# PHF12 regulates HDAC1 to promote tumorigenesis via EGFR/AKT signaling pathway in non-small cell lung cancer

**DOI:** 10.1186/s12967-024-05488-x

**Published:** 2024-07-29

**Authors:** Yiru Kong, Rongrong Jiang, Hui Zhou, Mengxi Ge, Hao Lin, Yu Wang, Rongrong Yao, Qing Wang, Xiaohua Liang, Jing Li, Xinli Zhou

**Affiliations:** 1 Department of Oncology, Huashan Hospital Fudan University, 12 Middle Urumqi Road, Shanghai, 200000 China; 2 Shanghai Medical College, Fudan University, Shanghai, 200032 China; 3 Department of Cardiothoracic Surgery, Huashan Hospital Fudan University, 12 Middle Urumqi Road, Shanghai, 200000 China

**Keywords:** PHF12, HDAC1, NSCLC, EGFR, Proliferation

## Abstract

**Background:**

Lung cancer stands as the second most prevalent malignant neoplasm worldwide. Addressing the underlying mechanisms propelling the progression of non-small cell lung cancer is of paramount importance. In this study, we have elucidated the pivotal role of PHF12 in this context.

**Materials and methods:**

We harnessed clinical lung cancer tissue samples and non-small cell lung cancer cell lines to discern the expression pattern of PHF12. In vitro assays probing cell proliferation were conducted to substantiate the functional impact of PHF12. Furthermore, an in vivo Xenograft model was employed to dissect the role of PHF12. Employing ChIP assays and qRT-PCR, we delved into the intricate binding dynamics between PHF12 and HDAC1. Mechanistic insights into the PHF12-HDAC1 axis in lung cancer progression were pursued via RNA-seq and GSEA analyses.

**Results:**

Notably, PHF12 exhibited a substantial upregulation within tumor tissue, concomitant with its correlation to HDAC1. The trilogy of cell proliferation assays, transwell assays, and the Xenograft model collectively underscored the promoting influence of PHF12 on lung cancer proliferation, both in vitro and in vivo. The ChIP assay unveiled the transcriptional regulatory role of PHF12 in governing HDAC1 expression. This correlation extended to both mRNA and protein levels. PHF12 promotes NSCLC progression through regulating HDCA1 expression. Intriguingly, the rescue of function within NSCLC cell lines post PHF12 knockdown was achievable through HDAC1 overexpression. Additionally, our findings unveiled the capacity of the PHF12-HDAC1 axis to activate the EGFR/AKT signaling pathway, thereby further corroborating its significance in lung cancer progression.

**Conclusion:**

Our study identified PHF12 as an oncogenic role in lung cancer proliferation and migration for the first time. PHF12 transcriptionally regulate HDAC1 and activate EGFR/AKT signaling pathway in NSCLC progression. PHF12 may serve as an important target in lung cancer therapy.

**Supplementary Information:**

The online version contains supplementary material available at 10.1186/s12967-024-05488-x.

## Introduction

Based on the comprehensive analysis of cancer statistics across 185 countries in the year 2020 encompassing 36 distinct cancer types, lung cancer emerges as a prominent concern, constituting 11.4% of all cancer cases. This places it firmly as the second most prevalent malignant neoplasm globally. Regrettably, lung cancer maintains its somber stature as the foremost contributor to cancer-related mortalities, commanding an 18% mortality rate. This substantial margin places it considerably ahead of other formidable contenders such as colon cancer with a mortality rate of 9.4%, liver cancer at 8.3%, and stomach cancer at 7.7% [1]. Although target molecular therapy and immunotherapy can inhibit tumor growth to a certain extent, poor efficacy and frequent drug resistance make the 5-year survival rate of lung cancer patients only 26% [2]. Unveiling novel molecular targets for targeted therapy and comprehending the intricate pathogenesis and evolution of lung cancer has unequivocally ascended to a paramount scientific imperative that demands immediate attention.

PHF12 (Plant Homeodomain Finger protein 12, PHF12), also known as KIAA1523, located in the long arm of human chromosome 17 (17q11.2), encodes a protein containing 1004 amino acids and has E3 ligase activity, located in the nucleus. The biological function and mechanistic role of PHF12 have garnered limited investigation thus far. Notably, PHF12 assumes significance in transcriptional regulation through its engagement with the Sin3B complex [3]. PHF12 is also reported as a bridge between two transcription systems, the transduction-like enhancer (TLE) and the Sin3A transcription system [4]. PHF12 functions as a transcriptional repressor, playing a pivotal role in orchestrating the DNA recruitment process of the active Sin3A complex. This engagement serves to uphold nucleolar integrity while also thwarting the onset of premature cellular senescence. [5]. PHF12 and Snail2 are believed to be recruited in the promoter region of Cad6b and are key regulators of neural crest epithelial-to-mesenchymal transition [6, 7]. Until now, there is still little research on the role of PHF12 in tumors, and previous study has found that disrupting the interaction between PHF12 and Sin3A complex can inhibit the migration and aggressiveness of triple negative breast cancer cells [8]. Overexpression of PHF12 effectively inhibits the growth of B-cell lymphomas with MYC dysregulation [9]. LncRNA RP11-116G8.5 may regulate the expression of PHF12 and FOXP4 by acting on miR-3150b-3p/miR-6870-5p [10].

Within the scope of our investigation, a substantial upregulation of PHF12 was discerned in non-small cell lung cancer (NSCLC) tumor specimens, displaying a noteworthy correlation with a less favorable clinical prognosis. Despite this, the precise function of PHF12 in the intricate landscape of lung cancer development remained enigmatic. Our study embarked on the elucidation of this ambiguity, unveiling that PHF12 collaboratively engages with HDAC1 to propel the proliferation of lung cancer cells. This collaborative action is executed through the activation of the EGFR/ErbB2 signaling pathway. This discovery not only augments our comprehension of lung cancer progression but also posits the PHF12-HDAC1 axis as an alluring candidate for therapeutic intervention in the realm of NSCLC treatment.

## Materials and methods

### Cell culture

Eight human NSCLC cell lines including A549, H1299, H292, PC9, H1975, H358, H460, and 293T were sourced from the American Tissue Culture Collection (ATCC) (Manassas, VA, USA). Eight cell lines were cultured with DMEM or 1640 medium supplemented with 10% fetal bovine serum (FBS) and penicillin sodium. Culturing was performed in a humidified incubator at 37 °C with 5% carbon dioxide. BSEA cells were obtained from Shanghai Cancer Institute. Cell lines were contamination free and routinely evaluated for cross-contamination.

### Human NSCLC samples

All of the 69 pairs of human NSCLC tissues and adjacent normal lung tissues were obtained from patients who had surgery in Huashan Hospital Fudan University from 2018 to 2019. The clinical information of all cases has been shown in Table 1. Because of sample size, only 40 pairs of samples can extract protein samples other than RNA samples. All human tissues were frozen immediately and stored in liquid nitrogen. Written informed consent has been collected from each patient. All procedures were carried out in accordance with the ethical code of the Declaration of Helsinki. This study was authorized by the Ethics Committee of Huashan Hospital Fudan University. The institutional approval number is 2021 − 922.

**Table 1:** Clinicopathologic features of 40 patients with lung cancer

Characteristics	Number of case%	PHF12 expression	
		Mean ± SD	*P* value
Age			
≤ 60	22 [32]	0.0060 ± 0.0060	0.879
$\:>\:$60	47 (68)	0.0063 ± 0.0057	
**Gender**			
Female	25 [36]	0.0065 ± 0.0071	0.740
Male	44 (64)	0.0060 ± 0.0049	
**Tumor size(cm)**			
≤ 3	26 [38]	0.0065 ± 0.0077	0.707
$\:>\:$3	43 (62)	0.0060 ± 0.0042	
**Tissue**			
NSCLC	69	0.0062 ± 0.005	**0.015**
Noncancerous	69	0.0057 ± 0.0053	
**Clinical stage**			
I + II	54 (78)	0.0057 ± 0.0050	0.271
III + IV	15 [22]	0.0081 ± 0.0078	
**Metastasis**			
No	35 [51]	0.0053 ± 0.0042	0.200
Yes	34 [49]	0.0071 ± 0.0069	

*P < 0.05, **P < 0.01 and ***P < 0.001.The bold formatting used in the table was considered to have a significant difference

### Western blot

Tissue Protein Extraction Reagent (Thermo Fisher, Waltham, USA) was used to extract protein from tumor tissues and cell lines. BCA Protein Assay Kit was used to measure the concentration of protein. Protein lysates were isolated with 6-10% SDS-PAGE electrophoresis. During protein extraction, cells are typically chosen at a density ranging from 50 to 60%. In a 10 cm cell culture dish, 200 µL of protein lysis buffer is typically added, while in a six-well plate, 60 µL of lysis buffer is added. Post-extraction, the sample concentration is carefully standardized. Following the initial test, the sample quantity is adjusted based on Actin, typically falling within the range of 10–20 µL. Marker and protein blot were transferred to PVDF membrane. All membranes were sealed with 5% milk and incubated with diluted primary antibody 4 °C overnight. Incubate a species-specific secondary antibody at room temperature for 1 h before fluorescent luminescence. Antibodies used for this study were anti-PHF12 (Proteintech, 24485-1-AP, 1:1000); anti-HDAC1 (Proteintech, 10197-1-AP, 1:1000); anti-EGFR (Proteintech, 18986-1-AP, 1:500); anti-ErbB2 (Proteintech, 18299-1-AP, 1:500); anti-p-AKT (Proteintech, 66,444, 1:500); anti-AKT (Proteintech, 10176-2-AP, 1:500)anti-p-mTOR(CST, 5536 S, 1:500); anti-mTOR (CST, 2983 S, 1:500); anti-β-actin (Proteintech, 81115-1-RR, 1:5000).

### RNA extraction and real-time qPCR

TRIzol reagent was used to extract total RNA samples according to the manufacturer’s protocol. PrimeScript RT Reagent Kit from TaKaRa was used to reverse transcribe all the RNA samples. cDNA samples were used to perform Real-time qPCR using SYBR Green Premix according to the protocol. When isolating RNA, DEPC water is incorporated based on the extent of precipitation. RNA concentration is typically maintained between 500-1000ng/mL. Similar to Western blot, the sample volume is modified relative to the Actin subsequent to the initial evaluation, commonly falling within the range of 10–20 µl. The primers used are listed below:

PHF12: F ATCGTGTACGACTTGGACACA.

R CCACCTTCCTTGCAGCTATCG.

HDAC1: F CTACTACGACGGGGATGTTGG.

R GAGTCATGCGGATTCGGTGAG.

EGFR: F AGGCACGAGTAACAAGCTCAC.

R ATGAGGACATAACCAGCCACC.

ErbB2: F TGCAGGGAAACCTGGAACTC.

R ACAGGGGTGGTATTGTTCAGC.

β-actin: F GTCATTCCAAATATGAGATGCGT.

R GCATTACATAATTACACGAAAGCA.

### RNA-sequence

In our experiment, Illumina was used for RNA sequencing. Before sequencing, it is necessary to build a library of RNA samples, including RNA reverse transcription, cDNA synthesis, library construction and PCR amplification. After the completion of sequencing, we first conducted quality control and filtering of the original sequencing data, and finally analyzed the data, including gene enrichment analysis, regulatory network analysis, etc. Raw data of RNA-seq could be downloaded from GSE225198.

### Transient transfection and Lentivirus constructs

All si-RNA oligonucleotides for PHF12 were designed and synthesized by RiboBio (Guangzhou, China). Cells were placed at 50–60% density in 6 dishes plates and transfected by siRNAs and Lipo2000 Reagent. The exposure time of siRNA is usually controlled within 6–8 h. Si-RNA used in this study were:

Si-PHF12#1: GTGTCCGAATCACATCGAA;

Si-PHF12#2: GGACCTCCGTTGACAGATT.

Sh-PHF12, HA-PHF12 and HA-HDAC1 was cloned into the lentiviral expression vector pWPXL. 12 µg Sh-PHF12, HA-PHF12 and HA-HDAC1 plasmid, 9 µg psPAX2, and 3.6 µg pMD2.G were transfected into 293T cells together with 60µL Lipo2000. Virus was collected after 48 h and filtered by membrane. Cells was infected with a 1:1 ratio of culture medium to viral liquid for 48 h.

### Cell proliferation, migration, and invasion assays

Cell proliferation is measured by CCK8 assay and Clone formation assay. Cells were plated in 6 dishes plate and transfected with si-RNAs for 48 h. In the CCK-8 assay, cells were seeded at a density of 800 cells per well, utilizing a 200µL volume of culture medium. In the colony assay, post cell counting, create a cell suspension in serum and antibiotic-infused medium at 5000 cells/mL concentration. Add 200µL of suspension per well in a six-well plate, seeding 1000 cells each. Supplement each well with 3mL of serum and antibiotic medium. In the Transwell assay, the upper chamber received 5 × 10^4^ cells for migration and 1 × 10^5^ cells for invasion, both suspended in 200µL of serum-free culture medium. Meanwhile, the lower chamber was supplemented with 800µL of culture medium containing 10% FBS. Difference between migration and invasion assays is that for invasion experiments, the Matrigel Matrix gel should be diluted 1:7 with culture medium without 10% serum 2–4 hours before the start of the experiment, and 60 µL of the diluted Matrigel Matrix gel should be spread on the chamber. This gel simulates the presence of a membrane, adding an obstacle to the process of cells transferring from the upper chamber to the lower chamber, so that it is no longer a simple migration, but an invasion. Chambers were placed at 37 °C for 24 h. In the proliferation assays, cells were fixed with methanol for 30 min and stained with 5% crystal violet for 10 min.

### Chromatin immunoprecipitation assay

A549, H1299, H292 cell lines were fixed and immunoprecipitated using ChIP assay kit (Beyotime) according to the manufacturer’s protocol. The condition of ultrasonic processing to cut DNA is 10 s a cycle, a total of three cycles. Illumina sequencing platform PE150 mode for high throughput sequencing. Results of ChIP-seq was attached in Additional file 1&2. Raw data of ChIP-seq could be downloaded from GSE225198.

Antibody used for immunoprecipitation is anti-PHF12 (Novus: NB100-81671, 1:100). Primers used to amplify the specific region of PHF12 is:

F: CCCATCAAGATTACCTCACGC.

R: TGGAGCGCCGATGGGAG.

### Immunofluorescence

A549, H1299, and H292 cells were placed in chambers with a density of 500 cells per well. All cells were fixed with 4% paraformaldehyde for 30 min and incubated with antibody 4 °C overnight. After washing with PBST for 3 times, cells were probed with species-specific secondary antibody. Anti-body used in this study was anti-PHF12 (Thermofisher, PA5-54400, 1:200).

### Xenograft model

3 × 10^6^ A549 and PC9 cells with stable PHF12 knockdown and overexpression were transplanted into five-week-old BALB/nu nude female mice. Cells are injected subcutaneously to mice. The diameter of the tumor was measured every two days. All the BALB nude mice were sacrificed 21 days after inoculation. All experiments were subject to approval by the Animal Care and Use Committee of Shanghai Cancer Institute.

### Statistical analysis

All data were collected and analyzed by Graphpad Prism version 5.0 (GraphPad Software, La Jolla, CA, USA). Comparisons between two groups should be made with student’s t-test. Kaplan-Meier was used to analyze survival curve and log-rank test. Spearman’s correlation test was used to analyze the correlations of PHF12 and HDAC1. When value of *P* < 0.05, it can be considered statistically significant. Every representative experiment was repeated three times.

## Results

### PHF12 is up regulated in cancer samples and is related with patient prognosis

**Fig. 1:**
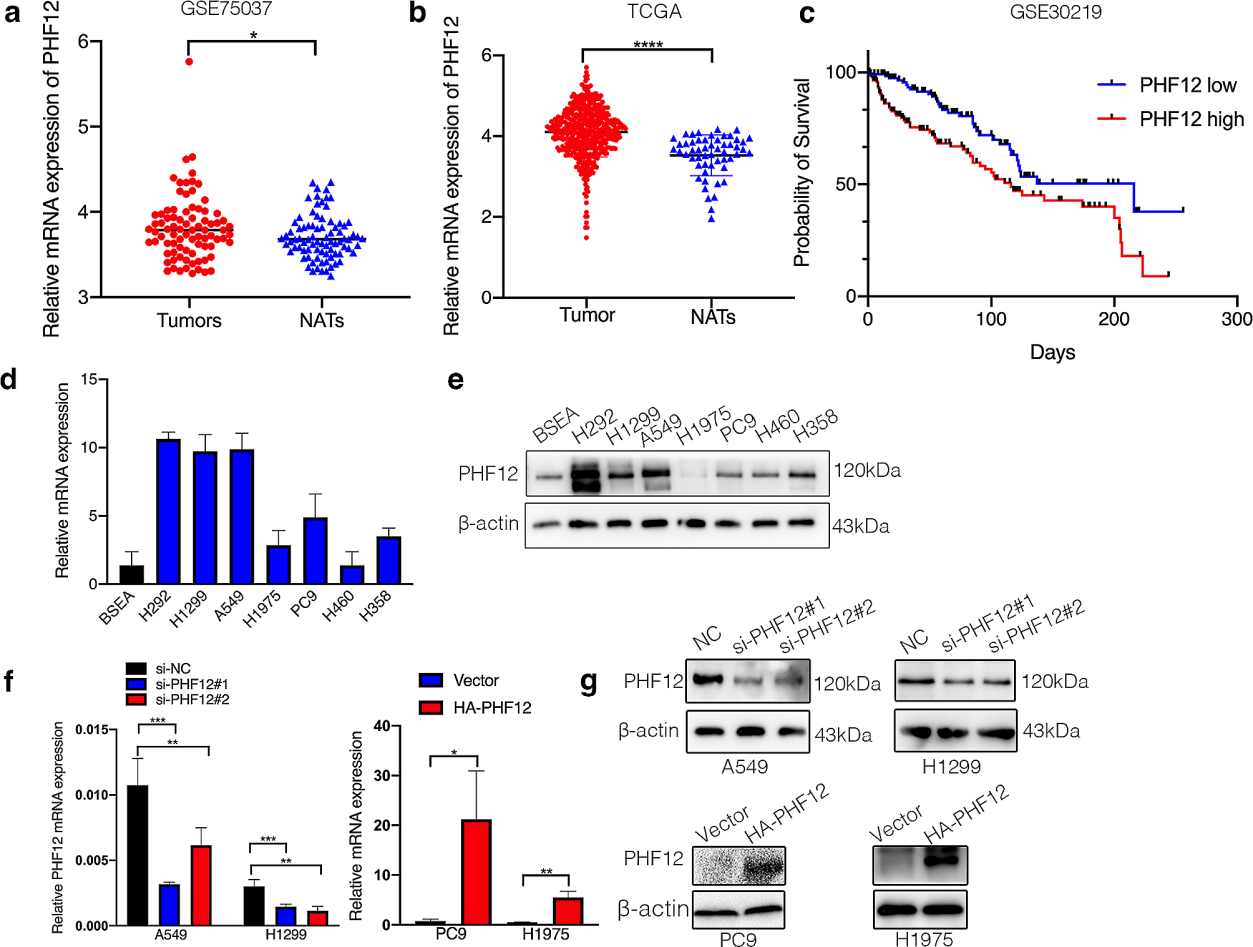
Screening for E3 ligases that promote lung cancer progression. a: Expression analysis of PHF12 in dataset GSE75037. b: Expression analysis of PHF12 in TCGA database. c: Survival analysis of PHF12 in dataset GSE30219. d-e: Expression of PHF12 at RNA level and protein level in lung cancer cell lines. f-g: Expression of PHF12 at protein level and RNA level in PHF12 knockdown in A549 and H1299 cells and PHF12 overexpression in PC9 and H1975. * *P* < 0.05; ** *P* < 0.01; *** *P* < 0.001, error bar represents SEM

We detected PHF12 expression in GSE75037 dataset and TCGA database and found that PHF12 is upregulated in lung adenocarcinoma samples compared with adjacent normal tissue (Fig. 1a). TCGA database also showed that PHF12 is significantly up regulated in lung cancer tissue compared with normal tissue (Fig. 1b). Kaplan-Meier’s survival analysis was performed to investigate the association between PHF12 expression and patient outcomes (Fig. 1c). Then, we detected PHF12 expression in non-small lung cancer cell lines both in protein level and mRNA level (Fig. 1d-e). To investigate the function of PHF12 in lung cancer cell lines, we knocked down PHF12 expression in high PHF12-expressing cell lines A549, H1299, and H292, while overexpressing PHF12 in low PHF12-expressing cell lines including PC9 and H1975 (Fig. 1f-g). Figures of H292 was shown in Additional file3.

### PHF12 promotes proliferation, metastasis, and invasion in lung cancer cell lines

**Fig. 2:**
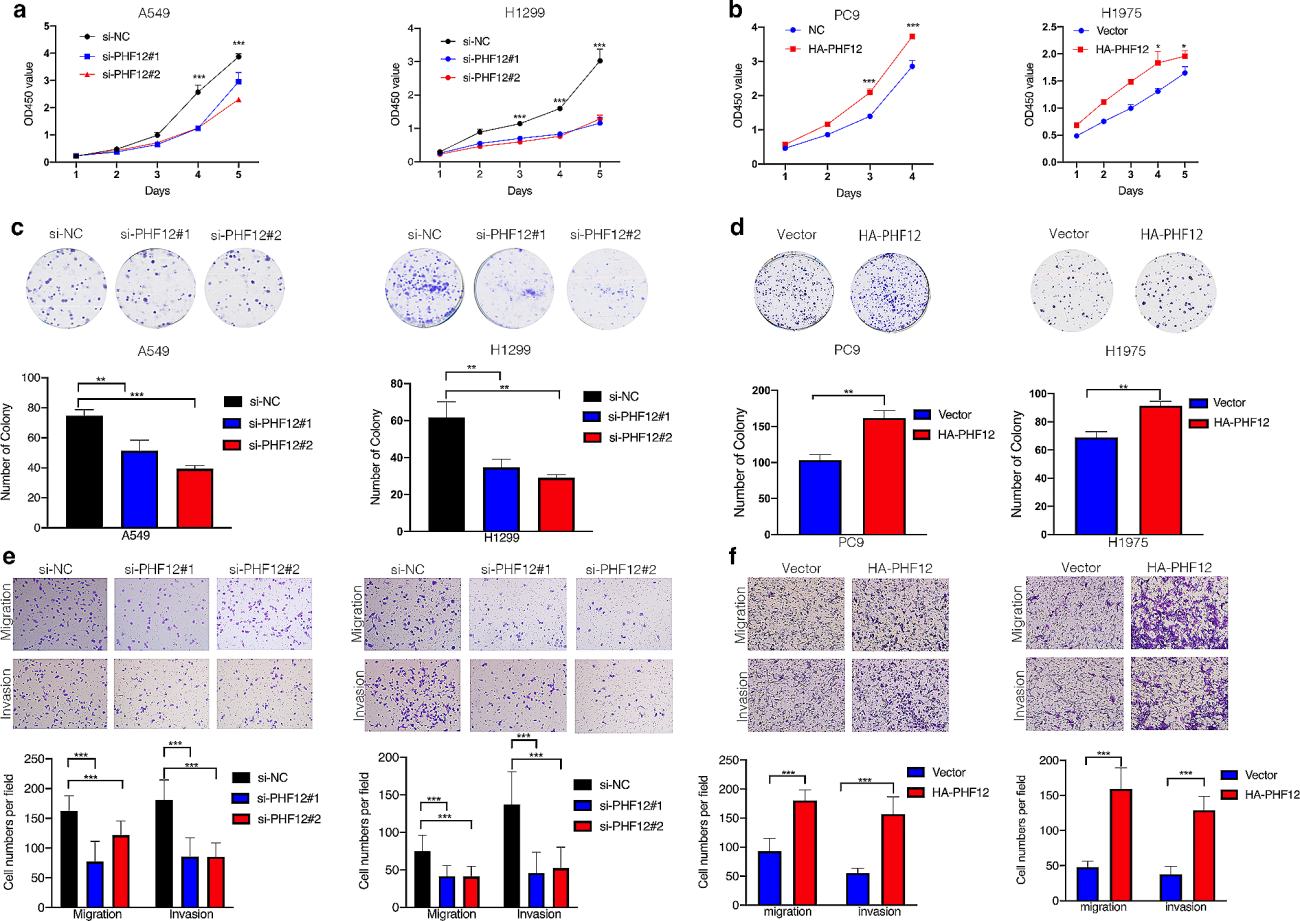
PHF12 promotes the proliferation, migration, and invasion of NSCLC. a: CCK8 assays of PHF12 knockdown in A549 and H1299 cell lines. b: CCK8 assays of PHF12 overexpression in PC9 and H1975 cell lines. c: Clone assays of PHF12 knockdown in A549 and H1299 cells. d: Clone assays of PHF12 overexpression in PC9 and H1975 cells. e: Transwell assays of PHF12 knockdown in A549 and H1299 cells. f: Transwell assays of PHF12 overexpression in PC9 and H1975 cells.* *P* < 0.05; ** *P* < 0.01; *** *P* < 0.001, error bar represents SEM

We performed cell proliferation assays to study PHF12 effect in cell function. CCK8 assay and clone formation assays were performed to examine the function of PHF12 in NSCLC cell lines. The results showed that PHF12 knockdown significantly inhibited cell proliferation while overexpression of PHF12 obviously promoted cell proliferation (Fig. 2a-d). Besides, we used transwell assay to detect the function of PHF12 in cell migration and invasion. We found that knockdown of PHF12 significantly suppressed the cell migration and invasion abilities in A549, H1299 cell lines (Fig. 2e). On the contrary, PHF12 overexpression distinctly enhanced migration and invasion rates compared with control cells in PC9 and H1975 cell lines (Fig. 2f). All these results demonstrated that PHF12 promotes the proliferation, migration, and invasion of NSCLC cells.

### PHF12 promotes NSCLC progression through regulating EGFR/ErbB2 signaling pathway

**Fig. 3:**
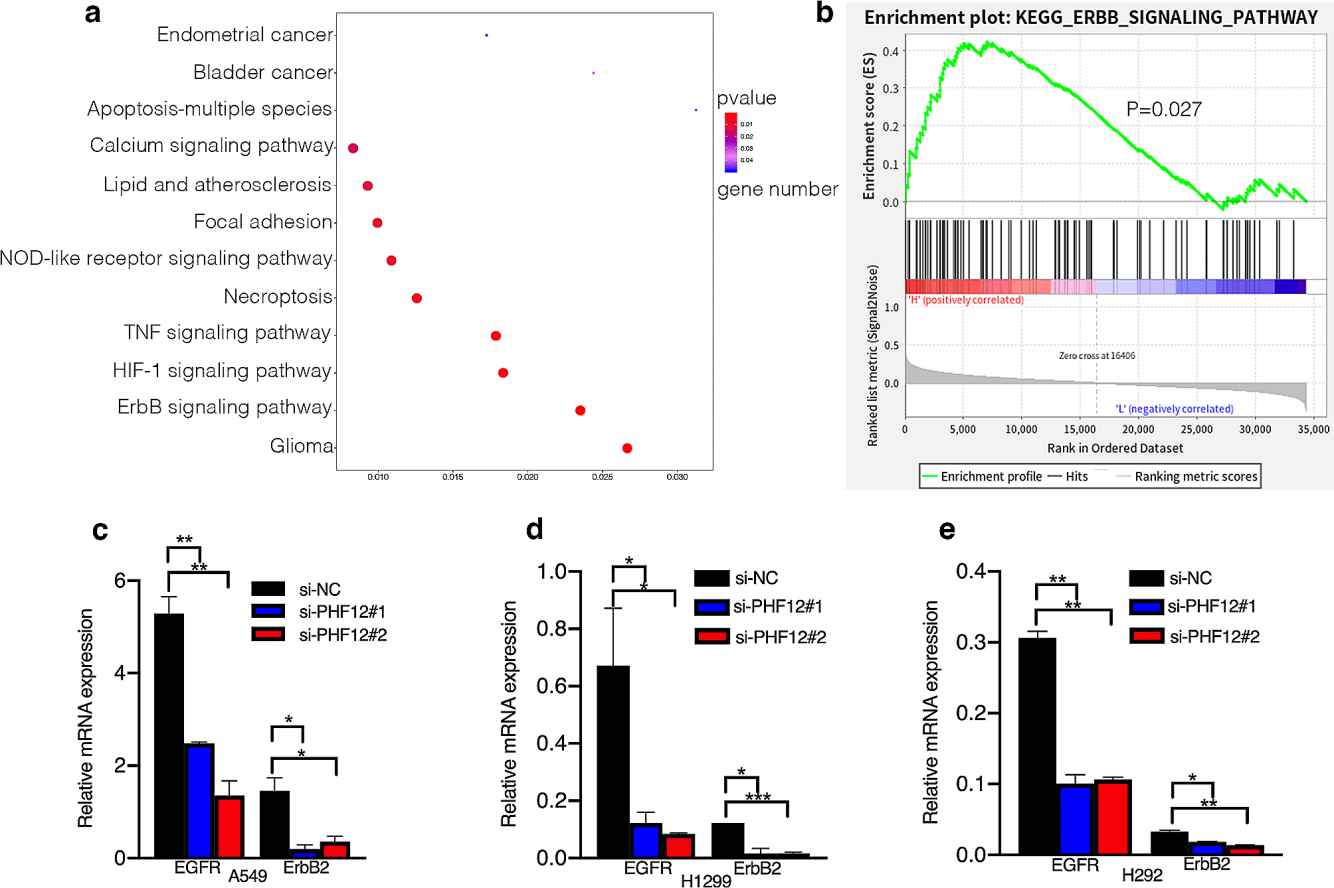
PHF12 promotes non-small cell lung cancer proliferation through activating EGFR signaling pathway. a: RNA-seq analysis showed that PHF12 regulation of lung cancer progression pathway prediction; The horizontal coordinate represents the proportion of enriched differential genes in the background genes of the channel, the size of points in the figure represents the number of enriched differential genes, and the color represents the p value. b: GSEA analysis showed that PHF12 was positively correlated with EGFR signaling pathway. c: Effects of PHF12 knockdown on EGFR signaling pathway in A549. d: Effect of PHF12 knockdown on EGFR signaling pathway in H1299. e: Effect of PHF12 knockdown in H292 on EGFR signaling pathway.* *P* < 0.05; ** *P* < 0.01; *** *P* < 0.001, error bar represents SEM

We performed RNA-seq analysis on control cells and PHF12 knockdown cells to explore the signaling pathway that PHF12 may regulate in the proliferation and metastasis of lung cancer. We conducted RNA-seq analysis using the KEGG pathway approach, revealing that PHF12 potentially modulates the progression of non-small cell lung cancer (NSCLC) through the EGFR/ErbB2 pathway, as illustrated in Fig. 3a. We also performed GSEA analysis to further explore the mechanisms of PHF12 in lung cancer development, and the results also showed that PHF12 was related to EGFR/ErbB2 signaling pathway (Fig. 3b). Both the KEGG analysis and GSEA analysis consistently indicated the potential regulatory role of PHF12 in the EGFR/ErbB2 signaling pathway. To validate this hypothesis, we conducted additional assays for further confirmation. Real-time qPCR was used to confirm that PHF12 knockdown significantly reduced mRNA expression of EGFR and ErbB2 compared to control cells in A549, H1299, and H292 cell lines (Fig. 3c-e).

### PHF12 regulates HDAC1 expression in both transcription and protein level

**Fig. 4:**
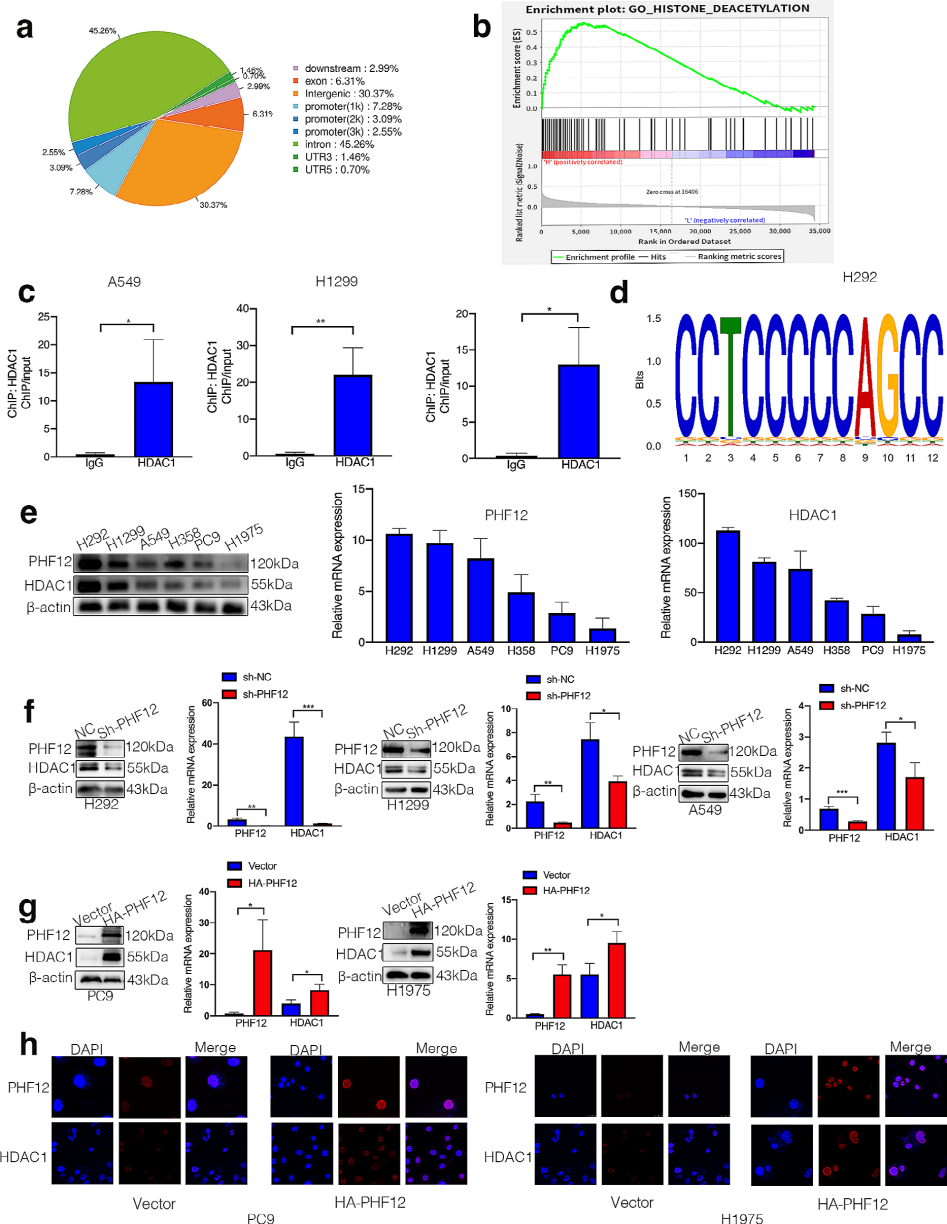
PHF12 is correlated with HDAC1 in both protein and RNA levels. a: Statistical map of PHF12 binding peak sites. b: GSEA enrichment analysis of PHF12. c: ChIP-qPCR verification of PHF12 regulates HDAC1 at the transcriptional level in A549, H1299, and H292 cell lines. d: Binding motif prediction of PHF12 and HDAC1. e: The expression of PHF12 was consistent with that of HDAC1 in non-small cell lung cancer cell lines. f: The expression level of HDAC1 expression was consistent with that of PHF12 in A549, H1299 and H292 cells. g: The expression of HDAC1 was consistent with that of PHF12 in PC9 and H1975. H: Colocalization pf PHF12 and HDAC1 in PC9 and HDAC1. * *P* < 0.05; ** *P* < 0.01; *** *P* < 0.001, error bar represents SEM

ChIP-seq was performed using H292 cells to find out the role of PHF12 in lung cancer progression as a transcription factor. After looking through the ChIP-seq results, we found that the most frequent binding peak sites of PHF12 in cancer cells were the intron region, accounting for 45.26% (Fig. 4a). The GSEA analysis was also conducted to elucidate the mechanisms of PHF12-regulated tumorigenesis. The results showed that PHF12 is highly related to histone deacetylation in cancer cells (Fig. 4b). According to the results, we determined 7 genes related to histone deacetylase, including HDAC1, HDAC5, HDAC6, HDAC7, HDAC8, HDAC10, HDAC11. We verified the non-binding relationship between the remaining 6 histone deacetylases except HDAC1 and PHF12 through ChIP-qPCR, and finally screened out HDAC1 for further study. We conducted ChIP assays using PHF12 antibody to test whether PHF12 binds to the intron region of HDAC1. According to the results of ChIP-seq (Supplementray file 1&2), we designed 2 sets of primers, and conducted chromatin immunoprecipitation experiment with PHF12 antibody. ChIP-qPCR assay was performed to assess the combination of PHF12 and HDAC1 in RNA level. The results showed that RNA level of the targeted intron region of HDAC1 in A549, H1299, and H292 cells increase significantly compared to IgG (Fig. 4c). The schematic shows the sequence logo of PHF12 and HDAC1 potential binding site indicated by STREME and MEME algorithm (Fig. 4d). We detected the expression of HDAC1 in NSCLC cell lines and the results showed that the HDAC1 expression is consistent with PHF12 in NSCLC cell lines (Fig. 4e). We further confirmed the protein and mRNA alterations in NSCLC cell lines. The expression of HDAC1 was observed downregulated after PHF12 knockdown in both protein and mRNA level in A549, H1299 and H292 cell lines (Fig. 4f). On the opposite, HDAC1 expression was significantly elevated in accordance with overexpression of PHF12 (Fig. 4g). Besides, IF assays were conducted to confirm the corresponding increase in HDAC1 expression after PHF12 overexpression in PC9 and H1975 cells (Fig. 4h). In addition, the IF assays also showed the colocalization of the two genes in the nucleus.

### PHF12 interacts with HDAC1 to promote lung cancer proliferation

**Fig. 5:**
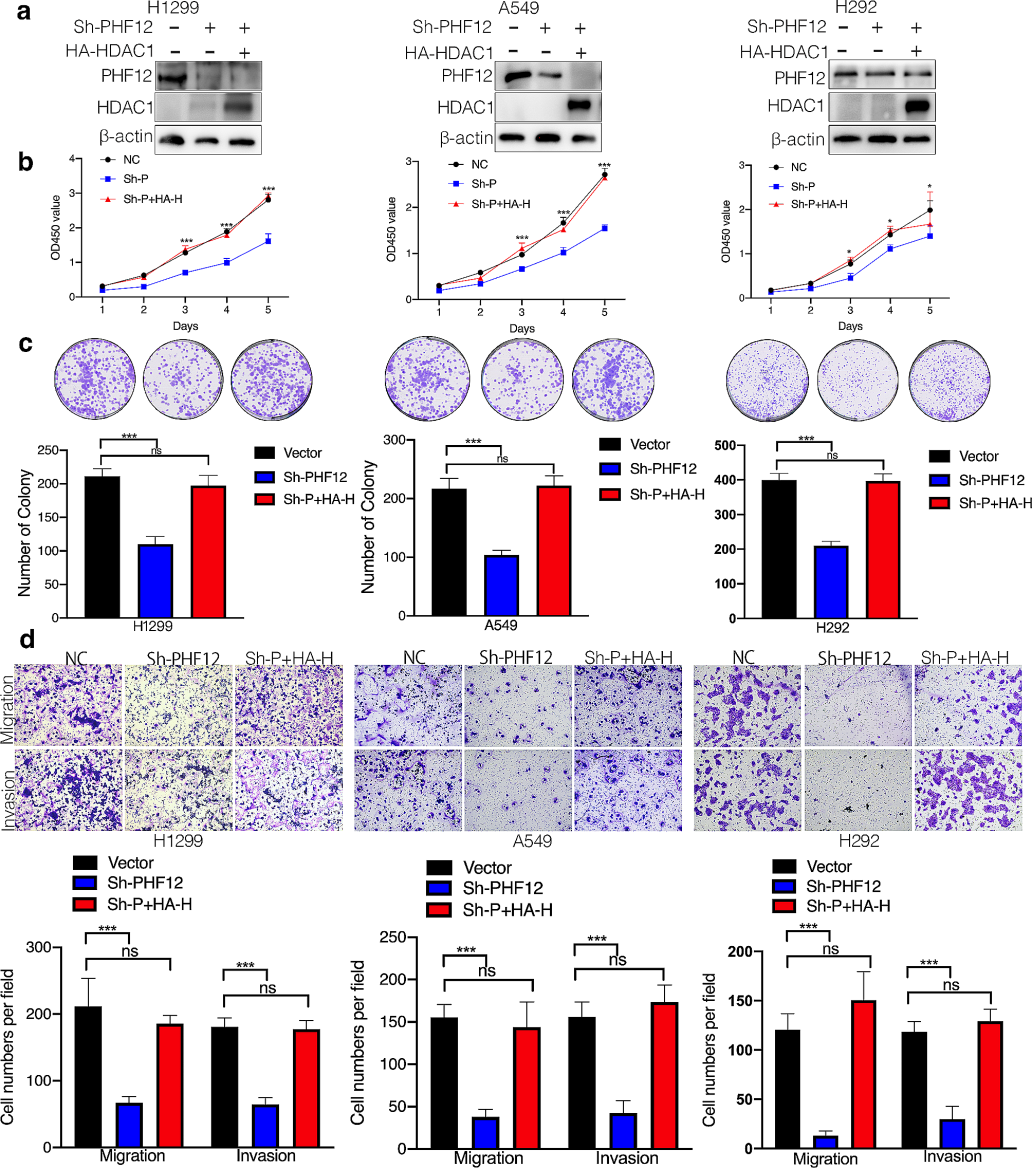
Effect of PHF12-HDAC1 axis on proliferation, migration, and invasion of lung cancer cells. a: Western Blot verified the efficiency of PHF12 knockdown and HDAC1 overexpression. b: Rescue assays with PHF12 knockdown and HDAC1 overexpression in CCK8 assays. c Rescue assays with PHF12 knockdown and HDAC1 overexpression in clone assays. d Rescue assays with PHF12 knockdown and HDAC1 overexpression in transwell assays. * *P* < 0.05; ** *P* < 0.01; *** *P* < 0.001, error bar represents SEM

To further study whether PHF12 exerts tumor-stimulative functions in lung cancer through HDAC1, rescue assays were used to detect the functions of NSCLC cells after PHF12 knockdown and HDAC1 overexpression. Firstly, the protein and mRNA levels of PHF12 and HDAC1 were detected in A549, H1299, and H292 (Fig. 5a). CCK8 assays showed that PHF12 knockdown significantly inhibited cell proliferation in H1299, A549, and H292 cell lines, while overexpression of HDAC1 after PHF12 knockdown can significantly rescue this inhibition (Fig. 5b). Overexpression of HDAC1 can rescue the reduced ability of clone formation in H1299, A549, and H292 cell lines in vitro after PHF12 knockdown (Fig. 5c). What’s more, the suppression of cell migration and invasion of A549, H1299 and H292 was also rescued by overexpression of HDAC1 in PHF12 knockdown cells (Fig. 5d).

### PHF12-HDAC1 axis regulates EGFR/AKT signaling pathway in NSCLC progression

**Fig. 6:**
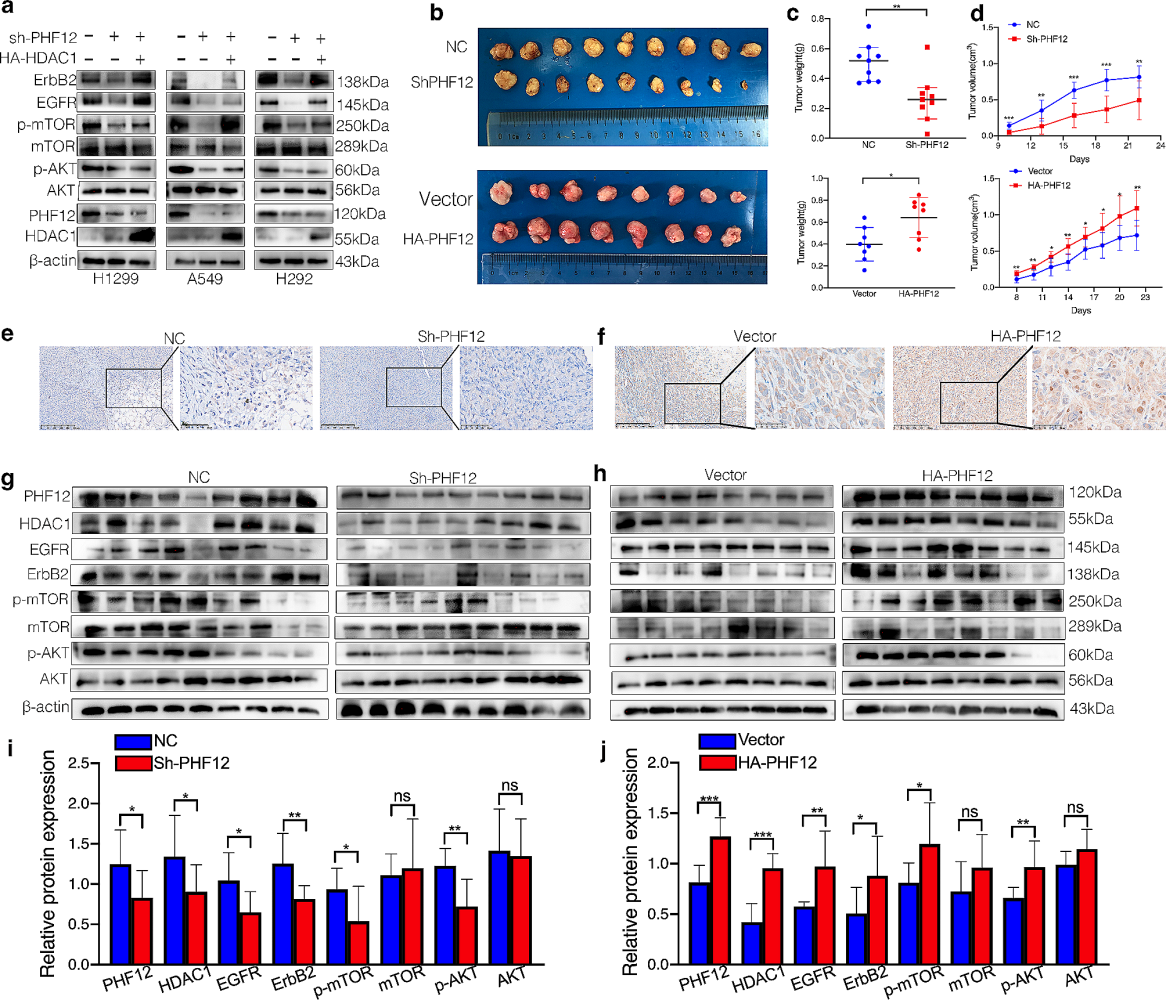
PHF12-HDAC1 axis can promote lung cancer cell proliferation by activating the EGFR/AKT signaling pathway. a: Signal pathway protein expression was detected in 3 groups of cells including: stable expression of Sh-PHF12, stable expression of Sh-PHF12 + HA-PHF12, and control group. b: Subcutaneous tumor model was established by cells with stable expression of Sh-PHF12/HA-PHF12 and control group. c: Tumor weight statistics of the two groups of subcutaneous tumor models. d: Tumor longitude statistics of the two groups of subcutaneous tumor model. e-f: The expression of PHF12 was verified by IHC in two groups of subcutaneous tumor models. g-h: Western Blot of EGFR/AKT signaling pathway protein expression in subcutaneous tumor models with stable PHF12 knockdown and PHF12 overexpression. i-j: Analysis of protein expression by Image J. * *P* < 0.05; ** *P* < 0.01; *** *P* < 0.001, error bar represents SEM

Since we found out that PHF12 may regulate the progression of NSCLC through EGFR signaling pathway. We used rescue assay to further detect whether HDAC1 exert in this regulation pathway. According to the results, overexpression of HDAC1 can rescue the suppress of EGFR signaling pathway and AKT-mTOR signaling pathway after PHF12 knockdown (Fig. 6a). Two subcutaneous tumor transplantation models were used to confirm the impact of PHF12 in tumorigenesis in vivo. A549 cells with stable PHF12 knockdown (Sh-PHF12 group) and control A549 cells (Control group) were transplanted into 2 groups of mice. PC9 cells with PHF12 overexpression (HA-PHF12 groups) and PC9 control cells (Vector groups) were transplanted into two other groups of mice (Fig. 6b). Tumor weight and tumor size of PHF12 knockdown group were significantly declined compared to control groups. On the contrary, tumor weight and tumor size of group with PHF12 stable overexpression were significantly increased compared to control group (Fig. 6c-d). IHC assay were performed to further confirm the expression of PHF12 in 4 groups (Fig. 6e-f). Then we extracted cell protein from 4 groups and tested the expression of PHF12 and HDAC1. The results showed that the expression of PHF12 and HDAC1 are consistent. Also, we tested the expression of EGFR, ErbB2, p-AKT, and p-mTOR in 4 groups of tumor tissues through western blot. Expression of EGFR, ErbB2, p-AKT, and p-mTOR is significantly reduced in Sh-PHF12 group. While expression of EGFR, ErbB2, p-AKT, and p-mTOR in HA-PHF12 group is higher than control group (Fig. 6g-h). Figure I-J shows the expression analysis of these protein.

### High expression of PHF12 and HDAC1 is related to worse clinical prognosis in NSCLC patients

**Fig. 7:**
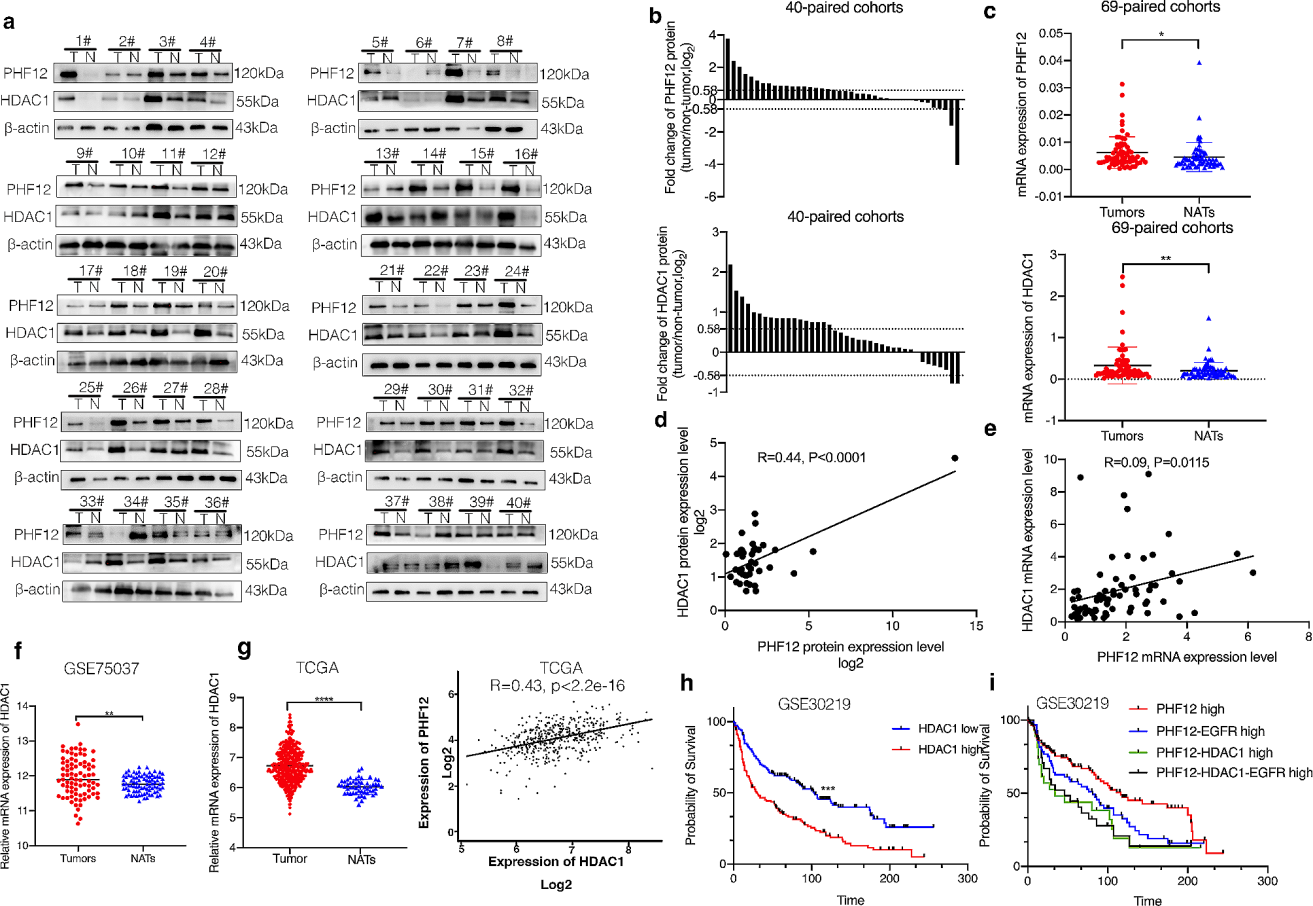
High expressions of PHF12 and HDAC1 were consistent in clinical samples and associated with poor clinical outcomes in patients with NSCLC. a: Protein level expression of PHF12 and HDAC1 in 40 pairs of clinical samples. b: Statistical analysis of PHF12 and HDAC1 expression in 40 pairs of clinical samples at protein level. C: Expression of PHF12 and HDAC1 in 69 pairs of clinical samples at RNA level. d: Statistical correlation between PHF12 and HDAC1 protein level expression in 40 pairs of clinical samples. e: Correlation statistics of RNA level expression of PHF12 and HDAC1 in 69 pairs of clinical samples. f: Detection of HDAC1 expression in tumor and normal lung tissue in GSE75037 and TCGA databases. g: Survival analysis of PHF12 and HDAC1 in dataset GSE30219. * *P* < 0.05; ** *P* < 0.01; *** *P* < 0.001, error bar represents SEM

To further explore the association with PHF12, HDAC1 and tumor proliferation, the expression of PHF12 and HDAC1 in clinical lung cancer tissues and adjacent normal lung tissues were detected through western blot and qRT-PCR. All samples were collected in Huashan Hospital Fudan University. Due to the size of these clinical samples, RNA samples could be extracted from all 69 pairs of clinical samples, but protein samples could only be extracted from 40 of the 69 pairs of clinical samples. The clinicopathologic characteristics of 69 patients have been shown in Table 1. The results showed that the protein expression of PHF12 is positively associated with HDAC1 in NSCLC samples (Fig. 7a-b, d). Besides, mRNA expression of PHF12 was also positively correlated with HDAC1 in 40 pairs of protein samples (Fig. 7c, e). We mined the GSE dataset and found that the expression of HDAC1 is significantly higher in NSCLC samples compared normal lung tissues in GSE75037 dataset (Fig. 7f). TCGA database also shows that HDAC1 expression is higher in tumor samples. Furthermore, PHF12 expression is positively correlated with HDAC1 in TCGA database (Fig. 7g). GSE30219 was used to detect the survival analysis of PHF12 and HDAC1. As expected, OS of HDAC1 low expression is better than high expression. More importantly, patients with PHF12-HDAC1 high expression have worse OS than patients with PHF12 high expression. The results also showed that patients with PHF12-HDAC1-EGFR high expression have worse OS than patients with PHF12 high expression. Although the survival of patients with high expression of PHF12-HDAC1-EGFR was not statistically worse than that of patients with high expression of PHF12-EGFR, the overall trend existed (Fig. 7h).

## Discussion

**Fig. 8:**
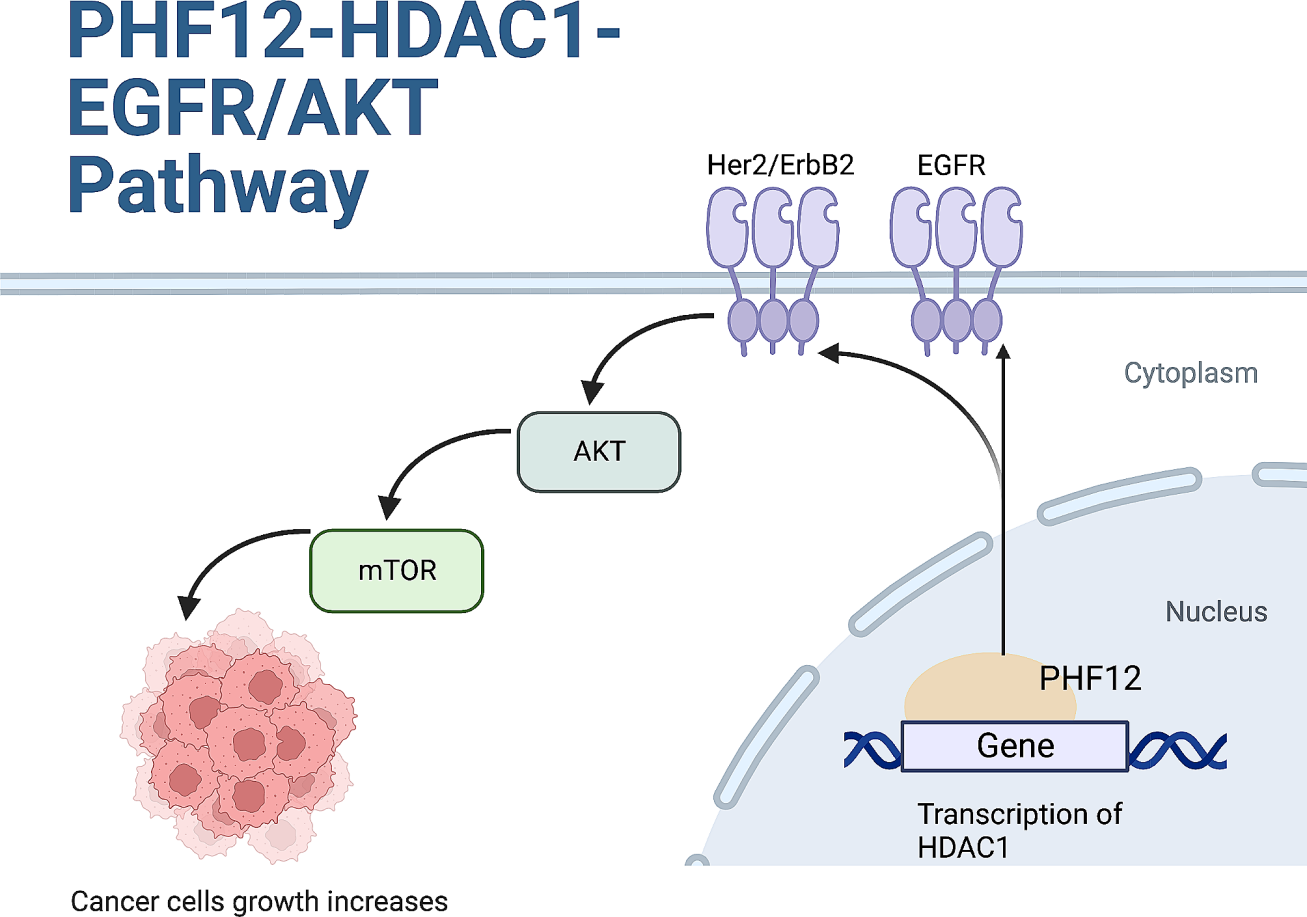
Potential mechanism of PHF12 regulating lung cancer proliferation

Lung cancer is the second most common cancer around the world. However, lung cancer still lacks effective therapy strategies. Therefore, unravelling the mechanisms of cancer progression is becoming increasingly urgent. Our study has first revealed that PHF12 interacts with HDAC1 to regulate EGFR/AKT signaling pathway and promote proliferation in non-small cell lung cancer (Fig. 8).

In our study, we detected PHF12 expression in public database and clinical samples collected in our hospital and confirmed that PHF12 exerts the role of tumorigenesis in NSCLC. What’s more, cell proliferation, migration and invasion assays were also used to verify the effect of promotor in the progression of NSCLC.

PHF12 has not only transcription factor activity but also E3 ligase activity. Increasingly studies have shown that histone deacetylase may be related to E3 ligase in cancer progression [11]. HDAC2 may mediate Parkin acetylation and activate mitophagy in cervical cancer [12]. Transcriptional changes caused by E3 ligase RNF5 inhibition are similar to changes caused by inhibition of histone deacetylase (HDAC)1 [13]. Inhibitor of HDAC can destabilize E3 ligase ZEB1 in a way that independent of histone acetylation [14]. E3 ligase may also regulate the effect of inhibitor of HDAC in many ways [15–18].

HDAC1, also known as HD1, KDAC1, and RPD3L. HDAC1 exhibited high expression in lung adenocarcinoma tissue compared to normal lung tissue [19]. It is reported that HDAC1 can be used as a biomarker for the efficacy and prognosis of platinum-based drugs in patients with non-small cell lung cancer [20]. Previously, it has been reported that PHF12 is an important part of the transcription complex Sin3B-PHF12, which contains Sin3B, PHF12, MRG15, and HDAC1 [3, 5]. Sin3B is considered as a bridge between PHF12 and HDAC1. However, it has also been reported that PHF12 links to the mSin3A complex instead of the Sin3B complex [4]. Here, we performed ChIP-seq and GSEA analysis and found the potential binding site of PHF12 and HDAC1. ChIP-qPCR assay was used to verify the binding of PHF12 and HDAC1. We noticed that the most binding peak sites of PHF12 were in the intron region, accounting for 45.26%. While according to the ChIP-seq results, PHF12 binds to the intron region of HDAC1 to regulate the expression of HDAC1. According to our results, we assume that different from many other transcription factors, PHF12 may bind to intron region to transcriptionally exert regulation effect. Other than the ChIP-qPCR analysis, we also found that the expression of HDAC1 in mRNA level and protein level coordinated with PHF12 knockdown and overexpression. HDAC1 expression is also consistent with PHF12 in cell lines and subcutaneous tumors of Xenograft model. However, restricted by our condition, more experiments are needed to perform to verify the activation of binding site of PHF12 and HDAC1.

HDAC1, also known as HD1, KDAC1, and RPD3L. HDAC1 exhibited high expression in lung adenocarcinoma tissue compared to normal lung tissue [19]. It is reported that HDAC1 can be used as a biomarker for the efficacy and prognosis of platinum-based drugs in patients with non-small cell lung cancer [20]. Previously, it has been reported that PHF12 is an important part of the transcription complex Sin3B-PHF12, which contains Sin3B, PHF12, MRG15, and HDAC1 [3, 5]. Sin3B is considered as a bridge between PHF12 and HDAC1. However, it has also been reported that PHF12 links to the mSin3A complex instead of the Sin3B complex [4]. Here, we performed ChIP-seq and GSEA analysis and found the potential binding site of PHF12 and HDAC1. ChIP-qPCR assay was used to verify the binding of PHF12 and HDAC1. We noticed that the most binding peak sites of PHF12 were in the intron region, accounting for 45.26%. While according to the ChIP-seq results, PHF12 binds to the intron region of HDAC1 to regulate the expression of HDAC1. According to our results, we assume that different from many other transcription factors, PHF12 may bind to intron region to transcriptionally exert regulation effect. Other than the ChIP-qPCR analysis, we also found that the expression of HDAC1 in mRNA level and protein level coordinated with PHF12 knockdown and overexpression. HDAC1 expression is also consistent with PHF12 in cell lines and subcutaneous tumors of Xenograft model. However, restricted by our condition, more experiments are needed to perform to verify the activation of binding site of PHF12 and HDAC1 (Fig. 8).

What’s more, according to recent studies, HDAC1 has developed many inhibitors. HDAC1 inhibitors have demonstrated efficacy in the treatment of several cancers, including gastric, breast, colorectal, prostate, colon, lung, ovarian, and pancreatic cancers, as well as in managing inflammation, with minimal associated toxic effects [21–24]. Despite the established efficacy of HDAC1 inhibitors in NSCLC and the acknowledged interaction with PHF12, we thought that confirming the utility of these inhibitors does not necessarily imply PHF12’s central role. However, given the confirmed interaction between HDAC1 and PHF12, we highly hypothesize that HDAC1 inhibitors could potentially be beneficial for patients exhibiting PHF12 overexpression.

EGFR and ErbB2 belong to the epidermal growth factor receptor (EGFR) family, which includes EGFR/Her1/ ErbB1, Her2/ErbB2, Her3/ErbB3, and Her4/ErbB4 [25]. EGFR/ErbB2 signaling pathway has been reported as important target in various kinds of cancer, including breast cancer, head and neck cancer, gastric cancer, pancreatic cancer, colon cancer, renal cell carcinoma, glioblastoma and non-small cell lung cancer, etc [26–37]. The downstream EGFR signaling pathway usually promotes cell proliferation [38], angiogenesis [39], tumor metastasis and invasion [40] and reduction of apoptosis [41].

E3 ligase plays a regulatory role in a variety of diseases and tumors by regulating EGFR signaling pathway, UPS11 promotes renal fibrosis by deubiquitination of EGFR [42]. PUF60 regulates E3 ligase by transcription in glioblastoma. STUB1 activates the EGFR/AKT signaling pathway and promotes tumorigenesis [43]. The cantoD and ZC3H15 can promote cancer by activating the EGFR signaling pathway [44, 45]. TRAF4 can lead to trastuzumab resistance in HER2-positive breast cancer by regulating the ErbB2 signaling pathway [46]. In lung cancer, FBXW2 can inhibit the proliferation and metastasis of lung cancer by activating EGFR signaling pathway [47]. EGFR signaling can promote the proliferation of lung adenocarcinoma by downregulating the E3 ligase NEDD4L [48]. Studies have shown that AKT signaling can be activated by EGFR signaling in a variety of ways [49, 50]. EGFR/AKT signaling pathway is thought to be involved in the development and development of various malignant tumors, including breast cancer and glioblastoma [43, 51].

Our results showed that expression of EGFR, ErbB2, p-AKT, and p-mTOR were reduced after PHF12 knockdown. Besides, the expression of EGFR, ErbB2, AKT, and mTOR were consistent with PHF12 and HDAC1. In rescue experiments, HDAC1 overexpression could rescue the effects of PHF12 knockdown in EGFR, ErbB2, p-AKT, and p-mTOR. Our results demonstrated that PHF12 interacts with HDAC1 to regulate the EGFR/AKT signaling pathway.

In our research, PHF12 was investigated for the first time in non-small cell lung cancer (NSCLC), representing a pivotal advancement in the therapeutic landscape. This pioneering investigation represents a crucial step forward in our understanding of NSCLC pathology and opens new avenues for targeted therapeutic interventions.

## Conclusion

In our study, we confirmed for the first time the promoter role of PHF12 in non-small cell lung cancer and proposed that PHF12-HDAC1 axis regulates the EGFR/AKT signaling pathway and promotes the development of lung cancer. Our study sheds light on the role of PHF12 in NSCLC and provided evidence for targeted therapy.

### Electronic supplementary material

Below is the link to the electronic supplementary material.

Supplementary file 1: Resuls of ChIP-seq part1
This file contains the first part of the results of ChIP-seq generated during this research.

Supplementary file 2: Resuls of ChIP-seq part2
This file contains the second part of the results of ChIP-seq generated during this research.

Supplementary file 3:Si-RNA experiments of H292 cell line
This file contains the results of si-RNA experiments of H292 cell line.

## Data Availability

The datasets generated during the current study are available in GSE225198.

## Declarations

All experiments were subject to approval by the Animal Care and Use Committee of Shanghai Cancer Institute.

The authors are accountable for all aspects of the work in ensuring that questions related to the accuracy or integrity of any part of the work are appropriately investigated and resolved.
