# Supplementary material for: PHF12 regulates HDAC1 to promote tumorigenesis via EGFR/AKT signaling pathway in non-small cell lung cancer
Source: J Transl Med. 2024 Jul 29;22:689. doi: 10.1186/s12967-024-05488-x (PMC11287983; doi:10.1186/s12967-024-05488-x)
Supplement: Supplementary file 2 — Resuls of ChIP-seq part2 This file contains the second part of the results of ChIP-seq generated during this research. [file 12967_2024_5488_MOESM2_ESM.pdf]

| motif_id | motif_alt_i | sequence_ | start | stop | strand | score | p-value | q-value |
|---|---|---|---|---|---|---|---|---|
| 169-GAGC1 | STREME-16 | Nc-1-IP_pe | 197 | 208 | + | 16.622 | 2.67E-06 | 0.0975 |
| 169-GAGC1 | STREME-16 | Nc-1-IP_pe | 201 | 212 | + | 11.1707 | 5.26E-05 | 0.25 |
| 43-AGTAG( | STREME-43 | Nc-1-IP_pe | 6 | 16 | + | 20.9342 | 2.49E-07 | 0.00162 |
| 43-AGTAG( | STREME-43 | Nc-1-IP_pe | 86 | 96 | + | 20.9342 | 2.49E-07 | 0.00162 |
| 43-AGTAG( | STREME-43 | Nc-1-IP_pe | 89 | 99 | + | 20.9342 | 2.49E-07 | 0.00162 |
| 43-AGTAG( | STREME-43 | Nc-1-IP_pe | 391 | 401 | + | 20.9342 | 2.49E-07 | 0.00162 |
| 43-AGTAG( | STREME-43 | Nc-1-IP_pe | 364 | 374 | - | 20.9342 | 2.49E-07 | 0.00162 |
| 43-AGTAG( | STREME-43 | Nc-1-IP_pe | 380 | 390 | + | 17.3289 | 9.57E-07 | 0.00525 |
| 42-CCTCCC | STREME-42 | Nc-1-IP_pe | 254 | 265 | - | 16.4939 | 1.52E-06 | 0.0605 |
| 42-CCTCCC | STREME-42 | Nc-1-IP_pe | 316 | 327 | - | 16.3476 | 3.71E-06 | 0.0734 |
| 42-CCTCCC | STREME-42 | Nc-1-IP_pe | 69 | 80 | + | 11.2073 | 3.07E-05 | 0.101 |
| 42-CCTCCC | STREME-42 | Nc-1-IP_pe | 203 | 214 | + | 11.2073 | 3.07E-05 | 0.101 |
| 42-CCTCCC | STREME-42 | Nc-1-IP_pe | 283 | 294 | - | 11.2073 | 3.07E-05 | 0.101 |
| 27-ACTAAA | STREME-27 | Nc-1-IP_pe | 132 | 143 | - | 24.6842 | 2.20E-08 | 0.000177 |
| 27-ACTAAA | STREME-27 | Nc-1-IP_pe | 136 | 147 | - | 24.6842 | 2.20E-08 | 0.000177 |
| 27-ACTAAA | STREME-27 | Nc-1-IP_pe | 50 | 61 | - | 17.75 | 4.06E-07 | 0.00233 |
| 27-ACTAAA | STREME-27 | Nc-1-IP_pe | 437 | 448 | - | 15.9737 | 8.62E-07 | 0.00459 |
| 27-ACTAAA | STREME-27 | Nc-1-IP_pe | 424 | 435 | - | 15.1447 | 1.09E-06 | 0.00568 |
| 27-ACTAAA | STREME-27 | Nc-1-IP_pe | 270 | 281 | - | 13.9342 | 2.03E-06 | 0.00982 |
| 27-ACTAAA | STREME-27 | Nc-1-IP_pe | 479 | 490 | + | 7.97368 | 2.31E-05 | 0.0732 |
| 75-GGCAT( | STREME-75 | Nc-1-IP_pe | 54 | 63 | - | 6.79268 | 4.30E-05 | 0.329 |
| 75-GGCAT( | STREME-75 | Nc-1-IP_pe | 57 | 66 | - | 6.79268 | 4.30E-05 | 0.329 |
| 75-GGCAT( | STREME-75 | Nc-1-IP_pe | 208 | 217 | - | 6.79268 | 4.30E-05 | 0.329 |
| 75-GGCAT( | STREME-75 | Nc-1-IP_pe | 407 | 416 | + | 6.73171 | 5.65E-05 | 0.38 |
| 75-GGCAT( | STREME-75 | Nc-1-IP_pe | 124 | 133 | + | 6.64634 | 5.97E-05 | 0.384 |
| 3-GCCACCF | STREME-3 | Nc-1-IP_pe | 29 | 38 | + | 16.1636 | 1.78E-06 | 0.0329 |
| 3-GCCACCF | STREME-3 | Nc-1-IP_pe | 109 | 118 | + | 16.1636 | 1.78E-06 | 0.0329 |
| 3-GCCACCF | STREME-3 | Nc-1-IP_pe | 247 | 256 | + | 15.8545 | 3.19E-06 | 0.033 |
| 3-GCCACCF | STREME-3 | Nc-1-IP_pe | 11 | 20 | - | 14.3818 | 1.98E-05 | 0.0511 |
| 3-GCCACCF | STREME-3 | Nc-1-IP_pe | 14 | 23 | - | 14.3818 | 1.98E-05 | 0.0511 |
| 3-GCCACCF | STREME-3 | Nc-1-IP_pe | 153 | 162 | + | 13.7818 | 2.46E-05 | 0.0569 |
| 3-GCCACCF | STREME-3 | Nc-1-IP_pe | 402 | 411 | + | 7.54545 | 5.92E-05 | 0.109 |
| 3-GCCACCF | STREME-3 | Nc-1-IP_pe | 440 | 449 | + | 6.05455 | 7.17E-05 | 0.13 |
| 4-CAAGYG/ | STREME-4 | Nc-1-IP_pe | 352 | 363 | + | 19.9636 | 5.51E-08 | 0.00217 |
| 4-CAAGYG/ | STREME-4 | Nc-1-IP_pe | 250 | 261 | - | 16.8364 | 1.60E-06 | 0.00939 |
| 4-CAAGYG/ | STREME-4 | Nc-1-IP_pe | 192 | 203 | + | 11.8545 | 2.42E-05 | 0.0652 |
| 4-CAAGYG/ | STREME-4 | Nc-1-IP_pe | 363 | 374 | + | 11.6182 | 2.64E-05 | 0.0705 |
| 4-CAAGYG/ | STREME-4 | Nc-1-IP_pe | 196 | 207 | + | 11.3091 | 2.97E-05 | 0.0766 |
| 4-CAAGYG/ | STREME-4 | Nc-1-IP_pe | 58 | 69 | + | 10.7818 | 3.68E-05 | 0.0901 |
| 4-CAAGYG/ | STREME-4 | Nc-1-IP_pe | 61 | 72 | + | 10.7818 | 3.68E-05 | 0.0901 |
| 4-CAAGYG/ | STREME-4 | Nc-1-IP_pe | 365 | 376 | - | 10.1455 | 4.60E-05 | 0.109 |
| 4-CAAGYG/ | STREME-4 | Nc-1-IP_pe | 391 | 402 | - | 8.54545 | 8.01E-05 | 0.168 |
| 20-GGCGC( | STREME-20 | Nc-1-IP_pe | 105 | 116 | + | 15.5286 | 2.44E-06 | 0.0427 |
| 20-GGCGC( | STREME-20 | Nc-1-IP_pe | 102 | 113 | + | 5.52857 | 3.88E-05 | 0.307 |

| | | | | | | | | |
|---|---|---|---|---|---|---|---|---|
| 99-GCCCA | STREME-99 | Nc-1-IP_pe | 362 | 373 | + | 11.061 | 5.73E-05 | 0.26 |
| 99-GCCCA | STREME-99 | Nc-1-IP_pe | 373 | 384 | + | 11.061 | 5.73E-05 | 0.26 |
| 6-AAAATTA | STREME-6 | Nc-1-IP_pe | 39 | 50 | - | 22.6724 | 4.38E-08 | 0.000741 |
| 6-AAAATTA | STREME-6 | Nc-1-IP_pe | 424 | 435 | - | 22.6724 | 4.38E-08 | 0.000741 |
| 6-AAAATTA | STREME-6 | Nc-1-IP_pe | 119 | 130 | - | 22.3103 | 7.85E-08 | 0.000741 |
| 6-AAAATTA | STREME-6 | Nc-1-IP_pe | 412 | 423 | - | 22.3103 | 7.85E-08 | 0.000741 |
| 6-AAAATTA | STREME-6 | Nc-1-IP_pe | 330 | 341 | + | 22.2069 | 1.13E-07 | 0.000741 |
| 6-AAAATTA | STREME-6 | Nc-1-IP_pe | 122 | 133 | - | 14.2241 | 3.99E-06 | 0.017 |
| 122-CTTGG | STREME-12 | Nc-1-IP_pe | 486 | 497 | + | 11.4939 | 2.03E-05 | 0.384 |
| 122-CTTGG | STREME-12 | Nc-1-IP_pe | 183 | 194 | + | 11.3476 | 3.03E-05 | 0.405 |
| 7-CCTGGC | STREME-7 | Nc-1-IP_pe | 287 | 297 | + | 18.9273 | 6.26E-07 | 0.00416 |
| 7-CCTGGC | STREME-7 | Nc-1-IP_pe | 455 | 465 | + | 18.9273 | 6.26E-07 | 0.00416 |
| 7-CCTGGC | STREME-7 | Nc-1-IP_pe | 467 | 477 | - | 18.9273 | 6.26E-07 | 0.00416 |
| 7-CCTGGC | STREME-7 | Nc-1-IP_pe | 166 | 176 | - | 18.7273 | 8.75E-07 | 0.00469 |
| 7-CCTGGC | STREME-7 | Nc-1-IP_pe | 70 | 80 | - | 16.2545 | 2.21E-06 | 0.01 |
| 7-CCTGGC | STREME-7 | Nc-1-IP_pe | 162 | 172 | - | 16.2545 | 2.21E-06 | 0.01 |
| 7-CCTGGC | STREME-7 | Nc-1-IP_pe | 300 | 310 | - | 14.4727 | 5.37E-06 | 0.0158 |
| 7-CCTGGC | STREME-7 | Nc-1-IP_pe | 454 | 464 | - | 13.5273 | 7.96E-06 | 0.0221 |
| 7-CCTGGC | STREME-7 | Nc-1-IP_pe | 289 | 299 | - | 10.4182 | 2.26E-05 | 0.0559 |
| 78-CCTCTG | STREME-78 | Nc-1-IP_pe | 276 | 284 | - | 11.2378 | 5.11E-05 | 0.251 |
| 14-GGCRT | STREME-14 | Nc-1-IP_pe | 236 | 247 | + | 21.2623 | 1.10E-07 | 0.00155 |
| 14-GGCRT | STREME-14 | Nc-1-IP_pe | 240 | 251 | + | 19.6066 | 2.84E-07 | 0.00181 |
| 14-GGCRT | STREME-14 | Nc-1-IP_pe | 206 | 217 | - | 11.5246 | 1.48E-05 | 0.0512 |
| 14-GGCRT | STREME-14 | Nc-1-IP_pe | 146 | 157 | + | 7.03279 | 7.24E-05 | 0.199 |
| 14-GGCRT | STREME-14 | Nc-1-IP_pe | 347 | 358 | - | 6.47541 | 8.63E-05 | 0.226 |
| 46-AGGGA | STREME-46 | Nc-1-IP_pe | 35 | 46 | - | 10.8902 | 6.10E-05 | 0.166 |
| 102-CAAG | STREME-10 | Nc-1-IP_pe | 314 | 325 | + | 11.9634 | 1.84E-05 | 0.27 |
| 102-CAAG | STREME-10 | Nc-1-IP_pe | 480 | 491 | + | 6.86585 | 7.53E-05 | 0.27 |
| 102-CAAG | STREME-10 | Nc-1-IP_pe | 71 | 82 | - | 6.83537 | 9.36E-05 | 0.27 |
| 102-CAAG | STREME-10 | Nc-1-IP_pe | 205 | 216 | - | 6.83537 | 9.36E-05 | 0.27 |
| 5-TCTCTAC | STREME-5 | Nc-1-IP_pe | 137 | 148 | - | 24.4494 | 2.76E-08 | 0.000195 |
| 5-TCTCTAC | STREME-5 | Nc-1-IP_pe | 141 | 152 | - | 24.4494 | 2.76E-08 | 0.000195 |
| 5-TCTCTAC | STREME-5 | Nc-1-IP_pe | 312 | 323 | + | 21.2697 | 5.53E-08 | 0.000346 |
| 5-TCTCTAC | STREME-5 | Nc-1-IP_pe | 429 | 440 | - | 16.8202 | 6.38E-07 | 0.00294 |
| 5-TCTCTAC | STREME-5 | Nc-1-IP_pe | 275 | 286 | - | 11.6629 | 5.08E-06 | 0.0191 |
| 5-TCTCTAC | STREME-5 | Nc-1-IP_pe | 442 | 453 | - | 7.79775 | 2.33E-05 | 0.0736 |
| 5-TCTCTAC | STREME-5 | Nc-1-IP_pe | 266 | 277 | - | 7.5618 | 2.50E-05 | 0.0783 |
| 77-GGTTCA | STREME-77 | Nc-1-IP_pe | 256 | 265 | - | 7.7439 | 3.16E-05 | 0.241 |
| 11-AACCC | STREME-11 | Nc-1-IP_pe | 49 | 60 | - | 20.36 | 1.10E-07 | 0.00347 |
| 11-AACCC | STREME-11 | Nc-1-IP_pe | 78 | 89 | - | 16.8267 | 1.55E-06 | 0.00847 |
| 11-AACCC | STREME-11 | Nc-1-IP_pe | 371 | 382 | + | 15.24 | 3.10E-06 | 0.0107 |
| 11-AACCC | STREME-11 | Nc-1-IP_pe | 46 | 57 | - | 14.0933 | 5.34E-06 | 0.0174 |
| 11-AACCC | STREME-11 | Nc-1-IP_pe | 81 | 92 | - | 12.9867 | 9.60E-06 | 0.0293 |
| 11-AACCC | STREME-11 | Nc-1-IP_pe | 280 | 291 | - | 12.8 | 1.07E-05 | 0.0322 |
| 11-AACCC | STREME-11 | Nc-1-IP_pe | 372 | 383 | - | 12.2667 | 1.44E-05 | 0.0416 |

| | | | | | | | | |
|---|---|---|---|---|---|---|---|---|
| 11-AACCCG | STREME-11 | Nc-1-IP_pe | 351 | 362 | - | 11.76 | 1.83E-05 | 0.0497 |
| 11-AACCCG | STREME-11 | Nc-1-IP_pe | 383 | 394 | - | 8.8 | 6.03E-05 | 0.123 |
| 10-CGGCTC | STREME-10 | Nc-1-IP_pe | 31 | 42 | + | 22.4 | 8.72E-08 | 0.00156 |
| 10-CGGCTC | STREME-10 | Nc-1-IP_pe | 421 | 432 | - | 21.0533 | 1.57E-07 | 0.00163 |
| 10-CGGCTC | STREME-10 | Nc-1-IP_pe | 28 | 39 | + | 13.1467 | 3.98E-06 | 0.0194 |
| 10-CGGCTC | STREME-10 | Nc-1-IP_pe | 333 | 344 | + | 12.9467 | 4.29E-06 | 0.0207 |
| 10-CGGCTC | STREME-10 | Nc-1-IP_pe | 322 | 333 | + | 12.1733 | 4.94E-06 | 0.0234 |
| 109-ACCTC | STREME-10 | Nc-1-IP_pe | 40 | 50 | + | 8.29333 | 1.15E-05 | 0.0809 |
| 109-ACCTC | STREME-10 | Nc-1-IP_pe | 377 | 387 | + | 6.68 | 2.72E-05 | 0.105 |
| 109-ACCTC | STREME-10 | Nc-1-IP_pe | 43 | 53 | + | 4.76 | 5.54E-05 | 0.158 |
| 109-ACCTC | STREME-10 | Nc-1-IP_pe | 345 | 355 | + | 4.42667 | 6.02E-05 | 0.158 |
| 39-ACTGCA | STREME-39 | Nc-1-IP_pe | 34 | 45 | + | 20.8533 | 1.94E-07 | 0.00168 |
| 39-ACTGCA | STREME-39 | Nc-1-IP_pe | 37 | 48 | + | 19.28 | 2.49E-07 | 0.00204 |
| 25-AGGAG. | STREME-25 | Nc-1-IP_pe | 62 | 71 | - | 19.4 | 8.93E-07 | 0.0243 |
| 25-AGGAG. | STREME-25 | Nc-1-IP_pe | 65 | 74 | - | 19.4 | 8.93E-07 | 0.0243 |
| 115-AGCCC | STREME-11 | Nc-1-IP_pe | 113 | 124 | - | 10.9605 | 8.84E-06 | 0.198 |
| 115-AGCCC | STREME-11 | Nc-1-IP_pe | 207 | 218 | - | 6.22368 | 5.97E-05 | 0.354 |
| 51-GGGTTT | STREME-51 | Nc-1-IP_pe | 456 | 466 | + | 20.9211 | 2.49E-07 | 0.00314 |
| 51-GGGTTT | STREME-51 | Nc-1-IP_pe | 155 | 165 | + | 17.3289 | 5.62E-07 | 0.00559 |
| 51-GGGTTT | STREME-51 | Nc-1-IP_pe | 151 | 161 | + | 13.4868 | 6.55E-06 | 0.0381 |
| 51-GGGTTT | STREME-51 | Nc-1-IP_pe | 59 | 69 | + | 12.7368 | 8.63E-06 | 0.0491 |
| 51-GGGTTT | STREME-51 | Nc-1-IP_pe | 443 | 453 | + | 8.23684 | 7.18E-05 | 0.244 |
| 181-CAAGC | STREME-18 | Nc-1-IP_pe | 250 | 261 | - | 4.0102 | 2.55E-05 | 0.185 |
| 181-CAAGC | STREME-18 | Nc-1-IP_pe | 461 | 472 | + | 3.92857 | 2.62E-05 | 0.185 |
| 181-CAAGC | STREME-18 | Nc-1-IP_pe | 58 | 69 | + | -0.86735 | 9.63E-05 | 0.341 |
| 181-CAAGC | STREME-18 | Nc-1-IP_pe | 61 | 72 | + | -0.86735 | 9.63E-05 | 0.341 |
| 62-CCTGCC | STREME-62 | Nc-1-IP_pe | 72 | 83 | + | 11.5244 | 4.23E-05 | 0.313 |
| 19-CCCCRN | STREME-19 | Nc-1-IP_pe | 192 | 200 | - | 14.0149 | 1.01E-05 | 0.157 |
| 19-CCCCRN | STREME-19 | Nc-1-IP_pe | 10 | 18 | - | 11.1493 | 9.44E-05 | 0.238 |
| 167-AACCC | STREME-16 | Nc-1-IP_pe | 153 | 159 | - | 13.2012 | 6.54E-05 | 0.391 |
| 167-AACCC | STREME-16 | Nc-1-IP_pe | 441 | 447 | - | 13.2012 | 6.54E-05 | 0.391 |
| 180-CACCT | STREME-18 | Nc-1-IP_pe | 114 | 124 | - | 0.797753 | 9.93E-05 | 0.636 |
| 145-CTGGT | STREME-14 | Nc-1-IP_pe | 81 | 90 | + | 18.1463 | 2.02E-06 | 0.0221 |
| 145-CTGGT | STREME-14 | Nc-1-IP_pe | 277 | 286 | - | 18.1463 | 2.02E-06 | 0.0221 |
| 145-CTGGT | STREME-14 | Nc-1-IP_pe | 465 | 474 | + | 18.1463 | 2.02E-06 | 0.0221 |
| 145-CTGGT | STREME-14 | Nc-1-IP_pe | 173 | 182 | + | 16.4634 | 2.91E-06 | 0.0244 |
| 145-CTGGT | STREME-14 | Nc-1-IP_pe | 478 | 487 | + | 7.04878 | 6.85E-05 | 0.257 |
| 145-CTGGT | STREME-14 | Nc-1-IP_pe | 259 | 268 | - | 6.84146 | 8.11E-05 | 0.257 |
| 145-CTGGT | STREME-14 | Nc-1-IP_pe | 345 | 354 | + | 6.84146 | 8.11E-05 | 0.257 |
| 145-CTGGT | STREME-14 | Nc-1-IP_pe | 356 | 365 | + | 6.84146 | 8.11E-05 | 0.257 |
| 156-GCCCA | STREME-15 | Nc-1-IP_pe | 2 | 13 | + | 16.7744 | 2.77E-06 | 0.0288 |
| 156-GCCCA | STREME-15 | Nc-1-IP_pe | 450 | 461 | - | 16.7744 | 2.77E-06 | 0.0288 |
| 156-GCCCA | STREME-15 | Nc-1-IP_pe | 304 | 315 | + | 11.5061 | 4.05E-05 | 0.155 |
| 156-GCCCA | STREME-15 | Nc-1-IP_pe | 263 | 274 | + | 11.1829 | 5.09E-05 | 0.155 |
| 156-GCCCA | STREME-15 | Nc-1-IP_pe | 282 | 293 | - | 11.1829 | 5.09E-05 | 0.155 |

| | | | | | | | | |
|---|---|---|---|---|---|---|---|---|
| 156-GCCCA | STREME-15 | Nc-1-IP_pe | 420 | 431 | - | 6.70732 | 7.95E-05 | 0.215 |
| 47-ATCTCG | STREME-47 | Nc-1-IP_pe | 27 | 37 | + | 20.7067 | 2.49E-07 | 0.00402 |
| 47-ATCTCG | STREME-47 | Nc-1-IP_pe | 329 | 339 | + | 8.62667 | 2.23E-05 | 0.105 |
| 47-ATCTCG | STREME-47 | Nc-1-IP_pe | 24 | 34 | + | 8.57333 | 2.47E-05 | 0.114 |
| 47-ATCTCG | STREME-47 | Nc-1-IP_pe | 426 | 436 | - | 7.54667 | 3.83E-05 | 0.153 |
| 66-TGGGG | STREME-66 | Nc-1-IP_pe | 287 | 294 | + | 12.4583 | 7.29E-05 | 0.359 |
| 66-TGGGG | STREME-66 | Nc-1-IP_pe | 358 | 365 | + | 12.4583 | 7.29E-05 | 0.359 |
| 17-CAYTGC | STREME-17 | Nc-1-IP_pe | 7 | 18 | - | 21.7846 | 1.24E-07 | 0.00088 |
| 17-CAYTGC | STREME-17 | Nc-1-IP_pe | 10 | 21 | - | 21.7846 | 1.24E-07 | 0.00088 |
| 17-CAYTGC | STREME-17 | Nc-1-IP_pe | 301 | 312 | - | 21.7846 | 1.24E-07 | 0.00088 |
| 17-CAYTGC | STREME-17 | Nc-1-IP_pe | 442 | 453 | + | 17.7846 | 4.10E-07 | 0.00261 |
| 163-TGCTA | STREME-16 | Nc-1-IP_pe | 399 | 408 | - | 8.65244 | 6.55E-05 | 0.89 |
| 171-ACAAA | STREME-17 | Nc-1-IP_pe | 415 | 426 | - | 7.06098 | 6.75E-05 | 0.271 |
| 48-AGGCCT | STREME-48 | Nc-1-IP_pe | 251 | 262 | + | 6.87195 | 7.53E-05 | 0.559 |
| 22-AGSYCA | STREME-22 | Nc-1-IP_pe | 90 | 101 | - | 17.7286 | 5.92E-07 | 0.00422 |
| 22-AGSYCA | STREME-22 | Nc-1-IP_pe | 487 | 498 | - | 17.0571 | 7.90E-07 | 0.0053 |
| 22-AGSYCA | STREME-22 | Nc-1-IP_pe | 182 | 193 | - | 8.68571 | 1.05E-05 | 0.0434 |
| 22-AGSYCA | STREME-22 | Nc-1-IP_pe | 186 | 197 | - | 8.68571 | 1.05E-05 | 0.0434 |
| 22-AGSYCA | STREME-22 | Nc-1-IP_pe | 266 | 277 | + | 4.71429 | 2.78E-05 | 0.0947 |
| 107-ATGAA | STREME-10 | Nc-1-IP_pe | 52 | 63 | - | 16.2135 | 8.92E-07 | 0.1 |
| 107-ATGAA | STREME-10 | Nc-1-IP_pe | 49 | 60 | - | 9.24719 | 1.02E-05 | 0.345 |
| 107-ATGAA | STREME-10 | Nc-1-IP_pe | 409 | 420 | - | 1.67416 | 8.59E-05 | 0.416 |
| 134-CAAGC | STREME-13 | Nc-1-IP_pe | 192 | 199 | - | 15.2683 | 1.45E-05 | 0.311 |
| 134-CAAGC | STREME-13 | Nc-1-IP_pe | 448 | 455 | - | 10.122 | 7.24E-05 | 0.617 |
| 76-AGCCGC | STREME-76 | Nc-1-IP_pe | 403 | 414 | + | 8.39024 | 1.08E-05 | 0.282 |
| 28-GAGCA | STREME-28 | Nc-1-IP_pe | 289 | 299 | - | 11.5429 | 2.15E-05 | 0.113 |
| 28-GAGCA | STREME-28 | Nc-1-IP_pe | 466 | 476 | + | 9.11429 | 5.55E-05 | 0.229 |
| 28-GAGCA | STREME-28 | Nc-1-IP_pe | 298 | 308 | + | 8.1 | 8.21E-05 | 0.298 |
| 29-CAAACT | STREME-29 | Nc-1-IP_pe | 224 | 235 | - | -0.50667 | 3.67E-05 | 0.573 |
| 32-ATCTCA | STREME-32 | Nc-1-IP_pe | 481 | 492 | + | -0.8 | 2.06E-05 | 0.575 |
| 12-CCTGAC | STREME-12 | Nc-1-IP_pe | 186 | 197 | + | 20.0492 | 2.44E-07 | 0.00285 |
| 12-CCTGAC | STREME-12 | Nc-1-IP_pe | 190 | 201 | + | 18.1803 | 7.04E-07 | 0.00577 |
| 12-CCTGAC | STREME-12 | Nc-1-IP_pe | 94 | 105 | + | 15.0492 | 2.45E-06 | 0.0157 |
| 12-CCTGAC | STREME-12 | Nc-1-IP_pe | 216 | 227 | + | 6.08197 | 5.20E-05 | 0.182 |
| 38-ACARAC | STREME-38 | Nc-1-IP_pe | 287 | 298 | - | 5.14667 | 2.80E-05 | 0.13 |
| 38-ACARAC | STREME-38 | Nc-1-IP_pe | 317 | 328 | + | 5.12 | 2.81E-05 | 0.13 |
| 38-ACARAC | STREME-38 | Nc-1-IP_pe | 321 | 332 | + | 0.88 | 9.73E-05 | 0.212 |
| 38-ACARAC | STREME-38 | Nc-1-IP_pe | 267 | 278 | - | 0.88 | 9.73E-05 | 0.212 |
| 117-AGGAC | STREME-11 | Nc-1-IP_pe | 235 | 246 | + | 8.37079 | 2.08E-05 | 0.449 |
| 164-CTGTT | STREME-16 | Nc-1-IP_pe | 310 | 321 | - | 12.1524 | 8.63E-06 | 0.324 |
| 44-GTCAGA | STREME-44 | Nc-1-IP_pe | 429 | 440 | - | 11.9878 | 1.84E-05 | 0.36 |
| 49-TAGGAA | STREME-49 | Nc-1-IP_pe | 291 | 302 | - | 0.512195 | 1.70E-05 | 0.383 |
| 50-AAAGAC | STREME-50 | Nc-1-IP_pe | 484 | 495 | + | 6.04878 | 3.51E-06 | 0.239 |
| 1-GCTGGG. | STREME-1 | Nc-1-IP_pe | 10 | 21 | + | 23 | 5.51E-08 | 0.000252 |
| 1-GCTGGG. | STREME-1 | Nc-1-IP_pe | 224 | 235 | + | 23 | 5.51E-08 | 0.000252 |

| | | | | | | | | |
|---|---|---|---|---|---|---|---|---|
| 1-GCTGGG. | STREME-1 | Nc-1-IP_pe | 228 | 239 | + | 23 | 5.51E-08 | 0.000252 |
| 1-GCTGGG. | STREME-1 | Nc-1-IP_pe | 90 | 101 | + | 21.1636 | 1.24E-07 | 0.000433 |
| 1-GCTGGG. | STREME-1 | Nc-1-IP_pe | 93 | 104 | + | 21.1636 | 1.24E-07 | 0.000433 |
| 1-GCTGGG. | STREME-1 | Nc-1-IP_pe | 134 | 145 | + | 19.6 | 1.68E-07 | 0.00055 |
| 1-GCTGGG. | STREME-1 | Nc-1-IP_pe | 218 | 229 | - | 19.1818 | 2.12E-07 | 0.000663 |
| 1-GCTGGG. | STREME-1 | Nc-1-IP_pe | 384 | 395 | + | 16.6545 | 8.01E-07 | 0.00205 |
| 1-GCTGGG. | STREME-1 | Nc-1-IP_pe | 359 | 370 | - | 15.7273 | 1.62E-06 | 0.00377 |
| 1-GCTGGG. | STREME-1 | Nc-1-IP_pe | 395 | 406 | + | 15.7273 | 1.62E-06 | 0.00377 |
| 61-AGATC( | STREME-61 | Nc-1-IP_pe | 22 | 30 | - | 16.7467 | 5.07E-06 | 0.232 |
| 61-AGATC( | STREME-61 | Nc-1-IP_pe | 120 | 128 | + | 4.62667 | 6.95E-05 | 0.963 |
| 82-CTTGCG | STREME-82 | Nc-1-IP_pe | 378 | 389 | - | 6.88 | 2.08E-05 | 0.478 |
| 63-GGCAG' | STREME-63 | Nc-1-IP_pe | 488 | 499 | + | 10.4268 | 3.51E-06 | 0.194 |
| 63-GGCAG' | STREME-63 | Nc-1-IP_pe | 87 | 98 | - | 6.68293 | 1.14E-05 | 0.321 |
| 65-AGATCT | STREME-65 | Nc-1-IP_pe | 47 | 58 | - | 0.280488 | 2.39E-05 | 0.542 |
| 147-GCCG( | STREME-14 | Nc-1-IP_pe | 186 | 196 | - | 8.65476 | 1.64E-05 | 0.371 |
| 2-GCCTCR( | STREME-2 | Nc-1-IP_pe | 72 | 83 | + | 21.4909 | 1.10E-07 | 0.000669 |
| 2-GCCTCR( | STREME-2 | Nc-1-IP_pe | 206 | 217 | + | 21.4909 | 1.10E-07 | 0.000669 |
| 2-GCCTCR( | STREME-2 | Nc-1-IP_pe | 226 | 237 | - | 21.4909 | 1.10E-07 | 0.000669 |
| 2-GCCTCR( | STREME-2 | Nc-1-IP_pe | 261 | 272 | - | 21.4909 | 1.10E-07 | 0.000669 |
| 2-GCCTCR( | STREME-2 | Nc-1-IP_pe | 210 | 221 | + | 20.2182 | 2.48E-07 | 0.00115 |
| 2-GCCTCR( | STREME-2 | Nc-1-IP_pe | 377 | 388 | + | 20.0545 | 3.35E-07 | 0.00134 |
| 2-GCCTCR( | STREME-2 | Nc-1-IP_pe | 345 | 356 | + | 17.0909 | 9.08E-07 | 0.00274 |
| 2-GCCTCR( | STREME-2 | Nc-1-IP_pe | 75 | 86 | + | 17.0727 | 9.95E-07 | 0.00293 |
| 2-GCCTCR( | STREME-2 | Nc-1-IP_pe | 116 | 127 | + | 16 | 1.77E-06 | 0.00441 |
| 2-GCCTCR( | STREME-2 | Nc-1-IP_pe | 366 | 377 | + | 15.8364 | 1.84E-06 | 0.00455 |
| 69-CTCAGT | STREME-69 | Nc-1-IP_pe | 382 | 393 | - | 7.15854 | 6.31E-05 | 0.636 |
| 79-GAAAA( | STREME-79 | Nc-1-IP_pe | 288 | 299 | - | 6.76829 | 1.82E-05 | 0.562 |
| 80-AGATY( | STREME-80 | Nc-1-IP_pe | 20 | 27 | - | 13.36 | 4.05E-05 | 0.643 |
| 80-AGATY( | STREME-80 | Nc-1-IP_pe | 23 | 30 | - | 12.3733 | 5.87E-05 | 0.688 |
| 86-CGTAG( | STREME-86 | Nc-1-IP_pe | 299 | 310 | + | 3.95122 | 4.71E-05 | 0.905 |
| 150-AGGC/ | STREME-15 | Nc-1-IP_pe | 367 | 378 | - | 1.5 | 2.41E-05 | 0.469 |
| 113-GGGCI | STREME-11 | Nc-1-IP_pe | 195 | 205 | - | 8.52439 | 1.45E-05 | 0.143 |
| 113-GGGCI | STREME-11 | Nc-1-IP_pe | 199 | 209 | - | 7.47561 | 2.44E-05 | 0.143 |
| 113-GGGCI | STREME-11 | Nc-1-IP_pe | 355 | 365 | - | 7.47561 | 2.44E-05 | 0.143 |

matched_sequence
gatctgcctgcc
gatccgcctgcc
agtagctggga
agtagctggga
agtagctggga
agtagctggga
AGTAGCTGGGA
agtagctggaa
CCTCCCGCAGCC
CCTCCCTCAGCC
cctgcctcagcc
cctgcctcagcc
CCTCTCCGAGCC
ACTAAAAATACA
ACTAAAAATACA
CCTAAAAATACA
ACCGAAAATACA
ATAAAAAATACA
ATTAAAAATAAA
aaaaaaaagaca
GGCATGAACC
GGCATGAACC
GGCATGAACC
ggcatacacc
GGCCTGCACC
gccaccacgc
gccaccacgc
gccaccacac
GCCACTGCAC
GCCACTGCAC
gccactgtgc
Gctactatac
actactgcac
caagtgatcctc
CAAGCAATCCTC
ctcgtgatctgc
caaacaatcctc
cttgtgatccgc
catgccattctc
catgccattctc
CACGCGATCCCC
CAAAAGATCCTC
ggcgtccaccac
ggcacctgccac

tcccacctcagc
tcccacctcagc
AAAATTAGCCGG
AAAATTAGCCGG
AAAATTAGCCAG
AAAATTAGCCAG
aaaattagctgg
AAAATTACCCAG
CTTGGTCAGGCC
CTTGGCCATCCT
cctgggcaaca
cctgggcaaca
CCTGGGCAACA
CCTGGCTAACA
CCTGACTAACA
CCTGACTAACA
CCTGGGTGACA
CTTGGGCAACA
CCTGGATGACA
CCTCTCCCC
ggcgtgagccac
ggtgtgagccac
GGCATGAACCGC
ggcctgagccac
GGTGTGCACCGC
AGGGGGGAGGGG
gaggctgaggga
GAATCTGATGGA
GAGGCTGAGGCA
GAGGCTGAGGCA
TCTCTACTAAAA
TCTCTACTAAAA
tctctacaaaaa
TCTCTATAAAAA
TCTCAATTAAAA
TCTCCACCGAAA
TATCTAAAAAAA
GGCTCAAGCA
AACCCGGGAGGC
TACTCGGGAGGC
tactcaggaggt
AACCCAAGAGGT
TACTCGGGAGAC
AACTAGGGAGGC
TACTCAGGAAGC

AGCCCAGGAGGC
TACTGAGGAGGC
cggctcactgca
TGGCTCACTGCA
cggcccactgca
cggctccctgca
catctcactgca
agctccacctc
acctcagcctc
agctctgcctc
gcctctgcctc
actgcaagctcc
actgcaagctct
AGGAGAATGG
AGGAGAATGG
AGCCGAGGCTGG
GGCCGAGGCAGG
gggtttcacca
gggtttcaccg
gggtttccccg
aggtttctcca
gggtttcacct
CAAGCAATCCTC
caagctggtctc
catgccattctc
catgccattctc
cctgcctcagtc
CCCCGCCCC
CCCCGCCCG
AACCCCA
AACCCCA
CATCTTGCTCG
ctggtctcaa
CTGGTCTCAA
ctggtctcaa
atggtctcga
ctgatctcaa
CTGGGCTCAA
ctgggctcaa
ctgggctcaa
gcccaggctgga
GCCCAGGCTGGA
acccaggctgga
gcccagccagga
GCCCAGGCTGGT

GGACACTCTGGA
atctcggctca
atctcggctcc
atctcggccca
ATCATGGCTCA
tggggtct
TGGGGTCT
CACTGCACTCCA
CACTGCACTCCA
CACTGCACTCCA
tactgcactcca
TAGTAGCACA
ACAAAAATTAGC
aggattgcttga
AGGTCGGGAGTT
AGCTTAGGAGTT
AGGTCAGGAGAT
AGGTCAGGAGAT
cagccaggagtt
ATGAACCCGGGA
ATGAACCCAAGA
ATTAGCCAGGTA
CAAGGTCA
CAAGGTGA
agcctggaaggt
GAGTGAGACCC
gagcgagacca
tagtgagaccc
CAAAGTGCTAGG
atctcaaactcc
cctgacctcgtg
cctgaccttgtg
cccgacctcagg
CCTGGCCTTGTC
AAAAGCAAACTA
acaaaaaaataa
aaaaataaaaaa
AAAAATAAAAAA
gggagcccaagg
TTGTTCTCCAGC
GCCACACAGGGA
TAGGAAAAGCAA
aaagacattagt
gctgggattaca
gctgggattaca

gctgggattaca
gctgggactaca
gctgggactaca
gctgggattata
GCTAGGATTACA
gctggaactaca
GCTGGGACCACA
gctgggaccaca
AGATCGCGC
AGATGGCGC
CTCACGCTTCCC
agcagtctgccc
GTCAGTCGGTCC
AGAAAAGAGAAC
GCCCCCGCCGC
gcctcagcctcc
gcctcagcctcc
GCCTCAGCCTCC
GCCTCAGCCTCC
gcctcggcctcc
acctcagcctcc
gcctctgcctcc
gcctcagtctcc
gcctcggcttcc
acctcagcttcc
CTCCCTCCTCAA
GAAAAGCAAACT
AGATTGCG
AGATCGCG
agtgagaccctc
AGGAAGCTGAGG
AGGCAGATCAC
AGGCGGATCAC
GGGAGGATCAC
