# Supplementary figures and images for: PHF12 regulates HDAC1 to promote tumorigenesis via EGFR/AKT signaling pathway in non-small cell lung cancer
Source: J Transl Med. 2024 Jul 29;22:689. doi: 10.1186/s12967-024-05488-x (PMC11287983; doi:10.1186/s12967-024-05488-x)

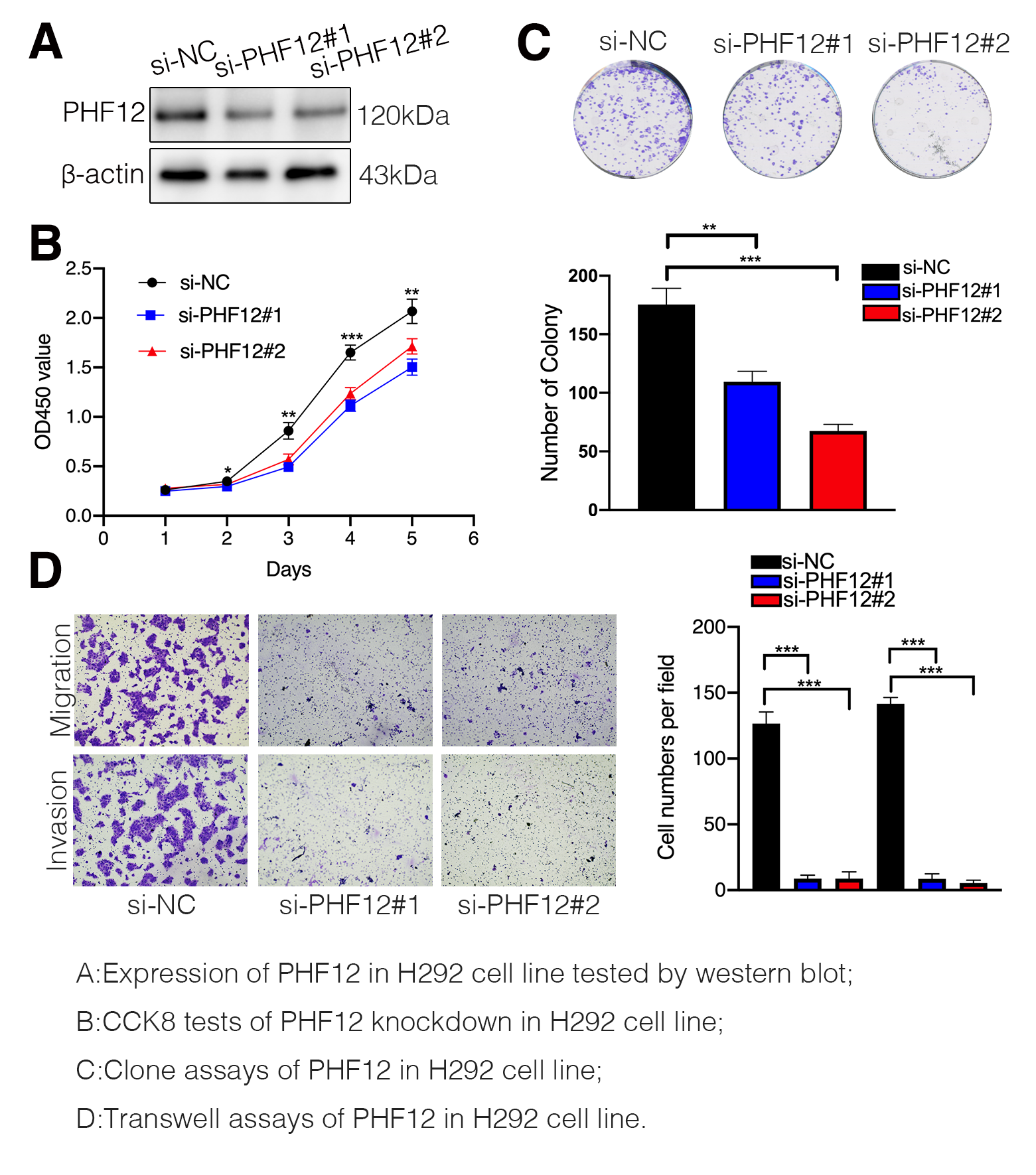

Supplement: Supplementary file 3 — Si-RNA experiments of H292 cell line This file contains the results of si-RNA experiments of H292 cell line. [file 12967_2024_5488_MOESM3_ESM.png]
