# Supplementary material for: PHF12 regulates HDAC1 to promote tumorigenesis via EGFR/AKT signaling pathway in non-small cell lung cancer
Source: J Transl Med. 2024 Jul 29;22:689. doi: 10.1186/s12967-024-05488-x (PMC11287983; doi:10.1186/s12967-024-05488-x)
Supplement: Supplementary file 1 — Resuls of ChIP-seq part1 This file contains the first part of the results of ChIP-seq generated during this research. [file 12967_2024_5488_MOESM1_ESM.pdf]

PeakID	chr	start	end	length	abs_summ	pileup	#NAME?	fold_enrich
Nc-1-IP_pe	chr1	11062	11385	324	11275	21	4.90204	2.59278
Nc-1-IP_pe	chr1	12195	13843	1649	12728	24	4.41955	2.3006
Nc-1-IP_pe	chr1	14104	14912	809	14788	28	7.18321	2.94535
Nc-1-IP_pe	chr1	16014	16273	260	16057	15	3.26922	2.27542
Nc-1-IP_pe	chr1	17116	17501	386	17324	25	6.28656	2.83671
Nc-1-IP_pe	chr1	19657	20436	780	19744	14	3.87746	2.60745
Nc-1-IP_pe	chr1	22421	22911	491	22439	13	3.35769	2.42919
Nc-1-IP_pe	chr1	25068	25562	495	25168	11	3.28475	2.53029
Nc-1-IP_pe	chr1	28978	29710	733	29141	29	18.6216	7.14745
Nc-1-IP_pe	chr1	32560	32818	259	32664	20	7.97282	3.87239
Nc-1-IP_pe	chr1	34747	35041	295	34839	18	6.15736	3.29676
Nc-1-IP_pe	chr1	37527	38105	579	37632	17	8.31233	4.43123
Nc-1-IP_pe	chr1	80811	81080	270	80920	11	5.77463	3.94557
Nc-1-IP_pe	chr1	129428	129704	277	129540	15	3.46204	2.35851
Nc-1-IP_pe	chr1	131308	131839	532	131401	17	7.02067	3.79543
Nc-1-IP_pe	chr1	133157	133555	399	133469	16	3.39261	2.27748
Nc-1-IP_pe	chr1	136576	136875	300	136806	22	4.80668	2.5094
Nc-1-IP_pe	chr1	137551	138140	590	138000	22	7.7403	3.56918
Nc-1-IP_pe	chr1	138448	138874	427	138778	20	4.68706	2.56965
Nc-1-IP_pe	chr1	169081	169514	434	169351	16	9.74731	5.37917
Nc-1-IP_pe	chr1	170231	170499	269	170352	13	6.92473	4.312
Nc-1-IP_pe	chr1	172201	172909	709	172763	14	6.69566	4.03025
Nc-1-IP_pe	chr1	175589	175844	256	175733	11	4.54404	3.2242
Nc-1-IP_pe	chr1	182877	184280	1404	183678	27	8.63021	3.46282
Nc-1-IP_pe	chr1	184681	185411	731	184783	24	6.14602	2.84828
Nc-1-IP_pe	chr1	187593	187847	255	187615	17	4.24737	2.5725
Nc-1-IP_pe	chr1	189468	190890	1423	189583	26	9.19542	3.73869
Nc-1-IP_pe	chr1	191338	191836	499	191465	24	8.65808	3.72951
Nc-1-IP_pe	chr1	192135	192443	309	192198	14	4.10345	2.71343
Nc-1-IP_pe	chr1	193009	193430	422	193076	13	3.72359	2.60585
Nc-1-IP_pe	chr1	195097	195566	470	195346	16	6.37501	3.60492
Nc-1-IP_pe	chr1	199221	200153	933	199680	25	8.31565	3.51608
Nc-1-IP_pe	chr1	259753	260147	395	259899	14	8.22927	4.89351
Nc-1-IP_pe	chr1	267315	267627	313	267376	26	5.56617	2.56496
Nc-1-IP_pe	chr1	280190	280546	357	280419	25	7.44236	3.21547
Nc-1-IP_pe	chr1	364167	365172	1006	364512	11	3.64839	2.72584
Nc-1-IP_pe	chr1	381002	382120	1119	381536	14	6.69566	4.03025
Nc-1-IP_pe	chr1	413981	414234	254	414131	13	7.7352	4.78626
Nc-1-IP_pe	chr1	434087	434552	466	434244	11	6.03439	4.10251
Nc-1-IP_pe	chr1	486957	487781	825	487730	13	5.29187	3.40559
Nc-1-IP_pe	chr1	490325	490600	276	490428	14	5.51541	3.4073
Nc-1-IP_pe	chr1	492812	493236	425	492876	12	4.24064	2.95299
Nc-1-IP_pe	chr1	493733	494115	383	493924	8	3.74505	3.07688
Nc-1-IP_pe	chr1	509484	509818	335	509702	15	5.66913	3.37372

Nc-1-IP_pe	chr1	511316	511693	378	511626	11	4.54404	3.2242
Nc-1-IP_pe	chr1	522437	522701	265	522527	13	6.62436	4.14003
Nc-1-IP_pe	chr1	525848	526273	426	525895	9	3.77975	2.99239
Nc-1-IP_pe	chr1	529087	529445	359	529271	22	7.7408	3.56938
Nc-1-IP_pe	chr1	530876	531335	460	530944	12	3.47963	2.55766
Nc-1-IP_pe	chr1	598276	598915	640	598834	20	4.71088	2.57831
Nc-1-IP_pe	chr1	599488	599864	377	599707	13	4.41954	2.952
Nc-1-IP_pe	chr1	603772	604604	833	603891	14	5.43414	3.36584
Nc-1-IP_pe	chr1	605292	605566	275	605409	19	4.8268	2.67939
Nc-1-IP_pe	chr1	606192	606519	328	606341	17	5.14803	2.94914
Nc-1-IP_pe	chr1	608193	611014	2822	610626	25	12.4232	5.11531
Nc-1-IP_pe	chr1	622773	623418	646	623355	12	4.53417	3.11004
Nc-1-IP_pe	chr1	649790	650167	378	650121	11	3.58722	2.69267
Nc-1-IP_pe	chr1	668341	669865	1525	669491	20	5.32999	2.80711
Nc-1-IP_pe	chr1	676778	677052	275	676836	11	4.06403	2.95415
Nc-1-IP_pe	chr1	726939	727440	502	727245	18	7.1522	3.73811
Nc-1-IP_pe	chr1	740747	741112	366	740923	13	7.7352	4.78626
Nc-1-IP_pe	chr1	780335	780798	464	780783	9	3.27326	2.68684
Nc-1-IP_pe	chr1	814721	815027	307	814784	11	6.03439	4.10251
Nc-1-IP_pe	chr1	822201	822480	280	822233	12	4.1728	2.91706
Nc-1-IP_pe	chr1	827701	828068	368	827955	21	10.7603	4.99737
Nc-1-IP_pe	chr1	836245	836547	303	836366	12	6.58417	4.27436
Nc-1-IP_pe	chr1	869879	870244	366	870046	17	11.4763	6.15376
Nc-1-IP_pe	chr1	899433	899825	393	899687	12	4.70082	3.20033
Nc-1-IP_pe	chr1	905594	906079	486	905773	14	6.13058	3.7272
Nc-1-IP_pe	chr1	910447	911052	606	910520	11	5.72344	3.91481
Nc-1-IP_pe	chr1	916261	916554	294	916362	12	5.61153	3.70794
Nc-1-IP_pe	chr1	922309	922806	498	922685	14	6.94277	4.16546
Nc-1-IP_pe	chr1	926075	926362	288	926198	12	5.45448	3.61875
Nc-1-IP_pe	chr1	926895	927309	415	926972	9	3.66792	2.92434
Nc-1-IP_pe	chr1	928224	928784	561	928313	8	2.99792	2.60557
Nc-1-IP_pe	chr1	932422	933006	585	932669	10	4.39571	3.25288
Nc-1-IP_pe	chr1	935326	936369	1044	935531	11	5.77463	3.94557
Nc-1-IP_pe	chr1	939843	940133	291	940001	15	8.26005	4.73146
Nc-1-IP_pe	chr1	945302	945670	369	945588	12	5.25962	3.50902
Nc-1-IP_pe	chr1	954084	954433	350	954250	10	4.68196	3.42446
Nc-1-IP_pe	chr1	954882	955277	396	955088	11	5.49185	3.77641
Nc-1-IP_pe	chr1	955793	956046	254	955937	10	5.08818	3.67138
Nc-1-IP_pe	chr1	959003	959866	864	959169	15	8.42791	4.82526
Nc-1-IP_pe	chr1	962916	963239	324	963069	15	5.66913	3.37372
Nc-1-IP_pe	chr1	967785	968059	275	967991	11	2.96397	2.36091
Nc-1-IP_pe	chr1	974513	974870	358	974862	9	3.74196	2.96936
Nc-1-IP_pe	chr1	980511	980898	388	980776	12	6.03452	3.95142
Nc-1-IP_pe	chr1	981710	982086	377	981763	9	4.20361	3.25317
Nc-1-IP_pe	chr1	982391	982904	514	982563	9	3.72192	2.95716

Nc-1-IP_pe	chr1	996397	997013	617	996590	12	4.24064	2.95299
Nc-1-IP_pe	chr1	997355	997634	280	997450	13	4.86362	3.18015
Nc-1-IP_pe	chr1	1006934	1007311	378	1007045	11	4.54404	3.2242
Nc-1-IP_pe	chr1	1007952	1008345	394	1007981	10	5.10552	3.682
Nc-1-IP_pe	chr1	1010024	1010434	411	1010253	14	7.90726	4.70771
Nc-1-IP_pe	chr1	1018242	1018779	538	1018404	13	6.59853	4.12535
Nc-1-IP_pe	chr1	1028544	1029197	654	1028627	10	4.34872	3.22492
Nc-1-IP_pe	chr1	1034536	1034887	352	1034685	12	6.86906	4.44438
Nc-1-IP_pe	chr1	1036346	1037117	772	1036798	9	4.46937	3.41876
Nc-1-IP_pe	chr1	1039093	1039355	263	1039250	10	5.08818	3.67138
Nc-1-IP_pe	chr1	1042918	1043232	315	1042946	9	4.04582	3.15559
Nc-1-IP_pe	chr1	1055342	1056009	668	1055595	15	8.06007	4.62056
Nc-1-IP_pe	chr1	1068287	1068703	417	1068481	15	6.19428	3.63445
Nc-1-IP_pe	chr1	1069585	1069887	303	1069705	12	4.51041	3.09723
Nc-1-IP_pe	chr1	1072671	1073238	568	1073128	14	4.21449	2.76599
Nc-1-IP_pe	chr1	1079037	1079403	367	1079152	17	4.78101	2.79343
Nc-1-IP_pe	chr1	1103846	1104314	469	1104052	11	4.54404	3.2242
Nc-1-IP_pe	chr1	1104664	1105124	461	1104984	8	3.74505	3.07688
Nc-1-IP_pe	chr1	1106636	1107218	583	1106685	8	3.08755	2.66145
Nc-1-IP_pe	chr1	1112467	1112851	385	1112575	9	2.89249	2.46179
Nc-1-IP_pe	chr1	1122940	1123212	273	1123074	9	3.12089	2.59629
Nc-1-IP_pe	chr1	1124263	1124748	486	1124394	10	4.12355	3.09176
Nc-1-IP_pe	chr1	1126085	1126514	430	1126223	11	4.73303	3.33236
Nc-1-IP_pe	chr1	1128989	1129679	691	1129134	10	4.33501	3.21677
Nc-1-IP_pe	chr1	1132064	1132476	413	1132268	15	7.77584	4.46458
Nc-1-IP_pe	chr1	1134275	1134538	264	1134389	15	7.46994	4.29894
Nc-1-IP_pe	chr1	1137022	1137316	295	1137157	12	5.15877	3.45264
Nc-1-IP_pe	chr1	1142491	1142767	277	1142662	14	7.40051	4.42001
Nc-1-IP_pe	chr1	1144816	1145119	304	1144900	21	7.72183	3.65936
Nc-1-IP_pe	chr1	1145608	1145910	303	1145759	15	4.7819	2.95039
Nc-1-IP_pe	chr1	1148529	1148885	357	1148640	15	6.78905	3.93887
Nc-1-IP_pe	chr1	1150430	1150698	269	1150526	11	4.11366	2.98176
Nc-1-IP_pe	chr1	1157745	1158098	354	1157958	15	8.29507	4.75097
Nc-1-IP_pe	chr1	1160294	1161911	1618	1160971	11	4.47871	3.18705
Nc-1-IP_pe	chr1	1164759	1165150	392	1164890	12	5.23082	3.49289
Nc-1-IP_pe	chr1	1171801	1172238	438	1171967	13	6.7109	4.18935
Nc-1-IP_pe	chr1	1173512	1173810	299	1173713	15	9.09658	5.20507
Nc-1-IP_pe	chr1	1177880	1178170	291	1177931	9	4.25966	3.28797
Nc-1-IP_pe	chr1	1180010	1180776	767	1180640	10	5.10552	3.682
Nc-1-IP_pe	chr1	1182404	1182752	349	1182550	13	7.70601	4.76894
Nc-1-IP_pe	chr1	1183764	1184049	286	1184024	10	3.08907	2.49869
Nc-1-IP_pe	chr1	1196769	1197206	438	1196946	9	4.46937	3.41876
Nc-1-IP_pe	chr1	1198300	1198567	268	1198419	9	2.89249	2.46179
Nc-1-IP_pe	chr1	1199562	1200207	646	1199676	13	5.9487	3.76157
Nc-1-IP_pe	chr1	1200555	1201252	698	1201111	14	7.42849	4.43574

Nc-1-IP_pe	chr1	1213234	1213811	578	1213362	11	5.52654	3.79706
Nc-1-IP_pe	chr1	1214144	1214437	294	1214255	12	5.23082	3.49289
Nc-1-IP_pe	chr1	1216296	1216720	425	1216488	12	5.23082	3.49289
Nc-1-IP_pe	chr1	1221879	1222475	597	1222213	9	3.88314	3.0556
Nc-1-IP_pe	chr1	1223516	1224276	761	1224104	13	7.00513	4.3584
Nc-1-IP_pe	chr1	1226716	1227267	552	1226844	12	5.23082	3.49289
Nc-1-IP_pe	chr1	1228783	1229297	515	1228843	9	3.43499	2.78365
Nc-1-IP_pe	chr1	1232613	1233602	990	1233374	17	8.82358	4.69439
Nc-1-IP_pe	chr1	1243555	1243849	295	1243705	11	5.16212	3.58161
Nc-1-IP_pe	chr1	1249229	1249602	374	1249589	10	4.99888	3.61677
Nc-1-IP_pe	chr1	1252574	1253106	533	1252736	10	5.23357	3.76063
Nc-1-IP_pe	chr1	1264093	1264350	258	1264191	12	6.86906	4.44438
Nc-1-IP_pe	chr1	1267632	1268643	1012	1268475	11	4.54404	3.2242
Nc-1-IP_pe	chr1	1270365	1271076	712	1270539	10	5.08818	3.67138
Nc-1-IP_pe	chr1	1273510	1273770	261	1273656	15	5.66913	3.37372
Nc-1-IP_pe	chr1	1274204	1275321	1118	1274372	16	6.8987	3.86161
Nc-1-IP_pe	chr1	1284546	1285107	562	1284648	11	4.54404	3.2242
Nc-1-IP_pe	chr1	1288225	1288963	739	1288404	12	5.72541	3.77304
Nc-1-IP_pe	chr1	1289225	1289648	424	1289298	15	7.46994	4.29894
Nc-1-IP_pe	chr1	1290144	1290572	429	1290361	13	4.41954	2.952
Nc-1-IP_pe	chr1	1291382	1292162	781	1291513	12	3.47963	2.55766
Nc-1-IP_pe	chr1	1297132	1298033	902	1297928	11	5.52654	3.79706
Nc-1-IP_pe	chr1	1298610	1299407	798	1299105	11	4.54404	3.2242
Nc-1-IP_pe	chr1	1303877	1304310	434	1304061	12	4.83276	3.2724
Nc-1-IP_pe	chr1	1309412	1310080	669	1309869	13	5.86991	3.71823
Nc-1-IP_pe	chr1	1311777	1312178	402	1312070	10	4.53273	3.33475
Nc-1-IP_pe	chr1	1312615	1312928	314	1312878	10	4.82246	3.50943
Nc-1-IP_pe	chr1	1313438	1313739	302	1313662	10	5.08818	3.67138
Nc-1-IP_pe	chr1	1314276	1315479	1204	1315104	10	4.99888	3.61677
Nc-1-IP_pe	chr1	1324236	1325153	918	1324434	14	7.73235	4.60774
Nc-1-IP_pe	chr1	1325684	1326165	482	1325720	13	5.36853	3.44651
Nc-1-IP_pe	chr1	1327396	1327914	519	1327560	13	6.67312	4.1678
Nc-1-IP_pe	chr1	1330379	1330996	618	1330657	13	5.36853	3.44651
Nc-1-IP_pe	chr1	1331339	1332189	851	1331503	13	6.21	3.90651
Nc-1-IP_pe	chr1	1333283	1333581	299	1333394	13	4.86362	3.18015
Nc-1-IP_pe	chr1	1336598	1337561	964	1336900	13	5.36853	3.44651
Nc-1-IP_pe	chr1	1339657	1339963	307	1339792	14	6.79972	4.087
Nc-1-IP_pe	chr1	1341555	1342228	674	1341764	14	6.14575	3.73522
Nc-1-IP_pe	chr1	1343685	1343966	282	1343769	14	6.0854	3.70334
Nc-1-IP_pe	chr1	1352333	1352771	439	1352401	11	3.89191	2.85898
Nc-1-IP_pe	chr1	1359451	1360354	904	1360231	14	8.18196	4.86608
Nc-1-IP_pe	chr1	1372182	1372751	570	1372356	15	8.38325	4.80024
Nc-1-IP_pe	chr1	1374241	1374619	379	1374547	10	3.94999	2.99009
Nc-1-IP_pe	chr1	1374955	1375447	493	1375202	21	10.8379	5.03418
Nc-1-IP_pe	chr1	1376328	1376645	318	1376568	11	4.61014	3.26192

Nc-1-IP_pe	chr1	1380550	1381086	537	1380723	14	8.1351	4.83895
Nc-1-IP_pe	chr1	1382377	1382758	382	1382663	23	5.5873	2.71945
Nc-1-IP_pe	chr1	1399998	1400251	254	1400073	15	5.58327	3.33181
Nc-1-IP_pe	chr1	1400889	1401526	638	1400986	11	5.40654	3.72575
Nc-1-IP_pe	chr1	1402716	1403052	337	1402828	10	4.39571	3.25288
Nc-1-IP_pe	chr1	1407123	1407776	654	1407241	12	4.75569	3.23024
Nc-1-IP_pe	chr1	1411436	1411944	509	1411903	10	5.23357	3.76063
Nc-1-IP_pe	chr1	1429823	1430114	292	1429907	14	7.40051	4.42001
Nc-1-IP_pe	chr1	1432268	1432578	311	1432418	12	3.47963	2.55766
Nc-1-IP_pe	chr1	1433990	1434482	493	1434254	13	6.19316	3.89711
Nc-1-IP_pe	chr1	1436472	1437091	620	1436474	10	3.94999	2.99009
Nc-1-IP_pe	chr1	1440951	1441811	861	1441072	12	4.70082	3.20033
Nc-1-IP_pe	chr1	1442376	1442695	320	1442451	10	3.4604	2.70797
Nc-1-IP_pe	chr1	1444491	1445055	565	1445018	12	5.84943	3.84431
Nc-1-IP_pe	chr1	1454160	1454520	361	1454334	10	4.99888	3.61677
Nc-1-IP_pe	chr1	1455600	1456009	410	1455903	9	4.4496	3.40639
Nc-1-IP_pe	chr1	1460387	1460656	270	1460567	14	6.69566	4.03025
Nc-1-IP_pe	chr1	1463917	1464203	287	1463948	8	3.58997	2.97809
Nc-1-IP_pe	chr1	1467197	1467923	727	1467396	9	4.46937	3.41876
Nc-1-IP_pe	chr1	1468599	1468964	366	1468822	10	5.23357	3.76063
Nc-1-IP_pe	chr1	1470757	1471082	326	1470857	9	4.46937	3.41876
Nc-1-IP_pe	chr1	1471455	1471883	429	1471811	15	9.19325	5.26076
Nc-1-IP_pe	chr1	1474579	1475101	523	1475006	11	6.03439	4.10251
Nc-1-IP_pe	chr1	1475633	1476174	542	1475795	10	5.23357	3.76063
Nc-1-IP_pe	chr1	1482249	1482566	318	1482469	10	5.23357	3.76063
Nc-1-IP_pe	chr1	1486181	1486676	496	1486273	13	4.86362	3.18015
Nc-1-IP_pe	chr1	1488913	1489627	715	1489450	12	6.86906	4.44438
Nc-1-IP_pe	chr1	1490689	1491120	432	1490976	16	6.8987	3.86161
Nc-1-IP_pe	chr1	1499779	1500044	266	1499887	10	4.39571	3.25288
Nc-1-IP_pe	chr1	1503339	1503772	434	1503671	10	4.39571	3.25288
Nc-1-IP_pe	chr1	1508868	1509124	257	1508975	14	4.21449	2.76599
Nc-1-IP_pe	chr1	1512300	1512875	576	1512702	15	8.92134	5.10462
Nc-1-IP_pe	chr1	1514313	1514747	435	1514501	13	7.27693	4.51635
Nc-1-IP_pe	chr1	1518878	1519515	638	1519079	20	13.6099	6.66308
Nc-1-IP_pe	chr1	1520118	1520420	303	1520313	10	3.89064	2.95552
Nc-1-IP_pe	chr1	1523141	1523488	348	1523464	9	3.72192	2.95716
Nc-1-IP_pe	chr1	1524341	1524641	301	1524365	9	3.54889	2.85226
Nc-1-IP_pe	chr1	1528594	1529753	1160	1529036	11	5.83542	3.98217
Nc-1-IP_pe	chr1	1533323	1533622	300	1533475	12	5.77527	3.80164
Nc-1-IP_pe	chr1	1536023	1536433	411	1536054	11	5.10577	3.5486
Nc-1-IP_pe	chr1	1538552	1538909	358	1538644	9	4.46937	3.41876
Nc-1-IP_pe	chr1	1540715	1541055	341	1540967	11	5.76039	3.93701
Nc-1-IP_pe	chr1	1543455	1544143	689	1543701	13	4.86362	3.18015
Nc-1-IP_pe	chr1	1546311	1546645	335	1546427	12	6.86906	4.44438
Nc-1-IP_pe	chr1	1551117	1551842	726	1551630	9	4.4496	3.40639

Nc-1-IP_pe	chr1	1553794	1554449	656	1554241	12	4.70082	3.20033
Nc-1-IP_pe	chr1	1561553	1561899	347	1561615	9	4.46937	3.41876
Nc-1-IP_pe	chr1	1563423	1563891	469	1563744	10	5.23357	3.76063
Nc-1-IP_pe	chr1	1564377	1564852	476	1564388	8	3.74505	3.07688
Nc-1-IP_pe	chr1	1574252	1575445	1194	1574958	17	7.02067	3.79543
Nc-1-IP_pe	chr1	1584365	1584781	417	1584629	8	3.74505	3.07688
Nc-1-IP_pe	chr1	1593859	1594241	383	1594078	8	3.74505	3.07688
Nc-1-IP_pe	chr1	1597768	1598104	337	1597896	8	3.51085	2.92785
Nc-1-IP_pe	chr1	1600530	1600809	280	1600586	10	4.90322	3.55848
Nc-1-IP_pe	chr1	1603403	1603693	291	1603450	10	4.79071	3.49018
Nc-1-IP_pe	chr1	1604843	1605145	303	1604920	8	3.74505	3.07688
Nc-1-IP_pe	chr1	1605855	1606940	1086	1606049	13	3.67377	2.58159
Nc-1-IP_pe	chr1	1609341	1609682	342	1609559	11	6.03439	4.10251
Nc-1-IP_pe	chr1	1613428	1613707	280	1613519	12	6.35031	4.1361
Nc-1-IP_pe	chr1	1615555	1616091	537	1615622	13	4.41954	2.952
Nc-1-IP_pe	chr1	1616364	1617010	647	1616465	14	7.58433	4.52368
Nc-1-IP_pe	chr1	1618506	1618762	257	1618710	10	4.60647	3.37901
Nc-1-IP_pe	chr1	1621902	1622209	308	1622105	11	5.93136	4.04009
Nc-1-IP_pe	chr1	1622776	1623171	396	1622828	8	3.56021	2.95918
Nc-1-IP_pe	chr1	1624849	1625235	387	1624932	8	3.58997	2.97809
Nc-1-IP_pe	chr1	1625636	1626751	1116	1625707	10	5.23357	3.76063
Nc-1-IP_pe	chr1	1627472	1628256	785	1627634	11	3.64839	2.72584
Nc-1-IP_pe	chr1	1630539	1632649	2111	1632423	11	6.03439	4.10251
Nc-1-IP_pe	chr1	1633026	1633696	671	1633297	19	10.8111	5.37367
Nc-1-IP_pe	chr1	1639557	1640451	895	1639717	12	6.6717	4.32642
Nc-1-IP_pe	chr1	1645796	1646070	275	1645947	11	5.66869	3.88198
Nc-1-IP_pe	chr1	1652999	1653539	541	1653100	10	5.23357	3.76063
Nc-1-IP_pe	chr1	1662953	1663384	432	1663363	11	3.64839	2.72584
Nc-1-IP_pe	chr1	1666565	1666932	368	1666646	13	6.62436	4.14003
Nc-1-IP_pe	chr1	1671058	1671333	276	1671195	11	6.03439	4.10251
Nc-1-IP_pe	chr1	1672949	1673228	280	1673171	9	4.46937	3.41876
Nc-1-IP_pe	chr1	1674651	1675025	375	1674961	10	4.47495	3.30017
Nc-1-IP_pe	chr1	1676207	1676630	424	1676374	12	5.23082	3.49289
Nc-1-IP_pe	chr1	1678482	1678740	259	1678669	9	3.92293	3.08
Nc-1-IP_pe	chr1	1685690	1686127	438	1685897	9	4.25966	3.28797
Nc-1-IP_pe	chr1	1697637	1697941	305	1697787	11	4.35236	3.11555
Nc-1-IP_pe	chr1	1699277	1699640	364	1699278	9	2.89249	2.46179
Nc-1-IP_pe	chr1	1700217	1701663	1447	1700516	16	8.26996	4.56762
Nc-1-IP_pe	chr1	1706651	1707285	635	1706751	12	6.44808	4.19375
Nc-1-IP_pe	chr1	1707895	1708196	302	1708062	11	4.54404	3.2242
Nc-1-IP_pe	chr1	1709492	1709775	284	1709711	13	6.36468	3.99316
Nc-1-IP_pe	chr1	1739323	1739598	276	1739475	9	2.89249	2.46179
Nc-1-IP_pe	chr1	1745657	1746026	370	1745902	12	6.31185	4.11348
Nc-1-IP_pe	chr1	1755479	1755992	514	1755830	13	7.02546	4.37016
Nc-1-IP_pe	chr1	1756670	1756949	280	1756823	16	9.06204	4.99663

Nc-1-IP_pe	chr1	1769209	1769517	309	1769447	9	4.46937	3.41876
Nc-1-IP_pe	chr1	1782352	1782982	631	1782496	14	6.06519	3.69269
Nc-1-IP_pe	chr1	1793268	1793903	636	1793836	9	3.72192	2.95716
Nc-1-IP_pe	chr1	1817909	1818181	273	1818088	10	5.23357	3.76063
Nc-1-IP_pe	chr1	1841186	1841511	326	1841379	9	4.25966	3.28797
Nc-1-IP_pe	chr1	1842025	1842297	273	1842141	10	5.23357	3.76063
Nc-1-IP_pe	chr1	1843010	1843339	330	1843088	7	2.9043	2.63038
Nc-1-IP_pe	chr1	1850987	1851387	401	1851150	10	5.23357	3.76063
Nc-1-IP_pe	chr1	1892075	1892476	402	1892218	17	9.09431	4.8363
Nc-1-IP_pe	chr1	1894780	1895566	787	1895220	11	4.74697	3.34038
Nc-1-IP_pe	chr1	1896412	1897054	643	1896828	12	5.23082	3.49289
Nc-1-IP_pe	chr1	1897597	1898028	432	1897999	9	4.40188	3.37657
Nc-1-IP_pe	chr1	1900415	1900669	255	1900484	8	3.74505	3.07688
Nc-1-IP_pe	chr1	1901293	1901853	561	1901777	12	4.70082	3.20033
Nc-1-IP_pe	chr1	1907761	1908376	616	1908009	10	4.47495	3.30017
Nc-1-IP_pe	chr1	1908718	1908982	265	1908750	10	3.83201	2.92147
Nc-1-IP_pe	chr1	1918294	1918553	260	1918381	13	5.9487	3.76157
Nc-1-IP_pe	chr1	1925619	1926301	683	1926101	12	6.86906	4.44438
Nc-1-IP_pe	chr1	1929636	1930009	374	1929702	11	4.54404	3.2242
Nc-1-IP_pe	chr1	1938737	1938992	256	1938834	10	5.23357	3.76063
Nc-1-IP_pe	chr1	1944497	1944930	434	1944788	11	6.03439	4.10251
Nc-1-IP_pe	chr1	1946373	1946923	551	1946528	11	6.03439	4.10251
Nc-1-IP_pe	chr1	1957761	1958340	580	1958278	9	2.89249	2.46179
Nc-1-IP_pe	chr1	1959922	1960252	331	1960120	10	3.89064	2.95552
Nc-1-IP_pe	chr1	1961949	1962222	274	1962044	12	6.58417	4.27436
Nc-1-IP_pe	chr1	1963501	1963846	346	1963688	11	5.76039	3.93701
Nc-1-IP_pe	chr1	1970280	1971022	743	1970925	10	5.07094	3.66082
Nc-1-IP_pe	chr1	1975917	1976297	381	1976257	10	4.79071	3.49018
Nc-1-IP_pe	chr1	1978791	1979089	299	1979034	11	5.50915	3.78671
Nc-1-IP_pe	chr1	1980665	1981198	534	1981061	10	4.48929	3.30875
Nc-1-IP_pe	chr1	2000324	2000596	273	2000459	10	5.17577	3.72509
Nc-1-IP_pe	chr1	2028048	2028359	312	2028286	8	3.74505	3.07688
Nc-1-IP_pe	chr1	2029702	2030589	888	2030463	10	5.23357	3.76063
Nc-1-IP_pe	chr1	2037612	2037952	341	2037726	10	3.89064	2.95552
Nc-1-IP_pe	chr1	2043269	2043610	342	2043356	13	5.57038	3.55505
Nc-1-IP_pe	chr1	2044664	2045038	375	2044799	14	7.38052	4.40879
Nc-1-IP_pe	chr1	2051305	2051856	552	2051720	10	3.89064	2.95552
Nc-1-IP_pe	chr1	2053682	2054006	325	2053848	11	5.49185	3.77641
Nc-1-IP_pe	chr1	2056241	2056635	395	2056437	12	5.23082	3.49289
Nc-1-IP_pe	chr1	2059390	2060702	1313	2060481	12	6.86906	4.44438
Nc-1-IP_pe	chr1	2067509	2067998	490	2067634	9	4.46937	3.41876
Nc-1-IP_pe	chr1	2075167	2075428	262	2075375	10	4.99888	3.61677
Nc-1-IP_pe	chr1	2076390	2077025	636	2076538	11	6.03439	4.10251
Nc-1-IP_pe	chr1	2080588	2080879	292	2080636	9	4.46937	3.41876
Nc-1-IP_pe	chr1	2089220	2089513	294	2089363	11	6.03439	4.10251

Nc-1-IP_pe	chr1	2091916	2092270	355	2091999	10	4.99888	3.61677
Nc-1-IP_pe	chr1	2094218	2094658	441	2094433	15	9.35725	5.35563
Nc-1-IP_pe	chr1	2101933	2102221	289	2102057	8	3.264	2.772
Nc-1-IP_pe	chr1	2106388	2106820	433	2106726	14	7.42849	4.43574
Nc-1-IP_pe	chr1	2113655	2114022	368	2113898	11	4.54404	3.2242
Nc-1-IP_pe	chr1	2126988	2127316	329	2127088	12	4.70082	3.20033
Nc-1-IP_pe	chr1	2134995	2135321	327	2135114	14	7.62616	4.54739
Nc-1-IP_pe	chr1	2137241	2137539	299	2137310	10	3.4604	2.70797
Nc-1-IP_pe	chr1	2161076	2161759	684	2161597	12	6.86906	4.44438
Nc-1-IP_pe	chr1	2173159	2173730	572	2173577	13	5.36853	3.44651
Nc-1-IP_pe	chr1	2174278	2174726	449	2174458	10	4.39571	3.25288
Nc-1-IP_pe	chr1	2177477	2177955	479	2177543	11	5.10577	3.5486
Nc-1-IP_pe	chr1	2188271	2188555	285	2188515	9	3.9633	3.10479
Nc-1-IP_pe	chr1	2192677	2193143	467	2193093	10	4.51818	3.32604
Nc-1-IP_pe	chr1	2193853	2194119	267	2194049	11	5.03835	3.5092
Nc-1-IP_pe	chr1	2198486	2199138	653	2198901	14	6.06519	3.69269
Nc-1-IP_pe	chr1	2200657	2200919	263	2200691	10	4.62142	3.388
Nc-1-IP_pe	chr1	2205610	2205884	275	2205783	11	4.35236	3.11555
Nc-1-IP_pe	chr1	2207355	2207624	270	2207439	12	6.31185	4.11348
Nc-1-IP_pe	chr1	2213878	2214372	495	2214057	13	6.54342	4.09407
Nc-1-IP_pe	chr1	2214825	2215319	495	2214880	11	4.06403	2.95415
Nc-1-IP_pe	chr1	2220615	2221114	500	2220822	9	4.25966	3.28797
Nc-1-IP_pe	chr1	2230132	2230419	288	2230331	9	3.12089	2.59629
Nc-1-IP_pe	chr1	2235266	2235539	274	2235428	9	3.53727	2.84525
Nc-1-IP_pe	chr1	2237041	2237308	268	2237119	11	4.61014	3.26192
Nc-1-IP_pe	chr1	2238026	2238458	433	2238267	15	6.78905	3.93887
Nc-1-IP_pe	chr1	2258829	2259265	437	2258850	8	3.64414	3.01254
Nc-1-IP_pe	chr1	2279012	2279331	320	2279167	13	4.41954	2.952
Nc-1-IP_pe	chr1	2280630	2281138	509	2281101	11	5.77463	3.94557
Nc-1-IP_pe	chr1	2283333	2283766	434	2283459	11	5.10577	3.5486
Nc-1-IP_pe	chr1	2284310	2284707	398	2284533	9	4.21839	3.26234
Nc-1-IP_pe	chr1	2291072	2291395	324	2291376	10	4.09813	3.07682
Nc-1-IP_pe	chr1	2292834	2293371	538	2292847	10	3.94999	2.99009
Nc-1-IP_pe	chr1	2294363	2294925	563	2294751	13	6.14303	3.86919
Nc-1-IP_pe	chr1	2298876	2299141	266	2298945	11	4.35236	3.11555
Nc-1-IP_pe	chr1	2304061	2304461	401	2304183	8	3.48502	2.91148
Nc-1-IP_pe	chr1	2305974	2306761	788	2306200	13	7.34205	4.55444
Nc-1-IP_pe	chr1	2307279	2307650	372	2307511	12	6.50787	4.22912
Nc-1-IP_pe	chr1	2310214	2310735	522	2310584	13	5.86991	3.71823
Nc-1-IP_pe	chr1	2313878	2314359	482	2314010	12	4.24064	2.95299
Nc-1-IP_pe	chr1	2317799	2318096	298	2317931	12	6.29276	4.10227
Nc-1-IP_pe	chr1	2322678	2323103	426	2323061	10	3.71867	2.85592
Nc-1-IP_pe	chr1	2324473	2324774	302	2324664	12	5.23082	3.49289
Nc-1-IP_pe	chr1	2333017	2333545	529	2333318	11	5.10577	3.5486
Nc-1-IP_pe	chr1	2335173	2335741	569	2335283	10	4.39571	3.25288

Nc-1-IP_pe	chr1	2339172	2339543	372	2339341	7	2.88417	2.61725
Nc-1-IP_pe	chr1	2339971	2340238	268	2340146	10	5.10552	3.682
Nc-1-IP_pe	chr1	2345594	2345967	374	2345903	8	3.56021	2.95918
Nc-1-IP_pe	chr1	2352734	2353178	445	2353131	11	4.35236	3.11555
Nc-1-IP_pe	chr1	2355313	2355670	358	2355599	11	5.77463	3.94557
Nc-1-IP_pe	chr1	2356359	2356622	264	2356515	8	3.74505	3.07688
Nc-1-IP_pe	chr1	2357014	2357322	309	2357153	11	6.03439	4.10251
Nc-1-IP_pe	chr1	2368315	2368590	276	2368331	8	3.56021	2.95918
Nc-1-IP_pe	chr1	2371553	2372432	880	2371719	10	5.23357	3.76063
Nc-1-IP_pe	chr1	2375317	2376276	960	2375710	13	7.7352	4.78626
Nc-1-IP_pe	chr1	2376935	2377844	910	2377089	11	6.03439	4.10251
Nc-1-IP_pe	chr1	2378204	2378471	268	2378352	13	7.7352	4.78626
Nc-1-IP_pe	chr1	2380901	2381258	358	2381179	11	4.54404	3.2242
Nc-1-IP_pe	chr1	2382250	2383344	1095	2383224	14	8.32526	4.94932
Nc-1-IP_pe	chr1	2384626	2385497	872	2385302	11	5.77463	3.94557
Nc-1-IP_pe	chr1	2387590	2388118	529	2388027	9	4.07387	3.17289
Nc-1-IP_pe	chr1	2390927	2391505	579	2391322	13	4.86362	3.18015
Nc-1-IP_pe	chr1	2391903	2392172	270	2392135	9	4.15971	3.22596
Nc-1-IP_pe	chr1	2393657	2393926	270	2393777	14	6.78215	4.0774
Nc-1-IP_pe	chr1	2395341	2395599	259	2395426	14	6.69566	4.03025
Nc-1-IP_pe	chr1	2411571	2412087	517	2411875	10	3.4604	2.70797
Nc-1-IP_pe	chr1	2414298	2414727	430	2414508	9	3.90961	3.07183
Nc-1-IP_pe	chr1	2418409	2418732	324	2418515	11	5.93136	4.04009
Nc-1-IP_pe	chr1	2427035	2427500	466	2427414	10	5.23357	3.76063
Nc-1-IP_pe	chr1	2429018	2429826	809	2429163	12	4.24064	2.95299
Nc-1-IP_pe	chr1	2431483	2432095	613	2431817	10	4.98602	3.60892
Nc-1-IP_pe	chr1	2442892	2443350	459	2443065	12	5.61153	3.70794
Nc-1-IP_pe	chr1	2444260	2445109	850	2444964	12	5.62759	3.7171
Nc-1-IP_pe	chr1	2446481	2446771	291	2446630	10	4.21426	3.14524
Nc-1-IP_pe	chr1	2453697	2454053	357	2453831	13	6.26093	3.93497
Nc-1-IP_pe	chr1	2454677	2455161	485	2455021	14	6.69566	4.03025
Nc-1-IP_pe	chr1	2459630	2460747	1118	2460561	12	6.07041	3.97229
Nc-1-IP_pe	chr1	2463655	2463923	269	2463788	9	3.83096	3.02367
Nc-1-IP_pe	chr1	2467899	2468177	279	2467970	13	6.36468	3.99316
Nc-1-IP_pe	chr1	2469174	2469847	674	2469847	10	3.08907	2.49869
Nc-1-IP_pe	chr1	2473129	2473775	647	2473325	11	5.10577	3.5486
Nc-1-IP_pe	chr1	2474084	2474356	273	2474112	10	4.66672	3.41527
Nc-1-IP_pe	chr1	2475676	2475963	288	2475945	10	3.03453	2.46828
Nc-1-IP_pe	chr1	2477858	2478535	678	2478336	13	6.39984	4.01295
Nc-1-IP_pe	chr1	2480666	2481579	914	2481526	10	4.63645	3.39704
Nc-1-IP_pe	chr1	2483779	2484058	280	2483853	9	4.20361	3.25317
Nc-1-IP_pe	chr1	2485107	2485563	457	2485466	12	6.50787	4.22912
Nc-1-IP_pe	chr1	2486015	2486442	428	2486111	8	3.74505	3.07688
Nc-1-IP_pe	chr1	2492944	2493592	649	2493369	12	5.23082	3.49289
Nc-1-IP_pe	chr1	2494851	2495207	357	2495052	14	5.51541	3.4073

Nc-1-IP_pe	chr1	2498741	2499447	707	2498960	10	4.95265	3.58858
Nc-1-IP_pe	chr1	2503605	2504023	419	2503713	9	3.99054	3.12154
Nc-1-IP_pe	chr1	2511489	2511973	485	2511551	10	4.39571	3.25288
Nc-1-IP_pe	chr1	2512742	2514259	1518	2513743	11	6.03439	4.10251
Nc-1-IP_pe	chr1	2519629	2519993	365	2519826	16	10.2925	5.69036
Nc-1-IP_pe	chr1	2521427	2521973	547	2521761	16	9.49293	5.236
Nc-1-IP_pe	chr1	2529708	2530020	313	2529973	10	3.77478	2.88832
Nc-1-IP_pe	chr1	2530674	2531050	377	2530843	13	5.77769	3.66773
Nc-1-IP_pe	chr1	2535634	2536004	371	2535967	12	5.07448	3.40574
Nc-1-IP_pe	chr1	2539176	2539500	325	2539342	14	6.49348	3.92083
Nc-1-IP_pe	chr1	2542676	2543017	342	2542873	11	4.59676	3.25427
Nc-1-IP_pe	chr1	2543456	2543860	405	2543510	12	5.23082	3.49289
Nc-1-IP_pe	chr1	2544148	2544468	321	2544388	13	4.86362	3.18015
Nc-1-IP_pe	chr1	2552772	2553959	1188	2553152	10	4.20113	3.13749
Nc-1-IP_pe	chr1	2555873	2556206	334	2556117	12	5.84943	3.84431
Nc-1-IP_pe	chr1	2558135	2558730	596	2558223	11	5.56156	3.81794
Nc-1-IP_pe	chr1	2562454	2562748	295	2562515	10	4.93609	3.57849
Nc-1-IP_pe	chr1	2564894	2565227	334	2565147	10	4.39571	3.25288
Nc-1-IP_pe	chr1	2566178	2566614	437	2566329	9	4.20361	3.25317
Nc-1-IP_pe	chr1	2567847	2568198	352	2567924	12	5.45448	3.61875
Nc-1-IP_pe	chr1	2575953	2576487	535	2576336	9	3.72192	2.95716
Nc-1-IP_pe	chr1	2579371	2579852	482	2579744	8	3.51085	2.92785
Nc-1-IP_pe	chr1	2583096	2583709	614	2583506	11	4.88957	3.42271
Nc-1-IP_pe	chr1	2585774	2586336	563	2585897	10	4.44645	3.28314
Nc-1-IP_pe	chr1	2586995	2587270	276	2587218	11	3.40056	2.59215
Nc-1-IP_pe	chr1	2588109	2588448	340	2588230	9	3.66792	2.92434
Nc-1-IP_pe	chr1	2591740	2592215	476	2592025	9	2.89249	2.46179
Nc-1-IP_pe	chr1	2593010	2594380	1371	2593208	12	6.58417	4.27436
Nc-1-IP_pe	chr1	2596450	2597062	613	2596511	9	4.25966	3.28797
Nc-1-IP_pe	chr1	2606989	2607560	572	2607116	12	5.84943	3.84431
Nc-1-IP_pe	chr1	2611593	2611970	378	2611748	11	6.03439	4.10251
Nc-1-IP_pe	chr1	2612878	2613272	395	2612915	8	3.64414	3.01254
Nc-1-IP_pe	chr1	2619255	2619510	256	2619443	11	6.03439	4.10251
Nc-1-IP_pe	chr1	2625302	2625649	348	2625538	12	6.31185	4.11348
Nc-1-IP_pe	chr1	2634073	2634662	590	2634498	11	6.03439	4.10251
Nc-1-IP_pe	chr1	2640452	2640772	321	2640688	9	4.14522	3.217
Nc-1-IP_pe	chr1	2641314	2641908	595	2641652	11	4.06403	2.95415
Nc-1-IP_pe	chr1	2642240	2643567	1328	2643174	14	5.51541	3.4073
Nc-1-IP_pe	chr1	2652144	2655087	2944	2653316	260	56.2573	3.19716
Nc-1-IP_pe	chr1	2655357	2656470	1114	2655958	115	20.9334	2.7509
Nc-1-IP_pe	chr1	2659749	2660212	464	2660060	19	3.71826	2.26621
Nc-1-IP_pe	chr1	2665841	2666103	263	2665936	34	7.35963	2.71148
Nc-1-IP_pe	chr1	2680996	2681408	413	2681089	64	20.9169	4.07009
Nc-1-IP_pe	chr1	2683352	2684454	1103	2684131	83	16.4471	2.83981
Nc-1-IP_pe	chr1	2684766	2685251	486	2684948	38	15.1596	4.53257

Nc-1-IP_pe	chr1	2687828	2688367	540	2688232	43	7.45691	2.44287
Nc-1-IP_pe	chr1	2690288	2690921	634	2690517	32	9.40838	3.35161
Nc-1-IP_pe	chr1	2693512	2693840	329	2693751	17	5.9911	3.31919
Nc-1-IP_pe	chr1	2696414	2697233	820	2696630	22	11.6971	5.27343
Nc-1-IP_pe	chr1	2697590	2697864	275	2697704	21	5.88587	2.94733
Nc-1-IP_pe	chr1	2699136	2699611	476	2699285	18	8.10208	4.1823
Nc-1-IP_pe	chr1	2701080	2701361	282	2701129	19	6.78661	3.47027
Nc-1-IP_pe	chr1	2748369	2748902	534	2748547	41	5.79468	2.16802
Nc-1-IP_pe	chr1	2750033	2751919	1887	2751747	73	16.7824	3.10983
Nc-1-IP_pe	chr1	2752298	2753457	1160	2752419	59	13.561	3.04372
Nc-1-IP_pe	chr1	2754690	2755299	610	2754831	53	14.6606	3.45491
Nc-1-IP_pe	chr1	2756546	2757645	1100	2757307	45	14.6567	3.86964
Nc-1-IP_pe	chr1	2758072	2758836	765	2758166	30	8.26892	3.14848
Nc-1-IP_pe	chr1	2759123	2760285	1163	2759594	27	6.99773	2.94558
Nc-1-IP_pe	chr1	2762361	2762673	313	2762615	14	4.742	3.02031
Nc-1-IP_pe	chr1	2763396	2763770	375	2763583	16	6.33285	3.58457
Nc-1-IP_pe	chr1	2764035	2765493	1459	2764422	16	6.24025	3.54005
Nc-1-IP_pe	chr1	2767784	2768352	569	2768061	15	4.7819	2.95039
Nc-1-IP_pe	chr1	2772315	2772621	307	2772617	15	3.00739	2.16374
Nc-1-IP_pe	chr1	2772912	2773978	1067	2773070	57	16.5832	3.63177
Nc-1-IP_pe	chr1	2785511	2785767	257	2785689	9	3.72192	2.95716
Nc-1-IP_pe	chr1	2787425	2787797	373	2787628	9	3.92293	3.08
Nc-1-IP_pe	chr1	2801786	2802377	592	2801984	14	8.63073	5.12814
Nc-1-IP_pe	chr1	2806165	2806491	327	2806420	9	3.27326	2.68684
Nc-1-IP_pe	chr1	2807603	2808149	547	2808028	10	5.14046	3.70342
Nc-1-IP_pe	chr1	2809028	2809281	254	2809250	10	3.4604	2.70797
Nc-1-IP_pe	chr1	2819715	2820188	474	2819840	8	3.74505	3.07688
Nc-1-IP_pe	chr1	2821184	2821514	331	2821396	11	5.10577	3.5486
Nc-1-IP_pe	chr1	2825073	2825520	448	2825174	11	6.03439	4.10251
Nc-1-IP_pe	chr1	2826802	2827329	528	2827072	9	3.72192	2.95716
Nc-1-IP_pe	chr1	2831144	2831573	430	2831298	7	3.06442	2.73501
Nc-1-IP_pe	chr1	2833836	2834235	400	2834193	9	4.46937	3.41876
Nc-1-IP_pe	chr1	2866983	2867246	264	2867057	8	3.56021	2.95918
Nc-1-IP_pe	chr1	2870660	2871014	355	2870910	10	5.23357	3.76063
Nc-1-IP_pe	chr1	2874631	2874945	315	2874641	9	4.46937	3.41876
Nc-1-IP_pe	chr1	2884995	2885300	306	2885148	9	4.35492	3.34727
Nc-1-IP_pe	chr1	2886761	2887024	264	2886808	8	3.74505	3.07688
Nc-1-IP_pe	chr1	2890090	2890564	475	2890367	14	8.29543	4.93196
Nc-1-IP_pe	chr1	2894594	2894942	349	2894838	9	4.46937	3.41876
Nc-1-IP_pe	chr1	2897937	2898227	291	2898132	8	3.64414	3.01254
Nc-1-IP_pe	chr1	2910295	2910694	400	2910391	8	3.74505	3.07688
Nc-1-IP_pe	chr1	2913826	2914144	319	2913932	11	4.54404	3.2242
Nc-1-IP_pe	chr1	2916704	2916968	265	2916824	9	4.46937	3.41876
Nc-1-IP_pe	chr1	2925063	2925327	265	2925206	8	3.74505	3.07688
Nc-1-IP_pe	chr1	2926497	2926771	275	2926691	11	5.77463	3.94557

Nc-1-IP_pe	chr1	2930249	2930768	520	2930599	12	6.86906	4.44438
Nc-1-IP_pe	chr1	2939177	2939494	318	2939317	10	4.99888	3.61677
Nc-1-IP_pe	chr1	2945741	2946618	878	2946199	8	3.74505	3.07688
Nc-1-IP_pe	chr1	2962200	2962649	450	2962270	8	3.74505	3.07688
Nc-1-IP_pe	chr1	2972558	2972890	333	2972761	7	3.06442	2.73501
Nc-1-IP_pe	chr1	2978890	2979338	449	2979033	9	4.46937	3.41876
Nc-1-IP_pe	chr1	2982572	2982975	404	2982599	9	4.46937	3.41876
Nc-1-IP_pe	chr1	2983899	2984357	459	2984041	9	4.46937	3.41876
Nc-1-IP_pe	chr1	3021639	3022133	495	3021897	10	4.62142	3.388
Nc-1-IP_pe	chr1	3032880	3033182	303	3033012	8	3.7276	3.06575
Nc-1-IP_pe	chr1	3038733	3039087	355	3038908	9	4.46937	3.41876
Nc-1-IP_pe	chr1	3060777	3061845	1069	3060991	12	6.86906	4.44438
Nc-1-IP_pe	chr1	3073619	3074251	633	3074088	12	6.86906	4.44438
Nc-1-IP_pe	chr1	3075761	3076593	833	3076380	11	4.54404	3.2242
Nc-1-IP_pe	chr1	3083154	3083426	273	3083359	9	4.46937	3.41876
Nc-1-IP_pe	chr1	3096083	3096774	692	3096376	8	3.56021	2.95918
Nc-1-IP_pe	chr1	3114881	3115433	553	3115338	8	3.74505	3.07688
Nc-1-IP_pe	chr1	3115864	3116182	319	3116033	13	7.7352	4.78626
Nc-1-IP_pe	chr1	3119019	3119299	281	3119211	12	6.86906	4.44438
Nc-1-IP_pe	chr1	3120229	3120841	613	3120645	10	5.23357	3.76063
Nc-1-IP_pe	chr1	3123409	3123734	326	3123458	9	4.46937	3.41876
Nc-1-IP_pe	chr1	3132258	3132561	304	3132462	14	8.63073	5.12814
Nc-1-IP_pe	chr1	3145013	3145559	547	3145344	12	6.86906	4.44438
Nc-1-IP_pe	chr1	3165175	3165694	520	3165573	11	5.10577	3.5486
Nc-1-IP_pe	chr1	3166344	3166651	308	3166544	10	4.39571	3.25288
Nc-1-IP_pe	chr1	3170576	3170973	398	3170719	13	7.7352	4.78626
Nc-1-IP_pe	chr1	3171447	3171723	277	3171607	10	4.99888	3.61677
Nc-1-IP_pe	chr1	3172429	3172759	331	3172584	8	3.74505	3.07688
Nc-1-IP_pe	chr1	3174644	3175173	530	3174669	8	3.56021	2.95918
Nc-1-IP_pe	chr1	3179329	3179884	556	3179767	9	3.27326	2.68684
Nc-1-IP_pe	chr1	3181282	3181790	509	3181592	19	8.4638	4.21715
Nc-1-IP_pe	chr1	3184448	3184839	392	3184578	8	3.74505	3.07688
Nc-1-IP_pe	chr1	3186882	3187245	364	3187174	7	3.06442	2.73501
Nc-1-IP_pe	chr1	3205141	3205399	259	3205317	10	5.23357	3.76063
Nc-1-IP_pe	chr1	3206526	3206980	455	3206752	8	3.74505	3.07688
Nc-1-IP_pe	chr1	3211781	3212078	298	3211815	9	4.46937	3.41876
Nc-1-IP_pe	chr1	3215405	3215675	271	3215480	7	2.9043	2.63038
Nc-1-IP_pe	chr1	3217120	3217457	338	3217266	10	5.23357	3.76063
Nc-1-IP_pe	chr1	3222325	3222943	619	3222712	10	5.23357	3.76063
Nc-1-IP_pe	chr1	3236777	3237185	409	3237036	13	5.9487	3.76157
Nc-1-IP_pe	chr1	3241163	3241605	443	3241441	10	3.89064	2.95552
Nc-1-IP_pe	chr1	3259554	3259927	374	3259733	10	5.10552	3.682
Nc-1-IP_pe	chr1	3267682	3268046	365	3267701	9	4.20361	3.25317
Nc-1-IP_pe	chr1	3276547	3277154	608	3276784	10	4.99888	3.61677
Nc-1-IP_pe	chr1	3283763	3284135	373	3283915	13	7.34205	4.55444

Nc-1-IP_pe	chr1	3288124	3288455	332	3288327	12	6.86906	4.44438
Nc-1-IP_pe	chr1	3291845	3292620	776	3291949	9	3.66792	2.92434
Nc-1-IP_pe	chr1	3296376	3296752	377	3296512	13	7.7352	4.78626
Nc-1-IP_pe	chr1	3297838	3298339	502	3297870	9	4.46937	3.41876
Nc-1-IP_pe	chr1	3298667	3299132	466	3298736	8	3.56021	2.95918
Nc-1-IP_pe	chr1	3319583	3320055	473	3320039	12	3.47963	2.55766
Nc-1-IP_pe	chr1	3320750	3321054	305	3320878	11	4.54404	3.2242
Nc-1-IP_pe	chr1	3327652	3328128	477	3327833	10	5.23357	3.76063
Nc-1-IP_pe	chr1	3329215	3329510	296	3329299	8	3.08755	2.66145
Nc-1-IP_pe	chr1	3330887	3331233	347	3331080	12	6.86906	4.44438
Nc-1-IP_pe	chr1	3335033	3335332	300	3335125	8	3.74505	3.07688
Nc-1-IP_pe	chr1	3338319	3339223	905	3338704	9	4.46937	3.41876
Nc-1-IP_pe	chr1	3339762	3340165	404	3339808	11	6.03439	4.10251
Nc-1-IP_pe	chr1	3344028	3344308	281	3344125	9	4.46937	3.41876
Nc-1-IP_pe	chr1	3346271	3346699	429	3346661	8	3.56021	2.95918
Nc-1-IP_pe	chr1	3349732	3349994	263	3349795	7	3.06442	2.73501
Nc-1-IP_pe	chr1	3351822	3352168	347	3352133	9	2.89249	2.46179
Nc-1-IP_pe	chr1	3352809	3353065	257	3352978	12	6.58417	4.27436
Nc-1-IP_pe	chr1	3353409	3353740	332	3353651	8	3.74505	3.07688
Nc-1-IP_pe	chr1	3361283	3361563	281	3361405	13	7.7352	4.78626
Nc-1-IP_pe	chr1	3394163	3394685	523	3394529	11	6.03439	4.10251
Nc-1-IP_pe	chr1	3404228	3404597	370	3404352	8	3.74505	3.07688
Nc-1-IP_pe	chr1	3409380	3409726	347	3409626	8	3.74505	3.07688
Nc-1-IP_pe	chr1	3414098	3414442	345	3414164	9	4.46937	3.41876
Nc-1-IP_pe	chr1	3422206	3422604	399	3422437	12	6.86906	4.44438
Nc-1-IP_pe	chr1	3424679	3424974	296	3424890	12	5.01934	3.37518
Nc-1-IP_pe	chr1	3430698	3431198	501	3430721	9	3.79246	3.00015
Nc-1-IP_pe	chr1	3433457	3433815	359	3433571	10	4.39571	3.25288
Nc-1-IP_pe	chr1	3444431	3444724	294	3444579	9	3.72192	2.95716
Nc-1-IP_pe	chr1	3451932	3452284	353	3452084	9	4.23325	3.27156
Nc-1-IP_pe	chr1	3453050	3453326	277	3453230	14	6.78215	4.0774
Nc-1-IP_pe	chr1	3453948	3454409	462	3454109	16	6.8987	3.86161
Nc-1-IP_pe	chr1	3463985	3464442	458	3464116	17	6.47874	3.54137
Nc-1-IP_pe	chr1	3474140	3474564	425	3474151	9	2.89249	2.46179
Nc-1-IP_pe	chr1	3475452	3475755	304	3475670	25	17.8013	7.68862
Nc-1-IP_pe	chr1	3481412	3481757	346	3481625	13	5.36853	3.44651
Nc-1-IP_pe	chr1	3484229	3484490	262	3484404	10	3.4604	2.70797
Nc-1-IP_pe	chr1	3485025	3485357	333	3485186	13	6.94469	4.32351
Nc-1-IP_pe	chr1	3490168	3490613	446	3490358	12	4.24064	2.95299
Nc-1-IP_pe	chr1	3491915	3492698	784	3492012	11	5.3563	3.696
Nc-1-IP_pe	chr1	3493908	3494389	482	3494173	11	4.61014	3.26192
Nc-1-IP_pe	chr1	3496093	3496441	349	3496321	13	7.14943	4.44205
Nc-1-IP_pe	chr1	3508901	3509184	284	3509154	11	3.40056	2.59215
Nc-1-IP_pe	chr1	3510102	3510394	293	3510317	13	4.02562	2.7544
Nc-1-IP_pe	chr1	3515261	3515616	356	3515327	10	4.83846	3.51913

Nc-1-IP_pe	chr1	3521111	3521479	369	3521393	10	4.62142	3.388
Nc-1-IP_pe	chr1	3525577	3526232	656	3525973	9	3.59586	2.88066
Nc-1-IP_pe	chr1	3533606	3533989	384	3533812	11	4.88957	3.42271
Nc-1-IP_pe	chr1	3534729	3535561	833	3535536	10	4.29423	3.19257
Nc-1-IP_pe	chr1	3538231	3539033	803	3538859	15	8.72788	4.99447
Nc-1-IP_pe	chr1	3539490	3539903	414	3539543	9	3.66792	2.92434
Nc-1-IP_pe	chr1	3542032	3542514	483	3542230	13	5.9487	3.76157
Nc-1-IP_pe	chr1	3544394	3544708	315	3544584	12	4.24064	2.95299
Nc-1-IP_pe	chr1	3546415	3546992	578	3546628	12	5.39344	3.58426
Nc-1-IP_pe	chr1	3553990	3554266	277	3554133	13	6.02837	3.80557
Nc-1-IP_pe	chr1	3564539	3566035	1497	3565809	11	5.10577	3.5486
Nc-1-IP_pe	chr1	3570181	3570563	383	3570195	8	3.14944	2.70014
Nc-1-IP_pe	chr1	3574059	3574711	653	3574111	11	5.10577	3.5486
Nc-1-IP_pe	chr1	3577309	3577870	562	3577362	13	6.62436	4.14003
Nc-1-IP_pe	chr1	3578563	3578961	399	3578822	14	5.51541	3.4073
Nc-1-IP_pe	chr1	3582715	3583124	410	3582966	14	5.06689	3.18081
Nc-1-IP_pe	chr1	3583589	3583841	253	3583687	24	5.47323	2.62998
Nc-1-IP_pe	chr1	3585442	3585856	415	3585694	11	4.6642	3.29286
Nc-1-IP_pe	chr1	3591012	3591278	267	3591012	7	2.86159	2.60254
Nc-1-IP_pe	chr1	3594805	3595106	302	3595102	9	3.27326	2.68684
Nc-1-IP_pe	chr1	3596063	3596901	839	3596318	12	6.86906	4.44438
Nc-1-IP_pe	chr1	3598065	3598895	831	3598246	11	4.54404	3.2242
Nc-1-IP_pe	chr1	3599188	3600018	831	3599634	14	7.42849	4.43574
Nc-1-IP_pe	chr1	3602072	3602524	453	3602316	9	4.3087	3.31847
Nc-1-IP_pe	chr1	3604013	3604286	274	3604116	14	5.03085	3.16286
Nc-1-IP_pe	chr1	3611485	3612413	929	3611929	12	6.6717	4.32642
Nc-1-IP_pe	chr1	3613100	3614274	1175	3613906	12	6.86906	4.44438
Nc-1-IP_pe	chr1	3615319	3615786	468	3615672	8	3.74505	3.07688
Nc-1-IP_pe	chr1	3617947	3618317	371	3618134	17	11.4763	6.15376
Nc-1-IP_pe	chr1	3620397	3621238	842	3620841	11	5.77463	3.94557
Nc-1-IP_pe	chr1	3624241	3625362	1122	3624603	14	6.28469	3.80898
Nc-1-IP_pe	chr1	3634056	3634712	657	3634550	9	3.27326	2.68684
Nc-1-IP_pe	chr1	3641292	3641594	303	3641361	11	4.54404	3.2242
Nc-1-IP_pe	chr1	3649572	3649857	286	3649725	15	6.19428	3.63445
Nc-1-IP_pe	chr1	3650764	3651202	439	3650993	12	6.58417	4.27436
Nc-1-IP_pe	chr1	3651484	3652044	561	3651668	9	4.24819	3.28084
Nc-1-IP_pe	chr1	3657873	3658147	275	3657929	7	3.06442	2.73501
Nc-1-IP_pe	chr1	3671017	3671389	373	3671035	10	4.39571	3.25288
Nc-1-IP_pe	chr1	3672418	3672947	530	3672571	12	6.07041	3.97229
Nc-1-IP_pe	chr1	3674185	3674698	514	3674541	11	3.64839	2.72584
Nc-1-IP_pe	chr1	3678855	3679225	371	3678980	12	6.50787	4.22912
Nc-1-IP_pe	chr1	3681138	3681426	289	3681188	10	5.12294	3.69268
Nc-1-IP_pe	chr1	3682374	3682761	388	3682614	13	6.62436	4.14003
Nc-1-IP_pe	chr1	3688687	3689338	652	3688930	19	9.15219	4.54307
Nc-1-IP_pe	chr1	3693533	3693888	356	3693768	11	5.5792	3.82847

Nc-1-IP_pe chr1	3694601	3694991	391	3694875	10	4.87068	3.5387
Nc-1-IP_pe chr1	3697104	3697619	516	3697444	9	4.3705	3.35698
Nc-1-IP_pe chr1	3703916	3704409	494	3704004	11	4.06403	2.95415
Nc-1-IP_pe chr1	3707673	3707932	260	3707731	10	4.20113	3.13749
Nc-1-IP_pe chr1	3708675	3708934	260	3708716	12	3.16137	2.39719
Nc-1-IP_pe chr1	3717863	3718155	293	3718038	9	3.54889	2.85226
Nc-1-IP_pe chr1	3719620	3720487	868	3719838	14	7.36061	4.39762
Nc-1-IP_pe chr1	3727877	3728432	556	3728061	11	5.7051	3.90381
Nc-1-IP_pe chr1	3734384	3734662	279	3734482	9	4.25966	3.28797
Nc-1-IP_pe chr1	3744667	3745243	577	3745216	10	5.23357	3.76063
Nc-1-IP_pe chr1	3746586	3747211	626	3746773	14	7.34079	4.38651
Nc-1-IP_pe chr1	3748078	3748359	282	3748153	7	3.06442	2.73501
Nc-1-IP_pe chr1	3754720	3755104	385	3754730	9	4.20361	3.25317
Nc-1-IP_pe chr1	3762959	3763474	516	3762979	10	3.08907	2.49869
Nc-1-IP_pe chr1	3772398	3772885	488	3772800	13	5.29187	3.40559
Nc-1-IP_pe chr1	3775143	3775401	259	3775276	12	4.70082	3.20033
Nc-1-IP_pe chr1	3786751	3787030	280	3786881	15	7.56326	4.34922
Nc-1-IP_pe chr1	3796019	3796431	413	3796083	14	3.55308	2.45762
Nc-1-IP_pe chr1	3816097	3816399	303	3816243	11	5.7051	3.90381
Nc-1-IP_pe chr1	3821999	3822621	623	3822131	9	4.46937	3.41876
Nc-1-IP_pe chr1	3832347	3832973	627	3832445	11	5.77463	3.94557
Nc-1-IP_pe chr1	3840032	3840337	306	3840216	13	7.7352	4.78626
Nc-1-IP_pe chr1	3856694	3857329	636	3857278	11	5.3563	3.696
Nc-1-IP_pe chr1	3868639	3868933	295	3868656	13	3.67377	2.58159
Nc-1-IP_pe chr1	3869407	3869991	585	3869804	10	3.08907	2.49869
Nc-1-IP_pe chr1	3870388	3870976	589	3870694	13	7.45271	4.61937
Nc-1-IP_pe chr1	3872620	3872910	291	3872880	12	5.84943	3.84431
Nc-1-IP_pe chr1	3881031	3881333	303	3881250	12	5.84943	3.84431
Nc-1-IP_pe chr1	3886370	3886652	283	3886598	9	4.25966	3.28797
Nc-1-IP_pe chr1	3899520	3900130	611	3899945	10	3.89064	2.95552
Nc-1-IP_pe chr1	3900623	3901116	494	3900764	13	4.41954	2.952
Nc-1-IP_pe chr1	3916706	3916959	254	3916812	10	5.23357	3.76063
Nc-1-IP_pe chr1	3923986	3924401	416	3924274	10	4.39571	3.25288
Nc-1-IP_pe chr1	3924878	3925282	405	3924897	9	3.72192	2.95716
Nc-1-IP_pe chr1	3933157	3933479	323	3933246	10	4.39571	3.25288
Nc-1-IP_pe chr1	3963360	3963741	382	3963694	7	2.9043	2.63038
Nc-1-IP_pe chr1	3987540	3988173	634	3988056	9	3.72192	2.95716
Nc-1-IP_pe chr1	3998235	3998528	294	3998382	9	4.46937	3.41876
Nc-1-IP_pe chr1	3999635	3999932	298	3999882	25	8.22195	3.4832
Nc-1-IP_pe chr1	4002565	4002845	281	4002709	9	4.46937	3.41876
Nc-1-IP_pe chr1	4020943	4021488	546	4021366	9	4.46937	3.41876
Nc-1-IP_pe chr1	4061729	4062011	283	4061832	8	3.74505	3.07688
Nc-1-IP_pe chr1	4077825	4078825	1001	4078012	14	8.63073	5.12814
Nc-1-IP_pe chr1	4107452	4107706	255	4107562	10	4.99888	3.61677
Nc-1-IP_pe chr1	4108865	4109206	342	4109051	9	4.46937	3.41876

Nc-1-IP_pe	chr1	4122328	4122998	671	4122613	8	3.74505	3.07688
Nc-1-IP_pe	chr1	4217663	4218103	441	4217943	11	6.03439	4.10251
Nc-1-IP_pe	chr1	4225209	4225672	464	4225312	8	3.74505	3.07688
Nc-1-IP_pe	chr1	4258358	4258617	260	4258445	8	3.74505	3.07688
Nc-1-IP_pe	chr1	4332404	4332863	460	4332515	11	6.03439	4.10251
Nc-1-IP_pe	chr1	4334124	4334428	305	4334262	8	3.56021	2.95918
Nc-1-IP_pe	chr1	4353190	4353488	299	4353427	9	4.46937	3.41876
Nc-1-IP_pe	chr1	4426339	4426856	518	4426395	10	4.39571	3.25288
Nc-1-IP_pe	chr1	4486709	4487253	545	4486954	9	4.46937	3.41876
Nc-1-IP_pe	chr1	4509409	4509669	261	4509496	10	5.23357	3.76063
Nc-1-IP_pe	chr1	4602066	4602325	260	4602257	11	6.03439	4.10251
Nc-1-IP_pe	chr1	4614818	4615236	419	4614924	8	3.74505	3.07688
Nc-1-IP_pe	chr1	4658125	4658406	282	4658330	10	4.99888	3.61677
Nc-1-IP_pe	chr1	4674587	4674924	338	4674879	9	4.46937	3.41876
Nc-1-IP_pe	chr1	4691533	4692320	788	4692234	13	7.7352	4.78626
Nc-1-IP_pe	chr1	4710031	4710376	346	4710085	7	3.06442	2.73501
Nc-1-IP_pe	chr1	4711457	4711800	344	4711797	8	3.38431	2.8478
Nc-1-IP_pe	chr1	4712459	4712875	417	4712465	8	3.08755	2.66145
Nc-1-IP_pe	chr1	4735901	4736302	402	4736114	9	4.46937	3.41876
Nc-1-IP_pe	chr1	4746807	4747478	672	4747071	11	6.03439	4.10251
Nc-1-IP_pe	chr1	4787380	4787822	443	4787717	9	4.35492	3.34727
Nc-1-IP_pe	chr1	4930410	4930668	259	4930477	8	3.74505	3.07688
Nc-1-IP_pe	chr1	5023585	5023895	311	5023730	9	4.46937	3.41876
Nc-1-IP_pe	chr1	5254022	5254457	436	5254230	7	2.9043	2.63038
Nc-1-IP_pe	chr1	5265039	5265590	552	5265419	11	6.03439	4.10251
Nc-1-IP_pe	chr1	5348823	5349142	320	5348900	10	4.99888	3.61677
Nc-1-IP_pe	chr1	5386296	5386559	264	5386455	11	6.03439	4.10251
Nc-1-IP_pe	chr1	5479092	5479413	322	5479195	11	6.03439	4.10251
Nc-1-IP_pe	chr1	5500105	5500656	552	5500128	9	4.46937	3.41876
Nc-1-IP_pe	chr1	5513328	5513691	364	5513592	8	3.56021	2.95918
Nc-1-IP_pe	chr1	5565491	5565804	314	5565548	10	5.23357	3.76063
Nc-1-IP_pe	chr1	5643550	5643963	414	5643801	9	4.46937	3.41876
Nc-1-IP_pe	chr1	5666405	5666932	528	5666584	10	4.20113	3.13749
Nc-1-IP_pe	chr1	5667279	5667534	256	5667456	10	3.89064	2.95552
Nc-1-IP_pe	chr1	5724500	5724828	329	5724762	11	6.03439	4.10251
Nc-1-IP_pe	chr1	5745623	5746561	939	5745728	10	3.89064	2.95552
Nc-1-IP_pe	chr1	5768515	5768913	399	5768752	7	3.06442	2.73501
Nc-1-IP_pe	chr1	5810128	5810408	281	5810159	7	3.06442	2.73501
Nc-1-IP_pe	chr1	5840689	5840943	255	5840735	8	3.56021	2.95918
Nc-1-IP_pe	chr1	5846371	5846665	295	5846478	11	4.54404	3.2242
Nc-1-IP_pe	chr1	5848014	5848277	264	5848130	8	3.68553	3.03891
Nc-1-IP_pe	chr1	5858498	5858911	414	5858756	9	4.35492	3.34727
Nc-1-IP_pe	chr1	5863049	5863338	290	5863220	10	3.08907	2.49869
Nc-1-IP_pe	chr1	5866354	5866619	266	5866481	11	6.03439	4.10251
Nc-1-IP_pe	chr1	5867202	5867781	580	5867241	9	4.46937	3.41876

Nc-1-IP_pe	chr1	5868653	5868954	302	5868753	9	4.46937	3.41876
Nc-1-IP_pe	chr1	5873710	5873983	274	5873725	9	3.22129	2.65588
Nc-1-IP_pe	chr1	5878598	5879002	405	5878640	8	3.14944	2.70014
Nc-1-IP_pe	chr1	5881986	5882765	780	5882625	9	4.05981	3.16422
Nc-1-IP_pe	chr1	5910015	5910536	522	5910477	9	4.46937	3.41876
Nc-1-IP_pe	chr1	5910832	5911123	292	5910994	9	4.46937	3.41876
Nc-1-IP_pe	chr1	5911974	5912275	302	5912142	11	6.03439	4.10251
Nc-1-IP_pe	chr1	5934323	5934688	366	5934639	8	3.74505	3.07688
Nc-1-IP_pe	chr1	5948165	5948490	326	5948173	8	3.56021	2.95918
Nc-1-IP_pe	chr1	5951309	5951648	340	5951610	8	3.56021	2.95918
Nc-1-IP_pe	chr1	5975913	5976168	256	5976061	10	5.23357	3.76063
Nc-1-IP_pe	chr1	5978140	5978461	322	5978377	11	6.03439	4.10251
Nc-1-IP_pe	chr1	6002559	6003304	746	6002710	11	4.54404	3.2242
Nc-1-IP_pe	chr1	6006922	6007286	365	6007079	9	4.46937	3.41876
Nc-1-IP_pe	chr1	6011154	6011502	349	6011258	9	3.97689	3.11315
Nc-1-IP_pe	chr1	6015558	6015877	320	6015646	11	5.03835	3.5092
Nc-1-IP_pe	chr1	6035123	6035534	412	6035140	9	2.89249	2.46179
Nc-1-IP_pe	chr1	6037566	6037819	254	6037787	7	2.9043	2.63038
Nc-1-IP_pe	chr1	6039276	6039643	368	6039377	8	3.74505	3.07688
Nc-1-IP_pe	chr1	6041545	6041965	421	6041734	10	5.23357	3.76063
Nc-1-IP_pe	chr1	6049380	6049679	300	6049505	9	4.46937	3.41876
Nc-1-IP_pe	chr1	6063998	6064257	260	6064138	10	4.82246	3.50943
Nc-1-IP_pe	chr1	6064861	6065178	318	6064902	8	3.58997	2.97809
Nc-1-IP_pe	chr1	6077342	6077621	280	6077581	9	3.72192	2.95716
Nc-1-IP_pe	chr1	6080977	6081754	778	6081535	12	6.58417	4.27436
Nc-1-IP_pe	chr1	6086156	6086553	398	6086384	11	6.03439	4.10251
Nc-1-IP_pe	chr1	6099392	6100367	976	6100271	12	5.61153	3.70794
Nc-1-IP_pe	chr1	6101183	6101539	357	6101399	10	5.05378	3.65032
Nc-1-IP_pe	chr1	6103414	6104088	675	6103698	14	8.32526	4.94932
Nc-1-IP_pe	chr1	6105496	6105827	332	6105622	9	3.72192	2.95716
Nc-1-IP_pe	chr1	6106381	6107037	657	6106427	9	3.94978	3.09648
Nc-1-IP_pe	chr1	6110032	6110288	257	6110033	9	3.12089	2.59629
Nc-1-IP_pe	chr1	6110604	6110874	271	6110701	9	3.84391	3.03159
Nc-1-IP_pe	chr1	6111919	6112260	342	6112076	9	3.72192	2.95716
Nc-1-IP_pe	chr1	6115430	6115704	275	6115544	11	4.06403	2.95415
Nc-1-IP_pe	chr1	6128927	6129573	647	6129380	12	4.24064	2.95299
Nc-1-IP_pe	chr1	6146381	6146816	436	6146526	13	6.02837	3.80557
Nc-1-IP_pe	chr1	6154337	6154807	471	6154348	11	3.64839	2.72584
Nc-1-IP_pe	chr1	6159758	6160382	625	6160049	12	5.59553	3.69882
Nc-1-IP_pe	chr1	6161387	6161698	312	6161617	13	6.43528	4.03292
Nc-1-IP_pe	chr1	6172479	6172755	277	6172646	12	6.12485	4.004
Nc-1-IP_pe	chr1	6173232	6173507	276	6173279	9	4.05981	3.16422
Nc-1-IP_pe	chr1	6177711	6178187	477	6177964	8	3.74505	3.07688
Nc-1-IP_pe	chr1	6179129	6179429	301	6179208	11	4.54404	3.2242
Nc-1-IP_pe	chr1	6207545	6207927	383	6207643	10	3.83201	2.92147

Nc-1-IP_pe	chr1	6209915	6210221	307	6210160	10	4.80655	3.49978
Nc-1-IP_pe	chr1	6212137	6212644	508	6212494	8	3.74505	3.07688
Nc-1-IP_pe	chr1	6213079	6213336	258	6213257	10	5.23357	3.76063
Nc-1-IP_pe	chr1	6217753	6218221	469	6218030	8	3.74505	3.07688
Nc-1-IP_pe	chr1	6245253	6245613	361	6245480	13	6.36468	3.99316
Nc-1-IP_pe	chr1	6248291	6248720	430	6248643	13	4.86362	3.18015
Nc-1-IP_pe	chr1	6252492	6253168	677	6252565	11	5.3563	3.696
Nc-1-IP_pe	chr1	6253621	6253936	316	6253822	13	7.27693	4.51635
Nc-1-IP_pe	chr1	6261134	6261744	611	6261622	12	5.5796	3.68975
Nc-1-IP_pe	chr1	6272542	6273040	499	6272670	12	6.31185	4.11348
Nc-1-IP_pe	chr1	6299163	6299548	386	6299438	9	4.46937	3.41876
Nc-1-IP_pe	chr1	6303920	6304240	321	6304026	11	5.3563	3.696
Nc-1-IP_pe	chr1	6304991	6306096	1106	6305926	16	8.79222	4.84884
Nc-1-IP_pe	chr1	6307771	6308321	551	6308153	13	6.88507	4.28917
Nc-1-IP_pe	chr1	6323142	6323464	323	6323198	9	4.35492	3.34727
Nc-1-IP_pe	chr1	6334150	6334682	533	6334371	15	8.26005	4.73146
Nc-1-IP_pe	chr1	6343141	6343511	371	6343194	11	3.64839	2.72584
Nc-1-IP_pe	chr1	6355000	6355280	281	6355098	10	3.4604	2.70797
Nc-1-IP_pe	chr1	6355783	6356880	1098	6356365	15	8.51839	4.87609
Nc-1-IP_pe	chr1	6359423	6359836	414	6359651	13	7.7352	4.78626
Nc-1-IP_pe	chr1	6360284	6360601	318	6360375	14	8.29543	4.93196
Nc-1-IP_pe	chr1	6374287	6374689	403	6374543	13	4.86362	3.18015
Nc-1-IP_pe	chr1	6378472	6378754	283	6378493	9	3.72192	2.95716
Nc-1-IP_pe	chr1	6391215	6391473	259	6391329	12	6.18005	4.03623
Nc-1-IP_pe	chr1	6392766	6393062	297	6393010	12	6.25484	4.08002
Nc-1-IP_pe	chr1	6400298	6400572	275	6400380	8	3.74505	3.07688
Nc-1-IP_pe	chr1	6416443	6416802	360	6416482	11	4.54404	3.2242
Nc-1-IP_pe	chr1	6418430	6419115	686	6418876	12	5.8598	3.85029
Nc-1-IP_pe	chr1	6420380	6421156	777	6420476	12	4.51041	3.09723
Nc-1-IP_pe	chr1	6425491	6426104	614	6425777	13	6.50707	4.07348
Nc-1-IP_pe	chr1	6426578	6427267	690	6426732	13	6.32982	3.97358
Nc-1-IP_pe	chr1	6434811	6435352	542	6435030	11	4.54404	3.2242
Nc-1-IP_pe	chr1	6435648	6436147	500	6435963	13	6.61706	4.13588
Nc-1-IP_pe	chr1	6437648	6438092	445	6437676	9	3.85693	3.03955
Nc-1-IP_pe	chr1	6441732	6442025	294	6441913	17	5.54967	3.12324
Nc-1-IP_pe	chr1	6448030	6448335	306	6448310	10	3.4604	2.70797
Nc-1-IP_pe	chr1	6453283	6453549	267	6453417	13	6.06081	3.82353
Nc-1-IP_pe	chr1	6454026	6454387	362	6454172	9	3.45738	2.79711
Nc-1-IP_pe	chr1	6456278	6456555	278	6456494	9	4.01804	3.13847
Nc-1-IP_pe	chr1	6464260	6464530	271	6464325	8	3.08755	2.66145
Nc-1-IP_pe	chr1	6467390	6467994	605	6467689	12	5.77527	3.80164
Nc-1-IP_pe	chr1	6472714	6473148	435	6472882	10	3.89064	2.95552
Nc-1-IP_pe	chr1	6474945	6475204	260	6475116	12	6.08847	3.9828
Nc-1-IP_pe	chr1	6476056	6476629	574	6476069	9	4.05981	3.16422
Nc-1-IP_pe	chr1	6477635	6478219	585	6478186	11	4.54404	3.2242

Nc-1-IP_pe	chr1	6490264	6490616	353	6490593	14	4.60011	2.95114
Nc-1-IP_pe	chr1	6492247	6492505	259	6492273	11	5.10577	3.5486
Nc-1-IP_pe	chr1	6492801	6493179	379	6493020	10	4.80655	3.49978
Nc-1-IP_pe	chr1	6495961	6496308	348	6496166	10	4.90322	3.55848
Nc-1-IP_pe	chr1	6498373	6498643	271	6498481	11	5.7051	3.90381
Nc-1-IP_pe	chr1	6502314	6502716	403	6502401	8	3.51085	2.92785
Nc-1-IP_pe	chr1	6503821	6504286	466	6504052	13	7.7352	4.78626
Nc-1-IP_pe	chr1	6504666	6505185	520	6505043	10	5.23357	3.76063
Nc-1-IP_pe	chr1	6510172	6510490	319	6510401	10	3.89064	2.95552
Nc-1-IP_pe	chr1	6519622	6520043	422	6519903	9	4.05981	3.16422
Nc-1-IP_pe	chr1	6552648	6553201	554	6552711	8	3.74505	3.07688
Nc-1-IP_pe	chr1	6553977	6554705	729	6554484	25	20.0142	8.88877
Nc-1-IP_pe	chr1	6588831	6589376	546	6589193	10	5.01973	3.6295
Nc-1-IP_pe	chr1	6592606	6593245	640	6593170	11	5.10577	3.5486
Nc-1-IP_pe	chr1	6597047	6597887	841	6597447	9	4.20361	3.25317
Nc-1-IP_pe	chr1	6598961	6599228	268	6599180	15	7.02624	4.06292
Nc-1-IP_pe	chr1	6602498	6603265	768	6602513	16	3.65575	2.38624
Nc-1-IP_pe	chr1	6607520	6607846	327	6607527	8	3.74505	3.07688
Nc-1-IP_pe	chr1	6608111	6608672	562	6608176	9	4.46937	3.41876
Nc-1-IP_pe	chr1	6612936	6613323	388	6613052	11	6.03439	4.10251
Nc-1-IP_pe	chr1	6613873	6614297	425	6614082	14	8.63073	5.12814
Nc-1-IP_pe	chr1	6619528	6619787	260	6619582	8	3.60341	2.98663
Nc-1-IP_pe	chr1	6626328	6626593	266	6626488	11	5.03835	3.5092
Nc-1-IP_pe	chr1	6629151	6629426	276	6629326	12	4.24064	2.95299
Nc-1-IP_pe	chr1	6634357	6634629	273	6634364	7	3.06442	2.73501
Nc-1-IP_pe	chr1	6650470	6650851	382	6650713	9	4.46937	3.41876
Nc-1-IP_pe	chr1	6669118	6669454	337	6669385	9	4.46937	3.41876
Nc-1-IP_pe	chr1	6701408	6701925	518	6701629	24	13.6493	5.83606
Nc-1-IP_pe	chr1	6720206	6720725	520	6720628	10	4.99888	3.61677
Nc-1-IP_pe	chr1	6907538	6907951	414	6907798	10	5.23357	3.76063
Nc-1-IP_pe	chr1	6909497	6909776	280	6909574	8	3.74505	3.07688
Nc-1-IP_pe	chr1	6913181	6913445	265	6913403	11	6.03439	4.10251
Nc-1-IP_pe	chr1	6925661	6925971	311	6925874	9	4.46937	3.41876
Nc-1-IP_pe	chr1	6995499	6995887	389	6995616	9	4.46937	3.41876
Nc-1-IP_pe	chr1	7009513	7010374	862	7009618	12	5.77527	3.80164
Nc-1-IP_pe	chr1	7043086	7043539	454	7043168	9	4.46937	3.41876
Nc-1-IP_pe	chr1	7044049	7044316	268	7044266	12	6.58417	4.27436
Nc-1-IP_pe	chr1	7049934	7050271	338	7050235	9	3.72192	2.95716
Nc-1-IP_pe	chr1	7102679	7102957	279	7102799	10	5.23357	3.76063
Nc-1-IP_pe	chr1	7149079	7149389	311	7149306	8	3.74505	3.07688
Nc-1-IP_pe	chr1	7153665	7153926	262	7153857	9	2.89249	2.46179
Nc-1-IP_pe	chr1	7174528	7174842	315	7174683	11	6.03439	4.10251
Nc-1-IP_pe	chr1	7177582	7177905	324	7177791	10	5.23357	3.76063
Nc-1-IP_pe	chr1	7192800	7193098	299	7193009	11	6.03439	4.10251
Nc-1-IP_pe	chr1	7220848	7221183	336	7220975	11	6.03439	4.10251

Nc-1-IP_pe	chr1	7224695	7224991	297	7224883	11	5.10577	3.5486
Nc-1-IP_pe	chr1	7228637	7229271	635	7228839	10	4.39571	3.25288
Nc-1-IP_pe	chr1	7236815	7237078	264	7236935	14	8.63073	5.12814
Nc-1-IP_pe	chr1	7256397	7256802	406	7256449	9	4.46937	3.41876
Nc-1-IP_pe	chr1	7301642	7302092	451	7301695	9	4.46937	3.41876
Nc-1-IP_pe	chr1	7331568	7331821	254	7331692	12	5.84943	3.84431
Nc-1-IP_pe	chr1	7349901	7350162	262	7350110	8	3.08755	2.66145
Nc-1-IP_pe	chr1	7389048	7389650	603	7389168	13	5.29187	3.40559
Nc-1-IP_pe	chr1	7403818	7404597	780	7404450	11	6.03439	4.10251
Nc-1-IP_pe	chr1	7409236	7409594	359	7409417	15	6.19428	3.63445
Nc-1-IP_pe	chr1	7431609	7431985	377	7431793	12	5.61153	3.70794
Nc-1-IP_pe	chr1	7439271	7439649	379	7439509	9	4.46937	3.41876
Nc-1-IP_pe	chr1	7440034	7440464	431	7440267	12	6.86906	4.44438
Nc-1-IP_pe	chr1	7454589	7455185	597	7454624	9	4.46937	3.41876
Nc-1-IP_pe	chr1	7471576	7471881	306	7471842	9	3.43499	2.78365
Nc-1-IP_pe	chr1	7473035	7474000	966	7473869	12	6.56855	4.26509
Nc-1-IP_pe	chr1	7478141	7478459	319	7478229	9	4.46937	3.41876
Nc-1-IP_pe	chr1	7479533	7479791	259	7479660	8	3.74505	3.07688
Nc-1-IP_pe	chr1	7485002	7485282	281	7485131	8	3.74505	3.07688
Nc-1-IP_pe	chr1	7493655	7493948	294	7493738	10	5.23357	3.76063
Nc-1-IP_pe	chr1	7495894	7496579	686	7496378	9	4.46937	3.41876
Nc-1-IP_pe	chr1	7503778	7504046	269	7503903	11	6.03439	4.10251
Nc-1-IP_pe	chr1	7504960	7505337	378	7505233	10	4.99888	3.61677
Nc-1-IP_pe	chr1	7518428	7518793	366	7518573	8	3.74505	3.07688
Nc-1-IP_pe	chr1	7570274	7570863	590	7570641	9	4.46937	3.41876
Nc-1-IP_pe	chr1	7578332	7578590	259	7578424	10	5.23357	3.76063
Nc-1-IP_pe	chr1	7583338	7583951	614	7583875	10	5.23357	3.76063
Nc-1-IP_pe	chr1	7586763	7587088	326	7586895	9	4.46937	3.41876
Nc-1-IP_pe	chr1	7601014	7601479	466	7601134	10	4.99888	3.61677
Nc-1-IP_pe	chr1	7628457	7628996	540	7628556	10	5.19356	3.73603
Nc-1-IP_pe	chr1	7630852	7631292	441	7631112	10	4.99888	3.61677
Nc-1-IP_pe	chr1	7647463	7648128	666	7647980	13	7.45271	4.61937
Nc-1-IP_pe	chr1	7661850	7662168	319	7661976	8	3.7135	3.05675
Nc-1-IP_pe	chr1	7667249	7668022	774	7667360	11	4.74697	3.34038
Nc-1-IP_pe	chr1	7668545	7668812	268	7668593	7	2.89555	2.62467
Nc-1-IP_pe	chr1	7683473	7683772	300	7683722	9	2.89249	2.46179
Nc-1-IP_pe	chr1	7687334	7687642	309	7687418	10	5.23357	3.76063
Nc-1-IP_pe	chr1	7695458	7695784	327	7695681	8	3.74505	3.07688
Nc-1-IP_pe	chr1	7703773	7704122	350	7703905	11	6.03439	4.10251
Nc-1-IP_pe	chr1	7725654	7726020	367	7725818	13	7.12853	4.42991
Nc-1-IP_pe	chr1	7771221	7772018	798	7771410	13	5.9487	3.76157
Nc-1-IP_pe	chr1	7776992	7777306	315	7776995	8	3.0402	2.6319
Nc-1-IP_pe	chr1	7827290	7827579	290	7827526	9	4.25966	3.28797
Nc-1-IP_pe	chr1	7894224	7894482	259	7894286	10	5.23357	3.76063
Nc-1-IP_pe	chr1	7925785	7926061	277	7925894	9	4.46937	3.41876

Nc-1-IP_pe	chr1	7952332	7953452	1121	7953280	22	10.0534	4.52509
Nc-1-IP_pe	chr1	7961710	7961972	263	7961804	12	5.23082	3.49289
Nc-1-IP_pe	chr1	7984154	7984432	279	7984180	9	4.20361	3.25317
Nc-1-IP_pe	chr1	8008524	8008830	307	8008537	8	3.08755	2.66145
Nc-1-IP_pe	chr1	8009405	8009681	277	8009523	11	6.03439	4.10251
Nc-1-IP_pe	chr1	8044913	8045384	472	8045091	11	6.03439	4.10251
Nc-1-IP_pe	chr1	8050552	8050821	270	8050596	9	4.25966	3.28797
Nc-1-IP_pe	chr1	8170164	8170496	333	8170388	10	5.23357	3.76063
Nc-1-IP_pe	chr1	8196167	8196423	257	8196239	11	6.03439	4.10251
Nc-1-IP_pe	chr1	8216973	8217345	373	8217307	10	3.94999	2.99009
Nc-1-IP_pe	chr1	8221962	8222366	405	8222011	9	4.27829	3.29955
Nc-1-IP_pe	chr1	8234583	8234873	291	8234607	8	3.38431	2.8478
Nc-1-IP_pe	chr1	8241687	8242280	594	8242128	13	7.7352	4.78626
Nc-1-IP_pe	chr1	8257201	8258291	1091	8257723	10	5.23357	3.76063
Nc-1-IP_pe	chr1	8271012	8271294	283	8271063	8	3.56021	2.95918
Nc-1-IP_pe	chr1	8349208	8349645	438	8349437	12	6.12485	4.004
Nc-1-IP_pe	chr1	8355288	8355579	292	8355553	8	3.52386	2.93611
Nc-1-IP_pe	chr1	8361586	8361929	344	8361739	11	5.10577	3.5486
Nc-1-IP_pe	chr1	8372728	8373000	273	8372900	11	6.03439	4.10251
Nc-1-IP_pe	chr1	8496349	8496758	410	8496702	8	3.56021	2.95918
Nc-1-IP_pe	chr1	8500396	8500719	324	8500477	11	5.3563	3.696
Nc-1-IP_pe	chr1	8501331	8502036	706	8501773	16	10.0135	5.53039
Nc-1-IP_pe	chr1	8552810	8553337	528	8552892	8	3.74505	3.07688
Nc-1-IP_pe	chr1	8798674	8799169	496	8799040	11	6.03439	4.10251
Nc-1-IP_pe	chr1	8800640	8800957	318	8800740	11	6.03439	4.10251
Nc-1-IP_pe	chr1	8816951	8817299	349	8817090	12	4.24064	2.95299
Nc-1-IP_pe	chr1	8825683	8825994	312	8825774	9	3.72192	2.95716
Nc-1-IP_pe	chr1	8860814	8861271	458	8861180	12	4.70082	3.20033
Nc-1-IP_pe	chr1	8869538	8869819	282	8869751	11	6.03439	4.10251
Nc-1-IP_pe	chr1	8870737	8871160	424	8871140	8	3.56021	2.95918
Nc-1-IP_pe	chr1	8887601	8888238	638	8888046	10	4.62142	3.388
Nc-1-IP_pe	chr1	8892801	8893080	280	8892912	10	5.23357	3.76063
Nc-1-IP_pe	chr1	8917734	8918131	398	8918065	8	3.74505	3.07688
Nc-1-IP_pe	chr1	8937378	8937659	282	8937639	8	3.74505	3.07688
Nc-1-IP_pe	chr1	8949202	8949480	279	8949285	9	4.46937	3.41876
Nc-1-IP_pe	chr1	8958208	8958652	445	8958491	10	4.93609	3.57849
Nc-1-IP_pe	chr1	8967425	8967908	484	8967579	12	6.58417	4.27436
Nc-1-IP_pe	chr1	8976826	8977338	513	8976887	8	3.74505	3.07688
Nc-1-IP_pe	chr1	8999441	8999851	411	8999676	8	3.74505	3.07688
Nc-1-IP_pe	chr1	9007805	9008390	586	9008164	12	6.86906	4.44438
Nc-1-IP_pe	chr1	9013410	9013667	258	9013501	9	4.46937	3.41876
Nc-1-IP_pe	chr1	9031547	9031905	359	9031696	14	6.61011	3.98382
Nc-1-IP_pe	chr1	9034267	9034590	324	9034291	10	3.08907	2.49869
Nc-1-IP_pe	chr1	9039619	9039878	260	9039631	9	3.79246	3.00015
Nc-1-IP_pe	chr1	9059238	9059528	291	9059364	10	4.99888	3.61677

Nc-1-IP_pe	chr1	9067085	9067367	283	9067366	8	3.08755	2.66145
Nc-1-IP_pe	chr1	9083668	9084056	389	9083848	9	3.27326	2.68684
Nc-1-IP_pe	chr1	9106462	9106883	422	9106720	11	5.10577	3.5486
Nc-1-IP_pe	chr1	9111949	9112259	311	9111993	7	2.9043	2.63038
Nc-1-IP_pe	chr1	9118049	9118435	387	9118141	9	4.46937	3.41876
Nc-1-IP_pe	chr1	9128284	9128918	635	9128464	13	5.42865	3.47872
Nc-1-IP_pe	chr1	9146234	9146489	256	9146283	9	4.46937	3.41876
Nc-1-IP_pe	chr1	9164216	9164536	321	9164354	13	7.49774	4.64586
Nc-1-IP_pe	chr1	9166688	9167043	356	9166767	10	3.89064	2.95552
Nc-1-IP_pe	chr1	9173951	9174223	273	9174158	10	4.99888	3.61677
Nc-1-IP_pe	chr1	9190694	9190963	270	9190876	8	3.74505	3.07688
Nc-1-IP_pe	chr1	9197443	9197826	384	9197728	11	4.54404	3.2242
Nc-1-IP_pe	chr1	9198400	9198700	301	9198583	11	4.54404	3.2242
Nc-1-IP_pe	chr1	9201475	9201818	344	9201680	14	8.20556	4.87976
Nc-1-IP_pe	chr1	9233060	9233683	624	9233319	12	4.24064	2.95299
Nc-1-IP_pe	chr1	9234195	9234569	375	9234356	9	4.25966	3.28797
Nc-1-IP_pe	chr1	9247136	9247496	361	9247166	10	3.4604	2.70797
Nc-1-IP_pe	chr1	9248879	9249214	336	9249037	14	8.63073	5.12814
Nc-1-IP_pe	chr1	9249674	9250028	355	9249847	9	4.46937	3.41876
Nc-1-IP_pe	chr1	9251243	9251813	571	9251661	11	6.03439	4.10251
Nc-1-IP_pe	chr1	9264443	9264815	373	9264772	10	4.62142	3.388
Nc-1-IP_pe	chr1	9271897	9272365	469	9272178	8	3.74505	3.07688
Nc-1-IP_pe	chr1	9281959	9282323	365	9282133	12	4.70082	3.20033
Nc-1-IP_pe	chr1	9283381	9284112	732	9283794	12	6.86906	4.44438
Nc-1-IP_pe	chr1	9292270	9292565	296	9292404	8	3.74505	3.07688
Nc-1-IP_pe	chr1	9293312	9293580	269	9293408	11	4.54404	3.2242
Nc-1-IP_pe	chr1	9294892	9295169	278	9295015	10	5.23357	3.76063
Nc-1-IP_pe	chr1	9307514	9307818	305	9307619	7	2.86159	2.60254
Nc-1-IP_pe	chr1	9316353	9316726	374	9316470	15	4.06016	2.62146
Nc-1-IP_pe	chr1	9319808	9320226	419	9320095	12	5.77527	3.80164
Nc-1-IP_pe	chr1	9326280	9326751	472	9326476	13	7.40812	4.59318
Nc-1-IP_pe	chr1	9332843	9333987	1145	9333679	10	5.23357	3.76063
Nc-1-IP_pe	chr1	9339110	9339452	343	9339287	15	6.78905	3.93887
Nc-1-IP_pe	chr1	9348999	9349327	329	9349283	8	3.08755	2.66145
Nc-1-IP_pe	chr1	9352146	9352580	435	9352178	10	4.93609	3.57849
Nc-1-IP_pe	chr1	9352889	9353281	393	9353256	9	4.46937	3.41876
Nc-1-IP_pe	chr1	9354157	9354457	301	9354205	8	3.74505	3.07688
Nc-1-IP_pe	chr1	9354903	9355277	375	9355132	11	5.77463	3.94557
Nc-1-IP_pe	chr1	9359895	9360741	847	9360653	11	6.03439	4.10251
Nc-1-IP_pe	chr1	9362440	9362722	283	9362575	10	5.23357	3.76063
Nc-1-IP_pe	chr1	9366241	9366505	265	9366364	14	8.63073	5.12814
Nc-1-IP_pe	chr1	9367414	9367948	535	9367582	12	4.24064	2.95299
Nc-1-IP_pe	chr1	9369596	9369943	348	9369896	11	4.06403	2.95415
Nc-1-IP_pe	chr1	9370845	9371183	339	9370905	9	3.27326	2.68684
Nc-1-IP_pe	chr1	9391548	9391841	294	9391587	8	3.52386	2.93611

Nc-1-IP_pe	chr1	9398846	9399604	759	9399177	9	4.3705	3.35698
Nc-1-IP_pe	chr1	9405271	9405889	619	9405772	11	6.03439	4.10251
Nc-1-IP_pe	chr1	9428672	9429121	450	9429026	10	4.39571	3.25288
Nc-1-IP_pe	chr1	9447490	9447889	400	9447733	12	6.86906	4.44438
Nc-1-IP_pe	chr1	9449693	9451267	1575	9450772	11	5.77463	3.94557
Nc-1-IP_pe	chr1	9455890	9456228	339	9456061	8	3.74505	3.07688
Nc-1-IP_pe	chr1	9459372	9459723	352	9459519	9	4.46937	3.41876
Nc-1-IP_pe	chr1	9511310	9511639	330	9511406	9	4.25966	3.28797
Nc-1-IP_pe	chr1	9528522	9529034	513	9528631	9	4.35492	3.34727
Nc-1-IP_pe	chr1	9595073	9595564	492	9595529	9	3.99054	3.12154
Nc-1-IP_pe	chr1	9602176	9602546	371	9602420	10	4.39571	3.25288
Nc-1-IP_pe	chr1	9608701	9609028	328	9608763	9	4.46937	3.41876
Nc-1-IP_pe	chr1	9612848	9613128	281	9612983	9	4.46937	3.41876
Nc-1-IP_pe	chr1	9626365	9626700	336	9626522	12	5.84943	3.84431
Nc-1-IP_pe	chr1	9629743	9630365	623	9629827	10	5.23357	3.76063
Nc-1-IP_pe	chr1	9631137	9631784	648	9631708	10	5.23357	3.76063
Nc-1-IP_pe	chr1	9632526	9633087	562	9632628	9	3.72192	2.95716
Nc-1-IP_pe	chr1	9640933	9641202	270	9641008	11	4.06403	2.95415
Nc-1-IP_pe	chr1	9716924	9717405	482	9716992	9	3.72192	2.95716
Nc-1-IP_pe	chr1	9720129	9720577	449	9720407	11	4.06403	2.95415
Nc-1-IP_pe	chr1	9721211	9721577	367	9721302	10	4.87068	3.5387
Nc-1-IP_pe	chr1	9722705	9723384	680	9723326	10	4.79071	3.49018
Nc-1-IP_pe	chr1	9724014	9724346	333	9724143	11	5.52654	3.79706
Nc-1-IP_pe	chr1	9727067	9727488	422	9727370	17	9.99923	5.32289
Nc-1-IP_pe	chr1	9734763	9735290	528	9735159	15	9.51988	5.45022
Nc-1-IP_pe	chr1	9772043	9772417	375	9772334	7	3.06442	2.73501
Nc-1-IP_pe	chr1	9823294	9823983	690	9823781	10	5.23357	3.76063
Nc-1-IP_pe	chr1	9834222	9834513	292	9834361	8	3.58997	2.97809
Nc-1-IP_pe	chr1	9835512	9836305	794	9836041	14	5.03085	3.16286
Nc-1-IP_pe	chr1	9843847	9844484	638	9843853	9	2.93173	2.4848
Nc-1-IP_pe	chr1	9850648	9851132	485	9851085	10	3.4604	2.70797
Nc-1-IP_pe	chr1	9869849	9870307	459	9870018	12	6.56855	4.26509
Nc-1-IP_pe	chr1	9874022	9874654	633	9874209	15	8.84797	5.06275
Nc-1-IP_pe	chr1	9895332	9895606	275	9895457	9	3.66792	2.92434
Nc-1-IP_pe	chr1	9900240	9900912	673	9900591	9	4.46937	3.41876
Nc-1-IP_pe	chr1	9901627	9901962	336	9901764	9	4.46937	3.41876
Nc-1-IP_pe	chr1	9941356	9941656	301	9941656	8	3.08755	2.66145
Nc-1-IP_pe	chr1	9943078	9943619	542	9943396	39	30.915	10.3852
Nc-1-IP_pe	chr1	9956479	9956882	404	9956791	9	2.89249	2.46179
Nc-1-IP_pe	chr1	9973292	9973640	349	9973365	11	6.03439	4.10251
Nc-1-IP_pe	chr1	9977996	9978410	415	9978076	9	3.72192	2.95716
Nc-1-IP_pe	chr1	10016999	10017501	503	10017447	9	4.46937	3.41876
Nc-1-IP_pe	chr1	10028666	10029030	365	10028753	14	4.60011	2.95114
Nc-1-IP_pe	chr1	10053512	10053803	292	10053631	9	4.46937	3.41876
Nc-1-IP_pe	chr1	10059501	10059939	439	10059652	13	6.62436	4.14003

Nc-1-IP_pe	chr1	10068566	10068820	255	10068618	12	6.86906	4.44438
Nc-1-IP_pe	chr1	10091644	10092309	666	10092178	10	4.39571	3.25288
Nc-1-IP_pe	chr1	10106289	10106591	303	10106412	10	3.89064	2.95552
Nc-1-IP_pe	chr1	10131143	10131498	356	10131466	8	3.64414	3.01254
Nc-1-IP_pe	chr1	10166025	10166301	277	10166126	8	3.56021	2.95918
Nc-1-IP_pe	chr1	10171289	10171869	581	10171593	10	5.23357	3.76063
Nc-1-IP_pe	chr1	10421915	10422378	464	10421990	8	3.56021	2.95918
Nc-1-IP_pe	chr1	10430951	10431416	466	10431234	9	4.38615	3.36674
Nc-1-IP_pe	chr1	10472279	10472580	302	10472380	12	4.70082	3.20033
Nc-1-IP_pe	chr1	10474532	10475177	646	10475018	16	8.26996	4.56762
Nc-1-IP_pe	chr1	10629939	10630531	593	10630130	11	5.52654	3.79706
Nc-1-IP_pe	chr1	10634980	10635627	648	10635556	8	3.14944	2.70014
Nc-1-IP_pe	chr1	10636104	10636479	376	10636293	13	6.72991	4.20021
Nc-1-IP_pe	chr1	10638213	10638549	337	10638413	13	7.65891	4.74103
Nc-1-IP_pe	chr1	10639378	10640174	797	10639526	15	9.19325	5.26076
Nc-1-IP_pe	chr1	10646008	10646966	959	10646679	12	5.23082	3.49289
Nc-1-IP_pe	chr1	10654241	10654854	614	10654481	9	4.35492	3.34727
Nc-1-IP_pe	chr1	10668762	10669283	522	10668956	9	3.66792	2.92434
Nc-1-IP_pe	chr1	10673784	10674403	620	10674269	13	4.02562	2.7544
Nc-1-IP_pe	chr1	10676965	10677256	292	10677045	14	6.13058	3.7272
Nc-1-IP_pe	chr1	10677740	10678152	413	10678069	10	4.71266	3.44298
Nc-1-IP_pe	chr1	10686818	10687370	553	10687247	11	3.64839	2.72584
Nc-1-IP_pe	chr1	10710552	10711055	504	10710741	11	5.76039	3.93701
Nc-1-IP_pe	chr1	10715351	10715747	397	10715507	12	4.70082	3.20033
Nc-1-IP_pe	chr1	10716175	10716626	452	10716414	11	5.7051	3.90381
Nc-1-IP_pe	chr1	10716988	10717273	286	10717051	10	4.47495	3.30017
Nc-1-IP_pe	chr1	10719521	10719954	434	10719612	9	4.00426	3.12999
Nc-1-IP_pe	chr1	10744543	10744809	267	10744670	13	4.86362	3.18015
Nc-1-IP_pe	chr1	10766081	10766480	400	10766389	9	3.27326	2.68684
Nc-1-IP_pe	chr1	10772375	10772632	258	10772479	10	4.99888	3.61677
Nc-1-IP_pe	chr1	10782617	10782872	256	10782694	10	5.14046	3.70342
Nc-1-IP_pe	chr1	10786037	10786443	407	10786148	10	5.23357	3.76063
Nc-1-IP_pe	chr1	10788679	10789166	488	10788822	14	7.9745	4.74633
Nc-1-IP_pe	chr1	10794974	10795586	613	10795544	14	4.21449	2.76599
Nc-1-IP_pe	chr1	10816601	10816858	258	10816647	11	4.23076	3.04719
Nc-1-IP_pe	chr1	10825679	10825986	308	10825964	12	3.47963	2.55766
Nc-1-IP_pe	chr1	10830075	10830432	358	10830281	10	4.98602	3.60892
Nc-1-IP_pe	chr1	10835488	10835850	363	10835619	10	4.39571	3.25288
Nc-1-IP_pe	chr1	10849286	10849608	323	10849462	11	6.03439	4.10251
Nc-1-IP_pe	chr1	10855329	10855746	418	10855695	10	3.4604	2.70797
Nc-1-IP_pe	chr1	10861549	10861848	300	10861760	10	4.99888	3.61677
Nc-1-IP_pe	chr1	10862971	10863333	363	10863277	9	2.89249	2.46179
Nc-1-IP_pe	chr1	10873432	10873710	279	10873536	9	4.07387	3.17289
Nc-1-IP_pe	chr1	10880225	10880479	255	10880422	9	4.46937	3.41876
Nc-1-IP_pe	chr1	10881448	10881860	413	10881689	12	6.50787	4.22912

Nc-1-IP_pe	chr1	10898319	10899015	697	10898866	12	5.23082	3.49289
Nc-1-IP_pe	chr1	10906100	10906384	285	10906213	12	5.99895	3.93077
Nc-1-IP_pe	chr1	10906923	10907229	307	10907066	10	4.98602	3.60892
Nc-1-IP_pe	chr1	10910028	10910311	284	10910292	9	2.89249	2.46179
Nc-1-IP_pe	chr1	10911386	10911760	375	10911650	9	4.46937	3.41876
Nc-1-IP_pe	chr1	10931735	10932003	269	10931885	10	5.23357	3.76063
Nc-1-IP_pe	chr1	10935430	10936197	768	10935932	8	3.74505	3.07688
Nc-1-IP_pe	chr1	10937276	10938030	755	10937522	14	7.42849	4.43574
Nc-1-IP_pe	chr1	10939909	10940176	268	10939973	9	4.33944	3.33761
Nc-1-IP_pe	chr1	10948643	10948911	269	10948777	12	5.77527	3.80164
Nc-1-IP_pe	chr1	10960293	10960626	334	10960537	14	5.51541	3.4073
Nc-1-IP_pe	chr1	10963923	10964205	283	10964113	12	5.84943	3.84431
Nc-1-IP_pe	chr1	10973752	10974036	285	10973910	8	3.56021	2.95918
Nc-1-IP_pe	chr1	10980767	10981102	336	10980971	10	5.23357	3.76063
Nc-1-IP_pe	chr1	11012609	11013072	464	11012752	21	10.0475	4.66519
Nc-1-IP_pe	chr1	11034782	11035091	310	11034964	9	4.46937	3.41876
Nc-1-IP_pe	chr1	11054705	11055040	336	11054850	14	7.34079	4.38651
Nc-1-IP_pe	chr1	11056801	11057084	284	11056855	10	5.07094	3.66082
Nc-1-IP_pe	chr1	11058933	11059790	858	11059510	12	6.71369	4.35145
Nc-1-IP_pe	chr1	11060219	11060481	263	11060424	8	3.56021	2.95918
Nc-1-IP_pe	chr1	11063359	11063795	437	11063504	10	4.39571	3.25288
Nc-1-IP_pe	chr1	11099444	11099863	420	11099697	14	5.03085	3.16286
Nc-1-IP_pe	chr1	11139336	11139825	490	11139500	10	5.23357	3.76063
Nc-1-IP_pe	chr1	11161047	11161556	510	11161142	13	4.86362	3.18015
Nc-1-IP_pe	chr1	11262206	11262589	384	11262355	13	4.86362	3.18015
Nc-1-IP_pe	chr1	11267373	11267645	273	11267515	10	5.23357	3.76063
Nc-1-IP_pe	chr1	11273206	11273489	284	11273326	20	12.9463	6.30032
Nc-1-IP_pe	chr1	11274304	11274763	460	11274490	11	6.03439	4.10251
Nc-1-IP_pe	chr1	11277440	11277803	364	11277484	9	4.46937	3.41876
Nc-1-IP_pe	chr1	11303705	11304039	335	11303857	13	5.9487	3.76157
Nc-1-IP_pe	chr1	11306658	11307016	359	11306779	11	6.03439	4.10251
Nc-1-IP_pe	chr1	11309123	11309719	597	11309558	11	6.03439	4.10251
Nc-1-IP_pe	chr1	11316413	11316721	309	11316450	8	3.74505	3.07688
Nc-1-IP_pe	chr1	11328860	11329169	310	11329066	8	3.74505	3.07688
Nc-1-IP_pe	chr1	11334363	11334700	338	11334479	8	3.74505	3.07688
Nc-1-IP_pe	chr1	11352098	11352383	286	11352201	10	5.23357	3.76063
Nc-1-IP_pe	chr1	11355539	11355843	305	11355594	9	4.46937	3.41876
Nc-1-IP_pe	chr1	11387854	11388330	477	11387988	8	3.74505	3.07688
Nc-1-IP_pe	chr1	11391810	11392163	354	11392029	8	3.74505	3.07688
Nc-1-IP_pe	chr1	11395811	11396212	402	11395881	10	5.23357	3.76063
Nc-1-IP_pe	chr1	11440819	11441250	432	11440948	11	6.03439	4.10251
Nc-1-IP_pe	chr1	11473588	11473840	253	11473687	9	4.46937	3.41876
Nc-1-IP_pe	chr1	11478845	11480238	1394	11479087	15	8.26005	4.73146
Nc-1-IP_pe	chr1	11488215	11488492	278	11488386	9	4.46937	3.41876
Nc-1-IP_pe	chr1	11500949	11501388	440	11501124	10	5.23357	3.76063

Nc-1-IP_pe	chr1	11515653	11516099	447	11515887	10	4.93609	3.57849
Nc-1-IP_pe	chr1	11516948	11517244	297	11516981	9	4.46937	3.41876
Nc-1-IP_pe	chr1	11522571	11523087	517	11522678	17	5.14803	2.94914
Nc-1-IP_pe	chr1	11531988	11532291	304	11532193	12	6.86906	4.44438
Nc-1-IP_pe	chr1	11535811	11536092	282	11535884	9	4.05981	3.16422
Nc-1-IP_pe	chr1	11541401	11541655	255	11541544	11	5.77463	3.94557
Nc-1-IP_pe	chr1	11564055	11564450	396	11564310	12	4.70082	3.20033
Nc-1-IP_pe	chr1	11592914	11593647	734	11593082	10	5.23357	3.76063
Nc-1-IP_pe	chr1	11601843	11602405	563	11601922	8	3.74505	3.07688
Nc-1-IP_pe	chr1	11621939	11622450	512	11622424	10	5.23357	3.76063
Nc-1-IP_pe	chr1	11630183	11630459	277	11630286	9	4.46937	3.41876
Nc-1-IP_pe	chr1	11634201	11634555	355	11634291	10	5.23357	3.76063
Nc-1-IP_pe	chr1	11635345	11635630	286	11635401	8	3.74505	3.07688
Nc-1-IP_pe	chr1	11636009	11636333	325	11636250	9	4.25966	3.28797
Nc-1-IP_pe	chr1	11646107	11646408	302	11646242	9	4.3087	3.31847
Nc-1-IP_pe	chr1	11649999	11650973	975	11650522	13	4.41954	2.952
Nc-1-IP_pe	chr1	11652508	11653185	678	11652775	16	7.53857	4.18505
Nc-1-IP_pe	chr1	11658685	11658995	311	11658709	10	4.95265	3.58858
Nc-1-IP_pe	chr1	11667182	11667614	433	11667376	9	4.46937	3.41876
Nc-1-IP_pe	chr1	11668961	11669247	287	11669243	7	2.9043	2.63038
Nc-1-IP_pe	chr1	11670841	11671141	301	11671071	8	3.74505	3.07688
Nc-1-IP_pe	chr1	11691080	11691471	392	11691190	9	4.25966	3.28797
Nc-1-IP_pe	chr1	11707176	11707904	729	11707448	10	5.23357	3.76063
Nc-1-IP_pe	chr1	11711959	11712361	403	11712055	9	4.46937	3.41876
Nc-1-IP_pe	chr1	11715055	11715382	328	11715248	9	2.89249	2.46179
Nc-1-IP_pe	chr1	11721365	11721823	459	11721416	8	3.74505	3.07688
Nc-1-IP_pe	chr1	11730211	11730496	286	11730272	9	4.25966	3.28797
Nc-1-IP_pe	chr1	11739707	11740131	425	11739814	8	3.52386	2.93611
Nc-1-IP_pe	chr1	11753511	11753787	277	11753591	13	4.86362	3.18015
Nc-1-IP_pe	chr1	11776498	11776911	414	11776753	9	4.46937	3.41876
Nc-1-IP_pe	chr1	11777350	11778036	687	11777839	10	5.23357	3.76063
Nc-1-IP_pe	chr1	11778335	11778678	344	11778504	10	5.23357	3.76063
Nc-1-IP_pe	chr1	11779491	11779978	488	11779918	9	4.46937	3.41876
Nc-1-IP_pe	chr1	11784835	11785281	447	11785094	11	5.3563	3.696
Nc-1-IP_pe	chr1	11787508	11788197	690	11787937	12	3.83688	2.74114
Nc-1-IP_pe	chr1	11796243	11796513	271	11796373	12	6.35031	4.1361
Nc-1-IP_pe	chr1	11829969	11830265	297	11830164	15	9.55381	5.47001
Nc-1-IP_pe	chr1	11841230	11841695	466	11841427	12	6.86906	4.44438
Nc-1-IP_pe	chr1	11871332	11871626	295	11871517	9	3.72192	2.95716
Nc-1-IP_pe	chr1	11878351	11878694	344	11878639	8	3.74505	3.07688
Nc-1-IP_pe	chr1	11908555	11908960	406	11908919	14	5.98179	3.64885
Nc-1-IP_pe	chr1	11916066	11916366	301	11916193	9	4.46937	3.41876
Nc-1-IP_pe	chr1	11921249	11921517	269	11921409	11	5.83542	3.98217
Nc-1-IP_pe	chr1	11922674	11923341	668	11922844	15	5.66913	3.37372
Nc-1-IP_pe	chr1	11925165	11926054	890	11925914	11	6.03439	4.10251

Nc-1-IP_pe	chr1	11932822	11933092	271	11932970	11	5.10577	3.5486
Nc-1-IP_pe	chr1	11934174	11934587	414	11934506	13	5.03208	3.26819
Nc-1-IP_pe	chr1	11936911	11937571	661	11937045	14	8.44792	5.0209
Nc-1-IP_pe	chr1	11943509	11943856	348	11943679	11	6.03439	4.10251
Nc-1-IP_pe	chr1	11950169	11950710	542	11950191	10	5.23357	3.76063
Nc-1-IP_pe	chr1	11964315	11964659	345	11964357	10	4.77495	3.48064
Nc-1-IP_pe	chr1	11980520	11980883	364	11980612	11	4.06403	2.95415
Nc-1-IP_pe	chr1	11984021	11984284	264	11984267	9	4.46937	3.41876
Nc-1-IP_pe	chr1	12001570	12002328	759	12001768	11	5.77463	3.94557
Nc-1-IP_pe	chr1	12003484	12004040	557	12003532	10	4.99888	3.61677
Nc-1-IP_pe	chr1	12008874	12009346	473	12009338	8	3.74505	3.07688
Nc-1-IP_pe	chr1	12009938	12010210	273	12010032	10	5.23357	3.76063
Nc-1-IP_pe	chr1	12010738	12011472	735	12010888	10	3.4604	2.70797
Nc-1-IP_pe	chr1	12012561	12012970	410	12012959	8	3.56021	2.95918
Nc-1-IP_pe	chr1	12020656	12021065	410	12020829	12	6.86906	4.44438
Nc-1-IP_pe	chr1	12028064	12028566	503	12028359	12	5.84943	3.84431
Nc-1-IP_pe	chr1	12029392	12029707	316	12029529	12	5.23082	3.49289
Nc-1-IP_pe	chr1	12043084	12043655	572	12043499	11	5.77463	3.94557
Nc-1-IP_pe	chr1	12047667	12048060	394	12047957	7	2.9043	2.63038
Nc-1-IP_pe	chr1	12052429	12052938	510	12052760	11	6.03439	4.10251
Nc-1-IP_pe	chr1	12057410	12057698	289	12057554	8	3.56021	2.95918
Nc-1-IP_pe	chr1	12059514	12059955	442	12059930	9	3.54889	2.85226
Nc-1-IP_pe	chr1	12062977	12063253	277	12063092	13	7.7352	4.78626
Nc-1-IP_pe	chr1	12064396	12064841	446	12064479	7	3.06442	2.73501
Nc-1-IP_pe	chr1	12068131	12068738	608	12068474	12	6.86906	4.44438
Nc-1-IP_pe	chr1	12079015	12079692	678	12079531	9	4.25966	3.28797
Nc-1-IP_pe	chr1	12096875	12097203	329	12097022	14	8.63073	5.12814
Nc-1-IP_pe	chr1	12111548	12111865	318	12111707	12	6.86906	4.44438
Nc-1-IP_pe	chr1	12123038	12123698	661	12123627	9	4.46937	3.41876
Nc-1-IP_pe	chr1	12127211	12127568	358	12127430	11	6.03439	4.10251
Nc-1-IP_pe	chr1	12130293	12130545	253	12130381	8	3.74505	3.07688
Nc-1-IP_pe	chr1	12167127	12167407	281	12167254	13	5.36853	3.44651
Nc-1-IP_pe	chr1	12168785	12169133	349	12168967	8	3.52386	2.93611
Nc-1-IP_pe	chr1	12170392	12170693	302	12170586	8	3.56021	2.95918
Nc-1-IP_pe	chr1	12176812	12177666	855	12177068	10	5.23357	3.76063
Nc-1-IP_pe	chr1	12179198	12179450	253	12179319	9	4.46937	3.41876
Nc-1-IP_pe	chr1	12180010	12180284	275	12180143	7	3.06442	2.73501
Nc-1-IP_pe	chr1	12182041	12182294	254	12182088	9	4.46937	3.41876
Nc-1-IP_pe	chr1	12188652	12188959	308	12188953	8	3.74505	3.07688
Nc-1-IP_pe	chr1	12192631	12193120	490	12192750	14	7.34079	4.38651
Nc-1-IP_pe	chr1	12194405	12194839	435	12194620	9	4.05981	3.16422
Nc-1-IP_pe	chr1	12202001	12202533	533	12202146	14	7.42849	4.43574
Nc-1-IP_pe	chr1	12205218	12205632	415	12205549	9	4.46937	3.41876
Nc-1-IP_pe	chr1	12212096	12212516	421	12212268	11	5.76039	3.93701
Nc-1-IP_pe	chr1	12215059	12215330	272	12215221	11	4.54404	3.2242

Nc-1-IP_pe	chr1	12230747	12231197	451	12230961	14	7.54286	4.50023
Nc-1-IP_pe	chr1	12250741	12251197	457	12251097	11	4.06403	2.95415
Nc-1-IP_pe	chr1	12323894	12324468	575	12323979	9	4.46937	3.41876
Nc-1-IP_pe	chr1	12503697	12503995	299	12503945	8	3.56021	2.95918
Nc-1-IP_pe	chr1	12506795	12507252	458	12507078	10	3.4604	2.70797
Nc-1-IP_pe	chr1	12513018	12513311	294	12513164	9	2.89249	2.46179
Nc-1-IP_pe	chr1	12520605	12520928	324	12520891	10	5.23357	3.76063
Nc-1-IP_pe	chr1	12540676	12540996	321	12540844	13	5.42865	3.47872
Nc-1-IP_pe	chr1	12547337	12548455	1119	12548049	17	9.09431	4.8363
Nc-1-IP_pe	chr1	12555127	12555638	512	12555487	9	3.72192	2.95716
Nc-1-IP_pe	chr1	12559991	12560849	859	12560652	12	6.31185	4.11348
Nc-1-IP_pe	chr1	12574362	12574745	384	12574395	11	3.28475	2.53029
Nc-1-IP_pe	chr1	12578843	12579313	471	12579008	11	5.52654	3.79706
Nc-1-IP_pe	chr1	12601436	12602200	765	12601651	9	4.20361	3.25317
Nc-1-IP_pe	chr1	12616938	12617246	309	12617089	13	7.6824	4.75494
Nc-1-IP_pe	chr1	12630843	12631151	309	12631105	9	4.46937	3.41876
Nc-1-IP_pe	chr1	12633143	12633493	351	12633429	9	4.46937	3.41876
Nc-1-IP_pe	chr1	12652224	12652586	363	12652426	10	5.23357	3.76063
Nc-1-IP_pe	chr1	12810047	12810356	310	12810104	10	5.23357	3.76063
Nc-1-IP_pe	chr1	12825700	12826200	501	12825786	10	4.33501	3.21677
Nc-1-IP_pe	chr1	13243553	13243876	324	13243630	13	5.9487	3.76157
Nc-1-IP_pe	chr1	13246095	13246635	541	13246131	11	3.64839	2.72584
Nc-1-IP_pe	chr1	13269026	13269334	309	13269207	11	6.03439	4.10251
Nc-1-IP_pe	chr1	13270783	13271447	665	13270982	9	4.46937	3.41876
Nc-1-IP_pe	chr1	13295985	13296248	264	13296168	12	3.83688	2.74114
Nc-1-IP_pe	chr1	13298675	13299046	372	13298946	14	6.61011	3.98382
Nc-1-IP_pe	chr1	13308264	13308549	286	13308301	14	5.03085	3.16286
Nc-1-IP_pe	chr1	13318405	13318659	255	13318580	7	3.06442	2.73501
Nc-1-IP_pe	chr1	13328414	13328692	279	13328512	15	6.19428	3.63445
Nc-1-IP_pe	chr1	13329119	13329607	489	13329298	13	4.41954	2.952
Nc-1-IP_pe	chr1	13371342	13371673	332	13371421	10	5.23357	3.76063
Nc-1-IP_pe	chr1	13394834	13395127	294	13395063	21	9.29881	4.32834
Nc-1-IP_pe	chr1	13430609	13431138	530	13431001	13	6.62436	4.14003
Nc-1-IP_pe	chr1	13595712	13595975	264	13595804	9	4.46937	3.41876
Nc-1-IP_pe	chr1	13657395	13657794	400	13657490	8	3.08755	2.66145
Nc-1-IP_pe	chr1	13658159	13658540	382	13658446	14	8.29543	4.93196
Nc-1-IP_pe	chr1	13668161	13668440	280	13668440	8	3.08755	2.66145
Nc-1-IP_pe	chr1	13801662	13802060	399	13801875	8	3.51085	2.92785
Nc-1-IP_pe	chr1	13811784	13812036	253	13811892	9	4.25966	3.28797
Nc-1-IP_pe	chr1	13814552	13814956	405	13814794	13	7.7352	4.78626
Nc-1-IP_pe	chr1	13960581	13960846	266	13960720	11	5.77463	3.94557
Nc-1-IP_pe	chr1	14415678	14416001	324	14415797	9	4.46937	3.41876
Nc-1-IP_pe	chr1	14519559	14519814	256	14519672	7	2.9043	2.63038
Nc-1-IP_pe	chr1	14599208	14599494	287	14599462	7	3.06442	2.73501
Nc-1-IP_pe	chr1	14665023	14665669	647	14665539	11	6.03439	4.10251

Nc-1-IP_pe	chr1	14677449	14677801	353	14677530	8	3.74505	3.07688
Nc-1-IP_pe	chr1	14901047	14901486	440	14901397	10	5.23357	3.76063
Nc-1-IP_pe	chr1	14904501	14904761	261	14904564	12	6.58417	4.27436
Nc-1-IP_pe	chr1	14920977	14921390	414	14921297	8	3.74505	3.07688
Nc-1-IP_pe	chr1	14923306	14923575	270	14923568	9	4.05981	3.16422
Nc-1-IP_pe	chr1	14924727	14925092	366	14925022	11	4.54404	3.2242
Nc-1-IP_pe	chr1	14950115	14950864	750	14950684	9	4.46937	3.41876
Nc-1-IP_pe	chr1	14978048	14978409	362	14978255	8	3.56021	2.95918
Nc-1-IP_pe	chr1	14997326	14997872	547	14997357	9	3.72192	2.95716
Nc-1-IP_pe	chr1	14999074	14999444	371	14999209	8	3.74505	3.07688
Nc-1-IP_pe	chr1	15022106	15022508	403	15022156	8	3.74505	3.07688
Nc-1-IP_pe	chr1	15075766	15076269	504	15075847	9	4.46937	3.41876
Nc-1-IP_pe	chr1	15096492	15096861	370	15096684	10	5.23357	3.76063
Nc-1-IP_pe	chr1	15110586	15111056	471	15110746	8	3.74505	3.07688
Nc-1-IP_pe	chr1	15119876	15120269	394	15120123	9	3.72192	2.95716
Nc-1-IP_pe	chr1	15153748	15154335	588	15154145	14	5.51541	3.4073
Nc-1-IP_pe	chr1	15223267	15223550	284	15223527	8	3.64414	3.01254
Nc-1-IP_pe	chr1	15259634	15259915	282	15259836	8	3.74505	3.07688
Nc-1-IP_pe	chr1	15260820	15261399	580	15260972	11	5.10577	3.5486
Nc-1-IP_pe	chr1	15276727	15276985	259	15276883	10	4.33501	3.21677
Nc-1-IP_pe	chr1	15289237	15289593	357	15289451	8	3.08755	2.66145
Nc-1-IP_pe	chr1	15309269	15309966	698	15309360	8	3.56021	2.95918
Nc-1-IP_pe	chr1	15346721	15347852	1132	15346851	10	5.23357	3.76063
Nc-1-IP_pe	chr1	15352797	15353055	259	15352803	8	2.93592	2.56703
Nc-1-IP_pe	chr1	15390672	15391017	346	15390840	10	5.23357	3.76063
Nc-1-IP_pe	chr1	15401706	15401965	260	15401791	10	5.23357	3.76063
Nc-1-IP_pe	chr1	15409023	15409325	303	15409162	12	6.58417	4.27436
Nc-1-IP_pe	chr1	15410419	15410797	379	15410551	8	3.74505	3.07688
Nc-1-IP_pe	chr1	15419057	15419456	400	15419407	11	5.03835	3.5092
Nc-1-IP_pe	chr1	15419711	15419975	265	15419825	9	4.24819	3.28084
Nc-1-IP_pe	chr1	15425346	15425735	390	15425566	11	5.1152	3.55411
Nc-1-IP_pe	chr1	15431088	15431911	824	15431783	14	7.75387	4.62
Nc-1-IP_pe	chr1	15433411	15433868	458	15433481	10	5.01973	3.6295
Nc-1-IP_pe	chr1	15444375	15444863	489	15444512	9	4.46937	3.41876
Nc-1-IP_pe	chr1	15452268	15453023	756	15452716	14	7.42849	4.43574
Nc-1-IP_pe	chr1	15465874	15466221	348	15466107	10	5.23357	3.76063
Nc-1-IP_pe	chr1	15467528	15467822	295	15467618	11	5.52654	3.79706
Nc-1-IP_pe	chr1	15469792	15470208	417	15470072	9	4.46937	3.41876
Nc-1-IP_pe	chr1	15472788	15473422	635	15472887	11	5.3563	3.696
Nc-1-IP_pe	chr1	15481004	15481261	258	15481135	8	3.74505	3.07688
Nc-1-IP_pe	chr1	15491776	15492037	262	15491920	9	4.46937	3.41876
Nc-1-IP_pe	chr1	15506041	15506322	282	15506121	7	3.06442	2.73501
Nc-1-IP_pe	chr1	15555769	15556076	308	15555841	9	3.72192	2.95716
Nc-1-IP_pe	chr1	15683246	15683573	328	15683352	11	6.03439	4.10251
Nc-1-IP_pe	chr1	15701985	15702385	401	15702328	13	5.36853	3.44651

Nc-1-IP_pe	chr1	15705483	15705819	337	15705589	10	5.23357	3.76063
Nc-1-IP_pe	chr1	15722895	15723233	339	15722946	8	3.74505	3.07688
Nc-1-IP_pe	chr1	15725192	15725717	526	15725609	11	5.10577	3.5486
Nc-1-IP_pe	chr1	15728229	15728621	393	15728431	8	3.08755	2.66145
Nc-1-IP_pe	chr1	15737861	15738340	480	15737988	13	7.00513	4.3584
Nc-1-IP_pe	chr1	15739855	15740209	355	15739893	8	3.58997	2.97809
Nc-1-IP_pe	chr1	15742928	15743330	403	15743231	9	4.46937	3.41876
Nc-1-IP_pe	chr1	15745084	15745474	391	15745376	12	6.31185	4.11348
Nc-1-IP_pe	chr1	15758777	15759404	628	15759198	16	6.8987	3.86161
Nc-1-IP_pe	chr1	15770279	15770782	504	15770384	12	5.84943	3.84431
Nc-1-IP_pe	chr1	15774746	15775013	268	15774988	8	3.74505	3.07688
Nc-1-IP_pe	chr1	15778775	15779031	257	15778924	8	3.74505	3.07688
Nc-1-IP_pe	chr1	15781063	15781355	293	15781210	9	3.27326	2.68684
Nc-1-IP_pe	chr1	15784562	15784838	277	15784665	11	5.3563	3.696
Nc-1-IP_pe	chr1	15941230	15941737	508	15941434	9	4.25966	3.28797
Nc-1-IP_pe	chr1	15942594	15942889	296	15942786	13	6.02837	3.80557
Nc-1-IP_pe	chr1	15948095	15948372	278	15948236	13	6.06081	3.82353
Nc-1-IP_pe	chr1	15958631	15958970	340	15958768	9	4.35492	3.34727
Nc-1-IP_pe	chr1	15961555	15961870	316	15961684	11	6.03439	4.10251
Nc-1-IP_pe	chr1	15972085	15972338	254	15972127	10	4.99888	3.61677
Nc-1-IP_pe	chr1	15975551	15975982	432	15975862	9	3.72192	2.95716
Nc-1-IP_pe	chr1	15991446	15992316	871	15991545	13	7.34205	4.55444
Nc-1-IP_pe	chr1	16006143	16006406	264	16006318	12	3.83688	2.74114
Nc-1-IP_pe	chr1	16021177	16021450	274	16021329	16	5.37454	3.13479
Nc-1-IP_pe	chr1	16022428	16023008	581	16022678	8	3.74178	3.0748
Nc-1-IP_pe	chr1	16027505	16027820	316	16027642	11	5.81652	3.97078
Nc-1-IP_pe	chr1	16031660	16032647	988	16032454	15	7.75613	4.45384
Nc-1-IP_pe	chr1	16034640	16034967	328	16034753	11	4.40231	3.14376
Nc-1-IP_pe	chr1	16036275	16037613	1339	16036711	16	6.8987	3.86161
Nc-1-IP_pe	chr1	16038266	16038552	287	16038403	13	4.45253	2.96876
Nc-1-IP_pe	chr1	16041941	16042203	263	16042109	13	5.40091	3.46384
Nc-1-IP_pe	chr1	16045058	16045655	598	16045272	15	6.19428	3.63445
Nc-1-IP_pe	chr1	16046558	16047530	973	16047082	13	5.99619	3.78778
Nc-1-IP_pe	chr1	16048655	16049040	386	16048828	15	7.46956	4.29873
Nc-1-IP_pe	chr1	16050845	16051868	1024	16051388	16	6.33285	3.58457
Nc-1-IP_pe	chr1	16057180	16057666	487	16057396	10	3.13988	2.52708
Nc-1-IP_pe	chr1	16058324	16058798	475	16058649	15	4.40338	2.77622
Nc-1-IP_pe	chr1	16066019	16066450	432	16066351	11	4.54404	3.2242
Nc-1-IP_pe	chr1	16066861	16067721	861	16067469	13	4.41954	2.952
Nc-1-IP_pe	chr1	16087800	16088222	423	16088000	12	5.18731	3.46856
Nc-1-IP_pe	chr1	16088600	16089376	777	16089249	14	5.51541	3.4073
Nc-1-IP_pe	chr1	16090977	16091366	390	16090999	12	3.16137	2.39719
Nc-1-IP_pe	chr1	16097725	16098017	293	16097877	16	8.17084	4.51499
Nc-1-IP_pe	chr1	16118681	16119208	528	16118798	13	4.67336	3.0817
Nc-1-IP_pe	chr1	16131834	16132723	890	16131948	13	4.86362	3.18015

Nc-1-IP_pe	chr1	16137742	16138807	1066	16138585	16	9.39429	5.18085
Nc-1-IP_pe	chr1	16140156	16140432	277	16140267	12	4.24064	2.95299
Nc-1-IP_pe	chr1	16144426	16144680	255	16144586	12	4.96504	3.34516
Nc-1-IP_pe	chr1	16150512	16150888	377	16150575	12	5.39344	3.58426
Nc-1-IP_pe	chr1	16162254	16162666	413	16162285	9	4.46937	3.41876
Nc-1-IP_pe	chr1	16165395	16165834	440	16165710	10	3.4604	2.70797
Nc-1-IP_pe	chr1	16176896	16177231	336	16177052	11	4.41492	3.1509
Nc-1-IP_pe	chr1	16184886	16185309	424	16185227	11	4.47871	3.18705
Nc-1-IP_pe	chr1	16191718	16192305	588	16191889	12	6.86906	4.44438
Nc-1-IP_pe	chr1	16201896	16202327	432	16202243	9	4.29346	3.30898
Nc-1-IP_pe	chr1	16205037	16205704	668	16205305	11	5.2904	3.65707
Nc-1-IP_pe	chr1	16206791	16207099	309	16206847	15	6.78905	3.93887
Nc-1-IP_pe	chr1	16226823	16227276	454	16227121	11	5.79772	3.95946
Nc-1-IP_pe	chr1	16236938	16237336	399	16237160	18	8.37884	4.31591
Nc-1-IP_pe	chr1	16244168	16244471	304	16244384	8	3.74505	3.07688
Nc-1-IP_pe	chr1	16305270	16305627	358	16305467	9	4.46937	3.41876
Nc-1-IP_pe	chr1	16331737	16332042	306	16331832	10	5.23357	3.76063
Nc-1-IP_pe	chr1	16359117	16359743	627	16359522	9	4.46937	3.41876
Nc-1-IP_pe	chr1	16367262	16367836	575	16367454	15	6.19428	3.63445
Nc-1-IP_pe	chr1	16392936	16393482	547	16393443	11	5.95085	4.05188
Nc-1-IP_pe	chr1	16407932	16408215	284	16407934	8	3.69948	3.0478
Nc-1-IP_pe	chr1	16417379	16418213	835	16418109	11	4.54404	3.2242
Nc-1-IP_pe	chr1	16425228	16425723	496	16425519	10	4.99888	3.61677
Nc-1-IP_pe	chr1	16433216	16433518	303	16433430	10	4.93609	3.57849
Nc-1-IP_pe	chr1	16473979	16474391	413	16474259	11	6.03439	4.10251
Nc-1-IP_pe	chr1	16476350	16477165	816	16477015	10	4.99888	3.61677
Nc-1-IP_pe	chr1	16483897	16484742	846	16484616	10	3.60969	2.79325
Nc-1-IP_pe	chr1	16490335	16490893	559	16490696	8	3.74505	3.07688
Nc-1-IP_pe	chr1	16498362	16499269	908	16498875	15	5.83691	3.4562
Nc-1-IP_pe	chr1	16514047	16514467	421	16514063	17	4.05031	2.49246
Nc-1-IP_pe	chr1	16532762	16533474	713	16532871	20	6.49719	3.259
Nc-1-IP_pe	chr1	16534885	16535145	261	16534994	29	7.66167	3.03012
Nc-1-IP_pe	chr1	16535597	16536179	583	16535779	33	8.45274	3.03383
Nc-1-IP_pe	chr1	16536583	16536975	393	16536974	19	2.87318	1.96343
Nc-1-IP_pe	chr1	16545100	16545627	528	16545355	10	4.39571	3.25288
Nc-1-IP_pe	chr1	16553939	16554195	257	16554025	9	4.25966	3.28797
Nc-1-IP_pe	chr1	16566935	16567294	360	16567019	31	6.5096	2.61702
Nc-1-IP_pe	chr1	16584550	16584841	292	16584735	14	5.65814	3.48057
Nc-1-IP_pe	chr1	16587303	16587636	334	16587494	17	5.9911	3.31919
Nc-1-IP_pe	chr1	16613011	16613853	843	16613571	32	7.59686	2.85784
Nc-1-IP_pe	chr1	16618857	16619119	263	16618947	23	7.62055	3.43424
Nc-1-IP_pe	chr1	16619607	16619918	312	16619818	22	4.91313	2.54525
Nc-1-IP_pe	chr1	16621279	16622028	750	16621375	21	5.27092	2.72369
Nc-1-IP_pe	chr1	16622429	16622797	369	16622565	21	5.14251	2.67786
Nc-1-IP_pe	chr1	16628460	16628866	407	16628742	18	3.29891	2.1529

Nc-1-IP_pe	chr1	16629670	16631373	1704	16630332	21	7.07439	3.40032
Nc-1-IP_pe	chr1	16632787	16633067	281	16632889	15	4.23553	2.70016
Nc-1-IP_pe	chr1	16637961	16638903	943	16638193	24	12.0237	5.09592
Nc-1-IP_pe	chr1	16642081	16643042	962	16642884	20	4.15754	2.37952
Nc-1-IP_pe	chr1	16644305	16644735	431	16644619	25	6.98838	3.0642
Nc-1-IP_pe	chr1	16645324	16646028	705	16645913	18	4.99031	2.80908
Nc-1-IP_pe	chr1	16646638	16648360	1723	16647329	40	9.73959	3.01724
Nc-1-IP_pe	chr1	16648617	16649471	855	16648917	26	6.47412	2.84038
Nc-1-IP_pe	chr1	16649875	16650244	370	16650096	33	15.505	5.19263
Nc-1-IP_pe	chr1	16656427	16657457	1031	16657123	30	9.49448	3.51262
Nc-1-IP_pe	chr1	16657951	16658507	557	16658024	30	6.32135	2.6078
Nc-1-IP_pe	chr1	16672533	16673185	653	16673091	25	5.37675	2.55246
Nc-1-IP_pe	chr1	16673456	16673860	405	16673736	18	4.62526	2.6628
Nc-1-IP_pe	chr1	16691595	16692018	424	16691786	12	4.99209	3.3601
Nc-1-IP_pe	chr1	16692695	16693376	682	16693045	13	5.15976	3.33548
Nc-1-IP_pe	chr1	16693757	16694605	849	16694032	13	4.41954	2.952
Nc-1-IP_pe	chr1	16696467	16696843	377	16696652	18	7.1522	3.73811
Nc-1-IP_pe	chr1	16697708	16697970	263	16697786	16	5.82165	3.34162
Nc-1-IP_pe	chr1	16700247	16701164	918	16700370	17	4.78101	2.79343
Nc-1-IP_pe	chr1	16704450	16705090	641	16704654	16	4.59224	2.7853
Nc-1-IP_pe	chr1	16709527	16709844	318	16709602	22	8.24538	3.76835
Nc-1-IP_pe	chr1	16710518	16710771	254	16710525	14	3.33731	2.35937
Nc-1-IP_pe	chr1	16711966	16712552	587	16712299	18	6.4429	3.42096
Nc-1-IP_pe	chr1	16713609	16714192	584	16713791	17	5.9775	3.31308
Nc-1-IP_pe	chr1	16714768	16715078	311	16714907	14	4.19452	2.75652
Nc-1-IP_pe	chr1	16718096	16718718	623	16718258	17	4.78101	2.79343
Nc-1-IP_pe	chr1	16724862	16725450	589	16725311	23	8.26098	3.67508
Nc-1-IP_pe	chr1	16727335	16728243	909	16727810	36	12.3242	3.89237
Nc-1-IP_pe	chr1	16734140	16734797	658	16734326	21	4.44756	2.43458
Nc-1-IP_pe	chr1	16748863	16749209	347	16749037	30	9.49448	3.51262
Nc-1-IP_pe	chr1	16749869	16751039	1171	16750105	38	14.1179	4.23486
Nc-1-IP_pe	chr1	16758318	16758704	387	16758534	25	8.42164	3.55345
Nc-1-IP_pe	chr1	16759171	16759771	601	16759289	21	4.34782	2.40029
Nc-1-IP_pe	chr1	16760612	16761673	1062	16760876	25	4.60609	2.31999
Nc-1-IP_pe	chr1	16765609	16766434	826	16765745	25	4.84935	2.39263
Nc-1-IP_pe	chr1	16770994	16771572	579	16771228	28	6.5317	2.75496
Nc-1-IP_pe	chr1	16775927	16776819	893	16776298	32	9.13953	3.27595
Nc-1-IP_pe	chr1	16777864	16778146	283	16778034	24	4.19057	2.23076
Nc-1-IP_pe	chr1	16789939	16790307	369	16790039	18	7.61002	3.94942
Nc-1-IP_pe	chr1	16796252	16796670	419	16796387	28	7.90909	3.16402
Nc-1-IP_pe	chr1	16856768	16857256	489	16857210	11	6.03439	4.10251
Nc-1-IP_pe	chr1	16859640	16859968	329	16859784	17	7.6273	4.08876
Nc-1-IP_pe	chr1	16862264	16862633	370	16862464	12	6.25484	4.08002
Nc-1-IP_pe	chr1	16872268	16873119	852	16872736	21	5.18226	2.69201
Nc-1-IP_pe	chr1	16874833	16875353	521	16874889	28	7.18321	2.94535

Nc-1-IP_pe	chr1	16877441	16877795	355	16877593	22	6.84408	3.22845
Nc-1-IP_pe	chr1	16895770	16896062	293	16895934	19	9.15219	4.54307
Nc-1-IP_pe	chr1	16906509	16907104	596	16907032	8	3.74505	3.07688
Nc-1-IP_pe	chr1	16913065	16913488	424	16913248	9	4.46937	3.41876
Nc-1-IP_pe	chr1	16916224	16916643	420	16916493	10	4.39571	3.25288
Nc-1-IP_pe	chr1	16921321	16921802	482	16921722	10	5.10552	3.682
Nc-1-IP_pe	chr1	16925726	16925985	260	16925811	10	5.23357	3.76063
Nc-1-IP_pe	chr1	16927098	16927799	702	16927371	11	5.77463	3.94557
Nc-1-IP_pe	chr1	16929477	16930135	659	16929990	12	6.86906	4.44438
Nc-1-IP_pe	chr1	16943485	16944003	519	16943688	9	4.46937	3.41876
Nc-1-IP_pe	chr1	16945188	16946084	897	16945704	14	8.63073	5.12814
Nc-1-IP_pe	chr1	16948256	16949287	1032	16948450	19	10.9318	5.43653
Nc-1-IP_pe	chr1	16950541	16950863	323	16950759	12	5.64372	3.72631
Nc-1-IP_pe	chr1	16958291	16958764	474	16958615	8	3.08755	2.66145
Nc-1-IP_pe	chr1	16960764	16961070	307	16960975	10	5.23357	3.76063
Nc-1-IP_pe	chr1	16965953	16966349	397	16966331	9	4.46937	3.41876
Nc-1-IP_pe	chr1	16967688	16967982	295	16967951	10	3.08907	2.49869
Nc-1-IP_pe	chr1	16970131	16970443	313	16970366	7	3.06442	2.73501
Nc-1-IP_pe	chr1	16971216	16972399	1184	16971322	11	5.77463	3.94557
Nc-1-IP_pe	chr1	16976035	16976610	576	16976575	9	3.72192	2.95716
Nc-1-IP_pe	chr1	16977763	16978289	527	16978262	9	3.92293	3.08
Nc-1-IP_pe	chr1	16979639	16979900	262	16979684	12	5.94619	3.9002
Nc-1-IP_pe	chr1	16986989	16987357	369	16987231	9	3.27326	2.68684
Nc-1-IP_pe	chr1	16996725	16997167	443	16997118	10	4.39571	3.25288
Nc-1-IP_pe	chr1	17004183	17004951	769	17004688	11	5.10577	3.5486
Nc-1-IP_pe	chr1	17011134	17011883	750	17011302	12	6.50787	4.22912
Nc-1-IP_pe	chr1	17087791	17088067	277	17087942	11	6.03439	4.10251
Nc-1-IP_pe	chr1	17094206	17094477	272	17094349	9	3.72192	2.95716
Nc-1-IP_pe	chr1	17117881	17118353	473	17117999	10	4.54734	3.34351
Nc-1-IP_pe	chr1	17138029	17138316	288	17138268	7	2.9043	2.63038
Nc-1-IP_pe	chr1	17149025	17149458	434	17149143	8	3.08755	2.66145
Nc-1-IP_pe	chr1	17164243	17164506	264	17164443	12	4.63088	3.16234
Nc-1-IP_pe	chr1	17183345	17184092	748	17183458	12	6.86906	4.44438
Nc-1-IP_pe	chr1	17198570	17198963	394	17198770	11	5.10577	3.5486
Nc-1-IP_pe	chr1	17212582	17212912	331	17212764	10	4.69727	3.4337
Nc-1-IP_pe	chr1	17230238	17230729	492	17230456	13	6.62436	4.14003
Nc-1-IP_pe	chr1	17232730	17233017	288	17232929	10	5.08818	3.67138
Nc-1-IP_pe	chr1	17233363	17233733	371	17233583	10	4.95265	3.58858
Nc-1-IP_pe	chr1	17238257	17238532	276	17238516	11	4.06403	2.95415
Nc-1-IP_pe	chr1	17259623	17260027	405	17259749	12	6.86906	4.44438
Nc-1-IP_pe	chr1	17267424	17267687	264	17267530	9	4.46937	3.41876
Nc-1-IP_pe	chr1	17271216	17271510	295	17271301	9	4.25966	3.28797
Nc-1-IP_pe	chr1	17274093	17274372	280	17274292	11	5.95085	4.05188
Nc-1-IP_pe	chr1	17297433	17297720	288	17297633	12	6.86906	4.44438
Nc-1-IP_pe	chr1	17303027	17303334	308	17303145	9	4.46937	3.41876

Nc-1-IP_pe	chr1	17310191	17310584	394	17310357	15	9.55381	5.47001
Nc-1-IP_pe	chr1	17313043	17313307	265	17313235	10	4.99888	3.61677
Nc-1-IP_pe	chr1	17315738	17316401	664	17316215	9	4.46937	3.41876
Nc-1-IP_pe	chr1	17321212	17321499	288	17321311	11	6.03439	4.10251
Nc-1-IP_pe	chr1	17330522	17331027	506	17330913	10	4.99888	3.61677
Nc-1-IP_pe	chr1	17344517	17345026	510	17344565	10	4.62142	3.388
Nc-1-IP_pe	chr1	17388518	17388857	340	17388704	11	6.03439	4.10251
Nc-1-IP_pe	chr1	17393596	17394446	851	17393959	12	6.86906	4.44438
Nc-1-IP_pe	chr1	17396111	17396611	501	17396318	10	5.23357	3.76063
Nc-1-IP_pe	chr1	17398937	17399222	286	17399101	11	6.03439	4.10251
Nc-1-IP_pe	chr1	17403509	17403865	357	17403693	12	5.01934	3.37518
Nc-1-IP_pe	chr1	17423078	17423636	559	17423156	9	3.72192	2.95716
Nc-1-IP_pe	chr1	17433318	17433595	278	17433468	11	6.03439	4.10251
Nc-1-IP_pe	chr1	17434721	17435133	413	17434885	12	5.23082	3.49289
Nc-1-IP_pe	chr1	17435479	17435733	255	17435552	10	4.99888	3.61677
Nc-1-IP_pe	chr1	17452238	17452614	377	17452375	9	3.72192	2.95716
Nc-1-IP_pe	chr1	17459964	17460787	824	17460068	10	5.23357	3.76063
Nc-1-IP_pe	chr1	17461935	17462451	517	17462114	11	6.03439	4.10251
Nc-1-IP_pe	chr1	17476241	17476601	361	17476416	10	4.77495	3.48064
Nc-1-IP_pe	chr1	17485461	17486187	727	17485739	9	3.72192	2.95716
Nc-1-IP_pe	chr1	17487318	17487807	490	17487608	9	4.46937	3.41876
Nc-1-IP_pe	chr1	17491283	17491663	381	17491504	11	6.03439	4.10251
Nc-1-IP_pe	chr1	17504665	17505159	495	17504702	8	3.74505	3.07688
Nc-1-IP_pe	chr1	17520689	17520949	261	17520868	12	5.45448	3.61875
Nc-1-IP_pe	chr1	17522403	17522680	278	17522536	10	4.99888	3.61677
Nc-1-IP_pe	chr1	17531942	17532454	513	17532104	9	3.72192	2.95716
Nc-1-IP_pe	chr1	17540019	17541090	1072	17540359	10	4.99888	3.61677
Nc-1-IP_pe	chr1	17551561	17552060	500	17551581	9	3.27326	2.68684
Nc-1-IP_pe	chr1	17552902	17553196	295	17553114	11	5.77463	3.94557
Nc-1-IP_pe	chr1	17566357	17567079	723	17566621	11	3.64839	2.72584
Nc-1-IP_pe	chr1	17569469	17570218	750	17569560	10	4.62142	3.388
Nc-1-IP_pe	chr1	17575570	17575967	398	17575901	10	4.33501	3.21677
Nc-1-IP_pe	chr1	17587594	17587930	337	17587655	8	3.74505	3.07688
Nc-1-IP_pe	chr1	17589763	17590116	354	17590102	8	3.74505	3.07688
Nc-1-IP_pe	chr1	17596220	17596659	440	17596328	11	4.06403	2.95415
Nc-1-IP_pe	chr1	17603491	17603823	333	17603651	12	6.86906	4.44438
Nc-1-IP_pe	chr1	17605355	17605777	423	17605676	8	3.74505	3.07688
Nc-1-IP_pe	chr1	17608524	17609351	828	17608927	11	6.03439	4.10251
Nc-1-IP_pe	chr1	17618054	17618765	712	17618327	12	6.82053	4.41531
Nc-1-IP_pe	chr1	17631445	17631736	292	17631568	9	4.46937	3.41876
Nc-1-IP_pe	chr1	17634979	17635240	262	17635075	9	4.35492	3.34727
Nc-1-IP_pe	chr1	17637494	17638589	1096	17638419	10	5.23357	3.76063
Nc-1-IP_pe	chr1	17640206	17640459	254	17640284	7	3.06442	2.73501
Nc-1-IP_pe	chr1	17644012	17644605	594	17644434	9	3.27326	2.68684
Nc-1-IP_pe	chr1	17647009	17647292	284	17647181	8	3.74505	3.07688

Nc-1-IP_pe	chr1	17653475	17653776	302	17653542	11	5.89269	4.01672
Nc-1-IP_pe	chr1	17657303	17657595	293	17657482	10	4.39571	3.25288
Nc-1-IP_pe	chr1	17677858	17678182	325	17678087	11	5.93136	4.04009
Nc-1-IP_pe	chr1	17688950	17689295	346	17689234	9	4.46937	3.41876
Nc-1-IP_pe	chr1	17694586	17694913	328	17694890	9	3.27326	2.68684
Nc-1-IP_pe	chr1	17699369	17699628	260	17699494	9	4.46937	3.41876
Nc-1-IP_pe	chr1	17737141	17737437	297	17737262	9	4.25966	3.28797
Nc-1-IP_pe	chr1	17750373	17751016	644	17750880	11	6.03439	4.10251
Nc-1-IP_pe	chr1	17774448	17774946	499	17774831	9	4.46937	3.41876
Nc-1-IP_pe	chr1	17778758	17779049	292	17779016	9	4.46937	3.41876
Nc-1-IP_pe	chr1	17802522	17802859	338	17802773	8	3.64414	3.01254
Nc-1-IP_pe	chr1	17819194	17819602	409	17819530	10	3.4604	2.70797
Nc-1-IP_pe	chr1	17833914	17834236	323	17834037	9	4.46937	3.41876
Nc-1-IP_pe	chr1	17842873	17843244	372	17843008	10	5.23357	3.76063
Nc-1-IP_pe	chr1	17852074	17852500	427	17852289	10	5.23357	3.76063
Nc-1-IP_pe	chr1	17854462	17854875	414	17854712	8	3.74505	3.07688
Nc-1-IP_pe	chr1	17887087	17887385	299	17887157	10	5.23357	3.76063
Nc-1-IP_pe	chr1	18015729	18016034	306	18015868	10	4.99888	3.61677
Nc-1-IP_pe	chr1	18033505	18033929	425	18033890	9	4.46937	3.41876
Nc-1-IP_pe	chr1	18057630	18058059	430	18057967	11	6.03439	4.10251
Nc-1-IP_pe	chr1	18107157	18107457	301	18107268	13	7.7352	4.78626
Nc-1-IP_pe	chr1	18181152	18181410	259	18181305	11	6.03439	4.10251
Nc-1-IP_pe	chr1	18222130	18222408	279	18222225	12	6.58417	4.27436
Nc-1-IP_pe	chr1	18295622	18295982	361	18295821	11	6.03439	4.10251
Nc-1-IP_pe	chr1	18346745	18347378	634	18347215	11	6.03439	4.10251
Nc-1-IP_pe	chr1	18363411	18363704	294	18363527	10	5.23357	3.76063
Nc-1-IP_pe	chr1	18434024	18434308	285	18434107	9	4.46937	3.41876
Nc-1-IP_pe	chr1	18453531	18453815	285	18453706	10	5.23357	3.76063
Nc-1-IP_pe	chr1	18575069	18575736	668	18575173	9	4.46937	3.41876
Nc-1-IP_pe	chr1	18606747	18607301	555	18606883	12	4.70082	3.20033
Nc-1-IP_pe	chr1	18616311	18616673	363	18616541	10	5.23357	3.76063
Nc-1-IP_pe	chr1	18617462	18617722	261	18617641	10	5.23357	3.76063
Nc-1-IP_pe	chr1	18619080	18619373	294	18619224	10	5.23357	3.76063
Nc-1-IP_pe	chr1	18622869	18623192	324	18622945	10	5.23357	3.76063
Nc-1-IP_pe	chr1	18633725	18634620	896	18633884	13	6.62436	4.14003
Nc-1-IP_pe	chr1	18650111	18650397	287	18650198	9	4.25966	3.28797
Nc-1-IP_pe	chr1	18664045	18664586	542	18664262	10	4.39571	3.25288
Nc-1-IP_pe	chr1	18668184	18668938	755	18668330	9	4.23325	3.27156
Nc-1-IP_pe	chr1	18680301	18680594	294	18680396	10	5.23357	3.76063
Nc-1-IP_pe	chr1	18689308	18689791	484	18689458	8	3.74505	3.07688
Nc-1-IP_pe	chr1	18712399	18713082	684	18712539	9	4.46937	3.41876
Nc-1-IP_pe	chr1	18718111	18718364	254	18718238	10	5.23357	3.76063
Nc-1-IP_pe	chr1	18718762	18719017	256	18718879	11	5.77463	3.94557
Nc-1-IP_pe	chr1	18720918	18721667	750	18721416	13	7.7352	4.78626
Nc-1-IP_pe	chr1	18722406	18722771	366	18722533	8	3.74505	3.07688

Nc-1-IP_pe	chr1	18728881	18729136	256	18728944	7	3.06442	2.73501
Nc-1-IP_pe	chr1	18737128	18737462	335	18737252	10	5.23357	3.76063
Nc-1-IP_pe	chr1	18746007	18746288	282	18746095	8	3.74505	3.07688
Nc-1-IP_pe	chr1	18798979	18799679	701	18799466	12	5.23082	3.49289
Nc-1-IP_pe	chr1	18800249	18800514	266	18800441	9	4.46568	3.41644
Nc-1-IP_pe	chr1	18821408	18821689	282	18821449	10	3.71867	2.85592
Nc-1-IP_pe	chr1	18830840	18831138	299	18831041	8	3.74505	3.07688
Nc-1-IP_pe	chr1	18838145	18838585	441	18838201	8	3.74505	3.07688
Nc-1-IP_pe	chr1	18859278	18859534	257	18859363	9	4.46937	3.41876
Nc-1-IP_pe	chr1	18869458	18869777	320	18869628	11	6.03439	4.10251
Nc-1-IP_pe	chr1	18902206	18902508	303	18902359	14	4.21449	2.76599
Nc-1-IP_pe	chr1	18915567	18916263	697	18915848	8	3.74505	3.07688
Nc-1-IP_pe	chr1	18926082	18926336	255	18926101	10	4.0855	3.0694
Nc-1-IP_pe	chr1	18939921	18940332	412	18939949	8	3.56021	2.95918
Nc-1-IP_pe	chr1	18945810	18946286	477	18946251	8	3.74505	3.07688
Nc-1-IP_pe	chr1	18949514	18949786	273	18949737	8	3.74505	3.07688
Nc-1-IP_pe	chr1	18951759	18952166	408	18951972	11	6.03439	4.10251
Nc-1-IP_pe	chr1	18955692	18956036	345	18955711	12	3.83688	2.74114
Nc-1-IP_pe	chr1	18964935	18965798	864	18965442	12	6.50787	4.22912
Nc-1-IP_pe	chr1	18978763	18979115	353	18978911	12	6.86906	4.44438
Nc-1-IP_pe	chr1	18986615	18987076	462	18987025	8	3.74505	3.07688
Nc-1-IP_pe	chr1	19032905	19033230	326	19033013	11	5.7051	3.90381
Nc-1-IP_pe	chr1	19047686	19048075	390	19047869	9	4.46937	3.41876
Nc-1-IP_pe	chr1	19053746	19053999	254	19053995	10	3.89064	2.95552
Nc-1-IP_pe	chr1	19055114	19055422	309	19055322	9	4.46937	3.41876
Nc-1-IP_pe	chr1	19058408	19058760	353	19058705	9	4.15971	3.22596
Nc-1-IP_pe	chr1	19070457	19070887	431	19070585	9	4.46568	3.41644
Nc-1-IP_pe	chr1	19140607	19141054	448	19140661	8	3.74505	3.07688
Nc-1-IP_pe	chr1	19209886	19210482	597	19210042	14	8.63073	5.12814
Nc-1-IP_pe	chr1	19214867	19215282	416	19215133	15	9.55381	5.47001
Nc-1-IP_pe	chr1	19251338	19252726	1389	19251574	16	9.11744	5.02718
Nc-1-IP_pe	chr1	19256396	19256792	397	19256683	11	4.54404	3.2242
Nc-1-IP_pe	chr1	19269231	19269567	337	19269236	8	3.0402	2.6319
Nc-1-IP_pe	chr1	19274199	19274456	258	19274250	9	3.92293	3.08
Nc-1-IP_pe	chr1	19288421	19288741	321	19288519	11	6.03439	4.10251
Nc-1-IP_pe	chr1	19315288	19315558	271	19315430	12	6.05242	3.96182
Nc-1-IP_pe	chr1	19331959	19332573	615	19332464	9	4.46937	3.41876
Nc-1-IP_pe	chr1	19337787	19338056	270	19337943	12	5.94619	3.9002
Nc-1-IP_pe	chr1	19338515	19338820	306	19338682	12	6.69264	4.3389
Nc-1-IP_pe	chr1	19342081	19342521	441	19342231	9	4.05981	3.16422
Nc-1-IP_pe	chr1	19351900	19352162	263	19352066	13	6.36468	3.99316
Nc-1-IP_pe	chr1	19359924	19360215	292	19360095	9	3.72192	2.95716
Nc-1-IP_pe	chr1	19371470	19371754	285	19371582	10	4.39571	3.25288
Nc-1-IP_pe	chr1	19372440	19372832	393	19372762	10	5.23357	3.76063
Nc-1-IP_pe	chr1	19383161	19383601	441	19383260	10	4.91962	3.56846

Nc-1-IP_pe	chr1	19386295	19386801	507	19386696	11	4.06403	2.95415
Nc-1-IP_pe	chr1	19402477	19402779	303	19402603	13	7.7352	4.78626
Nc-1-IP_pe	chr1	19408269	19408539	271	19408375	10	5.10552	3.682
Nc-1-IP_pe	chr1	19429627	19429880	254	19429844	7	3.06442	2.73501
Nc-1-IP_pe	chr1	19447603	19447862	260	19447782	11	6.03439	4.10251
Nc-1-IP_pe	chr1	19484353	19485536	1184	19484850	14	8.63073	5.12814
Nc-1-IP_pe	chr1	19635616	19635893	278	19635824	10	5.23357	3.76063
Nc-1-IP_pe	chr1	19653863	19654216	354	19654122	7	2.97697	2.67781
Nc-1-IP_pe	chr1	19654935	19655663	729	19655073	15	9.55381	5.47001
Nc-1-IP_pe	chr1	19657191	19658076	886	19657676	13	5.36853	3.44651
Nc-1-IP_pe	chr1	19666357	19666886	530	19666359	8	3.08755	2.66145
Nc-1-IP_pe	chr1	19667529	19668116	588	19667768	12	5.23082	3.49289
Nc-1-IP_pe	chr1	19669406	19669703	298	19669617	11	4.74697	3.34038
Nc-1-IP_pe	chr1	19722793	19723079	287	19723036	8	3.74505	3.07688
Nc-1-IP_pe	chr1	19780274	19780617	344	19780410	8	3.74505	3.07688
Nc-1-IP_pe	chr1	19802271	19802644	374	19802476	8	3.08755	2.66145
Nc-1-IP_pe	chr1	19813047	19813705	659	19813629	8	3.74505	3.07688
Nc-1-IP_pe	chr1	19920862	19921375	514	19921266	12	6.19862	4.04709
Nc-1-IP_pe	chr1	19948233	19948503	271	19948420	8	3.74505	3.07688
Nc-1-IP_pe	chr1	19953369	19954011	643	19953858	11	6.03439	4.10251
Nc-1-IP_pe	chr1	19976877	19977198	322	19976971	8	3.56021	2.95918
Nc-1-IP_pe	chr1	19977945	19978204	260	19978137	9	4.46937	3.41876
Nc-1-IP_pe	chr1	20100039	20101037	999	20100853	11	6.03439	4.10251
Nc-1-IP_pe	chr1	20101687	20101954	268	20101845	8	3.74505	3.07688
Nc-1-IP_pe	chr1	20112057	20112364	308	20112197	10	5.21145	3.74702
Nc-1-IP_pe	chr1	20116330	20116788	459	20116597	12	6.86906	4.44438
Nc-1-IP_pe	chr1	20126678	20127253	576	20127157	10	4.99888	3.61677
Nc-1-IP_pe	chr1	20146115	20146410	296	20146304	8	3.68553	3.03891
Nc-1-IP_pe	chr1	20149201	20149763	563	20149277	10	5.23357	3.76063
Nc-1-IP_pe	chr1	20151129	20151488	360	20151251	11	6.03439	4.10251
Nc-1-IP_pe	chr1	20152099	20152392	294	20152320	10	3.4604	2.70797
Nc-1-IP_pe	chr1	20156991	20157293	303	20157083	9	3.72192	2.95716
Nc-1-IP_pe	chr1	20183261	20183802	542	20183290	11	5.52654	3.79706
Nc-1-IP_pe	chr1	20191343	20191611	269	20191521	10	5.23357	3.76063
Nc-1-IP_pe	chr1	20203352	20203659	308	20203521	9	4.46937	3.41876
Nc-1-IP_pe	chr1	20204595	20204982	388	20204854	9	4.46937	3.41876
Nc-1-IP_pe	chr1	20205287	20205587	301	20205509	12	5.84943	3.84431
Nc-1-IP_pe	chr1	20220867	20221150	284	20221034	9	3.72192	2.95716
Nc-1-IP_pe	chr1	20223906	20224168	263	20224108	7	3.06442	2.73501
Nc-1-IP_pe	chr1	20230349	20230749	401	20230424	8	3.74505	3.07688
Nc-1-IP_pe	chr1	20262655	20262946	292	20262810	11	6.03439	4.10251
Nc-1-IP_pe	chr1	20283468	20283876	409	20283678	9	3.27326	2.68684
Nc-1-IP_pe	chr1	20302648	20302962	315	20302835	9	4.46937	3.41876
Nc-1-IP_pe	chr1	20311764	20312081	318	20311963	10	5.23357	3.76063
Nc-1-IP_pe	chr1	20342388	20342758	371	20342407	12	4.24064	2.95299

Nc-1-IP_pe	chr1	20345463	20345755	293	20345651	10	5.23357	3.76063
Nc-1-IP_pe	chr1	20366228	20366642	415	20366568	8	3.08755	2.66145
Nc-1-IP_pe	chr1	20368934	20369375	442	20369090	13	7.7352	4.78626
Nc-1-IP_pe	chr1	20375237	20375670	434	20375491	12	5.23082	3.49289
Nc-1-IP_pe	chr1	20379340	20379911	572	20379472	10	4.99888	3.61677
Nc-1-IP_pe	chr1	20387467	20387830	364	20387808	9	4.25966	3.28797
Nc-1-IP_pe	chr1	20401591	20402102	512	20401865	12	6.86906	4.44438
Nc-1-IP_pe	chr1	20404596	20404898	303	20404707	8	3.74505	3.07688
Nc-1-IP_pe	chr1	20430597	20431028	432	20430851	9	4.23325	3.27156
Nc-1-IP_pe	chr1	20463146	20463401	256	20463172	9	4.46937	3.41876
Nc-1-IP_pe	chr1	20472714	20473577	864	20473433	10	5.23357	3.76063
Nc-1-IP_pe	chr1	20481763	20482036	274	20481846	11	6.03439	4.10251
Nc-1-IP_pe	chr1	20516772	20517071	300	20516836	8	3.74505	3.07688
Nc-1-IP_pe	chr1	20553196	20554164	969	20554063	9	4.46937	3.41876
Nc-1-IP_pe	chr1	20566550	20566812	263	20566620	9	3.27326	2.68684
Nc-1-IP_pe	chr1	20589088	20589905	818	20589119	12	5.45448	3.61875
Nc-1-IP_pe	chr1	20633484	20633993	510	20633700	11	6.03439	4.10251
Nc-1-IP_pe	chr1	20636838	20637238	401	20637186	10	3.89064	2.95552
Nc-1-IP_pe	chr1	20640594	20640872	279	20640744	10	5.23357	3.76063
Nc-1-IP_pe	chr1	20652963	20653249	287	20653168	10	5.23357	3.76063
Nc-1-IP_pe	chr1	20657090	20657345	256	20657178	8	3.74505	3.07688
Nc-1-IP_pe	chr1	20682606	20682999	394	20682784	11	6.03439	4.10251
Nc-1-IP_pe	chr1	20701577	20701903	327	20701783	13	6.92473	4.312
Nc-1-IP_pe	chr1	20704718	20705039	322	20704991	8	3.74505	3.07688
Nc-1-IP_pe	chr1	20716952	20717611	660	20717335	14	7.34079	4.38651
Nc-1-IP_pe	chr1	20719151	20719582	432	20719462	9	4.46568	3.41644
Nc-1-IP_pe	chr1	20721478	20722245	768	20721705	10	5.23357	3.76063
Nc-1-IP_pe	chr1	20723484	20723745	262	20723578	10	5.23357	3.76063
Nc-1-IP_pe	chr1	20725083	20726642	1560	20725366	14	8.63073	5.12814
Nc-1-IP_pe	chr1	20727321	20727884	564	20727622	9	4.1889	3.24405
Nc-1-IP_pe	chr1	20731150	20731727	578	20731667	14	5.03085	3.16286
Nc-1-IP_pe	chr1	20786168	20786583	416	20786430	10	4.39571	3.25288
Nc-1-IP_pe	chr1	20820309	20820859	551	20820806	9	3.79246	3.00015
Nc-1-IP_pe	chr1	20838747	20839479	733	20838959	9	4.46937	3.41876
Nc-1-IP_pe	chr1	20875763	20876079	317	20875913	11	6.03439	4.10251
Nc-1-IP_pe	chr1	21035263	21035829	567	21035325	11	4.00079	2.91908
Nc-1-IP_pe	chr1	21052802	21053929	1128	21053409	19	7.29128	3.68799
Nc-1-IP_pe	chr1	21224966	21225244	279	21225166	9	3.72192	2.95716
Nc-1-IP_pe	chr1	21232916	21233672	757	21233503	12	6.50787	4.22912
Nc-1-IP_pe	chr1	21237940	21238354	415	21238201	11	5.03835	3.5092
Nc-1-IP_pe	chr1	21250654	21250924	271	21250724	8	3.7276	3.06575
Nc-1-IP_pe	chr1	21263475	21264754	1280	21263987	14	8.27703	4.92126
Nc-1-IP_pe	chr1	21274299	21274587	289	21274477	10	5.23357	3.76063
Nc-1-IP_pe	chr1	21288859	21290628	1770	21288994	12	4.24064	2.95299
Nc-1-IP_pe	chr1	21293498	21293893	396	21293701	14	8.63073	5.12814

Nc-1-IP_pe	chr1	21294185	21294507	323	21294379	8	3.74505	3.07688
Nc-1-IP_pe	chr1	21301420	21301684	265	21301620	10	4.69727	3.4337
Nc-1-IP_pe	chr1	21302102	21302365	264	21302228	11	5.10577	3.5486
Nc-1-IP_pe	chr1	21321693	21322059	367	21322051	10	4.47495	3.30017
Nc-1-IP_pe	chr1	21336673	21336927	255	21336759	11	4.69157	3.30855
Nc-1-IP_pe	chr1	21342758	21343231	474	21343032	14	5.51541	3.4073
Nc-1-IP_pe	chr1	21370719	21371140	422	21371062	9	4.46937	3.41876
Nc-1-IP_pe	chr1	21388096	21388427	332	21388376	29	10.8146	4.01908
Nc-1-IP_pe	chr1	21405197	21405604	408	21405423	12	6.50787	4.22912
Nc-1-IP_pe	chr1	21428541	21428948	408	21428732	14	7.42849	4.43574
Nc-1-IP_pe	chr1	21431013	21431800	788	21431238	10	5.23357	3.76063
Nc-1-IP_pe	chr1	21436370	21436816	447	21436566	15	8.61047	4.928
Nc-1-IP_pe	chr1	21491428	21491779	352	21491444	8	3.64414	3.01254
Nc-1-IP_pe	chr1	21496903	21497320	418	21497020	10	5.23357	3.76063
Nc-1-IP_pe	chr1	21508508	21508792	285	21508671	13	7.7352	4.78626
Nc-1-IP_pe	chr1	21509680	21510345	666	21509938	9	4.46937	3.41876
Nc-1-IP_pe	chr1	21511567	21512396	830	21511684	10	4.39571	3.25288
Nc-1-IP_pe	chr1	21514316	21514987	672	21514794	10	4.99888	3.61677
Nc-1-IP_pe	chr1	21515758	21516038	281	21515957	9	3.79246	3.00015
Nc-1-IP_pe	chr1	21525392	21525682	291	21525618	9	4.46937	3.41876
Nc-1-IP_pe	chr1	21531017	21531369	353	21531234	11	6.03439	4.10251
Nc-1-IP_pe	chr1	21537404	21537753	350	21537468	10	5.23357	3.76063
Nc-1-IP_pe	chr1	21538193	21538835	643	21538414	13	7.7352	4.78626
Nc-1-IP_pe	chr1	21562896	21563296	401	21563132	12	6.86906	4.44438
Nc-1-IP_pe	chr1	21567890	21568352	463	21568053	11	5.77463	3.94557
Nc-1-IP_pe	chr1	21573851	21574231	381	21573997	11	6.03439	4.10251
Nc-1-IP_pe	chr1	21584254	21584597	344	21584581	8	3.74505	3.07688
Nc-1-IP_pe	chr1	21585271	21585815	545	21585728	9	4.46937	3.41876
Nc-1-IP_pe	chr1	21597629	21597886	258	21597662	11	4.54404	3.2242
Nc-1-IP_pe	chr1	21598433	21599720	1288	21599294	10	4.39571	3.25288
Nc-1-IP_pe	chr1	21608144	21608576	433	21608352	12	5.77527	3.80164
Nc-1-IP_pe	chr1	21611170	21612111	942	21611468	14	6.69566	4.03025
Nc-1-IP_pe	chr1	21613657	21614328	672	21614160	15	6.19428	3.63445
Nc-1-IP_pe	chr1	21619053	21619337	285	21619269	12	6.35031	4.1361
Nc-1-IP_pe	chr1	21626422	21626709	288	21626500	10	4.39571	3.25288
Nc-1-IP_pe	chr1	21643972	21644320	349	21644274	9	4.25966	3.28797
Nc-1-IP_pe	chr1	21648794	21649201	408	21648931	15	9.54746	5.46631
Nc-1-IP_pe	chr1	21650260	21650833	574	21650716	10	4.62142	3.388
Nc-1-IP_pe	chr1	21652033	21652404	372	21652190	11	6.03439	4.10251
Nc-1-IP_pe	chr1	21663404	21663700	297	21663599	9	4.46937	3.41876
Nc-1-IP_pe	chr1	21668516	21669018	503	21668755	7	3.06442	2.73501
Nc-1-IP_pe	chr1	21674058	21674389	332	21674246	11	5.89269	4.01672
Nc-1-IP_pe	chr1	21787281	21787579	299	21787452	10	4.99888	3.61677
Nc-1-IP_pe	chr1	21814472	21815068	597	21814960	10	4.39571	3.25288
Nc-1-IP_pe	chr1	21823364	21824046	683	21824035	11	4.00079	2.91908

Nc-1-IP_pe	chr1	21828920	21829183	264	21828996	11	5.10577	3.5486
Nc-1-IP_pe	chr1	21833765	21834325	561	21834230	9	4.20361	3.25317
Nc-1-IP_pe	chr1	21836576	21837007	432	21836774	11	5.89269	4.01672
Nc-1-IP_pe	chr1	21842939	21843455	517	21843411	11	4.06403	2.95415
Nc-1-IP_pe	chr1	21844110	21844411	302	21844182	8	3.63049	3.00385
Nc-1-IP_pe	chr1	21852180	21852466	287	21852322	9	3.76709	2.98468
Nc-1-IP_pe	chr1	21854094	21854438	345	21854126	9	3.58404	2.87351
Nc-1-IP_pe	chr1	21855355	21855657	303	21855539	13	3.35769	2.42919
Nc-1-IP_pe	chr1	21864916	21865627	712	21865380	10	4.99888	3.61677
Nc-1-IP_pe	chr1	21870790	21871044	255	21870899	10	4.62142	3.388
Nc-1-IP_pe	chr1	21880048	21880567	520	21880274	14	7.9745	4.74633
Nc-1-IP_pe	chr1	21881212	21881541	330	21881225	10	3.89064	2.95552
Nc-1-IP_pe	chr1	21884532	21885302	771	21884703	10	5.23357	3.76063
Nc-1-IP_pe	chr1	21889936	21890458	523	21889989	11	6.00992	4.08766
Nc-1-IP_pe	chr1	21893934	21894592	659	21894356	12	6.46791	4.20548
Nc-1-IP_pe	chr1	21895755	21896069	315	21895782	12	3.47963	2.55766
Nc-1-IP_pe	chr1	21913391	21913743	353	21913722	10	4.20113	3.13749
Nc-1-IP_pe	chr1	21924932	21925326	395	21925030	8	3.74505	3.07688
Nc-1-IP_pe	chr1	21926947	21927340	394	21927300	11	5.63263	3.8604
Nc-1-IP_pe	chr1	21927699	21927976	278	21927789	11	5.10577	3.5486
Nc-1-IP_pe	chr1	21933363	21933694	332	21933528	19	11.7093	5.84867
Nc-1-IP_pe	chr1	21937449	21937722	274	21937620	9	3.72192	2.95716
Nc-1-IP_pe	chr1	21955799	21956059	261	21955947	10	5.23357	3.76063
Nc-1-IP_pe	chr1	21957237	21957894	658	21957277	9	2.93173	2.4848
Nc-1-IP_pe	chr1	21961782	21962145	364	21962097	12	5.23082	3.49289
Nc-1-IP_pe	chr1	21974119	21974499	381	21974254	11	6.03439	4.10251
Nc-1-IP_pe	chr1	21981445	21981899	455	21981603	12	6.86906	4.44438
Nc-1-IP_pe	chr1	21993261	21994113	853	21994041	9	3.79246	3.00015
Nc-1-IP_pe	chr1	22001432	22001981	550	22001533	9	3.72192	2.95716
Nc-1-IP_pe	chr1	22052090	22053406	1317	22052264	11	6.03439	4.10251
Nc-1-IP_pe	chr1	22108929	22109209	281	22109058	11	6.03439	4.10251
Nc-1-IP_pe	chr1	22132638	22133157	520	22132804	11	4.54404	3.2242
Nc-1-IP_pe	chr1	22142279	22142706	428	22142619	10	5.23357	3.76063
Nc-1-IP_pe	chr1	22182553	22183156	604	22182599	12	5.77527	3.80164
Nc-1-IP_pe	chr1	22186197	22186525	329	22186259	8	3.56021	2.95918
Nc-1-IP_pe	chr1	22192130	22192447	318	22192368	7	2.9043	2.63038
Nc-1-IP_pe	chr1	22209147	22209457	311	22209217	8	3.74505	3.07688
Nc-1-IP_pe	chr1	22218086	22218518	433	22218106	8	3.64414	3.01254
Nc-1-IP_pe	chr1	22226016	22226339	324	22226243	10	5.23357	3.76063
Nc-1-IP_pe	chr1	22233764	22234537	774	22233892	11	6.03439	4.10251
Nc-1-IP_pe	chr1	22237088	22237344	257	22237312	7	3.06442	2.73501
Nc-1-IP_pe	chr1	22239008	22239390	383	22239163	9	2.89249	2.46179
Nc-1-IP_pe	chr1	22250747	22251376	630	22251107	11	5.7051	3.90381
Nc-1-IP_pe	chr1	22261213	22261502	290	22261402	12	5.77527	3.80164
Nc-1-IP_pe	chr1	22272416	22272790	375	22272575	9	4.46937	3.41876

Nc-1-IP_pe	chr1	22343528	22343787	260	22343579	9	3.27326	2.68684
Nc-1-IP_pe	chr1	22363115	22363539	425	22363236	9	4.46937	3.41876
Nc-1-IP_pe	chr1	22370906	22371396	491	22371337	9	4.46937	3.41876
Nc-1-IP_pe	chr1	22383845	22384382	538	22383914	10	4.99888	3.61677
Nc-1-IP_pe	chr1	22402696	22403034	339	22402834	13	7.7352	4.78626
Nc-1-IP_pe	chr1	22451712	22452310	599	22451906	13	7.7352	4.78626
Nc-1-IP_pe	chr1	22529735	22530003	269	22529878	9	4.46937	3.41876
Nc-1-IP_pe	chr1	22531405	22531684	280	22531498	9	4.46937	3.41876
Nc-1-IP_pe	chr1	22543499	22543983	485	22543729	12	6.84223	4.4283
Nc-1-IP_pe	chr1	22553652	22553992	341	22553758	10	5.23357	3.76063
Nc-1-IP_pe	chr1	22564269	22564650	382	22564307	9	4.46937	3.41876
Nc-1-IP_pe	chr1	22565178	22565451	274	22565351	14	8.29543	4.93196
Nc-1-IP_pe	chr1	22613581	22613964	384	22613791	10	4.39571	3.25288
Nc-1-IP_pe	chr1	22636484	22637100	617	22636651	12	6.86906	4.44438
Nc-1-IP_pe	chr1	22638754	22639039	286	22638833	10	5.23357	3.76063
Nc-1-IP_pe	chr1	22641680	22642012	333	22641808	10	5.23357	3.76063
Nc-1-IP_pe	chr1	22642557	22642910	354	22642621	9	4.46937	3.41876
Nc-1-IP_pe	chr1	22643840	22644107	268	22643992	8	3.74505	3.07688
Nc-1-IP_pe	chr1	22647367	22647682	316	22647427	9	4.46937	3.41876
Nc-1-IP_pe	chr1	22653110	22653666	557	22653530	11	6.03439	4.10251
Nc-1-IP_pe	chr1	22669629	22669997	369	22669801	8	3.74505	3.07688
Nc-1-IP_pe	chr1	22673479	22673862	384	22673769	9	4.46937	3.41876
Nc-1-IP_pe	chr1	22676953	22677444	492	22677128	9	4.46937	3.41876
Nc-1-IP_pe	chr1	22679525	22679794	270	22679669	8	3.64414	3.01254
Nc-1-IP_pe	chr1	22680370	22680976	607	22680687	8	3.74505	3.07688
Nc-1-IP_pe	chr1	22681384	22681820	437	22681540	13	6.62436	4.14003
Nc-1-IP_pe	chr1	22717266	22717581	316	22717478	10	5.23357	3.76063
Nc-1-IP_pe	chr1	22731961	22732254	294	22732086	10	4.99888	3.61677
Nc-1-IP_pe	chr1	22746007	22746406	400	22746097	10	3.89064	2.95552
Nc-1-IP_pe	chr1	22777012	22777284	273	22777080	9	4.46937	3.41876
Nc-1-IP_pe	chr1	22792818	22793131	314	22792980	15	8.16556	4.67894
Nc-1-IP_pe	chr1	22805022	22805391	370	22805169	10	5.23357	3.76063
Nc-1-IP_pe	chr1	22807504	22807935	432	22807704	13	7.12853	4.42991
Nc-1-IP_pe	chr1	22825379	22825711	333	22825597	12	6.12485	4.004
Nc-1-IP_pe	chr1	22851590	22851846	257	22851792	7	3.06442	2.73501
Nc-1-IP_pe	chr1	22862976	22863339	364	22863022	11	4.54404	3.2242
Nc-1-IP_pe	chr1	22883599	22883854	256	22883657	8	3.64414	3.01254
Nc-1-IP_pe	chr1	22931025	22931316	292	22931124	9	4.46937	3.41876
Nc-1-IP_pe	chr1	22963386	22963885	500	22963489	10	4.39571	3.25288
Nc-1-IP_pe	chr1	22982205	22982500	296	22982286	8	3.56021	2.95918
Nc-1-IP_pe	chr1	23008496	23008881	386	23008822	11	5.77463	3.94557
Nc-1-IP_pe	chr1	23088625	23088911	287	23088787	10	5.23357	3.76063
Nc-1-IP_pe	chr1	23187726	23187985	260	23187762	8	3.74505	3.07688
Nc-1-IP_pe	chr1	23202995	23203494	500	23203137	9	4.46937	3.41876
Nc-1-IP_pe	chr1	23216836	23217792	957	23217662	13	4.41954	2.952

Nc-1-IP_pe	chr1	23245537	23245854	318	23245693	9	4.46937	3.41876
Nc-1-IP_pe	chr1	23375465	23375722	258	23375551	10	5.23357	3.76063
Nc-1-IP_pe	chr1	23379326	23379738	413	23379556	9	4.46937	3.41876
Nc-1-IP_pe	chr1	23384109	23384409	301	23384266	11	5.77463	3.94557
Nc-1-IP_pe	chr1	23418139	23418511	373	23418145	8	3.08755	2.66145
Nc-1-IP_pe	chr1	23424525	23425127	603	23424999	9	4.46937	3.41876
Nc-1-IP_pe	chr1	23436710	23437299	590	23436851	10	5.23357	3.76063
Nc-1-IP_pe	chr1	23442145	23442420	276	23442357	10	5.23357	3.76063
Nc-1-IP_pe	chr1	23450921	23451298	378	23451217	12	5.84943	3.84431
Nc-1-IP_pe	chr1	23454954	23455246	293	23455182	10	5.23357	3.76063
Nc-1-IP_pe	chr1	23462674	23463033	360	23462813	9	3.72192	2.95716
Nc-1-IP_pe	chr1	23470143	23470750	608	23470443	10	5.23357	3.76063
Nc-1-IP_pe	chr1	23483751	23484154	404	23484008	10	4.62142	3.388
Nc-1-IP_pe	chr1	23506400	23506710	311	23506559	9	4.25966	3.28797
Nc-1-IP_pe	chr1	23516835	23517154	320	23516906	9	4.25966	3.28797
Nc-1-IP_pe	chr1	23521793	23522126	334	23521903	11	6.03439	4.10251
Nc-1-IP_pe	chr1	23524748	23525294	547	23524927	9	3.72192	2.95716
Nc-1-IP_pe	chr1	23528004	23528322	319	23528146	11	6.03439	4.10251
Nc-1-IP_pe	chr1	23543162	23543516	355	23543360	10	5.23357	3.76063
Nc-1-IP_pe	chr1	23545246	23545580	335	23545496	10	4.93609	3.57849
Nc-1-IP_pe	chr1	23554318	23554890	573	23554354	11	5.03835	3.5092
Nc-1-IP_pe	chr1	23556149	23556462	314	23556277	12	6.528	4.24104
Nc-1-IP_pe	chr1	23558892	23559250	359	23559199	11	4.47871	3.18705
Nc-1-IP_pe	chr1	23586796	23587086	291	23586847	8	3.14944	2.70014
Nc-1-IP_pe	chr1	23598731	23599014	284	23598995	12	4.24064	2.95299
Nc-1-IP_pe	chr1	23606627	23607093	467	23606962	9	4.46937	3.41876
Nc-1-IP_pe	chr1	23618437	23618713	277	23618596	9	4.46937	3.41876
Nc-1-IP_pe	chr1	23619411	23619909	499	23619808	9	4.46937	3.41876
Nc-1-IP_pe	chr1	23634657	23635115	459	23634886	14	7.42849	4.43574
Nc-1-IP_pe	chr1	23644677	23645018	342	23644858	11	6.03439	4.10251
Nc-1-IP_pe	chr1	23652159	23652571	413	23652315	10	5.23357	3.76063
Nc-1-IP_pe	chr1	23691817	23692229	413	23691962	9	3.72192	2.95716
Nc-1-IP_pe	chr1	23740228	23740534	307	23740469	8	3.74505	3.07688
Nc-1-IP_pe	chr1	23743184	23744085	902	23743639	15	6.78905	3.93887
Nc-1-IP_pe	chr1	23793168	23793596	429	23793512	11	4.61014	3.26192
Nc-1-IP_pe	chr1	23798797	23799347	551	23798974	14	8.20556	4.87976
Nc-1-IP_pe	chr1	23800181	23801401	1221	23800522	12	5.61153	3.70794
Nc-1-IP_pe	chr1	23825095	23825496	402	23825315	14	6.69566	4.03025
Nc-1-IP_pe	chr1	23857527	23857792	266	23857728	7	3.06442	2.73501
Nc-1-IP_pe	chr1	23868592	23868869	278	23868775	11	6.03439	4.10251
Nc-1-IP_pe	chr1	23875140	23875485	346	23875387	11	5.10577	3.5486
Nc-1-IP_pe	chr1	23901725	23903142	1418	23902623	10	5.23357	3.76063
Nc-1-IP_pe	chr1	23909055	23909669	615	23909278	10	4.99888	3.61677
Nc-1-IP_pe	chr1	23912755	23913039	285	23912843	11	4.61014	3.26192
Nc-1-IP_pe	chr1	23916745	23917157	413	23916818	9	4.05981	3.16422

Nc-1-IP_pe	chr1	23927929	23928186	258	23928069	9	4.46937	3.41876
Nc-1-IP_pe	chr1	23987004	23987267	264	23987169	13	6.92473	4.312
Nc-1-IP_pe	chr1	23993175	23994468	1294	23993494	26	9.34034	3.78992
Nc-1-IP_pe	chr1	24026440	24026832	393	24026621	8	3.08755	2.66145
Nc-1-IP_pe	chr1	24029362	24029719	358	24029631	9	4.46937	3.41876
Nc-1-IP_pe	chr1	24050418	24050964	547	24050783	12	6.86906	4.44438
Nc-1-IP_pe	chr1	24064309	24064606	298	24064454	10	5.23357	3.76063
Nc-1-IP_pe	chr1	24079837	24080349	513	24079848	9	2.89249	2.46179
Nc-1-IP_pe	chr1	24086134	24086477	344	24086341	11	6.03439	4.10251
Nc-1-IP_pe	chr1	24089402	24089996	595	24089543	11	5.72344	3.91481
Nc-1-IP_pe	chr1	24094935	24095414	480	24095001	9	3.72192	2.95716
Nc-1-IP_pe	chr1	24101199	24101527	329	24101459	8	3.56332	2.96115
Nc-1-IP_pe	chr1	24105149	24105517	369	24105494	8	3.74505	3.07688
Nc-1-IP_pe	chr1	24107559	24107983	425	24107823	8	3.74505	3.07688
Nc-1-IP_pe	chr1	24108458	24108916	459	24108703	17	9.65211	5.13407
Nc-1-IP_pe	chr1	24121480	24121913	434	24121867	10	4.62142	3.388
Nc-1-IP_pe	chr1	24123140	24123427	288	24123387	8	3.74505	3.07688
Nc-1-IP_pe	chr1	24142045	24142300	256	24142086	10	5.23357	3.76063
Nc-1-IP_pe	chr1	24156939	24157301	363	24157111	18	12.0366	6.24715
Nc-1-IP_pe	chr1	24181079	24181376	298	24181301	9	3.79246	3.00015
Nc-1-IP_pe	chr1	24190515	24190877	363	24190632	10	5.23357	3.76063
Nc-1-IP_pe	chr1	24195895	24196173	279	24196005	12	6.86906	4.44438
Nc-1-IP_pe	chr1	24202435	24202767	333	24202637	9	4.46937	3.41876
Nc-1-IP_pe	chr1	24210816	24211409	594	24211174	10	5.23357	3.76063
Nc-1-IP_pe	chr1	24212121	24212538	418	24212455	20	6.49719	3.259
Nc-1-IP_pe	chr1	24216165	24216981	817	24216579	12	6.86906	4.44438
Nc-1-IP_pe	chr1	24245513	24245927	415	24245560	9	4.35492	3.34727
Nc-1-IP_pe	chr1	24254430	24255169	740	24254627	8	3.74505	3.07688
Nc-1-IP_pe	chr1	24274109	24274741	633	24274532	10	5.23357	3.76063
Nc-1-IP_pe	chr1	24278669	24279195	527	24278913	9	4.46937	3.41876
Nc-1-IP_pe	chr1	24340250	24340734	485	24340324	12	6.58417	4.27436
Nc-1-IP_pe	chr1	24350756	24351123	368	24350779	8	3.74505	3.07688
Nc-1-IP_pe	chr1	24353524	24353804	281	24353580	8	3.74505	3.07688
Nc-1-IP_pe	chr1	24452914	24453395	482	24453294	8	3.74505	3.07688
Nc-1-IP_pe	chr1	24489914	24490222	309	24490062	9	3.79246	3.00015
Nc-1-IP_pe	chr1	24496522	24496777	256	24496687	11	5.10577	3.5486
Nc-1-IP_pe	chr1	24556150	24556872	723	24556204	12	3.16137	2.39719
Nc-1-IP_pe	chr1	24558053	24558358	306	24558173	9	3.90961	3.07183
Nc-1-IP_pe	chr1	24583928	24584399	472	24584044	12	6.86906	4.44438
Nc-1-IP_pe	chr1	24585650	24585926	277	24585838	9	3.72192	2.95716
Nc-1-IP_pe	chr1	24597037	24597315	279	24597072	9	3.72192	2.95716
Nc-1-IP_pe	chr1	24642368	24642754	387	24642509	13	5.15976	3.33548
Nc-1-IP_pe	chr1	24669138	24669503	366	24669339	17	7.02067	3.79543
Nc-1-IP_pe	chr1	24685576	24686187	612	24685770	14	7.42849	4.43574
Nc-1-IP_pe	chr1	24700897	24701266	370	24701108	8	3.18323	2.7213

Nc-1-IP_pe	chr1	24704437	24704908	472	24704745	18	8.37884	4.31591
Nc-1-IP_pe	chr1	24712379	24712649	271	24712543	14	7.26231	4.34261
Nc-1-IP_pe	chr1	24720036	24720745	710	24720177	19	9.92774	4.92358
Nc-1-IP_pe	chr1	24721032	24721539	508	24721363	14	5.43414	3.36584
Nc-1-IP_pe	chr1	24725349	24725718	370	24725508	12	4.39405	3.03475
Nc-1-IP_pe	chr1	24736090	24736395	306	24736326	10	3.89064	2.95552
Nc-1-IP_pe	chr1	24742613	24742892	280	24742729	17	6.47874	3.54137
Nc-1-IP_pe	chr1	24745508	24745911	404	24745611	17	6.13258	3.38303
Nc-1-IP_pe	chr1	24781290	24781997	708	24781815	11	6.03439	4.10251
Nc-1-IP_pe	chr1	24797238	24797510	273	24797354	11	6.03439	4.10251
Nc-1-IP_pe	chr1	24799364	24799728	365	24799611	14	8.63073	5.12814
Nc-1-IP_pe	chr1	24813697	24813972	276	24813907	8	3.74505	3.07688
Nc-1-IP_pe	chr1	24832744	24833179	436	24832873	10	5.23357	3.76063
Nc-1-IP_pe	chr1	24833448	24834076	629	24833768	14	8.63073	5.12814
Nc-1-IP_pe	chr1	24848502	24848810	309	24848594	10	4.07292	3.06202
Nc-1-IP_pe	chr1	24862505	24863053	549	24862989	10	3.94999	2.99009
Nc-1-IP_pe	chr1	24891921	24892501	581	24892029	10	5.23357	3.76063
Nc-1-IP_pe	chr1	24895930	24896251	322	24896017	10	5.23357	3.76063
Nc-1-IP_pe	chr1	24898036	24898401	366	24898099	9	3.92293	3.08
Nc-1-IP_pe	chr1	24907136	24907430	295	24907367	10	5.23357	3.76063
Nc-1-IP_pe	chr1	24913588	24913854	267	24913697	10	4.62142	3.388
Nc-1-IP_pe	chr1	24914222	24914855	634	24914378	11	5.91198	4.02837
Nc-1-IP_pe	chr1	24930356	24930868	513	24930466	10	5.23357	3.76063
Nc-1-IP_pe	chr1	24931354	24931669	316	24931495	10	5.23357	3.76063
Nc-1-IP_pe	chr1	24932448	24932740	293	24932544	10	3.89064	2.95552
Nc-1-IP_pe	chr1	24935212	24935507	296	24935492	8	3.5501	2.95276
Nc-1-IP_pe	chr1	24951265	24951567	303	24951309	11	3.28475	2.53029
Nc-1-IP_pe	chr1	24965066	24965498	433	24965100	10	5.23357	3.76063
Nc-1-IP_pe	chr1	24966666	24967179	514	24966874	10	4.39571	3.25288
Nc-1-IP_pe	chr1	24971415	24971824	410	24971716	10	5.23357	3.76063
Nc-1-IP_pe	chr1	24989981	24990267	287	24990173	8	3.74505	3.07688
Nc-1-IP_pe	chr1	24993026	24993392	367	24993141	12	6.86906	4.44438
Nc-1-IP_pe	chr1	25006889	25007690	802	25007237	11	6.03439	4.10251
Nc-1-IP_pe	chr1	25011980	25012294	315	25012030	9	4.46937	3.41876
Nc-1-IP_pe	chr1	25018730	25019026	297	25018958	12	6.58417	4.27436
Nc-1-IP_pe	chr1	25020276	25020577	302	25020570	8	3.74505	3.07688
Nc-1-IP_pe	chr1	25031250	25031544	295	25031343	11	5.52654	3.79706
Nc-1-IP_pe	chr1	25033958	25034332	375	25034064	10	5.23357	3.76063
Nc-1-IP_pe	chr1	25049411	25049762	352	25049623	9	4.46937	3.41876
Nc-1-IP_pe	chr1	25051869	25052430	562	25052097	9	4.46937	3.41876
Nc-1-IP_pe	chr1	25063916	25064195	280	25064066	12	6.58417	4.27436
Nc-1-IP_pe	chr1	25065317	25065862	546	25065456	13	7.7352	4.78626
Nc-1-IP_pe	chr1	25067123	25067435	313	25067245	9	4.46937	3.41876
Nc-1-IP_pe	chr1	25088197	25088454	258	25088328	9	4.46937	3.41876
Nc-1-IP_pe	chr1	25091452	25091814	363	25091787	8	3.74505	3.07688

Nc-1-IP_pe	chr1	25139247	25139551	305	25139429	9	4.25966	3.28797
Nc-1-IP_pe	chr1	25195563	25196251	689	25195630	9	4.46937	3.41876
Nc-1-IP_pe	chr1	25197379	25197742	364	25197700	8	3.74505	3.07688
Nc-1-IP_pe	chr1	25198699	25199016	318	25198890	12	6.86906	4.44438
Nc-1-IP_pe	chr1	25286338	25286912	575	25286469	16	6.24025	3.54005
Nc-1-IP_pe	chr1	25294760	25295080	321	25294995	10	3.4604	2.70797
Nc-1-IP_pe	chr1	25336971	25337474	504	25337257	14	5.51541	3.4073
Nc-1-IP_pe	chr1	25338080	25338861	782	25338674	10	4.39571	3.25288
Nc-1-IP_pe	chr1	25375188	25375560	373	25375343	17	11.4763	6.15376
Nc-1-IP_pe	chr1	25391001	25391269	269	25391034	7	2.9043	2.63038
Nc-1-IP_pe	chr1	25413003	25413428	426	25413138	8	3.74505	3.07688
Nc-1-IP_pe	chr1	25424962	25425472	511	25425370	8	3.74505	3.07688
Nc-1-IP_pe	chr1	25518098	25518358	261	25518172	12	6.86906	4.44438
Nc-1-IP_pe	chr1	25523177	25523818	642	25523555	11	5.10577	3.5486
Nc-1-IP_pe	chr1	25524445	25524744	300	25524488	8	3.64414	3.01254
Nc-1-IP_pe	chr1	25540734	25541011	278	25540811	11	5.65062	3.87116
Nc-1-IP_pe	chr1	25543603	25544157	555	25543842	14	7.54286	4.50023
Nc-1-IP_pe	chr1	25549656	25550149	494	25549915	9	2.89249	2.46179
Nc-1-IP_pe	chr1	25555205	25555557	353	25555241	10	3.60969	2.79325
Nc-1-IP_pe	chr1	25560547	25560921	375	25560666	8	3.4091	2.86346
Nc-1-IP_pe	chr1	25583936	25584760	825	25584204	14	6.69566	4.03025
Nc-1-IP_pe	chr1	25587333	25587635	303	25587483	9	4.46937	3.41876
Nc-1-IP_pe	chr1	25616078	25616428	351	25616295	9	4.46937	3.41876
Nc-1-IP_pe	chr1	25685415	25685672	258	25685612	11	4.88957	3.42271
Nc-1-IP_pe	chr1	25692601	25692856	256	25692738	11	6.03439	4.10251
Nc-1-IP_pe	chr1	25695226	25695574	349	25695468	14	7.75387	4.62
Nc-1-IP_pe	chr1	25698151	25698689	539	25698336	8	3.74505	3.07688
Nc-1-IP_pe	chr1	25706308	25706564	257	25706377	10	5.23357	3.76063
Nc-1-IP_pe	chr1	25725693	25725951	259	25725833	10	5.23357	3.76063
Nc-1-IP_pe	chr1	25759024	25759716	693	25759346	10	5.23357	3.76063
Nc-1-IP_pe	chr1	25765901	25766225	325	25766096	8	3.74505	3.07688
Nc-1-IP_pe	chr1	25766496	25767015	520	25766608	10	4.99888	3.61677
Nc-1-IP_pe	chr1	25770109	25770593	485	25770500	11	5.77463	3.94557
Nc-1-IP_pe	chr1	25777918	25778368	451	25778038	11	6.03439	4.10251
Nc-1-IP_pe	chr1	25791559	25792054	496	25791900	9	4.46937	3.41876
Nc-1-IP_pe	chr1	25796110	25796570	461	25796410	9	4.46937	3.41876
Nc-1-IP_pe	chr1	25810472	25810953	482	25810913	9	3.42387	2.77697
Nc-1-IP_pe	chr1	25819986	25820555	570	25820416	11	4.61014	3.26192
Nc-1-IP_pe	chr1	25826440	25826824	385	25826655	10	3.89064	2.95552
Nc-1-IP_pe	chr1	25834337	25834602	266	25834435	9	4.46937	3.41876
Nc-1-IP_pe	chr1	25860401	25860675	275	25860489	9	3.72192	2.95716
Nc-1-IP_pe	chr1	25863514	25863858	345	25863652	11	6.03439	4.10251
Nc-1-IP_pe	chr1	25866913	25867226	314	25867019	12	6.86906	4.44438
Nc-1-IP_pe	chr1	25869845	25870106	262	25870061	10	3.08907	2.49869
Nc-1-IP_pe	chr1	25872269	25872713	445	25872698	8	3.56021	2.95918

Nc-1-IP_pe	chr1	25874310	25875527	1218	25875247	12	6.50787	4.22912
Nc-1-IP_pe	chr1	25906768	25907320	553	25906867	12	5.23082	3.49289
Nc-1-IP_pe	chr1	25937528	25937782	255	25937603	10	5.23357	3.76063
Nc-1-IP_pe	chr1	25947865	25948124	260	25948005	10	5.23357	3.76063
Nc-1-IP_pe	chr1	25953214	25953499	286	25953423	10	5.23357	3.76063
Nc-1-IP_pe	chr1	26021639	26023059	1421	26022610	11	6.03439	4.10251
Nc-1-IP_pe	chr1	26028272	26028844	573	26028443	10	4.99888	3.61677
Nc-1-IP_pe	chr1	26033475	26033752	278	26033659	9	4.2632	3.29017
Nc-1-IP_pe	chr1	26037833	26038388	556	26037917	9	3.27326	2.68684
Nc-1-IP_pe	chr1	26038651	26038973	323	26038772	11	4.06403	2.95415
Nc-1-IP_pe	chr1	26057215	26057552	338	26057364	14	5.69896	3.50164
Nc-1-IP_pe	chr1	26073664	26074043	380	26073792	12	6.58417	4.27436
Nc-1-IP_pe	chr1	26111042	26111336	295	26111276	16	7.79347	4.31685
Nc-1-IP_pe	chr1	26144149	26144425	277	26144314	11	6.03439	4.10251
Nc-1-IP_pe	chr1	26145649	26146312	664	26145778	11	5.3563	3.696
Nc-1-IP_pe	chr1	26156505	26157201	697	26156933	11	4.54404	3.2242
Nc-1-IP_pe	chr1	26161234	26161756	523	26161365	13	6.62436	4.14003
Nc-1-IP_pe	chr1	26168863	26169449	587	26169342	12	6.65086	4.31401
Nc-1-IP_pe	chr1	26171511	26171953	443	26171653	12	5.23082	3.49289
Nc-1-IP_pe	chr1	26180866	26181162	297	26180991	9	3.72192	2.95716
Nc-1-IP_pe	chr1	26202203	26203314	1112	26202311	9	3.72192	2.95716
Nc-1-IP_pe	chr1	26207276	26207545	270	26207402	11	6.03439	4.10251
Nc-1-IP_pe	chr1	26218653	26218978	326	26218866	11	6.03439	4.10251
Nc-1-IP_pe	chr1	26225083	26225718	636	26225220	12	6.58417	4.27436
Nc-1-IP_pe	chr1	26280850	26281283	434	26281230	12	3.47963	2.55766
Nc-1-IP_pe	chr1	26282002	26282440	439	26282172	11	5.25789	3.63791
Nc-1-IP_pe	chr1	26282838	26283862	1025	26283607	11	4.54404	3.2242
Nc-1-IP_pe	chr1	26286522	26286834	313	26286680	8	3.264	2.772
Nc-1-IP_pe	chr1	26289441	26289790	350	26289752	12	4.28158	2.97474
Nc-1-IP_pe	chr1	26294994	26295271	278	26295152	9	4.43361	3.39639
Nc-1-IP_pe	chr1	26301089	26301377	289	26301244	10	5.23357	3.76063
Nc-1-IP_pe	chr1	26303972	26304365	394	26304217	10	4.39571	3.25288
Nc-1-IP_pe	chr1	26307894	26308186	293	26308064	9	3.72192	2.95716
Nc-1-IP_pe	chr1	26310062	26310510	449	26310155	11	6.03439	4.10251
Nc-1-IP_pe	chr1	26336775	26337232	458	26336996	9	4.46937	3.41876
Nc-1-IP_pe	chr1	26341780	26342129	350	26341964	9	4.4177	3.38645
Nc-1-IP_pe	chr1	26343314	26343959	646	26343815	10	5.23357	3.76063
Nc-1-IP_pe	chr1	26346655	26346970	316	26346880	10	4.39571	3.25288
Nc-1-IP_pe	chr1	26350959	26351267	309	26351129	9	3.93633	3.08822
Nc-1-IP_pe	chr1	26367553	26367956	404	26367661	10	5.23357	3.76063
Nc-1-IP_pe	chr1	26370211	26370847	637	26370599	12	4.70082	3.20033
Nc-1-IP_pe	chr1	26372634	26373146	513	26372794	9	4.46937	3.41876
Nc-1-IP_pe	chr1	26411892	26412847	956	26412667	11	6.03439	4.10251
Nc-1-IP_pe	chr1	26471326	26471920	595	26471699	9	3.72192	2.95716
Nc-1-IP_pe	chr1	26523820	26524136	317	26523947	10	4.99888	3.61677

Nc-1-IP_pe	chr1	26530165	26530940	776	26530202	13	6.36468	3.99316
Nc-1-IP_pe	chr1	26532316	26532802	487	26532722	9	4.46937	3.41876
Nc-1-IP_pe	chr1	26541766	26542027	262	26541816	13	4.02562	2.7544
Nc-1-IP_pe	chr1	26545684	26546026	343	26545991	12	5.3037	3.53374
Nc-1-IP_pe	chr1	26573315	26573765	451	26573478	11	6.03439	4.10251
Nc-1-IP_pe	chr1	26577508	26577804	297	26577724	9	4.46937	3.41876
Nc-1-IP_pe	chr1	26596535	26596962	428	26596889	12	3.83688	2.74114
Nc-1-IP_pe	chr1	26695525	26695916	392	26695759	10	4.77495	3.48064
Nc-1-IP_pe	chr1	26696824	26697715	892	26697191	11	6.03439	4.10251
Nc-1-IP_pe	chr1	26705118	26705426	309	26705247	10	5.23357	3.76063
Nc-1-IP_pe	chr1	26708492	26708900	409	26708836	10	4.99888	3.61677
Nc-1-IP_pe	chr1	26755887	26756295	409	26756081	10	3.4604	2.70797
Nc-1-IP_pe	chr1	26773608	26773874	267	26773833	10	4.20113	3.13749
Nc-1-IP_pe	chr1	26779668	26780114	447	26779760	13	5.86991	3.71823
Nc-1-IP_pe	chr1	26803433	26804184	752	26803849	19	10.8111	5.37367
Nc-1-IP_pe	chr1	26818400	26819214	815	26818584	48	21.566	5.3461
Nc-1-IP_pe	chr1	26827823	26828100	278	26827954	11	6.03439	4.10251
Nc-1-IP_pe	chr1	26841021	26841611	591	26841190	8	3.08755	2.66145
Nc-1-IP_pe	chr1	26849754	26850093	340	26850012	14	4.42328	2.86573
Nc-1-IP_pe	chr1	26863416	26864325	910	26863857	10	4.33501	3.21677
Nc-1-IP_pe	chr1	26889808	26890263	456	26890163	11	2.96397	2.36091
Nc-1-IP_pe	chr1	26890548	26891125	578	26890992	12	6.86906	4.44438
Nc-1-IP_pe	chr1	26900080	26900546	467	26900373	23	16.2904	7.392
Nc-1-IP_pe	chr1	26901353	26901642	290	26901450	9	3.72192	2.95716
Nc-1-IP_pe	chr1	26942513	26942944	432	26942542	10	5.23357	3.76063
Nc-1-IP_pe	chr1	26951138	26951667	530	26951198	10	4.93609	3.57849
Nc-1-IP_pe	chr1	26959955	26961097	1143	26960625	16	10.5028	5.81189
Nc-1-IP_pe	chr1	26993157	26993416	260	26993308	10	4.99888	3.61677
Nc-1-IP_pe	chr1	26999452	26999767	316	26999608	9	3.27326	2.68684
Nc-1-IP_pe	chr1	27005313	27006540	1228	27005855	12	5.77527	3.80164
Nc-1-IP_pe	chr1	27010887	27011364	478	27011131	8	3.08755	2.66145
Nc-1-IP_pe	chr1	27033103	27033532	430	27033282	13	7.7352	4.78626
Nc-1-IP_pe	chr1	27035420	27036096	677	27036038	10	4.99888	3.61677
Nc-1-IP_pe	chr1	27037537	27038063	527	27037607	12	5.84943	3.84431
Nc-1-IP_pe	chr1	27061395	27061704	310	27061551	9	4.46937	3.41876
Nc-1-IP_pe	chr1	27063426	27063716	291	27063592	12	6.12485	4.004
Nc-1-IP_pe	chr1	27063974	27064509	536	27064470	11	2.96397	2.36091
Nc-1-IP_pe	chr1	27064831	27065097	267	27064948	12	5.61153	3.70794
Nc-1-IP_pe	chr1	27067088	27067348	261	27067175	10	5.10552	3.682
Nc-1-IP_pe	chr1	27106586	27106856	271	27106695	10	5.23357	3.76063
Nc-1-IP_pe	chr1	27110835	27111122	288	27110919	9	4.46937	3.41876
Nc-1-IP_pe	chr1	27122240	27122647	408	27122476	8	3.74505	3.07688
Nc-1-IP_pe	chr1	27137828	27138866	1039	27137830	9	4.25966	3.28797
Nc-1-IP_pe	chr1	27139154	27139671	518	27139538	11	4.47871	3.18705
Nc-1-IP_pe	chr1	27154740	27155162	423	27155047	11	5.7051	3.90381

Nc-1-IP_pe	chr1	27164893	27165145	253	27164897	9	2.89249	2.46179
Nc-1-IP_pe	chr1	27171489	27171770	282	27171708	10	5.23357	3.76063
Nc-1-IP_pe	chr1	27175580	27175843	264	27175736	8	3.74505	3.07688
Nc-1-IP_pe	chr1	27234758	27235192	435	27235017	11	3.64839	2.72584
Nc-1-IP_pe	chr1	27281342	27281857	516	27281749	9	4.46937	3.41876
Nc-1-IP_pe	chr1	27313830	27314145	316	27313970	12	6.86906	4.44438
Nc-1-IP_pe	chr1	27321330	27321666	337	27321570	10	5.23357	3.76063
Nc-1-IP_pe	chr1	27321936	27322327	392	27322099	16	8.26996	4.56762
Nc-1-IP_pe	chr1	27327312	27327583	272	27327468	13	7.7352	4.78626
Nc-1-IP_pe	chr1	27332849	27333300	452	27332904	15	7.95672	4.56362
Nc-1-IP_pe	chr1	27334005	27334831	827	27334765	10	4.39571	3.25288
Nc-1-IP_pe	chr1	27341052	27341323	272	27341132	10	4.88691	3.54856
Nc-1-IP_pe	chr1	27342741	27343069	329	27342898	19	8.68415	4.32024
Nc-1-IP_pe	chr1	27345326	27345625	300	27345486	11	5.20969	3.60954
Nc-1-IP_pe	chr1	27347191	27347480	290	27347434	12	5.3037	3.53374
Nc-1-IP_pe	chr1	27350265	27350701	437	27350511	11	5.10577	3.5486
Nc-1-IP_pe	chr1	27351164	27351505	342	27351385	9	4.40188	3.37657
Nc-1-IP_pe	chr1	27355978	27356947	970	27356856	12	5.77527	3.80164
Nc-1-IP_pe	chr1	27364086	27364556	471	27364362	13	6.92473	4.312
Nc-1-IP_pe	chr1	27394242	27394741	500	27394308	11	5.10577	3.5486
Nc-1-IP_pe	chr1	27397063	27397491	429	27397193	12	6.86906	4.44438
Nc-1-IP_pe	chr1	27401800	27402451	652	27402268	11	5.77463	3.94557
Nc-1-IP_pe	chr1	27405011	27405337	327	27405160	10	5.23357	3.76063
Nc-1-IP_pe	chr1	27409707	27410205	499	27410004	12	6.86906	4.44438
Nc-1-IP_pe	chr1	27515470	27515874	405	27515782	7	3.06442	2.73501
Nc-1-IP_pe	chr1	27519216	27520020	805	27519909	11	5.77463	3.94557
Nc-1-IP_pe	chr1	27525972	27526270	299	27526084	13	6.02837	3.80557
Nc-1-IP_pe	chr1	27539426	27539858	433	27539516	11	5.68685	3.89286
Nc-1-IP_pe	chr1	27541118	27541413	296	27541160	11	5.54401	3.80747
Nc-1-IP_pe	chr1	27543129	27543539	411	27543280	12	4.88508	3.30112
Nc-1-IP_pe	chr1	27545016	27545568	553	27545183	10	5.12294	3.69268
Nc-1-IP_pe	chr1	27549620	27550139	520	27549982	8	3.74505	3.07688
Nc-1-IP_pe	chr1	27551437	27551778	342	27551733	9	4.46937	3.41876
Nc-1-IP_pe	chr1	27552210	27552803	594	27552601	9	3.72192	2.95716
Nc-1-IP_pe	chr1	27555954	27556339	386	27556072	10	4.85453	3.52889
Nc-1-IP_pe	chr1	27568081	27568420	340	27568324	14	4.60011	2.95114
Nc-1-IP_pe	chr1	27570779	27571143	365	27571010	10	4.82246	3.50943
Nc-1-IP_pe	chr1	27571407	27571762	356	27571542	10	4.87068	3.5387
Nc-1-IP_pe	chr1	27584795	27585053	259	27584826	8	3.08755	2.66145
Nc-1-IP_pe	chr1	27588645	27589345	701	27588659	10	3.08907	2.49869
Nc-1-IP_pe	chr1	27589978	27590420	443	27590082	8	3.74505	3.07688
Nc-1-IP_pe	chr1	27592229	27592562	334	27592546	10	3.4604	2.70797
Nc-1-IP_pe	chr1	27593541	27593883	343	27593782	10	5.23357	3.76063
Nc-1-IP_pe	chr1	27594195	27595037	843	27594983	9	4.4496	3.40639
Nc-1-IP_pe	chr1	27597557	27598867	1311	27598655	17	8.31233	4.43123

Nc-1-IP_pe	chr1	27603336	27603643	308	27603445	9	3.65577	2.91697
Nc-1-IP_pe	chr1	27611584	27612027	444	27611674	8	3.74505	3.07688
Nc-1-IP_pe	chr1	27618736	27619055	320	27619002	8	3.74505	3.07688
Nc-1-IP_pe	chr1	27619520	27619786	267	27619581	8	3.74505	3.07688
Nc-1-IP_pe	chr1	27633154	27633607	454	27633519	11	6.03439	4.10251
Nc-1-IP_pe	chr1	27634399	27634694	296	27634670	8	3.74505	3.07688
Nc-1-IP_pe	chr1	27635006	27635690	685	27635478	11	6.03439	4.10251
Nc-1-IP_pe	chr1	27647762	27648152	391	27647957	11	6.03439	4.10251
Nc-1-IP_pe	chr1	27648574	27648926	353	27648627	9	3.92293	3.08
Nc-1-IP_pe	chr1	27655238	27655510	273	27655480	11	6.03439	4.10251
Nc-1-IP_pe	chr1	27726299	27726614	316	27726414	9	4.46937	3.41876
Nc-1-IP_pe	chr1	27773107	27773498	392	27773309	13	6.62436	4.14003
Nc-1-IP_pe	chr1	27791484	27791801	318	27791655	15	9.19325	5.26076
Nc-1-IP_pe	chr1	27796375	27796838	464	27796470	9	4.46937	3.41876
Nc-1-IP_pe	chr1	27830670	27831360	691	27830804	14	7.42849	4.43574
Nc-1-IP_pe	chr1	27858305	27859156	852	27859085	8	3.51085	2.92785
Nc-1-IP_pe	chr1	27859437	27859800	364	27859698	9	4.17427	3.23498
Nc-1-IP_pe	chr1	27873392	27873644	253	27873523	9	4.46937	3.41876
Nc-1-IP_pe	chr1	27874810	27875100	291	27874961	10	4.39571	3.25288
Nc-1-IP_pe	chr1	27876731	27877194	464	27876962	12	6.6095	4.28941
Nc-1-IP_pe	chr1	27877529	27877808	280	27877701	11	5.5792	3.82847
Nc-1-IP_pe	chr1	27885276	27885639	364	27885495	13	7.7352	4.78626
Nc-1-IP_pe	chr1	27899759	27900021	263	27899885	11	6.03439	4.10251
Nc-1-IP_pe	chr1	27935086	27935423	338	27935272	10	5.23357	3.76063
Nc-1-IP_pe	chr1	27954324	27955031	708	27954901	10	5.23357	3.76063
Nc-1-IP_pe	chr1	27966436	27966697	262	27966593	14	7.14701	4.27839
Nc-1-IP_pe	chr1	28090465	28090778	314	28090673	12	6.86906	4.44438
Nc-1-IP_pe	chr1	28121371	28121671	301	28121476	11	6.03439	4.10251
Nc-1-IP_pe	chr1	28143228	28143746	519	28143729	8	3.74505	3.07688
Nc-1-IP_pe	chr1	28148506	28149053	548	28148978	8	3.74505	3.07688
Nc-1-IP_pe	chr1	28153358	28153690	333	28153467	8	3.08755	2.66145
Nc-1-IP_pe	chr1	28171619	28172388	770	28171811	11	6.03439	4.10251
Nc-1-IP_pe	chr1	28174027	28174471	445	28174282	8	3.74505	3.07688
Nc-1-IP_pe	chr1	28194307	28194785	479	28194690	15	9.09658	5.20507
Nc-1-IP_pe	chr1	28232600	28233039	440	28232625	15	6.19428	3.63445
Nc-1-IP_pe	chr1	28233606	28233869	264	28233740	9	4.46937	3.41876
Nc-1-IP_pe	chr1	28259524	28259914	391	28259735	14	5.56442	3.43239
Nc-1-IP_pe	chr1	28265488	28265826	339	28265672	10	5.15807	3.71423
Nc-1-IP_pe	chr1	28276973	28277364	392	28277040	10	5.23357	3.76063
Nc-1-IP_pe	chr1	28280641	28281065	425	28280820	11	6.03439	4.10251
Nc-1-IP_pe	chr1	28282216	28283115	900	28282312	10	5.23357	3.76063
Nc-1-IP_pe	chr1	28326093	28326467	375	28326382	7	3.06442	2.73501
Nc-1-IP_pe	chr1	28353546	28353799	254	28353637	7	3.06442	2.73501
Nc-1-IP_pe	chr1	28466483	28466790	308	28466613	10	5.23357	3.76063
Nc-1-IP_pe	chr1	28490312	28490624	313	28490427	10	5.23357	3.76063

Nc-1-IP_pe	chr1	28507710	28508004	295	28507841	13	7.34205	4.55444
Nc-1-IP_pe	chr1	28515633	28516155	523	28516114	10	4.39571	3.25288
Nc-1-IP_pe	chr1	28530688	28530977	290	28530908	7	3.06442	2.73501
Nc-1-IP_pe	chr1	28534943	28535509	567	28535063	9	4.25966	3.28797
Nc-1-IP_pe	chr1	28563263	28563580	318	28563409	10	4.93609	3.57849
Nc-1-IP_pe	chr1	28571772	28572207	436	28572130	8	3.74505	3.07688
Nc-1-IP_pe	chr1	28581259	28581659	401	28581633	9	3.72192	2.95716
Nc-1-IP_pe	chr1	28593756	28594135	380	28593957	13	7.34205	4.55444
Nc-1-IP_pe	chr1	28610477	28610939	463	28610897	10	5.23357	3.76063
Nc-1-IP_pe	chr1	28620311	28620606	296	28620463	10	5.23357	3.76063
Nc-1-IP_pe	chr1	28647321	28647634	314	28647448	10	5.23357	3.76063
Nc-1-IP_pe	chr1	28666217	28666701	485	28666371	12	6.86906	4.44438
Nc-1-IP_pe	chr1	28674928	28675180	253	28675095	8	3.56021	2.95918
Nc-1-IP_pe	chr1	28708196	28708531	336	28708399	10	5.23357	3.76063
Nc-1-IP_pe	chr1	28737014	28737526	513	28737199	12	5.45448	3.61875
Nc-1-IP_pe	chr1	28780617	28780873	257	28780788	8	3.74505	3.07688
Nc-1-IP_pe	chr1	28796765	28797118	354	28797029	11	5.10577	3.5486
Nc-1-IP_pe	chr1	28816991	28817317	327	28817122	9	4.46937	3.41876
Nc-1-IP_pe	chr1	28818696	28819168	473	28818854	7	3.06442	2.73501
Nc-1-IP_pe	chr1	28820523	28820814	292	28820603	10	5.10552	3.682
Nc-1-IP_pe	chr1	28836467	28836723	257	28836532	7	3.06442	2.73501
Nc-1-IP_pe	chr1	28844933	28845272	340	28845217	8	3.74505	3.07688
Nc-1-IP_pe	chr1	28854688	28855022	335	28854908	8	3.74505	3.07688
Nc-1-IP_pe	chr1	28862898	28863482	585	28863387	12	6.86906	4.44438
Nc-1-IP_pe	chr1	28873572	28873842	271	28873711	11	6.03439	4.10251
Nc-1-IP_pe	chr1	28889277	28889553	277	28889313	9	4.25966	3.28797
Nc-1-IP_pe	chr1	28896703	28896976	274	28896833	9	4.25966	3.28797
Nc-1-IP_pe	chr1	28914642	28914989	348	28914978	15	4.06016	2.62146
Nc-1-IP_pe	chr1	29031784	29032069	286	29031934	11	6.03439	4.10251
Nc-1-IP_pe	chr1	29116359	29116618	260	29116528	8	3.74505	3.07688
Nc-1-IP_pe	chr1	29121239	29121540	302	29121391	10	4.41822	3.2663
Nc-1-IP_pe	chr1	29123416	29123771	356	29123569	16	8.17084	4.51499
Nc-1-IP_pe	chr1	29124976	29125296	321	29125151	13	6.45312	4.04299
Nc-1-IP_pe	chr1	29132015	29132429	415	29132316	9	4.46937	3.41876
Nc-1-IP_pe	chr1	29133895	29134820	926	29134732	11	5.89269	4.01672
Nc-1-IP_pe	chr1	29191970	29192285	316	29192145	7	3.06442	2.73501
Nc-1-IP_pe	chr1	29193185	29193690	506	29193617	9	4.25966	3.28797
Nc-1-IP_pe	chr1	29196307	29196635	329	29196566	9	4.46937	3.41876
Nc-1-IP_pe	chr1	29237086	29237641	556	29237483	12	5.82577	3.83068
Nc-1-IP_pe	chr1	29245338	29245698	361	29245392	9	3.32589	2.71826
Nc-1-IP_pe	chr1	29249199	29249705	507	29249559	9	3.12089	2.59629
Nc-1-IP_pe	chr1	29257425	29258028	604	29257838	11	6.03439	4.10251
Nc-1-IP_pe	chr1	29273375	29273641	267	29273442	9	3.27326	2.68684
Nc-1-IP_pe	chr1	29283563	29283936	374	29283749	9	4.2632	3.29017
Nc-1-IP_pe	chr1	29308805	29309099	295	29308938	10	4.39571	3.25288

Nc-1-IP_pe	chr1	29310714	29311090	377	29310929	12	6.86906	4.44438
Nc-1-IP_pe	chr1	29311370	29311669	300	29311382	8	3.14944	2.70014
Nc-1-IP_pe	chr1	29319788	29320116	329	29319922	11	5.91198	4.02837
Nc-1-IP_pe	chr1	29322098	29322438	341	29322155	7	3.06442	2.73501
Nc-1-IP_pe	chr1	29325262	29326241	980	29325725	12	6.50787	4.22912
Nc-1-IP_pe	chr1	29329767	29330090	324	29329858	11	6.03439	4.10251
Nc-1-IP_pe	chr1	29341443	29341730	288	29341652	11	4.54404	3.2242
Nc-1-IP_pe	chr1	29394681	29395090	410	29394748	8	3.74505	3.07688
Nc-1-IP_pe	chr1	29414326	29414593	268	29414498	9	4.25966	3.28797
Nc-1-IP_pe	chr1	29442604	29442920	317	29442699	9	4.25966	3.28797
Nc-1-IP_pe	chr1	29461262	29461717	456	29461625	8	3.74505	3.07688
Nc-1-IP_pe	chr1	29498137	29498405	269	29498338	9	4.46937	3.41876
Nc-1-IP_pe	chr1	29517718	29517973	256	29517935	8	3.74505	3.07688
Nc-1-IP_pe	chr1	29548814	29549323	510	29549026	10	3.89064	2.95552
Nc-1-IP_pe	chr1	29550860	29551595	736	29550870	10	3.40382	2.67582
Nc-1-IP_pe	chr1	29555171	29555505	335	29555344	8	3.74505	3.07688
Nc-1-IP_pe	chr1	29556572	29556926	355	29556793	8	3.08755	2.66145
Nc-1-IP_pe	chr1	29561195	29561497	303	29561386	11	6.03439	4.10251
Nc-1-IP_pe	chr1	29573520	29573814	295	29573612	8	3.74505	3.07688
Nc-1-IP_pe	chr1	29575067	29575330	264	29575130	10	5.10552	3.682
Nc-1-IP_pe	chr1	29580772	29581109	338	29580849	8	3.74505	3.07688
Nc-1-IP_pe	chr1	29593100	29593373	274	29593260	10	5.23357	3.76063
Nc-1-IP_pe	chr1	29593936	29594266	331	29593988	10	4.99888	3.61677
Nc-1-IP_pe	chr1	29626292	29626852	561	29626367	8	3.74505	3.07688
Nc-1-IP_pe	chr1	29686204	29686824	621	29686399	10	5.23357	3.76063
Nc-1-IP_pe	chr1	29688695	29689023	329	29688798	11	6.03439	4.10251
Nc-1-IP_pe	chr1	29713025	29713300	276	29713163	9	4.46937	3.41876
Nc-1-IP_pe	chr1	29720167	29720421	255	29720275	8	3.74505	3.07688
Nc-1-IP_pe	chr1	29783099	29783404	306	29783401	9	3.27326	2.68684
Nc-1-IP_pe	chr1	29787740	29788049	310	29787919	9	4.46937	3.41876
Nc-1-IP_pe	chr1	29789731	29790213	483	29789817	9	4.46937	3.41876
Nc-1-IP_pe	chr1	29826625	29826906	282	29826822	10	5.10552	3.682
Nc-1-IP_pe	chr1	29828006	29828321	316	29828251	7	3.06442	2.73501
Nc-1-IP_pe	chr1	29829636	29830109	474	29829696	9	4.35492	3.34727
Nc-1-IP_pe	chr1	29879225	29879608	384	29879555	10	4.99888	3.61677
Nc-1-IP_pe	chr1	29895224	29895540	317	29895399	11	6.03439	4.10251
Nc-1-IP_pe	chr1	29969736	29969992	257	29969776	9	4.46937	3.41876
Nc-1-IP_pe	chr1	30010526	30011082	557	30010837	10	5.23357	3.76063
Nc-1-IP_pe	chr1	30040059	30040558	500	30040208	11	5.7051	3.90381
Nc-1-IP_pe	chr1	30041795	30042285	491	30042047	11	6.03439	4.10251
Nc-1-IP_pe	chr1	30069880	30070164	285	30070091	9	4.25966	3.28797
Nc-1-IP_pe	chr1	30100892	30101239	348	30101022	13	7.42513	4.60316
Nc-1-IP_pe	chr1	30119933	30120203	271	30120108	8	3.74505	3.07688
Nc-1-IP_pe	chr1	30125400	30125822	423	30125697	7	3.06442	2.73501
Nc-1-IP_pe	chr1	30148860	30149383	524	30149255	10	5.23357	3.76063

Nc-1-IP_pe	chr1	30161951	30162385	435	30162211	13	7.7352	4.78626
Nc-1-IP_pe	chr1	30188043	30188445	403	30188257	8	3.74505	3.07688
Nc-1-IP_pe	chr1	30224456	30224756	301	30224544	10	5.23357	3.76063
Nc-1-IP_pe	chr1	30247960	30248390	431	30248311	8	3.56021	2.95918
Nc-1-IP_pe	chr1	30254098	30254380	283	30254253	14	6.61011	3.98382
Nc-1-IP_pe	chr1	30265189	30265638	450	30265299	9	4.46937	3.41876
Nc-1-IP_pe	chr1	30342593	30342961	369	30342705	12	4.70082	3.20033
Nc-1-IP_pe	chr1	30379183	30379499	317	30379358	13	7.42513	4.60316
Nc-1-IP_pe	chr1	30386793	30387133	341	30386857	8	3.74505	3.07688
Nc-1-IP_pe	chr1	30389570	30389951	382	30389791	14	8.63073	5.12814
Nc-1-IP_pe	chr1	30410203	30410874	672	30410294	10	5.23357	3.76063
Nc-1-IP_pe	chr1	30412038	30412490	453	30412381	9	4.46937	3.41876
Nc-1-IP_pe	chr1	30474486	30474775	290	30474706	9	4.05981	3.16422
Nc-1-IP_pe	chr1	30572957	30573288	332	30572987	9	4.20361	3.25317
Nc-1-IP_pe	chr1	30576710	30577024	315	30576910	10	4.99888	3.61677
Nc-1-IP_pe	chr1	30580572	30580855	284	30580748	10	5.23357	3.76063
Nc-1-IP_pe	chr1	30603216	30603539	324	30603380	10	4.99888	3.61677
Nc-1-IP_pe	chr1	30616759	30617261	503	30616934	13	4.86362	3.18015
Nc-1-IP_pe	chr1	30651400	30651940	541	30651812	18	4.6358	2.66698
Nc-1-IP_pe	chr1	30655633	30655918	286	30655851	13	3.07238	2.29378
Nc-1-IP_pe	chr1	30658511	30659194	684	30658711	11	5.74187	3.92588
Nc-1-IP_pe	chr1	30663799	30664051	253	30663900	9	4.46937	3.41876
Nc-1-IP_pe	chr1	30685033	30685323	291	30685309	10	3.83201	2.92147
Nc-1-IP_pe	chr1	30692933	30693644	712	30693049	10	5.23357	3.76063
Nc-1-IP_pe	chr1	30720302	30720808	507	30720669	9	4.11647	3.19921
Nc-1-IP_pe	chr1	30721729	30722178	450	30721937	9	3.66792	2.92434
Nc-1-IP_pe	chr1	30728572	30728916	345	30728726	12	6.86906	4.44438
Nc-1-IP_pe	chr1	30729885	30730203	319	30730114	9	3.66792	2.92434
Nc-1-IP_pe	chr1	30739738	30740160	423	30739755	9	4.05981	3.16422
Nc-1-IP_pe	chr1	30748522	30748960	439	30748747	11	6.03439	4.10251
Nc-1-IP_pe	chr1	30755940	30756311	372	30756030	9	4.46937	3.41876
Nc-1-IP_pe	chr1	30757539	30758136	598	30758034	11	5.10577	3.5486
Nc-1-IP_pe	chr1	30764712	30765238	527	30764844	8	3.74505	3.07688
Nc-1-IP_pe	chr1	30775571	30775981	411	30775815	11	6.03439	4.10251
Nc-1-IP_pe	chr1	30829085	30829436	352	30829229	10	5.23357	3.76063
Nc-1-IP_pe	chr1	30847445	30847754	310	30847535	11	5.3563	3.696
Nc-1-IP_pe	chr1	30881360	30881658	299	30881483	11	5.74187	3.92588
Nc-1-IP_pe	chr1	30906128	30906478	351	30906300	9	4.46937	3.41876
Nc-1-IP_pe	chr1	30906743	30907068	326	30906917	14	8.63073	5.12814
Nc-1-IP_pe	chr1	30911814	30912598	785	30912524	9	4.46937	3.41876
Nc-1-IP_pe	chr1	30929354	30929621	268	30929408	10	3.89064	2.95552
Nc-1-IP_pe	chr1	30949090	30949619	530	30949439	10	5.23357	3.76063
Nc-1-IP_pe	chr1	31104993	31105767	775	31105153	11	5.77463	3.94557
Nc-1-IP_pe	chr1	31113575	31113895	321	31113705	10	5.23357	3.76063
Nc-1-IP_pe	chr1	31120630	31120939	310	31120747	9	4.46937	3.41876

Nc-1-IP_pe	chr1	31135262	31135754	493	31135707	9	4.25966	3.28797
Nc-1-IP_pe	chr1	31141734	31142093	360	31141987	9	4.46937	3.41876
Nc-1-IP_pe	chr1	31161102	31161359	258	31161130	9	4.46937	3.41876
Nc-1-IP_pe	chr1	31164324	31164722	399	31164470	9	4.46937	3.41876
Nc-1-IP_pe	chr1	31176638	31177052	415	31176890	12	4.24064	2.95299
Nc-1-IP_pe	chr1	31180009	31180340	332	31180306	10	5.23357	3.76063
Nc-1-IP_pe	chr1	31182131	31182388	258	31182180	8	3.0402	2.6319
Nc-1-IP_pe	chr1	31183597	31184111	515	31183926	16	10.5028	5.81189
Nc-1-IP_pe	chr1	31195003	31195429	427	31195170	7	3.06442	2.73501
Nc-1-IP_pe	chr1	31229409	31229922	514	31229842	9	4.20361	3.25317
Nc-1-IP_pe	chr1	31296359	31296679	321	31296484	14	6.78215	4.0774
Nc-1-IP_pe	chr1	31367437	31367742	306	31367597	8	3.56021	2.95918
Nc-1-IP_pe	chr1	31374565	31374860	296	31374750	10	4.39571	3.25288
Nc-1-IP_pe	chr1	31395001	31395430	430	31395134	11	5.10577	3.5486
Nc-1-IP_pe	chr1	31408934	31409320	387	31409274	8	3.51085	2.92785
Nc-1-IP_pe	chr1	31413395	31414038	644	31413542	13	5.9487	3.76157
Nc-1-IP_pe	chr1	31424871	31425210	340	31424876	11	3.64839	2.72584
Nc-1-IP_pe	chr1	31431750	31432194	445	31432086	209	60.5025	3.98367
Nc-1-IP_pe	chr1	31439303	31439881	579	31439475	11	6.03439	4.10251
Nc-1-IP_pe	chr1	31440975	31441261	287	31441114	12	6.86906	4.44438
Nc-1-IP_pe	chr1	31445029	31445286	258	31445051	7	3.06442	2.73501
Nc-1-IP_pe	chr1	31501234	31501654	421	31501461	18	8.62313	4.4354
Nc-1-IP_pe	chr1	31506190	31506491	302	31506344	17	6.85055	3.71486
Nc-1-IP_pe	chr1	31514004	31514273	270	31514087	13	3.77558	2.63125
Nc-1-IP_pe	chr1	31563585	31564008	424	31563784	14	5.18557	3.24018
Nc-1-IP_pe	chr1	31568971	31569364	394	31569019	16	6.29369	3.56571
Nc-1-IP_pe	chr1	31572606	31573336	731	31573059	17	5.77097	3.22087
Nc-1-IP_pe	chr1	31576420	31576987	568	31576544	19	5.90435	3.10381
Nc-1-IP_pe	chr1	31584542	31585485	944	31585335	22	9.15678	4.14117
Nc-1-IP_pe	chr1	31588665	31589380	716	31589069	17	6.79464	3.68855
Nc-1-IP_pe	chr1	31590525	31590802	278	31590634	12	3.98572	2.81868
Nc-1-IP_pe	chr1	31593130	31593606	477	31593190	14	6.06519	3.69269
Nc-1-IP_pe	chr1	31594180	31594456	277	31594341	10	3.40382	2.67582
Nc-1-IP_pe	chr1	31612337	31612743	407	31612420	9	2.87781	2.45319
Nc-1-IP_pe	chr1	31614277	31614847	571	31614301	12	4.51041	3.09723
Nc-1-IP_pe	chr1	31615362	31615855	494	31615630	13	4.84835	3.17221
Nc-1-IP_pe	chr1	31617790	31618740	951	31618052	19	6.78661	3.47027
Nc-1-IP_pe	chr1	31619201	31619586	386	31619385	16	6.24025	3.54005
Nc-1-IP_pe	chr1	31622554	31622878	325	31622675	14	4.60011	2.95114
Nc-1-IP_pe	chr1	31623149	31623704	556	31623603	21	7.58657	3.60451
Nc-1-IP_pe	chr1	31624781	31625579	799	31625424	17	7.43048	3.99256
Nc-1-IP_pe	chr1	31626061	31626449	389	31626217	17	5.9911	3.31919
Nc-1-IP_pe	chr1	31627395	31627862	468	31627490	14	5.30793	3.30182
Nc-1-IP_pe	chr1	31629962	31630884	923	31630215	13	4.90859	3.20357
Nc-1-IP_pe	chr1	31631986	31632806	821	31632247	13	4.86362	3.18015

Nc-1-IP_pe	chr1	31635279	31635579	301	31635373	16	6.71503	3.77075
Nc-1-IP_pe	chr1	31644378	31644952	575	31644653	26	11.7219	4.68487
Nc-1-IP_pe	chr1	31655301	31655646	346	31655451	13	5.42865	3.47872
Nc-1-IP_pe	chr1	31656022	31656532	511	31656326	16	7.53857	4.18505
Nc-1-IP_pe	chr1	31659311	31659754	444	31659392	13	3.35769	2.42919
Nc-1-IP_pe	chr1	31660970	31661343	374	31661263	10	2.93344	2.41216
Nc-1-IP_pe	chr1	31664350	31665003	654	31664584	16	4.96462	2.94973
Nc-1-IP_pe	chr1	31665996	31666268	273	31666140	17	5.96578	3.30782
Nc-1-IP_pe	chr1	31667316	31667743	428	31667388	21	8.7477	4.08833
Nc-1-IP_pe	chr1	31668077	31668824	748	31668579	19	6.78661	3.47027
Nc-1-IP_pe	chr1	31671232	31671633	402	31671587	15	4.79042	2.95436
Nc-1-IP_pe	chr1	31672791	31673083	293	31672949	14	4.48284	2.8944
Nc-1-IP_pe	chr1	31684308	31685032	725	31684406	18	3.77591	2.33276
Nc-1-IP_pe	chr1	31688483	31688767	285	31688742	15	4.06016	2.62146
Nc-1-IP_pe	chr1	31689394	31689825	432	31689665	16	6.3887	3.61154
Nc-1-IP_pe	chr1	31690254	31692116	1863	31690617	18	3.77591	2.33276
Nc-1-IP_pe	chr1	31694540	31695039	500	31694944	16	6.1352	3.48981
Nc-1-IP_pe	chr1	31696116	31696942	827	31696375	13	4.53922	3.01292
Nc-1-IP_pe	chr1	31697379	31697672	294	31697486	15	4.90347	3.00714
Nc-1-IP_pe	chr1	31699592	31700130	539	31699804	22	6.44373	3.08129
Nc-1-IP_pe	chr1	31700771	31701227	457	31700815	16	5.60875	3.24248
Nc-1-IP_pe	chr1	31704144	31704485	342	31704206	16	4.96462	2.94973
Nc-1-IP_pe	chr1	31705374	31705884	511	31705576	16	5.43892	3.16425
Nc-1-IP_pe	chr1	31707721	31707984	264	31707849	21	6.66991	3.24296
Nc-1-IP_pe	chr1	31709172	31709684	513	31709486	19	7.05262	3.58428
Nc-1-IP_pe	chr1	31713390	31713644	255	31713532	16	2.92499	2.08721
Nc-1-IP_pe	chr1	31714390	31715573	1184	31715391	23	6.62483	3.0751
Nc-1-IP_pe	chr1	31725099	31725724	626	31725126	13	3.95529	2.71958
Nc-1-IP_pe	chr1	31732391	31732683	293	31732547	15	4.7819	2.95039
Nc-1-IP_pe	chr1	31735429	31735726	298	31735540	15	5.66913	3.37372
Nc-1-IP_pe	chr1	31736901	31737214	314	31736984	15	4.7819	2.95039
Nc-1-IP_pe	chr1	31739064	31739530	467	31739298	11	3.58722	2.69267
Nc-1-IP_pe	chr1	31741431	31741985	555	31741797	15	4.90347	3.00714
Nc-1-IP_pe	chr1	31742530	31743017	488	31742590	14	3.55308	2.45762
Nc-1-IP_pe	chr1	31744076	31744633	558	31744137	13	4.24481	2.8638
Nc-1-IP_pe	chr1	31745472	31746419	948	31745620	17	4.78101	2.79343
Nc-1-IP_pe	chr1	31747876	31748439	564	31748072	13	4.2147	2.84869
Nc-1-IP_pe	chr1	31750904	31751167	264	31751082	17	3.84884	2.41145
Nc-1-IP_pe	chr1	31752852	31753238	387	31753090	17	5.35342	3.03768
Nc-1-IP_pe	chr1	31753853	31754147	295	31753866	14	3.40897	2.39188
Nc-1-IP_pe	chr1	31762887	31763245	359	31763021	20	7.19732	3.54396
Nc-1-IP_pe	chr1	31764493	31764752	260	31764590	13	4.03953	2.7613
Nc-1-IP_pe	chr1	31765051	31765435	385	31765171	15	4.40338	2.77622
Nc-1-IP_pe	chr1	31765738	31766026	289	31765774	13	4.33674	2.9101
Nc-1-IP_pe	chr1	31770339	31771005	667	31770753	13	3.86418	2.67468

Nc-1-IP_pe	chr1	31771599	31772292	694	31772122	15	4.7819	2.95039
Nc-1-IP_pe	chr1	31773002	31773461	460	31773258	17	6.78076	3.68203
Nc-1-IP_pe	chr1	31775639	31776047	409	31775964	12	4.24064	2.95299
Nc-1-IP_pe	chr1	31783603	31783936	334	31783650	15	6.34879	3.71261
Nc-1-IP_pe	chr1	31787104	31787793	690	31787256	18	6.15736	3.29676
Nc-1-IP_pe	chr1	31789207	31789701	495	31789587	15	5.20142	3.14789
Nc-1-IP_pe	chr1	31790679	31791643	965	31791426	15	5.48589	3.28453
Nc-1-IP_pe	chr1	31795939	31796569	631	31796090	18	7.47506	3.88659
Nc-1-IP_pe	chr1	31799369	31800208	840	31799489	16	4.59224	2.7853
Nc-1-IP_pe	chr1	31811662	31812067	406	31811910	18	7.1522	3.73811
Nc-1-IP_pe	chr1	31813933	31814291	359	31814040	16	6.33285	3.58457
Nc-1-IP_pe	chr1	31828418	31828979	562	31828861	15	6.63607	3.85965
Nc-1-IP_pe	chr1	31829247	31829777	531	31829422	19	7.31609	3.69885
Nc-1-IP_pe	chr1	31837904	31838246	343	31838176	11	4.06403	2.95415
Nc-1-IP_pe	chr1	31848145	31849077	933	31848967	15	5.00915	3.05678
Nc-1-IP_pe	chr1	31854150	31854420	271	31854194	10	3.60969	2.79325
Nc-1-IP_pe	chr1	31861063	31861472	410	31861296	12	5.69253	3.7542
Nc-1-IP_pe	chr1	31865623	31866182	560	31865994	10	3.08907	2.49869
Nc-1-IP_pe	chr1	31890521	31890781	261	31890696	13	4.14549	2.81406
Nc-1-IP_pe	chr1	31904971	31905261	291	31905250	10	3.08907	2.49869
Nc-1-IP_pe	chr1	31914078	31914358	281	31914176	16	8.26996	4.56762
Nc-1-IP_pe	chr1	31937378	31937892	515	31937865	16	4.25252	2.63824
Nc-1-IP_pe	chr1	31942639	31942941	303	31942721	16	6.33285	3.58457
Nc-1-IP_pe	chr1	31945013	31945393	381	31945104	12	4.92484	3.32299
Nc-1-IP_pe	chr1	31956868	31957126	259	31956915	15	5.1177	3.10809
Nc-1-IP_pe	chr1	31958764	31959458	695	31959330	15	5.6578	3.36818
Nc-1-IP_pe	chr1	31973721	31973974	254	31973802	10	3.7298	2.86234
Nc-1-IP_pe	chr1	31974283	31974553	271	31974516	12	4.24064	2.95299
Nc-1-IP_pe	chr1	31985942	31986214	273	31986137	13	4.86362	3.18015
Nc-1-IP_pe	chr1	31986909	31987584	676	31987500	14	5.06689	3.18081
Nc-1-IP_pe	chr1	31992006	31992590	585	31992359	15	6.78905	3.93887
Nc-1-IP_pe	chr1	31994366	31994688	323	31994478	11	4.35236	3.11555
Nc-1-IP_pe	chr1	32004240	32004561	322	32004462	19	7.84735	3.93486
Nc-1-IP_pe	chr1	32013237	32014152	916	32013945	23	6.62483	3.0751
Nc-1-IP_pe	chr1	32014408	32014744	337	32014470	14	3.63457	2.49502
Nc-1-IP_pe	chr1	32021678	32022036	359	32021742	13	7.02546	4.37016
Nc-1-IP_pe	chr1	32040143	32040484	342	32040201	11	4.06403	2.95415
Nc-1-IP_pe	chr1	32055322	32055740	419	32055498	12	5.84943	3.84431
Nc-1-IP_pe	chr1	32064366	32064873	508	32064741	9	3.27326	2.68684
Nc-1-IP_pe	chr1	32067174	32067810	637	32067569	12	5.84943	3.84431
Nc-1-IP_pe	chr1	32068297	32068552	256	32068442	12	5.23082	3.49289
Nc-1-IP_pe	chr1	32070579	32071496	918	32070639	13	4.02562	2.7544
Nc-1-IP_pe	chr1	32072668	32073101	434	32072842	15	4.90347	3.00714
Nc-1-IP_pe	chr1	32076530	32076991	462	32076830	11	4.78913	3.36466
Nc-1-IP_pe	chr1	32081179	32081595	417	32081339	13	7.49774	4.64586

Nc-1-IP_pe	chr1	32083633	32083931	299	32083679	9	4.27829	3.29955
Nc-1-IP_pe	chr1	32090660	32090953	294	32090724	10	3.87863	2.94853
Nc-1-IP_pe	chr1	32098482	32099076	595	32098682	14	6.0854	3.70334
Nc-1-IP_pe	chr1	32108061	32108544	484	32108392	14	6.28469	3.80898
Nc-1-IP_pe	chr1	32113220	32113576	357	32113396	10	4.80655	3.49978
Nc-1-IP_pe	chr1	32132757	32133371	615	32133024	8	3.74505	3.07688
Nc-1-IP_pe	chr1	32133969	32134257	289	32134221	7	3.06442	2.73501
Nc-1-IP_pe	chr1	32135120	32135428	309	32135280	12	6.58417	4.27436
Nc-1-IP_pe	chr1	32162357	32162892	536	32162422	14	6.78215	4.0774
Nc-1-IP_pe	chr1	32179273	32179689	417	32179388	14	5.03085	3.16286
Nc-1-IP_pe	chr1	32180357	32180768	412	32180457	15	7.37761	4.24941
Nc-1-IP_pe	chr1	32187468	32187982	515	32187727	10	3.08907	2.49869
Nc-1-IP_pe	chr1	32192202	32192549	348	32192400	13	6.32982	3.97358
Nc-1-IP_pe	chr1	32205460	32205890	431	32205600	16	2.92499	2.08721
Nc-1-IP_pe	chr1	32206540	32206829	290	32206602	14	4.21449	2.76599
Nc-1-IP_pe	chr1	32225631	32226250	620	32226183	13	5.74741	3.65119
Nc-1-IP_pe	chr1	32235107	32235365	259	32235215	11	4.23076	3.04719
Nc-1-IP_pe	chr1	32250913	32251582	670	32251270	13	5.79292	3.67605
Nc-1-IP_pe	chr1	32263060	32263327	268	32263162	14	8.06563	4.79881
Nc-1-IP_pe	chr1	32267485	32268055	571	32267607	12	5.84943	3.84431
Nc-1-IP_pe	chr1	32274416	32274763	348	32274704	13	3.35769	2.42919
Nc-1-IP_pe	chr1	32279753	32280084	332	32279774	9	3.27326	2.68684
Nc-1-IP_pe	chr1	32285600	32286093	494	32285736	10	5.23357	3.76063
Nc-1-IP_pe	chr1	32287382	32287645	264	32287438	8	3.74505	3.07688
Nc-1-IP_pe	chr1	32292168	32292470	303	32292299	13	4.67336	3.0817
Nc-1-IP_pe	chr1	32308648	32308969	322	32308810	13	5.36853	3.44651
Nc-1-IP_pe	chr1	32318081	32318389	309	32318331	7	2.9043	2.63038
Nc-1-IP_pe	chr1	32327066	32327368	303	32327122	11	2.96397	2.36091
Nc-1-IP_pe	chr1	32336357	32337458	1102	32336927	17	5.14803	2.94914
Nc-1-IP_pe	chr1	32338018	32338663	646	32338559	17	8.1184	4.33308
Nc-1-IP_pe	chr1	32347778	32348056	279	32348009	10	3.91409	2.96917
Nc-1-IP_pe	chr1	32349609	32350106	498	32349621	11	3.28475	2.53029
Nc-1-IP_pe	chr1	32350611	32350905	295	32350770	13	5.51308	3.52412
Nc-1-IP_pe	chr1	32351877	32352627	751	32352500	14	5.51541	3.4073
Nc-1-IP_pe	chr1	32361274	32362098	825	32361558	19	10.9318	5.43653
Nc-1-IP_pe	chr1	32377871	32378289	419	32378180	11	4.54404	3.2242
Nc-1-IP_pe	chr1	32379403	32379676	274	32379626	9	4.05981	3.16422
Nc-1-IP_pe	chr1	32380306	32380785	480	32380405	12	3.83688	2.74114
Nc-1-IP_pe	chr1	32385547	32386135	589	32386072	12	6.19862	4.04709
Nc-1-IP_pe	chr1	32393975	32394428	454	32394227	16	6.24025	3.54005
Nc-1-IP_pe	chr1	32407345	32408051	707	32407508	15	9.41092	5.38679
Nc-1-IP_pe	chr1	32415596	32415859	264	32415758	12	6.31185	4.11348
Nc-1-IP_pe	chr1	32426893	32427157	265	32427028	13	4.86362	3.18015
Nc-1-IP_pe	chr1	32448775	32449361	587	32449052	11	5.10577	3.5486
Nc-1-IP_pe	chr1	32465246	32465648	403	32465426	14	6.06519	3.69269

Nc-1-IP_pe	chr1	32478009	32478446	438	32478268	9	3.72192	2.95716
Nc-1-IP_pe	chr1	32486413	32486778	366	32486624	13	7.7352	4.78626
Nc-1-IP_pe	chr1	32492334	32492943	610	32492812	12	6.58417	4.27436
Nc-1-IP_pe	chr1	32501773	32502050	278	32501898	14	7.42849	4.43574
Nc-1-IP_pe	chr1	32522080	32522441	362	32522308	9	4.46937	3.41876
Nc-1-IP_pe	chr1	32526804	32527142	339	32527087	9	4.25966	3.28797
Nc-1-IP_pe	chr1	32555076	32555681	606	32555147	10	4.99888	3.61677
Nc-1-IP_pe	chr1	32574798	32575088	291	32574920	10	5.23357	3.76063
Nc-1-IP_pe	chr1	32596194	32596532	339	32596482	9	3.72192	2.95716
Nc-1-IP_pe	chr1	32602043	32602771	729	32602109	10	5.23357	3.76063
Nc-1-IP_pe	chr1	32607080	32607557	478	32607403	9	4.46937	3.41876
Nc-1-IP_pe	chr1	32608831	32609224	394	32608963	18	7.1522	3.73811
Nc-1-IP_pe	chr1	32624748	32625607	860	32624801	9	4.21839	3.26234
Nc-1-IP_pe	chr1	32628175	32628552	378	32628485	12	4.51041	3.09723
Nc-1-IP_pe	chr1	32636078	32636732	655	32636207	11	6.03439	4.10251
Nc-1-IP_pe	chr1	32645990	32646904	915	32646523	11	5.8735	4.00514
Nc-1-IP_pe	chr1	32651281	32651790	510	32651591	18	9.19569	4.72112
Nc-1-IP_pe	chr1	32653829	32654311	483	32653939	11	5.56156	3.81794
Nc-1-IP_pe	chr1	32661996	32662780	785	32662653	13	7.12853	4.42991
Nc-1-IP_pe	chr1	32696137	32696460	324	32696260	11	4.70533	3.31645
Nc-1-IP_pe	chr1	32703136	32703775	640	32703452	16	8.83629	4.87286
Nc-1-IP_pe	chr1	32704212	32704752	541	32704595	10	3.89064	2.95552
Nc-1-IP_pe	chr1	32712555	32712845	291	32712659	16	7.27441	4.05022
Nc-1-IP_pe	chr1	32736620	32737033	414	32736925	11	4.06403	2.95415
Nc-1-IP_pe	chr1	32742150	32742465	316	32742233	15	4.06016	2.62146
Nc-1-IP_pe	chr1	32748169	32748628	460	32748300	17	7.02067	3.79543
Nc-1-IP_pe	chr1	32749596	32750197	602	32749784	15	6.69889	3.8921
Nc-1-IP_pe	chr1	32754681	32755032	352	32754712	11	3.77651	2.79567
Nc-1-IP_pe	chr1	32762706	32762960	255	32762939	13	5.19892	3.35621
Nc-1-IP_pe	chr1	32769837	32770319	483	32769914	16	6.33285	3.58457
Nc-1-IP_pe	chr1	32788582	32788911	330	32788631	10	3.89064	2.95552
Nc-1-IP_pe	chr1	32794834	32795358	525	32795039	10	4.93609	3.57849
Nc-1-IP_pe	chr1	32796475	32796728	254	32796591	12	6.50787	4.22912
Nc-1-IP_pe	chr1	32804389	32805195	807	32804508	16	7.61454	4.22416
Nc-1-IP_pe	chr1	32808113	32808376	264	32808279	10	4.83846	3.51913
Nc-1-IP_pe	chr1	32817230	32818194	965	32817319	22	5.72303	2.82387
Nc-1-IP_pe	chr1	32840579	32841090	512	32841022	10	5.23357	3.76063
Nc-1-IP_pe	chr1	32843476	32843759	284	32843690	10	4.93609	3.57849
Nc-1-IP_pe	chr1	32845109	32845454	346	32845359	12	6.58417	4.27436
Nc-1-IP_pe	chr1	32845759	32846013	255	32845886	10	5.10552	3.682
Nc-1-IP_pe	chr1	32869993	32870340	348	32870133	13	5.36853	3.44651
Nc-1-IP_pe	chr1	32871257	32871940	684	32871379	14	4.21449	2.76599
Nc-1-IP_pe	chr1	32872794	32873242	449	32872934	16	6.33285	3.58457
Nc-1-IP_pe	chr1	32879641	32879992	352	32879922	9	3.23155	2.66199
Nc-1-IP_pe	chr1	32881000	32881497	498	32881125	15	4.40338	2.77622

Nc-1-IP_pe	chr1	32885619	32886181	563	32885744	12	3.83688	2.74114
Nc-1-IP_pe	chr1	32886465	32886879	415	32886631	11	3.64839	2.72584
Nc-1-IP_pe	chr1	32887145	32887630	486	32887473	11	4.61014	3.26192
Nc-1-IP_pe	chr1	32895595	32896105	511	32896047	12	4.53417	3.11004
Nc-1-IP_pe	chr1	32905987	32906726	740	32906405	10	5.01973	3.6295
Nc-1-IP_pe	chr1	32916795	32917385	591	32916972	9	4.46937	3.41876
Nc-1-IP_pe	chr1	32918358	32919043	686	32918506	9	4.46937	3.41876
Nc-1-IP_pe	chr1	32930146	32930426	281	32930258	13	4.86362	3.18015
Nc-1-IP_pe	chr1	32936798	32937111	314	32937033	15	6.78905	3.93887
Nc-1-IP_pe	chr1	32973973	32975699	1727	32974490	19	9.92774	4.92358
Nc-1-IP_pe	chr1	32980232	32980518	287	32980467	9	3.59586	2.88066
Nc-1-IP_pe	chr1	33045052	33045516	465	33045480	11	3.64839	2.72584
Nc-1-IP_pe	chr1	33051763	33052033	271	33051903	13	6.21	3.90651
Nc-1-IP_pe	chr1	33052349	33052748	400	33052625	15	7.75613	4.45384
Nc-1-IP_pe	chr1	33058330	33058734	405	33058454	10	3.89064	2.95552
Nc-1-IP_pe	chr1	33059342	33059670	329	33059600	8	3.74505	3.07688
Nc-1-IP_pe	chr1	33062516	33062934	419	33062610	11	5.7051	3.90381
Nc-1-IP_pe	chr1	33081332	33081862	531	33081438	14	5.98179	3.64885
Nc-1-IP_pe	chr1	33086318	33086744	427	33086418	11	3.64839	2.72584
Nc-1-IP_pe	chr1	33087259	33087542	284	33087388	10	3.4604	2.70797
Nc-1-IP_pe	chr1	33092604	33092939	336	33092763	17	7.02067	3.79543
Nc-1-IP_pe	chr1	33094319	33094683	365	33094567	12	6.12485	4.004
Nc-1-IP_pe	chr1	33097354	33098037	684	33097596	12	5.84943	3.84431
Nc-1-IP_pe	chr1	33100995	33101267	273	33101099	9	4.1889	3.24405
Nc-1-IP_pe	chr1	33119837	33120190	354	33119940	11	5.76039	3.93701
Nc-1-IP_pe	chr1	33123724	33124222	499	33123952	11	5.77901	3.9482
Nc-1-IP_pe	chr1	33133110	33133682	573	33133596	14	6.28469	3.80898
Nc-1-IP_pe	chr1	33135360	33136086	727	33135616	14	7.2429	4.33177
Nc-1-IP_pe	chr1	33148928	33149316	389	33149165	15	7.37761	4.24941
Nc-1-IP_pe	chr1	33152164	33152497	334	33152185	9	2.89249	2.46179
Nc-1-IP_pe	chr1	33152982	33153515	534	33153083	12	5.67619	3.74486
Nc-1-IP_pe	chr1	33158008	33158429	422	33158187	10	4.4323	3.2747
Nc-1-IP_pe	chr1	33162538	33162793	256	33162692	11	4.23076	3.04719
Nc-1-IP_pe	chr1	33165077	33166095	1019	33165573	14	6.61011	3.98382
Nc-1-IP_pe	chr1	33167097	33167585	489	33167452	12	5.50092	3.64505
Nc-1-IP_pe	chr1	33171060	33171486	427	33171124	11	5.24175	3.6284
Nc-1-IP_pe	chr1	33172373	33172662	290	33172499	12	3.86684	2.7567
Nc-1-IP_pe	chr1	33174174	33174712	539	33174274	11	5.42344	3.73577
Nc-1-IP_pe	chr1	33178327	33178584	258	33178535	10	3.4604	2.70797
Nc-1-IP_pe	chr1	33181345	33182058	714	33181562	15	6.5434	3.81197
Nc-1-IP_pe	chr1	33216794	33217126	333	33216952	16	7.53857	4.18505
Nc-1-IP_pe	chr1	33253268	33253528	261	33253390	13	6.22691	3.91595
Nc-1-IP_pe	chr1	33255047	33255986	940	33255586	13	5.36853	3.44651
Nc-1-IP_pe	chr1	33257792	33258060	269	33257858	16	6.65665	3.74206
Nc-1-IP_pe	chr1	33270586	33271178	593	33270964	12	2.87625	2.25568

Nc-1-IP_pe	chr1	33280713	33280965	253	33280786	9	4.27829	3.29955
Nc-1-IP_pe	chr1	33292747	33293335	589	33293029	17	6.47874	3.54137
Nc-1-IP_pe	chr1	33298214	33298496	283	33298452	12	3.77112	2.7071
Nc-1-IP_pe	chr1	33303402	33303735	334	33303563	13	5.36853	3.44651
Nc-1-IP_pe	chr1	33306739	33307152	414	33306804	12	5.56373	3.68072
Nc-1-IP_pe	chr1	33309455	33309722	268	33309689	13	4.67336	3.0817
Nc-1-IP_pe	chr1	33319663	33319955	293	33319800	9	3.27326	2.68684
Nc-1-IP_pe	chr1	33320310	33320750	441	33320616	14	5.69896	3.50164
Nc-1-IP_pe	chr1	33324268	33324696	429	33324648	8	3.56332	2.96115
Nc-1-IP_pe	chr1	33325170	33325436	267	33325322	8	3.51085	2.92785
Nc-1-IP_pe	chr1	33340360	33340672	313	33340521	11	5.10577	3.5486
Nc-1-IP_pe	chr1	33350244	33350597	354	33350567	11	3.64839	2.72584
Nc-1-IP_pe	chr1	33354342	33354782	441	33354439	12	5.54793	3.67174
Nc-1-IP_pe	chr1	33361238	33361618	381	33361380	12	5.77527	3.80164
Nc-1-IP_pe	chr1	33366953	33367243	291	33367193	11	4.35236	3.11555
Nc-1-IP_pe	chr1	33367648	33368516	869	33367716	13	5.36853	3.44651
Nc-1-IP_pe	chr1	33369864	33370140	277	33369993	13	5.42865	3.47872
Nc-1-IP_pe	chr1	33386491	33386994	504	33386891	9	4.46937	3.41876
Nc-1-IP_pe	chr1	33430376	33430939	564	33430713	20	8.56319	4.1316
Nc-1-IP_pe	chr1	33454742	33455116	375	33454937	16	6.33285	3.58457
Nc-1-IP_pe	chr1	33466680	33467049	370	33466875	13	6.54342	4.09407
Nc-1-IP_pe	chr1	33488626	33489435	810	33489422	15	4.7819	2.95039
Nc-1-IP_pe	chr1	33499865	33500335	471	33500016	10	4.33501	3.21677
Nc-1-IP_pe	chr1	33508734	33510169	1436	33510029	17	9.65211	5.13407
Nc-1-IP_pe	chr1	33519301	33519560	260	33519521	11	4.54404	3.2242
Nc-1-IP_pe	chr1	33524825	33525171	347	33524958	13	4.86362	3.18015
Nc-1-IP_pe	chr1	33536524	33536879	356	33536670	12	6.50787	4.22912
Nc-1-IP_pe	chr1	33540991	33541585	595	33541286	9	4.46937	3.41876
Nc-1-IP_pe	chr1	33546046	33546472	427	33546219	11	4.06403	2.95415
Nc-1-IP_pe	chr1	33575344	33575659	316	33575464	10	5.23357	3.76063
Nc-1-IP_pe	chr1	33610890	33611164	275	33610971	8	3.5501	2.95276
Nc-1-IP_pe	chr1	33618904	33619379	476	33619225	9	3.27326	2.68684
Nc-1-IP_pe	chr1	33625023	33625501	479	33625347	13	5.15976	3.33548
Nc-1-IP_pe	chr1	33633287	33633754	468	33633595	19	10.9318	5.43653
Nc-1-IP_pe	chr1	33634364	33634988	625	33634787	13	5.29187	3.40559
Nc-1-IP_pe	chr1	33635727	33636380	654	33635847	12	4.63088	3.16234
Nc-1-IP_pe	chr1	33646622	33647011	390	33646927	9	3.54889	2.85226
Nc-1-IP_pe	chr1	33652210	33652515	306	33652437	11	5.3232	3.67643
Nc-1-IP_pe	chr1	33662838	33663092	255	33662949	11	3.64839	2.72584
Nc-1-IP_pe	chr1	33690117	33690416	300	33690216	11	5.10577	3.5486
Nc-1-IP_pe	chr1	33698667	33698938	272	33698709	8	3.74505	3.07688
Nc-1-IP_pe	chr1	33706720	33707204	485	33707031	9	3.72192	2.95716
Nc-1-IP_pe	chr1	33711722	33712404	683	33712062	11	6.03439	4.10251
Nc-1-IP_pe	chr1	33714710	33714998	289	33714908	9	4.3087	3.31847
Nc-1-IP_pe	chr1	33725082	33725406	325	33725201	12	6.86906	4.44438

Nc-1-IP_pe	chr1	33726300	33726612	313	33726428	16	9.11744	5.02718
Nc-1-IP_pe	chr1	33753202	33753455	254	33753296	9	4.46937	3.41876
Nc-1-IP_pe	chr1	33773685	33773975	291	33773692	10	3.08907	2.49869
Nc-1-IP_pe	chr1	33774564	33774877	314	33774731	14	8.29543	4.93196
Nc-1-IP_pe	chr1	33819517	33819796	280	33819753	11	4.47871	3.18705
Nc-1-IP_pe	chr1	33826486	33826778	293	33826614	11	5.10577	3.5486
Nc-1-IP_pe	chr1	33846975	33847259	285	33847044	10	4.99888	3.61677
Nc-1-IP_pe	chr1	33893847	33894426	580	33893934	12	6.86906	4.44438
Nc-1-IP_pe	chr1	33908578	33909061	484	33908731	11	6.02981	4.09973
Nc-1-IP_pe	chr1	33911831	33912113	283	33912096	8	3.74505	3.07688
Nc-1-IP_pe	chr1	33932354	33932631	278	33932543	14	8.63073	5.12814
Nc-1-IP_pe	chr1	33938934	33939356	423	33939048	8	3.74505	3.07688
Nc-1-IP_pe	chr1	33947061	33947336	276	33947151	11	5.99012	4.07567
Nc-1-IP_pe	chr1	33964742	33965250	509	33964838	8	3.08755	2.66145
Nc-1-IP_pe	chr1	33971403	33971701	299	33971618	12	6.12485	4.004
Nc-1-IP_pe	chr1	33997181	33997541	361	33997292	12	4.70082	3.20033
Nc-1-IP_pe	chr1	34014222	34014500	279	34014362	10	4.39571	3.25288
Nc-1-IP_pe	chr1	34029622	34029942	321	34029759	11	5.10577	3.5486
Nc-1-IP_pe	chr1	34031062	34031346	285	34031174	10	3.89064	2.95552
Nc-1-IP_pe	chr1	34072658	34072968	311	34072671	9	3.27326	2.68684
Nc-1-IP_pe	chr1	34092812	34093250	439	34093037	13	7.7352	4.78626
Nc-1-IP_pe	chr1	34153154	34153503	350	34153203	9	4.46937	3.41876
Nc-1-IP_pe	chr1	34177094	34177385	292	34177290	10	5.23357	3.76063
Nc-1-IP_pe	chr1	34179076	34179511	436	34179374	13	7.56612	4.68618
Nc-1-IP_pe	chr1	34189715	34190122	408	34189897	14	6.06519	3.69269
Nc-1-IP_pe	chr1	34200518	34201019	502	34200883	13	5.9487	3.76157
Nc-1-IP_pe	chr1	34210623	34210884	262	34210747	9	4.25966	3.28797
Nc-1-IP_pe	chr1	34212321	34212844	524	34212394	12	6.58417	4.27436
Nc-1-IP_pe	chr1	34250868	34251229	362	34251180	10	3.89064	2.95552
Nc-1-IP_pe	chr1	34266182	34266725	544	34266653	10	5.10552	3.682
Nc-1-IP_pe	chr1	34328407	34328839	433	34328513	9	4.46937	3.41876
Nc-1-IP_pe	chr1	34347723	34347977	255	34347901	10	5.23357	3.76063
Nc-1-IP_pe	chr1	34368916	34369515	600	34369331	9	4.46937	3.41876
Nc-1-IP_pe	chr1	34426002	34426476	475	34426055	8	3.51085	2.92785
Nc-1-IP_pe	chr1	34449016	34449278	263	34449140	11	5.8735	4.00514
Nc-1-IP_pe	chr1	34452450	34452897	448	34452560	9	3.72192	2.95716
Nc-1-IP_pe	chr1	34459941	34460495	555	34460464	9	3.72192	2.95716
Nc-1-IP_pe	chr1	34461377	34461634	258	34461502	8	3.38431	2.8478
Nc-1-IP_pe	chr1	34470318	34470578	261	34470460	8	3.74505	3.07688
Nc-1-IP_pe	chr1	34597630	34598087	458	34597832	13	4.02562	2.7544
Nc-1-IP_pe	chr1	34659411	34659770	360	34659516	12	4.70082	3.20033
Nc-1-IP_pe	chr1	34683637	34683901	265	34683765	14	8.63073	5.12814
Nc-1-IP_pe	chr1	34688087	34688448	362	34688255	8	3.48502	2.91148
Nc-1-IP_pe	chr1	34698000	34698307	308	34698184	18	8.62313	4.4354
Nc-1-IP_pe	chr1	34706060	34706372	313	34706257	10	5.23357	3.76063

Nc-1-IP_pe	chr1	34713553	34713869	317	34713705	12	5.84943	3.84431
Nc-1-IP_pe	chr1	34730870	34731317	448	34731174	13	6.92473	4.312
Nc-1-IP_pe	chr1	34738755	34739205	451	34739158	13	3.67377	2.58159
Nc-1-IP_pe	chr1	34741504	34741815	312	34741701	13	4.02562	2.7544
Nc-1-IP_pe	chr1	34747875	34748254	380	34748002	11	4.62357	3.2696
Nc-1-IP_pe	chr1	34761349	34762098	750	34761496	19	6.78661	3.47027
Nc-1-IP_pe	chr1	34770840	34771125	286	34770868	9	3.26261	2.68049
Nc-1-IP_pe	chr1	34776828	34777103	276	34777028	11	4.81752	3.38105
Nc-1-IP_pe	chr1	34779713	34780008	296	34779752	11	3.28475	2.53029
Nc-1-IP_pe	chr1	34781558	34782237	680	34782087	17	6.78076	3.68203
Nc-1-IP_pe	chr1	34785021	34785456	436	34785218	12	4.57012	3.12944
Nc-1-IP_pe	chr1	34794625	34794925	301	34794754	12	4.39405	3.03475
Nc-1-IP_pe	chr1	34808229	34808516	288	34808420	14	4.21449	2.76599
Nc-1-IP_pe	chr1	34825196	34825561	366	34825434	16	5.98198	3.41707
Nc-1-IP_pe	chr1	34844004	34844427	424	34844261	13	4.90859	3.20357
Nc-1-IP_pe	chr1	34850722	34851002	281	34850932	12	6.18005	4.03623
Nc-1-IP_pe	chr1	34852984	34853254	271	34853146	11	4.54404	3.2242
Nc-1-IP_pe	chr1	34855451	34855753	303	34855481	10	3.4604	2.70797
Nc-1-IP_pe	chr1	34859353	34859902	550	34859480	20	7.97282	3.87239
Nc-1-IP_pe	chr1	34865060	34865578	519	34865190	10	4.71266	3.44298
Nc-1-IP_pe	chr1	34868204	34868709	506	34868229	10	4.25398	3.16873
Nc-1-IP_pe	chr1	34871324	34871582	259	34871525	12	6.12485	4.004
Nc-1-IP_pe	chr1	34874927	34875206	280	34875130	11	6.03439	4.10251
Nc-1-IP_pe	chr1	34899062	34900073	1012	34899356	12	5.23082	3.49289
Nc-1-IP_pe	chr1	34900569	34900920	352	34900719	9	3.12089	2.59629
Nc-1-IP_pe	chr1	34904382	34904642	261	34904481	13	5.36853	3.44651
Nc-1-IP_pe	chr1	34911469	34911744	276	34911563	9	4.35492	3.34727
Nc-1-IP_pe	chr1	34928207	34928548	342	34928379	10	5.23357	3.76063
Nc-1-IP_pe	chr1	34929660	34930356	697	34930282	11	2.96397	2.36091
Nc-1-IP_pe	chr1	34948589	34948982	394	34948836	7	3.06442	2.73501
Nc-1-IP_pe	chr1	34967643	34968039	397	34967816	13	4.86362	3.18015
Nc-1-IP_pe	chr1	34976450	34977769	1320	34977401	13	4.02562	2.7544
Nc-1-IP_pe	chr1	34982073	34983249	1177	34982229	10	3.08907	2.49869
Nc-1-IP_pe	chr1	34984874	34985413	540	34985080	14	5.03085	3.16286
Nc-1-IP_pe	chr1	35004441	35005035	595	35004560	10	5.23357	3.76063
Nc-1-IP_pe	chr1	35034534	35034864	331	35034661	8	3.74505	3.07688
Nc-1-IP_pe	chr1	35060330	35060641	312	35060562	8	3.64414	3.01254
Nc-1-IP_pe	chr1	35068856	35069140	285	35069025	13	7.7352	4.78626
Nc-1-IP_pe	chr1	35105275	35105532	258	35105348	11	5.3563	3.696
Nc-1-IP_pe	chr1	35108550	35109044	495	35108836	10	3.50482	2.73328
Nc-1-IP_pe	chr1	35123774	35124557	784	35123918	11	5.76039	3.93701
Nc-1-IP_pe	chr1	35149012	35149391	380	35149294	12	6.31185	4.11348
Nc-1-IP_pe	chr1	35163953	35164262	310	35163994	8	3.74505	3.07688
Nc-1-IP_pe	chr1	35188696	35188956	261	35188820	14	8.15847	4.85247
Nc-1-IP_pe	chr1	35189808	35190092	285	35189951	8	3.52386	2.93611

Nc-1-IP_pe	chr1	35192071	35192994	924	35192791	17	4.44438	2.65333
Nc-1-IP_pe	chr1	35210396	35210850	455	35210591	12	5.77527	3.80164
Nc-1-IP_pe	chr1	35211428	35211730	303	35211609	14	8.63073	5.12814
Nc-1-IP_pe	chr1	35216379	35216764	386	35216440	9	4.1889	3.24405
Nc-1-IP_pe	chr1	35234495	35234771	277	35234630	13	5.9487	3.76157
Nc-1-IP_pe	chr1	35246313	35246697	385	35246530	11	6.03439	4.10251
Nc-1-IP_pe	chr1	35248115	35248732	618	35248613	11	5.10577	3.5486
Nc-1-IP_pe	chr1	35344082	35344336	255	35344195	12	6.86906	4.44438
Nc-1-IP_pe	chr1	35407173	35407426	254	35407297	9	4.46937	3.41876
Nc-1-IP_pe	chr1	35429660	35430184	525	35430116	11	5.47462	3.76617
Nc-1-IP_pe	chr1	35437061	35437499	439	35437063	13	3.95529	2.71958
Nc-1-IP_pe	chr1	35494621	35495222	602	35494669	10	5.10552	3.682
Nc-1-IP_pe	chr1	35541310	35541562	253	35541375	11	5.65062	3.87116
Nc-1-IP_pe	chr1	35564290	35564693	404	35564353	12	5.75858	3.79206
Nc-1-IP_pe	chr1	35565568	35566331	764	35565956	11	3.64839	2.72584
Nc-1-IP_pe	chr1	35573516	35574160	645	35573555	16	5.37454	3.13479
Nc-1-IP_pe	chr1	35574445	35574702	258	35574517	14	4.60011	2.95114
Nc-1-IP_pe	chr1	35576020	35576386	367	35576087	10	3.44377	2.69851
Nc-1-IP_pe	chr1	35577002	35577303	302	35577267	13	4.41954	2.952
Nc-1-IP_pe	chr1	35587826	35588830	1005	35588693	16	8.17084	4.51499
Nc-1-IP_pe	chr1	35589623	35590091	469	35589928	12	5.54793	3.67174
Nc-1-IP_pe	chr1	35610243	35610780	538	35610671	13	7.7352	4.78626
Nc-1-IP_pe	chr1	35641256	35641581	326	35641380	13	5.29187	3.40559
Nc-1-IP_pe	chr1	35660442	35660937	496	35660568	9	3.27326	2.68684
Nc-1-IP_pe	chr1	35666862	35667199	338	35667159	9	4.20361	3.25317
Nc-1-IP_pe	chr1	35685856	35686190	335	35686136	8	3.4091	2.86346
Nc-1-IP_pe	chr1	35688057	35688364	308	35688120	13	6.92473	4.312
Nc-1-IP_pe	chr1	35691045	35691431	387	35691235	11	5.54401	3.80747
Nc-1-IP_pe	chr1	35699617	35700131	515	35699712	9	4.25966	3.28797
Nc-1-IP_pe	chr1	35713336	35713638	303	35713484	11	4.86057	3.40592
Nc-1-IP_pe	chr1	35725319	35725737	419	35725488	12	6.25484	4.08002
Nc-1-IP_pe	chr1	35769320	35769575	256	35769473	15	6.78905	3.93887
Nc-1-IP_pe	chr1	35780135	35780599	465	35780217	9	4.40188	3.37657
Nc-1-IP_pe	chr1	35807826	35808168	343	35807872	10	3.94999	2.99009
Nc-1-IP_pe	chr1	35834480	35834906	427	35834817	9	4.46937	3.41876
Nc-1-IP_pe	chr1	35879154	35879597	444	35879208	10	4.47495	3.30017
Nc-1-IP_pe	chr1	35882735	35883037	303	35882965	18	7.1522	3.73811
Nc-1-IP_pe	chr1	35883400	35883985	586	35883747	14	4.62572	2.96359
Nc-1-IP_pe	chr1	35889769	35890070	302	35889828	8	3.21751	2.7428
Nc-1-IP_pe	chr1	35893516	35893871	356	35893814	11	3.64839	2.72584
Nc-1-IP_pe	chr1	35901969	35902363	395	35902050	14	7.42849	4.43574
Nc-1-IP_pe	chr1	35930880	35931530	651	35931060	20	7.97282	3.87239
Nc-1-IP_pe	chr1	35944733	35945004	272	35944750	9	3.27326	2.68684
Nc-1-IP_pe	chr1	35987000	35987313	314	35987050	11	5.77463	3.94557
Nc-1-IP_pe	chr1	36027478	36027741	264	36027598	9	4.38615	3.36674

Nc-1-IP_pe	chr1	36045340	36045642	303	36045395	8	3.74505	3.07688
Nc-1-IP_pe	chr1	36046086	36046411	326	36046103	9	3.72192	2.95716
Nc-1-IP_pe	chr1	36054358	36054861	504	36054659	8	3.08755	2.66145
Nc-1-IP_pe	chr1	36084010	36084441	432	36084121	13	4.90859	3.20357
Nc-1-IP_pe	chr1	36085832	36086422	591	36085895	13	5.17277	3.34236
Nc-1-IP_pe	chr1	36093273	36094157	885	36094089	14	5.51541	3.4073
Nc-1-IP_pe	chr1	36096700	36096972	273	36096857	14	5.51541	3.4073
Nc-1-IP_pe	chr1	36097353	36097906	554	36097809	10	3.08907	2.49869
Nc-1-IP_pe	chr1	36098204	36100576	2373	36100208	13	4.41954	2.952
Nc-1-IP_pe	chr1	36114185	36114683	499	36114355	12	5.01934	3.37518
Nc-1-IP_pe	chr1	36116227	36117007	781	36116279	11	4.00079	2.91908
Nc-1-IP_pe	chr1	36125967	36126241	275	36126131	19	8.4638	4.21715
Nc-1-IP_pe	chr1	36127943	36128565	623	36128406	12	6.07041	3.97229
Nc-1-IP_pe	chr1	36130612	36131414	803	36130987	11	5.10577	3.5486
Nc-1-IP_pe	chr1	36132361	36132735	375	36132431	9	2.89249	2.46179
Nc-1-IP_pe	chr1	36134714	36135129	416	36134847	13	7.42513	4.60316
Nc-1-IP_pe	chr1	36149198	36149728	531	36149340	18	7.73136	4.00629
Nc-1-IP_pe	chr1	36150486	36150743	258	36150667	13	4.14549	2.81406
Nc-1-IP_pe	chr1	36153019	36153445	427	36153429	13	4.98219	3.24203
Nc-1-IP_pe	chr1	36155602	36156638	1037	36156405	15	6.69889	3.8921
Nc-1-IP_pe	chr1	36159538	36159947	410	36159810	15	5.66913	3.37372
Nc-1-IP_pe	chr1	36170926	36171605	680	36171163	16	8.49334	4.68711
Nc-1-IP_pe	chr1	36172261	36172656	396	36172486	16	8.57707	4.73221
Nc-1-IP_pe	chr1	36174808	36175078	271	36174934	11	4.78913	3.36466
Nc-1-IP_pe	chr1	36175395	36175708	314	36175471	12	5.23082	3.49289
Nc-1-IP_pe	chr1	36176590	36177182	593	36176718	14	6.62702	3.99298
Nc-1-IP_pe	chr1	36177875	36178621	747	36178272	12	4.70082	3.20033
Nc-1-IP_pe	chr1	36194014	36194517	504	36194467	9	4.05981	3.16422
Nc-1-IP_pe	chr1	36196895	36197238	344	36197056	12	4.51041	3.09723
Nc-1-IP_pe	chr1	36200450	36200966	517	36200517	12	5.15877	3.45264
Nc-1-IP_pe	chr1	36204186	36204440	255	36204234	12	5.01934	3.37518
Nc-1-IP_pe	chr1	36208167	36208510	344	36208335	11	5.89269	4.01672
Nc-1-IP_pe	chr1	36211391	36211850	460	36211780	10	3.4604	2.70797
Nc-1-IP_pe	chr1	36212629	36213119	491	36212885	12	4.70082	3.20033
Nc-1-IP_pe	chr1	36214946	36215218	273	36215078	20	10.7672	5.16976
Nc-1-IP_pe	chr1	36215813	36216154	342	36215942	9	4.46937	3.41876
Nc-1-IP_pe	chr1	36221713	36222072	360	36221902	12	5.8598	3.85029
Nc-1-IP_pe	chr1	36227882	36228503	622	36227978	10	4.72812	3.45232
Nc-1-IP_pe	chr1	36238900	36239216	317	36239184	8	3.38431	2.8478
Nc-1-IP_pe	chr1	36252271	36252578	308	36252538	9	3.72192	2.95716
Nc-1-IP_pe	chr1	36257804	36258175	372	36257952	11	5.19376	3.60018
Nc-1-IP_pe	chr1	36260390	36260651	262	36260531	10	4.99888	3.61677
Nc-1-IP_pe	chr1	36271238	36271835	598	36271322	11	5.77463	3.94557
Nc-1-IP_pe	chr1	36306421	36306772	352	36306635	19	9.03708	4.48779
Nc-1-IP_pe	chr1	36308133	36308726	594	36308134	11	3.28475	2.53029

Nc-1-IP_pe	chr1	36319237	36319511	275	36319356	10	3.08907	2.49869
Nc-1-IP_pe	chr1	36320697	36321211	515	36321099	13	5.77769	3.66773
Nc-1-IP_pe	chr1	36321666	36322777	1112	36322619	14	4.23163	2.77414
Nc-1-IP_pe	chr1	36327708	36328001	294	36327717	15	5.00915	3.05678
Nc-1-IP_pe	chr1	36341395	36341724	330	36341503	11	3.89191	2.85898
Nc-1-IP_pe	chr1	36342068	36342594	527	36342451	11	3.64839	2.72584
Nc-1-IP_pe	chr1	36349591	36350460	870	36349978	14	5.03085	3.16286
Nc-1-IP_pe	chr1	36355040	36355343	304	36355166	16	6.89451	3.85952
Nc-1-IP_pe	chr1	36358087	36358342	256	36358181	13	5.42865	3.47872
Nc-1-IP_pe	chr1	36374882	36375282	401	36375031	17	9.53612	5.07157
Nc-1-IP_pe	chr1	36383345	36383598	254	36383476	18	9.58905	4.92191
Nc-1-IP_pe	chr1	36400802	36401201	400	36400804	13	3.07238	2.29378
Nc-1-IP_pe	chr1	36401804	36402415	612	36402165	15	8.26005	4.73146
Nc-1-IP_pe	chr1	36411408	36411700	293	36411559	12	4.91154	3.31567
Nc-1-IP_pe	chr1	36413892	36414176	285	36413971	17	7.02067	3.79543
Nc-1-IP_pe	chr1	36424129	36424435	307	36424208	9	4.23325	3.27156
Nc-1-IP_pe	chr1	36426811	36427084	274	36426920	10	4.62142	3.388
Nc-1-IP_pe	chr1	36430121	36430463	343	36430342	14	5.03085	3.16286
Nc-1-IP_pe	chr1	36437592	36437891	300	36437749	11	4.06403	2.95415
Nc-1-IP_pe	chr1	36441000	36441528	529	36441113	10	4.99888	3.61677
Nc-1-IP_pe	chr1	36451155	36451431	277	36451395	9	3.72947	2.96176
Nc-1-IP_pe	chr1	36461918	36462426	509	36462083	11	4.54404	3.2242
Nc-1-IP_pe	chr1	36463735	36464386	652	36464324	17	5.54967	3.12324
Nc-1-IP_pe	chr1	36466832	36467326	495	36467180	16	7.83	4.33588
Nc-1-IP_pe	chr1	36467702	36468300	599	36467962	16	5.82808	3.34463
Nc-1-IP_pe	chr1	36475206	36475586	381	36475460	10	3.54629	2.75696
Nc-1-IP_pe	chr1	36481309	36482935	1627	36482603	13	5.49888	3.51647
Nc-1-IP_pe	chr1	36485296	36485734	439	36485457	12	3.47963	2.55766
Nc-1-IP_pe	chr1	36492736	36493325	590	36493285	11	5.10577	3.5486
Nc-1-IP_pe	chr1	36505708	36506111	404	36505875	8	3.74505	3.07688
Nc-1-IP_pe	chr1	36509356	36509780	425	36509594	14	6.78215	4.0774
Nc-1-IP_pe	chr1	36515165	36515676	512	36515559	14	6.90659	4.14556
Nc-1-IP_pe	chr1	36520028	36520379	352	36520207	11	4.76096	3.34844
Nc-1-IP_pe	chr1	36529052	36529652	601	36529275	10	5.23357	3.76063
Nc-1-IP_pe	chr1	36530199	36530814	616	36530312	12	6.31185	4.11348
Nc-1-IP_pe	chr1	36542483	36543045	563	36542761	14	5.51541	3.4073
Nc-1-IP_pe	chr1	36553857	36554271	415	36553905	11	3.40056	2.59215
Nc-1-IP_pe	chr1	36558465	36558785	321	36558728	10	4.50371	3.31737
Nc-1-IP_pe	chr1	36565957	36566227	271	36566103	11	2.96397	2.36091
Nc-1-IP_pe	chr1	36568060	36568346	287	36568106	12	5.15877	3.45264
Nc-1-IP_pe	chr1	36572856	36573265	410	36573177	11	5.10577	3.5486
Nc-1-IP_pe	chr1	36576005	36576628	624	36576326	20	9.94634	4.77022
Nc-1-IP_pe	chr1	36581898	36582171	274	36581966	11	5.5792	3.82847
Nc-1-IP_pe	chr1	36583471	36583733	263	36583519	11	5.76039	3.93701
Nc-1-IP_pe	chr1	36584055	36584558	504	36584362	11	5.91198	4.02837

Nc-1-IP_pe	chr1	36585558	36585810	253	36585668	12	6.48784	4.21727
Nc-1-IP_pe	chr1	36590957	36591534	578	36591436	12	6.05242	3.96182
Nc-1-IP_pe	chr1	36596363	36596646	284	36596613	10	4.99888	3.61677
Nc-1-IP_pe	chr1	36599010	36599267	258	36599165	14	5.03085	3.16286
Nc-1-IP_pe	chr1	36606290	36606966	677	36606445	12	3.47963	2.55766
Nc-1-IP_pe	chr1	36623364	36624336	973	36623442	11	5.77463	3.94557
Nc-1-IP_pe	chr1	36630777	36631100	324	36630845	8	3.74505	3.07688
Nc-1-IP_pe	chr1	36635892	36636242	351	36636044	12	6.7561	4.37677
Nc-1-IP_pe	chr1	36650059	36650532	474	36650125	10	4.33501	3.21677
Nc-1-IP_pe	chr1	36652817	36653389	573	36653282	11	4.54404	3.2242
Nc-1-IP_pe	chr1	36656205	36656605	401	36656396	11	4.54404	3.2242
Nc-1-IP_pe	chr1	36658715	36659012	298	36658767	11	5.52654	3.79706
Nc-1-IP_pe	chr1	36664076	36664806	731	36664155	16	9.6187	5.30662
Nc-1-IP_pe	chr1	36677710	36678059	350	36677848	12	6.86906	4.44438
Nc-1-IP_pe	chr1	36686763	36687197	435	36687074	10	3.89064	2.95552
Nc-1-IP_pe	chr1	36694379	36694787	409	36694613	13	6.72991	4.20021
Nc-1-IP_pe	chr1	36696266	36696558	293	36696317	9	3.27326	2.68684
Nc-1-IP_pe	chr1	36715193	36715631	439	36715420	9	4.46937	3.41876
Nc-1-IP_pe	chr1	36719509	36719794	286	36719717	10	4.39571	3.25288
Nc-1-IP_pe	chr1	36722005	36722348	344	36722124	13	6.62436	4.14003
Nc-1-IP_pe	chr1	36729007	36729297	291	36729105	12	5.23082	3.49289
Nc-1-IP_pe	chr1	36729576	36729888	313	36729660	9	3.9633	3.10479
Nc-1-IP_pe	chr1	36733619	36733978	360	36733930	11	4.35236	3.11555
Nc-1-IP_pe	chr1	36735441	36735748	308	36735554	10	4.71266	3.44298
Nc-1-IP_pe	chr1	36738571	36738862	292	36738630	10	4.62142	3.388
Nc-1-IP_pe	chr1	36752617	36752966	350	36752716	8	3.264	2.772
Nc-1-IP_pe	chr1	36756445	36756701	257	36756488	11	5.44042	3.74585
Nc-1-IP_pe	chr1	36770721	36771053	333	36770951	10	4.39571	3.25288
Nc-1-IP_pe	chr1	36774804	36775193	390	36774965	11	5.10577	3.5486
Nc-1-IP_pe	chr1	36801505	36801911	407	36801911	10	3.08907	2.49869
Nc-1-IP_pe	chr1	36807031	36807577	547	36807135	14	4.60011	2.95114
Nc-1-IP_pe	chr1	36813860	36814285	426	36814128	10	5.23357	3.76063
Nc-1-IP_pe	chr1	36815960	36816254	295	36816141	10	5.23357	3.76063
Nc-1-IP_pe	chr1	36816769	36817240	472	36817179	11	4.06403	2.95415
Nc-1-IP_pe	chr1	36821437	36821800	364	36821547	11	5.7051	3.90381
Nc-1-IP_pe	chr1	36849222	36849618	397	36849568	9	3.27326	2.68684
Nc-1-IP_pe	chr1	36879588	36879967	380	36879891	9	3.27326	2.68684
Nc-1-IP_pe	chr1	36905948	36906231	284	36906092	10	5.23357	3.76063
Nc-1-IP_pe	chr1	36919682	36920051	370	36919832	14	8.29543	4.93196
Nc-1-IP_pe	chr1	36920381	36920659	279	36920531	9	4.46937	3.41876
Nc-1-IP_pe	chr1	36922435	36922852	418	36922544	11	4.35236	3.11555
Nc-1-IP_pe	chr1	36942210	36943067	858	36942383	10	4.39571	3.25288
Nc-1-IP_pe	chr1	36946012	36946359	348	36946187	13	7.72974	4.78302
Nc-1-IP_pe	chr1	36948217	36948756	540	36948339	11	4.06403	2.95415
Nc-1-IP_pe	chr1	36954861	36955299	439	36955114	11	5.20969	3.60954

Nc-1-IP_pe	chr1	36961952	36962228	277	36962140	11	4.54404	3.2242
Nc-1-IP_pe	chr1	36962772	36963134	363	36963036	10	4.77495	3.48064
Nc-1-IP_pe	chr1	36963708	36964403	696	36964166	11	5.95085	4.05188
Nc-1-IP_pe	chr1	36972817	36973212	396	36972901	11	5.03835	3.5092
Nc-1-IP_pe	chr1	36984354	36984784	431	36984624	13	6.38222	4.00303
Nc-1-IP_pe	chr1	36990029	36990351	323	36990094	14	7.30138	4.36445
Nc-1-IP_pe	chr1	36996998	36997616	619	36997192	9	4.21839	3.26234
Nc-1-IP_pe	chr1	37007512	37007896	385	37007888	9	3.27326	2.68684
Nc-1-IP_pe	chr1	37009498	37010152	655	37009979	13	6.62436	4.14003
Nc-1-IP_pe	chr1	37026039	37026387	349	37026158	8	3.08755	2.66145
Nc-1-IP_pe	chr1	37033203	37033527	325	37033435	12	5.23082	3.49289
Nc-1-IP_pe	chr1	37034611	37035325	715	37034848	12	6.33104	4.12476
Nc-1-IP_pe	chr1	37051805	37052191	387	37051934	10	4.99888	3.61677
Nc-1-IP_pe	chr1	37052886	37053367	482	37053164	14	8.29543	4.93196
Nc-1-IP_pe	chr1	37056367	37056736	370	37056531	19	9.92774	4.92358
Nc-1-IP_pe	chr1	37062318	37062616	299	37062492	9	4.46937	3.41876
Nc-1-IP_pe	chr1	37064389	37064677	289	37064547	11	5.77463	3.94557
Nc-1-IP_pe	chr1	37076049	37076392	344	37076209	10	4.59158	3.37006
Nc-1-IP_pe	chr1	37079945	37080655	711	37080302	12	5.45448	3.61875
Nc-1-IP_pe	chr1	37081162	37081705	544	37081591	9	3.72192	2.95716
Nc-1-IP_pe	chr1	37094557	37094963	407	37094895	9	2.89249	2.46179
Nc-1-IP_pe	chr1	37095642	37096453	812	37096224	12	4.70082	3.20033
Nc-1-IP_pe	chr1	37098275	37098622	348	37098361	10	5.23357	3.76063
Nc-1-IP_pe	chr1	37099345	37099812	468	37099633	13	7.7352	4.78626
Nc-1-IP_pe	chr1	37106066	37106387	322	37106207	12	4.70082	3.20033
Nc-1-IP_pe	chr1	37117606	37118116	511	37117828	8	3.08755	2.66145
Nc-1-IP_pe	chr1	37120406	37120665	260	37120492	10	5.23357	3.76063
Nc-1-IP_pe	chr1	37169800	37170155	356	37170014	13	5.36853	3.44651
Nc-1-IP_pe	chr1	37239043	37239427	385	37239338	12	5.23082	3.49289
Nc-1-IP_pe	chr1	37244322	37244680	359	37244334	9	3.27326	2.68684
Nc-1-IP_pe	chr1	37259805	37260173	369	37259878	9	4.46937	3.41876
Nc-1-IP_pe	chr1	37264054	37264594	541	37264170	9	4.3705	3.35698
Nc-1-IP_pe	chr1	37272382	37272750	369	37272430	11	5.76039	3.93701
Nc-1-IP_pe	chr1	37273552	37274356	805	37273883	16	8.79222	4.84884
Nc-1-IP_pe	chr1	37286605	37286966	362	37286869	12	4.70082	3.20033
Nc-1-IP_pe	chr1	37289424	37289803	380	37289609	12	5.23082	3.49289
Nc-1-IP_pe	chr1	37301923	37302244	322	37302020	10	4.41822	3.2663
Nc-1-IP_pe	chr1	37327536	37328206	671	37328136	11	6.03439	4.10251
Nc-1-IP_pe	chr1	37334306	37334597	292	37334536	9	4.35492	3.34727
Nc-1-IP_pe	chr1	37336194	37336454	261	37336408	9	2.89249	2.46179
Nc-1-IP_pe	chr1	37344171	37344486	316	37344238	10	4.39571	3.25288
Nc-1-IP_pe	chr1	37373961	37374419	459	37374068	10	4.39571	3.25288
Nc-1-IP_pe	chr1	37418547	37418967	421	37418552	9	2.89249	2.46179
Nc-1-IP_pe	chr1	37453339	37453727	389	37453564	14	4.8399	3.06836
Nc-1-IP_pe	chr1	37454766	37455422	657	37454828	11	3.64839	2.72584

Nc-1-IP_pe	chr1	37466392	37466830	439	37466720	9	4.46937	3.41876
Nc-1-IP_pe	chr1	37467111	37467648	538	37467391	13	6.62436	4.14003
Nc-1-IP_pe	chr1	37474695	37475796	1102	37475028	16	5.37454	3.13479
Nc-1-IP_pe	chr1	37476626	37476964	339	37476672	13	3.67377	2.58159
Nc-1-IP_pe	chr1	37480859	37481190	332	37481076	16	5.50606	3.19508
Nc-1-IP_pe	chr1	37482193	37483117	925	37482888	17	7.50977	4.03119
Nc-1-IP_pe	chr1	37484126	37484546	421	37484355	14	5.74019	3.52296
Nc-1-IP_pe	chr1	37486024	37486786	763	37486066	13	5.36853	3.44651
Nc-1-IP_pe	chr1	37487075	37487572	498	37487149	12	4.28158	2.97474
Nc-1-IP_pe	chr1	37502786	37503101	316	37502977	12	5.23082	3.49289
Nc-1-IP_pe	chr1	37506710	37507209	500	37507056	11	5.10577	3.5486
Nc-1-IP_pe	chr1	37518098	37518771	674	37518495	11	5.27411	3.64746
Nc-1-IP_pe	chr1	37530024	37530450	427	37530089	11	4.61014	3.26192
Nc-1-IP_pe	chr1	37532960	37533220	261	37533065	10	5.03671	3.63988
Nc-1-IP_pe	chr1	37553942	37554232	291	37554209	17	5.54967	3.12324
Nc-1-IP_pe	chr1	37557435	37557801	367	37557654	15	7.16374	4.13551
Nc-1-IP_pe	chr1	37562548	37562909	362	37562686	10	4.20113	3.13749
Nc-1-IP_pe	chr1	37606263	37606618	356	37606518	9	3.89634	3.06369
Nc-1-IP_pe	chr1	37612769	37613279	511	37613226	10	4.33501	3.21677
Nc-1-IP_pe	chr1	37616125	37616643	519	37616504	13	6.14303	3.86919
Nc-1-IP_pe	chr1	37625287	37625600	314	37625415	11	5.66869	3.88198
Nc-1-IP_pe	chr1	37631009	37631332	324	37631165	12	5.77527	3.80164
Nc-1-IP_pe	chr1	37633546	37634282	737	37633748	13	6.54342	4.09407
Nc-1-IP_pe	chr1	37634821	37635197	377	37635162	9	3.75449	2.977
Nc-1-IP_pe	chr1	37643637	37644205	569	37643714	12	5.01934	3.37518
Nc-1-IP_pe	chr1	37650668	37651033	366	37650878	10	4.33501	3.21677
Nc-1-IP_pe	chr1	37651579	37651930	352	37651620	9	3.70468	2.94668
Nc-1-IP_pe	chr1	37661496	37661751	256	37661519	11	3.28475	2.53029
Nc-1-IP_pe	chr1	37662946	37663323	378	37663214	9	3.77975	2.99239
Nc-1-IP_pe	chr1	37677505	37677881	377	37677660	10	4.62142	3.388
Nc-1-IP_pe	chr1	37690773	37691164	392	37690796	13	4.02562	2.7544
Nc-1-IP_pe	chr1	37692509	37692851	343	37692650	16	4.59224	2.7853
Nc-1-IP_pe	chr1	37706274	37706592	319	37706463	13	6.84576	4.26658
Nc-1-IP_pe	chr1	37708306	37709110	805	37708942	13	6.92473	4.312
Nc-1-IP_pe	chr1	37716685	37716951	267	37716741	10	3.82047	2.91478
Nc-1-IP_pe	chr1	37719347	37720277	931	37719482	13	3.67377	2.58159
Nc-1-IP_pe	chr1	37740581	37740900	320	37740692	11	3.64839	2.72584
Nc-1-IP_pe	chr1	37747302	37747706	405	37747404	10	4.47495	3.30017
Nc-1-IP_pe	chr1	37752906	37753649	744	37753282	12	3.47963	2.55766
Nc-1-IP_pe	chr1	37761385	37761965	581	37761885	12	5.84943	3.84431
Nc-1-IP_pe	chr1	37764879	37765153	275	37764971	10	4.39571	3.25288
Nc-1-IP_pe	chr1	37800261	37800539	279	37800438	12	3.83688	2.74114
Nc-1-IP_pe	chr1	37802143	37802716	574	37802333	10	4.4323	3.2747
Nc-1-IP_pe	chr1	37807588	37808034	447	37807788	15	5.00915	3.05678
Nc-1-IP_pe	chr1	37815452	37816109	658	37815543	10	3.4604	2.70797

Nc-1-IP_pe	chr1	37833857	37834211	355	37833979	11	6.03439	4.10251
Nc-1-IP_pe	chr1	37839828	37840148	321	37839948	9	4.46937	3.41876
Nc-1-IP_pe	chr1	37856312	37856569	258	37856472	11	5.72344	3.91481
Nc-1-IP_pe	chr1	37859072	37859635	564	37859428	18	6.63038	3.50359
Nc-1-IP_pe	chr1	37860012	37860272	261	37860209	14	4.52309	2.91384
Nc-1-IP_pe	chr1	37862803	37863082	280	37862880	11	3.28475	2.53029
Nc-1-IP_pe	chr1	37895711	37896342	632	37895851	17	7.02067	3.79543
Nc-1-IP_pe	chr1	37896623	37897154	532	37896961	37	13.508	4.14596
Nc-1-IP_pe	chr1	37927592	37928605	1014	37928264	15	8.26005	4.73146
Nc-1-IP_pe	chr1	37931431	37931718	288	37931543	13	6.63566	4.14646
Nc-1-IP_pe	chr1	37940344	37940614	271	37940578	11	4.54404	3.2242
Nc-1-IP_pe	chr1	37941316	37941783	468	37941618	13	6.56172	4.10444
Nc-1-IP_pe	chr1	37943500	37943978	479	37943851	12	5.48538	3.63624
Nc-1-IP_pe	chr1	37949859	37950391	533	37950214	10	4.07292	3.06202
Nc-1-IP_pe	chr1	37951076	37952572	1497	37951590	15	7.02624	4.06292
Nc-1-IP_pe	chr1	37955135	37955898	764	37955873	8	3.38431	2.8478
Nc-1-IP_pe	chr1	37973901	37974215	315	37974079	14	7.42849	4.43574
Nc-1-IP_pe	chr1	37989004	37990243	1240	37990041	19	8.4638	4.21715
Nc-1-IP_pe	chr1	37990629	37990997	369	37990864	12	5.84943	3.84431
Nc-1-IP_pe	chr1	37996187	37996556	370	37996481	7	2.9185	2.63964
Nc-1-IP_pe	chr1	37997875	37998542	668	37998031	10	4.96929	3.59872
Nc-1-IP_pe	chr1	38000450	38000844	395	38000620	11	5.08426	3.53601
Nc-1-IP_pe	chr1	38001415	38001858	444	38001721	13	6.36468	3.99316
Nc-1-IP_pe	chr1	38004463	38005348	886	38004852	12	4.51041	3.09723
Nc-1-IP_pe	chr1	38026031	38026302	272	38026155	11	4.49162	3.19439
Nc-1-IP_pe	chr1	38042336	38042670	335	38042424	12	6.48784	4.21727
Nc-1-IP_pe	chr1	38044808	38045154	347	38044976	9	4.05981	3.16422
Nc-1-IP_pe	chr1	38046968	38047612	645	38047171	13	4.41954	2.952
Nc-1-IP_pe	chr1	38066937	38067403	467	38067019	12	6.6095	4.28941
Nc-1-IP_pe	chr1	38068053	38068518	466	38068100	11	5.99012	4.07567
Nc-1-IP_pe	chr1	38070046	38070363	318	38070190	14	8.63073	5.12814
Nc-1-IP_pe	chr1	38071101	38071491	391	38071443	10	3.08907	2.49869
Nc-1-IP_pe	chr1	38072705	38072957	253	38072813	10	3.89064	2.95552
Nc-1-IP_pe	chr1	38073248	38074393	1146	38073694	11	6.00992	4.08766
Nc-1-IP_pe	chr1	38075201	38075610	410	38075456	14	7.7972	4.64473
Nc-1-IP_pe	chr1	38087067	38087488	422	38087176	14	7.16603	4.28896
Nc-1-IP_pe	chr1	38088381	38088825	445	38088560	14	6.06519	3.69269
Nc-1-IP_pe	chr1	38106759	38107368	610	38106925	12	6.38914	4.15897
Nc-1-IP_pe	chr1	38113351	38113884	534	38113860	10	3.08907	2.49869
Nc-1-IP_pe	chr1	38124713	38125020	308	38124941	11	4.62357	3.2696
Nc-1-IP_pe	chr1	38125831	38126145	315	38125947	15	6.69889	3.8921
Nc-1-IP_pe	chr1	38127645	38127959	315	38127851	17	5.54967	3.12324
Nc-1-IP_pe	chr1	38130420	38130714	295	38130659	11	4.00079	2.91908
Nc-1-IP_pe	chr1	38132217	38132516	300	38132270	12	5.01934	3.37518
Nc-1-IP_pe	chr1	38139156	38139570	415	38139381	15	5.20142	3.14789

Nc-1-IP_pe	chr1	38190707	38191124	418	38190886	13	6.82624	4.25537
Nc-1-IP_pe	chr1	38207286	38207680	395	38207508	11	4.00079	2.91908
Nc-1-IP_pe	chr1	38213729	38214083	355	38213910	15	7.56326	4.34922
Nc-1-IP_pe	chr1	38217592	38217885	294	38217666	12	6.79895	4.40238
Nc-1-IP_pe	chr1	38239354	38239608	255	38239476	9	4.46937	3.41876
Nc-1-IP_pe	chr1	38244015	38244270	256	38244109	10	4.82246	3.50943
Nc-1-IP_pe	chr1	38254373	38254701	329	38254442	10	4.20113	3.13749
Nc-1-IP_pe	chr1	38255879	38256520	642	38256421	11	5.65062	3.87116
Nc-1-IP_pe	chr1	38269172	38269484	313	38269243	9	4.46937	3.41876
Nc-1-IP_pe	chr1	38272172	38272448	277	38272304	13	6.62436	4.14003
Nc-1-IP_pe	chr1	38285656	38285908	253	38285774	13	6.92473	4.312
Nc-1-IP_pe	chr1	38377882	38378154	273	38378012	10	5.23357	3.76063
Nc-1-IP_pe	chr1	38420725	38421065	341	38421012	8	3.08755	2.66145
Nc-1-IP_pe	chr1	38485212	38485537	326	38485365	11	4.54404	3.2242
Nc-1-IP_pe	chr1	38487422	38487679	258	38487437	8	3.08755	2.66145
Nc-1-IP_pe	chr1	38514543	38514824	282	38514637	9	4.25966	3.28797
Nc-1-IP_pe	chr1	38522132	38522439	308	38522232	10	4.39571	3.25288
Nc-1-IP_pe	chr1	38542362	38542709	348	38542514	12	6.58417	4.27436
Nc-1-IP_pe	chr1	38572366	38572635	270	38572534	9	3.72192	2.95716
Nc-1-IP_pe	chr1	38577327	38577837	511	38577567	10	5.23357	3.76063
Nc-1-IP_pe	chr1	38578362	38578673	312	38578473	12	5.23082	3.49289
Nc-1-IP_pe	chr1	38580918	38581392	475	38581008	10	4.99888	3.61677
Nc-1-IP_pe	chr1	38582217	38582563	347	38582276	8	3.56021	2.95918
Nc-1-IP_pe	chr1	38623700	38624000	301	38623868	11	4.54404	3.2242
Nc-1-IP_pe	chr1	38624626	38625054	429	38624787	9	3.27326	2.68684
Nc-1-IP_pe	chr1	38634630	38635134	505	38634948	13	5.36853	3.44651
Nc-1-IP_pe	chr1	38646839	38647205	367	38647029	14	6.44441	3.89444
Nc-1-IP_pe	chr1	38670585	38670971	387	38670677	10	3.4604	2.70797
Nc-1-IP_pe	chr1	38674886	38675821	936	38675191	11	6.03439	4.10251
Nc-1-IP_pe	chr1	38696276	38696848	573	38696335	9	3.27326	2.68684
Nc-1-IP_pe	chr1	38719986	38720267	282	38720103	14	7.42849	4.43574
Nc-1-IP_pe	chr1	38736929	38737414	486	38736995	10	5.23357	3.76063
Nc-1-IP_pe	chr1	38755163	38755462	300	38755365	7	3.06442	2.73501
Nc-1-IP_pe	chr1	38772611	38772895	285	38772734	14	6.69566	4.03025
Nc-1-IP_pe	chr1	38802160	38802459	300	38802307	9	4.46937	3.41876
Nc-1-IP_pe	chr1	38819395	38819734	340	38819526	11	4.81752	3.38105
Nc-1-IP_pe	chr1	38830815	38831235	421	38831098	10	5.23357	3.76063
Nc-1-IP_pe	chr1	38848753	38849669	917	38849004	13	5.9487	3.76157
Nc-1-IP_pe	chr1	38850064	38850412	349	38850336	9	4.25966	3.28797
Nc-1-IP_pe	chr1	38859235	38860418	1184	38860119	18	8.62313	4.4354
Nc-1-IP_pe	chr1	38872924	38873276	353	38873026	17	7.36791	3.96218
Nc-1-IP_pe	chr1	38897992	38898271	280	38898210	10	5.23357	3.76063
Nc-1-IP_pe	chr1	38911917	38912629	713	38912540	13	7.7352	4.78626
Nc-1-IP_pe	chr1	38934107	38934473	367	38934402	9	3.88314	3.0556
Nc-1-IP_pe	chr1	38941426	38942120	695	38941771	14	5.18557	3.24018

Nc-1-IP_pe	chr1	38956208	38956492	285	38956359	13	5.9487	3.76157
Nc-1-IP_pe	chr1	38968103	38968474	372	38968193	7	3.03708	2.71711
Nc-1-IP_pe	chr1	38988019	38988513	495	38988130	10	3.89064	2.95552
Nc-1-IP_pe	chr1	38991332	38991960	629	38991640	14	6.78215	4.0774
Nc-1-IP_pe	chr1	39000073	39000622	550	39000547	11	6.03439	4.10251
Nc-1-IP_pe	chr1	39004779	39005066	288	39004935	10	4.96929	3.59872
Nc-1-IP_pe	chr1	39014116	39014560	445	39014439	13	3.67377	2.58159
Nc-1-IP_pe	chr1	39025544	39026473	930	39026418	13	4.56123	3.02417
Nc-1-IP_pe	chr1	39026850	39027359	510	39027301	11	5.03835	3.5092
Nc-1-IP_pe	chr1	39036809	39037359	551	39037284	10	5.23357	3.76063
Nc-1-IP_pe	chr1	39044037	39044304	268	39044162	10	3.89064	2.95552
Nc-1-IP_pe	chr1	39047144	39047460	317	39047280	13	6.41752	4.02291
Nc-1-IP_pe	chr1	39061194	39061666	473	39061322	7	3.06442	2.73501
Nc-1-IP_pe	chr1	39072705	39073169	465	39073019	10	4.39571	3.25288
Nc-1-IP_pe	chr1	39075906	39076950	1045	39076356	12	6.86906	4.44438
Nc-1-IP_pe	chr1	39080567	39081717	1151	39081445	11	4.54404	3.2242
Nc-1-IP_pe	chr1	39082188	39082745	558	39082696	11	2.96397	2.36091
Nc-1-IP_pe	chr1	39098284	39098711	428	39098521	17	7.6273	4.08876
Nc-1-IP_pe	chr1	39105993	39106372	380	39106105	15	7.02624	4.06292
Nc-1-IP_pe	chr1	39121471	39121757	287	39121631	14	7.75387	4.62
Nc-1-IP_pe	chr1	39133065	39133320	256	39133208	11	4.54404	3.2242
Nc-1-IP_pe	chr1	39198197	39198546	350	39198454	14	6.13058	3.7272
Nc-1-IP_pe	chr1	39234164	39234558	395	39234314	10	4.62142	3.388
Nc-1-IP_pe	chr1	39350836	39351241	406	39350920	9	4.21839	3.26234
Nc-1-IP_pe	chr1	39404190	39404468	279	39404399	11	5.45748	3.75598
Nc-1-IP_pe	chr1	39408126	39408486	361	39408255	11	5.03835	3.5092
Nc-1-IP_pe	chr1	39409048	39409693	646	39409076	13	3.35769	2.42919
Nc-1-IP_pe	chr1	39413209	39413579	371	39413374	10	3.94999	2.99009
Nc-1-IP_pe	chr1	39457656	39458028	373	39457713	11	4.61014	3.26192
Nc-1-IP_pe	chr1	39490389	39490825	437	39490806	13	4.02562	2.7544
Nc-1-IP_pe	chr1	39492573	39492957	385	39492738	11	4.29098	3.08099
Nc-1-IP_pe	chr1	39511067	39512076	1010	39511297	16	7.83	4.33588
Nc-1-IP_pe	chr1	39512596	39512864	269	39512745	12	5.07448	3.40574
Nc-1-IP_pe	chr1	39514571	39515534	964	39515264	20	10.7672	5.16976
Nc-1-IP_pe	chr1	39518463	39518814	352	39518601	13	4.86362	3.18015
Nc-1-IP_pe	chr1	39525622	39525910	289	39525777	12	4.24064	2.95299
Nc-1-IP_pe	chr1	39527282	39527535	254	39527475	11	4.74697	3.34038
Nc-1-IP_pe	chr1	39543073	39543393	321	39543319	12	6.33104	4.12476
Nc-1-IP_pe	chr1	39567908	39568197	290	39568002	12	5.89413	3.8701
Nc-1-IP_pe	chr1	39576290	39576657	368	39576467	17	9.65211	5.13407
Nc-1-IP_pe	chr1	39590982	39591393	412	39591334	11	4.8033	3.37283
Nc-1-IP_pe	chr1	39592315	39592666	352	39592501	11	4.06403	2.95415
Nc-1-IP_pe	chr1	39603406	39603794	389	39603552	10	5.23357	3.76063
Nc-1-IP_pe	chr1	39609700	39610063	364	39610002	13	6.02837	3.80557
Nc-1-IP_pe	chr1	39611804	39612142	339	39611991	14	8.32526	4.94932

Nc-1-IP_pe	chr1	39625364	39625924	561	39625816	17	8.31233	4.43123
Nc-1-IP_pe	chr1	39626455	39627300	846	39626704	14	5.51541	3.4073
Nc-1-IP_pe	chr1	39631926	39632179	254	39632040	10	3.89064	2.95552
Nc-1-IP_pe	chr1	39632647	39633212	566	39632881	11	5.14641	3.5724
Nc-1-IP_pe	chr1	39633570	39633889	320	39633746	17	8.31233	4.43123
Nc-1-IP_pe	chr1	39639442	39640631	1190	39639537	18	7.73136	4.00629
Nc-1-IP_pe	chr1	39644240	39644611	372	39644347	12	5.72541	3.77304
Nc-1-IP_pe	chr1	39646580	39646902	323	39646741	12	5.61153	3.70794
Nc-1-IP_pe	chr1	39669059	39669735	677	39669685	11	5.3232	3.67643
Nc-1-IP_pe	chr1	39672132	39672451	320	39672145	14	5.03085	3.16286
Nc-1-IP_pe	chr1	39687221	39687595	375	39687446	17	8.41245	4.48226
Nc-1-IP_pe	chr1	39690711	39691271	561	39691222	16	6.33285	3.58457
Nc-1-IP_pe	chr1	39712395	39712663	269	39712517	14	6.69566	4.03025
Nc-1-IP_pe	chr1	39727573	39728221	649	39728010	10	5.23357	3.76063
Nc-1-IP_pe	chr1	39752837	39753270	434	39753004	11	4.54404	3.2242
Nc-1-IP_pe	chr1	39755564	39756391	828	39756277	13	6.62436	4.14003
Nc-1-IP_pe	chr1	39758528	39758835	308	39758696	12	3.83688	2.74114
Nc-1-IP_pe	chr1	39760193	39761223	1031	39760494	14	6.44441	3.89444
Nc-1-IP_pe	chr1	39762579	39763236	658	39763056	15	5.96953	3.52194
Nc-1-IP_pe	chr1	39764566	39764906	341	39764824	18	4.96968	2.80074
Nc-1-IP_pe	chr1	39766667	39767597	931	39766749	18	3.29891	2.1529
Nc-1-IP_pe	chr1	39769563	39771595	2033	39769739	19	8.4638	4.21715
Nc-1-IP_pe	chr1	39774515	39774943	429	39774631	14	6.54309	3.94758
Nc-1-IP_pe	chr1	39778821	39779667	847	39779290	14	6.36383	3.85124
Nc-1-IP_pe	chr1	39784063	39784696	634	39784434	18	6.15736	3.29676
Nc-1-IP_pe	chr1	39788823	39789099	277	39789036	18	4.96968	2.80074
Nc-1-IP_pe	chr1	39799072	39799811	740	39799372	15	3.74763	2.48304
Nc-1-IP_pe	chr1	39805721	39805979	259	39805805	9	4.46937	3.41876
Nc-1-IP_pe	chr1	39820772	39821223	452	39821157	10	3.77478	2.88832
Nc-1-IP_pe	chr1	39823804	39824080	277	39823932	11	4.06403	2.95415
Nc-1-IP_pe	chr1	39834415	39834937	523	39834856	11	5.10577	3.5486
Nc-1-IP_pe	chr1	39845583	39845856	274	39845642	11	3.78682	2.80131
Nc-1-IP_pe	chr1	39868484	39868978	495	39868493	8	3.4722	2.90337
Nc-1-IP_pe	chr1	39869562	39870152	591	39869742	11	3.64839	2.72584
Nc-1-IP_pe	chr1	39871189	39871550	362	39871212	8	3.08755	2.66145
Nc-1-IP_pe	chr1	39884636	39885025	390	39884937	12	4.70082	3.20033
Nc-1-IP_pe	chr1	39891779	39892068	290	39891906	12	6.50787	4.22912
Nc-1-IP_pe	chr1	39901017	39901445	429	39901301	20	6.08336	3.09555
Nc-1-IP_pe	chr1	39903406	39904043	638	39903877	14	6.06519	3.69269
Nc-1-IP_pe	chr1	39912880	39913229	350	39913164	16	4.96462	2.94973
Nc-1-IP_pe	chr1	39928413	39928762	350	39928634	14	5.838	3.57373
Nc-1-IP_pe	chr1	39934495	39935104	610	39934566	13	4.86362	3.18015
Nc-1-IP_pe	chr1	39936643	39936941	299	39936816	16	7.53857	4.18505
Nc-1-IP_pe	chr1	39940856	39941164	309	39941063	14	5.51541	3.4073
Nc-1-IP_pe	chr1	39949103	39949357	255	39949288	15	7.83541	4.49711

Nc-1-IP_pe	chr1	39954812	39955078	267	39954986	17	5.14803	2.94914	
Nc-1-IP_pe	chr1	39955432	39955684	253	39955568	19	6.56181	3.3752	
Nc-1-IP_pe	chr1	39956220	39956573	354	39956405	17	8.9884	4.78058	
Nc-1-IP_pe	chr1	39957382	39957708	327	39957418	11	3.64839	2.72584	
Nc-1-IP_pe	chr1	39960557	39961045	489	39960787	21	13.4004	6.32172	
Nc-1-IP_pe	chr1	39965642	39966020	379	39965907	13	6.74901	4.21113	
Nc-1-IP_pe	chr1	39968123	39968790	668	39968313	10	4.37633	3.24134	
Nc-1-IP_pe	chr1	39979116	39979400	285	39979341	14	7.42849	4.43574	
Nc-1-IP_pe	chr1	39999283	40000038	756	39999960	14	4.60011	2.95114	
Nc-1-IP_pe	chr1	40012629	40012965	337	40012864	7	3.06442	2.73501	
Nc-1-IP_pe	chr1	40018256	40019120	865	40018384	11	6.03439	4.10251	
Nc-1-IP_pe	chr1	40029146	40029437	292	40029365	14	6.44441	3.89444	
Nc-1-IP_pe	chr1	40063258	40063541	284	40063377	8	3.74505	3.07688	
Nc-1-IP_pe	chr1	40071702	40072049	348	40071716	11	3.28475	2.53029	
Nc-1-IP_pe	chr1	40097009	40097462	454	40097162	19	7.84735	3.93486	
Nc-1-IP_pe	chr1	40127998	40128429	432	40128046	11	5.61473	3.84969	
Nc-1-IP_pe	chr1	40128963	40129286	324	40129102	14	7.86294	4.68231	
Nc-1-IP_pe	chr1	40137277	40137996	720	40137899	13	7.42513	4.60316	
Nc-1-IP_pe	chr1	40143816	40144075	260	40143944	10	5.23357	3.76063	
Nc-1-IP_pe	chr1	40169803	40170057	255	40169906	15	9.55381	5.47001	
Nc-1-IP_pe	chr1	40216133	40216385	253	40216238	11	6.03439	4.10251	
Nc-1-IP_pe	chr1	40253540	40254048	509	40253890	11	5.10577	3.5486	
Nc-1-IP_pe	chr1	40257682	40258577	896	40257802	17	6.47874	3.54137	
Nc-1-IP_pe	chr1	40291816	40292110	295	40291973	14	8.63073	5.12814	
Nc-1-IP_pe	chr1	40302537	40302982	446	40302695	14	4.21449	2.76599	
Nc-1-IP_pe	chr1	40303750	40304087	338	40303869	13	4.02562	2.7544	
Nc-1-IP_pe	chr1	40305718	40306073	356	40305913	14	4.60011	2.95114	
Nc-1-IP_pe	chr1	40307487	40307785	299	40307717	12	4.1728	2.91706	
Nc-1-IP_pe	chr1	40310461	40311135	675	40311076	12	4.24064	2.95299	
Nc-1-IP_pe	chr1	40313351	40314118	768	40313848	14	6.47706	3.91199	
Nc-1-IP_pe	chr1	40315247	40315695	449	40315639	12	4.51041	3.09723	
Nc-1-IP_pe	chr1	40316957	40317253	297	40317020	13	4.78908	3.14145	
Nc-1-IP_pe	chr1	40327677	40328230	554	40327736	11	3.64839	2.72584	
Nc-1-IP_pe	chr1	40353345	40353597	253	40353444	9	4.46937	3.41876	
Nc-1-IP_pe	chr1	40370915	40371172	258	40371018	12	4.70082	3.20033	
Nc-1-IP_pe	chr1	40373680	40374036	357	40373923	17	6.47874	3.54137	
Nc-1-IP_pe	chr1	40401011	40401349	339	40401188	11	6.03439	4.10251	
Nc-1-IP_pe	chr1	40421896	40422233	338	40422126	9	3.27326	2.68684	
Nc-1-IP_pe	chr1	40449850	40450348	499	40450259	12	4.24064	2.95299	
Nc-1-IP_pe	chr1	40474662	40474919	258	40474750	12	6.86906	4.44438	
Nc-1-IP_pe	chr1	40477137	40477630	494	40477314	13	6.36468	3.99316	
Nc-1-IP_pe	chr1	40483908	40484238	331	40484184	8	3.74505	3.07688	
Nc-1-IP_pe	chr1	40496837	40497106	270	40496969	12	6.86906	4.44438	
Nc-1-IP_pe	chr1	40508995	40509535	541	40509103	13	4.67336	3.0817	
Nc-1-IP_pe	chr1	40514515	40514768	254	40514632	8	3.69948	3.0478	

Nc-1-IP_pe	chr1	40534004	40534478	475	40534022	9	4.25966	3.28797
Nc-1-IP_pe	chr1	40554709	40554992	284	40554842	9	4.46937	3.41876
Nc-1-IP_pe	chr1	40557488	40557817	330	40557680	12	6.86906	4.44438
Nc-1-IP_pe	chr1	40558827	40559262	436	40559125	9	3.43499	2.78365
Nc-1-IP_pe	chr1	40572220	40572578	359	40572407	12	4.70082	3.20033
Nc-1-IP_pe	chr1	40580257	40580706	450	40580614	8	3.74505	3.07688
Nc-1-IP_pe	chr1	40595250	40595605	356	40595444	16	10.0135	5.53039
Nc-1-IP_pe	chr1	40601945	40602301	357	40602216	10	4.99888	3.61677
Nc-1-IP_pe	chr1	40610109	40610389	281	40610345	10	5.23357	3.76063
Nc-1-IP_pe	chr1	40612694	40613017	324	40612855	17	5.14803	2.94914
Nc-1-IP_pe	chr1	40613900	40614178	279	40614094	10	5.23357	3.76063
Nc-1-IP_pe	chr1	40619971	40620536	566	40620453	9	3.72192	2.95716
Nc-1-IP_pe	chr1	40621432	40621836	405	40621478	10	3.4604	2.70797
Nc-1-IP_pe	chr1	40625544	40625934	391	40625861	10	3.71867	2.85592
Nc-1-IP_pe	chr1	40626353	40626715	363	40626495	19	7.84735	3.93486
Nc-1-IP_pe	chr1	40630743	40631230	488	40631032	14	6.28469	3.80898
Nc-1-IP_pe	chr1	40641676	40642244	569	40641748	10	4.41822	3.2663
Nc-1-IP_pe	chr1	40645827	40646101	275	40645979	12	5.94619	3.9002
Nc-1-IP_pe	chr1	40646809	40647202	394	40647024	11	5.77463	3.94557
Nc-1-IP_pe	chr1	40648927	40649263	337	40649125	12	5.8598	3.85029
Nc-1-IP_pe	chr1	40649698	40650167	470	40649930	10	4.29423	3.19257
Nc-1-IP_pe	chr1	40653835	40654117	283	40653945	12	4.75569	3.23024
Nc-1-IP_pe	chr1	40654837	40655745	909	40655010	15	6.19428	3.63445
Nc-1-IP_pe	chr1	40658353	40658618	266	40658378	10	3.8904	2.95538
Nc-1-IP_pe	chr1	40662341	40662605	265	40662482	14	6.52649	3.93862
Nc-1-IP_pe	chr1	40667906	40668174	269	40668062	9	3.83096	3.02367
Nc-1-IP_pe	chr1	40678167	40678575	409	40678289	12	6.40869	4.1705
Nc-1-IP_pe	chr1	40691643	40692167	525	40692007	21	8.55202	4.00473
Nc-1-IP_pe	chr1	40726258	40726676	419	40726549	11	5.77463	3.94557
Nc-1-IP_pe	chr1	40727528	40728045	518	40727944	7	2.9043	2.63038
Nc-1-IP_pe	chr1	40728346	40728729	384	40728408	8	3.74505	3.07688
Nc-1-IP_pe	chr1	40729751	40730051	301	40729860	13	4.86362	3.18015
Nc-1-IP_pe	chr1	40738004	40738269	266	40738093	11	5.3563	3.696
Nc-1-IP_pe	chr1	40747963	40748257	295	40748030	15	3.46204	2.35851
Nc-1-IP_pe	chr1	40770485	40770898	414	40770544	11	4.35236	3.11555
Nc-1-IP_pe	chr1	40781145	40781535	391	40781335	16	7.8117	4.32634
Nc-1-IP_pe	chr1	40783257	40783685	429	40783498	20	9.94634	4.77022
Nc-1-IP_pe	chr1	40784329	40784888	560	40784782	16	5.85814	3.35873
Nc-1-IP_pe	chr1	40791557	40792157	601	40792101	11	4.67786	3.30068
Nc-1-IP_pe	chr1	40798657	40799718	1062	40799039	19	8.4638	4.21715
Nc-1-IP_pe	chr1	40800235	40800652	418	40800576	14	6.46071	3.9032
Nc-1-IP_pe	chr1	40810145	40810444	300	40810264	13	6.54342	4.09407
Nc-1-IP_pe	chr1	40823327	40823979	653	40823584	14	6.06519	3.69269
Nc-1-IP_pe	chr1	40824258	40824517	260	40824478	11	4.35236	3.11555
Nc-1-IP_pe	chr1	40832778	40833282	505	40832833	13	5.42865	3.47872

Nc-1-IP_pe	chr1	40837478	40837828	351	40837764	11	3.68532	2.74592
Nc-1-IP_pe	chr1	40855724	40856282	559	40855861	12	5.84943	3.84431
Nc-1-IP_pe	chr1	40862036	40862299	264	40862265	14	3.26761	2.32785
Nc-1-IP_pe	chr1	40863594	40863932	339	40863872	12	4.24064	2.95299
Nc-1-IP_pe	chr1	40869009	40869460	452	40869071	14	6.06519	3.69269
Nc-1-IP_pe	chr1	40874052	40874439	388	40874248	13	7.34205	4.55444
Nc-1-IP_pe	chr1	40877811	40878111	301	40878006	12	6.18005	4.03623
Nc-1-IP_pe	chr1	40879936	40880399	464	40880016	11	4.54404	3.2242
Nc-1-IP_pe	chr1	40890448	40890795	348	40890610	14	4.21449	2.76599
Nc-1-IP_pe	chr1	40910448	40910723	276	40910716	8	3.4091	2.86346
Nc-1-IP_pe	chr1	40975258	40975942	685	40975297	12	5.01934	3.37518
Nc-1-IP_pe	chr1	41013428	41013756	329	41013670	11	4.55694	3.23155
Nc-1-IP_pe	chr1	41027954	41028373	420	41027968	10	3.12201	2.51709
Nc-1-IP_pe	chr1	41046400	41046761	362	41046477	12	3.83688	2.74114
Nc-1-IP_pe	chr1	41059224	41059881	658	41059773	16	7.53857	4.18505
Nc-1-IP_pe	chr1	41106076	41106372	297	41106155	12	6.58417	4.27436
Nc-1-IP_pe	chr1	41122878	41123216	339	41123016	10	5.23357	3.76063
Nc-1-IP_pe	chr1	41150215	41150507	293	41150366	10	5.23357	3.76063
Nc-1-IP_pe	chr1	41176406	41177268	863	41177087	14	6.78215	4.0774
Nc-1-IP_pe	chr1	41205252	41205567	316	41205309	11	5.3563	3.696
Nc-1-IP_pe	chr1	41257465	41257776	312	41257668	13	4.56123	3.02417
Nc-1-IP_pe	chr1	41290708	41291009	302	41290817	13	5.71736	3.63481
Nc-1-IP_pe	chr1	41315697	41316115	419	41316081	9	3.37986	2.75057
Nc-1-IP_pe	chr1	41316973	41317481	509	41317093	14	6.16097	3.74327
Nc-1-IP_pe	chr1	41320440	41320823	384	41320793	13	4.9946	3.24853
Nc-1-IP_pe	chr1	41331329	41331583	255	41331526	10	4.16209	3.11446
Nc-1-IP_pe	chr1	41341994	41342502	509	41342394	15	4.7819	2.95039
Nc-1-IP_pe	chr1	41353309	41353583	275	41353435	14	6.67814	4.02073
Nc-1-IP_pe	chr1	41357579	41357878	300	41357619	14	4.60011	2.95114
Nc-1-IP_pe	chr1	41361949	41363018	1070	41362091	21	10.392	4.82431
Nc-1-IP_pe	chr1	41365396	41365954	559	41365517	19	8.4638	4.21715
Nc-1-IP_pe	chr1	41366656	41366953	298	41366897	11	3.68532	2.74592
Nc-1-IP_pe	chr1	41368249	41368503	255	41368397	14	5.24628	3.27071
Nc-1-IP_pe	chr1	41368847	41369243	397	41369035	17	6.47874	3.54137
Nc-1-IP_pe	chr1	41372839	41373153	315	41373033	19	7.84735	3.93486
Nc-1-IP_pe	chr1	41376934	41377475	542	41377387	16	7.20904	4.01714
Nc-1-IP_pe	chr1	41380309	41380742	434	41380431	13	4.41954	2.952
Nc-1-IP_pe	chr1	41381763	41382045	283	41381863	17	5.9911	3.31919
Nc-1-IP_pe	chr1	41394548	41395413	866	41394696	14	6.36383	3.85124
Nc-1-IP_pe	chr1	41404374	41404719	346	41404574	17	7.6273	4.08876
Nc-1-IP_pe	chr1	41406915	41407215	301	41407120	15	7.62034	4.38009
Nc-1-IP_pe	chr1	41407866	41408120	255	41407994	14	6.06519	3.69269
Nc-1-IP_pe	chr1	41411839	41412284	446	41411901	12	4.70082	3.20033
Nc-1-IP_pe	chr1	41414655	41414933	279	41414868	14	4.95172	3.12357
Nc-1-IP_pe	chr1	41415660	41416570	911	41416110	13	4.9946	3.24853

Nc-1-IP_pe	chr1	41423676	41424190	515	41423997	19	8.4638	4.21715
Nc-1-IP_pe	chr1	41427879	41428207	329	41428040	13	3.35769	2.42919
Nc-1-IP_pe	chr1	41431791	41432764	974	41431865	15	4.40338	2.77622
Nc-1-IP_pe	chr1	41435385	41435917	533	41435555	15	7.35943	4.23968
Nc-1-IP_pe	chr1	41448372	41448768	397	41448605	15	5.66913	3.37372
Nc-1-IP_pe	chr1	41456895	41457219	325	41456929	10	3.86691	2.94172
Nc-1-IP_pe	chr1	41463129	41463385	257	41463182	13	5.71736	3.63481
Nc-1-IP_pe	chr1	41466202	41466556	355	41466408	15	6.78905	3.93887
Nc-1-IP_pe	chr1	41474103	41474494	392	41474227	10	4.17505	3.1221
Nc-1-IP_pe	chr1	41475043	41475297	255	41475146	16	5.82808	3.34463
Nc-1-IP_pe	chr1	41476338	41476641	304	41476431	9	3.46864	2.80388
Nc-1-IP_pe	chr1	41478224	41478524	301	41478347	13	4.86362	3.18015
Nc-1-IP_pe	chr1	41480379	41480956	578	41480853	12	4.70082	3.20033
Nc-1-IP_pe	chr1	41484106	41484476	371	41484129	11	4.02303	2.9314
Nc-1-IP_pe	chr1	41484864	41485189	326	41485036	11	4.17167	3.01412
Nc-1-IP_pe	chr1	41491441	41491906	466	41491464	14	3.86727	2.6027
Nc-1-IP_pe	chr1	41493990	41494374	385	41494107	15	5.20142	3.14789
Nc-1-IP_pe	chr1	41502601	41503219	619	41502821	14	5.03085	3.16286
Nc-1-IP_pe	chr1	41505218	41505493	276	41505374	16	4.25252	2.63824
Nc-1-IP_pe	chr1	41507263	41507881	619	41507662	12	4.68027	3.18915
Nc-1-IP_pe	chr1	41511028	41511350	323	41511193	16	6.65665	3.74206
Nc-1-IP_pe	chr1	41512806	41513819	1014	41513212	18	7.47506	3.88659
Nc-1-IP_pe	chr1	41519149	41519442	294	41519266	13	4.87233	3.18468
Nc-1-IP_pe	chr1	41532993	41533271	279	41533091	16	8.21011	4.53581
Nc-1-IP_pe	chr1	41540183	41540533	351	41540394	12	5.61153	3.70794
Nc-1-IP_pe	chr1	41560212	41560577	366	41560340	13	5.9487	3.76157
Nc-1-IP_pe	chr1	41567436	41568450	1015	41568434	12	3.16137	2.39719
Nc-1-IP_pe	chr1	41574272	41574550	279	41574361	17	5.7822	3.22586
Nc-1-IP_pe	chr1	41576148	41576540	393	41576245	14	6.87069	4.12585
Nc-1-IP_pe	chr1	41579696	41580224	529	41580115	10	3.89064	2.95552
Nc-1-IP_pe	chr1	41584390	41584711	322	41584442	10	4.16209	3.11446
Nc-1-IP_pe	chr1	41585133	41585411	279	41585167	10	4.20113	3.13749
Nc-1-IP_pe	chr1	41589313	41589711	399	41589562	14	6.06519	3.69269
Nc-1-IP_pe	chr1	41592714	41593019	306	41592948	13	6.02837	3.80557
Nc-1-IP_pe	chr1	41595791	41596073	283	41595802	12	4.70082	3.20033
Nc-1-IP_pe	chr1	41603383	41603692	310	41603476	12	6.71369	4.35145
Nc-1-IP_pe	chr1	41612588	41612903	316	41612814	14	5.838	3.57373
Nc-1-IP_pe	chr1	41614051	41614374	324	41614235	14	6.69566	4.03025
Nc-1-IP_pe	chr1	41618434	41619060	627	41619020	13	6.63566	4.14646
Nc-1-IP_pe	chr1	41619566	41620854	1289	41619713	13	6.58008	4.11487
Nc-1-IP_pe	chr1	41628277	41628541	265	41628375	15	7.02624	4.06292
Nc-1-IP_pe	chr1	41656960	41657388	429	41657307	14	7.44075	4.44264
Nc-1-IP_pe	chr1	41657706	41657972	267	41657821	14	7.40051	4.42001
Nc-1-IP_pe	chr1	41663624	41664240	617	41664042	12	3.83688	2.74114
Nc-1-IP_pe	chr1	41666680	41667053	374	41666927	17	7.59026	4.07058

Nc-1-IP_pe	chr1	41671101	41671694	594	41671301	16	7.39088	4.10945
Nc-1-IP_pe	chr1	41672027	41672370	344	41672178	15	5.20142	3.14789
Nc-1-IP_pe	chr1	41675705	41676002	298	41675844	11	4.06403	2.95415
Nc-1-IP_pe	chr1	41698570	41698867	298	41698744	9	3.27326	2.68684
Nc-1-IP_pe	chr1	41711145	41711426	282	41711232	9	3.06256	2.56181
Nc-1-IP_pe	chr1	41729775	41730733	959	41730193	13	6.17638	3.88776
Nc-1-IP_pe	chr1	41738082	41738336	255	41738245	12	5.36329	3.56726
Nc-1-IP_pe	chr1	41745562	41746112	551	41746021	11	4.54404	3.2242
Nc-1-IP_pe	chr1	41746837	41747458	622	41747300	15	8.87231	5.07663
Nc-1-IP_pe	chr1	41751010	41751268	259	41751027	8	3.68553	3.03891
Nc-1-IP_pe	chr1	41752615	41752989	375	41752632	10	3.4604	2.70797
Nc-1-IP_pe	chr1	41786145	41786441	297	41786206	9	3.51419	2.83132
Nc-1-IP_pe	chr1	41806497	41807220	724	41806630	11	4.54404	3.2242
Nc-1-IP_pe	chr1	41830461	41830775	315	41830581	11	6.03439	4.10251
Nc-1-IP_pe	chr1	41833735	41834013	279	41833978	8	3.38431	2.8478
Nc-1-IP_pe	chr1	41839346	41839628	283	41839482	15	5.20142	3.14789
Nc-1-IP_pe	chr1	41844847	41845173	327	41845009	14	6.69566	4.03025
Nc-1-IP_pe	chr1	41848615	41849022	408	41848735	12	3.47963	2.55766
Nc-1-IP_pe	chr1	41854162	41854470	309	41854293	16	6.8987	3.86161
Nc-1-IP_pe	chr1	41866208	41866821	614	41866454	11	4.54404	3.2242
Nc-1-IP_pe	chr1	41917955	41918284	330	41918061	13	5.9487	3.76157
Nc-1-IP_pe	chr1	42011639	42012063	425	42011974	9	4.32403	3.32802
Nc-1-IP_pe	chr1	42036022	42036513	492	42036397	12	5.84943	3.84431
Nc-1-IP_pe	chr1	42040258	42041425	1168	42040340	15	8.26005	4.73146
Nc-1-IP_pe	chr1	42080986	42081464	479	42081132	19	8.35089	4.16476
Nc-1-IP_pe	chr1	42104498	42104839	342	42104791	9	3.72192	2.95716
Nc-1-IP_pe	chr1	42115808	42116108	301	42116038	11	5.19376	3.60018
Nc-1-IP_pe	chr1	42131169	42131657	489	42131287	12	6.42833	4.1821
Nc-1-IP_pe	chr1	42146254	42146770	517	42146301	12	4.24064	2.95299
Nc-1-IP_pe	chr1	42152526	42152932	407	42152773	17	7.36791	3.96218
Nc-1-IP_pe	chr1	42153241	42153654	414	42153449	15	4.90347	3.00714
Nc-1-IP_pe	chr1	42154766	42155026	261	42154992	13	3.14632	2.32868
Nc-1-IP_pe	chr1	42162663	42162972	310	42162688	14	4.60011	2.95114
Nc-1-IP_pe	chr1	42164287	42165188	902	42164538	18	9.19569	4.72112
Nc-1-IP_pe	chr1	42167448	42168162	715	42167686	13	4.9575	3.22911
Nc-1-IP_pe	chr1	42171577	42171831	255	42171714	13	4.41954	2.952
Nc-1-IP_pe	chr1	42172955	42173603	649	42173115	14	5.69896	3.50164
Nc-1-IP_pe	chr1	42191312	42191629	318	42191434	10	3.89064	2.95552
Nc-1-IP_pe	chr1	42217303	42217589	287	42217460	12	5.84943	3.84431
Nc-1-IP_pe	chr1	42226422	42226689	268	42226453	10	5.23357	3.76063
Nc-1-IP_pe	chr1	42257512	42257809	298	42257680	11	6.03439	4.10251
Nc-1-IP_pe	chr1	42314346	42314659	314	42314524	14	7.34079	4.38651
Nc-1-IP_pe	chr1	42334394	42334962	569	42334689	20	8.56319	4.1316
Nc-1-IP_pe	chr1	42336825	42337567	743	42337396	13	6.41752	4.02291
Nc-1-IP_pe	chr1	42356127	42356553	427	42356468	8	3.74505	3.07688

Nc-1-IP_pe	chr1	42359216	42359771	556	42359667	12	6.58417	4.27436
Nc-1-IP_pe	chr1	42375670	42375990	321	42375839	8	3.08755	2.66145
Nc-1-IP_pe	chr1	42380219	42381273	1055	42381036	18	8.37884	4.31591
Nc-1-IP_pe	chr1	42439287	42439576	290	42439454	12	3.47963	2.55766
Nc-1-IP_pe	chr1	42440393	42440671	279	42440539	13	5.9487	3.76157
Nc-1-IP_pe	chr1	42456209	42457000	792	42456651	18	7.1522	3.73811
Nc-1-IP_pe	chr1	42631351	42631731	381	42631421	10	4.98602	3.60892
Nc-1-IP_pe	chr1	42646683	42646947	265	42646730	8	3.74505	3.07688
Nc-1-IP_pe	chr1	42658466	42659156	691	42658567	20	5.34663	2.81336
Nc-1-IP_pe	chr1	42670710	42670970	261	42670773	9	4.46937	3.41876
Nc-1-IP_pe	chr1	42672977	42673274	298	42673054	12	5.84943	3.84431
Nc-1-IP_pe	chr1	42682511	42682788	278	42682561	15	6.78905	3.93887
Nc-1-IP_pe	chr1	42735676	42736692	1017	42736528	15	6.39297	3.73508
Nc-1-IP_pe	chr1	42739803	42740342	540	42739949	11	3.64839	2.72584
Nc-1-IP_pe	chr1	42742509	42742887	379	42742737	13	6.15968	3.87845
Nc-1-IP_pe	chr1	42744827	42745162	336	42744935	13	5.93256	3.75268
Nc-1-IP_pe	chr1	42746556	42746968	413	42746828	13	5.36853	3.44651
Nc-1-IP_pe	chr1	42750829	42751117	289	42751025	14	4.21449	2.76599
Nc-1-IP_pe	chr1	42752249	42752691	443	42752253	13	3.35769	2.42919
Nc-1-IP_pe	chr1	42754060	42754339	280	42754169	11	4.06403	2.95415
Nc-1-IP_pe	chr1	42766338	42766632	295	42766557	17	4.78101	2.79343
Nc-1-IP_pe	chr1	42767258	42767512	255	42767357	15	4.06016	2.62146
Nc-1-IP_pe	chr1	42784179	42784492	314	42784311	11	5.68685	3.89286
Nc-1-IP_pe	chr1	42785001	42785777	777	42785109	12	6.12485	4.004
Nc-1-IP_pe	chr1	42786456	42786820	365	42786637	11	3.64839	2.72584
Nc-1-IP_pe	chr1	42792919	42793176	258	42792983	12	5.84943	3.84431
Nc-1-IP_pe	chr1	42851234	42851785	552	42851286	13	5.9487	3.76157
Nc-1-IP_pe	chr1	42859374	42859934	561	42859812	14	5.69896	3.50164
Nc-1-IP_pe	chr1	42864226	42864517	292	42864428	10	5.23357	3.76063
Nc-1-IP_pe	chr1	42879990	42880463	474	42880300	11	6.03439	4.10251
Nc-1-IP_pe	chr1	42880960	42881290	331	42881172	9	4.46937	3.41876
Nc-1-IP_pe	chr1	42886880	42887160	281	42886981	12	3.47963	2.55766
Nc-1-IP_pe	chr1	42887462	42887895	434	42887715	12	5.23082	3.49289
Nc-1-IP_pe	chr1	42917191	42917482	292	42917352	16	9.74731	5.37917
Nc-1-IP_pe	chr1	42919024	42919320	297	42919134	10	4.39571	3.25288
Nc-1-IP_pe	chr1	42927633	42928362	730	42927966	13	5.45659	3.49372
Nc-1-IP_pe	chr1	42930839	42931144	306	42930892	14	5.03085	3.16286
Nc-1-IP_pe	chr1	42932542	42932861	320	42932661	15	4.7819	2.95039
Nc-1-IP_pe	chr1	42936988	42937471	484	42937235	12	3.47963	2.55766
Nc-1-IP_pe	chr1	42938669	42939060	392	42938986	13	4.30582	2.8945
Nc-1-IP_pe	chr1	42939733	42940013	281	42939885	15	5.58327	3.33181
Nc-1-IP_pe	chr1	42944823	42945124	302	42944947	12	3.83688	2.74114
Nc-1-IP_pe	chr1	42952649	42953296	648	42952715	11	4.54404	3.2242
Nc-1-IP_pe	chr1	42962424	42962809	386	42962755	12	5.25962	3.50902
Nc-1-IP_pe	chr1	42966031	42966602	572	42966434	13	7.08702	4.40581

Nc-1-IP_pe	chr1	42971696	42971970	275	42971847	11	6.02981	4.09973
Nc-1-IP_pe	chr1	42974849	42975581	733	42975301	16	10.5028	5.81189
Nc-1-IP_pe	chr1	42975985	42976323	339	42976194	7	3.02492	2.70916
Nc-1-IP_pe	chr1	42978572	42978992	421	42978627	12	4.24064	2.95299
Nc-1-IP_pe	chr1	42997396	42997704	309	42997436	10	4.91962	3.56846
Nc-1-IP_pe	chr1	42998790	42999198	409	42999041	10	5.15807	3.71423
Nc-1-IP_pe	chr1	43007359	43007762	404	43007620	12	2.87625	2.25568
Nc-1-IP_pe	chr1	43010634	43010913	280	43010829	14	7.28181	4.3535
Nc-1-IP_pe	chr1	43028997	43029609	613	43029284	18	9.94172	5.10499
Nc-1-IP_pe	chr1	43037736	43038163	428	43037820	12	6.35031	4.1361
Nc-1-IP_pe	chr1	43039250	43039618	369	43039455	15	8.84797	5.06275
Nc-1-IP_pe	chr1	43055827	43056121	295	43055947	16	6.33285	3.58457
Nc-1-IP_pe	chr1	43065149	43065436	288	43065275	13	5.36853	3.44651
Nc-1-IP_pe	chr1	43067671	43068161	491	43067774	10	4.47495	3.30017
Nc-1-IP_pe	chr1	43068924	43069178	255	43068985	9	3.71705	2.9542
Nc-1-IP_pe	chr1	43069496	43070094	599	43069626	13	3.07238	2.29378
Nc-1-IP_pe	chr1	43079543	43080017	475	43079944	12	3.83688	2.74114
Nc-1-IP_pe	chr1	43102532	43102828	297	43102629	10	4.47495	3.30017
Nc-1-IP_pe	chr1	43107743	43108061	319	43107918	13	5.9487	3.76157
Nc-1-IP_pe	chr1	43120071	43120397	327	43120242	17	5.14803	2.94914
Nc-1-IP_pe	chr1	43144647	43145125	479	43144778	14	6.44441	3.89444
Nc-1-IP_pe	chr1	43152317	43152708	392	43152609	10	3.83201	2.92147
Nc-1-IP_pe	chr1	43166352	43166742	391	43166486	11	6.03439	4.10251
Nc-1-IP_pe	chr1	43171828	43172236	409	43171876	13	3.67377	2.58159
Nc-1-IP_pe	chr1	43180619	43181206	588	43181063	13	6.54342	4.09407
Nc-1-IP_pe	chr1	43201557	43201844	288	43201610	10	4.65155	3.40613
Nc-1-IP_pe	chr1	43213907	43214197	291	43214034	13	7.7352	4.78626
Nc-1-IP_pe	chr1	43215426	43215691	266	43215524	13	5.9487	3.76157
Nc-1-IP_pe	chr1	43228941	43229225	285	43229140	10	3.83201	2.92147
Nc-1-IP_pe	chr1	43238305	43238870	566	43238617	14	6.44441	3.89444
Nc-1-IP_pe	chr1	43257107	43257448	342	43257357	14	7.6052	4.5355
Nc-1-IP_pe	chr1	43259168	43259621	454	43259271	10	4.93609	3.57849
Nc-1-IP_pe	chr1	43261042	43261712	671	43261675	9	4.46937	3.41876
Nc-1-IP_pe	chr1	43284952	43285234	283	43285169	13	6.01225	3.79665
Nc-1-IP_pe	chr1	43286237	43286602	366	43286439	13	3.35769	2.42919
Nc-1-IP_pe	chr1	43300800	43301094	295	43300948	14	6.78215	4.0774
Nc-1-IP_pe	chr1	43304828	43305280	453	43305128	12	4.31492	2.99249
Nc-1-IP_pe	chr1	43306918	43307187	270	43307055	14	5.7264	3.51582
Nc-1-IP_pe	chr1	43311634	43312452	819	43312099	12	4.37125	3.02256
Nc-1-IP_pe	chr1	43318003	43318448	446	43318232	13	4.86362	3.18015
Nc-1-IP_pe	chr1	43319828	43320105	278	43319936	14	5.69896	3.50164
Nc-1-IP_pe	chr1	43321633	43321895	263	43321822	18	9.94172	5.10499
Nc-1-IP_pe	chr1	43337717	43338007	291	43337828	12	5.23082	3.49289
Nc-1-IP_pe	chr1	43360108	43360500	393	43360368	12	4.87193	3.29389
Nc-1-IP_pe	chr1	43366561	43367073	513	43366950	13	3.44439	2.47074

Nc-1-IP_pe	chr1	43389875	43390128	254	43389958	20	7.43645	3.64378	
Nc-1-IP_pe	chr1	43399490	43400101	612	43399574	11	5.77901	3.9482	
Nc-1-IP_pe	chr1	43432102	43432684	583	43432270	11	4.25472	3.06062	
Nc-1-IP_pe	chr1	43438906	43439204	299	43438988	16	6.8987	3.86161	
Nc-1-IP_pe	chr1	43439668	43440273	606	43440123	13	5.83893	3.70124	
Nc-1-IP_pe	chr1	43450724	43450989	266	43450863	16	6.62772	3.72788	
Nc-1-IP_pe	chr1	43453300	43453697	398	43453434	14	4.8399	3.06836	
Nc-1-IP_pe	chr1	43471117	43471440	324	43471224	13	4.86362	3.18015	
Nc-1-IP_pe	chr1	43482763	43483033	271	43482868	9	4.46937	3.41876	
Nc-1-IP_pe	chr1	43501426	43501682	257	43501593	12	3.47963	2.55766	
Nc-1-IP_pe	chr1	43521034	43521315	282	43521116	10	3.89064	2.95552	
Nc-1-IP_pe	chr1	43522719	43522983	265	43522840	15	6.85973	3.97568	
Nc-1-IP_pe	chr1	43526603	43527306	704	43527149	17	8.08272	4.31512	
Nc-1-IP_pe	chr1	43543814	43544069	256	43543819	11	3.70534	2.75682	
Nc-1-IP_pe	chr1	43545031	43545314	284	43545183	12	4.08831	2.8725	
Nc-1-IP_pe	chr1	43546326	43546609	284	43546557	13	4.67336	3.0817	
Nc-1-IP_pe	chr1	43548868	43549161	294	43548889	15	4.7819	2.95039	
Nc-1-IP_pe	chr1	43551019	43551468	450	43551389	12	4.11973	2.88905	
Nc-1-IP_pe	chr1	43553953	43554254	302	43554160	13	4.67336	3.0817	
Nc-1-IP_pe	chr1	43561126	43561560	435	43561419	13	4.45253	2.96876	
Nc-1-IP_pe	chr1	43571490	43571824	335	43571679	13	4.86362	3.18015	
Nc-1-IP_pe	chr1	43574737	43575119	383	43574822	12	5.11638	3.42903	
Nc-1-IP_pe	chr1	43575480	43576230	751	43575553	14	6.54309	3.94758	
Nc-1-IP_pe	chr1	43577165	43578408	1244	43578278	17	8.82358	4.69439	
Nc-1-IP_pe	chr1	43591157	43591860	704	43591619	15	6.02372	3.54894	
Nc-1-IP_pe	chr1	43592376	43592876	501	43592643	16	6.92507	3.87472	
Nc-1-IP_pe	chr1	43595793	43596161	369	43595797	13	3.67377	2.58159	
Nc-1-IP_pe	chr1	43597578	43597900	323	43597873	12	4.23763	2.9514	
Nc-1-IP_pe	chr1	43603629	43604255	627	43603805	15	5.66913	3.37372	
Nc-1-IP_pe	chr1	43605227	43605697	471	43605290	13	5.06993	3.28809	
Nc-1-IP_pe	chr1	43608514	43608949	436	43608713	18	7.1522	3.73811	
Nc-1-IP_pe	chr1	43609970	43610223	254	43610102	11	4.20699	3.03387	
Nc-1-IP_pe	chr1	43611080	43611359	280	43611175	16	7.94118	4.39398	
Nc-1-IP_pe	chr1	43611700	43612031	332	43611882	17	7.6273	4.08876	
Nc-1-IP_pe	chr1	43617164	43618044	881	43617517	16	7.40774	4.11805	
Nc-1-IP_pe	chr1	43640856	43641165	310	43641068	16	5.82808	3.34463	
Nc-1-IP_pe	chr1	43644040	43644384	345	43644207	10	3.89064	2.95552	
Nc-1-IP_pe	chr1	43647517	43648048	532	43647581	13	5.36853	3.44651	
Nc-1-IP_pe	chr1	43667570	43668323	754	43668198	14	6.28469	3.80898	
Nc-1-IP_pe	chr1	43688743	43689030	288	43688824	9	3.72192	2.95716	
Nc-1-IP_pe	chr1	43704431	43704695	265	43704577	12	5.67619	3.74486	
Nc-1-IP_pe	chr1	43715351	43715635	285	43715476	12	6.86906	4.44438	
Nc-1-IP_pe	chr1	43725327	43725599	273	43725482	14	6.69566	4.03025	
Nc-1-IP_pe	chr1	43771544	43772300	757	43771597	14	5.51541	3.4073	
Nc-1-IP_pe	chr1	43778728	43779214	487	43778848	10	3.89064	2.95552	

Nc-1-IP_pe	chr1	43785429	43785889	461	43785777	15	9.35725	5.35563
Nc-1-IP_pe	chr1	43803048	43803324	277	43803232	14	7.54286	4.50023
Nc-1-IP_pe	chr1	43811374	43811638	265	43811376	8	3.08755	2.66145
Nc-1-IP_pe	chr1	43833731	43834163	433	43833974	13	6.98489	4.34671
Nc-1-IP_pe	chr1	43850335	43850590	256	43850407	10	3.08907	2.49869
Nc-1-IP_pe	chr1	43860004	43860267	264	43860057	12	4.39405	3.03475
Nc-1-IP_pe	chr1	43866869	43867134	266	43867052	12	5.23082	3.49289
Nc-1-IP_pe	chr1	43881595	43882054	460	43881901	13	4.45253	2.96876
Nc-1-IP_pe	chr1	43885351	43885646	296	43885466	16	8.37012	4.62105
Nc-1-IP_pe	chr1	43893141	43893547	407	43893428	13	5.9487	3.76157
Nc-1-IP_pe	chr1	43898309	43899151	843	43898511	13	5.71736	3.63481
Nc-1-IP_pe	chr1	43912673	43912993	321	43912882	11	3.28475	2.53029
Nc-1-IP_pe	chr1	43931661	43931976	316	43931736	12	3.83688	2.74114
Nc-1-IP_pe	chr1	43932517	43932867	351	43932665	15	6.30504	3.69041
Nc-1-IP_pe	chr1	43936663	43937105	443	43936731	13	5.38711	3.45645
Nc-1-IP_pe	chr1	43947214	43947812	599	43947221	16	3.78851	2.44164
Nc-1-IP_pe	chr1	43956435	43956957	523	43956633	13	6.67312	4.1678
Nc-1-IP_pe	chr1	43965929	43966361	433	43966101	12	4.24064	2.95299
Nc-1-IP_pe	chr1	43970911	43971176	266	43971050	16	6.54198	3.68597
Nc-1-IP_pe	chr1	43971459	43971746	288	43971566	11	3.3469	2.56344
Nc-1-IP_pe	chr1	43976135	43976502	368	43976393	17	6.47874	3.54137
Nc-1-IP_pe	chr1	43979687	43980455	769	43980365	14	4.42328	2.86573
Nc-1-IP_pe	chr1	43981459	43981858	400	43981760	14	4.60011	2.95114
Nc-1-IP_pe	chr1	43983783	43984078	296	43983927	16	7.44163	4.13536
Nc-1-IP_pe	chr1	43985749	43986210	462	43986043	15	7.2695	4.19169
Nc-1-IP_pe	chr1	43994097	43994356	260	43994189	14	5.85216	3.5811
Nc-1-IP_pe	chr1	43995175	43995456	282	43995312	12	3.47963	2.55766
Nc-1-IP_pe	chr1	43997180	43997436	257	43997290	12	4.63088	3.16234
Nc-1-IP_pe	chr1	44005004	44005607	604	44005079	12	4.24064	2.95299
Nc-1-IP_pe	chr1	44005968	44006348	381	44006135	10	3.86691	2.94172
Nc-1-IP_pe	chr1	44008315	44008617	303	44008453	19	9.15219	4.54307
Nc-1-IP_pe	chr1	44013160	44013558	399	44013238	13	4.41954	2.952
Nc-1-IP_pe	chr1	44018070	44018431	362	44018387	11	4.21886	3.04051
Nc-1-IP_pe	chr1	44020874	44021138	265	44021036	15	5.66913	3.37372
Nc-1-IP_pe	chr1	44029164	44029998	835	44029903	15	3.74763	2.48304
Nc-1-IP_pe	chr1	44033875	44034313	439	44033996	12	3.83688	2.74114
Nc-1-IP_pe	chr1	44044383	44044655	273	44044452	14	7.016	4.20583
Nc-1-IP_pe	chr1	44054250	44054572	323	44054508	10	4.33501	3.21677
Nc-1-IP_pe	chr1	44071575	44071872	298	44071584	8	3.43415	2.87929
Nc-1-IP_pe	chr1	44072348	44072755	408	44072599	12	6.16157	4.02543
Nc-1-IP_pe	chr1	44080084	44080382	299	44080208	11	6.03439	4.10251
Nc-1-IP_pe	chr1	44086036	44086366	331	44086185	12	6.71369	4.35145
Nc-1-IP_pe	chr1	44103400	44103672	273	44103551	18	7.73136	4.00629
Nc-1-IP_pe	chr1	44104292	44104646	355	44104487	13	7.34205	4.55444
Nc-1-IP_pe	chr1	44118795	44119303	509	44118998	13	7.43036	4.60624

Nc-1-IP_pe	chr1	44130730	44131276	547	44130980	11	4.06403	2.95415
Nc-1-IP_pe	chr1	44139696	44140072	377	44139753	11	3.28475	2.53029
Nc-1-IP_pe	chr1	44146671	44147077	407	44146911	11	3.64839	2.72584
Nc-1-IP_pe	chr1	44163168	44163644	477	44163356	10	4.99888	3.61677
Nc-1-IP_pe	chr1	44190192	44190448	257	44190349	10	3.89064	2.95552
Nc-1-IP_pe	chr1	44212529	44212973	445	44212666	8	3.56021	2.95918
Nc-1-IP_pe	chr1	44213271	44213941	671	44213514	19	12.7767	6.43399
Nc-1-IP_pe	chr1	44219856	44220302	447	44220175	15	4.06016	2.62146
Nc-1-IP_pe	chr1	44221384	44221890	507	44221451	12	5.28895	3.52546
Nc-1-IP_pe	chr1	44222704	44223494	791	44223122	13	5.82354	3.69281
Nc-1-IP_pe	chr1	44238222	44238563	342	44238337	13	5.71736	3.63481
Nc-1-IP_pe	chr1	44239247	44240050	804	44239472	13	5.36853	3.44651
Nc-1-IP_pe	chr1	44248085	44248739	655	44248143	10	4.39571	3.25288
Nc-1-IP_pe	chr1	44249513	44249830	318	44249768	9	3.76709	2.98468
Nc-1-IP_pe	chr1	44250226	44250661	436	44250433	12	6.10662	3.99337
Nc-1-IP_pe	chr1	44252126	44252594	469	44252457	12	5.77527	3.80164
Nc-1-IP_pe	chr1	44256482	44256750	269	44256551	9	3.02445	2.53932
Nc-1-IP_pe	chr1	44282782	44283147	366	44282795	14	4.60011	2.95114
Nc-1-IP_pe	chr1	44284577	44284836	260	44284778	11	4.88957	3.42271
Nc-1-IP_pe	chr1	44289694	44290086	393	44289809	12	3.47963	2.55766
Nc-1-IP_pe	chr1	44292656	44292911	256	44292849	10	4.56202	3.35231
Nc-1-IP_pe	chr1	44295861	44296381	521	44296004	12	5.94619	3.9002
Nc-1-IP_pe	chr1	44296707	44297011	305	44296774	11	5.25789	3.63791
Nc-1-IP_pe	chr1	44308146	44308663	518	44308201	11	4.54404	3.2242
Nc-1-IP_pe	chr1	44335117	44335549	433	44335217	11	5.5792	3.82847
Nc-1-IP_pe	chr1	44354946	44355389	444	44355246	16	6.8987	3.86161
Nc-1-IP_pe	chr1	44368135	44368887	753	44368176	12	5.65992	3.73556
Nc-1-IP_pe	chr1	44405365	44406191	827	44405484	14	4.21449	2.76599
Nc-1-IP_pe	chr1	44407408	44407910	503	44407506	16	7.53857	4.18505
Nc-1-IP_pe	chr1	44417510	44417792	283	44417650	11	4.35236	3.11555
Nc-1-IP_pe	chr1	44426366	44426710	345	44426526	12	6.86906	4.44438
Nc-1-IP_pe	chr1	44462817	44463223	407	44462995	15	9.55381	5.47001
Nc-1-IP_pe	chr1	44472814	44473114	301	44473026	10	3.4604	2.70797
Nc-1-IP_pe	chr1	44493102	44493379	278	44493259	11	6.03439	4.10251
Nc-1-IP_pe	chr1	44501440	44501770	331	44501731	10	3.89064	2.95552
Nc-1-IP_pe	chr1	44523233	44524092	860	44523306	13	4.86362	3.18015
Nc-1-IP_pe	chr1	44532911	44533433	523	44532966	11	3.78682	2.80131
Nc-1-IP_pe	chr1	44543957	44544304	348	44544015	15	5.4619	3.27292
Nc-1-IP_pe	chr1	44546394	44546698	305	44546672	11	3.68532	2.74592
Nc-1-IP_pe	chr1	44550883	44551332	450	44551216	16	7.83	4.33588
Nc-1-IP_pe	chr1	44562128	44562404	277	44562357	13	6.10995	3.8508
Nc-1-IP_pe	chr1	44565658	44566537	880	44566495	13	5.71736	3.63481
Nc-1-IP_pe	chr1	44581679	44581936	258	44581713	9	3.54889	2.85226
Nc-1-IP_pe	chr1	44582280	44582537	258	44582374	12	5.45448	3.61875
Nc-1-IP_pe	chr1	44587284	44587676	393	44587439	12	5.23082	3.49289

Nc-1-IP_pe	chr1	44591342	44592437	1096	44592348	13	4.02562	2.7544
Nc-1-IP_pe	chr1	44596208	44596511	304	44596373	13	6.36468	3.99316
Nc-1-IP_pe	chr1	44607878	44608215	338	44608018	10	3.83201	2.92147
Nc-1-IP_pe	chr1	44609391	44610655	1265	44609572	12	4.24064	2.95299
Nc-1-IP_pe	chr1	44616776	44617750	975	44617302	19	10.4598	5.19259
Nc-1-IP_pe	chr1	44619785	44620052	268	44619794	10	3.79754	2.90149
Nc-1-IP_pe	chr1	44625166	44625500	335	44625319	16	6.33285	3.58457
Nc-1-IP_pe	chr1	44631716	44632189	474	44631842	16	3.65575	2.38624
Nc-1-IP_pe	chr1	44635651	44635947	297	44635834	15	5.94269	3.50859
Nc-1-IP_pe	chr1	44640151	44640567	417	44640299	9	3.12089	2.59629
Nc-1-IP_pe	chr1	44645300	44645859	560	44645592	16	8.79222	4.84884
Nc-1-IP_pe	chr1	44649701	44651049	1349	44650915	13	5.36853	3.44651
Nc-1-IP_pe	chr1	44654248	44655460	1213	44655172	17	7.6885	4.11887
Nc-1-IP_pe	chr1	44658263	44658681	419	44658503	10	4.36249	3.23311
Nc-1-IP_pe	chr1	44662506	44663064	559	44662778	14	5.18557	3.24018
Nc-1-IP_pe	chr1	44706026	44706526	501	44706394	10	3.60969	2.79325
Nc-1-IP_pe	chr1	44715584	44715952	369	44715759	10	3.08907	2.49869
Nc-1-IP_pe	chr1	44723400	44724173	774	44724017	10	3.4604	2.70797
Nc-1-IP_pe	chr1	44738209	44738738	530	44738647	16	5.82808	3.34463
Nc-1-IP_pe	chr1	44744728	44745121	394	44744750	12	5.23082	3.49289
Nc-1-IP_pe	chr1	44759100	44759451	352	44759220	12	4.70082	3.20033
Nc-1-IP_pe	chr1	44764791	44765128	338	44765008	14	6.67814	4.02073
Nc-1-IP_pe	chr1	44777408	44777796	389	44777451	10	3.30648	2.62073
Nc-1-IP_pe	chr1	44784133	44784415	283	44784259	12	5.17301	3.46058
Nc-1-IP_pe	chr1	44785188	44785673	486	44785446	16	5.37454	3.13479
Nc-1-IP_pe	chr1	44786360	44786964	605	44786938	13	3.07238	2.29378
Nc-1-IP_pe	chr1	44791325	44791761	437	44791707	11	4.54404	3.2242
Nc-1-IP_pe	chr1	44800555	44800808	254	44800610	15	4.40338	2.77622
Nc-1-IP_pe	chr1	44802921	44803498	578	44803079	16	6.26689	3.55283
Nc-1-IP_pe	chr1	44805571	44807252	1682	44806741	16	6.74448	3.78526
Nc-1-IP_pe	chr1	44817977	44818234	258	44818007	10	3.54629	2.75696
Nc-1-IP_pe	chr1	44818832	44819087	256	44818947	12	4.1728	2.91706
Nc-1-IP_pe	chr1	44822339	44822633	295	44822511	14	6.94277	4.16546
Nc-1-IP_pe	chr1	44827093	44827357	265	44827158	14	5.838	3.57373
Nc-1-IP_pe	chr1	44827842	44828094	253	44827953	14	5.30793	3.30182
Nc-1-IP_pe	chr1	44829004	44829275	272	44829272	13	3.07238	2.29378
Nc-1-IP_pe	chr1	44832200	44832604	405	44832201	11	3.28475	2.53029
Nc-1-IP_pe	chr1	44833859	44834120	262	44833984	14	4.60011	2.95114
Nc-1-IP_pe	chr1	44848552	44849300	749	44849199	11	5.38972	3.71578
Nc-1-IP_pe	chr1	44850464	44850740	277	44850583	10	4.33501	3.21677
Nc-1-IP_pe	chr1	44901114	44901606	493	44901249	12	6.58417	4.27436
Nc-1-IP_pe	chr1	44925663	44926145	483	44925948	15	8.26005	4.73146
Nc-1-IP_pe	chr1	44967319	44967824	506	44967463	11	4.74697	3.34038
Nc-1-IP_pe	chr1	44986397	44986686	290	44986567	14	4.60011	2.95114
Nc-1-IP_pe	chr1	44993082	44993340	259	44993178	12	6.7561	4.37677

Nc-1-IP_pe	chr1	45003413	45003871	459	45003700	13	5.71736	3.63481
Nc-1-IP_pe	chr1	45004914	45005173	260	45005042	13	5.65792	3.60248
Nc-1-IP_pe	chr1	45013685	45014175	491	45014075	18	7.73136	4.00629
Nc-1-IP_pe	chr1	45038763	45039564	802	45039153	10	5.23357	3.76063
Nc-1-IP_pe	chr1	45061811	45062077	267	45061957	13	6.62436	4.14003
Nc-1-IP_pe	chr1	45081362	45081844	483	45081460	11	3.28475	2.53029
Nc-1-IP_pe	chr1	45120965	45121235	271	45121079	28	11.2618	4.27481
Nc-1-IP_pe	chr1	45135911	45136187	277	45136043	13	7.7352	4.78626
Nc-1-IP_pe	chr1	45159618	45159912	295	45159734	11	6.03439	4.10251
Nc-1-IP_pe	chr1	45206803	45207090	288	45206818	9	3.27326	2.68684
Nc-1-IP_pe	chr1	45218330	45218650	321	45218471	14	4.21449	2.76599
Nc-1-IP_pe	chr1	45223162	45223675	514	45223351	14	7.9745	4.74633
Nc-1-IP_pe	chr1	45224351	45224709	359	45224476	11	5.10577	3.5486
Nc-1-IP_pe	chr1	45236617	45236902	286	45236769	12	5.84943	3.84431
Nc-1-IP_pe	chr1	45247487	45247818	332	45247724	17	5.14803	2.94914
Nc-1-IP_pe	chr1	45254107	45254451	345	45254358	11	5.10577	3.5486
Nc-1-IP_pe	chr1	45269688	45269995	308	45269940	9	4.11647	3.19921
Nc-1-IP_pe	chr1	45295512	45295787	276	45295581	12	4.70082	3.20033
Nc-1-IP_pe	chr1	45296489	45296780	292	45296597	12	5.92876	3.89011
Nc-1-IP_pe	chr1	45309034	45309508	475	45309432	10	4.39571	3.25288
Nc-1-IP_pe	chr1	45331463	45331945	483	45331894	12	4.24064	2.95299
Nc-1-IP_pe	chr1	45333226	45333589	364	45333371	15	7.0945	4.09889
Nc-1-IP_pe	chr1	45339697	45340440	744	45340160	12	2.87625	2.25568
Nc-1-IP_pe	chr1	45360913	45361219	307	45361040	10	5.23357	3.76063
Nc-1-IP_pe	chr1	45382355	45382865	511	45382753	11	6.03439	4.10251
Nc-1-IP_pe	chr1	45484628	45485049	422	45484818	10	4.39571	3.25288
Nc-1-IP_pe	chr1	45508738	45509111	374	45508923	16	6.8987	3.86161
Nc-1-IP_pe	chr1	45521618	45522141	524	45522066	16	6.33285	3.58457
Nc-1-IP_pe	chr1	45548356	45548673	318	45548412	10	3.85523	2.93494
Nc-1-IP_pe	chr1	45550220	45550545	326	45550480	12	3.67829	2.65924
Nc-1-IP_pe	chr1	45556272	45556809	538	45556458	11	4.74697	3.34038
Nc-1-IP_pe	chr1	45564062	45564326	265	45564235	12	5.62759	3.7171
Nc-1-IP_pe	chr1	45568584	45568922	339	45568759	15	7.46994	4.29894
Nc-1-IP_pe	chr1	45587839	45588200	362	45588087	13	4.41954	2.952
Nc-1-IP_pe	chr1	45623139	45623399	261	45623327	9	4.1022	3.19039
Nc-1-IP_pe	chr1	45664704	45665135	432	45665021	10	3.94999	2.99009
Nc-1-IP_pe	chr1	45687996	45688440	445	45688366	14	5.03085	3.16286
Nc-1-IP_pe	chr1	45689688	45690405	718	45689829	21	7.58657	3.60451
Nc-1-IP_pe	chr1	45694950	45695293	344	45695257	11	4.06403	2.95415
Nc-1-IP_pe	chr1	45713171	45713489	319	45713212	8	3.06164	2.64527
Nc-1-IP_pe	chr1	45721993	45722415	423	45722315	9	3.79246	3.00015
Nc-1-IP_pe	chr1	45724281	45725612	1332	45724718	19	8.03556	4.02008
Nc-1-IP_pe	chr1	45731771	45732170	400	45731796	16	5.39474	3.14402
Nc-1-IP_pe	chr1	45732746	45733406	661	45733247	14	7.05305	4.22631
Nc-1-IP_pe	chr1	45734488	45734842	355	45734721	11	3.28475	2.53029

Nc-1-IP_pe	chr1	45739885	45740557	673	45740397	15	8.79961	5.03521
Nc-1-IP_pe	chr1	45748565	45749043	479	45748889	16	7.11257	3.96852
Nc-1-IP_pe	chr1	45751446	45751906	461	45751633	14	3.86727	2.6027
Nc-1-IP_pe	chr1	45765470	45765856	387	45765618	14	6.06519	3.69269
Nc-1-IP_pe	chr1	45771230	45771504	275	45771334	14	8.08868	4.81212
Nc-1-IP_pe	chr1	45776515	45777085	571	45776924	11	5.05359	3.5181
Nc-1-IP_pe	chr1	45782209	45782528	320	45782296	9	3.53727	2.84525
Nc-1-IP_pe	chr1	45803434	45804268	835	45803956	22	11.2469	5.06279
Nc-1-IP_pe	chr1	45806370	45806776	407	45806507	12	3.83688	2.74114
Nc-1-IP_pe	chr1	45809331	45809605	275	45809483	16	9.10831	5.02214
Nc-1-IP_pe	chr1	45816618	45816932	315	45816737	13	6.19316	3.89711
Nc-1-IP_pe	chr1	45836949	45837252	304	45837064	16	6.33285	3.58457
Nc-1-IP_pe	chr1	45839156	45839443	288	45839279	13	3.67377	2.58159
Nc-1-IP_pe	chr1	45873916	45874275	360	45874027	14	7.84093	4.66972
Nc-1-IP_pe	chr1	45909645	45909967	323	45909926	13	4.02562	2.7544
Nc-1-IP_pe	chr1	45922305	45922787	483	45922469	17	6.01283	3.32896
Nc-1-IP_pe	chr1	45939563	45940293	731	45939705	13	7.02546	4.37016
Nc-1-IP_pe	chr1	45947779	45948060	282	45947941	13	4.86362	3.18015
Nc-1-IP_pe	chr1	45972035	45972443	409	45972277	15	6.39297	3.73508
Nc-1-IP_pe	chr1	45994393	45994697	305	45994511	13	6.67312	4.1678
Nc-1-IP_pe	chr1	46034702	46035151	450	46034796	15	3.74763	2.48304
Nc-1-IP_pe	chr1	46058073	46058650	578	46058354	21	6.2632	3.08808
Nc-1-IP_pe	chr1	46103709	46104075	367	46103763	12	5.15877	3.45264
Nc-1-IP_pe	chr1	46106763	46107286	524	46107054	11	5.02318	3.50036
Nc-1-IP_pe	chr1	46118569	46119048	480	46118846	10	4.79071	3.49018
Nc-1-IP_pe	chr1	46124515	46124789	275	46124661	14	7.2043	4.31026
Nc-1-IP_pe	chr1	46132290	46132787	498	46132773	17	3.11196	2.12137
Nc-1-IP_pe	chr1	46173553	46173997	445	46173568	15	3.74763	2.48304
Nc-1-IP_pe	chr1	46174978	46175248	271	46175121	16	5.37454	3.13479
Nc-1-IP_pe	chr1	46176159	46176587	429	46176333	22	7.27519	3.39036
Nc-1-IP_pe	chr1	46184099	46184611	513	46184258	20	7.59756	3.71176
Nc-1-IP_pe	chr1	46185338	46186396	1059	46186181	18	6.22907	3.32777
Nc-1-IP_pe	chr1	46196828	46197282	455	46197242	17	3.58449	2.30632
Nc-1-IP_pe	chr1	46203198	46203514	317	46203318	16	6.90977	3.86711
Nc-1-IP_pe	chr1	46203795	46204561	767	46204079	13	4.67336	3.0817
Nc-1-IP_pe	chr1	46207479	46207895	417	46207796	13	3.07238	2.29378
Nc-1-IP_pe	chr1	46218722	46219063	342	46218771	12	4.24064	2.95299
Nc-1-IP_pe	chr1	46223172	46223485	314	46223315	15	6.73062	3.90854
Nc-1-IP_pe	chr1	46229276	46229639	364	46229440	16	5.82808	3.34463
Nc-1-IP_pe	chr1	46237698	46238048	351	46237848	13	5.83893	3.70124
Nc-1-IP_pe	chr1	46240754	46242175	1422	46242008	17	8.76298	4.66287
Nc-1-IP_pe	chr1	46247761	46248207	447	46247829	18	6.63038	3.50359
Nc-1-IP_pe	chr1	46260269	46260530	262	46260474	14	5.03085	3.16286
Nc-1-IP_pe	chr1	46263706	46264116	411	46264005	13	5.41476	3.47126
Nc-1-IP_pe	chr1	46267138	46267495	358	46267340	11	4.17167	3.01412

Nc-1-IP_pe	chr1	46273549	46273919	371	46273811	16	7.53857	4.18505
Nc-1-IP_pe	chr1	46278713	46279199	487	46278834	16	4.96462	2.94973
Nc-1-IP_pe	chr1	46285399	46286390	992	46286158	18	6.15736	3.29676
Nc-1-IP_pe	chr1	46303656	46303924	269	46303734	21	6.66991	3.24296
Nc-1-IP_pe	chr1	46312048	46312407	360	46312237	14	4.60011	2.95114
Nc-1-IP_pe	chr1	46319641	46320083	443	46319985	12	4.66786	3.1824
Nc-1-IP_pe	chr1	46340796	46341048	253	46341018	16	3.93641	2.50379
Nc-1-IP_pe	chr1	46343931	46344231	301	46344162	15	5.42616	3.25565
Nc-1-IP_pe	chr1	46344539	46344827	289	46344665	17	6.47874	3.54137
Nc-1-IP_pe	chr1	46347021	46347379	359	46347233	15	5.20142	3.14789
Nc-1-IP_pe	chr1	46347902	46348493	592	46348071	16	5.37454	3.13479
Nc-1-IP_pe	chr1	46349359	46349650	292	46349504	12	3.83688	2.74114
Nc-1-IP_pe	chr1	46361649	46362003	355	46361948	9	3.90961	3.07183
Nc-1-IP_pe	chr1	46388902	46389422	521	46389001	13	5.86991	3.71823
Nc-1-IP_pe	chr1	46394059	46394365	307	46394155	19	5.90435	3.10381
Nc-1-IP_pe	chr1	46395505	46395828	324	46395648	15	5.82387	3.44976
Nc-1-IP_pe	chr1	46400221	46400493	273	46400325	14	4.8731	3.08472
Nc-1-IP_pe	chr1	46401832	46402359	528	46402113	17	3.33947	2.20998
Nc-1-IP_pe	chr1	46405615	46406570	956	46406425	21	7.58657	3.60451
Nc-1-IP_pe	chr1	46410512	46410810	299	46410675	16	4.25252	2.63824
Nc-1-IP_pe	chr1	46414259	46414858	600	46414724	17	7.02067	3.79543
Nc-1-IP_pe	chr1	46425107	46425560	454	46425453	12	4.743	3.22332
Nc-1-IP_pe	chr1	46438195	46438488	294	46438425	15	5.4619	3.27292
Nc-1-IP_pe	chr1	46440380	46440816	437	46440714	13	4.68476	3.08757
Nc-1-IP_pe	chr1	46448471	46449191	721	46448622	17	8.10053	4.32408
Nc-1-IP_pe	chr1	46449615	46450075	461	46449771	15	4.7819	2.95039
Nc-1-IP_pe	chr1	46457935	46458815	881	46458027	16	5.37454	3.13479
Nc-1-IP_pe	chr1	46459511	46459771	261	46459536	14	3.86727	2.6027
Nc-1-IP_pe	chr1	46466999	46467677	679	46467480	15	5.20142	3.14789
Nc-1-IP_pe	chr1	46469661	46469972	312	46469888	14	5.80982	3.55907
Nc-1-IP_pe	chr1	46474640	46474953	314	46474868	15	6.19428	3.63445
Nc-1-IP_pe	chr1	46475558	46475916	359	46475702	14	5.03085	3.16286
Nc-1-IP_pe	chr1	46480040	46480353	314	46480145	13	5.49888	3.51647
Nc-1-IP_pe	chr1	46481085	46481405	321	46481303	12	3.83688	2.74114
Nc-1-IP_pe	chr1	46483985	46485203	1219	46484135	13	5.79292	3.67605
Nc-1-IP_pe	chr1	46487677	46488172	496	46487758	14	3.55308	2.45762
Nc-1-IP_pe	chr1	46488450	46488895	446	46488864	14	3.55308	2.45762
Nc-1-IP_pe	chr1	46489940	46491233	1294	46490578	19	5.51632	2.94814
Nc-1-IP_pe	chr1	46493216	46494204	989	46493986	18	8.10208	4.1823
Nc-1-IP_pe	chr1	46494659	46494974	316	46494861	16	6.8987	3.86161
Nc-1-IP_pe	chr1	46496741	46496997	257	46496766	11	3.64839	2.72584
Nc-1-IP_pe	chr1	46499030	46499329	300	46499159	14	6.22239	3.77584
Nc-1-IP_pe	chr1	46506888	46507314	427	46507079	18	7.1522	3.73811
Nc-1-IP_pe	chr1	46508043	46509011	969	46508744	15	6.69889	3.8921
Nc-1-IP_pe	chr1	46510315	46511193	879	46510627	19	9.22155	4.57653

Nc-1-IP_pe	chr1	46519497	46520213	717	46519909	20	6.49719	3.259
Nc-1-IP_pe	chr1	46522690	46523162	473	46523127	17	6.10838	3.37208
Nc-1-IP_pe	chr1	46531786	46532759	974	46532505	17	5.54967	3.12324
Nc-1-IP_pe	chr1	46533353	46533785	433	46533611	20	6.94658	3.44067
Nc-1-IP_pe	chr1	46539984	46540577	594	46540343	15	5.22925	3.16116
Nc-1-IP_pe	chr1	46543669	46544371	703	46543765	15	5.90272	3.48876
Nc-1-IP_pe	chr1	46547452	46547876	425	46547731	17	5.14803	2.94914
Nc-1-IP_pe	chr1	46548597	46548922	326	46548867	17	5.14803	2.94914
Nc-1-IP_pe	chr1	46552356	46552658	303	46552464	15	4.32392	2.74012
Nc-1-IP_pe	chr1	46554872	46555370	499	46554930	16	4.59224	2.7853
Nc-1-IP_pe	chr1	46558527	46558910	384	46558831	13	4.9575	3.22911
Nc-1-IP_pe	chr1	46561304	46561563	260	46561458	16	6.8987	3.86161
Nc-1-IP_pe	chr1	46569670	46569974	305	46569860	15	5.66913	3.37372
Nc-1-IP_pe	chr1	46571794	46572062	269	46571904	17	7.02067	3.79543
Nc-1-IP_pe	chr1	46585026	46585629	604	46585565	13	4.41954	2.952
Nc-1-IP_pe	chr1	46587281	46587676	396	46587445	15	7.62034	4.38009
Nc-1-IP_pe	chr1	46592393	46592659	267	46592524	12	3.83688	2.74114
Nc-1-IP_pe	chr1	46599275	46599554	280	46599357	11	3.89191	2.85898
Nc-1-IP_pe	chr1	46607729	46608058	330	46608010	14	4.21449	2.76599
Nc-1-IP_pe	chr1	46612884	46613198	315	46613095	21	5.2077	2.70109
Nc-1-IP_pe	chr1	46615024	46615388	365	46615216	17	6.47874	3.54137
Nc-1-IP_pe	chr1	46615805	46616276	472	46616158	14	3.86727	2.6027
Nc-1-IP_pe	chr1	46616578	46616835	258	46616722	16	5.37454	3.13479
Nc-1-IP_pe	chr1	46621635	46621949	315	46621807	12	4.45173	3.06567
Nc-1-IP_pe	chr1	46623399	46623670	272	46623564	15	6.62049	3.85162
Nc-1-IP_pe	chr1	46629679	46629992	314	46629746	15	6.85973	3.97568
Nc-1-IP_pe	chr1	46667852	46668555	704	46668462	19	6.92557	3.52962
Nc-1-IP_pe	chr1	46717905	46718519	615	46718228	17	6.78076	3.68203
Nc-1-IP_pe	chr1	46718787	46719042	256	46718917	24	5.77667	2.7276
Nc-1-IP_pe	chr1	46748479	46748993	515	46748600	14	4.95172	3.12357
Nc-1-IP_pe	chr1	46755984	46756304	321	46756117	17	6.47874	3.54137
Nc-1-IP_pe	chr1	46769473	46769766	294	46769498	12	4.39405	3.03475
Nc-1-IP_pe	chr1	46778422	46778982	561	46778658	16	6.8987	3.86161
Nc-1-IP_pe	chr1	46790590	46790898	309	46790836	12	5.92876	3.89011
Nc-1-IP_pe	chr1	46813334	46813625	292	46813562	11	3.89191	2.85898
Nc-1-IP_pe	chr1	46814100	46814480	381	46814308	15	6.39297	3.73508
Nc-1-IP_pe	chr1	46819458	46819730	273	46819551	14	5.51541	3.4073
Nc-1-IP_pe	chr1	46846196	46846605	410	46846451	17	4.44438	2.65333
Nc-1-IP_pe	chr1	46861720	46861989	270	46861816	16	5.37454	3.13479
Nc-1-IP_pe	chr1	46884068	46884447	380	46884190	12	5.65992	3.73556
Nc-1-IP_pe	chr1	46929899	46930322	424	46930227	12	4.70082	3.20033
Nc-1-IP_pe	chr1	46938932	46939204	273	46939006	9	3.56055	2.85931
Nc-1-IP_pe	chr1	46950447	46950773	327	46950595	17	9.09431	4.8363
Nc-1-IP_pe	chr1	47075720	47075973	254	47075796	12	5.84943	3.84431
Nc-1-IP_pe	chr1	47096817	47097090	274	47097009	10	5.23357	3.76063

Nc-1-IP_pe	chr1	47104325	47104680	356	47104585	13	3.67377	2.58159
Nc-1-IP_pe	chr1	47137430	47137695	266	47137569	17	7.02067	3.79543
Nc-1-IP_pe	chr1	47138479	47138948	470	47138829	16	6.65665	3.74206
Nc-1-IP_pe	chr1	47142188	47142663	476	47142621	14	5.78183	3.54454
Nc-1-IP_pe	chr1	47148593	47149450	858	47149211	17	8.20861	4.37862
Nc-1-IP_pe	chr1	47153728	47154001	274	47153911	12	4.24064	2.95299
Nc-1-IP_pe	chr1	47166229	47166756	528	47166330	11	5.49185	3.77641
Nc-1-IP_pe	chr1	47174648	47174973	326	47174843	17	7.6273	4.08876
Nc-1-IP_pe	chr1	47178813	47179593	781	47179370	17	5.9911	3.31919
Nc-1-IP_pe	chr1	47180852	47182390	1539	47181316	20	6.49719	3.259
Nc-1-IP_pe	chr1	47183765	47184298	534	47184221	16	4.25252	2.63824
Nc-1-IP_pe	chr1	47186803	47187107	305	47186917	15	4.40338	2.77622
Nc-1-IP_pe	chr1	47187840	47188590	751	47187868	13	4.69621	3.09346
Nc-1-IP_pe	chr1	47190091	47190722	632	47190668	15	4.7819	2.95039
Nc-1-IP_pe	chr1	47192970	47193255	286	47193088	16	4.25252	2.63824
Nc-1-IP_pe	chr1	47196644	47196919	276	47196706	21	11.2252	5.21997
Nc-1-IP_pe	chr1	47212926	47214025	1100	47213700	17	7.02067	3.79543
Nc-1-IP_pe	chr1	47217181	47217556	376	47217463	17	4.78101	2.79343
Nc-1-IP_pe	chr1	47224874	47225706	833	47225219	16	7.17668	4.0008
Nc-1-IP_pe	chr1	47229177	47229882	706	47229816	9	3.54889	2.85226
Nc-1-IP_pe	chr1	47231578	47232729	1152	47232588	13	4.86362	3.18015
Nc-1-IP_pe	chr1	47241016	47241315	300	47241171	14	5.51541	3.4073
Nc-1-IP_pe	chr1	47275364	47275827	464	47275700	12	4.70082	3.20033
Nc-1-IP_pe	chr1	47285598	47286046	449	47285801	10	5.23357	3.76063
Nc-1-IP_pe	chr1	47288314	47288566	253	47288444	14	7.54286	4.50023
Nc-1-IP_pe	chr1	47295062	47295571	510	47295236	18	8.37884	4.31591
Nc-1-IP_pe	chr1	47298818	47299481	664	47299289	10	4.79071	3.49018
Nc-1-IP_pe	chr1	47306970	47307323	354	47307087	10	4.62142	3.388
Nc-1-IP_pe	chr1	47311848	47312416	569	47311996	14	6.06519	3.69269
Nc-1-IP_pe	chr1	47313985	47314240	256	47314049	16	5.73763	3.30235
Nc-1-IP_pe	chr1	47319515	47320249	735	47319995	12	4.70082	3.20033
Nc-1-IP_pe	chr1	47329570	47330020	451	47329831	16	4.96462	2.94973
Nc-1-IP_pe	chr1	47341860	47342147	288	47342007	13	5.9487	3.76157
Nc-1-IP_pe	chr1	47345936	47346213	278	47345979	14	5.69896	3.50164
Nc-1-IP_pe	chr1	47346583	47347000	418	47346732	15	5.20142	3.14789
Nc-1-IP_pe	chr1	47363484	47363815	332	47363629	10	3.08907	2.49869
Nc-1-IP_pe	chr1	47388885	47389152	268	47388990	16	4.59224	2.7853
Nc-1-IP_pe	chr1	47391362	47391630	269	47391573	13	3.77558	2.63125
Nc-1-IP_pe	chr1	47405816	47406264	449	47406179	14	5.51541	3.4073
Nc-1-IP_pe	chr1	47412524	47412802	279	47412623	13	4.73076	3.11129
Nc-1-IP_pe	chr1	47429966	47430288	323	47430189	13	4.81267	3.15368
Nc-1-IP_pe	chr1	47431425	47432039	615	47431914	16	6.70037	3.76354
Nc-1-IP_pe	chr1	47432487	47432790	304	47432670	20	7.43645	3.64378
Nc-1-IP_pe	chr1	47439009	47439444	436	47439320	15	4.7819	2.95039
Nc-1-IP_pe	chr1	47440549	47440915	367	47440727	14	4.7852	3.04148

Nc-1-IP_pe	chr1	47443782	47444209	428	47444067	12	4.65549	3.17569
Nc-1-IP_pe	chr1	47456124	47456395	272	47456286	15	7.65877	4.40091
Nc-1-IP_pe	chr1	47459098	47459374	277	47459231	14	5.03085	3.16286
Nc-1-IP_pe	chr1	47466163	47466675	513	47466264	17	6.51104	3.5563
Nc-1-IP_pe	chr1	47468619	47469192	574	47468694	14	3.86727	2.6027
Nc-1-IP_pe	chr1	47469859	47470426	568	47470040	19	8.19109	4.09114
Nc-1-IP_pe	chr1	47490196	47490516	321	47490484	12	4.16211	2.91142
Nc-1-IP_pe	chr1	47492185	47492456	272	47492211	12	4.63088	3.16234
Nc-1-IP_pe	chr1	47507780	47509426	1647	47507899	16	7.27441	4.05022
Nc-1-IP_pe	chr1	47533753	47534031	279	47533829	14	4.95172	3.12357
Nc-1-IP_pe	chr1	47536248	47536509	262	47536335	15	5.66913	3.37372
Nc-1-IP_pe	chr1	47537041	47537404	364	47537270	15	6.97567	4.03634
Nc-1-IP_pe	chr1	47537857	47538209	353	47537892	12	3.16137	2.39719
Nc-1-IP_pe	chr1	47554270	47554592	323	47554479	12	3.83688	2.74114
Nc-1-IP_pe	chr1	47555935	47556517	583	47556107	13	5.03208	3.26819
Nc-1-IP_pe	chr1	47560430	47560873	444	47560818	13	4.96982	3.23556
Nc-1-IP_pe	chr1	47563365	47563731	367	47563668	10	2.95833	2.42595
Nc-1-IP_pe	chr1	47566947	47567367	421	47567085	17	7.32147	3.9397
Nc-1-IP_pe	chr1	47578306	47578644	339	47578432	16	6.95583	3.89005
Nc-1-IP_pe	chr1	47579499	47580344	846	47580167	12	4.70082	3.20033
Nc-1-IP_pe	chr1	47588066	47588396	331	47588136	13	4.86362	3.18015
Nc-1-IP_pe	chr1	47602302	47603017	716	47602440	18	7.73136	4.00629
Nc-1-IP_pe	chr1	47608390	47608695	306	47608593	12	6.12485	4.004
Nc-1-IP_pe	chr1	47621323	47621805	483	47621394	12	4.24064	2.95299
Nc-1-IP_pe	chr1	47623703	47624485	783	47624389	10	3.08907	2.49869
Nc-1-IP_pe	chr1	47630451	47630727	277	47630680	12	4.24064	2.95299
Nc-1-IP_pe	chr1	47632857	47633165	309	47633010	16	7.54467	4.18818
Nc-1-IP_pe	chr1	47633725	47634014	290	47633879	18	7.1522	3.73811
Nc-1-IP_pe	chr1	47635049	47635501	453	47635392	14	5.838	3.57373
Nc-1-IP_pe	chr1	47637892	47638298	407	47638236	12	4.70082	3.20033
Nc-1-IP_pe	chr1	47640634	47641362	729	47641260	14	4.95172	3.12357
Nc-1-IP_pe	chr1	47642917	47643578	662	47642990	15	4.41451	2.78128
Nc-1-IP_pe	chr1	47647472	47647810	339	47647617	12	3.77112	2.7071
Nc-1-IP_pe	chr1	47652154	47652544	391	47652408	13	5.8082	3.68441
Nc-1-IP_pe	chr1	47656721	47657139	419	47656903	16	8.79222	4.84884
Nc-1-IP_pe	chr1	47658234	47658600	367	47658439	17	7.6273	4.08876
Nc-1-IP_pe	chr1	47664222	47664604	383	47664523	15	6.10627	3.59023
Nc-1-IP_pe	chr1	47675663	47675934	272	47675755	17	7.6885	4.11887
Nc-1-IP_pe	chr1	47680953	47681320	368	47681168	19	9.62787	4.7748
Nc-1-IP_pe	chr1	47682006	47682295	290	47682120	15	5.83691	3.4562
Nc-1-IP_pe	chr1	47688200	47689066	867	47688374	19	9.15219	4.54307
Nc-1-IP_pe	chr1	47693171	47693433	263	47693370	10	3.59902	2.78714
Nc-1-IP_pe	chr1	47694129	47694627	499	47694286	11	4.11366	2.98176
Nc-1-IP_pe	chr1	47701003	47701290	288	47701270	12	3.47963	2.55766
Nc-1-IP_pe	chr1	47703192	47703454	263	47703358	14	5.51541	3.4073

Nc-1-IP_pe	chr1	47708722	47709993	1272	47709837	21	7.10961	3.41419
Nc-1-IP_pe	chr1	47710887	47711367	481	47711052	15	5.00915	3.05678
Nc-1-IP_pe	chr1	47712162	47712513	352	47712325	18	7.04825	3.69086
Nc-1-IP_pe	chr1	47723245	47723499	255	47723355	19	6.32622	3.27682
Nc-1-IP_pe	chr1	47724610	47725138	529	47725079	14	4.21449	2.76599
Nc-1-IP_pe	chr1	47734419	47734672	254	47734571	15	6.19428	3.63445
Nc-1-IP_pe	chr1	47752176	47752587	412	47752266	13	4.90859	3.20357
Nc-1-IP_pe	chr1	47764047	47764554	508	47764127	13	5.26508	3.39133
Nc-1-IP_pe	chr1	47764901	47765488	588	47765152	12	4.06753	2.86157
Nc-1-IP_pe	chr1	47765899	47766353	455	47766002	17	8.15431	4.35118
Nc-1-IP_pe	chr1	47767900	47768305	406	47768027	14	4.60011	2.95114
Nc-1-IP_pe	chr1	47774427	47774829	403	47774623	12	4.75569	3.23024
Nc-1-IP_pe	chr1	47775286	47775896	611	47775462	16	7.44163	4.13536
Nc-1-IP_pe	chr1	47777352	47778105	754	47777504	17	8.56555	4.56077
Nc-1-IP_pe	chr1	47783389	47783879	491	47783507	17	4.44438	2.65333
Nc-1-IP_pe	chr1	47785338	47785714	377	47785450	11	3.4645	2.62647
Nc-1-IP_pe	chr1	47791239	47791571	333	47791422	14	5.06689	3.18081
Nc-1-IP_pe	chr1	47808782	47809453	672	47809099	17	5.89608	3.2766
Nc-1-IP_pe	chr1	47814342	47814915	574	47814640	13	4.63935	3.06421
Nc-1-IP_pe	chr1	47815385	47815714	330	47815601	17	7.63916	4.09458
Nc-1-IP_pe	chr1	47817190	47817677	488	47817252	12	4.63088	3.16234
Nc-1-IP_pe	chr1	47818061	47818699	639	47818598	13	4.90859	3.20357
Nc-1-IP_pe	chr1	47822908	47823222	315	47823087	16	7.37408	4.10088
Nc-1-IP_pe	chr1	47825344	47825749	406	47825728	11	3.63586	2.71903
Nc-1-IP_pe	chr1	47826955	47827351	397	47827259	16	6.92507	3.87472
Nc-1-IP_pe	chr1	47828008	47828359	352	47828056	13	3.86418	2.67468
Nc-1-IP_pe	chr1	47840122	47840388	267	47840331	12	4.24064	2.95299
Nc-1-IP_pe	chr1	47841186	47841579	394	47841294	11	3.64839	2.72584
Nc-1-IP_pe	chr1	47858671	47858957	287	47858813	11	3.64839	2.72584
Nc-1-IP_pe	chr1	47860008	47860518	511	47860080	11	4.11366	2.98176
Nc-1-IP_pe	chr1	47866562	47866828	267	47866822	11	3.64839	2.72584
Nc-1-IP_pe	chr1	47876083	47876775	693	47876579	12	3.47963	2.55766
Nc-1-IP_pe	chr1	47887754	47888694	941	47888455	12	4.70082	3.20033
Nc-1-IP_pe	chr1	47890416	47890669	254	47890596	13	5.83893	3.70124
Nc-1-IP_pe	chr1	47895975	47896756	782	47896023	17	6.47874	3.54137
Nc-1-IP_pe	chr1	47902484	47902765	282	47902604	16	7.54467	4.18818
Nc-1-IP_pe	chr1	47913245	47913548	304	47913425	15	5.58327	3.33181
Nc-1-IP_pe	chr1	47914111	47914615	505	47914615	12	2.87625	2.25568
Nc-1-IP_pe	chr1	47916297	47916791	495	47916498	14	4.8399	3.06836
Nc-1-IP_pe	chr1	47922258	47922553	296	47922360	12	3.86684	2.7567
Nc-1-IP_pe	chr1	47924100	47924970	871	47924373	13	5.58484	3.56287
Nc-1-IP_pe	chr1	47929712	47930171	460	47929901	17	6.38155	3.49661
Nc-1-IP_pe	chr1	47932971	47933231	261	47932996	12	4.63088	3.16234
Nc-1-IP_pe	chr1	47934231	47935113	883	47934322	14	5.51541	3.4073
Nc-1-IP_pe	chr1	47940002	47940268	267	47940147	20	5.70098	2.94771

Nc-1-IP_pe	chr1	47984218	47984491	274	47984358	18	8.62313	4.4354
Nc-1-IP_pe	chr1	47988480	47988825	346	47988599	12	5.94619	3.9002
Nc-1-IP_pe	chr1	47996752	47997187	436	47996806	15	4.90347	3.00714
Nc-1-IP_pe	chr1	48019770	48020030	261	48019907	14	4.21449	2.76599
Nc-1-IP_pe	chr1	48027259	48027793	535	48027699	16	5.82808	3.34463
Nc-1-IP_pe	chr1	48051279	48051920	642	48051789	11	4.23076	3.04719
Nc-1-IP_pe	chr1	48055803	48056173	371	48056093	10	3.71867	2.85592
Nc-1-IP_pe	chr1	48066830	48067300	471	48067090	12	4.24064	2.95299
Nc-1-IP_pe	chr1	48089420	48090066	647	48089659	24	9.05651	3.87976
Nc-1-IP_pe	chr1	48150148	48150961	814	48150564	13	3.07238	2.29378
Nc-1-IP_pe	chr1	48172576	48173423	848	48173173	16	8.26996	4.56762
Nc-1-IP_pe	chr1	48192244	48193249	1006	48192612	42	19.0858	5.2794
Nc-1-IP_pe	chr1	48197886	48198256	371	48198209	9	4.27829	3.29955
Nc-1-IP_pe	chr1	48214327	48214614	288	48214443	15	7.45104	4.28878
Nc-1-IP_pe	chr1	48222755	48223467	713	48223227	12	5.45448	3.61875
Nc-1-IP_pe	chr1	48228319	48228614	296	48228504	12	3.16137	2.39719
Nc-1-IP_pe	chr1	48230496	48231363	868	48231316	14	4.21449	2.76599
Nc-1-IP_pe	chr1	48235702	48235960	259	48235890	11	4.37724	3.12959
Nc-1-IP_pe	chr1	48245569	48245879	311	48245763	15	8.26005	4.73146
Nc-1-IP_pe	chr1	48247218	48247521	304	48247385	10	4.39571	3.25288
Nc-1-IP_pe	chr1	48267473	48267750	278	48267611	13	7.7352	4.78626
Nc-1-IP_pe	chr1	48277306	48277712	407	48277640	12	4.24064	2.95299
Nc-1-IP_pe	chr1	48284768	48285156	389	48284792	10	3.4604	2.70797
Nc-1-IP_pe	chr1	48311808	48312163	356	48312001	16	10.117	5.58955
Nc-1-IP_pe	chr1	48372628	48372891	264	48372679	9	3.02445	2.53932
Nc-1-IP_pe	chr1	48382148	48383285	1138	48382217	14	4.60011	2.95114
Nc-1-IP_pe	chr1	48383614	48384250	637	48384143	15	5.66913	3.37372
Nc-1-IP_pe	chr1	48471636	48472393	758	48471899	16	6.33285	3.58457
Nc-1-IP_pe	chr1	48478798	48479168	371	48479101	18	5.33197	2.94862
Nc-1-IP_pe	chr1	48572674	48573106	433	48572906	15	7.46994	4.29894
Nc-1-IP_pe	chr1	48624607	48625050	444	48625004	9	4.35492	3.34727
Nc-1-IP_pe	chr1	48654532	48655168	637	48654629	12	5.87693	3.86017
Nc-1-IP_pe	chr1	48677719	48678079	361	48677903	17	7.6273	4.08876
Nc-1-IP_pe	chr1	48681400	48681770	371	48681740	11	4.06403	2.95415
Nc-1-IP_pe	chr1	48692356	48692634	279	48692510	15	8.16556	4.67894
Nc-1-IP_pe	chr1	48701002	48701395	394	48701250	10	3.4604	2.70797
Nc-1-IP_pe	chr1	48707079	48707423	345	48707121	15	3.74763	2.48304
Nc-1-IP_pe	chr1	48708207	48708528	322	48708376	10	4.72812	3.45232
Nc-1-IP_pe	chr1	48710971	48711263	293	48711046	16	5.37454	3.13479
Nc-1-IP_pe	chr1	48719230	48719558	329	48719464	14	5.98179	3.64885
Nc-1-IP_pe	chr1	48735691	48736094	404	48735806	13	5.71736	3.63481
Nc-1-IP_pe	chr1	48741415	48741687	273	48741518	12	6.86906	4.44438
Nc-1-IP_pe	chr1	48749965	48750661	697	48750520	10	4.99888	3.61677
Nc-1-IP_pe	chr1	48758902	48759667	766	48759034	13	5.86991	3.71823
Nc-1-IP_pe	chr1	48862150	48862611	462	48862289	11	6.03439	4.10251

Nc-1-IP_pe	chr1	48886714	48887010	297	48886794	8	3.64414	3.01254
Nc-1-IP_pe	chr1	48893307	48893600	294	48893428	8	3.08755	2.66145
Nc-1-IP_pe	chr1	48895172	48895475	304	48895314	16	8.26996	4.56762
Nc-1-IP_pe	chr1	48948135	48948508	374	48948338	17	7.02067	3.79543
Nc-1-IP_pe	chr1	48981567	48981901	335	48981797	10	5.23357	3.76063
Nc-1-IP_pe	chr1	48993241	48993517	277	48993426	10	4.74365	3.46171
Nc-1-IP_pe	chr1	49121604	49122285	682	49121795	16	7.53857	4.18505
Nc-1-IP_pe	chr1	49250499	49251822	1324	49251668	17	9.65211	5.13407
Nc-1-IP_pe	chr1	49257693	49258077	385	49258001	12	3.16137	2.39719
Nc-1-IP_pe	chr1	49282629	49283258	630	49283005	18	7.73136	4.00629
Nc-1-IP_pe	chr1	49331103	49331566	464	49331163	15	7.75613	4.45384
Nc-1-IP_pe	chr1	49348182	49348644	463	49348392	11	5.10577	3.5486
Nc-1-IP_pe	chr1	49383887	49384148	262	49384022	15	9.55381	5.47001
Nc-1-IP_pe	chr1	49503320	49503668	349	49503477	14	7.42849	4.43574
Nc-1-IP_pe	chr1	49552442	49552703	262	49552643	14	4.60011	2.95114
Nc-1-IP_pe	chr1	49556716	49557533	818	49557122	10	3.4604	2.70797
Nc-1-IP_pe	chr1	49558664	49559014	351	49558672	9	3.27326	2.68684
Nc-1-IP_pe	chr1	49566624	49567414	791	49566896	14	8.63073	5.12814
Nc-1-IP_pe	chr1	49650953	49651327	375	49651099	13	6.62436	4.14003
Nc-1-IP_pe	chr1	49652488	49653146	659	49652590	11	6.03439	4.10251
Nc-1-IP_pe	chr1	49708964	49709452	489	49709306	11	4.54404	3.2242
Nc-1-IP_pe	chr1	49730512	49730839	328	49730677	10	3.08907	2.49869
Nc-1-IP_pe	chr1	49737956	49738211	256	49738051	12	5.61153	3.70794
Nc-1-IP_pe	chr1	49763717	49764454	738	49763859	13	6.62436	4.14003
Nc-1-IP_pe	chr1	49773546	49774005	460	49773708	18	10.9042	5.61861
Nc-1-IP_pe	chr1	49864783	49865223	441	49865000	13	7.42513	4.60316
Nc-1-IP_pe	chr1	49880628	49881090	463	49880665	15	4.7819	2.95039
Nc-1-IP_pe	chr1	49893485	49893929	445	49893673	11	3.64839	2.72584
Nc-1-IP_pe	chr1	49913403	49913895	493	49913535	14	5.03085	3.16286
Nc-1-IP_pe	chr1	49924895	49925613	719	49925103	14	5.51541	3.4073
Nc-1-IP_pe	chr1	49934912	49935640	729	49935244	15	6.19428	3.63445
Nc-1-IP_pe	chr1	49982161	49982613	453	49982357	14	7.9745	4.74633
Nc-1-IP_pe	chr1	50004440	50005136	697	50004742	10	4.07292	3.06202
Nc-1-IP_pe	chr1	50023663	50024051	389	50024007	11	4.88957	3.42271
Nc-1-IP_pe	chr1	50099255	50099549	295	50099388	13	5.9487	3.76157
Nc-1-IP_pe	chr1	50102019	50102297	279	50102198	10	4.99888	3.61677
Nc-1-IP_pe	chr1	50190718	50190971	254	50190784	11	4.35236	3.11555
Nc-1-IP_pe	chr1	50195550	50195915	366	50195713	16	8.26996	4.56762
Nc-1-IP_pe	chr1	50225462	50225919	458	50225721	14	5.03085	3.16286
Nc-1-IP_pe	chr1	50244090	50244544	455	50244366	12	4.70082	3.20033
Nc-1-IP_pe	chr1	50272779	50273080	302	50272910	13	6.62436	4.14003
Nc-1-IP_pe	chr1	50289341	50289642	302	50289364	9	3.4128	2.77032
Nc-1-IP_pe	chr1	50315338	50316005	668	50315680	20	11.7013	5.64235
Nc-1-IP_pe	chr1	50323324	50323668	345	50323409	11	4.61014	3.26192
Nc-1-IP_pe	chr1	50326172	50326894	723	50326486	13	5.36853	3.44651

Nc-1-IP_pe	chr1	50329397	50329743	347	50329614	12	5.23082	3.49289
Nc-1-IP_pe	chr1	50332985	50334063	1079	50333293	20	4.15754	2.37952
Nc-1-IP_pe	chr1	50334459	50334760	302	50334641	11	4.54404	3.2242
Nc-1-IP_pe	chr1	50354909	50355171	263	50354942	11	3.96771	2.90079
Nc-1-IP_pe	chr1	50356871	50357126	256	50357115	11	2.96397	2.36091
Nc-1-IP_pe	chr1	50358776	50359182	407	50358887	13	4.02562	2.7544
Nc-1-IP_pe	chr1	50359502	50359879	378	50359543	14	5.98179	3.64885
Nc-1-IP_pe	chr1	50368441	50368958	518	50368550	14	4.21449	2.76599
Nc-1-IP_pe	chr1	50397918	50398177	260	50398047	12	6.6095	4.28941
Nc-1-IP_pe	chr1	50404195	50404839	645	50404290	13	7.34205	4.55444
Nc-1-IP_pe	chr1	50413864	50414158	295	50413977	17	8.7831	4.67333
Nc-1-IP_pe	chr1	50415085	50415347	263	50415139	13	6.94469	4.32351
Nc-1-IP_pe	chr1	50416301	50417433	1133	50417275	13	6.34721	3.98335
Nc-1-IP_pe	chr1	50418512	50418918	407	50418725	12	5.3037	3.53374
Nc-1-IP_pe	chr1	50423108	50423398	291	50423161	12	4.80688	3.25822
Nc-1-IP_pe	chr1	50427806	50428085	280	50427929	16	7.53857	4.18505
Nc-1-IP_pe	chr1	50432227	50432600	374	50432354	12	5.23082	3.49289
Nc-1-IP_pe	chr1	50435108	50435435	328	50435239	12	5.40861	3.59282
Nc-1-IP_pe	chr1	50532961	50533293	333	50533197	9	2.89249	2.46179
Nc-1-IP_pe	chr1	50539986	50540250	265	50540073	11	6.03439	4.10251
Nc-1-IP_pe	chr1	50588576	50588905	330	50588649	12	3.50082	2.56844
Nc-1-IP_pe	chr1	50609404	50609687	284	50609558	15	7.75613	4.45384
Nc-1-IP_pe	chr1	50648676	50649047	372	50648815	13	6.34721	3.98335
Nc-1-IP_pe	chr1	50653468	50653780	313	50653641	12	5.23082	3.49289
Nc-1-IP_pe	chr1	50681715	50682327	613	50681966	11	5.54401	3.80747
Nc-1-IP_pe	chr1	50691231	50691630	400	50691453	16	8.17084	4.51499
Nc-1-IP_pe	chr1	50707507	50708143	637	50707531	11	3.64839	2.72584
Nc-1-IP_pe	chr1	50710836	50711103	268	50710916	13	4.02562	2.7544
Nc-1-IP_pe	chr1	50725621	50726193	573	50726134	9	4.088	3.18162
Nc-1-IP_pe	chr1	50765811	50766117	307	50765955	15	8.26005	4.73146
Nc-1-IP_pe	chr1	50815735	50816495	761	50815894	13	7.7352	4.78626
Nc-1-IP_pe	chr1	50837780	50838047	268	50837874	12	6.86906	4.44438
Nc-1-IP_pe	chr1	50846380	50846635	256	50846454	12	5.15877	3.45264
Nc-1-IP_pe	chr1	50876585	50876837	253	50876702	11	5.10577	3.5486
Nc-1-IP_pe	chr1	50891929	50892332	404	50892125	20	5.70098	2.94771
Nc-1-IP_pe	chr1	50899511	50899868	358	50899702	16	8.26996	4.56762
Nc-1-IP_pe	chr1	50910105	50910431	327	50910355	16	6.37501	3.60492
Nc-1-IP_pe	chr1	50952130	50952997	868	50952397	20	6.94658	3.44067
Nc-1-IP_pe	chr1	50968935	50969241	307	50969118	23	6.25615	2.94664
Nc-1-IP_pe	chr1	50977606	50977936	331	50977828	18	6.52861	3.45863
Nc-1-IP_pe	chr1	50987238	50987563	326	50987398	11	5.03835	3.5092
Nc-1-IP_pe	chr1	51014331	51014685	355	51014485	13	7.7352	4.78626
Nc-1-IP_pe	chr1	51022258	51022564	307	51022369	9	4.46937	3.41876
Nc-1-IP_pe	chr1	51034222	51034493	272	51034387	11	4.06403	2.95415
Nc-1-IP_pe	chr1	51060594	51060898	305	51060744	15	5.66913	3.37372

Nc-1-IP_pe	chr1	51068928	51069357	430	51069276	12	5.21606	3.48463
Nc-1-IP_pe	chr1	51086336	51087000	665	51086832	14	6.06519	3.69269
Nc-1-IP_pe	chr1	51087661	51087987	327	51087838	14	8.1351	4.83895
Nc-1-IP_pe	chr1	51095080	51095453	374	51095258	14	5.56442	3.43239
Nc-1-IP_pe	chr1	51138644	51138944	301	51138836	14	5.43414	3.36584
Nc-1-IP_pe	chr1	51157690	51158117	428	51158008	16	4.96462	2.94973
Nc-1-IP_pe	chr1	51186465	51186796	332	51186718	12	4.70082	3.20033
Nc-1-IP_pe	chr1	51201058	51201324	267	51201261	11	4.06403	2.95415
Nc-1-IP_pe	chr1	51207071	51207513	443	51207327	9	3.94978	3.09648
Nc-1-IP_pe	chr1	51236178	51237174	997	51236773	22	13.1291	5.97147
Nc-1-IP_pe	chr1	51246050	51246581	532	51246229	14	3.86727	2.6027
Nc-1-IP_pe	chr1	51250868	51251183	316	51251073	15	7.19874	4.15407
Nc-1-IP_pe	chr1	51266573	51267108	536	51266694	11	4.74697	3.34038
Nc-1-IP_pe	chr1	51288629	51289135	507	51289051	11	3.58722	2.69267
Nc-1-IP_pe	chr1	51296582	51296915	334	51296676	21	6.99413	3.36883
Nc-1-IP_pe	chr1	51301515	51301818	304	51301558	17	5.9911	3.31919
Nc-1-IP_pe	chr1	51306637	51307208	572	51307169	14	5.49876	3.39879
Nc-1-IP_pe	chr1	51309121	51309440	320	51309295	15	5.20142	3.14789
Nc-1-IP_pe	chr1	51310926	51312119	1194	51312048	17	6.34337	3.47909
Nc-1-IP_pe	chr1	51314539	51314796	258	51314681	19	5.80235	3.06257
Nc-1-IP_pe	chr1	51320904	51321174	271	51321035	14	6.01114	3.66425
Nc-1-IP_pe	chr1	51321514	51321925	412	51321780	15	4.40338	2.77622
Nc-1-IP_pe	chr1	51326716	51327321	606	51326832	14	3.86727	2.6027
Nc-1-IP_pe	chr1	51329519	51329803	285	51329671	19	4.5193	2.56258
Nc-1-IP_pe	chr1	51336726	51337012	287	51336964	14	6.44441	3.89444
Nc-1-IP_pe	chr1	51342572	51343057	486	51342688	17	5.9911	3.31919
Nc-1-IP_pe	chr1	51344727	51344981	255	51344852	19	6.33279	3.27955
Nc-1-IP_pe	chr1	51345263	51345610	348	51345319	15	4.70031	2.91253
Nc-1-IP_pe	chr1	51346124	51346384	261	51346205	12	4.84576	3.27953
Nc-1-IP_pe	chr1	51353395	51353812	418	51353739	12	4.1728	2.91706
Nc-1-IP_pe	chr1	51358735	51359372	638	51359253	15	9.19325	5.26076
Nc-1-IP_pe	chr1	51379627	51380178	552	51379708	16	5.82808	3.34463
Nc-1-IP_pe	chr1	51393245	51393535	291	51393380	13	6.26093	3.93497
Nc-1-IP_pe	chr1	51408867	51409165	299	51409137	10	3.89064	2.95552
Nc-1-IP_pe	chr1	51434630	51434890	261	51434758	13	7.34205	4.55444
Nc-1-IP_pe	chr1	51483489	51483788	300	51483564	11	4.54404	3.2242
Nc-1-IP_pe	chr1	51510878	51511322	445	51511078	14	5.03085	3.16286
Nc-1-IP_pe	chr1	51518353	51518995	643	51518395	18	3.77591	2.33276
Nc-1-IP_pe	chr1	51546115	51546694	580	51546545	21	11.9704	5.58651
Nc-1-IP_pe	chr1	51563001	51563479	479	51563146	15	7.07734	4.08984
Nc-1-IP_pe	chr1	51575070	51575415	346	51575088	11	4.62357	3.2696
Nc-1-IP_pe	chr1	51576331	51576637	307	51576601	9	3.27326	2.68684
Nc-1-IP_pe	chr1	51582482	51582936	455	51582883	12	5.20166	3.47658
Nc-1-IP_pe	chr1	51585548	51585983	436	51585572	12	4.24064	2.95299
Nc-1-IP_pe	chr1	51607874	51608292	419	51608025	12	5.77527	3.80164

Nc-1-IP_pe	chr1	51617063	51617490	428	51617256	21	9.29881	4.32834
Nc-1-IP_pe	chr1	51666005	51666258	254	51666128	13	6.7109	4.18935
Nc-1-IP_pe	chr1	51678367	51678622	256	51678549	12	5.23082	3.49289
Nc-1-IP_pe	chr1	51692022	51692286	265	51692173	13	7.17043	4.45427
Nc-1-IP_pe	chr1	51709471	51709769	299	51709585	17	3.33947	2.20998
Nc-1-IP_pe	chr1	51711182	51712404	1223	51712221	23	6.25615	2.94664
Nc-1-IP_pe	chr1	51783474	51783772	299	51783666	12	5.45448	3.61875
Nc-1-IP_pe	chr1	51847417	51847748	332	51847632	17	7.6273	4.08876
Nc-1-IP_pe	chr1	51849588	51849991	404	51849679	11	3.64839	2.72584
Nc-1-IP_pe	chr1	51856428	51856734	307	51856596	14	6.69566	4.03025
Nc-1-IP_pe	chr1	51930741	51931086	346	51930852	14	6.83507	4.10633
Nc-1-IP_pe	chr1	51935961	51936216	256	51936025	13	3.35769	2.42919
Nc-1-IP_pe	chr1	51955366	51955663	298	51955474	12	4.63088	3.16234
Nc-1-IP_pe	chr1	51967018	51967312	295	51967178	16	8.55602	4.72085
Nc-1-IP_pe	chr1	51986631	51987055	425	51986771	12	4.24064	2.95299
Nc-1-IP_pe	chr1	51990395	51990700	306	51990692	12	3.41594	2.52533
Nc-1-IP_pe	chr1	52011067	52011498	432	52011359	12	6.03452	3.95142
Nc-1-IP_pe	chr1	52012200	52012650	451	52012590	11	4.61014	3.26192
Nc-1-IP_pe	chr1	52022767	52023087	321	52022944	16	9.11744	5.02718
Nc-1-IP_pe	chr1	52040180	52040456	277	52040253	13	5.70242	3.62667
Nc-1-IP_pe	chr1	52042689	52042993	305	52042901	13	4.02562	2.7544
Nc-1-IP_pe	chr1	52061754	52062051	298	52061855	11	5.14641	3.5724
Nc-1-IP_pe	chr1	52081061	52081373	313	52081156	14	6.28469	3.80898
Nc-1-IP_pe	chr1	52093339	52093591	253	52093373	11	4.54404	3.2242
Nc-1-IP_pe	chr1	52129761	52130068	308	52129924	12	5.50092	3.64505
Nc-1-IP_pe	chr1	52132254	52132506	253	52132448	10	4.07292	3.06202
Nc-1-IP_pe	chr1	52136464	52136857	394	52136824	11	4.54404	3.2242
Nc-1-IP_pe	chr1	52176681	52176963	283	52176811	16	7.61454	4.22416
Nc-1-IP_pe	chr1	52181305	52181670	366	52181563	18	5.72638	3.11298
Nc-1-IP_pe	chr1	52202872	52203316	445	52202949	10	3.40382	2.67582
Nc-1-IP_pe	chr1	52252334	52252768	435	52252684	13	5.42865	3.47872
Nc-1-IP_pe	chr1	52260514	52260808	295	52260611	12	6.50787	4.22912
Nc-1-IP_pe	chr1	52309161	52309425	265	52309318	14	6.66104	4.01144
Nc-1-IP_pe	chr1	52319450	52319884	435	52319604	9	3.27326	2.68684
Nc-1-IP_pe	chr1	52326814	52327097	284	52326886	13	6.56172	4.10444
Nc-1-IP_pe	chr1	52345096	52345357	262	52345273	16	7.34063	4.08385
Nc-1-IP_pe	chr1	52356944	52357226	283	52357020	14	5.7679	3.53731
Nc-1-IP_pe	chr1	52358881	52359487	607	52359121	20	9.94634	4.77022
Nc-1-IP_pe	chr1	52359838	52361097	1260	52360021	15	5.58327	3.33181
Nc-1-IP_pe	chr1	52362111	52362654	544	52362480	18	8.10208	4.1823
Nc-1-IP_pe	chr1	52365773	52366162	390	52366091	17	4.78065	2.79327
Nc-1-IP_pe	chr1	52367076	52367475	400	52367123	11	4.06403	2.95415
Nc-1-IP_pe	chr1	52383469	52383788	320	52383696	11	3.28475	2.53029
Nc-1-IP_pe	chr1	52403927	52404235	309	52404023	17	5.14803	2.94914
Nc-1-IP_pe	chr1	52428466	52428747	282	52428620	12	5.61153	3.70794

Nc-1-IP_pe	chr1	52429103	52429360	258	52429209	10	4.24068	3.16086
Nc-1-IP_pe	chr1	52432836	52433178	343	52433061	11	4.06403	2.95415
Nc-1-IP_pe	chr1	52443119	52443588	470	52443488	12	4.70082	3.20033
Nc-1-IP_pe	chr1	52459450	52459712	263	52459573	12	3.83688	2.74114
Nc-1-IP_pe	chr1	52524320	52524764	445	52524706	11	4.06403	2.95415
Nc-1-IP_pe	chr1	52537627	52538233	607	52538150	15	6.19428	3.63445
Nc-1-IP_pe	chr1	52544775	52545130	356	52544986	16	8.17084	4.51499
Nc-1-IP_pe	chr1	52552665	52553927	1263	52552852	21	9.29881	4.32834
Nc-1-IP_pe	chr1	52558094	52558473	380	52558141	10	4.46067	3.29163
Nc-1-IP_pe	chr1	52572636	52572958	323	52572862	14	6.97924	4.18554
Nc-1-IP_pe	chr1	52587596	52587925	330	52587793	14	7.34079	4.38651
Nc-1-IP_pe	chr1	52602136	52602550	415	52602459	15	5.20142	3.14789
Nc-1-IP_pe	chr1	52606650	52607386	737	52607130	13	5.83893	3.70124
Nc-1-IP_pe	chr1	52612177	52612664	488	52612577	14	4.60011	2.95114
Nc-1-IP_pe	chr1	52616583	52617090	508	52616703	10	3.64194	2.81176
Nc-1-IP_pe	chr1	52626693	52626956	264	52626742	10	4.02314	3.03283
Nc-1-IP_pe	chr1	52629257	52629543	287	52629439	16	4.59224	2.7853
Nc-1-IP_pe	chr1	52631934	52632329	396	52632322	10	3.97417	3.0042
Nc-1-IP_pe	chr1	52632644	52633883	1240	52633483	13	4.41954	2.952
Nc-1-IP_pe	chr1	52636893	52637262	370	52637033	19	8.51517	4.24108
Nc-1-IP_pe	chr1	52649972	52650278	307	52650039	15	4.7819	2.95039
Nc-1-IP_pe	chr1	52657392	52657803	412	52657539	11	4.06403	2.95415
Nc-1-IP_pe	chr1	52664410	52664949	540	52664754	17	7.46205	4.00792
Nc-1-IP_pe	chr1	52674287	52674571	285	52674478	13	6.09351	3.84167
Nc-1-IP_pe	chr1	52682098	52682466	369	52682171	12	3.16137	2.39719
Nc-1-IP_pe	chr1	52687714	52688104	391	52687987	14	4.60011	2.95114
Nc-1-IP_pe	chr1	52690890	52691399	510	52691016	11	3.64839	2.72584
Nc-1-IP_pe	chr1	52702977	52703314	338	52703149	14	3.26761	2.32785
Nc-1-IP_pe	chr1	52735286	52735711	426	52735625	14	6.54309	3.94758
Nc-1-IP_pe	chr1	52748664	52749162	499	52748794	14	6.06519	3.69269
Nc-1-IP_pe	chr1	52780985	52781387	403	52781301	13	4.41954	2.952
Nc-1-IP_pe	chr1	52805501	52805802	302	52805664	12	4.70082	3.20033
Nc-1-IP_pe	chr1	52841273	52841596	324	52841375	16	7.53857	4.18505
Nc-1-IP_pe	chr1	52843022	52843470	449	52843059	17	4.78101	2.79343
Nc-1-IP_pe	chr1	52851940	52852214	275	52852084	12	5.13046	3.43687
Nc-1-IP_pe	chr1	52858488	52858884	397	52858843	14	4.21449	2.76599
Nc-1-IP_pe	chr1	52864831	52865140	310	52865022	15	7.37761	4.24941
Nc-1-IP_pe	chr1	52869740	52870830	1091	52870620	25	9.19615	3.83259
Nc-1-IP_pe	chr1	52904611	52904920	310	52904724	14	5.03085	3.16286
Nc-1-IP_pe	chr1	52906536	52906814	279	52906677	16	7.59698	4.21511
Nc-1-IP_pe	chr1	52907118	52907388	271	52907254	13	5.36853	3.44651
Nc-1-IP_pe	chr1	52908182	52908500	319	52908377	15	6.19428	3.63445
Nc-1-IP_pe	chr1	52911572	52911880	309	52911609	11	3.64839	2.72584
Nc-1-IP_pe	chr1	52921780	52922060	281	52921783	13	3.07238	2.29378
Nc-1-IP_pe	chr1	52926549	52927919	1371	52926601	17	4.78101	2.79343

Nc-1-IP_pe	chr1	52943191	52943649	459	52943307	15	5.20142	3.14789
Nc-1-IP_pe	chr1	52966757	52967081	325	52966869	9	3.27326	2.68684
Nc-1-IP_pe	chr1	52979164	52979493	330	52979343	11	6.03439	4.10251
Nc-1-IP_pe	chr1	53026522	53026792	271	53026657	15	7.46994	4.29894
Nc-1-IP_pe	chr1	53063323	53063726	404	53063643	15	5.70822	3.39286
Nc-1-IP_pe	chr1	53069794	53070433	640	53070331	15	5.96953	3.52194
Nc-1-IP_pe	chr1	53074158	53074695	538	53074544	13	4.63935	3.06421
Nc-1-IP_pe	chr1	53075261	53075646	386	53075302	12	3.83688	2.74114
Nc-1-IP_pe	chr1	53084749	53085230	482	53084793	14	4.60011	2.95114
Nc-1-IP_pe	chr1	53087621	53088152	532	53088070	14	4.60011	2.95114
Nc-1-IP_pe	chr1	53088738	53089090	353	53088986	21	9.71982	4.51623
Nc-1-IP_pe	chr1	53089765	53090162	398	53090000	15	3.98281	2.58698
Nc-1-IP_pe	chr1	53093307	53093660	354	53093357	13	3.6894	2.58919
Nc-1-IP_pe	chr1	53094622	53095183	562	53094778	16	5.64353	3.25859
Nc-1-IP_pe	chr1	53100717	53101223	507	53100801	18	4.96968	2.80074
Nc-1-IP_pe	chr1	53101695	53101949	255	53101714	13	3.29152	2.39761
Nc-1-IP_pe	chr1	53102340	53102708	369	53102622	15	5.20142	3.14789
Nc-1-IP_pe	chr1	53109460	53109954	495	53109629	18	5.72638	3.11298
Nc-1-IP_pe	chr1	53114562	53115749	1188	53115059	18	6.15736	3.29676
Nc-1-IP_pe	chr1	53116423	53117205	783	53116976	17	5.7822	3.22586
Nc-1-IP_pe	chr1	53117852	53118185	334	53118024	19	6.78661	3.47027
Nc-1-IP_pe	chr1	53121132	53121458	327	53121347	13	4.33674	2.9101
Nc-1-IP_pe	chr1	53123408	53124014	607	53123882	16	5.82808	3.34463
Nc-1-IP_pe	chr1	53135270	53135563	294	53135365	13	3.35769	2.42919
Nc-1-IP_pe	chr1	53139142	53139427	286	53139302	13	4.56123	3.02417
Nc-1-IP_pe	chr1	53141466	53141749	284	53141588	18	4.96968	2.80074
Nc-1-IP_pe	chr1	53142348	53142622	275	53142412	16	4.25252	2.63824
Nc-1-IP_pe	chr1	53155003	53155420	418	53155261	18	11.081	5.71511
Nc-1-IP_pe	chr1	53196648	53197208	561	53196979	22	9.39426	4.24119
Nc-1-IP_pe	chr1	53205671	53206018	348	53205787	16	7.95994	4.40382
Nc-1-IP_pe	chr1	53211027	53211314	288	53211203	15	6.78905	3.93887
Nc-1-IP_pe	chr1	53211895	53212153	259	53212131	9	3.02445	2.53932
Nc-1-IP_pe	chr1	53222284	53222551	268	53222513	13	5.03208	3.26819
Nc-1-IP_pe	chr1	53238152	53239006	855	53238477	40	22.6623	6.71749
Nc-1-IP_pe	chr1	53260384	53260637	254	53260498	13	6.7109	4.18935
Nc-1-IP_pe	chr1	53262099	53262418	320	53262340	15	6.85973	3.97568
Nc-1-IP_pe	chr1	53263116	53263642	527	53263608	13	5.57038	3.55505
Nc-1-IP_pe	chr1	53264036	53264511	476	53264208	16	6.8987	3.86161
Nc-1-IP_pe	chr1	53271351	53271792	442	53271729	16	4.59224	2.7853
Nc-1-IP_pe	chr1	53274114	53274405	292	53274311	19	8.25448	4.12028
Nc-1-IP_pe	chr1	53274665	53276088	1424	53275826	18	5.72638	3.11298
Nc-1-IP_pe	chr1	53277001	53277449	449	53277233	16	6.08355	3.46522
Nc-1-IP_pe	chr1	53288010	53288443	434	53288055	14	5.22188	3.25843
Nc-1-IP_pe	chr1	53289110	53289704	595	53289395	17	4.78101	2.79343
Nc-1-IP_pe	chr1	53293885	53294241	357	53294022	18	7.79534	4.03642

Nc-1-IP_pe	chr1	53295228	53295602	375	53295429	13	3.95529	2.71958
Nc-1-IP_pe	chr1	53297298	53297562	265	53297488	15	5.49794	3.29036
Nc-1-IP_pe	chr1	53298009	53298319	311	53298258	14	4.21449	2.76599
Nc-1-IP_pe	chr1	53299116	53299556	441	53299163	15	4.40338	2.77622
Nc-1-IP_pe	chr1	53303235	53303494	260	53303408	15	5.1177	3.10809
Nc-1-IP_pe	chr1	53307139	53307675	537	53307380	15	5.36727	3.22728
Nc-1-IP_pe	chr1	53310856	53311618	763	53310966	15	5.72092	3.39909
Nc-1-IP_pe	chr1	53314350	53314839	490	53314826	12	4.19427	2.92842
Nc-1-IP_pe	chr1	53316539	53317218	680	53316899	13	4.41954	2.952
Nc-1-IP_pe	chr1	53319072	53320130	1059	53319247	16	6.1482	3.49601
Nc-1-IP_pe	chr1	53320615	53320879	265	53320723	16	5.73763	3.30235
Nc-1-IP_pe	chr1	53322434	53322720	287	53322448	15	5.00915	3.05678
Nc-1-IP_pe	chr1	53323389	53323702	314	53323395	12	3.31728	2.47546
Nc-1-IP_pe	chr1	53324215	53324673	459	53324244	16	4.59224	2.7853
Nc-1-IP_pe	chr1	53326291	53327762	1472	53327115	18	6.15736	3.29676
Nc-1-IP_pe	chr1	53332128	53332499	372	53332359	13	6.10995	3.8508
Nc-1-IP_pe	chr1	53334405	53334941	537	53334512	14	7.34079	4.38651
Nc-1-IP_pe	chr1	53342096	53343335	1240	53342231	15	6.19428	3.63445
Nc-1-IP_pe	chr1	53343980	53344495	516	53344453	12	4.44012	3.05944
Nc-1-IP_pe	chr1	53348748	53349187	440	53349096	17	5.54967	3.12324
Nc-1-IP_pe	chr1	53350639	53350916	278	53350857	17	6.13258	3.38303
Nc-1-IP_pe	chr1	53360963	53361226	264	53361221	11	4.11366	2.98176
Nc-1-IP_pe	chr1	53361865	53362289	425	53361901	12	3.16137	2.39719
Nc-1-IP_pe	chr1	53363156	53363496	341	53363325	18	7.77965	4.02903
Nc-1-IP_pe	chr1	53367095	53367880	786	53367860	14	3.86727	2.6027
Nc-1-IP_pe	chr1	53368925	53369718	794	53369270	14	4.21449	2.76599
Nc-1-IP_pe	chr1	53376551	53376960	410	53376825	10	3.00887	2.45401
Nc-1-IP_pe	chr1	53377415	53377965	551	53377873	15	6.29054	3.68307
Nc-1-IP_pe	chr1	53382752	53383174	423	53382879	14	5.98179	3.64885
Nc-1-IP_pe	chr1	53383517	53384060	544	53383641	14	6.16097	3.74327
Nc-1-IP_pe	chr1	53390204	53390555	352	53390493	16	4.59224	2.7853
Nc-1-IP_pe	chr1	53394265	53394587	323	53394485	9	2.98693	2.51723
Nc-1-IP_pe	chr1	53395684	53396415	732	53396083	14	5.51541	3.4073
Nc-1-IP_pe	chr1	53403459	53403780	322	53403588	14	5.69896	3.50164
Nc-1-IP_pe	chr1	53412482	53412748	267	53412741	12	3.47963	2.55766
Nc-1-IP_pe	chr1	53413812	53414162	351	53413887	17	4.78101	2.79343
Nc-1-IP_pe	chr1	53425043	53425313	271	53425184	14	5.03085	3.16286
Nc-1-IP_pe	chr1	53438680	53438935	256	53438822	21	6.2632	3.08808
Nc-1-IP_pe	chr1	53460043	53460486	444	53460338	14	5.03085	3.16286
Nc-1-IP_pe	chr1	53460986	53461465	480	53461017	12	4.64316	3.169
Nc-1-IP_pe	chr1	53466821	53467151	331	53466831	11	3.64839	2.72584
Nc-1-IP_pe	chr1	53469382	53469687	306	53469586	11	3.64839	2.72584
Nc-1-IP_pe	chr1	53470330	53470905	576	53470481	14	4.42328	2.86573
Nc-1-IP_pe	chr1	53472014	53472348	335	53472091	10	3.78613	2.89489
Nc-1-IP_pe	chr1	53478280	53478727	448	53478478	15	6.99247	4.04516

Nc-1-IP_pe	chr1	53482014	53482592	579	53482369	12	4.06753	2.86157
Nc-1-IP_pe	chr1	53484806	53485096	291	53484944	14	5.03085	3.16286
Nc-1-IP_pe	chr1	53490814	53491150	337	53490841	13	6.17638	3.88776
Nc-1-IP_pe	chr1	53491711	53492053	343	53491919	10	3.60969	2.79325
Nc-1-IP_pe	chr1	53495701	53496036	336	53495788	13	5.9487	3.76157
Nc-1-IP_pe	chr1	53498615	53498940	326	53498875	17	5.9911	3.31919
Nc-1-IP_pe	chr1	53499705	53500114	410	53500047	12	4.99209	3.3601
Nc-1-IP_pe	chr1	53500414	53500745	332	53500622	16	7.11257	3.96852
Nc-1-IP_pe	chr1	53503403	53503816	414	53503711	12	4.76841	3.23719
Nc-1-IP_pe	chr1	53505494	53506078	585	53505569	13	4.41954	2.952
Nc-1-IP_pe	chr1	53506499	53507547	1049	53507272	17	5.54967	3.12324
Nc-1-IP_pe	chr1	53508410	53508773	364	53508715	13	4.67336	3.0817
Nc-1-IP_pe	chr1	53509108	53509432	325	53509298	19	8.03556	4.02008
Nc-1-IP_pe	chr1	53511330	53511612	283	53511568	11	3.21718	2.49437
Nc-1-IP_pe	chr1	53512580	53513101	522	53512770	18	5.33197	2.94862
Nc-1-IP_pe	chr1	53513937	53515112	1176	53514640	15	5.52215	3.3021
Nc-1-IP_pe	chr1	53516243	53516496	254	53516448	14	3.26761	2.32785
Nc-1-IP_pe	chr1	53517799	53518224	426	53518098	12	3.60603	2.62216
Nc-1-IP_pe	chr1	53520341	53520796	456	53520598	21	7.10961	3.41419
Nc-1-IP_pe	chr1	53521819	53522519	701	53521994	15	5.20142	3.14789
Nc-1-IP_pe	chr1	53524793	53525241	449	53524967	16	5.37454	3.13479
Nc-1-IP_pe	chr1	53526923	53527244	322	53527047	20	8.41487	4.06572
Nc-1-IP_pe	chr1	53528706	53529264	559	53529262	15	3.20025	2.24588
Nc-1-IP_pe	chr1	53533340	53534608	1269	53534063	18	6.61581	3.49714
Nc-1-IP_pe	chr1	53536861	53537291	431	53537197	13	3.64718	2.56867
Nc-1-IP_pe	chr1	53538418	53538782	365	53538733	14	4.42328	2.86573
Nc-1-IP_pe	chr1	53542737	53543038	302	53542921	24	11.2835	4.77622
Nc-1-IP_pe	chr1	53544415	53544687	273	53544497	13	4.02562	2.7544
Nc-1-IP_pe	chr1	53545891	53546349	459	53546126	17	7.02067	3.79543
Nc-1-IP_pe	chr1	53546915	53547570	656	53547089	16	6.37501	3.60492
Nc-1-IP_pe	chr1	53559332	53559611	280	53559463	13	5.03208	3.26819
Nc-1-IP_pe	chr1	53560365	53561100	736	53560730	15	6.24734	3.66122
Nc-1-IP_pe	chr1	53561712	53562228	517	53562175	14	5.03085	3.16286
Nc-1-IP_pe	chr1	53567774	53568124	351	53567993	11	3.28475	2.53029
Nc-1-IP_pe	chr1	53572708	53573199	492	53572821	15	6.10627	3.59023
Nc-1-IP_pe	chr1	53579399	53579858	460	53579592	20	9.43239	4.52781
Nc-1-IP_pe	chr1	53583331	53583665	335	53583597	14	3.86727	2.6027
Nc-1-IP_pe	chr1	53588192	53588640	449	53588417	11	3.28475	2.53029
Nc-1-IP_pe	chr1	53592836	53593277	442	53593095	15	4.90347	3.00714
Nc-1-IP_pe	chr1	53593556	53593923	368	53593704	16	4.25252	2.63824
Nc-1-IP_pe	chr1	53600075	53600750	676	53600676	12	4.99209	3.3601
Nc-1-IP_pe	chr1	53603383	53603721	339	53603520	10	3.08907	2.49869
Nc-1-IP_pe	chr1	53616532	53617132	601	53616616	13	5.9487	3.76157
Nc-1-IP_pe	chr1	53617670	53618034	365	53617968	11	4.84616	3.39759
Nc-1-IP_pe	chr1	53618530	53619423	894	53618740	13	5.86991	3.71823

Nc-1-IP_pe	chr1	53621103	53621408	306	53621204	14	5.03085	3.16286
Nc-1-IP_pe	chr1	53631835	53632155	321	53632046	11	4.23076	3.04719
Nc-1-IP_pe	chr1	53632625	53633099	475	53633050	11	4.32767	3.10163
Nc-1-IP_pe	chr1	53633409	53634070	662	53633483	16	7.95994	4.40382
Nc-1-IP_pe	chr1	53636943	53637418	476	53637224	16	7.44163	4.13536
Nc-1-IP_pe	chr1	53640401	53640684	284	53640535	13	4.41954	2.952
Nc-1-IP_pe	chr1	53643556	53644185	630	53643557	12	2.87625	2.25568
Nc-1-IP_pe	chr1	53653377	53654031	655	53653500	10	3.60969	2.79325
Nc-1-IP_pe	chr1	53654336	53654972	637	53654719	16	7.53857	4.18505
Nc-1-IP_pe	chr1	53658664	53659526	863	53658856	12	5.3037	3.53374
Nc-1-IP_pe	chr1	53660597	53660959	363	53660701	17	6.47874	3.54137
Nc-1-IP_pe	chr1	53663782	53664186	405	53663799	16	5.37454	3.13479
Nc-1-IP_pe	chr1	53664781	53665045	265	53664947	13	4.41954	2.952
Nc-1-IP_pe	chr1	53672634	53672890	257	53672760	14	3.26761	2.32785
Nc-1-IP_pe	chr1	53675409	53675782	374	53675503	14	6.02589	3.672
Nc-1-IP_pe	chr1	53678625	53679132	508	53678955	11	4.32767	3.10163
Nc-1-IP_pe	chr1	53681043	53681484	442	53681261	14	5.63114	3.46667
Nc-1-IP_pe	chr1	53682211	53682612	402	53682498	17	7.62281	4.08655
Nc-1-IP_pe	chr1	53683787	53684364	578	53684242	14	3.63457	2.49502
Nc-1-IP_pe	chr1	53684911	53685170	260	53684991	18	5.33197	2.94862
Nc-1-IP_pe	chr1	53685844	53686366	523	53685965	15	4.40338	2.77622
Nc-1-IP_pe	chr1	53687589	53688014	426	53687982	15	5.4619	3.27292
Nc-1-IP_pe	chr1	53690957	53691719	763	53691538	21	6.66991	3.24296
Nc-1-IP_pe	chr1	53693448	53694552	1105	53694008	12	4.60645	3.1491
Nc-1-IP_pe	chr1	53698207	53698726	520	53698433	18	7.73136	4.00629
Nc-1-IP_pe	chr1	53702089	53703050	962	53702491	18	9.1091	4.6774
Nc-1-IP_pe	chr1	53710059	53710439	381	53710249	14	5.51541	3.4073
Nc-1-IP_pe	chr1	53718861	53719269	409	53719135	15	5.66913	3.37372
Nc-1-IP_pe	chr1	53726085	53726367	283	53726203	12	5.61153	3.70794
Nc-1-IP_pe	chr1	53737664	53738254	591	53738079	16	6.24025	3.54005
Nc-1-IP_pe	chr1	53738510	53739571	1062	53739435	18	6.78184	3.57098
Nc-1-IP_pe	chr1	53749107	53749436	330	53749208	14	6.28469	3.80898
Nc-1-IP_pe	chr1	53752328	53752767	440	53752531	11	3.28475	2.53029
Nc-1-IP_pe	chr1	53758868	53759292	425	53759143	14	7.18513	4.29958
Nc-1-IP_pe	chr1	53804413	53804725	313	53804561	12	6.29276	4.10227
Nc-1-IP_pe	chr1	53830904	53831198	295	53831134	10	4.07292	3.06202
Nc-1-IP_pe	chr1	53853995	53854534	540	53854328	14	6.06519	3.69269
Nc-1-IP_pe	chr1	53866031	53866363	333	53866049	8	3.10513	2.67242
Nc-1-IP_pe	chr1	53894147	53894636	490	53894341	17	7.32147	3.9397
Nc-1-IP_pe	chr1	53895918	53896435	518	53896053	15	5.70822	3.39286
Nc-1-IP_pe	chr1	53898024	53898378	355	53898163	22	8.24538	3.76835
Nc-1-IP_pe	chr1	53899757	53900019	263	53899872	12	4.06753	2.86157
Nc-1-IP_pe	chr1	53903063	53903426	364	53903160	14	5.61771	3.45976
Nc-1-IP_pe	chr1	53906972	53907243	272	53907024	17	7.36791	3.96218
Nc-1-IP_pe	chr1	53944485	53945135	651	53945041	17	3.58449	2.30632

Nc-1-IP_pe	chr1	53946918	53947266	349	53947076	19	9.15219	4.54307
Nc-1-IP_pe	chr1	53955319	53955937	619	53955887	16	4.96462	2.94973
Nc-1-IP_pe	chr1	53963143	53963703	561	53963345	14	4.21449	2.76599
Nc-1-IP_pe	chr1	53992688	53993101	414	53992905	20	9.21699	4.428
Nc-1-IP_pe	chr1	54003106	54003373	268	54003308	15	5.87628	3.47566
Nc-1-IP_pe	chr1	54004257	54004727	471	54004367	14	3.86727	2.6027
Nc-1-IP_pe	chr1	54009273	54009572	300	54009437	18	5.72638	3.11298
Nc-1-IP_pe	chr1	54010281	54010579	299	54010436	16	5.98198	3.41707
Nc-1-IP_pe	chr1	54010991	54011495	505	54011057	15	5.27475	3.1829
Nc-1-IP_pe	chr1	54013278	54013690	413	54013516	24	11.377	4.81598
Nc-1-IP_pe	chr1	54014170	54014664	495	54014192	15	4.7819	2.95039
Nc-1-IP_pe	chr1	54017524	54018169	646	54017845	13	3.6894	2.58919
Nc-1-IP_pe	chr1	54019010	54019661	652	54019642	16	3.65575	2.38624
Nc-1-IP_pe	chr1	54022235	54022696	462	54022496	15	4.40338	2.77622
Nc-1-IP_pe	chr1	54034464	54034772	309	54034613	14	6.06519	3.69269
Nc-1-IP_pe	chr1	54065480	54066115	636	54065542	12	5.3037	3.53374
Nc-1-IP_pe	chr1	54073095	54073432	338	54073252	12	3.16137	2.39719
Nc-1-IP_pe	chr1	54104842	54105443	602	54105394	10	3.08907	2.49869
Nc-1-IP_pe	chr1	54108337	54108909	573	54108688	15	5.20142	3.14789
Nc-1-IP_pe	chr1	54109718	54110207	490	54110054	14	5.51541	3.4073
Nc-1-IP_pe	chr1	54111196	54111596	401	54111277	12	4.91154	3.31567
Nc-1-IP_pe	chr1	54120575	54121598	1024	54121552	15	3.46204	2.35851
Nc-1-IP_pe	chr1	54130229	54130488	260	54130332	14	5.92369	3.61842
Nc-1-IP_pe	chr1	54134157	54134467	311	54134423	12	4.28158	2.97474
Nc-1-IP_pe	chr1	54136162	54136771	610	54136304	12	4.19427	2.92842
Nc-1-IP_pe	chr1	54150837	54151658	822	54151316	17	7.02067	3.79543
Nc-1-IP_pe	chr1	54152720	54153157	438	54152894	20	9.88243	4.73975
Nc-1-IP_pe	chr1	54161188	54161812	625	54161345	14	4.13959	2.7305
Nc-1-IP_pe	chr1	54163705	54163983	279	54163773	15	5.00915	3.05678
Nc-1-IP_pe	chr1	54168200	54168731	532	54168295	14	3.86727	2.6027
Nc-1-IP_pe	chr1	54174624	54174894	271	54174859	12	3.98572	2.81868
Nc-1-IP_pe	chr1	54177870	54178423	554	54178058	14	5.71266	3.50871
Nc-1-IP_pe	chr1	54185329	54185712	384	54185592	13	4.41954	2.952
Nc-1-IP_pe	chr1	54189373	54189910	538	54189748	14	3.55308	2.45762
Nc-1-IP_pe	chr1	54195358	54195733	376	54195542	14	5.30793	3.30182
Nc-1-IP_pe	chr1	54200198	54200615	418	54200323	20	4.42487	2.47493
Nc-1-IP_pe	chr1	54211333	54211654	322	54211545	14	6.14575	3.73522
Nc-1-IP_pe	chr1	54218201	54218504	304	54218348	19	7.29128	3.68799
Nc-1-IP_pe	chr1	54221117	54221480	364	54221234	14	5.25853	3.27688
Nc-1-IP_pe	chr1	54223404	54223870	467	54223534	12	4.0366	2.84534
Nc-1-IP_pe	chr1	54226061	54226383	323	54226180	17	7.13975	3.85227
Nc-1-IP_pe	chr1	54232626	54233892	1267	54233485	21	10.9221	5.07431
Nc-1-IP_pe	chr1	54238544	54238830	287	54238621	15	4.7819	2.95039
Nc-1-IP_pe	chr1	54239465	54239785	321	54239585	14	5.51541	3.4073
Nc-1-IP_pe	chr1	54244371	54245064	694	54244632	16	6.75928	3.79256

Nc-1-IP_pe	chr1	54248278	54248828	551	54248430	13	3.67377	2.58159
Nc-1-IP_pe	chr1	54250411	54252294	1884	54251606	17	7.52577	4.03901
Nc-1-IP_pe	chr1	54253339	54253832	494	54253421	16	5.98198	3.41707
Nc-1-IP_pe	chr1	54258525	54258784	260	54258544	12	3.67829	2.65924
Nc-1-IP_pe	chr1	54260337	54260803	467	54260605	11	3.7458	2.77889
Nc-1-IP_pe	chr1	54268045	54268321	277	54268295	11	3.4645	2.62647
Nc-1-IP_pe	chr1	54269078	54269345	268	54269241	12	3.83688	2.74114
Nc-1-IP_pe	chr1	54269879	54270134	256	54269959	16	6.54198	3.68597
Nc-1-IP_pe	chr1	54273056	54273353	298	54273313	12	4.24064	2.95299
Nc-1-IP_pe	chr1	54273808	54274202	395	54274071	15	6.23304	3.654
Nc-1-IP_pe	chr1	54274888	54275190	303	54275050	14	4.73128	3.01506
Nc-1-IP_pe	chr1	54280511	54280983	473	54280816	17	7.85637	4.20196
Nc-1-IP_pe	chr1	54281336	54281825	490	54281722	13	5.37336	3.44909
Nc-1-IP_pe	chr1	54286603	54286967	365	54286916	13	4.02562	2.7544
Nc-1-IP_pe	chr1	54287917	54288180	264	54288032	15	6.5434	3.81197
Nc-1-IP_pe	chr1	54294741	54295315	575	54295161	17	7.52577	4.03901
Nc-1-IP_pe	chr1	54297716	54298194	479	54297882	13	4.24481	2.8638
Nc-1-IP_pe	chr1	54304746	54305360	615	54305014	17	7.78859	4.16833
Nc-1-IP_pe	chr1	54310740	54311025	286	54310925	14	3.26761	2.32785
Nc-1-IP_pe	chr1	54314841	54315353	513	54315104	15	6.78905	3.93887
Nc-1-IP_pe	chr1	54329940	54330404	465	54330177	14	5.80982	3.55907
Nc-1-IP_pe	chr1	54335578	54335975	398	54335804	14	4.60011	2.95114
Nc-1-IP_pe	chr1	54337006	54337480	475	54337266	16	4.59224	2.7853
Nc-1-IP_pe	chr1	54342544	54343130	587	54343015	17	6.47874	3.54137
Nc-1-IP_pe	chr1	54348097	54348472	376	54348371	12	4.95159	3.33774
Nc-1-IP_pe	chr1	54354198	54354623	426	54354563	12	4.99209	3.3601
Nc-1-IP_pe	chr1	54355562	54355908	347	54355772	12	5.00569	3.36763
Nc-1-IP_pe	chr1	54356919	54357298	380	54357185	17	7.06589	3.81697
Nc-1-IP_pe	chr1	54381603	54381958	356	54381666	15	4.7819	2.95039
Nc-1-IP_pe	chr1	54404960	54405588	629	54405358	15	6.19428	3.63445
Nc-1-IP_pe	chr1	54414476	54414833	358	54414643	17	7.02067	3.79543
Nc-1-IP_pe	chr1	54417903	54418181	279	54418031	15	7.11172	4.10799
Nc-1-IP_pe	chr1	54419619	54419963	345	54419771	13	5.86991	3.71823
Nc-1-IP_pe	chr1	54420219	54421036	818	54420541	14	6.96097	4.17548
Nc-1-IP_pe	chr1	54423608	54424418	811	54423888	15	8.49563	4.86328
Nc-1-IP_pe	chr1	54424888	54425153	266	54425021	11	5.20969	3.60954
Nc-1-IP_pe	chr1	54430661	54431112	452	54430683	14	4.42328	2.86573
Nc-1-IP_pe	chr1	54448597	54448947	351	54448786	13	6.76819	4.2221
Nc-1-IP_pe	chr1	54457133	54457542	410	54457143	12	4.70082	3.20033
Nc-1-IP_pe	chr1	54458119	54458508	390	54458301	18	8.37884	4.31591
Nc-1-IP_pe	chr1	54462036	54462292	257	54462077	13	4.24481	2.8638
Nc-1-IP_pe	chr1	54471616	54471985	370	54471839	20	11.575	5.57735
Nc-1-IP_pe	chr1	54473032	54473305	274	54473147	13	5.86991	3.71823
Nc-1-IP_pe	chr1	54473853	54474118	266	54474045	13	4.02562	2.7544
Nc-1-IP_pe	chr1	54474617	54475186	570	54474994	22	12.3673	5.5949

Nc-1-IP_pe	chr1	54476415	54476779	365	54476723	13	5.8082	3.68441
Nc-1-IP_pe	chr1	54481631	54481904	274	54481785	20	5.01743	2.69071
Nc-1-IP_pe	chr1	54487835	54488227	393	54488027	19	8.35089	4.16476
Nc-1-IP_pe	chr1	54488983	54489667	685	54489623	14	5.03085	3.16286
Nc-1-IP_pe	chr1	54490743	54491572	830	54490855	15	5.00915	3.05678
Nc-1-IP_pe	chr1	54498581	54499169	589	54498851	16	7.35733	4.09235
Nc-1-IP_pe	chr1	54501375	54501709	335	54501512	17	7.83935	4.1935
Nc-1-IP_pe	chr1	54509692	54509990	299	54509798	15	6.57408	3.82774
Nc-1-IP_pe	chr1	54513487	54514343	857	54513935	12	4.24064	2.95299
Nc-1-IP_pe	chr1	54517599	54517863	265	54517715	13	5.99619	3.78778
Nc-1-IP_pe	chr1	54520006	54520259	254	54520045	17	5.9911	3.31919
Nc-1-IP_pe	chr1	54520659	54521004	346	54520892	9	3.75449	2.977
Nc-1-IP_pe	chr1	54522517	54522790	274	54522637	14	7.42059	4.4313
Nc-1-IP_pe	chr1	54527770	54528145	376	54527996	18	10.7634	5.54224
Nc-1-IP_pe	chr1	54540874	54541262	389	54541048	16	6.80394	3.81462
Nc-1-IP_pe	chr1	54541745	54542833	1089	54542030	18	9.3582	4.80363
Nc-1-IP_pe	chr1	54544979	54545279	301	54545034	12	4.35991	3.0165
Nc-1-IP_pe	chr1	54547594	54548131	538	54547976	18	8.33933	4.29672
Nc-1-IP_pe	chr1	54558551	54558935	385	54558754	13	5.86991	3.71823
Nc-1-IP_pe	chr1	54561587	54562693	1107	54561733	21	8.10596	3.8173
Nc-1-IP_pe	chr1	54564704	54565080	377	54564938	18	6.65364	3.51391
Nc-1-IP_pe	chr1	54567507	54568036	530	54567556	13	3.67377	2.58159
Nc-1-IP_pe	chr1	54577866	54578296	431	54578185	15	6.2617	3.66848
Nc-1-IP_pe	chr1	54583075	54583484	410	54583233	12	4.33734	3.00445
Nc-1-IP_pe	chr1	54585059	54585816	758	54585661	16	4.96462	2.94973
Nc-1-IP_pe	chr1	54593792	54594077	286	54593870	13	5.9487	3.76157
Nc-1-IP_pe	chr1	54594487	54594934	448	54594694	19	7.84735	3.93486
Nc-1-IP_pe	chr1	54595887	54596259	373	54596115	14	4.60011	2.95114
Nc-1-IP_pe	chr1	54596698	54597018	321	54596884	13	5.4426	3.4862
Nc-1-IP_pe	chr1	54598154	54598579	426	54598381	13	5.36853	3.44651
Nc-1-IP_pe	chr1	54601308	54601659	352	54601559	16	5.28625	3.09457
Nc-1-IP_pe	chr1	54604818	54605428	611	54605237	18	7.93853	4.10423
Nc-1-IP_pe	chr1	54606841	54607136	296	54606932	12	4.11973	2.88905
Nc-1-IP_pe	chr1	54607554	54607896	343	54607668	14	3.86727	2.6027
Nc-1-IP_pe	chr1	54610052	54610524	473	54610068	11	2.98297	2.37087
Nc-1-IP_pe	chr1	54612469	54612914	446	54612669	15	4.40338	2.77622
Nc-1-IP_pe	chr1	54619662	54620116	455	54619794	14	6.3004	3.81736
Nc-1-IP_pe	chr1	54620458	54620726	269	54620520	12	4.24064	2.95299
Nc-1-IP_pe	chr1	54622098	54622384	287	54622124	11	3.64839	2.72584
Nc-1-IP_pe	chr1	54622779	54623804	1026	54623682	14	6.96097	4.17548
Nc-1-IP_pe	chr1	54624305	54625019	715	54624739	15	4.40338	2.77622
Nc-1-IP_pe	chr1	54625770	54626156	387	54625868	13	5.86991	3.71823
Nc-1-IP_pe	chr1	54660531	54660789	259	54660613	15	8.22991	4.71468
Nc-1-IP_pe	chr1	54662075	54662385	311	54662235	13	4.86362	3.18015
Nc-1-IP_pe	chr1	54678832	54679533	702	54679360	17	4.78101	2.79343

Nc-1-IP_pe	chr1	54685176	54685528	353	54685264	13	4.34711	2.91533
Nc-1-IP_pe	chr1	54688617	54689117	501	54688811	13	5.47064	3.50127
Nc-1-IP_pe	chr1	54692147	54692644	498	54692520	16	4.96462	2.94973
Nc-1-IP_pe	chr1	54693640	54694065	426	54693715	15	6.24734	3.66122
Nc-1-IP_pe	chr1	54705106	54705729	624	54705727	11	2.96397	2.36091
Nc-1-IP_pe	chr1	54706520	54706886	367	54706689	12	4.69273	3.19593
Nc-1-IP_pe	chr1	54708543	54708878	336	54708549	13	3.35769	2.42919
Nc-1-IP_pe	chr1	54714645	54715331	687	54715051	13	4.78908	3.14145
Nc-1-IP_pe	chr1	54715719	54716169	451	54715952	17	5.14803	2.94914
Nc-1-IP_pe	chr1	54734091	54734627	537	54734503	9	3.27326	2.68684
Nc-1-IP_pe	chr1	54736986	54737239	254	54737177	14	4.73128	3.01506
Nc-1-IP_pe	chr1	54739164	54739484	321	54739350	14	6.04069	3.67979
Nc-1-IP_pe	chr1	54747726	54748458	733	54748361	14	5.51541	3.4073
Nc-1-IP_pe	chr1	54753947	54755194	1248	54754429	19	9.03708	4.48779
Nc-1-IP_pe	chr1	54757814	54758221	408	54758063	11	3.28475	2.53029
Nc-1-IP_pe	chr1	54761641	54761893	253	54761781	16	6.51374	3.67221
Nc-1-IP_pe	chr1	54781275	54781741	467	54781574	17	5.54967	3.12324
Nc-1-IP_pe	chr1	54787635	54787959	325	54787866	17	5.14803	2.94914
Nc-1-IP_pe	chr1	54792532	54793294	763	54793077	15	5.20142	3.14789
Nc-1-IP_pe	chr1	54795733	54796287	555	54795958	15	5.58327	3.33181
Nc-1-IP_pe	chr1	54799116	54799815	700	54799631	16	5.82808	3.34463
Nc-1-IP_pe	chr1	54800602	54801260	659	54800806	22	7.04484	3.3034
Nc-1-IP_pe	chr1	54804882	54805214	333	54804993	19	7.4805	3.77117
Nc-1-IP_pe	chr1	54815803	54816379	577	54816250	15	5.20142	3.14789
Nc-1-IP_pe	chr1	54819275	54819740	466	54819468	15	7.56326	4.34922
Nc-1-IP_pe	chr1	54825861	54826231	371	54826103	14	4.95172	3.12357
Nc-1-IP_pe	chr1	54835708	54836071	364	54836012	10	3.08907	2.49869
Nc-1-IP_pe	chr1	54846379	54846851	473	54846538	15	5.58327	3.33181
Nc-1-IP_pe	chr1	54848309	54848849	541	54848335	14	5.03085	3.16286
Nc-1-IP_pe	chr1	54851428	54851810	383	54851620	18	7.62523	3.95652
Nc-1-IP_pe	chr1	54852231	54852503	273	54852309	16	6.48567	3.65855
Nc-1-IP_pe	chr1	54853305	54854255	951	54853628	19	6.78661	3.47027
Nc-1-IP_pe	chr1	54854667	54855580	914	54855275	17	6.76692	3.67553
Nc-1-IP_pe	chr1	54860166	54860421	256	54860340	18	4.32723	2.54542
Nc-1-IP_pe	chr1	54861755	54862240	486	54862223	12	3.83688	2.74114
Nc-1-IP_pe	chr1	54865570	54866160	591	54865996	11	4.03422	2.9376
Nc-1-IP_pe	chr1	54866541	54867330	790	54866923	15	5.66913	3.37372
Nc-1-IP_pe	chr1	54872149	54872662	514	54872186	13	4.02562	2.7544
Nc-1-IP_pe	chr1	54918500	54919214	715	54919136	13	3.67377	2.58159
Nc-1-IP_pe	chr1	54923596	54924060	465	54923990	16	6.33285	3.58457
Nc-1-IP_pe	chr1	54924934	54925234	301	54925189	13	5.36853	3.44651
Nc-1-IP_pe	chr1	54925871	54926146	276	54925948	11	4.41492	3.1509
Nc-1-IP_pe	chr1	54945620	54945907	288	54945717	13	3.67377	2.58159
Nc-1-IP_pe	chr1	54949871	54950574	704	54950344	15	4.40338	2.77622
Nc-1-IP_pe	chr1	54972328	54972720	393	54972597	13	3.67377	2.58159

Nc-1-IP_pe	chr1	54980564	54981720	1157	54980807	22	6.72623	3.18481
Nc-1-IP_pe	chr1	54985449	54986598	1150	54986346	18	8.10208	4.1823
Nc-1-IP_pe	chr1	54988271	54988552	282	54988320	13	4.86362	3.18015
Nc-1-IP_pe	chr1	54990388	54991068	681	54990513	17	6.51104	3.5563
Nc-1-IP_pe	chr1	54994165	54994816	652	54994740	16	6.28027	3.55926
Nc-1-IP_pe	chr1	54995555	54996367	813	54996126	21	7.34645	3.50809
Nc-1-IP_pe	chr1	54997073	54997370	298	54997249	20	6.49719	3.259
Nc-1-IP_pe	chr1	54998775	54999218	444	54998799	14	4.8399	3.06836
Nc-1-IP_pe	chr1	55000818	55001365	548	55001205	17	6.47874	3.54137
Nc-1-IP_pe	chr1	55005823	55006207	385	55006016	20	7.97282	3.87239
Nc-1-IP_pe	chr1	55009004	55009538	535	55009185	14	4.88423	3.09021
Nc-1-IP_pe	chr1	55013799	55014052	254	55013996	16	5.85814	3.35873
Nc-1-IP_pe	chr1	55015097	55015466	370	55015404	12	3.98572	2.81868
Nc-1-IP_pe	chr1	55020086	55020414	329	55020275	12	4.00598	2.82928
Nc-1-IP_pe	chr1	55022327	55023338	1012	55022506	18	4.32723	2.54542
Nc-1-IP_pe	chr1	55029909	55030420	512	55030292	14	4.60011	2.95114
Nc-1-IP_pe	chr1	55037751	55038380	630	55037963	17	7.32147	3.9397
Nc-1-IP_pe	chr1	55039189	55039803	615	55039601	26	6.47412	2.84038
Nc-1-IP_pe	chr1	55040360	55040949	590	55040825	21	6.66991	3.24296
Nc-1-IP_pe	chr1	55044703	55045066	364	55044993	16	6.28027	3.55926
Nc-1-IP_pe	chr1	55046055	55046328	274	55046152	14	4.8399	3.06836
Nc-1-IP_pe	chr1	55047931	55048359	429	55047959	14	4.23163	2.77414
Nc-1-IP_pe	chr1	55049119	55049522	404	55049422	14	4.21449	2.76599
Nc-1-IP_pe	chr1	55049880	55050515	636	55050229	17	4.78101	2.79343
Nc-1-IP_pe	chr1	55051153	55051662	510	55051438	12	4.24856	2.9572
Nc-1-IP_pe	chr1	55053486	55054754	1269	55053581	16	6.84901	3.83694
Nc-1-IP_pe	chr1	55058883	55059950	1068	55059753	16	5.88264	3.37024
Nc-1-IP_pe	chr1	55061046	55061660	615	55061600	15	4.06016	2.62146
Nc-1-IP_pe	chr1	55063133	55063471	339	55063314	15	5.1177	3.10809
Nc-1-IP_pe	chr1	55182103	55182356	254	55182217	15	7.46994	4.29894
Nc-1-IP_pe	chr1	55208418	55208807	390	55208486	12	4.75569	3.23024
Nc-1-IP_pe	chr1	55214596	55215552	957	55214887	21	5.5349	2.81884
Nc-1-IP_pe	chr1	55252004	55252268	265	55252126	10	5.23357	3.76063
Nc-1-IP_pe	chr1	55260865	55261119	255	55260905	8	3.51085	2.92785
Nc-1-IP_pe	chr1	55273162	55273454	293	55273321	13	6.62436	4.14003
Nc-1-IP_pe	chr1	55307832	55308142	311	55307881	10	3.89064	2.95552
Nc-1-IP_pe	chr1	55313091	55313499	409	55313269	10	4.99888	3.61677
Nc-1-IP_pe	chr1	55343847	55344146	300	55343954	15	7.37761	4.24941
Nc-1-IP_pe	chr1	55377697	55378043	347	55377926	10	5.23357	3.76063
Nc-1-IP_pe	chr1	55399628	55400014	387	55399700	10	5.23357	3.76063
Nc-1-IP_pe	chr1	55431067	55431322	256	55431150	11	4.74697	3.34038
Nc-1-IP_pe	chr1	55476401	55476678	278	55476592	12	5.15877	3.45264
Nc-1-IP_pe	chr1	55491153	55491597	445	55491203	14	6.69566	4.03025
Nc-1-IP_pe	chr1	55507503	55507863	361	55507674	10	5.23357	3.76063
Nc-1-IP_pe	chr1	55558438	55558852	415	55558512	7	2.9417	2.65478

Nc-1-IP_pe	chr1	55574546	55574817	272	55574698	11	3.64839	2.72584
Nc-1-IP_pe	chr1	55582398	55582739	342	55582591	10	4.93609	3.57849
Nc-1-IP_pe	chr1	55745583	55745882	300	55745707	11	6.03439	4.10251
Nc-1-IP_pe	chr1	55760246	55760951	706	55760626	9	4.46937	3.41876
Nc-1-IP_pe	chr1	55783240	55783666	427	55783564	10	4.33501	3.21677
Nc-1-IP_pe	chr1	55788335	55788599	265	55788399	8	3.20603	2.7356
Nc-1-IP_pe	chr1	55793728	55794250	523	55794126	12	5.84943	3.84431
Nc-1-IP_pe	chr1	55912691	55912978	288	55912835	13	4.41954	2.952
Nc-1-IP_pe	chr1	55913390	55913642	253	55913560	11	4.06403	2.95415
Nc-1-IP_pe	chr1	55971801	55972080	280	55972070	8	3.64414	3.01254
Nc-1-IP_pe	chr1	56022135	56022467	333	56022346	11	6.03439	4.10251
Nc-1-IP_pe	chr1	56062661	56062937	277	56062809	14	7.14701	4.27839
Nc-1-IP_pe	chr1	56089963	56090445	483	56090306	16	6.95583	3.89005
Nc-1-IP_pe	chr1	56126221	56126500	280	56126368	11	5.77463	3.94557
Nc-1-IP_pe	chr1	56170925	56171316	392	56171093	11	6.03439	4.10251
Nc-1-IP_pe	chr1	56242391	56242649	259	56242522	15	9.55381	5.47001
Nc-1-IP_pe	chr1	56340359	56340736	378	56340588	14	5.30793	3.30182
Nc-1-IP_pe	chr1	56342540	56343259	720	56343068	12	6.31185	4.11348
Nc-1-IP_pe	chr1	56391948	56392217	270	56392079	13	7.7352	4.78626
Nc-1-IP_pe	chr1	56476477	56476808	332	56476596	13	7.63554	4.7272
Nc-1-IP_pe	chr1	56494350	56494703	354	56494598	11	6.03439	4.10251
Nc-1-IP_pe	chr1	56506241	56506941	701	56506416	10	3.08907	2.49869
Nc-1-IP_pe	chr1	56533069	56533343	275	56533192	8	3.56021	2.95918
Nc-1-IP_pe	chr1	56577825	56578362	538	56578316	10	3.83201	2.92147
Nc-1-IP_pe	chr1	56578797	56579385	589	56578890	15	4.40338	2.77622
Nc-1-IP_pe	chr1	56595074	56595351	278	56595138	12	6.86906	4.44438
Nc-1-IP_pe	chr1	56645388	56645908	521	56645514	15	5.83691	3.4562
Nc-1-IP_pe	chr1	56690198	56690471	274	56690312	10	5.23357	3.76063
Nc-1-IP_pe	chr1	56788732	56789103	372	56789011	12	5.3037	3.53374
Nc-1-IP_pe	chr1	56819313	56819657	345	56819565	12	4.70082	3.20033
Nc-1-IP_pe	chr1	56876060	56876339	280	56876179	11	5.10577	3.5486
Nc-1-IP_pe	chr1	56921467	56921731	265	56921692	12	5.23082	3.49289
Nc-1-IP_pe	chr1	56924294	56924802	509	56924708	9	4.46937	3.41876
Nc-1-IP_pe	chr1	57025012	57025277	266	57025135	10	5.23357	3.76063
Nc-1-IP_pe	chr1	57045310	57045646	337	57045545	10	4.99888	3.61677
Nc-1-IP_pe	chr1	57116023	57116390	368	57116180	9	4.46937	3.41876
Nc-1-IP_pe	chr1	57207466	57207745	280	57207631	11	6.03439	4.10251
Nc-1-IP_pe	chr1	57273469	57273846	378	57273546	10	5.23357	3.76063
Nc-1-IP_pe	chr1	57319063	57319355	293	57319115	10	4.39571	3.25288
Nc-1-IP_pe	chr1	57343306	57343690	385	57343457	13	6.62436	4.14003
Nc-1-IP_pe	chr1	57563657	57564112	456	57563959	11	4.06403	2.95415
Nc-1-IP_pe	chr1	57616991	57617270	280	57617028	8	3.74505	3.07688
Nc-1-IP_pe	chr1	57620664	57620922	259	57620782	10	4.39571	3.25288
Nc-1-IP_pe	chr1	57683049	57683382	334	57683213	11	6.03439	4.10251
Nc-1-IP_pe	chr1	57708110	57708657	548	57708654	12	2.87625	2.25568

Nc-1-IP_pe	chr1	57918521	57918807	287	57918610	10	5.23357	3.76063
Nc-1-IP_pe	chr1	58017555	58017827	273	58017619	10	3.89064	2.95552
Nc-1-IP_pe	chr1	58065729	58066041	313	58065982	8	3.74505	3.07688
Nc-1-IP_pe	chr1	58131757	58132245	489	58132220	11	3.64839	2.72584
Nc-1-IP_pe	chr1	58135838	58136326	489	58135953	10	5.23357	3.76063
Nc-1-IP_pe	chr1	58189498	58189897	400	58189625	11	6.03439	4.10251
Nc-1-IP_pe	chr1	58193495	58193802	308	58193762	9	3.27326	2.68684
Nc-1-IP_pe	chr1	58219382	58219644	263	58219644	11	2.96397	2.36091
Nc-1-IP_pe	chr1	58321367	58321639	273	58321441	9	4.46937	3.41876
Nc-1-IP_pe	chr1	58328326	58328762	437	58328468	13	5.71736	3.63481
Nc-1-IP_pe	chr1	58378557	58378830	274	58378647	19	7.84735	3.93486
Nc-1-IP_pe	chr1	58407140	58407695	556	58407269	9	4.46937	3.41876
Nc-1-IP_pe	chr1	58432603	58432865	263	58432688	11	5.89269	4.01672
Nc-1-IP_pe	chr1	58463204	58464003	800	58463666	10	5.23357	3.76063
Nc-1-IP_pe	chr1	58465981	58466264	284	58466137	10	5.23357	3.76063
Nc-1-IP_pe	chr1	58470284	58470566	283	58470427	12	5.15877	3.45264
Nc-1-IP_pe	chr1	58489613	58489878	266	58489764	13	6.72991	4.20021
Nc-1-IP_pe	chr1	58576113	58577641	1529	58576724	19	11.1811	5.5673
Nc-1-IP_pe	chr1	58590239	58590577	339	58590328	16	6.8987	3.86161
Nc-1-IP_pe	chr1	58597801	58598375	575	58598193	11	6.03439	4.10251
Nc-1-IP_pe	chr1	58623543	58623990	448	58623875	15	7.95672	4.56362
Nc-1-IP_pe	chr1	58630818	58631137	320	58630890	21	6.66991	3.24296
Nc-1-IP_pe	chr1	58691006	58691273	268	58691082	9	4.46937	3.41876
Nc-1-IP_pe	chr1	58699739	58700083	345	58699920	18	6.15736	3.29676
Nc-1-IP_pe	chr1	58700346	58700604	259	58700485	17	8.02964	4.28847
Nc-1-IP_pe	chr1	58714699	58714966	268	58714777	9	4.46937	3.41876
Nc-1-IP_pe	chr1	58721396	58721771	376	58721567	9	4.35492	3.34727
Nc-1-IP_pe	chr1	58735219	58735594	376	58735297	11	5.03835	3.5092
Nc-1-IP_pe	chr1	58747764	58748183	420	58747880	14	7.86294	4.68231
Nc-1-IP_pe	chr1	58784694	58784963	270	58784930	12	3.67829	2.65924
Nc-1-IP_pe	chr1	58808390	58808666	277	58808461	10	3.89064	2.95552
Nc-1-IP_pe	chr1	58815322	58815600	279	58815409	14	6.96097	4.17548
Nc-1-IP_pe	chr1	58837276	58837535	260	58837470	9	3.53727	2.84525
Nc-1-IP_pe	chr1	58893213	58893711	499	58893608	10	3.4604	2.70797
Nc-1-IP_pe	chr1	58896413	58896669	257	58896468	10	3.89064	2.95552
Nc-1-IP_pe	chr1	58899396	58899699	304	58899479	9	4.25966	3.28797
Nc-1-IP_pe	chr1	58903780	58904347	568	58904032	14	6.44441	3.89444
Nc-1-IP_pe	chr1	58935728	58936042	315	58935947	16	6.8987	3.86161
Nc-1-IP_pe	chr1	58965575	58965830	256	58965683	13	7.0664	4.39386
Nc-1-IP_pe	chr1	58971784	58972063	280	58971848	12	5.23082	3.49289
Nc-1-IP_pe	chr1	59015679	59015988	310	59015890	12	5.23082	3.49289
Nc-1-IP_pe	chr1	59106107	59106436	330	59106261	9	4.46937	3.41876
Nc-1-IP_pe	chr1	59146633	59146987	355	59146748	11	4.35236	3.11555
Nc-1-IP_pe	chr1	59260429	59260711	283	59260650	8	3.74505	3.07688
Nc-1-IP_pe	chr1	59263528	59263792	265	59263664	12	6.86906	4.44438

Nc-1-IP_pe	chr1	59283272	59283553	282	59283369	11	5.52654	3.79706
Nc-1-IP_pe	chr1	59369016	59369578	563	59369018	16	4.25252	2.63824
Nc-1-IP_pe	chr1	59477273	59477597	325	59477416	16	10.5028	5.81189
Nc-1-IP_pe	chr1	59520868	59521273	406	59521099	11	4.54404	3.2242
Nc-1-IP_pe	chr1	59525060	59525400	341	59525256	11	6.03439	4.10251
Nc-1-IP_pe	chr1	59533067	59533433	367	59533344	11	5.7051	3.90381
Nc-1-IP_pe	chr1	59542687	59542994	308	59542871	11	5.77463	3.94557
Nc-1-IP_pe	chr1	59587296	59587605	310	59587422	13	6.54342	4.09407
Nc-1-IP_pe	chr1	59599289	59599649	361	59599380	12	5.23082	3.49289
Nc-1-IP_pe	chr1	59610920	59611273	354	59610964	11	3.64839	2.72584
Nc-1-IP_pe	chr1	59653254	59653561	308	59653453	14	5.69896	3.50164
Nc-1-IP_pe	chr1	59668213	59668470	258	59668397	11	4.06403	2.95415
Nc-1-IP_pe	chr1	59673838	59674126	289	59673950	12	5.23082	3.49289
Nc-1-IP_pe	chr1	59709803	59710115	313	59709943	15	8.38325	4.80024
Nc-1-IP_pe	chr1	59712319	59712824	506	59712696	13	7.7352	4.78626
Nc-1-IP_pe	chr1	59737006	59737344	339	59737226	14	4.21449	2.76599
Nc-1-IP_pe	chr1	59806350	59806606	257	59806534	9	4.46937	3.41876
Nc-1-IP_pe	chr1	59814849	59815320	472	59815066	23	8.92223	3.93219
Nc-1-IP_pe	chr1	59926469	59926775	307	59926609	14	6.69566	4.03025
Nc-1-IP_pe	chr1	59969938	59970216	279	59969997	11	3.64839	2.72584
Nc-1-IP_pe	chr1	60020218	60020487	270	60020326	10	5.23357	3.76063
Nc-1-IP_pe	chr1	60184605	60185022	418	60184790	12	6.58417	4.27436
Nc-1-IP_pe	chr1	60454494	60454841	348	60454658	11	4.06403	2.95415
Nc-1-IP_pe	chr1	60455185	60456037	853	60455203	11	3.64839	2.72584
Nc-1-IP_pe	chr1	60640032	60640428	397	60640122	12	6.58417	4.27436
Nc-1-IP_pe	chr1	60721520	60721823	304	60721684	9	3.72192	2.95716
Nc-1-IP_pe	chr1	60812989	60813252	264	60813141	10	5.23357	3.76063
Nc-1-IP_pe	chr1	60924638	60925012	375	60924960	17	6.78076	3.68203
Nc-1-IP_pe	chr1	61001138	61001685	548	61001319	11	6.03439	4.10251
Nc-1-IP_pe	chr1	61004250	61004583	334	61004450	10	5.12294	3.69268
Nc-1-IP_pe	chr1	61050016	61051117	1102	61050238	23	11.5921	5.06057
Nc-1-IP_pe	chr1	61053641	61054052	412	61053999	12	5.89413	3.8701
Nc-1-IP_pe	chr1	61074593	61074870	278	61074750	16	7.53857	4.18505
Nc-1-IP_pe	chr1	61083236	61083531	296	61083297	16	4.59224	2.7853
Nc-1-IP_pe	chr1	61126915	61127350	436	61127030	10	4.99888	3.61677
Nc-1-IP_pe	chr1	61213752	61214013	262	61213919	12	6.86906	4.44438
Nc-1-IP_pe	chr1	61358508	61358788	281	61358621	10	4.93609	3.57849
Nc-1-IP_pe	chr1	61364889	61365206	318	61365132	11	6.03439	4.10251
Nc-1-IP_pe	chr1	61494257	61494567	311	61494423	10	5.17577	3.72509
Nc-1-IP_pe	chr1	61634516	61634801	286	61634537	9	4.46937	3.41876
Nc-1-IP_pe	chr1	61682507	61682822	316	61682696	13	6.62436	4.14003
Nc-1-IP_pe	chr1	61688399	61688713	315	61688576	13	5.36853	3.44651
Nc-1-IP_pe	chr1	61703930	61704210	281	61704077	10	5.23357	3.76063
Nc-1-IP_pe	chr1	61704502	61704769	268	61704575	12	6.86906	4.44438
Nc-1-IP_pe	chr1	61720988	61721507	520	61721144	11	6.03439	4.10251

Nc-1-IP_pe	chr1	61742994	61743260	267	61743115	13	4.56123	3.02417
Nc-1-IP_pe	chr1	61759351	61759629	279	61759558	11	6.03439	4.10251
Nc-1-IP_pe	chr1	61790705	61790991	287	61790825	10	5.23357	3.76063
Nc-1-IP_pe	chr1	61809404	61809656	253	61809515	9	4.46937	3.41876
Nc-1-IP_pe	chr1	61812459	61812866	408	61812596	9	4.46937	3.41876
Nc-1-IP_pe	chr1	61858329	61858692	364	61858425	11	5.10577	3.5486
Nc-1-IP_pe	chr1	61861911	61862284	374	61862099	12	6.58417	4.27436
Nc-1-IP_pe	chr1	61869727	61870033	307	61869833	9	2.89249	2.46179
Nc-1-IP_pe	chr1	61881430	61881742	313	61881620	12	6.86906	4.44438
Nc-1-IP_pe	chr1	61903835	61904092	258	61903880	11	6.03439	4.10251
Nc-1-IP_pe	chr1	61930620	61931023	404	61930757	11	6.03439	4.10251
Nc-1-IP_pe	chr1	61937716	61937989	274	61937913	9	2.89249	2.46179
Nc-1-IP_pe	chr1	61949767	61950149	383	61950072	16	5.37454	3.13479
Nc-1-IP_pe	chr1	62014641	62015688	1048	62015111	13	7.7352	4.78626
Nc-1-IP_pe	chr1	62086885	62087235	351	62087093	10	4.93609	3.57849
Nc-1-IP_pe	chr1	62134602	62134879	278	62134760	12	4.24064	2.95299
Nc-1-IP_pe	chr1	62139226	62139513	288	62139476	8	3.56021	2.95918
Nc-1-IP_pe	chr1	62158407	62158676	270	62158494	9	4.46937	3.41876
Nc-1-IP_pe	chr1	62159687	62159953	267	62159901	12	6.86906	4.44438
Nc-1-IP_pe	chr1	62164883	62165349	467	62165103	9	4.46937	3.41876
Nc-1-IP_pe	chr1	62166426	62166699	274	62166584	9	4.32403	3.32802
Nc-1-IP_pe	chr1	62173798	62174119	322	62174016	12	4.24064	2.95299
Nc-1-IP_pe	chr1	62177198	62177585	388	62177397	12	6.86906	4.44438
Nc-1-IP_pe	chr1	62185293	62185621	329	62185502	11	4.54404	3.2242
Nc-1-IP_pe	chr1	62194438	62195290	853	62195234	13	6.54342	4.09407
Nc-1-IP_pe	chr1	62216512	62216804	293	62216634	8	3.33547	2.81699
Nc-1-IP_pe	chr1	62217360	62217965	606	62217453	10	4.39571	3.25288
Nc-1-IP_pe	chr1	62227747	62228058	312	62227951	18	5.33197	2.94862
Nc-1-IP_pe	chr1	62242894	62243654	761	62243058	12	6.50787	4.22912
Nc-1-IP_pe	chr1	62249634	62250082	449	62249958	11	4.54404	3.2242
Nc-1-IP_pe	chr1	62253764	62254042	279	62253943	12	6.58898	4.27722
Nc-1-IP_pe	chr1	62264143	62264408	266	62264184	11	5.3232	3.67643
Nc-1-IP_pe	chr1	62268231	62269110	880	62269037	14	6.06519	3.69269
Nc-1-IP_pe	chr1	62273486	62273977	492	62273745	11	3.64839	2.72584
Nc-1-IP_pe	chr1	62291571	62291870	300	62291652	9	4.46937	3.41876
Nc-1-IP_pe	chr1	62294442	62294871	430	62294803	10	5.23357	3.76063
Nc-1-IP_pe	chr1	62308431	62309149	719	62308770	15	8.26005	4.73146
Nc-1-IP_pe	chr1	62310098	62310532	435	62310245	12	4.75569	3.23024
Nc-1-IP_pe	chr1	62318306	62318874	569	62318633	13	6.54342	4.09407
Nc-1-IP_pe	chr1	62327185	62327524	340	62327295	14	6.06519	3.69269
Nc-1-IP_pe	chr1	62361430	62361748	319	62361450	8	3.61691	2.99521
Nc-1-IP_pe	chr1	62377310	62377631	322	62377499	12	5.84943	3.84431
Nc-1-IP_pe	chr1	62414893	62415272	380	62415155	10	4.39571	3.25288
Nc-1-IP_pe	chr1	62436813	62437281	469	62437257	14	3.96259	2.64722
Nc-1-IP_pe	chr1	62453628	62454296	669	62453637	14	4.21449	2.76599

Nc-1-IP_pe	chr1	62562281	62562549	269	62562442	13	7.7352	4.78626
Nc-1-IP_pe	chr1	62562898	62563155	258	62562986	12	6.86906	4.44438
Nc-1-IP_pe	chr1	62574905	62575194	290	62575089	9	3.72192	2.95716
Nc-1-IP_pe	chr1	62641292	62641547	256	62641379	10	4.99888	3.61677
Nc-1-IP_pe	chr1	62687641	62688394	754	62687919	16	8.26996	4.56762
Nc-1-IP_pe	chr1	62704549	62705420	872	62704668	9	3.79246	3.00015
Nc-1-IP_pe	chr1	62706635	62706894	260	62706662	10	3.89064	2.95552
Nc-1-IP_pe	chr1	62761249	62761943	695	62761453	19	10.4598	5.19259
Nc-1-IP_pe	chr1	62798778	62799032	255	62798900	10	5.23357	3.76063
Nc-1-IP_pe	chr1	62815547	62815886	340	62815685	13	7.7352	4.78626
Nc-1-IP_pe	chr1	62826189	62826486	298	62826309	12	5.84943	3.84431
Nc-1-IP_pe	chr1	62945999	62946281	283	62946188	11	5.77463	3.94557
Nc-1-IP_pe	chr1	62964378	62964667	290	62964543	12	6.58417	4.27436
Nc-1-IP_pe	chr1	62967368	62967809	442	62967393	12	5.94619	3.9002
Nc-1-IP_pe	chr1	63027393	63027868	476	63027765	10	5.23357	3.76063
Nc-1-IP_pe	chr1	63028526	63028974	449	63028952	11	3.64839	2.72584
Nc-1-IP_pe	chr1	63073802	63074079	278	63073997	9	4.46937	3.41876
Nc-1-IP_pe	chr1	63120341	63120601	261	63120447	15	6.78905	3.93887
Nc-1-IP_pe	chr1	63286568	63286846	279	63286684	12	5.84943	3.84431
Nc-1-IP_pe	chr1	63287129	63287425	297	63287282	9	4.46937	3.41876
Nc-1-IP_pe	chr1	63292466	63292753	288	63292679	12	6.50787	4.22912
Nc-1-IP_pe	chr1	63297838	63298255	418	63298093	8	3.56021	2.95918
Nc-1-IP_pe	chr1	63316829	63317558	730	63317548	15	4.40338	2.77622
Nc-1-IP_pe	chr1	63323371	63323664	294	63323412	11	4.54404	3.2242
Nc-1-IP_pe	chr1	63324183	63324633	451	63324585	11	3.28475	2.53029
Nc-1-IP_pe	chr1	63329555	63329981	427	63329685	9	3.92293	3.08
Nc-1-IP_pe	chr1	63347601	63347936	336	63347745	10	4.99888	3.61677
Nc-1-IP_pe	chr1	63363965	63364232	268	63364144	10	4.68196	3.42446
Nc-1-IP_pe	chr1	63367237	63367723	487	63367424	14	4.13959	2.7305
Nc-1-IP_pe	chr1	63473832	63474291	460	63473968	13	5.57038	3.55505
Nc-1-IP_pe	chr1	63544166	63544656	491	63544578	9	4.46937	3.41876
Nc-1-IP_pe	chr1	63545102	63545689	588	63545583	15	9.55381	5.47001
Nc-1-IP_pe	chr1	63611641	63612185	545	63611988	10	5.23357	3.76063
Nc-1-IP_pe	chr1	63617299	63617591	293	63617484	13	7.7352	4.78626
Nc-1-IP_pe	chr1	63643588	63644301	714	63644200	11	6.03439	4.10251
Nc-1-IP_pe	chr1	63653643	63653902	260	63653796	9	4.46937	3.41876
Nc-1-IP_pe	chr1	63674611	63674877	267	63674705	12	3.83688	2.74114
Nc-1-IP_pe	chr1	63728743	63729037	295	63728776	13	3.67377	2.58159
Nc-1-IP_pe	chr1	63740558	63740907	350	63740756	14	7.54286	4.50023
Nc-1-IP_pe	chr1	63766691	63767131	441	63766840	15	6.78905	3.93887
Nc-1-IP_pe	chr1	63774560	63775117	558	63774737	15	4.70031	2.91253
Nc-1-IP_pe	chr1	63823633	63823934	302	63823858	9	4.46937	3.41876
Nc-1-IP_pe	chr1	63831030	63831409	380	63831175	13	6.54342	4.09407
Nc-1-IP_pe	chr1	63843107	63843402	296	63843289	16	6.8987	3.86161
Nc-1-IP_pe	chr1	63955872	63956125	254	63955996	11	5.02318	3.50036

Nc-1-IP_pe	chr1	64008631	64008927	297	64008813	10	4.99888	3.61677
Nc-1-IP_pe	chr1	64054128	64054397	270	64054371	8	3.74505	3.07688
Nc-1-IP_pe	chr1	64064314	64064692	379	64064620	10	4.39571	3.25288
Nc-1-IP_pe	chr1	64189960	64190215	256	64190134	10	5.00283	3.61918
Nc-1-IP_pe	chr1	64191798	64192418	621	64192008	10	3.08907	2.49869
Nc-1-IP_pe	chr1	64196411	64196752	342	64196544	10	5.14046	3.70342
Nc-1-IP_pe	chr1	64200503	64200999	497	64200992	11	3.28475	2.53029
Nc-1-IP_pe	chr1	64201286	64201809	524	64201482	12	4.70082	3.20033
Nc-1-IP_pe	chr1	64203514	64203825	312	64203675	12	6.86906	4.44438
Nc-1-IP_pe	chr1	64224377	64224718	342	64224504	12	6.58417	4.27436
Nc-1-IP_pe	chr1	64356956	64357299	344	64357042	10	5.05378	3.65032
Nc-1-IP_pe	chr1	64414375	64416024	1650	64414599	14	6.06519	3.69269
Nc-1-IP_pe	chr1	64491581	64491903	323	64491591	11	5.10577	3.5486
Nc-1-IP_pe	chr1	64663296	64663878	583	64663712	10	5.23357	3.76063
Nc-1-IP_pe	chr1	64700685	64700945	261	64700858	10	5.05378	3.65032
Nc-1-IP_pe	chr1	64745393	64745904	512	64745847	11	4.54404	3.2242
Nc-1-IP_pe	chr1	64792957	64793223	267	64793014	12	4.24064	2.95299
Nc-1-IP_pe	chr1	64845562	64845955	394	64845872	13	4.86362	3.18015
Nc-1-IP_pe	chr1	64849208	64849587	380	64849467	10	4.39571	3.25288
Nc-1-IP_pe	chr1	64854501	64854982	482	64854775	13	5.9487	3.76157
Nc-1-IP_pe	chr1	64873288	64873690	403	64873529	12	6.31185	4.11348
Nc-1-IP_pe	chr1	64896785	64897261	477	64897210	13	7.38599	4.58019
Nc-1-IP_pe	chr1	64957627	64957922	296	64957851	14	5.43414	3.36584
Nc-1-IP_pe	chr1	64965616	64965873	258	64965638	13	4.14549	2.81406
Nc-1-IP_pe	chr1	64975213	64975543	331	64975428	12	6.86906	4.44438
Nc-1-IP_pe	chr1	65000040	65000425	386	65000131	12	6.23601	4.06898
Nc-1-IP_pe	chr1	65001298	65001620	323	65001438	14	5.03085	3.16286
Nc-1-IP_pe	chr1	65027101	65027789	689	65027726	10	3.89064	2.95552
Nc-1-IP_pe	chr1	65051982	65052587	606	65052122	10	5.17577	3.72509
Nc-1-IP_pe	chr1	65066568	65067416	849	65067212	17	7.6273	4.08876
Nc-1-IP_pe	chr1	65096063	65096330	268	65096264	10	5.23357	3.76063
Nc-1-IP_pe	chr1	65103078	65103976	899	65103747	13	7.34205	4.55444
Nc-1-IP_pe	chr1	65111313	65111734	422	65111458	16	10.117	5.58955
Nc-1-IP_pe	chr1	65129312	65129737	426	65129434	11	4.54404	3.2242
Nc-1-IP_pe	chr1	65148637	65149034	398	65148735	18	7.1522	3.73811
Nc-1-IP_pe	chr1	65241620	65241877	258	65241762	10	5.23357	3.76063
Nc-1-IP_pe	chr1	65281667	65282115	449	65282082	10	4.60647	3.37901
Nc-1-IP_pe	chr1	65357638	65358070	433	65357669	9	4.46937	3.41876
Nc-1-IP_pe	chr1	65525776	65526197	422	65526067	15	5.66913	3.37372
Nc-1-IP_pe	chr1	65550141	65550873	733	65550471	14	5.51541	3.4073
Nc-1-IP_pe	chr1	65575982	65576245	264	65576077	10	5.23357	3.76063
Nc-1-IP_pe	chr1	65643298	65643632	335	65643314	9	3.27326	2.68684
Nc-1-IP_pe	chr1	65646473	65646743	271	65646667	9	4.46937	3.41876
Nc-1-IP_pe	chr1	65700257	65700521	265	65700405	12	6.86906	4.44438
Nc-1-IP_pe	chr1	65728086	65728441	356	65728257	11	6.03439	4.10251

Nc-1-IP_pe	chr1	65745148	65745487	340	65745245	11	4.54404	3.2242
Nc-1-IP_pe	chr1	65792226	65792660	435	65792484	12	6.86906	4.44438
Nc-1-IP_pe	chr1	66190310	66190725	416	66190592	9	4.46937	3.41876
Nc-1-IP_pe	chr1	66314386	66314643	258	66314471	9	4.46937	3.41876
Nc-1-IP_pe	chr1	66337331	66337670	340	66337511	15	9.55381	5.47001
Nc-1-IP_pe	chr1	66382067	66382390	324	66382189	9	4.46937	3.41876
Nc-1-IP_pe	chr1	66389620	66389892	273	66389684	11	6.03439	4.10251
Nc-1-IP_pe	chr1	66436892	66437164	273	66437052	12	5.23082	3.49289
Nc-1-IP_pe	chr1	66477399	66477725	327	66477563	9	4.20361	3.25317
Nc-1-IP_pe	chr1	66481116	66481410	295	66481232	13	6.62436	4.14003
Nc-1-IP_pe	chr1	66505381	66505682	302	66505456	10	4.99888	3.61677
Nc-1-IP_pe	chr1	66510179	66510452	274	66510222	12	4.24064	2.95299
Nc-1-IP_pe	chr1	66615001	66615356	356	66615167	15	7.46994	4.29894
Nc-1-IP_pe	chr1	66734458	66734759	302	66734731	9	4.46937	3.41876
Nc-1-IP_pe	chr1	66913699	66913970	272	66913850	11	4.06403	2.95415
Nc-1-IP_pe	chr1	66924190	66925082	893	66925017	13	4.90859	3.20357
Nc-1-IP_pe	chr1	66930213	66930700	488	66930424	16	10.2925	5.69036
Nc-1-IP_pe	chr1	66976016	66976291	276	66976183	10	5.23357	3.76063
Nc-1-IP_pe	chr1	66996362	66996655	294	66996471	16	10.117	5.58955
Nc-1-IP_pe	chr1	67112968	67113230	263	67113174	12	6.86906	4.44438
Nc-1-IP_pe	chr1	67181933	67182224	292	67182192	10	4.39571	3.25288
Nc-1-IP_pe	chr1	67183995	67184252	258	67184157	12	6.31185	4.11348
Nc-1-IP_pe	chr1	67208343	67208818	476	67208582	13	7.7352	4.78626
Nc-1-IP_pe	chr1	67212657	67212994	338	67212749	7	3.06442	2.73501
Nc-1-IP_pe	chr1	67289335	67289622	288	67289438	8	3.74505	3.07688
Nc-1-IP_pe	chr1	67307699	67307967	269	67307832	10	3.83201	2.92147
Nc-1-IP_pe	chr1	67393457	67393714	258	67393575	12	3.83688	2.74114
Nc-1-IP_pe	chr1	67395710	67396064	355	67395969	11	5.19376	3.60018
Nc-1-IP_pe	chr1	67401397	67401699	303	67401538	12	6.86906	4.44438
Nc-1-IP_pe	chr1	67409011	67409293	283	67409125	13	7.7352	4.78626
Nc-1-IP_pe	chr1	67429136	67429802	667	67429714	13	5.15976	3.33548
Nc-1-IP_pe	chr1	67480033	67480297	265	67480093	7	3.06442	2.73501
Nc-1-IP_pe	chr1	67490615	67491169	555	67490780	13	5.36853	3.44651
Nc-1-IP_pe	chr1	67524790	67525175	386	67525073	9	4.46937	3.41876
Nc-1-IP_pe	chr1	67554566	67555050	485	67554725	9	4.46937	3.41876
Nc-1-IP_pe	chr1	67561872	67562133	262	67561994	10	4.82246	3.50943
Nc-1-IP_pe	chr1	67592511	67592830	320	67592690	13	4.86362	3.18015
Nc-1-IP_pe	chr1	67610145	67610440	296	67610298	15	6.19428	3.63445
Nc-1-IP_pe	chr1	67625710	67626111	402	67625880	10	5.23357	3.76063
Nc-1-IP_pe	chr1	67634111	67634599	489	67634523	11	3.64839	2.72584
Nc-1-IP_pe	chr1	67649878	67650169	292	67650018	15	6.19428	3.63445
Nc-1-IP_pe	chr1	67685615	67685936	322	67685802	15	4.80057	2.95908
Nc-1-IP_pe	chr1	67700784	67701071	288	67700997	12	5.77527	3.80164
Nc-1-IP_pe	chr1	67736291	67736614	324	67736302	9	2.89249	2.46179
Nc-1-IP_pe	chr1	67754238	67754726	489	67754529	13	6.72991	4.20021

Nc-1-IP_pe	chr1	67832197	67832534	338	67832282	13	5.9487	3.76157
Nc-1-IP_pe	chr1	67939416	67939690	275	67939584	10	4.93609	3.57849
Nc-1-IP_pe	chr1	67959905	67960204	300	67960037	10	5.23357	3.76063
Nc-1-IP_pe	chr1	68008903	68009293	391	68008978	11	5.77463	3.94557
Nc-1-IP_pe	chr1	68043434	68044047	614	68043800	11	6.03439	4.10251
Nc-1-IP_pe	chr1	68078220	68078559	340	68078428	11	5.03835	3.5092
Nc-1-IP_pe	chr1	68132216	68132640	425	68132255	9	4.25966	3.28797
Nc-1-IP_pe	chr1	68230903	68231302	400	68231096	10	4.33501	3.21677
Nc-1-IP_pe	chr1	68232504	68232800	297	68232531	10	5.23357	3.76063
Nc-1-IP_pe	chr1	68238262	68238618	357	68238466	10	5.23357	3.76063
Nc-1-IP_pe	chr1	68373272	68373564	293	68373406	15	9.19325	5.26076
Nc-1-IP_pe	chr1	68387094	68387415	322	68387292	10	4.39571	3.25288
Nc-1-IP_pe	chr1	68405980	68406461	482	68406103	13	7.7352	4.78626
Nc-1-IP_pe	chr1	68547820	68548483	664	68547847	9	3.27326	2.68684
Nc-1-IP_pe	chr1	68710107	68710400	294	68710158	10	4.77495	3.48064
Nc-1-IP_pe	chr1	68742982	68743380	399	68743155	8	3.74505	3.07688
Nc-1-IP_pe	chr1	68863597	68863949	353	68863608	9	3.27326	2.68684
Nc-1-IP_pe	chr1	68881049	68881748	700	68881126	12	4.24064	2.95299
Nc-1-IP_pe	chr1	69071749	69072231	483	69071863	10	4.39571	3.25288
Nc-1-IP_pe	chr1	69169989	69170296	308	69170149	12	6.86906	4.44438
Nc-1-IP_pe	chr1	69373612	69374101	490	69373616	10	3.08907	2.49869
Nc-1-IP_pe	chr1	69567995	69568367	373	69568207	13	5.36853	3.44651
Nc-1-IP_pe	chr1	69663761	69664035	275	69663975	9	4.46937	3.41876
Nc-1-IP_pe	chr1	69888140	69888560	421	69888284	8	3.74505	3.07688
Nc-1-IP_pe	chr1	69919632	69920269	638	69919758	12	6.31185	4.11348
Nc-1-IP_pe	chr1	70038235	70038521	287	70038386	10	5.23357	3.76063
Nc-1-IP_pe	chr1	70167961	70168222	262	70168025	11	5.7051	3.90381
Nc-1-IP_pe	chr1	70180171	70180428	258	70180302	10	5.23357	3.76063
Nc-1-IP_pe	chr1	70205741	70205994	254	70205746	11	3.64839	2.72584
Nc-1-IP_pe	chr1	70221415	70221799	385	70221647	15	6.19428	3.63445
Nc-1-IP_pe	chr1	70281655	70281908	254	70281663	9	3.27326	2.68684
Nc-1-IP_pe	chr1	70343795	70344107	313	70344039	8	3.74505	3.07688
Nc-1-IP_pe	chr1	70365087	70365490	404	70365432	8	3.51085	2.92785
Nc-1-IP_pe	chr1	70397133	70397397	265	70397270	10	4.99888	3.61677
Nc-1-IP_pe	chr1	70487461	70487843	383	70487614	11	4.23076	3.04719
Nc-1-IP_pe	chr1	70566449	70566877	429	70566813	8	3.64414	3.01254
Nc-1-IP_pe	chr1	70600844	70601109	266	70601041	16	4.25252	2.63824
Nc-1-IP_pe	chr1	70628937	70629236	300	70629067	9	4.46937	3.41876
Nc-1-IP_pe	chr1	70675453	70676012	560	70675848	12	6.86906	4.44438
Nc-1-IP_pe	chr1	70727478	70728048	571	70727569	12	6.86906	4.44438
Nc-1-IP_pe	chr1	70752624	70753326	703	70753125	9	4.46937	3.41876
Nc-1-IP_pe	chr1	70753945	70754634	690	70754540	9	4.46937	3.41876
Nc-1-IP_pe	chr1	70803872	70804206	335	70804073	10	5.23357	3.76063
Nc-1-IP_pe	chr1	70806621	70807104	484	70807019	11	6.03439	4.10251
Nc-1-IP_pe	chr1	70883767	70884023	257	70883894	12	6.86906	4.44438

Nc-1-IP_pe	chr1	71046425	71046822	398	71046760	10	3.08907	2.49869
Nc-1-IP_pe	chr1	71187740	71188016	277	71187913	10	5.23357	3.76063
Nc-1-IP_pe	chr1	71331986	71332308	323	71332237	10	5.23357	3.76063
Nc-1-IP_pe	chr1	71513879	71514431	553	71514201	21	5.88587	2.94733
Nc-1-IP_pe	chr1	71602331	71602598	268	71602399	8	3.74505	3.07688
Nc-1-IP_pe	chr1	72147857	72148327	471	72147932	9	4.46937	3.41876
Nc-1-IP_pe	chr1	72419427	72419705	279	72419519	8	3.56021	2.95918
Nc-1-IP_pe	chr1	72450696	72451109	414	72450792	11	6.03439	4.10251
Nc-1-IP_pe	chr1	72725229	72725862	634	72725444	11	4.54404	3.2242
Nc-1-IP_pe	chr1	72898818	72899339	522	72898979	13	6.62436	4.14003
Nc-1-IP_pe	chr1	72982975	72983245	271	72983057	10	5.23357	3.76063
Nc-1-IP_pe	chr1	72984458	72984733	276	72984602	15	9.55381	5.47001
Nc-1-IP_pe	chr1	72990938	72991238	301	72991064	13	5.36853	3.44651
Nc-1-IP_pe	chr1	73045119	73045606	488	73045549	9	3.72192	2.95716
Nc-1-IP_pe	chr1	73181004	73181591	588	73181421	12	6.86906	4.44438
Nc-1-IP_pe	chr1	73205375	73205665	291	73205497	10	5.23357	3.76063
Nc-1-IP_pe	chr1	73344113	73344564	452	73344343	13	6.62436	4.14003
Nc-1-IP_pe	chr1	73448012	73448335	324	73448299	11	3.64839	2.72584
Nc-1-IP_pe	chr1	73526684	73527076	393	73526950	15	6.19428	3.63445
Nc-1-IP_pe	chr1	73570069	73570324	256	73570135	9	4.46937	3.41876
Nc-1-IP_pe	chr1	73626843	73627319	477	73627071	9	4.46937	3.41876
Nc-1-IP_pe	chr1	73666088	73666390	303	73666161	7	3.06442	2.73501
Nc-1-IP_pe	chr1	73844837	73845091	255	73844928	12	5.23082	3.49289
Nc-1-IP_pe	chr1	74247404	74247921	518	74247476	9	4.46937	3.41876
Nc-1-IP_pe	chr1	74280503	74281129	627	74280710	10	5.23357	3.76063
Nc-1-IP_pe	chr1	74673490	74673994	505	74673836	12	5.23082	3.49289
Nc-1-IP_pe	chr1	74725916	74726411	496	74726099	13	7.7352	4.78626
Nc-1-IP_pe	chr1	74733150	74733505	356	74733295	14	5.51541	3.4073
Nc-1-IP_pe	chr1	74756710	74757284	575	74756873	14	8.63073	5.12814
Nc-1-IP_pe	chr1	74885049	74885356	308	74885114	8	3.74505	3.07688
Nc-1-IP_pe	chr1	74913596	74914111	516	74913758	13	7.7352	4.78626
Nc-1-IP_pe	chr1	74952003	74952284	282	74952123	12	4.24064	2.95299
Nc-1-IP_pe	chr1	75037027	75037399	373	75037179	12	6.12485	4.004
Nc-1-IP_pe	chr1	75040098	75040702	605	75040468	12	5.84943	3.84431
Nc-1-IP_pe	chr1	75068460	75068797	338	75068504	10	3.89064	2.95552
Nc-1-IP_pe	chr1	75136869	75137175	307	75137059	13	6.54342	4.09407
Nc-1-IP_pe	chr1	75257981	75258403	423	75258090	11	5.89269	4.01672
Nc-1-IP_pe	chr1	75374090	75374691	602	75374535	10	5.23357	3.76063
Nc-1-IP_pe	chr1	75375801	75376425	625	75375952	10	5.23357	3.76063
Nc-1-IP_pe	chr1	75510924	75511211	288	75511054	14	8.63073	5.12814
Nc-1-IP_pe	chr1	75581999	75582654	656	75582290	14	8.29543	4.93196
Nc-1-IP_pe	chr1	75589790	75590112	323	75589954	13	7.42513	4.60316
Nc-1-IP_pe	chr1	75615601	75615898	298	75615715	12	4.88508	3.30112
Nc-1-IP_pe	chr1	75677866	75678282	417	75678278	11	2.96397	2.36091
Nc-1-IP_pe	chr1	75684799	75685298	500	75685111	14	8.63073	5.12814

Nc-1-IP_pe	chr1	75698086	75698362	277	75698168	10	4.20113	3.13749
Nc-1-IP_pe	chr1	75724291	75724867	577	75724431	15	6.10627	3.59023
Nc-1-IP_pe	chr1	75755246	75755532	287	75755375	13	5.03208	3.26819
Nc-1-IP_pe	chr1	75759968	75760231	264	75760141	10	5.23357	3.76063
Nc-1-IP_pe	chr1	75773483	75773767	285	75773680	8	3.74505	3.07688
Nc-1-IP_pe	chr1	75777790	75778508	719	75777935	9	3.27326	2.68684
Nc-1-IP_pe	chr1	75828965	75829445	481	75829363	10	4.99888	3.61677
Nc-1-IP_pe	chr1	75842762	75843140	379	75843021	17	7.02067	3.79543
Nc-1-IP_pe	chr1	75862067	75862414	348	75862260	12	6.58417	4.27436
Nc-1-IP_pe	chr1	75940039	75940328	290	75940200	11	6.03439	4.10251
Nc-1-IP_pe	chr1	76111983	76112881	899	76112093	14	7.34079	4.38651
Nc-1-IP_pe	chr1	76121216	76121815	600	76121359	11	6.03439	4.10251
Nc-1-IP_pe	chr1	76180740	76181103	364	76180857	10	3.89064	2.95552
Nc-1-IP_pe	chr1	76232619	76232928	310	76232793	10	5.23357	3.76063
Nc-1-IP_pe	chr1	76282826	76283142	317	76282961	14	7.42849	4.43574
Nc-1-IP_pe	chr1	76447691	76448090	400	76447968	11	5.77463	3.94557
Nc-1-IP_pe	chr1	76708246	76708634	389	76708295	10	5.23357	3.76063
Nc-1-IP_pe	chr1	76787344	76787668	325	76787525	10	5.23357	3.76063
Nc-1-IP_pe	chr1	76868213	76868780	568	76868709	14	5.51541	3.4073
Nc-1-IP_pe	chr1	76898022	76898292	271	76898113	10	5.23357	3.76063
Nc-1-IP_pe	chr1	76981071	76981451	381	76981219	12	5.84943	3.84431
Nc-1-IP_pe	chr1	77004678	77005368	691	77004798	15	6.19428	3.63445
Nc-1-IP_pe	chr1	77015757	77016131	375	77015943	11	6.03439	4.10251
Nc-1-IP_pe	chr1	77044171	77044656	486	77044423	16	7.53857	4.18505
Nc-1-IP_pe	chr1	77104757	77105232	476	77104869	11	5.10577	3.5486
Nc-1-IP_pe	chr1	77159059	77159577	519	77159169	10	5.23357	3.76063
Nc-1-IP_pe	chr1	77195132	77195408	277	77195209	12	5.23082	3.49289
Nc-1-IP_pe	chr1	77253622	77253956	335	77253773	11	6.03439	4.10251
Nc-1-IP_pe	chr1	77275224	77275484	261	77275371	13	7.7352	4.78626
Nc-1-IP_pe	chr1	77480990	77481289	300	77481043	9	2.89249	2.46179
Nc-1-IP_pe	chr1	77508379	77508895	517	77508658	14	7.42849	4.43574
Nc-1-IP_pe	chr1	77516757	77517121	365	77516946	14	4.60011	2.95114
Nc-1-IP_pe	chr1	77524988	77525533	546	77525078	9	4.46937	3.41876
Nc-1-IP_pe	chr1	77531759	77532146	388	77531959	12	5.61153	3.70794
Nc-1-IP_pe	chr1	77621564	77621816	253	77621710	9	4.46937	3.41876
Nc-1-IP_pe	chr1	77731764	77732041	278	77731986	8	3.74505	3.07688
Nc-1-IP_pe	chr1	77759584	77759902	319	77759694	13	4.86362	3.18015
Nc-1-IP_pe	chr1	77778788	77779042	255	77778854	9	4.24819	3.28084
Nc-1-IP_pe	chr1	77785497	77785809	313	77785568	8	3.56021	2.95918
Nc-1-IP_pe	chr1	77792870	77793281	412	77793139	8	3.74505	3.07688
Nc-1-IP_pe	chr1	77818711	77819003	293	77818890	12	6.86906	4.44438
Nc-1-IP_pe	chr1	77869488	77871208	1721	77870189	19	10.236	5.07864
Nc-1-IP_pe	chr1	77884842	77885118	277	77885021	10	4.80655	3.49978
Nc-1-IP_pe	chr1	77888709	77889123	415	77888875	20	9.45052	4.53626
Nc-1-IP_pe	chr1	77924033	77924300	268	77924231	10	4.62142	3.388

Nc-1-IP_pe	chr1	78014631	78014900	270	78014769	12	6.12485	4.004
Nc-1-IP_pe	chr1	78032097	78032407	311	78032260	13	7.7352	4.78626
Nc-1-IP_pe	chr1	78041845	78042344	500	78042015	10	3.89064	2.95552
Nc-1-IP_pe	chr1	78045908	78046538	631	78046159	13	4.41954	2.952
Nc-1-IP_pe	chr1	78107171	78107423	253	78107253	11	5.77463	3.94557
Nc-1-IP_pe	chr1	78168803	78169319	517	78169100	9	4.25966	3.28797
Nc-1-IP_pe	chr1	78178821	78179080	260	78178844	9	4.46937	3.41876
Nc-1-IP_pe	chr1	78202794	78203636	843	78202940	12	6.31185	4.11348
Nc-1-IP_pe	chr1	78211698	78212191	494	78211887	12	6.12485	4.004
Nc-1-IP_pe	chr1	78213925	78214657	733	78214153	13	5.9487	3.76157
Nc-1-IP_pe	chr1	78241642	78241965	324	78241820	12	6.58417	4.27436
Nc-1-IP_pe	chr1	78268651	78268955	305	78268901	9	4.46937	3.41876
Nc-1-IP_pe	chr1	78465045	78465443	399	78465374	12	6.12485	4.004
Nc-1-IP_pe	chr1	78484196	78484468	273	78484349	14	6.69566	4.03025
Nc-1-IP_pe	chr1	78553075	78553890	816	78553861	10	3.89064	2.95552
Nc-1-IP_pe	chr1	78647228	78647543	316	78647342	11	4.54404	3.2242
Nc-1-IP_pe	chr1	78701530	78702416	887	78701604	14	8.29543	4.93196
Nc-1-IP_pe	chr1	78840680	78840965	286	78840789	9	3.27326	2.68684
Nc-1-IP_pe	chr1	79037010	79037521	512	79037133	15	5.20142	3.14789
Nc-1-IP_pe	chr1	79045262	79045757	496	79045474	12	4.70082	3.20033
Nc-1-IP_pe	chr1	79165638	79165929	292	79165837	12	6.86906	4.44438
Nc-1-IP_pe	chr1	79271625	79272247	623	79272070	13	7.7352	4.78626
Nc-1-IP_pe	chr1	79461853	79462120	268	79461954	12	6.86906	4.44438
Nc-1-IP_pe	chr1	79464281	79464828	548	79464379	12	6.86906	4.44438
Nc-1-IP_pe	chr1	79478235	79478580	346	79478426	9	4.46937	3.41876
Nc-1-IP_pe	chr1	79516480	79517112	633	79516696	11	5.3563	3.696
Nc-1-IP_pe	chr1	79586074	79586505	432	79586150	9	3.72192	2.95716
Nc-1-IP_pe	chr1	79593965	79594417	453	79594251	9	4.46937	3.41876
Nc-1-IP_pe	chr1	79644099	79644425	327	79644243	15	8.26005	4.73146
Nc-1-IP_pe	chr1	79680200	79680813	614	79680716	10	5.23357	3.76063
Nc-1-IP_pe	chr1	79700356	79700612	257	79700462	11	6.03439	4.10251
Nc-1-IP_pe	chr1	79700872	79701192	321	79701003	9	3.72192	2.95716
Nc-1-IP_pe	chr1	79858657	79858920	264	79858694	10	4.93609	3.57849
Nc-1-IP_pe	chr1	79875300	79875622	323	79875449	15	7.37761	4.24941
Nc-1-IP_pe	chr1	79924075	79924538	464	79924382	12	4.24064	2.95299
Nc-1-IP_pe	chr1	80127270	80127556	287	80127393	16	10.5028	5.81189
Nc-1-IP_pe	chr1	80399733	80400108	376	80399955	13	6.62436	4.14003
Nc-1-IP_pe	chr1	80583950	80584217	268	80584115	9	4.25966	3.28797
Nc-1-IP_pe	chr1	80606848	80607230	383	80606914	9	3.66792	2.92434
Nc-1-IP_pe	chr1	80654029	80654388	360	80654210	9	4.46937	3.41876
Nc-1-IP_pe	chr1	80686033	80686586	554	80686407	13	5.9487	3.76157
Nc-1-IP_pe	chr1	80722798	80723348	551	80722917	13	6.92473	4.312
Nc-1-IP_pe	chr1	80944770	80945211	442	80944992	12	4.70082	3.20033
Nc-1-IP_pe	chr1	81027801	81028094	294	81027983	11	6.03439	4.10251
Nc-1-IP_pe	chr1	81175638	81175909	272	81175791	9	3.92293	3.08

Nc-1-IP_pe	chr1	81178577	81179006	430	81178718	12	4.75569	3.23024
Nc-1-IP_pe	chr1	81220594	81220888	295	81220703	11	4.47871	3.18705
Nc-1-IP_pe	chr1	81221538	81221904	367	81221628	14	7.42849	4.43574
Nc-1-IP_pe	chr1	81336943	81337337	395	81337082	13	4.02562	2.7544
Nc-1-IP_pe	chr1	81502847	81503594	748	81502996	13	5.03208	3.26819
Nc-1-IP_pe	chr1	81504658	81505353	696	81504800	10	5.23357	3.76063
Nc-1-IP_pe	chr1	81602284	81602895	612	81602856	10	4.20113	3.13749
Nc-1-IP_pe	chr1	81616697	81617240	544	81616985	12	6.58417	4.27436
Nc-1-IP_pe	chr1	81654981	81655260	280	81655032	11	6.03439	4.10251
Nc-1-IP_pe	chr1	81671571	81671862	292	81671768	9	4.46937	3.41876
Nc-1-IP_pe	chr1	81721489	81721759	271	81721615	11	6.03439	4.10251
Nc-1-IP_pe	chr1	81736984	81737543	560	81737084	11	5.77463	3.94557
Nc-1-IP_pe	chr1	81800931	81801325	395	81801195	12	5.15877	3.45264
Nc-1-IP_pe	chr1	81801674	81801998	325	81801809	15	6.69889	3.8921
Nc-1-IP_pe	chr1	82172703	82172965	263	82172774	10	5.23357	3.76063
Nc-1-IP_pe	chr1	82239896	82240320	425	82240147	12	6.31185	4.11348
Nc-1-IP_pe	chr1	82330332	82331062	731	82331007	10	4.99888	3.61677
Nc-1-IP_pe	chr1	82589362	82589647	286	82589460	12	6.86906	4.44438
Nc-1-IP_pe	chr1	82776545	82777836	1292	82776909	14	6.69566	4.03025
Nc-1-IP_pe	chr1	82956018	82956342	325	82956181	14	7.42849	4.43574
Nc-1-IP_pe	chr1	83050742	83051297	556	83051073	11	5.77463	3.94557
Nc-1-IP_pe	chr1	83068279	83068589	311	83068488	10	4.39571	3.25288
Nc-1-IP_pe	chr1	83143098	83143560	463	83143539	10	4.07292	3.06202
Nc-1-IP_pe	chr1	83436778	83437070	293	83436912	22	14.7336	6.80148
Nc-1-IP_pe	chr1	83441927	83442373	447	83442073	10	5.23357	3.76063
Nc-1-IP_pe	chr1	83554318	83554599	282	83554462	12	6.86906	4.44438
Nc-1-IP_pe	chr1	83583263	83583522	260	83583450	12	5.23082	3.49289
Nc-1-IP_pe	chr1	83593460	83593743	284	83593668	10	5.10552	3.682
Nc-1-IP_pe	chr1	83668304	83668695	392	83668550	9	4.46937	3.41876
Nc-1-IP_pe	chr1	83950821	83951085	265	83950966	11	5.3563	3.696
Nc-1-IP_pe	chr1	83998416	83999256	841	83998931	18	8.37884	4.31591
Nc-1-IP_pe	chr1	84038249	84038547	299	84038503	9	4.05981	3.16422
Nc-1-IP_pe	chr1	84077517	84078124	608	84077831	18	6.15736	3.29676
Nc-1-IP_pe	chr1	84224235	84224505	271	84224382	14	7.34079	4.38651
Nc-1-IP_pe	chr1	84234556	84234834	279	84234710	18	11.3299	5.852
Nc-1-IP_pe	chr1	84298793	84299342	550	84298889	15	3.46204	2.35851
Nc-1-IP_pe	chr1	84443268	84443882	615	84443318	12	4.24064	2.95299
Nc-1-IP_pe	chr1	84450337	84450644	308	84450450	10	4.99888	3.61677
Nc-1-IP_pe	chr1	84479289	84479775	487	84479494	18	6.15736	3.29676
Nc-1-IP_pe	chr1	84505612	84505977	366	84505800	14	5.03085	3.16286
Nc-1-IP_pe	chr1	84509691	84510155	465	84510062	10	5.23357	3.76063
Nc-1-IP_pe	chr1	84527779	84528038	260	84527891	10	5.23357	3.76063
Nc-1-IP_pe	chr1	84601250	84601521	272	84601368	13	6.56172	4.10444
Nc-1-IP_pe	chr1	84620248	84621087	840	84620588	15	5.66913	3.37372
Nc-1-IP_pe	chr1	84794525	84794801	277	84794698	11	6.03439	4.10251

Nc-1-IP_pe	chr1	84888846	84889239	394	84888993	12	4.70082	3.20033
Nc-1-IP_pe	chr1	84922600	84923248	649	84922713	11	6.03439	4.10251
Nc-1-IP_pe	chr1	84947442	84948091	650	84947745	13	5.57038	3.55505
Nc-1-IP_pe	chr1	84968746	84969054	309	84968925	13	6.72991	4.20021
Nc-1-IP_pe	chr1	85047852	85048558	707	85048132	20	9.21699	4.428
Nc-1-IP_pe	chr1	85136023	85136311	289	85136099	11	4.88957	3.42271
Nc-1-IP_pe	chr1	85200336	85201059	724	85200877	22	12.1294	5.47971
Nc-1-IP_pe	chr1	85211641	85212059	419	85211727	9	3.72192	2.95716
Nc-1-IP_pe	chr1	85241754	85242108	355	85241953	14	4.60011	2.95114
Nc-1-IP_pe	chr1	85259227	85259883	657	85259398	21	9.29881	4.32834
Nc-1-IP_pe	chr1	85332730	85333168	439	85333051	13	7.02546	4.37016
Nc-1-IP_pe	chr1	85555934	85556197	264	85556075	14	8.29543	4.93196
Nc-1-IP_pe	chr1	85576437	85577317	881	85577245	15	5.20142	3.14789
Nc-1-IP_pe	chr1	85580734	85581552	819	85581014	18	6.63038	3.50359
Nc-1-IP_pe	chr1	85596052	85596477	426	85596385	9	4.46937	3.41876
Nc-1-IP_pe	chr1	85602391	85602666	276	85602488	13	6.62436	4.14003
Nc-1-IP_pe	chr1	85615104	85615450	347	85615188	9	3.27326	2.68684
Nc-1-IP_pe	chr1	85629150	85629460	311	85629363	10	3.4604	2.70797
Nc-1-IP_pe	chr1	85641832	85642086	255	85641934	14	7.42849	4.43574
Nc-1-IP_pe	chr1	85676031	85676284	254	85676095	11	6.03439	4.10251
Nc-1-IP_pe	chr1	85683004	85683369	366	85683124	11	4.06403	2.95415
Nc-1-IP_pe	chr1	85708127	85708472	346	85708343	14	4.60011	2.95114
Nc-1-IP_pe	chr1	85830454	85830917	464	85830649	17	7.36791	3.96218
Nc-1-IP_pe	chr1	85896535	85896832	298	85896613	15	5.66913	3.37372
Nc-1-IP_pe	chr1	85955299	85955564	266	85955358	10	4.93609	3.57849
Nc-1-IP_pe	chr1	86105598	86105931	334	86105745	12	6.86906	4.44438
Nc-1-IP_pe	chr1	86110613	86110889	277	86110719	12	6.31185	4.11348
Nc-1-IP_pe	chr1	86167707	86168685	979	86168406	11	6.03439	4.10251
Nc-1-IP_pe	chr1	86216069	86216803	735	86216194	14	5.43414	3.36584
Nc-1-IP_pe	chr1	86377351	86377726	376	86377659	9	4.25966	3.28797
Nc-1-IP_pe	chr1	86412155	86412426	272	86412289	12	6.27375	4.09111
Nc-1-IP_pe	chr1	86523963	86524323	361	86524093	9	3.72192	2.95716
Nc-1-IP_pe	chr1	86551472	86551737	266	86551608	8	3.74505	3.07688
Nc-1-IP_pe	chr1	86552402	86552668	267	86552608	11	4.54404	3.2242
Nc-1-IP_pe	chr1	86594453	86594847	395	86594825	9	4.20361	3.25317
Nc-1-IP_pe	chr1	86623241	86623515	275	86623357	13	7.7352	4.78626
Nc-1-IP_pe	chr1	86664634	86665370	737	86665322	9	3.27326	2.68684
Nc-1-IP_pe	chr1	86666478	86666741	264	86666622	11	5.10577	3.5486
Nc-1-IP_pe	chr1	86704173	86704688	516	86704291	13	4.86362	3.18015
Nc-1-IP_pe	chr1	86704947	86705416	470	86705144	16	9.01614	4.97137
Nc-1-IP_pe	chr1	86798204	86798464	261	86798357	10	5.23357	3.76063
Nc-1-IP_pe	chr1	86813873	86814183	311	86813932	11	6.03439	4.10251
Nc-1-IP_pe	chr1	86837798	86838159	362	86837896	10	4.99888	3.61677
Nc-1-IP_pe	chr1	86842113	86842458	346	86842260	19	10.8111	5.37367
Nc-1-IP_pe	chr1	86899386	86900361	976	86900174	13	5.15976	3.33548

Nc-1-IP_pe	chr1	86914639	86914920	282	86914786	14	5.51541	3.4073
Nc-1-IP_pe	chr1	86924250	86924701	452	86924532	11	5.7051	3.90381
Nc-1-IP_pe	chr1	87118350	87118785	436	87118703	9	3.27326	2.68684
Nc-1-IP_pe	chr1	87125835	87126109	275	87126035	11	4.54404	3.2242
Nc-1-IP_pe	chr1	87132072	87133139	1068	87132579	18	5.72638	3.11298
Nc-1-IP_pe	chr1	87296801	87297165	365	87296962	14	8.63073	5.12814
Nc-1-IP_pe	chr1	87300179	87300443	265	87300233	12	6.50787	4.22912
Nc-1-IP_pe	chr1	87320163	87320430	268	87320325	10	5.23357	3.76063
Nc-1-IP_pe	chr1	87328665	87328917	253	87328817	13	5.86991	3.71823
Nc-1-IP_pe	chr1	87335271	87335699	429	87335481	15	8.26005	4.73146
Nc-1-IP_pe	chr1	87623392	87623668	277	87623507	12	6.86906	4.44438
Nc-1-IP_pe	chr1	87636246	87636704	459	87636468	14	6.69566	4.03025
Nc-1-IP_pe	chr1	87653778	87654143	366	87654103	8	3.38431	2.8478
Nc-1-IP_pe	chr1	88123913	88124289	377	88124085	11	5.10577	3.5486
Nc-1-IP_pe	chr1	88185616	88185936	321	88185774	13	5.9487	3.76157
Nc-1-IP_pe	chr1	88422715	88423043	329	88422921	12	4.75569	3.23024
Nc-1-IP_pe	chr1	88423304	88423561	258	88423444	10	3.89064	2.95552
Nc-1-IP_pe	chr1	88635269	88635558	290	88635442	17	8.7831	4.67333
Nc-1-IP_pe	chr1	88744463	88744715	253	88744628	11	6.03439	4.10251
Nc-1-IP_pe	chr1	88794145	88794433	289	88794311	12	6.86906	4.44438
Nc-1-IP_pe	chr1	88822725	88823106	382	88822805	10	3.89064	2.95552
Nc-1-IP_pe	chr1	88846306	88846623	318	88846449	11	6.03439	4.10251
Nc-1-IP_pe	chr1	88851512	88851788	277	88851622	13	5.42865	3.47872
Nc-1-IP_pe	chr1	88862371	88862866	496	88862470	11	6.03439	4.10251
Nc-1-IP_pe	chr1	88878050	88878309	260	88878196	12	5.77527	3.80164
Nc-1-IP_pe	chr1	88902679	88903190	512	88902738	9	4.46937	3.41876
Nc-1-IP_pe	chr1	88924909	88925421	513	88925274	11	5.95085	4.05188
Nc-1-IP_pe	chr1	88937032	88937316	285	88937197	13	5.9487	3.76157
Nc-1-IP_pe	chr1	88974507	88974957	451	88974570	7	2.9043	2.63038
Nc-1-IP_pe	chr1	89043705	89044158	454	89043921	13	5.86991	3.71823
Nc-1-IP_pe	chr1	89091868	89092172	305	89091889	8	3.74505	3.07688
Nc-1-IP_pe	chr1	89127982	89128461	480	89128207	15	5.20142	3.14789
Nc-1-IP_pe	chr1	89259636	89259984	349	89259824	12	6.86906	4.44438
Nc-1-IP_pe	chr1	89273513	89273995	483	89273650	12	4.70082	3.20033
Nc-1-IP_pe	chr1	89284270	89284543	274	89284339	8	3.74505	3.07688
Nc-1-IP_pe	chr1	89302934	89303340	407	89302955	11	2.96397	2.36091
Nc-1-IP_pe	chr1	89404836	89405763	928	89404902	13	6.62436	4.14003
Nc-1-IP_pe	chr1	89429782	89430232	451	89430176	9	4.46937	3.41876
Nc-1-IP_pe	chr1	89446238	89446739	502	89446313	11	6.03439	4.10251
Nc-1-IP_pe	chr1	89490997	89491537	541	89491381	12	4.24064	2.95299
Nc-1-IP_pe	chr1	89519233	89519638	406	89519437	9	4.46937	3.41876
Nc-1-IP_pe	chr1	89524253	89525211	959	89525058	25	11.6607	4.79439
Nc-1-IP_pe	chr1	89610008	89610480	473	89610038	13	3.67377	2.58159
Nc-1-IP_pe	chr1	89633446	89633762	317	89633683	14	5.56442	3.43239
Nc-1-IP_pe	chr1	89709436	89710016	581	89709954	11	5.3563	3.696

Nc-1-IP_pe	chr1	89739033	89739333	301	89739075	11	3.89191	2.85898
Nc-1-IP_pe	chr1	89752444	89752910	467	89752534	14	4.21449	2.76599
Nc-1-IP_pe	chr1	89774266	89774669	404	89774363	12	3.47963	2.55766
Nc-1-IP_pe	chr1	89781743	89782016	274	89781839	12	5.23082	3.49289
Nc-1-IP_pe	chr1	89786790	89787131	342	89787032	10	5.23357	3.76063
Nc-1-IP_pe	chr1	89843646	89844376	731	89843903	18	7.1522	3.73811
Nc-1-IP_pe	chr1	89889599	89889861	263	89889765	15	8.61047	4.928
Nc-1-IP_pe	chr1	89963645	89964115	471	89963809	9	4.05981	3.16422
Nc-1-IP_pe	chr1	89977627	89977896	270	89977802	8	3.08755	2.66145
Nc-1-IP_pe	chr1	89979184	89979442	259	89979329	12	6.86906	4.44438
Nc-1-IP_pe	chr1	89984636	89984983	348	89984767	13	3.35769	2.42919
Nc-1-IP_pe	chr1	89995055	89995573	519	89995374	19	7.29128	3.68799
Nc-1-IP_pe	chr1	90062546	90063093	548	90062793	15	8.84797	5.06275
Nc-1-IP_pe	chr1	90167157	90167543	387	90167222	9	4.25966	3.28797
Nc-1-IP_pe	chr1	90188548	90188901	354	90188592	10	4.99888	3.61677
Nc-1-IP_pe	chr1	90204164	90204418	255	90204321	10	5.23357	3.76063
Nc-1-IP_pe	chr1	90219424	90219706	283	90219560	18	7.1522	3.73811
Nc-1-IP_pe	chr1	90225606	90225980	375	90225725	10	5.23357	3.76063
Nc-1-IP_pe	chr1	90328446	90328710	265	90328505	10	3.4604	2.70797
Nc-1-IP_pe	chr1	90329157	90329508	352	90329271	11	6.03439	4.10251
Nc-1-IP_pe	chr1	90349356	90349825	470	90349779	8	3.74505	3.07688
Nc-1-IP_pe	chr1	90513360	90513738	379	90513457	8	3.74505	3.07688
Nc-1-IP_pe	chr1	90706394	90706778	385	90706526	11	6.03439	4.10251
Nc-1-IP_pe	chr1	90707516	90707871	356	90707705	12	6.58417	4.27436
Nc-1-IP_pe	chr1	90718286	90718941	656	90718919	8	3.74505	3.07688
Nc-1-IP_pe	chr1	90723580	90724136	557	90724082	11	6.03439	4.10251
Nc-1-IP_pe	chr1	90726883	90727156	274	90726935	9	4.20361	3.25317
Nc-1-IP_pe	chr1	90745476	90745746	271	90745577	12	6.86906	4.44438
Nc-1-IP_pe	chr1	90761243	90761547	305	90761442	9	4.25966	3.28797
Nc-1-IP_pe	chr1	90841444	90841773	330	90841497	9	4.46937	3.41876
Nc-1-IP_pe	chr1	90851268	90851568	301	90851491	15	8.16556	4.67894
Nc-1-IP_pe	chr1	90891877	90892428	552	90892153	11	6.03439	4.10251
Nc-1-IP_pe	chr1	90917460	90917801	342	90917658	13	7.7352	4.78626
Nc-1-IP_pe	chr1	90997632	90997915	284	90997812	10	4.99888	3.61677
Nc-1-IP_pe	chr1	91016782	91017119	338	91016941	14	8.29543	4.93196
Nc-1-IP_pe	chr1	91021222	91021504	283	91021284	12	5.77527	3.80164
Nc-1-IP_pe	chr1	91035287	91035581	295	91035361	11	5.10577	3.5486
Nc-1-IP_pe	chr1	91041887	91042242	356	91042073	11	6.03439	4.10251
Nc-1-IP_pe	chr1	91075357	91075613	257	91075422	8	3.51085	2.92785
Nc-1-IP_pe	chr1	91102793	91103121	329	91102919	9	4.46937	3.41876
Nc-1-IP_pe	chr1	91162644	91162898	255	91162644	9	2.89249	2.46179
Nc-1-IP_pe	chr1	91167972	91168247	276	91168093	12	6.86906	4.44438
Nc-1-IP_pe	chr1	91188384	91188706	323	91188561	9	4.35492	3.34727
Nc-1-IP_pe	chr1	91199741	91200000	260	91199954	8	3.56021	2.95918
Nc-1-IP_pe	chr1	91227251	91227660	410	91227529	8	3.74505	3.07688

Nc-1-IP_pe	chr1	91286791	91287214	424	91287172	11	4.06403	2.95415
Nc-1-IP_pe	chr1	91288902	91289229	328	91289010	11	6.03439	4.10251
Nc-1-IP_pe	chr1	91404390	91404791	402	91404410	10	3.89064	2.95552
Nc-1-IP_pe	chr1	91468120	91468393	274	91468223	10	3.89064	2.95552
Nc-1-IP_pe	chr1	91473416	91473790	375	91473659	10	3.89064	2.95552
Nc-1-IP_pe	chr1	91535528	91535877	350	91535696	13	7.7352	4.78626
Nc-1-IP_pe	chr1	91546272	91546737	466	91546517	15	9.55381	5.47001
Nc-1-IP_pe	chr1	91560780	91561100	321	91560983	10	5.23357	3.76063
Nc-1-IP_pe	chr1	91572036	91572335	300	91572166	12	5.84943	3.84431
Nc-1-IP_pe	chr1	91594853	91595140	288	91594898	13	5.36853	3.44651
Nc-1-IP_pe	chr1	91627522	91628390	869	91628319	15	8.84797	5.06275
Nc-1-IP_pe	chr1	91635862	91636284	423	91636266	10	5.23357	3.76063
Nc-1-IP_pe	chr1	91649042	91649575	534	91649144	9	4.46937	3.41876
Nc-1-IP_pe	chr1	91650799	91651256	458	91651044	10	3.89064	2.95552
Nc-1-IP_pe	chr1	91657168	91657447	280	91657328	11	4.61014	3.26192
Nc-1-IP_pe	chr1	91668838	91669317	480	91669064	41	9.27382	2.87489
Nc-1-IP_pe	chr1	91669938	91670257	320	91670015	11	3.64839	2.72584
Nc-1-IP_pe	chr1	91719951	91720351	401	91720131	12	5.84943	3.84431
Nc-1-IP_pe	chr1	91773393	91773710	318	91773531	10	5.23357	3.76063
Nc-1-IP_pe	chr1	91812567	91812823	257	91812701	12	6.77747	4.38954
Nc-1-IP_pe	chr1	91828663	91829341	679	91828744	12	5.84943	3.84431
Nc-1-IP_pe	chr1	91832892	91833243	352	91833125	10	5.14046	3.70342
Nc-1-IP_pe	chr1	91884834	91885131	298	91884983	14	7.016	4.20583
Nc-1-IP_pe	chr1	91907471	91908041	571	91907953	10	5.23357	3.76063
Nc-1-IP_pe	chr1	91920389	91920693	305	91920425	8	3.08755	2.66145
Nc-1-IP_pe	chr1	91922573	91923036	464	91923007	8	3.68553	3.03891
Nc-1-IP_pe	chr1	91931166	91931471	306	91931262	10	5.23357	3.76063
Nc-1-IP_pe	chr1	91932873	91933286	414	91933108	8	3.74505	3.07688
Nc-1-IP_pe	chr1	92026186	92027125	940	92026331	11	5.77463	3.94557
Nc-1-IP_pe	chr1	92111016	92111315	300	92111221	11	5.77463	3.94557
Nc-1-IP_pe	chr1	92214037	92214434	398	92214064	9	3.27326	2.68684
Nc-1-IP_pe	chr1	92229176	92229502	327	92229398	11	4.54404	3.2242
Nc-1-IP_pe	chr1	92298693	92299274	582	92298824	17	7.6273	4.08876
Nc-1-IP_pe	chr1	92328853	92329436	584	92329062	11	5.10577	3.5486
Nc-1-IP_pe	chr1	92347970	92348229	260	92348025	9	3.72192	2.95716
Nc-1-IP_pe	chr1	92422591	92423009	419	92422748	12	6.86906	4.44438
Nc-1-IP_pe	chr1	92454453	92454721	269	92454576	14	7.75387	4.62
Nc-1-IP_pe	chr1	92480184	92480447	264	92480285	13	5.45659	3.49372
Nc-1-IP_pe	chr1	92483316	92483851	536	92483760	14	4.32616	2.81919
Nc-1-IP_pe	chr1	92485347	92485806	460	92485721	17	5.9911	3.31919
Nc-1-IP_pe	chr1	92486791	92487044	254	92486946	13	5.36853	3.44651
Nc-1-IP_pe	chr1	92530199	92530612	414	92530570	12	3.83688	2.74114
Nc-1-IP_pe	chr1	92589229	92589985	757	92589301	15	5.20142	3.14789
Nc-1-IP_pe	chr1	92616860	92617233	374	92617119	12	3.47963	2.55766
Nc-1-IP_pe	chr1	92634976	92635624	649	92635462	15	5.20142	3.14789

Nc-1-IP_pe	chr1	92715552	92715881	330	92715725	10	4.62142	3.388
Nc-1-IP_pe	chr1	92803916	92804185	270	92804066	12	6.6717	4.32642
Nc-1-IP_pe	chr1	92831903	92832784	882	92832089	13	5.36853	3.44651
Nc-1-IP_pe	chr1	92849327	92849615	289	92849415	11	6.03439	4.10251
Nc-1-IP_pe	chr1	92878499	92878776	278	92878617	13	5.36853	3.44651
Nc-1-IP_pe	chr1	92961782	92962116	335	92961864	16	5.37454	3.13479
Nc-1-IP_pe	chr1	93007898	93008300	403	93007980	10	4.39571	3.25288
Nc-1-IP_pe	chr1	93027984	93028320	337	93028153	11	4.06403	2.95415
Nc-1-IP_pe	chr1	93035124	93035421	298	93035354	13	5.9487	3.76157
Nc-1-IP_pe	chr1	93078915	93079764	850	93079055	14	7.42849	4.43574
Nc-1-IP_pe	chr1	93089760	93090349	590	93090211	11	4.54404	3.2242
Nc-1-IP_pe	chr1	93145203	93145456	254	93145231	11	4.54404	3.2242
Nc-1-IP_pe	chr1	93160459	93160801	343	93160570	10	5.23357	3.76063
Nc-1-IP_pe	chr1	93170239	93170600	362	93170529	14	3.55308	2.45762
Nc-1-IP_pe	chr1	93170941	93171258	318	93171038	12	5.01934	3.37518
Nc-1-IP_pe	chr1	93180664	93180931	268	93180686	14	3.26761	2.32785
Nc-1-IP_pe	chr1	93199093	93199401	309	93199165	12	5.84943	3.84431
Nc-1-IP_pe	chr1	93252949	93253639	691	93253395	14	8.29543	4.93196
Nc-1-IP_pe	chr1	93262748	93263051	304	93262966	13	5.9487	3.76157
Nc-1-IP_pe	chr1	93345883	93346177	295	93346092	16	3.65575	2.38624
Nc-1-IP_pe	chr1	93352096	93352405	310	93352288	14	8.20556	4.87976
Nc-1-IP_pe	chr1	93375414	93376082	669	93375574	10	4.99888	3.61677
Nc-1-IP_pe	chr1	93407125	93407743	619	93407303	16	4.59224	2.7853
Nc-1-IP_pe	chr1	93408439	93408843	405	93408740	11	5.77463	3.94557
Nc-1-IP_pe	chr1	93419702	93420236	535	93420148	11	5.10577	3.5486
Nc-1-IP_pe	chr1	93423471	93423731	261	93423662	10	4.99888	3.61677
Nc-1-IP_pe	chr1	93426270	93426585	316	93426414	13	6.36468	3.99316
Nc-1-IP_pe	chr1	93434436	93434709	274	93434479	13	4.41954	2.952
Nc-1-IP_pe	chr1	93447887	93449266	1380	93448108	18	7.1522	3.73811
Nc-1-IP_pe	chr1	93455070	93455466	397	93455330	11	6.03439	4.10251
Nc-1-IP_pe	chr1	93496674	93497105	432	93496882	10	4.39571	3.25288
Nc-1-IP_pe	chr1	93517139	93517510	372	93517418	11	5.77463	3.94557
Nc-1-IP_pe	chr1	93532162	93532455	294	93532285	13	7.42513	4.60316
Nc-1-IP_pe	chr1	93566387	93566842	456	93566538	9	3.77975	2.99239
Nc-1-IP_pe	chr1	93569432	93569742	311	93569520	11	4.06403	2.95415
Nc-1-IP_pe	chr1	93575673	93576092	420	93575747	13	5.15976	3.33548
Nc-1-IP_pe	chr1	93576466	93576972	507	93576488	12	4.24064	2.95299
Nc-1-IP_pe	chr1	93578103	93578416	314	93578317	11	3.64839	2.72584
Nc-1-IP_pe	chr1	93579021	93579901	881	93579632	16	6.33285	3.58457
Nc-1-IP_pe	chr1	93588804	93589307	504	93589157	12	5.13046	3.43687
Nc-1-IP_pe	chr1	93607460	93607825	366	93607504	10	4.74365	3.46171
Nc-1-IP_pe	chr1	93608523	93608985	463	93608604	11	5.10577	3.5486
Nc-1-IP_pe	chr1	93609980	93610242	263	93610106	13	6.47103	4.0531
Nc-1-IP_pe	chr1	93619585	93619889	305	93619673	16	8.19044	4.52538
Nc-1-IP_pe	chr1	93624777	93625161	385	93624939	13	6.48901	4.06327

Nc-1-IP_pe	chr1	93637988	93638281	294	93638158	11	4.54404	3.2242
Nc-1-IP_pe	chr1	93639638	93640286	649	93639803	15	8.84797	5.06275
Nc-1-IP_pe	chr1	93650145	93650406	262	93650288	12	4.70082	3.20033
Nc-1-IP_pe	chr1	93653637	93654124	488	93653957	12	5.45448	3.61875
Nc-1-IP_pe	chr1	93680411	93680803	393	93680575	15	6.39297	3.73508
Nc-1-IP_pe	chr1	93697647	93698150	504	93697765	15	6.76257	3.92511
Nc-1-IP_pe	chr1	93698520	93698946	427	93698680	12	4.84576	3.27953
Nc-1-IP_pe	chr1	93740211	93740945	735	93740348	14	5.51541	3.4073
Nc-1-IP_pe	chr1	93751800	93752133	334	93752022	14	7.34079	4.38651
Nc-1-IP_pe	chr1	93756772	93757342	571	93757274	12	4.70082	3.20033
Nc-1-IP_pe	chr1	93757643	93758118	476	93757707	12	5.37834	3.57574
Nc-1-IP_pe	chr1	93761149	93761526	378	93761251	13	5.03208	3.26819
Nc-1-IP_pe	chr1	93766413	93766737	325	93766557	13	5.86991	3.71823
Nc-1-IP_pe	chr1	93770033	93770366	334	93770310	9	4.1889	3.24405
Nc-1-IP_pe	chr1	93785942	93786249	308	93786088	10	4.83846	3.51913
Nc-1-IP_pe	chr1	93832320	93832593	274	93832474	13	5.71736	3.63481
Nc-1-IP_pe	chr1	93847280	93848216	937	93847509	14	7.54286	4.50023
Nc-1-IP_pe	chr1	93878906	93879293	388	93879177	20	9.94634	4.77022
Nc-1-IP_pe	chr1	93884537	93884987	451	93884710	15	6.19428	3.63445
Nc-1-IP_pe	chr1	93949850	93950256	407	93950239	10	4.07292	3.06202
Nc-1-IP_pe	chr1	93975348	93975603	256	93975562	9	4.088	3.18162
Nc-1-IP_pe	chr1	93985419	93985761	343	93985637	14	7.42849	4.43574
Nc-1-IP_pe	chr1	94008977	94009585	609	94009218	13	7.58914	4.69977
Nc-1-IP_pe	chr1	94015615	94015923	309	94015793	11	4.06403	2.95415
Nc-1-IP_pe	chr1	94021862	94022438	577	94022233	15	5.20142	3.14789
Nc-1-IP_pe	chr1	94044644	94045146	503	94045093	11	3.64839	2.72584
Nc-1-IP_pe	chr1	94045799	94046156	358	94045872	11	5.02318	3.50036
Nc-1-IP_pe	chr1	94047764	94048075	312	94047844	11	5.08426	3.53601
Nc-1-IP_pe	chr1	94056555	94056874	320	94056747	16	7.61454	4.22416
Nc-1-IP_pe	chr1	94063176	94063591	416	94063424	11	5.5792	3.82847
Nc-1-IP_pe	chr1	94064446	94064876	431	94064712	16	7.53857	4.18505
Nc-1-IP_pe	chr1	94094866	94095578	713	94094961	10	4.65155	3.40613
Nc-1-IP_pe	chr1	94098696	94098986	291	94098880	10	4.37633	3.24134
Nc-1-IP_pe	chr1	94099865	94100179	315	94100077	16	6.8987	3.86161
Nc-1-IP_pe	chr1	94104477	94104810	334	94104593	17	5.54967	3.12324
Nc-1-IP_pe	chr1	94165258	94165559	302	94165377	13	6.62436	4.14003
Nc-1-IP_pe	chr1	94208503	94208765	263	94208657	11	6.03439	4.10251
Nc-1-IP_pe	chr1	94247136	94247548	413	94247370	19	7.29128	3.68799
Nc-1-IP_pe	chr1	94308793	94309122	330	94308951	11	4.54404	3.2242
Nc-1-IP_pe	chr1	94344028	94344461	434	94344342	14	8.63073	5.12814
Nc-1-IP_pe	chr1	94524535	94524885	351	94524768	10	5.23357	3.76063
Nc-1-IP_pe	chr1	94531448	94531901	454	94531743	10	4.39023	3.24962
Nc-1-IP_pe	chr1	94592220	94592474	255	94592412	11	3.64839	2.72584
Nc-1-IP_pe	chr1	94594033	94594373	341	94594245	12	5.61153	3.70794
Nc-1-IP_pe	chr1	94599416	94599699	284	94599488	10	4.91962	3.56846

Nc-1-IP_pe	chr1	94605247	94605733	487	94605377	12	5.77527	3.80164
Nc-1-IP_pe	chr1	94653025	94653910	886	94653596	12	3.83688	2.74114
Nc-1-IP_pe	chr1	94683217	94683672	456	94683539	15	5.66913	3.37372
Nc-1-IP_pe	chr1	94684351	94685183	833	94684436	13	7.12853	4.42991
Nc-1-IP_pe	chr1	94696468	94696730	263	94696569	13	7.42513	4.60316
Nc-1-IP_pe	chr1	94701132	94701481	350	94701278	13	7.43036	4.60624
Nc-1-IP_pe	chr1	94708546	94708904	359	94708720	14	6.96097	4.17548
Nc-1-IP_pe	chr1	94734803	94735550	748	94735345	13	5.9487	3.76157
Nc-1-IP_pe	chr1	94840506	94840949	444	94840775	11	5.10577	3.5486
Nc-1-IP_pe	chr1	94843064	94843471	408	94843168	11	5.3563	3.696
Nc-1-IP_pe	chr1	94848266	94848947	682	94848329	13	5.36853	3.44651
Nc-1-IP_pe	chr1	94877868	94878434	567	94878295	10	5.23357	3.76063
Nc-1-IP_pe	chr1	94886055	94886789	735	94886577	11	5.10577	3.5486
Nc-1-IP_pe	chr1	94915687	94916375	689	94915910	13	6.82624	4.25537
Nc-1-IP_pe	chr1	94927305	94927598	294	94927413	13	4.45253	2.96876
Nc-1-IP_pe	chr1	94935916	94936325	410	94936310	8	2.93592	2.56703
Nc-1-IP_pe	chr1	94976374	94976695	322	94976503	12	5.84943	3.84431
Nc-1-IP_pe	chr1	95004329	95004660	332	95004511	14	5.98179	3.64885
Nc-1-IP_pe	chr1	95056486	95056944	459	95056914	8	3.74505	3.07688
Nc-1-IP_pe	chr1	95092360	95092791	432	95092741	11	3.89191	2.85898
Nc-1-IP_pe	chr1	95117316	95117988	673	95117849	14	6.61011	3.98382
Nc-1-IP_pe	chr1	95134545	95134808	264	95134660	12	4.24064	2.95299
Nc-1-IP_pe	chr1	95188068	95188382	315	95188192	10	5.23357	3.76063
Nc-1-IP_pe	chr1	95232140	95232401	262	95232233	11	5.76039	3.93701
Nc-1-IP_pe	chr1	95234411	95234787	377	95234502	13	4.90859	3.20357
Nc-1-IP_pe	chr1	95483047	95483681	635	95483539	11	5.52654	3.79706
Nc-1-IP_pe	chr1	95646953	95647491	539	95647315	9	4.46937	3.41876
Nc-1-IP_pe	chr1	95668860	95669892	1033	95669623	15	7.46994	4.29894
Nc-1-IP_pe	chr1	95752588	95753457	870	95753265	12	5.77527	3.80164
Nc-1-IP_pe	chr1	95840388	95840712	325	95840708	9	3.66792	2.92434
Nc-1-IP_pe	chr1	95853516	95853783	268	95853641	12	4.70082	3.20033
Nc-1-IP_pe	chr1	96028106	96028443	338	96028179	8	3.74505	3.07688
Nc-1-IP_pe	chr1	96057090	96057519	430	96057231	11	2.96397	2.36091
Nc-1-IP_pe	chr1	96192633	96192965	333	96192824	12	6.86906	4.44438
Nc-1-IP_pe	chr1	96288663	96288994	332	96288783	12	6.12485	4.004
Nc-1-IP_pe	chr1	96447626	96448327	702	96448017	21	6.2632	3.08808
Nc-1-IP_pe	chr1	96469709	96470150	442	96469850	13	5.9487	3.76157
Nc-1-IP_pe	chr1	96571172	96571435	264	96571314	12	6.86906	4.44438
Nc-1-IP_pe	chr1	96574858	96575165	308	96574910	10	3.89064	2.95552
Nc-1-IP_pe	chr1	96659533	96659843	311	96659633	10	5.23357	3.76063
Nc-1-IP_pe	chr1	96662689	96662992	304	96662860	9	3.72192	2.95716
Nc-1-IP_pe	chr1	96682289	96682567	279	96682410	9	3.72192	2.95716
Nc-1-IP_pe	chr1	96736844	96737153	310	96737070	9	3.54889	2.85226
Nc-1-IP_pe	chr1	96762817	96763434	618	96763167	13	3.07238	2.29378
Nc-1-IP_pe	chr1	96861943	96862871	929	96862870	10	3.08907	2.49869

Nc-1-IP_pe	chr1	97056687	97057151	465	97056921	14	7.42849	4.43574
Nc-1-IP_pe	chr1	97287157	97287470	314	97287223	14	5.03085	3.16286
Nc-1-IP_pe	chr1	97358281	97358616	336	97358337	11	3.64839	2.72584
Nc-1-IP_pe	chr1	98049424	98049696	273	98049520	11	5.65062	3.87116
Nc-1-IP_pe	chr1	98317408	98317731	324	98317537	14	8.63073	5.12814
Nc-1-IP_pe	chr1	98367097	98367628	532	98367177	10	4.99888	3.61677
Nc-1-IP_pe	chr1	98512036	98512766	731	98512467	17	8.31233	4.43123
Nc-1-IP_pe	chr1	98513159	98513522	364	98513297	13	7.7352	4.78626
Nc-1-IP_pe	chr1	98588705	98589426	722	98588841	10	3.89064	2.95552
Nc-1-IP_pe	chr1	98624625	98624901	277	98624715	11	6.03439	4.10251
Nc-1-IP_pe	chr1	98723612	98723882	271	98723744	13	7.42513	4.60316
Nc-1-IP_pe	chr1	98809130	98809559	430	98809366	8	3.74505	3.07688
Nc-1-IP_pe	chr1	98827725	98827984	260	98827884	10	5.23357	3.76063
Nc-1-IP_pe	chr1	98866321	98866633	313	98866607	10	4.99888	3.61677
Nc-1-IP_pe	chr1	99036797	99037049	253	99036932	10	5.23357	3.76063
Nc-1-IP_pe	chr1	99051812	99052513	702	99052001	14	6.69566	4.03025
Nc-1-IP_pe	chr1	99078969	99079354	386	99079295	11	3.64839	2.72584
Nc-1-IP_pe	chr1	99089209	99089659	451	99089490	8	3.56021	2.95918
Nc-1-IP_pe	chr1	99161488	99161970	483	99161646	10	5.23357	3.76063
Nc-1-IP_pe	chr1	99264302	99264837	536	99264341	16	5.37454	3.13479
Nc-1-IP_pe	chr1	99378199	99378455	257	99378339	12	6.86906	4.44438
Nc-1-IP_pe	chr1	99417714	99418045	332	99417836	13	6.72991	4.20021
Nc-1-IP_pe	chr1	99508341	99508838	498	99508465	11	4.54404	3.2242
Nc-1-IP_pe	chr1	99510015	99510384	370	99510084	12	3.83688	2.74114
Nc-1-IP_pe	chr1	99753505	99753786	282	99753684	9	4.46937	3.41876
Nc-1-IP_pe	chr1	99823415	99823864	450	99823748	13	4.02562	2.7544
Nc-1-IP_pe	chr1	99856581	99856996	416	99856600	8	3.74505	3.07688
Nc-1-IP_pe	chr1	99938584	99938846	263	99938753	10	3.89064	2.95552
Nc-1-IP_pe	chr1	99949850	99950228	379	99950052	11	5.10577	3.5486
Nc-1-IP_pe	chr1	99969509	99970219	711	99970030	24	13.4032	5.72066
Nc-1-IP_pe	chr1	99971921	99972197	277	99972001	13	7.7352	4.78626
Nc-1-IP_pe	chr1	1E+08	1E+08	594	1E+08	8	3.56021	2.95918
Nc-1-IP_pe	chr1	1E+08	1E+08	369	1E+08	10	3.89064	2.95552
Nc-1-IP_pe	chr1	1E+08	1E+08	265	1E+08	13	7.7352	4.78626
Nc-1-IP_pe	chr1	1E+08	1E+08	454	1E+08	12	4.70082	3.20033
Nc-1-IP_pe	chr1	1E+08	1E+08	285	1E+08	12	6.58417	4.27436
Nc-1-IP_pe	chr1	1E+08	1E+08	660	1E+08	20	13.9136	6.83166
Nc-1-IP_pe	chr1	1E+08	1E+08	349	1E+08	14	6.06519	3.69269
Nc-1-IP_pe	chr1	1E+08	1E+08	431	1E+08	11	5.10577	3.5486
Nc-1-IP_pe	chr1	1E+08	1E+08	329	1E+08	10	5.23357	3.76063
Nc-1-IP_pe	chr1	1E+08	1E+08	346	1E+08	11	6.03439	4.10251
Nc-1-IP_pe	chr1	1E+08	1E+08	438	1E+08	15	4.7819	2.95039
Nc-1-IP_pe	chr1	1E+08	1E+08	271	1E+08	10	5.23357	3.76063
Nc-1-IP_pe	chr1	1E+08	1E+08	653	1E+08	9	4.25966	3.28797
Nc-1-IP_pe	chr1	1E+08	1E+08	282	1E+08	13	6.62436	4.14003

Nc-1-IP_pe	chr1	1.01E+08	1.01E+08	324	1.01E+08	10	5.23357	3.76063
Nc-1-IP_pe	chr1	1.01E+08	1.01E+08	669	1.01E+08	19	9.15219	4.54307
Nc-1-IP_pe	chr1	1.01E+08	1.01E+08	369	1.01E+08	13	7.7352	4.78626
Nc-1-IP_pe	chr1	1.01E+08	1.01E+08	390	1.01E+08	12	5.84943	3.84431
Nc-1-IP_pe	chr1	1.01E+08	1.01E+08	439	1.01E+08	10	3.4604	2.70797
Nc-1-IP_pe	chr1	1.01E+08	1.01E+08	269	1.01E+08	15	5.20142	3.14789
Nc-1-IP_pe	chr1	1.01E+08	1.01E+08	258	1.01E+08	14	8.29543	4.93196
Nc-1-IP_pe	chr1	1.01E+08	1.01E+08	309	1.01E+08	9	4.46937	3.41876
Nc-1-IP_pe	chr1	1.01E+08	1.01E+08	263	1.01E+08	13	2.94177	2.23242
Nc-1-IP_pe	chr1	1.01E+08	1.01E+08	264	1.01E+08	13	6.54342	4.09407
Nc-1-IP_pe	chr1	1.01E+08	1.01E+08	276	1.01E+08	11	6.03439	4.10251
Nc-1-IP_pe	chr1	1.01E+08	1.01E+08	264	1.01E+08	12	5.94619	3.9002
Nc-1-IP_pe	chr1	1.01E+08	1.01E+08	424	1.01E+08	11	5.77463	3.94557
Nc-1-IP_pe	chr1	1.01E+08	1.01E+08	475	1.01E+08	11	5.10577	3.5486
Nc-1-IP_pe	chr1	1.01E+08	1.01E+08	477	1.01E+08	9	3.54889	2.85226
Nc-1-IP_pe	chr1	1.01E+08	1.01E+08	332	1.01E+08	11	5.03835	3.5092
Nc-1-IP_pe	chr1	1.01E+08	1.01E+08	448	1.01E+08	9	4.20361	3.25317
Nc-1-IP_pe	chr1	1.01E+08	1.01E+08	258	1.01E+08	12	6.86906	4.44438
Nc-1-IP_pe	chr1	1.02E+08	1.02E+08	261	1.02E+08	12	6.86906	4.44438
Nc-1-IP_pe	chr1	1.02E+08	1.02E+08	300	1.02E+08	9	4.20361	3.25317
Nc-1-IP_pe	chr1	1.02E+08	1.02E+08	261	1.02E+08	9	4.46937	3.41876
Nc-1-IP_pe	chr1	1.02E+08	1.02E+08	771	1.02E+08	9	4.46937	3.41876
Nc-1-IP_pe	chr1	1.03E+08	1.03E+08	313	1.03E+08	15	8.26005	4.73146
Nc-1-IP_pe	chr1	1.03E+08	1.03E+08	359	1.03E+08	20	5.70098	2.94771
Nc-1-IP_pe	chr1	1.03E+08	1.03E+08	344	1.03E+08	12	3.83688	2.74114
Nc-1-IP_pe	chr1	1.03E+08	1.03E+08	555	1.03E+08	12	3.47963	2.55766
Nc-1-IP_pe	chr1	1.03E+08	1.03E+08	259	1.03E+08	12	6.86906	4.44438
Nc-1-IP_pe	chr1	1.03E+08	1.03E+08	274	1.03E+08	9	4.46937	3.41876
Nc-1-IP_pe	chr1	1.03E+08	1.03E+08	552	1.03E+08	15	9.35725	5.35563
Nc-1-IP_pe	chr1	1.03E+08	1.03E+08	444	1.03E+08	11	5.10577	3.5486
Nc-1-IP_pe	chr1	1.03E+08	1.03E+08	253	1.03E+08	11	3.64839	2.72584
Nc-1-IP_pe	chr1	1.03E+08	1.03E+08	773	1.03E+08	11	5.89269	4.01672
Nc-1-IP_pe	chr1	1.03E+08	1.03E+08	437	1.03E+08	11	5.77463	3.94557
Nc-1-IP_pe	chr1	1.03E+08	1.03E+08	446	1.03E+08	8	3.74505	3.07688
Nc-1-IP_pe	chr1	1.04E+08	1.04E+08	270	1.04E+08	13	5.36853	3.44651
Nc-1-IP_pe	chr1	1.04E+08	1.04E+08	323	1.04E+08	10	5.23357	3.76063
Nc-1-IP_pe	chr1	1.04E+08	1.04E+08	320	1.04E+08	20	8.29312	4.01203
Nc-1-IP_pe	chr1	1.04E+08	1.04E+08	298	1.04E+08	16	8.17084	4.51499
Nc-1-IP_pe	chr1	1.04E+08	1.04E+08	449	1.04E+08	16	4.59224	2.7853
Nc-1-IP_pe	chr1	1.04E+08	1.04E+08	256	1.04E+08	16	4.59224	2.7853
Nc-1-IP_pe	chr1	1.04E+08	1.04E+08	266	1.04E+08	11	6.03439	4.10251
Nc-1-IP_pe	chr1	1.04E+08	1.04E+08	622	1.04E+08	10	5.23357	3.76063
Nc-1-IP_pe	chr1	1.04E+08	1.04E+08	323	1.04E+08	11	5.10577	3.5486
Nc-1-IP_pe	chr1	1.04E+08	1.04E+08	766	1.04E+08	11	5.10577	3.5486
Nc-1-IP_pe	chr1	1.04E+08	1.04E+08	414	1.04E+08	16	10.5028	5.81189

Nc-1-IP_pe	chr1	1.04E+08	1.04E+08	678	1.04E+08	10	4.47495	3.30017
Nc-1-IP_pe	chr1	1.04E+08	1.04E+08	270	1.04E+08	15	8.26005	4.73146
Nc-1-IP_pe	chr1	1.04E+08	1.04E+08	677	1.04E+08	11	5.77463	3.94557
Nc-1-IP_pe	chr1	1.04E+08	1.04E+08	503	1.04E+08	10	5.10552	3.682
Nc-1-IP_pe	chr1	1.05E+08	1.05E+08	337	1.05E+08	9	4.25966	3.28797
Nc-1-IP_pe	chr1	1.05E+08	1.05E+08	316	1.05E+08	7	3.06442	2.73501
Nc-1-IP_pe	chr1	1.05E+08	1.05E+08	433	1.05E+08	10	4.07292	3.06202
Nc-1-IP_pe	chr1	1.05E+08	1.05E+08	542	1.05E+08	15	5.20142	3.14789
Nc-1-IP_pe	chr1	1.05E+08	1.05E+08	541	1.05E+08	13	5.36853	3.44651
Nc-1-IP_pe	chr1	1.05E+08	1.05E+08	403	1.05E+08	15	6.78905	3.93887
Nc-1-IP_pe	chr1	1.05E+08	1.05E+08	313	1.05E+08	13	7.7352	4.78626
Nc-1-IP_pe	chr1	1.05E+08	1.05E+08	399	1.05E+08	9	4.46937	3.41876
Nc-1-IP_pe	chr1	1.05E+08	1.05E+08	377	1.05E+08	15	7.46994	4.29894
Nc-1-IP_pe	chr1	1.05E+08	1.05E+08	361	1.05E+08	10	5.23357	3.76063
Nc-1-IP_pe	chr1	1.05E+08	1.05E+08	329	1.05E+08	9	3.27326	2.68684
Nc-1-IP_pe	chr1	1.05E+08	1.05E+08	521	1.05E+08	10	4.99888	3.61677
Nc-1-IP_pe	chr1	1.05E+08	1.05E+08	340	1.05E+08	10	4.99888	3.61677
Nc-1-IP_pe	chr1	1.05E+08	1.05E+08	854	1.05E+08	12	6.86906	4.44438
Nc-1-IP_pe	chr1	1.05E+08	1.05E+08	489	1.05E+08	12	5.77527	3.80164
Nc-1-IP_pe	chr1	1.06E+08	1.06E+08	260	1.06E+08	14	7.42849	4.43574
Nc-1-IP_pe	chr1	1.06E+08	1.06E+08	346	1.06E+08	10	4.39571	3.25288
Nc-1-IP_pe	chr1	1.06E+08	1.06E+08	306	1.06E+08	12	5.23082	3.49289
Nc-1-IP_pe	chr1	1.06E+08	1.06E+08	281	1.06E+08	8	3.08755	2.66145
Nc-1-IP_pe	chr1	1.06E+08	1.06E+08	548	1.06E+08	8	3.56021	2.95918
Nc-1-IP_pe	chr1	1.06E+08	1.06E+08	360	1.06E+08	9	4.46937	3.41876
Nc-1-IP_pe	chr1	1.06E+08	1.06E+08	610	1.06E+08	9	4.46937	3.41876
Nc-1-IP_pe	chr1	1.06E+08	1.06E+08	264	1.06E+08	13	7.7352	4.78626
Nc-1-IP_pe	chr1	1.06E+08	1.06E+08	521	1.06E+08	14	6.78215	4.0774
Nc-1-IP_pe	chr1	1.07E+08	1.07E+08	829	1.07E+08	11	3.64839	2.72584
Nc-1-IP_pe	chr1	1.07E+08	1.07E+08	322	1.07E+08	9	4.46937	3.41876
Nc-1-IP_pe	chr1	1.07E+08	1.07E+08	285	1.07E+08	9	3.72192	2.95716
Nc-1-IP_pe	chr1	1.07E+08	1.07E+08	569	1.07E+08	16	10.2925	5.69036
Nc-1-IP_pe	chr1	1.07E+08	1.07E+08	678	1.07E+08	15	8.26005	4.73146
Nc-1-IP_pe	chr1	1.07E+08	1.07E+08	266	1.07E+08	10	4.93609	3.57849
Nc-1-IP_pe	chr1	1.08E+08	1.08E+08	280	1.08E+08	13	7.7352	4.78626
Nc-1-IP_pe	chr1	1.08E+08	1.08E+08	683	1.08E+08	11	5.03835	3.5092
Nc-1-IP_pe	chr1	1.08E+08	1.08E+08	397	1.08E+08	13	5.36853	3.44651
Nc-1-IP_pe	chr1	1.08E+08	1.08E+08	380	1.08E+08	12	5.23082	3.49289
Nc-1-IP_pe	chr1	1.08E+08	1.08E+08	270	1.08E+08	8	3.74505	3.07688
Nc-1-IP_pe	chr1	1.08E+08	1.08E+08	314	1.08E+08	18	7.04825	3.69086
Nc-1-IP_pe	chr1	1.08E+08	1.08E+08	300	1.08E+08	10	3.40382	2.67582
Nc-1-IP_pe	chr1	1.08E+08	1.08E+08	454	1.08E+08	9	4.46937	3.41876
Nc-1-IP_pe	chr1	1.08E+08	1.08E+08	362	1.08E+08	10	3.4604	2.70797
Nc-1-IP_pe	chr1	1.08E+08	1.08E+08	332	1.08E+08	15	6.78905	3.93887
Nc-1-IP_pe	chr1	1.08E+08	1.08E+08	363	1.08E+08	16	5.82808	3.34463

Nc-1-IP_pe	chr1	1.08E+08	1.08E+08	430	1.08E+08	11	5.10577	3.5486
Nc-1-IP_pe	chr1	1.08E+08	1.08E+08	404	1.08E+08	12	5.94619	3.9002
Nc-1-IP_pe	chr1	1.08E+08	1.08E+08	272	1.08E+08	8	3.08755	2.66145
Nc-1-IP_pe	chr1	1.08E+08	1.08E+08	375	1.08E+08	9	4.46937	3.41876
Nc-1-IP_pe	chr1	1.08E+08	1.08E+08	336	1.08E+08	10	4.39571	3.25288
Nc-1-IP_pe	chr1	1.08E+08	1.08E+08	490	1.08E+08	10	5.23357	3.76063
Nc-1-IP_pe	chr1	1.08E+08	1.08E+08	262	1.08E+08	9	4.46937	3.41876
Nc-1-IP_pe	chr1	1.08E+08	1.08E+08	352	1.08E+08	10	5.23357	3.76063
Nc-1-IP_pe	chr1	1.08E+08	1.08E+08	309	1.08E+08	12	5.23082	3.49289
Nc-1-IP_pe	chr1	1.08E+08	1.08E+08	444	1.08E+08	13	4.86362	3.18015
Nc-1-IP_pe	chr1	1.08E+08	1.08E+08	550	1.08E+08	11	5.3563	3.696
Nc-1-IP_pe	chr1	1.08E+08	1.08E+08	572	1.08E+08	11	3.28475	2.53029
Nc-1-IP_pe	chr1	1.09E+08	1.09E+08	759	1.09E+08	15	6.69889	3.8921
Nc-1-IP_pe	chr1	1.09E+08	1.09E+08	270	1.09E+08	12	6.86906	4.44438
Nc-1-IP_pe	chr1	1.09E+08	1.09E+08	331	1.09E+08	28	9.63986	3.71574
Nc-1-IP_pe	chr1	1.09E+08	1.09E+08	314	1.09E+08	11	4.54404	3.2242
Nc-1-IP_pe	chr1	1.09E+08	1.09E+08	1419	1.09E+08	15	6.78905	3.93887
Nc-1-IP_pe	chr1	1.09E+08	1.09E+08	393	1.09E+08	9	4.46937	3.41876
Nc-1-IP_pe	chr1	1.09E+08	1.09E+08	293	1.09E+08	12	6.31185	4.11348
Nc-1-IP_pe	chr1	1.09E+08	1.09E+08	285	1.09E+08	13	4.24481	2.8638
Nc-1-IP_pe	chr1	1.09E+08	1.09E+08	340	1.09E+08	9	4.35492	3.34727
Nc-1-IP_pe	chr1	1.09E+08	1.09E+08	675	1.09E+08	12	5.84943	3.84431
Nc-1-IP_pe	chr1	1.09E+08	1.09E+08	440	1.09E+08	15	7.46994	4.29894
Nc-1-IP_pe	chr1	1.09E+08	1.09E+08	263	1.09E+08	11	6.03439	4.10251
Nc-1-IP_pe	chr1	1.09E+08	1.09E+08	395	1.09E+08	12	4.88508	3.30112
Nc-1-IP_pe	chr1	1.09E+08	1.09E+08	396	1.09E+08	15	3.74763	2.48304
Nc-1-IP_pe	chr1	1.09E+08	1.09E+08	628	1.09E+08	11	3.28475	2.53029
Nc-1-IP_pe	chr1	1.09E+08	1.09E+08	288	1.09E+08	10	5.23357	3.76063
Nc-1-IP_pe	chr1	1.09E+08	1.09E+08	571	1.09E+08	9	4.46937	3.41876
Nc-1-IP_pe	chr1	1.09E+08	1.09E+08	568	1.09E+08	11	5.77463	3.94557
Nc-1-IP_pe	chr1	1.09E+08	1.09E+08	282	1.09E+08	9	4.46937	3.41876
Nc-1-IP_pe	chr1	1.09E+08	1.09E+08	640	1.09E+08	15	4.90347	3.00714
Nc-1-IP_pe	chr1	1.09E+08	1.09E+08	328	1.09E+08	13	3.21799	2.36264
Nc-1-IP_pe	chr1	1.09E+08	1.09E+08	645	1.09E+08	9	4.3705	3.35698
Nc-1-IP_pe	chr1	1.09E+08	1.09E+08	261	1.09E+08	11	4.74697	3.34038
Nc-1-IP_pe	chr1	1.09E+08	1.09E+08	313	1.09E+08	10	3.71867	2.85592
Nc-1-IP_pe	chr1	1.09E+08	1.09E+08	299	1.09E+08	15	8.26005	4.73146
Nc-1-IP_pe	chr1	1.09E+08	1.09E+08	287	1.09E+08	9	3.93633	3.08822
Nc-1-IP_pe	chr1	1.09E+08	1.09E+08	403	1.09E+08	8	3.74505	3.07688
Nc-1-IP_pe	chr1	1.09E+08	1.09E+08	647	1.09E+08	10	4.88691	3.54856
Nc-1-IP_pe	chr1	1.09E+08	1.09E+08	374	1.09E+08	16	4.25252	2.63824
Nc-1-IP_pe	chr1	1.09E+08	1.09E+08	911	1.09E+08	12	6.46791	4.20548
Nc-1-IP_pe	chr1	1.09E+08	1.09E+08	273	1.09E+08	8	3.74505	3.07688
Nc-1-IP_pe	chr1	1.09E+08	1.09E+08	859	1.09E+08	13	4.41954	2.952
Nc-1-IP_pe	chr1	1.09E+08	1.09E+08	306	1.09E+08	21	7.58657	3.60451

Nc-1-IP_pe	chr1	1.09E+08	1.09E+08	379	1.09E+08	14	6.69566	4.03025
Nc-1-IP_pe	chr1	1.09E+08	1.09E+08	630	1.09E+08	13	5.36853	3.44651
Nc-1-IP_pe	chr1	1.09E+08	1.09E+08	305	1.09E+08	11	4.00079	2.91908
Nc-1-IP_pe	chr1	1.09E+08	1.09E+08	552	1.09E+08	15	5.20142	3.14789
Nc-1-IP_pe	chr1	1.09E+08	1.09E+08	485	1.09E+08	12	4.31492	2.99249
Nc-1-IP_pe	chr1	1.09E+08	1.09E+08	273	1.09E+08	15	4.80057	2.95908
Nc-1-IP_pe	chr1	1.09E+08	1.09E+08	299	1.09E+08	13	3.86418	2.67468
Nc-1-IP_pe	chr1	1.09E+08	1.09E+08	434	1.09E+08	19	8.19109	4.09114
Nc-1-IP_pe	chr1	1.09E+08	1.09E+08	406	1.09E+08	12	3.16137	2.39719
Nc-1-IP_pe	chr1	1.09E+08	1.09E+08	507	1.09E+08	11	4.17167	3.01412
Nc-1-IP_pe	chr1	1.09E+08	1.09E+08	430	1.09E+08	12	4.70523	3.20273
Nc-1-IP_pe	chr1	1.09E+08	1.09E+08	879	1.09E+08	13	5.42865	3.47872
Nc-1-IP_pe	chr1	1.09E+08	1.09E+08	471	1.09E+08	11	4.01189	2.92523
Nc-1-IP_pe	chr1	1.09E+08	1.09E+08	285	1.09E+08	16	4.59224	2.7853
Nc-1-IP_pe	chr1	1.09E+08	1.09E+08	409	1.09E+08	13	5.08264	3.29478
Nc-1-IP_pe	chr1	1.09E+08	1.09E+08	341	1.09E+08	17	3.84884	2.41145
Nc-1-IP_pe	chr1	1.09E+08	1.09E+08	399	1.09E+08	13	6.38222	4.00303
Nc-1-IP_pe	chr1	1.09E+08	1.09E+08	654	1.09E+08	11	6.03439	4.10251
Nc-1-IP_pe	chr1	1.09E+08	1.09E+08	405	1.09E+08	15	5.1177	3.10809
Nc-1-IP_pe	chr1	1.09E+08	1.09E+08	517	1.09E+08	9	2.89249	2.46179
Nc-1-IP_pe	chr1	1.09E+08	1.09E+08	327	1.09E+08	11	6.03439	4.10251
Nc-1-IP_pe	chr1	1.09E+08	1.09E+08	459	1.09E+08	13	7.7352	4.78626
Nc-1-IP_pe	chr1	1.09E+08	1.09E+08	439	1.09E+08	12	4.70082	3.20033
Nc-1-IP_pe	chr1	1.09E+08	1.09E+08	711	1.09E+08	19	8.68415	4.32024
Nc-1-IP_pe	chr1	1.09E+08	1.09E+08	299	1.09E+08	11	4.00079	2.91908
Nc-1-IP_pe	chr1	1.09E+08	1.09E+08	431	1.09E+08	13	4.41954	2.952
Nc-1-IP_pe	chr1	1.1E+08	1.1E+08	771	1.1E+08	17	5.89608	3.2766
Nc-1-IP_pe	chr1	1.1E+08	1.1E+08	300	1.1E+08	9	4.1889	3.24405
Nc-1-IP_pe	chr1	1.1E+08	1.1E+08	521	1.1E+08	11	3.64839	2.72584
Nc-1-IP_pe	chr1	1.1E+08	1.1E+08	280	1.1E+08	11	5.89269	4.01672
Nc-1-IP_pe	chr1	1.1E+08	1.1E+08	668	1.1E+08	13	5.25176	3.38425
Nc-1-IP_pe	chr1	1.1E+08	1.1E+08	325	1.1E+08	15	4.40338	2.77622
Nc-1-IP_pe	chr1	1.1E+08	1.1E+08	354	1.1E+08	11	4.23076	3.04719
Nc-1-IP_pe	chr1	1.1E+08	1.1E+08	1285	1.1E+08	14	6.06519	3.69269
Nc-1-IP_pe	chr1	1.1E+08	1.1E+08	695	1.1E+08	12	3.83688	2.74114
Nc-1-IP_pe	chr1	1.1E+08	1.1E+08	341	1.1E+08	13	6.59853	4.12535
Nc-1-IP_pe	chr1	1.1E+08	1.1E+08	779	1.1E+08	14	4.60011	2.95114
Nc-1-IP_pe	chr1	1.1E+08	1.1E+08	312	1.1E+08	11	4.87504	3.4143
Nc-1-IP_pe	chr1	1.1E+08	1.1E+08	1195	1.1E+08	19	10.9809	5.46219
Nc-1-IP_pe	chr1	1.1E+08	1.1E+08	383	1.1E+08	13	3.35769	2.42919
Nc-1-IP_pe	chr1	1.1E+08	1.1E+08	307	1.1E+08	8	3.74505	3.07688
Nc-1-IP_pe	chr1	1.1E+08	1.1E+08	333	1.1E+08	10	3.59902	2.78714
Nc-1-IP_pe	chr1	1.1E+08	1.1E+08	338	1.1E+08	12	4.88508	3.30112
Nc-1-IP_pe	chr1	1.1E+08	1.1E+08	284	1.1E+08	14	6.06519	3.69269
Nc-1-IP_pe	chr1	1.1E+08	1.1E+08	520	1.1E+08	16	7.39088	4.10945

Nc-1-IP_pe	chr1	1.1E+08	1.1E+08	700	1.1E+08	15	7.46994	4.29894
Nc-1-IP_pe	chr1	1.1E+08	1.1E+08	445	1.1E+08	11	3.64839	2.72584
Nc-1-IP_pe	chr1	1.1E+08	1.1E+08	1052	1.1E+08	15	5.00915	3.05678
Nc-1-IP_pe	chr1	1.1E+08	1.1E+08	379	1.1E+08	11	4.35236	3.11555
Nc-1-IP_pe	chr1	1.1E+08	1.1E+08	267	1.1E+08	9	4.05981	3.16422
Nc-1-IP_pe	chr1	1.1E+08	1.1E+08	340	1.1E+08	13	6.22691	3.91595
Nc-1-IP_pe	chr1	1.1E+08	1.1E+08	458	1.1E+08	12	3.16137	2.39719
Nc-1-IP_pe	chr1	1.1E+08	1.1E+08	413	1.1E+08	12	5.17301	3.46058
Nc-1-IP_pe	chr1	1.1E+08	1.1E+08	299	1.1E+08	14	6.06519	3.69269
Nc-1-IP_pe	chr1	1.1E+08	1.1E+08	528	1.1E+08	13	4.04902	2.76601
Nc-1-IP_pe	chr1	1.1E+08	1.1E+08	308	1.1E+08	16	3.94146	2.50592
Nc-1-IP_pe	chr1	1.1E+08	1.1E+08	427	1.1E+08	11	5.68685	3.89286
Nc-1-IP_pe	chr1	1.1E+08	1.1E+08	358	1.1E+08	14	5.03085	3.16286
Nc-1-IP_pe	chr1	1.1E+08	1.1E+08	350	1.1E+08	11	4.67786	3.30068
Nc-1-IP_pe	chr1	1.1E+08	1.1E+08	264	1.1E+08	13	5.41476	3.47126
Nc-1-IP_pe	chr1	1.1E+08	1.1E+08	287	1.1E+08	12	4.06753	2.86157
Nc-1-IP_pe	chr1	1.1E+08	1.1E+08	390	1.1E+08	16	8.26955	4.56741
Nc-1-IP_pe	chr1	1.1E+08	1.1E+08	280	1.1E+08	14	6.28469	3.80898
Nc-1-IP_pe	chr1	1.1E+08	1.1E+08	279	1.1E+08	16	8.09318	4.47392
Nc-1-IP_pe	chr1	1.1E+08	1.1E+08	374	1.1E+08	15	7.46994	4.29894
Nc-1-IP_pe	chr1	1.1E+08	1.1E+08	273	1.1E+08	12	6.77747	4.38954
Nc-1-IP_pe	chr1	1.1E+08	1.1E+08	361	1.1E+08	13	5.03208	3.26819
Nc-1-IP_pe	chr1	1.1E+08	1.1E+08	384	1.1E+08	14	5.51541	3.4073
Nc-1-IP_pe	chr1	1.1E+08	1.1E+08	285	1.1E+08	12	5.45448	3.61875
Nc-1-IP_pe	chr1	1.1E+08	1.1E+08	432	1.1E+08	12	4.51041	3.09723
Nc-1-IP_pe	chr1	1.1E+08	1.1E+08	260	1.1E+08	15	4.7819	2.95039
Nc-1-IP_pe	chr1	1.1E+08	1.1E+08	305	1.1E+08	9	4.24819	3.28084
Nc-1-IP_pe	chr1	1.1E+08	1.1E+08	279	1.1E+08	13	4.02562	2.7544
Nc-1-IP_pe	chr1	1.1E+08	1.1E+08	300	1.1E+08	11	4.54377	3.22405
Nc-1-IP_pe	chr1	1.1E+08	1.1E+08	484	1.1E+08	11	3.28475	2.53029
Nc-1-IP_pe	chr1	1.1E+08	1.1E+08	633	1.1E+08	11	2.96397	2.36091
Nc-1-IP_pe	chr1	1.1E+08	1.1E+08	340	1.1E+08	15	7.46994	4.29894
Nc-1-IP_pe	chr1	1.1E+08	1.1E+08	711	1.1E+08	14	7.42849	4.43574
Nc-1-IP_pe	chr1	1.1E+08	1.1E+08	1028	1.1E+08	18	10.7634	5.54224
Nc-1-IP_pe	chr1	1.1E+08	1.1E+08	415	1.1E+08	12	3.77112	2.7071
Nc-1-IP_pe	chr1	1.1E+08	1.1E+08	259	1.1E+08	13	3.07238	2.29378
Nc-1-IP_pe	chr1	1.1E+08	1.1E+08	513	1.1E+08	12	4.70082	3.20033
Nc-1-IP_pe	chr1	1.1E+08	1.1E+08	399	1.1E+08	12	5.21606	3.48463
Nc-1-IP_pe	chr1	1.1E+08	1.1E+08	292	1.1E+08	9	2.89249	2.46179
Nc-1-IP_pe	chr1	1.1E+08	1.1E+08	977	1.1E+08	12	5.84943	3.84431
Nc-1-IP_pe	chr1	1.1E+08	1.1E+08	301	1.1E+08	9	4.00426	3.12999
Nc-1-IP_pe	chr1	1.1E+08	1.1E+08	1061	1.1E+08	15	7.46994	4.29894
Nc-1-IP_pe	chr1	1.1E+08	1.1E+08	539	1.1E+08	10	4.0855	3.0694
Nc-1-IP_pe	chr1	1.1E+08	1.1E+08	418	1.1E+08	8	3.74505	3.07688
Nc-1-IP_pe	chr1	1.1E+08	1.1E+08	515	1.1E+08	9	2.89249	2.46179

Nc-1-IP_pe chr1	1.1E+08	1.1E+08	296	1.1E+08	11	5.52654	3.79706
Nc-1-IP_pe chr1	1.1E+08	1.1E+08	437	1.1E+08	12	5.23082	3.49289
Nc-1-IP_pe chr1	1.1E+08	1.1E+08	432	1.1E+08	13	4.41954	2.952
Nc-1-IP_pe chr1	1.1E+08	1.1E+08	409	1.1E+08	13	3.67377	2.58159
Nc-1-IP_pe chr1	1.1E+08	1.1E+08	316	1.1E+08	11	3.49235	2.64145
Nc-1-IP_pe chr1	1.1E+08	1.1E+08	287	1.1E+08	11	3.58722	2.69267
Nc-1-IP_pe chr1	1.1E+08	1.1E+08	639	1.1E+08	12	3.47963	2.55766
Nc-1-IP_pe chr1	1.1E+08	1.1E+08	288	1.1E+08	12	4.24064	2.95299
Nc-1-IP_pe chr1	1.1E+08	1.1E+08	494	1.1E+08	12	5.77527	3.80164
Nc-1-IP_pe chr1	1.1E+08	1.1E+08	312	1.1E+08	13	4.86362	3.18015
Nc-1-IP_pe chr1	1.1E+08	1.1E+08	778	1.1E+08	18	6.65364	3.51391
Nc-1-IP_pe chr1	1.1E+08	1.1E+08	270	1.1E+08	18	6.91333	3.62993
Nc-1-IP_pe chr1	1.1E+08	1.1E+08	542	1.1E+08	16	6.80394	3.81462
Nc-1-IP_pe chr1	1.1E+08	1.1E+08	260	1.1E+08	13	4.24481	2.8638
Nc-1-IP_pe chr1	1.1E+08	1.1E+08	567	1.1E+08	14	4.42328	2.86573
Nc-1-IP_pe chr1	1.1E+08	1.1E+08	947	1.1E+08	15	6.12018	3.5972
Nc-1-IP_pe chr1	1.1E+08	1.1E+08	375	1.1E+08	11	4.00079	2.91908
Nc-1-IP_pe chr1	1.1E+08	1.1E+08	475	1.1E+08	13	5.76252	3.65944
Nc-1-IP_pe chr1	1.1E+08	1.1E+08	352	1.1E+08	11	4.54404	3.2242
Nc-1-IP_pe chr1	1.1E+08	1.1E+08	309	1.1E+08	11	4.47871	3.18705
Nc-1-IP_pe chr1	1.1E+08	1.1E+08	265	1.1E+08	12	5.84943	3.84431
Nc-1-IP_pe chr1	1.1E+08	1.1E+08	304	1.1E+08	13	3.67377	2.58159
Nc-1-IP_pe chr1	1.1E+08	1.1E+08	282	1.1E+08	10	4.62142	3.388
Nc-1-IP_pe chr1	1.1E+08	1.1E+08	866	1.1E+08	15	5.00915	3.05678
Nc-1-IP_pe chr1	1.1E+08	1.1E+08	431	1.1E+08	11	5.10577	3.5486
Nc-1-IP_pe chr1	1.1E+08	1.1E+08	391	1.1E+08	12	4.70082	3.20033
Nc-1-IP_pe chr1	1.1E+08	1.1E+08	283	1.1E+08	10	5.23357	3.76063
Nc-1-IP_pe chr1	1.1E+08	1.1E+08	268	1.1E+08	10	3.4604	2.70797
Nc-1-IP_pe chr1	1.1E+08	1.1E+08	705	1.1E+08	14	6.06519	3.69269
Nc-1-IP_pe chr1	1.1E+08	1.1E+08	559	1.1E+08	14	8.29543	4.93196
Nc-1-IP_pe chr1	1.1E+08	1.1E+08	449	1.1E+08	12	5.61153	3.70794
Nc-1-IP_pe chr1	1.1E+08	1.1E+08	460	1.1E+08	12	3.83688	2.74114
Nc-1-IP_pe chr1	1.1E+08	1.1E+08	437	1.1E+08	12	2.87625	2.25568
Nc-1-IP_pe chr1	1.1E+08	1.1E+08	289	1.1E+08	11	3.64839	2.72584
Nc-1-IP_pe chr1	1.1E+08	1.1E+08	449	1.1E+08	13	6.36468	3.99316
Nc-1-IP_pe chr1	1.1E+08	1.1E+08	281	1.1E+08	21	5.88587	2.94733
Nc-1-IP_pe chr1	1.1E+08	1.1E+08	328	1.1E+08	13	5.57038	3.55505
Nc-1-IP_pe chr1	1.11E+08	1.11E+08	338	1.11E+08	9	3.61966	2.89507
Nc-1-IP_pe chr1	1.11E+08	1.11E+08	607	1.11E+08	14	8.04269	4.78558
Nc-1-IP_pe chr1	1.11E+08	1.11E+08	273	1.11E+08	12	5.84943	3.84431
Nc-1-IP_pe chr1	1.11E+08	1.11E+08	982	1.11E+08	14	6.78215	4.0774
Nc-1-IP_pe chr1	1.11E+08	1.11E+08	279	1.11E+08	9	3.79246	3.00015
Nc-1-IP_pe chr1	1.11E+08	1.11E+08	488	1.11E+08	10	3.89064	2.95552
Nc-1-IP_pe chr1	1.11E+08	1.11E+08	436	1.11E+08	10	5.23357	3.76063
Nc-1-IP_pe chr1	1.11E+08	1.11E+08	261	1.11E+08	9	3.54889	2.85226

Nc-1-IP_pe	chr1	1.11E+08	1.11E+08	599	1.11E+08	13	6.19316	3.89711
Nc-1-IP_pe	chr1	1.11E+08	1.11E+08	476	1.11E+08	12	4.24064	2.95299
Nc-1-IP_pe	chr1	1.11E+08	1.11E+08	384	1.11E+08	10	3.89064	2.95552
Nc-1-IP_pe	chr1	1.11E+08	1.11E+08	357	1.11E+08	10	5.10552	3.682
Nc-1-IP_pe	chr1	1.11E+08	1.11E+08	294	1.11E+08	11	5.49185	3.77641
Nc-1-IP_pe	chr1	1.11E+08	1.11E+08	290	1.11E+08	9	4.05981	3.16422
Nc-1-IP_pe	chr1	1.11E+08	1.11E+08	399	1.11E+08	13	7.00513	4.3584
Nc-1-IP_pe	chr1	1.11E+08	1.11E+08	286	1.11E+08	9	2.89249	2.46179
Nc-1-IP_pe	chr1	1.11E+08	1.11E+08	454	1.11E+08	8	3.74505	3.07688
Nc-1-IP_pe	chr1	1.11E+08	1.11E+08	566	1.11E+08	10	4.39571	3.25288
Nc-1-IP_pe	chr1	1.11E+08	1.11E+08	287	1.11E+08	13	7.7352	4.78626
Nc-1-IP_pe	chr1	1.11E+08	1.11E+08	587	1.11E+08	12	6.86906	4.44438
Nc-1-IP_pe	chr1	1.11E+08	1.11E+08	490	1.11E+08	10	4.99888	3.61677
Nc-1-IP_pe	chr1	1.11E+08	1.11E+08	354	1.11E+08	9	4.46937	3.41876
Nc-1-IP_pe	chr1	1.11E+08	1.11E+08	291	1.11E+08	11	6.03439	4.10251
Nc-1-IP_pe	chr1	1.11E+08	1.11E+08	282	1.11E+08	12	5.59553	3.69882
Nc-1-IP_pe	chr1	1.11E+08	1.11E+08	376	1.11E+08	13	6.19316	3.89711
Nc-1-IP_pe	chr1	1.11E+08	1.11E+08	260	1.11E+08	11	5.13077	3.56323
Nc-1-IP_pe	chr1	1.11E+08	1.11E+08	650	1.11E+08	12	3.47963	2.55766
Nc-1-IP_pe	chr1	1.11E+08	1.11E+08	328	1.11E+08	12	6.86906	4.44438
Nc-1-IP_pe	chr1	1.11E+08	1.11E+08	374	1.11E+08	11	4.23076	3.04719
Nc-1-IP_pe	chr1	1.11E+08	1.11E+08	715	1.11E+08	13	7.7352	4.78626
Nc-1-IP_pe	chr1	1.11E+08	1.11E+08	321	1.11E+08	10	3.08907	2.49869
Nc-1-IP_pe	chr1	1.11E+08	1.11E+08	343	1.11E+08	8	3.74505	3.07688
Nc-1-IP_pe	chr1	1.11E+08	1.11E+08	363	1.11E+08	14	6.28469	3.80898
Nc-1-IP_pe	chr1	1.11E+08	1.11E+08	439	1.11E+08	11	6.03439	4.10251
Nc-1-IP_pe	chr1	1.12E+08	1.12E+08	469	1.12E+08	15	5.66913	3.37372
Nc-1-IP_pe	chr1	1.12E+08	1.12E+08	1403	1.12E+08	13	6.54342	4.09407
Nc-1-IP_pe	chr1	1.12E+08	1.12E+08	488	1.12E+08	19	7.59289	3.82098
Nc-1-IP_pe	chr1	1.12E+08	1.12E+08	800	1.12E+08	13	4.90859	3.20357
Nc-1-IP_pe	chr1	1.12E+08	1.12E+08	401	1.12E+08	10	5.23357	3.76063
Nc-1-IP_pe	chr1	1.12E+08	1.12E+08	308	1.12E+08	10	5.23357	3.76063
Nc-1-IP_pe	chr1	1.12E+08	1.12E+08	261	1.12E+08	9	3.63165	2.90233
Nc-1-IP_pe	chr1	1.12E+08	1.12E+08	601	1.12E+08	12	5.69253	3.7542
Nc-1-IP_pe	chr1	1.12E+08	1.12E+08	295	1.12E+08	12	5.23082	3.49289
Nc-1-IP_pe	chr1	1.12E+08	1.12E+08	779	1.12E+08	13	5.9487	3.76157
Nc-1-IP_pe	chr1	1.12E+08	1.12E+08	416	1.12E+08	13	6.62436	4.14003
Nc-1-IP_pe	chr1	1.12E+08	1.12E+08	285	1.12E+08	10	4.62142	3.388
Nc-1-IP_pe	chr1	1.12E+08	1.12E+08	292	1.12E+08	9	3.93633	3.08822
Nc-1-IP_pe	chr1	1.12E+08	1.12E+08	377	1.12E+08	13	5.9487	3.76157
Nc-1-IP_pe	chr1	1.12E+08	1.12E+08	257	1.12E+08	13	5.9487	3.76157
Nc-1-IP_pe	chr1	1.12E+08	1.12E+08	546	1.12E+08	15	6.69889	3.8921
Nc-1-IP_pe	chr1	1.12E+08	1.12E+08	653	1.12E+08	10	4.4323	3.2747
Nc-1-IP_pe	chr1	1.12E+08	1.12E+08	351	1.12E+08	11	6.03439	4.10251
Nc-1-IP_pe	chr1	1.12E+08	1.12E+08	311	1.12E+08	11	5.77463	3.94557

Nc-1-IP_pe	chr1	1.12E+08	1.12E+08	685	1.12E+08	10	3.89064	2.95552
Nc-1-IP_pe	chr1	1.12E+08	1.12E+08	787	1.12E+08	14	7.9745	4.74633
Nc-1-IP_pe	chr1	1.12E+08	1.12E+08	464	1.12E+08	11	5.37297	3.70587
Nc-1-IP_pe	chr1	1.12E+08	1.12E+08	261	1.12E+08	10	4.72812	3.45232
Nc-1-IP_pe	chr1	1.12E+08	1.12E+08	267	1.12E+08	11	5.33971	3.68619
Nc-1-IP_pe	chr1	1.12E+08	1.12E+08	624	1.12E+08	13	7.19153	4.46655
Nc-1-IP_pe	chr1	1.12E+08	1.12E+08	299	1.12E+08	12	6.86906	4.44438
Nc-1-IP_pe	chr1	1.12E+08	1.12E+08	473	1.12E+08	8	3.14944	2.70014
Nc-1-IP_pe	chr1	1.12E+08	1.12E+08	496	1.12E+08	13	6.36468	3.99316
Nc-1-IP_pe	chr1	1.12E+08	1.12E+08	412	1.12E+08	9	4.33944	3.33761
Nc-1-IP_pe	chr1	1.12E+08	1.12E+08	294	1.12E+08	10	5.23357	3.76063
Nc-1-IP_pe	chr1	1.12E+08	1.12E+08	332	1.12E+08	10	4.95265	3.58858
Nc-1-IP_pe	chr1	1.12E+08	1.12E+08	679	1.12E+08	9	3.27326	2.68684
Nc-1-IP_pe	chr1	1.12E+08	1.12E+08	351	1.12E+08	13	7.7352	4.78626
Nc-1-IP_pe	chr1	1.12E+08	1.12E+08	257	1.12E+08	13	7.7352	4.78626
Nc-1-IP_pe	chr1	1.12E+08	1.12E+08	262	1.12E+08	11	4.23076	3.04719
Nc-1-IP_pe	chr1	1.12E+08	1.12E+08	280	1.12E+08	16	6.33285	3.58457
Nc-1-IP_pe	chr1	1.12E+08	1.12E+08	857	1.12E+08	9	4.46937	3.41876
Nc-1-IP_pe	chr1	1.12E+08	1.12E+08	296	1.12E+08	10	3.4604	2.70797
Nc-1-IP_pe	chr1	1.12E+08	1.12E+08	292	1.12E+08	10	4.39571	3.25288
Nc-1-IP_pe	chr1	1.12E+08	1.12E+08	435	1.12E+08	8	3.56021	2.95918
Nc-1-IP_pe	chr1	1.12E+08	1.12E+08	539	1.12E+08	12	6.86906	4.44438
Nc-1-IP_pe	chr1	1.12E+08	1.12E+08	318	1.12E+08	8	3.65786	3.02128
Nc-1-IP_pe	chr1	1.12E+08	1.12E+08	376	1.12E+08	11	6.03439	4.10251
Nc-1-IP_pe	chr1	1.12E+08	1.12E+08	416	1.12E+08	9	3.27326	2.68684
Nc-1-IP_pe	chr1	1.12E+08	1.12E+08	335	1.12E+08	8	3.68553	3.03891
Nc-1-IP_pe	chr1	1.12E+08	1.12E+08	414	1.12E+08	12	6.31185	4.11348
Nc-1-IP_pe	chr1	1.12E+08	1.12E+08	617	1.12E+08	11	3.28475	2.53029
Nc-1-IP_pe	chr1	1.12E+08	1.12E+08	1080	1.12E+08	17	7.02067	3.79543
Nc-1-IP_pe	chr1	1.12E+08	1.12E+08	289	1.12E+08	11	5.77463	3.94557
Nc-1-IP_pe	chr1	1.12E+08	1.12E+08	822	1.12E+08	10	4.39571	3.25288
Nc-1-IP_pe	chr1	1.13E+08	1.13E+08	380	1.13E+08	15	6.19428	3.63445
Nc-1-IP_pe	chr1	1.13E+08	1.13E+08	631	1.13E+08	15	4.7819	2.95039
Nc-1-IP_pe	chr1	1.13E+08	1.13E+08	841	1.13E+08	16	5.37454	3.13479
Nc-1-IP_pe	chr1	1.13E+08	1.13E+08	346	1.13E+08	10	4.22744	3.15303
Nc-1-IP_pe	chr1	1.13E+08	1.13E+08	361	1.13E+08	10	3.83201	2.92147
Nc-1-IP_pe	chr1	1.13E+08	1.13E+08	491	1.13E+08	13	7.7352	4.78626
Nc-1-IP_pe	chr1	1.13E+08	1.13E+08	366	1.13E+08	14	3.86727	2.6027
Nc-1-IP_pe	chr1	1.13E+08	1.13E+08	285	1.13E+08	9	2.89249	2.46179
Nc-1-IP_pe	chr1	1.13E+08	1.13E+08	312	1.13E+08	9	4.25966	3.28797
Nc-1-IP_pe	chr1	1.13E+08	1.13E+08	305	1.13E+08	10	3.52548	2.74507
Nc-1-IP_pe	chr1	1.13E+08	1.13E+08	396	1.13E+08	13	4.41954	2.952
Nc-1-IP_pe	chr1	1.13E+08	1.13E+08	376	1.13E+08	11	2.90689	2.33107
Nc-1-IP_pe	chr1	1.13E+08	1.13E+08	669	1.13E+08	16	6.33285	3.58457
Nc-1-IP_pe	chr1	1.13E+08	1.13E+08	406	1.13E+08	12	4.51041	3.09723

Nc-1-IP_pe	chr1	1.13E+08	1.13E+08	611	1.13E+08	10	4.39571	3.25288
Nc-1-IP_pe	chr1	1.13E+08	1.13E+08	367	1.13E+08	11	4.06403	2.95415
Nc-1-IP_pe	chr1	1.13E+08	1.13E+08	273	1.13E+08	13	5.86991	3.71823
Nc-1-IP_pe	chr1	1.13E+08	1.13E+08	282	1.13E+08	9	4.46937	3.41876
Nc-1-IP_pe	chr1	1.13E+08	1.13E+08	491	1.13E+08	10	4.39571	3.25288
Nc-1-IP_pe	chr1	1.13E+08	1.13E+08	375	1.13E+08	11	2.96397	2.36091
Nc-1-IP_pe	chr1	1.13E+08	1.13E+08	428	1.13E+08	12	5.3037	3.53374
Nc-1-IP_pe	chr1	1.13E+08	1.13E+08	368	1.13E+08	12	6.58417	4.27436
Nc-1-IP_pe	chr1	1.13E+08	1.13E+08	273	1.13E+08	10	4.39571	3.25288
Nc-1-IP_pe	chr1	1.13E+08	1.13E+08	406	1.13E+08	11	6.03439	4.10251
Nc-1-IP_pe	chr1	1.13E+08	1.13E+08	264	1.13E+08	11	5.10577	3.5486
Nc-1-IP_pe	chr1	1.13E+08	1.13E+08	663	1.13E+08	15	6.19428	3.63445
Nc-1-IP_pe	chr1	1.13E+08	1.13E+08	334	1.13E+08	14	5.03085	3.16286
Nc-1-IP_pe	chr1	1.13E+08	1.13E+08	362	1.13E+08	12	5.61153	3.70794
Nc-1-IP_pe	chr1	1.13E+08	1.13E+08	301	1.13E+08	14	5.51541	3.4073
Nc-1-IP_pe	chr1	1.13E+08	1.13E+08	383	1.13E+08	11	4.06403	2.95415
Nc-1-IP_pe	chr1	1.13E+08	1.13E+08	576	1.13E+08	17	4.44438	2.65333
Nc-1-IP_pe	chr1	1.13E+08	1.13E+08	700	1.13E+08	15	6.19428	3.63445
Nc-1-IP_pe	chr1	1.13E+08	1.13E+08	774	1.13E+08	11	4.54404	3.2242
Nc-1-IP_pe	chr1	1.13E+08	1.13E+08	628	1.13E+08	11	5.52654	3.79706
Nc-1-IP_pe	chr1	1.13E+08	1.13E+08	359	1.13E+08	13	6.19316	3.89711
Nc-1-IP_pe	chr1	1.13E+08	1.13E+08	295	1.13E+08	16	5.82808	3.34463
Nc-1-IP_pe	chr1	1.13E+08	1.13E+08	404	1.13E+08	14	4.21449	2.76599
Nc-1-IP_pe	chr1	1.13E+08	1.13E+08	449	1.13E+08	13	4.41954	2.952
Nc-1-IP_pe	chr1	1.13E+08	1.13E+08	286	1.13E+08	8	3.74505	3.07688
Nc-1-IP_pe	chr1	1.13E+08	1.13E+08	1257	1.13E+08	12	6.86906	4.44438
Nc-1-IP_pe	chr1	1.13E+08	1.13E+08	569	1.13E+08	10	4.39571	3.25288
Nc-1-IP_pe	chr1	1.13E+08	1.13E+08	328	1.13E+08	10	4.77495	3.48064
Nc-1-IP_pe	chr1	1.13E+08	1.13E+08	289	1.13E+08	11	6.03439	4.10251
Nc-1-IP_pe	chr1	1.13E+08	1.13E+08	625	1.13E+08	9	4.46937	3.41876
Nc-1-IP_pe	chr1	1.13E+08	1.13E+08	256	1.13E+08	15	5.20142	3.14789
Nc-1-IP_pe	chr1	1.13E+08	1.13E+08	381	1.13E+08	21	7.58657	3.60451
Nc-1-IP_pe	chr1	1.13E+08	1.13E+08	444	1.13E+08	13	3.36696	2.43362
Nc-1-IP_pe	chr1	1.13E+08	1.13E+08	263	1.13E+08	14	4.21449	2.76599
Nc-1-IP_pe	chr1	1.13E+08	1.13E+08	379	1.13E+08	15	3.74763	2.48304
Nc-1-IP_pe	chr1	1.13E+08	1.13E+08	370	1.13E+08	14	4.8399	3.06836
Nc-1-IP_pe	chr1	1.13E+08	1.13E+08	671	1.13E+08	15	4.60262	2.86743
Nc-1-IP_pe	chr1	1.13E+08	1.13E+08	285	1.13E+08	23	8.38947	3.72437
Nc-1-IP_pe	chr1	1.13E+08	1.13E+08	258	1.13E+08	23	7.89844	3.53777
Nc-1-IP_pe	chr1	1.13E+08	1.13E+08	263	1.13E+08	20	7.43645	3.64378
Nc-1-IP_pe	chr1	1.13E+08	1.13E+08	538	1.13E+08	14	4.51299	2.90896
Nc-1-IP_pe	chr1	1.13E+08	1.13E+08	646	1.13E+08	31	16.4257	5.82506
Nc-1-IP_pe	chr1	1.13E+08	1.13E+08	536	1.13E+08	18	6.49169	3.44239
Nc-1-IP_pe	chr1	1.13E+08	1.13E+08	1031	1.13E+08	24	6.96967	3.1252
Nc-1-IP_pe	chr1	1.13E+08	1.13E+08	656	1.13E+08	23	10.9655	4.78257

Nc-1-IP_pe	chr1	1.13E+08	1.13E+08	476	1.13E+08	27	2.91871	1.79143
Nc-1-IP_pe	chr1	1.13E+08	1.13E+08	345	1.13E+08	18	4.04135	2.43445
Nc-1-IP_pe	chr1	1.13E+08	1.13E+08	413	1.13E+08	19	6.44601	3.32669
Nc-1-IP_pe	chr1	1.13E+08	1.13E+08	403	1.13E+08	14	3.94539	2.63917
Nc-1-IP_pe	chr1	1.13E+08	1.13E+08	605	1.13E+08	19	5.51632	2.94814
Nc-1-IP_pe	chr1	1.13E+08	1.13E+08	256	1.13E+08	23	7.4429	3.36882
Nc-1-IP_pe	chr1	1.13E+08	1.13E+08	259	1.13E+08	18	6.05776	3.25389
Nc-1-IP_pe	chr1	1.13E+08	1.13E+08	412	1.13E+08	16	4.96462	2.94973
Nc-1-IP_pe	chr1	1.13E+08	1.13E+08	357	1.13E+08	20	7.88558	3.83476
Nc-1-IP_pe	chr1	1.13E+08	1.13E+08	379	1.13E+08	31	13.4578	4.71703
Nc-1-IP_pe	chr1	1.13E+08	1.13E+08	257	1.13E+08	20	6.49719	3.259
Nc-1-IP_pe	chr1	1.13E+08	1.13E+08	279	1.13E+08	27	6.99773	2.94558
Nc-1-IP_pe	chr1	1.13E+08	1.13E+08	253	1.13E+08	16	4.69824	2.83176
Nc-1-IP_pe	chr1	1.13E+08	1.13E+08	618	1.13E+08	18	6.60326	3.49159
Nc-1-IP_pe	chr1	1.13E+08	1.13E+08	529	1.13E+08	15	4.38709	2.7688
Nc-1-IP_pe	chr1	1.13E+08	1.13E+08	678	1.13E+08	19	4.23334	2.45554
Nc-1-IP_pe	chr1	1.13E+08	1.13E+08	792	1.13E+08	25	8.80005	3.68849
Nc-1-IP_pe	chr1	1.13E+08	1.13E+08	436	1.13E+08	18	4.32723	2.54542
Nc-1-IP_pe	chr1	1.13E+08	1.13E+08	359	1.13E+08	24	7.19588	3.20323
Nc-1-IP_pe	chr1	1.13E+08	1.13E+08	510	1.13E+08	13	4.09691	2.78983
Nc-1-IP_pe	chr1	1.13E+08	1.13E+08	564	1.13E+08	16	3.65575	2.38624
Nc-1-IP_pe	chr1	1.13E+08	1.13E+08	548	1.13E+08	24	6.09885	2.83276
Nc-1-IP_pe	chr1	1.13E+08	1.13E+08	352	1.13E+08	22	4.80668	2.5094
Nc-1-IP_pe	chr1	1.13E+08	1.13E+08	527	1.13E+08	19	5.70288	3.02257
Nc-1-IP_pe	chr1	1.13E+08	1.13E+08	261	1.13E+08	16	4.96462	2.94973
Nc-1-IP_pe	chr1	1.13E+08	1.13E+08	1115	1.13E+08	24	9.61644	4.09603
Nc-1-IP_pe	chr1	1.13E+08	1.13E+08	304	1.13E+08	18	3.08421	2.07298
Nc-1-IP_pe	chr1	1.13E+08	1.13E+08	415	1.13E+08	15	5.67035	3.37431
Nc-1-IP_pe	chr1	1.13E+08	1.13E+08	733	1.13E+08	28	6.5317	2.75496
Nc-1-IP_pe	chr1	1.13E+08	1.13E+08	655	1.13E+08	24	9.61644	4.09603
Nc-1-IP_pe	chr1	1.13E+08	1.13E+08	915	1.13E+08	23	11.5921	5.06057
Nc-1-IP_pe	chr1	1.13E+08	1.13E+08	871	1.13E+08	23	7.21012	3.28399
Nc-1-IP_pe	chr1	1.13E+08	1.13E+08	345	1.13E+08	30	9.06325	3.38223
Nc-1-IP_pe	chr1	1.13E+08	1.13E+08	254	1.13E+08	14	5.48576	3.39215
Nc-1-IP_pe	chr1	1.13E+08	1.13E+08	972	1.13E+08	29	9.32978	3.53561
Nc-1-IP_pe	chr1	1.13E+08	1.13E+08	349	1.13E+08	28	9.16687	3.56053
Nc-1-IP_pe	chr1	1.14E+08	1.14E+08	470	1.14E+08	17	7.02067	3.79543
Nc-1-IP_pe	chr1	1.14E+08	1.14E+08	631	1.14E+08	23	4.99703	2.52478
Nc-1-IP_pe	chr1	1.14E+08	1.14E+08	352	1.14E+08	18	4.31276	2.53976
Nc-1-IP_pe	chr1	1.14E+08	1.14E+08	322	1.14E+08	24	9.05596	3.87955
Nc-1-IP_pe	chr1	1.14E+08	1.14E+08	340	1.14E+08	15	6.76257	3.92511
Nc-1-IP_pe	chr1	1.14E+08	1.14E+08	521	1.14E+08	20	6.83566	3.39542
Nc-1-IP_pe	chr1	1.14E+08	1.14E+08	1367	1.14E+08	26	8.38703	3.45949
Nc-1-IP_pe	chr1	1.14E+08	1.14E+08	279	1.14E+08	21	9.70208	4.50823
Nc-1-IP_pe	chr1	1.14E+08	1.14E+08	566	1.14E+08	23	6.99955	3.20812

Nc-1-IP_pe	chr1	1.14E+08	1.14E+08	586	1.14E+08	17	5.7822	3.22586
Nc-1-IP_pe	chr1	1.14E+08	1.14E+08	328	1.14E+08	22	10.4413	4.69651
Nc-1-IP_pe	chr1	1.14E+08	1.14E+08	373	1.14E+08	18	6.28754	3.35314
Nc-1-IP_pe	chr1	1.14E+08	1.14E+08	612	1.14E+08	28	4.92952	2.30747
Nc-1-IP_pe	chr1	1.14E+08	1.14E+08	269	1.14E+08	18	8.65961	4.45337
Nc-1-IP_pe	chr1	1.14E+08	1.14E+08	361	1.14E+08	14	3.86727	2.6027
Nc-1-IP_pe	chr1	1.14E+08	1.14E+08	623	1.14E+08	22	7.27519	3.39036
Nc-1-IP_pe	chr1	1.14E+08	1.14E+08	826	1.14E+08	34	13.132	4.29719
Nc-1-IP_pe	chr1	1.14E+08	1.14E+08	284	1.14E+08	16	6.08355	3.46522
Nc-1-IP_pe	chr1	1.14E+08	1.14E+08	273	1.14E+08	21	8.26251	3.88258
Nc-1-IP_pe	chr1	1.14E+08	1.14E+08	278	1.14E+08	16	5.82808	3.34463
Nc-1-IP_pe	chr1	1.14E+08	1.14E+08	653	1.14E+08	20	5.70098	2.94771
Nc-1-IP_pe	chr1	1.14E+08	1.14E+08	482	1.14E+08	18	6.05776	3.25389
Nc-1-IP_pe	chr1	1.14E+08	1.14E+08	361	1.14E+08	25	3.04809	1.86664
Nc-1-IP_pe	chr1	1.14E+08	1.14E+08	707	1.14E+08	24	9.04231	3.87435
Nc-1-IP_pe	chr1	1.14E+08	1.14E+08	256	1.14E+08	12	3.33349	2.48364
Nc-1-IP_pe	chr1	1.14E+08	1.14E+08	532	1.14E+08	30	6.5377	2.66578
Nc-1-IP_pe	chr1	1.14E+08	1.14E+08	377	1.14E+08	17	4.13464	2.52661
Nc-1-IP_pe	chr1	1.14E+08	1.14E+08	317	1.14E+08	22	6.07094	2.94697
Nc-1-IP_pe	chr1	1.14E+08	1.14E+08	340	1.14E+08	12	3.67829	2.65924
Nc-1-IP_pe	chr1	1.14E+08	1.14E+08	1671	1.14E+08	25	7.37317	3.1922
Nc-1-IP_pe	chr1	1.14E+08	1.14E+08	335	1.14E+08	27	9.65592	3.8088
Nc-1-IP_pe	chr1	1.14E+08	1.14E+08	318	1.14E+08	27	8.12936	3.29991
Nc-1-IP_pe	chr1	1.14E+08	1.14E+08	753	1.14E+08	18	6.31113	3.3634
Nc-1-IP_pe	chr1	1.14E+08	1.14E+08	863	1.14E+08	18	6.65364	3.51391
Nc-1-IP_pe	chr1	1.14E+08	1.14E+08	669	1.14E+08	17	4.44438	2.65333
Nc-1-IP_pe	chr1	1.14E+08	1.14E+08	298	1.14E+08	20	8.41487	4.06572
Nc-1-IP_pe	chr1	1.14E+08	1.14E+08	649	1.14E+08	22	10.0441	4.52101
Nc-1-IP_pe	chr1	1.14E+08	1.14E+08	278	1.14E+08	17	5.54967	3.12324
Nc-1-IP_pe	chr1	1.14E+08	1.14E+08	322	1.14E+08	20	4.42487	2.47493
Nc-1-IP_pe	chr1	1.14E+08	1.14E+08	832	1.14E+08	24	8.93412	3.83328
Nc-1-IP_pe	chr1	1.14E+08	1.14E+08	493	1.14E+08	25	6.62684	2.94607
Nc-1-IP_pe	chr1	1.14E+08	1.14E+08	290	1.14E+08	20	3.05104	1.99497
Nc-1-IP_pe	chr1	1.14E+08	1.14E+08	352	1.14E+08	20	5.01743	2.69071
Nc-1-IP_pe	chr1	1.14E+08	1.14E+08	592	1.14E+08	17	4.13464	2.52661
Nc-1-IP_pe	chr1	1.14E+08	1.14E+08	282	1.14E+08	14	3.96259	2.64722
Nc-1-IP_pe	chr1	1.14E+08	1.14E+08	296	1.14E+08	19	7.01419	3.56771
Nc-1-IP_pe	chr1	1.14E+08	1.14E+08	380	1.14E+08	21	5.2077	2.70109
Nc-1-IP_pe	chr1	1.14E+08	1.14E+08	766	1.14E+08	24	7.19588	3.20323
Nc-1-IP_pe	chr1	1.14E+08	1.14E+08	450	1.14E+08	18	2.88361	1.99878
Nc-1-IP_pe	chr1	1.14E+08	1.14E+08	651	1.14E+08	24	9.61644	4.09603
Nc-1-IP_pe	chr1	1.14E+08	1.14E+08	448	1.14E+08	20	4.71088	2.57831
Nc-1-IP_pe	chr1	1.14E+08	1.14E+08	595	1.14E+08	19	5.54795	2.96071
Nc-1-IP_pe	chr1	1.14E+08	1.14E+08	323	1.14E+08	21	5.5349	2.81884
Nc-1-IP_pe	chr1	1.14E+08	1.14E+08	699	1.14E+08	22	6.24237	3.00842

Nc-1-IP_pe	chr1	1.14E+08	1.14E+08	471	1.14E+08	18	4.50518	2.61529
Nc-1-IP_pe	chr1	1.14E+08	1.14E+08	629	1.14E+08	18	4.6358	2.66698
Nc-1-IP_pe	chr1	1.14E+08	1.14E+08	255	1.14E+08	19	5.14691	2.80294
Nc-1-IP_pe	chr1	1.14E+08	1.14E+08	345	1.14E+08	29	9.32978	3.53561
Nc-1-IP_pe	chr1	1.14E+08	1.14E+08	430	1.14E+08	24	5.47323	2.62998
Nc-1-IP_pe	chr1	1.14E+08	1.14E+08	495	1.14E+08	24	8.53898	3.68518
Nc-1-IP_pe	chr1	1.14E+08	1.14E+08	2019	1.14E+08	30	12.7121	4.56962
Nc-1-IP_pe	chr1	1.14E+08	1.14E+08	1074	1.14E+08	22	7.49924	3.47596
Nc-1-IP_pe	chr1	1.14E+08	1.14E+08	529	1.14E+08	25	10.6867	4.4006
Nc-1-IP_pe	chr1	1.14E+08	1.14E+08	279	1.14E+08	13	3.62212	2.55651
Nc-1-IP_pe	chr1	1.14E+08	1.14E+08	374	1.14E+08	18	5.72638	3.11298
Nc-1-IP_pe	chr1	1.14E+08	1.14E+08	259	1.14E+08	17	6.01283	3.32896
Nc-1-IP_pe	chr1	1.14E+08	1.14E+08	351	1.14E+08	15	4.21811	2.69231
Nc-1-IP_pe	chr1	1.14E+08	1.14E+08	1148	1.14E+08	20	7.97282	3.87239
Nc-1-IP_pe	chr1	1.14E+08	1.14E+08	273	1.14E+08	19	7.84735	3.93486
Nc-1-IP_pe	chr1	1.14E+08	1.14E+08	313	1.14E+08	16	5.62031	3.24783
Nc-1-IP_pe	chr1	1.14E+08	1.14E+08	299	1.14E+08	23	7.89844	3.53777
Nc-1-IP_pe	chr1	1.14E+08	1.14E+08	364	1.14E+08	19	7.2357	3.66372
Nc-1-IP_pe	chr1	1.14E+08	1.14E+08	268	1.14E+08	19	7.29128	3.68799
Nc-1-IP_pe	chr1	1.14E+08	1.14E+08	256	1.14E+08	19	7.79517	3.91138
Nc-1-IP_pe	chr1	1.14E+08	1.14E+08	817	1.14E+08	24	9.33472	3.98646
Nc-1-IP_pe	chr1	1.14E+08	1.14E+08	930	1.14E+08	21	7.34645	3.50809
Nc-1-IP_pe	chr1	1.14E+08	1.14E+08	1016	1.14E+08	22	6.84408	3.22845
Nc-1-IP_pe	chr1	1.14E+08	1.14E+08	855	1.14E+08	16	4.25252	2.63824
Nc-1-IP_pe	chr1	1.14E+08	1.14E+08	312	1.14E+08	15	2.95965	2.14351
Nc-1-IP_pe	chr1	1.14E+08	1.14E+08	454	1.14E+08	20	9.65348	4.63135
Nc-1-IP_pe	chr1	1.14E+08	1.14E+08	643	1.14E+08	24	9.77496	4.15836
Nc-1-IP_pe	chr1	1.14E+08	1.14E+08	276	1.14E+08	22	7.5586	3.49881
Nc-1-IP_pe	chr1	1.14E+08	1.14E+08	321	1.14E+08	38	9.37169	3.02136
Nc-1-IP_pe	chr1	1.14E+08	1.14E+08	534	1.14E+08	23	6.62483	3.0751
Nc-1-IP_pe	chr1	1.14E+08	1.14E+08	723	1.14E+08	26	8.82426	3.60914
Nc-1-IP_pe	chr1	1.14E+08	1.14E+08	260	1.14E+08	16	5.67858	3.27486
Nc-1-IP_pe	chr1	1.14E+08	1.14E+08	335	1.14E+08	21	9.50999	4.42209
Nc-1-IP_pe	chr1	1.14E+08	1.14E+08	274	1.14E+08	16	6.75928	3.79256
Nc-1-IP_pe	chr1	1.14E+08	1.14E+08	767	1.14E+08	19	8.9112	4.42769
Nc-1-IP_pe	chr1	1.14E+08	1.14E+08	507	1.14E+08	17	4.44438	2.65333
Nc-1-IP_pe	chr1	1.14E+08	1.14E+08	414	1.14E+08	24	9.05651	3.87976
Nc-1-IP_pe	chr1	1.14E+08	1.14E+08	259	1.14E+08	13	5.36853	3.44651
Nc-1-IP_pe	chr1	1.14E+08	1.14E+08	263	1.14E+08	22	6.84408	3.22845
Nc-1-IP_pe	chr1	1.14E+08	1.14E+08	257	1.14E+08	19	8.44891	4.21022
Nc-1-IP_pe	chr1	1.14E+08	1.14E+08	273	1.14E+08	15	4.40338	2.77622
Nc-1-IP_pe	chr1	1.14E+08	1.14E+08	510	1.14E+08	20	9.56043	4.58764
Nc-1-IP_pe	chr1	1.14E+08	1.14E+08	329	1.14E+08	21	4.90204	2.59278
Nc-1-IP_pe	chr1	1.14E+08	1.14E+08	447	1.14E+08	17	3.33947	2.20998
Nc-1-IP_pe	chr1	1.14E+08	1.14E+08	261	1.14E+08	14	3.86727	2.6027

Nc-1-IP_pe	chr1	1.14E+08	1.14E+08	281	1.14E+08	25	8.69165	3.64955
Nc-1-IP_pe	chr1	1.14E+08	1.14E+08	415	1.14E+08	23	9.50079	4.16433
Nc-1-IP_pe	chr1	1.14E+08	1.14E+08	258	1.14E+08	16	4.96462	2.94973
Nc-1-IP_pe	chr1	1.15E+08	1.15E+08	732	1.15E+08	34	6.70877	2.55423
Nc-1-IP_pe	chr1	1.15E+08	1.15E+08	529	1.15E+08	16	4.96462	2.94973
Nc-1-IP_pe	chr1	1.15E+08	1.15E+08	286	1.15E+08	19	4.8268	2.67939
Nc-1-IP_pe	chr1	1.15E+08	1.15E+08	499	1.15E+08	16	6.75928	3.79256
Nc-1-IP_pe	chr1	1.15E+08	1.15E+08	258	1.15E+08	19	8.53186	4.24887
Nc-1-IP_pe	chr1	1.15E+08	1.15E+08	337	1.15E+08	27	12.4941	4.85838
Nc-1-IP_pe	chr1	1.15E+08	1.15E+08	299	1.15E+08	16	5.00661	2.96849
Nc-1-IP_pe	chr1	1.15E+08	1.15E+08	1120	1.15E+08	26	5.56617	2.56496
Nc-1-IP_pe	chr1	1.15E+08	1.15E+08	269	1.15E+08	14	3.00733	2.21111
Nc-1-IP_pe	chr1	1.15E+08	1.15E+08	273	1.15E+08	17	7.08058	3.82398
Nc-1-IP_pe	chr1	1.15E+08	1.15E+08	378	1.15E+08	17	4.78101	2.79343
Nc-1-IP_pe	chr1	1.15E+08	1.15E+08	466	1.15E+08	16	5.03676	2.98199
Nc-1-IP_pe	chr1	1.15E+08	1.15E+08	485	1.15E+08	17	5.9911	3.31919
Nc-1-IP_pe	chr1	1.15E+08	1.15E+08	266	1.15E+08	19	7.82467	3.92465
Nc-1-IP_pe	chr1	1.15E+08	1.15E+08	324	1.15E+08	15	3.46204	2.35851
Nc-1-IP_pe	chr1	1.15E+08	1.15E+08	378	1.15E+08	24	12.4198	5.27143
Nc-1-IP_pe	chr1	1.15E+08	1.15E+08	274	1.15E+08	16	6.80394	3.81462
Nc-1-IP_pe	chr1	1.15E+08	1.15E+08	484	1.15E+08	14	5.56442	3.43239
Nc-1-IP_pe	chr1	1.15E+08	1.15E+08	1070	1.15E+08	22	9.39426	4.24119
Nc-1-IP_pe	chr1	1.15E+08	1.15E+08	506	1.15E+08	29	8.0888	3.15597
Nc-1-IP_pe	chr1	1.15E+08	1.15E+08	258	1.15E+08	18	6.63038	3.50359
Nc-1-IP_pe	chr1	1.15E+08	1.15E+08	303	1.15E+08	18	4.32723	2.54542
Nc-1-IP_pe	chr1	1.15E+08	1.15E+08	606	1.15E+08	19	3.48582	2.18208
Nc-1-IP_pe	chr1	1.15E+08	1.15E+08	333	1.15E+08	15	4.40338	2.77622
Nc-1-IP_pe	chr1	1.15E+08	1.15E+08	381	1.15E+08	21	10.3334	4.79704
Nc-1-IP_pe	chr1	1.15E+08	1.15E+08	289	1.15E+08	16	4.25252	2.63824
Nc-1-IP_pe	chr1	1.15E+08	1.15E+08	331	1.15E+08	20	6.49719	3.259
Nc-1-IP_pe	chr1	1.15E+08	1.15E+08	663	1.15E+08	28	3.90103	2.03237
Nc-1-IP_pe	chr1	1.15E+08	1.15E+08	1080	1.15E+08	22	5.72303	2.82387
Nc-1-IP_pe	chr1	1.15E+08	1.15E+08	514	1.15E+08	19	5.41648	2.90862
Nc-1-IP_pe	chr1	1.15E+08	1.15E+08	273	1.15E+08	16	4.96462	2.94973
Nc-1-IP_pe	chr1	1.15E+08	1.15E+08	478	1.15E+08	27	6.99773	2.94558
Nc-1-IP_pe	chr1	1.15E+08	1.15E+08	276	1.15E+08	14	6.49348	3.92083
Nc-1-IP_pe	chr1	1.15E+08	1.15E+08	758	1.15E+08	16	5.37454	3.13479
Nc-1-IP_pe	chr1	1.15E+08	1.15E+08	615	1.15E+08	22	9.42371	4.25368
Nc-1-IP_pe	chr1	1.15E+08	1.15E+08	632	1.15E+08	18	8.71476	4.4806
Nc-1-IP_pe	chr1	1.15E+08	1.15E+08	277	1.15E+08	23	8.39	3.72457
Nc-1-IP_pe	chr1	1.15E+08	1.15E+08	804	1.15E+08	20	3.45251	2.13284
Nc-1-IP_pe	chr1	1.15E+08	1.15E+08	456	1.15E+08	28	12.2171	4.6243
Nc-1-IP_pe	chr1	1.15E+08	1.15E+08	318	1.15E+08	22	7.27519	3.39036
Nc-1-IP_pe	chr1	1.15E+08	1.15E+08	751	1.15E+08	31	9.23535	3.36637
Nc-1-IP_pe	chr1	1.15E+08	1.15E+08	276	1.15E+08	18	5.83846	3.16035

Nc-1-IP_pe	chr1	1.15E+08	1.15E+08	303	1.15E+08	18	5.9909	3.22526
Nc-1-IP_pe	chr1	1.15E+08	1.15E+08	369	1.15E+08	31	7.73963	2.94477
Nc-1-IP_pe	chr1	1.15E+08	1.15E+08	691	1.15E+08	20	6.43151	3.23281
Nc-1-IP_pe	chr1	1.15E+08	1.15E+08	444	1.15E+08	18	4.6358	2.66698
Nc-1-IP_pe	chr1	1.15E+08	1.15E+08	545	1.15E+08	32	11.992	4.12526
Nc-1-IP_pe	chr1	1.15E+08	1.15E+08	597	1.15E+08	19	5.90435	3.10381
Nc-1-IP_pe	chr1	1.15E+08	1.15E+08	291	1.15E+08	19	6.69226	3.43023
Nc-1-IP_pe	chr1	1.15E+08	1.15E+08	1284	1.15E+08	36	10.5102	3.40489
Nc-1-IP_pe	chr1	1.15E+08	1.15E+08	316	1.15E+08	21	7.7868	3.68585
Nc-1-IP_pe	chr1	1.15E+08	1.15E+08	675	1.15E+08	23	6.41066	3.00019
Nc-1-IP_pe	chr1	1.15E+08	1.15E+08	349	1.15E+08	33	6.61114	2.56636
Nc-1-IP_pe	chr1	1.15E+08	1.15E+08	540	1.15E+08	18	4.6358	2.66698
Nc-1-IP_pe	chr1	1.15E+08	1.15E+08	433	1.15E+08	23	5.91101	2.82849
Nc-1-IP_pe	chr1	1.15E+08	1.15E+08	481	1.15E+08	21	3.85951	2.23441
Nc-1-IP_pe	chr1	1.15E+08	1.15E+08	601	1.15E+08	26	4.55998	2.27131
Nc-1-IP_pe	chr1	1.15E+08	1.15E+08	327	1.15E+08	29	5.35148	2.38704
Nc-1-IP_pe	chr1	1.15E+08	1.15E+08	451	1.15E+08	22	6.23294	3.00503
Nc-1-IP_pe	chr1	1.15E+08	1.15E+08	263	1.15E+08	26	5.56617	2.56496
Nc-1-IP_pe	chr1	1.15E+08	1.15E+08	621	1.15E+08	23	7.01947	3.21526
Nc-1-IP_pe	chr1	1.15E+08	1.15E+08	327	1.15E+08	18	4.87655	2.76319
Nc-1-IP_pe	chr1	1.15E+08	1.15E+08	325	1.15E+08	24	8.05897	3.50918
Nc-1-IP_pe	chr1	1.15E+08	1.15E+08	309	1.15E+08	24	11.6162	4.91857
Nc-1-IP_pe	chr1	1.15E+08	1.15E+08	688	1.15E+08	25	7.24165	3.14818
Nc-1-IP_pe	chr1	1.15E+08	1.15E+08	259	1.15E+08	22	7.61855	3.52195
Nc-1-IP_pe	chr1	1.15E+08	1.15E+08	254	1.15E+08	18	4.04135	2.43445
Nc-1-IP_pe	chr1	1.15E+08	1.15E+08	540	1.15E+08	22	5.09279	2.60614
Nc-1-IP_pe	chr1	1.15E+08	1.15E+08	1143	1.15E+08	24	4.91679	2.45429
Nc-1-IP_pe	chr1	1.15E+08	1.15E+08	961	1.15E+08	28	6.84886	2.84698
Nc-1-IP_pe	chr1	1.15E+08	1.15E+08	616	1.15E+08	21	7.73476	3.66463
Nc-1-IP_pe	chr1	1.15E+08	1.15E+08	287	1.15E+08	20	6.55175	3.28082
Nc-1-IP_pe	chr1	1.15E+08	1.15E+08	457	1.15E+08	16	3.1497	2.1782
Nc-1-IP_pe	chr1	1.15E+08	1.15E+08	860	1.15E+08	25	8.51729	3.58735
Nc-1-IP_pe	chr1	1.15E+08	1.15E+08	1495	1.15E+08	38	8.40646	2.79996
Nc-1-IP_pe	chr1	1.15E+08	1.15E+08	333	1.15E+08	18	4.32723	2.54542
Nc-1-IP_pe	chr1	1.15E+08	1.15E+08	304	1.15E+08	23	8.92223	3.93219
Nc-1-IP_pe	chr1	1.15E+08	1.15E+08	558	1.15E+08	23	6.89706	3.17149
Nc-1-IP_pe	chr1	1.15E+08	1.15E+08	1605	1.15E+08	31	12.6113	4.42575
Nc-1-IP_pe	chr1	1.15E+08	1.15E+08	626	1.15E+08	22	5.72303	2.82387
Nc-1-IP_pe	chr1	1.15E+08	1.15E+08	275	1.15E+08	20	6.08336	3.09555
Nc-1-IP_pe	chr1	1.15E+08	1.15E+08	273	1.15E+08	21	8.10596	3.8173
Nc-1-IP_pe	chr1	1.15E+08	1.15E+08	639	1.15E+08	26	7.6132	3.20225
Nc-1-IP_pe	chr1	1.15E+08	1.15E+08	257	1.15E+08	23	6.22745	2.93674
Nc-1-IP_pe	chr1	1.15E+08	1.15E+08	352	1.15E+08	22	8.24538	3.76835
Nc-1-IP_pe	chr1	1.15E+08	1.15E+08	314	1.15E+08	23	7.4429	3.36882
Nc-1-IP_pe	chr1	1.15E+08	1.15E+08	536	1.15E+08	29	7.71914	3.04692

Nc-1-IP_pe	chr1	1.15E+08	1.15E+08	269	1.15E+08	21	6.05567	3.01032
Nc-1-IP_pe	chr1	1.15E+08	1.15E+08	413	1.15E+08	24	4.91679	2.45429
Nc-1-IP_pe	chr1	1.15E+08	1.15E+08	307	1.15E+08	21	2.86684	1.90523
Nc-1-IP_pe	chr1	1.15E+08	1.15E+08	691	1.15E+08	24	4.66132	2.37496
Nc-1-IP_pe	chr1	1.15E+08	1.15E+08	294	1.15E+08	23	4.99703	2.52478
Nc-1-IP_pe	chr1	1.15E+08	1.15E+08	539	1.15E+08	20	5.16039	2.74372
Nc-1-IP_pe	chr1	1.15E+08	1.15E+08	441	1.15E+08	29	10.0641	3.7706
Nc-1-IP_pe	chr1	1.15E+08	1.15E+08	673	1.15E+08	26	4.79255	2.33823
Nc-1-IP_pe	chr1	1.15E+08	1.15E+08	540	1.15E+08	33	9.58225	3.33783
Nc-1-IP_pe	chr1	1.15E+08	1.15E+08	1130	1.15E+08	25	7.64976	3.2857
Nc-1-IP_pe	chr1	1.15E+08	1.15E+08	329	1.15E+08	19	5.70288	3.02257
Nc-1-IP_pe	chr1	1.15E+08	1.15E+08	318	1.15E+08	22	6.52351	3.11038
Nc-1-IP_pe	chr1	1.15E+08	1.15E+08	336	1.15E+08	31	8.08569	3.03995
Nc-1-IP_pe	chr1	1.15E+08	1.15E+08	483	1.15E+08	23	6.62483	3.0751
Nc-1-IP_pe	chr1	1.15E+08	1.15E+08	662	1.15E+08	38	14.7676	4.4191
Nc-1-IP_pe	chr1	1.15E+08	1.15E+08	406	1.15E+08	19	2.87318	1.96343
Nc-1-IP_pe	chr1	1.16E+08	1.16E+08	597	1.16E+08	21	5.88587	2.94733
Nc-1-IP_pe	chr1	1.16E+08	1.16E+08	616	1.16E+08	15	4.06504	2.62364
Nc-1-IP_pe	chr1	1.16E+08	1.16E+08	351	1.16E+08	21	5.5349	2.81884
Nc-1-IP_pe	chr1	1.16E+08	1.16E+08	366	1.16E+08	24	6.09885	2.83276
Nc-1-IP_pe	chr1	1.16E+08	1.16E+08	543	1.16E+08	23	8.39	3.72457
Nc-1-IP_pe	chr1	1.16E+08	1.16E+08	353	1.16E+08	26	8.38703	3.45949
Nc-1-IP_pe	chr1	1.16E+08	1.16E+08	1070	1.16E+08	34	6.79851	2.5757
Nc-1-IP_pe	chr1	1.16E+08	1.16E+08	422	1.16E+08	21	7.98609	3.76767
Nc-1-IP_pe	chr1	1.16E+08	1.16E+08	802	1.16E+08	21	7.07439	3.40032
Nc-1-IP_pe	chr1	1.16E+08	1.16E+08	1461	1.16E+08	34	8.2959	2.94429
Nc-1-IP_pe	chr1	1.16E+08	1.16E+08	278	1.16E+08	23	6.25615	2.94664
Nc-1-IP_pe	chr1	1.16E+08	1.16E+08	445	1.16E+08	20	5.50604	2.87349
Nc-1-IP_pe	chr1	1.16E+08	1.16E+08	562	1.16E+08	23	5.17168	2.58184
Nc-1-IP_pe	chr1	1.16E+08	1.16E+08	759	1.16E+08	31	11.5347	4.07061
Nc-1-IP_pe	chr1	1.16E+08	1.16E+08	813	1.16E+08	34	9.98822	3.38732
Nc-1-IP_pe	chr1	1.16E+08	1.16E+08	511	1.16E+08	29	9.07321	3.45536
Nc-1-IP_pe	chr1	1.16E+08	1.16E+08	448	1.16E+08	29	7.03604	2.84996
Nc-1-IP_pe	chr1	1.16E+08	1.16E+08	282	1.16E+08	26	8.38703	3.45949
Nc-1-IP_pe	chr1	1.16E+08	1.16E+08	602	1.16E+08	21	3.63688	2.15978
Nc-1-IP_pe	chr1	1.16E+08	1.16E+08	386	1.16E+08	31	10.5859	3.77132
Nc-1-IP_pe	chr1	1.16E+08	1.16E+08	365	1.16E+08	17	4.46354	2.66123
Nc-1-IP_pe	chr1	1.16E+08	1.16E+08	303	1.16E+08	29	5.35148	2.38704
Nc-1-IP_pe	chr1	1.16E+08	1.16E+08	372	1.16E+08	21	4.0961	2.31438
Nc-1-IP_pe	chr1	1.16E+08	1.16E+08	414	1.16E+08	23	8.6544	3.827
Nc-1-IP_pe	chr1	1.16E+08	1.16E+08	348	1.16E+08	27	5.75532	2.57668
Nc-1-IP_pe	chr1	1.16E+08	1.16E+08	275	1.16E+08	19	4.5193	2.56258
Nc-1-IP_pe	chr1	1.16E+08	1.16E+08	420	1.16E+08	22	7.87712	3.62262
Nc-1-IP_pe	chr1	1.16E+08	1.16E+08	400	1.16E+08	18	6.15736	3.29676
Nc-1-IP_pe	chr1	1.16E+08	1.16E+08	551	1.16E+08	24	8.92072	3.82821

Nc-1-IP_pe	chr1	1.16E+08	1.16E+08	679	1.16E+08	29	11.2119	4.15411
Nc-1-IP_pe	chr1	1.16E+08	1.16E+08	480	1.16E+08	15	4.06504	2.62364
Nc-1-IP_pe	chr1	1.16E+08	1.16E+08	717	1.16E+08	28	7.30368	2.98116
Nc-1-IP_pe	chr1	1.16E+08	1.16E+08	450	1.16E+08	20	4.71088	2.57831
Nc-1-IP_pe	chr1	1.16E+08	1.16E+08	397	1.16E+08	21	4.0961	2.31438
Nc-1-IP_pe	chr1	1.16E+08	1.16E+08	2005	1.16E+08	33	12.6816	4.25027
Nc-1-IP_pe	chr1	1.16E+08	1.16E+08	381	1.16E+08	35	13.2527	4.24274
Nc-1-IP_pe	chr1	1.16E+08	1.16E+08	709	1.16E+08	24	10.2766	4.35885
Nc-1-IP_pe	chr1	1.16E+08	1.16E+08	381	1.16E+08	17	5.15375	2.9516
Nc-1-IP_pe	chr1	1.16E+08	1.16E+08	271	1.16E+08	23	11.2928	4.92679
Nc-1-IP_pe	chr1	1.16E+08	1.16E+08	275	1.16E+08	21	8.6741	4.05679
Nc-1-IP_pe	chr1	1.16E+08	1.16E+08	650	1.16E+08	18	6.65364	3.51391
Nc-1-IP_pe	chr1	1.16E+08	1.16E+08	362	1.16E+08	28	7.18321	2.94535
Nc-1-IP_pe	chr1	1.16E+08	1.16E+08	253	1.16E+08	18	5.72638	3.11298
Nc-1-IP_pe	chr1	1.16E+08	1.16E+08	446	1.16E+08	23	8.9305	3.93546
Nc-1-IP_pe	chr1	1.16E+08	1.16E+08	571	1.16E+08	25	5.37675	2.55246
Nc-1-IP_pe	chr1	1.16E+08	1.16E+08	706	1.16E+08	24	9.88175	4.20063
Nc-1-IP_pe	chr1	1.16E+08	1.16E+08	620	1.16E+08	25	11.4573	4.71063
Nc-1-IP_pe	chr1	1.16E+08	1.16E+08	276	1.16E+08	23	8.39	3.72457
Nc-1-IP_pe	chr1	1.16E+08	1.16E+08	634	1.16E+08	26	6.81227	2.94581
Nc-1-IP_pe	chr1	1.16E+08	1.16E+08	339	1.16E+08	20	6.49719	3.259
Nc-1-IP_pe	chr1	1.16E+08	1.16E+08	260	1.16E+08	17	4.60611	2.72032
Nc-1-IP_pe	chr1	1.16E+08	1.16E+08	885	1.16E+08	22	3.40842	2.0523
Nc-1-IP_pe	chr1	1.16E+08	1.16E+08	263	1.16E+08	23	4.4731	2.35612
Nc-1-IP_pe	chr1	1.16E+08	1.16E+08	535	1.16E+08	19	7.45276	3.75892
Nc-1-IP_pe	chr1	1.16E+08	1.16E+08	328	1.16E+08	23	6.50461	3.03295
Nc-1-IP_pe	chr1	1.16E+08	1.16E+08	680	1.16E+08	26	7.55119	3.18205
Nc-1-IP_pe	chr1	1.16E+08	1.16E+08	612	1.16E+08	23	7.54072	3.40476
Nc-1-IP_pe	chr1	1.16E+08	1.16E+08	437	1.16E+08	22	6.44373	3.08129
Nc-1-IP_pe	chr1	1.16E+08	1.16E+08	278	1.16E+08	22	4.80668	2.5094
Nc-1-IP_pe	chr1	1.16E+08	1.16E+08	689	1.16E+08	23	4.23294	2.27997
Nc-1-IP_pe	chr1	1.16E+08	1.16E+08	294	1.16E+08	30	8.26892	3.14848
Nc-1-IP_pe	chr1	1.16E+08	1.16E+08	730	1.16E+08	27	6.66155	2.84379
Nc-1-IP_pe	chr1	1.16E+08	1.16E+08	739	1.16E+08	23	7.4429	3.36882
Nc-1-IP_pe	chr1	1.16E+08	1.16E+08	955	1.16E+08	35	12.7006	4.07917
Nc-1-IP_pe	chr1	1.16E+08	1.16E+08	332	1.16E+08	23	6.79637	3.13568
Nc-1-IP_pe	chr1	1.16E+08	1.16E+08	362	1.16E+08	28	9.51016	3.67284
Nc-1-IP_pe	chr1	1.16E+08	1.16E+08	516	1.16E+08	25	2.89298	1.82213
Nc-1-IP_pe	chr1	1.16E+08	1.16E+08	1281	1.16E+08	39	12.3113	3.68097
Nc-1-IP_pe	chr1	1.16E+08	1.16E+08	275	1.16E+08	16	3.01888	2.12513
Nc-1-IP_pe	chr1	1.16E+08	1.16E+08	395	1.16E+08	20	6.20941	3.14495
Nc-1-IP_pe	chr1	1.16E+08	1.16E+08	394	1.16E+08	17	4.05031	2.49246
Nc-1-IP_pe	chr1	1.16E+08	1.16E+08	387	1.16E+08	22	3.60904	2.11656
Nc-1-IP_pe	chr1	1.16E+08	1.16E+08	369	1.16E+08	19	3.26827	2.10398
Nc-1-IP_pe	chr1	1.16E+08	1.16E+08	256	1.16E+08	24	8.05897	3.50918

Nc-1-IP_pe	chr1	1.16E+08	1.16E+08	478	1.16E+08	28	9.16687	3.56053
Nc-1-IP_pe	chr1	1.16E+08	1.16E+08	281	1.16E+08	21	8.30508	3.90043
Nc-1-IP_pe	chr1	1.16E+08	1.16E+08	330	1.16E+08	14	3.26761	2.32785
Nc-1-IP_pe	chr1	1.16E+08	1.16E+08	549	1.16E+08	19	5.14691	2.80294
Nc-1-IP_pe	chr1	1.16E+08	1.16E+08	271	1.16E+08	23	4.1883	2.26588
Nc-1-IP_pe	chr1	1.16E+08	1.16E+08	324	1.16E+08	27	8.08969	3.28717
Nc-1-IP_pe	chr1	1.16E+08	1.16E+08	292	1.16E+08	28	8.36155	3.30401
Nc-1-IP_pe	chr1	1.16E+08	1.16E+08	923	1.16E+08	26	5.0372	2.40922
Nc-1-IP_pe	chr1	1.16E+08	1.16E+08	266	1.16E+08	26	7.55119	3.18205
Nc-1-IP_pe	chr1	1.16E+08	1.16E+08	572	1.16E+08	29	8.0888	3.15597
Nc-1-IP_pe	chr1	1.16E+08	1.16E+08	771	1.16E+08	30	9.06325	3.38223
Nc-1-IP_pe	chr1	1.16E+08	1.16E+08	255	1.16E+08	20	6.49719	3.259
Nc-1-IP_pe	chr1	1.16E+08	1.16E+08	529	1.16E+08	26	12.3122	4.92264
Nc-1-IP_pe	chr1	1.16E+08	1.16E+08	254	1.16E+08	16	3.1497	2.1782
Nc-1-IP_pe	chr1	1.16E+08	1.16E+08	1704	1.16E+08	39	13.6948	4.04016
Nc-1-IP_pe	chr1	1.16E+08	1.16E+08	270	1.16E+08	22	6.84408	3.22845
Nc-1-IP_pe	chr1	1.16E+08	1.16E+08	253	1.16E+08	21	7.4442	3.54719
Nc-1-IP_pe	chr1	1.16E+08	1.16E+08	315	1.16E+08	17	3.33947	2.20998
Nc-1-IP_pe	chr1	1.16E+08	1.16E+08	278	1.16E+08	20	4.42487	2.47493
Nc-1-IP_pe	chr1	1.16E+08	1.16E+08	783	1.16E+08	22	7.17746	3.35334
Nc-1-IP_pe	chr1	1.16E+08	1.16E+08	306	1.16E+08	19	3.96692	2.35708
Nc-1-IP_pe	chr1	1.16E+08	1.16E+08	390	1.16E+08	18	3.08421	2.07298
Nc-1-IP_pe	chr1	1.16E+08	1.16E+08	418	1.16E+08	33	8.81131	3.12882
Nc-1-IP_pe	chr1	1.16E+08	1.16E+08	445	1.16E+08	23	4.23294	2.27997
Nc-1-IP_pe	chr1	1.16E+08	1.16E+08	297	1.16E+08	29	8.73808	3.35194
Nc-1-IP_pe	chr1	1.16E+08	1.16E+08	397	1.16E+08	20	6.08336	3.09555
Nc-1-IP_pe	chr1	1.16E+08	1.16E+08	502	1.16E+08	25	5.66296	2.64066
Nc-1-IP_pe	chr1	1.16E+08	1.16E+08	256	1.16E+08	19	4.98424	2.7399
Nc-1-IP_pe	chr1	1.16E+08	1.16E+08	298	1.16E+08	22	7.08871	3.31988
Nc-1-IP_pe	chr1	1.16E+08	1.16E+08	430	1.16E+08	19	4.42591	2.52746
Nc-1-IP_pe	chr1	1.16E+08	1.16E+08	695	1.16E+08	19	5.04347	2.76279
Nc-1-IP_pe	chr1	1.16E+08	1.16E+08	907	1.16E+08	32	9.40838	3.35161
Nc-1-IP_pe	chr1	1.16E+08	1.16E+08	498	1.16E+08	28	5.16525	2.37168
Nc-1-IP_pe	chr1	1.16E+08	1.16E+08	589	1.16E+08	29	5.99646	2.56076
Nc-1-IP_pe	chr1	1.16E+08	1.16E+08	335	1.16E+08	18	3.77591	2.33276
Nc-1-IP_pe	chr1	1.16E+08	1.16E+08	253	1.16E+08	26	5.85241	2.65063
Nc-1-IP_pe	chr1	1.17E+08	1.17E+08	523	1.17E+08	24	5.14265	2.5251
Nc-1-IP_pe	chr1	1.17E+08	1.17E+08	935	1.17E+08	26	6.10504	2.72711
Nc-1-IP_pe	chr1	1.17E+08	1.17E+08	311	1.17E+08	29	6.71992	2.76073
Nc-1-IP_pe	chr1	1.17E+08	1.17E+08	348	1.17E+08	33	8.81131	3.12882
Nc-1-IP_pe	chr1	1.17E+08	1.17E+08	1110	1.17E+08	26	6.15464	2.74223
Nc-1-IP_pe	chr1	1.17E+08	1.17E+08	697	1.17E+08	32	15.5328	5.33565
Nc-1-IP_pe	chr1	1.17E+08	1.17E+08	307	1.17E+08	24	4.66132	2.37496
Nc-1-IP_pe	chr1	1.17E+08	1.17E+08	719	1.17E+08	18	5.52764	3.02971
Nc-1-IP_pe	chr1	1.17E+08	1.17E+08	299	1.17E+08	19	3.26827	2.10398

Nc-1-IP_pe	chr1	1.17E+08	1.17E+08	450	1.17E+08	31	12.9461	4.53969
Nc-1-IP_pe	chr1	1.17E+08	1.17E+08	1060	1.17E+08	29	4.46367	2.15381
Nc-1-IP_pe	chr1	1.17E+08	1.17E+08	530	1.17E+08	38	8.71565	2.87006
Nc-1-IP_pe	chr1	1.17E+08	1.17E+08	272	1.17E+08	23	6.62483	3.0751
Nc-1-IP_pe	chr1	1.17E+08	1.17E+08	263	1.17E+08	18	4.6358	2.66698
Nc-1-IP_pe	chr1	1.17E+08	1.17E+08	265	1.17E+08	21	5.88587	2.94733
Nc-1-IP_pe	chr1	1.17E+08	1.17E+08	411	1.17E+08	28	7.18321	2.94535
Nc-1-IP_pe	chr1	1.17E+08	1.17E+08	378	1.17E+08	18	5.3798	2.96836
Nc-1-IP_pe	chr1	1.17E+08	1.17E+08	540	1.17E+08	20	6.36707	3.20721
Nc-1-IP_pe	chr1	1.17E+08	1.17E+08	335	1.17E+08	23	6.41066	3.00019
Nc-1-IP_pe	chr1	1.17E+08	1.17E+08	776	1.17E+08	33	9.58225	3.33783
Nc-1-IP_pe	chr1	1.17E+08	1.17E+08	552	1.17E+08	27	6.04159	2.65996
Nc-1-IP_pe	chr1	1.17E+08	1.17E+08	346	1.17E+08	22	6.4249	3.07445
Nc-1-IP_pe	chr1	1.17E+08	1.17E+08	333	1.17E+08	25	7.34119	3.18147
Nc-1-IP_pe	chr1	1.17E+08	1.17E+08	461	1.17E+08	22	7.27519	3.39036
Nc-1-IP_pe	chr1	1.17E+08	1.17E+08	579	1.17E+08	24	6.87207	3.0918
Nc-1-IP_pe	chr1	1.17E+08	1.17E+08	840	1.17E+08	22	5.78164	2.84446
Nc-1-IP_pe	chr1	1.17E+08	1.17E+08	436	1.17E+08	35	16.569	5.30666
Nc-1-IP_pe	chr1	1.17E+08	1.17E+08	586	1.17E+08	30	12.0847	4.35139
Nc-1-IP_pe	chr1	1.17E+08	1.17E+08	272	1.17E+08	20	5.70098	2.94771
Nc-1-IP_pe	chr1	1.17E+08	1.17E+08	456	1.17E+08	23	4.99703	2.52478
Nc-1-IP_pe	chr1	1.17E+08	1.17E+08	272	1.17E+08	29	9.32978	3.53561
Nc-1-IP_pe	chr1	1.17E+08	1.17E+08	689	1.17E+08	33	8.45274	3.03383
Nc-1-IP_pe	chr1	1.17E+08	1.17E+08	279	1.17E+08	26	8.59896	3.53163
Nc-1-IP_pe	chr1	1.17E+08	1.17E+08	877	1.17E+08	26	8.26874	3.41954
Nc-1-IP_pe	chr1	1.17E+08	1.17E+08	417	1.17E+08	23	5.28318	2.6185
Nc-1-IP_pe	chr1	1.17E+08	1.17E+08	477	1.17E+08	28	9.10684	3.54107
Nc-1-IP_pe	chr1	1.17E+08	1.17E+08	458	1.17E+08	26	5.56617	2.56496
Nc-1-IP_pe	chr1	1.17E+08	1.17E+08	522	1.17E+08	25	4.26554	2.21932
Nc-1-IP_pe	chr1	1.17E+08	1.17E+08	351	1.17E+08	26	5.0372	2.40922
Nc-1-IP_pe	chr1	1.17E+08	1.17E+08	777	1.17E+08	34	9.38324	3.22547
Nc-1-IP_pe	chr1	1.17E+08	1.17E+08	465	1.17E+08	26	8.38703	3.45949
Nc-1-IP_pe	chr1	1.17E+08	1.17E+08	285	1.17E+08	26	7.17069	3.05938
Nc-1-IP_pe	chr1	1.17E+08	1.17E+08	404	1.17E+08	19	3.6913	2.25641
Nc-1-IP_pe	chr1	1.17E+08	1.17E+08	1002	1.17E+08	22	3.82136	2.18497
Nc-1-IP_pe	chr1	1.17E+08	1.17E+08	604	1.17E+08	21	4.58491	2.48205
Nc-1-IP_pe	chr1	1.17E+08	1.17E+08	292	1.17E+08	25	8.26908	3.49972
Nc-1-IP_pe	chr1	1.17E+08	1.17E+08	307	1.17E+08	18	4.04135	2.43445
Nc-1-IP_pe	chr1	1.17E+08	1.17E+08	270	1.17E+08	24	6.44146	2.94634
Nc-1-IP_pe	chr1	1.17E+08	1.17E+08	274	1.17E+08	17	5.9911	3.31919
Nc-1-IP_pe	chr1	1.17E+08	1.17E+08	320	1.17E+08	26	6.47412	2.84038
Nc-1-IP_pe	chr1	1.17E+08	1.17E+08	1330	1.17E+08	24	6.77607	3.05911
Nc-1-IP_pe	chr1	1.17E+08	1.17E+08	487	1.17E+08	25	7.78348	3.33136
Nc-1-IP_pe	chr1	1.17E+08	1.17E+08	552	1.17E+08	21	6.51474	3.18348
Nc-1-IP_pe	chr1	1.17E+08	1.17E+08	975	1.17E+08	31	12.0201	4.22865

Nc-1-IP_pe	chr1	1.17E+08	1.17E+08	1060	1.17E+08	24	7.16986	3.19421
Nc-1-IP_pe	chr1	1.17E+08	1.17E+08	259	1.17E+08	24	7.59141	3.34179
Nc-1-IP_pe	chr1	1.17E+08	1.17E+08	354	1.17E+08	17	3.33947	2.20998
Nc-1-IP_pe	chr1	1.17E+08	1.17E+08	315	1.17E+08	19	3.96692	2.35708
Nc-1-IP_pe	chr1	1.17E+08	1.17E+08	310	1.17E+08	21	7.43189	3.54225
Nc-1-IP_pe	chr1	1.17E+08	1.17E+08	559	1.17E+08	18	4.1654	2.48241
Nc-1-IP_pe	chr1	1.17E+08	1.17E+08	286	1.17E+08	18	5.46792	3.00487
Nc-1-IP_pe	chr1	1.17E+08	1.17E+08	447	1.17E+08	18	4.96968	2.80074
Nc-1-IP_pe	chr1	1.17E+08	1.17E+08	306	1.17E+08	21	7.10961	3.41419
Nc-1-IP_pe	chr1	1.17E+08	1.17E+08	611	1.17E+08	29	8.89222	3.39931
Nc-1-IP_pe	chr1	1.17E+08	1.17E+08	307	1.17E+08	22	6.62394	3.14716
Nc-1-IP_pe	chr1	1.17E+08	1.17E+08	321	1.17E+08	21	8.37669	3.93054
Nc-1-IP_pe	chr1	1.17E+08	1.17E+08	286	1.17E+08	22	7.27519	3.39036
Nc-1-IP_pe	chr1	1.17E+08	1.17E+08	1009	1.17E+08	24	5.58146	2.66465
Nc-1-IP_pe	chr1	1.17E+08	1.17E+08	443	1.17E+08	25	5.1059	2.46996
Nc-1-IP_pe	chr1	1.17E+08	1.17E+08	537	1.17E+08	19	4.23334	2.45554
Nc-1-IP_pe	chr1	1.17E+08	1.17E+08	573	1.17E+08	21	5.77693	2.9072
Nc-1-IP_pe	chr1	1.17E+08	1.17E+08	268	1.17E+08	20	7.43645	3.64378
Nc-1-IP_pe	chr1	1.17E+08	1.17E+08	335	1.17E+08	23	6.62483	3.0751
Nc-1-IP_pe	chr1	1.17E+08	1.17E+08	285	1.17E+08	26	6.47412	2.84038
Nc-1-IP_pe	chr1	1.17E+08	1.17E+08	292	1.17E+08	21	2.86684	1.90523
Nc-1-IP_pe	chr1	1.17E+08	1.17E+08	604	1.17E+08	23	5.91101	2.82849
Nc-1-IP_pe	chr1	1.17E+08	1.17E+08	267	1.17E+08	25	6.98838	3.0642
Nc-1-IP_pe	chr1	1.17E+08	1.17E+08	310	1.17E+08	19	2.87318	1.96343
Nc-1-IP_pe	chr1	1.17E+08	1.17E+08	637	1.17E+08	16	5.10799	3.01397
Nc-1-IP_pe	chr1	1.17E+08	1.17E+08	334	1.17E+08	22	5.39765	2.71064
Nc-1-IP_pe	chr1	1.17E+08	1.17E+08	914	1.17E+08	32	10.9313	3.79712
Nc-1-IP_pe	chr1	1.17E+08	1.17E+08	374	1.17E+08	20	5.16039	2.74372
Nc-1-IP_pe	chr1	1.17E+08	1.17E+08	279	1.17E+08	20	5.70098	2.94771
Nc-1-IP_pe	chr1	1.17E+08	1.17E+08	287	1.17E+08	14	4.10345	2.71343
Nc-1-IP_pe	chr1	1.17E+08	1.17E+08	596	1.17E+08	25	9.19615	3.83259
Nc-1-IP_pe	chr1	1.17E+08	1.17E+08	438	1.17E+08	27	7.72987	3.17269
Nc-1-IP_pe	chr1	1.17E+08	1.17E+08	271	1.17E+08	21	6.66991	3.24296
Nc-1-IP_pe	chr1	1.17E+08	1.17E+08	273	1.17E+08	21	7.58657	3.60451
Nc-1-IP_pe	chr1	1.17E+08	1.17E+08	559	1.17E+08	22	6.84408	3.22845
Nc-1-IP_pe	chr1	1.17E+08	1.17E+08	280	1.17E+08	26	10.0575	4.04875
Nc-1-IP_pe	chr1	1.17E+08	1.17E+08	355	1.17E+08	16	4.3356	2.67395
Nc-1-IP_pe	chr1	1.17E+08	1.17E+08	310	1.17E+08	22	7.7408	3.56938
Nc-1-IP_pe	chr1	1.17E+08	1.17E+08	435	1.17E+08	24	5.50559	2.64033
Nc-1-IP_pe	chr1	1.17E+08	1.17E+08	384	1.17E+08	22	5.39765	2.71064
Nc-1-IP_pe	chr1	1.17E+08	1.17E+08	322	1.17E+08	23	5.91101	2.82849
Nc-1-IP_pe	chr1	1.17E+08	1.17E+08	530	1.17E+08	20	6.35641	3.20298
Nc-1-IP_pe	chr1	1.17E+08	1.17E+08	581	1.17E+08	21	7.4442	3.54719
Nc-1-IP_pe	chr1	1.17E+08	1.17E+08	765	1.17E+08	19	4.23334	2.45554
Nc-1-IP_pe	chr1	1.17E+08	1.17E+08	485	1.17E+08	17	3.58449	2.30632

Nc-1-IP_pe chr1	1.17E+08	1.17E+08	390	1.17E+08	34	10.5986	3.55474
Nc-1-IP_pe chr1	1.17E+08	1.17E+08	316	1.17E+08	28	8.72239	3.41776
Nc-1-IP_pe chr1	1.17E+08	1.17E+08	364	1.17E+08	20	5.34663	2.81336
Nc-1-IP_pe chr1	1.17E+08	1.17E+08	316	1.17E+08	23	9.94766	4.34828
Nc-1-IP_pe chr1	1.17E+08	1.17E+08	331	1.17E+08	19	5.15822	2.80735
Nc-1-IP_pe chr1	1.17E+08	1.17E+08	302	1.17E+08	22	8.40205	3.8312
Nc-1-IP_pe chr1	1.17E+08	1.17E+08	304	1.17E+08	23	7.89844	3.53777
Nc-1-IP_pe chr1	1.17E+08	1.17E+08	256	1.17E+08	20	5.34663	2.81336
Nc-1-IP_pe chr1	1.17E+08	1.17E+08	253	1.17E+08	22	6.84408	3.22845
Nc-1-IP_pe chr1	1.17E+08	1.17E+08	743	1.17E+08	25	6.98838	3.0642
Nc-1-IP_pe chr1	1.17E+08	1.17E+08	694	1.17E+08	33	10.834	3.69193
Nc-1-IP_pe chr1	1.17E+08	1.17E+08	378	1.17E+08	25	9.7397	4.03495
Nc-1-IP_pe chr1	1.17E+08	1.17E+08	420	1.17E+08	15	5.58327	3.33181
Nc-1-IP_pe chr1	1.17E+08	1.17E+08	616	1.17E+08	22	7.04484	3.3034
Nc-1-IP_pe chr1	1.17E+08	1.17E+08	443	1.17E+08	22	4.80668	2.5094
Nc-1-IP_pe chr1	1.17E+08	1.17E+08	315	1.17E+08	21	6.66275	3.2402
Nc-1-IP_pe chr1	1.17E+08	1.17E+08	535	1.17E+08	21	7.10961	3.41419
Nc-1-IP_pe chr1	1.17E+08	1.17E+08	377	1.17E+08	23	5.87882	2.81757
Nc-1-IP_pe chr1	1.17E+08	1.17E+08	750	1.17E+08	28	10.1443	3.88511
Nc-1-IP_pe chr1	1.17E+08	1.17E+08	495	1.17E+08	19	6.11371	3.18917
Nc-1-IP_pe chr1	1.17E+08	1.17E+08	286	1.17E+08	23	6.62483	3.0751
Nc-1-IP_pe chr1	1.17E+08	1.17E+08	385	1.17E+08	21	3.63688	2.15978
Nc-1-IP_pe chr1	1.17E+08	1.17E+08	527	1.17E+08	34	11.0523	3.68197
Nc-1-IP_pe chr1	1.17E+08	1.17E+08	425	1.17E+08	20	5.18544	2.75305
Nc-1-IP_pe chr1	1.17E+08	1.17E+08	758	1.17E+08	21	5.77693	2.9072
Nc-1-IP_pe chr1	1.17E+08	1.17E+08	447	1.17E+08	24	7.61242	3.34923
Nc-1-IP_pe chr1	1.17E+08	1.17E+08	361	1.17E+08	18	4.09381	2.4547
Nc-1-IP_pe chr1	1.17E+08	1.17E+08	290	1.17E+08	27	8.55392	3.43775
Nc-1-IP_pe chr1	1.17E+08	1.17E+08	546	1.17E+08	21	4.0961	2.31438
Nc-1-IP_pe chr1	1.17E+08	1.17E+08	378	1.17E+08	21	6.35047	3.12103
Nc-1-IP_pe chr1	1.17E+08	1.17E+08	413	1.17E+08	21	5.5349	2.81884
Nc-1-IP_pe chr1	1.17E+08	1.17E+08	342	1.17E+08	22	6.84408	3.22845
Nc-1-IP_pe chr1	1.17E+08	1.17E+08	357	1.17E+08	17	5.58414	3.13837
Nc-1-IP_pe chr1	1.17E+08	1.17E+08	257	1.17E+08	17	5.46727	3.08721
Nc-1-IP_pe chr1	1.17E+08	1.17E+08	420	1.17E+08	16	3.1497	2.1782
Nc-1-IP_pe chr1	1.17E+08	1.17E+08	425	1.17E+08	23	5.5873	2.71945
Nc-1-IP_pe chr1	1.17E+08	1.17E+08	424	1.17E+08	14	3.26761	2.32785
Nc-1-IP_pe chr1	1.17E+08	1.17E+08	322	1.17E+08	21	5.88587	2.94733
Nc-1-IP_pe chr1	1.17E+08	1.17E+08	275	1.17E+08	16	5.48356	3.18474
Nc-1-IP_pe chr1	1.17E+08	1.17E+08	786	1.17E+08	25	7.78348	3.33136
Nc-1-IP_pe chr1	1.17E+08	1.17E+08	274	1.17E+08	16	2.92499	2.08721
Nc-1-IP_pe chr1	1.17E+08	1.17E+08	1336	1.17E+08	33	6.88554	2.63401
Nc-1-IP_pe chr1	1.17E+08	1.17E+08	299	1.17E+08	16	3.39261	2.27748
Nc-1-IP_pe chr1	1.17E+08	1.17E+08	2132	1.17E+08	30	14.718	5.30899
Nc-1-IP_pe chr1	1.17E+08	1.17E+08	274	1.17E+08	18	6.15736	3.29676

Nc-1-IP_pe	chr1	1.17E+08	1.17E+08	536	1.17E+08	20	6.49675	3.25882
Nc-1-IP_pe	chr1	1.17E+08	1.17E+08	261	1.17E+08	21	8.6741	4.05679
Nc-1-IP_pe	chr1	1.18E+08	1.18E+08	707	1.18E+08	21	6.04612	3.00677
Nc-1-IP_pe	chr1	1.18E+08	1.18E+08	280	1.18E+08	22	7.15514	3.34491
Nc-1-IP_pe	chr1	1.18E+08	1.18E+08	337	1.18E+08	18	5.33158	2.94846
Nc-1-IP_pe	chr1	1.18E+08	1.18E+08	297	1.18E+08	20	6.94658	3.44067
Nc-1-IP_pe	chr1	1.18E+08	1.18E+08	769	1.18E+08	27	9.46411	3.74281
Nc-1-IP_pe	chr1	1.18E+08	1.18E+08	581	1.18E+08	27	8.12936	3.29991
Nc-1-IP_pe	chr1	1.18E+08	1.18E+08	267	1.18E+08	20	4.71088	2.57831
Nc-1-IP_pe	chr1	1.18E+08	1.18E+08	684	1.18E+08	22	5.09279	2.60614
Nc-1-IP_pe	chr1	1.18E+08	1.18E+08	964	1.18E+08	28	9.36531	3.62523
Nc-1-IP_pe	chr1	1.18E+08	1.18E+08	499	1.18E+08	26	9.37548	3.80239
Nc-1-IP_pe	chr1	1.18E+08	1.18E+08	550	1.18E+08	25	6.24427	2.82324
Nc-1-IP_pe	chr1	1.18E+08	1.18E+08	377	1.18E+08	26	8.24655	3.41207
Nc-1-IP_pe	chr1	1.18E+08	1.18E+08	437	1.18E+08	23	5.39637	2.65591
Nc-1-IP_pe	chr1	1.18E+08	1.18E+08	694	1.18E+08	23	5.79517	2.78927
Nc-1-IP_pe	chr1	1.18E+08	1.18E+08	432	1.18E+08	22	2.86933	1.88099
Nc-1-IP_pe	chr1	1.18E+08	1.18E+08	543	1.18E+08	23	5.5873	2.71945
Nc-1-IP_pe	chr1	1.18E+08	1.18E+08	1965	1.18E+08	23	5.91101	2.82849
Nc-1-IP_pe	chr1	1.18E+08	1.18E+08	483	1.18E+08	27	6.66155	2.84379
Nc-1-IP_pe	chr1	1.18E+08	1.18E+08	717	1.18E+08	20	4.42487	2.47493
Nc-1-IP_pe	chr1	1.18E+08	1.18E+08	738	1.18E+08	27	10.4903	4.103
Nc-1-IP_pe	chr1	1.18E+08	1.18E+08	384	1.18E+08	21	4.61597	2.49283
Nc-1-IP_pe	chr1	1.18E+08	1.18E+08	1128	1.18E+08	36	9.92208	3.25429
Nc-1-IP_pe	chr1	1.18E+08	1.18E+08	748	1.18E+08	27	6.04159	2.65996
Nc-1-IP_pe	chr1	1.18E+08	1.18E+08	302	1.18E+08	20	4.71088	2.57831
Nc-1-IP_pe	chr1	1.18E+08	1.18E+08	333	1.18E+08	35	11.2118	3.6563
Nc-1-IP_pe	chr1	1.18E+08	1.18E+08	734	1.18E+08	22	5.44743	2.72785
Nc-1-IP_pe	chr1	1.18E+08	1.18E+08	258	1.18E+08	25	4.84935	2.39263
Nc-1-IP_pe	chr1	1.18E+08	1.18E+08	383	1.18E+08	29	7.36869	2.94515
Nc-1-IP_pe	chr1	1.18E+08	1.18E+08	306	1.18E+08	25	7.37137	3.1916
Nc-1-IP_pe	chr1	1.18E+08	1.18E+08	479	1.18E+08	20	3.24562	2.0616
Nc-1-IP_pe	chr1	1.18E+08	1.18E+08	854	1.18E+08	25	7.78348	3.33136
Nc-1-IP_pe	chr1	1.18E+08	1.18E+08	646	1.18E+08	23	5.5873	2.71945
Nc-1-IP_pe	chr1	1.18E+08	1.18E+08	353	1.18E+08	20	4.3291	2.44062
Nc-1-IP_pe	chr1	1.18E+08	1.18E+08	267	1.18E+08	22	5.09279	2.60614
Nc-1-IP_pe	chr1	1.18E+08	1.18E+08	348	1.18E+08	20	6.08336	3.09555
Nc-1-IP_pe	chr1	1.18E+08	1.18E+08	254	1.18E+08	20	6.08336	3.09555
Nc-1-IP_pe	chr1	1.18E+08	1.18E+08	479	1.18E+08	16	5.1389	3.02788
Nc-1-IP_pe	chr1	1.18E+08	1.18E+08	436	1.18E+08	19	3.48582	2.18208
Nc-1-IP_pe	chr1	1.18E+08	1.18E+08	457	1.18E+08	24	5.77667	2.7276
Nc-1-IP_pe	chr1	1.18E+08	1.18E+08	585	1.18E+08	32	9.0095	3.23966
Nc-1-IP_pe	chr1	1.18E+08	1.18E+08	563	1.18E+08	19	4.8268	2.67939
Nc-1-IP_pe	chr1	1.18E+08	1.18E+08	681	1.18E+08	23	7.3183	3.32328
Nc-1-IP_pe	chr1	1.18E+08	1.18E+08	465	1.18E+08	16	3.39261	2.27748

Nc-1-IP_pe	chr1	1.18E+08	1.18E+08	378	1.18E+08	21	5.26279	2.72078
Nc-1-IP_pe	chr1	1.18E+08	1.18E+08	414	1.18E+08	22	5.39765	2.71064
Nc-1-IP_pe	chr1	1.18E+08	1.18E+08	690	1.18E+08	26	7.17069	3.05938
Nc-1-IP_pe	chr1	1.18E+08	1.18E+08	430	1.18E+08	25	6.98838	3.0642
Nc-1-IP_pe	chr1	1.18E+08	1.18E+08	303	1.18E+08	18	7.47506	3.88659
Nc-1-IP_pe	chr1	1.18E+08	1.18E+08	473	1.18E+08	17	4.78065	2.79327
Nc-1-IP_pe	chr1	1.18E+08	1.18E+08	670	1.18E+08	23	4.4731	2.35612
Nc-1-IP_pe	chr1	1.18E+08	1.18E+08	814	1.18E+08	26	8.80019	3.60082
Nc-1-IP_pe	chr1	1.18E+08	1.18E+08	264	1.18E+08	19	4.50014	2.55537
Nc-1-IP_pe	chr1	1.18E+08	1.18E+08	319	1.18E+08	34	13.132	4.29719
Nc-1-IP_pe	chr1	1.18E+08	1.18E+08	610	1.18E+08	24	7.06892	3.15933
Nc-1-IP_pe	chr1	1.18E+08	1.18E+08	420	1.18E+08	27	7.72987	3.17269
Nc-1-IP_pe	chr1	1.18E+08	1.18E+08	284	1.18E+08	20	6.08336	3.09555
Nc-1-IP_pe	chr1	1.18E+08	1.18E+08	328	1.18E+08	19	4.5193	2.56258
Nc-1-IP_pe	chr1	1.18E+08	1.18E+08	310	1.18E+08	14	5.44699	3.37238
Nc-1-IP_pe	chr1	1.18E+08	1.18E+08	1172	1.18E+08	45	12.0854	3.30251
Nc-1-IP_pe	chr1	1.18E+08	1.18E+08	254	1.18E+08	20	5.70098	2.94771
Nc-1-IP_pe	chr1	1.18E+08	1.18E+08	306	1.18E+08	16	2.92499	2.08721
Nc-1-IP_pe	chr1	1.18E+08	1.18E+08	329	1.18E+08	20	6.49719	3.259
Nc-1-IP_pe	chr1	1.18E+08	1.18E+08	367	1.18E+08	17	3.33947	2.20998
Nc-1-IP_pe	chr1	1.18E+08	1.18E+08	451	1.18E+08	26	7.92224	3.30384
Nc-1-IP_pe	chr1	1.18E+08	1.18E+08	976	1.18E+08	30	8.26892	3.14848
Nc-1-IP_pe	chr1	1.18E+08	1.18E+08	387	1.18E+08	22	10.0534	4.52509
Nc-1-IP_pe	chr1	1.18E+08	1.18E+08	302	1.18E+08	24	8.05897	3.50918
Nc-1-IP_pe	chr1	1.18E+08	1.18E+08	255	1.18E+08	20	6.08336	3.09555
Nc-1-IP_pe	chr1	1.18E+08	1.18E+08	541	1.18E+08	18	4.96968	2.80074
Nc-1-IP_pe	chr1	1.18E+08	1.18E+08	468	1.18E+08	16	6.03248	3.44098
Nc-1-IP_pe	chr1	1.18E+08	1.18E+08	260	1.18E+08	17	7.97708	4.26215
Nc-1-IP_pe	chr1	1.18E+08	1.18E+08	361	1.18E+08	21	5.88587	2.94733
Nc-1-IP_pe	chr1	1.18E+08	1.18E+08	404	1.18E+08	17	4.35793	2.61776
Nc-1-IP_pe	chr1	1.18E+08	1.18E+08	344	1.18E+08	19	6.33279	3.27955
Nc-1-IP_pe	chr1	1.18E+08	1.18E+08	413	1.18E+08	12	3.33349	2.48364
Nc-1-IP_pe	chr1	1.18E+08	1.18E+08	686	1.18E+08	23	6.41066	3.00019
Nc-1-IP_pe	chr1	1.18E+08	1.18E+08	305	1.18E+08	20	6.07662	3.09292
Nc-1-IP_pe	chr1	1.18E+08	1.18E+08	259	1.18E+08	18	5.72638	3.11298
Nc-1-IP_pe	chr1	1.18E+08	1.18E+08	313	1.18E+08	21	9.29881	4.32834
Nc-1-IP_pe	chr1	1.18E+08	1.18E+08	430	1.18E+08	20	4.42487	2.47493
Nc-1-IP_pe	chr1	1.18E+08	1.18E+08	1017	1.18E+08	24	4.66132	2.37496
Nc-1-IP_pe	chr1	1.19E+08	1.19E+08	392	1.19E+08	18	4.32723	2.54542
Nc-1-IP_pe	chr1	1.19E+08	1.19E+08	500	1.19E+08	20	5.34663	2.81336
Nc-1-IP_pe	chr1	1.19E+08	1.19E+08	602	1.19E+08	28	7.18321	2.94535
Nc-1-IP_pe	chr1	1.19E+08	1.19E+08	432	1.19E+08	22	9.752	4.39412
Nc-1-IP_pe	chr1	1.19E+08	1.19E+08	275	1.19E+08	18	4.6358	2.66698
Nc-1-IP_pe	chr1	1.19E+08	1.19E+08	579	1.19E+08	17	6.73936	3.66261
Nc-1-IP_pe	chr1	1.19E+08	1.19E+08	527	1.19E+08	20	6.08336	3.09555

Nc-1-IP_pe	chr1	1.19E+08	1.19E+08	327	1.19E+08	16	6.33285	3.58457
Nc-1-IP_pe	chr1	1.19E+08	1.19E+08	286	1.19E+08	20	10.5351	5.05526
Nc-1-IP_pe	chr1	1.19E+08	1.19E+08	279	1.19E+08	25	9.19615	3.83259
Nc-1-IP_pe	chr1	1.19E+08	1.19E+08	346	1.19E+08	15	4.80057	2.95908
Nc-1-IP_pe	chr1	1.19E+08	1.19E+08	493	1.19E+08	24	9.05651	3.87976
Nc-1-IP_pe	chr1	1.19E+08	1.19E+08	293	1.19E+08	18	7.73136	4.00629
Nc-1-IP_pe	chr1	1.19E+08	1.19E+08	523	1.19E+08	24	7.19588	3.20323
Nc-1-IP_pe	chr1	1.19E+08	1.19E+08	301	1.19E+08	15	5.85	3.46266
Nc-1-IP_pe	chr1	1.19E+08	1.19E+08	267	1.19E+08	16	4.25252	2.63824
Nc-1-IP_pe	chr1	1.19E+08	1.19E+08	324	1.19E+08	19	8.85804	4.40242
Nc-1-IP_pe	chr1	1.19E+08	1.19E+08	477	1.19E+08	21	8.6741	4.05679
Nc-1-IP_pe	chr1	1.19E+08	1.19E+08	347	1.19E+08	19	3.71826	2.26621
Nc-1-IP_pe	chr1	1.19E+08	1.19E+08	323	1.19E+08	26	8.84747	3.61717
Nc-1-IP_pe	chr1	1.19E+08	1.19E+08	598	1.19E+08	24	8.2826	3.59065
Nc-1-IP_pe	chr1	1.19E+08	1.19E+08	381	1.19E+08	17	5.60572	3.14786
Nc-1-IP_pe	chr1	1.19E+08	1.19E+08	846	1.19E+08	20	7.19732	3.54396
Nc-1-IP_pe	chr1	1.19E+08	1.19E+08	480	1.19E+08	21	4.90204	2.59278
Nc-1-IP_pe	chr1	1.19E+08	1.19E+08	703	1.19E+08	29	11.0682	4.10499
Nc-1-IP_pe	chr1	1.19E+08	1.19E+08	971	1.19E+08	19	6.45747	3.33148
Nc-1-IP_pe	chr1	1.19E+08	1.19E+08	922	1.19E+08	25	10.8182	4.4527
Nc-1-IP_pe	chr1	1.19E+08	1.19E+08	774	1.19E+08	20	6.72028	3.34863
Nc-1-IP_pe	chr1	1.19E+08	1.19E+08	348	1.19E+08	22	8.01709	3.67768
Nc-1-IP_pe	chr1	1.19E+08	1.19E+08	474	1.19E+08	26	8.38703	3.45949
Nc-1-IP_pe	chr1	1.19E+08	1.19E+08	635	1.19E+08	24	5.77667	2.7276
Nc-1-IP_pe	chr1	1.19E+08	1.19E+08	623	1.19E+08	25	10.3273	4.25987
Nc-1-IP_pe	chr1	1.19E+08	1.19E+08	1090	1.19E+08	25	7.86419	3.35906
Nc-1-IP_pe	chr1	1.19E+08	1.19E+08	320	1.19E+08	24	11.8619	5.0251
Nc-1-IP_pe	chr1	1.19E+08	1.19E+08	563	1.19E+08	20	5.70098	2.94771
Nc-1-IP_pe	chr1	1.19E+08	1.19E+08	330	1.19E+08	21	6.66991	3.24296
Nc-1-IP_pe	chr1	1.19E+08	1.19E+08	308	1.19E+08	21	7.34645	3.50809
Nc-1-IP_pe	chr1	1.19E+08	1.19E+08	361	1.19E+08	20	5.70098	2.94771
Nc-1-IP_pe	chr1	1.19E+08	1.19E+08	941	1.19E+08	22	6.23294	3.00503
Nc-1-IP_pe	chr1	1.19E+08	1.19E+08	737	1.19E+08	33	5.85346	2.38278
Nc-1-IP_pe	chr1	1.19E+08	1.19E+08	289	1.19E+08	22	7.7408	3.56938
Nc-1-IP_pe	chr1	1.19E+08	1.19E+08	693	1.19E+08	29	8.84453	3.38462
Nc-1-IP_pe	chr1	1.19E+08	1.19E+08	278	1.19E+08	21	5.02528	2.63626
Nc-1-IP_pe	chr1	1.19E+08	1.19E+08	483	1.19E+08	27	5.48341	2.49845
Nc-1-IP_pe	chr1	1.19E+08	1.19E+08	335	1.19E+08	19	4.50014	2.55537
Nc-1-IP_pe	chr1	1.19E+08	1.19E+08	304	1.19E+08	24	6.87207	3.0918
Nc-1-IP_pe	chr1	1.19E+08	1.19E+08	764	1.19E+08	34	8.6364	3.03105
Nc-1-IP_pe	chr1	1.19E+08	1.19E+08	453	1.19E+08	28	5.16525	2.37168
Nc-1-IP_pe	chr1	1.19E+08	1.19E+08	500	1.19E+08	32	5.43745	2.3127
Nc-1-IP_pe	chr1	1.19E+08	1.19E+08	274	1.19E+08	20	7.97282	3.87239
Nc-1-IP_pe	chr1	1.19E+08	1.19E+08	553	1.19E+08	22	7.27519	3.39036
Nc-1-IP_pe	chr1	1.19E+08	1.19E+08	542	1.19E+08	17	5.54967	3.12324

Nc-1-IP_pe	chr1	1.19E+08	1.19E+08	360	1.19E+08	15	5.20142	3.14789
Nc-1-IP_pe	chr1	1.19E+08	1.19E+08	324	1.19E+08	17	5.9911	3.31919
Nc-1-IP_pe	chr1	1.19E+08	1.19E+08	359	1.19E+08	22	9.39426	4.24119
Nc-1-IP_pe	chr1	1.19E+08	1.19E+08	308	1.19E+08	23	4.99703	2.52478
Nc-1-IP_pe	chr1	1.19E+08	1.19E+08	616	1.19E+08	18	6.56579	3.47503
Nc-1-IP_pe	chr1	1.19E+08	1.19E+08	254	1.19E+08	18	6.63038	3.50359
Nc-1-IP_pe	chr1	1.19E+08	1.19E+08	269	1.19E+08	18	5.72638	3.11298
Nc-1-IP_pe	chr1	1.19E+08	1.19E+08	310	1.19E+08	24	9.91256	4.21286
Nc-1-IP_pe	chr1	1.19E+08	1.19E+08	698	1.19E+08	27	6.34328	2.7488
Nc-1-IP_pe	chr1	1.19E+08	1.19E+08	287	1.19E+08	18	3.08421	2.07298
Nc-1-IP_pe	chr1	1.19E+08	1.19E+08	258	1.19E+08	24	7.61242	3.34923
Nc-1-IP_pe	chr1	1.19E+08	1.19E+08	586	1.19E+08	23	9.17456	4.0326
Nc-1-IP_pe	chr1	1.19E+08	1.19E+08	378	1.19E+08	19	6.12444	3.19357
Nc-1-IP_pe	chr1	1.19E+08	1.19E+08	549	1.19E+08	24	7.70148	3.38085
Nc-1-IP_pe	chr1	1.19E+08	1.19E+08	335	1.19E+08	19	6.0077	3.14582
Nc-1-IP_pe	chr1	1.19E+08	1.19E+08	321	1.19E+08	21	4.34782	2.40029
Nc-1-IP_pe	chr1	1.19E+08	1.19E+08	358	1.19E+08	16	3.64675	2.3825
Nc-1-IP_pe	chr1	1.19E+08	1.19E+08	550	1.19E+08	19	5.15822	2.80735
Nc-1-IP_pe	chr1	1.19E+08	1.19E+08	734	1.19E+08	21	4.34782	2.40029
Nc-1-IP_pe	chr1	1.2E+08	1.2E+08	590	1.2E+08	16	4.25252	2.63824
Nc-1-IP_pe	chr1	1.2E+08	1.2E+08	290	1.2E+08	18	5.33197	2.94862
Nc-1-IP_pe	chr1	1.2E+08	1.2E+08	269	1.2E+08	15	4.70031	2.91253
Nc-1-IP_pe	chr1	1.2E+08	1.2E+08	655	1.2E+08	18	4.32723	2.54542
Nc-1-IP_pe	chr1	1.2E+08	1.2E+08	323	1.2E+08	17	6.40717	3.50839
Nc-1-IP_pe	chr1	1.2E+08	1.2E+08	323	1.2E+08	22	7.27519	3.39036
Nc-1-IP_pe	chr1	1.2E+08	1.2E+08	339	1.2E+08	23	9.58124	4.19714
Nc-1-IP_pe	chr1	1.2E+08	1.2E+08	611	1.2E+08	20	7.0741	3.49302
Nc-1-IP_pe	chr1	1.2E+08	1.2E+08	269	1.2E+08	15	5.2862	3.18838
Nc-1-IP_pe	chr1	1.2E+08	1.2E+08	522	1.2E+08	19	7.29128	3.68799
Nc-1-IP_pe	chr1	1.2E+08	1.2E+08	426	1.2E+08	19	6.32622	3.27682
Nc-1-IP_pe	chr1	1.2E+08	1.2E+08	329	1.2E+08	19	4.23334	2.45554
Nc-1-IP_pe	chr1	1.2E+08	1.2E+08	480	1.2E+08	14	4.85093	3.07379
Nc-1-IP_pe	chr1	1.2E+08	1.2E+08	386	1.2E+08	17	3.69914	2.35176
Nc-1-IP_pe	chr1	1.2E+08	1.2E+08	275	1.2E+08	27	9.006	3.58762
Nc-1-IP_pe	chr1	1.2E+08	1.2E+08	1286	1.2E+08	19	5.70288	3.02257
Nc-1-IP_pe	chr1	1.2E+08	1.2E+08	882	1.2E+08	18	4.6358	2.66698
Nc-1-IP_pe	chr1	1.2E+08	1.2E+08	785	1.2E+08	24	3.57212	2.04455
Nc-1-IP_pe	chr1	1.2E+08	1.2E+08	334	1.2E+08	19	8.78787	4.36918
Nc-1-IP_pe	chr1	1.2E+08	1.2E+08	374	1.2E+08	12	4.1728	2.91706
Nc-1-IP_pe	chr1	1.2E+08	1.2E+08	360	1.2E+08	16	4.3356	2.67395
Nc-1-IP_pe	chr1	1.2E+08	1.2E+08	265	1.2E+08	18	4.87655	2.76319
Nc-1-IP_pe	chr1	1.2E+08	1.2E+08	515	1.2E+08	27	8.19444	3.32086
Nc-1-IP_pe	chr1	1.2E+08	1.2E+08	1157	1.2E+08	18	4.79108	2.72889
Nc-1-IP_pe	chr1	1.2E+08	1.2E+08	291	1.2E+08	17	3.90564	2.43421
Nc-1-IP_pe	chr1	1.2E+08	1.2E+08	286	1.2E+08	17	3.90564	2.43421

Nc-1-IP_pe	chr1	1.2E+08	1.2E+08	974	1.2E+08	20	4.71088	2.57831
Nc-1-IP_pe	chr1	1.2E+08	1.2E+08	342	1.2E+08	20	7.11078	3.50815
Nc-1-IP_pe	chr1	1.2E+08	1.2E+08	2014	1.2E+08	22	8.52649	3.88148
Nc-1-IP_pe	chr1	1.2E+08	1.2E+08	365	1.2E+08	19	6.93814	3.53501
Nc-1-IP_pe	chr1	1.2E+08	1.2E+08	1051	1.2E+08	16	3.94146	2.50592
Nc-1-IP_pe	chr1	1.2E+08	1.2E+08	732	1.2E+08	19	5.60545	2.98361
Nc-1-IP_pe	chr1	1.2E+08	1.2E+08	458	1.2E+08	19	6.32622	3.27682
Nc-1-IP_pe	chr1	1.2E+08	1.2E+08	606	1.2E+08	21	6.66991	3.24296
Nc-1-IP_pe	chr1	1.2E+08	1.2E+08	423	1.2E+08	15	2.95965	2.14351
Nc-1-IP_pe	chr1	1.2E+08	1.2E+08	290	1.2E+08	21	5.5349	2.81884
Nc-1-IP_pe	chr1	1.2E+08	1.2E+08	421	1.2E+08	19	7.75121	3.89165
Nc-1-IP_pe	chr1	1.2E+08	1.2E+08	743	1.2E+08	24	8.53898	3.68518
Nc-1-IP_pe	chr1	1.2E+08	1.2E+08	940	1.2E+08	21	7.98609	3.76767
Nc-1-IP_pe	chr1	1.2E+08	1.2E+08	543	1.2E+08	19	7.14338	3.62357
Nc-1-IP_pe	chr1	1.2E+08	1.2E+08	273	1.2E+08	18	8.37884	4.31591
Nc-1-IP_pe	chr1	1.2E+08	1.2E+08	849	1.2E+08	19	5.90435	3.10381
Nc-1-IP_pe	chr1	1.2E+08	1.2E+08	470	1.2E+08	16	4.96462	2.94973
Nc-1-IP_pe	chr1	1.2E+08	1.2E+08	291	1.2E+08	18	3.52899	2.23922
Nc-1-IP_pe	chr1	1.2E+08	1.2E+08	271	1.2E+08	15	4.7819	2.95039
Nc-1-IP_pe	chr1	1.2E+08	1.2E+08	579	1.2E+08	18	4.6358	2.66698
Nc-1-IP_pe	chr1	1.2E+08	1.2E+08	383	1.2E+08	15	5.92932	3.50195
Nc-1-IP_pe	chr1	1.2E+08	1.2E+08	391	1.2E+08	17	5.05731	2.91036
Nc-1-IP_pe	chr1	1.2E+08	1.2E+08	508	1.2E+08	15	4.40338	2.77622
Nc-1-IP_pe	chr1	1.2E+08	1.2E+08	393	1.2E+08	18	6.15736	3.29676
Nc-1-IP_pe	chr1	1.2E+08	1.2E+08	304	1.2E+08	22	8.79433	3.99081
Nc-1-IP_pe	chr1	1.2E+08	1.2E+08	361	1.2E+08	18	4.96379	2.79835
Nc-1-IP_pe	chr1	1.2E+08	1.2E+08	441	1.2E+08	17	8.1184	4.33308
Nc-1-IP_pe	chr1	1.2E+08	1.2E+08	298	1.2E+08	19	9.78988	4.85493
Nc-1-IP_pe	chr1	1.2E+08	1.2E+08	270	1.2E+08	17	9.49036	5.04699
Nc-1-IP_pe	chr1	1.2E+08	1.2E+08	604	1.2E+08	13	3.60553	2.54847
Nc-1-IP_pe	chr1	1.2E+08	1.2E+08	257	1.2E+08	17	5.54967	3.12324
Nc-1-IP_pe	chr1	1.2E+08	1.2E+08	582	1.2E+08	18	3.77591	2.33276
Nc-1-IP_pe	chr1	1.2E+08	1.2E+08	273	1.2E+08	19	5.90435	3.10381
Nc-1-IP_pe	chr1	1.2E+08	1.2E+08	1140	1.2E+08	29	7.71914	3.04692
Nc-1-IP_pe	chr1	1.2E+08	1.2E+08	275	1.2E+08	21	8.10596	3.8173
Nc-1-IP_pe	chr1	1.2E+08	1.2E+08	361	1.2E+08	15	4.70031	2.91253
Nc-1-IP_pe	chr1	1.2E+08	1.2E+08	375	1.2E+08	17	5.14803	2.94914
Nc-1-IP_pe	chr1	1.2E+08	1.2E+08	499	1.2E+08	21	5.96104	2.97514
Nc-1-IP_pe	chr1	1.2E+08	1.2E+08	269	1.2E+08	19	9.11021	4.52287
Nc-1-IP_pe	chr1	1.2E+08	1.2E+08	454	1.2E+08	20	6.07662	3.09292
Nc-1-IP_pe	chr1	1.2E+08	1.2E+08	932	1.2E+08	31	4.82266	2.19039
Nc-1-IP_pe	chr1	1.2E+08	1.2E+08	264	1.2E+08	21	5.5349	2.81884
Nc-1-IP_pe	chr1	1.2E+08	1.2E+08	450	1.2E+08	18	3.08421	2.07298
Nc-1-IP_pe	chr1	1.2E+08	1.2E+08	359	1.2E+08	22	8.79433	3.99081
Nc-1-IP_pe	chr1	1.2E+08	1.2E+08	491	1.2E+08	12	6.86906	4.44438

Nc-1-IP_pe chr1	1.2E+08	1.2E+08	292	1.2E+08	13	7.7352	4.78626
Nc-1-IP_pe chr1	1.2E+08	1.2E+08	712	1.2E+08	14	5.03085	3.16286
Nc-1-IP_pe chr1	1.2E+08	1.2E+08	315	1.2E+08	22	7.27519	3.39036
Nc-1-IP_pe chr1	1.2E+08	1.2E+08	312	1.2E+08	13	6.15968	3.87845
Nc-1-IP_pe chr1	1.2E+08	1.2E+08	1099	1.2E+08	22	10.6487	4.78948
Nc-1-IP_pe chr1	1.2E+08	1.2E+08	641	1.2E+08	17	7.6273	4.08876
Nc-1-IP_pe chr1	1.2E+08	1.2E+08	475	1.2E+08	9	4.00426	3.12999
Nc-1-IP_pe chr1	1.2E+08	1.2E+08	280	1.2E+08	21	10.0475	4.66519
Nc-1-IP_pe chr1	1.2E+08	1.2E+08	722	1.2E+08	20	7.43645	3.64378
Nc-1-IP_pe chr1	1.2E+08	1.2E+08	894	1.2E+08	16	7.52735	4.17929
Nc-1-IP_pe chr1	1.21E+08	1.21E+08	376	1.21E+08	16	5.82808	3.34463
Nc-1-IP_pe chr1	1.21E+08	1.21E+08	323	1.21E+08	18	9.17568	4.711
Nc-1-IP_pe chr1	1.21E+08	1.21E+08	531	1.21E+08	20	5.34663	2.81336
Nc-1-IP_pe chr1	1.21E+08	1.21E+08	279	1.21E+08	21	7.58657	3.60451
Nc-1-IP_pe chr1	1.21E+08	1.21E+08	469	1.21E+08	18	7.1522	3.73811
Nc-1-IP_pe chr1	1.21E+08	1.21E+08	269	1.21E+08	18	8.10208	4.1823
Nc-1-IP_pe chr1	1.21E+08	1.21E+08	368	1.21E+08	24	14.9722	6.4762
Nc-1-IP_pe chr1	1.21E+08	1.21E+08	270	1.21E+08	12	5.37834	3.57574
Nc-1-IP_pe chr1	1.21E+08	1.21E+08	444	1.21E+08	25	11.6607	4.79439
Nc-1-IP_pe chr1	1.21E+08	1.21E+08	590	1.21E+08	20	6.38846	3.2157
Nc-1-IP_pe chr1	1.21E+08	1.21E+08	258	1.21E+08	11	5.7051	3.90381
Nc-1-IP_pe chr1	1.21E+08	1.21E+08	269	1.21E+08	14	5.03085	3.16286
Nc-1-IP_pe chr1	1.21E+08	1.21E+08	429	1.21E+08	24	10.2246	4.33784
Nc-1-IP_pe chr1	1.21E+08	1.21E+08	541	1.21E+08	17	5.14803	2.94914
Nc-1-IP_pe chr1	1.21E+08	1.21E+08	256	1.21E+08	25	5.1059	2.46996
Nc-1-IP_pe chr1	1.21E+08	1.21E+08	360	1.21E+08	27	6.86154	2.90416
Nc-1-IP_pe chr1	1.21E+08	1.21E+08	651	1.21E+08	20	5.70098	2.94771
Nc-1-IP_pe chr1	1.21E+08	1.21E+08	445	1.21E+08	17	5.14803	2.94914
Nc-1-IP_pe chr1	1.21E+08	1.21E+08	657	1.21E+08	30	12.0847	4.35139
Nc-1-IP_pe chr1	1.21E+08	1.21E+08	575	1.21E+08	13	5.9487	3.76157
Nc-1-IP_pe chr1	1.21E+08	1.21E+08	298	1.21E+08	15	4.40338	2.77622
Nc-1-IP_pe chr1	1.21E+08	1.21E+08	666	1.21E+08	16	8.26996	4.56762
Nc-1-IP_pe chr1	1.21E+08	1.21E+08	477	1.21E+08	13	4.86362	3.18015
Nc-1-IP_pe chr1	1.21E+08	1.21E+08	942	1.21E+08	18	8.99859	4.62187
Nc-1-IP_pe chr1	1.21E+08	1.21E+08	473	1.21E+08	13	3.07238	2.29378
Nc-1-IP_pe chr1	1.21E+08	1.21E+08	253	1.21E+08	12	5.23082	3.49289
Nc-1-IP_pe chr1	1.21E+08	1.21E+08	472	1.21E+08	11	4.23076	3.04719
Nc-1-IP_pe chr1	1.21E+08	1.21E+08	274	1.21E+08	16	7.53857	4.18505
Nc-1-IP_pe chr1	1.21E+08	1.21E+08	307	1.21E+08	15	8.26005	4.73146
Nc-1-IP_pe chr1	1.21E+08	1.21E+08	283	1.21E+08	12	5.23082	3.49289
Nc-1-IP_pe chr1	1.21E+08	1.21E+08	344	1.21E+08	12	5.3037	3.53374
Nc-1-IP_pe chr1	1.21E+08	1.21E+08	1187	1.21E+08	15	5.66913	3.37372
Nc-1-IP_pe chr1	1.21E+08	1.21E+08	720	1.21E+08	12	5.61153	3.70794
Nc-1-IP_pe chr1	1.21E+08	1.21E+08	494	1.21E+08	9	4.20361	3.25317
Nc-1-IP_pe chr1	1.21E+08	1.21E+08	726	1.21E+08	18	7.73136	4.00629

Nc-1-IP_pe	chr1	1.21E+08	1.21E+08	303	1.21E+08	21	7.10961	3.41419
Nc-1-IP_pe	chr1	1.21E+08	1.21E+08	611	1.21E+08	16	8.37012	4.62105
Nc-1-IP_pe	chr1	1.21E+08	1.21E+08	678	1.21E+08	18	7.1522	3.73811
Nc-1-IP_pe	chr1	1.21E+08	1.21E+08	395	1.21E+08	11	3.28475	2.53029
Nc-1-IP_pe	chr1	1.21E+08	1.21E+08	290	1.21E+08	16	8.26996	4.56762
Nc-1-IP_pe	chr1	1.21E+08	1.21E+08	277	1.21E+08	17	9.65211	5.13407
Nc-1-IP_pe	chr1	1.21E+08	1.21E+08	290	1.21E+08	13	6.36468	3.99316
Nc-1-IP_pe	chr1	1.21E+08	1.21E+08	313	1.21E+08	14	6.79972	4.087
Nc-1-IP_pe	chr1	1.21E+08	1.21E+08	408	1.21E+08	12	5.67619	3.74486
Nc-1-IP_pe	chr1	1.21E+08	1.21E+08	443	1.21E+08	21	7.58657	3.60451
Nc-1-IP_pe	chr1	1.21E+08	1.21E+08	353	1.21E+08	14	6.69566	4.03025
Nc-1-IP_pe	chr1	1.21E+08	1.21E+08	258	1.21E+08	8	3.08755	2.66145
Nc-1-IP_pe	chr1	1.21E+08	1.21E+08	314	1.21E+08	12	5.61153	3.70794
Nc-1-IP_pe	chr1	1.21E+08	1.21E+08	282	1.21E+08	11	5.10577	3.5486
Nc-1-IP_pe	chr1	1.21E+08	1.21E+08	1281	1.21E+08	20	6.49719	3.259
Nc-1-IP_pe	chr1	1.21E+08	1.21E+08	257	1.21E+08	12	6.58417	4.27436
Nc-1-IP_pe	chr1	1.21E+08	1.21E+08	675	1.21E+08	10	4.77495	3.48064
Nc-1-IP_pe	chr1	1.21E+08	1.21E+08	341	1.21E+08	14	8.63073	5.12814
Nc-1-IP_pe	chr1	1.21E+08	1.21E+08	590	1.21E+08	12	5.23082	3.49289
Nc-1-IP_pe	chr1	1.21E+08	1.21E+08	439	1.21E+08	9	3.32589	2.71826
Nc-1-IP_pe	chr1	1.22E+08	1.22E+08	670	1.22E+08	17	8.31233	4.43123
Nc-1-IP_pe	chr1	1.22E+08	1.22E+08	432	1.22E+08	11	3.64839	2.72584
Nc-1-IP_pe	chr1	1.25E+08	1.25E+08	304	1.25E+08	14	4.60011	2.95114
Nc-1-IP_pe	chr1	1.25E+08	1.25E+08	262	1.25E+08	15	4.7819	2.95039
Nc-1-IP_pe	chr1	1.25E+08	1.25E+08	365	1.25E+08	11	3.52048	2.65661
Nc-1-IP_pe	chr1	1.25E+08	1.25E+08	344	1.25E+08	16	4.96462	2.94973
Nc-1-IP_pe	chr1	1.25E+08	1.25E+08	1077	1.25E+08	13	5.94838	3.76139
Nc-1-IP_pe	chr1	1.25E+08	1.25E+08	577	1.25E+08	13	6.01225	3.79665
Nc-1-IP_pe	chr1	1.25E+08	1.25E+08	261	1.25E+08	16	7.73914	4.28862
Nc-1-IP_pe	chr1	1.25E+08	1.25E+08	1474	1.25E+08	38	12.1514	3.70494
Nc-1-IP_pe	chr1	1.25E+08	1.25E+08	440	1.25E+08	30	9.06325	3.38223
Nc-1-IP_pe	chr1	1.43E+08	1.43E+08	548	1.43E+08	14	6.13058	3.7272
Nc-1-IP_pe	chr1	1.43E+08	1.43E+08	516	1.43E+08	11	3.68532	2.74592
Nc-1-IP_pe	chr1	1.43E+08	1.43E+08	266	1.43E+08	13	5.36853	3.44651
Nc-1-IP_pe	chr1	1.43E+08	1.43E+08	729	1.43E+08	10	5.23357	3.76063
Nc-1-IP_pe	chr1	1.43E+08	1.43E+08	410	1.43E+08	13	5.36853	3.44651
Nc-1-IP_pe	chr1	1.43E+08	1.43E+08	308	1.43E+08	12	6.58417	4.27436
Nc-1-IP_pe	chr1	1.43E+08	1.43E+08	283	1.43E+08	12	4.70082	3.20033
Nc-1-IP_pe	chr1	1.43E+08	1.43E+08	626	1.43E+08	12	4.70082	3.20033
Nc-1-IP_pe	chr1	1.43E+08	1.43E+08	292	1.43E+08	8	3.56021	2.95918
Nc-1-IP_pe	chr1	1.43E+08	1.43E+08	275	1.43E+08	12	6.31185	4.11348
Nc-1-IP_pe	chr1	1.43E+08	1.43E+08	547	1.43E+08	11	6.03439	4.10251
Nc-1-IP_pe	chr1	1.43E+08	1.43E+08	638	1.43E+08	14	8.29543	4.93196
Nc-1-IP_pe	chr1	1.43E+08	1.43E+08	735	1.43E+08	10	3.89064	2.95552
Nc-1-IP_pe	chr1	1.44E+08	1.44E+08	261	1.44E+08	13	7.42513	4.60316

Nc-1-IP_pe	chr1	1.44E+08	1.44E+08	331	1.44E+08	10	5.23357	3.76063
Nc-1-IP_pe	chr1	1.44E+08	1.44E+08	319	1.44E+08	10	5.23357	3.76063
Nc-1-IP_pe	chr1	1.44E+08	1.44E+08	294	1.44E+08	16	10.117	5.58955
Nc-1-IP_pe	chr1	1.44E+08	1.44E+08	258	1.44E+08	11	4.54404	3.2242
Nc-1-IP_pe	chr1	1.44E+08	1.44E+08	358	1.44E+08	11	4.06403	2.95415
Nc-1-IP_pe	chr1	1.44E+08	1.44E+08	281	1.44E+08	12	3.47963	2.55766
Nc-1-IP_pe	chr1	1.44E+08	1.44E+08	273	1.44E+08	12	5.23082	3.49289
Nc-1-IP_pe	chr1	1.44E+08	1.44E+08	261	1.44E+08	12	6.46791	4.20548
Nc-1-IP_pe	chr1	1.44E+08	1.44E+08	472	1.44E+08	17	7.6273	4.08876
Nc-1-IP_pe	chr1	1.44E+08	1.44E+08	304	1.44E+08	23	4.4731	2.35612
Nc-1-IP_pe	chr1	1.44E+08	1.44E+08	601	1.44E+08	38	11.7	3.58894
Nc-1-IP_pe	chr1	1.44E+08	1.44E+08	500	1.44E+08	59	13.2358	2.99208
Nc-1-IP_pe	chr1	1.44E+08	1.44E+08	697	1.44E+08	26	7.41514	3.13793
Nc-1-IP_pe	chr1	1.44E+08	1.44E+08	697	1.44E+08	15	7.56326	4.34922
Nc-1-IP_pe	chr1	1.44E+08	1.44E+08	415	1.44E+08	10	4.39571	3.25288
Nc-1-IP_pe	chr1	1.44E+08	1.44E+08	282	1.44E+08	11	6.03439	4.10251
Nc-1-IP_pe	chr1	1.44E+08	1.44E+08	484	1.44E+08	19	3.71826	2.26621
Nc-1-IP_pe	chr1	1.44E+08	1.44E+08	668	1.44E+08	12	4.1728	2.91706
Nc-1-IP_pe	chr1	1.44E+08	1.44E+08	307	1.44E+08	13	4.86362	3.18015
Nc-1-IP_pe	chr1	1.44E+08	1.44E+08	291	1.44E+08	13	4.41954	2.952
Nc-1-IP_pe	chr1	1.44E+08	1.44E+08	525	1.44E+08	11	5.52654	3.79706
Nc-1-IP_pe	chr1	1.44E+08	1.44E+08	336	1.44E+08	8	3.0402	2.6319
Nc-1-IP_pe	chr1	1.44E+08	1.44E+08	339	1.44E+08	14	8.63073	5.12814
Nc-1-IP_pe	chr1	1.44E+08	1.44E+08	495	1.44E+08	14	8.29543	4.93196
Nc-1-IP_pe	chr1	1.44E+08	1.44E+08	315	1.44E+08	11	6.03439	4.10251
Nc-1-IP_pe	chr1	1.44E+08	1.44E+08	265	1.44E+08	9	4.46937	3.41876
Nc-1-IP_pe	chr1	1.44E+08	1.44E+08	468	1.44E+08	12	5.23082	3.49289
Nc-1-IP_pe	chr1	1.44E+08	1.44E+08	620	1.44E+08	12	4.24064	2.95299
Nc-1-IP_pe	chr1	1.44E+08	1.44E+08	257	1.44E+08	9	4.46937	3.41876
Nc-1-IP_pe	chr1	1.44E+08	1.44E+08	277	1.44E+08	7	3.06442	2.73501
Nc-1-IP_pe	chr1	1.44E+08	1.44E+08	257	1.44E+08	13	4.02562	2.7544
Nc-1-IP_pe	chr1	1.44E+08	1.44E+08	790	1.44E+08	14	3.86727	2.6027
Nc-1-IP_pe	chr1	1.44E+08	1.44E+08	905	1.44E+08	10	4.62142	3.388
Nc-1-IP_pe	chr1	1.44E+08	1.44E+08	437	1.44E+08	14	5.30793	3.30182
Nc-1-IP_pe	chr1	1.44E+08	1.44E+08	667	1.44E+08	10	3.40382	2.67582
Nc-1-IP_pe	chr1	1.44E+08	1.44E+08	470	1.44E+08	13	3.67377	2.58159
Nc-1-IP_pe	chr1	1.44E+08	1.44E+08	344	1.44E+08	11	4.96316	3.46542
Nc-1-IP_pe	chr1	1.44E+08	1.44E+08	887	1.44E+08	20	9.21699	4.428
Nc-1-IP_pe	chr1	1.44E+08	1.44E+08	358	1.44E+08	15	7.46994	4.29894
Nc-1-IP_pe	chr1	1.44E+08	1.44E+08	484	1.44E+08	17	5.9911	3.31919
Nc-1-IP_pe	chr1	1.44E+08	1.44E+08	337	1.44E+08	15	4.7819	2.95039
Nc-1-IP_pe	chr1	1.44E+08	1.44E+08	441	1.44E+08	12	5.23082	3.49289
Nc-1-IP_pe	chr1	1.45E+08	1.45E+08	493	1.45E+08	9	4.46937	3.41876
Nc-1-IP_pe	chr1	1.45E+08	1.45E+08	884	1.45E+08	11	4.54404	3.2242
Nc-1-IP_pe	chr1	1.45E+08	1.45E+08	297	1.45E+08	8	3.68553	3.03891

Nc-1-IP_pe	chr1	1.45E+08	1.45E+08	841	1.45E+08	18	7.73136	4.00629
Nc-1-IP_pe	chr1	1.45E+08	1.45E+08	795	1.45E+08	13	4.41954	2.952
Nc-1-IP_pe	chr1	1.45E+08	1.45E+08	284	1.45E+08	12	6.86906	4.44438
Nc-1-IP_pe	chr1	1.45E+08	1.45E+08	320	1.45E+08	12	6.86906	4.44438
Nc-1-IP_pe	chr1	1.45E+08	1.45E+08	881	1.45E+08	19	5.15822	2.80735
Nc-1-IP_pe	chr1	1.45E+08	1.45E+08	496	1.45E+08	14	5.838	3.57373
Nc-1-IP_pe	chr1	1.45E+08	1.45E+08	297	1.45E+08	13	4.41954	2.952
Nc-1-IP_pe	chr1	1.45E+08	1.45E+08	1689	1.45E+08	15	7.46994	4.29894
Nc-1-IP_pe	chr1	1.45E+08	1.45E+08	410	1.45E+08	15	8.49563	4.86328
Nc-1-IP_pe	chr1	1.45E+08	1.45E+08	359	1.45E+08	8	3.74505	3.07688
Nc-1-IP_pe	chr1	1.45E+08	1.45E+08	325	1.45E+08	11	4.54404	3.2242
Nc-1-IP_pe	chr1	1.45E+08	1.45E+08	461	1.45E+08	12	6.50787	4.22912
Nc-1-IP_pe	chr1	1.45E+08	1.45E+08	344	1.45E+08	14	7.54286	4.50023
Nc-1-IP_pe	chr1	1.45E+08	1.45E+08	325	1.45E+08	10	4.99888	3.61677
Nc-1-IP_pe	chr1	1.45E+08	1.45E+08	545	1.45E+08	9	3.72192	2.95716
Nc-1-IP_pe	chr1	1.45E+08	1.45E+08	822	1.45E+08	11	6.03439	4.10251
Nc-1-IP_pe	chr1	1.45E+08	1.45E+08	283	1.45E+08	11	5.42344	3.73577
Nc-1-IP_pe	chr1	1.45E+08	1.45E+08	379	1.45E+08	12	3.16137	2.39719
Nc-1-IP_pe	chr1	1.45E+08	1.45E+08	335	1.45E+08	16	8.26996	4.56762
Nc-1-IP_pe	chr1	1.45E+08	1.45E+08	894	1.45E+08	13	5.90111	3.73538
Nc-1-IP_pe	chr1	1.45E+08	1.45E+08	796	1.45E+08	14	4.21449	2.76599
Nc-1-IP_pe	chr1	1.45E+08	1.45E+08	273	1.45E+08	14	6.06519	3.69269
Nc-1-IP_pe	chr1	1.45E+08	1.45E+08	904	1.45E+08	12	3.96559	2.80816
Nc-1-IP_pe	chr1	1.45E+08	1.45E+08	739	1.45E+08	17	6.78076	3.68203
Nc-1-IP_pe	chr1	1.45E+08	1.45E+08	270	1.45E+08	13	4.90859	3.20357
Nc-1-IP_pe	chr1	1.45E+08	1.45E+08	330	1.45E+08	17	5.9911	3.31919
Nc-1-IP_pe	chr1	1.45E+08	1.45E+08	432	1.45E+08	12	5.01934	3.37518
Nc-1-IP_pe	chr1	1.45E+08	1.45E+08	537	1.45E+08	24	5.77667	2.7276
Nc-1-IP_pe	chr1	1.45E+08	1.45E+08	532	1.45E+08	10	4.60647	3.37901
Nc-1-IP_pe	chr1	1.45E+08	1.45E+08	318	1.45E+08	14	7.2429	4.33177
Nc-1-IP_pe	chr1	1.45E+08	1.45E+08	423	1.45E+08	16	9.11744	5.02718
Nc-1-IP_pe	chr1	1.45E+08	1.45E+08	350	1.45E+08	10	5.23357	3.76063
Nc-1-IP_pe	chr1	1.45E+08	1.45E+08	638	1.45E+08	13	7.63554	4.7272
Nc-1-IP_pe	chr1	1.45E+08	1.45E+08	288	1.45E+08	12	3.47963	2.55766
Nc-1-IP_pe	chr1	1.45E+08	1.45E+08	330	1.45E+08	15	5.20142	3.14789
Nc-1-IP_pe	chr1	1.45E+08	1.45E+08	599	1.45E+08	21	10.7603	4.99737
Nc-1-IP_pe	chr1	1.45E+08	1.45E+08	343	1.45E+08	11	6.03439	4.10251
Nc-1-IP_pe	chr1	1.45E+08	1.45E+08	264	1.45E+08	13	4.02562	2.7544
Nc-1-IP_pe	chr1	1.45E+08	1.45E+08	743	1.45E+08	17	5.54967	3.12324
Nc-1-IP_pe	chr1	1.45E+08	1.45E+08	756	1.45E+08	17	5.54967	3.12324
Nc-1-IP_pe	chr1	1.45E+08	1.45E+08	732	1.45E+08	10	3.71867	2.85592
Nc-1-IP_pe	chr1	1.45E+08	1.45E+08	506	1.45E+08	17	6.64406	3.61808
Nc-1-IP_pe	chr1	1.45E+08	1.45E+08	635	1.45E+08	11	2.96397	2.36091
Nc-1-IP_pe	chr1	1.45E+08	1.45E+08	854	1.45E+08	16	6.8987	3.86161
Nc-1-IP_pe	chr1	1.45E+08	1.45E+08	537	1.45E+08	22	6.07094	2.94697

Nc-1-IP_pe	chr1	1.45E+08	1.45E+08	602	1.45E+08	15	4.06016	2.62146
Nc-1-IP_pe	chr1	1.45E+08	1.45E+08	271	1.45E+08	13	5.9487	3.76157
Nc-1-IP_pe	chr1	1.45E+08	1.45E+08	862	1.45E+08	20	9.94634	4.77022
Nc-1-IP_pe	chr1	1.45E+08	1.45E+08	259	1.45E+08	19	6.78661	3.47027
Nc-1-IP_pe	chr1	1.45E+08	1.45E+08	621	1.45E+08	17	7.52577	4.03901
Nc-1-IP_pe	chr1	1.45E+08	1.45E+08	557	1.45E+08	8	3.7276	3.06575
Nc-1-IP_pe	chr1	1.45E+08	1.45E+08	272	1.45E+08	13	7.43036	4.60624
Nc-1-IP_pe	chr1	1.45E+08	1.45E+08	300	1.45E+08	10	3.4604	2.70797
Nc-1-IP_pe	chr1	1.45E+08	1.45E+08	583	1.45E+08	11	4.74697	3.34038
Nc-1-IP_pe	chr1	1.45E+08	1.45E+08	296	1.45E+08	10	3.79754	2.90149
Nc-1-IP_pe	chr1	1.45E+08	1.45E+08	339	1.45E+08	13	5.36853	3.44651
Nc-1-IP_pe	chr1	1.45E+08	1.45E+08	269	1.45E+08	12	5.77527	3.80164
Nc-1-IP_pe	chr1	1.45E+08	1.45E+08	263	1.45E+08	12	4.70082	3.20033
Nc-1-IP_pe	chr1	1.45E+08	1.45E+08	1296	1.45E+08	14	6.69566	4.03025
Nc-1-IP_pe	chr1	1.46E+08	1.46E+08	565	1.46E+08	12	6.31185	4.11348
Nc-1-IP_pe	chr1	1.46E+08	1.46E+08	630	1.46E+08	15	7.46994	4.29894
Nc-1-IP_pe	chr1	1.46E+08	1.46E+08	513	1.46E+08	12	5.01934	3.37518
Nc-1-IP_pe	chr1	1.46E+08	1.46E+08	297	1.46E+08	17	5.14803	2.94914
Nc-1-IP_pe	chr1	1.46E+08	1.46E+08	260	1.46E+08	10	5.23357	3.76063
Nc-1-IP_pe	chr1	1.46E+08	1.46E+08	771	1.46E+08	14	8.15847	4.85247
Nc-1-IP_pe	chr1	1.46E+08	1.46E+08	638	1.46E+08	13	6.36468	3.99316
Nc-1-IP_pe	chr1	1.46E+08	1.46E+08	257	1.46E+08	14	6.8886	4.13568
Nc-1-IP_pe	chr1	1.46E+08	1.46E+08	717	1.46E+08	14	6.61011	3.98382
Nc-1-IP_pe	chr1	1.46E+08	1.46E+08	1004	1.46E+08	14	7.42849	4.43574
Nc-1-IP_pe	chr1	1.46E+08	1.46E+08	460	1.46E+08	9	4.46937	3.41876
Nc-1-IP_pe	chr1	1.46E+08	1.46E+08	321	1.46E+08	9	4.05981	3.16422
Nc-1-IP_pe	chr1	1.46E+08	1.46E+08	409	1.46E+08	9	4.35492	3.34727
Nc-1-IP_pe	chr1	1.46E+08	1.46E+08	343	1.46E+08	9	4.05981	3.16422
Nc-1-IP_pe	chr1	1.46E+08	1.46E+08	380	1.46E+08	15	4.7819	2.95039
Nc-1-IP_pe	chr1	1.46E+08	1.46E+08	256	1.46E+08	12	5.33338	3.55042
Nc-1-IP_pe	chr1	1.46E+08	1.46E+08	561	1.46E+08	13	4.41954	2.952
Nc-1-IP_pe	chr1	1.46E+08	1.46E+08	489	1.46E+08	17	7.21469	3.88822
Nc-1-IP_pe	chr1	1.46E+08	1.46E+08	546	1.46E+08	13	5.36853	3.44651
Nc-1-IP_pe	chr1	1.46E+08	1.46E+08	453	1.46E+08	14	3.55308	2.45762
Nc-1-IP_pe	chr1	1.46E+08	1.46E+08	374	1.46E+08	15	8.25154	4.72672
Nc-1-IP_pe	chr1	1.46E+08	1.46E+08	259	1.46E+08	11	4.06403	2.95415
Nc-1-IP_pe	chr1	1.46E+08	1.46E+08	332	1.46E+08	14	5.51541	3.4073
Nc-1-IP_pe	chr1	1.46E+08	1.46E+08	260	1.46E+08	9	4.46937	3.41876
Nc-1-IP_pe	chr1	1.46E+08	1.46E+08	713	1.46E+08	13	4.56123	3.02417
Nc-1-IP_pe	chr1	1.46E+08	1.46E+08	363	1.46E+08	11	2.96397	2.36091
Nc-1-IP_pe	chr1	1.46E+08	1.46E+08	263	1.46E+08	11	3.64839	2.72584
Nc-1-IP_pe	chr1	1.46E+08	1.46E+08	489	1.46E+08	12	5.74196	3.78252
Nc-1-IP_pe	chr1	1.46E+08	1.46E+08	319	1.46E+08	9	4.46937	3.41876
Nc-1-IP_pe	chr1	1.46E+08	1.46E+08	904	1.46E+08	10	4.82246	3.50943
Nc-1-IP_pe	chr1	1.46E+08	1.46E+08	256	1.46E+08	11	4.88957	3.42271

Nc-1-IP_pe	chr1	1.46E+08	1.46E+08	727	1.46E+08	18	11.2458	5.80565
Nc-1-IP_pe	chr1	1.46E+08	1.46E+08	257	1.46E+08	13	3.07238	2.29378
Nc-1-IP_pe	chr1	1.46E+08	1.46E+08	274	1.46E+08	22	9.92229	4.46789
Nc-1-IP_pe	chr1	1.46E+08	1.46E+08	429	1.46E+08	13	5.9487	3.76157
Nc-1-IP_pe	chr1	1.46E+08	1.46E+08	706	1.46E+08	15	5.20142	3.14789
Nc-1-IP_pe	chr1	1.46E+08	1.46E+08	628	1.46E+08	18	4.32723	2.54542
Nc-1-IP_pe	chr1	1.46E+08	1.46E+08	334	1.46E+08	17	6.47874	3.54137
Nc-1-IP_pe	chr1	1.46E+08	1.46E+08	883	1.46E+08	12	5.15877	3.45264
Nc-1-IP_pe	chr1	1.46E+08	1.46E+08	525	1.46E+08	10	3.89064	2.95552
Nc-1-IP_pe	chr1	1.46E+08	1.46E+08	253	1.46E+08	15	6.78905	3.93887
Nc-1-IP_pe	chr1	1.46E+08	1.46E+08	339	1.46E+08	12	6.86404	4.44138
Nc-1-IP_pe	chr1	1.46E+08	1.46E+08	329	1.46E+08	13	4.41954	2.952
Nc-1-IP_pe	chr1	1.47E+08	1.47E+08	341	1.47E+08	17	9.09431	4.8363
Nc-1-IP_pe	chr1	1.47E+08	1.47E+08	345	1.47E+08	14	6.06519	3.69269
Nc-1-IP_pe	chr1	1.47E+08	1.47E+08	346	1.47E+08	14	6.69566	4.03025
Nc-1-IP_pe	chr1	1.47E+08	1.47E+08	260	1.47E+08	15	7.46994	4.29894
Nc-1-IP_pe	chr1	1.47E+08	1.47E+08	373	1.47E+08	13	6.02837	3.80557
Nc-1-IP_pe	chr1	1.47E+08	1.47E+08	553	1.47E+08	10	4.93609	3.57849
Nc-1-IP_pe	chr1	1.47E+08	1.47E+08	271	1.47E+08	19	5.15822	2.80735
Nc-1-IP_pe	chr1	1.47E+08	1.47E+08	415	1.47E+08	17	11.4763	6.15376
Nc-1-IP_pe	chr1	1.47E+08	1.47E+08	321	1.47E+08	14	7.7972	4.64473
Nc-1-IP_pe	chr1	1.47E+08	1.47E+08	266	1.47E+08	10	3.89064	2.95552
Nc-1-IP_pe	chr1	1.47E+08	1.47E+08	520	1.47E+08	13	7.49774	4.64586
Nc-1-IP_pe	chr1	1.47E+08	1.47E+08	446	1.47E+08	10	4.39571	3.25288
Nc-1-IP_pe	chr1	1.47E+08	1.47E+08	354	1.47E+08	8	3.74505	3.07688
Nc-1-IP_pe	chr1	1.47E+08	1.47E+08	330	1.47E+08	10	5.23357	3.76063
Nc-1-IP_pe	chr1	1.47E+08	1.47E+08	321	1.47E+08	16	8.17084	4.51499
Nc-1-IP_pe	chr1	1.47E+08	1.47E+08	679	1.47E+08	17	7.02067	3.79543
Nc-1-IP_pe	chr1	1.47E+08	1.47E+08	511	1.47E+08	19	5.15822	2.80735
Nc-1-IP_pe	chr1	1.47E+08	1.47E+08	385	1.47E+08	9	3.27326	2.68684
Nc-1-IP_pe	chr1	1.47E+08	1.47E+08	788	1.47E+08	13	6.72991	4.20021
Nc-1-IP_pe	chr1	1.47E+08	1.47E+08	319	1.47E+08	9	4.05981	3.16422
Nc-1-IP_pe	chr1	1.47E+08	1.47E+08	559	1.47E+08	12	6.65086	4.31401
Nc-1-IP_pe	chr1	1.47E+08	1.47E+08	630	1.47E+08	10	4.39571	3.25288
Nc-1-IP_pe	chr1	1.47E+08	1.47E+08	264	1.47E+08	16	4.59224	2.7853
Nc-1-IP_pe	chr1	1.47E+08	1.47E+08	278	1.47E+08	11	5.44042	3.74585
Nc-1-IP_pe	chr1	1.47E+08	1.47E+08	372	1.47E+08	15	9.55381	5.47001
Nc-1-IP_pe	chr1	1.47E+08	1.47E+08	274	1.47E+08	10	4.39571	3.25288
Nc-1-IP_pe	chr1	1.47E+08	1.47E+08	644	1.47E+08	15	8.61047	4.928
Nc-1-IP_pe	chr1	1.47E+08	1.47E+08	266	1.47E+08	11	4.61014	3.26192
Nc-1-IP_pe	chr1	1.47E+08	1.47E+08	271	1.47E+08	15	6.5434	3.81197
Nc-1-IP_pe	chr1	1.47E+08	1.47E+08	328	1.47E+08	9	3.02445	2.53932
Nc-1-IP_pe	chr1	1.48E+08	1.48E+08	422	1.48E+08	17	5.54967	3.12324
Nc-1-IP_pe	chr1	1.48E+08	1.48E+08	352	1.48E+08	8	3.56021	2.95918
Nc-1-IP_pe	chr1	1.48E+08	1.48E+08	291	1.48E+08	11	4.06403	2.95415

Nc-1-IP_pe	chr1	1.48E+08	1.48E+08	899	1.48E+08	14	6.06519	3.69269
Nc-1-IP_pe	chr1	1.48E+08	1.48E+08	496	1.48E+08	12	5.15877	3.45264
Nc-1-IP_pe	chr1	1.48E+08	1.48E+08	593	1.48E+08	10	3.4604	2.70797
Nc-1-IP_pe	chr1	1.48E+08	1.48E+08	394	1.48E+08	10	3.08907	2.49869
Nc-1-IP_pe	chr1	1.48E+08	1.48E+08	289	1.48E+08	14	8.57364	5.09458
Nc-1-IP_pe	chr1	1.48E+08	1.48E+08	514	1.48E+08	17	7.6273	4.08876
Nc-1-IP_pe	chr1	1.48E+08	1.48E+08	454	1.48E+08	16	7.11257	3.96852
Nc-1-IP_pe	chr1	1.48E+08	1.48E+08	391	1.48E+08	15	7.46994	4.29894
Nc-1-IP_pe	chr1	1.48E+08	1.48E+08	304	1.48E+08	11	4.06403	2.95415
Nc-1-IP_pe	chr1	1.48E+08	1.48E+08	360	1.48E+08	11	5.10577	3.5486
Nc-1-IP_pe	chr1	1.48E+08	1.48E+08	449	1.48E+08	13	5.36853	3.44651
Nc-1-IP_pe	chr1	1.48E+08	1.48E+08	338	1.48E+08	10	3.08907	2.49869
Nc-1-IP_pe	chr1	1.48E+08	1.48E+08	565	1.48E+08	13	5.86991	3.71823
Nc-1-IP_pe	chr1	1.48E+08	1.48E+08	609	1.48E+08	9	3.79246	3.00015
Nc-1-IP_pe	chr1	1.48E+08	1.48E+08	405	1.48E+08	9	3.72192	2.95716
Nc-1-IP_pe	chr1	1.48E+08	1.48E+08	644	1.48E+08	15	7.46994	4.29894
Nc-1-IP_pe	chr1	1.48E+08	1.48E+08	801	1.48E+08	13	5.36853	3.44651
Nc-1-IP_pe	chr1	1.48E+08	1.48E+08	742	1.48E+08	13	5.36853	3.44651
Nc-1-IP_pe	chr1	1.48E+08	1.48E+08	254	1.48E+08	9	4.46937	3.41876
Nc-1-IP_pe	chr1	1.48E+08	1.48E+08	397	1.48E+08	9	3.72192	2.95716
Nc-1-IP_pe	chr1	1.48E+08	1.48E+08	303	1.48E+08	11	4.06403	2.95415
Nc-1-IP_pe	chr1	1.48E+08	1.48E+08	603	1.48E+08	13	7.40812	4.59318
Nc-1-IP_pe	chr1	1.48E+08	1.48E+08	914	1.48E+08	14	6.69566	4.03025
Nc-1-IP_pe	chr1	1.48E+08	1.48E+08	458	1.48E+08	8	3.74505	3.07688
Nc-1-IP_pe	chr1	1.48E+08	1.48E+08	634	1.48E+08	12	4.70082	3.20033
Nc-1-IP_pe	chr1	1.48E+08	1.48E+08	393	1.48E+08	18	7.62523	3.95652
Nc-1-IP_pe	chr1	1.48E+08	1.48E+08	286	1.48E+08	14	8.29543	4.93196
Nc-1-IP_pe	chr1	1.48E+08	1.48E+08	286	1.48E+08	17	11.2522	6.02508
Nc-1-IP_pe	chr1	1.48E+08	1.48E+08	375	1.48E+08	13	6.19316	3.89711
Nc-1-IP_pe	chr1	1.48E+08	1.48E+08	289	1.48E+08	12	5.92876	3.89011
Nc-1-IP_pe	chr1	1.48E+08	1.48E+08	357	1.48E+08	13	5.29187	3.40559
Nc-1-IP_pe	chr1	1.48E+08	1.48E+08	263	1.48E+08	13	4.86362	3.18015
Nc-1-IP_pe	chr1	1.48E+08	1.48E+08	329	1.48E+08	13	4.86362	3.18015
Nc-1-IP_pe	chr1	1.48E+08	1.48E+08	278	1.48E+08	14	5.51541	3.4073
Nc-1-IP_pe	chr1	1.48E+08	1.48E+08	307	1.48E+08	12	4.70082	3.20033
Nc-1-IP_pe	chr1	1.48E+08	1.48E+08	302	1.48E+08	12	3.83688	2.74114
Nc-1-IP_pe	chr1	1.48E+08	1.48E+08	331	1.48E+08	10	4.48929	3.30875
Nc-1-IP_pe	chr1	1.48E+08	1.48E+08	655	1.48E+08	18	4.96968	2.80074
Nc-1-IP_pe	chr1	1.48E+08	1.48E+08	865	1.48E+08	15	4.90347	3.00714
Nc-1-IP_pe	chr1	1.48E+08	1.48E+08	577	1.48E+08	18	8.62313	4.4354
Nc-1-IP_pe	chr1	1.48E+08	1.48E+08	255	1.48E+08	13	6.8068	4.24423
Nc-1-IP_pe	chr1	1.48E+08	1.48E+08	657	1.48E+08	11	4.47871	3.18705
Nc-1-IP_pe	chr1	1.48E+08	1.48E+08	394	1.48E+08	10	5.23357	3.76063
Nc-1-IP_pe	chr1	1.48E+08	1.48E+08	268	1.48E+08	12	3.47963	2.55766
Nc-1-IP_pe	chr1	1.48E+08	1.48E+08	465	1.48E+08	10	4.22744	3.15303

Nc-1-IP_pe chr1	1.48E+08	1.48E+08	807	1.48E+08	15	6.19428	3.63445
Nc-1-IP_pe chr1	1.48E+08	1.48E+08	395	1.48E+08	14	7.42849	4.43574
Nc-1-IP_pe chr1	1.48E+08	1.48E+08	542	1.48E+08	15	6.57408	3.82774
Nc-1-IP_pe chr1	1.48E+08	1.48E+08	451	1.48E+08	10	3.4604	2.70797
Nc-1-IP_pe chr1	1.48E+08	1.48E+08	579	1.48E+08	12	3.83688	2.74114
Nc-1-IP_pe chr1	1.48E+08	1.48E+08	581	1.48E+08	11	5.77463	3.94557
Nc-1-IP_pe chr1	1.48E+08	1.48E+08	259	1.48E+08	16	5.82808	3.34463
Nc-1-IP_pe chr1	1.48E+08	1.48E+08	318	1.48E+08	14	4.21449	2.76599
Nc-1-IP_pe chr1	1.48E+08	1.48E+08	563	1.48E+08	12	5.94619	3.9002
Nc-1-IP_pe chr1	1.48E+08	1.48E+08	315	1.48E+08	10	3.89064	2.95552
Nc-1-IP_pe chr1	1.48E+08	1.48E+08	407	1.48E+08	11	5.3563	3.696
Nc-1-IP_pe chr1	1.48E+08	1.48E+08	445	1.48E+08	10	3.89064	2.95552
Nc-1-IP_pe chr1	1.48E+08	1.48E+08	340	1.48E+08	15	6.78905	3.93887
Nc-1-IP_pe chr1	1.48E+08	1.48E+08	277	1.48E+08	10	5.23357	3.76063
Nc-1-IP_pe chr1	1.48E+08	1.48E+08	323	1.48E+08	13	6.62436	4.14003
Nc-1-IP_pe chr1	1.48E+08	1.48E+08	329	1.48E+08	20	3.90729	2.29119
Nc-1-IP_pe chr1	1.49E+08	1.49E+08	606	1.49E+08	13	6.62436	4.14003
Nc-1-IP_pe chr1	1.49E+08	1.49E+08	369	1.49E+08	20	8.56319	4.1316
Nc-1-IP_pe chr1	1.49E+08	1.49E+08	360	1.49E+08	9	3.27326	2.68684
Nc-1-IP_pe chr1	1.49E+08	1.49E+08	283	1.49E+08	14	7.62616	4.54739
Nc-1-IP_pe chr1	1.49E+08	1.49E+08	352	1.49E+08	14	7.81901	4.65719
Nc-1-IP_pe chr1	1.49E+08	1.49E+08	261	1.49E+08	9	3.92293	3.08
Nc-1-IP_pe chr1	1.49E+08	1.49E+08	1166	1.49E+08	21	8.6741	4.05679
Nc-1-IP_pe chr1	1.49E+08	1.49E+08	330	1.49E+08	15	8.65712	4.95438
Nc-1-IP_pe chr1	1.49E+08	1.49E+08	273	1.49E+08	12	6.86404	4.44138
Nc-1-IP_pe chr1	1.49E+08	1.49E+08	288	1.49E+08	10	5.23357	3.76063
Nc-1-IP_pe chr1	1.49E+08	1.49E+08	622	1.49E+08	17	7.6273	4.08876
Nc-1-IP_pe chr1	1.49E+08	1.49E+08	320	1.49E+08	13	5.9487	3.76157
Nc-1-IP_pe chr1	1.49E+08	1.49E+08	528	1.49E+08	18	12.2352	6.35981
Nc-1-IP_pe chr1	1.49E+08	1.49E+08	290	1.49E+08	15	7.02624	4.06292
Nc-1-IP_pe chr1	1.49E+08	1.49E+08	314	1.49E+08	17	5.9911	3.31919
Nc-1-IP_pe chr1	1.49E+08	1.49E+08	264	1.49E+08	14	4.21449	2.76599
Nc-1-IP_pe chr1	1.49E+08	1.49E+08	372	1.49E+08	11	4.00079	2.91908
Nc-1-IP_pe chr1	1.49E+08	1.49E+08	400	1.49E+08	15	4.40338	2.77622
Nc-1-IP_pe chr1	1.49E+08	1.49E+08	309	1.49E+08	11	5.81652	3.97078
Nc-1-IP_pe chr1	1.49E+08	1.49E+08	273	1.49E+08	15	6.19428	3.63445
Nc-1-IP_pe chr1	1.49E+08	1.49E+08	633	1.49E+08	13	6.62436	4.14003
Nc-1-IP_pe chr1	1.49E+08	1.49E+08	313	1.49E+08	11	5.7051	3.90381
Nc-1-IP_pe chr1	1.49E+08	1.49E+08	859	1.49E+08	11	5.10577	3.5486
Nc-1-IP_pe chr1	1.49E+08	1.49E+08	393	1.49E+08	13	4.02562	2.7544
Nc-1-IP_pe chr1	1.49E+08	1.49E+08	256	1.49E+08	16	5.37454	3.13479
Nc-1-IP_pe chr1	1.49E+08	1.49E+08	384	1.49E+08	29	9.79421	3.68331
Nc-1-IP_pe chr1	1.49E+08	1.49E+08	475	1.49E+08	12	4.70082	3.20033
Nc-1-IP_pe chr1	1.49E+08	1.49E+08	582	1.49E+08	12	5.84943	3.84431
Nc-1-IP_pe chr1	1.49E+08	1.49E+08	268	1.49E+08	15	5.66913	3.37372

Nc-1-IP_pe	chr1	1.49E+08	1.49E+08	398	1.49E+08	18	3.77591	2.33276
Nc-1-IP_pe	chr1	1.49E+08	1.49E+08	984	1.49E+08	17	10.1401	5.40027
Nc-1-IP_pe	chr1	1.49E+08	1.49E+08	676	1.49E+08	23	8.6544	3.827
Nc-1-IP_pe	chr1	1.49E+08	1.49E+08	479	1.49E+08	11	3.28475	2.53029
Nc-1-IP_pe	chr1	1.49E+08	1.49E+08	273	1.49E+08	17	7.6273	4.08876
Nc-1-IP_pe	chr1	1.5E+08	1.5E+08	260	1.5E+08	14	5.30793	3.30182
Nc-1-IP_pe	chr1	1.5E+08	1.5E+08	261	1.5E+08	12	5.79203	3.81127
Nc-1-IP_pe	chr1	1.5E+08	1.5E+08	298	1.5E+08	15	4.40338	2.77622
Nc-1-IP_pe	chr1	1.5E+08	1.5E+08	325	1.5E+08	12	6.86906	4.44438
Nc-1-IP_pe	chr1	1.5E+08	1.5E+08	323	1.5E+08	19	6.32622	3.27682
Nc-1-IP_pe	chr1	1.5E+08	1.5E+08	326	1.5E+08	10	3.08907	2.49869
Nc-1-IP_pe	chr1	1.5E+08	1.5E+08	311	1.5E+08	12	3.83688	2.74114
Nc-1-IP_pe	chr1	1.5E+08	1.5E+08	328	1.5E+08	13	4.90859	3.20357
Nc-1-IP_pe	chr1	1.5E+08	1.5E+08	322	1.5E+08	13	6.62436	4.14003
Nc-1-IP_pe	chr1	1.5E+08	1.5E+08	952	1.5E+08	11	6.03439	4.10251
Nc-1-IP_pe	chr1	1.5E+08	1.5E+08	503	1.5E+08	14	5.51541	3.4073
Nc-1-IP_pe	chr1	1.5E+08	1.5E+08	978	1.5E+08	13	4.41954	2.952
Nc-1-IP_pe	chr1	1.5E+08	1.5E+08	314	1.5E+08	13	5.9487	3.76157
Nc-1-IP_pe	chr1	1.5E+08	1.5E+08	278	1.5E+08	8	3.08755	2.66145
Nc-1-IP_pe	chr1	1.5E+08	1.5E+08	264	1.5E+08	10	5.23357	3.76063
Nc-1-IP_pe	chr1	1.5E+08	1.5E+08	484	1.5E+08	15	3.74763	2.48304
Nc-1-IP_pe	chr1	1.5E+08	1.5E+08	626	1.5E+08	14	5.03085	3.16286
Nc-1-IP_pe	chr1	1.5E+08	1.5E+08	274	1.5E+08	10	3.77478	2.88832
Nc-1-IP_pe	chr1	1.5E+08	1.5E+08	955	1.5E+08	17	7.02067	3.79543
Nc-1-IP_pe	chr1	1.5E+08	1.5E+08	1099	1.5E+08	18	7.62523	3.95652
Nc-1-IP_pe	chr1	1.5E+08	1.5E+08	307	1.5E+08	19	7.29128	3.68799
Nc-1-IP_pe	chr1	1.5E+08	1.5E+08	671	1.5E+08	21	9.0131	4.20308
Nc-1-IP_pe	chr1	1.5E+08	1.5E+08	467	1.5E+08	12	5.15877	3.45264
Nc-1-IP_pe	chr1	1.5E+08	1.5E+08	257	1.5E+08	8	3.74505	3.07688
Nc-1-IP_pe	chr1	1.5E+08	1.5E+08	272	1.5E+08	10	4.60647	3.37901
Nc-1-IP_pe	chr1	1.5E+08	1.5E+08	283	1.5E+08	17	7.6273	4.08876
Nc-1-IP_pe	chr1	1.5E+08	1.5E+08	340	1.5E+08	9	4.11647	3.19921
Nc-1-IP_pe	chr1	1.5E+08	1.5E+08	497	1.5E+08	13	6.17638	3.88776
Nc-1-IP_pe	chr1	1.5E+08	1.5E+08	421	1.5E+08	12	4.70082	3.20033
Nc-1-IP_pe	chr1	1.5E+08	1.5E+08	466	1.5E+08	11	5.08426	3.53601
Nc-1-IP_pe	chr1	1.5E+08	1.5E+08	294	1.5E+08	10	3.4604	2.70797
Nc-1-IP_pe	chr1	1.5E+08	1.5E+08	356	1.5E+08	14	4.21449	2.76599
Nc-1-IP_pe	chr1	1.5E+08	1.5E+08	262	1.5E+08	11	5.77463	3.94557
Nc-1-IP_pe	chr1	1.5E+08	1.5E+08	842	1.5E+08	9	4.46937	3.41876
Nc-1-IP_pe	chr1	1.5E+08	1.5E+08	526	1.5E+08	11	6.03439	4.10251
Nc-1-IP_pe	chr1	1.5E+08	1.5E+08	309	1.5E+08	11	5.77463	3.94557
Nc-1-IP_pe	chr1	1.5E+08	1.5E+08	352	1.5E+08	10	5.23357	3.76063
Nc-1-IP_pe	chr1	1.5E+08	1.5E+08	439	1.5E+08	12	5.23082	3.49289
Nc-1-IP_pe	chr1	1.5E+08	1.5E+08	276	1.5E+08	11	5.10577	3.5486
Nc-1-IP_pe	chr1	1.5E+08	1.5E+08	355	1.5E+08	11	5.10577	3.5486

Nc-1-IP_pe	chr1	1.5E+08	1.5E+08	304	1.5E+08	10	5.23357	3.76063
Nc-1-IP_pe	chr1	1.5E+08	1.5E+08	743	1.5E+08	10	5.23357	3.76063
Nc-1-IP_pe	chr1	1.5E+08	1.5E+08	359	1.5E+08	14	5.03085	3.16286
Nc-1-IP_pe	chr1	1.5E+08	1.5E+08	344	1.5E+08	9	3.68012	2.93175
Nc-1-IP_pe	chr1	1.5E+08	1.5E+08	403	1.5E+08	10	4.39571	3.25288
Nc-1-IP_pe	chr1	1.5E+08	1.5E+08	332	1.5E+08	7	2.87285	2.60987
Nc-1-IP_pe	chr1	1.5E+08	1.5E+08	372	1.5E+08	12	5.84275	3.84046
Nc-1-IP_pe	chr1	1.5E+08	1.5E+08	385	1.5E+08	11	4.5176	3.20915
Nc-1-IP_pe	chr1	1.5E+08	1.5E+08	253	1.5E+08	13	5.40091	3.46384
Nc-1-IP_pe	chr1	1.5E+08	1.5E+08	328	1.5E+08	12	5.74196	3.78252
Nc-1-IP_pe	chr1	1.5E+08	1.5E+08	315	1.5E+08	15	9.55381	5.47001
Nc-1-IP_pe	chr1	1.5E+08	1.5E+08	317	1.5E+08	10	5.23357	3.76063
Nc-1-IP_pe	chr1	1.5E+08	1.5E+08	287	1.5E+08	10	5.23357	3.76063
Nc-1-IP_pe	chr1	1.5E+08	1.5E+08	285	1.5E+08	14	7.75387	4.62
Nc-1-IP_pe	chr1	1.5E+08	1.5E+08	298	1.5E+08	14	8.63073	5.12814
Nc-1-IP_pe	chr1	1.5E+08	1.5E+08	608	1.5E+08	9	4.20361	3.25317
Nc-1-IP_pe	chr1	1.5E+08	1.5E+08	782	1.5E+08	11	4.61014	3.26192
Nc-1-IP_pe	chr1	1.5E+08	1.5E+08	279	1.5E+08	12	6.86906	4.44438
Nc-1-IP_pe	chr1	1.5E+08	1.5E+08	328	1.5E+08	12	6.82053	4.41531
Nc-1-IP_pe	chr1	1.5E+08	1.5E+08	276	1.5E+08	11	4.54404	3.2242
Nc-1-IP_pe	chr1	1.5E+08	1.5E+08	278	1.5E+08	14	8.29543	4.93196
Nc-1-IP_pe	chr1	1.5E+08	1.5E+08	411	1.5E+08	13	7.52042	4.65922
Nc-1-IP_pe	chr1	1.5E+08	1.5E+08	313	1.5E+08	10	4.99888	3.61677
Nc-1-IP_pe	chr1	1.5E+08	1.5E+08	424	1.5E+08	12	5.84943	3.84431
Nc-1-IP_pe	chr1	1.5E+08	1.5E+08	302	1.5E+08	8	3.56021	2.95918
Nc-1-IP_pe	chr1	1.5E+08	1.5E+08	364	1.5E+08	10	4.39571	3.25288
Nc-1-IP_pe	chr1	1.5E+08	1.5E+08	635	1.5E+08	9	4.4496	3.40639
Nc-1-IP_pe	chr1	1.5E+08	1.5E+08	863	1.5E+08	18	7.62523	3.95652
Nc-1-IP_pe	chr1	1.5E+08	1.5E+08	273	1.5E+08	10	4.39023	3.24962
Nc-1-IP_pe	chr1	1.51E+08	1.51E+08	256	1.51E+08	10	4.71266	3.44298
Nc-1-IP_pe	chr1	1.51E+08	1.51E+08	366	1.51E+08	15	7.02624	4.06292
Nc-1-IP_pe	chr1	1.51E+08	1.51E+08	273	1.51E+08	17	6.51104	3.5563
Nc-1-IP_pe	chr1	1.51E+08	1.51E+08	324	1.51E+08	9	3.43499	2.78365
Nc-1-IP_pe	chr1	1.51E+08	1.51E+08	258	1.51E+08	14	4.60011	2.95114
Nc-1-IP_pe	chr1	1.51E+08	1.51E+08	255	1.51E+08	9	3.53727	2.84525
Nc-1-IP_pe	chr1	1.51E+08	1.51E+08	285	1.51E+08	16	4.50821	2.74867
Nc-1-IP_pe	chr1	1.51E+08	1.51E+08	685	1.51E+08	18	8.27052	4.2634
Nc-1-IP_pe	chr1	1.51E+08	1.51E+08	300	1.51E+08	14	5.89493	3.6034
Nc-1-IP_pe	chr1	1.51E+08	1.51E+08	282	1.51E+08	15	6.10627	3.59023
Nc-1-IP_pe	chr1	1.51E+08	1.51E+08	315	1.51E+08	11	5.1152	3.55411
Nc-1-IP_pe	chr1	1.51E+08	1.51E+08	280	1.51E+08	11	6.03439	4.10251
Nc-1-IP_pe	chr1	1.51E+08	1.51E+08	254	1.51E+08	11	5.8735	4.00514
Nc-1-IP_pe	chr1	1.51E+08	1.51E+08	380	1.51E+08	9	4.46937	3.41876
Nc-1-IP_pe	chr1	1.51E+08	1.51E+08	269	1.51E+08	9	4.46937	3.41876
Nc-1-IP_pe	chr1	1.51E+08	1.51E+08	934	1.51E+08	36	19.1017	6.06213

Nc-1-IP_pe	chr1	1.51E+08	1.51E+08	399	1.51E+08	9	4.20361	3.25317
Nc-1-IP_pe	chr1	1.51E+08	1.51E+08	640	1.51E+08	12	4.24064	2.95299
Nc-1-IP_pe	chr1	1.51E+08	1.51E+08	450	1.51E+08	8	3.38431	2.8478
Nc-1-IP_pe	chr1	1.51E+08	1.51E+08	442	1.51E+08	9	4.46937	3.41876
Nc-1-IP_pe	chr1	1.51E+08	1.51E+08	510	1.51E+08	9	4.46937	3.41876
Nc-1-IP_pe	chr1	1.51E+08	1.51E+08	663	1.51E+08	10	5.23357	3.76063
Nc-1-IP_pe	chr1	1.51E+08	1.51E+08	255	1.51E+08	9	4.25966	3.28797
Nc-1-IP_pe	chr1	1.51E+08	1.51E+08	267	1.51E+08	11	5.10577	3.5486
Nc-1-IP_pe	chr1	1.51E+08	1.51E+08	309	1.51E+08	12	4.88508	3.30112
Nc-1-IP_pe	chr1	1.51E+08	1.51E+08	274	1.51E+08	8	3.51085	2.92785
Nc-1-IP_pe	chr1	1.51E+08	1.51E+08	291	1.51E+08	12	6.86906	4.44438
Nc-1-IP_pe	chr1	1.51E+08	1.51E+08	439	1.51E+08	9	4.46937	3.41876
Nc-1-IP_pe	chr1	1.51E+08	1.51E+08	464	1.51E+08	10	3.4604	2.70797
Nc-1-IP_pe	chr1	1.51E+08	1.51E+08	289	1.51E+08	11	6.03439	4.10251
Nc-1-IP_pe	chr1	1.51E+08	1.51E+08	278	1.51E+08	10	4.39571	3.25288
Nc-1-IP_pe	chr1	1.51E+08	1.51E+08	342	1.51E+08	8	3.74505	3.07688
Nc-1-IP_pe	chr1	1.51E+08	1.51E+08	538	1.51E+08	10	3.4604	2.70797
Nc-1-IP_pe	chr1	1.51E+08	1.51E+08	368	1.51E+08	10	3.89064	2.95552
Nc-1-IP_pe	chr1	1.51E+08	1.51E+08	402	1.51E+08	12	4.06753	2.86157
Nc-1-IP_pe	chr1	1.51E+08	1.51E+08	489	1.51E+08	13	4.86362	3.18015
Nc-1-IP_pe	chr1	1.51E+08	1.51E+08	268	1.51E+08	13	5.21206	3.36317
Nc-1-IP_pe	chr1	1.51E+08	1.51E+08	1750	1.51E+08	16	6.95583	3.89005
Nc-1-IP_pe	chr1	1.51E+08	1.51E+08	519	1.51E+08	11	4.24272	3.05389
Nc-1-IP_pe	chr1	1.51E+08	1.51E+08	337	1.51E+08	13	5.71736	3.63481
Nc-1-IP_pe	chr1	1.51E+08	1.51E+08	503	1.51E+08	10	4.07292	3.06202
Nc-1-IP_pe	chr1	1.51E+08	1.51E+08	331	1.51E+08	14	6.644	4.00219
Nc-1-IP_pe	chr1	1.51E+08	1.51E+08	730	1.51E+08	18	9.89612	5.08115
Nc-1-IP_pe	chr1	1.51E+08	1.51E+08	541	1.51E+08	21	8.26251	3.88258
Nc-1-IP_pe	chr1	1.51E+08	1.51E+08	391	1.51E+08	17	7.85637	4.20196
Nc-1-IP_pe	chr1	1.51E+08	1.51E+08	882	1.51E+08	13	4.41954	2.952
Nc-1-IP_pe	chr1	1.51E+08	1.51E+08	292	1.51E+08	9	3.72192	2.95716
Nc-1-IP_pe	chr1	1.51E+08	1.51E+08	460	1.51E+08	9	4.46937	3.41876
Nc-1-IP_pe	chr1	1.51E+08	1.51E+08	535	1.51E+08	9	4.05981	3.16422
Nc-1-IP_pe	chr1	1.51E+08	1.51E+08	515	1.51E+08	12	3.16137	2.39719
Nc-1-IP_pe	chr1	1.51E+08	1.51E+08	647	1.51E+08	10	3.50482	2.73328
Nc-1-IP_pe	chr1	1.51E+08	1.51E+08	493	1.51E+08	15	6.69889	3.8921
Nc-1-IP_pe	chr1	1.51E+08	1.51E+08	353	1.51E+08	12	3.83688	2.74114
Nc-1-IP_pe	chr1	1.51E+08	1.51E+08	525	1.51E+08	11	4.06403	2.95415
Nc-1-IP_pe	chr1	1.51E+08	1.51E+08	336	1.51E+08	12	5.61153	3.70794
Nc-1-IP_pe	chr1	1.51E+08	1.51E+08	448	1.51E+08	14	6.96097	4.17548
Nc-1-IP_pe	chr1	1.51E+08	1.51E+08	282	1.51E+08	11	3.64839	2.72584
Nc-1-IP_pe	chr1	1.51E+08	1.51E+08	641	1.51E+08	18	5.33197	2.94862
Nc-1-IP_pe	chr1	1.51E+08	1.51E+08	517	1.51E+08	16	5.37454	3.13479
Nc-1-IP_pe	chr1	1.51E+08	1.51E+08	279	1.51E+08	16	5.82808	3.34463
Nc-1-IP_pe	chr1	1.51E+08	1.51E+08	323	1.51E+08	11	4.01189	2.92523

Nc-1-IP_pe chr1	1.51E+08	1.51E+08	612	1.51E+08	13	6.09351	3.84167
Nc-1-IP_pe chr1	1.51E+08	1.51E+08	345	1.51E+08	13	6.41752	4.02291
Nc-1-IP_pe chr1	1.51E+08	1.51E+08	373	1.51E+08	12	5.23082	3.49289
Nc-1-IP_pe chr1	1.51E+08	1.51E+08	424	1.51E+08	10	4.62142	3.388
Nc-1-IP_pe chr1	1.51E+08	1.51E+08	669	1.51E+08	13	6.72991	4.20021
Nc-1-IP_pe chr1	1.51E+08	1.51E+08	534	1.51E+08	10	3.4604	2.70797
Nc-1-IP_pe chr1	1.51E+08	1.51E+08	424	1.51E+08	8	3.68553	3.03891
Nc-1-IP_pe chr1	1.51E+08	1.51E+08	363	1.51E+08	13	6.62436	4.14003
Nc-1-IP_pe chr1	1.51E+08	1.51E+08	536	1.51E+08	14	6.06519	3.69269
Nc-1-IP_pe chr1	1.51E+08	1.51E+08	431	1.51E+08	11	3.64839	2.72584
Nc-1-IP_pe chr1	1.51E+08	1.51E+08	253	1.51E+08	15	6.10627	3.59023
Nc-1-IP_pe chr1	1.51E+08	1.51E+08	288	1.51E+08	16	4.50821	2.74867
Nc-1-IP_pe chr1	1.51E+08	1.51E+08	911	1.51E+08	16	6.37501	3.60492
Nc-1-IP_pe chr1	1.51E+08	1.51E+08	275	1.51E+08	14	6.69566	4.03025
Nc-1-IP_pe chr1	1.51E+08	1.51E+08	316	1.51E+08	11	5.13077	3.56323
Nc-1-IP_pe chr1	1.51E+08	1.51E+08	353	1.51E+08	16	4.87846	2.91138
Nc-1-IP_pe chr1	1.51E+08	1.51E+08	612	1.51E+08	11	5.52654	3.79706
Nc-1-IP_pe chr1	1.51E+08	1.51E+08	261	1.51E+08	8	3.7276	3.06575
Nc-1-IP_pe chr1	1.51E+08	1.51E+08	568	1.51E+08	9	3.9633	3.10479
Nc-1-IP_pe chr1	1.52E+08	1.52E+08	504	1.52E+08	9	4.17427	3.23498
Nc-1-IP_pe chr1	1.52E+08	1.52E+08	304	1.52E+08	11	4.06403	2.95415
Nc-1-IP_pe chr1	1.52E+08	1.52E+08	505	1.52E+08	12	4.24064	2.95299
Nc-1-IP_pe chr1	1.52E+08	1.52E+08	301	1.52E+08	13	7.42513	4.60316
Nc-1-IP_pe chr1	1.52E+08	1.52E+08	699	1.52E+08	13	7.29853	4.52898
Nc-1-IP_pe chr1	1.52E+08	1.52E+08	350	1.52E+08	11	4.11366	2.98176
Nc-1-IP_pe chr1	1.52E+08	1.52E+08	452	1.52E+08	11	4.11366	2.98176
Nc-1-IP_pe chr1	1.52E+08	1.52E+08	340	1.52E+08	10	3.4604	2.70797
Nc-1-IP_pe chr1	1.52E+08	1.52E+08	271	1.52E+08	15	8.54126	4.88897
Nc-1-IP_pe chr1	1.52E+08	1.52E+08	271	1.52E+08	19	11.831	5.91433
Nc-1-IP_pe chr1	1.52E+08	1.52E+08	1120	1.52E+08	18	6.15736	3.29676
Nc-1-IP_pe chr1	1.52E+08	1.52E+08	561	1.52E+08	9	4.46937	3.41876
Nc-1-IP_pe chr1	1.52E+08	1.52E+08	770	1.52E+08	13	3.67377	2.58159
Nc-1-IP_pe chr1	1.52E+08	1.52E+08	338	1.52E+08	15	7.02624	4.06292
Nc-1-IP_pe chr1	1.52E+08	1.52E+08	885	1.52E+08	11	4.54404	3.2242
Nc-1-IP_pe chr1	1.52E+08	1.52E+08	623	1.52E+08	17	8.86434	4.71565
Nc-1-IP_pe chr1	1.52E+08	1.52E+08	1103	1.52E+08	14	6.14575	3.73522
Nc-1-IP_pe chr1	1.52E+08	1.52E+08	516	1.52E+08	11	3.78682	2.80131
Nc-1-IP_pe chr1	1.52E+08	1.52E+08	272	1.52E+08	12	5.84943	3.84431
Nc-1-IP_pe chr1	1.52E+08	1.52E+08	458	1.52E+08	10	4.07292	3.06202
Nc-1-IP_pe chr1	1.52E+08	1.52E+08	576	1.52E+08	19	6.32622	3.27682
Nc-1-IP_pe chr1	1.52E+08	1.52E+08	681	1.52E+08	13	6.54342	4.09407
Nc-1-IP_pe chr1	1.52E+08	1.52E+08	283	1.52E+08	13	7.02546	4.37016
Nc-1-IP_pe chr1	1.52E+08	1.52E+08	719	1.52E+08	12	5.01934	3.37518
Nc-1-IP_pe chr1	1.52E+08	1.52E+08	396	1.52E+08	13	5.57038	3.55505
Nc-1-IP_pe chr1	1.52E+08	1.52E+08	617	1.52E+08	12	5.45448	3.61875

Nc-1-IP_pe	chr1	1.52E+08	1.52E+08	298	1.52E+08	11	4.11366	2.98176
Nc-1-IP_pe	chr1	1.52E+08	1.52E+08	470	1.52E+08	12	4.75569	3.23024
Nc-1-IP_pe	chr1	1.52E+08	1.52E+08	346	1.52E+08	12	5.3483	3.55882
Nc-1-IP_pe	chr1	1.52E+08	1.52E+08	317	1.52E+08	11	4.65061	3.28507
Nc-1-IP_pe	chr1	1.52E+08	1.52E+08	651	1.52E+08	14	4.52309	2.91384
Nc-1-IP_pe	chr1	1.52E+08	1.52E+08	374	1.52E+08	14	6.06519	3.69269
Nc-1-IP_pe	chr1	1.52E+08	1.52E+08	307	1.52E+08	13	4.86362	3.18015
Nc-1-IP_pe	chr1	1.52E+08	1.52E+08	431	1.52E+08	11	4.88957	3.42271
Nc-1-IP_pe	chr1	1.52E+08	1.52E+08	374	1.52E+08	12	6.18005	4.03623
Nc-1-IP_pe	chr1	1.52E+08	1.52E+08	374	1.52E+08	13	6.96475	4.33508
Nc-1-IP_pe	chr1	1.52E+08	1.52E+08	524	1.52E+08	9	4.46937	3.41876
Nc-1-IP_pe	chr1	1.52E+08	1.52E+08	511	1.52E+08	11	5.13077	3.56323
Nc-1-IP_pe	chr1	1.52E+08	1.52E+08	270	1.52E+08	16	6.51374	3.67221
Nc-1-IP_pe	chr1	1.52E+08	1.52E+08	254	1.52E+08	14	8.30109	4.93525
Nc-1-IP_pe	chr1	1.52E+08	1.52E+08	340	1.52E+08	12	6.40869	4.1705
Nc-1-IP_pe	chr1	1.52E+08	1.52E+08	274	1.52E+08	12	6.86906	4.44438
Nc-1-IP_pe	chr1	1.52E+08	1.52E+08	285	1.52E+08	12	3.16137	2.39719
Nc-1-IP_pe	chr1	1.52E+08	1.52E+08	334	1.52E+08	8	3.74505	3.07688
Nc-1-IP_pe	chr1	1.52E+08	1.52E+08	253	1.52E+08	17	4.78101	2.79343
Nc-1-IP_pe	chr1	1.52E+08	1.52E+08	259	1.52E+08	12	4.24064	2.95299
Nc-1-IP_pe	chr1	1.52E+08	1.52E+08	698	1.52E+08	14	7.34079	4.38651
Nc-1-IP_pe	chr1	1.52E+08	1.52E+08	882	1.52E+08	16	10.0135	5.53039
Nc-1-IP_pe	chr1	1.52E+08	1.52E+08	403	1.52E+08	11	5.03835	3.5092
Nc-1-IP_pe	chr1	1.52E+08	1.52E+08	272	1.52E+08	12	4.31492	2.99249
Nc-1-IP_pe	chr1	1.52E+08	1.52E+08	268	1.52E+08	17	6.47874	3.54137
Nc-1-IP_pe	chr1	1.52E+08	1.52E+08	341	1.52E+08	16	4.59224	2.7853
Nc-1-IP_pe	chr1	1.52E+08	1.52E+08	1699	1.52E+08	59	9.66984	2.45138
Nc-1-IP_pe	chr1	1.52E+08	1.52E+08	874	1.52E+08	59	10.9099	2.63449
Nc-1-IP_pe	chr1	1.52E+08	1.52E+08	488	1.52E+08	30	5.53764	2.40159
Nc-1-IP_pe	chr1	1.52E+08	1.52E+08	268	1.52E+08	19	5.16439	2.80975
Nc-1-IP_pe	chr1	1.52E+08	1.52E+08	302	1.52E+08	9	4.46937	3.41876
Nc-1-IP_pe	chr1	1.52E+08	1.52E+08	877	1.52E+08	14	5.30793	3.30182
Nc-1-IP_pe	chr1	1.52E+08	1.52E+08	646	1.52E+08	14	5.30793	3.30182
Nc-1-IP_pe	chr1	1.52E+08	1.52E+08	603	1.52E+08	12	5.23082	3.49289
Nc-1-IP_pe	chr1	1.52E+08	1.52E+08	393	1.52E+08	14	6.69566	4.03025
Nc-1-IP_pe	chr1	1.52E+08	1.52E+08	589	1.52E+08	13	6.41752	4.02291
Nc-1-IP_pe	chr1	1.52E+08	1.52E+08	316	1.52E+08	12	5.01934	3.37518
Nc-1-IP_pe	chr1	1.52E+08	1.52E+08	574	1.52E+08	10	5.23357	3.76063
Nc-1-IP_pe	chr1	1.52E+08	1.52E+08	325	1.52E+08	13	6.54342	4.09407
Nc-1-IP_pe	chr1	1.52E+08	1.52E+08	517	1.52E+08	9	4.46937	3.41876
Nc-1-IP_pe	chr1	1.52E+08	1.52E+08	352	1.52E+08	10	3.89064	2.95552
Nc-1-IP_pe	chr1	1.53E+08	1.53E+08	815	1.53E+08	9	4.40188	3.37657
Nc-1-IP_pe	chr1	1.53E+08	1.53E+08	473	1.53E+08	11	6.03439	4.10251
Nc-1-IP_pe	chr1	1.53E+08	1.53E+08	306	1.53E+08	8	3.74505	3.07688
Nc-1-IP_pe	chr1	1.53E+08	1.53E+08	435	1.53E+08	11	3.28475	2.53029

Nc-1-IP_pe	chr1	1.53E+08	1.53E+08	285	1.53E+08	11	4.06403	2.95415
Nc-1-IP_pe	chr1	1.53E+08	1.53E+08	444	1.53E+08	13	7.7352	4.78626
Nc-1-IP_pe	chr1	1.53E+08	1.53E+08	527	1.53E+08	11	5.77463	3.94557
Nc-1-IP_pe	chr1	1.53E+08	1.53E+08	664	1.53E+08	14	8.29543	4.93196
Nc-1-IP_pe	chr1	1.53E+08	1.53E+08	374	1.53E+08	10	4.99888	3.61677
Nc-1-IP_pe	chr1	1.53E+08	1.53E+08	408	1.53E+08	11	4.06403	2.95415
Nc-1-IP_pe	chr1	1.53E+08	1.53E+08	274	1.53E+08	10	5.23357	3.76063
Nc-1-IP_pe	chr1	1.53E+08	1.53E+08	582	1.53E+08	10	4.39571	3.25288
Nc-1-IP_pe	chr1	1.53E+08	1.53E+08	469	1.53E+08	14	6.69566	4.03025
Nc-1-IP_pe	chr1	1.53E+08	1.53E+08	288	1.53E+08	11	5.77463	3.94557
Nc-1-IP_pe	chr1	1.53E+08	1.53E+08	322	1.53E+08	11	5.10577	3.5486
Nc-1-IP_pe	chr1	1.53E+08	1.53E+08	362	1.53E+08	20	7.43645	3.64378
Nc-1-IP_pe	chr1	1.53E+08	1.53E+08	379	1.53E+08	13	3.67377	2.58159
Nc-1-IP_pe	chr1	1.53E+08	1.53E+08	321	1.53E+08	12	5.61153	3.70794
Nc-1-IP_pe	chr1	1.53E+08	1.53E+08	286	1.53E+08	11	5.52654	3.79706
Nc-1-IP_pe	chr1	1.53E+08	1.53E+08	450	1.53E+08	13	4.86362	3.18015
Nc-1-IP_pe	chr1	1.53E+08	1.53E+08	301	1.53E+08	9	4.46937	3.41876
Nc-1-IP_pe	chr1	1.53E+08	1.53E+08	303	1.53E+08	12	6.86906	4.44438
Nc-1-IP_pe	chr1	1.53E+08	1.53E+08	282	1.53E+08	9	4.46937	3.41876
Nc-1-IP_pe	chr1	1.53E+08	1.53E+08	417	1.53E+08	11	6.03439	4.10251
Nc-1-IP_pe	chr1	1.53E+08	1.53E+08	278	1.53E+08	12	4.24064	2.95299
Nc-1-IP_pe	chr1	1.53E+08	1.53E+08	277	1.53E+08	10	3.89064	2.95552
Nc-1-IP_pe	chr1	1.53E+08	1.53E+08	362	1.53E+08	12	6.86906	4.44438
Nc-1-IP_pe	chr1	1.53E+08	1.53E+08	518	1.53E+08	11	5.52654	3.79706
Nc-1-IP_pe	chr1	1.53E+08	1.53E+08	359	1.53E+08	14	7.42849	4.43574
Nc-1-IP_pe	chr1	1.53E+08	1.53E+08	581	1.53E+08	11	4.54404	3.2242
Nc-1-IP_pe	chr1	1.53E+08	1.53E+08	592	1.53E+08	12	6.58417	4.27436
Nc-1-IP_pe	chr1	1.53E+08	1.53E+08	503	1.53E+08	9	4.25966	3.28797
Nc-1-IP_pe	chr1	1.53E+08	1.53E+08	903	1.53E+08	16	8.79222	4.84884
Nc-1-IP_pe	chr1	1.53E+08	1.53E+08	507	1.53E+08	12	4.51041	3.09723
Nc-1-IP_pe	chr1	1.53E+08	1.53E+08	837	1.53E+08	13	4.86362	3.18015
Nc-1-IP_pe	chr1	1.53E+08	1.53E+08	319	1.53E+08	11	4.88957	3.42271
Nc-1-IP_pe	chr1	1.53E+08	1.53E+08	299	1.53E+08	16	7.97877	4.4137
Nc-1-IP_pe	chr1	1.53E+08	1.53E+08	263	1.53E+08	13	6.61706	4.13588
Nc-1-IP_pe	chr1	1.53E+08	1.53E+08	801	1.53E+08	13	6.61706	4.13588
Nc-1-IP_pe	chr1	1.53E+08	1.53E+08	382	1.53E+08	12	5.51653	3.6539
Nc-1-IP_pe	chr1	1.53E+08	1.53E+08	314	1.53E+08	16	8.26996	4.56762
Nc-1-IP_pe	chr1	1.53E+08	1.53E+08	279	1.53E+08	15	6.78905	3.93887
Nc-1-IP_pe	chr1	1.53E+08	1.53E+08	583	1.53E+08	11	6.03439	4.10251
Nc-1-IP_pe	chr1	1.53E+08	1.53E+08	507	1.53E+08	9	3.27326	2.68684
Nc-1-IP_pe	chr1	1.53E+08	1.53E+08	271	1.53E+08	12	5.84943	3.84431
Nc-1-IP_pe	chr1	1.53E+08	1.53E+08	279	1.53E+08	8	3.08755	2.66145
Nc-1-IP_pe	chr1	1.53E+08	1.53E+08	260	1.53E+08	8	3.74505	3.07688
Nc-1-IP_pe	chr1	1.53E+08	1.53E+08	286	1.53E+08	13	6.19316	3.89711
Nc-1-IP_pe	chr1	1.53E+08	1.53E+08	570	1.53E+08	12	6.12485	4.004

Nc-1-IP_pe	chr1	1.53E+08	1.53E+08	349	1.53E+08	13	5.36853	3.44651
Nc-1-IP_pe	chr1	1.53E+08	1.53E+08	256	1.53E+08	11	5.63263	3.8604
Nc-1-IP_pe	chr1	1.53E+08	1.53E+08	559	1.53E+08	14	7.18513	4.29958
Nc-1-IP_pe	chr1	1.54E+08	1.54E+08	279	1.54E+08	13	3.67377	2.58159
Nc-1-IP_pe	chr1	1.54E+08	1.54E+08	399	1.54E+08	12	4.75569	3.23024
Nc-1-IP_pe	chr1	1.54E+08	1.54E+08	390	1.54E+08	13	3.35769	2.42919
Nc-1-IP_pe	chr1	1.54E+08	1.54E+08	500	1.54E+08	13	7.43036	4.60624
Nc-1-IP_pe	chr1	1.54E+08	1.54E+08	638	1.54E+08	13	5.29187	3.40559
Nc-1-IP_pe	chr1	1.54E+08	1.54E+08	347	1.54E+08	13	4.73076	3.11129
Nc-1-IP_pe	chr1	1.54E+08	1.54E+08	290	1.54E+08	12	4.42854	3.05323
Nc-1-IP_pe	chr1	1.54E+08	1.54E+08	777	1.54E+08	12	4.70523	3.20273
Nc-1-IP_pe	chr1	1.54E+08	1.54E+08	1147	1.54E+08	13	5.70242	3.62667
Nc-1-IP_pe	chr1	1.54E+08	1.54E+08	595	1.54E+08	13	4.02562	2.7544
Nc-1-IP_pe	chr1	1.54E+08	1.54E+08	347	1.54E+08	14	6.52649	3.93862
Nc-1-IP_pe	chr1	1.54E+08	1.54E+08	443	1.54E+08	24	12.4198	5.27143
Nc-1-IP_pe	chr1	1.54E+08	1.54E+08	884	1.54E+08	13	5.15976	3.33548
Nc-1-IP_pe	chr1	1.54E+08	1.54E+08	270	1.54E+08	10	3.55676	2.76294
Nc-1-IP_pe	chr1	1.54E+08	1.54E+08	638	1.54E+08	15	3.46204	2.35851
Nc-1-IP_pe	chr1	1.54E+08	1.54E+08	401	1.54E+08	13	4.04902	2.76601
Nc-1-IP_pe	chr1	1.54E+08	1.54E+08	441	1.54E+08	15	5.94269	3.50859
Nc-1-IP_pe	chr1	1.54E+08	1.54E+08	409	1.54E+08	10	4.33501	3.21677
Nc-1-IP_pe	chr1	1.54E+08	1.54E+08	546	1.54E+08	11	5.47462	3.76617
Nc-1-IP_pe	chr1	1.54E+08	1.54E+08	452	1.54E+08	12	5.79203	3.81127
Nc-1-IP_pe	chr1	1.54E+08	1.54E+08	1621	1.54E+08	14	6.66104	4.01144
Nc-1-IP_pe	chr1	1.54E+08	1.54E+08	1195	1.54E+08	14	6.42818	3.88572
Nc-1-IP_pe	chr1	1.54E+08	1.54E+08	285	1.54E+08	10	4.12355	3.09176
Nc-1-IP_pe	chr1	1.54E+08	1.54E+08	452	1.54E+08	8	3.68553	3.03891
Nc-1-IP_pe	chr1	1.54E+08	1.54E+08	253	1.54E+08	10	3.08907	2.49869
Nc-1-IP_pe	chr1	1.54E+08	1.54E+08	318	1.54E+08	17	9.09431	4.8363
Nc-1-IP_pe	chr1	1.54E+08	1.54E+08	437	1.54E+08	11	4.88957	3.42271
Nc-1-IP_pe	chr1	1.54E+08	1.54E+08	296	1.54E+08	9	4.088	3.18162
Nc-1-IP_pe	chr1	1.54E+08	1.54E+08	265	1.54E+08	12	3.83688	2.74114
Nc-1-IP_pe	chr1	1.54E+08	1.54E+08	268	1.54E+08	12	4.24064	2.95299
Nc-1-IP_pe	chr1	1.54E+08	1.54E+08	404	1.54E+08	16	5.85814	3.35873
Nc-1-IP_pe	chr1	1.54E+08	1.54E+08	376	1.54E+08	12	4.60645	3.1491
Nc-1-IP_pe	chr1	1.54E+08	1.54E+08	274	1.54E+08	14	5.51541	3.4073
Nc-1-IP_pe	chr1	1.54E+08	1.54E+08	481	1.54E+08	9	3.85693	3.03955
Nc-1-IP_pe	chr1	1.54E+08	1.54E+08	417	1.54E+08	19	10.6211	5.27544
Nc-1-IP_pe	chr1	1.54E+08	1.54E+08	631	1.54E+08	14	4.60011	2.95114
Nc-1-IP_pe	chr1	1.54E+08	1.54E+08	465	1.54E+08	10	3.89064	2.95552
Nc-1-IP_pe	chr1	1.54E+08	1.54E+08	270	1.54E+08	13	7.7352	4.78626
Nc-1-IP_pe	chr1	1.54E+08	1.54E+08	635	1.54E+08	9	4.46937	3.41876
Nc-1-IP_pe	chr1	1.54E+08	1.54E+08	256	1.54E+08	11	5.10577	3.5486
Nc-1-IP_pe	chr1	1.54E+08	1.54E+08	362	1.54E+08	11	4.06403	2.95415
Nc-1-IP_pe	chr1	1.54E+08	1.54E+08	394	1.54E+08	12	6.86906	4.44438

Nc-1-IP_pe	chr1	1.54E+08	1.54E+08	272	1.54E+08	9	4.01804	3.13847
Nc-1-IP_pe	chr1	1.54E+08	1.54E+08	421	1.54E+08	15	8.26005	4.73146
Nc-1-IP_pe	chr1	1.54E+08	1.54E+08	451	1.54E+08	10	4.07292	3.06202
Nc-1-IP_pe	chr1	1.54E+08	1.54E+08	710	1.54E+08	14	5.98179	3.64885
Nc-1-IP_pe	chr1	1.54E+08	1.54E+08	367	1.54E+08	12	3.83688	2.74114
Nc-1-IP_pe	chr1	1.54E+08	1.54E+08	301	1.54E+08	18	6.15736	3.29676
Nc-1-IP_pe	chr1	1.54E+08	1.54E+08	849	1.54E+08	16	5.82808	3.34463
Nc-1-IP_pe	chr1	1.54E+08	1.54E+08	294	1.54E+08	16	6.33285	3.58457
Nc-1-IP_pe	chr1	1.54E+08	1.54E+08	382	1.54E+08	17	9.3108	4.95102
Nc-1-IP_pe	chr1	1.54E+08	1.54E+08	494	1.54E+08	15	5.58327	3.33181
Nc-1-IP_pe	chr1	1.54E+08	1.54E+08	459	1.54E+08	10	4.83846	3.51913
Nc-1-IP_pe	chr1	1.54E+08	1.54E+08	414	1.54E+08	9	2.89249	2.46179
Nc-1-IP_pe	chr1	1.54E+08	1.54E+08	503	1.54E+08	9	4.25966	3.28797
Nc-1-IP_pe	chr1	1.54E+08	1.54E+08	338	1.54E+08	9	4.33944	3.33761
Nc-1-IP_pe	chr1	1.54E+08	1.54E+08	505	1.54E+08	11	6.03439	4.10251
Nc-1-IP_pe	chr1	1.54E+08	1.54E+08	486	1.54E+08	9	4.25966	3.28797
Nc-1-IP_pe	chr1	1.54E+08	1.54E+08	579	1.54E+08	9	4.46937	3.41876
Nc-1-IP_pe	chr1	1.54E+08	1.54E+08	256	1.54E+08	11	4.54404	3.2242
Nc-1-IP_pe	chr1	1.54E+08	1.54E+08	283	1.54E+08	7	3.06442	2.73501
Nc-1-IP_pe	chr1	1.54E+08	1.54E+08	342	1.54E+08	11	4.74697	3.34038
Nc-1-IP_pe	chr1	1.54E+08	1.54E+08	336	1.54E+08	12	6.86906	4.44438
Nc-1-IP_pe	chr1	1.54E+08	1.54E+08	254	1.54E+08	11	4.06403	2.95415
Nc-1-IP_pe	chr1	1.54E+08	1.54E+08	256	1.54E+08	11	4.96316	3.46542
Nc-1-IP_pe	chr1	1.54E+08	1.54E+08	303	1.54E+08	11	5.44042	3.74585
Nc-1-IP_pe	chr1	1.54E+08	1.54E+08	265	1.54E+08	10	3.89064	2.95552
Nc-1-IP_pe	chr1	1.54E+08	1.54E+08	655	1.54E+08	12	4.70082	3.20033
Nc-1-IP_pe	chr1	1.54E+08	1.54E+08	329	1.54E+08	15	6.19428	3.63445
Nc-1-IP_pe	chr1	1.54E+08	1.54E+08	280	1.54E+08	11	6.03439	4.10251
Nc-1-IP_pe	chr1	1.54E+08	1.54E+08	399	1.54E+08	12	4.24064	2.95299
Nc-1-IP_pe	chr1	1.54E+08	1.54E+08	711	1.54E+08	11	5.7051	3.90381
Nc-1-IP_pe	chr1	1.54E+08	1.54E+08	476	1.54E+08	15	5.66913	3.37372
Nc-1-IP_pe	chr1	1.54E+08	1.54E+08	513	1.54E+08	11	4.06403	2.95415
Nc-1-IP_pe	chr1	1.54E+08	1.54E+08	681	1.54E+08	18	10.7376	5.5283
Nc-1-IP_pe	chr1	1.54E+08	1.54E+08	1618	1.54E+08	16	5.82808	3.34463
Nc-1-IP_pe	chr1	1.54E+08	1.54E+08	279	1.54E+08	10	4.53273	3.33475
Nc-1-IP_pe	chr1	1.54E+08	1.54E+08	311	1.54E+08	13	6.62436	4.14003
Nc-1-IP_pe	chr1	1.54E+08	1.54E+08	719	1.54E+08	13	5.90111	3.73538
Nc-1-IP_pe	chr1	1.54E+08	1.54E+08	1155	1.54E+08	14	5.43414	3.36584
Nc-1-IP_pe	chr1	1.54E+08	1.54E+08	287	1.54E+08	8	3.64414	3.01254
Nc-1-IP_pe	chr1	1.54E+08	1.54E+08	553	1.54E+08	11	5.3563	3.696
Nc-1-IP_pe	chr1	1.54E+08	1.54E+08	521	1.54E+08	15	5.34394	3.21607
Nc-1-IP_pe	chr1	1.54E+08	1.54E+08	376	1.54E+08	13	4.86362	3.18015
Nc-1-IP_pe	chr1	1.54E+08	1.54E+08	299	1.54E+08	11	4.91882	3.43967
Nc-1-IP_pe	chr1	1.54E+08	1.54E+08	439	1.54E+08	14	5.98179	3.64885
Nc-1-IP_pe	chr1	1.54E+08	1.54E+08	726	1.54E+08	11	4.06403	2.95415

Nc-1-IP_pe	chr1	1.54E+08	1.54E+08	748	1.54E+08	10	4.60647	3.37901
Nc-1-IP_pe	chr1	1.54E+08	1.54E+08	288	1.54E+08	10	4.69727	3.4337
Nc-1-IP_pe	chr1	1.54E+08	1.54E+08	870	1.54E+08	13	5.36853	3.44651
Nc-1-IP_pe	chr1	1.54E+08	1.54E+08	673	1.54E+08	10	5.23357	3.76063
Nc-1-IP_pe	chr1	1.54E+08	1.54E+08	261	1.54E+08	12	5.84943	3.84431
Nc-1-IP_pe	chr1	1.54E+08	1.54E+08	602	1.54E+08	11	4.06403	2.95415
Nc-1-IP_pe	chr1	1.54E+08	1.54E+08	434	1.54E+08	13	6.62436	4.14003
Nc-1-IP_pe	chr1	1.54E+08	1.54E+08	263	1.54E+08	9	4.14522	3.217
Nc-1-IP_pe	chr1	1.55E+08	1.55E+08	421	1.55E+08	8	3.74505	3.07688
Nc-1-IP_pe	chr1	1.55E+08	1.55E+08	381	1.55E+08	13	6.62436	4.14003
Nc-1-IP_pe	chr1	1.55E+08	1.55E+08	352	1.55E+08	11	5.10577	3.5486
Nc-1-IP_pe	chr1	1.55E+08	1.55E+08	434	1.55E+08	12	5.15877	3.45264
Nc-1-IP_pe	chr1	1.55E+08	1.55E+08	308	1.55E+08	9	4.1022	3.19039
Nc-1-IP_pe	chr1	1.55E+08	1.55E+08	1095	1.55E+08	12	5.77527	3.80164
Nc-1-IP_pe	chr1	1.55E+08	1.55E+08	480	1.55E+08	13	6.62436	4.14003
Nc-1-IP_pe	chr1	1.55E+08	1.55E+08	596	1.55E+08	12	5.15877	3.45264
Nc-1-IP_pe	chr1	1.55E+08	1.55E+08	467	1.55E+08	12	6.69264	4.3389
Nc-1-IP_pe	chr1	1.55E+08	1.55E+08	297	1.55E+08	10	3.89064	2.95552
Nc-1-IP_pe	chr1	1.55E+08	1.55E+08	447	1.55E+08	10	4.39571	3.25288
Nc-1-IP_pe	chr1	1.55E+08	1.55E+08	256	1.55E+08	8	3.56021	2.95918
Nc-1-IP_pe	chr1	1.55E+08	1.55E+08	355	1.55E+08	12	5.84943	3.84431
Nc-1-IP_pe	chr1	1.55E+08	1.55E+08	303	1.55E+08	13	5.29187	3.40559
Nc-1-IP_pe	chr1	1.55E+08	1.55E+08	279	1.55E+08	8	3.264	2.772
Nc-1-IP_pe	chr1	1.55E+08	1.55E+08	1140	1.55E+08	12	4.24064	2.95299
Nc-1-IP_pe	chr1	1.55E+08	1.55E+08	257	1.55E+08	11	4.54404	3.2242
Nc-1-IP_pe	chr1	1.55E+08	1.55E+08	468	1.55E+08	12	5.23082	3.49289
Nc-1-IP_pe	chr1	1.55E+08	1.55E+08	674	1.55E+08	12	5.61153	3.70794
Nc-1-IP_pe	chr1	1.55E+08	1.55E+08	342	1.55E+08	11	4.06403	2.95415
Nc-1-IP_pe	chr1	1.55E+08	1.55E+08	299	1.55E+08	12	6.58417	4.27436
Nc-1-IP_pe	chr1	1.55E+08	1.55E+08	285	1.55E+08	10	5.01973	3.6295
Nc-1-IP_pe	chr1	1.55E+08	1.55E+08	333	1.55E+08	15	9.55381	5.47001
Nc-1-IP_pe	chr1	1.55E+08	1.55E+08	258	1.55E+08	11	3.28475	2.53029
Nc-1-IP_pe	chr1	1.55E+08	1.55E+08	411	1.55E+08	11	3.64839	2.72584
Nc-1-IP_pe	chr1	1.55E+08	1.55E+08	282	1.55E+08	13	7.58914	4.69977
Nc-1-IP_pe	chr1	1.55E+08	1.55E+08	437	1.55E+08	14	5.51541	3.4073
Nc-1-IP_pe	chr1	1.55E+08	1.55E+08	255	1.55E+08	9	4.05981	3.16422
Nc-1-IP_pe	chr1	1.55E+08	1.55E+08	265	1.55E+08	11	4.06403	2.95415
Nc-1-IP_pe	chr1	1.55E+08	1.55E+08	279	1.55E+08	12	5.15877	3.45264
Nc-1-IP_pe	chr1	1.55E+08	1.55E+08	279	1.55E+08	14	7.56355	4.51193
Nc-1-IP_pe	chr1	1.55E+08	1.55E+08	703	1.55E+08	12	5.84943	3.84431
Nc-1-IP_pe	chr1	1.55E+08	1.55E+08	521	1.55E+08	12	4.70082	3.20033
Nc-1-IP_pe	chr1	1.55E+08	1.55E+08	475	1.55E+08	10	3.4604	2.70797
Nc-1-IP_pe	chr1	1.55E+08	1.55E+08	557	1.55E+08	13	7.00513	4.3584
Nc-1-IP_pe	chr1	1.55E+08	1.55E+08	341	1.55E+08	12	4.70082	3.20033
Nc-1-IP_pe	chr1	1.55E+08	1.55E+08	283	1.55E+08	12	5.61153	3.70794

Nc-1-IP_pe	chr1	1.55E+08	1.55E+08	805	1.55E+08	12	5.15877	3.45264
Nc-1-IP_pe	chr1	1.55E+08	1.55E+08	265	1.55E+08	15	7.43258	4.27887
Nc-1-IP_pe	chr1	1.55E+08	1.55E+08	332	1.55E+08	13	5.36853	3.44651
Nc-1-IP_pe	chr1	1.55E+08	1.55E+08	378	1.55E+08	17	9.72281	5.17231
Nc-1-IP_pe	chr1	1.55E+08	1.55E+08	403	1.55E+08	16	9.11744	5.02718
Nc-1-IP_pe	chr1	1.55E+08	1.55E+08	547	1.55E+08	15	7.56326	4.34922
Nc-1-IP_pe	chr1	1.55E+08	1.55E+08	283	1.55E+08	13	7.34205	4.55444
Nc-1-IP_pe	chr1	1.55E+08	1.55E+08	428	1.55E+08	11	4.54404	3.2242
Nc-1-IP_pe	chr1	1.55E+08	1.55E+08	314	1.55E+08	15	7.56326	4.34922
Nc-1-IP_pe	chr1	1.55E+08	1.55E+08	303	1.55E+08	10	4.57677	3.36116
Nc-1-IP_pe	chr1	1.55E+08	1.55E+08	281	1.55E+08	14	6.06519	3.69269
Nc-1-IP_pe	chr1	1.55E+08	1.55E+08	272	1.55E+08	11	4.71915	3.32439
Nc-1-IP_pe	chr1	1.55E+08	1.55E+08	378	1.55E+08	13	5.6432	3.59449
Nc-1-IP_pe	chr1	1.55E+08	1.55E+08	282	1.55E+08	13	3.95529	2.71958
Nc-1-IP_pe	chr1	1.55E+08	1.55E+08	274	1.55E+08	12	4.84576	3.27953
Nc-1-IP_pe	chr1	1.55E+08	1.55E+08	747	1.55E+08	13	3.86418	2.67468
Nc-1-IP_pe	chr1	1.55E+08	1.55E+08	400	1.55E+08	12	4.53417	3.11004
Nc-1-IP_pe	chr1	1.55E+08	1.55E+08	357	1.55E+08	12	2.87625	2.25568
Nc-1-IP_pe	chr1	1.55E+08	1.55E+08	338	1.55E+08	15	5.00915	3.05678
Nc-1-IP_pe	chr1	1.55E+08	1.55E+08	263	1.55E+08	17	7.21469	3.88822
Nc-1-IP_pe	chr1	1.55E+08	1.55E+08	410	1.55E+08	13	5.36853	3.44651
Nc-1-IP_pe	chr1	1.55E+08	1.55E+08	258	1.55E+08	10	4.16209	3.11446
Nc-1-IP_pe	chr1	1.55E+08	1.55E+08	330	1.55E+08	13	4.02562	2.7544
Nc-1-IP_pe	chr1	1.55E+08	1.55E+08	400	1.55E+08	12	4.70082	3.20033
Nc-1-IP_pe	chr1	1.55E+08	1.55E+08	461	1.55E+08	12	3.47963	2.55766
Nc-1-IP_pe	chr1	1.55E+08	1.55E+08	434	1.55E+08	15	4.60262	2.86743
Nc-1-IP_pe	chr1	1.55E+08	1.55E+08	902	1.55E+08	16	5.37454	3.13479
Nc-1-IP_pe	chr1	1.55E+08	1.55E+08	313	1.55E+08	11	3.91338	2.8708
Nc-1-IP_pe	chr1	1.55E+08	1.55E+08	871	1.55E+08	16	6.33285	3.58457
Nc-1-IP_pe	chr1	1.55E+08	1.55E+08	407	1.55E+08	13	5.29187	3.40559
Nc-1-IP_pe	chr1	1.55E+08	1.55E+08	380	1.55E+08	16	7.19283	4.00896
Nc-1-IP_pe	chr1	1.55E+08	1.55E+08	541	1.55E+08	13	5.31884	3.41997
Nc-1-IP_pe	chr1	1.55E+08	1.55E+08	664	1.55E+08	12	3.47963	2.55766
Nc-1-IP_pe	chr1	1.55E+08	1.55E+08	269	1.55E+08	12	4.89828	3.30838
Nc-1-IP_pe	chr1	1.55E+08	1.55E+08	322	1.55E+08	14	6.44441	3.89444
Nc-1-IP_pe	chr1	1.55E+08	1.55E+08	815	1.55E+08	14	5.5512	3.42562
Nc-1-IP_pe	chr1	1.55E+08	1.55E+08	261	1.55E+08	16	4.59224	2.7853
Nc-1-IP_pe	chr1	1.55E+08	1.55E+08	355	1.55E+08	11	3.68532	2.74592
Nc-1-IP_pe	chr1	1.55E+08	1.55E+08	349	1.55E+08	13	5.15976	3.33548
Nc-1-IP_pe	chr1	1.55E+08	1.55E+08	318	1.55E+08	12	4.24856	2.9572
Nc-1-IP_pe	chr1	1.55E+08	1.55E+08	463	1.55E+08	13	4.67336	3.0817
Nc-1-IP_pe	chr1	1.55E+08	1.55E+08	550	1.55E+08	15	4.70031	2.91253
Nc-1-IP_pe	chr1	1.55E+08	1.55E+08	449	1.55E+08	15	5.57097	3.32583
Nc-1-IP_pe	chr1	1.55E+08	1.55E+08	302	1.55E+08	15	5.1177	3.10809
Nc-1-IP_pe	chr1	1.55E+08	1.55E+08	551	1.55E+08	15	5.20142	3.14789

Nc-1-IP_pe	chr1	1.55E+08	1.55E+08	1063	1.55E+08	14	7.14701	4.27839
Nc-1-IP_pe	chr1	1.55E+08	1.55E+08	395	1.55E+08	19	9.92774	4.92358
Nc-1-IP_pe	chr1	1.55E+08	1.55E+08	321	1.55E+08	10	4.33501	3.21677
Nc-1-IP_pe	chr1	1.55E+08	1.55E+08	353	1.55E+08	12	5.84943	3.84431
Nc-1-IP_pe	chr1	1.55E+08	1.55E+08	877	1.55E+08	13	6.82624	4.25537
Nc-1-IP_pe	chr1	1.55E+08	1.55E+08	347	1.55E+08	16	3.65575	2.38624
Nc-1-IP_pe	chr1	1.55E+08	1.55E+08	365	1.55E+08	17	4.78101	2.79343
Nc-1-IP_pe	chr1	1.55E+08	1.55E+08	306	1.55E+08	16	4.96462	2.94973
Nc-1-IP_pe	chr1	1.55E+08	1.55E+08	297	1.55E+08	12	4.65549	3.17569
Nc-1-IP_pe	chr1	1.55E+08	1.55E+08	442	1.55E+08	12	4.24064	2.95299
Nc-1-IP_pe	chr1	1.55E+08	1.55E+08	737	1.55E+08	12	6.58898	4.27722
Nc-1-IP_pe	chr1	1.55E+08	1.55E+08	300	1.55E+08	9	2.89249	2.46179
Nc-1-IP_pe	chr1	1.55E+08	1.55E+08	436	1.55E+08	13	6.02837	3.80557
Nc-1-IP_pe	chr1	1.55E+08	1.55E+08	394	1.55E+08	10	3.69653	2.84316
Nc-1-IP_pe	chr1	1.55E+08	1.55E+08	363	1.55E+08	16	5.37454	3.13479
Nc-1-IP_pe	chr1	1.55E+08	1.55E+08	1136	1.55E+08	20	10.2211	4.90229
Nc-1-IP_pe	chr1	1.55E+08	1.55E+08	748	1.55E+08	11	4.8033	3.37283
Nc-1-IP_pe	chr1	1.55E+08	1.55E+08	345	1.55E+08	14	6.49348	3.92083
Nc-1-IP_pe	chr1	1.55E+08	1.55E+08	1146	1.55E+08	18	7.1522	3.73811
Nc-1-IP_pe	chr1	1.55E+08	1.55E+08	312	1.55E+08	11	3.62606	2.71372
Nc-1-IP_pe	chr1	1.55E+08	1.55E+08	294	1.55E+08	13	5.29187	3.40559
Nc-1-IP_pe	chr1	1.55E+08	1.55E+08	524	1.55E+08	12	4.39405	3.03475
Nc-1-IP_pe	chr1	1.55E+08	1.55E+08	520	1.55E+08	21	6.66991	3.24296
Nc-1-IP_pe	chr1	1.55E+08	1.55E+08	346	1.55E+08	18	8.25345	4.25515
Nc-1-IP_pe	chr1	1.55E+08	1.55E+08	253	1.55E+08	14	5.03085	3.16286
Nc-1-IP_pe	chr1	1.55E+08	1.55E+08	329	1.55E+08	11	3.3117	2.54465
Nc-1-IP_pe	chr1	1.55E+08	1.55E+08	537	1.55E+08	13	5.93256	3.75268
Nc-1-IP_pe	chr1	1.55E+08	1.55E+08	1458	1.55E+08	12	5.15877	3.45264
Nc-1-IP_pe	chr1	1.55E+08	1.55E+08	280	1.55E+08	10	3.21261	2.56787
Nc-1-IP_pe	chr1	1.55E+08	1.55E+08	490	1.55E+08	10	3.89064	2.95552
Nc-1-IP_pe	chr1	1.55E+08	1.55E+08	271	1.55E+08	11	4.06403	2.95415
Nc-1-IP_pe	chr1	1.55E+08	1.55E+08	585	1.55E+08	13	5.36853	3.44651
Nc-1-IP_pe	chr1	1.55E+08	1.55E+08	258	1.55E+08	10	3.03453	2.46828
Nc-1-IP_pe	chr1	1.55E+08	1.55E+08	324	1.55E+08	13	4.02562	2.7544
Nc-1-IP_pe	chr1	1.55E+08	1.55E+08	361	1.55E+08	13	5.49888	3.51647
Nc-1-IP_pe	chr1	1.55E+08	1.55E+08	761	1.55E+08	15	4.40338	2.77622
Nc-1-IP_pe	chr1	1.55E+08	1.55E+08	293	1.55E+08	18	7.73136	4.00629
Nc-1-IP_pe	chr1	1.55E+08	1.55E+08	258	1.55E+08	11	4.06403	2.95415
Nc-1-IP_pe	chr1	1.55E+08	1.55E+08	1283	1.55E+08	15	7.19874	4.15407
Nc-1-IP_pe	chr1	1.55E+08	1.55E+08	429	1.55E+08	13	4.41954	2.952
Nc-1-IP_pe	chr1	1.55E+08	1.55E+08	608	1.55E+08	13	6.09351	3.84167
Nc-1-IP_pe	chr1	1.55E+08	1.55E+08	258	1.55E+08	13	4.41954	2.952
Nc-1-IP_pe	chr1	1.55E+08	1.55E+08	839	1.55E+08	14	7.54286	4.50023
Nc-1-IP_pe	chr1	1.55E+08	1.55E+08	263	1.55E+08	10	5.03671	3.63988
Nc-1-IP_pe	chr1	1.55E+08	1.55E+08	953	1.55E+08	13	7.70601	4.76894

Nc-1-IP_pe	chr1	1.55E+08	1.55E+08	988	1.55E+08	12	5.23082	3.49289
Nc-1-IP_pe	chr1	1.55E+08	1.55E+08	326	1.55E+08	13	4.41954	2.952
Nc-1-IP_pe	chr1	1.55E+08	1.55E+08	288	1.55E+08	11	4.69157	3.30855
Nc-1-IP_pe	chr1	1.55E+08	1.55E+08	355	1.55E+08	12	3.47963	2.55766
Nc-1-IP_pe	chr1	1.55E+08	1.55E+08	275	1.55E+08	12	4.46339	3.07193
Nc-1-IP_pe	chr1	1.55E+08	1.55E+08	277	1.55E+08	16	5.37454	3.13479
Nc-1-IP_pe	chr1	1.55E+08	1.55E+08	1788	1.55E+08	19	8.19109	4.09114
Nc-1-IP_pe	chr1	1.55E+08	1.55E+08	303	1.55E+08	9	3.75449	2.977
Nc-1-IP_pe	chr1	1.55E+08	1.55E+08	348	1.55E+08	8	3.74505	3.07688
Nc-1-IP_pe	chr1	1.55E+08	1.55E+08	1101	1.55E+08	11	5.95085	4.05188
Nc-1-IP_pe	chr1	1.55E+08	1.55E+08	276	1.55E+08	10	5.23357	3.76063
Nc-1-IP_pe	chr1	1.55E+08	1.55E+08	408	1.55E+08	9	3.54889	2.85226
Nc-1-IP_pe	chr1	1.55E+08	1.55E+08	404	1.55E+08	13	5.9487	3.76157
Nc-1-IP_pe	chr1	1.55E+08	1.55E+08	423	1.55E+08	13	5.36853	3.44651
Nc-1-IP_pe	chr1	1.55E+08	1.55E+08	289	1.55E+08	7	3.06442	2.73501
Nc-1-IP_pe	chr1	1.55E+08	1.55E+08	331	1.55E+08	14	8.29543	4.93196
Nc-1-IP_pe	chr1	1.56E+08	1.56E+08	276	1.56E+08	13	5.36853	3.44651
Nc-1-IP_pe	chr1	1.56E+08	1.56E+08	280	1.56E+08	11	5.72344	3.91481
Nc-1-IP_pe	chr1	1.56E+08	1.56E+08	276	1.56E+08	9	4.25966	3.28797
Nc-1-IP_pe	chr1	1.56E+08	1.56E+08	300	1.56E+08	12	6.86906	4.44438
Nc-1-IP_pe	chr1	1.56E+08	1.56E+08	255	1.56E+08	9	4.46937	3.41876
Nc-1-IP_pe	chr1	1.56E+08	1.56E+08	368	1.56E+08	15	3.46204	2.35851
Nc-1-IP_pe	chr1	1.56E+08	1.56E+08	434	1.56E+08	8	3.74505	3.07688
Nc-1-IP_pe	chr1	1.56E+08	1.56E+08	286	1.56E+08	13	6.62436	4.14003
Nc-1-IP_pe	chr1	1.56E+08	1.56E+08	332	1.56E+08	9	3.27326	2.68684
Nc-1-IP_pe	chr1	1.56E+08	1.56E+08	459	1.56E+08	9	4.05981	3.16422
Nc-1-IP_pe	chr1	1.56E+08	1.56E+08	306	1.56E+08	14	4.23163	2.77414
Nc-1-IP_pe	chr1	1.56E+08	1.56E+08	583	1.56E+08	13	4.41954	2.952
Nc-1-IP_pe	chr1	1.56E+08	1.56E+08	652	1.56E+08	9	4.46937	3.41876
Nc-1-IP_pe	chr1	1.56E+08	1.56E+08	409	1.56E+08	11	6.03439	4.10251
Nc-1-IP_pe	chr1	1.56E+08	1.56E+08	384	1.56E+08	17	5.14803	2.94914
Nc-1-IP_pe	chr1	1.56E+08	1.56E+08	530	1.56E+08	17	9.20132	4.89288
Nc-1-IP_pe	chr1	1.56E+08	1.56E+08	410	1.56E+08	10	4.0855	3.0694
Nc-1-IP_pe	chr1	1.56E+08	1.56E+08	543	1.56E+08	16	5.82808	3.34463
Nc-1-IP_pe	chr1	1.56E+08	1.56E+08	394	1.56E+08	20	7.85753	3.8227
Nc-1-IP_pe	chr1	1.56E+08	1.56E+08	371	1.56E+08	10	3.08907	2.49869
Nc-1-IP_pe	chr1	1.56E+08	1.56E+08	395	1.56E+08	8	3.51085	2.92785
Nc-1-IP_pe	chr1	1.56E+08	1.56E+08	349	1.56E+08	10	4.39571	3.25288
Nc-1-IP_pe	chr1	1.56E+08	1.56E+08	562	1.56E+08	15	7.46994	4.29894
Nc-1-IP_pe	chr1	1.56E+08	1.56E+08	282	1.56E+08	15	8.26005	4.73146
Nc-1-IP_pe	chr1	1.56E+08	1.56E+08	439	1.56E+08	12	6.19862	4.04709
Nc-1-IP_pe	chr1	1.56E+08	1.56E+08	354	1.56E+08	11	5.09969	3.54504
Nc-1-IP_pe	chr1	1.56E+08	1.56E+08	542	1.56E+08	15	8.72788	4.99447
Nc-1-IP_pe	chr1	1.56E+08	1.56E+08	582	1.56E+08	12	6.58417	4.27436
Nc-1-IP_pe	chr1	1.56E+08	1.56E+08	368	1.56E+08	12	5.23082	3.49289

Nc-1-IP_pe	chr1	1.56E+08	1.56E+08	315	1.56E+08	10	5.23357	3.76063
Nc-1-IP_pe	chr1	1.56E+08	1.56E+08	270	1.56E+08	8	3.74505	3.07688
Nc-1-IP_pe	chr1	1.56E+08	1.56E+08	336	1.56E+08	10	5.23357	3.76063
Nc-1-IP_pe	chr1	1.56E+08	1.56E+08	262	1.56E+08	11	3.64839	2.72584
Nc-1-IP_pe	chr1	1.56E+08	1.56E+08	258	1.56E+08	10	3.4604	2.70797
Nc-1-IP_pe	chr1	1.56E+08	1.56E+08	980	1.56E+08	14	5.98179	3.64885
Nc-1-IP_pe	chr1	1.56E+08	1.56E+08	361	1.56E+08	12	5.84943	3.84431
Nc-1-IP_pe	chr1	1.56E+08	1.56E+08	274	1.56E+08	13	7.27693	4.51635
Nc-1-IP_pe	chr1	1.56E+08	1.56E+08	1067	1.56E+08	17	11.4763	6.15376
Nc-1-IP_pe	chr1	1.56E+08	1.56E+08	458	1.56E+08	14	6.13058	3.7272
Nc-1-IP_pe	chr1	1.56E+08	1.56E+08	300	1.56E+08	18	6.63038	3.50359
Nc-1-IP_pe	chr1	1.56E+08	1.56E+08	673	1.56E+08	13	4.86362	3.18015
Nc-1-IP_pe	chr1	1.56E+08	1.56E+08	377	1.56E+08	11	4.06403	2.95415
Nc-1-IP_pe	chr1	1.56E+08	1.56E+08	333	1.56E+08	16	6.90977	3.86711
Nc-1-IP_pe	chr1	1.56E+08	1.56E+08	477	1.56E+08	13	3.60553	2.54847
Nc-1-IP_pe	chr1	1.56E+08	1.56E+08	347	1.56E+08	14	3.55308	2.45762
Nc-1-IP_pe	chr1	1.56E+08	1.56E+08	428	1.56E+08	13	6.38222	4.00303
Nc-1-IP_pe	chr1	1.56E+08	1.56E+08	669	1.56E+08	13	3.35769	2.42919
Nc-1-IP_pe	chr1	1.56E+08	1.56E+08	302	1.56E+08	15	4.7819	2.95039
Nc-1-IP_pe	chr1	1.56E+08	1.56E+08	414	1.56E+08	8	2.91563	2.55444
Nc-1-IP_pe	chr1	1.56E+08	1.56E+08	406	1.56E+08	11	4.54404	3.2242
Nc-1-IP_pe	chr1	1.56E+08	1.56E+08	277	1.56E+08	11	5.47462	3.76617
Nc-1-IP_pe	chr1	1.56E+08	1.56E+08	363	1.56E+08	8	3.20603	2.7356
Nc-1-IP_pe	chr1	1.56E+08	1.56E+08	765	1.56E+08	12	3.47963	2.55766
Nc-1-IP_pe	chr1	1.56E+08	1.56E+08	288	1.56E+08	15	4.40338	2.77622
Nc-1-IP_pe	chr1	1.56E+08	1.56E+08	422	1.56E+08	19	7.84735	3.93486
Nc-1-IP_pe	chr1	1.56E+08	1.56E+08	287	1.56E+08	14	5.56442	3.43239
Nc-1-IP_pe	chr1	1.56E+08	1.56E+08	425	1.56E+08	13	3.35769	2.42919
Nc-1-IP_pe	chr1	1.56E+08	1.56E+08	307	1.56E+08	10	3.85523	2.93494
Nc-1-IP_pe	chr1	1.56E+08	1.56E+08	1077	1.56E+08	13	5.9487	3.76157
Nc-1-IP_pe	chr1	1.56E+08	1.56E+08	934	1.56E+08	13	4.41954	2.952
Nc-1-IP_pe	chr1	1.56E+08	1.56E+08	1140	1.56E+08	14	5.56442	3.43239
Nc-1-IP_pe	chr1	1.56E+08	1.56E+08	416	1.56E+08	13	5.29187	3.40559
Nc-1-IP_pe	chr1	1.56E+08	1.56E+08	467	1.56E+08	17	8.37483	4.46305
Nc-1-IP_pe	chr1	1.56E+08	1.56E+08	607	1.56E+08	14	3.55308	2.45762
Nc-1-IP_pe	chr1	1.56E+08	1.56E+08	275	1.56E+08	12	4.24064	2.95299
Nc-1-IP_pe	chr1	1.56E+08	1.56E+08	376	1.56E+08	11	4.01189	2.92523
Nc-1-IP_pe	chr1	1.56E+08	1.56E+08	309	1.56E+08	16	7.64983	4.24237
Nc-1-IP_pe	chr1	1.56E+08	1.56E+08	547	1.56E+08	14	5.51541	3.4073
Nc-1-IP_pe	chr1	1.56E+08	1.56E+08	407	1.56E+08	17	4.52162	2.68525
Nc-1-IP_pe	chr1	1.56E+08	1.56E+08	302	1.56E+08	14	5.03085	3.16286
Nc-1-IP_pe	chr1	1.56E+08	1.56E+08	507	1.56E+08	14	5.5249	3.41215
Nc-1-IP_pe	chr1	1.56E+08	1.56E+08	263	1.56E+08	13	4.56123	3.02417
Nc-1-IP_pe	chr1	1.56E+08	1.56E+08	264	1.56E+08	11	3.64839	2.72584
Nc-1-IP_pe	chr1	1.56E+08	1.56E+08	784	1.56E+08	15	6.10627	3.59023

Nc-1-IP_pe	chr1	1.56E+08	1.56E+08	433	1.56E+08	13	4.41954	2.952
Nc-1-IP_pe	chr1	1.56E+08	1.56E+08	266	1.56E+08	9	3.27326	2.68684
Nc-1-IP_pe	chr1	1.56E+08	1.56E+08	393	1.56E+08	12	5.23082	3.49289
Nc-1-IP_pe	chr1	1.56E+08	1.56E+08	344	1.56E+08	12	3.83688	2.74114
Nc-1-IP_pe	chr1	1.56E+08	1.56E+08	847	1.56E+08	12	5.18731	3.46856
Nc-1-IP_pe	chr1	1.56E+08	1.56E+08	463	1.56E+08	13	6.62436	4.14003
Nc-1-IP_pe	chr1	1.56E+08	1.56E+08	290	1.56E+08	11	4.06403	2.95415
Nc-1-IP_pe	chr1	1.56E+08	1.56E+08	497	1.56E+08	9	3.42387	2.77697
Nc-1-IP_pe	chr1	1.56E+08	1.56E+08	279	1.56E+08	10	4.14919	3.10686
Nc-1-IP_pe	chr1	1.56E+08	1.56E+08	260	1.56E+08	11	4.54404	3.2242
Nc-1-IP_pe	chr1	1.56E+08	1.56E+08	340	1.56E+08	44	11.9084	3.31161
Nc-1-IP_pe	chr1	1.56E+08	1.56E+08	271	1.56E+08	14	7.50175	4.47701
Nc-1-IP_pe	chr1	1.56E+08	1.56E+08	391	1.56E+08	11	4.88957	3.42271
Nc-1-IP_pe	chr1	1.56E+08	1.56E+08	608	1.56E+08	11	3.64839	2.72584
Nc-1-IP_pe	chr1	1.56E+08	1.56E+08	311	1.56E+08	15	5.70822	3.39286
Nc-1-IP_pe	chr1	1.56E+08	1.56E+08	289	1.56E+08	15	4.7819	2.95039
Nc-1-IP_pe	chr1	1.56E+08	1.56E+08	255	1.56E+08	15	6.05107	3.5626
Nc-1-IP_pe	chr1	1.56E+08	1.56E+08	436	1.56E+08	12	5.84943	3.84431
Nc-1-IP_pe	chr1	1.56E+08	1.56E+08	553	1.56E+08	12	5.43913	3.61006
Nc-1-IP_pe	chr1	1.56E+08	1.56E+08	543	1.56E+08	10	4.39571	3.25288
Nc-1-IP_pe	chr1	1.56E+08	1.56E+08	297	1.56E+08	17	6.47874	3.54137
Nc-1-IP_pe	chr1	1.56E+08	1.56E+08	265	1.56E+08	11	4.99304	3.4828
Nc-1-IP_pe	chr1	1.56E+08	1.56E+08	831	1.56E+08	16	10.2925	5.69036
Nc-1-IP_pe	chr1	1.56E+08	1.56E+08	702	1.56E+08	11	4.06403	2.95415
Nc-1-IP_pe	chr1	1.56E+08	1.56E+08	532	1.56E+08	19	10.4598	5.19259
Nc-1-IP_pe	chr1	1.56E+08	1.56E+08	619	1.56E+08	12	6.46791	4.20548
Nc-1-IP_pe	chr1	1.56E+08	1.56E+08	334	1.56E+08	10	5.17577	3.72509
Nc-1-IP_pe	chr1	1.56E+08	1.56E+08	336	1.56E+08	11	5.00808	3.49156
Nc-1-IP_pe	chr1	1.56E+08	1.56E+08	346	1.56E+08	9	2.89249	2.46179
Nc-1-IP_pe	chr1	1.56E+08	1.56E+08	335	1.56E+08	11	3.64839	2.72584
Nc-1-IP_pe	chr1	1.56E+08	1.56E+08	386	1.56E+08	13	3.52389	2.509
Nc-1-IP_pe	chr1	1.56E+08	1.56E+08	668	1.56E+08	17	5.14803	2.94914
Nc-1-IP_pe	chr1	1.56E+08	1.56E+08	734	1.56E+08	17	4.78101	2.79343
Nc-1-IP_pe	chr1	1.56E+08	1.56E+08	425	1.56E+08	15	7.19874	4.15407
Nc-1-IP_pe	chr1	1.56E+08	1.56E+08	264	1.56E+08	11	4.06403	2.95415
Nc-1-IP_pe	chr1	1.56E+08	1.56E+08	1249	1.56E+08	14	4.60011	2.95114
Nc-1-IP_pe	chr1	1.56E+08	1.56E+08	303	1.56E+08	10	3.44377	2.69851
Nc-1-IP_pe	chr1	1.56E+08	1.56E+08	254	1.56E+08	15	6.85973	3.97568
Nc-1-IP_pe	chr1	1.56E+08	1.56E+08	343	1.56E+08	12	2.87625	2.25568
Nc-1-IP_pe	chr1	1.56E+08	1.56E+08	330	1.56E+08	14	3.87746	2.60745
Nc-1-IP_pe	chr1	1.56E+08	1.56E+08	260	1.56E+08	16	5.37454	3.13479
Nc-1-IP_pe	chr1	1.56E+08	1.56E+08	352	1.56E+08	11	4.47871	3.18705
Nc-1-IP_pe	chr1	1.56E+08	1.56E+08	401	1.56E+08	14	7.34079	4.38651
Nc-1-IP_pe	chr1	1.56E+08	1.56E+08	368	1.56E+08	11	4.74697	3.34038
Nc-1-IP_pe	chr1	1.56E+08	1.56E+08	934	1.56E+08	12	5.23082	3.49289

Nc-1-IP_pe	chr1	1.56E+08	1.56E+08	290	1.56E+08	16	9.49293	5.236
Nc-1-IP_pe	chr1	1.56E+08	1.56E+08	1185	1.56E+08	18	11.2181	5.79036
Nc-1-IP_pe	chr1	1.56E+08	1.56E+08	448	1.56E+08	14	5.56442	3.43239
Nc-1-IP_pe	chr1	1.56E+08	1.56E+08	274	1.56E+08	14	5.51541	3.4073
Nc-1-IP_pe	chr1	1.56E+08	1.56E+08	596	1.56E+08	14	6.14575	3.73522
Nc-1-IP_pe	chr1	1.56E+08	1.56E+08	1404	1.56E+08	16	7.90386	4.37444
Nc-1-IP_pe	chr1	1.56E+08	1.56E+08	771	1.56E+08	13	4.39942	2.9418
Nc-1-IP_pe	chr1	1.56E+08	1.56E+08	638	1.56E+08	17	6.38155	3.49661
Nc-1-IP_pe	chr1	1.57E+08	1.57E+08	268	1.57E+08	13	5.36853	3.44651
Nc-1-IP_pe	chr1	1.57E+08	1.57E+08	268	1.57E+08	10	4.20113	3.13749
Nc-1-IP_pe	chr1	1.57E+08	1.57E+08	769	1.57E+08	15	8.08099	4.63212
Nc-1-IP_pe	chr1	1.57E+08	1.57E+08	486	1.57E+08	11	4.61014	3.26192
Nc-1-IP_pe	chr1	1.57E+08	1.57E+08	333	1.57E+08	14	6.06519	3.69269
Nc-1-IP_pe	chr1	1.57E+08	1.57E+08	284	1.57E+08	17	5.54967	3.12324
Nc-1-IP_pe	chr1	1.57E+08	1.57E+08	334	1.57E+08	12	5.80886	3.82095
Nc-1-IP_pe	chr1	1.57E+08	1.57E+08	482	1.57E+08	12	3.83688	2.74114
Nc-1-IP_pe	chr1	1.57E+08	1.57E+08	352	1.57E+08	11	3.64839	2.72584
Nc-1-IP_pe	chr1	1.57E+08	1.57E+08	343	1.57E+08	15	4.40338	2.77622
Nc-1-IP_pe	chr1	1.57E+08	1.57E+08	408	1.57E+08	10	3.89064	2.95552
Nc-1-IP_pe	chr1	1.57E+08	1.57E+08	326	1.57E+08	13	6.63566	4.14646
Nc-1-IP_pe	chr1	1.57E+08	1.57E+08	310	1.57E+08	12	5.45448	3.61875
Nc-1-IP_pe	chr1	1.57E+08	1.57E+08	312	1.57E+08	15	7.52557	4.32889
Nc-1-IP_pe	chr1	1.57E+08	1.57E+08	318	1.57E+08	14	6.06519	3.69269
Nc-1-IP_pe	chr1	1.57E+08	1.57E+08	602	1.57E+08	14	7.54286	4.50023
Nc-1-IP_pe	chr1	1.57E+08	1.57E+08	254	1.57E+08	10	4.39571	3.25288
Nc-1-IP_pe	chr1	1.57E+08	1.57E+08	590	1.57E+08	16	8.83629	4.87286
Nc-1-IP_pe	chr1	1.57E+08	1.57E+08	806	1.57E+08	14	3.86727	2.6027
Nc-1-IP_pe	chr1	1.57E+08	1.57E+08	675	1.57E+08	9	2.89249	2.46179
Nc-1-IP_pe	chr1	1.57E+08	1.57E+08	540	1.57E+08	12	4.24064	2.95299
Nc-1-IP_pe	chr1	1.57E+08	1.57E+08	654	1.57E+08	15	6.10627	3.59023
Nc-1-IP_pe	chr1	1.57E+08	1.57E+08	519	1.57E+08	14	5.47279	3.38553
Nc-1-IP_pe	chr1	1.57E+08	1.57E+08	409	1.57E+08	14	5.45987	3.37894
Nc-1-IP_pe	chr1	1.57E+08	1.57E+08	462	1.57E+08	13	5.19892	3.35621
Nc-1-IP_pe	chr1	1.57E+08	1.57E+08	427	1.57E+08	12	4.24064	2.95299
Nc-1-IP_pe	chr1	1.57E+08	1.57E+08	532	1.57E+08	18	7.1522	3.73811
Nc-1-IP_pe	chr1	1.57E+08	1.57E+08	268	1.57E+08	11	4.06403	2.95415
Nc-1-IP_pe	chr1	1.57E+08	1.57E+08	324	1.57E+08	11	4.36477	3.12256
Nc-1-IP_pe	chr1	1.57E+08	1.57E+08	704	1.57E+08	14	5.43414	3.36584
Nc-1-IP_pe	chr1	1.57E+08	1.57E+08	1542	1.57E+08	15	7.75613	4.45384
Nc-1-IP_pe	chr1	1.57E+08	1.57E+08	321	1.57E+08	15	6.39297	3.73508
Nc-1-IP_pe	chr1	1.57E+08	1.57E+08	384	1.57E+08	11	4.06403	2.95415
Nc-1-IP_pe	chr1	1.57E+08	1.57E+08	1476	1.57E+08	17	7.02067	3.79543
Nc-1-IP_pe	chr1	1.57E+08	1.57E+08	308	1.57E+08	13	3.35769	2.42919
Nc-1-IP_pe	chr1	1.57E+08	1.57E+08	348	1.57E+08	15	6.78905	3.93887
Nc-1-IP_pe	chr1	1.57E+08	1.57E+08	290	1.57E+08	13	5.18582	3.34927

Nc-1-IP_pe	chr1	1.57E+08	1.57E+08	452	1.57E+08	13	5.08264	3.29478
Nc-1-IP_pe	chr1	1.57E+08	1.57E+08	466	1.57E+08	17	6.85055	3.71486
Nc-1-IP_pe	chr1	1.57E+08	1.57E+08	787	1.57E+08	15	5.34394	3.21607
Nc-1-IP_pe	chr1	1.57E+08	1.57E+08	304	1.57E+08	16	8.05476	4.45367
Nc-1-IP_pe	chr1	1.57E+08	1.57E+08	702	1.57E+08	14	3.86727	2.6027
Nc-1-IP_pe	chr1	1.57E+08	1.57E+08	293	1.57E+08	10	3.08907	2.49869
Nc-1-IP_pe	chr1	1.57E+08	1.57E+08	587	1.57E+08	13	7.04588	4.38198
Nc-1-IP_pe	chr1	1.57E+08	1.57E+08	878	1.57E+08	16	6.8987	3.86161
Nc-1-IP_pe	chr1	1.57E+08	1.57E+08	1030	1.57E+08	18	7.62523	3.95652
Nc-1-IP_pe	chr1	1.57E+08	1.57E+08	504	1.57E+08	17	5.9911	3.31919
Nc-1-IP_pe	chr1	1.57E+08	1.57E+08	562	1.57E+08	14	5.51541	3.4073
Nc-1-IP_pe	chr1	1.57E+08	1.57E+08	415	1.57E+08	17	6.92131	3.74829
Nc-1-IP_pe	chr1	1.57E+08	1.57E+08	282	1.57E+08	12	4.24064	2.95299
Nc-1-IP_pe	chr1	1.57E+08	1.57E+08	704	1.57E+08	13	5.9487	3.76157
Nc-1-IP_pe	chr1	1.57E+08	1.57E+08	793	1.57E+08	8	3.64414	3.01254
Nc-1-IP_pe	chr1	1.57E+08	1.57E+08	253	1.57E+08	11	6.03439	4.10251
Nc-1-IP_pe	chr1	1.57E+08	1.57E+08	594	1.57E+08	12	4.70082	3.20033
Nc-1-IP_pe	chr1	1.57E+08	1.57E+08	409	1.57E+08	16	5.62031	3.24783
Nc-1-IP_pe	chr1	1.57E+08	1.57E+08	262	1.57E+08	11	4.06403	2.95415
Nc-1-IP_pe	chr1	1.57E+08	1.57E+08	315	1.57E+08	10	3.89064	2.95552
Nc-1-IP_pe	chr1	1.57E+08	1.57E+08	267	1.57E+08	10	3.60969	2.79325
Nc-1-IP_pe	chr1	1.57E+08	1.57E+08	384	1.57E+08	11	3.64839	2.72584
Nc-1-IP_pe	chr1	1.57E+08	1.57E+08	301	1.57E+08	12	5.03305	3.38277
Nc-1-IP_pe	chr1	1.57E+08	1.57E+08	964	1.57E+08	14	6.19157	3.75949
Nc-1-IP_pe	chr1	1.57E+08	1.57E+08	334	1.57E+08	12	3.83688	2.74114
Nc-1-IP_pe	chr1	1.57E+08	1.57E+08	296	1.57E+08	11	3.28475	2.53029
Nc-1-IP_pe	chr1	1.57E+08	1.57E+08	275	1.57E+08	11	2.96397	2.36091
Nc-1-IP_pe	chr1	1.57E+08	1.57E+08	320	1.57E+08	12	4.97854	3.35262
Nc-1-IP_pe	chr1	1.57E+08	1.57E+08	933	1.57E+08	15	6.78905	3.93887
Nc-1-IP_pe	chr1	1.57E+08	1.57E+08	469	1.57E+08	14	5.13767	3.21617
Nc-1-IP_pe	chr1	1.57E+08	1.57E+08	903	1.57E+08	16	5.98198	3.41707
Nc-1-IP_pe	chr1	1.57E+08	1.57E+08	286	1.57E+08	14	4.91783	3.1068
Nc-1-IP_pe	chr1	1.57E+08	1.57E+08	864	1.57E+08	15	6.39297	3.73508
Nc-1-IP_pe	chr1	1.57E+08	1.57E+08	269	1.57E+08	11	4.06403	2.95415
Nc-1-IP_pe	chr1	1.57E+08	1.57E+08	397	1.57E+08	14	5.03085	3.16286
Nc-1-IP_pe	chr1	1.57E+08	1.57E+08	401	1.57E+08	19	6.32622	3.27682
Nc-1-IP_pe	chr1	1.57E+08	1.57E+08	291	1.57E+08	15	6.78905	3.93887
Nc-1-IP_pe	chr1	1.57E+08	1.57E+08	283	1.57E+08	12	5.01934	3.37518
Nc-1-IP_pe	chr1	1.57E+08	1.57E+08	710	1.57E+08	12	5.84943	3.84431
Nc-1-IP_pe	chr1	1.57E+08	1.57E+08	520	1.57E+08	11	6.03439	4.10251
Nc-1-IP_pe	chr1	1.57E+08	1.57E+08	260	1.57E+08	14	7.42849	4.43574
Nc-1-IP_pe	chr1	1.57E+08	1.57E+08	283	1.57E+08	13	4.41954	2.952
Nc-1-IP_pe	chr1	1.57E+08	1.57E+08	271	1.57E+08	10	4.48929	3.30875
Nc-1-IP_pe	chr1	1.57E+08	1.57E+08	338	1.57E+08	12	3.16137	2.39719
Nc-1-IP_pe	chr1	1.57E+08	1.57E+08	621	1.57E+08	12	6.50787	4.22912

Nc-1-IP_pe	chr1	1.57E+08	1.57E+08	265	1.57E+08	11	5.10577	3.5486
Nc-1-IP_pe	chr1	1.57E+08	1.57E+08	543	1.57E+08	16	6.24025	3.54005
Nc-1-IP_pe	chr1	1.57E+08	1.57E+08	290	1.57E+08	11	6.03439	4.10251
Nc-1-IP_pe	chr1	1.57E+08	1.57E+08	294	1.57E+08	14	5.838	3.57373
Nc-1-IP_pe	chr1	1.57E+08	1.57E+08	424	1.57E+08	10	3.89064	2.95552
Nc-1-IP_pe	chr1	1.57E+08	1.57E+08	527	1.57E+08	18	9.07665	4.66107
Nc-1-IP_pe	chr1	1.57E+08	1.57E+08	355	1.57E+08	12	5.89413	3.8701
Nc-1-IP_pe	chr1	1.57E+08	1.57E+08	333	1.57E+08	17	5.9911	3.31919
Nc-1-IP_pe	chr1	1.57E+08	1.57E+08	450	1.57E+08	15	4.7819	2.95039
Nc-1-IP_pe	chr1	1.57E+08	1.57E+08	276	1.57E+08	10	4.48929	3.30875
Nc-1-IP_pe	chr1	1.57E+08	1.57E+08	326	1.57E+08	14	6.78215	4.0774
Nc-1-IP_pe	chr1	1.57E+08	1.57E+08	307	1.57E+08	9	3.66792	2.92434
Nc-1-IP_pe	chr1	1.57E+08	1.57E+08	270	1.57E+08	10	4.91962	3.56846
Nc-1-IP_pe	chr1	1.57E+08	1.57E+08	307	1.57E+08	9	4.11647	3.19921
Nc-1-IP_pe	chr1	1.57E+08	1.57E+08	278	1.57E+08	12	4.39405	3.03475
Nc-1-IP_pe	chr1	1.57E+08	1.57E+08	395	1.57E+08	14	5.03085	3.16286
Nc-1-IP_pe	chr1	1.57E+08	1.57E+08	849	1.57E+08	13	6.72991	4.20021
Nc-1-IP_pe	chr1	1.57E+08	1.57E+08	387	1.57E+08	12	5.23082	3.49289
Nc-1-IP_pe	chr1	1.57E+08	1.57E+08	278	1.57E+08	9	3.64368	2.90963
Nc-1-IP_pe	chr1	1.57E+08	1.57E+08	470	1.57E+08	8	3.74505	3.07688
Nc-1-IP_pe	chr1	1.57E+08	1.57E+08	303	1.57E+08	12	4.70082	3.20033
Nc-1-IP_pe	chr1	1.57E+08	1.57E+08	267	1.57E+08	7	2.9043	2.63038
Nc-1-IP_pe	chr1	1.57E+08	1.57E+08	406	1.57E+08	17	11.4763	6.15376
Nc-1-IP_pe	chr1	1.57E+08	1.57E+08	346	1.57E+08	12	6.86906	4.44438
Nc-1-IP_pe	chr1	1.57E+08	1.57E+08	392	1.57E+08	9	4.46937	3.41876
Nc-1-IP_pe	chr1	1.57E+08	1.57E+08	368	1.57E+08	8	3.56021	2.95918
Nc-1-IP_pe	chr1	1.57E+08	1.57E+08	536	1.57E+08	11	6.03439	4.10251
Nc-1-IP_pe	chr1	1.57E+08	1.57E+08	290	1.57E+08	9	2.89249	2.46179
Nc-1-IP_pe	chr1	1.57E+08	1.57E+08	253	1.57E+08	13	7.19153	4.46655
Nc-1-IP_pe	chr1	1.57E+08	1.57E+08	447	1.57E+08	10	5.10552	3.682
Nc-1-IP_pe	chr1	1.57E+08	1.57E+08	271	1.57E+08	11	3.28475	2.53029
Nc-1-IP_pe	chr1	1.57E+08	1.57E+08	279	1.57E+08	10	4.99888	3.61677
Nc-1-IP_pe	chr1	1.57E+08	1.57E+08	314	1.57E+08	11	6.03439	4.10251
Nc-1-IP_pe	chr1	1.58E+08	1.58E+08	313	1.58E+08	10	5.23357	3.76063
Nc-1-IP_pe	chr1	1.58E+08	1.58E+08	258	1.58E+08	11	6.03439	4.10251
Nc-1-IP_pe	chr1	1.58E+08	1.58E+08	348	1.58E+08	8	3.74505	3.07688
Nc-1-IP_pe	chr1	1.58E+08	1.58E+08	316	1.58E+08	10	5.15807	3.71423
Nc-1-IP_pe	chr1	1.58E+08	1.58E+08	609	1.58E+08	12	6.86906	4.44438
Nc-1-IP_pe	chr1	1.58E+08	1.58E+08	282	1.58E+08	9	3.72192	2.95716
Nc-1-IP_pe	chr1	1.58E+08	1.58E+08	267	1.58E+08	10	4.47495	3.30017
Nc-1-IP_pe	chr1	1.58E+08	1.58E+08	307	1.58E+08	11	4.06403	2.95415
Nc-1-IP_pe	chr1	1.58E+08	1.58E+08	372	1.58E+08	10	3.89064	2.95552
Nc-1-IP_pe	chr1	1.58E+08	1.58E+08	352	1.58E+08	9	3.72192	2.95716
Nc-1-IP_pe	chr1	1.58E+08	1.58E+08	305	1.58E+08	10	4.39571	3.25288
Nc-1-IP_pe	chr1	1.58E+08	1.58E+08	593	1.58E+08	16	9.74731	5.37917

Nc-1-IP_pe	chr1	1.58E+08	1.58E+08	261	1.58E+08	12	5.99895	3.93077
Nc-1-IP_pe	chr1	1.58E+08	1.58E+08	315	1.58E+08	10	4.56202	3.35231
Nc-1-IP_pe	chr1	1.58E+08	1.58E+08	548	1.58E+08	9	3.27326	2.68684
Nc-1-IP_pe	chr1	1.58E+08	1.58E+08	332	1.58E+08	9	4.27829	3.29955
Nc-1-IP_pe	chr1	1.58E+08	1.58E+08	459	1.58E+08	13	7.52042	4.65922
Nc-1-IP_pe	chr1	1.58E+08	1.58E+08	404	1.58E+08	11	4.06403	2.95415
Nc-1-IP_pe	chr1	1.58E+08	1.58E+08	327	1.58E+08	11	4.06403	2.95415
Nc-1-IP_pe	chr1	1.58E+08	1.58E+08	287	1.58E+08	10	3.83201	2.92147
Nc-1-IP_pe	chr1	1.58E+08	1.58E+08	603	1.58E+08	13	5.9487	3.76157
Nc-1-IP_pe	chr1	1.58E+08	1.58E+08	330	1.58E+08	9	3.27326	2.68684
Nc-1-IP_pe	chr1	1.58E+08	1.58E+08	286	1.58E+08	10	3.30648	2.62073
Nc-1-IP_pe	chr1	1.58E+08	1.58E+08	422	1.58E+08	9	4.00426	3.12999
Nc-1-IP_pe	chr1	1.58E+08	1.58E+08	490	1.58E+08	8	3.51085	2.92785
Nc-1-IP_pe	chr1	1.58E+08	1.58E+08	329	1.58E+08	13	7.7352	4.78626
Nc-1-IP_pe	chr1	1.58E+08	1.58E+08	305	1.58E+08	9	4.46937	3.41876
Nc-1-IP_pe	chr1	1.58E+08	1.58E+08	403	1.58E+08	15	7.46994	4.29894
Nc-1-IP_pe	chr1	1.58E+08	1.58E+08	333	1.58E+08	8	3.74505	3.07688
Nc-1-IP_pe	chr1	1.58E+08	1.58E+08	417	1.58E+08	9	3.72192	2.95716
Nc-1-IP_pe	chr1	1.59E+08	1.59E+08	271	1.59E+08	9	4.25966	3.28797
Nc-1-IP_pe	chr1	1.59E+08	1.59E+08	339	1.59E+08	11	6.03439	4.10251
Nc-1-IP_pe	chr1	1.59E+08	1.59E+08	277	1.59E+08	12	6.50787	4.22912
Nc-1-IP_pe	chr1	1.59E+08	1.59E+08	335	1.59E+08	8	3.74505	3.07688
Nc-1-IP_pe	chr1	1.59E+08	1.59E+08	307	1.59E+08	11	6.03439	4.10251
Nc-1-IP_pe	chr1	1.59E+08	1.59E+08	614	1.59E+08	11	5.10577	3.5486
Nc-1-IP_pe	chr1	1.59E+08	1.59E+08	371	1.59E+08	11	6.03439	4.10251
Nc-1-IP_pe	chr1	1.59E+08	1.59E+08	255	1.59E+08	9	4.46937	3.41876
Nc-1-IP_pe	chr1	1.59E+08	1.59E+08	417	1.59E+08	12	5.84943	3.84431
Nc-1-IP_pe	chr1	1.59E+08	1.59E+08	269	1.59E+08	9	3.72192	2.95716
Nc-1-IP_pe	chr1	1.59E+08	1.59E+08	305	1.59E+08	15	5.20142	3.14789
Nc-1-IP_pe	chr1	1.59E+08	1.59E+08	420	1.59E+08	13	5.9487	3.76157
Nc-1-IP_pe	chr1	1.59E+08	1.59E+08	315	1.59E+08	9	4.46937	3.41876
Nc-1-IP_pe	chr1	1.59E+08	1.59E+08	394	1.59E+08	7	3.06442	2.73501
Nc-1-IP_pe	chr1	1.59E+08	1.59E+08	358	1.59E+08	13	7.7352	4.78626
Nc-1-IP_pe	chr1	1.59E+08	1.59E+08	270	1.59E+08	9	3.72192	2.95716
Nc-1-IP_pe	chr1	1.59E+08	1.59E+08	466	1.59E+08	11	5.03835	3.5092
Nc-1-IP_pe	chr1	1.6E+08	1.6E+08	509	1.6E+08	10	4.99888	3.61677
Nc-1-IP_pe	chr1	1.6E+08	1.6E+08	270	1.6E+08	7	3.06442	2.73501
Nc-1-IP_pe	chr1	1.6E+08	1.6E+08	354	1.6E+08	10	3.89064	2.95552
Nc-1-IP_pe	chr1	1.6E+08	1.6E+08	292	1.6E+08	11	4.54404	3.2242
Nc-1-IP_pe	chr1	1.6E+08	1.6E+08	978	1.6E+08	20	9.27144	4.45313
Nc-1-IP_pe	chr1	1.6E+08	1.6E+08	443	1.6E+08	9	3.68012	2.93175
Nc-1-IP_pe	chr1	1.6E+08	1.6E+08	911	1.6E+08	11	5.17791	3.59087
Nc-1-IP_pe	chr1	1.6E+08	1.6E+08	444	1.6E+08	10	4.65155	3.40613
Nc-1-IP_pe	chr1	1.6E+08	1.6E+08	316	1.6E+08	12	5.62759	3.7171
Nc-1-IP_pe	chr1	1.6E+08	1.6E+08	335	1.6E+08	12	3.86684	2.7567

Nc-1-IP_pe	chr1	1.6E+08	1.6E+08	298	1.6E+08	11	5.95085	4.05188
Nc-1-IP_pe	chr1	1.6E+08	1.6E+08	274	1.6E+08	15	7.56326	4.34922
Nc-1-IP_pe	chr1	1.6E+08	1.6E+08	422	1.6E+08	10	3.83201	2.92147
Nc-1-IP_pe	chr1	1.6E+08	1.6E+08	263	1.6E+08	11	3.64839	2.72584
Nc-1-IP_pe	chr1	1.6E+08	1.6E+08	410	1.6E+08	17	8.86434	4.71565
Nc-1-IP_pe	chr1	1.6E+08	1.6E+08	372	1.6E+08	15	5.83691	3.4562
Nc-1-IP_pe	chr1	1.6E+08	1.6E+08	374	1.6E+08	14	5.30793	3.30182
Nc-1-IP_pe	chr1	1.6E+08	1.6E+08	790	1.6E+08	15	5.70822	3.39286
Nc-1-IP_pe	chr1	1.6E+08	1.6E+08	465	1.6E+08	11	4.47871	3.18705
Nc-1-IP_pe	chr1	1.6E+08	1.6E+08	266	1.6E+08	12	5.8598	3.85029
Nc-1-IP_pe	chr1	1.6E+08	1.6E+08	263	1.6E+08	10	3.89064	2.95552
Nc-1-IP_pe	chr1	1.6E+08	1.6E+08	317	1.6E+08	9	3.27326	2.68684
Nc-1-IP_pe	chr1	1.6E+08	1.6E+08	458	1.6E+08	10	3.92601	2.97611
Nc-1-IP_pe	chr1	1.6E+08	1.6E+08	529	1.6E+08	11	3.28475	2.53029
Nc-1-IP_pe	chr1	1.6E+08	1.6E+08	350	1.6E+08	9	3.32589	2.71826
Nc-1-IP_pe	chr1	1.6E+08	1.6E+08	256	1.6E+08	11	5.10577	3.5486
Nc-1-IP_pe	chr1	1.6E+08	1.6E+08	331	1.6E+08	8	3.74178	3.0748
Nc-1-IP_pe	chr1	1.6E+08	1.6E+08	269	1.6E+08	14	8.29543	4.93196
Nc-1-IP_pe	chr1	1.6E+08	1.6E+08	552	1.6E+08	13	5.15976	3.33548
Nc-1-IP_pe	chr1	1.6E+08	1.6E+08	1078	1.6E+08	8	2.93592	2.56703
Nc-1-IP_pe	chr1	1.6E+08	1.6E+08	308	1.6E+08	11	5.17791	3.59087
Nc-1-IP_pe	chr1	1.6E+08	1.6E+08	536	1.6E+08	10	4.39571	3.25288
Nc-1-IP_pe	chr1	1.6E+08	1.6E+08	431	1.6E+08	12	6.10662	3.99337
Nc-1-IP_pe	chr1	1.6E+08	1.6E+08	423	1.6E+08	14	6.61011	3.98382
Nc-1-IP_pe	chr1	1.6E+08	1.6E+08	584	1.6E+08	16	5.85814	3.35873
Nc-1-IP_pe	chr1	1.6E+08	1.6E+08	319	1.6E+08	11	4.58343	3.24666
Nc-1-IP_pe	chr1	1.6E+08	1.6E+08	668	1.6E+08	14	5.03085	3.16286
Nc-1-IP_pe	chr1	1.6E+08	1.6E+08	537	1.6E+08	14	6.13058	3.7272
Nc-1-IP_pe	chr1	1.6E+08	1.6E+08	343	1.6E+08	10	4.20113	3.13749
Nc-1-IP_pe	chr1	1.6E+08	1.6E+08	274	1.6E+08	14	7.42849	4.43574
Nc-1-IP_pe	chr1	1.6E+08	1.6E+08	291	1.6E+08	12	4.24064	2.95299
Nc-1-IP_pe	chr1	1.6E+08	1.6E+08	383	1.6E+08	12	4.70082	3.20033
Nc-1-IP_pe	chr1	1.6E+08	1.6E+08	277	1.6E+08	12	5.67619	3.74486
Nc-1-IP_pe	chr1	1.6E+08	1.6E+08	433	1.6E+08	15	7.83541	4.49711
Nc-1-IP_pe	chr1	1.6E+08	1.6E+08	376	1.6E+08	14	7.14701	4.27839
Nc-1-IP_pe	chr1	1.6E+08	1.6E+08	336	1.6E+08	17	7.02067	3.79543
Nc-1-IP_pe	chr1	1.6E+08	1.6E+08	280	1.6E+08	14	5.03085	3.16286
Nc-1-IP_pe	chr1	1.6E+08	1.6E+08	570	1.6E+08	13	4.86362	3.18015
Nc-1-IP_pe	chr1	1.6E+08	1.6E+08	392	1.6E+08	14	8.29543	4.93196
Nc-1-IP_pe	chr1	1.6E+08	1.6E+08	329	1.6E+08	9	3.72192	2.95716
Nc-1-IP_pe	chr1	1.6E+08	1.6E+08	270	1.6E+08	12	6.58417	4.27436
Nc-1-IP_pe	chr1	1.6E+08	1.6E+08	330	1.6E+08	9	4.4496	3.40639
Nc-1-IP_pe	chr1	1.6E+08	1.6E+08	825	1.6E+08	13	4.86362	3.18015
Nc-1-IP_pe	chr1	1.6E+08	1.6E+08	348	1.6E+08	11	5.06889	3.52703
Nc-1-IP_pe	chr1	1.6E+08	1.6E+08	345	1.6E+08	10	4.40419	3.25794

Nc-1-IP_pe	chr1	1.6E+08	1.6E+08	256	1.6E+08	12	5.15877	3.45264
Nc-1-IP_pe	chr1	1.6E+08	1.6E+08	455	1.6E+08	11	4.73303	3.33236
Nc-1-IP_pe	chr1	1.6E+08	1.6E+08	445	1.6E+08	14	5.51541	3.4073
Nc-1-IP_pe	chr1	1.6E+08	1.6E+08	306	1.6E+08	12	5.54793	3.67174
Nc-1-IP_pe	chr1	1.6E+08	1.6E+08	346	1.6E+08	14	6.61011	3.98382
Nc-1-IP_pe	chr1	1.6E+08	1.6E+08	312	1.6E+08	10	5.23357	3.76063
Nc-1-IP_pe	chr1	1.6E+08	1.6E+08	339	1.6E+08	10	5.23357	3.76063
Nc-1-IP_pe	chr1	1.6E+08	1.6E+08	595	1.6E+08	15	6.19428	3.63445
Nc-1-IP_pe	chr1	1.6E+08	1.6E+08	450	1.6E+08	18	9.94172	5.10499
Nc-1-IP_pe	chr1	1.6E+08	1.6E+08	461	1.6E+08	12	4.70082	3.20033
Nc-1-IP_pe	chr1	1.6E+08	1.6E+08	335	1.6E+08	10	4.68196	3.42446
Nc-1-IP_pe	chr1	1.6E+08	1.6E+08	725	1.6E+08	12	4.63088	3.16234
Nc-1-IP_pe	chr1	1.6E+08	1.6E+08	267	1.6E+08	7	3.03708	2.71711
Nc-1-IP_pe	chr1	1.6E+08	1.6E+08	295	1.6E+08	11	5.06889	3.52703
Nc-1-IP_pe	chr1	1.6E+08	1.6E+08	373	1.6E+08	11	4.87504	3.4143
Nc-1-IP_pe	chr1	1.6E+08	1.6E+08	477	1.6E+08	17	9.24487	4.91597
Nc-1-IP_pe	chr1	1.6E+08	1.6E+08	436	1.6E+08	12	3.47963	2.55766
Nc-1-IP_pe	chr1	1.6E+08	1.6E+08	273	1.6E+08	12	5.84943	3.84431
Nc-1-IP_pe	chr1	1.6E+08	1.6E+08	658	1.6E+08	11	5.97044	4.06374
Nc-1-IP_pe	chr1	1.6E+08	1.6E+08	681	1.6E+08	10	4.39571	3.25288
Nc-1-IP_pe	chr1	1.61E+08	1.61E+08	293	1.61E+08	10	5.23357	3.76063
Nc-1-IP_pe	chr1	1.61E+08	1.61E+08	345	1.61E+08	12	5.45448	3.61875
Nc-1-IP_pe	chr1	1.61E+08	1.61E+08	333	1.61E+08	8	3.61691	2.99521
Nc-1-IP_pe	chr1	1.61E+08	1.61E+08	386	1.61E+08	12	5.23082	3.49289
Nc-1-IP_pe	chr1	1.61E+08	1.61E+08	443	1.61E+08	10	5.23357	3.76063
Nc-1-IP_pe	chr1	1.61E+08	1.61E+08	663	1.61E+08	12	5.23082	3.49289
Nc-1-IP_pe	chr1	1.61E+08	1.61E+08	279	1.61E+08	10	3.71867	2.85592
Nc-1-IP_pe	chr1	1.61E+08	1.61E+08	686	1.61E+08	18	9.94172	5.10499
Nc-1-IP_pe	chr1	1.61E+08	1.61E+08	262	1.61E+08	11	4.74697	3.34038
Nc-1-IP_pe	chr1	1.61E+08	1.61E+08	355	1.61E+08	14	8.63073	5.12814
Nc-1-IP_pe	chr1	1.61E+08	1.61E+08	450	1.61E+08	9	4.46937	3.41876
Nc-1-IP_pe	chr1	1.61E+08	1.61E+08	257	1.61E+08	10	3.89064	2.95552
Nc-1-IP_pe	chr1	1.61E+08	1.61E+08	403	1.61E+08	9	4.46937	3.41876
Nc-1-IP_pe	chr1	1.61E+08	1.61E+08	289	1.61E+08	9	3.43499	2.78365
Nc-1-IP_pe	chr1	1.61E+08	1.61E+08	332	1.61E+08	11	4.54404	3.2242
Nc-1-IP_pe	chr1	1.61E+08	1.61E+08	269	1.61E+08	13	6.54342	4.09407
Nc-1-IP_pe	chr1	1.61E+08	1.61E+08	360	1.61E+08	11	3.49235	2.64145
Nc-1-IP_pe	chr1	1.61E+08	1.61E+08	310	1.61E+08	14	6.06519	3.69269
Nc-1-IP_pe	chr1	1.61E+08	1.61E+08	408	1.61E+08	14	7.14701	4.27839
Nc-1-IP_pe	chr1	1.61E+08	1.61E+08	543	1.61E+08	11	6.03439	4.10251
Nc-1-IP_pe	chr1	1.61E+08	1.61E+08	303	1.61E+08	10	4.77495	3.48064
Nc-1-IP_pe	chr1	1.61E+08	1.61E+08	282	1.61E+08	9	4.24819	3.28084
Nc-1-IP_pe	chr1	1.61E+08	1.61E+08	278	1.61E+08	14	5.03085	3.16286
Nc-1-IP_pe	chr1	1.61E+08	1.61E+08	349	1.61E+08	10	3.08907	2.49869
Nc-1-IP_pe	chr1	1.61E+08	1.61E+08	457	1.61E+08	14	5.51541	3.4073

Nc-1-IP_pe chr1	1.61E+08	1.61E+08	403	1.61E+08	19	7.29128	3.68799
Nc-1-IP_pe chr1	1.61E+08	1.61E+08	440	1.61E+08	14	7.14701	4.27839
Nc-1-IP_pe chr1	1.61E+08	1.61E+08	271	1.61E+08	10	4.0855	3.0694
Nc-1-IP_pe chr1	1.61E+08	1.61E+08	349	1.61E+08	12	5.65992	3.73556
Nc-1-IP_pe chr1	1.61E+08	1.61E+08	422	1.61E+08	10	3.30648	2.62073
Nc-1-IP_pe chr1	1.61E+08	1.61E+08	269	1.61E+08	10	4.39571	3.25288
Nc-1-IP_pe chr1	1.61E+08	1.61E+08	321	1.61E+08	14	4.21449	2.76599
Nc-1-IP_pe chr1	1.61E+08	1.61E+08	1277	1.61E+08	15	6.19428	3.63445
Nc-1-IP_pe chr1	1.61E+08	1.61E+08	777	1.61E+08	11	3.49235	2.64145
Nc-1-IP_pe chr1	1.61E+08	1.61E+08	260	1.61E+08	15	6.78905	3.93887
Nc-1-IP_pe chr1	1.61E+08	1.61E+08	272	1.61E+08	13	5.6432	3.59449
Nc-1-IP_pe chr1	1.61E+08	1.61E+08	305	1.61E+08	18	7.93853	4.10423
Nc-1-IP_pe chr1	1.61E+08	1.61E+08	345	1.61E+08	15	3.74763	2.48304
Nc-1-IP_pe chr1	1.61E+08	1.61E+08	715	1.61E+08	11	5.63263	3.8604
Nc-1-IP_pe chr1	1.61E+08	1.61E+08	340	1.61E+08	12	6.79895	4.40238
Nc-1-IP_pe chr1	1.61E+08	1.61E+08	412	1.61E+08	13	6.62436	4.14003
Nc-1-IP_pe chr1	1.61E+08	1.61E+08	725	1.61E+08	13	4.41954	2.952
Nc-1-IP_pe chr1	1.61E+08	1.61E+08	661	1.61E+08	15	6.10627	3.59023
Nc-1-IP_pe chr1	1.61E+08	1.61E+08	336	1.61E+08	12	4.63088	3.16234
Nc-1-IP_pe chr1	1.61E+08	1.61E+08	360	1.61E+08	14	7.32104	4.37545
Nc-1-IP_pe chr1	1.61E+08	1.61E+08	429	1.61E+08	12	3.47963	2.55766
Nc-1-IP_pe chr1	1.61E+08	1.61E+08	292	1.61E+08	15	5.70822	3.39286
Nc-1-IP_pe chr1	1.61E+08	1.61E+08	595	1.61E+08	15	6.78905	3.93887
Nc-1-IP_pe chr1	1.61E+08	1.61E+08	1355	1.61E+08	17	7.02067	3.79543
Nc-1-IP_pe chr1	1.61E+08	1.61E+08	269	1.61E+08	10	4.51818	3.32604
Nc-1-IP_pe chr1	1.61E+08	1.61E+08	254	1.61E+08	11	4.06403	2.95415
Nc-1-IP_pe chr1	1.61E+08	1.61E+08	360	1.61E+08	14	6.11547	3.71921
Nc-1-IP_pe chr1	1.61E+08	1.61E+08	264	1.61E+08	9	3.27326	2.68684
Nc-1-IP_pe chr1	1.61E+08	1.61E+08	526	1.61E+08	14	7.75387	4.62
Nc-1-IP_pe chr1	1.61E+08	1.61E+08	646	1.61E+08	11	5.10577	3.5486
Nc-1-IP_pe chr1	1.61E+08	1.61E+08	450	1.61E+08	13	5.9487	3.76157
Nc-1-IP_pe chr1	1.61E+08	1.61E+08	628	1.61E+08	13	7.12853	4.42991
Nc-1-IP_pe chr1	1.61E+08	1.61E+08	291	1.61E+08	10	3.08907	2.49869
Nc-1-IP_pe chr1	1.61E+08	1.61E+08	255	1.61E+08	8	3.74505	3.07688
Nc-1-IP_pe chr1	1.61E+08	1.61E+08	505	1.61E+08	8	3.74505	3.07688
Nc-1-IP_pe chr1	1.61E+08	1.61E+08	253	1.61E+08	8	3.74505	3.07688
Nc-1-IP_pe chr1	1.61E+08	1.61E+08	300	1.61E+08	10	3.4604	2.70797
Nc-1-IP_pe chr1	1.61E+08	1.61E+08	266	1.61E+08	9	4.46937	3.41876
Nc-1-IP_pe chr1	1.61E+08	1.61E+08	274	1.61E+08	8	3.51085	2.92785
Nc-1-IP_pe chr1	1.61E+08	1.61E+08	306	1.61E+08	17	9.99923	5.32289
Nc-1-IP_pe chr1	1.61E+08	1.61E+08	380	1.61E+08	17	11.4763	6.15376
Nc-1-IP_pe chr1	1.61E+08	1.61E+08	401	1.61E+08	13	5.9487	3.76157
Nc-1-IP_pe chr1	1.61E+08	1.61E+08	1395	1.61E+08	16	6.8987	3.86161
Nc-1-IP_pe chr1	1.61E+08	1.61E+08	2567	1.61E+08	80	17.41	3.01591
Nc-1-IP_pe chr1	1.61E+08	1.61E+08	586	1.61E+08	57	14.3545	3.24134

Nc-1-IP_pe	chr1	1.61E+08	1.61E+08	1115	1.61E+08	82	8.17859	1.96832
Nc-1-IP_pe	chr1	1.61E+08	1.61E+08	1515	1.61E+08	81	18.24	3.09232
Nc-1-IP_pe	chr1	1.61E+08	1.61E+08	4439	1.61E+08	79	18.5502	3.1801
Nc-1-IP_pe	chr1	1.61E+08	1.61E+08	361	1.61E+08	42	3.98634	1.80706
Nc-1-IP_pe	chr1	1.61E+08	1.61E+08	1441	1.61E+08	105	31.8988	3.99738
Nc-1-IP_pe	chr1	1.61E+08	1.61E+08	4283	1.61E+08	80	25.4914	4.07417
Nc-1-IP_pe	chr1	1.61E+08	1.61E+08	1567	1.61E+08	81	16.6676	2.90589
Nc-1-IP_pe	chr1	1.61E+08	1.61E+08	5186	1.61E+08	87	18.3803	2.97504
Nc-1-IP_pe	chr1	1.61E+08	1.61E+08	4387	1.61E+08	104	29.9284	3.81291
Nc-1-IP_pe	chr1	1.61E+08	1.61E+08	424	1.61E+08	9	3.12089	2.59629
Nc-1-IP_pe	chr1	1.62E+08	1.62E+08	533	1.62E+08	10	3.08907	2.49869
Nc-1-IP_pe	chr1	1.62E+08	1.62E+08	578	1.62E+08	10	4.39571	3.25288
Nc-1-IP_pe	chr1	1.62E+08	1.62E+08	309	1.62E+08	18	8.37884	4.31591
Nc-1-IP_pe	chr1	1.62E+08	1.62E+08	306	1.62E+08	13	3.07238	2.29378
Nc-1-IP_pe	chr1	1.62E+08	1.62E+08	290	1.62E+08	19	7.29128	3.68799
Nc-1-IP_pe	chr1	1.62E+08	1.62E+08	373	1.62E+08	19	8.98281	4.46183
Nc-1-IP_pe	chr1	1.62E+08	1.62E+08	422	1.62E+08	15	5.94269	3.50859
Nc-1-IP_pe	chr1	1.62E+08	1.62E+08	254	1.62E+08	17	7.54182	4.04685
Nc-1-IP_pe	chr1	1.62E+08	1.62E+08	472	1.62E+08	18	4.6358	2.66698
Nc-1-IP_pe	chr1	1.62E+08	1.62E+08	1289	1.62E+08	16	3.94146	2.50592
Nc-1-IP_pe	chr1	1.62E+08	1.62E+08	256	1.62E+08	13	4.45253	2.96876
Nc-1-IP_pe	chr1	1.62E+08	1.62E+08	257	1.62E+08	15	2.95965	2.14351
Nc-1-IP_pe	chr1	1.62E+08	1.62E+08	428	1.62E+08	14	3.26761	2.32785
Nc-1-IP_pe	chr1	1.62E+08	1.62E+08	308	1.62E+08	19	10.258	5.08981
Nc-1-IP_pe	chr1	1.62E+08	1.62E+08	342	1.62E+08	18	6.63038	3.50359
Nc-1-IP_pe	chr1	1.62E+08	1.62E+08	356	1.62E+08	20	6.49719	3.259
Nc-1-IP_pe	chr1	1.62E+08	1.62E+08	441	1.62E+08	12	5.84943	3.84431
Nc-1-IP_pe	chr1	1.62E+08	1.62E+08	313	1.62E+08	10	4.39571	3.25288
Nc-1-IP_pe	chr1	1.62E+08	1.62E+08	1074	1.62E+08	17	5.14803	2.94914
Nc-1-IP_pe	chr1	1.62E+08	1.62E+08	319	1.62E+08	12	6.58417	4.27436
Nc-1-IP_pe	chr1	1.62E+08	1.62E+08	260	1.62E+08	13	5.9487	3.76157
Nc-1-IP_pe	chr1	1.62E+08	1.62E+08	318	1.62E+08	8	3.38431	2.8478
Nc-1-IP_pe	chr1	1.62E+08	1.62E+08	295	1.62E+08	11	5.72344	3.91481
Nc-1-IP_pe	chr1	1.62E+08	1.62E+08	670	1.62E+08	12	6.0167	3.94107
Nc-1-IP_pe	chr1	1.62E+08	1.62E+08	255	1.62E+08	8	3.65786	3.02128
Nc-1-IP_pe	chr1	1.62E+08	1.62E+08	539	1.62E+08	14	7.54286	4.50023
Nc-1-IP_pe	chr1	1.62E+08	1.62E+08	287	1.62E+08	11	4.54404	3.2242
Nc-1-IP_pe	chr1	1.62E+08	1.62E+08	314	1.62E+08	9	4.1889	3.24405
Nc-1-IP_pe	chr1	1.62E+08	1.62E+08	404	1.62E+08	12	3.16137	2.39719
Nc-1-IP_pe	chr1	1.62E+08	1.62E+08	328	1.62E+08	10	5.12294	3.69268
Nc-1-IP_pe	chr1	1.62E+08	1.62E+08	778	1.62E+08	11	6.03439	4.10251
Nc-1-IP_pe	chr1	1.62E+08	1.62E+08	590	1.62E+08	13	5.71736	3.63481
Nc-1-IP_pe	chr1	1.62E+08	1.62E+08	1281	1.62E+08	16	6.8987	3.86161
Nc-1-IP_pe	chr1	1.62E+08	1.62E+08	336	1.62E+08	10	4.77495	3.48064
Nc-1-IP_pe	chr1	1.62E+08	1.62E+08	317	1.62E+08	12	6.58417	4.27436

Nc-1-IP_pe	chr1	1.62E+08	1.62E+08	370	1.62E+08	15	9.55381	5.47001
Nc-1-IP_pe	chr1	1.62E+08	1.62E+08	510	1.62E+08	10	4.62142	3.388
Nc-1-IP_pe	chr1	1.62E+08	1.62E+08	513	1.62E+08	10	5.10552	3.682
Nc-1-IP_pe	chr1	1.62E+08	1.62E+08	263	1.62E+08	12	3.83688	2.74114
Nc-1-IP_pe	chr1	1.62E+08	1.62E+08	520	1.62E+08	16	9.24948	5.10026
Nc-1-IP_pe	chr1	1.62E+08	1.62E+08	284	1.62E+08	10	4.62142	3.388
Nc-1-IP_pe	chr1	1.62E+08	1.62E+08	297	1.62E+08	13	7.56612	4.68618
Nc-1-IP_pe	chr1	1.62E+08	1.62E+08	402	1.62E+08	10	4.99888	3.61677
Nc-1-IP_pe	chr1	1.62E+08	1.62E+08	266	1.62E+08	7	3.06442	2.73501
Nc-1-IP_pe	chr1	1.62E+08	1.62E+08	295	1.62E+08	11	5.3563	3.696
Nc-1-IP_pe	chr1	1.62E+08	1.62E+08	278	1.62E+08	11	5.52654	3.79706
Nc-1-IP_pe	chr1	1.62E+08	1.62E+08	363	1.62E+08	13	6.86537	4.27785
Nc-1-IP_pe	chr1	1.62E+08	1.62E+08	475	1.62E+08	13	4.67336	3.0817
Nc-1-IP_pe	chr1	1.62E+08	1.62E+08	275	1.62E+08	10	4.39571	3.25288
Nc-1-IP_pe	chr1	1.62E+08	1.62E+08	370	1.62E+08	14	5.03085	3.16286
Nc-1-IP_pe	chr1	1.62E+08	1.62E+08	383	1.62E+08	11	5.7051	3.90381
Nc-1-IP_pe	chr1	1.62E+08	1.62E+08	738	1.62E+08	13	3.67377	2.58159
Nc-1-IP_pe	chr1	1.62E+08	1.62E+08	367	1.62E+08	27	10.0045	3.93029
Nc-1-IP_pe	chr1	1.63E+08	1.63E+08	364	1.63E+08	9	3.72192	2.95716
Nc-1-IP_pe	chr1	1.63E+08	1.63E+08	516	1.63E+08	18	8.44412	4.3477
Nc-1-IP_pe	chr1	1.63E+08	1.63E+08	331	1.63E+08	10	4.99888	3.61677
Nc-1-IP_pe	chr1	1.63E+08	1.63E+08	398	1.63E+08	15	8.26005	4.73146
Nc-1-IP_pe	chr1	1.63E+08	1.63E+08	356	1.63E+08	9	3.72192	2.95716
Nc-1-IP_pe	chr1	1.63E+08	1.63E+08	361	1.63E+08	12	5.23082	3.49289
Nc-1-IP_pe	chr1	1.63E+08	1.63E+08	335	1.63E+08	13	6.54342	4.09407
Nc-1-IP_pe	chr1	1.63E+08	1.63E+08	343	1.63E+08	12	6.71369	4.35145
Nc-1-IP_pe	chr1	1.63E+08	1.63E+08	263	1.63E+08	10	5.23357	3.76063
Nc-1-IP_pe	chr1	1.63E+08	1.63E+08	303	1.63E+08	8	3.56021	2.95918
Nc-1-IP_pe	chr1	1.63E+08	1.63E+08	268	1.63E+08	9	3.27326	2.68684
Nc-1-IP_pe	chr1	1.63E+08	1.63E+08	418	1.63E+08	10	4.99888	3.61677
Nc-1-IP_pe	chr1	1.63E+08	1.63E+08	271	1.63E+08	11	6.03439	4.10251
Nc-1-IP_pe	chr1	1.63E+08	1.63E+08	454	1.63E+08	14	5.03085	3.16286
Nc-1-IP_pe	chr1	1.63E+08	1.63E+08	257	1.63E+08	15	9.19325	5.26076
Nc-1-IP_pe	chr1	1.64E+08	1.64E+08	397	1.64E+08	11	4.54404	3.2242
Nc-1-IP_pe	chr1	1.64E+08	1.64E+08	273	1.64E+08	10	5.23357	3.76063
Nc-1-IP_pe	chr1	1.64E+08	1.64E+08	508	1.64E+08	14	8.63073	5.12814
Nc-1-IP_pe	chr1	1.64E+08	1.64E+08	258	1.64E+08	11	4.54404	3.2242
Nc-1-IP_pe	chr1	1.64E+08	1.64E+08	468	1.64E+08	16	8.26996	4.56762
Nc-1-IP_pe	chr1	1.64E+08	1.64E+08	581	1.64E+08	14	8.63073	5.12814
Nc-1-IP_pe	chr1	1.64E+08	1.64E+08	343	1.64E+08	15	8.26005	4.73146
Nc-1-IP_pe	chr1	1.64E+08	1.64E+08	274	1.64E+08	10	4.07292	3.06202
Nc-1-IP_pe	chr1	1.64E+08	1.64E+08	641	1.64E+08	10	4.39571	3.25288
Nc-1-IP_pe	chr1	1.64E+08	1.64E+08	299	1.64E+08	9	4.46937	3.41876
Nc-1-IP_pe	chr1	1.64E+08	1.64E+08	379	1.64E+08	14	8.63073	5.12814
Nc-1-IP_pe	chr1	1.64E+08	1.64E+08	734	1.64E+08	15	7.46994	4.29894

Nc-1-IP_pe	chr1	1.64E+08	1.64E+08	291	1.64E+08	12	5.84943	3.84431
Nc-1-IP_pe	chr1	1.65E+08	1.65E+08	256	1.65E+08	9	4.46937	3.41876
Nc-1-IP_pe	chr1	1.65E+08	1.65E+08	352	1.65E+08	11	4.06403	2.95415
Nc-1-IP_pe	chr1	1.65E+08	1.65E+08	425	1.65E+08	13	4.86362	3.18015
Nc-1-IP_pe	chr1	1.65E+08	1.65E+08	275	1.65E+08	10	4.99888	3.61677
Nc-1-IP_pe	chr1	1.65E+08	1.65E+08	263	1.65E+08	13	7.42513	4.60316
Nc-1-IP_pe	chr1	1.65E+08	1.65E+08	295	1.65E+08	9	3.65577	2.91697
Nc-1-IP_pe	chr1	1.65E+08	1.65E+08	275	1.65E+08	12	5.23082	3.49289
Nc-1-IP_pe	chr1	1.65E+08	1.65E+08	328	1.65E+08	10	3.4604	2.70797
Nc-1-IP_pe	chr1	1.65E+08	1.65E+08	299	1.65E+08	11	5.89269	4.01672
Nc-1-IP_pe	chr1	1.65E+08	1.65E+08	454	1.65E+08	11	5.89269	4.01672
Nc-1-IP_pe	chr1	1.65E+08	1.65E+08	611	1.65E+08	10	4.93609	3.57849
Nc-1-IP_pe	chr1	1.65E+08	1.65E+08	255	1.65E+08	7	3.06442	2.73501
Nc-1-IP_pe	chr1	1.65E+08	1.65E+08	438	1.65E+08	11	5.10577	3.5486
Nc-1-IP_pe	chr1	1.65E+08	1.65E+08	266	1.65E+08	10	4.99888	3.61677
Nc-1-IP_pe	chr1	1.65E+08	1.65E+08	280	1.65E+08	9	4.46937	3.41876
Nc-1-IP_pe	chr1	1.65E+08	1.65E+08	342	1.65E+08	9	4.46937	3.41876
Nc-1-IP_pe	chr1	1.65E+08	1.65E+08	253	1.65E+08	9	4.46937	3.41876
Nc-1-IP_pe	chr1	1.65E+08	1.65E+08	577	1.65E+08	10	3.89064	2.95552
Nc-1-IP_pe	chr1	1.65E+08	1.65E+08	893	1.65E+08	9	4.46937	3.41876
Nc-1-IP_pe	chr1	1.65E+08	1.65E+08	443	1.65E+08	10	5.23357	3.76063
Nc-1-IP_pe	chr1	1.65E+08	1.65E+08	391	1.65E+08	10	3.94999	2.99009
Nc-1-IP_pe	chr1	1.65E+08	1.65E+08	385	1.65E+08	11	6.03439	4.10251
Nc-1-IP_pe	chr1	1.65E+08	1.65E+08	350	1.65E+08	10	4.99888	3.61677
Nc-1-IP_pe	chr1	1.65E+08	1.65E+08	268	1.65E+08	8	3.38431	2.8478
Nc-1-IP_pe	chr1	1.66E+08	1.66E+08	313	1.66E+08	7	3.06442	2.73501
Nc-1-IP_pe	chr1	1.66E+08	1.66E+08	443	1.66E+08	10	5.23357	3.76063
Nc-1-IP_pe	chr1	1.66E+08	1.66E+08	380	1.66E+08	11	6.03439	4.10251
Nc-1-IP_pe	chr1	1.66E+08	1.66E+08	428	1.66E+08	11	4.54404	3.2242
Nc-1-IP_pe	chr1	1.66E+08	1.66E+08	413	1.66E+08	12	6.86906	4.44438
Nc-1-IP_pe	chr1	1.66E+08	1.66E+08	374	1.66E+08	12	3.96559	2.80816
Nc-1-IP_pe	chr1	1.66E+08	1.66E+08	477	1.66E+08	12	4.70082	3.20033
Nc-1-IP_pe	chr1	1.66E+08	1.66E+08	267	1.66E+08	9	4.25966	3.28797
Nc-1-IP_pe	chr1	1.66E+08	1.66E+08	451	1.66E+08	9	3.72192	2.95716
Nc-1-IP_pe	chr1	1.66E+08	1.66E+08	308	1.66E+08	11	4.54404	3.2242
Nc-1-IP_pe	chr1	1.66E+08	1.66E+08	322	1.66E+08	11	5.63263	3.8604
Nc-1-IP_pe	chr1	1.66E+08	1.66E+08	410	1.66E+08	14	8.63073	5.12814
Nc-1-IP_pe	chr1	1.66E+08	1.66E+08	416	1.66E+08	9	4.25966	3.28797
Nc-1-IP_pe	chr1	1.66E+08	1.66E+08	321	1.66E+08	16	5.50606	3.19508
Nc-1-IP_pe	chr1	1.66E+08	1.66E+08	489	1.66E+08	12	5.84943	3.84431
Nc-1-IP_pe	chr1	1.66E+08	1.66E+08	335	1.66E+08	9	4.46937	3.41876
Nc-1-IP_pe	chr1	1.66E+08	1.66E+08	273	1.66E+08	11	5.77463	3.94557
Nc-1-IP_pe	chr1	1.66E+08	1.66E+08	604	1.66E+08	12	5.9637	3.91034
Nc-1-IP_pe	chr1	1.66E+08	1.66E+08	355	1.66E+08	11	4.54404	3.2242
Nc-1-IP_pe	chr1	1.66E+08	1.66E+08	428	1.66E+08	10	4.99888	3.61677

Nc-1-IP_pe	chr1	1.66E+08	1.66E+08	336	1.66E+08	13	6.62436	4.14003
Nc-1-IP_pe	chr1	1.66E+08	1.66E+08	280	1.66E+08	11	3.64839	2.72584
Nc-1-IP_pe	chr1	1.66E+08	1.66E+08	271	1.66E+08	11	4.54404	3.2242
Nc-1-IP_pe	chr1	1.66E+08	1.66E+08	302	1.66E+08	11	6.03439	4.10251
Nc-1-IP_pe	chr1	1.66E+08	1.66E+08	383	1.66E+08	11	5.77463	3.94557
Nc-1-IP_pe	chr1	1.66E+08	1.66E+08	295	1.66E+08	10	5.23357	3.76063
Nc-1-IP_pe	chr1	1.66E+08	1.66E+08	458	1.66E+08	9	4.46937	3.41876
Nc-1-IP_pe	chr1	1.66E+08	1.66E+08	319	1.66E+08	8	3.08755	2.66145
Nc-1-IP_pe	chr1	1.66E+08	1.66E+08	258	1.66E+08	11	5.7051	3.90381
Nc-1-IP_pe	chr1	1.66E+08	1.66E+08	460	1.66E+08	9	4.25966	3.28797
Nc-1-IP_pe	chr1	1.67E+08	1.67E+08	789	1.67E+08	9	4.46937	3.41876
Nc-1-IP_pe	chr1	1.67E+08	1.67E+08	415	1.67E+08	13	7.7352	4.78626
Nc-1-IP_pe	chr1	1.67E+08	1.67E+08	276	1.67E+08	13	5.71736	3.63481
Nc-1-IP_pe	chr1	1.67E+08	1.67E+08	421	1.67E+08	12	5.23082	3.49289
Nc-1-IP_pe	chr1	1.67E+08	1.67E+08	561	1.67E+08	10	5.23357	3.76063
Nc-1-IP_pe	chr1	1.67E+08	1.67E+08	752	1.67E+08	12	6.86906	4.44438
Nc-1-IP_pe	chr1	1.67E+08	1.67E+08	265	1.67E+08	9	4.46937	3.41876
Nc-1-IP_pe	chr1	1.67E+08	1.67E+08	383	1.67E+08	14	7.42849	4.43574
Nc-1-IP_pe	chr1	1.67E+08	1.67E+08	389	1.67E+08	12	6.86906	4.44438
Nc-1-IP_pe	chr1	1.67E+08	1.67E+08	280	1.67E+08	11	3.64839	2.72584
Nc-1-IP_pe	chr1	1.67E+08	1.67E+08	604	1.67E+08	15	9.55381	5.47001
Nc-1-IP_pe	chr1	1.67E+08	1.67E+08	285	1.67E+08	13	5.86991	3.71823
Nc-1-IP_pe	chr1	1.67E+08	1.67E+08	585	1.67E+08	12	3.16137	2.39719
Nc-1-IP_pe	chr1	1.67E+08	1.67E+08	320	1.67E+08	14	7.90726	4.70771
Nc-1-IP_pe	chr1	1.67E+08	1.67E+08	824	1.67E+08	12	5.77527	3.80164
Nc-1-IP_pe	chr1	1.67E+08	1.67E+08	302	1.67E+08	11	5.3563	3.696
Nc-1-IP_pe	chr1	1.67E+08	1.67E+08	615	1.67E+08	14	6.06519	3.69269
Nc-1-IP_pe	chr1	1.67E+08	1.67E+08	446	1.67E+08	13	6.90485	4.30056
Nc-1-IP_pe	chr1	1.67E+08	1.67E+08	298	1.67E+08	7	3.06442	2.73501
Nc-1-IP_pe	chr1	1.67E+08	1.67E+08	379	1.67E+08	11	3.89191	2.85898
Nc-1-IP_pe	chr1	1.67E+08	1.67E+08	524	1.67E+08	14	7.34079	4.38651
Nc-1-IP_pe	chr1	1.67E+08	1.67E+08	340	1.67E+08	14	7.34079	4.38651
Nc-1-IP_pe	chr1	1.67E+08	1.67E+08	259	1.67E+08	11	5.77463	3.94557
Nc-1-IP_pe	chr1	1.67E+08	1.67E+08	618	1.67E+08	10	3.4604	2.70797
Nc-1-IP_pe	chr1	1.67E+08	1.67E+08	266	1.67E+08	13	7.7352	4.78626
Nc-1-IP_pe	chr1	1.67E+08	1.67E+08	426	1.67E+08	8	3.74505	3.07688
Nc-1-IP_pe	chr1	1.67E+08	1.67E+08	320	1.67E+08	11	5.7051	3.90381
Nc-1-IP_pe	chr1	1.67E+08	1.67E+08	321	1.67E+08	12	5.23082	3.49289
Nc-1-IP_pe	chr1	1.67E+08	1.67E+08	485	1.67E+08	8	3.5501	2.95276
Nc-1-IP_pe	chr1	1.67E+08	1.67E+08	400	1.67E+08	11	4.88957	3.42271
Nc-1-IP_pe	chr1	1.67E+08	1.67E+08	641	1.67E+08	12	5.59553	3.69882
Nc-1-IP_pe	chr1	1.67E+08	1.67E+08	996	1.67E+08	17	8.9884	4.78058
Nc-1-IP_pe	chr1	1.67E+08	1.67E+08	392	1.67E+08	10	3.40382	2.67582
Nc-1-IP_pe	chr1	1.67E+08	1.67E+08	494	1.67E+08	10	3.89064	2.95552
Nc-1-IP_pe	chr1	1.67E+08	1.67E+08	367	1.67E+08	10	4.39571	3.25288

Nc-1-IP_pe	chr1	1.67E+08	1.67E+08	382	1.67E+08	8	3.08755	2.66145
Nc-1-IP_pe	chr1	1.67E+08	1.67E+08	463	1.67E+08	12	6.23601	4.06898
Nc-1-IP_pe	chr1	1.67E+08	1.67E+08	279	1.67E+08	15	8.84797	5.06275
Nc-1-IP_pe	chr1	1.67E+08	1.67E+08	497	1.67E+08	10	5.23357	3.76063
Nc-1-IP_pe	chr1	1.67E+08	1.67E+08	385	1.67E+08	10	5.00283	3.61918
Nc-1-IP_pe	chr1	1.68E+08	1.68E+08	280	1.68E+08	12	6.86906	4.44438
Nc-1-IP_pe	chr1	1.68E+08	1.68E+08	303	1.68E+08	12	3.77112	2.7071
Nc-1-IP_pe	chr1	1.68E+08	1.68E+08	347	1.68E+08	12	5.94619	3.9002
Nc-1-IP_pe	chr1	1.68E+08	1.68E+08	356	1.68E+08	14	8.63073	5.12814
Nc-1-IP_pe	chr1	1.68E+08	1.68E+08	563	1.68E+08	11	4.61014	3.26192
Nc-1-IP_pe	chr1	1.68E+08	1.68E+08	279	1.68E+08	9	4.35492	3.34727
Nc-1-IP_pe	chr1	1.68E+08	1.68E+08	314	1.68E+08	10	5.10552	3.682
Nc-1-IP_pe	chr1	1.68E+08	1.68E+08	541	1.68E+08	8	3.74505	3.07688
Nc-1-IP_pe	chr1	1.68E+08	1.68E+08	290	1.68E+08	11	5.10577	3.5486
Nc-1-IP_pe	chr1	1.68E+08	1.68E+08	544	1.68E+08	12	4.24064	2.95299
Nc-1-IP_pe	chr1	1.68E+08	1.68E+08	258	1.68E+08	11	3.64839	2.72584
Nc-1-IP_pe	chr1	1.68E+08	1.68E+08	393	1.68E+08	14	6.44441	3.89444
Nc-1-IP_pe	chr1	1.68E+08	1.68E+08	283	1.68E+08	12	4.24064	2.95299
Nc-1-IP_pe	chr1	1.68E+08	1.68E+08	596	1.68E+08	16	4.25252	2.63824
Nc-1-IP_pe	chr1	1.68E+08	1.68E+08	774	1.68E+08	17	9.99923	5.32289
Nc-1-IP_pe	chr1	1.68E+08	1.68E+08	390	1.68E+08	16	6.51374	3.67221
Nc-1-IP_pe	chr1	1.68E+08	1.68E+08	261	1.68E+08	11	3.64839	2.72584
Nc-1-IP_pe	chr1	1.68E+08	1.68E+08	485	1.68E+08	10	5.23357	3.76063
Nc-1-IP_pe	chr1	1.68E+08	1.68E+08	457	1.68E+08	9	3.72192	2.95716
Nc-1-IP_pe	chr1	1.68E+08	1.68E+08	475	1.68E+08	14	5.43414	3.36584
Nc-1-IP_pe	chr1	1.68E+08	1.68E+08	260	1.68E+08	11	6.03439	4.10251
Nc-1-IP_pe	chr1	1.68E+08	1.68E+08	411	1.68E+08	9	4.4496	3.40639
Nc-1-IP_pe	chr1	1.68E+08	1.68E+08	275	1.68E+08	13	5.9487	3.76157
Nc-1-IP_pe	chr1	1.68E+08	1.68E+08	313	1.68E+08	15	7.83541	4.49711
Nc-1-IP_pe	chr1	1.68E+08	1.68E+08	332	1.68E+08	12	5.67619	3.74486
Nc-1-IP_pe	chr1	1.68E+08	1.68E+08	379	1.68E+08	15	6.78905	3.93887
Nc-1-IP_pe	chr1	1.68E+08	1.68E+08	532	1.68E+08	13	4.86362	3.18015
Nc-1-IP_pe	chr1	1.68E+08	1.68E+08	298	1.68E+08	13	4.86362	3.18015
Nc-1-IP_pe	chr1	1.68E+08	1.68E+08	315	1.68E+08	10	5.23357	3.76063
Nc-1-IP_pe	chr1	1.68E+08	1.68E+08	253	1.68E+08	22	12.505	5.66212
Nc-1-IP_pe	chr1	1.68E+08	1.68E+08	408	1.68E+08	15	3.46204	2.35851
Nc-1-IP_pe	chr1	1.68E+08	1.68E+08	781	1.68E+08	11	6.03439	4.10251
Nc-1-IP_pe	chr1	1.68E+08	1.68E+08	355	1.68E+08	12	5.77527	3.80164
Nc-1-IP_pe	chr1	1.68E+08	1.68E+08	572	1.68E+08	13	4.86362	3.18015
Nc-1-IP_pe	chr1	1.68E+08	1.68E+08	477	1.68E+08	9	4.46937	3.41876
Nc-1-IP_pe	chr1	1.68E+08	1.68E+08	423	1.68E+08	10	4.95265	3.58858
Nc-1-IP_pe	chr1	1.68E+08	1.68E+08	374	1.68E+08	11	6.03439	4.10251
Nc-1-IP_pe	chr1	1.68E+08	1.68E+08	270	1.68E+08	7	3.06442	2.73501
Nc-1-IP_pe	chr1	1.68E+08	1.68E+08	283	1.68E+08	12	5.23082	3.49289
Nc-1-IP_pe	chr1	1.68E+08	1.68E+08	302	1.68E+08	11	4.06403	2.95415

Nc-1-IP_pe	chr1	1.68E+08	1.68E+08	329	1.68E+08	11	5.83542	3.98217
Nc-1-IP_pe	chr1	1.68E+08	1.68E+08	504	1.68E+08	12	3.47963	2.55766
Nc-1-IP_pe	chr1	1.69E+08	1.69E+08	401	1.69E+08	14	5.51541	3.4073
Nc-1-IP_pe	chr1	1.69E+08	1.69E+08	257	1.69E+08	9	4.38615	3.36674
Nc-1-IP_pe	chr1	1.69E+08	1.69E+08	279	1.69E+08	11	4.35236	3.11555
Nc-1-IP_pe	chr1	1.69E+08	1.69E+08	267	1.69E+08	12	6.86906	4.44438
Nc-1-IP_pe	chr1	1.69E+08	1.69E+08	489	1.69E+08	12	4.70082	3.20033
Nc-1-IP_pe	chr1	1.69E+08	1.69E+08	279	1.69E+08	10	4.77495	3.48064
Nc-1-IP_pe	chr1	1.69E+08	1.69E+08	640	1.69E+08	12	6.86906	4.44438
Nc-1-IP_pe	chr1	1.69E+08	1.69E+08	266	1.69E+08	15	8.61047	4.928
Nc-1-IP_pe	chr1	1.69E+08	1.69E+08	962	1.69E+08	12	6.86906	4.44438
Nc-1-IP_pe	chr1	1.69E+08	1.69E+08	439	1.69E+08	11	5.10577	3.5486
Nc-1-IP_pe	chr1	1.69E+08	1.69E+08	271	1.69E+08	12	6.6717	4.32642
Nc-1-IP_pe	chr1	1.69E+08	1.69E+08	261	1.69E+08	8	3.56021	2.95918
Nc-1-IP_pe	chr1	1.69E+08	1.69E+08	424	1.69E+08	14	4.95172	3.12357
Nc-1-IP_pe	chr1	1.69E+08	1.69E+08	1053	1.69E+08	17	6.47874	3.54137
Nc-1-IP_pe	chr1	1.69E+08	1.69E+08	420	1.69E+08	11	5.19376	3.60018
Nc-1-IP_pe	chr1	1.69E+08	1.69E+08	255	1.69E+08	12	6.86906	4.44438
Nc-1-IP_pe	chr1	1.69E+08	1.69E+08	382	1.69E+08	12	6.86906	4.44438
Nc-1-IP_pe	chr1	1.69E+08	1.69E+08	516	1.69E+08	12	5.23082	3.49289
Nc-1-IP_pe	chr1	1.69E+08	1.69E+08	337	1.69E+08	10	3.89064	2.95552
Nc-1-IP_pe	chr1	1.69E+08	1.69E+08	466	1.69E+08	10	5.23357	3.76063
Nc-1-IP_pe	chr1	1.69E+08	1.69E+08	480	1.69E+08	8	3.264	2.772
Nc-1-IP_pe	chr1	1.69E+08	1.69E+08	332	1.69E+08	20	8.4457	4.07937
Nc-1-IP_pe	chr1	1.7E+08	1.7E+08	289	1.7E+08	13	4.86362	3.18015
Nc-1-IP_pe	chr1	1.7E+08	1.7E+08	542	1.7E+08	14	6.06519	3.69269
Nc-1-IP_pe	chr1	1.7E+08	1.7E+08	374	1.7E+08	13	6.62436	4.14003
Nc-1-IP_pe	chr1	1.7E+08	1.7E+08	533	1.7E+08	12	5.23082	3.49289
Nc-1-IP_pe	chr1	1.7E+08	1.7E+08	301	1.7E+08	14	6.69566	4.03025
Nc-1-IP_pe	chr1	1.7E+08	1.7E+08	253	1.7E+08	9	3.72192	2.95716
Nc-1-IP_pe	chr1	1.7E+08	1.7E+08	279	1.7E+08	9	4.4177	3.38645
Nc-1-IP_pe	chr1	1.7E+08	1.7E+08	471	1.7E+08	10	3.89064	2.95552
Nc-1-IP_pe	chr1	1.7E+08	1.7E+08	254	1.7E+08	9	4.46937	3.41876
Nc-1-IP_pe	chr1	1.7E+08	1.7E+08	299	1.7E+08	13	6.36468	3.99316
Nc-1-IP_pe	chr1	1.7E+08	1.7E+08	632	1.7E+08	16	5.37454	3.13479
Nc-1-IP_pe	chr1	1.7E+08	1.7E+08	520	1.7E+08	15	6.78905	3.93887
Nc-1-IP_pe	chr1	1.7E+08	1.7E+08	479	1.7E+08	9	4.25966	3.28797
Nc-1-IP_pe	chr1	1.7E+08	1.7E+08	306	1.7E+08	16	6.37501	3.60492
Nc-1-IP_pe	chr1	1.7E+08	1.7E+08	1375	1.7E+08	11	6.03439	4.10251
Nc-1-IP_pe	chr1	1.7E+08	1.7E+08	315	1.7E+08	14	8.29543	4.93196
Nc-1-IP_pe	chr1	1.7E+08	1.7E+08	654	1.7E+08	13	5.36853	3.44651
Nc-1-IP_pe	chr1	1.7E+08	1.7E+08	650	1.7E+08	12	6.86906	4.44438
Nc-1-IP_pe	chr1	1.71E+08	1.71E+08	322	1.71E+08	8	3.74505	3.07688
Nc-1-IP_pe	chr1	1.71E+08	1.71E+08	354	1.71E+08	10	3.89064	2.95552
Nc-1-IP_pe	chr1	1.71E+08	1.71E+08	416	1.71E+08	8	3.74505	3.07688

Nc-1-IP_pe	chr1	1.71E+08	1.71E+08	272	1.71E+08	10	5.23357	3.76063
Nc-1-IP_pe	chr1	1.71E+08	1.71E+08	362	1.71E+08	12	6.86906	4.44438
Nc-1-IP_pe	chr1	1.71E+08	1.71E+08	354	1.71E+08	10	5.10552	3.682
Nc-1-IP_pe	chr1	1.71E+08	1.71E+08	810	1.71E+08	10	5.23357	3.76063
Nc-1-IP_pe	chr1	1.71E+08	1.71E+08	265	1.71E+08	9	2.89249	2.46179
Nc-1-IP_pe	chr1	1.71E+08	1.71E+08	264	1.71E+08	13	5.36853	3.44651
Nc-1-IP_pe	chr1	1.71E+08	1.71E+08	464	1.71E+08	10	4.39571	3.25288
Nc-1-IP_pe	chr1	1.71E+08	1.71E+08	595	1.71E+08	9	3.72192	2.95716
Nc-1-IP_pe	chr1	1.71E+08	1.71E+08	258	1.71E+08	10	4.39571	3.25288
Nc-1-IP_pe	chr1	1.71E+08	1.71E+08	257	1.71E+08	10	5.23357	3.76063
Nc-1-IP_pe	chr1	1.71E+08	1.71E+08	456	1.71E+08	11	5.7051	3.90381
Nc-1-IP_pe	chr1	1.71E+08	1.71E+08	254	1.71E+08	9	4.46937	3.41876
Nc-1-IP_pe	chr1	1.71E+08	1.71E+08	272	1.71E+08	14	4.60011	2.95114
Nc-1-IP_pe	chr1	1.71E+08	1.71E+08	318	1.71E+08	15	9.55381	5.47001
Nc-1-IP_pe	chr1	1.72E+08	1.72E+08	261	1.72E+08	10	5.23357	3.76063
Nc-1-IP_pe	chr1	1.72E+08	1.72E+08	295	1.72E+08	7	3.06442	2.73501
Nc-1-IP_pe	chr1	1.72E+08	1.72E+08	276	1.72E+08	9	4.46937	3.41876
Nc-1-IP_pe	chr1	1.72E+08	1.72E+08	329	1.72E+08	10	3.89064	2.95552
Nc-1-IP_pe	chr1	1.72E+08	1.72E+08	495	1.72E+08	10	5.23357	3.76063
Nc-1-IP_pe	chr1	1.72E+08	1.72E+08	266	1.72E+08	10	5.23357	3.76063
Nc-1-IP_pe	chr1	1.72E+08	1.72E+08	262	1.72E+08	8	3.74505	3.07688
Nc-1-IP_pe	chr1	1.72E+08	1.72E+08	266	1.72E+08	8	3.56021	2.95918
Nc-1-IP_pe	chr1	1.72E+08	1.72E+08	697	1.72E+08	14	6.69566	4.03025
Nc-1-IP_pe	chr1	1.72E+08	1.72E+08	331	1.72E+08	9	4.46937	3.41876
Nc-1-IP_pe	chr1	1.72E+08	1.72E+08	293	1.72E+08	10	4.99888	3.61677
Nc-1-IP_pe	chr1	1.72E+08	1.72E+08	315	1.72E+08	18	9.94172	5.10499
Nc-1-IP_pe	chr1	1.72E+08	1.72E+08	348	1.72E+08	11	5.33971	3.68619
Nc-1-IP_pe	chr1	1.72E+08	1.72E+08	324	1.72E+08	9	3.27326	2.68684
Nc-1-IP_pe	chr1	1.72E+08	1.72E+08	257	1.72E+08	12	5.84943	3.84431
Nc-1-IP_pe	chr1	1.72E+08	1.72E+08	350	1.72E+08	9	4.46937	3.41876
Nc-1-IP_pe	chr1	1.72E+08	1.72E+08	344	1.72E+08	17	6.47874	3.54137
Nc-1-IP_pe	chr1	1.72E+08	1.72E+08	1057	1.72E+08	9	3.66792	2.92434
Nc-1-IP_pe	chr1	1.72E+08	1.72E+08	272	1.72E+08	12	4.24064	2.95299
Nc-1-IP_pe	chr1	1.72E+08	1.72E+08	415	1.72E+08	11	4.74697	3.34038
Nc-1-IP_pe	chr1	1.73E+08	1.73E+08	356	1.73E+08	12	4.1728	2.91706
Nc-1-IP_pe	chr1	1.73E+08	1.73E+08	568	1.73E+08	17	7.02067	3.79543
Nc-1-IP_pe	chr1	1.73E+08	1.73E+08	945	1.73E+08	11	4.06403	2.95415
Nc-1-IP_pe	chr1	1.73E+08	1.73E+08	270	1.73E+08	13	7.54321	4.67266
Nc-1-IP_pe	chr1	1.73E+08	1.73E+08	436	1.73E+08	10	4.93609	3.57849
Nc-1-IP_pe	chr1	1.73E+08	1.73E+08	352	1.73E+08	13	5.36853	3.44651
Nc-1-IP_pe	chr1	1.73E+08	1.73E+08	288	1.73E+08	13	6.54342	4.09407
Nc-1-IP_pe	chr1	1.73E+08	1.73E+08	264	1.73E+08	13	7.7352	4.78626
Nc-1-IP_pe	chr1	1.73E+08	1.73E+08	428	1.73E+08	13	6.62436	4.14003
Nc-1-IP_pe	chr1	1.73E+08	1.73E+08	264	1.73E+08	16	10.0135	5.53039
Nc-1-IP_pe	chr1	1.73E+08	1.73E+08	443	1.73E+08	12	5.84943	3.84431

Nc-1-IP_pe	chr1	1.73E+08	1.73E+08	324	1.73E+08	13	5.9487	3.76157
Nc-1-IP_pe	chr1	1.73E+08	1.73E+08	481	1.73E+08	8	3.56021	2.95918
Nc-1-IP_pe	chr1	1.73E+08	1.73E+08	543	1.73E+08	12	6.58417	4.27436
Nc-1-IP_pe	chr1	1.73E+08	1.73E+08	278	1.73E+08	9	4.4177	3.38645
Nc-1-IP_pe	chr1	1.73E+08	1.73E+08	392	1.73E+08	14	6.06519	3.69269
Nc-1-IP_pe	chr1	1.73E+08	1.73E+08	564	1.73E+08	10	4.66672	3.41527
Nc-1-IP_pe	chr1	1.73E+08	1.73E+08	402	1.73E+08	11	3.64839	2.72584
Nc-1-IP_pe	chr1	1.73E+08	1.73E+08	328	1.73E+08	9	2.89249	2.46179
Nc-1-IP_pe	chr1	1.73E+08	1.73E+08	526	1.73E+08	11	5.91198	4.02837
Nc-1-IP_pe	chr1	1.73E+08	1.73E+08	692	1.73E+08	20	10.7672	5.16976
Nc-1-IP_pe	chr1	1.73E+08	1.73E+08	253	1.73E+08	10	5.23357	3.76063
Nc-1-IP_pe	chr1	1.74E+08	1.74E+08	296	1.74E+08	10	3.08907	2.49869
Nc-1-IP_pe	chr1	1.74E+08	1.74E+08	344	1.74E+08	13	7.7352	4.78626
Nc-1-IP_pe	chr1	1.74E+08	1.74E+08	286	1.74E+08	7	2.97697	2.67781
Nc-1-IP_pe	chr1	1.74E+08	1.74E+08	315	1.74E+08	9	4.46937	3.41876
Nc-1-IP_pe	chr1	1.74E+08	1.74E+08	264	1.74E+08	11	6.03439	4.10251
Nc-1-IP_pe	chr1	1.74E+08	1.74E+08	422	1.74E+08	12	6.50787	4.22912
Nc-1-IP_pe	chr1	1.74E+08	1.74E+08	284	1.74E+08	11	4.54404	3.2242
Nc-1-IP_pe	chr1	1.74E+08	1.74E+08	362	1.74E+08	11	5.77463	3.94557
Nc-1-IP_pe	chr1	1.74E+08	1.74E+08	312	1.74E+08	12	6.86906	4.44438
Nc-1-IP_pe	chr1	1.74E+08	1.74E+08	472	1.74E+08	10	5.23357	3.76063
Nc-1-IP_pe	chr1	1.74E+08	1.74E+08	261	1.74E+08	13	5.36853	3.44651
Nc-1-IP_pe	chr1	1.74E+08	1.74E+08	274	1.74E+08	10	5.23357	3.76063
Nc-1-IP_pe	chr1	1.74E+08	1.74E+08	308	1.74E+08	10	5.23357	3.76063
Nc-1-IP_pe	chr1	1.74E+08	1.74E+08	261	1.74E+08	9	3.54889	2.85226
Nc-1-IP_pe	chr1	1.74E+08	1.74E+08	315	1.74E+08	12	6.86906	4.44438
Nc-1-IP_pe	chr1	1.74E+08	1.74E+08	302	1.74E+08	9	4.46937	3.41876
Nc-1-IP_pe	chr1	1.74E+08	1.74E+08	851	1.74E+08	12	6.31185	4.11348
Nc-1-IP_pe	chr1	1.74E+08	1.74E+08	257	1.74E+08	11	6.03439	4.10251
Nc-1-IP_pe	chr1	1.74E+08	1.74E+08	334	1.74E+08	14	6.06519	3.69269
Nc-1-IP_pe	chr1	1.74E+08	1.74E+08	280	1.74E+08	17	7.02067	3.79543
Nc-1-IP_pe	chr1	1.74E+08	1.74E+08	657	1.74E+08	11	4.47871	3.18705
Nc-1-IP_pe	chr1	1.74E+08	1.74E+08	438	1.74E+08	9	4.46937	3.41876
Nc-1-IP_pe	chr1	1.74E+08	1.74E+08	403	1.74E+08	13	4.86362	3.18015
Nc-1-IP_pe	chr1	1.74E+08	1.74E+08	287	1.74E+08	10	5.23357	3.76063
Nc-1-IP_pe	chr1	1.74E+08	1.74E+08	291	1.74E+08	12	4.70082	3.20033
Nc-1-IP_pe	chr1	1.74E+08	1.74E+08	505	1.74E+08	11	4.54404	3.2242
Nc-1-IP_pe	chr1	1.74E+08	1.74E+08	892	1.74E+08	12	6.86906	4.44438
Nc-1-IP_pe	chr1	1.74E+08	1.74E+08	266	1.74E+08	8	3.74505	3.07688
Nc-1-IP_pe	chr1	1.74E+08	1.74E+08	429	1.74E+08	9	4.46937	3.41876
Nc-1-IP_pe	chr1	1.74E+08	1.74E+08	275	1.74E+08	12	5.45448	3.61875
Nc-1-IP_pe	chr1	1.74E+08	1.74E+08	547	1.74E+08	9	4.46937	3.41876
Nc-1-IP_pe	chr1	1.74E+08	1.74E+08	339	1.74E+08	10	5.23357	3.76063
Nc-1-IP_pe	chr1	1.74E+08	1.74E+08	425	1.74E+08	18	7.73136	4.00629
Nc-1-IP_pe	chr1	1.74E+08	1.74E+08	349	1.74E+08	12	6.86906	4.44438

Nc-1-IP_pe	chr1	1.74E+08	1.74E+08	558	1.74E+08	11	5.10577	3.5486
Nc-1-IP_pe	chr1	1.74E+08	1.74E+08	342	1.74E+08	10	5.23357	3.76063
Nc-1-IP_pe	chr1	1.74E+08	1.74E+08	1568	1.74E+08	20	14.0444	6.90474
Nc-1-IP_pe	chr1	1.75E+08	1.75E+08	472	1.75E+08	9	4.25966	3.28797
Nc-1-IP_pe	chr1	1.75E+08	1.75E+08	256	1.75E+08	8	3.08755	2.66145
Nc-1-IP_pe	chr1	1.75E+08	1.75E+08	461	1.75E+08	15	8.26005	4.73146
Nc-1-IP_pe	chr1	1.75E+08	1.75E+08	442	1.75E+08	9	4.46937	3.41876
Nc-1-IP_pe	chr1	1.75E+08	1.75E+08	270	1.75E+08	11	5.10577	3.5486
Nc-1-IP_pe	chr1	1.75E+08	1.75E+08	276	1.75E+08	9	4.46937	3.41876
Nc-1-IP_pe	chr1	1.75E+08	1.75E+08	272	1.75E+08	9	4.46937	3.41876
Nc-1-IP_pe	chr1	1.75E+08	1.75E+08	561	1.75E+08	12	4.70082	3.20033
Nc-1-IP_pe	chr1	1.75E+08	1.75E+08	259	1.75E+08	7	2.9043	2.63038
Nc-1-IP_pe	chr1	1.75E+08	1.75E+08	259	1.75E+08	11	5.77463	3.94557
Nc-1-IP_pe	chr1	1.75E+08	1.75E+08	434	1.75E+08	11	4.88957	3.42271
Nc-1-IP_pe	chr1	1.75E+08	1.75E+08	343	1.75E+08	14	5.03085	3.16286
Nc-1-IP_pe	chr1	1.75E+08	1.75E+08	549	1.75E+08	13	6.62436	4.14003
Nc-1-IP_pe	chr1	1.75E+08	1.75E+08	1018	1.75E+08	11	5.10577	3.5486
Nc-1-IP_pe	chr1	1.75E+08	1.75E+08	969	1.75E+08	13	5.36853	3.44651
Nc-1-IP_pe	chr1	1.75E+08	1.75E+08	513	1.75E+08	10	3.4604	2.70797
Nc-1-IP_pe	chr1	1.75E+08	1.75E+08	366	1.75E+08	14	6.06519	3.69269
Nc-1-IP_pe	chr1	1.75E+08	1.75E+08	290	1.75E+08	10	5.23357	3.76063
Nc-1-IP_pe	chr1	1.75E+08	1.75E+08	586	1.75E+08	12	6.31185	4.11348
Nc-1-IP_pe	chr1	1.75E+08	1.75E+08	283	1.75E+08	10	5.23357	3.76063
Nc-1-IP_pe	chr1	1.75E+08	1.75E+08	772	1.75E+08	11	5.77463	3.94557
Nc-1-IP_pe	chr1	1.75E+08	1.75E+08	275	1.75E+08	9	4.4177	3.38645
Nc-1-IP_pe	chr1	1.75E+08	1.75E+08	986	1.75E+08	13	4.86362	3.18015
Nc-1-IP_pe	chr1	1.75E+08	1.75E+08	318	1.75E+08	8	3.69948	3.0478
Nc-1-IP_pe	chr1	1.75E+08	1.75E+08	364	1.75E+08	11	4.54404	3.2242
Nc-1-IP_pe	chr1	1.75E+08	1.75E+08	425	1.75E+08	11	5.74187	3.92588
Nc-1-IP_pe	chr1	1.75E+08	1.75E+08	395	1.75E+08	12	6.65086	4.31401
Nc-1-IP_pe	chr1	1.75E+08	1.75E+08	291	1.75E+08	9	4.25966	3.28797
Nc-1-IP_pe	chr1	1.75E+08	1.75E+08	270	1.75E+08	9	4.46937	3.41876
Nc-1-IP_pe	chr1	1.75E+08	1.75E+08	368	1.75E+08	9	3.75449	2.977
Nc-1-IP_pe	chr1	1.75E+08	1.75E+08	321	1.75E+08	12	4.88508	3.30112
Nc-1-IP_pe	chr1	1.75E+08	1.75E+08	344	1.75E+08	15	9.55381	5.47001
Nc-1-IP_pe	chr1	1.75E+08	1.75E+08	519	1.75E+08	12	6.58417	4.27436
Nc-1-IP_pe	chr1	1.75E+08	1.75E+08	342	1.75E+08	10	3.4604	2.70797
Nc-1-IP_pe	chr1	1.75E+08	1.75E+08	342	1.75E+08	9	3.27326	2.68684
Nc-1-IP_pe	chr1	1.75E+08	1.75E+08	391	1.75E+08	12	4.24064	2.95299
Nc-1-IP_pe	chr1	1.75E+08	1.75E+08	292	1.75E+08	11	5.7051	3.90381
Nc-1-IP_pe	chr1	1.75E+08	1.75E+08	464	1.75E+08	11	5.89269	4.01672
Nc-1-IP_pe	chr1	1.75E+08	1.75E+08	312	1.75E+08	9	4.46937	3.41876
Nc-1-IP_pe	chr1	1.76E+08	1.76E+08	271	1.76E+08	10	5.23357	3.76063
Nc-1-IP_pe	chr1	1.76E+08	1.76E+08	273	1.76E+08	8	3.63049	3.00385
Nc-1-IP_pe	chr1	1.76E+08	1.76E+08	276	1.76E+08	13	5.9487	3.76157

Nc-1-IP_pe	chr1	1.76E+08	1.76E+08	565	1.76E+08	8	3.64414	3.01254
Nc-1-IP_pe	chr1	1.76E+08	1.76E+08	331	1.76E+08	9	4.46937	3.41876
Nc-1-IP_pe	chr1	1.76E+08	1.76E+08	266	1.76E+08	9	4.23325	3.27156
Nc-1-IP_pe	chr1	1.76E+08	1.76E+08	284	1.76E+08	12	6.12485	4.004
Nc-1-IP_pe	chr1	1.76E+08	1.76E+08	591	1.76E+08	9	3.27326	2.68684
Nc-1-IP_pe	chr1	1.76E+08	1.76E+08	384	1.76E+08	14	5.43414	3.36584
Nc-1-IP_pe	chr1	1.76E+08	1.76E+08	265	1.76E+08	11	6.03439	4.10251
Nc-1-IP_pe	chr1	1.76E+08	1.76E+08	253	1.76E+08	12	5.23082	3.49289
Nc-1-IP_pe	chr1	1.76E+08	1.76E+08	266	1.76E+08	13	7.7352	4.78626
Nc-1-IP_pe	chr1	1.76E+08	1.76E+08	261	1.76E+08	13	7.7352	4.78626
Nc-1-IP_pe	chr1	1.76E+08	1.76E+08	257	1.76E+08	15	6.78905	3.93887
Nc-1-IP_pe	chr1	1.76E+08	1.76E+08	330	1.76E+08	10	5.23357	3.76063
Nc-1-IP_pe	chr1	1.76E+08	1.76E+08	313	1.76E+08	11	3.64839	2.72584
Nc-1-IP_pe	chr1	1.76E+08	1.76E+08	467	1.76E+08	10	3.89064	2.95552
Nc-1-IP_pe	chr1	1.76E+08	1.76E+08	285	1.76E+08	9	4.46937	3.41876
Nc-1-IP_pe	chr1	1.76E+08	1.76E+08	965	1.76E+08	15	7.46994	4.29894
Nc-1-IP_pe	chr1	1.76E+08	1.76E+08	788	1.76E+08	11	6.03439	4.10251
Nc-1-IP_pe	chr1	1.76E+08	1.76E+08	331	1.76E+08	12	4.24064	2.95299
Nc-1-IP_pe	chr1	1.76E+08	1.76E+08	269	1.76E+08	9	4.25966	3.28797
Nc-1-IP_pe	chr1	1.76E+08	1.76E+08	340	1.76E+08	10	4.99888	3.61677
Nc-1-IP_pe	chr1	1.76E+08	1.76E+08	605	1.76E+08	11	5.77463	3.94557
Nc-1-IP_pe	chr1	1.76E+08	1.76E+08	487	1.76E+08	9	3.72192	2.95716
Nc-1-IP_pe	chr1	1.77E+08	1.77E+08	282	1.77E+08	10	5.23357	3.76063
Nc-1-IP_pe	chr1	1.77E+08	1.77E+08	306	1.77E+08	10	5.23357	3.76063
Nc-1-IP_pe	chr1	1.77E+08	1.77E+08	360	1.77E+08	11	6.03439	4.10251
Nc-1-IP_pe	chr1	1.77E+08	1.77E+08	296	1.77E+08	11	6.03439	4.10251
Nc-1-IP_pe	chr1	1.77E+08	1.77E+08	258	1.77E+08	16	8.26996	4.56762
Nc-1-IP_pe	chr1	1.77E+08	1.77E+08	390	1.77E+08	9	3.27326	2.68684
Nc-1-IP_pe	chr1	1.77E+08	1.77E+08	304	1.77E+08	9	3.79246	3.00015
Nc-1-IP_pe	chr1	1.77E+08	1.77E+08	457	1.77E+08	13	4.41954	2.952
Nc-1-IP_pe	chr1	1.77E+08	1.77E+08	692	1.77E+08	14	8.63073	5.12814
Nc-1-IP_pe	chr1	1.77E+08	1.77E+08	441	1.77E+08	11	5.52654	3.79706
Nc-1-IP_pe	chr1	1.78E+08	1.78E+08	388	1.78E+08	11	6.03439	4.10251
Nc-1-IP_pe	chr1	1.78E+08	1.78E+08	511	1.78E+08	10	5.23357	3.76063
Nc-1-IP_pe	chr1	1.78E+08	1.78E+08	258	1.78E+08	11	6.03439	4.10251
Nc-1-IP_pe	chr1	1.78E+08	1.78E+08	551	1.78E+08	10	5.23357	3.76063
Nc-1-IP_pe	chr1	1.78E+08	1.78E+08	459	1.78E+08	9	2.89249	2.46179
Nc-1-IP_pe	chr1	1.78E+08	1.78E+08	260	1.78E+08	9	4.46937	3.41876
Nc-1-IP_pe	chr1	1.78E+08	1.78E+08	660	1.78E+08	9	4.25966	3.28797
Nc-1-IP_pe	chr1	1.78E+08	1.78E+08	445	1.78E+08	15	6.78905	3.93887
Nc-1-IP_pe	chr1	1.78E+08	1.78E+08	404	1.78E+08	9	4.46937	3.41876
Nc-1-IP_pe	chr1	1.78E+08	1.78E+08	258	1.78E+08	11	4.06403	2.95415
Nc-1-IP_pe	chr1	1.78E+08	1.78E+08	728	1.78E+08	13	5.9487	3.76157
Nc-1-IP_pe	chr1	1.78E+08	1.78E+08	254	1.78E+08	11	3.64839	2.72584
Nc-1-IP_pe	chr1	1.78E+08	1.78E+08	253	1.78E+08	12	5.23082	3.49289

Nc-1-IP_pe	chr1	1.78E+08	1.78E+08	418	1.78E+08	8	3.74505	3.07688
Nc-1-IP_pe	chr1	1.78E+08	1.78E+08	650	1.78E+08	16	5.62031	3.24783
Nc-1-IP_pe	chr1	1.78E+08	1.78E+08	446	1.78E+08	12	6.86906	4.44438
Nc-1-IP_pe	chr1	1.78E+08	1.78E+08	283	1.78E+08	10	4.99888	3.61677
Nc-1-IP_pe	chr1	1.78E+08	1.78E+08	572	1.78E+08	16	6.33285	3.58457
Nc-1-IP_pe	chr1	1.78E+08	1.78E+08	323	1.78E+08	10	3.87863	2.94853
Nc-1-IP_pe	chr1	1.79E+08	1.79E+08	675	1.79E+08	12	4.70082	3.20033
Nc-1-IP_pe	chr1	1.79E+08	1.79E+08	363	1.79E+08	13	4.41954	2.952
Nc-1-IP_pe	chr1	1.79E+08	1.79E+08	305	1.79E+08	14	4.62572	2.96359
Nc-1-IP_pe	chr1	1.79E+08	1.79E+08	447	1.79E+08	8	3.74505	3.07688
Nc-1-IP_pe	chr1	1.79E+08	1.79E+08	381	1.79E+08	12	5.23082	3.49289
Nc-1-IP_pe	chr1	1.79E+08	1.79E+08	262	1.79E+08	10	4.99888	3.61677
Nc-1-IP_pe	chr1	1.79E+08	1.79E+08	380	1.79E+08	10	4.99888	3.61677
Nc-1-IP_pe	chr1	1.79E+08	1.79E+08	287	1.79E+08	9	4.46937	3.41876
Nc-1-IP_pe	chr1	1.79E+08	1.79E+08	327	1.79E+08	11	6.03439	4.10251
Nc-1-IP_pe	chr1	1.79E+08	1.79E+08	438	1.79E+08	11	4.54404	3.2242
Nc-1-IP_pe	chr1	1.79E+08	1.79E+08	286	1.79E+08	8	3.74505	3.07688
Nc-1-IP_pe	chr1	1.79E+08	1.79E+08	482	1.79E+08	11	4.23076	3.04719
Nc-1-IP_pe	chr1	1.79E+08	1.79E+08	1358	1.79E+08	11	4.06403	2.95415
Nc-1-IP_pe	chr1	1.79E+08	1.79E+08	457	1.79E+08	10	5.23357	3.76063
Nc-1-IP_pe	chr1	1.79E+08	1.79E+08	376	1.79E+08	11	4.35236	3.11555
Nc-1-IP_pe	chr1	1.79E+08	1.79E+08	449	1.79E+08	10	4.33501	3.21677
Nc-1-IP_pe	chr1	1.79E+08	1.79E+08	447	1.79E+08	10	5.21145	3.74702
Nc-1-IP_pe	chr1	1.79E+08	1.79E+08	332	1.79E+08	11	4.06403	2.95415
Nc-1-IP_pe	chr1	1.79E+08	1.79E+08	462	1.79E+08	10	3.08907	2.49869
Nc-1-IP_pe	chr1	1.79E+08	1.79E+08	597	1.79E+08	16	7.53857	4.18505
Nc-1-IP_pe	chr1	1.79E+08	1.79E+08	330	1.79E+08	8	3.08755	2.66145
Nc-1-IP_pe	chr1	1.79E+08	1.79E+08	279	1.79E+08	14	7.42849	4.43574
Nc-1-IP_pe	chr1	1.79E+08	1.79E+08	708	1.79E+08	18	6.63038	3.50359
Nc-1-IP_pe	chr1	1.79E+08	1.79E+08	261	1.79E+08	19	5.90435	3.10381
Nc-1-IP_pe	chr1	1.79E+08	1.79E+08	426	1.79E+08	10	3.7298	2.86234
Nc-1-IP_pe	chr1	1.79E+08	1.79E+08	497	1.79E+08	12	5.00569	3.36763
Nc-1-IP_pe	chr1	1.79E+08	1.79E+08	352	1.79E+08	9	3.56055	2.85931
Nc-1-IP_pe	chr1	1.79E+08	1.79E+08	610	1.79E+08	14	7.62616	4.54739
Nc-1-IP_pe	chr1	1.79E+08	1.79E+08	522	1.79E+08	8	3.74505	3.07688
Nc-1-IP_pe	chr1	1.79E+08	1.79E+08	1952	1.79E+08	15	6.85973	3.97568
Nc-1-IP_pe	chr1	1.79E+08	1.79E+08	607	1.79E+08	13	5.9487	3.76157
Nc-1-IP_pe	chr1	1.79E+08	1.79E+08	430	1.79E+08	7	3.06442	2.73501
Nc-1-IP_pe	chr1	1.79E+08	1.79E+08	265	1.79E+08	11	5.52654	3.79706
Nc-1-IP_pe	chr1	1.79E+08	1.79E+08	460	1.79E+08	10	3.4604	2.70797
Nc-1-IP_pe	chr1	1.79E+08	1.79E+08	529	1.79E+08	9	4.35492	3.34727
Nc-1-IP_pe	chr1	1.79E+08	1.79E+08	622	1.79E+08	9	3.72192	2.95716
Nc-1-IP_pe	chr1	1.79E+08	1.79E+08	358	1.79E+08	17	6.64406	3.61808
Nc-1-IP_pe	chr1	1.79E+08	1.79E+08	346	1.79E+08	9	4.46937	3.41876
Nc-1-IP_pe	chr1	1.79E+08	1.79E+08	355	1.79E+08	9	3.79246	3.00015

Nc-1-IP_pe	chr1	1.79E+08	1.79E+08	264	1.79E+08	11	5.56156	3.81794
Nc-1-IP_pe	chr1	1.79E+08	1.79E+08	294	1.79E+08	32	11.7583	4.05164
Nc-1-IP_pe	chr1	1.79E+08	1.79E+08	799	1.79E+08	9	4.46937	3.41876
Nc-1-IP_pe	chr1	1.79E+08	1.79E+08	495	1.79E+08	12	4.24064	2.95299
Nc-1-IP_pe	chr1	1.8E+08	1.8E+08	615	1.8E+08	10	5.23357	3.76063
Nc-1-IP_pe	chr1	1.8E+08	1.8E+08	362	1.8E+08	9	3.72192	2.95716
Nc-1-IP_pe	chr1	1.8E+08	1.8E+08	360	1.8E+08	14	7.34079	4.38651
Nc-1-IP_pe	chr1	1.8E+08	1.8E+08	581	1.8E+08	13	5.03208	3.26819
Nc-1-IP_pe	chr1	1.8E+08	1.8E+08	893	1.8E+08	13	7.7352	4.78626
Nc-1-IP_pe	chr1	1.8E+08	1.8E+08	376	1.8E+08	8	3.74505	3.07688
Nc-1-IP_pe	chr1	1.8E+08	1.8E+08	414	1.8E+08	9	4.25966	3.28797
Nc-1-IP_pe	chr1	1.8E+08	1.8E+08	719	1.8E+08	12	6.86906	4.44438
Nc-1-IP_pe	chr1	1.8E+08	1.8E+08	571	1.8E+08	12	6.86906	4.44438
Nc-1-IP_pe	chr1	1.8E+08	1.8E+08	282	1.8E+08	12	5.23082	3.49289
Nc-1-IP_pe	chr1	1.8E+08	1.8E+08	404	1.8E+08	11	4.00079	2.91908
Nc-1-IP_pe	chr1	1.8E+08	1.8E+08	263	1.8E+08	12	5.23082	3.49289
Nc-1-IP_pe	chr1	1.8E+08	1.8E+08	624	1.8E+08	13	5.47064	3.50127
Nc-1-IP_pe	chr1	1.8E+08	1.8E+08	318	1.8E+08	12	4.24064	2.95299
Nc-1-IP_pe	chr1	1.8E+08	1.8E+08	396	1.8E+08	11	4.6642	3.29286
Nc-1-IP_pe	chr1	1.8E+08	1.8E+08	681	1.8E+08	11	3.64839	2.72584
Nc-1-IP_pe	chr1	1.8E+08	1.8E+08	259	1.8E+08	9	3.27326	2.68684
Nc-1-IP_pe	chr1	1.8E+08	1.8E+08	315	1.8E+08	14	6.06519	3.69269
Nc-1-IP_pe	chr1	1.8E+08	1.8E+08	339	1.8E+08	10	3.89064	2.95552
Nc-1-IP_pe	chr1	1.8E+08	1.8E+08	673	1.8E+08	20	6.49719	3.259
Nc-1-IP_pe	chr1	1.8E+08	1.8E+08	359	1.8E+08	9	3.27326	2.68684
Nc-1-IP_pe	chr1	1.8E+08	1.8E+08	515	1.8E+08	15	6.78905	3.93887
Nc-1-IP_pe	chr1	1.8E+08	1.8E+08	287	1.8E+08	10	4.07292	3.06202
Nc-1-IP_pe	chr1	1.8E+08	1.8E+08	314	1.8E+08	12	5.23082	3.49289
Nc-1-IP_pe	chr1	1.8E+08	1.8E+08	829	1.8E+08	9	4.46568	3.41644
Nc-1-IP_pe	chr1	1.8E+08	1.8E+08	301	1.8E+08	11	6.03439	4.10251
Nc-1-IP_pe	chr1	1.8E+08	1.8E+08	388	1.8E+08	18	6.15736	3.29676
Nc-1-IP_pe	chr1	1.8E+08	1.8E+08	522	1.8E+08	11	4.47871	3.18705
Nc-1-IP_pe	chr1	1.8E+08	1.8E+08	476	1.8E+08	14	4.60011	2.95114
Nc-1-IP_pe	chr1	1.8E+08	1.8E+08	484	1.8E+08	11	5.09969	3.54504
Nc-1-IP_pe	chr1	1.8E+08	1.8E+08	454	1.8E+08	14	5.51541	3.4073
Nc-1-IP_pe	chr1	1.8E+08	1.8E+08	317	1.8E+08	8	3.32341	2.80939
Nc-1-IP_pe	chr1	1.8E+08	1.8E+08	331	1.8E+08	12	5.23082	3.49289
Nc-1-IP_pe	chr1	1.8E+08	1.8E+08	373	1.8E+08	10	4.41822	3.2663
Nc-1-IP_pe	chr1	1.8E+08	1.8E+08	309	1.8E+08	14	6.42818	3.88572
Nc-1-IP_pe	chr1	1.8E+08	1.8E+08	607	1.8E+08	12	4.88508	3.30112
Nc-1-IP_pe	chr1	1.8E+08	1.8E+08	798	1.8E+08	15	7.52557	4.32889
Nc-1-IP_pe	chr1	1.8E+08	1.8E+08	323	1.8E+08	12	4.88508	3.30112
Nc-1-IP_pe	chr1	1.8E+08	1.8E+08	303	1.8E+08	13	6.36468	3.99316
Nc-1-IP_pe	chr1	1.8E+08	1.8E+08	406	1.8E+08	13	7.00513	4.3584
Nc-1-IP_pe	chr1	1.8E+08	1.8E+08	721	1.8E+08	14	5.03085	3.16286

Nc-1-IP_pe	chr1	1.8E+08	1.8E+08	314	1.8E+08	9	4.46937	3.41876
Nc-1-IP_pe	chr1	1.8E+08	1.8E+08	326	1.8E+08	12	6.31185	4.11348
Nc-1-IP_pe	chr1	1.8E+08	1.8E+08	324	1.8E+08	16	4.96462	2.94973
Nc-1-IP_pe	chr1	1.8E+08	1.8E+08	333	1.8E+08	15	5.66913	3.37372
Nc-1-IP_pe	chr1	1.8E+08	1.8E+08	427	1.8E+08	7	2.97697	2.67781
Nc-1-IP_pe	chr1	1.8E+08	1.8E+08	308	1.8E+08	10	5.05378	3.65032
Nc-1-IP_pe	chr1	1.8E+08	1.8E+08	391	1.8E+08	14	6.44441	3.89444
Nc-1-IP_pe	chr1	1.8E+08	1.8E+08	438	1.8E+08	10	3.89064	2.95552
Nc-1-IP_pe	chr1	1.8E+08	1.8E+08	292	1.8E+08	12	5.82577	3.83068
Nc-1-IP_pe	chr1	1.8E+08	1.8E+08	343	1.8E+08	10	4.17505	3.1221
Nc-1-IP_pe	chr1	1.8E+08	1.8E+08	452	1.8E+08	15	9.02077	5.16154
Nc-1-IP_pe	chr1	1.8E+08	1.8E+08	1178	1.8E+08	12	6.46791	4.20548
Nc-1-IP_pe	chr1	1.8E+08	1.8E+08	495	1.8E+08	11	5.59692	3.83905
Nc-1-IP_pe	chr1	1.8E+08	1.8E+08	632	1.8E+08	12	5.61153	3.70794
Nc-1-IP_pe	chr1	1.8E+08	1.8E+08	287	1.8E+08	10	3.89064	2.95552
Nc-1-IP_pe	chr1	1.8E+08	1.8E+08	267	1.8E+08	12	6.71369	4.35145
Nc-1-IP_pe	chr1	1.8E+08	1.8E+08	275	1.8E+08	10	5.23357	3.76063
Nc-1-IP_pe	chr1	1.8E+08	1.8E+08	263	1.8E+08	14	8.63073	5.12814
Nc-1-IP_pe	chr1	1.81E+08	1.81E+08	847	1.81E+08	19	8.68415	4.32024
Nc-1-IP_pe	chr1	1.81E+08	1.81E+08	616	1.81E+08	14	8.63073	5.12814
Nc-1-IP_pe	chr1	1.81E+08	1.81E+08	395	1.81E+08	11	5.3563	3.696
Nc-1-IP_pe	chr1	1.81E+08	1.81E+08	371	1.81E+08	10	4.39571	3.25288
Nc-1-IP_pe	chr1	1.81E+08	1.81E+08	303	1.81E+08	16	10.5028	5.81189
Nc-1-IP_pe	chr1	1.81E+08	1.81E+08	291	1.81E+08	12	6.86404	4.44138
Nc-1-IP_pe	chr1	1.81E+08	1.81E+08	375	1.81E+08	10	5.23357	3.76063
Nc-1-IP_pe	chr1	1.81E+08	1.81E+08	510	1.81E+08	14	5.03085	3.16286
Nc-1-IP_pe	chr1	1.81E+08	1.81E+08	417	1.81E+08	13	7.56612	4.68618
Nc-1-IP_pe	chr1	1.81E+08	1.81E+08	303	1.81E+08	9	4.46937	3.41876
Nc-1-IP_pe	chr1	1.81E+08	1.81E+08	653	1.81E+08	11	6.03439	4.10251
Nc-1-IP_pe	chr1	1.81E+08	1.81E+08	291	1.81E+08	11	6.03439	4.10251
Nc-1-IP_pe	chr1	1.81E+08	1.81E+08	269	1.81E+08	11	6.03439	4.10251
Nc-1-IP_pe	chr1	1.81E+08	1.81E+08	257	1.81E+08	10	5.23357	3.76063
Nc-1-IP_pe	chr1	1.81E+08	1.81E+08	443	1.81E+08	9	3.27326	2.68684
Nc-1-IP_pe	chr1	1.81E+08	1.81E+08	457	1.81E+08	13	5.36853	3.44651
Nc-1-IP_pe	chr1	1.81E+08	1.81E+08	359	1.81E+08	12	6.86906	4.44438
Nc-1-IP_pe	chr1	1.81E+08	1.81E+08	285	1.81E+08	9	3.43499	2.78365
Nc-1-IP_pe	chr1	1.81E+08	1.81E+08	1207	1.81E+08	17	9.09431	4.8363
Nc-1-IP_pe	chr1	1.81E+08	1.81E+08	294	1.81E+08	13	4.41954	2.952
Nc-1-IP_pe	chr1	1.81E+08	1.81E+08	500	1.81E+08	9	2.89249	2.46179
Nc-1-IP_pe	chr1	1.81E+08	1.81E+08	526	1.81E+08	12	3.16137	2.39719
Nc-1-IP_pe	chr1	1.81E+08	1.81E+08	329	1.81E+08	11	5.74187	3.92588
Nc-1-IP_pe	chr1	1.81E+08	1.81E+08	385	1.81E+08	10	3.4604	2.70797
Nc-1-IP_pe	chr1	1.81E+08	1.81E+08	1028	1.81E+08	18	6.15736	3.29676
Nc-1-IP_pe	chr1	1.81E+08	1.81E+08	256	1.81E+08	11	5.7051	3.90381
Nc-1-IP_pe	chr1	1.81E+08	1.81E+08	267	1.81E+08	11	3.28475	2.53029

Nc-1-IP_pe	chr1	1.81E+08	1.81E+08	291	1.81E+08	15	6.19428	3.63445
Nc-1-IP_pe	chr1	1.81E+08	1.81E+08	454	1.81E+08	11	4.29098	3.08099
Nc-1-IP_pe	chr1	1.81E+08	1.81E+08	654	1.81E+08	12	5.23082	3.49289
Nc-1-IP_pe	chr1	1.81E+08	1.81E+08	638	1.81E+08	12	3.83688	2.74114
Nc-1-IP_pe	chr1	1.81E+08	1.81E+08	374	1.81E+08	14	7.38052	4.40879
Nc-1-IP_pe	chr1	1.81E+08	1.81E+08	466	1.81E+08	10	4.26734	3.17663
Nc-1-IP_pe	chr1	1.81E+08	1.81E+08	603	1.81E+08	15	9.55381	5.47001
Nc-1-IP_pe	chr1	1.81E+08	1.81E+08	401	1.81E+08	8	3.264	2.772
Nc-1-IP_pe	chr1	1.81E+08	1.81E+08	403	1.81E+08	14	7.42849	4.43574
Nc-1-IP_pe	chr1	1.81E+08	1.81E+08	254	1.81E+08	11	5.76039	3.93701
Nc-1-IP_pe	chr1	1.81E+08	1.81E+08	350	1.81E+08	12	5.23082	3.49289
Nc-1-IP_pe	chr1	1.81E+08	1.81E+08	540	1.81E+08	9	4.46937	3.41876
Nc-1-IP_pe	chr1	1.81E+08	1.81E+08	384	1.81E+08	13	7.7352	4.78626
Nc-1-IP_pe	chr1	1.81E+08	1.81E+08	295	1.81E+08	12	5.23082	3.49289
Nc-1-IP_pe	chr1	1.81E+08	1.81E+08	259	1.81E+08	9	3.72192	2.95716
Nc-1-IP_pe	chr1	1.81E+08	1.81E+08	376	1.81E+08	15	5.1177	3.10809
Nc-1-IP_pe	chr1	1.81E+08	1.81E+08	466	1.81E+08	10	4.39571	3.25288
Nc-1-IP_pe	chr1	1.81E+08	1.81E+08	313	1.81E+08	13	6.62436	4.14003
Nc-1-IP_pe	chr1	1.81E+08	1.81E+08	389	1.81E+08	8	3.48502	2.91148
Nc-1-IP_pe	chr1	1.81E+08	1.81E+08	256	1.81E+08	13	7.7352	4.78626
Nc-1-IP_pe	chr1	1.81E+08	1.81E+08	364	1.81E+08	9	4.46937	3.41876
Nc-1-IP_pe	chr1	1.81E+08	1.81E+08	324	1.81E+08	8	3.74505	3.07688
Nc-1-IP_pe	chr1	1.81E+08	1.81E+08	290	1.81E+08	9	3.72192	2.95716
Nc-1-IP_pe	chr1	1.81E+08	1.81E+08	320	1.81E+08	8	3.60341	2.98663
Nc-1-IP_pe	chr1	1.81E+08	1.81E+08	361	1.81E+08	10	5.23357	3.76063
Nc-1-IP_pe	chr1	1.81E+08	1.81E+08	640	1.81E+08	8	3.08755	2.66145
Nc-1-IP_pe	chr1	1.82E+08	1.82E+08	500	1.82E+08	10	5.23357	3.76063
Nc-1-IP_pe	chr1	1.82E+08	1.82E+08	268	1.82E+08	12	5.84943	3.84431
Nc-1-IP_pe	chr1	1.82E+08	1.82E+08	261	1.82E+08	9	4.46937	3.41876
Nc-1-IP_pe	chr1	1.82E+08	1.82E+08	485	1.82E+08	11	6.03439	4.10251
Nc-1-IP_pe	chr1	1.82E+08	1.82E+08	344	1.82E+08	13	7.42513	4.60316
Nc-1-IP_pe	chr1	1.82E+08	1.82E+08	287	1.82E+08	7	3.06442	2.73501
Nc-1-IP_pe	chr1	1.82E+08	1.82E+08	274	1.82E+08	12	6.86906	4.44438
Nc-1-IP_pe	chr1	1.82E+08	1.82E+08	1103	1.82E+08	18	6.63038	3.50359
Nc-1-IP_pe	chr1	1.82E+08	1.82E+08	326	1.82E+08	18	8.99859	4.62187
Nc-1-IP_pe	chr1	1.82E+08	1.82E+08	272	1.82E+08	8	2.93592	2.56703
Nc-1-IP_pe	chr1	1.82E+08	1.82E+08	611	1.82E+08	12	4.70082	3.20033
Nc-1-IP_pe	chr1	1.82E+08	1.82E+08	702	1.82E+08	10	5.23357	3.76063
Nc-1-IP_pe	chr1	1.82E+08	1.82E+08	312	1.82E+08	8	3.08755	2.66145
Nc-1-IP_pe	chr1	1.82E+08	1.82E+08	684	1.82E+08	9	3.61966	2.89507
Nc-1-IP_pe	chr1	1.82E+08	1.82E+08	618	1.82E+08	11	5.77463	3.94557
Nc-1-IP_pe	chr1	1.82E+08	1.82E+08	307	1.82E+08	11	6.03439	4.10251
Nc-1-IP_pe	chr1	1.82E+08	1.82E+08	305	1.82E+08	9	3.72192	2.95716
Nc-1-IP_pe	chr1	1.82E+08	1.82E+08	264	1.82E+08	12	5.5796	3.68975
Nc-1-IP_pe	chr1	1.82E+08	1.82E+08	590	1.82E+08	12	5.3037	3.53374

Nc-1-IP_pe	chr1	1.82E+08	1.82E+08	285	1.82E+08	12	5.84943	3.84431
Nc-1-IP_pe	chr1	1.82E+08	1.82E+08	480	1.82E+08	11	2.96397	2.36091
Nc-1-IP_pe	chr1	1.82E+08	1.82E+08	276	1.82E+08	11	3.64839	2.72584
Nc-1-IP_pe	chr1	1.82E+08	1.82E+08	301	1.82E+08	12	5.45448	3.61875
Nc-1-IP_pe	chr1	1.82E+08	1.82E+08	347	1.82E+08	11	4.47871	3.18705
Nc-1-IP_pe	chr1	1.82E+08	1.82E+08	281	1.82E+08	11	4.54404	3.2242
Nc-1-IP_pe	chr1	1.82E+08	1.82E+08	253	1.82E+08	8	3.74505	3.07688
Nc-1-IP_pe	chr1	1.82E+08	1.82E+08	256	1.82E+08	10	3.89064	2.95552
Nc-1-IP_pe	chr1	1.82E+08	1.82E+08	253	1.82E+08	11	4.54404	3.2242
Nc-1-IP_pe	chr1	1.82E+08	1.82E+08	386	1.82E+08	10	4.39571	3.25288
Nc-1-IP_pe	chr1	1.83E+08	1.83E+08	381	1.83E+08	10	5.23357	3.76063
Nc-1-IP_pe	chr1	1.83E+08	1.83E+08	385	1.83E+08	9	3.54889	2.85226
Nc-1-IP_pe	chr1	1.83E+08	1.83E+08	268	1.83E+08	11	5.77901	3.9482
Nc-1-IP_pe	chr1	1.83E+08	1.83E+08	427	1.83E+08	14	6.55975	3.95658
Nc-1-IP_pe	chr1	1.83E+08	1.83E+08	1413	1.83E+08	16	9.27336	5.11352
Nc-1-IP_pe	chr1	1.83E+08	1.83E+08	290	1.83E+08	11	5.20969	3.60954
Nc-1-IP_pe	chr1	1.83E+08	1.83E+08	319	1.83E+08	10	3.71867	2.85592
Nc-1-IP_pe	chr1	1.83E+08	1.83E+08	293	1.83E+08	15	9.19325	5.26076
Nc-1-IP_pe	chr1	1.83E+08	1.83E+08	294	1.83E+08	11	5.10577	3.5486
Nc-1-IP_pe	chr1	1.83E+08	1.83E+08	369	1.83E+08	8	3.74505	3.07688
Nc-1-IP_pe	chr1	1.83E+08	1.83E+08	778	1.83E+08	12	6.71369	4.35145
Nc-1-IP_pe	chr1	1.83E+08	1.83E+08	456	1.83E+08	12	6.528	4.24104
Nc-1-IP_pe	chr1	1.83E+08	1.83E+08	567	1.83E+08	14	3.55308	2.45762
Nc-1-IP_pe	chr1	1.83E+08	1.83E+08	713	1.83E+08	13	5.42865	3.47872
Nc-1-IP_pe	chr1	1.83E+08	1.83E+08	356	1.83E+08	9	4.46937	3.41876
Nc-1-IP_pe	chr1	1.83E+08	1.83E+08	360	1.83E+08	20	14.0444	6.90474
Nc-1-IP_pe	chr1	1.83E+08	1.83E+08	715	1.83E+08	14	6.69566	4.03025
Nc-1-IP_pe	chr1	1.83E+08	1.83E+08	259	1.83E+08	14	8.29543	4.93196
Nc-1-IP_pe	chr1	1.83E+08	1.83E+08	374	1.83E+08	14	4.60011	2.95114
Nc-1-IP_pe	chr1	1.83E+08	1.83E+08	311	1.83E+08	10	3.89064	2.95552
Nc-1-IP_pe	chr1	1.83E+08	1.83E+08	302	1.83E+08	14	6.28469	3.80898
Nc-1-IP_pe	chr1	1.83E+08	1.83E+08	463	1.83E+08	12	3.83688	2.74114
Nc-1-IP_pe	chr1	1.83E+08	1.83E+08	272	1.83E+08	10	4.47495	3.30017
Nc-1-IP_pe	chr1	1.83E+08	1.83E+08	314	1.83E+08	12	3.47963	2.55766
Nc-1-IP_pe	chr1	1.83E+08	1.83E+08	473	1.83E+08	11	5.77463	3.94557
Nc-1-IP_pe	chr1	1.83E+08	1.83E+08	439	1.83E+08	8	3.08755	2.66145
Nc-1-IP_pe	chr1	1.83E+08	1.83E+08	300	1.83E+08	9	3.72192	2.95716
Nc-1-IP_pe	chr1	1.83E+08	1.83E+08	386	1.83E+08	10	4.39571	3.25288
Nc-1-IP_pe	chr1	1.83E+08	1.83E+08	470	1.83E+08	9	3.60774	2.88785
Nc-1-IP_pe	chr1	1.83E+08	1.83E+08	434	1.83E+08	13	6.48901	4.06327
Nc-1-IP_pe	chr1	1.83E+08	1.83E+08	380	1.83E+08	15	7.91596	4.54123
Nc-1-IP_pe	chr1	1.83E+08	1.83E+08	330	1.83E+08	20	9.82443	4.71218
Nc-1-IP_pe	chr1	1.83E+08	1.83E+08	364	1.83E+08	14	7.6052	4.5355
Nc-1-IP_pe	chr1	1.83E+08	1.83E+08	327	1.83E+08	13	6.62436	4.14003
Nc-1-IP_pe	chr1	1.83E+08	1.83E+08	254	1.83E+08	10	5.01973	3.6295

Nc-1-IP_pe	chr1	1.83E+08	1.83E+08	322	1.83E+08	12	5.01934	3.37518
Nc-1-IP_pe	chr1	1.83E+08	1.83E+08	259	1.83E+08	16	6.80394	3.81462
Nc-1-IP_pe	chr1	1.83E+08	1.83E+08	481	1.83E+08	10	3.40382	2.67582
Nc-1-IP_pe	chr1	1.83E+08	1.83E+08	390	1.83E+08	13	4.86362	3.18015
Nc-1-IP_pe	chr1	1.83E+08	1.83E+08	417	1.83E+08	9	3.72192	2.95716
Nc-1-IP_pe	chr1	1.83E+08	1.83E+08	1673	1.83E+08	22	8.24538	3.76835
Nc-1-IP_pe	chr1	1.83E+08	1.83E+08	358	1.83E+08	9	4.25966	3.28797
Nc-1-IP_pe	chr1	1.83E+08	1.83E+08	275	1.83E+08	11	6.03439	4.10251
Nc-1-IP_pe	chr1	1.83E+08	1.83E+08	950	1.83E+08	20	12.7789	6.21004
Nc-1-IP_pe	chr1	1.83E+08	1.83E+08	449	1.83E+08	11	4.54404	3.2242
Nc-1-IP_pe	chr1	1.84E+08	1.84E+08	530	1.84E+08	9	4.46937	3.41876
Nc-1-IP_pe	chr1	1.84E+08	1.84E+08	342	1.84E+08	10	3.03453	2.46828
Nc-1-IP_pe	chr1	1.84E+08	1.84E+08	312	1.84E+08	16	5.62031	3.24783
Nc-1-IP_pe	chr1	1.84E+08	1.84E+08	862	1.84E+08	10	5.23357	3.76063
Nc-1-IP_pe	chr1	1.84E+08	1.84E+08	305	1.84E+08	11	5.19376	3.60018
Nc-1-IP_pe	chr1	1.84E+08	1.84E+08	345	1.84E+08	10	5.23357	3.76063
Nc-1-IP_pe	chr1	1.84E+08	1.84E+08	287	1.84E+08	12	5.45448	3.61875
Nc-1-IP_pe	chr1	1.84E+08	1.84E+08	260	1.84E+08	12	5.94619	3.9002
Nc-1-IP_pe	chr1	1.84E+08	1.84E+08	287	1.84E+08	13	7.7352	4.78626
Nc-1-IP_pe	chr1	1.84E+08	1.84E+08	398	1.84E+08	12	3.16137	2.39719
Nc-1-IP_pe	chr1	1.84E+08	1.84E+08	253	1.84E+08	13	4.86362	3.18015
Nc-1-IP_pe	chr1	1.84E+08	1.84E+08	421	1.84E+08	13	4.41954	2.952
Nc-1-IP_pe	chr1	1.84E+08	1.84E+08	431	1.84E+08	9	4.46568	3.41644
Nc-1-IP_pe	chr1	1.84E+08	1.84E+08	257	1.84E+08	13	4.86362	3.18015
Nc-1-IP_pe	chr1	1.84E+08	1.84E+08	330	1.84E+08	13	5.9487	3.76157
Nc-1-IP_pe	chr1	1.84E+08	1.84E+08	376	1.84E+08	15	9.35725	5.35563
Nc-1-IP_pe	chr1	1.84E+08	1.84E+08	383	1.84E+08	11	5.10577	3.5486
Nc-1-IP_pe	chr1	1.84E+08	1.84E+08	269	1.84E+08	13	5.9487	3.76157
Nc-1-IP_pe	chr1	1.84E+08	1.84E+08	655	1.84E+08	16	9.49293	5.236
Nc-1-IP_pe	chr1	1.84E+08	1.84E+08	397	1.84E+08	12	6.58417	4.27436
Nc-1-IP_pe	chr1	1.84E+08	1.84E+08	479	1.84E+08	23	11.5921	5.06057
Nc-1-IP_pe	chr1	1.84E+08	1.84E+08	606	1.84E+08	12	6.86906	4.44438
Nc-1-IP_pe	chr1	1.84E+08	1.84E+08	431	1.84E+08	13	5.9487	3.76157
Nc-1-IP_pe	chr1	1.84E+08	1.84E+08	303	1.84E+08	9	3.27326	2.68684
Nc-1-IP_pe	chr1	1.84E+08	1.84E+08	451	1.84E+08	12	3.83688	2.74114
Nc-1-IP_pe	chr1	1.84E+08	1.84E+08	256	1.84E+08	12	6.58417	4.27436
Nc-1-IP_pe	chr1	1.84E+08	1.84E+08	266	1.84E+08	10	4.39571	3.25288
Nc-1-IP_pe	chr1	1.85E+08	1.85E+08	473	1.85E+08	9	4.46937	3.41876
Nc-1-IP_pe	chr1	1.85E+08	1.85E+08	392	1.85E+08	11	4.35236	3.11555
Nc-1-IP_pe	chr1	1.85E+08	1.85E+08	496	1.85E+08	17	5.14803	2.94914
Nc-1-IP_pe	chr1	1.85E+08	1.85E+08	410	1.85E+08	9	3.27326	2.68684
Nc-1-IP_pe	chr1	1.85E+08	1.85E+08	480	1.85E+08	18	5.33197	2.94862
Nc-1-IP_pe	chr1	1.85E+08	1.85E+08	326	1.85E+08	10	5.23357	3.76063
Nc-1-IP_pe	chr1	1.85E+08	1.85E+08	309	1.85E+08	11	4.54404	3.2242
Nc-1-IP_pe	chr1	1.85E+08	1.85E+08	338	1.85E+08	9	4.46937	3.41876

Nc-1-IP_pe	chr1	1.85E+08	1.85E+08	481	1.85E+08	11	4.54404	3.2242
Nc-1-IP_pe	chr1	1.85E+08	1.85E+08	370	1.85E+08	14	4.21449	2.76599
Nc-1-IP_pe	chr1	1.85E+08	1.85E+08	407	1.85E+08	12	3.58823	2.61305
Nc-1-IP_pe	chr1	1.85E+08	1.85E+08	390	1.85E+08	11	6.03439	4.10251
Nc-1-IP_pe	chr1	1.85E+08	1.85E+08	337	1.85E+08	13	7.7352	4.78626
Nc-1-IP_pe	chr1	1.85E+08	1.85E+08	257	1.85E+08	10	5.23357	3.76063
Nc-1-IP_pe	chr1	1.85E+08	1.85E+08	528	1.85E+08	13	7.7352	4.78626
Nc-1-IP_pe	chr1	1.85E+08	1.85E+08	270	1.85E+08	9	4.25966	3.28797
Nc-1-IP_pe	chr1	1.85E+08	1.85E+08	321	1.85E+08	12	6.86906	4.44438
Nc-1-IP_pe	chr1	1.85E+08	1.85E+08	273	1.85E+08	12	6.86906	4.44438
Nc-1-IP_pe	chr1	1.85E+08	1.85E+08	293	1.85E+08	13	5.86991	3.71823
Nc-1-IP_pe	chr1	1.85E+08	1.85E+08	267	1.85E+08	10	4.39571	3.25288
Nc-1-IP_pe	chr1	1.85E+08	1.85E+08	311	1.85E+08	11	5.19376	3.60018
Nc-1-IP_pe	chr1	1.85E+08	1.85E+08	451	1.85E+08	10	4.99888	3.61677
Nc-1-IP_pe	chr1	1.86E+08	1.86E+08	600	1.86E+08	10	5.23357	3.76063
Nc-1-IP_pe	chr1	1.86E+08	1.86E+08	283	1.86E+08	13	5.29187	3.40559
Nc-1-IP_pe	chr1	1.86E+08	1.86E+08	282	1.86E+08	11	3.64839	2.72584
Nc-1-IP_pe	chr1	1.86E+08	1.86E+08	274	1.86E+08	12	6.58417	4.27436
Nc-1-IP_pe	chr1	1.86E+08	1.86E+08	336	1.86E+08	12	6.31185	4.11348
Nc-1-IP_pe	chr1	1.86E+08	1.86E+08	292	1.86E+08	12	2.87625	2.25568
Nc-1-IP_pe	chr1	1.86E+08	1.86E+08	300	1.86E+08	9	4.46937	3.41876
Nc-1-IP_pe	chr1	1.86E+08	1.86E+08	320	1.86E+08	10	5.23357	3.76063
Nc-1-IP_pe	chr1	1.86E+08	1.86E+08	330	1.86E+08	11	4.54404	3.2242
Nc-1-IP_pe	chr1	1.86E+08	1.86E+08	256	1.86E+08	15	4.40338	2.77622
Nc-1-IP_pe	chr1	1.86E+08	1.86E+08	837	1.86E+08	11	4.54404	3.2242
Nc-1-IP_pe	chr1	1.86E+08	1.86E+08	339	1.86E+08	11	2.96397	2.36091
Nc-1-IP_pe	chr1	1.86E+08	1.86E+08	285	1.86E+08	13	7.42513	4.60316
Nc-1-IP_pe	chr1	1.87E+08	1.87E+08	254	1.87E+08	9	4.35492	3.34727
Nc-1-IP_pe	chr1	1.87E+08	1.87E+08	750	1.87E+08	12	6.86906	4.44438
Nc-1-IP_pe	chr1	1.87E+08	1.87E+08	466	1.87E+08	14	8.63073	5.12814
Nc-1-IP_pe	chr1	1.87E+08	1.87E+08	1514	1.87E+08	17	9.42237	5.01057
Nc-1-IP_pe	chr1	1.87E+08	1.87E+08	616	1.87E+08	14	6.06519	3.69269
Nc-1-IP_pe	chr1	1.87E+08	1.87E+08	896	1.87E+08	14	7.34079	4.38651
Nc-1-IP_pe	chr1	1.88E+08	1.88E+08	611	1.88E+08	12	6.58417	4.27436
Nc-1-IP_pe	chr1	1.88E+08	1.88E+08	520	1.88E+08	10	4.39571	3.25288
Nc-1-IP_pe	chr1	1.88E+08	1.88E+08	278	1.88E+08	11	4.54404	3.2242
Nc-1-IP_pe	chr1	1.88E+08	1.88E+08	267	1.88E+08	9	4.46937	3.41876
Nc-1-IP_pe	chr1	1.88E+08	1.88E+08	255	1.88E+08	10	5.23357	3.76063
Nc-1-IP_pe	chr1	1.88E+08	1.88E+08	330	1.88E+08	15	6.39297	3.73508
Nc-1-IP_pe	chr1	1.88E+08	1.88E+08	274	1.88E+08	13	6.62436	4.14003
Nc-1-IP_pe	chr1	1.88E+08	1.88E+08	259	1.88E+08	10	5.23357	3.76063
Nc-1-IP_pe	chr1	1.89E+08	1.89E+08	519	1.89E+08	11	6.03439	4.10251
Nc-1-IP_pe	chr1	1.89E+08	1.89E+08	305	1.89E+08	11	6.03439	4.10251
Nc-1-IP_pe	chr1	1.89E+08	1.89E+08	263	1.89E+08	14	6.69566	4.03025
Nc-1-IP_pe	chr1	1.89E+08	1.89E+08	339	1.89E+08	10	3.89064	2.95552

Nc-1-IP_pe	chr1	1.89E+08	1.89E+08	277	1.89E+08	12	5.45448	3.61875
Nc-1-IP_pe	chr1	1.89E+08	1.89E+08	282	1.89E+08	12	6.58417	4.27436
Nc-1-IP_pe	chr1	1.89E+08	1.89E+08	580	1.89E+08	14	7.54286	4.50023
Nc-1-IP_pe	chr1	1.89E+08	1.89E+08	501	1.89E+08	10	3.89064	2.95552
Nc-1-IP_pe	chr1	1.89E+08	1.89E+08	289	1.89E+08	10	4.47495	3.30017
Nc-1-IP_pe	chr1	1.89E+08	1.89E+08	393	1.89E+08	13	5.9487	3.76157
Nc-1-IP_pe	chr1	1.89E+08	1.89E+08	293	1.89E+08	14	5.51541	3.4073
Nc-1-IP_pe	chr1	1.89E+08	1.89E+08	632	1.89E+08	11	4.06403	2.95415
Nc-1-IP_pe	chr1	1.89E+08	1.89E+08	257	1.89E+08	10	5.23357	3.76063
Nc-1-IP_pe	chr1	1.89E+08	1.89E+08	436	1.89E+08	13	3.67377	2.58159
Nc-1-IP_pe	chr1	1.89E+08	1.89E+08	680	1.89E+08	12	6.58417	4.27436
Nc-1-IP_pe	chr1	1.89E+08	1.89E+08	436	1.89E+08	12	6.31185	4.11348
Nc-1-IP_pe	chr1	1.9E+08	1.9E+08	309	1.9E+08	10	3.89064	2.95552
Nc-1-IP_pe	chr1	1.9E+08	1.9E+08	786	1.9E+08	14	7.42849	4.43574
Nc-1-IP_pe	chr1	1.9E+08	1.9E+08	376	1.9E+08	13	3.35769	2.42919
Nc-1-IP_pe	chr1	1.9E+08	1.9E+08	431	1.9E+08	31	13.4578	4.71703
Nc-1-IP_pe	chr1	1.9E+08	1.9E+08	420	1.9E+08	13	7.34205	4.55444
Nc-1-IP_pe	chr1	1.9E+08	1.9E+08	268	1.9E+08	12	6.58417	4.27436
Nc-1-IP_pe	chr1	1.9E+08	1.9E+08	365	1.9E+08	10	5.23357	3.76063
Nc-1-IP_pe	chr1	1.9E+08	1.9E+08	363	1.9E+08	12	6.86906	4.44438
Nc-1-IP_pe	chr1	1.91E+08	1.91E+08	476	1.91E+08	9	3.27326	2.68684
Nc-1-IP_pe	chr1	1.91E+08	1.91E+08	262	1.91E+08	8	3.74505	3.07688
Nc-1-IP_pe	chr1	1.91E+08	1.91E+08	386	1.91E+08	14	8.63073	5.12814
Nc-1-IP_pe	chr1	1.91E+08	1.91E+08	394	1.91E+08	8	3.74505	3.07688
Nc-1-IP_pe	chr1	1.91E+08	1.91E+08	611	1.91E+08	11	6.03439	4.10251
Nc-1-IP_pe	chr1	1.91E+08	1.91E+08	267	1.91E+08	8	3.0402	2.6319
Nc-1-IP_pe	chr1	1.91E+08	1.91E+08	409	1.91E+08	14	7.42849	4.43574
Nc-1-IP_pe	chr1	1.91E+08	1.91E+08	762	1.91E+08	11	4.54404	3.2242
Nc-1-IP_pe	chr1	1.91E+08	1.91E+08	267	1.91E+08	9	4.46937	3.41876
Nc-1-IP_pe	chr1	1.91E+08	1.91E+08	867	1.91E+08	12	6.86906	4.44438
Nc-1-IP_pe	chr1	1.92E+08	1.92E+08	298	1.92E+08	10	5.23357	3.76063
Nc-1-IP_pe	chr1	1.92E+08	1.92E+08	601	1.92E+08	8	3.74505	3.07688
Nc-1-IP_pe	chr1	1.92E+08	1.92E+08	269	1.92E+08	13	6.54342	4.09407
Nc-1-IP_pe	chr1	1.92E+08	1.92E+08	427	1.92E+08	8	3.74505	3.07688
Nc-1-IP_pe	chr1	1.92E+08	1.92E+08	556	1.92E+08	8	3.74505	3.07688
Nc-1-IP_pe	chr1	1.92E+08	1.92E+08	644	1.92E+08	10	5.23357	3.76063
Nc-1-IP_pe	chr1	1.92E+08	1.92E+08	271	1.92E+08	14	7.42849	4.43574
Nc-1-IP_pe	chr1	1.92E+08	1.92E+08	264	1.92E+08	14	5.03085	3.16286
Nc-1-IP_pe	chr1	1.92E+08	1.92E+08	454	1.92E+08	15	6.78905	3.93887
Nc-1-IP_pe	chr1	1.93E+08	1.93E+08	336	1.93E+08	17	7.52577	4.03901
Nc-1-IP_pe	chr1	1.93E+08	1.93E+08	508	1.93E+08	10	3.89064	2.95552
Nc-1-IP_pe	chr1	1.93E+08	1.93E+08	265	1.93E+08	10	5.23357	3.76063
Nc-1-IP_pe	chr1	1.93E+08	1.93E+08	346	1.93E+08	14	8.63073	5.12814
Nc-1-IP_pe	chr1	1.93E+08	1.93E+08	266	1.93E+08	11	6.03439	4.10251
Nc-1-IP_pe	chr1	1.93E+08	1.93E+08	519	1.93E+08	10	4.39571	3.25288

Nc-1-IP_pe	chr1	1.93E+08	1.93E+08	1629	1.93E+08	28	11.2618	4.27481
Nc-1-IP_pe	chr1	1.93E+08	1.93E+08	501	1.93E+08	13	6.62436	4.14003
Nc-1-IP_pe	chr1	1.93E+08	1.93E+08	598	1.93E+08	13	6.92473	4.312
Nc-1-IP_pe	chr1	1.93E+08	1.93E+08	276	1.93E+08	12	4.51041	3.09723
Nc-1-IP_pe	chr1	1.93E+08	1.93E+08	488	1.93E+08	14	4.60011	2.95114
Nc-1-IP_pe	chr1	1.93E+08	1.93E+08	354	1.93E+08	12	6.58417	4.27436
Nc-1-IP_pe	chr1	1.93E+08	1.93E+08	293	1.93E+08	11	5.03835	3.5092
Nc-1-IP_pe	chr1	1.93E+08	1.93E+08	486	1.93E+08	13	7.7352	4.78626
Nc-1-IP_pe	chr1	1.94E+08	1.94E+08	288	1.94E+08	13	4.02562	2.7544
Nc-1-IP_pe	chr1	1.94E+08	1.94E+08	490	1.94E+08	13	7.42513	4.60316
Nc-1-IP_pe	chr1	1.94E+08	1.94E+08	320	1.94E+08	8	3.74505	3.07688
Nc-1-IP_pe	chr1	1.94E+08	1.94E+08	266	1.94E+08	8	3.74505	3.07688
Nc-1-IP_pe	chr1	1.94E+08	1.94E+08	488	1.94E+08	14	8.63073	5.12814
Nc-1-IP_pe	chr1	1.94E+08	1.94E+08	302	1.94E+08	12	4.70082	3.20033
Nc-1-IP_pe	chr1	1.94E+08	1.94E+08	594	1.94E+08	13	5.15976	3.33548
Nc-1-IP_pe	chr1	1.95E+08	1.95E+08	374	1.95E+08	10	3.89064	2.95552
Nc-1-IP_pe	chr1	1.95E+08	1.95E+08	566	1.95E+08	14	6.69566	4.03025
Nc-1-IP_pe	chr1	1.95E+08	1.95E+08	447	1.95E+08	7	3.06442	2.73501
Nc-1-IP_pe	chr1	1.95E+08	1.95E+08	757	1.95E+08	17	11.4763	6.15376
Nc-1-IP_pe	chr1	1.95E+08	1.95E+08	413	1.95E+08	10	3.08907	2.49869
Nc-1-IP_pe	chr1	1.95E+08	1.95E+08	491	1.95E+08	13	7.7352	4.78626
Nc-1-IP_pe	chr1	1.96E+08	1.96E+08	294	1.96E+08	12	6.86906	4.44438
Nc-1-IP_pe	chr1	1.96E+08	1.96E+08	302	1.96E+08	10	3.4604	2.70797
Nc-1-IP_pe	chr1	1.96E+08	1.96E+08	355	1.96E+08	10	3.4604	2.70797
Nc-1-IP_pe	chr1	1.96E+08	1.96E+08	375	1.96E+08	12	6.86906	4.44438
Nc-1-IP_pe	chr1	1.96E+08	1.96E+08	293	1.96E+08	14	7.9745	4.74633
Nc-1-IP_pe	chr1	1.96E+08	1.96E+08	503	1.96E+08	16	4.96462	2.94973
Nc-1-IP_pe	chr1	1.97E+08	1.97E+08	1183	1.97E+08	15	6.19428	3.63445
Nc-1-IP_pe	chr1	1.97E+08	1.97E+08	439	1.97E+08	10	3.89064	2.95552
Nc-1-IP_pe	chr1	1.97E+08	1.97E+08	485	1.97E+08	13	7.7352	4.78626
Nc-1-IP_pe	chr1	1.97E+08	1.97E+08	272	1.97E+08	13	7.42513	4.60316
Nc-1-IP_pe	chr1	1.97E+08	1.97E+08	253	1.97E+08	12	5.77527	3.80164
Nc-1-IP_pe	chr1	1.97E+08	1.97E+08	286	1.97E+08	12	6.86906	4.44438
Nc-1-IP_pe	chr1	1.97E+08	1.97E+08	310	1.97E+08	9	3.72192	2.95716
Nc-1-IP_pe	chr1	1.97E+08	1.97E+08	1196	1.97E+08	12	5.15877	3.45264
Nc-1-IP_pe	chr1	1.97E+08	1.97E+08	307	1.97E+08	11	4.54404	3.2242
Nc-1-IP_pe	chr1	1.97E+08	1.97E+08	358	1.97E+08	16	6.33285	3.58457
Nc-1-IP_pe	chr1	1.97E+08	1.97E+08	309	1.97E+08	15	5.22925	3.16116
Nc-1-IP_pe	chr1	1.98E+08	1.98E+08	714	1.98E+08	16	5.82808	3.34463
Nc-1-IP_pe	chr1	1.98E+08	1.98E+08	255	1.98E+08	10	4.39571	3.25288
Nc-1-IP_pe	chr1	1.98E+08	1.98E+08	280	1.98E+08	12	6.58417	4.27436
Nc-1-IP_pe	chr1	1.98E+08	1.98E+08	514	1.98E+08	16	5.82808	3.34463
Nc-1-IP_pe	chr1	1.98E+08	1.98E+08	255	1.98E+08	13	5.9487	3.76157
Nc-1-IP_pe	chr1	1.98E+08	1.98E+08	414	1.98E+08	15	8.26005	4.73146
Nc-1-IP_pe	chr1	1.98E+08	1.98E+08	700	1.98E+08	15	8.26005	4.73146

Nc-1-IP_pe	chr1	1.98E+08	1.98E+08	610	1.98E+08	12	3.83688	2.74114
Nc-1-IP_pe	chr1	1.98E+08	1.98E+08	841	1.98E+08	16	5.37454	3.13479
Nc-1-IP_pe	chr1	1.98E+08	1.98E+08	566	1.98E+08	13	6.06081	3.82353
Nc-1-IP_pe	chr1	1.98E+08	1.98E+08	433	1.98E+08	11	4.23076	3.04719
Nc-1-IP_pe	chr1	1.98E+08	1.98E+08	262	1.98E+08	9	3.72192	2.95716
Nc-1-IP_pe	chr1	1.98E+08	1.98E+08	316	1.98E+08	10	4.99888	3.61677
Nc-1-IP_pe	chr1	1.98E+08	1.98E+08	686	1.98E+08	12	5.84943	3.84431
Nc-1-IP_pe	chr1	1.98E+08	1.98E+08	269	1.98E+08	12	4.24064	2.95299
Nc-1-IP_pe	chr1	1.98E+08	1.98E+08	368	1.98E+08	11	6.03439	4.10251
Nc-1-IP_pe	chr1	1.98E+08	1.98E+08	289	1.98E+08	11	3.28475	2.53029
Nc-1-IP_pe	chr1	1.98E+08	1.98E+08	279	1.98E+08	14	6.69566	4.03025
Nc-1-IP_pe	chr1	1.98E+08	1.98E+08	271	1.98E+08	10	5.23357	3.76063
Nc-1-IP_pe	chr1	1.98E+08	1.98E+08	712	1.98E+08	30	10.9504	3.972
Nc-1-IP_pe	chr1	1.98E+08	1.98E+08	580	1.98E+08	16	6.33285	3.58457
Nc-1-IP_pe	chr1	1.98E+08	1.98E+08	515	1.98E+08	20	7.43645	3.64378
Nc-1-IP_pe	chr1	1.99E+08	1.99E+08	265	1.99E+08	14	7.9745	4.74633
Nc-1-IP_pe	chr1	1.99E+08	1.99E+08	296	1.99E+08	8	3.08755	2.66145
Nc-1-IP_pe	chr1	1.99E+08	1.99E+08	263	1.99E+08	11	3.64839	2.72584
Nc-1-IP_pe	chr1	1.99E+08	1.99E+08	551	1.99E+08	11	3.64839	2.72584
Nc-1-IP_pe	chr1	1.99E+08	1.99E+08	425	1.99E+08	23	8.92223	3.93219
Nc-1-IP_pe	chr1	1.99E+08	1.99E+08	265	1.99E+08	10	5.23357	3.76063
Nc-1-IP_pe	chr1	1.99E+08	1.99E+08	823	1.99E+08	18	6.15736	3.29676
Nc-1-IP_pe	chr1	1.99E+08	1.99E+08	285	1.99E+08	13	6.54342	4.09407
Nc-1-IP_pe	chr1	1.99E+08	1.99E+08	430	1.99E+08	17	5.54967	3.12324
Nc-1-IP_pe	chr1	1.99E+08	1.99E+08	431	1.99E+08	11	4.61014	3.26192
Nc-1-IP_pe	chr1	1.99E+08	1.99E+08	335	1.99E+08	14	3.86727	2.6027
Nc-1-IP_pe	chr1	1.99E+08	1.99E+08	314	1.99E+08	12	3.83688	2.74114
Nc-1-IP_pe	chr1	1.99E+08	1.99E+08	348	1.99E+08	13	4.41954	2.952
Nc-1-IP_pe	chr1	1.99E+08	1.99E+08	288	1.99E+08	10	3.08907	2.49869
Nc-1-IP_pe	chr1	1.99E+08	1.99E+08	267	1.99E+08	17	7.06589	3.81697
Nc-1-IP_pe	chr1	1.99E+08	1.99E+08	369	1.99E+08	11	4.06403	2.95415
Nc-1-IP_pe	chr1	2E+08	2E+08	271	2E+08	18	7.1522	3.73811
Nc-1-IP_pe	chr1	2E+08	2E+08	270	2E+08	9	4.25966	3.28797
Nc-1-IP_pe	chr1	2E+08	2E+08	271	2E+08	10	3.08907	2.49869
Nc-1-IP_pe	chr1	2E+08	2E+08	414	2E+08	11	5.77463	3.94557
Nc-1-IP_pe	chr1	2E+08	2E+08	287	2E+08	11	3.64839	2.72584
Nc-1-IP_pe	chr1	2E+08	2E+08	361	2E+08	18	12.4731	6.49564
Nc-1-IP_pe	chr1	2E+08	2E+08	340	2E+08	11	4.99304	3.4828
Nc-1-IP_pe	chr1	2E+08	2E+08	308	2E+08	11	4.54404	3.2242
Nc-1-IP_pe	chr1	2E+08	2E+08	393	2E+08	11	5.24175	3.6284
Nc-1-IP_pe	chr1	2E+08	2E+08	465	2E+08	12	3.16137	2.39719
Nc-1-IP_pe	chr1	2E+08	2E+08	263	2E+08	11	5.52654	3.79706
Nc-1-IP_pe	chr1	2E+08	2E+08	291	2E+08	12	5.61153	3.70794
Nc-1-IP_pe	chr1	2E+08	2E+08	454	2E+08	11	5.7051	3.90381
Nc-1-IP_pe	chr1	2E+08	2E+08	314	2E+08	13	6.36468	3.99316

Nc-1-IP_pe	chr1	2E+08	2E+08	455	2E+08	18	8.37884	4.31591
Nc-1-IP_pe	chr1	2E+08	2E+08	352	2E+08	11	3.40056	2.59215
Nc-1-IP_pe	chr1	2E+08	2E+08	736	2E+08	20	9.21699	4.428
Nc-1-IP_pe	chr1	2E+08	2E+08	460	2E+08	21	8.10596	3.8173
Nc-1-IP_pe	chr1	2E+08	2E+08	376	2E+08	14	7.9745	4.74633
Nc-1-IP_pe	chr1	2E+08	2E+08	265	2E+08	13	6.02837	3.80557
Nc-1-IP_pe	chr1	2E+08	2E+08	346	2E+08	13	4.86362	3.18015
Nc-1-IP_pe	chr1	2E+08	2E+08	254	2E+08	12	4.24064	2.95299
Nc-1-IP_pe	chr1	2E+08	2E+08	686	2E+08	14	3.86727	2.6027
Nc-1-IP_pe	chr1	2E+08	2E+08	583	2E+08	14	5.99644	3.65653
Nc-1-IP_pe	chr1	2E+08	2E+08	1102	2E+08	16	6.51374	3.67221
Nc-1-IP_pe	chr1	2E+08	2E+08	491	2E+08	12	4.76841	3.23719
Nc-1-IP_pe	chr1	2E+08	2E+08	311	2E+08	15	3.74763	2.48304
Nc-1-IP_pe	chr1	2E+08	2E+08	308	2E+08	12	4.24064	2.95299
Nc-1-IP_pe	chr1	2E+08	2E+08	506	2E+08	17	7.36791	3.96218
Nc-1-IP_pe	chr1	2E+08	2E+08	320	2E+08	11	6.03439	4.10251
Nc-1-IP_pe	chr1	2E+08	2E+08	408	2E+08	19	6.32622	3.27682
Nc-1-IP_pe	chr1	2E+08	2E+08	297	2E+08	11	4.35236	3.11555
Nc-1-IP_pe	chr1	2E+08	2E+08	373	2E+08	16	5.82808	3.34463
Nc-1-IP_pe	chr1	2E+08	2E+08	451	2E+08	14	5.03085	3.16286
Nc-1-IP_pe	chr1	2E+08	2E+08	432	2E+08	12	3.83688	2.74114
Nc-1-IP_pe	chr1	2E+08	2E+08	599	2E+08	14	5.51541	3.4073
Nc-1-IP_pe	chr1	2E+08	2E+08	386	2E+08	13	4.86362	3.18015
Nc-1-IP_pe	chr1	2E+08	2E+08	579	2E+08	11	3.64839	2.72584
Nc-1-IP_pe	chr1	2E+08	2E+08	304	2E+08	17	9.72281	5.17231
Nc-1-IP_pe	chr1	2E+08	2E+08	260	2E+08	12	5.23082	3.49289
Nc-1-IP_pe	chr1	2E+08	2E+08	341	2E+08	13	5.42865	3.47872
Nc-1-IP_pe	chr1	2E+08	2E+08	402	2E+08	15	6.46757	3.77313
Nc-1-IP_pe	chr1	2E+08	2E+08	637	2E+08	12	5.94619	3.9002
Nc-1-IP_pe	chr1	2E+08	2E+08	302	2E+08	10	4.51818	3.32604
Nc-1-IP_pe	chr1	2E+08	2E+08	325	2E+08	18	9.1091	4.6774
Nc-1-IP_pe	chr1	2E+08	2E+08	262	2E+08	14	6.69566	4.03025
Nc-1-IP_pe	chr1	2E+08	2E+08	519	2E+08	14	7.34079	4.38651
Nc-1-IP_pe	chr1	2E+08	2E+08	296	2E+08	10	4.62142	3.388
Nc-1-IP_pe	chr1	2E+08	2E+08	262	2E+08	11	4.06403	2.95415
Nc-1-IP_pe	chr1	2E+08	2E+08	285	2E+08	13	4.02562	2.7544
Nc-1-IP_pe	chr1	2E+08	2E+08	359	2E+08	21	5.88587	2.94733
Nc-1-IP_pe	chr1	2E+08	2E+08	378	2E+08	13	5.9487	3.76157
Nc-1-IP_pe	chr1	2E+08	2E+08	253	2E+08	10	5.12294	3.69268
Nc-1-IP_pe	chr1	2.01E+08	2.01E+08	339	2.01E+08	11	4.49162	3.19439
Nc-1-IP_pe	chr1	2.01E+08	2.01E+08	318	2.01E+08	14	6.61011	3.98382
Nc-1-IP_pe	chr1	2.01E+08	2.01E+08	696	2.01E+08	13	5.9487	3.76157
Nc-1-IP_pe	chr1	2.01E+08	2.01E+08	475	2.01E+08	13	6.36468	3.99316
Nc-1-IP_pe	chr1	2.01E+08	2.01E+08	389	2.01E+08	12	6.63013	4.30168
Nc-1-IP_pe	chr1	2.01E+08	2.01E+08	294	2.01E+08	12	6.6095	4.28941

Nc-1-IP_pe	chr1	2.01E+08	2.01E+08	311	2.01E+08	9	4.25966	3.28797
Nc-1-IP_pe	chr1	2.01E+08	2.01E+08	293	2.01E+08	11	5.81652	3.97078
Nc-1-IP_pe	chr1	2.01E+08	2.01E+08	366	2.01E+08	23	7.89844	3.53777
Nc-1-IP_pe	chr1	2.01E+08	2.01E+08	503	2.01E+08	15	8.14428	4.66715
Nc-1-IP_pe	chr1	2.01E+08	2.01E+08	312	2.01E+08	19	7.29128	3.68799
Nc-1-IP_pe	chr1	2.01E+08	2.01E+08	374	2.01E+08	13	7.12853	4.42991
Nc-1-IP_pe	chr1	2.01E+08	2.01E+08	383	2.01E+08	14	5.69896	3.50164
Nc-1-IP_pe	chr1	2.01E+08	2.01E+08	259	2.01E+08	11	5.13077	3.56323
Nc-1-IP_pe	chr1	2.01E+08	2.01E+08	282	2.01E+08	14	8.32526	4.94932
Nc-1-IP_pe	chr1	2.01E+08	2.01E+08	1095	2.01E+08	20	5.34663	2.81336
Nc-1-IP_pe	chr1	2.01E+08	2.01E+08	305	2.01E+08	14	4.60011	2.95114
Nc-1-IP_pe	chr1	2.01E+08	2.01E+08	471	2.01E+08	11	5.19376	3.60018
Nc-1-IP_pe	chr1	2.01E+08	2.01E+08	284	2.01E+08	14	5.51541	3.4073
Nc-1-IP_pe	chr1	2.01E+08	2.01E+08	617	2.01E+08	16	9.24948	5.10026
Nc-1-IP_pe	chr1	2.01E+08	2.01E+08	342	2.01E+08	11	5.77463	3.94557
Nc-1-IP_pe	chr1	2.01E+08	2.01E+08	267	2.01E+08	12	6.6095	4.28941
Nc-1-IP_pe	chr1	2.01E+08	2.01E+08	1720	2.01E+08	18	5.83846	3.16035
Nc-1-IP_pe	chr1	2.01E+08	2.01E+08	438	2.01E+08	15	5.64529	3.36206
Nc-1-IP_pe	chr1	2.01E+08	2.01E+08	279	2.01E+08	17	4.13464	2.52661
Nc-1-IP_pe	chr1	2.01E+08	2.01E+08	288	2.01E+08	17	5.85019	3.25611
Nc-1-IP_pe	chr1	2.01E+08	2.01E+08	1276	2.01E+08	19	5.60545	2.98361
Nc-1-IP_pe	chr1	2.01E+08	2.01E+08	643	2.01E+08	15	4.7819	2.95039
Nc-1-IP_pe	chr1	2.01E+08	2.01E+08	449	2.01E+08	21	5.2077	2.70109
Nc-1-IP_pe	chr1	2.01E+08	2.01E+08	1088	2.01E+08	27	8.12936	3.29991
Nc-1-IP_pe	chr1	2.01E+08	2.01E+08	276	2.01E+08	22	8.24538	3.76835
Nc-1-IP_pe	chr1	2.01E+08	2.01E+08	840	2.01E+08	14	3.55308	2.45762
Nc-1-IP_pe	chr1	2.01E+08	2.01E+08	604	2.01E+08	13	6.50707	4.07348
Nc-1-IP_pe	chr1	2.01E+08	2.01E+08	273	2.01E+08	11	3.28475	2.53029
Nc-1-IP_pe	chr1	2.01E+08	2.01E+08	302	2.01E+08	16	6.95583	3.89005
Nc-1-IP_pe	chr1	2.01E+08	2.01E+08	328	2.01E+08	14	5.98179	3.64885
Nc-1-IP_pe	chr1	2.01E+08	2.01E+08	259	2.01E+08	16	5.82808	3.34463
Nc-1-IP_pe	chr1	2.01E+08	2.01E+08	343	2.01E+08	17	5.14803	2.94914
Nc-1-IP_pe	chr1	2.01E+08	2.01E+08	496	2.01E+08	17	7.08058	3.82398
Nc-1-IP_pe	chr1	2.01E+08	2.01E+08	330	2.01E+08	14	5.43414	3.36584
Nc-1-IP_pe	chr1	2.01E+08	2.01E+08	795	2.01E+08	13	3.07238	2.29378
Nc-1-IP_pe	chr1	2.01E+08	2.01E+08	321	2.01E+08	13	4.80086	3.14755
Nc-1-IP_pe	chr1	2.01E+08	2.01E+08	570	2.01E+08	17	7.54182	4.04685
Nc-1-IP_pe	chr1	2.01E+08	2.01E+08	627	2.01E+08	12	3.96559	2.80816
Nc-1-IP_pe	chr1	2.01E+08	2.01E+08	676	2.01E+08	14	5.00888	3.15193
Nc-1-IP_pe	chr1	2.01E+08	2.01E+08	353	2.01E+08	16	6.75928	3.79256
Nc-1-IP_pe	chr1	2.01E+08	2.01E+08	364	2.01E+08	14	5.47279	3.38553
Nc-1-IP_pe	chr1	2.01E+08	2.01E+08	844	2.01E+08	18	7.47506	3.88659
Nc-1-IP_pe	chr1	2.01E+08	2.01E+08	570	2.01E+08	13	4.24481	2.8638
Nc-1-IP_pe	chr1	2.01E+08	2.01E+08	377	2.01E+08	18	8.67794	4.46241
Nc-1-IP_pe	chr1	2.01E+08	2.01E+08	454	2.01E+08	12	4.28158	2.97474

Nc-1-IP_pe	chr1	2.01E+08	2.01E+08	318	2.01E+08	14	3.00733	2.21111
Nc-1-IP_pe	chr1	2.01E+08	2.01E+08	511	2.01E+08	26	12.2933	4.9149
Nc-1-IP_pe	chr1	2.01E+08	2.01E+08	435	2.01E+08	19	8.17534	4.08393
Nc-1-IP_pe	chr1	2.01E+08	2.01E+08	955	2.01E+08	14	5.18557	3.24018
Nc-1-IP_pe	chr1	2.01E+08	2.01E+08	362	2.01E+08	12	4.32611	2.99846
Nc-1-IP_pe	chr1	2.01E+08	2.01E+08	495	2.01E+08	18	8.37884	4.31591
Nc-1-IP_pe	chr1	2.01E+08	2.01E+08	272	2.01E+08	14	4.21449	2.76599
Nc-1-IP_pe	chr1	2.01E+08	2.01E+08	355	2.01E+08	13	5.30533	3.41277
Nc-1-IP_pe	chr1	2.01E+08	2.01E+08	324	2.01E+08	13	5.42865	3.47872
Nc-1-IP_pe	chr1	2.01E+08	2.01E+08	417	2.01E+08	14	4.21449	2.76599
Nc-1-IP_pe	chr1	2.01E+08	2.01E+08	770	2.01E+08	11	4.11366	2.98176
Nc-1-IP_pe	chr1	2.01E+08	2.01E+08	890	2.01E+08	13	3.07238	2.29378
Nc-1-IP_pe	chr1	2.01E+08	2.01E+08	453	2.01E+08	11	4.13673	2.99462
Nc-1-IP_pe	chr1	2.01E+08	2.01E+08	328	2.01E+08	14	5.51541	3.4073
Nc-1-IP_pe	chr1	2.01E+08	2.01E+08	321	2.01E+08	14	3.00733	2.21111
Nc-1-IP_pe	chr1	2.01E+08	2.01E+08	613	2.01E+08	15	6.10627	3.59023
Nc-1-IP_pe	chr1	2.01E+08	2.01E+08	264	2.01E+08	15	4.40338	2.77622
Nc-1-IP_pe	chr1	2.01E+08	2.01E+08	294	2.01E+08	20	9.39627	4.511
Nc-1-IP_pe	chr1	2.01E+08	2.01E+08	572	2.01E+08	10	3.4604	2.70797
Nc-1-IP_pe	chr1	2.01E+08	2.01E+08	315	2.01E+08	13	4.86362	3.18015
Nc-1-IP_pe	chr1	2.01E+08	2.01E+08	298	2.01E+08	14	6.69566	4.03025
Nc-1-IP_pe	chr1	2.01E+08	2.01E+08	308	2.01E+08	14	4.21449	2.76599
Nc-1-IP_pe	chr1	2.01E+08	2.01E+08	300	2.01E+08	11	4.27885	3.07417
Nc-1-IP_pe	chr1	2.01E+08	2.01E+08	648	2.01E+08	16	7.53857	4.18505
Nc-1-IP_pe	chr1	2.01E+08	2.01E+08	976	2.01E+08	17	5.54967	3.12324
Nc-1-IP_pe	chr1	2.01E+08	2.01E+08	516	2.01E+08	11	4.10219	2.97537
Nc-1-IP_pe	chr1	2.01E+08	2.01E+08	554	2.01E+08	15	6.19428	3.63445
Nc-1-IP_pe	chr1	2.01E+08	2.01E+08	446	2.01E+08	13	4.5945	3.0412
Nc-1-IP_pe	chr1	2.01E+08	2.01E+08	627	2.01E+08	19	9.31591	4.62223
Nc-1-IP_pe	chr1	2.01E+08	2.01E+08	260	2.01E+08	18	4.79108	2.72889
Nc-1-IP_pe	chr1	2.01E+08	2.01E+08	302	2.01E+08	14	5.18557	3.24018
Nc-1-IP_pe	chr1	2.01E+08	2.01E+08	263	2.01E+08	16	5.73763	3.30235
Nc-1-IP_pe	chr1	2.01E+08	2.01E+08	263	2.01E+08	12	4.16211	2.91142
Nc-1-IP_pe	chr1	2.01E+08	2.01E+08	337	2.01E+08	21	6.2632	3.08808
Nc-1-IP_pe	chr1	2.01E+08	2.01E+08	292	2.01E+08	16	6.1093	3.47747
Nc-1-IP_pe	chr1	2.01E+08	2.01E+08	333	2.01E+08	16	7.56205	4.19712
Nc-1-IP_pe	chr1	2.01E+08	2.01E+08	436	2.01E+08	12	5.23082	3.49289
Nc-1-IP_pe	chr1	2.01E+08	2.01E+08	299	2.01E+08	16	6.24025	3.54005
Nc-1-IP_pe	chr1	2.01E+08	2.01E+08	256	2.01E+08	15	6.16217	3.61829
Nc-1-IP_pe	chr1	2.01E+08	2.01E+08	672	2.01E+08	16	7.49289	4.16161
Nc-1-IP_pe	chr1	2.01E+08	2.01E+08	297	2.01E+08	15	2.9484	2.13875
Nc-1-IP_pe	chr1	2.01E+08	2.01E+08	501	2.01E+08	14	4.21449	2.76599
Nc-1-IP_pe	chr1	2.01E+08	2.01E+08	272	2.01E+08	10	3.53586	2.751
Nc-1-IP_pe	chr1	2.01E+08	2.01E+08	577	2.01E+08	12	3.16137	2.39719
Nc-1-IP_pe	chr1	2.01E+08	2.01E+08	271	2.01E+08	16	6.33285	3.58457

Nc-1-IP_pe	chr1	2.01E+08	2.01E+08	643	2.01E+08	13	4.67336	3.0817
Nc-1-IP_pe	chr1	2.01E+08	2.01E+08	504	2.01E+08	13	3.07238	2.29378
Nc-1-IP_pe	chr1	2.01E+08	2.01E+08	524	2.01E+08	13	4.30582	2.8945
Nc-1-IP_pe	chr1	2.01E+08	2.01E+08	286	2.01E+08	17	6.47874	3.54137
Nc-1-IP_pe	chr1	2.01E+08	2.01E+08	292	2.01E+08	11	3.29427	2.53536
Nc-1-IP_pe	chr1	2.01E+08	2.01E+08	528	2.01E+08	12	4.24064	2.95299
Nc-1-IP_pe	chr1	2.01E+08	2.01E+08	411	2.01E+08	23	7.89844	3.53777
Nc-1-IP_pe	chr1	2.01E+08	2.01E+08	625	2.01E+08	13	5.36853	3.44651
Nc-1-IP_pe	chr1	2.01E+08	2.01E+08	614	2.01E+08	14	6.42818	3.88572
Nc-1-IP_pe	chr1	2.01E+08	2.01E+08	434	2.01E+08	15	6.19428	3.63445
Nc-1-IP_pe	chr1	2.01E+08	2.01E+08	1035	2.01E+08	14	4.95172	3.12357
Nc-1-IP_pe	chr1	2.01E+08	2.01E+08	343	2.01E+08	14	6.44441	3.89444
Nc-1-IP_pe	chr1	2.01E+08	2.01E+08	834	2.01E+08	14	4.60011	2.95114
Nc-1-IP_pe	chr1	2.01E+08	2.01E+08	407	2.01E+08	19	8.35089	4.16476
Nc-1-IP_pe	chr1	2.01E+08	2.01E+08	739	2.01E+08	14	5.03198	3.16342
Nc-1-IP_pe	chr1	2.01E+08	2.01E+08	900	2.01E+08	19	8.12837	4.06242
Nc-1-IP_pe	chr1	2.01E+08	2.01E+08	407	2.01E+08	21	9.0131	4.20308
Nc-1-IP_pe	chr1	2.01E+08	2.01E+08	913	2.01E+08	18	5.33197	2.94862
Nc-1-IP_pe	chr1	2.01E+08	2.01E+08	310	2.01E+08	13	5.121	3.315
Nc-1-IP_pe	chr1	2.01E+08	2.01E+08	436	2.01E+08	14	4.21449	2.76599
Nc-1-IP_pe	chr1	2.01E+08	2.01E+08	393	2.01E+08	13	4.78908	3.14145
Nc-1-IP_pe	chr1	2.01E+08	2.01E+08	426	2.01E+08	14	5.75402	3.53012
Nc-1-IP_pe	chr1	2.01E+08	2.01E+08	1136	2.01E+08	14	5.51541	3.4073
Nc-1-IP_pe	chr1	2.01E+08	2.01E+08	578	2.01E+08	15	4.70031	2.91253
Nc-1-IP_pe	chr1	2.01E+08	2.01E+08	770	2.01E+08	14	5.06689	3.18081
Nc-1-IP_pe	chr1	2.01E+08	2.01E+08	285	2.01E+08	13	5.13388	3.3218
Nc-1-IP_pe	chr1	2.01E+08	2.01E+08	679	2.01E+08	16	5.60875	3.24248
Nc-1-IP_pe	chr1	2.01E+08	2.01E+08	473	2.01E+08	12	3.77112	2.7071
Nc-1-IP_pe	chr1	2.01E+08	2.01E+08	544	2.01E+08	16	7.11257	3.96852
Nc-1-IP_pe	chr1	2.01E+08	2.01E+08	340	2.01E+08	14	5.45987	3.37894
Nc-1-IP_pe	chr1	2.01E+08	2.01E+08	524	2.01E+08	15	6.03738	3.55576
Nc-1-IP_pe	chr1	2.01E+08	2.01E+08	405	2.01E+08	14	4.13959	2.7305
Nc-1-IP_pe	chr1	2.01E+08	2.01E+08	263	2.01E+08	13	5.15976	3.33548
Nc-1-IP_pe	chr1	2.01E+08	2.01E+08	356	2.01E+08	15	7.21633	4.16341
Nc-1-IP_pe	chr1	2.01E+08	2.01E+08	872	2.01E+08	13	5.76252	3.65944
Nc-1-IP_pe	chr1	2.01E+08	2.01E+08	306	2.01E+08	11	3.89191	2.85898
Nc-1-IP_pe	chr1	2.01E+08	2.01E+08	612	2.01E+08	14	6.13058	3.7272
Nc-1-IP_pe	chr1	2.01E+08	2.01E+08	643	2.01E+08	14	5.98179	3.64885
Nc-1-IP_pe	chr1	2.01E+08	2.01E+08	253	2.01E+08	10	2.86829	2.37613
Nc-1-IP_pe	chr1	2.01E+08	2.01E+08	470	2.01E+08	16	6.59897	3.7138
Nc-1-IP_pe	chr1	2.01E+08	2.01E+08	417	2.01E+08	13	3.67377	2.58159
Nc-1-IP_pe	chr1	2.01E+08	2.01E+08	508	2.01E+08	18	7.73136	4.00629
Nc-1-IP_pe	chr1	2.01E+08	2.01E+08	419	2.01E+08	15	3.74763	2.48304
Nc-1-IP_pe	chr1	2.01E+08	2.01E+08	404	2.01E+08	15	3.46204	2.35851
Nc-1-IP_pe	chr1	2.01E+08	2.01E+08	675	2.01E+08	17	5.9911	3.31919

Nc-1-IP_pe	chr1	2.01E+08	2.01E+08	698	2.01E+08	14	4.73128	3.01506
Nc-1-IP_pe	chr1	2.01E+08	2.01E+08	780	2.01E+08	19	7.84735	3.93486
Nc-1-IP_pe	chr1	2.01E+08	2.01E+08	383	2.01E+08	17	6.97865	3.77546
Nc-1-IP_pe	chr1	2.01E+08	2.01E+08	400	2.01E+08	16	4.68897	2.82769
Nc-1-IP_pe	chr1	2.01E+08	2.01E+08	254	2.01E+08	14	5.18557	3.24018
Nc-1-IP_pe	chr1	2.01E+08	2.01E+08	376	2.01E+08	13	3.07238	2.29378
Nc-1-IP_pe	chr1	2.01E+08	2.01E+08	534	2.01E+08	16	3.65575	2.38624
Nc-1-IP_pe	chr1	2.01E+08	2.01E+08	272	2.01E+08	17	6.89289	3.73485
Nc-1-IP_pe	chr1	2.01E+08	2.01E+08	464	2.01E+08	18	5.72638	3.11298
Nc-1-IP_pe	chr1	2.01E+08	2.01E+08	1116	2.01E+08	19	4.23334	2.45554
Nc-1-IP_pe	chr1	2.01E+08	2.01E+08	264	2.01E+08	16	5.37454	3.13479
Nc-1-IP_pe	chr1	2.01E+08	2.01E+08	1199	2.01E+08	21	7.10961	3.41419
Nc-1-IP_pe	chr1	2.01E+08	2.01E+08	289	2.01E+08	17	4.44438	2.65333
Nc-1-IP_pe	chr1	2.01E+08	2.01E+08	408	2.01E+08	16	6.89451	3.85952
Nc-1-IP_pe	chr1	2.01E+08	2.01E+08	279	2.01E+08	16	7.44163	4.13536
Nc-1-IP_pe	chr1	2.01E+08	2.01E+08	358	2.01E+08	16	5.37454	3.13479
Nc-1-IP_pe	chr1	2.01E+08	2.01E+08	406	2.01E+08	12	3.47963	2.55766
Nc-1-IP_pe	chr1	2.01E+08	2.01E+08	257	2.01E+08	11	4.30316	3.08784
Nc-1-IP_pe	chr1	2.01E+08	2.01E+08	497	2.01E+08	16	8.15132	4.50466
Nc-1-IP_pe	chr1	2.01E+08	2.01E+08	273	2.01E+08	11	3.49235	2.64145
Nc-1-IP_pe	chr1	2.01E+08	2.01E+08	283	2.01E+08	16	7.08082	3.95257
Nc-1-IP_pe	chr1	2.01E+08	2.01E+08	398	2.01E+08	13	4.78908	3.14145
Nc-1-IP_pe	chr1	2.01E+08	2.01E+08	1986	2.01E+08	28	9.16687	3.56053
Nc-1-IP_pe	chr1	2.01E+08	2.01E+08	306	2.01E+08	17	4.44438	2.65333
Nc-1-IP_pe	chr1	2.01E+08	2.01E+08	289	2.01E+08	14	5.10212	3.19839
Nc-1-IP_pe	chr1	2.01E+08	2.01E+08	279	2.01E+08	18	6.15736	3.29676
Nc-1-IP_pe	chr1	2.01E+08	2.01E+08	907	2.01E+08	22	4.80668	2.5094
Nc-1-IP_pe	chr1	2.01E+08	2.01E+08	565	2.01E+08	16	5.82808	3.34463
Nc-1-IP_pe	chr1	2.01E+08	2.01E+08	559	2.01E+08	14	5.03085	3.16286
Nc-1-IP_pe	chr1	2.01E+08	2.01E+08	302	2.01E+08	11	3.8706	2.84726
Nc-1-IP_pe	chr1	2.01E+08	2.01E+08	339	2.01E+08	14	5.88063	3.59594
Nc-1-IP_pe	chr1	2.01E+08	2.01E+08	791	2.01E+08	15	6.14813	3.61124
Nc-1-IP_pe	chr1	2.02E+08	2.02E+08	629	2.02E+08	26	12.2933	4.9149
Nc-1-IP_pe	chr1	2.02E+08	2.02E+08	543	2.02E+08	12	4.70082	3.20033
Nc-1-IP_pe	chr1	2.02E+08	2.02E+08	463	2.02E+08	16	6.33285	3.58457
Nc-1-IP_pe	chr1	2.02E+08	2.02E+08	299	2.02E+08	16	4.25252	2.63824
Nc-1-IP_pe	chr1	2.02E+08	2.02E+08	408	2.02E+08	16	4.96462	2.94973
Nc-1-IP_pe	chr1	2.02E+08	2.02E+08	549	2.02E+08	19	8.4638	4.21715
Nc-1-IP_pe	chr1	2.02E+08	2.02E+08	602	2.02E+08	14	4.60011	2.95114
Nc-1-IP_pe	chr1	2.02E+08	2.02E+08	550	2.02E+08	15	4.06016	2.62146
Nc-1-IP_pe	chr1	2.02E+08	2.02E+08	412	2.02E+08	14	5.74019	3.52296
Nc-1-IP_pe	chr1	2.02E+08	2.02E+08	811	2.02E+08	16	7.56205	4.19712
Nc-1-IP_pe	chr1	2.02E+08	2.02E+08	292	2.02E+08	17	6.64406	3.61808
Nc-1-IP_pe	chr1	2.02E+08	2.02E+08	255	2.02E+08	14	6.28469	3.80898
Nc-1-IP_pe	chr1	2.02E+08	2.02E+08	376	2.02E+08	13	4.69621	3.09346

Nc-1-IP_pe	chr1	2.02E+08	2.02E+08	345	2.02E+08	21	7.22656	3.4604
Nc-1-IP_pe	chr1	2.02E+08	2.02E+08	436	2.02E+08	12	4.66786	3.1824
Nc-1-IP_pe	chr1	2.02E+08	2.02E+08	363	2.02E+08	12	3.16137	2.39719
Nc-1-IP_pe	chr1	2.02E+08	2.02E+08	370	2.02E+08	13	5.17277	3.34236
Nc-1-IP_pe	chr1	2.02E+08	2.02E+08	656	2.02E+08	15	6.57408	3.82774
Nc-1-IP_pe	chr1	2.02E+08	2.02E+08	428	2.02E+08	14	5.82389	3.56638
Nc-1-IP_pe	chr1	2.02E+08	2.02E+08	742	2.02E+08	18	4.62526	2.6628
Nc-1-IP_pe	chr1	2.02E+08	2.02E+08	738	2.02E+08	22	5.72303	2.82387
Nc-1-IP_pe	chr1	2.02E+08	2.02E+08	541	2.02E+08	15	5.66913	3.37372
Nc-1-IP_pe	chr1	2.02E+08	2.02E+08	618	2.02E+08	11	4.87504	3.4143
Nc-1-IP_pe	chr1	2.02E+08	2.02E+08	410	2.02E+08	14	5.99644	3.65653
Nc-1-IP_pe	chr1	2.02E+08	2.02E+08	261	2.02E+08	12	5.15877	3.45264
Nc-1-IP_pe	chr1	2.02E+08	2.02E+08	866	2.02E+08	15	4.40338	2.77622
Nc-1-IP_pe	chr1	2.02E+08	2.02E+08	732	2.02E+08	18	7.62523	3.95652
Nc-1-IP_pe	chr1	2.02E+08	2.02E+08	358	2.02E+08	22	10.8623	4.88619
Nc-1-IP_pe	chr1	2.02E+08	2.02E+08	428	2.02E+08	15	5.66913	3.37372
Nc-1-IP_pe	chr1	2.02E+08	2.02E+08	670	2.02E+08	18	7.47506	3.88659
Nc-1-IP_pe	chr1	2.02E+08	2.02E+08	517	2.02E+08	19	7.29128	3.68799
Nc-1-IP_pe	chr1	2.02E+08	2.02E+08	255	2.02E+08	14	5.51181	3.40546
Nc-1-IP_pe	chr1	2.02E+08	2.02E+08	675	2.02E+08	15	6.73062	3.90854
Nc-1-IP_pe	chr1	2.02E+08	2.02E+08	474	2.02E+08	12	4.69273	3.19593
Nc-1-IP_pe	chr1	2.02E+08	2.02E+08	570	2.02E+08	13	4.02562	2.7544
Nc-1-IP_pe	chr1	2.02E+08	2.02E+08	429	2.02E+08	13	4.53922	3.01292
Nc-1-IP_pe	chr1	2.02E+08	2.02E+08	569	2.02E+08	15	6.06481	3.56947
Nc-1-IP_pe	chr1	2.02E+08	2.02E+08	565	2.02E+08	17	5.7822	3.22586
Nc-1-IP_pe	chr1	2.02E+08	2.02E+08	537	2.02E+08	13	5.73236	3.64298
Nc-1-IP_pe	chr1	2.02E+08	2.02E+08	340	2.02E+08	13	5.57038	3.55505
Nc-1-IP_pe	chr1	2.02E+08	2.02E+08	662	2.02E+08	13	6.36468	3.99316
Nc-1-IP_pe	chr1	2.02E+08	2.02E+08	516	2.02E+08	13	4.02562	2.7544
Nc-1-IP_pe	chr1	2.02E+08	2.02E+08	470	2.02E+08	14	5.18557	3.24018
Nc-1-IP_pe	chr1	2.02E+08	2.02E+08	258	2.02E+08	14	5.51541	3.4073
Nc-1-IP_pe	chr1	2.02E+08	2.02E+08	256	2.02E+08	16	6.51374	3.67221
Nc-1-IP_pe	chr1	2.02E+08	2.02E+08	255	2.02E+08	13	5.36853	3.44651
Nc-1-IP_pe	chr1	2.02E+08	2.02E+08	264	2.02E+08	16	7.53857	4.18505
Nc-1-IP_pe	chr1	2.02E+08	2.02E+08	287	2.02E+08	14	6.69566	4.03025
Nc-1-IP_pe	chr1	2.02E+08	2.02E+08	601	2.02E+08	13	4.86362	3.18015
Nc-1-IP_pe	chr1	2.02E+08	2.02E+08	318	2.02E+08	14	6.06519	3.69269
Nc-1-IP_pe	chr1	2.02E+08	2.02E+08	278	2.02E+08	14	6.69566	4.03025
Nc-1-IP_pe	chr1	2.02E+08	2.02E+08	351	2.02E+08	21	7.58657	3.60451
Nc-1-IP_pe	chr1	2.02E+08	2.02E+08	266	2.02E+08	19	5.51632	2.94814
Nc-1-IP_pe	chr1	2.02E+08	2.02E+08	472	2.02E+08	14	3.87746	2.60745
Nc-1-IP_pe	chr1	2.02E+08	2.02E+08	303	2.02E+08	14	5.19764	3.24624
Nc-1-IP_pe	chr1	2.02E+08	2.02E+08	690	2.02E+08	14	4.8399	3.06836
Nc-1-IP_pe	chr1	2.02E+08	2.02E+08	618	2.02E+08	16	5.28625	3.09457
Nc-1-IP_pe	chr1	2.02E+08	2.02E+08	270	2.02E+08	11	4.15998	3.00759

Nc-1-IP_pe	chr1	2.02E+08	2.02E+08	264	2.02E+08	13	4.75398	3.12328
Nc-1-IP_pe	chr1	2.02E+08	2.02E+08	261	2.02E+08	14	3.55308	2.45762
Nc-1-IP_pe	chr1	2.02E+08	2.02E+08	495	2.02E+08	25	9.19615	3.83259
Nc-1-IP_pe	chr1	2.02E+08	2.02E+08	289	2.02E+08	14	6.10041	3.71126
Nc-1-IP_pe	chr1	2.02E+08	2.02E+08	565	2.02E+08	15	7.67809	4.4114
Nc-1-IP_pe	chr1	2.02E+08	2.02E+08	548	2.02E+08	13	5.9487	3.76157
Nc-1-IP_pe	chr1	2.02E+08	2.02E+08	327	2.02E+08	9	4.29346	3.30898
Nc-1-IP_pe	chr1	2.02E+08	2.02E+08	613	2.02E+08	27	14.085	5.5088
Nc-1-IP_pe	chr1	2.02E+08	2.02E+08	298	2.02E+08	14	3.86727	2.6027
Nc-1-IP_pe	chr1	2.02E+08	2.02E+08	276	2.02E+08	13	4.02562	2.7544
Nc-1-IP_pe	chr1	2.02E+08	2.02E+08	329	2.02E+08	22	7.27519	3.39036
Nc-1-IP_pe	chr1	2.02E+08	2.02E+08	1152	2.02E+08	21	8.61158	4.03009
Nc-1-IP_pe	chr1	2.02E+08	2.02E+08	376	2.02E+08	17	5.90762	3.28176
Nc-1-IP_pe	chr1	2.02E+08	2.02E+08	782	2.02E+08	20	7.45226	3.65043
Nc-1-IP_pe	chr1	2.02E+08	2.02E+08	337	2.02E+08	13	4.42057	2.95252
Nc-1-IP_pe	chr1	2.02E+08	2.02E+08	518	2.02E+08	13	4.58338	3.0355
Nc-1-IP_pe	chr1	2.02E+08	2.02E+08	312	2.02E+08	13	4.67336	3.0817
Nc-1-IP_pe	chr1	2.02E+08	2.02E+08	267	2.02E+08	13	4.98219	3.24203
Nc-1-IP_pe	chr1	2.02E+08	2.02E+08	412	2.02E+08	12	3.83688	2.74114
Nc-1-IP_pe	chr1	2.02E+08	2.02E+08	629	2.02E+08	13	3.07238	2.29378
Nc-1-IP_pe	chr1	2.02E+08	2.02E+08	306	2.02E+08	13	3.67377	2.58159
Nc-1-IP_pe	chr1	2.02E+08	2.02E+08	296	2.02E+08	13	5.29187	3.40559
Nc-1-IP_pe	chr1	2.02E+08	2.02E+08	259	2.02E+08	11	3.64839	2.72584
Nc-1-IP_pe	chr1	2.02E+08	2.02E+08	262	2.02E+08	16	4.25252	2.63824
Nc-1-IP_pe	chr1	2.02E+08	2.02E+08	442	2.02E+08	17	5.54967	3.12324
Nc-1-IP_pe	chr1	2.02E+08	2.02E+08	686	2.02E+08	16	5.73763	3.30235
Nc-1-IP_pe	chr1	2.02E+08	2.02E+08	366	2.02E+08	19	7.18275	3.64066
Nc-1-IP_pe	chr1	2.02E+08	2.02E+08	625	2.02E+08	15	4.40338	2.77622
Nc-1-IP_pe	chr1	2.02E+08	2.02E+08	450	2.02E+08	17	6.25544	3.43888
Nc-1-IP_pe	chr1	2.02E+08	2.02E+08	431	2.02E+08	13	3.60553	2.54847
Nc-1-IP_pe	chr1	2.02E+08	2.02E+08	497	2.02E+08	22	8.79433	3.99081
Nc-1-IP_pe	chr1	2.02E+08	2.02E+08	303	2.02E+08	17	6.97865	3.77546
Nc-1-IP_pe	chr1	2.02E+08	2.02E+08	494	2.02E+08	16	5.39474	3.14402
Nc-1-IP_pe	chr1	2.02E+08	2.02E+08	312	2.02E+08	19	6.78661	3.47027
Nc-1-IP_pe	chr1	2.02E+08	2.02E+08	1589	2.02E+08	19	7.84735	3.93486
Nc-1-IP_pe	chr1	2.02E+08	2.02E+08	594	2.02E+08	13	4.34711	2.91533
Nc-1-IP_pe	chr1	2.02E+08	2.02E+08	992	2.02E+08	15	4.7819	2.95039
Nc-1-IP_pe	chr1	2.02E+08	2.02E+08	962	2.02E+08	13	3.67377	2.58159
Nc-1-IP_pe	chr1	2.02E+08	2.02E+08	417	2.02E+08	15	4.80057	2.95908
Nc-1-IP_pe	chr1	2.02E+08	2.02E+08	627	2.02E+08	17	4.78101	2.79343
Nc-1-IP_pe	chr1	2.02E+08	2.02E+08	524	2.02E+08	14	5.18557	3.24018
Nc-1-IP_pe	chr1	2.02E+08	2.02E+08	316	2.02E+08	20	6.08336	3.09555
Nc-1-IP_pe	chr1	2.02E+08	2.02E+08	573	2.02E+08	15	5.35559	3.22166
Nc-1-IP_pe	chr1	2.02E+08	2.02E+08	273	2.02E+08	15	5.20142	3.14789
Nc-1-IP_pe	chr1	2.02E+08	2.02E+08	261	2.02E+08	15	5.58327	3.33181

Nc-1-IP_pe	chr1	2.02E+08	2.02E+08	1068	2.02E+08		18	4.96968	2.80074
Nc-1-IP_pe	chr1	2.02E+08	2.02E+08	1626	2.02E+08		19	8.80534	4.37744
Nc-1-IP_pe	chr1	2.02E+08	2.02E+08	565	2.02E+08		15	5.52215	3.3021
Nc-1-IP_pe	chr1	2.02E+08	2.02E+08	472	2.02E+08		20	9.06303	4.35732
Nc-1-IP_pe	chr1	2.02E+08	2.02E+08	384	2.02E+08		16	5.73763	3.30235
Nc-1-IP_pe	chr1	2.02E+08	2.02E+08	496	2.02E+08		15	4.40338	2.77622
Nc-1-IP_pe	chr1	2.02E+08	2.02E+08	524	2.02E+08		12	4.24064	2.95299
Nc-1-IP_pe	chr1	2.02E+08	2.02E+08	417	2.02E+08		11	3.69531	2.75136
Nc-1-IP_pe	chr1	2.02E+08	2.02E+08	302	2.02E+08		14	5.60432	3.45288
Nc-1-IP_pe	chr1	2.02E+08	2.02E+08	940	2.02E+08		17	7.6273	4.08876
Nc-1-IP_pe	chr1	2.02E+08	2.02E+08	500	2.02E+08		15	5.83691	3.4562
Nc-1-IP_pe	chr1	2.02E+08	2.02E+08	288	2.02E+08		18	7.32895	3.81908
Nc-1-IP_pe	chr1	2.02E+08	2.02E+08	690	2.02E+08		17	8.7831	4.67333
Nc-1-IP_pe	chr1	2.02E+08	2.02E+08	292	2.02E+08		9	3.84391	3.03159
Nc-1-IP_pe	chr1	2.02E+08	2.02E+08	528	2.02E+08		15	6.95893	4.02756
Nc-1-IP_pe	chr1	2.02E+08	2.02E+08	343	2.02E+08		12	3.83688	2.74114
Nc-1-IP_pe	chr1	2.02E+08	2.02E+08	303	2.02E+08		11	3.14224	2.45469
Nc-1-IP_pe	chr1	2.02E+08	2.02E+08	412	2.02E+08		14	5.5249	3.41215
Nc-1-IP_pe	chr1	2.02E+08	2.02E+08	271	2.02E+08		15	5.88948	3.4822
Nc-1-IP_pe	chr1	2.02E+08	2.02E+08	387	2.02E+08		15	5.4025	3.24424
Nc-1-IP_pe	chr1	2.02E+08	2.02E+08	1126	2.02E+08		17	6.72563	3.65618
Nc-1-IP_pe	chr1	2.02E+08	2.02E+08	552	2.02E+08		13	5.13388	3.3218
Nc-1-IP_pe	chr1	2.02E+08	2.02E+08	418	2.02E+08		12	4.70082	3.20033
Nc-1-IP_pe	chr1	2.02E+08	2.02E+08	599	2.02E+08		12	5.3483	3.55882
Nc-1-IP_pe	chr1	2.02E+08	2.02E+08	360	2.02E+08		22	5.09279	2.60614
Nc-1-IP_pe	chr1	2.02E+08	2.02E+08	264	2.02E+08		15	9.17347	5.24934
Nc-1-IP_pe	chr1	2.02E+08	2.02E+08	301	2.02E+08		21	8.10596	3.8173
Nc-1-IP_pe	chr1	2.02E+08	2.02E+08	479	2.02E+08		11	3.64839	2.72584
Nc-1-IP_pe	chr1	2.02E+08	2.02E+08	832	2.02E+08		11	4.54404	3.2242
Nc-1-IP_pe	chr1	2.02E+08	2.02E+08	564	2.02E+08		13	7.42513	4.60316
Nc-1-IP_pe	chr1	2.02E+08	2.02E+08	341	2.02E+08		11	5.03835	3.5092
Nc-1-IP_pe	chr1	2.02E+08	2.02E+08	313	2.02E+08		14	6.96097	4.17548
Nc-1-IP_pe	chr1	2.02E+08	2.02E+08	335	2.02E+08		18	5.72638	3.11298
Nc-1-IP_pe	chr1	2.02E+08	2.02E+08	263	2.02E+08		13	3.67377	2.58159
Nc-1-IP_pe	chr1	2.02E+08	2.02E+08	373	2.02E+08		11	3.64839	2.72584
Nc-1-IP_pe	chr1	2.02E+08	2.02E+08	758	2.02E+08		19	4.23334	2.45554
Nc-1-IP_pe	chr1	2.02E+08	2.02E+08	293	2.02E+08		17	5.9911	3.31919
Nc-1-IP_pe	chr1	2.03E+08	2.03E+08	335	2.03E+08		15	5.83691	3.4562
Nc-1-IP_pe	chr1	2.03E+08	2.03E+08	464	2.03E+08		14	5.63114	3.46667
Nc-1-IP_pe	chr1	2.03E+08	2.03E+08	798	2.03E+08		16	7.11257	3.96852
Nc-1-IP_pe	chr1	2.03E+08	2.03E+08	309	2.03E+08		15	5.20142	3.14789
Nc-1-IP_pe	chr1	2.03E+08	2.03E+08	280	2.03E+08		10	4.04792	3.04735
Nc-1-IP_pe	chr1	2.03E+08	2.03E+08	552	2.03E+08		16	3.1497	2.1782
Nc-1-IP_pe	chr1	2.03E+08	2.03E+08	302	2.03E+08		13	4.41954	2.952
Nc-1-IP_pe	chr1	2.03E+08	2.03E+08	791	2.03E+08		18	8.71476	4.4806

Nc-1-IP_pe	chr1	2.03E+08	2.03E+08	360	2.03E+08	19	5.15822	2.80735
Nc-1-IP_pe	chr1	2.03E+08	2.03E+08	544	2.03E+08	14	5.03085	3.16286
Nc-1-IP_pe	chr1	2.03E+08	2.03E+08	369	2.03E+08	15	6.33416	3.70518
Nc-1-IP_pe	chr1	2.03E+08	2.03E+08	311	2.03E+08	12	4.54611	3.11648
Nc-1-IP_pe	chr1	2.03E+08	2.03E+08	288	2.03E+08	12	3.83688	2.74114
Nc-1-IP_pe	chr1	2.03E+08	2.03E+08	256	2.03E+08	14	4.32616	2.81919
Nc-1-IP_pe	chr1	2.03E+08	2.03E+08	622	2.03E+08	15	6.33416	3.70518
Nc-1-IP_pe	chr1	2.03E+08	2.03E+08	272	2.03E+08	17	6.47874	3.54137
Nc-1-IP_pe	chr1	2.03E+08	2.03E+08	409	2.03E+08	14	6.25343	3.79234
Nc-1-IP_pe	chr1	2.03E+08	2.03E+08	298	2.03E+08	9	3.21106	2.6498
Nc-1-IP_pe	chr1	2.03E+08	2.03E+08	1369	2.03E+08	12	5.23082	3.49289
Nc-1-IP_pe	chr1	2.03E+08	2.03E+08	477	2.03E+08	14	5.56442	3.43239
Nc-1-IP_pe	chr1	2.03E+08	2.03E+08	750	2.03E+08	16	6.33285	3.58457
Nc-1-IP_pe	chr1	2.03E+08	2.03E+08	269	2.03E+08	14	6.44441	3.89444
Nc-1-IP_pe	chr1	2.03E+08	2.03E+08	392	2.03E+08	18	4.32723	2.54542
Nc-1-IP_pe	chr1	2.03E+08	2.03E+08	482	2.03E+08	14	7.14701	4.27839
Nc-1-IP_pe	chr1	2.03E+08	2.03E+08	306	2.03E+08	12	3.47963	2.55766
Nc-1-IP_pe	chr1	2.03E+08	2.03E+08	356	2.03E+08	20	8.60223	4.14902
Nc-1-IP_pe	chr1	2.03E+08	2.03E+08	835	2.03E+08	14	5.43414	3.36584
Nc-1-IP_pe	chr1	2.03E+08	2.03E+08	637	2.03E+08	12	4.69273	3.19593
Nc-1-IP_pe	chr1	2.03E+08	2.03E+08	424	2.03E+08	13	5.57038	3.55505
Nc-1-IP_pe	chr1	2.03E+08	2.03E+08	429	2.03E+08	17	8.7831	4.67333
Nc-1-IP_pe	chr1	2.03E+08	2.03E+08	352	2.03E+08	11	3.64839	2.72584
Nc-1-IP_pe	chr1	2.03E+08	2.03E+08	306	2.03E+08	13	6.62436	4.14003
Nc-1-IP_pe	chr1	2.03E+08	2.03E+08	264	2.03E+08	13	4.02562	2.7544
Nc-1-IP_pe	chr1	2.03E+08	2.03E+08	392	2.03E+08	17	4.13464	2.52661
Nc-1-IP_pe	chr1	2.03E+08	2.03E+08	359	2.03E+08	27	10.1235	3.97223
Nc-1-IP_pe	chr1	2.03E+08	2.03E+08	376	2.03E+08	19	5.51632	2.94814
Nc-1-IP_pe	chr1	2.03E+08	2.03E+08	291	2.03E+08	15	4.40338	2.77622
Nc-1-IP_pe	chr1	2.03E+08	2.03E+08	467	2.03E+08	13	5.13388	3.3218
Nc-1-IP_pe	chr1	2.03E+08	2.03E+08	684	2.03E+08	18	7.1522	3.73811
Nc-1-IP_pe	chr1	2.03E+08	2.03E+08	274	2.03E+08	14	3.79446	2.56886
Nc-1-IP_pe	chr1	2.03E+08	2.03E+08	374	2.03E+08	14	5.88063	3.59594
Nc-1-IP_pe	chr1	2.03E+08	2.03E+08	285	2.03E+08	13	3.21799	2.36264
Nc-1-IP_pe	chr1	2.03E+08	2.03E+08	253	2.03E+08	17	6.38155	3.49661
Nc-1-IP_pe	chr1	2.03E+08	2.03E+08	422	2.03E+08	13	3.29152	2.39761
Nc-1-IP_pe	chr1	2.03E+08	2.03E+08	408	2.03E+08	13	3.86418	2.67468
Nc-1-IP_pe	chr1	2.03E+08	2.03E+08	273	2.03E+08	14	3.79446	2.56886
Nc-1-IP_pe	chr1	2.03E+08	2.03E+08	492	2.03E+08	13	5.21206	3.36317
Nc-1-IP_pe	chr1	2.03E+08	2.03E+08	558	2.03E+08	10	5.08818	3.67138
Nc-1-IP_pe	chr1	2.03E+08	2.03E+08	280	2.03E+08	12	5.72541	3.77304
Nc-1-IP_pe	chr1	2.03E+08	2.03E+08	298	2.03E+08	13	6.09351	3.84167
Nc-1-IP_pe	chr1	2.03E+08	2.03E+08	680	2.03E+08	13	4.41954	2.952
Nc-1-IP_pe	chr1	2.03E+08	2.03E+08	742	2.03E+08	19	9.15219	4.54307
Nc-1-IP_pe	chr1	2.03E+08	2.03E+08	537	2.03E+08	9	3.70468	2.94668

Nc-1-IP_pe	chr1	2.03E+08	2.03E+08	670	2.03E+08	14	4.60011	2.95114
Nc-1-IP_pe	chr1	2.03E+08	2.03E+08	378	2.03E+08	14	3.26761	2.32785
Nc-1-IP_pe	chr1	2.03E+08	2.03E+08	554	2.03E+08	20	6.07662	3.09292
Nc-1-IP_pe	chr1	2.03E+08	2.03E+08	309	2.03E+08	16	6.33285	3.58457
Nc-1-IP_pe	chr1	2.03E+08	2.03E+08	419	2.03E+08	13	5.36853	3.44651
Nc-1-IP_pe	chr1	2.03E+08	2.03E+08	360	2.03E+08	19	5.90435	3.10381
Nc-1-IP_pe	chr1	2.03E+08	2.03E+08	610	2.03E+08	14	4.60011	2.95114
Nc-1-IP_pe	chr1	2.03E+08	2.03E+08	320	2.03E+08	14	5.7679	3.53731
Nc-1-IP_pe	chr1	2.03E+08	2.03E+08	474	2.03E+08	15	6.19428	3.63445
Nc-1-IP_pe	chr1	2.03E+08	2.03E+08	326	2.03E+08	12	4.58219	3.13597
Nc-1-IP_pe	chr1	2.03E+08	2.03E+08	306	2.03E+08	13	4.67336	3.0817
Nc-1-IP_pe	chr1	2.03E+08	2.03E+08	1130	2.03E+08	14	5.95264	3.63357
Nc-1-IP_pe	chr1	2.03E+08	2.03E+08	354	2.03E+08	19	7.29128	3.68799
Nc-1-IP_pe	chr1	2.03E+08	2.03E+08	388	2.03E+08	13	4.56123	3.02417
Nc-1-IP_pe	chr1	2.03E+08	2.03E+08	650	2.03E+08	17	8.06497	4.3062
Nc-1-IP_pe	chr1	2.03E+08	2.03E+08	320	2.03E+08	16	4.68897	2.82769
Nc-1-IP_pe	chr1	2.03E+08	2.03E+08	480	2.03E+08	13	4.56123	3.02417
Nc-1-IP_pe	chr1	2.03E+08	2.03E+08	371	2.03E+08	14	6.66104	4.01144
Nc-1-IP_pe	chr1	2.03E+08	2.03E+08	321	2.03E+08	13	5.9487	3.76157
Nc-1-IP_pe	chr1	2.03E+08	2.03E+08	577	2.03E+08	15	5.20142	3.14789
Nc-1-IP_pe	chr1	2.03E+08	2.03E+08	354	2.03E+08	14	3.86727	2.6027
Nc-1-IP_pe	chr1	2.03E+08	2.03E+08	335	2.03E+08	13	5.42865	3.47872
Nc-1-IP_pe	chr1	2.03E+08	2.03E+08	622	2.03E+08	17	7.02067	3.79543
Nc-1-IP_pe	chr1	2.03E+08	2.03E+08	374	2.03E+08	12	4.78119	3.24417
Nc-1-IP_pe	chr1	2.03E+08	2.03E+08	279	2.03E+08	17	4.13464	2.52661
Nc-1-IP_pe	chr1	2.03E+08	2.03E+08	1872	2.03E+08	15	5.83691	3.4562
Nc-1-IP_pe	chr1	2.03E+08	2.03E+08	308	2.03E+08	19	9.91418	4.91681
Nc-1-IP_pe	chr1	2.03E+08	2.03E+08	430	2.03E+08	11	3.64839	2.72584
Nc-1-IP_pe	chr1	2.03E+08	2.03E+08	572	2.03E+08	13	4.02562	2.7544
Nc-1-IP_pe	chr1	2.03E+08	2.03E+08	500	2.03E+08	18	7.1522	3.73811
Nc-1-IP_pe	chr1	2.03E+08	2.03E+08	410	2.03E+08	15	5.72092	3.39909
Nc-1-IP_pe	chr1	2.03E+08	2.03E+08	584	2.03E+08	12	3.46657	2.55102
Nc-1-IP_pe	chr1	2.03E+08	2.03E+08	307	2.03E+08	23	7.01947	3.21526
Nc-1-IP_pe	chr1	2.03E+08	2.03E+08	629	2.03E+08	15	3.46204	2.35851
Nc-1-IP_pe	chr1	2.03E+08	2.03E+08	254	2.03E+08	14	4.67812	2.9891
Nc-1-IP_pe	chr1	2.03E+08	2.03E+08	288	2.03E+08	14	4.36469	2.83763
Nc-1-IP_pe	chr1	2.03E+08	2.03E+08	462	2.03E+08	13	5.08264	3.29478
Nc-1-IP_pe	chr1	2.03E+08	2.03E+08	499	2.03E+08	11	3.7458	2.77889
Nc-1-IP_pe	chr1	2.03E+08	2.03E+08	272	2.03E+08	12	3.96559	2.80816
Nc-1-IP_pe	chr1	2.03E+08	2.03E+08	874	2.03E+08	18	7.82684	4.0513
Nc-1-IP_pe	chr1	2.03E+08	2.03E+08	1400	2.03E+08	16	5.93204	3.39349
Nc-1-IP_pe	chr1	2.03E+08	2.03E+08	434	2.03E+08	14	5.09034	3.19251
Nc-1-IP_pe	chr1	2.03E+08	2.03E+08	270	2.03E+08	10	2.96668	2.43058
Nc-1-IP_pe	chr1	2.03E+08	2.03E+08	692	2.03E+08	15	5.20142	3.14789
Nc-1-IP_pe	chr1	2.03E+08	2.03E+08	632	2.03E+08	14	5.98179	3.64885

Nc-1-IP_pe	chr1	2.03E+08	2.03E+08	332	2.03E+08	14	7.54286	4.50023
Nc-1-IP_pe	chr1	2.03E+08	2.03E+08	317	2.03E+08	15	6.19428	3.63445
Nc-1-IP_pe	chr1	2.03E+08	2.03E+08	498	2.03E+08	12	4.1728	2.91706
Nc-1-IP_pe	chr1	2.03E+08	2.03E+08	347	2.03E+08	15	5.20142	3.14789
Nc-1-IP_pe	chr1	2.03E+08	2.03E+08	275	2.03E+08	11	4.19517	3.02726
Nc-1-IP_pe	chr1	2.03E+08	2.03E+08	262	2.03E+08	12	4.70082	3.20033
Nc-1-IP_pe	chr1	2.03E+08	2.03E+08	823	2.03E+08	14	4.60011	2.95114
Nc-1-IP_pe	chr1	2.03E+08	2.03E+08	259	2.03E+08	13	3.67377	2.58159
Nc-1-IP_pe	chr1	2.03E+08	2.03E+08	412	2.03E+08	11	4.62357	3.2696
Nc-1-IP_pe	chr1	2.03E+08	2.03E+08	355	2.03E+08	13	6.24389	3.92544
Nc-1-IP_pe	chr1	2.03E+08	2.03E+08	388	2.03E+08	9	3.66792	2.92434
Nc-1-IP_pe	chr1	2.03E+08	2.03E+08	372	2.03E+08	13	5.9487	3.76157
Nc-1-IP_pe	chr1	2.03E+08	2.03E+08	368	2.03E+08	16	7.27441	4.05022
Nc-1-IP_pe	chr1	2.03E+08	2.03E+08	295	2.03E+08	23	7.3183	3.32328
Nc-1-IP_pe	chr1	2.03E+08	2.03E+08	741	2.03E+08	17	3.83544	2.40609
Nc-1-IP_pe	chr1	2.03E+08	2.03E+08	271	2.03E+08	14	5.56442	3.43239
Nc-1-IP_pe	chr1	2.03E+08	2.03E+08	693	2.03E+08	16	7.97877	4.4137
Nc-1-IP_pe	chr1	2.03E+08	2.03E+08	528	2.03E+08	15	7.43258	4.27887
Nc-1-IP_pe	chr1	2.03E+08	2.03E+08	273	2.03E+08	13	5.47064	3.50127
Nc-1-IP_pe	chr1	2.03E+08	2.03E+08	843	2.03E+08	21	7.22656	3.4604
Nc-1-IP_pe	chr1	2.03E+08	2.03E+08	675	2.03E+08	20	7.97282	3.87239
Nc-1-IP_pe	chr1	2.03E+08	2.03E+08	337	2.03E+08	14	3.86727	2.6027
Nc-1-IP_pe	chr1	2.03E+08	2.03E+08	274	2.03E+08	16	4.3356	2.67395
Nc-1-IP_pe	chr1	2.03E+08	2.03E+08	265	2.03E+08	9	3.25221	2.67429
Nc-1-IP_pe	chr1	2.03E+08	2.03E+08	289	2.03E+08	17	8.01206	4.27966
Nc-1-IP_pe	chr1	2.03E+08	2.03E+08	713	2.03E+08	15	7.69749	4.42193
Nc-1-IP_pe	chr1	2.03E+08	2.03E+08	568	2.03E+08	13	5.03208	3.26819
Nc-1-IP_pe	chr1	2.03E+08	2.03E+08	319	2.03E+08	9	2.89249	2.46179
Nc-1-IP_pe	chr1	2.03E+08	2.03E+08	583	2.03E+08	13	3.07238	2.29378
Nc-1-IP_pe	chr1	2.03E+08	2.03E+08	751	2.03E+08	16	4.96462	2.94973
Nc-1-IP_pe	chr1	2.03E+08	2.03E+08	279	2.03E+08	10	3.08907	2.49869
Nc-1-IP_pe	chr1	2.03E+08	2.03E+08	404	2.03E+08	15	5.58327	3.33181
Nc-1-IP_pe	chr1	2.03E+08	2.03E+08	281	2.03E+08	11	4.27885	3.07417
Nc-1-IP_pe	chr1	2.03E+08	2.03E+08	699	2.03E+08	12	4.95159	3.33774
Nc-1-IP_pe	chr1	2.03E+08	2.03E+08	1302	2.03E+08	16	5.50606	3.19508
Nc-1-IP_pe	chr1	2.03E+08	2.03E+08	403	2.03E+08	10	3.47412	2.71578
Nc-1-IP_pe	chr1	2.03E+08	2.03E+08	546	2.03E+08	16	6.8987	3.86161
Nc-1-IP_pe	chr1	2.03E+08	2.03E+08	535	2.03E+08	11	4.54404	3.2242
Nc-1-IP_pe	chr1	2.03E+08	2.03E+08	256	2.03E+08	14	4.60011	2.95114
Nc-1-IP_pe	chr1	2.03E+08	2.03E+08	678	2.03E+08	17	5.9911	3.31919
Nc-1-IP_pe	chr1	2.03E+08	2.03E+08	353	2.03E+08	11	2.96397	2.36091
Nc-1-IP_pe	chr1	2.03E+08	2.03E+08	372	2.03E+08	11	3.58722	2.69267
Nc-1-IP_pe	chr1	2.04E+08	2.04E+08	381	2.04E+08	18	6.29932	3.35826
Nc-1-IP_pe	chr1	2.04E+08	2.04E+08	487	2.04E+08	16	4.59224	2.7853
Nc-1-IP_pe	chr1	2.04E+08	2.04E+08	656	2.04E+08	18	6.50397	3.44778

Nc-1-IP_pe	chr1	2.04E+08	2.04E+08	566	2.04E+08	16	4.25252	2.63824
Nc-1-IP_pe	chr1	2.04E+08	2.04E+08	371	2.04E+08	18	4.96968	2.80074
Nc-1-IP_pe	chr1	2.04E+08	2.04E+08	389	2.04E+08	14	4.21449	2.76599
Nc-1-IP_pe	chr1	2.04E+08	2.04E+08	1474	2.04E+08	17	7.35238	3.95466
Nc-1-IP_pe	chr1	2.04E+08	2.04E+08	602	2.04E+08	12	4.1728	2.91706
Nc-1-IP_pe	chr1	2.04E+08	2.04E+08	504	2.04E+08	17	5.9911	3.31919
Nc-1-IP_pe	chr1	2.04E+08	2.04E+08	354	2.04E+08	13	4.02562	2.7544
Nc-1-IP_pe	chr1	2.04E+08	2.04E+08	253	2.04E+08	11	3.79717	2.80697
Nc-1-IP_pe	chr1	2.04E+08	2.04E+08	273	2.04E+08	16	6.8987	3.86161
Nc-1-IP_pe	chr1	2.04E+08	2.04E+08	363	2.04E+08	12	4.66786	3.1824
Nc-1-IP_pe	chr1	2.04E+08	2.04E+08	458	2.04E+08	11	3.80755	2.81266
Nc-1-IP_pe	chr1	2.04E+08	2.04E+08	904	2.04E+08	15	4.40338	2.77622
Nc-1-IP_pe	chr1	2.04E+08	2.04E+08	358	2.04E+08	14	6.05554	3.68761
Nc-1-IP_pe	chr1	2.04E+08	2.04E+08	331	2.04E+08	16	3.78851	2.44164
Nc-1-IP_pe	chr1	2.04E+08	2.04E+08	285	2.04E+08	12	4.15147	2.90579
Nc-1-IP_pe	chr1	2.04E+08	2.04E+08	320	2.04E+08	13	4.14549	2.81406
Nc-1-IP_pe	chr1	2.04E+08	2.04E+08	328	2.04E+08	18	8.37884	4.31591
Nc-1-IP_pe	chr1	2.04E+08	2.04E+08	385	2.04E+08	11	4.71915	3.32439
Nc-1-IP_pe	chr1	2.04E+08	2.04E+08	504	2.04E+08	13	5.85439	3.70972
Nc-1-IP_pe	chr1	2.04E+08	2.04E+08	621	2.04E+08	14	5.51541	3.4073
Nc-1-IP_pe	chr1	2.04E+08	2.04E+08	721	2.04E+08	12	4.84576	3.27953
Nc-1-IP_pe	chr1	2.04E+08	2.04E+08	349	2.04E+08	18	10.6609	5.48691
Nc-1-IP_pe	chr1	2.04E+08	2.04E+08	287	2.04E+08	13	4.86362	3.18015
Nc-1-IP_pe	chr1	2.04E+08	2.04E+08	337	2.04E+08	17	8.72293	4.64208
Nc-1-IP_pe	chr1	2.04E+08	2.04E+08	611	2.04E+08	13	5.15976	3.33548
Nc-1-IP_pe	chr1	2.04E+08	2.04E+08	553	2.04E+08	14	6.06519	3.69269
Nc-1-IP_pe	chr1	2.04E+08	2.04E+08	256	2.04E+08	13	5.9487	3.76157
Nc-1-IP_pe	chr1	2.04E+08	2.04E+08	253	2.04E+08	17	6.47874	3.54137
Nc-1-IP_pe	chr1	2.04E+08	2.04E+08	632	2.04E+08	15	5.70822	3.39286
Nc-1-IP_pe	chr1	2.04E+08	2.04E+08	512	2.04E+08	13	6.72991	4.20021
Nc-1-IP_pe	chr1	2.04E+08	2.04E+08	1191	2.04E+08	68	24.6439	4.51284
Nc-1-IP_pe	chr1	2.04E+08	2.04E+08	359	2.04E+08	12	6.6095	4.28941
Nc-1-IP_pe	chr1	2.04E+08	2.04E+08	264	2.04E+08	10	3.89064	2.95552
Nc-1-IP_pe	chr1	2.04E+08	2.04E+08	284	2.04E+08	13	5.9487	3.76157
Nc-1-IP_pe	chr1	2.04E+08	2.04E+08	403	2.04E+08	15	6.82712	3.95868
Nc-1-IP_pe	chr1	2.04E+08	2.04E+08	401	2.04E+08	15	6.19428	3.63445
Nc-1-IP_pe	chr1	2.04E+08	2.04E+08	344	2.04E+08	17	9.09431	4.8363
Nc-1-IP_pe	chr1	2.04E+08	2.04E+08	342	2.04E+08	13	5.36853	3.44651
Nc-1-IP_pe	chr1	2.04E+08	2.04E+08	424	2.04E+08	11	4.54404	3.2242
Nc-1-IP_pe	chr1	2.04E+08	2.04E+08	450	2.04E+08	11	4.86057	3.40592
Nc-1-IP_pe	chr1	2.04E+08	2.04E+08	503	2.04E+08	13	5.29187	3.40559
Nc-1-IP_pe	chr1	2.04E+08	2.04E+08	322	2.04E+08	19	6.32622	3.27682
Nc-1-IP_pe	chr1	2.04E+08	2.04E+08	253	2.04E+08	18	6.15736	3.29676
Nc-1-IP_pe	chr1	2.04E+08	2.04E+08	431	2.04E+08	19	9.60791	4.76497
Nc-1-IP_pe	chr1	2.04E+08	2.04E+08	2129	2.04E+08	19	4.8268	2.67939

Nc-1-IP_pe	chr1	2.04E+08	2.04E+08	1361	2.04E+08	17	4.96303	2.87025
Nc-1-IP_pe	chr1	2.04E+08	2.04E+08	287	2.04E+08	14	4.25033	2.78303
Nc-1-IP_pe	chr1	2.04E+08	2.04E+08	287	2.04E+08	17	6.26791	3.44457
Nc-1-IP_pe	chr1	2.04E+08	2.04E+08	274	2.04E+08	15	5.20142	3.14789
Nc-1-IP_pe	chr1	2.04E+08	2.04E+08	677	2.04E+08	15	4.7819	2.95039
Nc-1-IP_pe	chr1	2.04E+08	2.04E+08	881	2.04E+08	18	7.73136	4.00629
Nc-1-IP_pe	chr1	2.04E+08	2.04E+08	257	2.04E+08	14	5.90928	3.6109
Nc-1-IP_pe	chr1	2.04E+08	2.04E+08	730	2.04E+08	15	4.40338	2.77622
Nc-1-IP_pe	chr1	2.04E+08	2.04E+08	1170	2.04E+08	20	7.43645	3.64378
Nc-1-IP_pe	chr1	2.04E+08	2.04E+08	587	2.04E+08	16	5.82808	3.34463
Nc-1-IP_pe	chr1	2.04E+08	2.04E+08	691	2.04E+08	18	5.52764	3.02971
Nc-1-IP_pe	chr1	2.04E+08	2.04E+08	260	2.04E+08	17	4.13464	2.52661
Nc-1-IP_pe	chr1	2.04E+08	2.04E+08	265	2.04E+08	19	6.32622	3.27682
Nc-1-IP_pe	chr1	2.04E+08	2.04E+08	417	2.04E+08	16	5.37282	3.134
Nc-1-IP_pe	chr1	2.04E+08	2.04E+08	394	2.04E+08	16	2.92499	2.08721
Nc-1-IP_pe	chr1	2.04E+08	2.04E+08	349	2.04E+08	13	4.3784	2.93116
Nc-1-IP_pe	chr1	2.04E+08	2.04E+08	768	2.04E+08	15	3.20025	2.24588
Nc-1-IP_pe	chr1	2.04E+08	2.04E+08	286	2.04E+08	14	5.05522	3.17499
Nc-1-IP_pe	chr1	2.04E+08	2.04E+08	499	2.04E+08	13	3.67377	2.58159
Nc-1-IP_pe	chr1	2.04E+08	2.04E+08	481	2.04E+08	14	5.18557	3.24018
Nc-1-IP_pe	chr1	2.04E+08	2.04E+08	753	2.04E+08	16	6.40243	3.61818
Nc-1-IP_pe	chr1	2.04E+08	2.04E+08	1361	2.04E+08	23	9.50079	4.16433
Nc-1-IP_pe	chr1	2.04E+08	2.04E+08	317	2.04E+08	17	6.68468	3.63703
Nc-1-IP_pe	chr1	2.04E+08	2.04E+08	449	2.04E+08	14	4.60011	2.95114
Nc-1-IP_pe	chr1	2.04E+08	2.04E+08	379	2.04E+08	14	3.86727	2.6027
Nc-1-IP_pe	chr1	2.04E+08	2.04E+08	401	2.04E+08	15	4.60262	2.86743
Nc-1-IP_pe	chr1	2.04E+08	2.04E+08	1103	2.04E+08	15	5.54649	3.31392
Nc-1-IP_pe	chr1	2.04E+08	2.04E+08	489	2.04E+08	15	5.49794	3.29036
Nc-1-IP_pe	chr1	2.04E+08	2.04E+08	288	2.04E+08	11	3.28475	2.53029
Nc-1-IP_pe	chr1	2.04E+08	2.04E+08	1340	2.04E+08	15	5.83691	3.4562
Nc-1-IP_pe	chr1	2.04E+08	2.04E+08	697	2.04E+08	15	4.40338	2.77622
Nc-1-IP_pe	chr1	2.04E+08	2.04E+08	317	2.04E+08	10	3.15787	2.53716
Nc-1-IP_pe	chr1	2.04E+08	2.04E+08	856	2.04E+08	16	5.82808	3.34463
Nc-1-IP_pe	chr1	2.04E+08	2.04E+08	1179	2.04E+08	19	5.51632	2.94814
Nc-1-IP_pe	chr1	2.04E+08	2.04E+08	262	2.04E+08	11	4.09075	2.96901
Nc-1-IP_pe	chr1	2.04E+08	2.04E+08	293	2.04E+08	10	3.40382	2.67582
Nc-1-IP_pe	chr1	2.04E+08	2.04E+08	269	2.04E+08	16	4.59224	2.7853
Nc-1-IP_pe	chr1	2.04E+08	2.04E+08	619	2.04E+08	18	8.25345	4.25515
Nc-1-IP_pe	chr1	2.04E+08	2.04E+08	355	2.04E+08	17	8.20861	4.37862
Nc-1-IP_pe	chr1	2.04E+08	2.04E+08	299	2.04E+08	18	5.72638	3.11298
Nc-1-IP_pe	chr1	2.04E+08	2.04E+08	289	2.04E+08	14	5.56442	3.43239
Nc-1-IP_pe	chr1	2.04E+08	2.04E+08	2047	2.04E+08	16	6.33285	3.58457
Nc-1-IP_pe	chr1	2.04E+08	2.04E+08	272	2.04E+08	15	6.36347	3.72007
Nc-1-IP_pe	chr1	2.04E+08	2.04E+08	370	2.04E+08	12	4.35991	3.0165
Nc-1-IP_pe	chr1	2.04E+08	2.04E+08	820	2.04E+08	20	9.70997	4.65798

Nc-1-IP_pe	chr1	2.04E+08	2.04E+08	331	2.04E+08	14	4.60011	2.95114
Nc-1-IP_pe	chr1	2.04E+08	2.04E+08	490	2.04E+08	17	6.47874	3.54137
Nc-1-IP_pe	chr1	2.04E+08	2.04E+08	300	2.04E+08	16	5.62031	3.24783
Nc-1-IP_pe	chr1	2.04E+08	2.04E+08	304	2.04E+08	20	7.43645	3.64378
Nc-1-IP_pe	chr1	2.04E+08	2.04E+08	611	2.04E+08	17	6.47874	3.54137
Nc-1-IP_pe	chr1	2.04E+08	2.04E+08	382	2.04E+08	13	3.67377	2.58159
Nc-1-IP_pe	chr1	2.04E+08	2.04E+08	255	2.04E+08	12	3.4751	2.55535
Nc-1-IP_pe	chr1	2.04E+08	2.04E+08	303	2.04E+08	20	7.0741	3.49302
Nc-1-IP_pe	chr1	2.04E+08	2.04E+08	299	2.04E+08	14	4.60011	2.95114
Nc-1-IP_pe	chr1	2.04E+08	2.04E+08	342	2.04E+08	14	4.60011	2.95114
Nc-1-IP_pe	chr1	2.04E+08	2.04E+08	489	2.04E+08	15	4.14926	2.66135
Nc-1-IP_pe	chr1	2.04E+08	2.04E+08	260	2.04E+08	15	5.22925	3.16116
Nc-1-IP_pe	chr1	2.04E+08	2.04E+08	267	2.04E+08	13	4.93296	3.21629
Nc-1-IP_pe	chr1	2.04E+08	2.04E+08	462	2.04E+08	17	6.78076	3.68203
Nc-1-IP_pe	chr1	2.04E+08	2.04E+08	1097	2.04E+08	16	5.82808	3.34463
Nc-1-IP_pe	chr1	2.04E+08	2.04E+08	958	2.04E+08	18	8.21946	4.23874
Nc-1-IP_pe	chr1	2.04E+08	2.04E+08	854	2.04E+08	20	9.21699	4.428
Nc-1-IP_pe	chr1	2.04E+08	2.04E+08	355	2.04E+08	14	6.05554	3.68761
Nc-1-IP_pe	chr1	2.04E+08	2.04E+08	266	2.04E+08	16	9.24948	5.10026
Nc-1-IP_pe	chr1	2.04E+08	2.04E+08	271	2.04E+08	16	2.92499	2.08721
Nc-1-IP_pe	chr1	2.04E+08	2.04E+08	285	2.04E+08	15	5.73365	3.40534
Nc-1-IP_pe	chr1	2.04E+08	2.04E+08	314	2.04E+08	13	3.35769	2.42919
Nc-1-IP_pe	chr1	2.04E+08	2.04E+08	659	2.04E+08	17	5.14803	2.94914
Nc-1-IP_pe	chr1	2.04E+08	2.04E+08	372	2.04E+08	13	3.35769	2.42919
Nc-1-IP_pe	chr1	2.04E+08	2.04E+08	727	2.04E+08	18	8.42652	4.33912
Nc-1-IP_pe	chr1	2.04E+08	2.04E+08	310	2.04E+08	16	4.96462	2.94973
Nc-1-IP_pe	chr1	2.04E+08	2.04E+08	382	2.04E+08	14	5.7264	3.51582
Nc-1-IP_pe	chr1	2.04E+08	2.04E+08	276	2.04E+08	13	5.15976	3.33548
Nc-1-IP_pe	chr1	2.04E+08	2.04E+08	258	2.04E+08	15	4.06016	2.62146
Nc-1-IP_pe	chr1	2.04E+08	2.04E+08	286	2.04E+08	14	5.0786	3.18665
Nc-1-IP_pe	chr1	2.04E+08	2.04E+08	340	2.04E+08	16	6.26689	3.55283
Nc-1-IP_pe	chr1	2.04E+08	2.04E+08	478	2.04E+08	15	5.6578	3.36818
Nc-1-IP_pe	chr1	2.04E+08	2.04E+08	282	2.04E+08	20	6.08336	3.09555
Nc-1-IP_pe	chr1	2.05E+08	2.05E+08	429	2.05E+08	17	7.02067	3.79543
Nc-1-IP_pe	chr1	2.05E+08	2.05E+08	420	2.05E+08	15	7.37761	4.24941
Nc-1-IP_pe	chr1	2.05E+08	2.05E+08	310	2.05E+08	12	5.84943	3.84431
Nc-1-IP_pe	chr1	2.05E+08	2.05E+08	609	2.05E+08	14	6.69566	4.03025
Nc-1-IP_pe	chr1	2.05E+08	2.05E+08	295	2.05E+08	10	3.4604	2.70797
Nc-1-IP_pe	chr1	2.05E+08	2.05E+08	335	2.05E+08	17	8.39361	4.47264
Nc-1-IP_pe	chr1	2.05E+08	2.05E+08	276	2.05E+08	14	4.60011	2.95114
Nc-1-IP_pe	chr1	2.05E+08	2.05E+08	583	2.05E+08	13	5.26508	3.39133
Nc-1-IP_pe	chr1	2.05E+08	2.05E+08	352	2.05E+08	13	4.55021	3.01854
Nc-1-IP_pe	chr1	2.05E+08	2.05E+08	265	2.05E+08	17	8.10053	4.32408
Nc-1-IP_pe	chr1	2.05E+08	2.05E+08	1280	2.05E+08	15	6.89257	3.99282
Nc-1-IP_pe	chr1	2.05E+08	2.05E+08	439	2.05E+08	13	5.76252	3.65944

Nc-1-IP_pe	chr1	2.05E+08	2.05E+08	273	2.05E+08	13	4.90859	3.20357
Nc-1-IP_pe	chr1	2.05E+08	2.05E+08	317	2.05E+08	15	5.98302	3.52865
Nc-1-IP_pe	chr1	2.05E+08	2.05E+08	1153	2.05E+08	14	5.17354	3.23415
Nc-1-IP_pe	chr1	2.05E+08	2.05E+08	253	2.05E+08	16	5.50606	3.19508
Nc-1-IP_pe	chr1	2.05E+08	2.05E+08	385	2.05E+08	13	4.39942	2.9418
Nc-1-IP_pe	chr1	2.05E+08	2.05E+08	296	2.05E+08	12	3.83688	2.74114
Nc-1-IP_pe	chr1	2.05E+08	2.05E+08	416	2.05E+08	12	3.83688	2.74114
Nc-1-IP_pe	chr1	2.05E+08	2.05E+08	265	2.05E+08	12	4.06753	2.86157
Nc-1-IP_pe	chr1	2.05E+08	2.05E+08	423	2.05E+08	16	5.37454	3.13479
Nc-1-IP_pe	chr1	2.05E+08	2.05E+08	292	2.05E+08	14	6.06519	3.69269
Nc-1-IP_pe	chr1	2.05E+08	2.05E+08	641	2.05E+08	16	5.50606	3.19508
Nc-1-IP_pe	chr1	2.05E+08	2.05E+08	443	2.05E+08	12	4.1728	2.91706
Nc-1-IP_pe	chr1	2.05E+08	2.05E+08	410	2.05E+08	16	5.98198	3.41707
Nc-1-IP_pe	chr1	2.05E+08	2.05E+08	407	2.05E+08	19	9.81043	4.86513
Nc-1-IP_pe	chr1	2.05E+08	2.05E+08	424	2.05E+08	12	4.24064	2.95299
Nc-1-IP_pe	chr1	2.05E+08	2.05E+08	378	2.05E+08	12	4.24064	2.95299
Nc-1-IP_pe	chr1	2.05E+08	2.05E+08	541	2.05E+08	17	7.02067	3.79543
Nc-1-IP_pe	chr1	2.05E+08	2.05E+08	266	2.05E+08	10	3.03453	2.46828
Nc-1-IP_pe	chr1	2.05E+08	2.05E+08	253	2.05E+08	15	5.20142	3.14789
Nc-1-IP_pe	chr1	2.05E+08	2.05E+08	489	2.05E+08	14	3.71352	2.5314
Nc-1-IP_pe	chr1	2.05E+08	2.05E+08	285	2.05E+08	21	5.34474	2.75017
Nc-1-IP_pe	chr1	2.05E+08	2.05E+08	457	2.05E+08	16	5.85814	3.35873
Nc-1-IP_pe	chr1	2.05E+08	2.05E+08	426	2.05E+08	11	4.40231	3.14376
Nc-1-IP_pe	chr1	2.05E+08	2.05E+08	353	2.05E+08	14	6.06519	3.69269
Nc-1-IP_pe	chr1	2.05E+08	2.05E+08	1376	2.05E+08	15	6.12018	3.5972
Nc-1-IP_pe	chr1	2.05E+08	2.05E+08	338	2.05E+08	17	4.78101	2.79343
Nc-1-IP_pe	chr1	2.05E+08	2.05E+08	449	2.05E+08	14	5.5512	3.42562
Nc-1-IP_pe	chr1	2.05E+08	2.05E+08	600	2.05E+08	14	5.60432	3.45288
Nc-1-IP_pe	chr1	2.05E+08	2.05E+08	287	2.05E+08	11	4.1834	3.02067
Nc-1-IP_pe	chr1	2.05E+08	2.05E+08	260	2.05E+08	13	3.77558	2.63125
Nc-1-IP_pe	chr1	2.05E+08	2.05E+08	264	2.05E+08	12	3.47963	2.55766
Nc-1-IP_pe	chr1	2.05E+08	2.05E+08	290	2.05E+08	12	3.83688	2.74114
Nc-1-IP_pe	chr1	2.05E+08	2.05E+08	434	2.05E+08	9	3.27326	2.68684
Nc-1-IP_pe	chr1	2.05E+08	2.05E+08	549	2.05E+08	16	3.39261	2.27748
Nc-1-IP_pe	chr1	2.05E+08	2.05E+08	754	2.05E+08	12	4.83276	3.2724
Nc-1-IP_pe	chr1	2.05E+08	2.05E+08	381	2.05E+08	11	3.64839	2.72584
Nc-1-IP_pe	chr1	2.05E+08	2.05E+08	309	2.05E+08	12	5.23082	3.49289
Nc-1-IP_pe	chr1	2.05E+08	2.05E+08	263	2.05E+08	9	3.92293	3.08
Nc-1-IP_pe	chr1	2.05E+08	2.05E+08	270	2.05E+08	14	3.55308	2.45762
Nc-1-IP_pe	chr1	2.05E+08	2.05E+08	1137	2.05E+08	18	10.8413	5.58447
Nc-1-IP_pe	chr1	2.05E+08	2.05E+08	345	2.05E+08	14	6.92464	4.15548
Nc-1-IP_pe	chr1	2.05E+08	2.05E+08	380	2.05E+08	16	9.2257	5.08707
Nc-1-IP_pe	chr1	2.05E+08	2.05E+08	296	2.05E+08	14	5.03085	3.16286
Nc-1-IP_pe	chr1	2.05E+08	2.05E+08	302	2.05E+08	15	6.69889	3.8921
Nc-1-IP_pe	chr1	2.05E+08	2.05E+08	490	2.05E+08	12	4.88508	3.30112

Nc-1-IP_pe	chr1	2.05E+08	2.05E+08	693	2.05E+08	13	5.36853	3.44651
Nc-1-IP_pe	chr1	2.05E+08	2.05E+08	407	2.05E+08	16	7.49289	4.16161
Nc-1-IP_pe	chr1	2.05E+08	2.05E+08	395	2.05E+08	13	5.40091	3.46384
Nc-1-IP_pe	chr1	2.05E+08	2.05E+08	523	2.05E+08	14	6.72985	4.04886
Nc-1-IP_pe	chr1	2.05E+08	2.05E+08	357	2.05E+08	13	3.77558	2.63125
Nc-1-IP_pe	chr1	2.05E+08	2.05E+08	920	2.05E+08	14	6.06519	3.69269
Nc-1-IP_pe	chr1	2.05E+08	2.05E+08	781	2.05E+08	16	4.59224	2.7853
Nc-1-IP_pe	chr1	2.05E+08	2.05E+08	271	2.05E+08	13	4.02562	2.7544
Nc-1-IP_pe	chr1	2.05E+08	2.05E+08	277	2.05E+08	17	7.6885	4.11887
Nc-1-IP_pe	chr1	2.05E+08	2.05E+08	496	2.05E+08	15	6.62049	3.85162
Nc-1-IP_pe	chr1	2.05E+08	2.05E+08	397	2.05E+08	14	4.42328	2.86573
Nc-1-IP_pe	chr1	2.05E+08	2.05E+08	334	2.05E+08	18	6.63038	3.50359
Nc-1-IP_pe	chr1	2.05E+08	2.05E+08	643	2.05E+08	18	6.65364	3.51391
Nc-1-IP_pe	chr1	2.05E+08	2.05E+08	752	2.05E+08	15	6.12018	3.5972
Nc-1-IP_pe	chr1	2.05E+08	2.05E+08	372	2.05E+08	13	4.02562	2.7544
Nc-1-IP_pe	chr1	2.05E+08	2.05E+08	377	2.05E+08	11	3.22564	2.49886
Nc-1-IP_pe	chr1	2.05E+08	2.05E+08	254	2.05E+08	12	4.16211	2.91142
Nc-1-IP_pe	chr1	2.05E+08	2.05E+08	270	2.05E+08	13	3.86418	2.67468
Nc-1-IP_pe	chr1	2.05E+08	2.05E+08	987	2.05E+08	17	7.87345	4.21046
Nc-1-IP_pe	chr1	2.05E+08	2.05E+08	549	2.05E+08	13	6.31249	3.96385
Nc-1-IP_pe	chr1	2.05E+08	2.05E+08	307	2.05E+08	11	3.28475	2.53029
Nc-1-IP_pe	chr1	2.05E+08	2.05E+08	398	2.05E+08	17	9.09431	4.8363
Nc-1-IP_pe	chr1	2.05E+08	2.05E+08	315	2.05E+08	13	4.02562	2.7544
Nc-1-IP_pe	chr1	2.05E+08	2.05E+08	619	2.05E+08	12	4.28158	2.97474
Nc-1-IP_pe	chr1	2.05E+08	2.05E+08	659	2.05E+08	13	5.29187	3.40559
Nc-1-IP_pe	chr1	2.05E+08	2.05E+08	324	2.05E+08	16	7.8117	4.32634
Nc-1-IP_pe	chr1	2.05E+08	2.05E+08	533	2.05E+08	17	4.78101	2.79343
Nc-1-IP_pe	chr1	2.05E+08	2.05E+08	673	2.05E+08	19	9.15219	4.54307
Nc-1-IP_pe	chr1	2.05E+08	2.05E+08	340	2.05E+08	18	8.37884	4.31591
Nc-1-IP_pe	chr1	2.05E+08	2.05E+08	815	2.05E+08	12	4.06753	2.86157
Nc-1-IP_pe	chr1	2.05E+08	2.05E+08	281	2.05E+08	15	6.69889	3.8921
Nc-1-IP_pe	chr1	2.05E+08	2.05E+08	397	2.05E+08	13	4.67336	3.0817
Nc-1-IP_pe	chr1	2.05E+08	2.05E+08	675	2.05E+08	15	6.19428	3.63445
Nc-1-IP_pe	chr1	2.05E+08	2.05E+08	956	2.05E+08	13	7.47517	4.63258
Nc-1-IP_pe	chr1	2.05E+08	2.05E+08	364	2.05E+08	16	8.26996	4.56762
Nc-1-IP_pe	chr1	2.05E+08	2.05E+08	377	2.05E+08	14	7.14701	4.27839
Nc-1-IP_pe	chr1	2.05E+08	2.05E+08	263	2.05E+08	16	6.80394	3.81462
Nc-1-IP_pe	chr1	2.05E+08	2.05E+08	312	2.05E+08	12	5.74196	3.78252
Nc-1-IP_pe	chr1	2.05E+08	2.05E+08	437	2.05E+08	13	4.02562	2.7544
Nc-1-IP_pe	chr1	2.05E+08	2.05E+08	525	2.05E+08	13	5.36853	3.44651
Nc-1-IP_pe	chr1	2.05E+08	2.05E+08	468	2.05E+08	11	4.70533	3.31645
Nc-1-IP_pe	chr1	2.05E+08	2.05E+08	285	2.05E+08	15	6.19428	3.63445
Nc-1-IP_pe	chr1	2.05E+08	2.05E+08	443	2.05E+08	12	4.24064	2.95299
Nc-1-IP_pe	chr1	2.05E+08	2.05E+08	425	2.05E+08	11	4.06403	2.95415
Nc-1-IP_pe	chr1	2.05E+08	2.05E+08	329	2.05E+08	23	9.36735	4.11018

Nc-1-IP_pe	chr1	2.05E+08	2.05E+08	436	2.05E+08	24	9.05651	3.87976
Nc-1-IP_pe	chr1	2.05E+08	2.05E+08	262	2.05E+08	15	9.35725	5.35563
Nc-1-IP_pe	chr1	2.05E+08	2.05E+08	315	2.05E+08	18	9.1091	4.6774
Nc-1-IP_pe	chr1	2.05E+08	2.05E+08	285	2.05E+08	18	7.73136	4.00629
Nc-1-IP_pe	chr1	2.05E+08	2.05E+08	540	2.05E+08	16	6.58466	3.7068
Nc-1-IP_pe	chr1	2.05E+08	2.05E+08	403	2.05E+08	10	2.88442	2.38504
Nc-1-IP_pe	chr1	2.05E+08	2.05E+08	279	2.05E+08	10	3.08907	2.49869
Nc-1-IP_pe	chr1	2.05E+08	2.05E+08	479	2.05E+08	14	3.55308	2.45762
Nc-1-IP_pe	chr1	2.05E+08	2.05E+08	793	2.05E+08	16	5.82808	3.34463
Nc-1-IP_pe	chr1	2.05E+08	2.05E+08	886	2.05E+08	13	3.67377	2.58159
Nc-1-IP_pe	chr1	2.05E+08	2.05E+08	288	2.05E+08	24	8.53053	3.68205
Nc-1-IP_pe	chr1	2.05E+08	2.05E+08	330	2.05E+08	15	4.40338	2.77622
Nc-1-IP_pe	chr1	2.05E+08	2.05E+08	954	2.05E+08	14	4.23163	2.77414
Nc-1-IP_pe	chr1	2.05E+08	2.05E+08	681	2.05E+08	16	6.37501	3.60492
Nc-1-IP_pe	chr1	2.05E+08	2.05E+08	965	2.05E+08	15	6.02372	3.54894
Nc-1-IP_pe	chr1	2.05E+08	2.05E+08	545	2.05E+08	13	4.41954	2.952
Nc-1-IP_pe	chr1	2.05E+08	2.05E+08	254	2.05E+08	13	4.60566	3.04692
Nc-1-IP_pe	chr1	2.05E+08	2.05E+08	634	2.05E+08	16	5.37454	3.13479
Nc-1-IP_pe	chr1	2.05E+08	2.05E+08	329	2.05E+08	11	3.94586	2.88872
Nc-1-IP_pe	chr1	2.05E+08	2.05E+08	300	2.05E+08	12	4.24064	2.95299
Nc-1-IP_pe	chr1	2.05E+08	2.05E+08	376	2.05E+08	16	4.96462	2.94973
Nc-1-IP_pe	chr1	2.05E+08	2.05E+08	392	2.05E+08	15	7.46994	4.29894
Nc-1-IP_pe	chr1	2.05E+08	2.05E+08	616	2.05E+08	11	3.64839	2.72584
Nc-1-IP_pe	chr1	2.05E+08	2.05E+08	299	2.05E+08	12	6.528	4.24104
Nc-1-IP_pe	chr1	2.05E+08	2.05E+08	1190	2.05E+08	14	5.18557	3.24018
Nc-1-IP_pe	chr1	2.05E+08	2.05E+08	576	2.05E+08	12	4.24064	2.95299
Nc-1-IP_pe	chr1	2.05E+08	2.05E+08	489	2.05E+08	11	3.06138	2.41205
Nc-1-IP_pe	chr1	2.05E+08	2.05E+08	342	2.05E+08	13	4.41954	2.952
Nc-1-IP_pe	chr1	2.05E+08	2.05E+08	758	2.05E+08	14	4.60011	2.95114
Nc-1-IP_pe	chr1	2.05E+08	2.05E+08	311	2.05E+08	16	3.1497	2.1782
Nc-1-IP_pe	chr1	2.05E+08	2.05E+08	478	2.05E+08	15	5.33233	3.21049
Nc-1-IP_pe	chr1	2.05E+08	2.05E+08	494	2.05E+08	17	5.7822	3.22586
Nc-1-IP_pe	chr1	2.05E+08	2.05E+08	322	2.05E+08	16	5.31851	3.10924
Nc-1-IP_pe	chr1	2.05E+08	2.05E+08	469	2.05E+08	13	3.35769	2.42919
Nc-1-IP_pe	chr1	2.05E+08	2.05E+08	772	2.05E+08	15	5.99655	3.53539
Nc-1-IP_pe	chr1	2.05E+08	2.05E+08	320	2.05E+08	12	3.67829	2.65924
Nc-1-IP_pe	chr1	2.05E+08	2.05E+08	477	2.05E+08	10	3.49455	2.72742
Nc-1-IP_pe	chr1	2.05E+08	2.05E+08	727	2.05E+08	17	4.44438	2.65333
Nc-1-IP_pe	chr1	2.05E+08	2.05E+08	377	2.05E+08	15	6.03738	3.55576
Nc-1-IP_pe	chr1	2.05E+08	2.05E+08	298	2.05E+08	11	3.59688	2.6979
Nc-1-IP_pe	chr1	2.05E+08	2.05E+08	584	2.05E+08	12	4.25953	2.96302
Nc-1-IP_pe	chr1	2.05E+08	2.05E+08	304	2.05E+08	15	6.39297	3.73508
Nc-1-IP_pe	chr1	2.05E+08	2.05E+08	345	2.05E+08	12	4.24856	2.9572
Nc-1-IP_pe	chr1	2.06E+08	2.06E+08	353	2.06E+08	16	3.44488	2.29898
Nc-1-IP_pe	chr1	2.06E+08	2.06E+08	371	2.06E+08	21	7.4689	3.5571

Nc-1-IP_pe	chr1	2.06E+08	2.06E+08	812	2.06E+08	23	12.1465	5.31323
Nc-1-IP_pe	chr1	2.06E+08	2.06E+08	412	2.06E+08	15	4.40338	2.77622
Nc-1-IP_pe	chr1	2.06E+08	2.06E+08	898	2.06E+08	18	7.46027	3.87973
Nc-1-IP_pe	chr1	2.06E+08	2.06E+08	736	2.06E+08	16	3.94146	2.50592
Nc-1-IP_pe	chr1	2.06E+08	2.06E+08	633	2.06E+08	17	3.84884	2.41145
Nc-1-IP_pe	chr1	2.06E+08	2.06E+08	443	2.06E+08	16	3.65575	2.38624
Nc-1-IP_pe	chr1	2.06E+08	2.06E+08	543	2.06E+08	16	4.96462	2.94973
Nc-1-IP_pe	chr1	2.06E+08	2.06E+08	355	2.06E+08	12	4.06753	2.86157
Nc-1-IP_pe	chr1	2.06E+08	2.06E+08	291	2.06E+08	10	3.00887	2.45401
Nc-1-IP_pe	chr1	2.06E+08	2.06E+08	1083	2.06E+08	19	8.19109	4.09114
Nc-1-IP_pe	chr1	2.06E+08	2.06E+08	381	2.06E+08	14	3.55308	2.45762
Nc-1-IP_pe	chr1	2.06E+08	2.06E+08	468	2.06E+08	20	9.94634	4.77022
Nc-1-IP_pe	chr1	2.06E+08	2.06E+08	354	2.06E+08	17	8.17235	4.36029
Nc-1-IP_pe	chr1	2.06E+08	2.06E+08	637	2.06E+08	11	3.64839	2.72584
Nc-1-IP_pe	chr1	2.06E+08	2.06E+08	620	2.06E+08	20	9.21699	4.428
Nc-1-IP_pe	chr1	2.06E+08	2.06E+08	1352	2.06E+08	15	7.18121	4.14477
Nc-1-IP_pe	chr1	2.06E+08	2.06E+08	726	2.06E+08	16	5.73763	3.30235
Nc-1-IP_pe	chr1	2.06E+08	2.06E+08	279	2.06E+08	16	3.65575	2.38624
Nc-1-IP_pe	chr1	2.06E+08	2.06E+08	626	2.06E+08	14	4.47284	2.88959
Nc-1-IP_pe	chr1	2.06E+08	2.06E+08	998	2.06E+08	22	8.79433	3.99081
Nc-1-IP_pe	chr1	2.06E+08	2.06E+08	533	2.06E+08	20	8.11501	3.93409
Nc-1-IP_pe	chr1	2.06E+08	2.06E+08	529	2.06E+08	15	4.92438	3.01694
Nc-1-IP_pe	chr1	2.06E+08	2.06E+08	473	2.06E+08	18	9.07665	4.66107
Nc-1-IP_pe	chr1	2.06E+08	2.06E+08	261	2.06E+08	13	4.86362	3.18015
Nc-1-IP_pe	chr1	2.06E+08	2.06E+08	316	2.06E+08	11	5.22568	3.61895
Nc-1-IP_pe	chr1	2.06E+08	2.06E+08	649	2.06E+08	16	7.53857	4.18505
Nc-1-IP_pe	chr1	2.06E+08	2.06E+08	632	2.06E+08	11	4.54404	3.2242
Nc-1-IP_pe	chr1	2.06E+08	2.06E+08	588	2.06E+08	16	8.26996	4.56762
Nc-1-IP_pe	chr1	2.06E+08	2.06E+08	341	2.06E+08	12	5.20166	3.47658
Nc-1-IP_pe	chr1	2.06E+08	2.06E+08	303	2.06E+08	10	3.4604	2.70797
Nc-1-IP_pe	chr1	2.06E+08	2.06E+08	485	2.06E+08	12	4.93819	3.33035
Nc-1-IP_pe	chr1	2.06E+08	2.06E+08	305	2.06E+08	18	6.15736	3.29676
Nc-1-IP_pe	chr1	2.06E+08	2.06E+08	327	2.06E+08	14	5.78183	3.54454
Nc-1-IP_pe	chr1	2.06E+08	2.06E+08	501	2.06E+08	14	5.03085	3.16286
Nc-1-IP_pe	chr1	2.06E+08	2.06E+08	473	2.06E+08	15	4.06016	2.62146
Nc-1-IP_pe	chr1	2.06E+08	2.06E+08	403	2.06E+08	22	8.79433	3.99081
Nc-1-IP_pe	chr1	2.06E+08	2.06E+08	256	2.06E+08	15	2.95965	2.14351
Nc-1-IP_pe	chr1	2.06E+08	2.06E+08	416	2.06E+08	15	4.06016	2.62146
Nc-1-IP_pe	chr1	2.06E+08	2.06E+08	963	2.06E+08	17	6.47874	3.54137
Nc-1-IP_pe	chr1	2.06E+08	2.06E+08	552	2.06E+08	15	4.40338	2.77622
Nc-1-IP_pe	chr1	2.06E+08	2.06E+08	362	2.06E+08	13	4.02562	2.7544
Nc-1-IP_pe	chr1	2.06E+08	2.06E+08	482	2.06E+08	16	4.25252	2.63824
Nc-1-IP_pe	chr1	2.06E+08	2.06E+08	408	2.06E+08	14	5.51541	3.4073
Nc-1-IP_pe	chr1	2.06E+08	2.06E+08	781	2.06E+08	17	8.46937	4.51138
Nc-1-IP_pe	chr1	2.06E+08	2.06E+08	288	2.06E+08	15	6.42267	3.75021

Nc-1-IP_pe	chr1	2.06E+08	2.06E+08	388	2.06E+08	15	8.26005	4.73146
Nc-1-IP_pe	chr1	2.06E+08	2.06E+08	407	2.06E+08	19	5.14691	2.80294
Nc-1-IP_pe	chr1	2.06E+08	2.06E+08	289	2.06E+08	14	6.06519	3.69269
Nc-1-IP_pe	chr1	2.06E+08	2.06E+08	253	2.06E+08	14	5.56442	3.43239
Nc-1-IP_pe	chr1	2.06E+08	2.06E+08	380	2.06E+08	16	6.33285	3.58457
Nc-1-IP_pe	chr1	2.06E+08	2.06E+08	288	2.06E+08	15	5.58327	3.33181
Nc-1-IP_pe	chr1	2.06E+08	2.06E+08	419	2.06E+08	12	3.97564	2.81341
Nc-1-IP_pe	chr1	2.06E+08	2.06E+08	263	2.06E+08	13	4.9575	3.22911
Nc-1-IP_pe	chr1	2.06E+08	2.06E+08	335	2.06E+08	14	5.48576	3.39215
Nc-1-IP_pe	chr1	2.06E+08	2.06E+08	364	2.06E+08	13	4.02562	2.7544
Nc-1-IP_pe	chr1	2.06E+08	2.06E+08	352	2.06E+08	16	3.65575	2.38624
Nc-1-IP_pe	chr1	2.06E+08	2.06E+08	590	2.06E+08	14	5.03085	3.16286
Nc-1-IP_pe	chr1	2.06E+08	2.06E+08	422	2.06E+08	11	5.10577	3.5486
Nc-1-IP_pe	chr1	2.06E+08	2.06E+08	613	2.06E+08	15	4.40338	2.77622
Nc-1-IP_pe	chr1	2.06E+08	2.06E+08	360	2.06E+08	10	4.26734	3.17663
Nc-1-IP_pe	chr1	2.06E+08	2.06E+08	300	2.06E+08	15	6.03738	3.55576
Nc-1-IP_pe	chr1	2.06E+08	2.06E+08	255	2.06E+08	15	5.66913	3.37372
Nc-1-IP_pe	chr1	2.06E+08	2.06E+08	358	2.06E+08	16	6.68575	3.75635
Nc-1-IP_pe	chr1	2.06E+08	2.06E+08	356	2.06E+08	16	5.28625	3.09457
Nc-1-IP_pe	chr1	2.06E+08	2.06E+08	253	2.06E+08	14	3.55308	2.45762
Nc-1-IP_pe	chr1	2.06E+08	2.06E+08	343	2.06E+08	13	4.56123	3.02417
Nc-1-IP_pe	chr1	2.06E+08	2.06E+08	301	2.06E+08	14	4.8399	3.06836
Nc-1-IP_pe	chr1	2.06E+08	2.06E+08	1065	2.06E+08	19	7.18275	3.64066
Nc-1-IP_pe	chr1	2.06E+08	2.06E+08	274	2.06E+08	16	6.37501	3.60492
Nc-1-IP_pe	chr1	2.06E+08	2.06E+08	262	2.06E+08	12	2.8846	2.25979
Nc-1-IP_pe	chr1	2.06E+08	2.06E+08	901	2.06E+08	18	6.15736	3.29676
Nc-1-IP_pe	chr1	2.06E+08	2.06E+08	290	2.06E+08	12	4.5943	3.14252
Nc-1-IP_pe	chr1	2.06E+08	2.06E+08	320	2.06E+08	12	4.24064	2.95299
Nc-1-IP_pe	chr1	2.06E+08	2.06E+08	801	2.06E+08	20	7.85753	3.8227
Nc-1-IP_pe	chr1	2.06E+08	2.06E+08	330	2.06E+08	14	5.03085	3.16286
Nc-1-IP_pe	chr1	2.06E+08	2.06E+08	347	2.06E+08	19	8.03556	4.02008
Nc-1-IP_pe	chr1	2.06E+08	2.06E+08	481	2.06E+08	15	4.90347	3.00714
Nc-1-IP_pe	chr1	2.06E+08	2.06E+08	353	2.06E+08	15	4.06016	2.62146
Nc-1-IP_pe	chr1	2.06E+08	2.06E+08	325	2.06E+08	14	6.69566	4.03025
Nc-1-IP_pe	chr1	2.06E+08	2.06E+08	449	2.06E+08	11	3.58722	2.69267
Nc-1-IP_pe	chr1	2.06E+08	2.06E+08	364	2.06E+08	15	4.40338	2.77622
Nc-1-IP_pe	chr1	2.06E+08	2.06E+08	306	2.06E+08	17	8.1184	4.33308
Nc-1-IP_pe	chr1	2.06E+08	2.06E+08	951	2.06E+08	16	8.43138	4.65384
Nc-1-IP_pe	chr1	2.06E+08	2.06E+08	296	2.06E+08	16	5.37454	3.13479
Nc-1-IP_pe	chr1	2.06E+08	2.06E+08	314	2.06E+08	14	6.19157	3.75949
Nc-1-IP_pe	chr1	2.06E+08	2.06E+08	355	2.06E+08	11	3.28475	2.53029
Nc-1-IP_pe	chr1	2.06E+08	2.06E+08	922	2.06E+08	19	5.23499	2.8373
Nc-1-IP_pe	chr1	2.06E+08	2.06E+08	277	2.06E+08	16	7.53857	4.18505
Nc-1-IP_pe	chr1	2.06E+08	2.06E+08	339	2.06E+08	13	4.86362	3.18015
Nc-1-IP_pe	chr1	2.06E+08	2.06E+08	456	2.06E+08	13	5.70242	3.62667

Nc-1-IP_pe	chr1	2.06E+08	2.06E+08	332	2.06E+08	14	5.03085	3.16286
Nc-1-IP_pe	chr1	2.06E+08	2.06E+08	434	2.06E+08	16	5.37454	3.13479
Nc-1-IP_pe	chr1	2.06E+08	2.06E+08	681	2.06E+08	19	7.18275	3.64066
Nc-1-IP_pe	chr1	2.06E+08	2.06E+08	280	2.06E+08	10	3.4604	2.70797
Nc-1-IP_pe	chr1	2.06E+08	2.06E+08	442	2.06E+08	10	4.25398	3.16873
Nc-1-IP_pe	chr1	2.06E+08	2.06E+08	262	2.06E+08	11	4.49162	3.19439
Nc-1-IP_pe	chr1	2.06E+08	2.06E+08	277	2.06E+08	14	6.61011	3.98382
Nc-1-IP_pe	chr1	2.06E+08	2.06E+08	829	2.06E+08	18	5.23669	2.90944
Nc-1-IP_pe	chr1	2.06E+08	2.06E+08	1006	2.06E+08	18	7.18674	3.75388
Nc-1-IP_pe	chr1	2.06E+08	2.06E+08	269	2.06E+08	14	6.06519	3.69269
Nc-1-IP_pe	chr1	2.06E+08	2.06E+08	273	2.06E+08	14	6.61011	3.98382
Nc-1-IP_pe	chr1	2.06E+08	2.06E+08	545	2.06E+08	17	7.02067	3.79543
Nc-1-IP_pe	chr1	2.06E+08	2.06E+08	256	2.06E+08	10	4.39571	3.25288
Nc-1-IP_pe	chr1	2.06E+08	2.06E+08	275	2.06E+08	17	7.52577	4.03901
Nc-1-IP_pe	chr1	2.06E+08	2.06E+08	407	2.06E+08	13	4.41954	2.952
Nc-1-IP_pe	chr1	2.06E+08	2.06E+08	411	2.06E+08	17	5.14803	2.94914
Nc-1-IP_pe	chr1	2.06E+08	2.06E+08	278	2.06E+08	12	4.24064	2.95299
Nc-1-IP_pe	chr1	2.06E+08	2.06E+08	316	2.06E+08	14	5.19764	3.24624
Nc-1-IP_pe	chr1	2.06E+08	2.06E+08	866	2.06E+08	15	5.51003	3.29622
Nc-1-IP_pe	chr1	2.06E+08	2.06E+08	762	2.06E+08	24	11.1	4.69865
Nc-1-IP_pe	chr1	2.06E+08	2.06E+08	303	2.06E+08	14	4.8399	3.06836
Nc-1-IP_pe	chr1	2.06E+08	2.06E+08	299	2.06E+08	12	4.20505	2.93413
Nc-1-IP_pe	chr1	2.06E+08	2.06E+08	299	2.06E+08	14	5.92369	3.61842
Nc-1-IP_pe	chr1	2.06E+08	2.06E+08	256	2.06E+08	11	3.28475	2.53029
Nc-1-IP_pe	chr1	2.06E+08	2.06E+08	302	2.06E+08	14	3.86727	2.6027
Nc-1-IP_pe	chr1	2.06E+08	2.06E+08	338	2.06E+08	15	6.21877	3.6468
Nc-1-IP_pe	chr1	2.06E+08	2.06E+08	630	2.06E+08	18	6.63038	3.50359
Nc-1-IP_pe	chr1	2.07E+08	2.07E+08	319	2.07E+08	15	3.20025	2.24588
Nc-1-IP_pe	chr1	2.07E+08	2.07E+08	1389	2.07E+08	19	4.8268	2.67939
Nc-1-IP_pe	chr1	2.07E+08	2.07E+08	886	2.07E+08	17	4.44438	2.65333
Nc-1-IP_pe	chr1	2.07E+08	2.07E+08	697	2.07E+08	15	6.19428	3.63445
Nc-1-IP_pe	chr1	2.07E+08	2.07E+08	280	2.07E+08	16	6.68575	3.75635
Nc-1-IP_pe	chr1	2.07E+08	2.07E+08	457	2.07E+08	14	4.60011	2.95114
Nc-1-IP_pe	chr1	2.07E+08	2.07E+08	382	2.07E+08	17	6.47874	3.54137
Nc-1-IP_pe	chr1	2.07E+08	2.07E+08	603	2.07E+08	12	4.68027	3.18915
Nc-1-IP_pe	chr1	2.07E+08	2.07E+08	362	2.07E+08	16	6.33285	3.58457
Nc-1-IP_pe	chr1	2.07E+08	2.07E+08	464	2.07E+08	17	6.47874	3.54137
Nc-1-IP_pe	chr1	2.07E+08	2.07E+08	353	2.07E+08	13	4.44185	2.96333
Nc-1-IP_pe	chr1	2.07E+08	2.07E+08	429	2.07E+08	11	3.28475	2.53029
Nc-1-IP_pe	chr1	2.07E+08	2.07E+08	524	2.07E+08	12	4.70082	3.20033
Nc-1-IP_pe	chr1	2.07E+08	2.07E+08	312	2.07E+08	17	7.02067	3.79543
Nc-1-IP_pe	chr1	2.07E+08	2.07E+08	839	2.07E+08	13	5.9487	3.76157
Nc-1-IP_pe	chr1	2.07E+08	2.07E+08	388	2.07E+08	15	6.85973	3.97568
Nc-1-IP_pe	chr1	2.07E+08	2.07E+08	664	2.07E+08	60	41.1403	9.46661
Nc-1-IP_pe	chr1	2.07E+08	2.07E+08	410	2.07E+08	11	4.49162	3.19439

Nc-1-IP_pe	chr1	2.07E+08	2.07E+08	255	2.07E+08	11	5.06889	3.52703
Nc-1-IP_pe	chr1	2.07E+08	2.07E+08	412	2.07E+08	20	6.08336	3.09555
Nc-1-IP_pe	chr1	2.07E+08	2.07E+08	262	2.07E+08	12	5.45448	3.61875
Nc-1-IP_pe	chr1	2.07E+08	2.07E+08	326	2.07E+08	17	4.44438	2.65333
Nc-1-IP_pe	chr1	2.07E+08	2.07E+08	356	2.07E+08	10	3.4604	2.70797
Nc-1-IP_pe	chr1	2.07E+08	2.07E+08	385	2.07E+08	14	5.838	3.57373
Nc-1-IP_pe	chr1	2.07E+08	2.07E+08	378	2.07E+08	14	4.32616	2.81919
Nc-1-IP_pe	chr1	2.07E+08	2.07E+08	334	2.07E+08	15	6.19428	3.63445
Nc-1-IP_pe	chr1	2.07E+08	2.07E+08	269	2.07E+08	13	5.74741	3.65119
Nc-1-IP_pe	chr1	2.07E+08	2.07E+08	293	2.07E+08	16	7.53857	4.18505
Nc-1-IP_pe	chr1	2.07E+08	2.07E+08	253	2.07E+08	16	3.94146	2.50592
Nc-1-IP_pe	chr1	2.07E+08	2.07E+08	867	2.07E+08	18	8.37884	4.31591
Nc-1-IP_pe	chr1	2.07E+08	2.07E+08	866	2.07E+08	18	7.73136	4.00629
Nc-1-IP_pe	chr1	2.07E+08	2.07E+08	531	2.07E+08	13	4.14549	2.81406
Nc-1-IP_pe	chr1	2.07E+08	2.07E+08	260	2.07E+08	15	5.82387	3.44976
Nc-1-IP_pe	chr1	2.07E+08	2.07E+08	779	2.07E+08	16	6.24025	3.54005
Nc-1-IP_pe	chr1	2.07E+08	2.07E+08	284	2.07E+08	16	5.82808	3.34463
Nc-1-IP_pe	chr1	2.07E+08	2.07E+08	367	2.07E+08	16	7.53857	4.18505
Nc-1-IP_pe	chr1	2.07E+08	2.07E+08	415	2.07E+08	15	6.19428	3.63445
Nc-1-IP_pe	chr1	2.07E+08	2.07E+08	262	2.07E+08	12	3.47963	2.55766
Nc-1-IP_pe	chr1	2.07E+08	2.07E+08	563	2.07E+08	12	5.9637	3.91034
Nc-1-IP_pe	chr1	2.07E+08	2.07E+08	271	2.07E+08	15	7.77584	4.46458
Nc-1-IP_pe	chr1	2.07E+08	2.07E+08	269	2.07E+08	12	5.3037	3.53374
Nc-1-IP_pe	chr1	2.07E+08	2.07E+08	295	2.07E+08	13	6.90485	4.30056
Nc-1-IP_pe	chr1	2.07E+08	2.07E+08	560	2.07E+08	13	5.35966	3.44177
Nc-1-IP_pe	chr1	2.07E+08	2.07E+08	323	2.07E+08	11	4.30316	3.08784
Nc-1-IP_pe	chr1	2.07E+08	2.07E+08	378	2.07E+08	20	5.34663	2.81336
Nc-1-IP_pe	chr1	2.07E+08	2.07E+08	343	2.07E+08	17	7.30608	3.93226
Nc-1-IP_pe	chr1	2.07E+08	2.07E+08	498	2.07E+08	26	9.86937	3.97999
Nc-1-IP_pe	chr1	2.07E+08	2.07E+08	706	2.07E+08	13	5.31884	3.41997
Nc-1-IP_pe	chr1	2.07E+08	2.07E+08	367	2.07E+08	12	4.81979	3.26529
Nc-1-IP_pe	chr1	2.07E+08	2.07E+08	547	2.07E+08	13	4.41954	2.952
Nc-1-IP_pe	chr1	2.07E+08	2.07E+08	273	2.07E+08	12	5.00569	3.36763
Nc-1-IP_pe	chr1	2.07E+08	2.07E+08	472	2.07E+08	11	4.44029	3.16526
Nc-1-IP_pe	chr1	2.07E+08	2.07E+08	366	2.07E+08	14	6.28469	3.80898
Nc-1-IP_pe	chr1	2.07E+08	2.07E+08	268	2.07E+08	16	6.33285	3.58457
Nc-1-IP_pe	chr1	2.07E+08	2.07E+08	413	2.07E+08	12	5.01934	3.37518
Nc-1-IP_pe	chr1	2.07E+08	2.07E+08	273	2.07E+08	16	5.82808	3.34463
Nc-1-IP_pe	chr1	2.07E+08	2.07E+08	493	2.07E+08	13	4.74235	3.11727
Nc-1-IP_pe	chr1	2.07E+08	2.07E+08	278	2.07E+08	14	5.61771	3.45976
Nc-1-IP_pe	chr1	2.07E+08	2.07E+08	324	2.07E+08	12	4.63088	3.16234
Nc-1-IP_pe	chr1	2.07E+08	2.07E+08	318	2.07E+08	15	5.66913	3.37372
Nc-1-IP_pe	chr1	2.07E+08	2.07E+08	797	2.07E+08	14	3.86727	2.6027
Nc-1-IP_pe	chr1	2.07E+08	2.07E+08	513	2.07E+08	16	7.8117	4.32634
Nc-1-IP_pe	chr1	2.07E+08	2.07E+08	565	2.07E+08	15	5.4619	3.27292

Nc-1-IP_pe	chr1	2.07E+08	2.07E+08	412	2.07E+08	18	6.63038	3.50359
Nc-1-IP_pe	chr1	2.07E+08	2.07E+08	780	2.07E+08	19	8.4638	4.21715
Nc-1-IP_pe	chr1	2.07E+08	2.07E+08	257	2.07E+08	15	5.4619	3.27292
Nc-1-IP_pe	chr1	2.07E+08	2.07E+08	259	2.07E+08	16	8.26996	4.56762
Nc-1-IP_pe	chr1	2.07E+08	2.07E+08	351	2.07E+08	15	5.20142	3.14789
Nc-1-IP_pe	chr1	2.07E+08	2.07E+08	389	2.07E+08	14	7.2043	4.31026
Nc-1-IP_pe	chr1	2.07E+08	2.07E+08	282	2.07E+08	16	9.10831	5.02214
Nc-1-IP_pe	chr1	2.07E+08	2.07E+08	424	2.07E+08	8	3.08755	2.66145
Nc-1-IP_pe	chr1	2.07E+08	2.07E+08	579	2.07E+08	10	4.79071	3.49018
Nc-1-IP_pe	chr1	2.07E+08	2.07E+08	437	2.07E+08	21	9.17453	4.27363
Nc-1-IP_pe	chr1	2.07E+08	2.07E+08	439	2.07E+08	12	6.40869	4.1705
Nc-1-IP_pe	chr1	2.07E+08	2.07E+08	308	2.07E+08	13	5.86991	3.71823
Nc-1-IP_pe	chr1	2.08E+08	2.08E+08	385	2.08E+08	9	3.72192	2.95716
Nc-1-IP_pe	chr1	2.08E+08	2.08E+08	943	2.08E+08	17	6.13258	3.38303
Nc-1-IP_pe	chr1	2.08E+08	2.08E+08	413	2.08E+08	12	4.24064	2.95299
Nc-1-IP_pe	chr1	2.08E+08	2.08E+08	620	2.08E+08	12	4.24064	2.95299
Nc-1-IP_pe	chr1	2.08E+08	2.08E+08	374	2.08E+08	17	8.7831	4.67333
Nc-1-IP_pe	chr1	2.08E+08	2.08E+08	295	2.08E+08	19	11.1305	5.54065
Nc-1-IP_pe	chr1	2.08E+08	2.08E+08	264	2.08E+08	15	4.70031	2.91253
Nc-1-IP_pe	chr1	2.08E+08	2.08E+08	973	2.08E+08	18	9.94172	5.10499
Nc-1-IP_pe	chr1	2.08E+08	2.08E+08	264	2.08E+08	12	6.58898	4.27722
Nc-1-IP_pe	chr1	2.08E+08	2.08E+08	492	2.08E+08	14	4.21449	2.76599
Nc-1-IP_pe	chr1	2.08E+08	2.08E+08	322	2.08E+08	15	5.66913	3.37372
Nc-1-IP_pe	chr1	2.08E+08	2.08E+08	740	2.08E+08	18	9.05704	4.65121
Nc-1-IP_pe	chr1	2.08E+08	2.08E+08	1025	2.08E+08	17	5.7822	3.22586
Nc-1-IP_pe	chr1	2.08E+08	2.08E+08	478	2.08E+08	24	12.0853	5.12299
Nc-1-IP_pe	chr1	2.08E+08	2.08E+08	584	2.08E+08	16	4.59224	2.7853
Nc-1-IP_pe	chr1	2.08E+08	2.08E+08	375	2.08E+08	14	4.21449	2.76599
Nc-1-IP_pe	chr1	2.08E+08	2.08E+08	608	2.08E+08	19	9.81043	4.86513
Nc-1-IP_pe	chr1	2.08E+08	2.08E+08	361	2.08E+08	19	6.78661	3.47027
Nc-1-IP_pe	chr1	2.08E+08	2.08E+08	280	2.08E+08	10	3.8436	2.92819
Nc-1-IP_pe	chr1	2.08E+08	2.08E+08	465	2.08E+08	16	5.85814	3.35873
Nc-1-IP_pe	chr1	2.08E+08	2.08E+08	295	2.08E+08	11	4.74697	3.34038
Nc-1-IP_pe	chr1	2.08E+08	2.08E+08	280	2.08E+08	13	6.29523	3.95418
Nc-1-IP_pe	chr1	2.08E+08	2.08E+08	275	2.08E+08	11	4.54404	3.2242
Nc-1-IP_pe	chr1	2.08E+08	2.08E+08	759	2.08E+08	12	5.45448	3.61875
Nc-1-IP_pe	chr1	2.08E+08	2.08E+08	813	2.08E+08	12	4.63088	3.16234
Nc-1-IP_pe	chr1	2.08E+08	2.08E+08	313	2.08E+08	13	6.92473	4.312
Nc-1-IP_pe	chr1	2.08E+08	2.08E+08	335	2.08E+08	15	9.55381	5.47001
Nc-1-IP_pe	chr1	2.08E+08	2.08E+08	300	2.08E+08	9	4.46937	3.41876
Nc-1-IP_pe	chr1	2.08E+08	2.08E+08	333	2.08E+08	12	6.07041	3.97229
Nc-1-IP_pe	chr1	2.08E+08	2.08E+08	273	2.08E+08	12	5.15877	3.45264
Nc-1-IP_pe	chr1	2.08E+08	2.08E+08	1144	2.08E+08	21	7.10961	3.41419
Nc-1-IP_pe	chr1	2.08E+08	2.08E+08	626	2.08E+08	13	3.67377	2.58159
Nc-1-IP_pe	chr1	2.08E+08	2.08E+08	798	2.08E+08	14	7.34079	4.38651

Nc-1-IP_pe	chr1	2.08E+08	2.08E+08	255	2.08E+08	10	4.77495	3.48064
Nc-1-IP_pe	chr1	2.08E+08	2.08E+08	440	2.08E+08	18	8.37884	4.31591
Nc-1-IP_pe	chr1	2.08E+08	2.08E+08	275	2.08E+08	14	5.98179	3.64885
Nc-1-IP_pe	chr1	2.08E+08	2.08E+08	354	2.08E+08	13	4.02562	2.7544
Nc-1-IP_pe	chr1	2.08E+08	2.08E+08	299	2.08E+08	14	6.06519	3.69269
Nc-1-IP_pe	chr1	2.08E+08	2.08E+08	493	2.08E+08	14	5.5512	3.42562
Nc-1-IP_pe	chr1	2.08E+08	2.08E+08	531	2.08E+08	14	6.81736	4.09664
Nc-1-IP_pe	chr1	2.08E+08	2.08E+08	310	2.08E+08	15	4.7819	2.95039
Nc-1-IP_pe	chr1	2.08E+08	2.08E+08	359	2.08E+08	14	5.48576	3.39215
Nc-1-IP_pe	chr1	2.08E+08	2.08E+08	276	2.08E+08	13	4.92076	3.20992
Nc-1-IP_pe	chr1	2.08E+08	2.08E+08	350	2.08E+08	19	5.51632	2.94814
Nc-1-IP_pe	chr1	2.08E+08	2.08E+08	370	2.08E+08	12	3.16137	2.39719
Nc-1-IP_pe	chr1	2.08E+08	2.08E+08	526	2.08E+08	18	8.80785	4.52672
Nc-1-IP_pe	chr1	2.08E+08	2.08E+08	560	2.08E+08	17	6.47874	3.54137
Nc-1-IP_pe	chr1	2.08E+08	2.08E+08	431	2.08E+08	15	6.5434	3.81197
Nc-1-IP_pe	chr1	2.08E+08	2.08E+08	446	2.08E+08	15	6.42267	3.75021
Nc-1-IP_pe	chr1	2.08E+08	2.08E+08	462	2.08E+08	15	3.46204	2.35851
Nc-1-IP_pe	chr1	2.08E+08	2.08E+08	253	2.08E+08	13	5.17277	3.34236
Nc-1-IP_pe	chr1	2.08E+08	2.08E+08	406	2.08E+08	13	4.02562	2.7544
Nc-1-IP_pe	chr1	2.08E+08	2.08E+08	451	2.08E+08	10	3.57781	2.77499
Nc-1-IP_pe	chr1	2.08E+08	2.08E+08	569	2.08E+08	13	5.42865	3.47872
Nc-1-IP_pe	chr1	2.08E+08	2.08E+08	295	2.08E+08	15	5.34394	3.21607
Nc-1-IP_pe	chr1	2.08E+08	2.08E+08	463	2.08E+08	13	5.17277	3.34236
Nc-1-IP_pe	chr1	2.08E+08	2.08E+08	492	2.08E+08	15	4.06016	2.62146
Nc-1-IP_pe	chr1	2.08E+08	2.08E+08	786	2.08E+08	13	4.86362	3.18015
Nc-1-IP_pe	chr1	2.08E+08	2.08E+08	282	2.08E+08	15	4.06016	2.62146
Nc-1-IP_pe	chr1	2.08E+08	2.08E+08	357	2.08E+08	13	4.56123	3.02417
Nc-1-IP_pe	chr1	2.08E+08	2.08E+08	476	2.08E+08	12	4.39405	3.03475
Nc-1-IP_pe	chr1	2.08E+08	2.08E+08	273	2.08E+08	10	3.21261	2.56787
Nc-1-IP_pe	chr1	2.08E+08	2.08E+08	466	2.08E+08	12	3.47963	2.55766
Nc-1-IP_pe	chr1	2.08E+08	2.08E+08	272	2.08E+08	12	4.28158	2.97474
Nc-1-IP_pe	chr1	2.08E+08	2.08E+08	389	2.08E+08	11	4.46586	3.17976
Nc-1-IP_pe	chr1	2.08E+08	2.08E+08	446	2.08E+08	16	4.59224	2.7853
Nc-1-IP_pe	chr1	2.08E+08	2.08E+08	506	2.08E+08	15	6.3782	3.72756
Nc-1-IP_pe	chr1	2.08E+08	2.08E+08	1033	2.08E+08	20	10.4288	5.00322
Nc-1-IP_pe	chr1	2.08E+08	2.08E+08	573	2.08E+08	17	5.7822	3.22586
Nc-1-IP_pe	chr1	2.08E+08	2.08E+08	444	2.08E+08	13	4.69621	3.09346
Nc-1-IP_pe	chr1	2.08E+08	2.08E+08	315	2.08E+08	19	9.29692	4.61301
Nc-1-IP_pe	chr1	2.08E+08	2.08E+08	915	2.08E+08	20	5.70098	2.94771
Nc-1-IP_pe	chr1	2.08E+08	2.08E+08	273	2.08E+08	13	5.71736	3.63481
Nc-1-IP_pe	chr1	2.08E+08	2.08E+08	302	2.08E+08	10	3.89064	2.95552
Nc-1-IP_pe	chr1	2.08E+08	2.08E+08	277	2.08E+08	13	7.12853	4.42991
Nc-1-IP_pe	chr1	2.08E+08	2.08E+08	283	2.08E+08	12	4.70082	3.20033
Nc-1-IP_pe	chr1	2.08E+08	2.08E+08	387	2.08E+08	10	4.20113	3.13749
Nc-1-IP_pe	chr1	2.08E+08	2.08E+08	412	2.08E+08	15	6.78905	3.93887

Nc-1-IP_pe	chr1	2.09E+08	2.09E+08	394	2.09E+08	11	4.35236	3.11555
Nc-1-IP_pe	chr1	2.09E+08	2.09E+08	309	2.09E+08	8	3.7276	3.06575
Nc-1-IP_pe	chr1	2.09E+08	2.09E+08	281	2.09E+08	14	7.42849	4.43574
Nc-1-IP_pe	chr1	2.09E+08	2.09E+08	531	2.09E+08	13	6.72991	4.20021
Nc-1-IP_pe	chr1	2.09E+08	2.09E+08	256	2.09E+08	9	4.24819	3.28084
Nc-1-IP_pe	chr1	2.09E+08	2.09E+08	264	2.09E+08	12	5.23082	3.49289
Nc-1-IP_pe	chr1	2.09E+08	2.09E+08	275	2.09E+08	10	4.39571	3.25288
Nc-1-IP_pe	chr1	2.09E+08	2.09E+08	406	2.09E+08	13	4.86362	3.18015
Nc-1-IP_pe	chr1	2.09E+08	2.09E+08	439	2.09E+08	10	3.89064	2.95552
Nc-1-IP_pe	chr1	2.09E+08	2.09E+08	350	2.09E+08	10	5.23357	3.76063
Nc-1-IP_pe	chr1	2.09E+08	2.09E+08	303	2.09E+08	11	2.96397	2.36091
Nc-1-IP_pe	chr1	2.09E+08	2.09E+08	338	2.09E+08	9	4.46937	3.41876
Nc-1-IP_pe	chr1	2.09E+08	2.09E+08	276	2.09E+08	10	3.83201	2.92147
Nc-1-IP_pe	chr1	2.09E+08	2.09E+08	456	2.09E+08	13	3.07238	2.29378
Nc-1-IP_pe	chr1	2.09E+08	2.09E+08	621	2.09E+08	13	6.69197	4.17855
Nc-1-IP_pe	chr1	2.09E+08	2.09E+08	887	2.09E+08	15	5.20142	3.14789
Nc-1-IP_pe	chr1	2.09E+08	2.09E+08	281	2.09E+08	12	3.47963	2.55766
Nc-1-IP_pe	chr1	2.09E+08	2.09E+08	259	2.09E+08	12	4.70082	3.20033
Nc-1-IP_pe	chr1	2.09E+08	2.09E+08	263	2.09E+08	13	4.02562	2.7544
Nc-1-IP_pe	chr1	2.09E+08	2.09E+08	389	2.09E+08	12	4.63088	3.16234
Nc-1-IP_pe	chr1	2.09E+08	2.09E+08	258	2.09E+08	10	4.39571	3.25288
Nc-1-IP_pe	chr1	2.09E+08	2.09E+08	608	2.09E+08	15	6.78905	3.93887
Nc-1-IP_pe	chr1	2.09E+08	2.09E+08	412	2.09E+08	14	6.69566	4.03025
Nc-1-IP_pe	chr1	2.09E+08	2.09E+08	269	2.09E+08	13	5.77769	3.66773
Nc-1-IP_pe	chr1	2.09E+08	2.09E+08	315	2.09E+08	11	5.10577	3.5486
Nc-1-IP_pe	chr1	2.09E+08	2.09E+08	298	2.09E+08	12	6.10662	3.99337
Nc-1-IP_pe	chr1	2.1E+08	2.1E+08	330	2.1E+08	13	7.29853	4.52898
Nc-1-IP_pe	chr1	2.1E+08	2.1E+08	518	2.1E+08	14	3.86727	2.6027
Nc-1-IP_pe	chr1	2.1E+08	2.1E+08	330	2.1E+08	13	3.35769	2.42919
Nc-1-IP_pe	chr1	2.1E+08	2.1E+08	308	2.1E+08	13	5.71736	3.63481
Nc-1-IP_pe	chr1	2.1E+08	2.1E+08	1041	2.1E+08	13	4.67336	3.0817
Nc-1-IP_pe	chr1	2.1E+08	2.1E+08	439	2.1E+08	15	4.06016	2.62146
Nc-1-IP_pe	chr1	2.1E+08	2.1E+08	267	2.1E+08	14	5.03085	3.16286
Nc-1-IP_pe	chr1	2.1E+08	2.1E+08	872	2.1E+08	14	6.69566	4.03025
Nc-1-IP_pe	chr1	2.1E+08	2.1E+08	1311	2.1E+08	17	7.21469	3.88822
Nc-1-IP_pe	chr1	2.1E+08	2.1E+08	255	2.1E+08	14	5.49876	3.39879
Nc-1-IP_pe	chr1	2.1E+08	2.1E+08	262	2.1E+08	12	4.18351	2.92273
Nc-1-IP_pe	chr1	2.1E+08	2.1E+08	732	2.1E+08	16	4.59224	2.7853
Nc-1-IP_pe	chr1	2.1E+08	2.1E+08	922	2.1E+08	21	7.10961	3.41419
Nc-1-IP_pe	chr1	2.1E+08	2.1E+08	374	2.1E+08	16	7.01793	3.92106
Nc-1-IP_pe	chr1	2.1E+08	2.1E+08	319	2.1E+08	15	5.1177	3.10809
Nc-1-IP_pe	chr1	2.1E+08	2.1E+08	312	2.1E+08	18	7.04825	3.69086
Nc-1-IP_pe	chr1	2.1E+08	2.1E+08	280	2.1E+08	12	3.47963	2.55766
Nc-1-IP_pe	chr1	2.1E+08	2.1E+08	267	2.1E+08	11	4.10219	2.97537
Nc-1-IP_pe	chr1	2.1E+08	2.1E+08	546	2.1E+08	13	6.24389	3.92544

Nc-1-IP_pe	chr1	2.1E+08	2.1E+08	628	2.1E+08	16	5.73763	3.30235
Nc-1-IP_pe	chr1	2.1E+08	2.1E+08	291	2.1E+08	11	4.06403	2.95415
Nc-1-IP_pe	chr1	2.1E+08	2.1E+08	537	2.1E+08	18	7.73136	4.00629
Nc-1-IP_pe	chr1	2.1E+08	2.1E+08	472	2.1E+08	19	10.6915	5.31176
Nc-1-IP_pe	chr1	2.1E+08	2.1E+08	639	2.1E+08	20	3.24562	2.0616
Nc-1-IP_pe	chr1	2.1E+08	2.1E+08	276	2.1E+08	13	5.36853	3.44651
Nc-1-IP_pe	chr1	2.1E+08	2.1E+08	674	2.1E+08	14	3.86727	2.6027
Nc-1-IP_pe	chr1	2.1E+08	2.1E+08	416	2.1E+08	13	7.42513	4.60316
Nc-1-IP_pe	chr1	2.1E+08	2.1E+08	626	2.1E+08	13	6.72991	4.20021
Nc-1-IP_pe	chr1	2.1E+08	2.1E+08	461	2.1E+08	15	7.41419	4.269
Nc-1-IP_pe	chr1	2.1E+08	2.1E+08	561	2.1E+08	11	5.02318	3.50036
Nc-1-IP_pe	chr1	2.1E+08	2.1E+08	456	2.1E+08	13	4.67336	3.0817
Nc-1-IP_pe	chr1	2.1E+08	2.1E+08	301	2.1E+08	16	4.59224	2.7853
Nc-1-IP_pe	chr1	2.1E+08	2.1E+08	318	2.1E+08	16	4.25252	2.63824
Nc-1-IP_pe	chr1	2.1E+08	2.1E+08	329	2.1E+08	10	5.23357	3.76063
Nc-1-IP_pe	chr1	2.1E+08	2.1E+08	928	2.1E+08	16	7.53857	4.18505
Nc-1-IP_pe	chr1	2.1E+08	2.1E+08	887	2.1E+08	16	10.0682	5.56166
Nc-1-IP_pe	chr1	2.1E+08	2.1E+08	730	2.1E+08	12	5.15877	3.45264
Nc-1-IP_pe	chr1	2.1E+08	2.1E+08	462	2.1E+08	11	4.06403	2.95415
Nc-1-IP_pe	chr1	2.1E+08	2.1E+08	344	2.1E+08	15	6.19428	3.63445
Nc-1-IP_pe	chr1	2.1E+08	2.1E+08	441	2.1E+08	12	6.86906	4.44438
Nc-1-IP_pe	chr1	2.1E+08	2.1E+08	453	2.1E+08	13	5.82354	3.69281
Nc-1-IP_pe	chr1	2.1E+08	2.1E+08	387	2.1E+08	21	10.7603	4.99737
Nc-1-IP_pe	chr1	2.1E+08	2.1E+08	309	2.1E+08	14	6.69566	4.03025
Nc-1-IP_pe	chr1	2.1E+08	2.1E+08	375	2.1E+08	12	6.86906	4.44438
Nc-1-IP_pe	chr1	2.1E+08	2.1E+08	605	2.1E+08	20	7.0741	3.49302
Nc-1-IP_pe	chr1	2.1E+08	2.1E+08	450	2.1E+08	13	4.02562	2.7544
Nc-1-IP_pe	chr1	2.1E+08	2.1E+08	585	2.1E+08	14	7.42849	4.43574
Nc-1-IP_pe	chr1	2.1E+08	2.1E+08	346	2.1E+08	9	3.85693	3.03955
Nc-1-IP_pe	chr1	2.1E+08	2.1E+08	733	2.1E+08	13	5.9487	3.76157
Nc-1-IP_pe	chr1	2.1E+08	2.1E+08	300	2.1E+08	15	4.7819	2.95039
Nc-1-IP_pe	chr1	2.1E+08	2.1E+08	291	2.1E+08	11	4.94832	3.45679
Nc-1-IP_pe	chr1	2.1E+08	2.1E+08	368	2.1E+08	9	3.56055	2.85931
Nc-1-IP_pe	chr1	2.1E+08	2.1E+08	457	2.1E+08	13	7.54321	4.67266
Nc-1-IP_pe	chr1	2.1E+08	2.1E+08	321	2.1E+08	10	3.08907	2.49869
Nc-1-IP_pe	chr1	2.1E+08	2.1E+08	269	2.1E+08	12	5.23082	3.49289
Nc-1-IP_pe	chr1	2.1E+08	2.1E+08	330	2.1E+08	14	7.26231	4.34261
Nc-1-IP_pe	chr1	2.1E+08	2.1E+08	607	2.1E+08	10	4.77495	3.48064
Nc-1-IP_pe	chr1	2.1E+08	2.1E+08	571	2.1E+08	13	6.02837	3.80557
Nc-1-IP_pe	chr1	2.1E+08	2.1E+08	259	2.1E+08	11	6.03439	4.10251
Nc-1-IP_pe	chr1	2.11E+08	2.11E+08	809	2.11E+08	13	7.12853	4.42991
Nc-1-IP_pe	chr1	2.11E+08	2.11E+08	285	2.11E+08	8	3.51085	2.92785
Nc-1-IP_pe	chr1	2.11E+08	2.11E+08	721	2.11E+08	17	8.9884	4.78058
Nc-1-IP_pe	chr1	2.11E+08	2.11E+08	601	2.11E+08	12	4.70082	3.20033
Nc-1-IP_pe	chr1	2.11E+08	2.11E+08	400	2.11E+08	10	3.89064	2.95552

Nc-1-IP_pe	chr1	2.11E+08	2.11E+08	615	2.11E+08	11	4.77502	3.35653
Nc-1-IP_pe	chr1	2.11E+08	2.11E+08	521	2.11E+08	12	5.23082	3.49289
Nc-1-IP_pe	chr1	2.11E+08	2.11E+08	326	2.11E+08	14	7.2043	4.31026
Nc-1-IP_pe	chr1	2.11E+08	2.11E+08	365	2.11E+08	9	4.29346	3.30898
Nc-1-IP_pe	chr1	2.11E+08	2.11E+08	592	2.11E+08	14	8.27703	4.92126
Nc-1-IP_pe	chr1	2.11E+08	2.11E+08	301	2.11E+08	11	5.19376	3.60018
Nc-1-IP_pe	chr1	2.11E+08	2.11E+08	331	2.11E+08	12	6.48784	4.21727
Nc-1-IP_pe	chr1	2.11E+08	2.11E+08	334	2.11E+08	12	6.6095	4.28941
Nc-1-IP_pe	chr1	2.11E+08	2.11E+08	294	2.11E+08	13	5.9487	3.76157
Nc-1-IP_pe	chr1	2.11E+08	2.11E+08	495	2.11E+08	12	4.24064	2.95299
Nc-1-IP_pe	chr1	2.11E+08	2.11E+08	325	2.11E+08	13	6.86537	4.27785
Nc-1-IP_pe	chr1	2.11E+08	2.11E+08	303	2.11E+08	12	5.84943	3.84431
Nc-1-IP_pe	chr1	2.11E+08	2.11E+08	512	2.11E+08	11	4.06403	2.95415
Nc-1-IP_pe	chr1	2.11E+08	2.11E+08	268	2.11E+08	9	4.46937	3.41876
Nc-1-IP_pe	chr1	2.11E+08	2.11E+08	265	2.11E+08	8	3.74505	3.07688
Nc-1-IP_pe	chr1	2.11E+08	2.11E+08	881	2.11E+08	14	7.54286	4.50023
Nc-1-IP_pe	chr1	2.11E+08	2.11E+08	890	2.11E+08	11	4.61014	3.26192
Nc-1-IP_pe	chr1	2.11E+08	2.11E+08	377	2.11E+08	13	4.41954	2.952
Nc-1-IP_pe	chr1	2.11E+08	2.11E+08	457	2.11E+08	10	3.30648	2.62073
Nc-1-IP_pe	chr1	2.11E+08	2.11E+08	616	2.11E+08	10	4.83846	3.51913
Nc-1-IP_pe	chr1	2.11E+08	2.11E+08	415	2.11E+08	13	6.19316	3.89711
Nc-1-IP_pe	chr1	2.11E+08	2.11E+08	418	2.11E+08	8	3.74505	3.07688
Nc-1-IP_pe	chr1	2.11E+08	2.11E+08	752	2.11E+08	14	4.42328	2.86573
Nc-1-IP_pe	chr1	2.11E+08	2.11E+08	385	2.11E+08	14	7.42849	4.43574
Nc-1-IP_pe	chr1	2.11E+08	2.11E+08	340	2.11E+08	18	10.9042	5.61861
Nc-1-IP_pe	chr1	2.11E+08	2.11E+08	504	2.11E+08	14	6.44441	3.89444
Nc-1-IP_pe	chr1	2.11E+08	2.11E+08	322	2.11E+08	17	9.33294	4.96282
Nc-1-IP_pe	chr1	2.11E+08	2.11E+08	253	2.11E+08	15	4.06016	2.62146
Nc-1-IP_pe	chr1	2.11E+08	2.11E+08	360	2.11E+08	14	4.21449	2.76599
Nc-1-IP_pe	chr1	2.11E+08	2.11E+08	258	2.11E+08	10	4.39571	3.25288
Nc-1-IP_pe	chr1	2.11E+08	2.11E+08	588	2.11E+08	15	3.46204	2.35851
Nc-1-IP_pe	chr1	2.11E+08	2.11E+08	350	2.11E+08	10	2.95	2.42133
Nc-1-IP_pe	chr1	2.11E+08	2.11E+08	322	2.11E+08	10	3.89064	2.95552
Nc-1-IP_pe	chr1	2.11E+08	2.11E+08	516	2.11E+08	13	4.90859	3.20357
Nc-1-IP_pe	chr1	2.11E+08	2.11E+08	567	2.11E+08	14	7.16603	4.28896
Nc-1-IP_pe	chr1	2.11E+08	2.11E+08	413	2.11E+08	18	11.9194	6.18102
Nc-1-IP_pe	chr1	2.11E+08	2.11E+08	292	2.11E+08	15	5.20142	3.14789
Nc-1-IP_pe	chr1	2.11E+08	2.11E+08	507	2.11E+08	12	3.16137	2.39719
Nc-1-IP_pe	chr1	2.11E+08	2.11E+08	598	2.11E+08	11	4.88957	3.42271
Nc-1-IP_pe	chr1	2.11E+08	2.11E+08	348	2.11E+08	13	4.56123	3.02417
Nc-1-IP_pe	chr1	2.11E+08	2.11E+08	371	2.11E+08	13	4.02562	2.7544
Nc-1-IP_pe	chr1	2.11E+08	2.11E+08	1232	2.11E+08	12	6.23601	4.06898
Nc-1-IP_pe	chr1	2.11E+08	2.11E+08	413	2.11E+08	9	3.27326	2.68684
Nc-1-IP_pe	chr1	2.11E+08	2.11E+08	271	2.11E+08	17	5.54967	3.12324
Nc-1-IP_pe	chr1	2.11E+08	2.11E+08	379	2.11E+08	16	4.96462	2.94973

Nc-1-IP_pe chr1	2.11E+08	2.11E+08	369	2.11E+08	11	4.06403	2.95415
Nc-1-IP_pe chr1	2.11E+08	2.11E+08	306	2.11E+08	15	6.19428	3.63445
Nc-1-IP_pe chr1	2.11E+08	2.11E+08	512	2.11E+08	16	7.11257	3.96852
Nc-1-IP_pe chr1	2.11E+08	2.11E+08	609	2.11E+08	11	4.06403	2.95415
Nc-1-IP_pe chr1	2.12E+08	2.12E+08	444	2.12E+08	14	6.71255	4.03944
Nc-1-IP_pe chr1	2.12E+08	2.12E+08	310	2.12E+08	16	6.37501	3.60492
Nc-1-IP_pe chr1	2.12E+08	2.12E+08	279	2.12E+08	16	5.82808	3.34463
Nc-1-IP_pe chr1	2.12E+08	2.12E+08	261	2.12E+08	17	5.56266	3.12894
Nc-1-IP_pe chr1	2.12E+08	2.12E+08	366	2.12E+08	17	6.64406	3.61808
Nc-1-IP_pe chr1	2.12E+08	2.12E+08	549	2.12E+08	15	6.5895	3.83567
Nc-1-IP_pe chr1	2.12E+08	2.12E+08	559	2.12E+08	12	4.91154	3.31567
Nc-1-IP_pe chr1	2.12E+08	2.12E+08	400	2.12E+08	19	9.01894	4.4791
Nc-1-IP_pe chr1	2.12E+08	2.12E+08	366	2.12E+08	12	5.23082	3.49289
Nc-1-IP_pe chr1	2.12E+08	2.12E+08	326	2.12E+08	11	5.10577	3.5486
Nc-1-IP_pe chr1	2.12E+08	2.12E+08	971	2.12E+08	19	4.5193	2.56258
Nc-1-IP_pe chr1	2.12E+08	2.12E+08	303	2.12E+08	12	4.24064	2.95299
Nc-1-IP_pe chr1	2.12E+08	2.12E+08	667	2.12E+08	19	8.4638	4.21715
Nc-1-IP_pe chr1	2.12E+08	2.12E+08	605	2.12E+08	17	7.06589	3.81697
Nc-1-IP_pe chr1	2.12E+08	2.12E+08	1006	2.12E+08	16	3.94146	2.50592
Nc-1-IP_pe chr1	2.12E+08	2.12E+08	496	2.12E+08	17	6.47874	3.54137
Nc-1-IP_pe chr1	2.12E+08	2.12E+08	636	2.12E+08	14	4.60011	2.95114
Nc-1-IP_pe chr1	2.12E+08	2.12E+08	640	2.12E+08	16	7.39088	4.10945
Nc-1-IP_pe chr1	2.12E+08	2.12E+08	623	2.12E+08	15	7.46994	4.29894
Nc-1-IP_pe chr1	2.12E+08	2.12E+08	683	2.12E+08	17	6.47874	3.54137
Nc-1-IP_pe chr1	2.12E+08	2.12E+08	788	2.12E+08	15	6.78905	3.93887
Nc-1-IP_pe chr1	2.12E+08	2.12E+08	494	2.12E+08	12	4.70523	3.20273
Nc-1-IP_pe chr1	2.12E+08	2.12E+08	291	2.12E+08	14	3.71352	2.5314
Nc-1-IP_pe chr1	2.12E+08	2.12E+08	256	2.12E+08	13	5.9487	3.76157
Nc-1-IP_pe chr1	2.12E+08	2.12E+08	736	2.12E+08	27	11.1537	4.34533
Nc-1-IP_pe chr1	2.12E+08	2.12E+08	350	2.12E+08	11	4.32767	3.10163
Nc-1-IP_pe chr1	2.12E+08	2.12E+08	354	2.12E+08	11	3.95677	2.89474
Nc-1-IP_pe chr1	2.12E+08	2.12E+08	516	2.12E+08	11	2.96397	2.36091
Nc-1-IP_pe chr1	2.12E+08	2.12E+08	486	2.12E+08	12	5.51653	3.6539
Nc-1-IP_pe chr1	2.12E+08	2.12E+08	367	2.12E+08	16	7.42466	4.12669
Nc-1-IP_pe chr1	2.12E+08	2.12E+08	385	2.12E+08	12	4.24064	2.95299
Nc-1-IP_pe chr1	2.12E+08	2.12E+08	794	2.12E+08	14	5.51541	3.4073
Nc-1-IP_pe chr1	2.12E+08	2.12E+08	274	2.12E+08	18	6.91333	3.62993
Nc-1-IP_pe chr1	2.12E+08	2.12E+08	711	2.12E+08	19	7.83948	3.93131
Nc-1-IP_pe chr1	2.12E+08	2.12E+08	410	2.12E+08	21	6.55662	3.19948
Nc-1-IP_pe chr1	2.12E+08	2.12E+08	401	2.12E+08	14	7.42849	4.43574
Nc-1-IP_pe chr1	2.12E+08	2.12E+08	253	2.12E+08	11	6.03439	4.10251
Nc-1-IP_pe chr1	2.12E+08	2.12E+08	513	2.12E+08	18	4.6358	2.66698
Nc-1-IP_pe chr1	2.12E+08	2.12E+08	401	2.12E+08	14	5.51541	3.4073
Nc-1-IP_pe chr1	2.12E+08	2.12E+08	520	2.12E+08	16	4.96462	2.94973
Nc-1-IP_pe chr1	2.12E+08	2.12E+08	614	2.12E+08	11	3.78682	2.80131

Nc-1-IP_pe	chr1	2.12E+08	2.12E+08	362	2.12E+08	14	5.51541	3.4073
Nc-1-IP_pe	chr1	2.12E+08	2.12E+08	529	2.12E+08	17	8.20861	4.37862
Nc-1-IP_pe	chr1	2.12E+08	2.12E+08	299	2.12E+08	13	5.91681	3.74401
Nc-1-IP_pe	chr1	2.12E+08	2.12E+08	290	2.12E+08	14	6.06519	3.69269
Nc-1-IP_pe	chr1	2.12E+08	2.12E+08	596	2.12E+08	16	5.37454	3.13479
Nc-1-IP_pe	chr1	2.12E+08	2.12E+08	574	2.12E+08	11	4.54404	3.2242
Nc-1-IP_pe	chr1	2.12E+08	2.12E+08	260	2.12E+08	12	5.3037	3.53374
Nc-1-IP_pe	chr1	2.12E+08	2.12E+08	273	2.12E+08	12	6.21727	4.05801
Nc-1-IP_pe	chr1	2.12E+08	2.12E+08	471	2.12E+08	15	4.70031	2.91253
Nc-1-IP_pe	chr1	2.12E+08	2.12E+08	592	2.12E+08	13	4.41954	2.952
Nc-1-IP_pe	chr1	2.12E+08	2.12E+08	426	2.12E+08	14	7.1092	4.2574
Nc-1-IP_pe	chr1	2.12E+08	2.12E+08	540	2.12E+08	12	5.65992	3.73556
Nc-1-IP_pe	chr1	2.12E+08	2.12E+08	286	2.12E+08	14	4.95172	3.12357
Nc-1-IP_pe	chr1	2.12E+08	2.12E+08	304	2.12E+08	13	5.9487	3.76157
Nc-1-IP_pe	chr1	2.12E+08	2.12E+08	263	2.12E+08	10	4.12355	3.09176
Nc-1-IP_pe	chr1	2.12E+08	2.12E+08	301	2.12E+08	9	3.53727	2.84525
Nc-1-IP_pe	chr1	2.12E+08	2.12E+08	574	2.12E+08	14	5.51541	3.4073
Nc-1-IP_pe	chr1	2.12E+08	2.12E+08	303	2.12E+08	12	5.23082	3.49289
Nc-1-IP_pe	chr1	2.12E+08	2.12E+08	352	2.12E+08	10	3.68553	2.83683
Nc-1-IP_pe	chr1	2.12E+08	2.12E+08	272	2.12E+08	11	4.06403	2.95415
Nc-1-IP_pe	chr1	2.12E+08	2.12E+08	271	2.12E+08	11	5.10577	3.5486
Nc-1-IP_pe	chr1	2.12E+08	2.12E+08	296	2.12E+08	12	5.77527	3.80164
Nc-1-IP_pe	chr1	2.12E+08	2.12E+08	399	2.12E+08	22	6.23294	3.00503
Nc-1-IP_pe	chr1	2.12E+08	2.12E+08	941	2.12E+08	19	9.15219	4.54307
Nc-1-IP_pe	chr1	2.12E+08	2.12E+08	1625	2.12E+08	18	8.37884	4.31591
Nc-1-IP_pe	chr1	2.12E+08	2.12E+08	644	2.12E+08	12	6.86906	4.44438
Nc-1-IP_pe	chr1	2.12E+08	2.12E+08	322	2.12E+08	11	3.64839	2.72584
Nc-1-IP_pe	chr1	2.12E+08	2.12E+08	435	2.12E+08	18	6.15736	3.29676
Nc-1-IP_pe	chr1	2.12E+08	2.12E+08	630	2.12E+08	16	7.27441	4.05022
Nc-1-IP_pe	chr1	2.12E+08	2.12E+08	293	2.12E+08	11	2.96397	2.36091
Nc-1-IP_pe	chr1	2.12E+08	2.12E+08	549	2.12E+08	14	5.18557	3.24018
Nc-1-IP_pe	chr1	2.12E+08	2.12E+08	772	2.12E+08	13	6.58008	4.11487
Nc-1-IP_pe	chr1	2.12E+08	2.12E+08	256	2.12E+08	16	9.01614	4.97137
Nc-1-IP_pe	chr1	2.12E+08	2.12E+08	521	2.12E+08	13	3.07238	2.29378
Nc-1-IP_pe	chr1	2.12E+08	2.12E+08	346	2.12E+08	15	6.45255	3.76546
Nc-1-IP_pe	chr1	2.12E+08	2.12E+08	363	2.12E+08	11	4.47871	3.18705
Nc-1-IP_pe	chr1	2.12E+08	2.12E+08	342	2.12E+08	10	3.80898	2.90812
Nc-1-IP_pe	chr1	2.12E+08	2.12E+08	356	2.12E+08	14	5.98179	3.64885
Nc-1-IP_pe	chr1	2.12E+08	2.12E+08	357	2.12E+08	12	3.47963	2.55766
Nc-1-IP_pe	chr1	2.12E+08	2.12E+08	262	2.12E+08	18	8.10208	4.1823
Nc-1-IP_pe	chr1	2.12E+08	2.12E+08	415	2.12E+08	13	3.67377	2.58159
Nc-1-IP_pe	chr1	2.12E+08	2.12E+08	544	2.12E+08	13	4.02562	2.7544
Nc-1-IP_pe	chr1	2.12E+08	2.12E+08	412	2.12E+08	17	8.20861	4.37862
Nc-1-IP_pe	chr1	2.12E+08	2.12E+08	307	2.12E+08	11	4.14834	3.00109
Nc-1-IP_pe	chr1	2.12E+08	2.12E+08	265	2.12E+08	11	4.06403	2.95415

Nc-1-IP_pe	chr1	2.12E+08	2.12E+08	452	2.12E+08	13	5.18582	3.34927
Nc-1-IP_pe	chr1	2.12E+08	2.12E+08	375	2.12E+08	15	4.06016	2.62146
Nc-1-IP_pe	chr1	2.12E+08	2.12E+08	667	2.12E+08	15	4.14926	2.66135
Nc-1-IP_pe	chr1	2.12E+08	2.12E+08	275	2.12E+08	17	7.21469	3.88822
Nc-1-IP_pe	chr1	2.12E+08	2.12E+08	341	2.12E+08	17	5.14803	2.94914
Nc-1-IP_pe	chr1	2.12E+08	2.12E+08	422	2.12E+08	12	4.24064	2.95299
Nc-1-IP_pe	chr1	2.13E+08	2.13E+08	389	2.13E+08	15	6.78905	3.93887
Nc-1-IP_pe	chr1	2.13E+08	2.13E+08	405	2.13E+08	18	10.1486	5.21369
Nc-1-IP_pe	chr1	2.13E+08	2.13E+08	769	2.13E+08	16	6.51374	3.67221
Nc-1-IP_pe	chr1	2.13E+08	2.13E+08	613	2.13E+08	15	5.66913	3.37372
Nc-1-IP_pe	chr1	2.13E+08	2.13E+08	385	2.13E+08	13	3.67377	2.58159
Nc-1-IP_pe	chr1	2.13E+08	2.13E+08	292	2.13E+08	13	5.71736	3.63481
Nc-1-IP_pe	chr1	2.13E+08	2.13E+08	384	2.13E+08	16	3.94146	2.50592
Nc-1-IP_pe	chr1	2.13E+08	2.13E+08	414	2.13E+08	14	5.03085	3.16286
Nc-1-IP_pe	chr1	2.13E+08	2.13E+08	354	2.13E+08	10	4.20113	3.13749
Nc-1-IP_pe	chr1	2.13E+08	2.13E+08	726	2.13E+08	18	9.94172	5.10499
Nc-1-IP_pe	chr1	2.13E+08	2.13E+08	897	2.13E+08	19	8.68415	4.32024
Nc-1-IP_pe	chr1	2.13E+08	2.13E+08	350	2.13E+08	13	5.47064	3.50127
Nc-1-IP_pe	chr1	2.13E+08	2.13E+08	271	2.13E+08	12	5.5322	3.6628
Nc-1-IP_pe	chr1	2.13E+08	2.13E+08	2309	2.13E+08	13	3.35769	2.42919
Nc-1-IP_pe	chr1	2.13E+08	2.13E+08	882	2.13E+08	13	4.41954	2.952
Nc-1-IP_pe	chr1	2.13E+08	2.13E+08	406	2.13E+08	12	5.50092	3.64505
Nc-1-IP_pe	chr1	2.13E+08	2.13E+08	585	2.13E+08	10	4.20113	3.13749
Nc-1-IP_pe	chr1	2.13E+08	2.13E+08	422	2.13E+08	12	3.83688	2.74114
Nc-1-IP_pe	chr1	2.13E+08	2.13E+08	382	2.13E+08	16	7.53857	4.18505
Nc-1-IP_pe	chr1	2.13E+08	2.13E+08	406	2.13E+08	14	3.79446	2.56886
Nc-1-IP_pe	chr1	2.13E+08	2.13E+08	494	2.13E+08	17	4.60611	2.72032
Nc-1-IP_pe	chr1	2.13E+08	2.13E+08	568	2.13E+08	14	5.06689	3.18081
Nc-1-IP_pe	chr1	2.13E+08	2.13E+08	433	2.13E+08	12	5.77527	3.80164
Nc-1-IP_pe	chr1	2.13E+08	2.13E+08	348	2.13E+08	13	5.36853	3.44651
Nc-1-IP_pe	chr1	2.13E+08	2.13E+08	1312	2.13E+08	16	6.80394	3.81462
Nc-1-IP_pe	chr1	2.13E+08	2.13E+08	595	2.13E+08	32	12.3151	4.22826
Nc-1-IP_pe	chr1	2.13E+08	2.13E+08	383	2.13E+08	13	3.67377	2.58159
Nc-1-IP_pe	chr1	2.13E+08	2.13E+08	577	2.13E+08	13	4.41954	2.952
Nc-1-IP_pe	chr1	2.13E+08	2.13E+08	676	2.13E+08	13	4.41954	2.952
Nc-1-IP_pe	chr1	2.13E+08	2.13E+08	318	2.13E+08	21	5.5349	2.81884
Nc-1-IP_pe	chr1	2.13E+08	2.13E+08	253	2.13E+08	11	5.77463	3.94557
Nc-1-IP_pe	chr1	2.13E+08	2.13E+08	262	2.13E+08	9	3.27326	2.68684
Nc-1-IP_pe	chr1	2.13E+08	2.13E+08	288	2.13E+08	10	4.47495	3.30017
Nc-1-IP_pe	chr1	2.13E+08	2.13E+08	390	2.13E+08	16	8.81421	4.86082
Nc-1-IP_pe	chr1	2.13E+08	2.13E+08	462	2.13E+08	13	6.65435	4.1571
Nc-1-IP_pe	chr1	2.13E+08	2.13E+08	301	2.13E+08	10	3.89064	2.95552
Nc-1-IP_pe	chr1	2.13E+08	2.13E+08	484	2.13E+08	16	6.33285	3.58457
Nc-1-IP_pe	chr1	2.13E+08	2.13E+08	579	2.13E+08	14	4.60011	2.95114
Nc-1-IP_pe	chr1	2.13E+08	2.13E+08	525	2.13E+08	13	4.34711	2.91533

Nc-1-IP_pe	chr1	2.13E+08	2.13E+08	288	2.13E+08	13	5.73236	3.64298
Nc-1-IP_pe	chr1	2.13E+08	2.13E+08	353	2.13E+08	11	4.00079	2.91908
Nc-1-IP_pe	chr1	2.13E+08	2.13E+08	257	2.13E+08	11	5.74187	3.92588
Nc-1-IP_pe	chr1	2.13E+08	2.13E+08	344	2.13E+08	23	7.01947	3.21526
Nc-1-IP_pe	chr1	2.13E+08	2.13E+08	306	2.13E+08	12	5.84943	3.84431
Nc-1-IP_pe	chr1	2.13E+08	2.13E+08	378	2.13E+08	9	4.46937	3.41876
Nc-1-IP_pe	chr1	2.13E+08	2.13E+08	339	2.13E+08	12	6.86906	4.44438
Nc-1-IP_pe	chr1	2.13E+08	2.13E+08	723	2.13E+08	17	10.6711	5.69559
Nc-1-IP_pe	chr1	2.13E+08	2.13E+08	311	2.13E+08	15	8.61047	4.928
Nc-1-IP_pe	chr1	2.13E+08	2.13E+08	1383	2.13E+08	126	59.9758	6.44254
Nc-1-IP_pe	chr1	2.13E+08	2.13E+08	803	2.13E+08	22	8.24538	3.76835
Nc-1-IP_pe	chr1	2.13E+08	2.13E+08	467	2.13E+08	10	4.99888	3.61677
Nc-1-IP_pe	chr1	2.13E+08	2.13E+08	786	2.13E+08	13	3.35769	2.42919
Nc-1-IP_pe	chr1	2.13E+08	2.13E+08	297	2.13E+08	11	6.03439	4.10251
Nc-1-IP_pe	chr1	2.13E+08	2.13E+08	383	2.13E+08	11	5.03835	3.5092
Nc-1-IP_pe	chr1	2.13E+08	2.13E+08	296	2.13E+08	12	5.84943	3.84431
Nc-1-IP_pe	chr1	2.13E+08	2.13E+08	421	2.13E+08	13	4.02562	2.7544
Nc-1-IP_pe	chr1	2.13E+08	2.13E+08	1178	2.13E+08	11	3.3117	2.54465
Nc-1-IP_pe	chr1	2.13E+08	2.13E+08	265	2.13E+08	15	7.99781	4.58622
Nc-1-IP_pe	chr1	2.13E+08	2.13E+08	502	2.13E+08	14	6.06519	3.69269
Nc-1-IP_pe	chr1	2.13E+08	2.13E+08	499	2.13E+08	14	6.81736	4.09664
Nc-1-IP_pe	chr1	2.13E+08	2.13E+08	526	2.13E+08	14	5.03085	3.16286
Nc-1-IP_pe	chr1	2.13E+08	2.13E+08	270	2.13E+08	11	5.08426	3.53601
Nc-1-IP_pe	chr1	2.13E+08	2.13E+08	364	2.13E+08	11	5.50915	3.78671
Nc-1-IP_pe	chr1	2.13E+08	2.13E+08	345	2.13E+08	12	5.23082	3.49289
Nc-1-IP_pe	chr1	2.13E+08	2.13E+08	498	2.13E+08	10	3.89064	2.95552
Nc-1-IP_pe	chr1	2.13E+08	2.13E+08	314	2.13E+08	15	5.66913	3.37372
Nc-1-IP_pe	chr1	2.13E+08	2.13E+08	358	2.13E+08	15	7.71696	4.43252
Nc-1-IP_pe	chr1	2.13E+08	2.13E+08	253	2.13E+08	11	4.06403	2.95415
Nc-1-IP_pe	chr1	2.13E+08	2.13E+08	481	2.13E+08	11	4.54404	3.2242
Nc-1-IP_pe	chr1	2.14E+08	2.14E+08	282	2.14E+08	11	5.79772	3.95946
Nc-1-IP_pe	chr1	2.14E+08	2.14E+08	570	2.14E+08	11	5.10577	3.5486
Nc-1-IP_pe	chr1	2.14E+08	2.14E+08	407	2.14E+08	9	4.1022	3.19039
Nc-1-IP_pe	chr1	2.14E+08	2.14E+08	315	2.14E+08	14	5.51541	3.4073
Nc-1-IP_pe	chr1	2.14E+08	2.14E+08	253	2.14E+08	10	3.4604	2.70797
Nc-1-IP_pe	chr1	2.14E+08	2.14E+08	265	2.14E+08	17	10.8684	5.80674
Nc-1-IP_pe	chr1	2.14E+08	2.14E+08	309	2.14E+08	12	6.07041	3.97229
Nc-1-IP_pe	chr1	2.14E+08	2.14E+08	484	2.14E+08	9	3.27326	2.68684
Nc-1-IP_pe	chr1	2.14E+08	2.14E+08	428	2.14E+08	12	6.86906	4.44438
Nc-1-IP_pe	chr1	2.14E+08	2.14E+08	254	2.14E+08	14	8.15847	4.85247
Nc-1-IP_pe	chr1	2.14E+08	2.14E+08	302	2.14E+08	10	4.99888	3.61677
Nc-1-IP_pe	chr1	2.14E+08	2.14E+08	528	2.14E+08	11	3.28475	2.53029
Nc-1-IP_pe	chr1	2.14E+08	2.14E+08	288	2.14E+08	12	5.23082	3.49289
Nc-1-IP_pe	chr1	2.14E+08	2.14E+08	571	2.14E+08	16	7.97877	4.4137
Nc-1-IP_pe	chr1	2.14E+08	2.14E+08	725	2.14E+08	13	5.36853	3.44651

Nc-1-IP_pe	chr1	2.14E+08	2.14E+08	308	2.14E+08	10	4.79071	3.49018
Nc-1-IP_pe	chr1	2.14E+08	2.14E+08	276	2.14E+08	13	6.62436	4.14003
Nc-1-IP_pe	chr1	2.14E+08	2.14E+08	253	2.14E+08	10	4.80655	3.49978
Nc-1-IP_pe	chr1	2.14E+08	2.14E+08	480	2.14E+08	15	3.46204	2.35851
Nc-1-IP_pe	chr1	2.14E+08	2.14E+08	352	2.14E+08	9	3.87	3.04756
Nc-1-IP_pe	chr1	2.14E+08	2.14E+08	547	2.14E+08	17	5.6711	3.17666
Nc-1-IP_pe	chr1	2.14E+08	2.14E+08	367	2.14E+08	10	4.07292	3.06202
Nc-1-IP_pe	chr1	2.14E+08	2.14E+08	285	2.14E+08	14	6.06519	3.69269
Nc-1-IP_pe	chr1	2.14E+08	2.14E+08	628	2.14E+08	17	7.02067	3.79543
Nc-1-IP_pe	chr1	2.14E+08	2.14E+08	455	2.14E+08	11	3.28475	2.53029
Nc-1-IP_pe	chr1	2.14E+08	2.14E+08	596	2.14E+08	15	6.68311	3.88394
Nc-1-IP_pe	chr1	2.14E+08	2.14E+08	453	2.14E+08	17	7.02067	3.79543
Nc-1-IP_pe	chr1	2.14E+08	2.14E+08	274	2.14E+08	10	4.39571	3.25288
Nc-1-IP_pe	chr1	2.14E+08	2.14E+08	640	2.14E+08	13	6.54342	4.09407
Nc-1-IP_pe	chr1	2.14E+08	2.14E+08	381	2.14E+08	12	5.94619	3.9002
Nc-1-IP_pe	chr1	2.14E+08	2.14E+08	427	2.14E+08	10	3.71867	2.85592
Nc-1-IP_pe	chr1	2.14E+08	2.14E+08	330	2.14E+08	10	4.33501	3.21677
Nc-1-IP_pe	chr1	2.14E+08	2.14E+08	511	2.14E+08	10	3.83201	2.92147
Nc-1-IP_pe	chr1	2.14E+08	2.14E+08	257	2.14E+08	14	6.69566	4.03025
Nc-1-IP_pe	chr1	2.15E+08	2.15E+08	304	2.15E+08	18	5.33197	2.94862
Nc-1-IP_pe	chr1	2.15E+08	2.15E+08	296	2.15E+08	18	8.99859	4.62187
Nc-1-IP_pe	chr1	2.15E+08	2.15E+08	791	2.15E+08	25	10.4937	4.3247
Nc-1-IP_pe	chr1	2.15E+08	2.15E+08	323	2.15E+08	9	2.89249	2.46179
Nc-1-IP_pe	chr1	2.15E+08	2.15E+08	325	2.15E+08	14	6.96097	4.17548
Nc-1-IP_pe	chr1	2.15E+08	2.15E+08	268	2.15E+08	15	5.20142	3.14789
Nc-1-IP_pe	chr1	2.15E+08	2.15E+08	401	2.15E+08	18	7.18674	3.75388
Nc-1-IP_pe	chr1	2.15E+08	2.15E+08	2327	2.15E+08	17	4.78101	2.79343
Nc-1-IP_pe	chr1	2.15E+08	2.15E+08	259	2.15E+08	15	9.55381	5.47001
Nc-1-IP_pe	chr1	2.15E+08	2.15E+08	339	2.15E+08	15	9.33061	5.34018
Nc-1-IP_pe	chr1	2.15E+08	2.15E+08	263	2.15E+08	13	6.47103	4.0531
Nc-1-IP_pe	chr1	2.15E+08	2.15E+08	344	2.15E+08	20	4.15754	2.37952
Nc-1-IP_pe	chr1	2.15E+08	2.15E+08	282	2.15E+08	14	8.29543	4.93196
Nc-1-IP_pe	chr1	2.15E+08	2.15E+08	1138	2.15E+08	15	6.78905	3.93887
Nc-1-IP_pe	chr1	2.15E+08	2.15E+08	273	2.15E+08	11	5.10577	3.5486
Nc-1-IP_pe	chr1	2.15E+08	2.15E+08	287	2.15E+08	13	6.62436	4.14003
Nc-1-IP_pe	chr1	2.15E+08	2.15E+08	635	2.15E+08	14	3.86727	2.6027
Nc-1-IP_pe	chr1	2.15E+08	2.15E+08	283	2.15E+08	13	6.62436	4.14003
Nc-1-IP_pe	chr1	2.15E+08	2.15E+08	390	2.15E+08	14	7.9745	4.74633
Nc-1-IP_pe	chr1	2.15E+08	2.15E+08	422	2.15E+08	12	6.86906	4.44438
Nc-1-IP_pe	chr1	2.15E+08	2.15E+08	356	2.15E+08	13	4.41954	2.952
Nc-1-IP_pe	chr1	2.15E+08	2.15E+08	585	2.15E+08	18	7.73136	4.00629
Nc-1-IP_pe	chr1	2.15E+08	2.15E+08	263	2.15E+08	10	4.99888	3.61677
Nc-1-IP_pe	chr1	2.15E+08	2.15E+08	847	2.15E+08	12	5.84943	3.84431
Nc-1-IP_pe	chr1	2.15E+08	2.15E+08	298	2.15E+08	12	5.23082	3.49289
Nc-1-IP_pe	chr1	2.15E+08	2.15E+08	262	2.15E+08	10	3.4604	2.70797

Nc-1-IP_pe	chr1	2.15E+08	2.15E+08	601	2.15E+08	12	4.70082	3.20033
Nc-1-IP_pe	chr1	2.15E+08	2.15E+08	295	2.15E+08	10	3.89064	2.95552
Nc-1-IP_pe	chr1	2.15E+08	2.15E+08	467	2.15E+08	10	4.77495	3.48064
Nc-1-IP_pe	chr1	2.16E+08	2.16E+08	326	2.16E+08	9	4.46568	3.41644
Nc-1-IP_pe	chr1	2.16E+08	2.16E+08	521	2.16E+08	11	5.83542	3.98217
Nc-1-IP_pe	chr1	2.16E+08	2.16E+08	261	2.16E+08	10	4.46067	3.29163
Nc-1-IP_pe	chr1	2.16E+08	2.16E+08	400	2.16E+08	11	5.72344	3.91481
Nc-1-IP_pe	chr1	2.16E+08	2.16E+08	305	2.16E+08	13	5.9487	3.76157
Nc-1-IP_pe	chr1	2.16E+08	2.16E+08	390	2.16E+08	17	5.14803	2.94914
Nc-1-IP_pe	chr1	2.16E+08	2.16E+08	257	2.16E+08	13	5.9487	3.76157
Nc-1-IP_pe	chr1	2.16E+08	2.16E+08	313	2.16E+08	10	3.08907	2.49869
Nc-1-IP_pe	chr1	2.16E+08	2.16E+08	271	2.16E+08	10	5.23357	3.76063
Nc-1-IP_pe	chr1	2.16E+08	2.16E+08	398	2.16E+08	18	11.3299	5.852
Nc-1-IP_pe	chr1	2.16E+08	2.16E+08	529	2.16E+08	12	4.70082	3.20033
Nc-1-IP_pe	chr1	2.16E+08	2.16E+08	492	2.16E+08	10	5.23357	3.76063
Nc-1-IP_pe	chr1	2.16E+08	2.16E+08	278	2.16E+08	13	5.9487	3.76157
Nc-1-IP_pe	chr1	2.17E+08	2.17E+08	277	2.17E+08	12	5.23082	3.49289
Nc-1-IP_pe	chr1	2.17E+08	2.17E+08	452	2.17E+08	14	8.44792	5.0209
Nc-1-IP_pe	chr1	2.17E+08	2.17E+08	372	2.17E+08	12	5.84943	3.84431
Nc-1-IP_pe	chr1	2.17E+08	2.17E+08	280	2.17E+08	8	3.74505	3.07688
Nc-1-IP_pe	chr1	2.17E+08	2.17E+08	728	2.17E+08	11	6.03439	4.10251
Nc-1-IP_pe	chr1	2.17E+08	2.17E+08	904	2.17E+08	12	5.84943	3.84431
Nc-1-IP_pe	chr1	2.17E+08	2.17E+08	351	2.17E+08	10	3.4604	2.70797
Nc-1-IP_pe	chr1	2.17E+08	2.17E+08	257	2.17E+08	9	4.46937	3.41876
Nc-1-IP_pe	chr1	2.17E+08	2.17E+08	260	2.17E+08	11	5.10577	3.5486
Nc-1-IP_pe	chr1	2.17E+08	2.17E+08	257	2.17E+08	10	5.00283	3.61918
Nc-1-IP_pe	chr1	2.17E+08	2.17E+08	278	2.17E+08	17	11.0652	5.91835
Nc-1-IP_pe	chr1	2.17E+08	2.17E+08	827	2.17E+08	15	9.41092	5.38679
Nc-1-IP_pe	chr1	2.17E+08	2.17E+08	326	2.17E+08	11	4.06403	2.95415
Nc-1-IP_pe	chr1	2.17E+08	2.17E+08	692	2.17E+08	16	6.33285	3.58457
Nc-1-IP_pe	chr1	2.17E+08	2.17E+08	271	2.17E+08	11	6.03439	4.10251
Nc-1-IP_pe	chr1	2.18E+08	2.18E+08	543	2.18E+08	39	14.8827	4.36417
Nc-1-IP_pe	chr1	2.18E+08	2.18E+08	266	2.18E+08	14	4.60011	2.95114
Nc-1-IP_pe	chr1	2.18E+08	2.18E+08	256	2.18E+08	13	7.49774	4.64586
Nc-1-IP_pe	chr1	2.18E+08	2.18E+08	438	2.18E+08	10	4.39571	3.25288
Nc-1-IP_pe	chr1	2.18E+08	2.18E+08	430	2.18E+08	11	3.64839	2.72584
Nc-1-IP_pe	chr1	2.18E+08	2.18E+08	317	2.18E+08	13	7.7352	4.78626
Nc-1-IP_pe	chr1	2.18E+08	2.18E+08	257	2.18E+08	12	6.86906	4.44438
Nc-1-IP_pe	chr1	2.18E+08	2.18E+08	489	2.18E+08	10	4.99888	3.61677
Nc-1-IP_pe	chr1	2.18E+08	2.18E+08	266	2.18E+08	13	6.62436	4.14003
Nc-1-IP_pe	chr1	2.18E+08	2.18E+08	665	2.18E+08	17	8.58498	4.57078
Nc-1-IP_pe	chr1	2.18E+08	2.18E+08	1322	2.18E+08	12	5.23082	3.49289
Nc-1-IP_pe	chr1	2.18E+08	2.18E+08	464	2.18E+08	10	4.39571	3.25288
Nc-1-IP_pe	chr1	2.18E+08	2.18E+08	260	2.18E+08	13	6.62436	4.14003
Nc-1-IP_pe	chr1	2.18E+08	2.18E+08	937	2.18E+08	11	3.64839	2.72584

Nc-1-IP_pe	chr1	2.18E+08	2.18E+08	748	2.18E+08	11	3.64839	2.72584
Nc-1-IP_pe	chr1	2.18E+08	2.18E+08	259	2.18E+08	9	4.46937	3.41876
Nc-1-IP_pe	chr1	2.18E+08	2.18E+08	287	2.18E+08	15	5.66913	3.37372
Nc-1-IP_pe	chr1	2.18E+08	2.18E+08	271	2.18E+08	13	7.47517	4.63258
Nc-1-IP_pe	chr1	2.18E+08	2.18E+08	441	2.18E+08	9	2.89249	2.46179
Nc-1-IP_pe	chr1	2.18E+08	2.18E+08	285	2.18E+08	12	6.58417	4.27436
Nc-1-IP_pe	chr1	2.18E+08	2.18E+08	386	2.18E+08	32	12.9078	4.42099
Nc-1-IP_pe	chr1	2.18E+08	2.18E+08	295	2.18E+08	13	5.36853	3.44651
Nc-1-IP_pe	chr1	2.18E+08	2.18E+08	496	2.18E+08	14	3.86727	2.6027
Nc-1-IP_pe	chr1	2.18E+08	2.18E+08	295	2.18E+08	13	5.86991	3.71823
Nc-1-IP_pe	chr1	2.18E+08	2.18E+08	476	2.18E+08	12	5.42384	3.60142
Nc-1-IP_pe	chr1	2.18E+08	2.18E+08	512	2.18E+08	12	5.15877	3.45264
Nc-1-IP_pe	chr1	2.18E+08	2.18E+08	699	2.18E+08	12	5.45448	3.61875
Nc-1-IP_pe	chr1	2.19E+08	2.19E+08	562	2.19E+08	13	5.29187	3.40559
Nc-1-IP_pe	chr1	2.19E+08	2.19E+08	507	2.19E+08	16	7.44163	4.13536
Nc-1-IP_pe	chr1	2.19E+08	2.19E+08	258	2.19E+08	12	6.79895	4.40238
Nc-1-IP_pe	chr1	2.19E+08	2.19E+08	675	2.19E+08	13	7.17043	4.45427
Nc-1-IP_pe	chr1	2.19E+08	2.19E+08	455	2.19E+08	14	5.03085	3.16286
Nc-1-IP_pe	chr1	2.19E+08	2.19E+08	580	2.19E+08	14	4.95172	3.12357
Nc-1-IP_pe	chr1	2.19E+08	2.19E+08	639	2.19E+08	15	3.46204	2.35851
Nc-1-IP_pe	chr1	2.19E+08	2.19E+08	466	2.19E+08	11	3.28475	2.53029
Nc-1-IP_pe	chr1	2.19E+08	2.19E+08	261	2.19E+08	13	5.36853	3.44651
Nc-1-IP_pe	chr1	2.19E+08	2.19E+08	470	2.19E+08	11	3.89191	2.85898
Nc-1-IP_pe	chr1	2.19E+08	2.19E+08	299	2.19E+08	12	6.86906	4.44438
Nc-1-IP_pe	chr1	2.19E+08	2.19E+08	271	2.19E+08	12	6.86906	4.44438
Nc-1-IP_pe	chr1	2.19E+08	2.19E+08	484	2.19E+08	15	5.66913	3.37372
Nc-1-IP_pe	chr1	2.19E+08	2.19E+08	323	2.19E+08	18	8.99859	4.62187
Nc-1-IP_pe	chr1	2.19E+08	2.19E+08	344	2.19E+08	21	15.5913	7.52127
Nc-1-IP_pe	chr1	2.19E+08	2.19E+08	506	2.19E+08	12	4.70082	3.20033
Nc-1-IP_pe	chr1	2.19E+08	2.19E+08	289	2.19E+08	10	3.89064	2.95552
Nc-1-IP_pe	chr1	2.19E+08	2.19E+08	324	2.19E+08	12	4.24064	2.95299
Nc-1-IP_pe	chr1	2.19E+08	2.19E+08	299	2.19E+08	13	3.67377	2.58159
Nc-1-IP_pe	chr1	2.19E+08	2.19E+08	355	2.19E+08	16	6.65665	3.74206
Nc-1-IP_pe	chr1	2.19E+08	2.19E+08	538	2.19E+08	14	4.60011	2.95114
Nc-1-IP_pe	chr1	2.19E+08	2.19E+08	324	2.19E+08	13	5.9487	3.76157
Nc-1-IP_pe	chr1	2.2E+08	2.2E+08	354	2.2E+08	16	9.6698	5.33541
Nc-1-IP_pe	chr1	2.2E+08	2.2E+08	1176	2.2E+08	16	5.82808	3.34463
Nc-1-IP_pe	chr1	2.2E+08	2.2E+08	285	2.2E+08	17	4.78101	2.79343
Nc-1-IP_pe	chr1	2.2E+08	2.2E+08	388	2.2E+08	10	4.20113	3.13749
Nc-1-IP_pe	chr1	2.2E+08	2.2E+08	346	2.2E+08	14	5.03085	3.16286
Nc-1-IP_pe	chr1	2.2E+08	2.2E+08	266	2.2E+08	13	7.7352	4.78626
Nc-1-IP_pe	chr1	2.2E+08	2.2E+08	371	2.2E+08	8	3.0402	2.6319
Nc-1-IP_pe	chr1	2.2E+08	2.2E+08	692	2.2E+08	45	24.1357	6.4569
Nc-1-IP_pe	chr1	2.2E+08	2.2E+08	364	2.2E+08	16	6.51374	3.67221
Nc-1-IP_pe	chr1	2.2E+08	2.2E+08	257	2.2E+08	15	4.06016	2.62146

Nc-1-IP_pe chr1	2.2E+08	2.2E+08	633	2.2E+08	10	4.33501	3.21677
Nc-1-IP_pe chr1	2.2E+08	2.2E+08	570	2.2E+08	17	5.6711	3.17666
Nc-1-IP_pe chr1	2.2E+08	2.2E+08	270	2.2E+08	14	7.42849	4.43574
Nc-1-IP_pe chr1	2.2E+08	2.2E+08	296	2.2E+08	11	4.06403	2.95415
Nc-1-IP_pe chr1	2.2E+08	2.2E+08	277	2.2E+08	18	7.73136	4.00629
Nc-1-IP_pe chr1	2.2E+08	2.2E+08	264	2.2E+08	11	4.06403	2.95415
Nc-1-IP_pe chr1	2.2E+08	2.2E+08	567	2.2E+08	10	4.99888	3.61677
Nc-1-IP_pe chr1	2.2E+08	2.2E+08	323	2.2E+08	14	6.69566	4.03025
Nc-1-IP_pe chr1	2.2E+08	2.2E+08	281	2.2E+08	12	6.86906	4.44438
Nc-1-IP_pe chr1	2.2E+08	2.2E+08	440	2.2E+08	12	5.84943	3.84431
Nc-1-IP_pe chr1	2.2E+08	2.2E+08	260	2.2E+08	19	7.84735	3.93486
Nc-1-IP_pe chr1	2.2E+08	2.2E+08	365	2.2E+08	12	5.75858	3.79206
Nc-1-IP_pe chr1	2.2E+08	2.2E+08	288	2.2E+08	14	3.26761	2.32785
Nc-1-IP_pe chr1	2.2E+08	2.2E+08	266	2.2E+08	15	6.78905	3.93887
Nc-1-IP_pe chr1	2.2E+08	2.2E+08	501	2.2E+08	15	6.78905	3.93887
Nc-1-IP_pe chr1	2.2E+08	2.2E+08	289	2.2E+08	10	3.89064	2.95552
Nc-1-IP_pe chr1	2.2E+08	2.2E+08	276	2.2E+08	11	6.03439	4.10251
Nc-1-IP_pe chr1	2.2E+08	2.2E+08	465	2.2E+08	13	6.62436	4.14003
Nc-1-IP_pe chr1	2.2E+08	2.2E+08	583	2.2E+08	11	6.03439	4.10251
Nc-1-IP_pe chr1	2.2E+08	2.2E+08	467	2.2E+08	9	4.46937	3.41876
Nc-1-IP_pe chr1	2.2E+08	2.2E+08	270	2.2E+08	13	6.36468	3.99316
Nc-1-IP_pe chr1	2.2E+08	2.2E+08	287	2.2E+08	10	3.89064	2.95552
Nc-1-IP_pe chr1	2.2E+08	2.2E+08	257	2.2E+08	12	5.84943	3.84431
Nc-1-IP_pe chr1	2.2E+08	2.2E+08	499	2.2E+08	16	10.0135	5.53039
Nc-1-IP_pe chr1	2.2E+08	2.2E+08	272	2.2E+08	15	4.06016	2.62146
Nc-1-IP_pe chr1	2.2E+08	2.2E+08	532	2.2E+08	15	3.46204	2.35851
Nc-1-IP_pe chr1	2.2E+08	2.2E+08	808	2.2E+08	13	4.86362	3.18015
Nc-1-IP_pe chr1	2.2E+08	2.2E+08	305	2.2E+08	18	6.63038	3.50359
Nc-1-IP_pe chr1	2.2E+08	2.2E+08	390	2.2E+08	15	7.56326	4.34922
Nc-1-IP_pe chr1	2.2E+08	2.2E+08	282	2.2E+08	10	4.07292	3.06202
Nc-1-IP_pe chr1	2.2E+08	2.2E+08	265	2.2E+08	15	4.7819	2.95039
Nc-1-IP_pe chr1	2.2E+08	2.2E+08	407	2.2E+08	18	7.47506	3.88659
Nc-1-IP_pe chr1	2.2E+08	2.2E+08	867	2.2E+08	19	9.03708	4.48779
Nc-1-IP_pe chr1	2.2E+08	2.2E+08	1054	2.2E+08	15	5.83691	3.4562
Nc-1-IP_pe chr1	2.2E+08	2.2E+08	327	2.2E+08	12	5.5322	3.6628
Nc-1-IP_pe chr1	2.21E+08	2.21E+08	256	2.21E+08	20	7.97282	3.87239
Nc-1-IP_pe chr1	2.21E+08	2.21E+08	980	2.21E+08	19	4.5193	2.56258
Nc-1-IP_pe chr1	2.21E+08	2.21E+08	265	2.21E+08	10	3.4604	2.70797
Nc-1-IP_pe chr1	2.21E+08	2.21E+08	500	2.21E+08	21	7.10961	3.41419
Nc-1-IP_pe chr1	2.21E+08	2.21E+08	358	2.21E+08	13	5.29187	3.40559
Nc-1-IP_pe chr1	2.21E+08	2.21E+08	622	2.21E+08	17	8.31233	4.43123
Nc-1-IP_pe chr1	2.21E+08	2.21E+08	359	2.21E+08	19	9.15219	4.54307
Nc-1-IP_pe chr1	2.21E+08	2.21E+08	368	2.21E+08	14	6.78215	4.0774
Nc-1-IP_pe chr1	2.21E+08	2.21E+08	318	2.21E+08	16	7.53857	4.18505
Nc-1-IP_pe chr1	2.21E+08	2.21E+08	255	2.21E+08	11	4.54404	3.2242

Nc-1-IP_pe	chr1	2.21E+08	2.21E+08	387	2.21E+08	15	5.66913	3.37372
Nc-1-IP_pe	chr1	2.21E+08	2.21E+08	609	2.21E+08	25	10.6542	4.38776
Nc-1-IP_pe	chr1	2.21E+08	2.21E+08	347	2.21E+08	14	5.51541	3.4073
Nc-1-IP_pe	chr1	2.21E+08	2.21E+08	256	2.21E+08	13	3.6894	2.58919
Nc-1-IP_pe	chr1	2.21E+08	2.21E+08	291	2.21E+08	14	6.16097	3.74327
Nc-1-IP_pe	chr1	2.21E+08	2.21E+08	389	2.21E+08	20	9.21699	4.428
Nc-1-IP_pe	chr1	2.21E+08	2.21E+08	301	2.21E+08	12	5.03305	3.38277
Nc-1-IP_pe	chr1	2.21E+08	2.21E+08	307	2.21E+08	12	3.83688	2.74114
Nc-1-IP_pe	chr1	2.21E+08	2.21E+08	450	2.21E+08	12	4.1728	2.91706
Nc-1-IP_pe	chr1	2.21E+08	2.21E+08	807	2.21E+08	19	8.19109	4.09114
Nc-1-IP_pe	chr1	2.21E+08	2.21E+08	272	2.21E+08	14	5.51541	3.4073
Nc-1-IP_pe	chr1	2.21E+08	2.21E+08	607	2.21E+08	15	7.14634	4.1263
Nc-1-IP_pe	chr1	2.21E+08	2.21E+08	258	2.21E+08	13	5.6727	3.61051
Nc-1-IP_pe	chr1	2.21E+08	2.21E+08	256	2.21E+08	12	3.83688	2.74114
Nc-1-IP_pe	chr1	2.21E+08	2.21E+08	632	2.21E+08	12	5.51653	3.6539
Nc-1-IP_pe	chr1	2.21E+08	2.21E+08	350	2.21E+08	13	4.02562	2.7544
Nc-1-IP_pe	chr1	2.21E+08	2.21E+08	534	2.21E+08	14	4.73128	3.01506
Nc-1-IP_pe	chr1	2.21E+08	2.21E+08	309	2.21E+08	10	5.19356	3.73603
Nc-1-IP_pe	chr1	2.21E+08	2.21E+08	499	2.21E+08	14	4.21449	2.76599
Nc-1-IP_pe	chr1	2.21E+08	2.21E+08	357	2.21E+08	13	4.86362	3.18015
Nc-1-IP_pe	chr1	2.21E+08	2.21E+08	298	2.21E+08	16	5.82808	3.34463
Nc-1-IP_pe	chr1	2.21E+08	2.21E+08	431	2.21E+08	12	6.10662	3.99337
Nc-1-IP_pe	chr1	2.21E+08	2.21E+08	284	2.21E+08	17	7.6273	4.08876
Nc-1-IP_pe	chr1	2.21E+08	2.21E+08	366	2.21E+08	10	3.08907	2.49869
Nc-1-IP_pe	chr1	2.21E+08	2.21E+08	571	2.21E+08	13	7.47517	4.63258
Nc-1-IP_pe	chr1	2.21E+08	2.21E+08	258	2.21E+08	12	6.86906	4.44438
Nc-1-IP_pe	chr1	2.21E+08	2.21E+08	293	2.21E+08	12	3.16137	2.39719
Nc-1-IP_pe	chr1	2.22E+08	2.22E+08	273	2.22E+08	15	6.78905	3.93887
Nc-1-IP_pe	chr1	2.22E+08	2.22E+08	253	2.22E+08	13	6.36468	3.99316
Nc-1-IP_pe	chr1	2.22E+08	2.22E+08	257	2.22E+08	11	5.10577	3.5486
Nc-1-IP_pe	chr1	2.22E+08	2.22E+08	419	2.22E+08	11	4.87504	3.4143
Nc-1-IP_pe	chr1	2.22E+08	2.22E+08	271	2.22E+08	10	3.60969	2.79325
Nc-1-IP_pe	chr1	2.22E+08	2.22E+08	320	2.22E+08	15	6.78905	3.93887
Nc-1-IP_pe	chr1	2.22E+08	2.22E+08	264	2.22E+08	13	5.9487	3.76157
Nc-1-IP_pe	chr1	2.22E+08	2.22E+08	444	2.22E+08	14	4.73128	3.01506
Nc-1-IP_pe	chr1	2.22E+08	2.22E+08	390	2.22E+08	14	6.28469	3.80898
Nc-1-IP_pe	chr1	2.22E+08	2.22E+08	271	2.22E+08	12	4.70082	3.20033
Nc-1-IP_pe	chr1	2.22E+08	2.22E+08	353	2.22E+08	17	4.44438	2.65333
Nc-1-IP_pe	chr1	2.22E+08	2.22E+08	417	2.22E+08	14	3.00733	2.21111
Nc-1-IP_pe	chr1	2.22E+08	2.22E+08	968	2.22E+08	16	4.25252	2.63824
Nc-1-IP_pe	chr1	2.22E+08	2.22E+08	285	2.22E+08	20	5.34663	2.81336
Nc-1-IP_pe	chr1	2.22E+08	2.22E+08	374	2.22E+08	16	6.33285	3.58457
Nc-1-IP_pe	chr1	2.22E+08	2.22E+08	674	2.22E+08	14	5.51541	3.4073
Nc-1-IP_pe	chr1	2.22E+08	2.22E+08	321	2.22E+08	17	7.02067	3.79543
Nc-1-IP_pe	chr1	2.22E+08	2.22E+08	285	2.22E+08	12	5.01934	3.37518

Nc-1-IP_pe chr1	2.22E+08	2.22E+08	289	2.22E+08	16	6.47169	3.65176
Nc-1-IP_pe chr1	2.22E+08	2.22E+08	312	2.22E+08	22	10.276	4.6231
Nc-1-IP_pe chr1	2.22E+08	2.22E+08	288	2.22E+08	14	6.69566	4.03025
Nc-1-IP_pe chr1	2.22E+08	2.22E+08	1192	2.22E+08	18	9.50408	4.87823
Nc-1-IP_pe chr1	2.22E+08	2.22E+08	1119	2.22E+08	19	8.4638	4.21715
Nc-1-IP_pe chr1	2.22E+08	2.22E+08	259	2.22E+08	17	6.64406	3.61808
Nc-1-IP_pe chr1	2.22E+08	2.22E+08	329	2.22E+08	16	4.68897	2.82769
Nc-1-IP_pe chr1	2.22E+08	2.22E+08	261	2.22E+08	17	8.02964	4.28847
Nc-1-IP_pe chr1	2.22E+08	2.22E+08	762	2.22E+08	18	7.62523	3.95652
Nc-1-IP_pe chr1	2.22E+08	2.22E+08	290	2.22E+08	16	7.27441	4.05022
Nc-1-IP_pe chr1	2.22E+08	2.22E+08	354	2.22E+08	17	5.14803	2.94914
Nc-1-IP_pe chr1	2.22E+08	2.22E+08	373	2.22E+08	10	3.59902	2.78714
Nc-1-IP_pe chr1	2.22E+08	2.22E+08	300	2.22E+08	16	3.94146	2.50592
Nc-1-IP_pe chr1	2.22E+08	2.22E+08	264	2.22E+08	13	6.41752	4.02291
Nc-1-IP_pe chr1	2.22E+08	2.22E+08	265	2.22E+08	17	5.89608	3.2766
Nc-1-IP_pe chr1	2.22E+08	2.22E+08	482	2.22E+08	15	4.7819	2.95039
Nc-1-IP_pe chr1	2.22E+08	2.22E+08	298	2.22E+08	15	5.47388	3.27871
Nc-1-IP_pe chr1	2.22E+08	2.22E+08	258	2.22E+08	15	5.20142	3.14789
Nc-1-IP_pe chr1	2.22E+08	2.22E+08	295	2.22E+08	19	4.35302	2.50016
Nc-1-IP_pe chr1	2.22E+08	2.22E+08	1140	2.22E+08	20	6.17846	3.13279
Nc-1-IP_pe chr1	2.22E+08	2.22E+08	573	2.22E+08	26	7.95585	3.31498
Nc-1-IP_pe chr1	2.22E+08	2.22E+08	375	2.22E+08	21	7.72183	3.65936
Nc-1-IP_pe chr1	2.22E+08	2.22E+08	541	2.22E+08	17	7.19962	3.88098
Nc-1-IP_pe chr1	2.22E+08	2.22E+08	253	2.22E+08	20	9.45052	4.53626
Nc-1-IP_pe chr1	2.22E+08	2.22E+08	446	2.22E+08	14	5.7679	3.53731
Nc-1-IP_pe chr1	2.22E+08	2.22E+08	261	2.22E+08	13	4.34711	2.91533
Nc-1-IP_pe chr1	2.23E+08	2.23E+08	501	2.23E+08	20	8.63402	4.16324
Nc-1-IP_pe chr1	2.23E+08	2.23E+08	275	2.23E+08	26	9.34034	3.78992
Nc-1-IP_pe chr1	2.23E+08	2.23E+08	297	2.23E+08	11	4.06403	2.95415
Nc-1-IP_pe chr1	2.23E+08	2.23E+08	431	2.23E+08	17	5.54967	3.12324
Nc-1-IP_pe chr1	2.23E+08	2.23E+08	430	2.23E+08	18	4.96968	2.80074
Nc-1-IP_pe chr1	2.23E+08	2.23E+08	503	2.23E+08	11	4.24272	3.05389
Nc-1-IP_pe chr1	2.23E+08	2.23E+08	281	2.23E+08	15	5.66913	3.37372
Nc-1-IP_pe chr1	2.23E+08	2.23E+08	592	2.23E+08	12	4.75569	3.23024
Nc-1-IP_pe chr1	2.23E+08	2.23E+08	424	2.23E+08	15	5.96953	3.52194
Nc-1-IP_pe chr1	2.23E+08	2.23E+08	599	2.23E+08	20	4.42487	2.47493
Nc-1-IP_pe chr1	2.23E+08	2.23E+08	272	2.23E+08	18	8.37884	4.31591
Nc-1-IP_pe chr1	2.23E+08	2.23E+08	290	2.23E+08	12	5.23082	3.49289
Nc-1-IP_pe chr1	2.23E+08	2.23E+08	278	2.23E+08	13	7.42513	4.60316
Nc-1-IP_pe chr1	2.23E+08	2.23E+08	498	2.23E+08	13	5.9487	3.76157
Nc-1-IP_pe chr1	2.23E+08	2.23E+08	310	2.23E+08	13	5.36853	3.44651
Nc-1-IP_pe chr1	2.23E+08	2.23E+08	264	2.23E+08	23	7.01947	3.21526
Nc-1-IP_pe chr1	2.23E+08	2.23E+08	389	2.23E+08	19	9.22155	4.57653
Nc-1-IP_pe chr1	2.23E+08	2.23E+08	289	2.23E+08	12	5.84943	3.84431
Nc-1-IP_pe chr1	2.23E+08	2.23E+08	253	2.23E+08	15	7.46994	4.29894

Nc-1-IP_pe	chr1	2.23E+08	2.23E+08	274	2.23E+08	14	3.86727	2.6027
Nc-1-IP_pe	chr1	2.23E+08	2.23E+08	617	2.23E+08	11	3.78682	2.80131
Nc-1-IP_pe	chr1	2.23E+08	2.23E+08	355	2.23E+08	13	4.41954	2.952
Nc-1-IP_pe	chr1	2.23E+08	2.23E+08	357	2.23E+08	14	6.06519	3.69269
Nc-1-IP_pe	chr1	2.23E+08	2.23E+08	441	2.23E+08	17	7.6273	4.08876
Nc-1-IP_pe	chr1	2.23E+08	2.23E+08	718	2.23E+08	24	6.44146	2.94634
Nc-1-IP_pe	chr1	2.23E+08	2.23E+08	554	2.23E+08	14	3.86727	2.6027
Nc-1-IP_pe	chr1	2.23E+08	2.23E+08	307	2.23E+08	16	8.39046	4.63193
Nc-1-IP_pe	chr1	2.23E+08	2.23E+08	263	2.23E+08	14	6.50995	3.92971
Nc-1-IP_pe	chr1	2.23E+08	2.23E+08	669	2.23E+08	17	6.47874	3.54137
Nc-1-IP_pe	chr1	2.23E+08	2.23E+08	372	2.23E+08	15	7.19874	4.15407
Nc-1-IP_pe	chr1	2.23E+08	2.23E+08	496	2.23E+08	18	6.65364	3.51391
Nc-1-IP_pe	chr1	2.23E+08	2.23E+08	387	2.23E+08	19	8.33471	4.15728
Nc-1-IP_pe	chr1	2.23E+08	2.23E+08	467	2.23E+08	16	4.96462	2.94973
Nc-1-IP_pe	chr1	2.23E+08	2.23E+08	898	2.23E+08	20	7.85753	3.8227
Nc-1-IP_pe	chr1	2.23E+08	2.23E+08	401	2.23E+08	22	10.6487	4.78948
Nc-1-IP_pe	chr1	2.23E+08	2.23E+08	302	2.23E+08	16	2.92499	2.08721
Nc-1-IP_pe	chr1	2.23E+08	2.23E+08	656	2.23E+08	17	5.9911	3.31919
Nc-1-IP_pe	chr1	2.23E+08	2.23E+08	396	2.23E+08	14	5.27082	3.28308
Nc-1-IP_pe	chr1	2.23E+08	2.23E+08	427	2.23E+08	14	5.06689	3.18081
Nc-1-IP_pe	chr1	2.23E+08	2.23E+08	483	2.23E+08	15	6.02372	3.54894
Nc-1-IP_pe	chr1	2.23E+08	2.23E+08	438	2.23E+08	14	5.00888	3.15193
Nc-1-IP_pe	chr1	2.23E+08	2.23E+08	506	2.23E+08	12	4.04688	2.85073
Nc-1-IP_pe	chr1	2.23E+08	2.23E+08	261	2.23E+08	17	7.02067	3.79543
Nc-1-IP_pe	chr1	2.23E+08	2.23E+08	253	2.23E+08	13	3.07238	2.29378
Nc-1-IP_pe	chr1	2.23E+08	2.23E+08	1137	2.23E+08	20	8.56319	4.1316
Nc-1-IP_pe	chr1	2.23E+08	2.23E+08	519	2.23E+08	18	4.87655	2.76319
Nc-1-IP_pe	chr1	2.23E+08	2.23E+08	641	2.23E+08	17	6.01283	3.32896
Nc-1-IP_pe	chr1	2.23E+08	2.23E+08	338	2.23E+08	16	3.65575	2.38624
Nc-1-IP_pe	chr1	2.23E+08	2.23E+08	420	2.23E+08	14	5.14959	3.22214
Nc-1-IP_pe	chr1	2.23E+08	2.23E+08	512	2.23E+08	17	7.15465	3.8594
Nc-1-IP_pe	chr1	2.23E+08	2.23E+08	425	2.23E+08	12	3.33349	2.48364
Nc-1-IP_pe	chr1	2.23E+08	2.23E+08	515	2.23E+08	21	8.10596	3.8173
Nc-1-IP_pe	chr1	2.23E+08	2.23E+08	435	2.23E+08	20	3.05104	1.99497
Nc-1-IP_pe	chr1	2.23E+08	2.23E+08	554	2.23E+08	14	5.33286	3.31443
Nc-1-IP_pe	chr1	2.23E+08	2.23E+08	545	2.23E+08	22	7.7408	3.56938
Nc-1-IP_pe	chr1	2.23E+08	2.23E+08	304	2.23E+08	15	4.7819	2.95039
Nc-1-IP_pe	chr1	2.23E+08	2.23E+08	287	2.23E+08	18	4.96968	2.80074
Nc-1-IP_pe	chr1	2.23E+08	2.23E+08	400	2.23E+08	14	4.8399	3.06836
Nc-1-IP_pe	chr1	2.23E+08	2.23E+08	323	2.23E+08	22	10.0534	4.52509
Nc-1-IP_pe	chr1	2.23E+08	2.23E+08	254	2.23E+08	15	6.10627	3.59023
Nc-1-IP_pe	chr1	2.23E+08	2.23E+08	278	2.23E+08	13	5.22525	3.37017
Nc-1-IP_pe	chr1	2.23E+08	2.23E+08	684	2.23E+08	19	7.05262	3.58428
Nc-1-IP_pe	chr1	2.23E+08	2.23E+08	298	2.23E+08	14	4.8399	3.06836
Nc-1-IP_pe	chr1	2.23E+08	2.23E+08	630	2.23E+08	14	3.55308	2.45762

Nc-1-IP_pe	chr1	2.23E+08	2.23E+08	292	2.23E+08	14	5.40858	3.35284
Nc-1-IP_pe	chr1	2.23E+08	2.23E+08	576	2.23E+08	17	7.02067	3.79543
Nc-1-IP_pe	chr1	2.23E+08	2.23E+08	801	2.23E+08	18	8.46177	4.35631
Nc-1-IP_pe	chr1	2.23E+08	2.23E+08	406	2.23E+08	20	9.21699	4.428
Nc-1-IP_pe	chr1	2.23E+08	2.23E+08	277	2.23E+08	15	4.75011	2.93562
Nc-1-IP_pe	chr1	2.23E+08	2.23E+08	580	2.23E+08	17	6.47874	3.54137
Nc-1-IP_pe	chr1	2.23E+08	2.23E+08	295	2.23E+08	16	4.59224	2.7853
Nc-1-IP_pe	chr1	2.23E+08	2.23E+08	468	2.23E+08	18	6.63038	3.50359
Nc-1-IP_pe	chr1	2.23E+08	2.23E+08	343	2.23E+08	17	5.4568	3.08264
Nc-1-IP_pe	chr1	2.23E+08	2.23E+08	392	2.23E+08	16	6.59897	3.7138
Nc-1-IP_pe	chr1	2.23E+08	2.23E+08	412	2.23E+08	18	6.91333	3.62993
Nc-1-IP_pe	chr1	2.23E+08	2.23E+08	258	2.23E+08	17	5.14803	2.94914
Nc-1-IP_pe	chr1	2.23E+08	2.23E+08	1552	2.23E+08	21	8.6741	4.05679
Nc-1-IP_pe	chr1	2.23E+08	2.23E+08	772	2.23E+08	16	5.37454	3.13479
Nc-1-IP_pe	chr1	2.23E+08	2.23E+08	663	2.23E+08	15	5.00915	3.05678
Nc-1-IP_pe	chr1	2.23E+08	2.23E+08	313	2.23E+08	18	3.52899	2.23922
Nc-1-IP_pe	chr1	2.23E+08	2.23E+08	318	2.23E+08	23	6.50461	3.03295
Nc-1-IP_pe	chr1	2.23E+08	2.23E+08	408	2.23E+08	18	8.39148	4.32206
Nc-1-IP_pe	chr1	2.23E+08	2.23E+08	767	2.23E+08	21	10.6316	4.93656
Nc-1-IP_pe	chr1	2.23E+08	2.23E+08	335	2.23E+08	19	10.8111	5.37367
Nc-1-IP_pe	chr1	2.23E+08	2.23E+08	394	2.23E+08	22	13.4841	6.15087
Nc-1-IP_pe	chr1	2.23E+08	2.23E+08	595	2.23E+08	21	5.5349	2.81884
Nc-1-IP_pe	chr1	2.23E+08	2.23E+08	312	2.23E+08	16	5.37454	3.13479
Nc-1-IP_pe	chr1	2.23E+08	2.23E+08	320	2.23E+08	14	5.98179	3.64885
Nc-1-IP_pe	chr1	2.23E+08	2.23E+08	431	2.23E+08	17	5.9911	3.31919
Nc-1-IP_pe	chr1	2.23E+08	2.23E+08	383	2.23E+08	17	6.13258	3.38303
Nc-1-IP_pe	chr1	2.23E+08	2.23E+08	636	2.23E+08	18	5.33197	2.94862
Nc-1-IP_pe	chr1	2.24E+08	2.24E+08	660	2.24E+08	16	3.94146	2.50592
Nc-1-IP_pe	chr1	2.24E+08	2.24E+08	317	2.24E+08	19	7.29128	3.68799
Nc-1-IP_pe	chr1	2.24E+08	2.24E+08	264	2.24E+08	17	6.47874	3.54137
Nc-1-IP_pe	chr1	2.24E+08	2.24E+08	373	2.24E+08	13	3.07238	2.29378
Nc-1-IP_pe	chr1	2.24E+08	2.24E+08	362	2.24E+08	15	6.21877	3.6468
Nc-1-IP_pe	chr1	2.24E+08	2.24E+08	598	2.24E+08	15	6.21877	3.6468
Nc-1-IP_pe	chr1	2.24E+08	2.24E+08	374	2.24E+08	12	4.29266	2.98064
Nc-1-IP_pe	chr1	2.24E+08	2.24E+08	261	2.24E+08	14	4.60011	2.95114
Nc-1-IP_pe	chr1	2.24E+08	2.24E+08	282	2.24E+08	11	2.96397	2.36091
Nc-1-IP_pe	chr1	2.24E+08	2.24E+08	483	2.24E+08	15	5.20142	3.14789
Nc-1-IP_pe	chr1	2.24E+08	2.24E+08	506	2.24E+08	20	5.70098	2.94771
Nc-1-IP_pe	chr1	2.24E+08	2.24E+08	934	2.24E+08	17	4.78101	2.79343
Nc-1-IP_pe	chr1	2.24E+08	2.24E+08	487	2.24E+08	14	5.00888	3.15193
Nc-1-IP_pe	chr1	2.24E+08	2.24E+08	331	2.24E+08	13	4.14549	2.81406
Nc-1-IP_pe	chr1	2.24E+08	2.24E+08	652	2.24E+08	17	6.60377	3.59932
Nc-1-IP_pe	chr1	2.24E+08	2.24E+08	338	2.24E+08	16	6.07073	3.45913
Nc-1-IP_pe	chr1	2.24E+08	2.24E+08	277	2.24E+08	17	4.13464	2.52661
Nc-1-IP_pe	chr1	2.24E+08	2.24E+08	857	2.24E+08	14	3.26761	2.32785

Nc-1-IP_pe	chr1	2.24E+08	2.24E+08	434	2.24E+08	21	9.1909	4.28082
Nc-1-IP_pe	chr1	2.24E+08	2.24E+08	437	2.24E+08	22	6.84408	3.22845
Nc-1-IP_pe	chr1	2.24E+08	2.24E+08	298	2.24E+08	20	9.57894	4.59631
Nc-1-IP_pe	chr1	2.24E+08	2.24E+08	327	2.24E+08	18	6.15736	3.29676
Nc-1-IP_pe	chr1	2.24E+08	2.24E+08	287	2.24E+08	15	4.7819	2.95039
Nc-1-IP_pe	chr1	2.24E+08	2.24E+08	261	2.24E+08	17	3.58449	2.30632
Nc-1-IP_pe	chr1	2.24E+08	2.24E+08	1073	2.24E+08	22	6.44373	3.08129
Nc-1-IP_pe	chr1	2.24E+08	2.24E+08	319	2.24E+08	15	2.9484	2.13875
Nc-1-IP_pe	chr1	2.24E+08	2.24E+08	437	2.24E+08	16	5.14926	3.03255
Nc-1-IP_pe	chr1	2.24E+08	2.24E+08	257	2.24E+08	19	7.2357	3.66372
Nc-1-IP_pe	chr1	2.24E+08	2.24E+08	753	2.24E+08	26	6.15464	2.74223
Nc-1-IP_pe	chr1	2.24E+08	2.24E+08	258	2.24E+08	15	4.06016	2.62146
Nc-1-IP_pe	chr1	2.24E+08	2.24E+08	564	2.24E+08	22	6.4249	3.07445
Nc-1-IP_pe	chr1	2.24E+08	2.24E+08	317	2.24E+08	23	7.89844	3.53777
Nc-1-IP_pe	chr1	2.24E+08	2.24E+08	321	2.24E+08	21	5.5349	2.81884
Nc-1-IP_pe	chr1	2.24E+08	2.24E+08	577	2.24E+08	23	6.99955	3.20812
Nc-1-IP_pe	chr1	2.24E+08	2.24E+08	626	2.24E+08	18	7.27161	3.79273
Nc-1-IP_pe	chr1	2.24E+08	2.24E+08	492	2.24E+08	26	8.38703	3.45949
Nc-1-IP_pe	chr1	2.24E+08	2.24E+08	403	2.24E+08	17	6.47874	3.54137
Nc-1-IP_pe	chr1	2.24E+08	2.24E+08	297	2.24E+08	20	6.94658	3.44067
Nc-1-IP_pe	chr1	2.24E+08	2.24E+08	475	2.24E+08	20	6.94658	3.44067
Nc-1-IP_pe	chr1	2.24E+08	2.24E+08	529	2.24E+08	16	5.56281	3.22124
Nc-1-IP_pe	chr1	2.24E+08	2.24E+08	405	2.24E+08	18	6.40661	3.40507
Nc-1-IP_pe	chr1	2.24E+08	2.24E+08	469	2.24E+08	25	5.1059	2.46996
Nc-1-IP_pe	chr1	2.24E+08	2.24E+08	456	2.24E+08	18	4.96379	2.79835
Nc-1-IP_pe	chr1	2.24E+08	2.24E+08	1409	2.24E+08	22	7.7403	3.56918
Nc-1-IP_pe	chr1	2.24E+08	2.24E+08	298	2.24E+08	15	4.14926	2.66135
Nc-1-IP_pe	chr1	2.24E+08	2.24E+08	514	2.24E+08	15	3.74763	2.48304
Nc-1-IP_pe	chr1	2.24E+08	2.24E+08	272	2.24E+08	13	4.50646	2.99621
Nc-1-IP_pe	chr1	2.24E+08	2.24E+08	255	2.24E+08	18	6.63038	3.50359
Nc-1-IP_pe	chr1	2.24E+08	2.24E+08	539	2.24E+08	15	5.66913	3.37372
Nc-1-IP_pe	chr1	2.24E+08	2.24E+08	956	2.24E+08	13	4.39942	2.9418
Nc-1-IP_pe	chr1	2.24E+08	2.24E+08	273	2.24E+08	10	3.03453	2.46828
Nc-1-IP_pe	chr1	2.24E+08	2.24E+08	522	2.24E+08	26	9.86937	3.97999
Nc-1-IP_pe	chr1	2.24E+08	2.24E+08	681	2.24E+08	29	11.3772	4.21102
Nc-1-IP_pe	chr1	2.24E+08	2.24E+08	304	2.24E+08	19	5.51001	2.94564
Nc-1-IP_pe	chr1	2.24E+08	2.24E+08	818	2.24E+08	14	5.03085	3.16286
Nc-1-IP_pe	chr1	2.24E+08	2.24E+08	368	2.24E+08	14	5.32037	3.30811
Nc-1-IP_pe	chr1	2.24E+08	2.24E+08	256	2.24E+08	23	8.92223	3.93219
Nc-1-IP_pe	chr1	2.24E+08	2.24E+08	277	2.24E+08	17	5.54967	3.12324
Nc-1-IP_pe	chr1	2.24E+08	2.24E+08	516	2.24E+08	15	5.34394	3.21607
Nc-1-IP_pe	chr1	2.24E+08	2.24E+08	487	2.24E+08	21	10.3724	4.81519
Nc-1-IP_pe	chr1	2.24E+08	2.24E+08	398	2.24E+08	18	5.33197	2.94862
Nc-1-IP_pe	chr1	2.24E+08	2.24E+08	482	2.24E+08	22	7.27519	3.39036
Nc-1-IP_pe	chr1	2.24E+08	2.24E+08	638	2.24E+08	25	5.37675	2.55246

Nc-1-IP_pe	chr1	2.24E+08	2.24E+08	483	2.24E+08	10	3.94999	2.99009
Nc-1-IP_pe	chr1	2.24E+08	2.24E+08	291	2.24E+08	15	7.46994	4.29894
Nc-1-IP_pe	chr1	2.24E+08	2.24E+08	497	2.24E+08	15	5.66913	3.37372
Nc-1-IP_pe	chr1	2.24E+08	2.24E+08	266	2.24E+08	13	4.67336	3.0817
Nc-1-IP_pe	chr1	2.24E+08	2.24E+08	268	2.24E+08	16	8.79222	4.84884
Nc-1-IP_pe	chr1	2.24E+08	2.24E+08	297	2.24E+08	17	8.88483	4.72635
Nc-1-IP_pe	chr1	2.24E+08	2.24E+08	891	2.24E+08	26	10.439	4.19014
Nc-1-IP_pe	chr1	2.24E+08	2.24E+08	291	2.24E+08	12	5.62759	3.7171
Nc-1-IP_pe	chr1	2.24E+08	2.24E+08	275	2.24E+08	13	6.39984	4.01295
Nc-1-IP_pe	chr1	2.24E+08	2.24E+08	314	2.24E+08	16	7.27441	4.05022
Nc-1-IP_pe	chr1	2.24E+08	2.24E+08	283	2.24E+08	12	5.21606	3.48463
Nc-1-IP_pe	chr1	2.24E+08	2.24E+08	306	2.24E+08	17	5.9911	3.31919
Nc-1-IP_pe	chr1	2.24E+08	2.24E+08	299	2.24E+08	18	9.1091	4.6774
Nc-1-IP_pe	chr1	2.24E+08	2.24E+08	301	2.24E+08	19	8.17534	4.08393
Nc-1-IP_pe	chr1	2.24E+08	2.24E+08	312	2.24E+08	20	6.49719	3.259
Nc-1-IP_pe	chr1	2.24E+08	2.24E+08	330	2.24E+08	20	4.15754	2.37952
Nc-1-IP_pe	chr1	2.24E+08	2.24E+08	1078	2.24E+08	13	4.65065	3.07002
Nc-1-IP_pe	chr1	2.24E+08	2.24E+08	430	2.24E+08	15	4.70031	2.91253
Nc-1-IP_pe	chr1	2.24E+08	2.24E+08	310	2.24E+08	14	4.36469	2.83763
Nc-1-IP_pe	chr1	2.24E+08	2.24E+08	293	2.24E+08	20	7.73292	3.76934
Nc-1-IP_pe	chr1	2.24E+08	2.24E+08	564	2.24E+08	16	6.22699	3.53369
Nc-1-IP_pe	chr1	2.24E+08	2.24E+08	526	2.24E+08	12	4.51041	3.09723
Nc-1-IP_pe	chr1	2.24E+08	2.24E+08	320	2.24E+08	12	4.75569	3.23024
Nc-1-IP_pe	chr1	2.24E+08	2.24E+08	302	2.24E+08	17	5.54967	3.12324
Nc-1-IP_pe	chr1	2.24E+08	2.24E+08	276	2.24E+08	18	8.37884	4.31591
Nc-1-IP_pe	chr1	2.24E+08	2.24E+08	452	2.24E+08	12	4.24064	2.95299
Nc-1-IP_pe	chr1	2.24E+08	2.24E+08	366	2.24E+08	15	5.20142	3.14789
Nc-1-IP_pe	chr1	2.24E+08	2.24E+08	635	2.24E+08	20	6.08336	3.09555
Nc-1-IP_pe	chr1	2.24E+08	2.24E+08	304	2.24E+08	22	8.79433	3.99081
Nc-1-IP_pe	chr1	2.24E+08	2.24E+08	314	2.24E+08	21	4.34782	2.40029
Nc-1-IP_pe	chr1	2.24E+08	2.24E+08	588	2.24E+08	19	6.78661	3.47027
Nc-1-IP_pe	chr1	2.24E+08	2.24E+08	302	2.24E+08	15	4.40338	2.77622
Nc-1-IP_pe	chr1	2.24E+08	2.24E+08	369	2.24E+08	16	4.59224	2.7853
Nc-1-IP_pe	chr1	2.24E+08	2.24E+08	462	2.24E+08	17	4.78101	2.79343
Nc-1-IP_pe	chr1	2.24E+08	2.24E+08	444	2.24E+08	11	4.06403	2.95415
Nc-1-IP_pe	chr1	2.24E+08	2.24E+08	519	2.24E+08	13	4.70769	3.09938
Nc-1-IP_pe	chr1	2.24E+08	2.24E+08	476	2.24E+08	21	13.8282	6.5494
Nc-1-IP_pe	chr1	2.24E+08	2.24E+08	273	2.24E+08	14	6.14575	3.73522
Nc-1-IP_pe	chr1	2.24E+08	2.24E+08	506	2.24E+08	16	4.96462	2.94973
Nc-1-IP_pe	chr1	2.25E+08	2.25E+08	458	2.25E+08	18	6.28754	3.35314
Nc-1-IP_pe	chr1	2.25E+08	2.25E+08	263	2.25E+08	16	4.96462	2.94973
Nc-1-IP_pe	chr1	2.25E+08	2.25E+08	385	2.25E+08	18	5.83846	3.16035
Nc-1-IP_pe	chr1	2.25E+08	2.25E+08	332	2.25E+08	20	7.97282	3.87239
Nc-1-IP_pe	chr1	2.25E+08	2.25E+08	561	2.25E+08	15	6.05107	3.5626
Nc-1-IP_pe	chr1	2.25E+08	2.25E+08	476	2.25E+08	13	3.35769	2.42919

Nc-1-IP_pe	chr1	2.25E+08	2.25E+08	326	2.25E+08	20	8.14433	3.94687
Nc-1-IP_pe	chr1	2.25E+08	2.25E+08	339	2.25E+08	12	4.04688	2.85073
Nc-1-IP_pe	chr1	2.25E+08	2.25E+08	641	2.25E+08	19	4.8268	2.67939
Nc-1-IP_pe	chr1	2.25E+08	2.25E+08	256	2.25E+08	13	6.02837	3.80557
Nc-1-IP_pe	chr1	2.25E+08	2.25E+08	324	2.25E+08	17	5.14803	2.94914
Nc-1-IP_pe	chr1	2.25E+08	2.25E+08	393	2.25E+08	18	8.62313	4.4354
Nc-1-IP_pe	chr1	2.25E+08	2.25E+08	552	2.25E+08	25	7.64976	3.2857
Nc-1-IP_pe	chr1	2.25E+08	2.25E+08	266	2.25E+08	21	6.88144	3.32483
Nc-1-IP_pe	chr1	2.25E+08	2.25E+08	633	2.25E+08	20	9.45052	4.53626
Nc-1-IP_pe	chr1	2.25E+08	2.25E+08	396	2.25E+08	19	7.84735	3.93486
Nc-1-IP_pe	chr1	2.25E+08	2.25E+08	363	2.25E+08	18	6.15736	3.29676
Nc-1-IP_pe	chr1	2.25E+08	2.25E+08	540	2.25E+08	21	7.10961	3.41419
Nc-1-IP_pe	chr1	2.25E+08	2.25E+08	545	2.25E+08	14	4.34537	2.82838
Nc-1-IP_pe	chr1	2.25E+08	2.25E+08	465	2.25E+08	14	4.24097	2.77858
Nc-1-IP_pe	chr1	2.25E+08	2.25E+08	523	2.25E+08	16	3.1497	2.1782
Nc-1-IP_pe	chr1	2.25E+08	2.25E+08	309	2.25E+08	16	4.25252	2.63824
Nc-1-IP_pe	chr1	2.25E+08	2.25E+08	653	2.25E+08	18	5.33197	2.94862
Nc-1-IP_pe	chr1	2.25E+08	2.25E+08	256	2.25E+08	14	4.21449	2.76599
Nc-1-IP_pe	chr1	2.25E+08	2.25E+08	340	2.25E+08	22	8.79433	3.99081
Nc-1-IP_pe	chr1	2.25E+08	2.25E+08	253	2.25E+08	17	5.9911	3.31919
Nc-1-IP_pe	chr1	2.25E+08	2.25E+08	496	2.25E+08	13	4.92076	3.20992
Nc-1-IP_pe	chr1	2.25E+08	2.25E+08	613	2.25E+08	15	6.05107	3.5626
Nc-1-IP_pe	chr1	2.25E+08	2.25E+08	936	2.25E+08	20	6.38846	3.2157
Nc-1-IP_pe	chr1	2.25E+08	2.25E+08	509	2.25E+08	21	4.90204	2.59278
Nc-1-IP_pe	chr1	2.25E+08	2.25E+08	380	2.25E+08	12	3.47963	2.55766
Nc-1-IP_pe	chr1	2.25E+08	2.25E+08	271	2.25E+08	13	4.02562	2.7544
Nc-1-IP_pe	chr1	2.25E+08	2.25E+08	343	2.25E+08	19	8.35089	4.16476
Nc-1-IP_pe	chr1	2.25E+08	2.25E+08	286	2.25E+08	19	4.8268	2.67939
Nc-1-IP_pe	chr1	2.25E+08	2.25E+08	318	2.25E+08	14	4.60011	2.95114
Nc-1-IP_pe	chr1	2.25E+08	2.25E+08	432	2.25E+08	13	5.57038	3.55505
Nc-1-IP_pe	chr1	2.25E+08	2.25E+08	420	2.25E+08	14	5.03085	3.16286
Nc-1-IP_pe	chr1	2.25E+08	2.25E+08	345	2.25E+08	19	6.32622	3.27682
Nc-1-IP_pe	chr1	2.25E+08	2.25E+08	576	2.25E+08	14	6.06519	3.69269
Nc-1-IP_pe	chr1	2.25E+08	2.25E+08	284	2.25E+08	16	6.33285	3.58457
Nc-1-IP_pe	chr1	2.25E+08	2.25E+08	580	2.25E+08	15	6.5434	3.81197
Nc-1-IP_pe	chr1	2.25E+08	2.25E+08	415	2.25E+08	17	7.50977	4.03119
Nc-1-IP_pe	chr1	2.25E+08	2.25E+08	564	2.25E+08	21	7.58657	3.60451
Nc-1-IP_pe	chr1	2.25E+08	2.25E+08	304	2.25E+08	10	3.89064	2.95552
Nc-1-IP_pe	chr1	2.25E+08	2.25E+08	333	2.25E+08	11	5.44042	3.74585
Nc-1-IP_pe	chr1	2.25E+08	2.25E+08	258	2.25E+08	14	5.03085	3.16286
Nc-1-IP_pe	chr1	2.25E+08	2.25E+08	544	2.25E+08	16	7.53857	4.18505
Nc-1-IP_pe	chr1	2.25E+08	2.25E+08	267	2.25E+08	14	3.87746	2.60745
Nc-1-IP_pe	chr1	2.25E+08	2.25E+08	562	2.25E+08	18	5.33197	2.94862
Nc-1-IP_pe	chr1	2.25E+08	2.25E+08	1188	2.25E+08	19	9.41173	4.66885
Nc-1-IP_pe	chr1	2.25E+08	2.25E+08	490	2.25E+08	21	6.66991	3.24296

Nc-1-IP_pe	chr1	2.25E+08	2.25E+08	461	2.25E+08	16	8.22985	4.5463
Nc-1-IP_pe	chr1	2.25E+08	2.25E+08	772	2.25E+08	15	5.20142	3.14789
Nc-1-IP_pe	chr1	2.25E+08	2.25E+08	335	2.25E+08	15	6.78905	3.93887
Nc-1-IP_pe	chr1	2.25E+08	2.25E+08	281	2.25E+08	13	3.35769	2.42919
Nc-1-IP_pe	chr1	2.25E+08	2.25E+08	685	2.25E+08	22	8.38838	3.82569
Nc-1-IP_pe	chr1	2.25E+08	2.25E+08	921	2.25E+08	13	4.02562	2.7544
Nc-1-IP_pe	chr1	2.25E+08	2.25E+08	440	2.25E+08	13	5.73236	3.64298
Nc-1-IP_pe	chr1	2.25E+08	2.25E+08	419	2.25E+08	13	4.02562	2.7544
Nc-1-IP_pe	chr1	2.25E+08	2.25E+08	271	2.25E+08	12	4.70082	3.20033
Nc-1-IP_pe	chr1	2.25E+08	2.25E+08	599	2.25E+08	18	10.9042	5.61861
Nc-1-IP_pe	chr1	2.25E+08	2.25E+08	290	2.25E+08	16	7.61454	4.22416
Nc-1-IP_pe	chr1	2.25E+08	2.25E+08	655	2.25E+08	18	9.1091	4.6774
Nc-1-IP_pe	chr1	2.25E+08	2.25E+08	268	2.25E+08	17	8.9884	4.78058
Nc-1-IP_pe	chr1	2.25E+08	2.25E+08	487	2.25E+08	21	7.10961	3.41419
Nc-1-IP_pe	chr1	2.25E+08	2.25E+08	610	2.25E+08	25	7.37317	3.1922
Nc-1-IP_pe	chr1	2.25E+08	2.25E+08	719	2.25E+08	22	4.53777	2.41958
Nc-1-IP_pe	chr1	2.25E+08	2.25E+08	668	2.25E+08	15	4.06016	2.62146
Nc-1-IP_pe	chr1	2.25E+08	2.25E+08	425	2.25E+08	14	3.55308	2.45762
Nc-1-IP_pe	chr1	2.25E+08	2.25E+08	431	2.25E+08	24	8.92072	3.82821
Nc-1-IP_pe	chr1	2.25E+08	2.25E+08	416	2.25E+08	19	5.15822	2.80735
Nc-1-IP_pe	chr1	2.25E+08	2.25E+08	548	2.25E+08	21	5.5349	2.81884
Nc-1-IP_pe	chr1	2.25E+08	2.25E+08	636	2.25E+08	26	9.32357	3.78397
Nc-1-IP_pe	chr1	2.25E+08	2.25E+08	629	2.25E+08	21	6.2632	3.08808
Nc-1-IP_pe	chr1	2.25E+08	2.25E+08	348	2.25E+08	18	5.52764	3.02971
Nc-1-IP_pe	chr1	2.25E+08	2.25E+08	344	2.25E+08	23	8.52058	3.77499
Nc-1-IP_pe	chr1	2.25E+08	2.25E+08	254	2.25E+08	17	3.33947	2.20998
Nc-1-IP_pe	chr1	2.25E+08	2.25E+08	309	2.25E+08	19	5.70288	3.02257
Nc-1-IP_pe	chr1	2.25E+08	2.25E+08	526	2.25E+08	15	4.40535	2.77711
Nc-1-IP_pe	chr1	2.25E+08	2.25E+08	331	2.25E+08	14	3.72153	2.5351
Nc-1-IP_pe	chr1	2.25E+08	2.25E+08	426	2.25E+08	18	5.72638	3.11298
Nc-1-IP_pe	chr1	2.25E+08	2.25E+08	416	2.25E+08	16	3.44488	2.29898
Nc-1-IP_pe	chr1	2.25E+08	2.25E+08	1217	2.25E+08	27	9.98694	3.92412
Nc-1-IP_pe	chr1	2.25E+08	2.25E+08	398	2.25E+08	14	4.12147	2.72194
Nc-1-IP_pe	chr1	2.25E+08	2.25E+08	299	2.25E+08	18	6.65364	3.51391
Nc-1-IP_pe	chr1	2.26E+08	2.26E+08	342	2.26E+08	17	5.54967	3.12324
Nc-1-IP_pe	chr1	2.26E+08	2.26E+08	530	2.26E+08	21	7.4689	3.5571
Nc-1-IP_pe	chr1	2.26E+08	2.26E+08	705	2.26E+08	18	7.04825	3.69086
Nc-1-IP_pe	chr1	2.26E+08	2.26E+08	339	2.26E+08	14	6.46071	3.9032
Nc-1-IP_pe	chr1	2.26E+08	2.26E+08	322	2.26E+08	12	3.16137	2.39719
Nc-1-IP_pe	chr1	2.26E+08	2.26E+08	301	2.26E+08	14	6.06519	3.69269
Nc-1-IP_pe	chr1	2.26E+08	2.26E+08	389	2.26E+08	13	5.36853	3.44651
Nc-1-IP_pe	chr1	2.26E+08	2.26E+08	355	2.26E+08	15	5.1177	3.10809
Nc-1-IP_pe	chr1	2.26E+08	2.26E+08	262	2.26E+08	20	6.49719	3.259
Nc-1-IP_pe	chr1	2.26E+08	2.26E+08	272	2.26E+08	17	5.9911	3.31919
Nc-1-IP_pe	chr1	2.26E+08	2.26E+08	602	2.26E+08	28	8.30387	3.286

Nc-1-IP_pe	chr1	2.26E+08	2.26E+08	507	2.26E+08	23	6.25615	2.94664
Nc-1-IP_pe	chr1	2.26E+08	2.26E+08	451	2.26E+08	17	5.54967	3.12324
Nc-1-IP_pe	chr1	2.26E+08	2.26E+08	255	2.26E+08	15	5.34394	3.21607
Nc-1-IP_pe	chr1	2.26E+08	2.26E+08	301	2.26E+08	15	3.46204	2.35851
Nc-1-IP_pe	chr1	2.26E+08	2.26E+08	491	2.26E+08	17	6.38155	3.49661
Nc-1-IP_pe	chr1	2.26E+08	2.26E+08	264	2.26E+08	12	3.47963	2.55766
Nc-1-IP_pe	chr1	2.26E+08	2.26E+08	346	2.26E+08	15	6.19428	3.63445
Nc-1-IP_pe	chr1	2.26E+08	2.26E+08	467	2.26E+08	20	6.08336	3.09555
Nc-1-IP_pe	chr1	2.26E+08	2.26E+08	289	2.26E+08	15	5.34394	3.21607
Nc-1-IP_pe	chr1	2.26E+08	2.26E+08	822	2.26E+08	15	2.95965	2.14351
Nc-1-IP_pe	chr1	2.26E+08	2.26E+08	727	2.26E+08	15	4.68057	2.9034
Nc-1-IP_pe	chr1	2.26E+08	2.26E+08	879	2.26E+08	19	7.28916	3.68706
Nc-1-IP_pe	chr1	2.26E+08	2.26E+08	308	2.26E+08	18	6.40661	3.40507
Nc-1-IP_pe	chr1	2.26E+08	2.26E+08	969	2.26E+08	18	7.02099	3.67851
Nc-1-IP_pe	chr1	2.26E+08	2.26E+08	281	2.26E+08	13	3.86418	2.67468
Nc-1-IP_pe	chr1	2.26E+08	2.26E+08	262	2.26E+08	20	7.85753	3.8227
Nc-1-IP_pe	chr1	2.26E+08	2.26E+08	974	2.26E+08	22	5.72303	2.82387
Nc-1-IP_pe	chr1	2.26E+08	2.26E+08	258	2.26E+08	18	6.27579	3.34803
Nc-1-IP_pe	chr1	2.26E+08	2.26E+08	757	2.26E+08	22	6.07094	2.94697
Nc-1-IP_pe	chr1	2.26E+08	2.26E+08	823	2.26E+08	16	7.53857	4.18505
Nc-1-IP_pe	chr1	2.26E+08	2.26E+08	711	2.26E+08	17	6.47874	3.54137
Nc-1-IP_pe	chr1	2.26E+08	2.26E+08	555	2.26E+08	22	5.72303	2.82387
Nc-1-IP_pe	chr1	2.26E+08	2.26E+08	321	2.26E+08	15	5.58327	3.33181
Nc-1-IP_pe	chr1	2.26E+08	2.26E+08	402	2.26E+08	29	13.3265	4.91524
Nc-1-IP_pe	chr1	2.26E+08	2.26E+08	469	2.26E+08	19	4.23334	2.45554
Nc-1-IP_pe	chr1	2.26E+08	2.26E+08	702	2.26E+08	22	6.84408	3.22845
Nc-1-IP_pe	chr1	2.26E+08	2.26E+08	348	2.26E+08	17	5.14803	2.94914
Nc-1-IP_pe	chr1	2.26E+08	2.26E+08	848	2.26E+08	19	4.97584	2.73666
Nc-1-IP_pe	chr1	2.26E+08	2.26E+08	257	2.26E+08	19	7.31609	3.69885
Nc-1-IP_pe	chr1	2.26E+08	2.26E+08	281	2.26E+08	16	5.18047	3.04664
Nc-1-IP_pe	chr1	2.26E+08	2.26E+08	590	2.26E+08	19	6.78661	3.47027
Nc-1-IP_pe	chr1	2.26E+08	2.26E+08	354	2.26E+08	15	4.7819	2.95039
Nc-1-IP_pe	chr1	2.26E+08	2.26E+08	1000	2.26E+08	16	4.25252	2.63824
Nc-1-IP_pe	chr1	2.26E+08	2.26E+08	664	2.26E+08	13	4.9946	3.24853
Nc-1-IP_pe	chr1	2.26E+08	2.26E+08	1774	2.26E+08	23	11.3126	4.93555
Nc-1-IP_pe	chr1	2.26E+08	2.26E+08	693	2.26E+08	22	6.44373	3.08129
Nc-1-IP_pe	chr1	2.26E+08	2.26E+08	417	2.26E+08	14	3.90277	2.61926
Nc-1-IP_pe	chr1	2.26E+08	2.26E+08	383	2.26E+08	16	3.65575	2.38624
Nc-1-IP_pe	chr1	2.26E+08	2.26E+08	277	2.26E+08	14	3.66592	2.50944
Nc-1-IP_pe	chr1	2.26E+08	2.26E+08	629	2.26E+08	19	4.23334	2.45554
Nc-1-IP_pe	chr1	2.26E+08	2.26E+08	786	2.26E+08	14	3.55308	2.45762
Nc-1-IP_pe	chr1	2.26E+08	2.26E+08	546	2.26E+08	19	5.51632	2.94814
Nc-1-IP_pe	chr1	2.26E+08	2.26E+08	279	2.26E+08	16	6.08355	3.46522
Nc-1-IP_pe	chr1	2.26E+08	2.26E+08	631	2.26E+08	17	4.78101	2.79343
Nc-1-IP_pe	chr1	2.26E+08	2.26E+08	294	2.26E+08	16	5.85814	3.35873

Nc-1-IP_pe	chr1	2.26E+08	2.26E+08	473	2.26E+08	22	6.52351	3.11038
Nc-1-IP_pe	chr1	2.26E+08	2.26E+08	480	2.26E+08	16	5.18047	3.04664
Nc-1-IP_pe	chr1	2.26E+08	2.26E+08	784	2.26E+08	25	9.7397	4.03495
Nc-1-IP_pe	chr1	2.26E+08	2.26E+08	319	2.26E+08	20	7.45226	3.65043
Nc-1-IP_pe	chr1	2.26E+08	2.26E+08	265	2.26E+08	23	8.42853	3.73941
Nc-1-IP_pe	chr1	2.26E+08	2.26E+08	432	2.26E+08	23	5.28318	2.6185
Nc-1-IP_pe	chr1	2.26E+08	2.26E+08	441	2.26E+08	20	5.07791	2.71309
Nc-1-IP_pe	chr1	2.26E+08	2.26E+08	572	2.26E+08	23	4.72742	2.43753
Nc-1-IP_pe	chr1	2.26E+08	2.26E+08	445	2.26E+08	27	9.006	3.58762
Nc-1-IP_pe	chr1	2.26E+08	2.26E+08	812	2.26E+08	17	5.79346	3.23086
Nc-1-IP_pe	chr1	2.26E+08	2.26E+08	517	2.26E+08	24	7.61242	3.34923
Nc-1-IP_pe	chr1	2.26E+08	2.26E+08	540	2.26E+08	23	6.89706	3.17149
Nc-1-IP_pe	chr1	2.26E+08	2.26E+08	533	2.26E+08	20	5.01743	2.69071
Nc-1-IP_pe	chr1	2.26E+08	2.26E+08	282	2.26E+08	17	4.78101	2.79343
Nc-1-IP_pe	chr1	2.26E+08	2.26E+08	548	2.26E+08	20	6.49719	3.259
Nc-1-IP_pe	chr1	2.26E+08	2.26E+08	392	2.26E+08	14	4.31659	2.81462
Nc-1-IP_pe	chr1	2.26E+08	2.26E+08	704	2.26E+08	21	8.702	4.06873
Nc-1-IP_pe	chr1	2.26E+08	2.26E+08	281	2.26E+08	18	6.63038	3.50359
Nc-1-IP_pe	chr1	2.26E+08	2.26E+08	623	2.26E+08	16	5.37454	3.13479
Nc-1-IP_pe	chr1	2.26E+08	2.26E+08	387	2.26E+08	18	4.96968	2.80074
Nc-1-IP_pe	chr1	2.26E+08	2.26E+08	873	2.26E+08	20	5.87894	3.01614
Nc-1-IP_pe	chr1	2.26E+08	2.26E+08	595	2.26E+08	19	3.96692	2.35708
Nc-1-IP_pe	chr1	2.26E+08	2.26E+08	487	2.26E+08	26	6.42685	2.82576
Nc-1-IP_pe	chr1	2.26E+08	2.26E+08	413	2.26E+08	17	5.35342	3.03768
Nc-1-IP_pe	chr1	2.26E+08	2.26E+08	278	2.26E+08	22	10.0534	4.52509
Nc-1-IP_pe	chr1	2.26E+08	2.26E+08	758	2.26E+08	31	12.1968	4.28702
Nc-1-IP_pe	chr1	2.26E+08	2.26E+08	580	2.26E+08	17	4.78101	2.79343
Nc-1-IP_pe	chr1	2.26E+08	2.26E+08	500	2.26E+08	14	3.96259	2.64722
Nc-1-IP_pe	chr1	2.26E+08	2.26E+08	527	2.26E+08	17	7.00757	3.7892
Nc-1-IP_pe	chr1	2.26E+08	2.26E+08	370	2.26E+08	16	6.28027	3.55926
Nc-1-IP_pe	chr1	2.26E+08	2.26E+08	408	2.26E+08	15	5.63281	3.35597
Nc-1-IP_pe	chr1	2.26E+08	2.26E+08	511	2.26E+08	13	4.41954	2.952
Nc-1-IP_pe	chr1	2.26E+08	2.26E+08	1131	2.26E+08	16	6.64217	3.73495
Nc-1-IP_pe	chr1	2.26E+08	2.26E+08	451	2.26E+08	19	7.84735	3.93486
Nc-1-IP_pe	chr1	2.26E+08	2.26E+08	1318	2.26E+08	20	6.94658	3.44067
Nc-1-IP_pe	chr1	2.26E+08	2.26E+08	396	2.26E+08	18	5.23669	2.90944
Nc-1-IP_pe	chr1	2.26E+08	2.26E+08	837	2.26E+08	29	8.89222	3.39931
Nc-1-IP_pe	chr1	2.26E+08	2.26E+08	375	2.26E+08	22	10.7644	4.84175
Nc-1-IP_pe	chr1	2.26E+08	2.26E+08	625	2.26E+08	20	9.39627	4.511
Nc-1-IP_pe	chr1	2.26E+08	2.26E+08	520	2.26E+08	20	7.97282	3.87239
Nc-1-IP_pe	chr1	2.26E+08	2.26E+08	332	2.26E+08	15	5.58327	3.33181
Nc-1-IP_pe	chr1	2.26E+08	2.26E+08	445	2.26E+08	19	6.32622	3.27682
Nc-1-IP_pe	chr1	2.26E+08	2.26E+08	471	2.26E+08	13	5.06993	3.28809
Nc-1-IP_pe	chr1	2.26E+08	2.26E+08	309	2.26E+08	17	7.02067	3.79543
Nc-1-IP_pe	chr1	2.26E+08	2.26E+08	652	2.26E+08	21	4.0961	2.31438

Nc-1-IP_pe	chr1	2.26E+08	2.26E+08	362	2.26E+08	15	5.20142	3.14789
Nc-1-IP_pe	chr1	2.26E+08	2.26E+08	557	2.26E+08	17	4.78101	2.79343
Nc-1-IP_pe	chr1	2.26E+08	2.26E+08	456	2.26E+08	20	5.70098	2.94771
Nc-1-IP_pe	chr1	2.26E+08	2.26E+08	738	2.26E+08	16	4.96462	2.94973
Nc-1-IP_pe	chr1	2.26E+08	2.26E+08	528	2.26E+08	14	3.55308	2.45762
Nc-1-IP_pe	chr1	2.26E+08	2.26E+08	394	2.26E+08	23	10.7795	4.7016
Nc-1-IP_pe	chr1	2.26E+08	2.26E+08	449	2.26E+08	27	5.2249	2.42484
Nc-1-IP_pe	chr1	2.26E+08	2.26E+08	568	2.26E+08	20	7.97282	3.87239
Nc-1-IP_pe	chr1	2.26E+08	2.26E+08	395	2.26E+08	18	6.05776	3.25389
Nc-1-IP_pe	chr1	2.26E+08	2.26E+08	308	2.26E+08	20	6.07662	3.09292
Nc-1-IP_pe	chr1	2.26E+08	2.26E+08	383	2.26E+08	15	4.69043	2.90796
Nc-1-IP_pe	chr1	2.26E+08	2.26E+08	805	2.26E+08	21	4.90204	2.59278
Nc-1-IP_pe	chr1	2.26E+08	2.26E+08	317	2.26E+08	19	5.80235	3.06257
Nc-1-IP_pe	chr1	2.26E+08	2.26E+08	260	2.26E+08	19	5.93483	3.11617
Nc-1-IP_pe	chr1	2.26E+08	2.26E+08	395	2.26E+08	18	3.08421	2.07298
Nc-1-IP_pe	chr1	2.26E+08	2.26E+08	434	2.26E+08	18	4.6358	2.66698
Nc-1-IP_pe	chr1	2.26E+08	2.26E+08	354	2.26E+08	20	7.0741	3.49302
Nc-1-IP_pe	chr1	2.26E+08	2.26E+08	315	2.26E+08	11	3.82842	2.8241
Nc-1-IP_pe	chr1	2.26E+08	2.26E+08	461	2.26E+08	12	3.58823	2.61305
Nc-1-IP_pe	chr1	2.26E+08	2.26E+08	625	2.26E+08	14	6.06519	3.69269
Nc-1-IP_pe	chr1	2.26E+08	2.26E+08	526	2.26E+08	13	4.86362	3.18015
Nc-1-IP_pe	chr1	2.26E+08	2.26E+08	929	2.26E+08	15	5.64529	3.36206
Nc-1-IP_pe	chr1	2.26E+08	2.26E+08	881	2.26E+08	23	3.39514	2.01894
Nc-1-IP_pe	chr1	2.26E+08	2.26E+08	332	2.26E+08	18	7.18674	3.75388
Nc-1-IP_pe	chr1	2.26E+08	2.26E+08	825	2.26E+08	21	5.2077	2.70109
Nc-1-IP_pe	chr1	2.26E+08	2.26E+08	273	2.26E+08	18	5.72638	3.11298
Nc-1-IP_pe	chr1	2.26E+08	2.26E+08	429	2.26E+08	17	4.13464	2.52661
Nc-1-IP_pe	chr1	2.26E+08	2.26E+08	317	2.26E+08	18	4.6358	2.66698
Nc-1-IP_pe	chr1	2.26E+08	2.26E+08	347	2.26E+08	18	4.96968	2.80074
Nc-1-IP_pe	chr1	2.26E+08	2.26E+08	309	2.26E+08	16	4.59224	2.7853
Nc-1-IP_pe	chr1	2.26E+08	2.26E+08	916	2.26E+08	21	7.58657	3.60451
Nc-1-IP_pe	chr1	2.26E+08	2.26E+08	313	2.26E+08	21	9.98999	4.63886
Nc-1-IP_pe	chr1	2.26E+08	2.26E+08	368	2.26E+08	14	5.71266	3.50871
Nc-1-IP_pe	chr1	2.26E+08	2.26E+08	728	2.26E+08	14	4.60011	2.95114
Nc-1-IP_pe	chr1	2.26E+08	2.26E+08	541	2.26E+08	15	5.20142	3.14789
Nc-1-IP_pe	chr1	2.26E+08	2.26E+08	723	2.26E+08	15	4.23553	2.70016
Nc-1-IP_pe	chr1	2.26E+08	2.26E+08	417	2.26E+08	15	5.20142	3.14789
Nc-1-IP_pe	chr1	2.26E+08	2.26E+08	450	2.26E+08	12	4.24064	2.95299
Nc-1-IP_pe	chr1	2.26E+08	2.26E+08	536	2.26E+08	15	6.10627	3.59023
Nc-1-IP_pe	chr1	2.26E+08	2.26E+08	770	2.26E+08	28	6.5317	2.75496
Nc-1-IP_pe	chr1	2.26E+08	2.26E+08	303	2.26E+08	17	6.1569	3.39405
Nc-1-IP_pe	chr1	2.26E+08	2.26E+08	891	2.26E+08	19	8.4638	4.21715
Nc-1-IP_pe	chr1	2.26E+08	2.26E+08	314	2.26E+08	15	6.14813	3.61124
Nc-1-IP_pe	chr1	2.26E+08	2.26E+08	786	2.26E+08	14	5.29552	3.29555
Nc-1-IP_pe	chr1	2.26E+08	2.26E+08	622	2.26E+08	15	3.74763	2.48304

Nc-1-IP_pe chr1	2.26E+08	2.26E+08	491	2.26E+08	14	3.26761	2.32785
Nc-1-IP_pe chr1	2.26E+08	2.26E+08	445	2.26E+08	15	5.98302	3.52865
Nc-1-IP_pe chr1	2.26E+08	2.26E+08	332	2.26E+08	17	4.13464	2.52661
Nc-1-IP_pe chr1	2.26E+08	2.26E+08	420	2.26E+08	13	4.02562	2.7544
Nc-1-IP_pe chr1	2.26E+08	2.26E+08	583	2.26E+08	18	7.62523	3.95652
Nc-1-IP_pe chr1	2.26E+08	2.26E+08	346	2.26E+08	16	4.96462	2.94973
Nc-1-IP_pe chr1	2.26E+08	2.26E+08	493	2.26E+08	17	6.43293	3.52024
Nc-1-IP_pe chr1	2.26E+08	2.26E+08	340	2.26E+08	19	6.78661	3.47027
Nc-1-IP_pe chr1	2.26E+08	2.26E+08	870	2.26E+08	16	5.09774	3.00936
Nc-1-IP_pe chr1	2.26E+08	2.26E+08	1085	2.26E+08	20	8.39952	4.05893
Nc-1-IP_pe chr1	2.26E+08	2.26E+08	351	2.26E+08	17	4.96303	2.87025
Nc-1-IP_pe chr1	2.26E+08	2.26E+08	357	2.26E+08	18	6.71734	3.54221
Nc-1-IP_pe chr1	2.26E+08	2.26E+08	278	2.26E+08	16	5.20142	3.05611
Nc-1-IP_pe chr1	2.26E+08	2.26E+08	419	2.26E+08	16	5.49479	3.1899
Nc-1-IP_pe chr1	2.26E+08	2.26E+08	403	2.26E+08	24	8.04377	3.50368
Nc-1-IP_pe chr1	2.26E+08	2.26E+08	276	2.26E+08	17	3.33947	2.20998
Nc-1-IP_pe chr1	2.26E+08	2.26E+08	338	2.26E+08	27	7.35328	3.05492
Nc-1-IP_pe chr1	2.26E+08	2.26E+08	669	2.26E+08	18	6.15736	3.29676
Nc-1-IP_pe chr1	2.26E+08	2.26E+08	375	2.26E+08	13	4.24481	2.8638
Nc-1-IP_pe chr1	2.26E+08	2.26E+08	332	2.26E+08	17	6.25544	3.43888
Nc-1-IP_pe chr1	2.26E+08	2.26E+08	298	2.26E+08	15	5.63281	3.35597
Nc-1-IP_pe chr1	2.26E+08	2.26E+08	652	2.26E+08	15	3.74763	2.48304
Nc-1-IP_pe chr1	2.26E+08	2.26E+08	425	2.26E+08	20	7.43645	3.64378
Nc-1-IP_pe chr1	2.26E+08	2.26E+08	324	2.26E+08	11	3.64839	2.72584
Nc-1-IP_pe chr1	2.26E+08	2.26E+08	305	2.26E+08	13	4.78908	3.14145
Nc-1-IP_pe chr1	2.26E+08	2.26E+08	311	2.26E+08	15	4.7819	2.95039
Nc-1-IP_pe chr1	2.26E+08	2.26E+08	411	2.26E+08	16	4.25252	2.63824
Nc-1-IP_pe chr1	2.26E+08	2.26E+08	563	2.26E+08	13	3.67377	2.58159
Nc-1-IP_pe chr1	2.26E+08	2.26E+08	260	2.26E+08	19	8.4638	4.21715
Nc-1-IP_pe chr1	2.26E+08	2.26E+08	361	2.26E+08	14	5.19764	3.24624
Nc-1-IP_pe chr1	2.26E+08	2.26E+08	347	2.26E+08	17	5.14803	2.94914
Nc-1-IP_pe chr1	2.26E+08	2.26E+08	508	2.26E+08	16	6.65665	3.74206
Nc-1-IP_pe chr1	2.26E+08	2.26E+08	914	2.26E+08	19	6.78661	3.47027
Nc-1-IP_pe chr1	2.27E+08	2.27E+08	353	2.27E+08	13	4.02562	2.7544
Nc-1-IP_pe chr1	2.27E+08	2.27E+08	321	2.27E+08	11	3.49235	2.64145
Nc-1-IP_pe chr1	2.27E+08	2.27E+08	781	2.27E+08	19	3.71826	2.26621
Nc-1-IP_pe chr1	2.27E+08	2.27E+08	514	2.27E+08	16	4.96462	2.94973
Nc-1-IP_pe chr1	2.27E+08	2.27E+08	393	2.27E+08	21	11.0937	5.15654
Nc-1-IP_pe chr1	2.27E+08	2.27E+08	275	2.27E+08	15	5.20142	3.14789
Nc-1-IP_pe chr1	2.27E+08	2.27E+08	918	2.27E+08	27	9.006	3.58762
Nc-1-IP_pe chr1	2.27E+08	2.27E+08	952	2.27E+08	14	6.34788	3.84271
Nc-1-IP_pe chr1	2.27E+08	2.27E+08	347	2.27E+08	20	6.49719	3.259
Nc-1-IP_pe chr1	2.27E+08	2.27E+08	411	2.27E+08	17	6.47874	3.54137
Nc-1-IP_pe chr1	2.27E+08	2.27E+08	774	2.27E+08	14	5.35794	3.32713
Nc-1-IP_pe chr1	2.27E+08	2.27E+08	271	2.27E+08	12	3.86684	2.7567

Nc-1-IP_pe	chr1	2.27E+08	2.27E+08	279	2.27E+08	17	5.54967	3.12324
Nc-1-IP_pe	chr1	2.27E+08	2.27E+08	979	2.27E+08	16	6.21376	3.52735
Nc-1-IP_pe	chr1	2.27E+08	2.27E+08	403	2.27E+08	19	6.32622	3.27682
Nc-1-IP_pe	chr1	2.27E+08	2.27E+08	346	2.27E+08	15	5.52215	3.3021
Nc-1-IP_pe	chr1	2.27E+08	2.27E+08	420	2.27E+08	15	5.58327	3.33181
Nc-1-IP_pe	chr1	2.27E+08	2.27E+08	334	2.27E+08	16	6.70037	3.76354
Nc-1-IP_pe	chr1	2.27E+08	2.27E+08	1172	2.27E+08	14	4.52309	2.91384
Nc-1-IP_pe	chr1	2.27E+08	2.27E+08	574	2.27E+08	19	7.29128	3.68799
Nc-1-IP_pe	chr1	2.27E+08	2.27E+08	291	2.27E+08	16	5.82808	3.34463
Nc-1-IP_pe	chr1	2.27E+08	2.27E+08	304	2.27E+08	15	5.07393	3.08736
Nc-1-IP_pe	chr1	2.27E+08	2.27E+08	576	2.27E+08	21	6.45254	3.15977
Nc-1-IP_pe	chr1	2.27E+08	2.27E+08	806	2.27E+08	19	5.60545	2.98361
Nc-1-IP_pe	chr1	2.27E+08	2.27E+08	664	2.27E+08	18	4.32723	2.54542
Nc-1-IP_pe	chr1	2.27E+08	2.27E+08	989	2.27E+08	19	6.11371	3.18917
Nc-1-IP_pe	chr1	2.27E+08	2.27E+08	1810	2.27E+08	24	8.04377	3.50368
Nc-1-IP_pe	chr1	2.27E+08	2.27E+08	263	2.27E+08	15	4.35086	2.75234
Nc-1-IP_pe	chr1	2.27E+08	2.27E+08	1195	2.27E+08	23	10.3515	4.51804
Nc-1-IP_pe	chr1	2.27E+08	2.27E+08	575	2.27E+08	20	7.45226	3.65043
Nc-1-IP_pe	chr1	2.27E+08	2.27E+08	431	2.27E+08	16	5.30773	3.10433
Nc-1-IP_pe	chr1	2.27E+08	2.27E+08	289	2.27E+08	19	4.5193	2.56258
Nc-1-IP_pe	chr1	2.27E+08	2.27E+08	463	2.27E+08	18	6.84717	3.60021
Nc-1-IP_pe	chr1	2.27E+08	2.27E+08	458	2.27E+08	15	4.40338	2.77622
Nc-1-IP_pe	chr1	2.27E+08	2.27E+08	317	2.27E+08	15	4.06016	2.62146
Nc-1-IP_pe	chr1	2.27E+08	2.27E+08	522	2.27E+08	19	5.15822	2.80735
Nc-1-IP_pe	chr1	2.27E+08	2.27E+08	370	2.27E+08	16	4.68897	2.82769
Nc-1-IP_pe	chr1	2.27E+08	2.27E+08	336	2.27E+08	17	5.14803	2.94914
Nc-1-IP_pe	chr1	2.27E+08	2.27E+08	577	2.27E+08	17	5.6711	3.17666
Nc-1-IP_pe	chr1	2.27E+08	2.27E+08	414	2.27E+08	14	4.82889	3.06294
Nc-1-IP_pe	chr1	2.27E+08	2.27E+08	952	2.27E+08	25	12.4735	5.13688
Nc-1-IP_pe	chr1	2.27E+08	2.27E+08	1229	2.27E+08	19	4.89276	2.70468
Nc-1-IP_pe	chr1	2.27E+08	2.27E+08	419	2.27E+08	15	7.19874	4.15407
Nc-1-IP_pe	chr1	2.27E+08	2.27E+08	366	2.27E+08	10	3.80898	2.90812
Nc-1-IP_pe	chr1	2.27E+08	2.27E+08	276	2.27E+08	11	2.96397	2.36091
Nc-1-IP_pe	chr1	2.27E+08	2.27E+08	318	2.27E+08	13	4.82453	3.15983
Nc-1-IP_pe	chr1	2.27E+08	2.27E+08	457	2.27E+08	15	5.66913	3.37372
Nc-1-IP_pe	chr1	2.27E+08	2.27E+08	835	2.27E+08	17	4.78101	2.79343
Nc-1-IP_pe	chr1	2.27E+08	2.27E+08	285	2.27E+08	16	6.8987	3.86161
Nc-1-IP_pe	chr1	2.27E+08	2.27E+08	850	2.27E+08	15	6.02372	3.54894
Nc-1-IP_pe	chr1	2.27E+08	2.27E+08	451	2.27E+08	17	7.06589	3.81697
Nc-1-IP_pe	chr1	2.27E+08	2.27E+08	440	2.27E+08	18	4.96968	2.80074
Nc-1-IP_pe	chr1	2.27E+08	2.27E+08	949	2.27E+08	21	7.10961	3.41419
Nc-1-IP_pe	chr1	2.27E+08	2.27E+08	303	2.27E+08	17	6.47874	3.54137
Nc-1-IP_pe	chr1	2.27E+08	2.27E+08	660	2.27E+08	13	4.3784	2.93116
Nc-1-IP_pe	chr1	2.27E+08	2.27E+08	665	2.27E+08	11	3.32925	2.55401
Nc-1-IP_pe	chr1	2.27E+08	2.27E+08	349	2.27E+08	17	6.47874	3.54137

Nc-1-IP_pe	chr1	2.27E+08	2.27E+08	445	2.27E+08	24	6.44146	2.94634
Nc-1-IP_pe	chr1	2.27E+08	2.27E+08	495	2.27E+08	18	6.02422	3.23951
Nc-1-IP_pe	chr1	2.27E+08	2.27E+08	568	2.27E+08	17	6.78076	3.68203
Nc-1-IP_pe	chr1	2.27E+08	2.27E+08	799	2.27E+08	14	5.22188	3.25843
Nc-1-IP_pe	chr1	2.27E+08	2.27E+08	406	2.27E+08	15	4.7819	2.95039
Nc-1-IP_pe	chr1	2.27E+08	2.27E+08	267	2.27E+08	14	4.96308	3.1292
Nc-1-IP_pe	chr1	2.27E+08	2.27E+08	288	2.27E+08	12	3.47963	2.55766
Nc-1-IP_pe	chr1	2.27E+08	2.27E+08	271	2.27E+08	19	3.96692	2.35708
Nc-1-IP_pe	chr1	2.27E+08	2.27E+08	356	2.27E+08	20	5.34663	2.81336
Nc-1-IP_pe	chr1	2.27E+08	2.27E+08	287	2.27E+08	13	3.07238	2.29378
Nc-1-IP_pe	chr1	2.27E+08	2.27E+08	261	2.27E+08	16	6.24025	3.54005
Nc-1-IP_pe	chr1	2.27E+08	2.27E+08	335	2.27E+08	13	3.35769	2.42919
Nc-1-IP_pe	chr1	2.27E+08	2.27E+08	392	2.27E+08	16	5.37454	3.13479
Nc-1-IP_pe	chr1	2.27E+08	2.27E+08	384	2.27E+08	15	5.43804	3.26139
Nc-1-IP_pe	chr1	2.27E+08	2.27E+08	316	2.27E+08	21	5.5349	2.81884
Nc-1-IP_pe	chr1	2.27E+08	2.27E+08	269	2.27E+08	20	6.97747	3.45331
Nc-1-IP_pe	chr1	2.27E+08	2.27E+08	477	2.27E+08	17	5.96578	3.30782
Nc-1-IP_pe	chr1	2.27E+08	2.27E+08	923	2.27E+08	23	6.89706	3.17149
Nc-1-IP_pe	chr1	2.27E+08	2.27E+08	426	2.27E+08	20	6.94658	3.44067
Nc-1-IP_pe	chr1	2.27E+08	2.27E+08	268	2.27E+08	16	4.3356	2.67395
Nc-1-IP_pe	chr1	2.27E+08	2.27E+08	365	2.27E+08	17	4.13464	2.52661
Nc-1-IP_pe	chr1	2.27E+08	2.27E+08	388	2.27E+08	12	3.77112	2.7071
Nc-1-IP_pe	chr1	2.27E+08	2.27E+08	647	2.27E+08	21	6.66991	3.24296
Nc-1-IP_pe	chr1	2.27E+08	2.27E+08	290	2.27E+08	16	3.65575	2.38624
Nc-1-IP_pe	chr1	2.27E+08	2.27E+08	342	2.27E+08	15	4.06016	2.62146
Nc-1-IP_pe	chr1	2.27E+08	2.27E+08	584	2.27E+08	16	3.39261	2.27748
Nc-1-IP_pe	chr1	2.27E+08	2.27E+08	576	2.27E+08	19	6.80149	3.4766
Nc-1-IP_pe	chr1	2.27E+08	2.27E+08	739	2.27E+08	16	5.57425	3.22652
Nc-1-IP_pe	chr1	2.27E+08	2.27E+08	260	2.27E+08	15	4.80057	2.95908
Nc-1-IP_pe	chr1	2.27E+08	2.27E+08	707	2.27E+08	18	5.33197	2.94862
Nc-1-IP_pe	chr1	2.27E+08	2.27E+08	825	2.27E+08	17	6.38155	3.49661
Nc-1-IP_pe	chr1	2.27E+08	2.27E+08	275	2.27E+08	13	3.67377	2.58159
Nc-1-IP_pe	chr1	2.27E+08	2.27E+08	270	2.27E+08	16	7.53857	4.18505
Nc-1-IP_pe	chr1	2.27E+08	2.27E+08	254	2.27E+08	11	4.06403	2.95415
Nc-1-IP_pe	chr1	2.27E+08	2.27E+08	402	2.27E+08	14	5.51541	3.4073
Nc-1-IP_pe	chr1	2.27E+08	2.27E+08	278	2.27E+08	13	6.62436	4.14003
Nc-1-IP_pe	chr1	2.27E+08	2.27E+08	345	2.27E+08	12	6.48784	4.21727
Nc-1-IP_pe	chr1	2.27E+08	2.27E+08	442	2.27E+08	19	4.8268	2.67939
Nc-1-IP_pe	chr1	2.27E+08	2.27E+08	466	2.27E+08	13	4.86362	3.18015
Nc-1-IP_pe	chr1	2.27E+08	2.27E+08	322	2.27E+08	17	7.6273	4.08876
Nc-1-IP_pe	chr1	2.27E+08	2.27E+08	532	2.27E+08	17	4.78101	2.79343
Nc-1-IP_pe	chr1	2.27E+08	2.27E+08	554	2.27E+08	11	5.10577	3.5486
Nc-1-IP_pe	chr1	2.27E+08	2.27E+08	463	2.27E+08	17	9.09431	4.8363
Nc-1-IP_pe	chr1	2.27E+08	2.27E+08	312	2.27E+08	16	8.37012	4.62105
Nc-1-IP_pe	chr1	2.27E+08	2.27E+08	896	2.27E+08	21	4.90204	2.59278

Nc-1-IP_pe	chr1	2.27E+08	2.27E+08	282	2.27E+08	15	6.19428	3.63445
Nc-1-IP_pe	chr1	2.27E+08	2.27E+08	277	2.27E+08	15	5.20142	3.14789
Nc-1-IP_pe	chr1	2.27E+08	2.27E+08	1012	2.27E+08	21	5.88587	2.94733
Nc-1-IP_pe	chr1	2.27E+08	2.27E+08	636	2.27E+08	22	8.24538	3.76835
Nc-1-IP_pe	chr1	2.27E+08	2.27E+08	290	2.27E+08	17	8.41245	4.48226
Nc-1-IP_pe	chr1	2.27E+08	2.27E+08	284	2.27E+08	14	6.44441	3.89444
Nc-1-IP_pe	chr1	2.27E+08	2.27E+08	539	2.27E+08	20	9.21699	4.428
Nc-1-IP_pe	chr1	2.27E+08	2.27E+08	306	2.27E+08	13	4.67336	3.0817
Nc-1-IP_pe	chr1	2.27E+08	2.27E+08	303	2.27E+08	16	5.37454	3.13479
Nc-1-IP_pe	chr1	2.27E+08	2.27E+08	378	2.27E+08	17	6.25544	3.43888
Nc-1-IP_pe	chr1	2.27E+08	2.27E+08	413	2.27E+08	17	6.13258	3.38303
Nc-1-IP_pe	chr1	2.27E+08	2.27E+08	456	2.27E+08	11	4.61014	3.26192
Nc-1-IP_pe	chr1	2.27E+08	2.27E+08	921	2.27E+08	14	5.18557	3.24018
Nc-1-IP_pe	chr1	2.27E+08	2.27E+08	951	2.27E+08	14	4.60011	2.95114
Nc-1-IP_pe	chr1	2.27E+08	2.27E+08	993	2.27E+08	21	7.10961	3.41419
Nc-1-IP_pe	chr1	2.27E+08	2.27E+08	396	2.27E+08	16	4.96462	2.94973
Nc-1-IP_pe	chr1	2.27E+08	2.27E+08	636	2.27E+08	23	9.9969	4.3688
Nc-1-IP_pe	chr1	2.27E+08	2.27E+08	293	2.27E+08	22	9.42371	4.25368
Nc-1-IP_pe	chr1	2.28E+08	2.28E+08	1369	2.28E+08	26	8.58731	3.52764
Nc-1-IP_pe	chr1	2.28E+08	2.28E+08	440	2.28E+08	17	6.47874	3.54137
Nc-1-IP_pe	chr1	2.28E+08	2.28E+08	558	2.28E+08	19	8.4638	4.21715
Nc-1-IP_pe	chr1	2.28E+08	2.28E+08	785	2.28E+08	15	4.41451	2.78128
Nc-1-IP_pe	chr1	2.28E+08	2.28E+08	1754	2.28E+08	28	9.16687	3.56053
Nc-1-IP_pe	chr1	2.28E+08	2.28E+08	568	2.28E+08	15	4.06504	2.62364
Nc-1-IP_pe	chr1	2.28E+08	2.28E+08	464	2.28E+08	18	6.63038	3.50359
Nc-1-IP_pe	chr1	2.28E+08	2.28E+08	430	2.28E+08	21	8.85564	4.13481
Nc-1-IP_pe	chr1	2.28E+08	2.28E+08	300	2.28E+08	16	3.39261	2.27748
Nc-1-IP_pe	chr1	2.28E+08	2.28E+08	1176	2.28E+08	15	2.95965	2.14351
Nc-1-IP_pe	chr1	2.28E+08	2.28E+08	1489	2.28E+08	19	4.97584	2.73666
Nc-1-IP_pe	chr1	2.28E+08	2.28E+08	799	2.28E+08	20	9.94634	4.77022
Nc-1-IP_pe	chr1	2.28E+08	2.28E+08	752	2.28E+08	17	7.02067	3.79543
Nc-1-IP_pe	chr1	2.28E+08	2.28E+08	344	2.28E+08	12	4.70082	3.20033
Nc-1-IP_pe	chr1	2.28E+08	2.28E+08	660	2.28E+08	15	5.20142	3.14789
Nc-1-IP_pe	chr1	2.28E+08	2.28E+08	687	2.28E+08	11	4.01189	2.92523
Nc-1-IP_pe	chr1	2.28E+08	2.28E+08	259	2.28E+08	9	3.50273	2.82441
Nc-1-IP_pe	chr1	2.28E+08	2.28E+08	331	2.28E+08	10	3.4604	2.70797
Nc-1-IP_pe	chr1	2.28E+08	2.28E+08	451	2.28E+08	15	7.46994	4.29894
Nc-1-IP_pe	chr1	2.28E+08	2.28E+08	996	2.28E+08	22	12.3673	5.5949
Nc-1-IP_pe	chr1	2.28E+08	2.28E+08	883	2.28E+08	11	3.28475	2.53029
Nc-1-IP_pe	chr1	2.28E+08	2.28E+08	275	2.28E+08	17	6.64406	3.61808
Nc-1-IP_pe	chr1	2.28E+08	2.28E+08	613	2.28E+08	18	7.73136	4.00629
Nc-1-IP_pe	chr1	2.28E+08	2.28E+08	271	2.28E+08	19	4.50014	2.55537
Nc-1-IP_pe	chr1	2.28E+08	2.28E+08	868	2.28E+08	20	6.49719	3.259
Nc-1-IP_pe	chr1	2.28E+08	2.28E+08	915	2.28E+08	20	6.81239	3.38595
Nc-1-IP_pe	chr1	2.28E+08	2.28E+08	1651	2.28E+08	37	16.9855	5.19965

Nc-1-IP_pe	chr1	2.28E+08	2.28E+08	575	2.28E+08	18	3.77591	2.33276
Nc-1-IP_pe	chr1	2.28E+08	2.28E+08	311	2.28E+08	20	4.76247	2.59711
Nc-1-IP_pe	chr1	2.28E+08	2.28E+08	292	2.28E+08	21	8.40557	3.94271
Nc-1-IP_pe	chr1	2.28E+08	2.28E+08	394	2.28E+08	13	3.67244	2.58094
Nc-1-IP_pe	chr1	2.28E+08	2.28E+08	409	2.28E+08	18	6.76888	3.56519
Nc-1-IP_pe	chr1	2.28E+08	2.28E+08	264	2.28E+08	14	4.97448	3.13485
Nc-1-IP_pe	chr1	2.28E+08	2.28E+08	508	2.28E+08	15	5.00915	3.05678
Nc-1-IP_pe	chr1	2.28E+08	2.28E+08	381	2.28E+08	19	6.32622	3.27682
Nc-1-IP_pe	chr1	2.28E+08	2.28E+08	361	2.28E+08	15	5.21796	3.15577
Nc-1-IP_pe	chr1	2.28E+08	2.28E+08	405	2.28E+08	16	6.09641	3.47134
Nc-1-IP_pe	chr1	2.28E+08	2.28E+08	432	2.28E+08	16	5.37454	3.13479
Nc-1-IP_pe	chr1	2.28E+08	2.28E+08	266	2.28E+08	17	4.44438	2.65333
Nc-1-IP_pe	chr1	2.28E+08	2.28E+08	358	2.28E+08	24	5.47323	2.62998
Nc-1-IP_pe	chr1	2.28E+08	2.28E+08	329	2.28E+08	14	3.38729	2.38203
Nc-1-IP_pe	chr1	2.28E+08	2.28E+08	535	2.28E+08	19	5.15822	2.80735
Nc-1-IP_pe	chr1	2.28E+08	2.28E+08	590	2.28E+08	19	3.96692	2.35708
Nc-1-IP_pe	chr1	2.28E+08	2.28E+08	389	2.28E+08	14	4.49286	2.89924
Nc-1-IP_pe	chr1	2.28E+08	2.28E+08	444	2.28E+08	14	3.96259	2.64722
Nc-1-IP_pe	chr1	2.28E+08	2.28E+08	286	2.28E+08	13	3.36696	2.43362
Nc-1-IP_pe	chr1	2.28E+08	2.28E+08	360	2.28E+08	20	7.0741	3.49302
Nc-1-IP_pe	chr1	2.28E+08	2.28E+08	256	2.28E+08	15	4.50738	2.8237
Nc-1-IP_pe	chr1	2.28E+08	2.28E+08	263	2.28E+08	14	4.53323	2.91874
Nc-1-IP_pe	chr1	2.28E+08	2.28E+08	1182	2.28E+08	27	8.63021	3.46282
Nc-1-IP_pe	chr1	2.28E+08	2.28E+08	411	2.28E+08	17	4.83452	2.81593
Nc-1-IP_pe	chr1	2.28E+08	2.28E+08	641	2.28E+08	20	6.92979	3.4338
Nc-1-IP_pe	chr1	2.28E+08	2.28E+08	536	2.28E+08	19	4.73127	2.64291
Nc-1-IP_pe	chr1	2.28E+08	2.28E+08	682	2.28E+08	22	7.27519	3.39036
Nc-1-IP_pe	chr1	2.28E+08	2.28E+08	322	2.28E+08	13	6.96475	4.33508
Nc-1-IP_pe	chr1	2.28E+08	2.28E+08	731	2.28E+08	21	5.88587	2.94733
Nc-1-IP_pe	chr1	2.28E+08	2.28E+08	293	2.28E+08	11	2.96397	2.36091
Nc-1-IP_pe	chr1	2.28E+08	2.28E+08	479	2.28E+08	20	7.58419	3.7061
Nc-1-IP_pe	chr1	2.28E+08	2.28E+08	451	2.28E+08	24	9.33472	3.98646
Nc-1-IP_pe	chr1	2.28E+08	2.28E+08	417	2.28E+08	16	5.82808	3.34463
Nc-1-IP_pe	chr1	2.28E+08	2.28E+08	373	2.28E+08	20	5.59659	2.90787
Nc-1-IP_pe	chr1	2.28E+08	2.28E+08	330	2.28E+08	20	5.50604	2.87349
Nc-1-IP_pe	chr1	2.28E+08	2.28E+08	714	2.28E+08	23	3.21249	1.96277
Nc-1-IP_pe	chr1	2.28E+08	2.28E+08	530	2.28E+08	26	10.3785	4.16756
Nc-1-IP_pe	chr1	2.28E+08	2.28E+08	959	2.28E+08	28	11.0712	4.20692
Nc-1-IP_pe	chr1	2.28E+08	2.28E+08	986	2.28E+08	35	15.1161	4.8229
Nc-1-IP_pe	chr1	2.28E+08	2.28E+08	303	2.28E+08	21	4.87356	2.58277
Nc-1-IP_pe	chr1	2.28E+08	2.28E+08	258	2.28E+08	23	6.41066	3.00019
Nc-1-IP_pe	chr1	2.28E+08	2.28E+08	306	2.28E+08	27	7.72987	3.17269
Nc-1-IP_pe	chr1	2.28E+08	2.28E+08	648	2.28E+08	18	4.88519	2.76666
Nc-1-IP_pe	chr1	2.28E+08	2.28E+08	338	2.28E+08	19	5.51001	2.94564
Nc-1-IP_pe	chr1	2.28E+08	2.28E+08	358	2.28E+08	17	4.85263	2.82357

Nc-1-IP_pe	chr1	2.28E+08	2.28E+08	342	2.28E+08	19	4.8268	2.67939
Nc-1-IP_pe	chr1	2.28E+08	2.28E+08	3724	2.28E+08	29	14.0832	5.20541
Nc-1-IP_pe	chr1	2.28E+08	2.28E+08	580	2.28E+08	13	4.41954	2.952
Nc-1-IP_pe	chr1	2.28E+08	2.28E+08	567	2.28E+08	16	6.48567	3.65855
Nc-1-IP_pe	chr1	2.28E+08	2.28E+08	571	2.28E+08	19	5.70288	3.02257
Nc-1-IP_pe	chr1	2.28E+08	2.28E+08	533	2.28E+08	17	3.58449	2.30632
Nc-1-IP_pe	chr1	2.28E+08	2.28E+08	428	2.28E+08	15	2.95965	2.14351
Nc-1-IP_pe	chr1	2.28E+08	2.28E+08	535	2.28E+08	25	9.18728	3.82933
Nc-1-IP_pe	chr1	2.28E+08	2.28E+08	2594	2.28E+08	21	3.85951	2.23441
Nc-1-IP_pe	chr1	2.28E+08	2.28E+08	283	2.28E+08	18	6.46724	3.43164
Nc-1-IP_pe	chr1	2.28E+08	2.28E+08	274	2.28E+08	19	6.80149	3.4766
Nc-1-IP_pe	chr1	2.28E+08	2.28E+08	777	2.28E+08	22	6.44373	3.08129
Nc-1-IP_pe	chr1	2.28E+08	2.28E+08	1373	2.28E+08	17	5.54967	3.12324
Nc-1-IP_pe	chr1	2.28E+08	2.28E+08	890	2.28E+08	22	8.25322	3.77148
Nc-1-IP_pe	chr1	2.28E+08	2.28E+08	1543	2.28E+08	24	6.80641	3.06942
Nc-1-IP_pe	chr1	2.28E+08	2.28E+08	780	2.28E+08	23	7.3183	3.32328
Nc-1-IP_pe	chr1	2.28E+08	2.28E+08	328	2.28E+08	19	3.26827	2.10398
Nc-1-IP_pe	chr1	2.28E+08	2.28E+08	508	2.28E+08	19	6.70429	3.43532
Nc-1-IP_pe	chr1	2.28E+08	2.28E+08	313	2.28E+08	16	5.37454	3.13479
Nc-1-IP_pe	chr1	2.28E+08	2.28E+08	697	2.28E+08	18	8.10208	4.1823
Nc-1-IP_pe	chr1	2.28E+08	2.28E+08	533	2.28E+08	17	7.85637	4.20196
Nc-1-IP_pe	chr1	2.28E+08	2.28E+08	525	2.28E+08	17	7.02067	3.79543
Nc-1-IP_pe	chr1	2.28E+08	2.28E+08	416	2.28E+08	20	5.34663	2.81336
Nc-1-IP_pe	chr1	2.28E+08	2.28E+08	461	2.28E+08	18	4.04135	2.43445
Nc-1-IP_pe	chr1	2.28E+08	2.28E+08	404	2.28E+08	27	10.1235	3.97223
Nc-1-IP_pe	chr1	2.28E+08	2.28E+08	320	2.28E+08	20	4.68706	2.56965
Nc-1-IP_pe	chr1	2.28E+08	2.28E+08	426	2.28E+08	22	5.09279	2.60614
Nc-1-IP_pe	chr1	2.28E+08	2.28E+08	338	2.28E+08	22	4.53777	2.41958
Nc-1-IP_pe	chr1	2.28E+08	2.28E+08	595	2.28E+08	22	7.7408	3.56938
Nc-1-IP_pe	chr1	2.28E+08	2.28E+08	496	2.28E+08	16	4.25252	2.63824
Nc-1-IP_pe	chr1	2.28E+08	2.28E+08	946	2.28E+08	25	5.66296	2.64066
Nc-1-IP_pe	chr1	2.28E+08	2.28E+08	1345	2.28E+08	20	6.94658	3.44067
Nc-1-IP_pe	chr1	2.28E+08	2.28E+08	1030	2.28E+08	21	7.10914	3.414
Nc-1-IP_pe	chr1	2.28E+08	2.28E+08	731	2.28E+08	19	6.43457	3.32191
Nc-1-IP_pe	chr1	2.28E+08	2.28E+08	568	2.28E+08	19	6.11371	3.18917
Nc-1-IP_pe	chr1	2.28E+08	2.28E+08	387	2.28E+08	18	4.1654	2.48241
Nc-1-IP_pe	chr1	2.28E+08	2.28E+08	588	2.28E+08	18	3.52899	2.23922
Nc-1-IP_pe	chr1	2.28E+08	2.28E+08	572	2.28E+08	19	6.78661	3.47027
Nc-1-IP_pe	chr1	2.28E+08	2.28E+08	556	2.28E+08	17	4.87083	2.83124
Nc-1-IP_pe	chr1	2.28E+08	2.28E+08	458	2.28E+08	22	5.72303	2.82387
Nc-1-IP_pe	chr1	2.28E+08	2.28E+08	1676	2.28E+08	19	6.78661	3.47027
Nc-1-IP_pe	chr1	2.28E+08	2.28E+08	414	2.28E+08	17	5.9911	3.31919
Nc-1-IP_pe	chr1	2.28E+08	2.28E+08	3045	2.28E+08	24	10.2246	4.33784
Nc-1-IP_pe	chr1	2.28E+08	2.28E+08	289	2.28E+08	22	7.27519	3.39036
Nc-1-IP_pe	chr1	2.28E+08	2.28E+08	436	2.28E+08	25	10.3209	4.25738

Nc-1-IP_pe	chr1	2.28E+08	2.28E+08	272	2.28E+08	22	6.84408	3.22845
Nc-1-IP_pe	chr1	2.28E+08	2.28E+08	364	2.28E+08	20	7.06192	3.48801
Nc-1-IP_pe	chr1	2.28E+08	2.28E+08	1034	2.28E+08	27	9.006	3.58762
Nc-1-IP_pe	chr1	2.28E+08	2.28E+08	617	2.28E+08	28	11.101	4.2175
Nc-1-IP_pe	chr1	2.28E+08	2.28E+08	821	2.28E+08	16	4.4556	2.72582
Nc-1-IP_pe	chr1	2.28E+08	2.28E+08	600	2.28E+08	18	5.96882	3.21582
Nc-1-IP_pe	chr1	2.28E+08	2.28E+08	593	2.28E+08	20	5.24441	2.77505
Nc-1-IP_pe	chr1	2.28E+08	2.28E+08	328	2.28E+08	17	3.90564	2.43421
Nc-1-IP_pe	chr1	2.28E+08	2.28E+08	261	2.28E+08	17	5.6711	3.17666
Nc-1-IP_pe	chr1	2.28E+08	2.28E+08	977	2.28E+08	22	7.88972	3.62756
Nc-1-IP_pe	chr1	2.28E+08	2.28E+08	1056	2.28E+08	25	8.69165	3.64955
Nc-1-IP_pe	chr1	2.28E+08	2.28E+08	415	2.28E+08	25	6.76714	2.99167
Nc-1-IP_pe	chr1	2.28E+08	2.28E+08	272	2.28E+08	19	3.06441	2.03128
Nc-1-IP_pe	chr1	2.28E+08	2.28E+08	274	2.28E+08	22	6.44373	3.08129
Nc-1-IP_pe	chr1	2.28E+08	2.28E+08	2069	2.28E+08	26	11.7219	4.68487
Nc-1-IP_pe	chr1	2.28E+08	2.28E+08	490	2.28E+08	20	7.23483	3.55954
Nc-1-IP_pe	chr1	2.28E+08	2.28E+08	1486	2.28E+08	19	4.8268	2.67939
Nc-1-IP_pe	chr1	2.28E+08	2.28E+08	465	2.28E+08	16	4.52591	2.75637
Nc-1-IP_pe	chr1	2.28E+08	2.28E+08	497	2.28E+08	27	8.55392	3.43775
Nc-1-IP_pe	chr1	2.28E+08	2.28E+08	267	2.28E+08	15	3.59911	2.41806
Nc-1-IP_pe	chr1	2.28E+08	2.28E+08	705	2.28E+08	23	4.00593	2.20858
Nc-1-IP_pe	chr1	2.28E+08	2.28E+08	259	2.28E+08	10	3.40382	2.67582
Nc-1-IP_pe	chr1	2.28E+08	2.28E+08	774	2.28E+08	16	4.59754	2.78762
Nc-1-IP_pe	chr1	2.28E+08	2.28E+08	375	2.28E+08	26	5.80081	2.63511
Nc-1-IP_pe	chr1	2.28E+08	2.28E+08	1251	2.28E+08	27	6.99773	2.94558
Nc-1-IP_pe	chr1	2.28E+08	2.28E+08	311	2.28E+08	17	4.36596	2.62105
Nc-1-IP_pe	chr1	2.28E+08	2.28E+08	2766	2.28E+08	20	6.62973	3.31212
Nc-1-IP_pe	chr1	2.28E+08	2.28E+08	366	2.28E+08	23	9.24806	4.06209
Nc-1-IP_pe	chr1	2.28E+08	2.28E+08	302	2.28E+08	20	7.40034	3.62863
Nc-1-IP_pe	chr1	2.28E+08	2.28E+08	290	2.28E+08	18	6.22907	3.32777
Nc-1-IP_pe	chr1	2.28E+08	2.28E+08	854	2.28E+08	19	6.32622	3.27682
Nc-1-IP_pe	chr1	2.28E+08	2.28E+08	291	2.28E+08	14	3.55308	2.45762
Nc-1-IP_pe	chr1	2.28E+08	2.28E+08	946	2.28E+08	19	6.68027	3.42515
Nc-1-IP_pe	chr1	2.28E+08	2.28E+08	300	2.28E+08	15	4.32392	2.74012
Nc-1-IP_pe	chr1	2.28E+08	2.28E+08	634	2.28E+08	21	6.66991	3.24296
Nc-1-IP_pe	chr1	2.28E+08	2.28E+08	256	2.28E+08	18	4.96968	2.80074
Nc-1-IP_pe	chr1	2.28E+08	2.28E+08	277	2.28E+08	21	5.42814	2.78021
Nc-1-IP_pe	chr1	2.28E+08	2.28E+08	755	2.28E+08	27	8.08969	3.28717
Nc-1-IP_pe	chr1	2.28E+08	2.28E+08	327	2.28E+08	17	5.23251	2.98544
Nc-1-IP_pe	chr1	2.28E+08	2.28E+08	383	2.28E+08	17	5.49884	3.10099
Nc-1-IP_pe	chr1	2.28E+08	2.28E+08	379	2.28E+08	20	4.42487	2.47493
Nc-1-IP_pe	chr1	2.28E+08	2.28E+08	1356	2.28E+08	24	8.53898	3.68518
Nc-1-IP_pe	chr1	2.28E+08	2.28E+08	259	2.28E+08	13	3.89124	2.688
Nc-1-IP_pe	chr1	2.28E+08	2.28E+08	344	2.28E+08	17	7.06589	3.81697
Nc-1-IP_pe	chr1	2.28E+08	2.28E+08	440	2.28E+08	15	4.06016	2.62146

Nc-1-IP_pe	chr1	2.28E+08	2.28E+08	392	2.28E+08	14	4.13959	2.7305
Nc-1-IP_pe	chr1	2.28E+08	2.28E+08	343	2.28E+08	16	6.24025	3.54005
Nc-1-IP_pe	chr1	2.28E+08	2.28E+08	530	2.28E+08	13	4.02562	2.7544
Nc-1-IP_pe	chr1	2.28E+08	2.28E+08	352	2.28E+08	12	5.03305	3.38277
Nc-1-IP_pe	chr1	2.28E+08	2.28E+08	275	2.28E+08	16	3.65575	2.38624
Nc-1-IP_pe	chr1	2.28E+08	2.28E+08	1812	2.28E+08	23	7.89844	3.53777
Nc-1-IP_pe	chr1	2.28E+08	2.28E+08	828	2.28E+08	17	2.90036	2.03959
Nc-1-IP_pe	chr1	2.28E+08	2.28E+08	464	2.28E+08	22	6.49374	3.09951
Nc-1-IP_pe	chr1	2.28E+08	2.28E+08	1304	2.28E+08	24	8.05897	3.50918
Nc-1-IP_pe	chr1	2.28E+08	2.28E+08	493	2.28E+08	17	3.83544	2.40609
Nc-1-IP_pe	chr1	2.28E+08	2.28E+08	337	2.28E+08	19	6.60887	3.39501
Nc-1-IP_pe	chr1	2.28E+08	2.28E+08	591	2.28E+08	17	3.63296	2.32551
Nc-1-IP_pe	chr1	2.28E+08	2.28E+08	263	2.28E+08	20	8.23315	3.98571
Nc-1-IP_pe	chr1	2.28E+08	2.28E+08	739	2.28E+08	17	5.9911	3.31919
Nc-1-IP_pe	chr1	2.28E+08	2.28E+08	294	2.28E+08	20	9.21699	4.428
Nc-1-IP_pe	chr1	2.28E+08	2.28E+08	2164	2.28E+08	20	10.7672	5.16976
Nc-1-IP_pe	chr1	2.28E+08	2.28E+08	267	2.28E+08	14	5.43414	3.36584
Nc-1-IP_pe	chr1	2.28E+08	2.28E+08	286	2.28E+08	14	5.74019	3.52296
Nc-1-IP_pe	chr1	2.28E+08	2.28E+08	343	2.28E+08	23	9.50079	4.16433
Nc-1-IP_pe	chr1	2.28E+08	2.28E+08	267	2.28E+08	19	6.78661	3.47027
Nc-1-IP_pe	chr1	2.28E+08	2.28E+08	288	2.28E+08	15	4.50738	2.8237
Nc-1-IP_pe	chr1	2.28E+08	2.28E+08	343	2.28E+08	16	5.98198	3.41707
Nc-1-IP_pe	chr1	2.28E+08	2.28E+08	273	2.28E+08	10	3.35471	2.64799
Nc-1-IP_pe	chr1	2.28E+08	2.28E+08	597	2.28E+08	16	6.74448	3.78526
Nc-1-IP_pe	chr1	2.28E+08	2.28E+08	820	2.28E+08	21	4.90204	2.59278
Nc-1-IP_pe	chr1	2.28E+08	2.28E+08	1339	2.28E+08	20	6.08336	3.09555
Nc-1-IP_pe	chr1	2.28E+08	2.28E+08	502	2.28E+08	17	5.4568	3.08264
Nc-1-IP_pe	chr1	2.28E+08	2.28E+08	526	2.28E+08	19	5.15822	2.80735
Nc-1-IP_pe	chr1	2.28E+08	2.28E+08	483	2.28E+08	25	9.89861	4.09514
Nc-1-IP_pe	chr1	2.28E+08	2.28E+08	364	2.28E+08	17	4.7363	2.77467
Nc-1-IP_pe	chr1	2.28E+08	2.28E+08	323	2.28E+08	21	6.25033	3.08324
Nc-1-IP_pe	chr1	2.28E+08	2.28E+08	657	2.28E+08	23	8.6544	3.827
Nc-1-IP_pe	chr1	2.28E+08	2.28E+08	816	2.28E+08	24	6.80641	3.06942
Nc-1-IP_pe	chr1	2.28E+08	2.28E+08	2043	2.28E+08	19	6.32622	3.27682
Nc-1-IP_pe	chr1	2.28E+08	2.28E+08	260	2.28E+08	18	5.33158	2.94846
Nc-1-IP_pe	chr1	2.28E+08	2.28E+08	676	2.28E+08	18	5.83846	3.16035
Nc-1-IP_pe	chr1	2.28E+08	2.28E+08	313	2.28E+08	17	5.59492	3.14311
Nc-1-IP_pe	chr1	2.28E+08	2.28E+08	515	2.28E+08	16	5.34015	3.1191
Nc-1-IP_pe	chr1	2.28E+08	2.28E+08	358	2.28E+08	14	3.71352	2.5314
Nc-1-IP_pe	chr1	2.28E+08	2.28E+08	278	2.28E+08	17	5.70414	3.19126
Nc-1-IP_pe	chr1	2.28E+08	2.28E+08	770	2.28E+08	19	4.8268	2.67939
Nc-1-IP_pe	chr1	2.28E+08	2.28E+08	1878	2.28E+08	20	7.43645	3.64378
Nc-1-IP_pe	chr1	2.28E+08	2.28E+08	540	2.28E+08	19	6.22205	3.23373
Nc-1-IP_pe	chr1	2.28E+08	2.28E+08	302	2.28E+08	16	4.3356	2.67395
Nc-1-IP_pe	chr1	2.28E+08	2.28E+08	366	2.28E+08	19	6.22205	3.23373

Nc-1-IP_pe	chr1	2.28E+08	2.28E+08	399	2.28E+08	22	8.59669	3.90999
Nc-1-IP_pe	chr1	2.28E+08	2.28E+08	368	2.28E+08	13	3.28408	2.39406
Nc-1-IP_pe	chr1	2.28E+08	2.28E+08	799	2.28E+08	29	12.7994	4.71873
Nc-1-IP_pe	chr1	2.28E+08	2.28E+08	1117	2.28E+08	17	4.96303	2.87025
Nc-1-IP_pe	chr1	2.28E+08	2.28E+08	382	2.28E+08	15	3.20025	2.24588
Nc-1-IP_pe	chr1	2.28E+08	2.28E+08	293	2.28E+08	21	8.10596	3.8173
Nc-1-IP_pe	chr1	2.28E+08	2.28E+08	310	2.28E+08	18	5.94685	3.20644
Nc-1-IP_pe	chr1	2.28E+08	2.28E+08	632	2.28E+08	23	6.25615	2.94664
Nc-1-IP_pe	chr1	2.28E+08	2.28E+08	2127	2.28E+08	17	6.33071	3.47329
Nc-1-IP_pe	chr1	2.28E+08	2.28E+08	482	2.28E+08	16	4.96462	2.94973
Nc-1-IP_pe	chr1	2.28E+08	2.28E+08	258	2.28E+08	17	5.15375	2.9516
Nc-1-IP_pe	chr1	2.28E+08	2.28E+08	527	2.28E+08	15	4.32392	2.74012
Nc-1-IP_pe	chr1	2.28E+08	2.28E+08	317	2.28E+08	20	6.28238	3.1737
Nc-1-IP_pe	chr1	2.28E+08	2.28E+08	296	2.28E+08	22	6.04768	2.93867
Nc-1-IP_pe	chr1	2.28E+08	2.28E+08	1265	2.28E+08	22	7.13291	3.33652
Nc-1-IP_pe	chr1	2.28E+08	2.28E+08	893	2.28E+08	31	9.66075	3.49133
Nc-1-IP_pe	chr1	2.28E+08	2.28E+08	1473	2.28E+08	22	6.91523	3.25492
Nc-1-IP_pe	chr1	2.28E+08	2.28E+08	1076	2.28E+08	26	9.32357	3.78397
Nc-1-IP_pe	chr1	2.28E+08	2.28E+08	1498	2.28E+08	24	6.09885	2.83276
Nc-1-IP_pe	chr1	2.28E+08	2.28E+08	340	2.28E+08	21	3.63688	2.15978
Nc-1-IP_pe	chr1	2.28E+08	2.28E+08	295	2.28E+08	19	6.83841	3.49234
Nc-1-IP_pe	chr1	2.28E+08	2.28E+08	286	2.28E+08	12	3.83688	2.74114
Nc-1-IP_pe	chr1	2.28E+08	2.28E+08	426	2.28E+08	23	8.38947	3.72437
Nc-1-IP_pe	chr1	2.28E+08	2.28E+08	397	2.28E+08	14	3.80267	2.57267
Nc-1-IP_pe	chr1	2.28E+08	2.28E+08	816	2.28E+08	20	6.08336	3.09555
Nc-1-IP_pe	chr1	2.28E+08	2.28E+08	441	2.28E+08	19	5.90435	3.10381
Nc-1-IP_pe	chr1	2.28E+08	2.28E+08	1980	2.28E+08	21	6.66991	3.24296
Nc-1-IP_pe	chr1	2.28E+08	2.28E+08	382	2.28E+08	18	2.88361	1.99878
Nc-1-IP_pe	chr1	2.28E+08	2.28E+08	744	2.28E+08	20	5.41719	2.83991
Nc-1-IP_pe	chr1	2.28E+08	2.28E+08	1023	2.28E+08	20	6.8943	3.4193
Nc-1-IP_pe	chr1	2.28E+08	2.28E+08	1087	2.28E+08	20	6.49719	3.259
Nc-1-IP_pe	chr1	2.28E+08	2.28E+08	496	2.28E+08	16	5.04686	2.98652
Nc-1-IP_pe	chr1	2.28E+08	2.28E+08	455	2.28E+08	19	3.06441	2.03128
Nc-1-IP_pe	chr1	2.28E+08	2.28E+08	923	2.28E+08	17	5.14803	2.94914
Nc-1-IP_pe	chr1	2.28E+08	2.28E+08	384	2.28E+08	17	5.54967	3.12324
Nc-1-IP_pe	chr1	2.28E+08	2.28E+08	547	2.28E+08	15	5.00915	3.05678
Nc-1-IP_pe	chr1	2.28E+08	2.28E+08	297	2.28E+08	14	4.60011	2.95114
Nc-1-IP_pe	chr1	2.28E+08	2.28E+08	298	2.28E+08	19	4.23334	2.45554
Nc-1-IP_pe	chr1	2.28E+08	2.28E+08	650	2.28E+08	21	7.58657	3.60451
Nc-1-IP_pe	chr1	2.28E+08	2.28E+08	286	2.28E+08	16	5.37454	3.13479
Nc-1-IP_pe	chr1	2.28E+08	2.28E+08	563	2.28E+08	14	5.43414	3.36584
Nc-1-IP_pe	chr1	2.28E+08	2.28E+08	777	2.28E+08	27	8.12936	3.29991
Nc-1-IP_pe	chr1	2.28E+08	2.28E+08	816	2.28E+08	18	6.15736	3.29676
Nc-1-IP_pe	chr1	2.28E+08	2.28E+08	486	2.28E+08	17	5.14803	2.94914
Nc-1-IP_pe	chr1	2.28E+08	2.28E+08	370	2.28E+08	16	3.39261	2.27748

Nc-1-IP_pe	chr1	2.28E+08	2.28E+08	516	2.28E+08	13	3.6894	2.58919
Nc-1-IP_pe	chr1	2.28E+08	2.28E+08	641	2.28E+08	17	7.6273	4.08876
Nc-1-IP_pe	chr1	2.28E+08	2.28E+08	253	2.28E+08	14	6.10041	3.71126
Nc-1-IP_pe	chr1	2.28E+08	2.28E+08	621	2.28E+08	25	5.84305	2.69672
Nc-1-IP_pe	chr1	2.28E+08	2.28E+08	630	2.28E+08	25	8.69165	3.64955
Nc-1-IP_pe	chr1	2.28E+08	2.28E+08	916	2.28E+08	18	5.78519	3.1378
Nc-1-IP_pe	chr1	2.28E+08	2.28E+08	793	2.28E+08	24	10.9385	4.63096
Nc-1-IP_pe	chr1	2.28E+08	2.28E+08	335	2.28E+08	16	3.39261	2.27748
Nc-1-IP_pe	chr1	2.29E+08	2.29E+08	486	2.29E+08	16	4.25218	2.63809
Nc-1-IP_pe	chr1	2.29E+08	2.29E+08	885	2.29E+08	20	5.34663	2.81336
Nc-1-IP_pe	chr1	2.29E+08	2.29E+08	1011	2.29E+08	15	6.24734	3.66122
Nc-1-IP_pe	chr1	2.29E+08	2.29E+08	478	2.29E+08	17	7.06589	3.81697
Nc-1-IP_pe	chr1	2.29E+08	2.29E+08	877	2.29E+08	16	5.21193	3.06086
Nc-1-IP_pe	chr1	2.29E+08	2.29E+08	462	2.29E+08	16	4.74488	2.85228
Nc-1-IP_pe	chr1	2.29E+08	2.29E+08	2045	2.29E+08	79	14.866	2.73605
Nc-1-IP_pe	chr1	2.29E+08	2.29E+08	9474	2.29E+08	140	34.4547	3.42671
Nc-1-IP_pe	chr1	2.29E+08	2.29E+08	1537	2.29E+08	102	23.7352	3.22267
Nc-1-IP_pe	chr1	2.29E+08	2.29E+08	6152	2.29E+08	152	38.8311	3.54259
Nc-1-IP_pe	chr1	2.29E+08	2.29E+08	12513	2.29E+08	121	21.2334	2.69732
Nc-1-IP_pe	chr1	2.29E+08	2.29E+08	4264	2.29E+08	123	29.6153	3.34552
Nc-1-IP_pe	chr1	2.29E+08	2.29E+08	1910	2.29E+08	129	29.9752	3.26745
Nc-1-IP_pe	chr1	2.29E+08	2.29E+08	531	2.29E+08	18	7.18674	3.75388
Nc-1-IP_pe	chr1	2.29E+08	2.29E+08	277	2.29E+08	19	8.85804	4.40242
Nc-1-IP_pe	chr1	2.29E+08	2.29E+08	278	2.29E+08	17	10.1912	5.42842
Nc-1-IP_pe	chr1	2.29E+08	2.29E+08	282	2.29E+08	113	33.6045	3.94478
Nc-1-IP_pe	chr1	2.29E+08	2.29E+08	742	2.29E+08	15	5.66913	3.37372
Nc-1-IP_pe	chr1	2.29E+08	2.29E+08	520	2.29E+08	22	4.2847	2.33597
Nc-1-IP_pe	chr1	2.29E+08	2.29E+08	336	2.29E+08	13	3.35769	2.42919
Nc-1-IP_pe	chr1	2.29E+08	2.29E+08	1171	2.29E+08	20	9.45052	4.53626
Nc-1-IP_pe	chr1	2.29E+08	2.29E+08	342	2.29E+08	16	7.27441	4.05022
Nc-1-IP_pe	chr1	2.29E+08	2.29E+08	376	2.29E+08	15	4.06016	2.62146
Nc-1-IP_pe	chr1	2.29E+08	2.29E+08	270	2.29E+08	14	6.17624	3.75136
Nc-1-IP_pe	chr1	2.29E+08	2.29E+08	268	2.29E+08	18	8.27052	4.2634
Nc-1-IP_pe	chr1	2.29E+08	2.29E+08	435	2.29E+08	15	6.17625	3.62538
Nc-1-IP_pe	chr1	2.29E+08	2.29E+08	597	2.29E+08	16	4.25252	2.63824
Nc-1-IP_pe	chr1	2.29E+08	2.29E+08	746	2.29E+08	18	7.1522	3.73811
Nc-1-IP_pe	chr1	2.29E+08	2.29E+08	347	2.29E+08	19	8.19109	4.09114
Nc-1-IP_pe	chr1	2.29E+08	2.29E+08	855	2.29E+08	16	6.51374	3.67221
Nc-1-IP_pe	chr1	2.29E+08	2.29E+08	268	2.29E+08	18	9.01801	4.63161
Nc-1-IP_pe	chr1	2.29E+08	2.29E+08	342	2.29E+08	18	7.31456	3.81246
Nc-1-IP_pe	chr1	2.29E+08	2.29E+08	353	2.29E+08	18	7.07566	3.7033
Nc-1-IP_pe	chr1	2.29E+08	2.29E+08	283	2.29E+08	22	8.79433	3.99081
Nc-1-IP_pe	chr1	2.29E+08	2.29E+08	279	2.29E+08	17	8.68317	4.62148
Nc-1-IP_pe	chr1	2.29E+08	2.29E+08	365	2.29E+08	15	2.95965	2.14351
Nc-1-IP_pe	chr1	2.29E+08	2.29E+08	417	2.29E+08	20	8.14433	3.94687

Nc-1-IP_pe	chr1	2.29E+08	2.29E+08	484	2.29E+08	18	4.96968	2.80074
Nc-1-IP_pe	chr1	2.29E+08	2.29E+08	891	2.29E+08	18	4.96968	2.80074
Nc-1-IP_pe	chr1	2.29E+08	2.29E+08	695	2.29E+08	11	4.31539	3.09472
Nc-1-IP_pe	chr1	2.29E+08	2.29E+08	324	2.29E+08	16	5.82808	3.34463
Nc-1-IP_pe	chr1	2.29E+08	2.29E+08	300	2.29E+08	12	3.88634	2.76684
Nc-1-IP_pe	chr1	2.29E+08	2.29E+08	513	2.29E+08	16	5.95694	3.40524
Nc-1-IP_pe	chr1	2.29E+08	2.29E+08	322	2.29E+08	16	6.74448	3.78526
Nc-1-IP_pe	chr1	2.29E+08	2.29E+08	344	2.29E+08	16	4.96462	2.94973
Nc-1-IP_pe	chr1	2.29E+08	2.29E+08	1041	2.29E+08	16	6.92507	3.87472
Nc-1-IP_pe	chr1	2.29E+08	2.29E+08	678	2.29E+08	19	7.84735	3.93486
Nc-1-IP_pe	chr1	2.29E+08	2.29E+08	433	2.29E+08	14	4.21449	2.76599
Nc-1-IP_pe	chr1	2.29E+08	2.29E+08	801	2.29E+08	14	4.50291	2.90409
Nc-1-IP_pe	chr1	2.29E+08	2.29E+08	304	2.29E+08	17	6.23061	3.42756
Nc-1-IP_pe	chr1	2.29E+08	2.29E+08	645	2.29E+08	16	4.59224	2.7853
Nc-1-IP_pe	chr1	2.29E+08	2.29E+08	518	2.29E+08	19	8.03556	4.02008
Nc-1-IP_pe	chr1	2.29E+08	2.29E+08	276	2.29E+08	13	4.86362	3.18015
Nc-1-IP_pe	chr1	2.29E+08	2.29E+08	428	2.29E+08	14	5.03085	3.16286
Nc-1-IP_pe	chr1	2.29E+08	2.29E+08	590	2.29E+08	18	6.63038	3.50359
Nc-1-IP_pe	chr1	2.29E+08	2.29E+08	664	2.29E+08	15	4.06016	2.62146
Nc-1-IP_pe	chr1	2.29E+08	2.29E+08	454	2.29E+08	16	5.82808	3.34463
Nc-1-IP_pe	chr1	2.29E+08	2.29E+08	256	2.29E+08	20	6.08336	3.09555
Nc-1-IP_pe	chr1	2.29E+08	2.29E+08	264	2.29E+08	17	5.4568	3.08264
Nc-1-IP_pe	chr1	2.29E+08	2.29E+08	770	2.29E+08	17	4.44438	2.65333
Nc-1-IP_pe	chr1	2.29E+08	2.29E+08	402	2.29E+08	18	6.05776	3.25389
Nc-1-IP_pe	chr1	2.29E+08	2.29E+08	284	2.29E+08	17	5.9911	3.31919
Nc-1-IP_pe	chr1	2.29E+08	2.29E+08	332	2.29E+08	16	5.32931	3.11416
Nc-1-IP_pe	chr1	2.29E+08	2.29E+08	634	2.29E+08	17	4.96303	2.87025
Nc-1-IP_pe	chr1	2.29E+08	2.29E+08	423	2.29E+08	13	4.96982	3.23556
Nc-1-IP_pe	chr1	2.29E+08	2.29E+08	597	2.29E+08	17	4.13464	2.52661
Nc-1-IP_pe	chr1	2.29E+08	2.29E+08	273	2.29E+08	17	7.32147	3.9397
Nc-1-IP_pe	chr1	2.29E+08	2.29E+08	288	2.29E+08	17	4.44438	2.65333
Nc-1-IP_pe	chr1	2.29E+08	2.29E+08	1213	2.29E+08	16	5.94447	3.39936
Nc-1-IP_pe	chr1	2.29E+08	2.29E+08	299	2.29E+08	12	3.83688	2.74114
Nc-1-IP_pe	chr1	2.29E+08	2.29E+08	851	2.29E+08	14	3.96259	2.64722
Nc-1-IP_pe	chr1	2.29E+08	2.29E+08	287	2.29E+08	15	4.7819	2.95039
Nc-1-IP_pe	chr1	2.29E+08	2.29E+08	597	2.29E+08	18	2.88361	1.99878
Nc-1-IP_pe	chr1	2.29E+08	2.29E+08	462	2.29E+08	14	5.03085	3.16286
Nc-1-IP_pe	chr1	2.29E+08	2.29E+08	541	2.29E+08	17	6.5771	3.58692
Nc-1-IP_pe	chr1	2.29E+08	2.29E+08	591	2.29E+08	18	5.83846	3.16035
Nc-1-IP_pe	chr1	2.29E+08	2.29E+08	720	2.29E+08	15	4.23553	2.70016
Nc-1-IP_pe	chr1	2.29E+08	2.29E+08	426	2.29E+08	22	7.7408	3.56938
Nc-1-IP_pe	chr1	2.29E+08	2.29E+08	359	2.29E+08	22	8.24538	3.76835
Nc-1-IP_pe	chr1	2.29E+08	2.29E+08	876	2.29E+08	13	4.02562	2.7544
Nc-1-IP_pe	chr1	2.29E+08	2.29E+08	379	2.29E+08	23	6.25615	2.94664
Nc-1-IP_pe	chr1	2.29E+08	2.29E+08	426	2.29E+08	19	7.59289	3.82098

Nc-1-IP_pe	chr1	2.29E+08	2.29E+08	574	2.29E+08	14	3.13218	2.26693
Nc-1-IP_pe	chr1	2.29E+08	2.29E+08	707	2.29E+08	16	3.65575	2.38624
Nc-1-IP_pe	chr1	2.29E+08	2.29E+08	291	2.29E+08	12	4.69273	3.19593
Nc-1-IP_pe	chr1	2.29E+08	2.29E+08	284	2.29E+08	13	4.24481	2.8638
Nc-1-IP_pe	chr1	2.29E+08	2.29E+08	630	2.29E+08	19	3.06441	2.03128
Nc-1-IP_pe	chr1	2.29E+08	2.29E+08	277	2.29E+08	15	6.06481	3.56947
Nc-1-IP_pe	chr1	2.29E+08	2.29E+08	466	2.29E+08	11	4.5176	3.20915
Nc-1-IP_pe	chr1	2.29E+08	2.29E+08	331	2.29E+08	16	4.59224	2.7853
Nc-1-IP_pe	chr1	2.29E+08	2.29E+08	256	2.29E+08	12	4.4986	3.09087
Nc-1-IP_pe	chr1	2.29E+08	2.29E+08	602	2.29E+08	13	3.67377	2.58159
Nc-1-IP_pe	chr1	2.29E+08	2.29E+08	343	2.29E+08	15	5.22925	3.16116
Nc-1-IP_pe	chr1	2.29E+08	2.29E+08	355	2.29E+08	19	6.32622	3.27682
Nc-1-IP_pe	chr1	2.29E+08	2.29E+08	407	2.29E+08	13	4.41954	2.952
Nc-1-IP_pe	chr1	2.29E+08	2.29E+08	631	2.29E+08	15	6.19428	3.63445
Nc-1-IP_pe	chr1	2.29E+08	2.29E+08	707	2.29E+08	17	4.35793	2.61776
Nc-1-IP_pe	chr1	2.29E+08	2.29E+08	419	2.29E+08	17	5.9911	3.31919
Nc-1-IP_pe	chr1	2.29E+08	2.29E+08	321	2.29E+08	15	3.46204	2.35851
Nc-1-IP_pe	chr1	2.29E+08	2.29E+08	259	2.29E+08	18	6.63038	3.50359
Nc-1-IP_pe	chr1	2.29E+08	2.29E+08	337	2.29E+08	29	8.89222	3.39931
Nc-1-IP_pe	chr1	2.29E+08	2.29E+08	592	2.29E+08	22	5.95746	2.90658
Nc-1-IP_pe	chr1	2.29E+08	2.29E+08	296	2.29E+08	10	3.23111	2.57827
Nc-1-IP_pe	chr1	2.29E+08	2.29E+08	408	2.29E+08	13	4.02562	2.7544
Nc-1-IP_pe	chr1	2.29E+08	2.29E+08	464	2.29E+08	13	4.14549	2.81406
Nc-1-IP_pe	chr1	2.29E+08	2.29E+08	284	2.29E+08	14	4.23163	2.77414
Nc-1-IP_pe	chr1	2.29E+08	2.29E+08	258	2.29E+08	14	4.32616	2.81919
Nc-1-IP_pe	chr1	2.29E+08	2.29E+08	718	2.29E+08	23	11.8653	5.18429
Nc-1-IP_pe	chr1	2.29E+08	2.29E+08	512	2.29E+08	15	4.14926	2.66135
Nc-1-IP_pe	chr1	2.29E+08	2.29E+08	343	2.29E+08	19	4.8268	2.67939
Nc-1-IP_pe	chr1	2.29E+08	2.29E+08	401	2.29E+08	17	4.13464	2.52661
Nc-1-IP_pe	chr1	2.29E+08	2.29E+08	624	2.29E+08	16	5.50606	3.19508
Nc-1-IP_pe	chr1	2.29E+08	2.29E+08	654	2.29E+08	18	7.04825	3.69086
Nc-1-IP_pe	chr1	2.29E+08	2.29E+08	261	2.29E+08	14	3.26761	2.32785
Nc-1-IP_pe	chr1	2.29E+08	2.29E+08	316	2.29E+08	20	6.38846	3.2157
Nc-1-IP_pe	chr1	2.29E+08	2.29E+08	333	2.29E+08	13	4.96982	3.23556
Nc-1-IP_pe	chr1	2.29E+08	2.29E+08	699	2.29E+08	17	6.92131	3.74829
Nc-1-IP_pe	chr1	2.29E+08	2.29E+08	346	2.29E+08	16	6.8987	3.86161
Nc-1-IP_pe	chr1	2.29E+08	2.29E+08	729	2.29E+08	18	4.6358	2.66698
Nc-1-IP_pe	chr1	2.29E+08	2.29E+08	829	2.29E+08	19	6.92557	3.52962
Nc-1-IP_pe	chr1	2.29E+08	2.29E+08	839	2.29E+08	20	7.58419	3.7061
Nc-1-IP_pe	chr1	2.29E+08	2.29E+08	362	2.29E+08	11	4.23076	3.04719
Nc-1-IP_pe	chr1	2.29E+08	2.29E+08	378	2.29E+08	15	4.40338	2.77622
Nc-1-IP_pe	chr1	2.29E+08	2.29E+08	382	2.29E+08	12	3.83688	2.74114
Nc-1-IP_pe	chr1	2.29E+08	2.29E+08	458	2.29E+08	12	5.23082	3.49289
Nc-1-IP_pe	chr1	2.3E+08	2.3E+08	448	2.3E+08	27	9.48843	3.75114
Nc-1-IP_pe	chr1	2.3E+08	2.3E+08	562	2.3E+08	14	5.03085	3.16286

Nc-1-IP_pe	chr1	2.3E+08	2.3E+08	425	2.3E+08	18	9.1091	4.6774
Nc-1-IP_pe	chr1	2.3E+08	2.3E+08	281	2.3E+08	14	5.43414	3.36584
Nc-1-IP_pe	chr1	2.3E+08	2.3E+08	1351	2.3E+08	21	7.72183	3.65936
Nc-1-IP_pe	chr1	2.3E+08	2.3E+08	392	2.3E+08	13	5.13388	3.3218
Nc-1-IP_pe	chr1	2.3E+08	2.3E+08	520	2.3E+08	12	5.43913	3.61006
Nc-1-IP_pe	chr1	2.3E+08	2.3E+08	325	2.3E+08	19	9.92774	4.92358
Nc-1-IP_pe	chr1	2.3E+08	2.3E+08	270	2.3E+08	11	3.78682	2.80131
Nc-1-IP_pe	chr1	2.3E+08	2.3E+08	332	2.3E+08	13	5.79292	3.67605
Nc-1-IP_pe	chr1	2.3E+08	2.3E+08	1165	2.3E+08	19	9.15219	4.54307
Nc-1-IP_pe	chr1	2.3E+08	2.3E+08	267	2.3E+08	12	4.93819	3.33035
Nc-1-IP_pe	chr1	2.3E+08	2.3E+08	274	2.3E+08	17	8.20861	4.37862
Nc-1-IP_pe	chr1	2.3E+08	2.3E+08	576	2.3E+08	24	5.77667	2.7276
Nc-1-IP_pe	chr1	2.3E+08	2.3E+08	343	2.3E+08	23	10.8261	4.72183
Nc-1-IP_pe	chr1	2.3E+08	2.3E+08	559	2.3E+08	16	5.82808	3.34463
Nc-1-IP_pe	chr1	2.3E+08	2.3E+08	503	2.3E+08	18	6.15736	3.29676
Nc-1-IP_pe	chr1	2.3E+08	2.3E+08	325	2.3E+08	19	5.90435	3.10381
Nc-1-IP_pe	chr1	2.3E+08	2.3E+08	404	2.3E+08	13	3.07238	2.29378
Nc-1-IP_pe	chr1	2.3E+08	2.3E+08	669	2.3E+08	14	3.86727	2.6027
Nc-1-IP_pe	chr1	2.3E+08	2.3E+08	291	2.3E+08	14	4.60011	2.95114
Nc-1-IP_pe	chr1	2.3E+08	2.3E+08	268	2.3E+08	19	5.90435	3.10381
Nc-1-IP_pe	chr1	2.3E+08	2.3E+08	278	2.3E+08	11	5.49185	3.77641
Nc-1-IP_pe	chr1	2.3E+08	2.3E+08	259	2.3E+08	15	6.19428	3.63445
Nc-1-IP_pe	chr1	2.3E+08	2.3E+08	485	2.3E+08	16	8.39046	4.63193
Nc-1-IP_pe	chr1	2.3E+08	2.3E+08	259	2.3E+08	16	4.25252	2.63824
Nc-1-IP_pe	chr1	2.3E+08	2.3E+08	400	2.3E+08	15	4.7819	2.95039
Nc-1-IP_pe	chr1	2.3E+08	2.3E+08	321	2.3E+08	15	6.5434	3.81197
Nc-1-IP_pe	chr1	2.3E+08	2.3E+08	265	2.3E+08	12	5.77527	3.80164
Nc-1-IP_pe	chr1	2.3E+08	2.3E+08	298	2.3E+08	19	9.05528	4.49651
Nc-1-IP_pe	chr1	2.3E+08	2.3E+08	425	2.3E+08	14	6.06519	3.69269
Nc-1-IP_pe	chr1	2.3E+08	2.3E+08	850	2.3E+08	14	6.05554	3.68761
Nc-1-IP_pe	chr1	2.3E+08	2.3E+08	510	2.3E+08	15	6.69889	3.8921
Nc-1-IP_pe	chr1	2.3E+08	2.3E+08	513	2.3E+08	12	4.51041	3.09723
Nc-1-IP_pe	chr1	2.3E+08	2.3E+08	308	2.3E+08	12	4.24064	2.95299
Nc-1-IP_pe	chr1	2.3E+08	2.3E+08	505	2.3E+08	16	5.82808	3.34463
Nc-1-IP_pe	chr1	2.3E+08	2.3E+08	336	2.3E+08	17	6.47874	3.54137
Nc-1-IP_pe	chr1	2.3E+08	2.3E+08	355	2.3E+08	12	4.70082	3.20033
Nc-1-IP_pe	chr1	2.3E+08	2.3E+08	319	2.3E+08	11	3.78682	2.80131
Nc-1-IP_pe	chr1	2.3E+08	2.3E+08	253	2.3E+08	11	4.81752	3.38105
Nc-1-IP_pe	chr1	2.3E+08	2.3E+08	437	2.3E+08	17	7.02067	3.79543
Nc-1-IP_pe	chr1	2.3E+08	2.3E+08	708	2.3E+08	16	5.28625	3.09457
Nc-1-IP_pe	chr1	2.3E+08	2.3E+08	460	2.3E+08	17	4.44438	2.65333
Nc-1-IP_pe	chr1	2.3E+08	2.3E+08	282	2.3E+08	15	4.40338	2.77622
Nc-1-IP_pe	chr1	2.3E+08	2.3E+08	265	2.3E+08	12	3.33349	2.48364
Nc-1-IP_pe	chr1	2.3E+08	2.3E+08	328	2.3E+08	16	6.33285	3.58457
Nc-1-IP_pe	chr1	2.3E+08	2.3E+08	595	2.3E+08	12	5.08839	3.41347

Nc-1-IP_pe	chr1	2.3E+08	2.3E+08	451	2.3E+08	14	7.34079	4.38651
Nc-1-IP_pe	chr1	2.3E+08	2.3E+08	369	2.3E+08	17	3.11196	2.12137
Nc-1-IP_pe	chr1	2.3E+08	2.3E+08	300	2.3E+08	20	11.0932	5.33258
Nc-1-IP_pe	chr1	2.3E+08	2.3E+08	715	2.3E+08	16	4.25252	2.63824
Nc-1-IP_pe	chr1	2.3E+08	2.3E+08	1013	2.3E+08	19	6.78661	3.47027
Nc-1-IP_pe	chr1	2.3E+08	2.3E+08	400	2.3E+08	17	5.9911	3.31919
Nc-1-IP_pe	chr1	2.3E+08	2.3E+08	756	2.3E+08	15	5.20142	3.14789
Nc-1-IP_pe	chr1	2.3E+08	2.3E+08	834	2.3E+08	17	6.51104	3.5563
Nc-1-IP_pe	chr1	2.3E+08	2.3E+08	541	2.3E+08	16	5.37454	3.13479
Nc-1-IP_pe	chr1	2.3E+08	2.3E+08	373	2.3E+08	15	5.20142	3.14789
Nc-1-IP_pe	chr1	2.3E+08	2.3E+08	583	2.3E+08	13	4.98219	3.24203
Nc-1-IP_pe	chr1	2.3E+08	2.3E+08	562	2.3E+08	14	3.86727	2.6027
Nc-1-IP_pe	chr1	2.3E+08	2.3E+08	945	2.3E+08	19	9.03708	4.48779
Nc-1-IP_pe	chr1	2.3E+08	2.3E+08	1064	2.3E+08	23	10.1636	4.43862
Nc-1-IP_pe	chr1	2.3E+08	2.3E+08	494	2.3E+08	15	3.74763	2.48304
Nc-1-IP_pe	chr1	2.3E+08	2.3E+08	368	2.3E+08	16	5.15964	3.03723
Nc-1-IP_pe	chr1	2.3E+08	2.3E+08	514	2.3E+08	12	3.90597	2.77706
Nc-1-IP_pe	chr1	2.3E+08	2.3E+08	484	2.3E+08	17	5.14803	2.94914
Nc-1-IP_pe	chr1	2.3E+08	2.3E+08	389	2.3E+08	17	6.52418	3.56239
Nc-1-IP_pe	chr1	2.3E+08	2.3E+08	479	2.3E+08	19	5.15822	2.80735
Nc-1-IP_pe	chr1	2.3E+08	2.3E+08	259	2.3E+08	14	4.64659	2.97374
Nc-1-IP_pe	chr1	2.3E+08	2.3E+08	609	2.3E+08	16	5.73763	3.30235
Nc-1-IP_pe	chr1	2.3E+08	2.3E+08	258	2.3E+08	15	6.19428	3.63445
Nc-1-IP_pe	chr1	2.3E+08	2.3E+08	304	2.3E+08	13	4.78908	3.14145
Nc-1-IP_pe	chr1	2.3E+08	2.3E+08	1004	2.3E+08	17	6.47874	3.54137
Nc-1-IP_pe	chr1	2.3E+08	2.3E+08	258	2.3E+08	14	3.86727	2.6027
Nc-1-IP_pe	chr1	2.3E+08	2.3E+08	949	2.3E+08	15	5.22925	3.16116
Nc-1-IP_pe	chr1	2.3E+08	2.3E+08	402	2.3E+08	18	7.7019	3.99245
Nc-1-IP_pe	chr1	2.3E+08	2.3E+08	1023	2.3E+08	18	6.40661	3.40507
Nc-1-IP_pe	chr1	2.3E+08	2.3E+08	344	2.3E+08	14	5.06689	3.18081
Nc-1-IP_pe	chr1	2.3E+08	2.3E+08	486	2.3E+08	19	8.19109	4.09114
Nc-1-IP_pe	chr1	2.3E+08	2.3E+08	442	2.3E+08	13	4.02562	2.7544
Nc-1-IP_pe	chr1	2.3E+08	2.3E+08	274	2.3E+08	17	5.54967	3.12324
Nc-1-IP_pe	chr1	2.3E+08	2.3E+08	782	2.3E+08	19	5.15822	2.80735
Nc-1-IP_pe	chr1	2.3E+08	2.3E+08	253	2.3E+08	15	4.40338	2.77622
Nc-1-IP_pe	chr1	2.3E+08	2.3E+08	467	2.3E+08	15	5.00915	3.05678
Nc-1-IP_pe	chr1	2.3E+08	2.3E+08	285	2.3E+08	19	9.07354	4.50526
Nc-1-IP_pe	chr1	2.3E+08	2.3E+08	313	2.3E+08	14	6.20695	3.76765
Nc-1-IP_pe	chr1	2.3E+08	2.3E+08	278	2.3E+08	12	3.47963	2.55766
Nc-1-IP_pe	chr1	2.3E+08	2.3E+08	652	2.3E+08	12	5.01934	3.37518
Nc-1-IP_pe	chr1	2.3E+08	2.3E+08	280	2.3E+08	20	9.21699	4.428
Nc-1-IP_pe	chr1	2.3E+08	2.3E+08	322	2.3E+08	15	5.58327	3.33181
Nc-1-IP_pe	chr1	2.3E+08	2.3E+08	1501	2.3E+08	23	9.50079	4.16433
Nc-1-IP_pe	chr1	2.3E+08	2.3E+08	343	2.3E+08	16	4.25252	2.63824
Nc-1-IP_pe	chr1	2.3E+08	2.3E+08	435	2.3E+08	18	5.33197	2.94862

Nc-1-IP_pe	chr1	2.3E+08	2.3E+08	1568	2.3E+08	27	14.0512	5.49451
Nc-1-IP_pe	chr1	2.3E+08	2.3E+08	628	2.3E+08	18	5.33197	2.94862
Nc-1-IP_pe	chr1	2.3E+08	2.3E+08	280	2.3E+08	14	3.55308	2.45762
Nc-1-IP_pe	chr1	2.3E+08	2.3E+08	426	2.3E+08	13	3.35769	2.42919
Nc-1-IP_pe	chr1	2.3E+08	2.3E+08	388	2.3E+08	15	5.60797	3.34384
Nc-1-IP_pe	chr1	2.3E+08	2.3E+08	273	2.3E+08	12	3.99583	2.82397
Nc-1-IP_pe	chr1	2.3E+08	2.3E+08	364	2.3E+08	16	4.50821	2.74867
Nc-1-IP_pe	chr1	2.3E+08	2.3E+08	309	2.3E+08	19	7.84735	3.93486
Nc-1-IP_pe	chr1	2.3E+08	2.3E+08	485	2.3E+08	14	3.26761	2.32785
Nc-1-IP_pe	chr1	2.3E+08	2.3E+08	369	2.3E+08	21	10.0289	4.65665
Nc-1-IP_pe	chr1	2.3E+08	2.3E+08	300	2.3E+08	18	7.77965	4.02903
Nc-1-IP_pe	chr1	2.3E+08	2.3E+08	901	2.3E+08	20	6.08336	3.09555
Nc-1-IP_pe	chr1	2.3E+08	2.3E+08	265	2.3E+08	13	3.67377	2.58159
Nc-1-IP_pe	chr1	2.3E+08	2.3E+08	325	2.3E+08	15	3.74763	2.48304
Nc-1-IP_pe	chr1	2.3E+08	2.3E+08	401	2.3E+08	13	3.95529	2.71958
Nc-1-IP_pe	chr1	2.3E+08	2.3E+08	396	2.3E+08	15	4.7819	2.95039
Nc-1-IP_pe	chr1	2.3E+08	2.3E+08	310	2.3E+08	15	5.20142	3.14789
Nc-1-IP_pe	chr1	2.3E+08	2.3E+08	382	2.3E+08	19	7.29128	3.68799
Nc-1-IP_pe	chr1	2.3E+08	2.3E+08	315	2.3E+08	18	8.44412	4.3477
Nc-1-IP_pe	chr1	2.3E+08	2.3E+08	368	2.3E+08	16	5.37454	3.13479
Nc-1-IP_pe	chr1	2.3E+08	2.3E+08	431	2.3E+08	12	3.47963	2.55766
Nc-1-IP_pe	chr1	2.3E+08	2.3E+08	439	2.3E+08	14	5.35794	3.32713
Nc-1-IP_pe	chr1	2.3E+08	2.3E+08	262	2.3E+08	14	4.21449	2.76599
Nc-1-IP_pe	chr1	2.3E+08	2.3E+08	634	2.3E+08	16	4.59224	2.7853
Nc-1-IP_pe	chr1	2.3E+08	2.3E+08	279	2.3E+08	14	4.60011	2.95114
Nc-1-IP_pe	chr1	2.3E+08	2.3E+08	268	2.3E+08	12	4.05718	2.85614
Nc-1-IP_pe	chr1	2.3E+08	2.3E+08	724	2.3E+08	19	7.29128	3.68799
Nc-1-IP_pe	chr1	2.3E+08	2.3E+08	330	2.3E+08	15	6.55872	3.81984
Nc-1-IP_pe	chr1	2.3E+08	2.3E+08	359	2.3E+08	17	6.38155	3.49661
Nc-1-IP_pe	chr1	2.3E+08	2.3E+08	363	2.3E+08	15	4.06016	2.62146
Nc-1-IP_pe	chr1	2.3E+08	2.3E+08	536	2.3E+08	15	6.63607	3.85965
Nc-1-IP_pe	chr1	2.3E+08	2.3E+08	260	2.3E+08	15	6.78905	3.93887
Nc-1-IP_pe	chr1	2.3E+08	2.3E+08	488	2.3E+08	11	3.28475	2.53029
Nc-1-IP_pe	chr1	2.3E+08	2.3E+08	345	2.3E+08	14	6.04069	3.67979
Nc-1-IP_pe	chr1	2.3E+08	2.3E+08	289	2.3E+08	18	7.73136	4.00629
Nc-1-IP_pe	chr1	2.3E+08	2.3E+08	272	2.3E+08	14	5.98179	3.64885
Nc-1-IP_pe	chr1	2.3E+08	2.3E+08	310	2.3E+08	12	5.03305	3.38277
Nc-1-IP_pe	chr1	2.3E+08	2.3E+08	300	2.3E+08	13	6.10995	3.8508
Nc-1-IP_pe	chr1	2.3E+08	2.3E+08	277	2.3E+08	14	6.61011	3.98382
Nc-1-IP_pe	chr1	2.3E+08	2.3E+08	465	2.3E+08	13	5.18582	3.34927
Nc-1-IP_pe	chr1	2.3E+08	2.3E+08	401	2.3E+08	18	6.15736	3.29676
Nc-1-IP_pe	chr1	2.3E+08	2.3E+08	713	2.3E+08	16	4.96462	2.94973
Nc-1-IP_pe	chr1	2.3E+08	2.3E+08	282	2.3E+08	16	6.33285	3.58457
Nc-1-IP_pe	chr1	2.3E+08	2.3E+08	263	2.3E+08	14	6.06519	3.69269
Nc-1-IP_pe	chr1	2.3E+08	2.3E+08	286	2.3E+08	11	4.81752	3.38105

Nc-1-IP_pe	chr1	2.3E+08	2.3E+08	390	2.3E+08	14	5.51541	3.4073
Nc-1-IP_pe	chr1	2.3E+08	2.3E+08	270	2.3E+08	18	5.33197	2.94862
Nc-1-IP_pe	chr1	2.3E+08	2.3E+08	757	2.3E+08	13	5.36853	3.44651
Nc-1-IP_pe	chr1	2.31E+08	2.31E+08	304	2.31E+08	13	5.76252	3.65944
Nc-1-IP_pe	chr1	2.31E+08	2.31E+08	540	2.31E+08	14	6.66104	4.01144
Nc-1-IP_pe	chr1	2.31E+08	2.31E+08	384	2.31E+08	12	4.24064	2.95299
Nc-1-IP_pe	chr1	2.31E+08	2.31E+08	329	2.31E+08	22	11.4578	5.16095
Nc-1-IP_pe	chr1	2.31E+08	2.31E+08	424	2.31E+08	11	4.54404	3.2242
Nc-1-IP_pe	chr1	2.31E+08	2.31E+08	405	2.31E+08	12	4.70523	3.20273
Nc-1-IP_pe	chr1	2.31E+08	2.31E+08	277	2.31E+08	19	5.90435	3.10381
Nc-1-IP_pe	chr1	2.31E+08	2.31E+08	910	2.31E+08	35	12.1779	3.92775
Nc-1-IP_pe	chr1	2.31E+08	2.31E+08	424	2.31E+08	21	9.98999	4.63886
Nc-1-IP_pe	chr1	2.31E+08	2.31E+08	342	2.31E+08	19	7.29128	3.68799
Nc-1-IP_pe	chr1	2.31E+08	2.31E+08	775	2.31E+08	22	8.17351	3.73969
Nc-1-IP_pe	chr1	2.31E+08	2.31E+08	306	2.31E+08	15	4.40338	2.77622
Nc-1-IP_pe	chr1	2.31E+08	2.31E+08	735	2.31E+08	15	5.41431	3.24994
Nc-1-IP_pe	chr1	2.31E+08	2.31E+08	799	2.31E+08	14	4.73128	3.01506
Nc-1-IP_pe	chr1	2.31E+08	2.31E+08	370	2.31E+08	14	5.56442	3.43239
Nc-1-IP_pe	chr1	2.31E+08	2.31E+08	270	2.31E+08	18	7.32895	3.81908
Nc-1-IP_pe	chr1	2.31E+08	2.31E+08	892	2.31E+08	17	5.14803	2.94914
Nc-1-IP_pe	chr1	2.31E+08	2.31E+08	254	2.31E+08	12	3.96559	2.80816
Nc-1-IP_pe	chr1	2.31E+08	2.31E+08	307	2.31E+08	18	6.91333	3.62993
Nc-1-IP_pe	chr1	2.31E+08	2.31E+08	420	2.31E+08	19	4.8268	2.67939
Nc-1-IP_pe	chr1	2.31E+08	2.31E+08	280	2.31E+08	15	4.54518	2.84103
Nc-1-IP_pe	chr1	2.31E+08	2.31E+08	689	2.31E+08	17	3.84884	2.41145
Nc-1-IP_pe	chr1	2.31E+08	2.31E+08	539	2.31E+08	17	5.98924	3.31836
Nc-1-IP_pe	chr1	2.31E+08	2.31E+08	267	2.31E+08	13	3.62212	2.55651
Nc-1-IP_pe	chr1	2.31E+08	2.31E+08	375	2.31E+08	15	5.49794	3.29036
Nc-1-IP_pe	chr1	2.31E+08	2.31E+08	325	2.31E+08	16	4.25252	2.63824
Nc-1-IP_pe	chr1	2.31E+08	2.31E+08	298	2.31E+08	15	4.40338	2.77622
Nc-1-IP_pe	chr1	2.31E+08	2.31E+08	880	2.31E+08	15	3.46204	2.35851
Nc-1-IP_pe	chr1	2.31E+08	2.31E+08	255	2.31E+08	15	5.90272	3.48876
Nc-1-IP_pe	chr1	2.31E+08	2.31E+08	526	2.31E+08	20	5.9768	3.05405
Nc-1-IP_pe	chr1	2.31E+08	2.31E+08	466	2.31E+08	18	4.6358	2.66698
Nc-1-IP_pe	chr1	2.31E+08	2.31E+08	293	2.31E+08	16	6.18743	3.51475
Nc-1-IP_pe	chr1	2.31E+08	2.31E+08	349	2.31E+08	19	9.60791	4.76497
Nc-1-IP_pe	chr1	2.31E+08	2.31E+08	1036	2.31E+08	15	4.40338	2.77622
Nc-1-IP_pe	chr1	2.31E+08	2.31E+08	837	2.31E+08	19	5.23499	2.8373
Nc-1-IP_pe	chr1	2.31E+08	2.31E+08	653	2.31E+08	20	5.01743	2.69071
Nc-1-IP_pe	chr1	2.31E+08	2.31E+08	374	2.31E+08	23	10.8261	4.72183
Nc-1-IP_pe	chr1	2.31E+08	2.31E+08	370	2.31E+08	18	4.32723	2.54542
Nc-1-IP_pe	chr1	2.31E+08	2.31E+08	421	2.31E+08	25	13.8001	5.72315
Nc-1-IP_pe	chr1	2.31E+08	2.31E+08	266	2.31E+08	13	4.86362	3.18015
Nc-1-IP_pe	chr1	2.31E+08	2.31E+08	557	2.31E+08	13	3.21799	2.36264
Nc-1-IP_pe	chr1	2.31E+08	2.31E+08	345	2.31E+08	13	5.86991	3.71823

Nc-1-IP_pe	chr1	2.31E+08	2.31E+08	699	2.31E+08	16	6.33285	3.58457
Nc-1-IP_pe	chr1	2.31E+08	2.31E+08	385	2.31E+08	13	3.67377	2.58159
Nc-1-IP_pe	chr1	2.31E+08	2.31E+08	596	2.31E+08	18	7.32895	3.81908
Nc-1-IP_pe	chr1	2.31E+08	2.31E+08	725	2.31E+08	17	5.9911	3.31919
Nc-1-IP_pe	chr1	2.31E+08	2.31E+08	266	2.31E+08	13	4.41954	2.952
Nc-1-IP_pe	chr1	2.31E+08	2.31E+08	309	2.31E+08	18	4.6358	2.66698
Nc-1-IP_pe	chr1	2.31E+08	2.31E+08	926	2.31E+08	19	8.4638	4.21715
Nc-1-IP_pe	chr1	2.31E+08	2.31E+08	289	2.31E+08	11	3.3117	2.54465
Nc-1-IP_pe	chr1	2.31E+08	2.31E+08	277	2.31E+08	16	4.96462	2.94973
Nc-1-IP_pe	chr1	2.31E+08	2.31E+08	324	2.31E+08	14	5.74019	3.52296
Nc-1-IP_pe	chr1	2.31E+08	2.31E+08	462	2.31E+08	18	7.1522	3.73811
Nc-1-IP_pe	chr1	2.31E+08	2.31E+08	294	2.31E+08	15	6.62049	3.85162
Nc-1-IP_pe	chr1	2.31E+08	2.31E+08	267	2.31E+08	13	4.02562	2.7544
Nc-1-IP_pe	chr1	2.31E+08	2.31E+08	493	2.31E+08	18	9.8513	5.05778
Nc-1-IP_pe	chr1	2.31E+08	2.31E+08	392	2.31E+08	18	7.73136	4.00629
Nc-1-IP_pe	chr1	2.31E+08	2.31E+08	359	2.31E+08	13	3.07238	2.29378
Nc-1-IP_pe	chr1	2.31E+08	2.31E+08	978	2.31E+08	14	4.60011	2.95114
Nc-1-IP_pe	chr1	2.31E+08	2.31E+08	1571	2.31E+08	18	4.04135	2.43445
Nc-1-IP_pe	chr1	2.31E+08	2.31E+08	264	2.31E+08	16	8.09318	4.47392
Nc-1-IP_pe	chr1	2.31E+08	2.31E+08	271	2.31E+08	13	6.47103	4.0531
Nc-1-IP_pe	chr1	2.31E+08	2.31E+08	333	2.31E+08	13	3.07238	2.29378
Nc-1-IP_pe	chr1	2.31E+08	2.31E+08	378	2.31E+08	13	4.41954	2.952
Nc-1-IP_pe	chr1	2.31E+08	2.31E+08	271	2.31E+08	14	6.44441	3.89444
Nc-1-IP_pe	chr1	2.31E+08	2.31E+08	857	2.31E+08	19	10.3023	5.1123
Nc-1-IP_pe	chr1	2.31E+08	2.31E+08	279	2.31E+08	16	6.44386	3.63825
Nc-1-IP_pe	chr1	2.31E+08	2.31E+08	292	2.31E+08	14	4.91783	3.1068
Nc-1-IP_pe	chr1	2.31E+08	2.31E+08	430	2.31E+08	15	4.80057	2.95908
Nc-1-IP_pe	chr1	2.31E+08	2.31E+08	430	2.31E+08	15	5.66913	3.37372
Nc-1-IP_pe	chr1	2.31E+08	2.31E+08	466	2.31E+08	17	6.92131	3.74829
Nc-1-IP_pe	chr1	2.31E+08	2.31E+08	684	2.31E+08	13	4.5945	3.0412
Nc-1-IP_pe	chr1	2.31E+08	2.31E+08	260	2.31E+08	14	5.03085	3.16286
Nc-1-IP_pe	chr1	2.31E+08	2.31E+08	293	2.31E+08	21	7.58657	3.60451
Nc-1-IP_pe	chr1	2.31E+08	2.31E+08	360	2.31E+08	13	4.49561	2.99068
Nc-1-IP_pe	chr1	2.31E+08	2.31E+08	755	2.31E+08	17	7.21469	3.88822
Nc-1-IP_pe	chr1	2.31E+08	2.31E+08	770	2.31E+08	16	7.20904	4.01714
Nc-1-IP_pe	chr1	2.31E+08	2.31E+08	387	2.31E+08	15	6.39297	3.73508
Nc-1-IP_pe	chr1	2.31E+08	2.31E+08	757	2.31E+08	21	11.0504	5.13573
Nc-1-IP_pe	chr1	2.31E+08	2.31E+08	338	2.31E+08	13	3.35769	2.42919
Nc-1-IP_pe	chr1	2.31E+08	2.31E+08	495	2.31E+08	22	5.28851	2.67305
Nc-1-IP_pe	chr1	2.31E+08	2.31E+08	279	2.31E+08	22	12.3191	5.57148
Nc-1-IP_pe	chr1	2.31E+08	2.31E+08	290	2.31E+08	13	4.92076	3.20992
Nc-1-IP_pe	chr1	2.31E+08	2.31E+08	298	2.31E+08	13	4.56123	3.02417
Nc-1-IP_pe	chr1	2.31E+08	2.31E+08	329	2.31E+08	14	3.86727	2.6027
Nc-1-IP_pe	chr1	2.31E+08	2.31E+08	589	2.31E+08	15	4.63166	2.88081
Nc-1-IP_pe	chr1	2.31E+08	2.31E+08	341	2.31E+08	16	6.33285	3.58457

Nc-1-IP_pe	chr1	2.31E+08	2.31E+08	496	2.31E+08	18	5.72638	3.11298
Nc-1-IP_pe	chr1	2.31E+08	2.31E+08	492	2.31E+08	25	5.69058	2.64923
Nc-1-IP_pe	chr1	2.31E+08	2.31E+08	368	2.31E+08	22	3.82136	2.18497
Nc-1-IP_pe	chr1	2.31E+08	2.31E+08	263	2.31E+08	15	7.46994	4.29894
Nc-1-IP_pe	chr1	2.31E+08	2.31E+08	284	2.31E+08	21	9.29881	4.32834
Nc-1-IP_pe	chr1	2.31E+08	2.31E+08	311	2.31E+08	17	8.56555	4.56077
Nc-1-IP_pe	chr1	2.31E+08	2.31E+08	282	2.31E+08	20	6.07662	3.09292
Nc-1-IP_pe	chr1	2.31E+08	2.31E+08	325	2.31E+08	18	4.04135	2.43445
Nc-1-IP_pe	chr1	2.31E+08	2.31E+08	489	2.31E+08	16	3.39261	2.27748
Nc-1-IP_pe	chr1	2.31E+08	2.31E+08	421	2.31E+08	10	3.63115	2.80557
Nc-1-IP_pe	chr1	2.31E+08	2.31E+08	287	2.31E+08	14	5.03085	3.16286
Nc-1-IP_pe	chr1	2.31E+08	2.31E+08	467	2.31E+08	13	4.78908	3.14145
Nc-1-IP_pe	chr1	2.32E+08	2.32E+08	482	2.32E+08	14	6.06519	3.69269
Nc-1-IP_pe	chr1	2.32E+08	2.32E+08	551	2.32E+08	21	6.45254	3.15977
Nc-1-IP_pe	chr1	2.32E+08	2.32E+08	347	2.32E+08	10	3.71867	2.85592
Nc-1-IP_pe	chr1	2.32E+08	2.32E+08	659	2.32E+08	16	6.80394	3.81462
Nc-1-IP_pe	chr1	2.32E+08	2.32E+08	667	2.32E+08	20	8.29312	4.01203
Nc-1-IP_pe	chr1	2.32E+08	2.32E+08	537	2.32E+08	31	12.8059	4.49176
Nc-1-IP_pe	chr1	2.32E+08	2.32E+08	261	2.32E+08	23	7.01947	3.21526
Nc-1-IP_pe	chr1	2.32E+08	2.32E+08	268	2.32E+08	20	8.69811	4.19196
Nc-1-IP_pe	chr1	2.32E+08	2.32E+08	574	2.32E+08	17	5.54967	3.12324
Nc-1-IP_pe	chr1	2.32E+08	2.32E+08	258	2.32E+08	14	5.92369	3.61842
Nc-1-IP_pe	chr1	2.32E+08	2.32E+08	410	2.32E+08	16	3.39261	2.27748
Nc-1-IP_pe	chr1	2.32E+08	2.32E+08	404	2.32E+08	14	4.60011	2.95114
Nc-1-IP_pe	chr1	2.32E+08	2.32E+08	859	2.32E+08	16	7.44163	4.13536
Nc-1-IP_pe	chr1	2.32E+08	2.32E+08	317	2.32E+08	16	4.42092	2.71079
Nc-1-IP_pe	chr1	2.32E+08	2.32E+08	270	2.32E+08	16	5.37454	3.13479
Nc-1-IP_pe	chr1	2.32E+08	2.32E+08	312	2.32E+08	15	6.19428	3.63445
Nc-1-IP_pe	chr1	2.32E+08	2.32E+08	368	2.32E+08	18	7.32895	3.81908
Nc-1-IP_pe	chr1	2.32E+08	2.32E+08	256	2.32E+08	16	5.37454	3.13479
Nc-1-IP_pe	chr1	2.32E+08	2.32E+08	395	2.32E+08	19	9.15219	4.54307
Nc-1-IP_pe	chr1	2.32E+08	2.32E+08	276	2.32E+08	15	5.63281	3.35597
Nc-1-IP_pe	chr1	2.32E+08	2.32E+08	279	2.32E+08	16	6.8987	3.86161
Nc-1-IP_pe	chr1	2.32E+08	2.32E+08	293	2.32E+08	23	10.1326	4.42559
Nc-1-IP_pe	chr1	2.32E+08	2.32E+08	329	2.32E+08	16	6.8987	3.86161
Nc-1-IP_pe	chr1	2.32E+08	2.32E+08	305	2.32E+08	14	4.60011	2.95114
Nc-1-IP_pe	chr1	2.32E+08	2.32E+08	280	2.32E+08	9	3.70468	2.94668
Nc-1-IP_pe	chr1	2.32E+08	2.32E+08	288	2.32E+08	15	8.26005	4.73146
Nc-1-IP_pe	chr1	2.32E+08	2.32E+08	274	2.32E+08	9	3.81807	3.01579
Nc-1-IP_pe	chr1	2.32E+08	2.32E+08	638	2.32E+08	18	9.09632	4.67097
Nc-1-IP_pe	chr1	2.32E+08	2.32E+08	416	2.32E+08	11	4.06403	2.95415
Nc-1-IP_pe	chr1	2.32E+08	2.32E+08	358	2.32E+08	15	4.7819	2.95039
Nc-1-IP_pe	chr1	2.32E+08	2.32E+08	266	2.32E+08	12	3.47963	2.55766
Nc-1-IP_pe	chr1	2.32E+08	2.32E+08	345	2.32E+08	15	2.95965	2.14351
Nc-1-IP_pe	chr1	2.32E+08	2.32E+08	510	2.32E+08	18	10.1486	5.21369

Nc-1-IP_pe	chr1	2.32E+08	2.32E+08	294	2.32E+08	12	5.15877	3.45264
Nc-1-IP_pe	chr1	2.32E+08	2.32E+08	303	2.32E+08	18	6.63038	3.50359
Nc-1-IP_pe	chr1	2.32E+08	2.32E+08	295	2.32E+08	14	5.03085	3.16286
Nc-1-IP_pe	chr1	2.32E+08	2.32E+08	670	2.32E+08	18	6.78184	3.57098
Nc-1-IP_pe	chr1	2.32E+08	2.32E+08	428	2.32E+08	12	3.68745	2.66395
Nc-1-IP_pe	chr1	2.32E+08	2.32E+08	566	2.32E+08	19	6.78661	3.47027
Nc-1-IP_pe	chr1	2.32E+08	2.32E+08	541	2.32E+08	16	7.44163	4.13536
Nc-1-IP_pe	chr1	2.32E+08	2.32E+08	407	2.32E+08	16	6.18743	3.51475
Nc-1-IP_pe	chr1	2.32E+08	2.32E+08	328	2.32E+08	15	5.66913	3.37372
Nc-1-IP_pe	chr1	2.32E+08	2.32E+08	320	2.32E+08	15	5.58327	3.33181
Nc-1-IP_pe	chr1	2.32E+08	2.32E+08	395	2.32E+08	20	7.97282	3.87239
Nc-1-IP_pe	chr1	2.32E+08	2.32E+08	516	2.32E+08	12	3.71508	2.67818
Nc-1-IP_pe	chr1	2.32E+08	2.32E+08	365	2.32E+08	16	3.39261	2.27748
Nc-1-IP_pe	chr1	2.32E+08	2.32E+08	458	2.32E+08	20	6.08336	3.09555
Nc-1-IP_pe	chr1	2.32E+08	2.32E+08	518	2.32E+08	14	3.55308	2.45762
Nc-1-IP_pe	chr1	2.32E+08	2.32E+08	456	2.32E+08	13	4.51735	3.00176
Nc-1-IP_pe	chr1	2.32E+08	2.32E+08	390	2.32E+08	18	4.04135	2.43445
Nc-1-IP_pe	chr1	2.32E+08	2.32E+08	1482	2.32E+08	21	7.98609	3.76767
Nc-1-IP_pe	chr1	2.32E+08	2.32E+08	336	2.32E+08	19	7.73663	3.88512
Nc-1-IP_pe	chr1	2.32E+08	2.32E+08	391	2.32E+08	14	4.60011	2.95114
Nc-1-IP_pe	chr1	2.32E+08	2.32E+08	270	2.32E+08	16	3.3803	2.27242
Nc-1-IP_pe	chr1	2.32E+08	2.32E+08	518	2.32E+08	15	6.19428	3.63445
Nc-1-IP_pe	chr1	2.32E+08	2.32E+08	326	2.32E+08	18	7.1522	3.73811
Nc-1-IP_pe	chr1	2.32E+08	2.32E+08	328	2.32E+08	16	3.65575	2.38624
Nc-1-IP_pe	chr1	2.32E+08	2.32E+08	278	2.32E+08	18	9.1091	4.6774
Nc-1-IP_pe	chr1	2.33E+08	2.33E+08	334	2.33E+08	20	6.49719	3.259
Nc-1-IP_pe	chr1	2.33E+08	2.33E+08	579	2.33E+08	12	3.58823	2.61305
Nc-1-IP_pe	chr1	2.33E+08	2.33E+08	286	2.33E+08	15	6.40779	3.74263
Nc-1-IP_pe	chr1	2.33E+08	2.33E+08	488	2.33E+08	12	4.46339	3.07193
Nc-1-IP_pe	chr1	2.33E+08	2.33E+08	405	2.33E+08	17	5.54967	3.12324
Nc-1-IP_pe	chr1	2.33E+08	2.33E+08	299	2.33E+08	16	3.1497	2.1782
Nc-1-IP_pe	chr1	2.33E+08	2.33E+08	316	2.33E+08	15	5.66913	3.37372
Nc-1-IP_pe	chr1	2.33E+08	2.33E+08	376	2.33E+08	22	13.2637	6.03922
Nc-1-IP_pe	chr1	2.33E+08	2.33E+08	304	2.33E+08	19	3.26827	2.10398
Nc-1-IP_pe	chr1	2.33E+08	2.33E+08	371	2.33E+08	14	3.33731	2.35937
Nc-1-IP_pe	chr1	2.33E+08	2.33E+08	304	2.33E+08	16	4.59224	2.7853
Nc-1-IP_pe	chr1	2.33E+08	2.33E+08	541	2.33E+08	18	6.63038	3.50359
Nc-1-IP_pe	chr1	2.33E+08	2.33E+08	440	2.33E+08	13	3.67377	2.58159
Nc-1-IP_pe	chr1	2.33E+08	2.33E+08	772	2.33E+08	17	6.47874	3.54137
Nc-1-IP_pe	chr1	2.33E+08	2.33E+08	574	2.33E+08	15	4.60262	2.86743
Nc-1-IP_pe	chr1	2.33E+08	2.33E+08	254	2.33E+08	19	6.78661	3.47027
Nc-1-IP_pe	chr1	2.33E+08	2.33E+08	262	2.33E+08	20	9.21699	4.428
Nc-1-IP_pe	chr1	2.33E+08	2.33E+08	453	2.33E+08	21	5.5349	2.81884
Nc-1-IP_pe	chr1	2.33E+08	2.33E+08	402	2.33E+08	12	3.47963	2.55766
Nc-1-IP_pe	chr1	2.33E+08	2.33E+08	313	2.33E+08	16	5.82808	3.34463

Nc-1-IP_pe	chr1	2.33E+08	2.33E+08	260	2.33E+08	20	5.70098	2.94771
Nc-1-IP_pe	chr1	2.33E+08	2.33E+08	310	2.33E+08	17	3.84884	2.41145
Nc-1-IP_pe	chr1	2.33E+08	2.33E+08	263	2.33E+08	12	4.97854	3.35262
Nc-1-IP_pe	chr1	2.33E+08	2.33E+08	570	2.33E+08	11	3.64839	2.72584
Nc-1-IP_pe	chr1	2.33E+08	2.33E+08	302	2.33E+08	21	6.66991	3.24296
Nc-1-IP_pe	chr1	2.33E+08	2.33E+08	277	2.33E+08	18	6.63038	3.50359
Nc-1-IP_pe	chr1	2.33E+08	2.33E+08	820	2.33E+08	17	5.54967	3.12324
Nc-1-IP_pe	chr1	2.33E+08	2.33E+08	397	2.33E+08	22	11.0016	4.9498
Nc-1-IP_pe	chr1	2.33E+08	2.33E+08	1049	2.33E+08	23	5.28318	2.6185
Nc-1-IP_pe	chr1	2.33E+08	2.33E+08	730	2.33E+08	16	4.25252	2.63824
Nc-1-IP_pe	chr1	2.33E+08	2.33E+08	701	2.33E+08	17	5.9911	3.31919
Nc-1-IP_pe	chr1	2.33E+08	2.33E+08	558	2.33E+08	14	4.52309	2.91384
Nc-1-IP_pe	chr1	2.33E+08	2.33E+08	273	2.33E+08	17	4.44438	2.65333
Nc-1-IP_pe	chr1	2.33E+08	2.33E+08	929	2.33E+08	15	3.74763	2.48304
Nc-1-IP_pe	chr1	2.33E+08	2.33E+08	328	2.33E+08	14	7.44075	4.44264
Nc-1-IP_pe	chr1	2.33E+08	2.33E+08	409	2.33E+08	15	5.20142	3.14789
Nc-1-IP_pe	chr1	2.33E+08	2.33E+08	317	2.33E+08	16	7.53857	4.18505
Nc-1-IP_pe	chr1	2.33E+08	2.33E+08	295	2.33E+08	20	11.7262	5.65522
Nc-1-IP_pe	chr1	2.33E+08	2.33E+08	307	2.33E+08	20	6.08336	3.09555
Nc-1-IP_pe	chr1	2.33E+08	2.33E+08	601	2.33E+08	18	6.63038	3.50359
Nc-1-IP_pe	chr1	2.33E+08	2.33E+08	255	2.33E+08	13	5.03208	3.26819
Nc-1-IP_pe	chr1	2.33E+08	2.33E+08	374	2.33E+08	20	6.49719	3.259
Nc-1-IP_pe	chr1	2.33E+08	2.33E+08	313	2.33E+08	12	5.15877	3.45264
Nc-1-IP_pe	chr1	2.33E+08	2.33E+08	290	2.33E+08	12	5.23082	3.49289
Nc-1-IP_pe	chr1	2.33E+08	2.33E+08	259	2.33E+08	15	5.66913	3.37372
Nc-1-IP_pe	chr1	2.33E+08	2.33E+08	298	2.33E+08	13	7.7352	4.78626
Nc-1-IP_pe	chr1	2.33E+08	2.33E+08	314	2.33E+08	20	12.7789	6.21004
Nc-1-IP_pe	chr1	2.33E+08	2.33E+08	281	2.33E+08	18	7.04825	3.69086
Nc-1-IP_pe	chr1	2.33E+08	2.33E+08	401	2.33E+08	19	6.32622	3.27682
Nc-1-IP_pe	chr1	2.33E+08	2.33E+08	299	2.33E+08	17	9.20132	4.89288
Nc-1-IP_pe	chr1	2.33E+08	2.33E+08	268	2.33E+08	12	4.70082	3.20033
Nc-1-IP_pe	chr1	2.33E+08	2.33E+08	416	2.33E+08	26	5.85241	2.65063
Nc-1-IP_pe	chr1	2.33E+08	2.33E+08	287	2.33E+08	16	7.51009	4.17043
Nc-1-IP_pe	chr1	2.33E+08	2.33E+08	357	2.33E+08	11	4.94832	3.45679
Nc-1-IP_pe	chr1	2.33E+08	2.33E+08	518	2.33E+08	12	4.75569	3.23024
Nc-1-IP_pe	chr1	2.33E+08	2.33E+08	329	2.33E+08	21	8.6741	4.05679
Nc-1-IP_pe	chr1	2.33E+08	2.33E+08	1121	2.33E+08	26	9.34034	3.78992
Nc-1-IP_pe	chr1	2.33E+08	2.33E+08	294	2.33E+08	19	10.1269	5.02353
Nc-1-IP_pe	chr1	2.33E+08	2.33E+08	390	2.33E+08	15	6.77862	3.93345
Nc-1-IP_pe	chr1	2.33E+08	2.33E+08	547	2.33E+08	15	6.19428	3.63445
Nc-1-IP_pe	chr1	2.33E+08	2.33E+08	320	2.33E+08	14	3.55754	2.45966
Nc-1-IP_pe	chr1	2.33E+08	2.33E+08	691	2.33E+08	21	5.88587	2.94733
Nc-1-IP_pe	chr1	2.34E+08	2.34E+08	372	2.34E+08	16	4.59224	2.7853
Nc-1-IP_pe	chr1	2.34E+08	2.34E+08	345	2.34E+08	12	4.44012	3.05944
Nc-1-IP_pe	chr1	2.34E+08	2.34E+08	1431	2.34E+08	20	7.85753	3.8227

Nc-1-IP_pe	chr1	2.34E+08	2.34E+08	297	2.34E+08	17	9.09431	4.8363
Nc-1-IP_pe	chr1	2.34E+08	2.34E+08	402	2.34E+08	18	8.62313	4.4354
Nc-1-IP_pe	chr1	2.34E+08	2.34E+08	328	2.34E+08	20	8.4457	4.07937
Nc-1-IP_pe	chr1	2.34E+08	2.34E+08	273	2.34E+08	11	3.40056	2.59215
Nc-1-IP_pe	chr1	2.34E+08	2.34E+08	314	2.34E+08	11	5.79772	3.95946
Nc-1-IP_pe	chr1	2.34E+08	2.34E+08	352	2.34E+08	17	9.94011	5.29055
Nc-1-IP_pe	chr1	2.34E+08	2.34E+08	527	2.34E+08	15	6.19428	3.63445
Nc-1-IP_pe	chr1	2.34E+08	2.34E+08	968	2.34E+08	27	9.006	3.58762
Nc-1-IP_pe	chr1	2.34E+08	2.34E+08	549	2.34E+08	13	4.56123	3.02417
Nc-1-IP_pe	chr1	2.34E+08	2.34E+08	254	2.34E+08	21	7.10914	3.414
Nc-1-IP_pe	chr1	2.34E+08	2.34E+08	262	2.34E+08	15	6.19428	3.63445
Nc-1-IP_pe	chr1	2.34E+08	2.34E+08	504	2.34E+08	14	3.86727	2.6027
Nc-1-IP_pe	chr1	2.34E+08	2.34E+08	267	2.34E+08	29	10.2881	3.84387
Nc-1-IP_pe	chr1	2.34E+08	2.34E+08	282	2.34E+08	12	5.50092	3.64505
Nc-1-IP_pe	chr1	2.34E+08	2.34E+08	325	2.34E+08	17	5.6711	3.17666
Nc-1-IP_pe	chr1	2.34E+08	2.34E+08	286	2.34E+08	13	5.73236	3.64298
Nc-1-IP_pe	chr1	2.34E+08	2.34E+08	706	2.34E+08	17	6.25544	3.43888
Nc-1-IP_pe	chr1	2.34E+08	2.34E+08	395	2.34E+08	13	4.04902	2.76601
Nc-1-IP_pe	chr1	2.34E+08	2.34E+08	564	2.34E+08	13	6.98489	4.34671
Nc-1-IP_pe	chr1	2.34E+08	2.34E+08	564	2.34E+08	15	3.74763	2.48304
Nc-1-IP_pe	chr1	2.34E+08	2.34E+08	346	2.34E+08	19	5.90435	3.10381
Nc-1-IP_pe	chr1	2.34E+08	2.34E+08	1051	2.34E+08	20	6.72028	3.34863
Nc-1-IP_pe	chr1	2.34E+08	2.34E+08	265	2.34E+08	16	6.37501	3.60492
Nc-1-IP_pe	chr1	2.34E+08	2.34E+08	267	2.34E+08	14	3.26761	2.32785
Nc-1-IP_pe	chr1	2.34E+08	2.34E+08	278	2.34E+08	14	4.21449	2.76599
Nc-1-IP_pe	chr1	2.34E+08	2.34E+08	267	2.34E+08	15	7.56326	4.34922
Nc-1-IP_pe	chr1	2.34E+08	2.34E+08	421	2.34E+08	17	8.7831	4.67333
Nc-1-IP_pe	chr1	2.34E+08	2.34E+08	257	2.34E+08	17	7.6273	4.08876
Nc-1-IP_pe	chr1	2.34E+08	2.34E+08	297	2.34E+08	17	3.33947	2.20998
Nc-1-IP_pe	chr1	2.34E+08	2.34E+08	492	2.34E+08	18	7.74842	4.01431
Nc-1-IP_pe	chr1	2.34E+08	2.34E+08	774	2.34E+08	20	9.09729	4.373
Nc-1-IP_pe	chr1	2.34E+08	2.34E+08	436	2.34E+08	17	8.43136	4.49193
Nc-1-IP_pe	chr1	2.34E+08	2.34E+08	271	2.34E+08	13	5.54163	3.53952
Nc-1-IP_pe	chr1	2.34E+08	2.34E+08	382	2.34E+08	18	10.2908	5.28894
Nc-1-IP_pe	chr1	2.34E+08	2.34E+08	351	2.34E+08	21	5.2077	2.70109
Nc-1-IP_pe	chr1	2.34E+08	2.34E+08	284	2.34E+08	14	5.43414	3.36584
Nc-1-IP_pe	chr1	2.34E+08	2.34E+08	283	2.34E+08	15	5.81087	3.44334
Nc-1-IP_pe	chr1	2.34E+08	2.34E+08	658	2.34E+08	17	5.15375	2.9516
Nc-1-IP_pe	chr1	2.34E+08	2.34E+08	254	2.34E+08	16	4.96462	2.94973
Nc-1-IP_pe	chr1	2.34E+08	2.34E+08	457	2.34E+08	16	5.85814	3.35873
Nc-1-IP_pe	chr1	2.34E+08	2.34E+08	253	2.34E+08	14	5.43414	3.36584
Nc-1-IP_pe	chr1	2.34E+08	2.34E+08	1858	2.34E+08	27	8.55392	3.43775
Nc-1-IP_pe	chr1	2.34E+08	2.34E+08	343	2.34E+08	21	8.6741	4.05679
Nc-1-IP_pe	chr1	2.34E+08	2.34E+08	757	2.34E+08	24	8.53898	3.68518
Nc-1-IP_pe	chr1	2.34E+08	2.34E+08	585	2.34E+08	13	4.80086	3.14755

Nc-1-IP_pe	chr1	2.34E+08	2.34E+08	266	2.34E+08	16	5.37454	3.13479
Nc-1-IP_pe	chr1	2.34E+08	2.34E+08	300	2.34E+08	14	5.838	3.57373
Nc-1-IP_pe	chr1	2.34E+08	2.34E+08	734	2.34E+08	20	5.34663	2.81336
Nc-1-IP_pe	chr1	2.34E+08	2.34E+08	447	2.34E+08	13	3.95529	2.71958
Nc-1-IP_pe	chr1	2.34E+08	2.34E+08	788	2.34E+08	16	5.37454	3.13479
Nc-1-IP_pe	chr1	2.34E+08	2.34E+08	782	2.34E+08	20	7.97282	3.87239
Nc-1-IP_pe	chr1	2.34E+08	2.34E+08	310	2.34E+08	14	4.32616	2.81919
Nc-1-IP_pe	chr1	2.34E+08	2.34E+08	502	2.34E+08	23	9.83409	4.30114
Nc-1-IP_pe	chr1	2.34E+08	2.34E+08	284	2.34E+08	20	8.56319	4.1316
Nc-1-IP_pe	chr1	2.34E+08	2.34E+08	337	2.34E+08	18	8.37884	4.31591
Nc-1-IP_pe	chr1	2.34E+08	2.34E+08	835	2.34E+08	14	7.34079	4.38651
Nc-1-IP_pe	chr1	2.34E+08	2.34E+08	255	2.34E+08	15	5.1177	3.10809
Nc-1-IP_pe	chr1	2.34E+08	2.34E+08	383	2.34E+08	20	5.70098	2.94771
Nc-1-IP_pe	chr1	2.34E+08	2.34E+08	493	2.34E+08	16	5.82808	3.34463
Nc-1-IP_pe	chr1	2.34E+08	2.34E+08	774	2.34E+08	39	19.8689	5.89629
Nc-1-IP_pe	chr1	2.34E+08	2.34E+08	322	2.34E+08	18	6.52861	3.45863
Nc-1-IP_pe	chr1	2.34E+08	2.34E+08	391	2.34E+08	14	3.96259	2.64722
Nc-1-IP_pe	chr1	2.34E+08	2.34E+08	416	2.34E+08	14	6.59326	3.9747
Nc-1-IP_pe	chr1	2.34E+08	2.34E+08	347	2.34E+08	14	5.51541	3.4073
Nc-1-IP_pe	chr1	2.34E+08	2.34E+08	652	2.34E+08	13	4.90859	3.20357
Nc-1-IP_pe	chr1	2.34E+08	2.34E+08	732	2.34E+08	21	9.50999	4.42209
Nc-1-IP_pe	chr1	2.34E+08	2.34E+08	537	2.34E+08	23	6.25615	2.94664
Nc-1-IP_pe	chr1	2.34E+08	2.34E+08	327	2.34E+08	16	6.95583	3.89005
Nc-1-IP_pe	chr1	2.34E+08	2.34E+08	492	2.34E+08	17	6.51104	3.5563
Nc-1-IP_pe	chr1	2.35E+08	2.35E+08	446	2.35E+08	23	7.4429	3.36882
Nc-1-IP_pe	chr1	2.35E+08	2.35E+08	435	2.35E+08	13	4.29558	2.88934
Nc-1-IP_pe	chr1	2.35E+08	2.35E+08	392	2.35E+08	17	5.14803	2.94914
Nc-1-IP_pe	chr1	2.35E+08	2.35E+08	357	2.35E+08	14	3.26761	2.32785
Nc-1-IP_pe	chr1	2.35E+08	2.35E+08	271	2.35E+08	13	3.86418	2.67468
Nc-1-IP_pe	chr1	2.35E+08	2.35E+08	444	2.35E+08	16	3.65575	2.38624
Nc-1-IP_pe	chr1	2.35E+08	2.35E+08	563	2.35E+08	16	3.94146	2.50592
Nc-1-IP_pe	chr1	2.35E+08	2.35E+08	299	2.35E+08	14	3.86727	2.6027
Nc-1-IP_pe	chr1	2.35E+08	2.35E+08	375	2.35E+08	19	6.78661	3.47027
Nc-1-IP_pe	chr1	2.35E+08	2.35E+08	411	2.35E+08	18	5.33197	2.94862
Nc-1-IP_pe	chr1	2.35E+08	2.35E+08	570	2.35E+08	14	3.55308	2.45762
Nc-1-IP_pe	chr1	2.35E+08	2.35E+08	254	2.35E+08	16	7.324	4.07539
Nc-1-IP_pe	chr1	2.35E+08	2.35E+08	256	2.35E+08	15	3.46204	2.35851
Nc-1-IP_pe	chr1	2.35E+08	2.35E+08	329	2.35E+08	18	9.33764	4.79316
Nc-1-IP_pe	chr1	2.35E+08	2.35E+08	331	2.35E+08	11	3.69531	2.75136
Nc-1-IP_pe	chr1	2.35E+08	2.35E+08	381	2.35E+08	19	5.90435	3.10381
Nc-1-IP_pe	chr1	2.35E+08	2.35E+08	423	2.35E+08	21	6.66991	3.24296
Nc-1-IP_pe	chr1	2.35E+08	2.35E+08	306	2.35E+08	16	6.80394	3.81462
Nc-1-IP_pe	chr1	2.35E+08	2.35E+08	292	2.35E+08	23	8.26098	3.67508
Nc-1-IP_pe	chr1	2.35E+08	2.35E+08	259	2.35E+08	21	4.90204	2.59278
Nc-1-IP_pe	chr1	2.35E+08	2.35E+08	428	2.35E+08	17	6.47874	3.54137

Nc-1-IP_pe	chr1	2.35E+08	2.35E+08	280	2.35E+08	15	5.00915	3.05678
Nc-1-IP_pe	chr1	2.35E+08	2.35E+08	414	2.35E+08	18	6.91333	3.62993
Nc-1-IP_pe	chr1	2.35E+08	2.35E+08	317	2.35E+08	20	3.90729	2.29119
Nc-1-IP_pe	chr1	2.35E+08	2.35E+08	287	2.35E+08	17	4.44438	2.65333
Nc-1-IP_pe	chr1	2.35E+08	2.35E+08	464	2.35E+08	20	4.42487	2.47493
Nc-1-IP_pe	chr1	2.35E+08	2.35E+08	1133	2.35E+08	18	5.49768	3.01724
Nc-1-IP_pe	chr1	2.35E+08	2.35E+08	283	2.35E+08	15	3.68723	2.45656
Nc-1-IP_pe	chr1	2.35E+08	2.35E+08	278	2.35E+08	18	5.28388	2.92882
Nc-1-IP_pe	chr1	2.35E+08	2.35E+08	311	2.35E+08	24	10.8883	4.60999
Nc-1-IP_pe	chr1	2.35E+08	2.35E+08	409	2.35E+08	16	6.58466	3.7068
Nc-1-IP_pe	chr1	2.35E+08	2.35E+08	256	2.35E+08	15	4.7819	2.95039
Nc-1-IP_pe	chr1	2.35E+08	2.35E+08	834	2.35E+08	14	4.60011	2.95114
Nc-1-IP_pe	chr1	2.35E+08	2.35E+08	289	2.35E+08	34	7.12622	2.65467
Nc-1-IP_pe	chr1	2.35E+08	2.35E+08	543	2.35E+08	43	10.2916	3.01179
Nc-1-IP_pe	chr1	2.35E+08	2.35E+08	291	2.35E+08	17	5.14803	2.94914
Nc-1-IP_pe	chr1	2.35E+08	2.35E+08	867	2.35E+08	25	6.98838	3.0642
Nc-1-IP_pe	chr1	2.35E+08	2.35E+08	468	2.35E+08	19	4.8268	2.67939
Nc-1-IP_pe	chr1	2.35E+08	2.35E+08	614	2.35E+08	17	4.44438	2.65333
Nc-1-IP_pe	chr1	2.35E+08	2.35E+08	746	2.35E+08	19	3.26827	2.10398
Nc-1-IP_pe	chr1	2.35E+08	2.35E+08	709	2.35E+08	18	3.52899	2.23922
Nc-1-IP_pe	chr1	2.35E+08	2.35E+08	794	2.35E+08	15	4.40338	2.77622
Nc-1-IP_pe	chr1	2.35E+08	2.35E+08	341	2.35E+08	18	7.73136	4.00629
Nc-1-IP_pe	chr1	2.35E+08	2.35E+08	526	2.35E+08	15	5.62037	3.34989
Nc-1-IP_pe	chr1	2.35E+08	2.35E+08	256	2.35E+08	15	5.4619	3.27292
Nc-1-IP_pe	chr1	2.35E+08	2.35E+08	683	2.35E+08	15	4.80057	2.95908
Nc-1-IP_pe	chr1	2.35E+08	2.35E+08	795	2.35E+08	22	9.11077	4.12193
Nc-1-IP_pe	chr1	2.35E+08	2.35E+08	380	2.35E+08	16	3.39261	2.27748
Nc-1-IP_pe	chr1	2.35E+08	2.35E+08	288	2.35E+08	15	4.23553	2.70016
Nc-1-IP_pe	chr1	2.35E+08	2.35E+08	346	2.35E+08	14	3.85234	2.59575
Nc-1-IP_pe	chr1	2.35E+08	2.35E+08	351	2.35E+08	22	5.39765	2.71064
Nc-1-IP_pe	chr1	2.35E+08	2.35E+08	289	2.35E+08	21	7.22656	3.4604
Nc-1-IP_pe	chr1	2.35E+08	2.35E+08	311	2.35E+08	16	5.37454	3.13479
Nc-1-IP_pe	chr1	2.35E+08	2.35E+08	715	2.35E+08	17	5.89608	3.2766
Nc-1-IP_pe	chr1	2.35E+08	2.35E+08	978	2.35E+08	15	3.74763	2.48304
Nc-1-IP_pe	chr1	2.35E+08	2.35E+08	394	2.35E+08	18	6.65364	3.51391
Nc-1-IP_pe	chr1	2.35E+08	2.35E+08	282	2.35E+08	16	5.60875	3.24248
Nc-1-IP_pe	chr1	2.35E+08	2.35E+08	478	2.35E+08	19	4.23334	2.45554
Nc-1-IP_pe	chr1	2.35E+08	2.35E+08	453	2.35E+08	14	4.862	3.07924
Nc-1-IP_pe	chr1	2.35E+08	2.35E+08	256	2.35E+08	20	5.16039	2.74372
Nc-1-IP_pe	chr1	2.35E+08	2.35E+08	265	2.35E+08	17	5.14803	2.94914
Nc-1-IP_pe	chr1	2.35E+08	2.35E+08	417	2.35E+08	17	6.25544	3.43888
Nc-1-IP_pe	chr1	2.35E+08	2.35E+08	649	2.35E+08	18	5.33197	2.94862
Nc-1-IP_pe	chr1	2.35E+08	2.35E+08	299	2.35E+08	24	8.05897	3.50918
Nc-1-IP_pe	chr1	2.35E+08	2.35E+08	627	2.35E+08	23	8.92223	3.93219
Nc-1-IP_pe	chr1	2.35E+08	2.35E+08	690	2.35E+08	23	6.25615	2.94664

Nc-1-IP_pe	chr1	2.35E+08	2.35E+08	259	2.35E+08	19	7.67869	3.85921
Nc-1-IP_pe	chr1	2.35E+08	2.35E+08	361	2.35E+08	16	4.95684	2.94626
Nc-1-IP_pe	chr1	2.35E+08	2.35E+08	287	2.35E+08	19	6.58528	3.38508
Nc-1-IP_pe	chr1	2.35E+08	2.35E+08	358	2.35E+08	19	3.96692	2.35708
Nc-1-IP_pe	chr1	2.35E+08	2.35E+08	357	2.35E+08	13	4.42057	2.95252
Nc-1-IP_pe	chr1	2.35E+08	2.35E+08	525	2.35E+08	19	7.84735	3.93486
Nc-1-IP_pe	chr1	2.35E+08	2.35E+08	438	2.35E+08	20	8.56319	4.1316
Nc-1-IP_pe	chr1	2.35E+08	2.35E+08	755	2.35E+08	22	6.07094	2.94697
Nc-1-IP_pe	chr1	2.35E+08	2.35E+08	255	2.35E+08	20	6.49719	3.259
Nc-1-IP_pe	chr1	2.35E+08	2.35E+08	323	2.35E+08	20	4.71088	2.57831
Nc-1-IP_pe	chr1	2.35E+08	2.35E+08	392	2.35E+08	22	8.76839	3.98016
Nc-1-IP_pe	chr1	2.35E+08	2.35E+08	717	2.35E+08	24	6.44146	2.94634
Nc-1-IP_pe	chr1	2.35E+08	2.35E+08	532	2.35E+08	14	4.25972	2.7875
Nc-1-IP_pe	chr1	2.35E+08	2.35E+08	337	2.35E+08	16	3.65575	2.38624
Nc-1-IP_pe	chr1	2.35E+08	2.35E+08	662	2.35E+08	16	6.33285	3.58457
Nc-1-IP_pe	chr1	2.35E+08	2.35E+08	888	2.35E+08	15	4.06016	2.62146
Nc-1-IP_pe	chr1	2.35E+08	2.35E+08	270	2.35E+08	13	3.67377	2.58159
Nc-1-IP_pe	chr1	2.35E+08	2.35E+08	379	2.35E+08	17	5.14803	2.94914
Nc-1-IP_pe	chr1	2.35E+08	2.35E+08	603	2.35E+08	15	2.95965	2.14351
Nc-1-IP_pe	chr1	2.35E+08	2.35E+08	322	2.35E+08	21	6.66275	3.2402
Nc-1-IP_pe	chr1	2.35E+08	2.35E+08	320	2.35E+08	19	6.0077	3.14582
Nc-1-IP_pe	chr1	2.35E+08	2.35E+08	366	2.35E+08	15	5.36727	3.22728
Nc-1-IP_pe	chr1	2.35E+08	2.35E+08	272	2.35E+08	13	4.02562	2.7544
Nc-1-IP_pe	chr1	2.35E+08	2.35E+08	254	2.35E+08	17	5.54967	3.12324
Nc-1-IP_pe	chr1	2.35E+08	2.35E+08	543	2.35E+08	15	3.74763	2.48304
Nc-1-IP_pe	chr1	2.35E+08	2.35E+08	677	2.35E+08	24	10.8883	4.60999
Nc-1-IP_pe	chr1	2.35E+08	2.35E+08	281	2.35E+08	11	4.06403	2.95415
Nc-1-IP_pe	chr1	2.35E+08	2.35E+08	564	2.35E+08	12	4.70082	3.20033
Nc-1-IP_pe	chr1	2.35E+08	2.35E+08	506	2.35E+08	16	4.50821	2.74867
Nc-1-IP_pe	chr1	2.35E+08	2.35E+08	290	2.35E+08	17	5.6931	3.18638
Nc-1-IP_pe	chr1	2.35E+08	2.35E+08	378	2.35E+08	14	3.96259	2.64722
Nc-1-IP_pe	chr1	2.35E+08	2.35E+08	458	2.35E+08	16	3.94146	2.50592
Nc-1-IP_pe	chr1	2.35E+08	2.35E+08	274	2.35E+08	20	5.34663	2.81336
Nc-1-IP_pe	chr1	2.35E+08	2.35E+08	613	2.35E+08	19	5.90394	3.10364
Nc-1-IP_pe	chr1	2.35E+08	2.35E+08	318	2.35E+08	24	7.9276	3.46175
Nc-1-IP_pe	chr1	2.35E+08	2.35E+08	1220	2.35E+08	19	6.78661	3.47027
Nc-1-IP_pe	chr1	2.35E+08	2.35E+08	334	2.35E+08	16	4.25252	2.63824
Nc-1-IP_pe	chr1	2.35E+08	2.35E+08	699	2.35E+08	16	4.74488	2.85228
Nc-1-IP_pe	chr1	2.35E+08	2.35E+08	452	2.35E+08	24	4.91679	2.45429
Nc-1-IP_pe	chr1	2.35E+08	2.35E+08	269	2.35E+08	18	3.77591	2.33276
Nc-1-IP_pe	chr1	2.35E+08	2.35E+08	417	2.35E+08	27	6.66155	2.84379
Nc-1-IP_pe	chr1	2.35E+08	2.35E+08	412	2.35E+08	14	4.19452	2.75652
Nc-1-IP_pe	chr1	2.35E+08	2.35E+08	262	2.35E+08	16	6.16124	3.50224
Nc-1-IP_pe	chr1	2.35E+08	2.35E+08	444	2.35E+08	22	4.80668	2.5094
Nc-1-IP_pe	chr1	2.35E+08	2.35E+08	268	2.35E+08	14	4.21449	2.76599

Nc-1-IP_pe	chr1	2.35E+08	2.35E+08	331	2.35E+08	16	5.50606	3.19508
Nc-1-IP_pe	chr1	2.35E+08	2.35E+08	265	2.35E+08	15	4.7819	2.95039
Nc-1-IP_pe	chr1	2.35E+08	2.35E+08	440	2.35E+08	19	3.96692	2.35708
Nc-1-IP_pe	chr1	2.35E+08	2.35E+08	318	2.35E+08	12	3.83688	2.74114
Nc-1-IP_pe	chr1	2.35E+08	2.35E+08	487	2.35E+08	17	4.78101	2.79343
Nc-1-IP_pe	chr1	2.35E+08	2.35E+08	319	2.35E+08	13	5.4426	3.4862
Nc-1-IP_pe	chr1	2.35E+08	2.35E+08	588	2.35E+08	15	6.78905	3.93887
Nc-1-IP_pe	chr1	2.35E+08	2.35E+08	567	2.35E+08	12	2.87625	2.25568
Nc-1-IP_pe	chr1	2.35E+08	2.35E+08	316	2.35E+08	14	5.51541	3.4073
Nc-1-IP_pe	chr1	2.35E+08	2.35E+08	399	2.35E+08	16	5.82808	3.34463
Nc-1-IP_pe	chr1	2.35E+08	2.35E+08	599	2.35E+08	18	7.73136	4.00629
Nc-1-IP_pe	chr1	2.35E+08	2.35E+08	265	2.35E+08	15	5.83691	3.4562
Nc-1-IP_pe	chr1	2.35E+08	2.35E+08	764	2.35E+08	13	5.21206	3.36317
Nc-1-IP_pe	chr1	2.35E+08	2.35E+08	423	2.35E+08	19	5.90435	3.10381
Nc-1-IP_pe	chr1	2.35E+08	2.35E+08	411	2.35E+08	19	6.78661	3.47027
Nc-1-IP_pe	chr1	2.35E+08	2.35E+08	363	2.35E+08	14	6.28469	3.80898
Nc-1-IP_pe	chr1	2.35E+08	2.35E+08	576	2.35E+08	15	5.20142	3.14789
Nc-1-IP_pe	chr1	2.35E+08	2.35E+08	393	2.35E+08	18	2.88361	1.99878
Nc-1-IP_pe	chr1	2.35E+08	2.35E+08	376	2.35E+08	18	8.65961	4.45337
Nc-1-IP_pe	chr1	2.35E+08	2.35E+08	1263	2.35E+08	28	6.84886	2.84698
Nc-1-IP_pe	chr1	2.35E+08	2.35E+08	322	2.35E+08	15	4.40338	2.77622
Nc-1-IP_pe	chr1	2.35E+08	2.35E+08	667	2.35E+08	31	8.83213	3.25005
Nc-1-IP_pe	chr1	2.35E+08	2.35E+08	290	2.35E+08	17	8.58498	4.57078
Nc-1-IP_pe	chr1	2.35E+08	2.35E+08	311	2.35E+08	20	6.94658	3.44067
Nc-1-IP_pe	chr1	2.35E+08	2.35E+08	343	2.35E+08	14	6.06519	3.69269
Nc-1-IP_pe	chr1	2.35E+08	2.35E+08	965	2.35E+08	15	6.19428	3.63445
Nc-1-IP_pe	chr1	2.35E+08	2.35E+08	493	2.35E+08	17	9.09431	4.8363
Nc-1-IP_pe	chr1	2.35E+08	2.35E+08	340	2.35E+08	14	4.60011	2.95114
Nc-1-IP_pe	chr1	2.35E+08	2.35E+08	363	2.35E+08	19	5.90435	3.10381
Nc-1-IP_pe	chr1	2.35E+08	2.35E+08	485	2.35E+08	14	5.33286	3.31443
Nc-1-IP_pe	chr1	2.35E+08	2.35E+08	344	2.35E+08	13	4.43119	2.95792
Nc-1-IP_pe	chr1	2.35E+08	2.35E+08	365	2.35E+08	13	4.50646	2.99621
Nc-1-IP_pe	chr1	2.35E+08	2.35E+08	465	2.35E+08	22	9.95686	4.48294
Nc-1-IP_pe	chr1	2.35E+08	2.35E+08	313	2.35E+08	19	7.05262	3.58428
Nc-1-IP_pe	chr1	2.35E+08	2.35E+08	365	2.35E+08	14	3.55308	2.45762
Nc-1-IP_pe	chr1	2.35E+08	2.35E+08	378	2.35E+08	20	6.49719	3.259
Nc-1-IP_pe	chr1	2.35E+08	2.35E+08	281	2.35E+08	12	4.05718	2.85614
Nc-1-IP_pe	chr1	2.35E+08	2.35E+08	340	2.35E+08	14	4.8399	3.06836
Nc-1-IP_pe	chr1	2.35E+08	2.35E+08	514	2.35E+08	19	5.15822	2.80735
Nc-1-IP_pe	chr1	2.35E+08	2.35E+08	426	2.35E+08	15	3.74763	2.48304
Nc-1-IP_pe	chr1	2.35E+08	2.35E+08	589	2.35E+08	15	3.20025	2.24588
Nc-1-IP_pe	chr1	2.35E+08	2.35E+08	285	2.35E+08	15	5.1177	3.10809
Nc-1-IP_pe	chr1	2.35E+08	2.35E+08	431	2.35E+08	20	6.94658	3.44067
Nc-1-IP_pe	chr1	2.36E+08	2.36E+08	519	2.36E+08	17	8.24512	4.3971
Nc-1-IP_pe	chr1	2.36E+08	2.36E+08	271	2.36E+08	18	7.1522	3.73811

Nc-1-IP_pe	chr1	2.36E+08	2.36E+08	265	2.36E+08	17	6.64406	3.61808
Nc-1-IP_pe	chr1	2.36E+08	2.36E+08	607	2.36E+08	17	5.15375	2.9516
Nc-1-IP_pe	chr1	2.36E+08	2.36E+08	275	2.36E+08	18	6.63038	3.50359
Nc-1-IP_pe	chr1	2.36E+08	2.36E+08	557	2.36E+08	14	3.55308	2.45762
Nc-1-IP_pe	chr1	2.36E+08	2.36E+08	599	2.36E+08	14	5.29552	3.29555
Nc-1-IP_pe	chr1	2.36E+08	2.36E+08	764	2.36E+08	14	5.43414	3.36584
Nc-1-IP_pe	chr1	2.36E+08	2.36E+08	462	2.36E+08	16	6.22699	3.53369
Nc-1-IP_pe	chr1	2.36E+08	2.36E+08	1231	2.36E+08	18	4.04135	2.43445
Nc-1-IP_pe	chr1	2.36E+08	2.36E+08	722	2.36E+08	21	6.2632	3.08808
Nc-1-IP_pe	chr1	2.36E+08	2.36E+08	522	2.36E+08	13	4.44185	2.96333
Nc-1-IP_pe	chr1	2.36E+08	2.36E+08	405	2.36E+08	18	7.14481	3.73475
Nc-1-IP_pe	chr1	2.36E+08	2.36E+08	370	2.36E+08	16	4.68897	2.82769
Nc-1-IP_pe	chr1	2.36E+08	2.36E+08	481	2.36E+08	17	5.9911	3.31919
Nc-1-IP_pe	chr1	2.36E+08	2.36E+08	330	2.36E+08	12	3.47963	2.55766
Nc-1-IP_pe	chr1	2.36E+08	2.36E+08	645	2.36E+08	16	6.8987	3.86161
Nc-1-IP_pe	chr1	2.36E+08	2.36E+08	261	2.36E+08	13	5.03208	3.26819
Nc-1-IP_pe	chr1	2.36E+08	2.36E+08	279	2.36E+08	19	7.29128	3.68799
Nc-1-IP_pe	chr1	2.36E+08	2.36E+08	719	2.36E+08	16	4.59224	2.7853
Nc-1-IP_pe	chr1	2.36E+08	2.36E+08	473	2.36E+08	14	3.26761	2.32785
Nc-1-IP_pe	chr1	2.36E+08	2.36E+08	342	2.36E+08	21	7.40735	3.53242
Nc-1-IP_pe	chr1	2.36E+08	2.36E+08	826	2.36E+08	19	5.15822	2.80735
Nc-1-IP_pe	chr1	2.36E+08	2.36E+08	294	2.36E+08	18	7.1522	3.73811
Nc-1-IP_pe	chr1	2.36E+08	2.36E+08	320	2.36E+08	16	7.97877	4.4137
Nc-1-IP_pe	chr1	2.36E+08	2.36E+08	337	2.36E+08	15	7.02624	4.06292
Nc-1-IP_pe	chr1	2.36E+08	2.36E+08	265	2.36E+08	20	6.49719	3.259
Nc-1-IP_pe	chr1	2.36E+08	2.36E+08	566	2.36E+08	18	8.37884	4.31591
Nc-1-IP_pe	chr1	2.36E+08	2.36E+08	501	2.36E+08	24	7.9276	3.46175
Nc-1-IP_pe	chr1	2.36E+08	2.36E+08	294	2.36E+08	13	6.31249	3.96385
Nc-1-IP_pe	chr1	2.36E+08	2.36E+08	512	2.36E+08	13	4.02562	2.7544
Nc-1-IP_pe	chr1	2.36E+08	2.36E+08	305	2.36E+08	13	6.38222	4.00303
Nc-1-IP_pe	chr1	2.36E+08	2.36E+08	310	2.36E+08	14	5.03085	3.16286
Nc-1-IP_pe	chr1	2.36E+08	2.36E+08	290	2.36E+08	20	8.56319	4.1316
Nc-1-IP_pe	chr1	2.36E+08	2.36E+08	314	2.36E+08	19	6.32622	3.27682
Nc-1-IP_pe	chr1	2.36E+08	2.36E+08	275	2.36E+08	17	5.9911	3.31919
Nc-1-IP_pe	chr1	2.36E+08	2.36E+08	364	2.36E+08	16	5.90727	3.38183
Nc-1-IP_pe	chr1	2.36E+08	2.36E+08	256	2.36E+08	13	5.38711	3.45645
Nc-1-IP_pe	chr1	2.36E+08	2.36E+08	289	2.36E+08	12	3.47963	2.55766
Nc-1-IP_pe	chr1	2.36E+08	2.36E+08	253	2.36E+08	14	6.55975	3.95658
Nc-1-IP_pe	chr1	2.36E+08	2.36E+08	679	2.36E+08	21	11.4957	5.35164
Nc-1-IP_pe	chr1	2.36E+08	2.36E+08	291	2.36E+08	16	5.6319	3.2532
Nc-1-IP_pe	chr1	2.36E+08	2.36E+08	459	2.36E+08	14	4.43314	2.87047
Nc-1-IP_pe	chr1	2.36E+08	2.36E+08	313	2.36E+08	17	5.89608	3.2766
Nc-1-IP_pe	chr1	2.36E+08	2.36E+08	293	2.36E+08	12	4.39405	3.03475
Nc-1-IP_pe	chr1	2.36E+08	2.36E+08	360	2.36E+08	9	2.90462	2.46889
Nc-1-IP_pe	chr1	2.36E+08	2.36E+08	297	2.36E+08	16	4.59224	2.7853

Nc-1-IP_pe	chr1	2.36E+08	2.36E+08	380	2.36E+08	15	7.28736	4.2012
Nc-1-IP_pe	chr1	2.36E+08	2.36E+08	351	2.36E+08	18	9.27639	4.76202
Nc-1-IP_pe	chr1	2.36E+08	2.36E+08	342	2.36E+08	16	4.59224	2.7853
Nc-1-IP_pe	chr1	2.36E+08	2.36E+08	713	2.36E+08	15	5.66913	3.37372
Nc-1-IP_pe	chr1	2.36E+08	2.36E+08	271	2.36E+08	14	4.60011	2.95114
Nc-1-IP_pe	chr1	2.36E+08	2.36E+08	443	2.36E+08	13	4.41954	2.952
Nc-1-IP_pe	chr1	2.36E+08	2.36E+08	486	2.36E+08	14	6.06519	3.69269
Nc-1-IP_pe	chr1	2.36E+08	2.36E+08	346	2.36E+08	12	4.34861	3.01046
Nc-1-IP_pe	chr1	2.36E+08	2.36E+08	451	2.36E+08	19	8.85804	4.40242
Nc-1-IP_pe	chr1	2.36E+08	2.36E+08	469	2.36E+08	20	7.19732	3.54396
Nc-1-IP_pe	chr1	2.36E+08	2.36E+08	353	2.36E+08	13	5.36853	3.44651
Nc-1-IP_pe	chr1	2.36E+08	2.36E+08	512	2.36E+08	24	11.6162	4.91857
Nc-1-IP_pe	chr1	2.36E+08	2.36E+08	265	2.36E+08	21	6.23053	3.07579
Nc-1-IP_pe	chr1	2.36E+08	2.36E+08	873	2.36E+08	21	9.3401	4.34659
Nc-1-IP_pe	chr1	2.36E+08	2.36E+08	1278	2.36E+08	16	4.50821	2.74867
Nc-1-IP_pe	chr1	2.36E+08	2.36E+08	271	2.36E+08	16	4.59224	2.7853
Nc-1-IP_pe	chr1	2.36E+08	2.36E+08	505	2.36E+08	18	6.91333	3.62993
Nc-1-IP_pe	chr1	2.36E+08	2.36E+08	402	2.36E+08	13	5.15976	3.33548
Nc-1-IP_pe	chr1	2.36E+08	2.36E+08	688	2.36E+08	31	10.0584	3.61026
Nc-1-IP_pe	chr1	2.36E+08	2.36E+08	349	2.36E+08	21	5.5349	2.81884
Nc-1-IP_pe	chr1	2.36E+08	2.36E+08	293	2.36E+08	18	5.72638	3.11298
Nc-1-IP_pe	chr1	2.36E+08	2.36E+08	414	2.36E+08	19	7.22241	3.65793
Nc-1-IP_pe	chr1	2.36E+08	2.36E+08	736	2.36E+08	14	5.03085	3.16286
Nc-1-IP_pe	chr1	2.36E+08	2.36E+08	264	2.36E+08	13	4.04902	2.76601
Nc-1-IP_pe	chr1	2.36E+08	2.36E+08	572	2.36E+08	19	6.56181	3.3752
Nc-1-IP_pe	chr1	2.36E+08	2.36E+08	272	2.36E+08	11	3.63586	2.71903
Nc-1-IP_pe	chr1	2.36E+08	2.36E+08	366	2.36E+08	12	3.77112	2.7071
Nc-1-IP_pe	chr1	2.36E+08	2.36E+08	351	2.36E+08	20	7.43645	3.64378
Nc-1-IP_pe	chr1	2.36E+08	2.36E+08	735	2.36E+08	15	4.90347	3.00714
Nc-1-IP_pe	chr1	2.36E+08	2.36E+08	507	2.36E+08	13	4.41954	2.952
Nc-1-IP_pe	chr1	2.36E+08	2.36E+08	345	2.36E+08	12	3.47963	2.55766
Nc-1-IP_pe	chr1	2.36E+08	2.36E+08	323	2.36E+08	9	3.27326	2.68684
Nc-1-IP_pe	chr1	2.36E+08	2.36E+08	266	2.36E+08	12	5.84943	3.84431
Nc-1-IP_pe	chr1	2.36E+08	2.36E+08	380	2.36E+08	35	13.3425	4.26967
Nc-1-IP_pe	chr1	2.36E+08	2.36E+08	561	2.36E+08	16	7.53857	4.18505
Nc-1-IP_pe	chr1	2.36E+08	2.36E+08	278	2.36E+08	13	3.35769	2.42919
Nc-1-IP_pe	chr1	2.36E+08	2.36E+08	264	2.36E+08	15	6.19428	3.63445
Nc-1-IP_pe	chr1	2.36E+08	2.36E+08	257	2.36E+08	13	3.67377	2.58159
Nc-1-IP_pe	chr1	2.36E+08	2.36E+08	322	2.36E+08	12	3.33349	2.48364
Nc-1-IP_pe	chr1	2.36E+08	2.36E+08	272	2.36E+08	11	3.90263	2.86488
Nc-1-IP_pe	chr1	2.36E+08	2.36E+08	321	2.36E+08	17	7.6273	4.08876
Nc-1-IP_pe	chr1	2.36E+08	2.36E+08	450	2.36E+08	23	10.1326	4.42559
Nc-1-IP_pe	chr1	2.36E+08	2.36E+08	292	2.36E+08	17	7.02067	3.79543
Nc-1-IP_pe	chr1	2.36E+08	2.36E+08	426	2.36E+08	10	3.49455	2.72742
Nc-1-IP_pe	chr1	2.36E+08	2.36E+08	518	2.36E+08	13	4.86362	3.18015

Nc-1-IP_pe	chr1	2.36E+08	2.36E+08	730	2.36E+08	16	7.79347	4.31685
Nc-1-IP_pe	chr1	2.36E+08	2.36E+08	980	2.36E+08	22	7.27519	3.39036
Nc-1-IP_pe	chr1	2.36E+08	2.36E+08	553	2.36E+08	19	7.56458	3.8084
Nc-1-IP_pe	chr1	2.36E+08	2.36E+08	411	2.36E+08	13	4.06809	2.77549
Nc-1-IP_pe	chr1	2.36E+08	2.36E+08	931	2.36E+08	21	10.7603	4.99737
Nc-1-IP_pe	chr1	2.36E+08	2.36E+08	268	2.36E+08	15	4.41451	2.78128
Nc-1-IP_pe	chr1	2.36E+08	2.36E+08	263	2.36E+08	17	6.64406	3.61808
Nc-1-IP_pe	chr1	2.36E+08	2.36E+08	334	2.36E+08	16	6.80394	3.81462
Nc-1-IP_pe	chr1	2.36E+08	2.36E+08	429	2.36E+08	16	4.25252	2.63824
Nc-1-IP_pe	chr1	2.36E+08	2.36E+08	460	2.36E+08	18	7.1522	3.73811
Nc-1-IP_pe	chr1	2.36E+08	2.36E+08	644	2.36E+08	18	8.84551	4.54544
Nc-1-IP_pe	chr1	2.36E+08	2.36E+08	359	2.36E+08	14	3.86727	2.6027
Nc-1-IP_pe	chr1	2.36E+08	2.36E+08	732	2.36E+08	18	6.63038	3.50359
Nc-1-IP_pe	chr1	2.36E+08	2.36E+08	288	2.36E+08	16	6.54198	3.68597
Nc-1-IP_pe	chr1	2.36E+08	2.36E+08	509	2.36E+08	18	5.42854	2.98853
Nc-1-IP_pe	chr1	2.36E+08	2.36E+08	401	2.36E+08	24	6.44146	2.94634
Nc-1-IP_pe	chr1	2.36E+08	2.36E+08	387	2.36E+08	18	8.20254	4.23058
Nc-1-IP_pe	chr1	2.36E+08	2.36E+08	597	2.36E+08	13	4.41954	2.952
Nc-1-IP_pe	chr1	2.37E+08	2.37E+08	437	2.37E+08	12	3.09974	2.36643
Nc-1-IP_pe	chr1	2.37E+08	2.37E+08	575	2.37E+08	22	9.39426	4.24119
Nc-1-IP_pe	chr1	2.37E+08	2.37E+08	348	2.37E+08	12	4.66786	3.1824
Nc-1-IP_pe	chr1	2.37E+08	2.37E+08	901	2.37E+08	25	5.37675	2.55246
Nc-1-IP_pe	chr1	2.37E+08	2.37E+08	290	2.37E+08	20	7.71924	3.7635
Nc-1-IP_pe	chr1	2.37E+08	2.37E+08	477	2.37E+08	14	4.21449	2.76599
Nc-1-IP_pe	chr1	2.37E+08	2.37E+08	425	2.37E+08	12	4.27054	2.96887
Nc-1-IP_pe	chr1	2.37E+08	2.37E+08	334	2.37E+08	12	3.47963	2.55766
Nc-1-IP_pe	chr1	2.37E+08	2.37E+08	380	2.37E+08	12	4.39405	3.03475
Nc-1-IP_pe	chr1	2.37E+08	2.37E+08	435	2.37E+08	11	5.10577	3.5486
Nc-1-IP_pe	chr1	2.37E+08	2.37E+08	345	2.37E+08	21	3.63688	2.15978
Nc-1-IP_pe	chr1	2.37E+08	2.37E+08	721	2.37E+08	15	7.02624	4.06292
Nc-1-IP_pe	chr1	2.37E+08	2.37E+08	300	2.37E+08	10	3.8436	2.92819
Nc-1-IP_pe	chr1	2.37E+08	2.37E+08	316	2.37E+08	14	4.52309	2.91384
Nc-1-IP_pe	chr1	2.37E+08	2.37E+08	520	2.37E+08	16	4.59224	2.7853
Nc-1-IP_pe	chr1	2.37E+08	2.37E+08	514	2.37E+08	12	5.01934	3.37518
Nc-1-IP_pe	chr1	2.37E+08	2.37E+08	429	2.37E+08	14	6.06519	3.69269
Nc-1-IP_pe	chr1	2.37E+08	2.37E+08	310	2.37E+08	15	4.40338	2.77622
Nc-1-IP_pe	chr1	2.37E+08	2.37E+08	490	2.37E+08	16	6.94043	3.88237
Nc-1-IP_pe	chr1	2.37E+08	2.37E+08	289	2.37E+08	16	6.8987	3.86161
Nc-1-IP_pe	chr1	2.37E+08	2.37E+08	880	2.37E+08	16	5.82808	3.34463
Nc-1-IP_pe	chr1	2.37E+08	2.37E+08	306	2.37E+08	14	6.25343	3.79234
Nc-1-IP_pe	chr1	2.37E+08	2.37E+08	565	2.37E+08	17	5.14803	2.94914
Nc-1-IP_pe	chr1	2.37E+08	2.37E+08	324	2.37E+08	19	7.29128	3.68799
Nc-1-IP_pe	chr1	2.37E+08	2.37E+08	290	2.37E+08	21	4.34782	2.40029
Nc-1-IP_pe	chr1	2.37E+08	2.37E+08	322	2.37E+08	14	6.06519	3.69269
Nc-1-IP_pe	chr1	2.37E+08	2.37E+08	350	2.37E+08	11	3.28475	2.53029

Nc-1-IP_pe	chr1	2.37E+08	2.37E+08	605	2.37E+08	18	7.47506	3.88659
Nc-1-IP_pe	chr1	2.37E+08	2.37E+08	344	2.37E+08	28	8.05622	3.20922
Nc-1-IP_pe	chr1	2.37E+08	2.37E+08	580	2.37E+08	14	5.56442	3.43239
Nc-1-IP_pe	chr1	2.37E+08	2.37E+08	288	2.37E+08	14	4.21449	2.76599
Nc-1-IP_pe	chr1	2.37E+08	2.37E+08	432	2.37E+08	13	4.41954	2.952
Nc-1-IP_pe	chr1	2.37E+08	2.37E+08	308	2.37E+08	17	8.9884	4.78058
Nc-1-IP_pe	chr1	2.37E+08	2.37E+08	271	2.37E+08	15	4.06016	2.62146
Nc-1-IP_pe	chr1	2.37E+08	2.37E+08	362	2.37E+08	15	8.20837	4.70271
Nc-1-IP_pe	chr1	2.37E+08	2.37E+08	431	2.37E+08	12	4.06753	2.86157
Nc-1-IP_pe	chr1	2.37E+08	2.37E+08	676	2.37E+08	14	6.50995	3.92971
Nc-1-IP_pe	chr1	2.37E+08	2.37E+08	270	2.37E+08	15	7.19874	4.15407
Nc-1-IP_pe	chr1	2.37E+08	2.37E+08	267	2.37E+08	12	5.23082	3.49289
Nc-1-IP_pe	chr1	2.37E+08	2.37E+08	345	2.37E+08	14	5.30793	3.30182
Nc-1-IP_pe	chr1	2.37E+08	2.37E+08	802	2.37E+08	12	5.01934	3.37518
Nc-1-IP_pe	chr1	2.37E+08	2.37E+08	339	2.37E+08	12	4.75569	3.23024
Nc-1-IP_pe	chr1	2.37E+08	2.37E+08	289	2.37E+08	12	4.24064	2.95299
Nc-1-IP_pe	chr1	2.37E+08	2.37E+08	335	2.37E+08	14	8.20556	4.87976
Nc-1-IP_pe	chr1	2.37E+08	2.37E+08	347	2.37E+08	16	10.117	5.58955
Nc-1-IP_pe	chr1	2.37E+08	2.37E+08	328	2.37E+08	14	5.51541	3.4073
Nc-1-IP_pe	chr1	2.37E+08	2.37E+08	301	2.37E+08	12	6.35031	4.1361
Nc-1-IP_pe	chr1	2.37E+08	2.37E+08	253	2.37E+08	16	5.37454	3.13479
Nc-1-IP_pe	chr1	2.37E+08	2.37E+08	291	2.37E+08	15	8.26005	4.73146
Nc-1-IP_pe	chr1	2.37E+08	2.37E+08	523	2.37E+08	19	7.29128	3.68799
Nc-1-IP_pe	chr1	2.37E+08	2.37E+08	506	2.37E+08	8	3.74505	3.07688
Nc-1-IP_pe	chr1	2.37E+08	2.37E+08	293	2.37E+08	10	5.10552	3.682
Nc-1-IP_pe	chr1	2.37E+08	2.37E+08	286	2.37E+08	14	7.34079	4.38651
Nc-1-IP_pe	chr1	2.38E+08	2.38E+08	272	2.38E+08	10	5.23357	3.76063
Nc-1-IP_pe	chr1	2.38E+08	2.38E+08	370	2.38E+08	13	4.86362	3.18015
Nc-1-IP_pe	chr1	2.38E+08	2.38E+08	681	2.38E+08	19	9.03708	4.48779
Nc-1-IP_pe	chr1	2.38E+08	2.38E+08	331	2.38E+08	13	7.7352	4.78626
Nc-1-IP_pe	chr1	2.38E+08	2.38E+08	514	2.38E+08	12	5.94619	3.9002
Nc-1-IP_pe	chr1	2.38E+08	2.38E+08	875	2.38E+08	15	8.16556	4.67894
Nc-1-IP_pe	chr1	2.38E+08	2.38E+08	432	2.38E+08	19	7.88414	3.95145
Nc-1-IP_pe	chr1	2.38E+08	2.38E+08	731	2.38E+08	16	5.82808	3.34463
Nc-1-IP_pe	chr1	2.38E+08	2.38E+08	298	2.38E+08	14	6.69566	4.03025
Nc-1-IP_pe	chr1	2.38E+08	2.38E+08	267	2.38E+08	10	3.21261	2.56787
Nc-1-IP_pe	chr1	2.38E+08	2.38E+08	287	2.38E+08	9	3.27326	2.68684
Nc-1-IP_pe	chr1	2.38E+08	2.38E+08	292	2.38E+08	11	4.06403	2.95415
Nc-1-IP_pe	chr1	2.38E+08	2.38E+08	577	2.38E+08	19	12.6812	6.38078
Nc-1-IP_pe	chr1	2.38E+08	2.38E+08	554	2.38E+08	17	9.09431	4.8363
Nc-1-IP_pe	chr1	2.38E+08	2.38E+08	373	2.38E+08	16	3.39261	2.27748
Nc-1-IP_pe	chr1	2.38E+08	2.38E+08	298	2.38E+08	14	8.29543	4.93196
Nc-1-IP_pe	chr1	2.38E+08	2.38E+08	322	2.38E+08	12	3.16137	2.39719
Nc-1-IP_pe	chr1	2.38E+08	2.38E+08	286	2.38E+08	11	4.06403	2.95415
Nc-1-IP_pe	chr1	2.38E+08	2.38E+08	614	2.38E+08	19	8.4638	4.21715

Nc-1-IP_pe	chr1	2.38E+08	2.38E+08	292	2.38E+08	11	5.7051	3.90381
Nc-1-IP_pe	chr1	2.38E+08	2.38E+08	254	2.38E+08	12	4.24064	2.95299
Nc-1-IP_pe	chr1	2.38E+08	2.38E+08	874	2.38E+08	10	5.01973	3.6295
Nc-1-IP_pe	chr1	2.38E+08	2.38E+08	307	2.38E+08	11	4.06403	2.95415
Nc-1-IP_pe	chr1	2.38E+08	2.38E+08	611	2.38E+08	14	5.43414	3.36584
Nc-1-IP_pe	chr1	2.38E+08	2.38E+08	254	2.38E+08	16	9.01614	4.97137
Nc-1-IP_pe	chr1	2.38E+08	2.38E+08	561	2.38E+08	11	4.06403	2.95415
Nc-1-IP_pe	chr1	2.38E+08	2.38E+08	279	2.38E+08	11	5.77463	3.94557
Nc-1-IP_pe	chr1	2.38E+08	2.38E+08	258	2.38E+08	13	7.7352	4.78626
Nc-1-IP_pe	chr1	2.38E+08	2.38E+08	318	2.38E+08	16	7.53857	4.18505
Nc-1-IP_pe	chr1	2.38E+08	2.38E+08	469	2.38E+08	9	3.27326	2.68684
Nc-1-IP_pe	chr1	2.38E+08	2.38E+08	256	2.38E+08	14	6.06519	3.69269
Nc-1-IP_pe	chr1	2.38E+08	2.38E+08	276	2.38E+08	12	5.84943	3.84431
Nc-1-IP_pe	chr1	2.38E+08	2.38E+08	283	2.38E+08	12	6.12485	4.004
Nc-1-IP_pe	chr1	2.38E+08	2.38E+08	495	2.38E+08	11	4.06403	2.95415
Nc-1-IP_pe	chr1	2.38E+08	2.38E+08	389	2.38E+08	13	5.15976	3.33548
Nc-1-IP_pe	chr1	2.38E+08	2.38E+08	276	2.38E+08	14	6.69566	4.03025
Nc-1-IP_pe	chr1	2.38E+08	2.38E+08	277	2.38E+08	12	6.86906	4.44438
Nc-1-IP_pe	chr1	2.39E+08	2.39E+08	596	2.39E+08	14	3.00733	2.21111
Nc-1-IP_pe	chr1	2.39E+08	2.39E+08	324	2.39E+08	15	7.02624	4.06292
Nc-1-IP_pe	chr1	2.39E+08	2.39E+08	273	2.39E+08	12	6.86906	4.44438
Nc-1-IP_pe	chr1	2.39E+08	2.39E+08	340	2.39E+08	12	6.82053	4.41531
Nc-1-IP_pe	chr1	2.39E+08	2.39E+08	273	2.39E+08	9	4.46937	3.41876
Nc-1-IP_pe	chr1	2.39E+08	2.39E+08	525	2.39E+08	20	8.56319	4.1316
Nc-1-IP_pe	chr1	2.39E+08	2.39E+08	301	2.39E+08	11	3.89191	2.85898
Nc-1-IP_pe	chr1	2.39E+08	2.39E+08	265	2.39E+08	12	5.84943	3.84431
Nc-1-IP_pe	chr1	2.39E+08	2.39E+08	526	2.39E+08	13	5.86991	3.71823
Nc-1-IP_pe	chr1	2.39E+08	2.39E+08	543	2.39E+08	15	5.83691	3.4562
Nc-1-IP_pe	chr1	2.39E+08	2.39E+08	293	2.39E+08	16	7.53857	4.18505
Nc-1-IP_pe	chr1	2.39E+08	2.39E+08	442	2.39E+08	14	5.98179	3.64885
Nc-1-IP_pe	chr1	2.39E+08	2.39E+08	334	2.39E+08	10	4.75926	3.47115
Nc-1-IP_pe	chr1	2.39E+08	2.39E+08	407	2.39E+08	9	3.92293	3.08
Nc-1-IP_pe	chr1	2.39E+08	2.39E+08	464	2.39E+08	13	3.36696	2.43362
Nc-1-IP_pe	chr1	2.39E+08	2.39E+08	275	2.39E+08	14	5.51541	3.4073
Nc-1-IP_pe	chr1	2.4E+08	2.4E+08	666	2.4E+08	14	7.42849	4.43574
Nc-1-IP_pe	chr1	2.4E+08	2.4E+08	279	2.4E+08	11	5.10577	3.5486
Nc-1-IP_pe	chr1	2.4E+08	2.4E+08	531	2.4E+08	10	3.4604	2.70797
Nc-1-IP_pe	chr1	2.4E+08	2.4E+08	309	2.4E+08	15	5.66913	3.37372
Nc-1-IP_pe	chr1	2.4E+08	2.4E+08	532	2.4E+08	14	4.73128	3.01506
Nc-1-IP_pe	chr1	2.4E+08	2.4E+08	279	2.4E+08	16	6.8987	3.86161
Nc-1-IP_pe	chr1	2.4E+08	2.4E+08	263	2.4E+08	15	7.46994	4.29894
Nc-1-IP_pe	chr1	2.4E+08	2.4E+08	291	2.4E+08	15	6.78905	3.93887
Nc-1-IP_pe	chr1	2.4E+08	2.4E+08	310	2.4E+08	16	10.117	5.58955
Nc-1-IP_pe	chr1	2.4E+08	2.4E+08	337	2.4E+08	8	3.67166	3.03007
Nc-1-IP_pe	chr1	2.4E+08	2.4E+08	615	2.4E+08	13	5.36853	3.44651

Nc-1-IP_pe chr1	2.4E+08	2.4E+08	357	2.4E+08	14	7.461	4.45404
Nc-1-IP_pe chr1	2.4E+08	2.4E+08	314	2.4E+08	14	7.77549	4.63233
Nc-1-IP_pe chr1	2.4E+08	2.4E+08	310	2.4E+08	12	6.07041	3.97229
Nc-1-IP_pe chr1	2.4E+08	2.4E+08	389	2.4E+08	12	4.24064	2.95299
Nc-1-IP_pe chr1	2.4E+08	2.4E+08	333	2.4E+08	15	7.56326	4.34922
Nc-1-IP_pe chr1	2.4E+08	2.4E+08	295	2.4E+08	12	5.84943	3.84431
Nc-1-IP_pe chr1	2.4E+08	2.4E+08	478	2.4E+08	10	5.03671	3.63988
Nc-1-IP_pe chr1	2.4E+08	2.4E+08	409	2.4E+08	15	5.66913	3.37372
Nc-1-IP_pe chr1	2.4E+08	2.4E+08	330	2.4E+08	14	7.38052	4.40879
Nc-1-IP_pe chr1	2.4E+08	2.4E+08	350	2.4E+08	16	5.98198	3.41707
Nc-1-IP_pe chr1	2.4E+08	2.4E+08	525	2.4E+08	16	7.53857	4.18505
Nc-1-IP_pe chr1	2.4E+08	2.4E+08	543	2.4E+08	14	6.25343	3.79234
Nc-1-IP_pe chr1	2.4E+08	2.4E+08	514	2.4E+08	16	6.33285	3.58457
Nc-1-IP_pe chr1	2.4E+08	2.4E+08	403	2.4E+08	14	5.03085	3.16286
Nc-1-IP_pe chr1	2.4E+08	2.4E+08	256	2.4E+08	13	3.35769	2.42919
Nc-1-IP_pe chr1	2.4E+08	2.4E+08	865	2.4E+08	21	9.29881	4.32834
Nc-1-IP_pe chr1	2.4E+08	2.4E+08	443	2.4E+08	14	6.06519	3.69269
Nc-1-IP_pe chr1	2.4E+08	2.4E+08	509	2.4E+08	11	2.96397	2.36091
Nc-1-IP_pe chr1	2.4E+08	2.4E+08	298	2.4E+08	10	3.89064	2.95552
Nc-1-IP_pe chr1	2.4E+08	2.4E+08	335	2.4E+08	14	6.13058	3.7272
Nc-1-IP_pe chr1	2.4E+08	2.4E+08	367	2.4E+08	13	6.19316	3.89711
Nc-1-IP_pe chr1	2.4E+08	2.4E+08	1547	2.4E+08	15	5.58327	3.33181
Nc-1-IP_pe chr1	2.4E+08	2.4E+08	296	2.4E+08	17	8.7831	4.67333
Nc-1-IP_pe chr1	2.4E+08	2.4E+08	904	2.4E+08	15	5.66913	3.37372
Nc-1-IP_pe chr1	2.4E+08	2.4E+08	579	2.4E+08	9	3.27326	2.68684
Nc-1-IP_pe chr1	2.4E+08	2.4E+08	602	2.4E+08	14	7.77549	4.63233
Nc-1-IP_pe chr1	2.4E+08	2.4E+08	726	2.4E+08	11	5.77463	3.94557
Nc-1-IP_pe chr1	2.4E+08	2.4E+08	343	2.4E+08	10	3.4604	2.70797
Nc-1-IP_pe chr1	2.4E+08	2.4E+08	288	2.4E+08	11	5.37297	3.70587
Nc-1-IP_pe chr1	2.4E+08	2.4E+08	1197	2.4E+08	17	9.42237	5.01057
Nc-1-IP_pe chr1	2.4E+08	2.4E+08	308	2.4E+08	13	5.9487	3.76157
Nc-1-IP_pe chr1	2.4E+08	2.4E+08	253	2.4E+08	15	7.46994	4.29894
Nc-1-IP_pe chr1	2.4E+08	2.4E+08	291	2.4E+08	12	5.23082	3.49289
Nc-1-IP_pe chr1	2.4E+08	2.4E+08	540	2.4E+08	11	4.61014	3.26192
Nc-1-IP_pe chr1	2.4E+08	2.4E+08	341	2.4E+08	16	3.94146	2.50592
Nc-1-IP_pe chr1	2.4E+08	2.4E+08	335	2.4E+08	12	4.24064	2.95299
Nc-1-IP_pe chr1	2.4E+08	2.4E+08	308	2.4E+08	16	6.8987	3.86161
Nc-1-IP_pe chr1	2.4E+08	2.4E+08	325	2.4E+08	14	7.42059	4.4313
Nc-1-IP_pe chr1	2.4E+08	2.4E+08	311	2.4E+08	14	7.1092	4.2574
Nc-1-IP_pe chr1	2.4E+08	2.4E+08	770	2.4E+08	15	7.46994	4.29894
Nc-1-IP_pe chr1	2.41E+08	2.41E+08	299	2.41E+08	10	4.65155	3.40613
Nc-1-IP_pe chr1	2.41E+08	2.41E+08	260	2.41E+08	12	4.06753	2.86157
Nc-1-IP_pe chr1	2.41E+08	2.41E+08	560	2.41E+08	16	6.33285	3.58457
Nc-1-IP_pe chr1	2.41E+08	2.41E+08	351	2.41E+08	15	8.97083	5.13293
Nc-1-IP_pe chr1	2.41E+08	2.41E+08	445	2.41E+08	16	7.53857	4.18505

Nc-1-IP_pe	chr1	2.41E+08	2.41E+08	373	2.41E+08	13	5.36853	3.44651
Nc-1-IP_pe	chr1	2.41E+08	2.41E+08	263	2.41E+08	13	6.72991	4.20021
Nc-1-IP_pe	chr1	2.41E+08	2.41E+08	300	2.41E+08	13	4.78908	3.14145
Nc-1-IP_pe	chr1	2.41E+08	2.41E+08	354	2.41E+08	13	4.86362	3.18015
Nc-1-IP_pe	chr1	2.41E+08	2.41E+08	269	2.41E+08	19	6.78661	3.47027
Nc-1-IP_pe	chr1	2.41E+08	2.41E+08	260	2.41E+08	12	5.98129	3.92053
Nc-1-IP_pe	chr1	2.41E+08	2.41E+08	359	2.41E+08	16	8.77032	4.83692
Nc-1-IP_pe	chr1	2.41E+08	2.41E+08	296	2.41E+08	14	7.12806	4.26787
Nc-1-IP_pe	chr1	2.41E+08	2.41E+08	286	2.41E+08	12	5.77527	3.80164
Nc-1-IP_pe	chr1	2.41E+08	2.41E+08	285	2.41E+08	13	6.14303	3.86919
Nc-1-IP_pe	chr1	2.41E+08	2.41E+08	311	2.41E+08	15	5.66913	3.37372
Nc-1-IP_pe	chr1	2.41E+08	2.41E+08	306	2.41E+08	15	6.39297	3.73508
Nc-1-IP_pe	chr1	2.41E+08	2.41E+08	628	2.41E+08	16	4.96462	2.94973
Nc-1-IP_pe	chr1	2.41E+08	2.41E+08	384	2.41E+08	11	4.41492	3.1509
Nc-1-IP_pe	chr1	2.41E+08	2.41E+08	429	2.41E+08	12	4.73037	3.21642
Nc-1-IP_pe	chr1	2.41E+08	2.41E+08	598	2.41E+08	12	4.28158	2.97474
Nc-1-IP_pe	chr1	2.41E+08	2.41E+08	412	2.41E+08	13	4.41954	2.952
Nc-1-IP_pe	chr1	2.41E+08	2.41E+08	441	2.41E+08	15	5.20142	3.14789
Nc-1-IP_pe	chr1	2.41E+08	2.41E+08	772	2.41E+08	24	6.77607	3.05911
Nc-1-IP_pe	chr1	2.41E+08	2.41E+08	321	2.41E+08	17	4.27863	2.58527
Nc-1-IP_pe	chr1	2.41E+08	2.41E+08	333	2.41E+08	20	11.5999	5.59018
Nc-1-IP_pe	chr1	2.41E+08	2.41E+08	267	2.41E+08	15	5.66913	3.37372
Nc-1-IP_pe	chr1	2.41E+08	2.41E+08	347	2.41E+08	19	7.29128	3.68799
Nc-1-IP_pe	chr1	2.41E+08	2.41E+08	307	2.41E+08	14	6.76465	4.06784
Nc-1-IP_pe	chr1	2.41E+08	2.41E+08	276	2.41E+08	19	10.3693	5.1464
Nc-1-IP_pe	chr1	2.41E+08	2.41E+08	279	2.41E+08	16	5.82808	3.34463
Nc-1-IP_pe	chr1	2.41E+08	2.41E+08	1369	2.41E+08	17	7.02067	3.79543
Nc-1-IP_pe	chr1	2.41E+08	2.41E+08	466	2.41E+08	18	5.33197	2.94862
Nc-1-IP_pe	chr1	2.41E+08	2.41E+08	353	2.41E+08	13	3.67377	2.58159
Nc-1-IP_pe	chr1	2.41E+08	2.41E+08	311	2.41E+08	13	6.65435	4.1571
Nc-1-IP_pe	chr1	2.41E+08	2.41E+08	269	2.41E+08	14	7.42059	4.4313
Nc-1-IP_pe	chr1	2.41E+08	2.41E+08	526	2.41E+08	19	9.15219	4.54307
Nc-1-IP_pe	chr1	2.41E+08	2.41E+08	297	2.41E+08	13	4.86362	3.18015
Nc-1-IP_pe	chr1	2.41E+08	2.41E+08	340	2.41E+08	13	4.24481	2.8638
Nc-1-IP_pe	chr1	2.41E+08	2.41E+08	257	2.41E+08	11	4.06403	2.95415
Nc-1-IP_pe	chr1	2.41E+08	2.41E+08	554	2.41E+08	17	8.20861	4.37862
Nc-1-IP_pe	chr1	2.41E+08	2.41E+08	334	2.41E+08	11	4.35236	3.11555
Nc-1-IP_pe	chr1	2.41E+08	2.41E+08	298	2.41E+08	14	4.21449	2.76599
Nc-1-IP_pe	chr1	2.41E+08	2.41E+08	317	2.41E+08	18	5.72638	3.11298
Nc-1-IP_pe	chr1	2.41E+08	2.41E+08	357	2.41E+08	19	6.78661	3.47027
Nc-1-IP_pe	chr1	2.41E+08	2.41E+08	610	2.41E+08	14	4.21449	2.76599
Nc-1-IP_pe	chr1	2.41E+08	2.41E+08	253	2.41E+08	13	4.86362	3.18015
Nc-1-IP_pe	chr1	2.41E+08	2.41E+08	267	2.41E+08	15	8.26005	4.73146
Nc-1-IP_pe	chr1	2.42E+08	2.42E+08	559	2.42E+08	19	5.51632	2.94814
Nc-1-IP_pe	chr1	2.42E+08	2.42E+08	340	2.42E+08	17	5.14803	2.94914

Nc-1-IP_pe	chr1	2.42E+08	2.42E+08	573	2.42E+08	18	6.15736	3.29676
Nc-1-IP_pe	chr1	2.42E+08	2.42E+08	337	2.42E+08	15	3.74763	2.48304
Nc-1-IP_pe	chr1	2.42E+08	2.42E+08	500	2.42E+08	17	5.7822	3.22586
Nc-1-IP_pe	chr1	2.42E+08	2.42E+08	331	2.42E+08	16	7.47575	4.15282
Nc-1-IP_pe	chr1	2.42E+08	2.42E+08	323	2.42E+08	17	7.6273	4.08876
Nc-1-IP_pe	chr1	2.42E+08	2.42E+08	582	2.42E+08	15	5.20142	3.14789
Nc-1-IP_pe	chr1	2.42E+08	2.42E+08	341	2.42E+08	15	8.33897	4.77548
Nc-1-IP_pe	chr1	2.42E+08	2.42E+08	420	2.42E+08	19	8.9469	4.44469
Nc-1-IP_pe	chr1	2.42E+08	2.42E+08	316	2.42E+08	19	9.41173	4.66885
Nc-1-IP_pe	chr1	2.42E+08	2.42E+08	359	2.42E+08	20	9.21699	4.428
Nc-1-IP_pe	chr1	2.42E+08	2.42E+08	311	2.42E+08	10	4.56202	3.35231
Nc-1-IP_pe	chr1	2.42E+08	2.42E+08	513	2.42E+08	13	4.67336	3.0817
Nc-1-IP_pe	chr1	2.42E+08	2.42E+08	253	2.42E+08	13	4.86362	3.18015
Nc-1-IP_pe	chr1	2.42E+08	2.42E+08	762	2.42E+08	15	7.69749	4.42193
Nc-1-IP_pe	chr1	2.42E+08	2.42E+08	356	2.42E+08	18	9.5677	4.91092
Nc-1-IP_pe	chr1	2.42E+08	2.42E+08	278	2.42E+08	18	10.2193	5.25104
Nc-1-IP_pe	chr1	2.42E+08	2.42E+08	559	2.42E+08	12	3.47963	2.55766
Nc-1-IP_pe	chr1	2.42E+08	2.42E+08	263	2.42E+08	16	6.30714	3.57219
Nc-1-IP_pe	chr1	2.42E+08	2.42E+08	271	2.42E+08	15	5.72092	3.39909
Nc-1-IP_pe	chr1	2.42E+08	2.42E+08	392	2.42E+08	11	3.82842	2.8241
Nc-1-IP_pe	chr1	2.42E+08	2.42E+08	314	2.42E+08	20	9.94634	4.77022
Nc-1-IP_pe	chr1	2.42E+08	2.42E+08	492	2.42E+08	12	4.63088	3.16234
Nc-1-IP_pe	chr1	2.42E+08	2.42E+08	417	2.42E+08	13	5.98019	3.77894
Nc-1-IP_pe	chr1	2.42E+08	2.42E+08	579	2.42E+08	15	4.7819	2.95039
Nc-1-IP_pe	chr1	2.42E+08	2.42E+08	844	2.42E+08	14	6.13058	3.7272
Nc-1-IP_pe	chr1	2.42E+08	2.42E+08	623	2.42E+08	13	4.78908	3.14145
Nc-1-IP_pe	chr1	2.42E+08	2.42E+08	412	2.42E+08	11	3.44608	2.61657
Nc-1-IP_pe	chr1	2.42E+08	2.42E+08	641	2.42E+08	19	3.48582	2.18208
Nc-1-IP_pe	chr1	2.42E+08	2.42E+08	565	2.42E+08	20	9.23622	4.43687
Nc-1-IP_pe	chr1	2.42E+08	2.42E+08	611	2.42E+08	16	6.77412	3.79988
Nc-1-IP_pe	chr1	2.42E+08	2.42E+08	412	2.42E+08	16	5.37454	3.13479
Nc-1-IP_pe	chr1	2.42E+08	2.42E+08	477	2.42E+08	15	6.39297	3.73508
Nc-1-IP_pe	chr1	2.42E+08	2.42E+08	300	2.42E+08	13	4.80086	3.14755
Nc-1-IP_pe	chr1	2.42E+08	2.42E+08	337	2.42E+08	16	5.62031	3.24783
Nc-1-IP_pe	chr1	2.42E+08	2.42E+08	623	2.42E+08	15	7.02624	4.06292
Nc-1-IP_pe	chr1	2.42E+08	2.42E+08	463	2.42E+08	9	3.27326	2.68684
Nc-1-IP_pe	chr1	2.42E+08	2.42E+08	270	2.42E+08	13	4.86362	3.18015
Nc-1-IP_pe	chr1	2.42E+08	2.42E+08	743	2.42E+08	16	9.06204	4.99663
Nc-1-IP_pe	chr1	2.42E+08	2.42E+08	285	2.42E+08	14	5.51541	3.4073
Nc-1-IP_pe	chr1	2.42E+08	2.42E+08	308	2.42E+08	18	7.73136	4.00629
Nc-1-IP_pe	chr1	2.42E+08	2.42E+08	405	2.42E+08	12	4.70082	3.20033
Nc-1-IP_pe	chr1	2.42E+08	2.42E+08	269	2.42E+08	13	4.41954	2.952
Nc-1-IP_pe	chr1	2.42E+08	2.42E+08	374	2.42E+08	17	8.31233	4.43123
Nc-1-IP_pe	chr1	2.42E+08	2.42E+08	293	2.42E+08	11	4.54404	3.2242
Nc-1-IP_pe	chr1	2.42E+08	2.42E+08	297	2.42E+08	19	8.4638	4.21715

Nc-1-IP_pe	chr1	2.42E+08	2.42E+08	311	2.42E+08	11	5.3232	3.67643
Nc-1-IP_pe	chr1	2.42E+08	2.42E+08	469	2.42E+08	12	5.77527	3.80164
Nc-1-IP_pe	chr1	2.42E+08	2.42E+08	309	2.42E+08	10	4.99888	3.61677
Nc-1-IP_pe	chr1	2.42E+08	2.42E+08	294	2.42E+08	13	7.08702	4.40581
Nc-1-IP_pe	chr1	2.42E+08	2.42E+08	278	2.42E+08	15	7.46994	4.29894
Nc-1-IP_pe	chr1	2.42E+08	2.42E+08	269	2.42E+08	11	5.16212	3.58161
Nc-1-IP_pe	chr1	2.42E+08	2.42E+08	275	2.42E+08	15	8.26005	4.73146
Nc-1-IP_pe	chr1	2.42E+08	2.42E+08	283	2.42E+08	14	5.51541	3.4073
Nc-1-IP_pe	chr1	2.42E+08	2.42E+08	1131	2.42E+08	17	8.31233	4.43123
Nc-1-IP_pe	chr1	2.43E+08	2.43E+08	1035	2.43E+08	14	6.69566	4.03025
Nc-1-IP_pe	chr1	2.43E+08	2.43E+08	496	2.43E+08	12	6.18005	4.03623
Nc-1-IP_pe	chr1	2.43E+08	2.43E+08	564	2.43E+08	14	6.44441	3.89444
Nc-1-IP_pe	chr1	2.43E+08	2.43E+08	603	2.43E+08	17	5.54967	3.12324
Nc-1-IP_pe	chr1	2.43E+08	2.43E+08	430	2.43E+08	15	6.78905	3.93887
Nc-1-IP_pe	chr1	2.43E+08	2.43E+08	528	2.43E+08	15	7.02624	4.06292
Nc-1-IP_pe	chr1	2.43E+08	2.43E+08	304	2.43E+08	12	5.23082	3.49289
Nc-1-IP_pe	chr1	2.43E+08	2.43E+08	340	2.43E+08	16	4.17061	2.60318
Nc-1-IP_pe	chr1	2.43E+08	2.43E+08	425	2.43E+08	17	3.58449	2.30632
Nc-1-IP_pe	chr1	2.43E+08	2.43E+08	499	2.43E+08	12	5.77527	3.80164
Nc-1-IP_pe	chr1	2.43E+08	2.43E+08	357	2.43E+08	14	7.6052	4.5355
Nc-1-IP_pe	chr1	2.43E+08	2.43E+08	421	2.43E+08	15	6.19428	3.63445
Nc-1-IP_pe	chr1	2.43E+08	2.43E+08	395	2.43E+08	11	4.90416	3.43117
Nc-1-IP_pe	chr1	2.43E+08	2.43E+08	392	2.43E+08	15	3.46204	2.35851
Nc-1-IP_pe	chr1	2.43E+08	2.43E+08	505	2.43E+08	16	8.01663	4.43359
Nc-1-IP_pe	chr1	2.43E+08	2.43E+08	428	2.43E+08	16	5.82808	3.34463
Nc-1-IP_pe	chr1	2.43E+08	2.43E+08	370	2.43E+08	12	6.12485	4.004
Nc-1-IP_pe	chr1	2.43E+08	2.43E+08	333	2.43E+08	10	2.95	2.42133
Nc-1-IP_pe	chr1	2.43E+08	2.43E+08	503	2.43E+08	17	9.22305	4.9044
Nc-1-IP_pe	chr1	2.43E+08	2.43E+08	592	2.43E+08	13	6.52521	4.08375
Nc-1-IP_pe	chr1	2.43E+08	2.43E+08	288	2.43E+08	9	3.45738	2.79711
Nc-1-IP_pe	chr1	2.43E+08	2.43E+08	477	2.43E+08	14	4.95172	3.12357
Nc-1-IP_pe	chr1	2.43E+08	2.43E+08	261	2.43E+08	12	5.84275	3.84046
Nc-1-IP_pe	chr1	2.43E+08	2.43E+08	262	2.43E+08	14	7.30138	4.36445
Nc-1-IP_pe	chr1	2.43E+08	2.43E+08	443	2.43E+08	23	8.39	3.72457
Nc-1-IP_pe	chr1	2.43E+08	2.43E+08	570	2.43E+08	14	8.20556	4.87976
Nc-1-IP_pe	chr1	2.43E+08	2.43E+08	413	2.43E+08	15	4.7819	2.95039
Nc-1-IP_pe	chr1	2.43E+08	2.43E+08	466	2.43E+08	12	5.40861	3.59282
Nc-1-IP_pe	chr1	2.43E+08	2.43E+08	285	2.43E+08	26	16.0292	6.56793
Nc-1-IP_pe	chr1	2.43E+08	2.43E+08	538	2.43E+08	13	4.41954	2.952
Nc-1-IP_pe	chr1	2.43E+08	2.43E+08	855	2.43E+08	16	5.99456	3.42302
Nc-1-IP_pe	chr1	2.43E+08	2.43E+08	752	2.43E+08	19	7.59289	3.82098
Nc-1-IP_pe	chr1	2.43E+08	2.43E+08	459	2.43E+08	14	3.19897	2.29693
Nc-1-IP_pe	chr1	2.43E+08	2.43E+08	316	2.43E+08	15	5.83691	3.4562
Nc-1-IP_pe	chr1	2.43E+08	2.43E+08	309	2.43E+08	21	8.6741	4.05679
Nc-1-IP_pe	chr1	2.43E+08	2.43E+08	328	2.43E+08	17	9.20132	4.89288

Nc-1-IP_pe	chr1	2.43E+08	2.43E+08	558	2.43E+08	13	4.41954	2.952
Nc-1-IP_pe	chr1	2.43E+08	2.43E+08	899	2.43E+08	15	5.66913	3.37372
Nc-1-IP_pe	chr1	2.43E+08	2.43E+08	903	2.43E+08	17	4.78101	2.79343
Nc-1-IP_pe	chr1	2.43E+08	2.43E+08	316	2.43E+08	16	4.59224	2.7853
Nc-1-IP_pe	chr1	2.43E+08	2.43E+08	513	2.43E+08	11	4.12518	2.98817
Nc-1-IP_pe	chr1	2.43E+08	2.43E+08	455	2.43E+08	15	6.76257	3.92511
Nc-1-IP_pe	chr1	2.43E+08	2.43E+08	1516	2.43E+08	16	5.37454	3.13479
Nc-1-IP_pe	chr1	2.43E+08	2.43E+08	331	2.43E+08	17	9.09431	4.8363
Nc-1-IP_pe	chr1	2.43E+08	2.43E+08	277	2.43E+08	14	7.42849	4.43574
Nc-1-IP_pe	chr1	2.43E+08	2.43E+08	416	2.43E+08	24	6.09885	2.83276
Nc-1-IP_pe	chr1	2.43E+08	2.43E+08	306	2.43E+08	21	6.2632	3.08808
Nc-1-IP_pe	chr1	2.43E+08	2.43E+08	285	2.43E+08	12	4.75569	3.23024
Nc-1-IP_pe	chr1	2.43E+08	2.43E+08	275	2.43E+08	17	5.6711	3.17666
Nc-1-IP_pe	chr1	2.43E+08	2.43E+08	300	2.43E+08	13	4.86362	3.18015
Nc-1-IP_pe	chr1	2.43E+08	2.43E+08	278	2.43E+08	9	4.17427	3.23498
Nc-1-IP_pe	chr1	2.43E+08	2.43E+08	462	2.43E+08	15	6.5434	3.81197
Nc-1-IP_pe	chr1	2.43E+08	2.43E+08	488	2.43E+08	12	5.01934	3.37518
Nc-1-IP_pe	chr1	2.43E+08	2.43E+08	265	2.43E+08	12	4.70082	3.20033
Nc-1-IP_pe	chr1	2.43E+08	2.43E+08	512	2.43E+08	20	6.49719	3.259
Nc-1-IP_pe	chr1	2.43E+08	2.43E+08	384	2.43E+08	18	5.33197	2.94862
Nc-1-IP_pe	chr1	2.43E+08	2.43E+08	1643	2.43E+08	18	5.23669	2.90944
Nc-1-IP_pe	chr1	2.43E+08	2.43E+08	958	2.43E+08	19	5.51632	2.94814
Nc-1-IP_pe	chr1	2.43E+08	2.43E+08	599	2.43E+08	19	5.90435	3.10381
Nc-1-IP_pe	chr1	2.43E+08	2.43E+08	625	2.43E+08	15	5.20142	3.14789
Nc-1-IP_pe	chr1	2.43E+08	2.43E+08	596	2.43E+08	11	3.83891	2.82985
Nc-1-IP_pe	chr1	2.43E+08	2.43E+08	609	2.43E+08	21	8.6741	4.05679
Nc-1-IP_pe	chr1	2.43E+08	2.43E+08	549	2.43E+08	12	4.70082	3.20033
Nc-1-IP_pe	chr1	2.44E+08	2.44E+08	440	2.44E+08	12	4.24064	2.95299
Nc-1-IP_pe	chr1	2.44E+08	2.44E+08	399	2.44E+08	13	4.41954	2.952
Nc-1-IP_pe	chr1	2.44E+08	2.44E+08	285	2.44E+08	14	4.60011	2.95114
Nc-1-IP_pe	chr1	2.44E+08	2.44E+08	292	2.44E+08	15	5.70822	3.39286
Nc-1-IP_pe	chr1	2.44E+08	2.44E+08	459	2.44E+08	10	3.40382	2.67582
Nc-1-IP_pe	chr1	2.44E+08	2.44E+08	673	2.44E+08	22	11.5934	5.22453
Nc-1-IP_pe	chr1	2.44E+08	2.44E+08	331	2.44E+08	19	6.80149	3.4766
Nc-1-IP_pe	chr1	2.44E+08	2.44E+08	304	2.44E+08	17	9.94011	5.29055
Nc-1-IP_pe	chr1	2.44E+08	2.44E+08	253	2.44E+08	20	7.97282	3.87239
Nc-1-IP_pe	chr1	2.44E+08	2.44E+08	550	2.44E+08	28	11.8835	4.50052
Nc-1-IP_pe	chr1	2.44E+08	2.44E+08	559	2.44E+08	24	7.61242	3.34923
Nc-1-IP_pe	chr1	2.44E+08	2.44E+08	319	2.44E+08	10	4.18807	3.12977
Nc-1-IP_pe	chr1	2.44E+08	2.44E+08	403	2.44E+08	17	9.65211	5.13407
Nc-1-IP_pe	chr1	2.44E+08	2.44E+08	431	2.44E+08	16	7.53857	4.18505
Nc-1-IP_pe	chr1	2.44E+08	2.44E+08	564	2.44E+08	16	6.52784	3.67908
Nc-1-IP_pe	chr1	2.44E+08	2.44E+08	756	2.44E+08	16	8.39046	4.63193
Nc-1-IP_pe	chr1	2.44E+08	2.44E+08	415	2.44E+08	13	4.02562	2.7544
Nc-1-IP_pe	chr1	2.44E+08	2.44E+08	359	2.44E+08	13	5.74741	3.65119

Nc-1-IP_pe	chr1	2.44E+08	2.44E+08	258	2.44E+08	14	5.03085	3.16286
Nc-1-IP_pe	chr1	2.44E+08	2.44E+08	315	2.44E+08	22	12.6129	5.71503
Nc-1-IP_pe	chr1	2.44E+08	2.44E+08	288	2.44E+08	11	4.53066	3.21658
Nc-1-IP_pe	chr1	2.44E+08	2.44E+08	325	2.44E+08	14	6.31617	3.82577
Nc-1-IP_pe	chr1	2.44E+08	2.44E+08	491	2.44E+08	12	4.99209	3.3601
Nc-1-IP_pe	chr1	2.44E+08	2.44E+08	259	2.44E+08	12	5.25962	3.50902
Nc-1-IP_pe	chr1	2.44E+08	2.44E+08	253	2.44E+08	23	4.4731	2.35612
Nc-1-IP_pe	chr1	2.44E+08	2.44E+08	295	2.44E+08	17	4.78101	2.79343
Nc-1-IP_pe	chr1	2.44E+08	2.44E+08	461	2.44E+08	15	5.66913	3.37372
Nc-1-IP_pe	chr1	2.44E+08	2.44E+08	354	2.44E+08	13	4.86362	3.18015
Nc-1-IP_pe	chr1	2.44E+08	2.44E+08	280	2.44E+08	16	3.39261	2.27748
Nc-1-IP_pe	chr1	2.44E+08	2.44E+08	291	2.44E+08	16	8.01663	4.43359
Nc-1-IP_pe	chr1	2.44E+08	2.44E+08	347	2.44E+08	19	6.32622	3.27682
Nc-1-IP_pe	chr1	2.44E+08	2.44E+08	278	2.44E+08	17	4.78065	2.79327
Nc-1-IP_pe	chr1	2.44E+08	2.44E+08	406	2.44E+08	16	4.96462	2.94973
Nc-1-IP_pe	chr1	2.44E+08	2.44E+08	266	2.44E+08	16	4.25252	2.63824
Nc-1-IP_pe	chr1	2.44E+08	2.44E+08	301	2.44E+08	16	6.49968	3.66537
Nc-1-IP_pe	chr1	2.44E+08	2.44E+08	475	2.44E+08	15	5.4619	3.27292
Nc-1-IP_pe	chr1	2.44E+08	2.44E+08	274	2.44E+08	18	7.47506	3.88659
Nc-1-IP_pe	chr1	2.44E+08	2.44E+08	386	2.44E+08	18	7.56466	3.92825
Nc-1-IP_pe	chr1	2.44E+08	2.44E+08	321	2.44E+08	12	3.47963	2.55766
Nc-1-IP_pe	chr1	2.44E+08	2.44E+08	401	2.44E+08	15	3.74763	2.48304
Nc-1-IP_pe	chr1	2.44E+08	2.44E+08	279	2.44E+08	19	4.8268	2.67939
Nc-1-IP_pe	chr1	2.44E+08	2.44E+08	345	2.44E+08	17	3.84884	2.41145
Nc-1-IP_pe	chr1	2.44E+08	2.44E+08	332	2.44E+08	15	4.40338	2.77622
Nc-1-IP_pe	chr1	2.44E+08	2.44E+08	390	2.44E+08	12	4.743	3.22332
Nc-1-IP_pe	chr1	2.44E+08	2.44E+08	319	2.44E+08	15	4.7819	2.95039
Nc-1-IP_pe	chr1	2.44E+08	2.44E+08	581	2.44E+08	16	6.33417	3.58521
Nc-1-IP_pe	chr1	2.44E+08	2.44E+08	365	2.44E+08	17	4.78065	2.79327
Nc-1-IP_pe	chr1	2.44E+08	2.44E+08	293	2.44E+08	17	7.6273	4.08876
Nc-1-IP_pe	chr1	2.44E+08	2.44E+08	281	2.44E+08	14	4.21449	2.76599
Nc-1-IP_pe	chr1	2.44E+08	2.44E+08	501	2.44E+08	16	4.96462	2.94973
Nc-1-IP_pe	chr1	2.44E+08	2.44E+08	817	2.44E+08	14	3.55308	2.45762
Nc-1-IP_pe	chr1	2.44E+08	2.44E+08	532	2.44E+08	15	6.01011	3.54215
Nc-1-IP_pe	chr1	2.44E+08	2.44E+08	400	2.44E+08	10	3.03453	2.46828
Nc-1-IP_pe	chr1	2.44E+08	2.44E+08	277	2.44E+08	18	5.72638	3.11298
Nc-1-IP_pe	chr1	2.44E+08	2.44E+08	475	2.44E+08	18	6.87353	3.61204
Nc-1-IP_pe	chr1	2.44E+08	2.44E+08	387	2.44E+08	16	7.53857	4.18505
Nc-1-IP_pe	chr1	2.44E+08	2.44E+08	277	2.44E+08	16	10.2074	5.6414
Nc-1-IP_pe	chr1	2.44E+08	2.44E+08	469	2.44E+08	16	4.25252	2.63824
Nc-1-IP_pe	chr1	2.44E+08	2.44E+08	730	2.44E+08	26	9.86937	3.97999
Nc-1-IP_pe	chr1	2.44E+08	2.44E+08	336	2.44E+08	16	4.25252	2.63824
Nc-1-IP_pe	chr1	2.44E+08	2.44E+08	416	2.44E+08	11	4.06403	2.95415
Nc-1-IP_pe	chr1	2.44E+08	2.44E+08	295	2.44E+08	11	5.10577	3.5486
Nc-1-IP_pe	chr1	2.44E+08	2.44E+08	273	2.44E+08	23	6.25615	2.94664

Nc-1-IP_pe	chr1	2.44E+08	2.44E+08	578	2.44E+08	19	4.97584	2.73666
Nc-1-IP_pe	chr1	2.44E+08	2.44E+08	293	2.44E+08	21	13.0803	6.15364
Nc-1-IP_pe	chr1	2.45E+08	2.45E+08	561	2.45E+08	13	4.56123	3.02417
Nc-1-IP_pe	chr1	2.45E+08	2.45E+08	289	2.45E+08	14	4.60011	2.95114
Nc-1-IP_pe	chr1	2.45E+08	2.45E+08	326	2.45E+08	20	12.9463	6.30032
Nc-1-IP_pe	chr1	2.45E+08	2.45E+08	487	2.45E+08	18	8.80785	4.52672
Nc-1-IP_pe	chr1	2.45E+08	2.45E+08	1001	2.45E+08	12	5.77527	3.80164
Nc-1-IP_pe	chr1	2.45E+08	2.45E+08	464	2.45E+08	19	7.45276	3.75892
Nc-1-IP_pe	chr1	2.45E+08	2.45E+08	279	2.45E+08	10	3.71867	2.85592
Nc-1-IP_pe	chr1	2.45E+08	2.45E+08	342	2.45E+08	13	4.41954	2.952
Nc-1-IP_pe	chr1	2.45E+08	2.45E+08	453	2.45E+08	16	7.53857	4.18505
Nc-1-IP_pe	chr1	2.45E+08	2.45E+08	599	2.45E+08	12	4.63088	3.16234
Nc-1-IP_pe	chr1	2.45E+08	2.45E+08	285	2.45E+08	13	5.40091	3.46384
Nc-1-IP_pe	chr1	2.45E+08	2.45E+08	259	2.45E+08	16	7.11257	3.96852
Nc-1-IP_pe	chr1	2.45E+08	2.45E+08	369	2.45E+08	13	4.47401	2.97968
Nc-1-IP_pe	chr1	2.45E+08	2.45E+08	1254	2.45E+08	17	6.64406	3.61808
Nc-1-IP_pe	chr1	2.45E+08	2.45E+08	417	2.45E+08	16	4.96462	2.94973
Nc-1-IP_pe	chr1	2.45E+08	2.45E+08	582	2.45E+08	18	6.63038	3.50359
Nc-1-IP_pe	chr1	2.45E+08	2.45E+08	344	2.45E+08	15	4.70031	2.91253
Nc-1-IP_pe	chr1	2.45E+08	2.45E+08	827	2.45E+08	15	3.74763	2.48304
Nc-1-IP_pe	chr1	2.45E+08	2.45E+08	257	2.45E+08	10	3.4604	2.70797
Nc-1-IP_pe	chr1	2.45E+08	2.45E+08	627	2.45E+08	15	5.58327	3.33181
Nc-1-IP_pe	chr1	2.45E+08	2.45E+08	569	2.45E+08	16	6.1093	3.47747
Nc-1-IP_pe	chr1	2.45E+08	2.45E+08	728	2.45E+08	15	6.43758	3.75782
Nc-1-IP_pe	chr1	2.45E+08	2.45E+08	267	2.45E+08	17	4.78101	2.79343
Nc-1-IP_pe	chr1	2.45E+08	2.45E+08	301	2.45E+08	15	6.8109	3.95023
Nc-1-IP_pe	chr1	2.45E+08	2.45E+08	499	2.45E+08	20	7.99918	3.88379
Nc-1-IP_pe	chr1	2.45E+08	2.45E+08	1066	2.45E+08	21	10.7603	4.99737
Nc-1-IP_pe	chr1	2.45E+08	2.45E+08	580	2.45E+08	13	5.47064	3.50127
Nc-1-IP_pe	chr1	2.45E+08	2.45E+08	378	2.45E+08	16	4.25252	2.63824
Nc-1-IP_pe	chr1	2.45E+08	2.45E+08	439	2.45E+08	14	4.60011	2.95114
Nc-1-IP_pe	chr1	2.45E+08	2.45E+08	291	2.45E+08	10	3.08907	2.49869
Nc-1-IP_pe	chr1	2.45E+08	2.45E+08	573	2.45E+08	21	6.2632	3.08808
Nc-1-IP_pe	chr1	2.45E+08	2.45E+08	273	2.45E+08	11	4.90416	3.43117
Nc-1-IP_pe	chr1	2.45E+08	2.45E+08	332	2.45E+08	29	6.41921	2.67691
Nc-1-IP_pe	chr1	2.45E+08	2.45E+08	329	2.45E+08	17	5.14803	2.94914
Nc-1-IP_pe	chr1	2.45E+08	2.45E+08	254	2.45E+08	14	4.60011	2.95114
Nc-1-IP_pe	chr1	2.45E+08	2.45E+08	399	2.45E+08	14	4.21449	2.76599
Nc-1-IP_pe	chr1	2.45E+08	2.45E+08	593	2.45E+08	13	3.67377	2.58159
Nc-1-IP_pe	chr1	2.45E+08	2.45E+08	370	2.45E+08	15	3.46204	2.35851
Nc-1-IP_pe	chr1	2.45E+08	2.45E+08	563	2.45E+08	14	6.37983	3.8598
Nc-1-IP_pe	chr1	2.45E+08	2.45E+08	551	2.45E+08	18	7.73136	4.00629
Nc-1-IP_pe	chr1	2.45E+08	2.45E+08	291	2.45E+08	15	4.7819	2.95039
Nc-1-IP_pe	chr1	2.45E+08	2.45E+08	289	2.45E+08	22	7.7408	3.56938
Nc-1-IP_pe	chr1	2.45E+08	2.45E+08	563	2.45E+08	12	3.83688	2.74114

Nc-1-IP_pe	chr1	2.45E+08	2.45E+08	874	2.45E+08	16	5.82808	3.34463
Nc-1-IP_pe	chr1	2.45E+08	2.45E+08	481	2.45E+08	11	5.03835	3.5092
Nc-1-IP_pe	chr1	2.45E+08	2.45E+08	301	2.45E+08	16	8.26996	4.56762
Nc-1-IP_pe	chr1	2.45E+08	2.45E+08	376	2.45E+08	17	9.00934	4.79158
Nc-1-IP_pe	chr1	2.45E+08	2.45E+08	748	2.45E+08	17	4.13464	2.52661
Nc-1-IP_pe	chr1	2.45E+08	2.45E+08	298	2.45E+08	19	5.90435	3.10381
Nc-1-IP_pe	chr1	2.45E+08	2.45E+08	463	2.45E+08	11	3.49235	2.64145
Nc-1-IP_pe	chr1	2.45E+08	2.45E+08	456	2.45E+08	15	7.63952	4.39047
Nc-1-IP_pe	chr1	2.45E+08	2.45E+08	297	2.45E+08	15	7.02624	4.06292
Nc-1-IP_pe	chr1	2.45E+08	2.45E+08	265	2.45E+08	15	6.82712	3.95868
Nc-1-IP_pe	chr1	2.45E+08	2.45E+08	292	2.45E+08	16	4.96462	2.94973
Nc-1-IP_pe	chr1	2.45E+08	2.45E+08	1210	2.45E+08	20	4.71088	2.57831
Nc-1-IP_pe	chr1	2.45E+08	2.45E+08	609	2.45E+08	18	6.52861	3.45863
Nc-1-IP_pe	chr1	2.45E+08	2.45E+08	420	2.45E+08	22	6.83044	3.22338
Nc-1-IP_pe	chr1	2.45E+08	2.45E+08	631	2.45E+08	20	7.37456	3.61783
Nc-1-IP_pe	chr1	2.45E+08	2.45E+08	472	2.45E+08	15	4.49799	2.8194
Nc-1-IP_pe	chr1	2.45E+08	2.45E+08	381	2.45E+08	18	5.83846	3.16035
Nc-1-IP_pe	chr1	2.45E+08	2.45E+08	307	2.45E+08	22	10.5348	4.73832
Nc-1-IP_pe	chr1	2.45E+08	2.45E+08	337	2.45E+08	20	7.85753	3.8227
Nc-1-IP_pe	chr1	2.45E+08	2.45E+08	465	2.45E+08	21	8.6741	4.05679
Nc-1-IP_pe	chr1	2.45E+08	2.45E+08	447	2.45E+08	22	7.48744	3.47143
Nc-1-IP_pe	chr1	2.45E+08	2.45E+08	699	2.45E+08	34	5.80432	2.34122
Nc-1-IP_pe	chr1	2.45E+08	2.45E+08	270	2.45E+08	16	3.53753	2.33721
Nc-1-IP_pe	chr1	2.45E+08	2.45E+08	310	2.45E+08	17	2.90036	2.03959
Nc-1-IP_pe	chr1	2.45E+08	2.45E+08	346	2.45E+08	18	5.3798	2.96836
Nc-1-IP_pe	chr1	2.45E+08	2.45E+08	324	2.45E+08	15	4.45138	2.79809
Nc-1-IP_pe	chr1	2.45E+08	2.45E+08	399	2.45E+08	18	4.96968	2.80074
Nc-1-IP_pe	chr1	2.45E+08	2.45E+08	288	2.45E+08	19	6.56181	3.3752
Nc-1-IP_pe	chr1	2.45E+08	2.45E+08	376	2.45E+08	17	4.78101	2.79343
Nc-1-IP_pe	chr1	2.45E+08	2.45E+08	476	2.45E+08	17	4.13464	2.52661
Nc-1-IP_pe	chr1	2.45E+08	2.45E+08	562	2.45E+08	20	4.42487	2.47493
Nc-1-IP_pe	chr1	2.45E+08	2.45E+08	447	2.45E+08	19	3.71826	2.26621
Nc-1-IP_pe	chr1	2.45E+08	2.45E+08	339	2.45E+08	24	4.66132	2.37496
Nc-1-IP_pe	chr1	2.45E+08	2.45E+08	295	2.45E+08	17	5.14803	2.94914
Nc-1-IP_pe	chr1	2.45E+08	2.45E+08	333	2.45E+08	21	4.90204	2.59278
Nc-1-IP_pe	chr1	2.45E+08	2.45E+08	464	2.45E+08	22	5.09279	2.60614
Nc-1-IP_pe	chr1	2.45E+08	2.45E+08	269	2.45E+08	24	9.47843	4.04216
Nc-1-IP_pe	chr1	2.45E+08	2.45E+08	344	2.45E+08	25	5.1059	2.46996
Nc-1-IP_pe	chr1	2.45E+08	2.45E+08	260	2.45E+08	29	5.66014	2.46968
Nc-1-IP_pe	chr1	2.45E+08	2.45E+08	281	2.45E+08	29	6.1329	2.59804
Nc-1-IP_pe	chr1	2.45E+08	2.45E+08	372	2.45E+08	30	9.06325	3.38223
Nc-1-IP_pe	chr1	2.45E+08	2.45E+08	1270	2.45E+08	28	6.23052	2.6687
Nc-1-IP_pe	chr1	2.45E+08	2.45E+08	712	2.45E+08	19	5.90394	3.10364
Nc-1-IP_pe	chr1	2.45E+08	2.45E+08	485	2.45E+08	17	5.61655	3.15262
Nc-1-IP_pe	chr1	2.45E+08	2.45E+08	277	2.45E+08	19	6.78661	3.47027

Nc-1-IP_pe	chr1	2.45E+08	2.45E+08	366	2.45E+08	24	9.61644	4.09603
Nc-1-IP_pe	chr1	2.45E+08	2.45E+08	588	2.45E+08	17	5.47777	3.09179
Nc-1-IP_pe	chr1	2.45E+08	2.45E+08	258	2.45E+08	20	6.28238	3.1737
Nc-1-IP_pe	chr1	2.45E+08	2.45E+08	313	2.45E+08	23	10.1326	4.42559
Nc-1-IP_pe	chr1	2.45E+08	2.45E+08	541	2.45E+08	18	6.15736	3.29676
Nc-1-IP_pe	chr1	2.45E+08	2.45E+08	397	2.45E+08	14	5.5512	3.42562
Nc-1-IP_pe	chr1	2.45E+08	2.45E+08	492	2.45E+08	17	5.9911	3.31919
Nc-1-IP_pe	chr1	2.45E+08	2.45E+08	318	2.45E+08	19	7.84735	3.93486
Nc-1-IP_pe	chr1	2.45E+08	2.45E+08	270	2.45E+08	13	3.35769	2.42919
Nc-1-IP_pe	chr1	2.45E+08	2.45E+08	1013	2.45E+08	15	5.20142	3.14789
Nc-1-IP_pe	chr1	2.45E+08	2.45E+08	316	2.45E+08	19	8.4638	4.21715
Nc-1-IP_pe	chr1	2.45E+08	2.45E+08	261	2.45E+08	10	4.57677	3.36116
Nc-1-IP_pe	chr1	2.45E+08	2.45E+08	712	2.45E+08	16	5.73763	3.30235
Nc-1-IP_pe	chr1	2.45E+08	2.45E+08	300	2.45E+08	10	3.89064	2.95552
Nc-1-IP_pe	chr1	2.45E+08	2.45E+08	368	2.45E+08	13	4.86362	3.18015
Nc-1-IP_pe	chr1	2.45E+08	2.45E+08	299	2.45E+08	9	3.59586	2.88066
Nc-1-IP_pe	chr1	2.45E+08	2.45E+08	298	2.45E+08	13	5.36853	3.44651
Nc-1-IP_pe	chr1	2.45E+08	2.45E+08	331	2.45E+08	15	5.96953	3.52194
Nc-1-IP_pe	chr1	2.45E+08	2.45E+08	300	2.45E+08	12	4.63088	3.16234
Nc-1-IP_pe	chr1	2.45E+08	2.45E+08	638	2.45E+08	10	4.09813	3.07682
Nc-1-IP_pe	chr1	2.45E+08	2.45E+08	438	2.45E+08	14	4.60011	2.95114
Nc-1-IP_pe	chr1	2.45E+08	2.45E+08	349	2.45E+08	12	5.92876	3.89011
Nc-1-IP_pe	chr1	2.45E+08	2.45E+08	276	2.45E+08	15	6.19428	3.63445
Nc-1-IP_pe	chr1	2.45E+08	2.45E+08	444	2.45E+08	13	6.62436	4.14003
Nc-1-IP_pe	chr1	2.45E+08	2.45E+08	317	2.45E+08	11	4.54404	3.2242
Nc-1-IP_pe	chr1	2.45E+08	2.45E+08	284	2.45E+08	15	8.26005	4.73146
Nc-1-IP_pe	chr1	2.45E+08	2.45E+08	261	2.45E+08	12	6.56855	4.26509
Nc-1-IP_pe	chr1	2.45E+08	2.45E+08	403	2.45E+08	12	4.1728	2.91706
Nc-1-IP_pe	chr1	2.45E+08	2.45E+08	261	2.45E+08	11	4.06403	2.95415
Nc-1-IP_pe	chr1	2.46E+08	2.46E+08	328	2.46E+08	18	4.04135	2.43445
Nc-1-IP_pe	chr1	2.46E+08	2.46E+08	326	2.46E+08	17	6.47874	3.54137
Nc-1-IP_pe	chr1	2.46E+08	2.46E+08	392	2.46E+08	16	6.8987	3.86161
Nc-1-IP_pe	chr1	2.46E+08	2.46E+08	1121	2.46E+08	19	9.15219	4.54307
Nc-1-IP_pe	chr1	2.46E+08	2.46E+08	681	2.46E+08	16	6.8987	3.86161
Nc-1-IP_pe	chr1	2.46E+08	2.46E+08	353	2.46E+08	11	3.58722	2.69267
Nc-1-IP_pe	chr1	2.46E+08	2.46E+08	440	2.46E+08	13	6.31249	3.96385
Nc-1-IP_pe	chr1	2.46E+08	2.46E+08	358	2.46E+08	10	4.25398	3.16873
Nc-1-IP_pe	chr1	2.46E+08	2.46E+08	427	2.46E+08	16	6.33285	3.58457
Nc-1-IP_pe	chr1	2.46E+08	2.46E+08	760	2.46E+08	12	5.84943	3.84431
Nc-1-IP_pe	chr1	2.46E+08	2.46E+08	323	2.46E+08	15	6.5434	3.81197
Nc-1-IP_pe	chr1	2.46E+08	2.46E+08	343	2.46E+08	17	9.09431	4.8363
Nc-1-IP_pe	chr1	2.46E+08	2.46E+08	559	2.46E+08	13	6.19316	3.89711
Nc-1-IP_pe	chr1	2.46E+08	2.46E+08	835	2.46E+08	14	5.03085	3.16286
Nc-1-IP_pe	chr1	2.46E+08	2.46E+08	440	2.46E+08	13	4.86362	3.18015
Nc-1-IP_pe	chr1	2.46E+08	2.46E+08	426	2.46E+08	13	5.22525	3.37017

Nc-1-IP_pe	chr1	2.46E+08	2.46E+08	532	2.46E+08	14	4.21449	2.76599
Nc-1-IP_pe	chr1	2.46E+08	2.46E+08	500	2.46E+08	15	5.96953	3.52194
Nc-1-IP_pe	chr1	2.46E+08	2.46E+08	638	2.46E+08	13	5.29187	3.40559
Nc-1-IP_pe	chr1	2.46E+08	2.46E+08	516	2.46E+08	15	4.60262	2.86743
Nc-1-IP_pe	chr1	2.46E+08	2.46E+08	550	2.46E+08	18	4.96968	2.80074
Nc-1-IP_pe	chr1	2.46E+08	2.46E+08	259	2.46E+08	17	5.14803	2.94914
Nc-1-IP_pe	chr1	2.46E+08	2.46E+08	415	2.46E+08	14	5.14959	3.22214
Nc-1-IP_pe	chr1	2.46E+08	2.46E+08	579	2.46E+08	14	4.60011	2.95114
Nc-1-IP_pe	chr1	2.46E+08	2.46E+08	358	2.46E+08	19	6.78661	3.47027
Nc-1-IP_pe	chr1	2.46E+08	2.46E+08	418	2.46E+08	14	3.79446	2.56886
Nc-1-IP_pe	chr1	2.46E+08	2.46E+08	271	2.46E+08	13	6.58008	4.11487
Nc-1-IP_pe	chr1	2.46E+08	2.46E+08	703	2.46E+08	21	7.58657	3.60451
Nc-1-IP_pe	chr1	2.46E+08	2.46E+08	255	2.46E+08	16	4.96462	2.94973
Nc-1-IP_pe	chr1	2.46E+08	2.46E+08	376	2.46E+08	13	5.36853	3.44651
Nc-1-IP_pe	chr1	2.46E+08	2.46E+08	323	2.46E+08	12	5.23082	3.49289
Nc-1-IP_pe	chr1	2.46E+08	2.46E+08	419	2.46E+08	15	5.34394	3.21607
Nc-1-IP_pe	chr1	2.46E+08	2.46E+08	487	2.46E+08	10	4.16209	3.11446
Nc-1-IP_pe	chr1	2.46E+08	2.46E+08	258	2.46E+08	10	3.50482	2.73328
Nc-1-IP_pe	chr1	2.46E+08	2.46E+08	366	2.46E+08	14	5.03085	3.16286
Nc-1-IP_pe	chr1	2.46E+08	2.46E+08	314	2.46E+08	10	4.20113	3.13749
Nc-1-IP_pe	chr1	2.46E+08	2.46E+08	294	2.46E+08	12	3.83688	2.74114
Nc-1-IP_pe	chr1	2.46E+08	2.46E+08	495	2.46E+08	14	4.21449	2.76599
Nc-1-IP_pe	chr1	2.46E+08	2.46E+08	569	2.46E+08	48	22.3274	5.55221
Nc-1-IP_pe	chr1	2.46E+08	2.46E+08	382	2.46E+08	9	3.09155	2.57893
Nc-1-IP_pe	chr1	2.46E+08	2.46E+08	562	2.46E+08	14	6.06519	3.69269
Nc-1-IP_pe	chr1	2.46E+08	2.46E+08	471	2.46E+08	14	6.23788	3.78407
Nc-1-IP_pe	chr1	2.46E+08	2.46E+08	546	2.46E+08	10	3.08907	2.49869
Nc-1-IP_pe	chr1	2.46E+08	2.46E+08	323	2.46E+08	11	3.78682	2.80131
Nc-1-IP_pe	chr1	2.46E+08	2.46E+08	519	2.46E+08	10	3.8436	2.92819
Nc-1-IP_pe	chr1	2.46E+08	2.46E+08	291	2.46E+08	12	4.51041	3.09723
Nc-1-IP_pe	chr1	2.46E+08	2.46E+08	325	2.46E+08	15	5.20142	3.14789
Nc-1-IP_pe	chr1	2.46E+08	2.46E+08	640	2.46E+08	18	7.04825	3.69086
Nc-1-IP_pe	chr1	2.46E+08	2.46E+08	526	2.46E+08	11	4.05671	2.95009
Nc-1-IP_pe	chr1	2.46E+08	2.46E+08	255	2.46E+08	13	4.41954	2.952
Nc-1-IP_pe	chr1	2.46E+08	2.46E+08	301	2.46E+08	17	7.6273	4.08876
Nc-1-IP_pe	chr1	2.46E+08	2.46E+08	450	2.46E+08	11	4.06403	2.95415
Nc-1-IP_pe	chr1	2.46E+08	2.46E+08	515	2.46E+08	15	4.06016	2.62146
Nc-1-IP_pe	chr1	2.46E+08	2.46E+08	278	2.46E+08	13	4.41954	2.952
Nc-1-IP_pe	chr1	2.46E+08	2.46E+08	322	2.46E+08	17	8.9884	4.78058
Nc-1-IP_pe	chr1	2.46E+08	2.46E+08	273	2.46E+08	12	3.16137	2.39719
Nc-1-IP_pe	chr1	2.46E+08	2.46E+08	352	2.46E+08	15	4.90347	3.00714
Nc-1-IP_pe	chr1	2.46E+08	2.46E+08	344	2.46E+08	34	15.0468	4.91286
Nc-1-IP_pe	chr1	2.46E+08	2.46E+08	777	2.46E+08	18	5.72638	3.11298
Nc-1-IP_pe	chr1	2.46E+08	2.46E+08	586	2.46E+08	16	7.53857	4.18505
Nc-1-IP_pe	chr1	2.46E+08	2.46E+08	319	2.46E+08	17	9.55913	5.08395

Nc-1-IP_pe	chr1	2.46E+08	2.46E+08	743	2.46E+08	16	5.50606	3.19508
Nc-1-IP_pe	chr1	2.46E+08	2.46E+08	340	2.46E+08	17	8.7831	4.67333
Nc-1-IP_pe	chr1	2.47E+08	2.47E+08	831	2.47E+08	24	9.19393	3.93228
Nc-1-IP_pe	chr1	2.47E+08	2.47E+08	393	2.47E+08	15	4.60262	2.86743
Nc-1-IP_pe	chr1	2.47E+08	2.47E+08	465	2.47E+08	17	4.13464	2.52661
Nc-1-IP_pe	chr1	2.47E+08	2.47E+08	737	2.47E+08	17	5.54967	3.12324
Nc-1-IP_pe	chr1	2.47E+08	2.47E+08	662	2.47E+08	16	5.77344	3.31907
Nc-1-IP_pe	chr1	2.47E+08	2.47E+08	765	2.47E+08	16	5.80955	3.33595
Nc-1-IP_pe	chr1	2.47E+08	2.47E+08	334	2.47E+08	17	6.31808	3.4675
Nc-1-IP_pe	chr1	2.47E+08	2.47E+08	703	2.47E+08	18	7.40151	3.85254
Nc-1-IP_pe	chr1	2.47E+08	2.47E+08	501	2.47E+08	16	4.25252	2.63824
Nc-1-IP_pe	chr1	2.47E+08	2.47E+08	314	2.47E+08	10	3.16691	2.54222
Nc-1-IP_pe	chr1	2.47E+08	2.47E+08	673	2.47E+08	15	5.20142	3.14789
Nc-1-IP_pe	chr1	2.47E+08	2.47E+08	350	2.47E+08	12	5.01934	3.37518
Nc-1-IP_pe	chr1	2.47E+08	2.47E+08	967	2.47E+08	22	6.44373	3.08129
Nc-1-IP_pe	chr1	2.47E+08	2.47E+08	265	2.47E+08	17	7.6273	4.08876
Nc-1-IP_pe	chr1	2.47E+08	2.47E+08	377	2.47E+08	16	8.85845	4.88496
Nc-1-IP_pe	chr1	2.47E+08	2.47E+08	649	2.47E+08	14	3.26761	2.32785
Nc-1-IP_pe	chr1	2.47E+08	2.47E+08	347	2.47E+08	13	3.52389	2.509
Nc-1-IP_pe	chr1	2.47E+08	2.47E+08	261	2.47E+08	23	8.39	3.72457
Nc-1-IP_pe	chr1	2.47E+08	2.47E+08	334	2.47E+08	16	6.33285	3.58457
Nc-1-IP_pe	chr1	2.47E+08	2.47E+08	287	2.47E+08	11	4.10219	2.97537
Nc-1-IP_pe	chr1	2.47E+08	2.47E+08	564	2.47E+08	17	8.45033	4.50163
Nc-1-IP_pe	chr1	2.47E+08	2.47E+08	430	2.47E+08	15	7.48816	4.30874
Nc-1-IP_pe	chr1	2.47E+08	2.47E+08	282	2.47E+08	14	6.19157	3.75949
Nc-1-IP_pe	chr1	2.47E+08	2.47E+08	841	2.47E+08	16	7.8853	4.36474
Nc-1-IP_pe	chr1	2.47E+08	2.47E+08	438	2.47E+08	17	3.33947	2.20998
Nc-1-IP_pe	chr1	2.47E+08	2.47E+08	309	2.47E+08	15	4.7819	2.95039
Nc-1-IP_pe	chr1	2.47E+08	2.47E+08	1245	2.47E+08	17	7.83935	4.1935
Nc-1-IP_pe	chr1	2.47E+08	2.47E+08	409	2.47E+08	11	3.9787	2.90686
Nc-1-IP_pe	chr1	2.47E+08	2.47E+08	498	2.47E+08	13	4.96982	3.23556
Nc-1-IP_pe	chr1	2.47E+08	2.47E+08	310	2.47E+08	15	3.46204	2.35851
Nc-1-IP_pe	chr1	2.47E+08	2.47E+08	606	2.47E+08	20	10.6431	5.10839
Nc-1-IP_pe	chr1	2.47E+08	2.47E+08	575	2.47E+08	13	3.35769	2.42919
Nc-1-IP_pe	chr1	2.47E+08	2.47E+08	300	2.47E+08	12	5.33338	3.55042
Nc-1-IP_pe	chr1	2.47E+08	2.47E+08	264	2.47E+08	14	5.838	3.57373
Nc-1-IP_pe	chr1	2.47E+08	2.47E+08	553	2.47E+08	12	4.63088	3.16234
Nc-1-IP_pe	chr1	2.47E+08	2.47E+08	452	2.47E+08	13	4.34711	2.91533
Nc-1-IP_pe	chr1	2.47E+08	2.47E+08	347	2.47E+08	9	3.12089	2.59629
Nc-1-IP_pe	chr1	2.47E+08	2.47E+08	578	2.47E+08	16	4.59224	2.7853
Nc-1-IP_pe	chr1	2.47E+08	2.47E+08	268	2.47E+08	16	6.54198	3.68597
Nc-1-IP_pe	chr1	2.47E+08	2.47E+08	321	2.47E+08	18	4.96968	2.80074
Nc-1-IP_pe	chr1	2.47E+08	2.47E+08	302	2.47E+08	77	11.7773	2.41476
Nc-1-IP_pe	chr1	2.47E+08	2.47E+08	383	2.47E+08	15	3.98281	2.58698
Nc-1-IP_pe	chr1	2.47E+08	2.47E+08	263	2.47E+08	20	7.06192	3.48801

Nc-1-IP_pe	chr1	2.47E+08	2.47E+08	721	2.47E+08	19	6.16758	3.2113
Nc-1-IP_pe	chr1	2.47E+08	2.47E+08	439	2.47E+08	21	6.66991	3.24296
Nc-1-IP_pe	chr1	2.47E+08	2.47E+08	281	2.47E+08	16	4.25252	2.63824
Nc-1-IP_pe	chr1	2.47E+08	2.47E+08	330	2.47E+08	23	7.65509	3.44702
Nc-1-IP_pe	chr1	2.47E+08	2.47E+08	495	2.47E+08	18	5.83846	3.16035
Nc-1-IP_pe	chr1	2.47E+08	2.47E+08	942	2.47E+08	18	6.28754	3.35314
Nc-1-IP_pe	chr1	2.47E+08	2.47E+08	288	2.47E+08	11	2.98297	2.37087
Nc-1-IP_pe	chr1	2.47E+08	2.47E+08	813	2.47E+08	17	5.6711	3.17666
Nc-1-IP_pe	chr1	2.47E+08	2.47E+08	285	2.47E+08	16	6.8987	3.86161
Nc-1-IP_pe	chr1	2.47E+08	2.47E+08	300	2.47E+08	12	4.25953	2.96302
Nc-1-IP_pe	chr1	2.47E+08	2.47E+08	275	2.47E+08	11	3.58722	2.69267
Nc-1-IP_pe	chr1	2.47E+08	2.47E+08	365	2.47E+08	13	6.62436	4.14003
Nc-1-IP_pe	chr1	2.47E+08	2.47E+08	445	2.47E+08	12	2.87625	2.25568
Nc-1-IP_pe	chr1	2.47E+08	2.47E+08	438	2.47E+08	17	7.02067	3.79543
Nc-1-IP_pe	chr1	2.47E+08	2.47E+08	377	2.47E+08	14	7.58433	4.52368
Nc-1-IP_pe	chr1	2.47E+08	2.47E+08	373	2.47E+08	16	9.87887	5.45373
Nc-1-IP_pe	chr1	2.47E+08	2.47E+08	371	2.47E+08	14	4.21449	2.76599
Nc-1-IP_pe	chr1	2.47E+08	2.47E+08	511	2.47E+08	11	3.64839	2.72584
Nc-1-IP_pe	chr1	2.47E+08	2.47E+08	264	2.47E+08	20	7.43645	3.64378
Nc-1-IP_pe	chr1	2.47E+08	2.47E+08	421	2.47E+08	15	5.00915	3.05678
Nc-1-IP_pe	chr1	2.47E+08	2.47E+08	295	2.47E+08	12	3.83688	2.74114
Nc-1-IP_pe	chr1	2.47E+08	2.47E+08	271	2.47E+08	16	7.94118	4.39398
Nc-1-IP_pe	chr1	2.47E+08	2.47E+08	322	2.47E+08	15	5.66913	3.37372
Nc-1-IP_pe	chr1	2.47E+08	2.47E+08	341	2.47E+08	12	3.47963	2.55766
Nc-1-IP_pe	chr1	2.47E+08	2.47E+08	271	2.47E+08	15	6.23304	3.654
Nc-1-IP_pe	chr1	2.47E+08	2.47E+08	675	2.47E+08	24	14.138	6.06866
Nc-1-IP_pe	chr1	2.47E+08	2.47E+08	352	2.47E+08	19	9.15219	4.54307
Nc-1-IP_pe	chr1	2.47E+08	2.47E+08	419	2.47E+08	11	5.10577	3.5486
Nc-1-IP_pe	chr1	2.47E+08	2.47E+08	345	2.47E+08	20	10.7672	5.16976
Nc-1-IP_pe	chr1	2.47E+08	2.47E+08	314	2.47E+08	15	5.66913	3.37372
Nc-1-IP_pe	chr1	2.47E+08	2.47E+08	254	2.47E+08	14	5.51541	3.4073
Nc-1-IP_pe	chr1	2.47E+08	2.47E+08	369	2.47E+08	17	3.84884	2.41145
Nc-1-IP_pe	chr1	2.47E+08	2.47E+08	609	2.47E+08	15	3.46204	2.35851
Nc-1-IP_pe	chr1	2.47E+08	2.47E+08	792	2.47E+08	21	5.5349	2.81884
Nc-1-IP_pe	chr1	2.47E+08	2.47E+08	413	2.47E+08	12	4.63088	3.16234
Nc-1-IP_pe	chr1	2.47E+08	2.47E+08	1111	2.47E+08	16	6.24025	3.54005
Nc-1-IP_pe	chr1	2.47E+08	2.47E+08	1092	2.47E+08	22	7.7403	3.56918
Nc-1-IP_pe	chr1	2.47E+08	2.47E+08	385	2.47E+08	15	5.66913	3.37372
Nc-1-IP_pe	chr1	2.47E+08	2.47E+08	675	2.47E+08	16	6.33285	3.58457
Nc-1-IP_pe	chr1	2.47E+08	2.47E+08	320	2.47E+08	18	7.1522	3.73811
Nc-1-IP_pe	chr1	2.47E+08	2.47E+08	734	2.47E+08	14	3.00733	2.21111
Nc-1-IP_pe	chr1	2.47E+08	2.47E+08	288	2.47E+08	16	7.95994	4.40382
Nc-1-IP_pe	chr1	2.47E+08	2.47E+08	696	2.47E+08	20	10.6431	5.10839
Nc-1-IP_pe	chr1	2.47E+08	2.47E+08	531	2.47E+08	24	7.61242	3.34923
Nc-1-IP_pe	chr1	2.47E+08	2.47E+08	914	2.47E+08	17	4.44438	2.65333

Nc-1-IP_pe	chr1	2.47E+08	2.47E+08	968	2.47E+08	18	5.33197	2.94862
Nc-1-IP_pe	chr1	2.47E+08	2.47E+08	410	2.47E+08	18	8.05251	4.15857
Nc-1-IP_pe	chr1	2.47E+08	2.47E+08	639	2.47E+08	17	5.9911	3.31919
Nc-1-IP_pe	chr1	2.47E+08	2.47E+08	448	2.47E+08	24	5.18704	2.5391
Nc-1-IP_pe	chr1	2.47E+08	2.47E+08	1089	2.47E+08	24	9.05596	3.87955
Nc-1-IP_pe	chr1	2.47E+08	2.47E+08	567	2.47E+08	18	7.18674	3.75388
Nc-1-IP_pe	chr1	2.47E+08	2.47E+08	722	2.47E+08	21	8.10596	3.8173
Nc-1-IP_pe	chr1	2.47E+08	2.47E+08	367	2.47E+08	17	8.02964	4.28847
Nc-1-IP_pe	chr1	2.47E+08	2.47E+08	366	2.47E+08	23	11.6559	5.08929
Nc-1-IP_pe	chr1	2.47E+08	2.47E+08	301	2.47E+08	13	6.62436	4.14003
Nc-1-IP_pe	chr1	2.47E+08	2.47E+08	347	2.47E+08	13	4.86362	3.18015
Nc-1-IP_pe	chr1	2.47E+08	2.47E+08	373	2.47E+08	13	5.29187	3.40559
Nc-1-IP_pe	chr1	2.47E+08	2.47E+08	494	2.47E+08	22	6.84408	3.22845
Nc-1-IP_pe	chr1	2.47E+08	2.47E+08	282	2.47E+08	18	9.94172	5.10499
Nc-1-IP_pe	chr1	2.47E+08	2.47E+08	582	2.47E+08	23	7.01947	3.21526
Nc-1-IP_pe	chr1	2.47E+08	2.47E+08	265	2.47E+08	13	6.62436	4.14003
Nc-1-IP_pe	chr1	2.47E+08	2.47E+08	345	2.47E+08	13	5.57038	3.55505
Nc-1-IP_pe	chr1	2.47E+08	2.47E+08	492	2.47E+08	16	6.65665	3.74206
Nc-1-IP_pe	chr1	2.47E+08	2.47E+08	1177	2.47E+08	15	6.77862	3.93345
Nc-1-IP_pe	chr1	2.47E+08	2.47E+08	978	2.47E+08	13	5.35966	3.44177
Nc-1-IP_pe	chr1	2.47E+08	2.47E+08	263	2.47E+08	12	4.51041	3.09723
Nc-1-IP_pe	chr1	2.47E+08	2.47E+08	554	2.47E+08	15	5.22925	3.16116
Nc-1-IP_pe	chr1	2.47E+08	2.47E+08	589	2.47E+08	12	2.87625	2.25568
Nc-1-IP_pe	chr1	2.47E+08	2.47E+08	293	2.47E+08	17	6.76692	3.67553
Nc-1-IP_pe	chr1	2.47E+08	2.47E+08	397	2.47E+08	14	4.73128	3.01506
Nc-1-IP_pe	chr1	2.47E+08	2.47E+08	526	2.47E+08	18	7.56466	3.92825
Nc-1-IP_pe	chr1	2.47E+08	2.47E+08	1715	2.47E+08	16	5.37454	3.13479
Nc-1-IP_pe	chr1	2.47E+08	2.47E+08	291	2.47E+08	14	5.98179	3.64885
Nc-1-IP_pe	chr1	2.47E+08	2.47E+08	906	2.47E+08	25	5.1059	2.46996
Nc-1-IP_pe	chr1	2.47E+08	2.47E+08	1564	2.47E+08	23	6.62483	3.0751
Nc-1-IP_pe	chr1	2.47E+08	2.47E+08	701	2.47E+08	18	6.08025	3.26355
Nc-1-IP_pe	chr1	2.47E+08	2.47E+08	795	2.47E+08	15	6.09241	3.58328
Nc-1-IP_pe	chr1	2.47E+08	2.47E+08	256	2.47E+08	15	6.24734	3.66122
Nc-1-IP_pe	chr1	2.47E+08	2.47E+08	390	2.47E+08	12	4.39405	3.03475
Nc-1-IP_pe	chr1	2.47E+08	2.47E+08	713	2.47E+08	14	5.69896	3.50164
Nc-1-IP_pe	chr1	2.47E+08	2.47E+08	257	2.47E+08	13	6.58008	4.11487
Nc-1-IP_pe	chr1	2.47E+08	2.47E+08	303	2.47E+08	11	4.54404	3.2242
Nc-1-IP_pe	chr1	2.47E+08	2.47E+08	583	2.47E+08	11	3.64839	2.72584
Nc-1-IP_pe	chr1	2.47E+08	2.47E+08	290	2.47E+08	16	7.37408	4.10088
Nc-1-IP_pe	chr1	2.47E+08	2.47E+08	292	2.47E+08	21	9.29881	4.32834
Nc-1-IP_pe	chr1	2.47E+08	2.47E+08	266	2.47E+08	14	6.13058	3.7272
Nc-1-IP_pe	chr1	2.47E+08	2.47E+08	262	2.47E+08	16	7.97877	4.4137
Nc-1-IP_pe	chr1	2.47E+08	2.47E+08	265	2.47E+08	13	6.47103	4.0531
Nc-1-IP_pe	chr1	2.47E+08	2.47E+08	299	2.47E+08	12	5.59553	3.69882
Nc-1-IP_pe	chr1	2.47E+08	2.47E+08	265	2.47E+08	12	5.23082	3.49289

Nc-1-IP_pe	chr1	2.47E+08	2.47E+08	412	2.47E+08	11	3.28475	2.53029
Nc-1-IP_pe	chr1	2.47E+08	2.47E+08	1379	2.47E+08	17	9.62872	5.12145
Nc-1-IP_pe	chr1	2.47E+08	2.47E+08	326	2.47E+08	14	5.838	3.57373
Nc-1-IP_pe	chr1	2.47E+08	2.47E+08	318	2.47E+08	13	5.36853	3.44651
Nc-1-IP_pe	chr1	2.47E+08	2.47E+08	667	2.47E+08	20	10.6431	5.10839
Nc-1-IP_pe	chr1	2.47E+08	2.47E+08	352	2.47E+08	14	4.21449	2.76599
Nc-1-IP_pe	chr1	2.47E+08	2.47E+08	560	2.47E+08	12	5.23082	3.49289
Nc-1-IP_pe	chr1	2.47E+08	2.47E+08	285	2.47E+08	14	7.77549	4.63233
Nc-1-IP_pe	chr1	2.47E+08	2.47E+08	290	2.47E+08	15	9.09658	5.20507
Nc-1-IP_pe	chr1	2.48E+08	2.48E+08	776	2.48E+08	15	4.7819	2.95039
Nc-1-IP_pe	chr1	2.48E+08	2.48E+08	368	2.48E+08	13	4.90859	3.20357
Nc-1-IP_pe	chr1	2.48E+08	2.48E+08	348	2.48E+08	17	5.54967	3.12324
Nc-1-IP_pe	chr1	2.48E+08	2.48E+08	328	2.48E+08	12	5.01934	3.37518
Nc-1-IP_pe	chr1	2.48E+08	2.48E+08	308	2.48E+08	12	3.96559	2.80816
Nc-1-IP_pe	chr1	2.48E+08	2.48E+08	325	2.48E+08	10	3.94999	2.99009
Nc-1-IP_pe	chr1	2.48E+08	2.48E+08	309	2.48E+08	19	8.4638	4.21715
Nc-1-IP_pe	chr1	2.48E+08	2.48E+08	263	2.48E+08	13	7.42513	4.60316
Nc-1-IP_pe	chr1	2.48E+08	2.48E+08	409	2.48E+08	14	8.63073	5.12814
Nc-1-IP_pe	chr1	2.48E+08	2.48E+08	605	2.48E+08	13	6.98489	4.34671
Nc-1-IP_pe	chr1	2.48E+08	2.48E+08	1082	2.48E+08	17	6.47874	3.54137
Nc-1-IP_pe	chr1	2.48E+08	2.48E+08	262	2.48E+08	38	6.53856	2.39111
Nc-1-IP_pe	chr1	2.48E+08	2.48E+08	398	2.48E+08	13	5.36853	3.44651
Nc-1-IP_pe	chr1	2.48E+08	2.48E+08	429	2.48E+08	15	5.66913	3.37372
Nc-1-IP_pe	chr1	2.48E+08	2.48E+08	254	2.48E+08	12	5.23082	3.49289
Nc-1-IP_pe	chr1	2.48E+08	2.48E+08	263	2.48E+08	10	4.99888	3.61677
Nc-1-IP_pe	chr1	2.48E+08	2.48E+08	817	2.48E+08	21	4.90204	2.59278
Nc-1-IP_pe	chr1	2.48E+08	2.48E+08	414	2.48E+08	13	5.9487	3.76157
Nc-1-IP_pe	chr1	2.48E+08	2.48E+08	261	2.48E+08	13	7.7352	4.78626
Nc-1-IP_pe	chr1	2.48E+08	2.48E+08	263	2.48E+08	18	9.1091	4.6774
Nc-1-IP_pe	chr1	2.48E+08	2.48E+08	274	2.48E+08	11	4.06403	2.95415
Nc-1-IP_pe	chr1	2.48E+08	2.48E+08	507	2.48E+08	21	6.2632	3.08808
Nc-1-IP_pe	chr1	2.48E+08	2.48E+08	771	2.48E+08	16	6.33285	3.58457
Nc-1-IP_pe	chr1	2.48E+08	2.48E+08	285	2.48E+08	16	6.80394	3.81462
Nc-1-IP_pe	chr1	2.48E+08	2.48E+08	705	2.48E+08	12	5.77527	3.80164
Nc-1-IP_pe	chr1	2.48E+08	2.48E+08	1371	2.48E+08	19	9.92774	4.92358
Nc-1-IP_pe	chr1	2.48E+08	2.48E+08	269	2.48E+08	15	4.7819	2.95039
Nc-1-IP_pe	chr1	2.48E+08	2.48E+08	782	2.48E+08	13	4.41954	2.952
Nc-1-IP_pe	chr1	2.48E+08	2.48E+08	709	2.48E+08	19	9.81043	4.86513
Nc-1-IP_pe	chr1	2.48E+08	2.48E+08	730	2.48E+08	17	6.47874	3.54137
Nc-1-IP_pe	chr1	2.48E+08	2.48E+08	746	2.48E+08	19	6.78661	3.47027
Nc-1-IP_pe	chr1	2.48E+08	2.48E+08	325	2.48E+08	15	7.46994	4.29894
Nc-1-IP_pe	chr1	2.48E+08	2.48E+08	276	2.48E+08	13	4.41954	2.952
Nc-1-IP_pe	chr1	2.48E+08	2.48E+08	284	2.48E+08	13	7.42513	4.60316
Nc-1-IP_pe	chr1	2.48E+08	2.48E+08	737	2.48E+08	371	64.3765	2.78965
Nc-1-IP_pe	chr1	2.48E+08	2.48E+08	455	2.48E+08	24	8.53898	3.68518

Nc-1-IP_pe	chr1	2.48E+08	2.48E+08	347	2.48E+08	16	4.59224	2.7853
Nc-1-IP_pe	chr1	2.48E+08	2.48E+08	411	2.48E+08	19	4.8268	2.67939
Nc-1-IP_pe	chr1	2.48E+08	2.48E+08	332	2.48E+08	12	4.75569	3.23024
Nc-1-IP_pe	chr1	2.48E+08	2.48E+08	260	2.48E+08	14	5.30793	3.30182
Nc-1-IP_pe	chr1	2.48E+08	2.48E+08	260	2.48E+08	17	8.31233	4.43123
Nc-1-IP_pe	chr1	2.48E+08	2.48E+08	461	2.48E+08	14	3.26761	2.32785
Nc-1-IP_pe	chr1	2.48E+08	2.48E+08	988	2.48E+08	16	4.59224	2.7853
Nc-1-IP_pe	chr1	2.49E+08	2.49E+08	379	2.49E+08	30	15.7544	5.71639
Nc-1-IP_pe	chr1	2.49E+08	2.49E+08	535	2.49E+08	14	5.03085	3.16286
Nc-1-IP_pe	chr1	2.49E+08	2.49E+08	656	2.49E+08	17	7.6273	4.08876
Nc-1-IP_pe	chr1	2.49E+08	2.49E+08	504	2.49E+08	19	8.4638	4.21715
Nc-1-IP_pe	chr1	2.49E+08	2.49E+08	315	2.49E+08	16	6.33285	3.58457
Nc-1-IP_pe	chr1	2.49E+08	2.49E+08	568	2.49E+08	17	7.02067	3.79543
Nc-1-IP_pe	chr1	2.49E+08	2.49E+08	484	2.49E+08	14	5.03085	3.16286
Nc-1-IP_pe	chr1	2.49E+08	2.49E+08	325	2.49E+08	9	4.46937	3.41876
Nc-1-IP_pe	chr1	2.49E+08	2.49E+08	646	2.49E+08	11	5.03835	3.5092
Nc-1-IP_pe	chr1	2.49E+08	2.49E+08	295	2.49E+08	16	10.5028	5.81189
Nc-1-IP_pe	chr1	2.49E+08	2.49E+08	622	2.49E+08	12	4.63088	3.16234
Nc-1-IP_pe	chr1	2.49E+08	2.49E+08	422	2.49E+08	11	4.13673	2.99462
Nc-1-IP_pe	chr1	2.49E+08	2.49E+08	285	2.49E+08	13	5.03208	3.26819
Nc-1-IP_pe	chr1	2.49E+08	2.49E+08	676	2.49E+08	16	5.82808	3.34463
Nc-1-IP_pe	chr1	2.49E+08	2.49E+08	1938	2.49E+08	17	8.31233	4.43123
Nc-1-IP_pe	chr1	2.49E+08	2.49E+08	354	2.49E+08	21	9.29881	4.32834
Nc-1-IP_pe	chr1	2.49E+08	2.49E+08	320	2.49E+08	11	3.64839	2.72584
Nc-1-IP_pe	chr1	2.49E+08	2.49E+08	651	2.49E+08	12	4.24064	2.95299
Nc-1-IP_pe	chr1	2.49E+08	2.49E+08	291	2.49E+08	14	5.03085	3.16286
Nc-1-IP_pe	chr1	2.49E+08	2.49E+08	279	2.49E+08	12	4.63088	3.16234
Nc-1-IP_pe	chr1	2.49E+08	2.49E+08	608	2.49E+08	18	6.63038	3.50359
Nc-1-IP_pe	chr1	2.49E+08	2.49E+08	262	2.49E+08	16	7.44163	4.13536
Nc-1-IP_pe	chr1	2.49E+08	2.49E+08	607	2.49E+08	12	4.63088	3.16234
Nc-1-IP_pe	chr1	2.49E+08	2.49E+08	482	2.49E+08	18	9.1091	4.6774
Nc-1-IP_pe	chr1	2.49E+08	2.49E+08	613	2.49E+08	14	4.47284	2.88959
Nc-1-IP_pe	chr1	2.49E+08	2.49E+08	260	2.49E+08	19	7.18275	3.64066
Nc-1-IP_pe	chr1	2.49E+08	2.49E+08	609	2.49E+08	23	8.13503	3.62709
Nc-1-IP_pe	chr1	2.49E+08	2.49E+08	391	2.49E+08	14	4.8954	3.09572
Nc-1-IP_pe	chr1	2.49E+08	2.49E+08	458	2.49E+08	20	9.25381	4.44499
Nc-1-IP_pe	chr1	2.49E+08	2.49E+08	674	2.49E+08	19	4.5193	2.56258
Nc-1-IP_pe	chr1	2.49E+08	2.49E+08	365	2.49E+08	21	4.61597	2.49283
Nc-1-IP_pe	chr1	2.49E+08	2.49E+08	266	2.49E+08	18	8.37884	4.31591
Nc-1-IP_pe	chr1	2.49E+08	2.49E+08	369	2.49E+08	19	4.8268	2.67939
Nc-1-IP_pe	chr1	2.49E+08	2.49E+08	421	2.49E+08	20	6.07662	3.09292
Nc-1-IP_pe	chr1	2.49E+08	2.49E+08	485	2.49E+08	14	4.60011	2.95114
Nc-1-IP_pe	chr1	2.49E+08	2.49E+08	562	2.49E+08	19	6.22205	3.23373
Nc-1-IP_pe	chr1	2.49E+08	2.49E+08	1136	2.49E+08	16	5.37454	3.13479
Nc-1-IP_pe	chr1	2.49E+08	2.49E+08	338	2.49E+08	19	7.20916	3.65215

Nc-1-IP_pe	chr1	2.49E+08	2.49E+08	329	2.49E+08	17	5.6711	3.17666
Nc-1-IP_pe	chr1	2.49E+08	2.49E+08	286	2.49E+08	22	8.61083	3.91575
Nc-1-IP_pe	chr1	2.49E+08	2.49E+08	390	2.49E+08	20	6.49719	3.259
Nc-1-IP_pe	chr1	2.49E+08	2.49E+08	494	2.49E+08	23	3.79116	2.14153
Nc-1-IP_pe	chr1	2.49E+08	2.49E+08	293	2.49E+08	20	5.16039	2.74372
Nc-1-IP_pe	chr1	2.49E+08	2.49E+08	689	2.49E+08	23	7.01947	3.21526
Nc-1-IP_pe	chr1	2.49E+08	2.49E+08	683	2.49E+08	12	6.12485	4.004
Nc-1-IP_pe	chr1	2.49E+08	2.49E+08	255	2.49E+08	15	7.46994	4.29894
Nc-1-IP_pe	chr1	2.49E+08	2.49E+08	758	2.49E+08	15	6.19428	3.63445
Nc-1-IP_pe	chr1	2.49E+08	2.49E+08	527	2.49E+08	24	6.44146	2.94634
Nc-1-IP_pe	chr1	2.49E+08	2.49E+08	326	2.49E+08	20	6.49719	3.259
Nc-1-IP_pe	chr1	2.49E+08	2.49E+08	390	2.49E+08	12	2.87625	2.25568
Nc-1-IP_pe	chr1	2.49E+08	2.49E+08	351	2.49E+08	15	7.30527	4.21075
Nc-1-IP_pe	chr1	2.49E+08	2.49E+08	643	2.49E+08	33	11.8946	4.00703
Nc-1-IP_pe	chr1	2.49E+08	2.49E+08	559	2.49E+08	20	9.21699	4.428
Nc-1-IP_pe	chr10	10030	10371	342	10246	68	11.5491	2.53697
Nc-1-IP_pe	chr10	11215	11989	775	11324	12	5.82577	3.83068
Nc-1-IP_pe	chr10	27717	28551	835	28463	15	7.46994	4.29894
Nc-1-IP_pe	chr10	57258	57619	362	57408	8	3.74505	3.07688
Nc-1-IP_pe	chr10	73350	74152	803	74031	15	5.66913	3.37372
Nc-1-IP_pe	chr10	86294	86584	291	86543	9	3.66792	2.92434
Nc-1-IP_pe	chr10	87274	87564	291	87554	9	3.27326	2.68684
Nc-1-IP_pe	chr10	88054	88950	897	88566	13	4.41954	2.952
Nc-1-IP_pe	chr10	104506	105487	982	105163	10	4.62142	3.388
Nc-1-IP_pe	chr10	108659	109191	533	108833	10	5.23357	3.76063
Nc-1-IP_pe	chr10	271400	272083	684	271434	10	4.07292	3.06202
Nc-1-IP_pe	chr10	278044	278326	283	278105	11	3.64839	2.72584
Nc-1-IP_pe	chr10	280464	280756	293	280563	11	4.54404	3.2242
Nc-1-IP_pe	chr10	281455	281725	271	281623	11	3.28475	2.53029
Nc-1-IP_pe	chr10	285175	285760	586	285214	9	4.21839	3.26234
Nc-1-IP_pe	chr10	288449	288906	458	288513	10	5.05378	3.65032
Nc-1-IP_pe	chr10	289688	290134	447	289969	10	4.93609	3.57849
Nc-1-IP_pe	chr10	290447	290764	318	290645	12	6.77747	4.38954
Nc-1-IP_pe	chr10	307825	308141	317	307923	11	4.06403	2.95415
Nc-1-IP_pe	chr10	308653	308959	307	308757	14	8.22927	4.89351
Nc-1-IP_pe	chr10	309463	309804	342	309592	11	5.74187	3.92588
Nc-1-IP_pe	chr10	311862	312300	439	312232	11	4.06403	2.95415
Nc-1-IP_pe	chr10	322796	323430	635	323269	53	15.9241	3.69629
Nc-1-IP_pe	chr10	338650	338987	338	338835	11	5.52654	3.79706
Nc-1-IP_pe	chr10	341456	342093	638	341704	10	4.20113	3.13749
Nc-1-IP_pe	chr10	347189	347534	346	347493	11	4.87504	3.4143
Nc-1-IP_pe	chr10	356460	356738	279	356633	14	5.03085	3.16286
Nc-1-IP_pe	chr10	363155	363580	426	363301	14	5.51541	3.4073
Nc-1-IP_pe	chr10	368526	370010	1485	368632	13	5.36853	3.44651
Nc-1-IP_pe	chr10	371351	371706	356	371660	12	4.70082	3.20033

Nc-1-IP_pe chr10	376039	376417	379	376239	10	4.77495	3.48064
Nc-1-IP_pe chr10	378872	379124	253	378911	10	4.33501	3.21677
Nc-1-IP_pe chr10	379924	380517	594	380420	9	3.27326	2.68684
Nc-1-IP_pe chr10	384716	385080	365	384996	11	2.96397	2.36091
Nc-1-IP_pe chr10	386107	386488	382	386149	12	4.70082	3.20033
Nc-1-IP_pe chr10	388710	389048	339	388801	14	6.44441	3.89444
Nc-1-IP_pe chr10	406936	407196	261	407054	12	6.50787	4.22912
Nc-1-IP_pe chr10	413767	414025	259	413845	12	5.23082	3.49289
Nc-1-IP_pe chr10	419015	419548	534	419072	10	4.82246	3.50943
Nc-1-IP_pe chr10	420170	420493	324	420376	12	4.70082	3.20033
Nc-1-IP_pe chr10	436277	437564	1288	437333	14	7.54286	4.50023
Nc-1-IP_pe chr10	438574	439479	906	439324	14	8.06563	4.79881
Nc-1-IP_pe chr10	447145	447714	570	447205	13	4.86362	3.18015
Nc-1-IP_pe chr10	452644	452896	253	452681	11	5.10577	3.5486
Nc-1-IP_pe chr10	458138	458773	636	458772	11	2.96397	2.36091
Nc-1-IP_pe chr10	459773	460030	258	459796	11	3.68532	2.74592
Nc-1-IP_pe chr10	489487	489840	354	489635	21	9.29881	4.32834
Nc-1-IP_pe chr10	497451	498043	593	497648	12	5.23082	3.49289
Nc-1-IP_pe chr10	506662	507223	562	506730	13	5.42865	3.47872
Nc-1-IP_pe chr10	509418	509990	573	509633	15	7.19874	4.15407
Nc-1-IP_pe chr10	523185	523705	521	523344	11	5.17791	3.59087
Nc-1-IP_pe chr10	527046	527342	297	527208	11	4.74697	3.34038
Nc-1-IP_pe chr10	534046	534320	275	534113	11	5.77463	3.94557
Nc-1-IP_pe chr10	537432	537855	424	537698	9	4.46937	3.41876
Nc-1-IP_pe chr10	541617	542232	616	542112	12	5.94619	3.9002
Nc-1-IP_pe chr10	543957	544232	276	544121	12	5.23082	3.49289
Nc-1-IP_pe chr10	556975	557421	447	557276	13	6.58008	4.11487
Nc-1-IP_pe chr10	566381	566814	434	566652	10	3.08907	2.49869
Nc-1-IP_pe chr10	571143	571458	316	571261	20	11.575	5.57735
Nc-1-IP_pe chr10	586875	587306	432	587187	16	4.25252	2.63824
Nc-1-IP_pe chr10	587690	588205	516	587822	14	7.75387	4.62
Nc-1-IP_pe chr10	588573	589131	559	588671	10	4.39571	3.25288
Nc-1-IP_pe chr10	593636	593918	283	593842	9	4.15971	3.22596
Nc-1-IP_pe chr10	596942	597390	449	597305	15	4.40338	2.77622
Nc-1-IP_pe chr10	597958	598342	385	598282	11	4.06403	2.95415
Nc-1-IP_pe chr10	599588	599945	358	599744	13	6.69197	4.17855
Nc-1-IP_pe chr10	600315	600644	330	600540	9	3.76709	2.98468
Nc-1-IP_pe chr10	610693	611002	310	610833	12	4.92484	3.32299
Nc-1-IP_pe chr10	628671	628969	299	628849	12	3.47963	2.55766
Nc-1-IP_pe chr10	629492	629816	325	629575	10	3.12201	2.51709
Nc-1-IP_pe chr10	635522	635821	300	635664	18	10.5351	5.41929
Nc-1-IP_pe chr10	639176	639466	291	639460	10	3.30648	2.62073
Nc-1-IP_pe chr10	641515	641861	347	641629	14	6.69566	4.03025
Nc-1-IP_pe chr10	650883	651277	395	651000	13	5.94838	3.76139
Nc-1-IP_pe chr10	661248	661599	352	661443	15	7.95672	4.56362

Nc-1-IP_pe	chr10	677858	678199	342	677994	11	4.06403	2.95415
Nc-1-IP_pe	chr10	680107	680393	287	680285	11	4.88957	3.42271
Nc-1-IP_pe	chr10	688759	689668	910	689539	15	9.02077	5.16154
Nc-1-IP_pe	chr10	690399	690764	366	690680	13	5.36853	3.44651
Nc-1-IP_pe	chr10	695132	695684	553	695537	17	8.02964	4.28847
Nc-1-IP_pe	chr10	697269	697754	486	697655	13	6.92473	4.312
Nc-1-IP_pe	chr10	726214	726597	384	726249	9	3.72192	2.95716
Nc-1-IP_pe	chr10	726871	727489	619	727027	14	5.03085	3.16286
Nc-1-IP_pe	chr10	735355	735658	304	735413	13	5.9487	3.76157
Nc-1-IP_pe	chr10	745172	745433	262	745243	12	6.86404	4.44138
Nc-1-IP_pe	chr10	757165	757650	486	757530	14	8.63073	5.12814
Nc-1-IP_pe	chr10	759154	759816	663	759245	13	5.36853	3.44651
Nc-1-IP_pe	chr10	772326	772625	300	772465	16	9.41879	5.19453
Nc-1-IP_pe	chr10	776948	777434	487	777080	12	3.83688	2.74114
Nc-1-IP_pe	chr10	778196	778966	771	778280	13	6.06081	3.82353
Nc-1-IP_pe	chr10	779627	779965	339	779726	11	3.58722	2.69267
Nc-1-IP_pe	chr10	783781	785659	1879	784732	15	4.7819	2.95039
Nc-1-IP_pe	chr10	789698	790032	335	789892	12	5.18731	3.46856
Nc-1-IP_pe	chr10	805030	806159	1130	805429	13	4.86362	3.18015
Nc-1-IP_pe	chr10	807430	808037	608	807916	12	4.70082	3.20033
Nc-1-IP_pe	chr10	809771	810041	271	809892	12	5.84943	3.84431
Nc-1-IP_pe	chr10	815908	816175	268	816007	10	4.75926	3.47115
Nc-1-IP_pe	chr10	817893	818201	309	818017	11	5.33971	3.68619
Nc-1-IP_pe	chr10	822053	822971	919	822518	12	5.77527	3.80164
Nc-1-IP_pe	chr10	847919	848285	367	848231	12	6.08847	3.9828
Nc-1-IP_pe	chr10	862491	862789	299	862588	9	4.20361	3.25317
Nc-1-IP_pe	chr10	871359	871683	325	871470	11	5.77463	3.94557
Nc-1-IP_pe	chr10	875588	875851	264	875700	12	6.56855	4.26509
Nc-1-IP_pe	chr10	887914	888229	316	888087	12	6.58417	4.27436
Nc-1-IP_pe	chr10	905697	905949	253	905760	10	5.23357	3.76063
Nc-1-IP_pe	chr10	932750	933054	305	932997	12	5.79203	3.81127
Nc-1-IP_pe	chr10	933909	934391	483	933997	11	5.1152	3.55411
Nc-1-IP_pe	chr10	941444	941717	274	941532	11	5.74187	3.92588
Nc-1-IP_pe	chr10	943200	943511	312	943301	10	5.22943	3.75809
Nc-1-IP_pe	chr10	954990	955633	644	955127	13	7.54321	4.67266
Nc-1-IP_pe	chr10	956714	957064	351	956872	17	9.99923	5.32289
Nc-1-IP_pe	chr10	959692	960085	394	959925	10	4.46067	3.29163
Nc-1-IP_pe	chr10	964883	965151	269	965034	11	4.91882	3.43967
Nc-1-IP_pe	chr10	988258	988920	663	988292	15	2.95965	2.14351
Nc-1-IP_pe	chr10	998516	998876	361	998832	8	3.74505	3.07688
Nc-1-IP_pe	chr10	1003772	1005100	1329	1004644	13	6.02837	3.80557
Nc-1-IP_pe	chr10	1006537	1007287	751	1007204	11	4.74697	3.34038
Nc-1-IP_pe	chr10	1010558	1011021	464	1010866	11	6.03439	4.10251
Nc-1-IP_pe	chr10	1011763	1012042	280	1011878	13	7.42513	4.60316
Nc-1-IP_pe	chr10	1014418	1014895	478	1014462	11	6.03439	4.10251

Nc-1-IP_pe	chr10	1015307	1015962	656	1015533	22	11.5934	5.22453
Nc-1-IP_pe	chr10	1020387	1021300	914	1020628	13	7.42513	4.60316
Nc-1-IP_pe	chr10	1021864	1022181	318	1021957	11	5.10577	3.5486
Nc-1-IP_pe	chr10	1022528	1022852	325	1022620	10	5.23357	3.76063
Nc-1-IP_pe	chr10	1035625	1035890	266	1035723	10	4.47495	3.30017
Nc-1-IP_pe	chr10	1036503	1036777	275	1036558	14	4.21449	2.76599
Nc-1-IP_pe	chr10	1046970	1047705	736	1047458	17	10.3998	5.544
Nc-1-IP_pe	chr10	1056385	1057384	1000	1056533	21	9.68438	4.50026
Nc-1-IP_pe	chr10	1059075	1059344	270	1059250	12	6.46791	4.20548
Nc-1-IP_pe	chr10	1074536	1074986	451	1074769	12	5.84943	3.84431
Nc-1-IP_pe	chr10	1086950	1087298	349	1087178	13	7.49774	4.64586
Nc-1-IP_pe	chr10	1096344	1096894	551	1096802	13	6.54342	4.09407
Nc-1-IP_pe	chr10	1100732	1101290	559	1100921	14	7.95199	4.73339
Nc-1-IP_pe	chr10	1127670	1127995	326	1127852	12	6.46791	4.20548
Nc-1-IP_pe	chr10	1128751	1129038	288	1128933	12	6.44808	4.19375
Nc-1-IP_pe	chr10	1130505	1130862	358	1130823	8	3.74505	3.07688
Nc-1-IP_pe	chr10	1147618	1148057	440	1147911	9	4.20361	3.25317
Nc-1-IP_pe	chr10	1148966	1149348	383	1149175	9	3.72192	2.95716
Nc-1-IP_pe	chr10	1159752	1160035	284	1159839	18	6.15736	3.29676
Nc-1-IP_pe	chr10	1160617	1160971	355	1160690	10	5.23357	3.76063
Nc-1-IP_pe	chr10	1169867	1170157	291	1170033	9	3.27326	2.68684
Nc-1-IP_pe	chr10	1172028	1172470	443	1172327	9	4.07387	3.17289
Nc-1-IP_pe	chr10	1174334	1174908	575	1174436	12	3.47963	2.55766
Nc-1-IP_pe	chr10	1185679	1185949	271	1185928	11	4.06403	2.95415
Nc-1-IP_pe	chr10	1188994	1189832	839	1189179	13	5.9487	3.76157
Nc-1-IP_pe	chr10	1204455	1204939	485	1204569	11	4.54404	3.2242
Nc-1-IP_pe	chr10	1206441	1206808	368	1206689	12	5.61153	3.70794
Nc-1-IP_pe	chr10	1209473	1209725	253	1209651	12	4.92484	3.32299
Nc-1-IP_pe	chr10	1215342	1215678	337	1215485	10	4.16209	3.11446
Nc-1-IP_pe	chr10	1217392	1217674	283	1217669	10	3.08907	2.49869
Nc-1-IP_pe	chr10	1227100	1227464	365	1227132	10	3.4604	2.70797
Nc-1-IP_pe	chr10	1236237	1236923	687	1236620	8	3.74505	3.07688
Nc-1-IP_pe	chr10	1237370	1237683	314	1237625	11	5.77463	3.94557
Nc-1-IP_pe	chr10	1241444	1241814	371	1241550	12	5.77527	3.80164
Nc-1-IP_pe	chr10	1243643	1244025	383	1243933	12	5.84943	3.84431
Nc-1-IP_pe	chr10	1244574	1244851	278	1244643	12	5.23082	3.49289
Nc-1-IP_pe	chr10	1255720	1256398	679	1255774	11	4.11366	2.98176
Nc-1-IP_pe	chr10	1265449	1266132	684	1265985	17	7.85637	4.20196
Nc-1-IP_pe	chr10	1267511	1267849	339	1267748	9	4.46937	3.41876
Nc-1-IP_pe	chr10	1272665	1273047	383	1272947	10	4.99888	3.61677
Nc-1-IP_pe	chr10	1274576	1275372	797	1274950	11	6.02981	4.09973
Nc-1-IP_pe	chr10	1278027	1278279	253	1278132	10	5.23357	3.76063
Nc-1-IP_pe	chr10	1297238	1297896	659	1297872	9	4.20361	3.25317
Nc-1-IP_pe	chr10	1302016	1302754	739	1302661	14	6.06519	3.69269
Nc-1-IP_pe	chr10	1313984	1315029	1046	1314060	10	5.10552	3.682

Nc-1-IP_pe	chr10	1340665	1341885	1221	1341541	20	9.21699	4.428
Nc-1-IP_pe	chr10	1348440	1349460	1021	1349390	14	5.69896	3.50164
Nc-1-IP_pe	chr10	1354432	1355080	649	1354630	10	4.77495	3.48064
Nc-1-IP_pe	chr10	1363775	1364155	381	1363855	13	4.34711	2.91533
Nc-1-IP_pe	chr10	1373329	1374110	782	1373859	15	6.78905	3.93887
Nc-1-IP_pe	chr10	1374977	1375280	304	1375109	14	8.20556	4.87976
Nc-1-IP_pe	chr10	1377887	1378306	420	1378157	11	4.54404	3.2242
Nc-1-IP_pe	chr10	1378620	1379004	385	1378721	14	6.69566	4.03025
Nc-1-IP_pe	chr10	1379711	1380368	658	1379832	9	4.3705	3.35698
Nc-1-IP_pe	chr10	1391164	1391427	264	1391336	10	5.23357	3.76063
Nc-1-IP_pe	chr10	1394696	1394980	285	1394787	11	5.19376	3.60018
Nc-1-IP_pe	chr10	1404581	1404956	376	1404584	9	3.72192	2.95716
Nc-1-IP_pe	chr10	1413049	1413624	576	1413309	10	5.10552	3.682
Nc-1-IP_pe	chr10	1413902	1414403	502	1414047	14	8.29543	4.93196
Nc-1-IP_pe	chr10	1420690	1421082	393	1420857	15	8.84797	5.06275
Nc-1-IP_pe	chr10	1439102	1439728	627	1439212	11	5.10577	3.5486
Nc-1-IP_pe	chr10	1466115	1466496	382	1466179	11	4.06403	2.95415
Nc-1-IP_pe	chr10	1473967	1474500	534	1474305	9	4.35492	3.34727
Nc-1-IP_pe	chr10	1478908	1479425	518	1479333	11	6.03439	4.10251
Nc-1-IP_pe	chr10	1492354	1492961	608	1492910	10	4.62142	3.388
Nc-1-IP_pe	chr10	1513459	1513731	273	1513548	10	4.39571	3.25288
Nc-1-IP_pe	chr10	1525677	1526112	436	1526083	10	4.65155	3.40613
Nc-1-IP_pe	chr10	1551656	1553067	1412	1551871	17	10.984	5.87222
Nc-1-IP_pe	chr10	1557903	1558523	621	1558392	12	6.86906	4.44438
Nc-1-IP_pe	chr10	1559239	1559503	265	1559341	11	6.03439	4.10251
Nc-1-IP_pe	chr10	1589148	1589631	484	1589366	13	5.36853	3.44651
Nc-1-IP_pe	chr10	1592674	1593322	649	1592930	14	8.63073	5.12814
Nc-1-IP_pe	chr10	1595436	1595732	297	1595529	12	6.86906	4.44438
Nc-1-IP_pe	chr10	1612071	1612351	281	1612268	9	4.25966	3.28797
Nc-1-IP_pe	chr10	1616792	1617707	916	1616947	18	6.65364	3.51391
Nc-1-IP_pe	chr10	1632751	1633120	370	1632793	8	3.56021	2.95918
Nc-1-IP_pe	chr10	1633879	1634179	301	1634037	12	5.84943	3.84431
Nc-1-IP_pe	chr10	1669574	1670061	488	1670012	10	4.93609	3.57849
Nc-1-IP_pe	chr10	1671222	1671576	355	1671399	10	4.33501	3.21677
Nc-1-IP_pe	chr10	1672363	1672736	374	1672621	12	6.58417	4.27436
Nc-1-IP_pe	chr10	1677353	1677663	311	1677457	12	6.86906	4.44438
Nc-1-IP_pe	chr10	1678255	1678517	263	1678366	10	5.23357	3.76063
Nc-1-IP_pe	chr10	1707502	1708139	638	1707598	11	5.10577	3.5486
Nc-1-IP_pe	chr10	1739887	1740255	369	1740165	11	4.61014	3.26192
Nc-1-IP_pe	chr10	1746878	1747469	592	1747101	12	4.70082	3.20033
Nc-1-IP_pe	chr10	1754750	1755177	428	1755105	10	3.89064	2.95552
Nc-1-IP_pe	chr10	1771816	1772180	365	1772119	7	3.06442	2.73501
Nc-1-IP_pe	chr10	1790550	1791354	805	1791257	11	6.03439	4.10251
Nc-1-IP_pe	chr10	1800487	1800851	365	1800554	12	4.88508	3.30112
Nc-1-IP_pe	chr10	1807121	1807921	801	1807546	9	4.25966	3.28797

Nc-1-IP_pe	chr10	1874616	1874901	286	1874754	9	4.46937	3.41876
Nc-1-IP_pe	chr10	1923921	1924336	416	1923998	11	3.64839	2.72584
Nc-1-IP_pe	chr10	1935755	1936222	468	1935813	8	3.74505	3.07688
Nc-1-IP_pe	chr10	1961474	1961947	474	1961912	9	4.46937	3.41876
Nc-1-IP_pe	chr10	1992430	1992706	277	1992566	10	5.23357	3.76063
Nc-1-IP_pe	chr10	2019995	2020542	548	2020504	10	4.39571	3.25288
Nc-1-IP_pe	chr10	2023940	2024335	396	2024150	10	4.39571	3.25288
Nc-1-IP_pe	chr10	2076029	2076321	293	2076133	12	6.86906	4.44438
Nc-1-IP_pe	chr10	2096653	2096960	308	2096812	10	5.23357	3.76063
Nc-1-IP_pe	chr10	2158302	2158597	296	2158440	11	5.10577	3.5486
Nc-1-IP_pe	chr10	2175839	2176190	352	2176039	20	9.94634	4.77022
Nc-1-IP_pe	chr10	2231682	2232013	332	2231877	20	8.56319	4.1316
Nc-1-IP_pe	chr10	2256363	2256639	277	2256473	8	3.08755	2.66145
Nc-1-IP_pe	chr10	2266425	2266878	454	2266610	11	5.10577	3.5486
Nc-1-IP_pe	chr10	2315097	2315383	287	2315279	12	6.86906	4.44438
Nc-1-IP_pe	chr10	2328971	2329229	259	2329017	9	4.20361	3.25317
Nc-1-IP_pe	chr10	2381712	2382054	343	2381878	11	6.03439	4.10251
Nc-1-IP_pe	chr10	2501381	2501692	312	2501636	18	4.04135	2.43445
Nc-1-IP_pe	chr10	2521071	2521542	472	2521251	22	12.505	5.66212
Nc-1-IP_pe	chr10	2580705	2581012	308	2580902	10	5.23357	3.76063
Nc-1-IP_pe	chr10	2595798	2596082	285	2595935	11	6.03439	4.10251
Nc-1-IP_pe	chr10	2622766	2623314	549	2623145	9	4.46937	3.41876
Nc-1-IP_pe	chr10	2671594	2671968	375	2671777	10	5.23357	3.76063
Nc-1-IP_pe	chr10	2712823	2713158	336	2713035	9	4.20361	3.25317
Nc-1-IP_pe	chr10	2730928	2731211	284	2731118	9	4.46937	3.41876
Nc-1-IP_pe	chr10	2733535	2733853	319	2733583	9	3.72192	2.95716
Nc-1-IP_pe	chr10	2736121	2736415	295	2736324	11	5.77463	3.94557
Nc-1-IP_pe	chr10	2748129	2748417	289	2748288	9	4.25966	3.28797
Nc-1-IP_pe	chr10	2800296	2800549	254	2800388	8	3.08755	2.66145
Nc-1-IP_pe	chr10	2813391	2813725	335	2813599	10	4.39571	3.25288
Nc-1-IP_pe	chr10	2887457	2887950	494	2887582	15	8.16556	4.67894
Nc-1-IP_pe	chr10	2903232	2903774	543	2903624	10	3.89064	2.95552
Nc-1-IP_pe	chr10	2905978	2906296	319	2906238	10	3.89064	2.95552
Nc-1-IP_pe	chr10	2907151	2907747	597	2907276	10	4.93609	3.57849
Nc-1-IP_pe	chr10	2910269	2910572	304	2910395	12	5.84943	3.84431
Nc-1-IP_pe	chr10	2912780	2913130	351	2912949	13	5.9487	3.76157
Nc-1-IP_pe	chr10	2913401	2913679	279	2913496	14	8.29543	4.93196
Nc-1-IP_pe	chr10	2915252	2915876	625	2915606	15	8.84797	5.06275
Nc-1-IP_pe	chr10	2944460	2944991	532	2944644	10	4.39571	3.25288
Nc-1-IP_pe	chr10	2962422	2962851	430	2962685	12	6.86906	4.44438
Nc-1-IP_pe	chr10	2977631	2977896	266	2977762	12	6.86906	4.44438
Nc-1-IP_pe	chr10	2987866	2988536	671	2987940	11	5.52654	3.79706
Nc-1-IP_pe	chr10	2996145	2996407	263	2996313	10	5.23357	3.76063
Nc-1-IP_pe	chr10	3020168	3020556	389	3020299	13	7.7352	4.78626
Nc-1-IP_pe	chr10	3043904	3044252	349	3044143	10	5.12294	3.69268

Nc-1-IP_pe	chr10	3046098	3046499	402	3046432	11	3.64839	2.72584
Nc-1-IP_pe	chr10	3066721	3067910	1190	3067058	15	6.19428	3.63445
Nc-1-IP_pe	chr10	3069095	3069378	284	3069209	16	6.37501	3.60492
Nc-1-IP_pe	chr10	3073019	3073596	578	3073117	13	5.36853	3.44651
Nc-1-IP_pe	chr10	3079773	3080125	353	3080013	9	4.3087	3.31847
Nc-1-IP_pe	chr10	3081317	3081632	316	3081600	9	3.72192	2.95716
Nc-1-IP_pe	chr10	3082478	3082739	262	3082611	13	6.92473	4.312
Nc-1-IP_pe	chr10	3099266	3099753	488	3099359	10	4.39571	3.25288
Nc-1-IP_pe	chr10	3100964	3102075	1112	3101474	18	7.73136	4.00629
Nc-1-IP_pe	chr10	3102819	3103093	275	3102953	12	5.99895	3.93077
Nc-1-IP_pe	chr10	3108786	3109144	359	3108992	12	5.17301	3.46058
Nc-1-IP_pe	chr10	3114547	3115405	859	3115240	18	6.17129	3.30277
Nc-1-IP_pe	chr10	3119591	3119877	287	3119726	14	6.76465	4.06784
Nc-1-IP_pe	chr10	3122199	3122980	782	3122670	14	5.838	3.57373
Nc-1-IP_pe	chr10	3123768	3124100	333	3123925	16	7.61454	4.22416
Nc-1-IP_pe	chr10	3124801	3125151	351	3124902	15	7.45104	4.28878
Nc-1-IP_pe	chr10	3125515	3125786	272	3125683	13	5.15976	3.33548
Nc-1-IP_pe	chr10	3128613	3128896	284	3128717	11	4.06403	2.95415
Nc-1-IP_pe	chr10	3132100	3132403	304	3132236	16	9.32142	5.14024
Nc-1-IP_pe	chr10	3136121	3136376	256	3136182	9	3.22129	2.65588
Nc-1-IP_pe	chr10	3142511	3142805	295	3142554	10	4.25398	3.16873
Nc-1-IP_pe	chr10	3150773	3151138	366	3151021	9	3.27326	2.68684
Nc-1-IP_pe	chr10	3151784	3152596	813	3152311	9	4.25966	3.28797
Nc-1-IP_pe	chr10	3154945	3155479	535	3155072	11	6.03439	4.10251
Nc-1-IP_pe	chr10	3172326	3172720	395	3172636	15	4.40338	2.77622
Nc-1-IP_pe	chr10	3188448	3189155	708	3188984	10	4.99888	3.61677
Nc-1-IP_pe	chr10	3196711	3197611	901	3196993	19	9.92774	4.92358
Nc-1-IP_pe	chr10	3205777	3206301	525	3206177	10	3.4604	2.70797
Nc-1-IP_pe	chr10	3208554	3208945	392	3208692	12	6.77747	4.38954
Nc-1-IP_pe	chr10	3241902	3242461	560	3241998	12	6.46791	4.20548
Nc-1-IP_pe	chr10	3245354	3245608	255	3245416	8	3.56021	2.95918
Nc-1-IP_pe	chr10	3254013	3254462	450	3254323	10	4.62142	3.388
Nc-1-IP_pe	chr10	3263771	3264444	674	3264276	13	7.34205	4.55444
Nc-1-IP_pe	chr10	3274069	3274405	337	3274325	10	3.71867	2.85592
Nc-1-IP_pe	chr10	3275881	3276505	625	3276348	9	4.25966	3.28797
Nc-1-IP_pe	chr10	3284409	3284669	261	3284552	8	3.74505	3.07688
Nc-1-IP_pe	chr10	3286041	3286384	344	3286174	12	6.58417	4.27436
Nc-1-IP_pe	chr10	3288133	3288393	261	3288185	13	5.86991	3.71823
Nc-1-IP_pe	chr10	3289710	3290037	328	3289843	11	5.30676	3.66673
Nc-1-IP_pe	chr10	3314602	3315031	430	3314947	24	5.47323	2.62998
Nc-1-IP_pe	chr10	3320138	3320634	497	3320382	14	5.03085	3.16286
Nc-1-IP_pe	chr10	3331274	3331534	261	3331519	8	3.56021	2.95918
Nc-1-IP_pe	chr10	3339343	3339950	608	3339742	8	3.74505	3.07688
Nc-1-IP_pe	chr10	3349760	3350291	532	3350136	12	6.86906	4.44438
Nc-1-IP_pe	chr10	3394789	3395059	271	3394918	9	4.1889	3.24405

Nc-1-IP_pe	chr10	3397772	3398231	460	3397995	14	8.59917	5.10958
Nc-1-IP_pe	chr10	3404231	3404685	455	3404401	10	5.23357	3.76063
Nc-1-IP_pe	chr10	3407157	3407496	340	3407372	14	7.42849	4.43574
Nc-1-IP_pe	chr10	3408161	3409122	962	3408268	15	8.20837	4.70271
Nc-1-IP_pe	chr10	3412556	3413447	892	3413024	16	8.45195	4.66488
Nc-1-IP_pe	chr10	3416151	3416532	382	3416443	11	5.1152	3.55411
Nc-1-IP_pe	chr10	3420959	3421235	277	3421219	9	4.05981	3.16422
Nc-1-IP_pe	chr10	3421773	3422042	270	3421948	11	3.28475	2.53029
Nc-1-IP_pe	chr10	3423424	3423693	270	3423452	10	3.89064	2.95552
Nc-1-IP_pe	chr10	3456822	3457141	320	3456912	9	2.89249	2.46179
Nc-1-IP_pe	chr10	3458623	3459337	715	3458723	17	5.54967	3.12324
Nc-1-IP_pe	chr10	3494881	3495242	362	3494976	11	5.03835	3.5092
Nc-1-IP_pe	chr10	3537842	3538249	408	3538094	10	4.66672	3.41527
Nc-1-IP_pe	chr10	3539313	3539923	611	3539471	11	3.06138	2.41205
Nc-1-IP_pe	chr10	3592998	3593275	278	3593007	10	3.08907	2.49869
Nc-1-IP_pe	chr10	3653593	3654389	797	3653946	11	4.54404	3.2242
Nc-1-IP_pe	chr10	3666251	3666789	539	3666420	14	6.06519	3.69269
Nc-1-IP_pe	chr10	3671064	3671322	259	3671179	11	4.54404	3.2242
Nc-1-IP_pe	chr10	3682040	3682332	293	3682097	11	5.66869	3.88198
Nc-1-IP_pe	chr10	3689768	3690023	256	3689831	13	4.86362	3.18015
Nc-1-IP_pe	chr10	3693235	3693659	425	3693584	10	3.08907	2.49869
Nc-1-IP_pe	chr10	3706599	3706857	259	3706689	9	4.46937	3.41876
Nc-1-IP_pe	chr10	3730119	3730415	297	3730162	14	5.03085	3.16286
Nc-1-IP_pe	chr10	3744678	3744951	274	3744752	13	4.86362	3.18015
Nc-1-IP_pe	chr10	3747000	3747533	534	3747353	12	6.71369	4.35145
Nc-1-IP_pe	chr10	3758449	3758872	424	3758674	17	6.92131	3.74829
Nc-1-IP_pe	chr10	3759833	3760328	496	3760197	10	3.89064	2.95552
Nc-1-IP_pe	chr10	3780171	3780498	328	3780247	12	4.70082	3.20033
Nc-1-IP_pe	chr10	3783405	3783844	440	3783524	11	4.06403	2.95415
Nc-1-IP_pe	chr10	3790720	3791005	286	3790777	9	4.35492	3.34727
Nc-1-IP_pe	chr10	3807360	3808245	886	3808051	14	8.11183	4.82549
Nc-1-IP_pe	chr10	3826531	3826789	259	3826592	13	6.19316	3.89711
Nc-1-IP_pe	chr10	3855753	3856402	650	3856200	8	3.7135	3.05675
Nc-1-IP_pe	chr10	3888878	3889288	411	3889045	16	9.11744	5.02718
Nc-1-IP_pe	chr10	3895859	3896112	254	3895989	13	7.7352	4.78626
Nc-1-IP_pe	chr10	3961733	3961994	262	3961824	12	6.86906	4.44438
Nc-1-IP_pe	chr10	3979531	3979862	332	3979680	11	5.52654	3.79706
Nc-1-IP_pe	chr10	3992404	3992910	507	3992829	13	6.62436	4.14003
Nc-1-IP_pe	chr10	4088225	4088718	494	4088492	10	5.23357	3.76063
Nc-1-IP_pe	chr10	4141738	4142016	279	4141901	11	5.10577	3.5486
Nc-1-IP_pe	chr10	4145357	4145684	328	4145593	12	4.24064	2.95299
Nc-1-IP_pe	chr10	4149443	4149710	268	4149577	16	8.26996	4.56762
Nc-1-IP_pe	chr10	4194942	4195374	433	4195039	9	3.92293	3.08
Nc-1-IP_pe	chr10	4199759	4200094	336	4199881	11	5.10577	3.5486
Nc-1-IP_pe	chr10	4205653	4206145	493	4205707	8	3.08755	2.66145

Nc-1-IP_pe	chr10	4233465	4233729	265	4233624	10	5.23357	3.76063
Nc-1-IP_pe	chr10	4258020	4258274	255	4258131	13	7.42513	4.60316
Nc-1-IP_pe	chr10	4265306	4265574	269	4265496	9	4.46937	3.41876
Nc-1-IP_pe	chr10	4409645	4409898	254	4409775	12	6.86906	4.44438
Nc-1-IP_pe	chr10	4459136	4459442	307	4459369	9	4.46937	3.41876
Nc-1-IP_pe	chr10	4587502	4587858	357	4587683	15	9.19325	5.26076
Nc-1-IP_pe	chr10	4604495	4604855	361	4604613	10	5.23357	3.76063
Nc-1-IP_pe	chr10	4607778	4608098	321	4607965	10	5.23357	3.76063
Nc-1-IP_pe	chr10	4801477	4801869	393	4801629	10	5.23357	3.76063
Nc-1-IP_pe	chr10	4807404	4807781	378	4807717	9	4.46937	3.41876
Nc-1-IP_pe	chr10	4814147	4814418	272	4814329	13	7.7352	4.78626
Nc-1-IP_pe	chr10	4825157	4825753	597	4825657	9	4.05981	3.16422
Nc-1-IP_pe	chr10	4826674	4826980	307	4826770	12	6.86906	4.44438
Nc-1-IP_pe	chr10	4837770	4838052	283	4837892	14	7.56355	4.51193
Nc-1-IP_pe	chr10	4844890	4845185	296	4845131	18	6.63038	3.50359
Nc-1-IP_pe	chr10	4845635	4846313	679	4845798	14	6.49348	3.92083
Nc-1-IP_pe	chr10	4911189	4911467	279	4911272	12	6.12485	4.004
Nc-1-IP_pe	chr10	4936668	4937542	875	4937410	13	5.86991	3.71823
Nc-1-IP_pe	chr10	4950946	4951199	254	4951015	8	3.74505	3.07688
Nc-1-IP_pe	chr10	4956435	4956761	327	4956556	13	3.67377	2.58159
Nc-1-IP_pe	chr10	4972379	4972648	270	4972527	20	5.34663	2.81336
Nc-1-IP_pe	chr10	4984169	4984868	700	4984446	16	6.33285	3.58457
Nc-1-IP_pe	chr10	4989436	4989953	518	4989526	13	6.62436	4.14003
Nc-1-IP_pe	chr10	5009833	5010170	338	5010013	13	3.35769	2.42919
Nc-1-IP_pe	chr10	5032420	5032703	284	5032509	15	5.66913	3.37372
Nc-1-IP_pe	chr10	5046645	5047247	603	5046835	15	7.46956	4.29873
Nc-1-IP_pe	chr10	5053043	5053688	646	5053236	23	9.83409	4.30114
Nc-1-IP_pe	chr10	5060606	5061015	410	5060860	19	6.32622	3.27682
Nc-1-IP_pe	chr10	5102377	5102800	424	5102644	18	9.94172	5.10499
Nc-1-IP_pe	chr10	5110016	5110336	321	5110213	12	5.84943	3.84431
Nc-1-IP_pe	chr10	5147797	5148091	295	5147952	12	6.86906	4.44438
Nc-1-IP_pe	chr10	5199349	5199729	381	5199527	12	5.84943	3.84431
Nc-1-IP_pe	chr10	5245416	5246034	619	5245591	14	6.13058	3.7272
Nc-1-IP_pe	chr10	5296775	5297043	269	5296940	12	5.07448	3.40574
Nc-1-IP_pe	chr10	5311095	5311497	403	5311400	9	4.21839	3.26234
Nc-1-IP_pe	chr10	5325118	5325761	644	5325274	12	6.86906	4.44438
Nc-1-IP_pe	chr10	5327518	5327949	432	5327635	13	6.92473	4.312
Nc-1-IP_pe	chr10	5329769	5330215	447	5330115	12	4.24064	2.95299
Nc-1-IP_pe	chr10	5334137	5334394	258	5334238	10	4.99888	3.61677
Nc-1-IP_pe	chr10	5364941	5365356	416	5365175	11	3.28475	2.53029
Nc-1-IP_pe	chr10	5380303	5380984	682	5380434	13	7.7352	4.78626
Nc-1-IP_pe	chr10	5466186	5466567	382	5466496	11	4.06403	2.95415
Nc-1-IP_pe	chr10	5473341	5473595	255	5473440	13	6.29523	3.95418
Nc-1-IP_pe	chr10	5477820	5478177	358	5478076	13	5.36853	3.44651
Nc-1-IP_pe	chr10	5487594	5488502	909	5488237	11	4.06403	2.95415

Nc-1-IP_pe chr10	5492517	5493762	1246	5492697	15	6.78905	3.93887
Nc-1-IP_pe chr10	5498321	5498652	332	5498476	20	6.08336	3.09555
Nc-1-IP_pe chr10	5500643	5500984	342	5500942	13	4.02562	2.7544
Nc-1-IP_pe chr10	5506654	5507016	363	5506939	12	4.24064	2.95299
Nc-1-IP_pe chr10	5508043	5508383	341	5508296	10	3.70758	2.84953
Nc-1-IP_pe chr10	5514896	5515149	254	5515036	13	6.29523	3.95418
Nc-1-IP_pe chr10	5521550	5522060	511	5521646	11	4.06403	2.95415
Nc-1-IP_pe chr10	5523016	5523672	657	5523076	12	4.70082	3.20033
Nc-1-IP_pe chr10	5525271	5526158	888	5525423	16	6.8987	3.86161
Nc-1-IP_pe chr10	5527526	5527992	467	5527901	12	5.3483	3.55882
Nc-1-IP_pe chr10	5533209	5533744	536	5533537	17	5.14803	2.94914
Nc-1-IP_pe chr10	5546323	5546667	345	5546396	14	4.60011	2.95114
Nc-1-IP_pe chr10	5550390	5550760	371	5550507	17	7.43048	3.99256
Nc-1-IP_pe chr10	5553309	5553640	332	5553391	14	4.60011	2.95114
Nc-1-IP_pe chr10	5557181	5557482	302	5557385	13	5.03208	3.26819
Nc-1-IP_pe chr10	5557925	5558694	770	5558207	17	7.85637	4.20196
Nc-1-IP_pe chr10	5560925	5561334	410	5560997	14	4.21449	2.76599
Nc-1-IP_pe chr10	5564326	5564823	498	5564668	11	4.21886	3.04051
Nc-1-IP_pe chr10	5570110	5570531	422	5570184	14	5.03085	3.16286
Nc-1-IP_pe chr10	5574828	5575176	349	5574959	14	6.06519	3.69269
Nc-1-IP_pe chr10	5575561	5575999	439	5575744	9	4.11647	3.19921
Nc-1-IP_pe chr10	5576845	5577177	333	5576848	9	2.89249	2.46179
Nc-1-IP_pe chr10	5582937	5583230	294	5583078	15	7.19874	4.15407
Nc-1-IP_pe chr10	5587875	5588735	861	5588058	12	4.87193	3.29389
Nc-1-IP_pe chr10	5589359	5589639	281	5589513	18	8.80785	4.52672
Nc-1-IP_pe chr10	5598666	5598939	274	5598868	13	5.73236	3.64298
Nc-1-IP_pe chr10	5601562	5602206	645	5601618	9	3.58404	2.87351
Nc-1-IP_pe chr10	5604104	5604502	399	5604275	12	5.61153	3.70794
Nc-1-IP_pe chr10	5606773	5607153	381	5607057	10	4.39571	3.25288
Nc-1-IP_pe chr10	5607870	5608321	452	5607987	8	3.21751	2.7428
Nc-1-IP_pe chr10	5621045	5621371	327	5621099	9	2.91362	2.47417
Nc-1-IP_pe chr10	5632022	5632293	272	5632184	11	5.3563	3.696
Nc-1-IP_pe chr10	5640080	5640927	848	5640422	12	5.84943	3.84431
Nc-1-IP_pe chr10	5646095	5646529	435	5646237	16	8.26996	4.56762
Nc-1-IP_pe chr10	5659296	5659587	292	5659420	13	5.29187	3.40559
Nc-1-IP_pe chr10	5660870	5661256	387	5660929	12	3.83688	2.74114
Nc-1-IP_pe chr10	5666205	5666615	411	5666325	14	5.56442	3.43239
Nc-1-IP_pe chr10	5692862	5693226	365	5692984	13	6.72991	4.20021
Nc-1-IP_pe chr10	5749238	5749548	311	5749402	12	6.528	4.24104
Nc-1-IP_pe chr10	5812878	5813585	708	5813147	19	6.32622	3.27682
Nc-1-IP_pe chr10	5836445	5836705	261	5836506	12	5.21606	3.48463
Nc-1-IP_pe chr10	5840391	5841129	739	5841006	13	5.36853	3.44651
Nc-1-IP_pe chr10	5862734	5863144	411	5863135	9	2.89249	2.46179
Nc-1-IP_pe chr10	5882998	5883309	312	5883098	12	5.84943	3.84431
Nc-1-IP_pe chr10	5884129	5884570	442	5884316	11	4.54404	3.2242

Nc-1-IP_pe	chr10	5889068	5889733	666	5889385	13	3.07238	2.29378
Nc-1-IP_pe	chr10	5890425	5890758	334	5890539	13	4.78908	3.14145
Nc-1-IP_pe	chr10	5896093	5896562	470	5896407	8	3.61691	2.99521
Nc-1-IP_pe	chr10	5910523	5911490	968	5911241	12	5.84943	3.84431
Nc-1-IP_pe	chr10	5915136	5915421	286	5915391	9	4.25966	3.28797
Nc-1-IP_pe	chr10	5916394	5916714	321	5916612	13	7.40812	4.59318
Nc-1-IP_pe	chr10	5923825	5924223	399	5924144	11	5.05359	3.5181
Nc-1-IP_pe	chr10	5930562	5931256	695	5931194	11	5.76039	3.93701
Nc-1-IP_pe	chr10	5959889	5960683	795	5960235	12	5.45448	3.61875
Nc-1-IP_pe	chr10	5976967	5977463	497	5977194	14	5.43414	3.36584
Nc-1-IP_pe	chr10	6019548	6019827	280	6019746	16	8.97063	4.94637
Nc-1-IP_pe	chr10	6032426	6032683	258	6032546	12	6.86906	4.44438
Nc-1-IP_pe	chr10	6049931	6050232	302	6050213	8	3.4979	2.91965
Nc-1-IP_pe	chr10	6050656	6051035	380	6050873	14	7.42849	4.43574
Nc-1-IP_pe	chr10	6052637	6053013	377	6052928	10	4.62142	3.388
Nc-1-IP_pe	chr10	6074879	6075251	373	6074886	10	3.4604	2.70797
Nc-1-IP_pe	chr10	6088902	6089572	671	6089415	19	8.35089	4.16476
Nc-1-IP_pe	chr10	6111277	6111635	359	6111487	12	6.71369	4.35145
Nc-1-IP_pe	chr10	6127487	6127774	288	6127537	11	2.96397	2.36091
Nc-1-IP_pe	chr10	6128454	6128771	318	6128669	12	3.47963	2.55766
Nc-1-IP_pe	chr10	6130155	6130554	400	6130500	10	4.32135	3.20866
Nc-1-IP_pe	chr10	6132396	6132653	258	6132454	10	4.22744	3.15303
Nc-1-IP_pe	chr10	6136118	6136389	272	6136285	12	5.61153	3.70794
Nc-1-IP_pe	chr10	6140098	6140441	344	6140233	11	4.11366	2.98176
Nc-1-IP_pe	chr10	6142923	6143317	395	6143116	16	6.33285	3.58457
Nc-1-IP_pe	chr10	6144859	6145928	1070	6145633	15	7.30527	4.21075
Nc-1-IP_pe	chr10	6153177	6153533	357	6153333	10	3.4604	2.70797
Nc-1-IP_pe	chr10	6156261	6156659	399	6156295	8	3.67166	3.03007
Nc-1-IP_pe	chr10	6178583	6179076	494	6178943	15	7.56326	4.34922
Nc-1-IP_pe	chr10	6179751	6180014	264	6179965	9	3.50273	2.82441
Nc-1-IP_pe	chr10	6195772	6196157	386	6195990	12	5.61153	3.70794
Nc-1-IP_pe	chr10	6201044	6201582	539	6201547	11	4.35236	3.11555
Nc-1-IP_pe	chr10	6202997	6204262	1266	6203197	18	8.27052	4.2634
Nc-1-IP_pe	chr10	6208596	6209039	444	6208702	9	3.94978	3.09648
Nc-1-IP_pe	chr10	6211542	6212210	669	6211624	13	6.69197	4.17855
Nc-1-IP_pe	chr10	6215651	6216197	547	6215929	12	4.24064	2.95299
Nc-1-IP_pe	chr10	6217265	6217566	302	6217367	11	5.03835	3.5092
Nc-1-IP_pe	chr10	6223928	6224379	452	6224063	13	5.9487	3.76157
Nc-1-IP_pe	chr10	6225256	6225723	468	6225424	15	7.75613	4.45384
Nc-1-IP_pe	chr10	6228624	6228930	307	6228636	10	4.28075	3.18458
Nc-1-IP_pe	chr10	6231536	6232541	1006	6232347	12	5.3483	3.55882
Nc-1-IP_pe	chr10	6279680	6280028	349	6279944	9	3.66792	2.92434
Nc-1-IP_pe	chr10	6340602	6341057	456	6340742	13	6.90485	4.30056
Nc-1-IP_pe	chr10	6422772	6423296	525	6422916	10	5.00283	3.61918
Nc-1-IP_pe	chr10	6463632	6464377	746	6463798	12	6.71369	4.35145

Nc-1-IP_pe	chr10	6464978	6465418	441	6465341	9	2.89249	2.46179
Nc-1-IP_pe	chr10	6488631	6488931	301	6488921	8	3.74505	3.07688
Nc-1-IP_pe	chr10	6489886	6490180	295	6489894	8	2.93592	2.56703
Nc-1-IP_pe	chr10	6491331	6491645	315	6491460	11	5.77463	3.94557
Nc-1-IP_pe	chr10	6495037	6495401	365	6495038	10	3.08907	2.49869
Nc-1-IP_pe	chr10	6520445	6520888	444	6520761	9	3.54889	2.85226
Nc-1-IP_pe	chr10	6526480	6526996	517	6526587	14	7.9745	4.74633
Nc-1-IP_pe	chr10	6543691	6543943	253	6543728	9	3.02445	2.53932
Nc-1-IP_pe	chr10	6563237	6564019	783	6563380	10	3.89064	2.95552
Nc-1-IP_pe	chr10	6578915	6579493	579	6579018	10	5.23357	3.76063
Nc-1-IP_pe	chr10	6594413	6594692	280	6594462	9	4.46937	3.41876
Nc-1-IP_pe	chr10	6634637	6635274	638	6634685	10	4.47495	3.30017
Nc-1-IP_pe	chr10	6642761	6643027	267	6642967	13	7.42513	4.60316
Nc-1-IP_pe	chr10	6772738	6773036	299	6772882	10	4.39571	3.25288
Nc-1-IP_pe	chr10	6792743	6793327	585	6792880	12	6.58417	4.27436
Nc-1-IP_pe	chr10	6800387	6800641	255	6800531	10	5.23357	3.76063
Nc-1-IP_pe	chr10	6809793	6810242	450	6809902	15	6.78905	3.93887
Nc-1-IP_pe	chr10	6890811	6891227	417	6890875	9	4.25966	3.28797
Nc-1-IP_pe	chr10	6976113	6976531	419	6976374	9	4.20361	3.25317
Nc-1-IP_pe	chr10	7075068	7075339	272	7075127	9	4.05981	3.16422
Nc-1-IP_pe	chr10	7137478	7137810	333	7137639	21	9.29881	4.32834
Nc-1-IP_pe	chr10	7147669	7148300	632	7148096	13	7.42513	4.60316
Nc-1-IP_pe	chr10	7194067	7194319	253	7194149	11	5.10577	3.5486
Nc-1-IP_pe	chr10	7210699	7211151	453	7211040	8	3.56332	2.96115
Nc-1-IP_pe	chr10	7220852	7221146	295	7221020	10	5.23357	3.76063
Nc-1-IP_pe	chr10	7246499	7246876	378	7246612	9	4.46937	3.41876
Nc-1-IP_pe	chr10	7251831	7252322	492	7252234	11	3.64839	2.72584
Nc-1-IP_pe	chr10	7263086	7263430	345	7263210	8	3.57661	2.96959
Nc-1-IP_pe	chr10	7267182	7267517	336	7267201	9	3.54889	2.85226
Nc-1-IP_pe	chr10	7272384	7272781	398	7272584	11	5.10577	3.5486
Nc-1-IP_pe	chr10	7275202	7275498	297	7275344	9	4.35492	3.34727
Nc-1-IP_pe	chr10	7329435	7329963	529	7329819	9	3.72192	2.95716
Nc-1-IP_pe	chr10	7346855	7347353	499	7347218	10	5.23357	3.76063
Nc-1-IP_pe	chr10	7352613	7352867	255	7352736	11	4.54404	3.2242
Nc-1-IP_pe	chr10	7357985	7358839	855	7358610	10	4.07292	3.06202
Nc-1-IP_pe	chr10	7363424	7363700	277	7363536	17	11.2828	6.04256
Nc-1-IP_pe	chr10	7373879	7374314	436	7374058	14	6.61011	3.98382
Nc-1-IP_pe	chr10	7406691	7407465	775	7407317	12	4.70082	3.20033
Nc-1-IP_pe	chr10	7408597	7409174	578	7408805	10	4.93609	3.57849
Nc-1-IP_pe	chr10	7410251	7410567	317	7410423	10	4.39571	3.25288
Nc-1-IP_pe	chr10	7412399	7412696	298	7412479	15	4.7819	2.95039
Nc-1-IP_pe	chr10	7442515	7442791	277	7442577	12	5.45448	3.61875
Nc-1-IP_pe	chr10	7463937	7464284	348	7464134	11	6.03439	4.10251
Nc-1-IP_pe	chr10	7474522	7475486	965	7474646	12	6.86906	4.44438
Nc-1-IP_pe	chr10	7508193	7508589	397	7508380	10	3.89064	2.95552

Nc-1-IP_pe	chr10	7557372	7557802	431	7557643	11	4.54404	3.2242
Nc-1-IP_pe	chr10	7569400	7569718	319	7569595	9	4.46937	3.41876
Nc-1-IP_pe	chr10	7570686	7570956	271	7570845	9	4.46937	3.41876
Nc-1-IP_pe	chr10	7576187	7577109	923	7576463	14	8.63073	5.12814
Nc-1-IP_pe	chr10	7593328	7593719	392	7593613	11	5.52654	3.79706
Nc-1-IP_pe	chr10	7607234	7607512	279	7607375	10	5.23357	3.76063
Nc-1-IP_pe	chr10	7619618	7619997	380	7619718	8	3.56021	2.95918
Nc-1-IP_pe	chr10	7625972	7626245	274	7626042	10	5.23357	3.76063
Nc-1-IP_pe	chr10	7651118	7651431	314	7651285	14	8.63073	5.12814
Nc-1-IP_pe	chr10	7651722	7652021	300	7651998	9	4.46937	3.41876
Nc-1-IP_pe	chr10	7667459	7668041	583	7667987	10	5.23357	3.76063
Nc-1-IP_pe	chr10	7698622	7699101	480	7699048	9	4.25966	3.28797
Nc-1-IP_pe	chr10	7739870	7740150	281	7739976	10	4.99888	3.61677
Nc-1-IP_pe	chr10	7754301	7754735	435	7754317	8	3.74505	3.07688
Nc-1-IP_pe	chr10	7768442	7768784	343	7768617	11	5.97044	4.06374
Nc-1-IP_pe	chr10	7782389	7782653	265	7782515	10	4.99888	3.61677
Nc-1-IP_pe	chr10	7798291	7798567	277	7798454	14	5.51541	3.4073
Nc-1-IP_pe	chr10	7800531	7800817	287	7800662	11	6.03439	4.10251
Nc-1-IP_pe	chr10	7814113	7814495	383	7814227	10	5.23357	3.76063
Nc-1-IP_pe	chr10	7818397	7818724	328	7818571	12	5.94619	3.9002
Nc-1-IP_pe	chr10	7833715	7834067	353	7833787	11	4.06403	2.95415
Nc-1-IP_pe	chr10	7908815	7909167	353	7908958	11	4.06403	2.95415
Nc-1-IP_pe	chr10	7915091	7915356	266	7915220	11	6.03439	4.10251
Nc-1-IP_pe	chr10	7935827	7936101	275	7936013	8	3.74505	3.07688
Nc-1-IP_pe	chr10	7942891	7943180	290	7943001	11	6.03439	4.10251
Nc-1-IP_pe	chr10	8000347	8000696	350	8000615	8	3.74505	3.07688
Nc-1-IP_pe	chr10	8008172	8008654	483	8008332	9	4.25966	3.28797
Nc-1-IP_pe	chr10	8033825	8034086	262	8033877	9	2.89249	2.46179
Nc-1-IP_pe	chr10	8043050	8043630	581	8043234	15	8.26005	4.73146
Nc-1-IP_pe	chr10	8046390	8046805	416	8046527	13	4.86362	3.18015
Nc-1-IP_pe	chr10	8048248	8048746	499	8048387	14	4.60011	2.95114
Nc-1-IP_pe	chr10	8049971	8050709	739	8050072	12	5.23053	3.49272
Nc-1-IP_pe	chr10	8051382	8051668	287	8051479	14	6.06519	3.69269
Nc-1-IP_pe	chr10	8055595	8056322	728	8056110	15	7.56326	4.34922
Nc-1-IP_pe	chr10	8058374	8058831	458	8058612	19	10.236	5.07864
Nc-1-IP_pe	chr10	8062973	8063294	322	8063129	9	4.20361	3.25317
Nc-1-IP_pe	chr10	8069566	8069832	267	8069718	11	5.10577	3.5486
Nc-1-IP_pe	chr10	8094159	8094414	256	8094380	10	3.4604	2.70797
Nc-1-IP_pe	chr10	8097643	8098195	553	8097769	10	5.23357	3.76063
Nc-1-IP_pe	chr10	8102019	8102539	521	8102111	9	4.35492	3.34727
Nc-1-IP_pe	chr10	8103007	8103268	262	8103102	10	5.05378	3.65032
Nc-1-IP_pe	chr10	8105381	8105681	301	8105540	15	5.20142	3.14789
Nc-1-IP_pe	chr10	8154289	8154625	337	8154358	10	4.93609	3.57849
Nc-1-IP_pe	chr10	8161842	8162144	303	8161912	12	5.23082	3.49289
Nc-1-IP_pe	chr10	8172672	8173187	516	8172949	15	9.19934	5.26427

Nc-1-IP_pe	chr10	8181608	8182035	428	8181731	9	3.72192	2.95716
Nc-1-IP_pe	chr10	8188455	8188728	274	8188544	9	4.05981	3.16422
Nc-1-IP_pe	chr10	8199681	8200070	390	8199867	14	8.63073	5.12814
Nc-1-IP_pe	chr10	8242278	8242671	394	8242550	12	5.77527	3.80164
Nc-1-IP_pe	chr10	8260754	8261014	261	8260863	11	6.03439	4.10251
Nc-1-IP_pe	chr10	8261346	8261606	261	8261466	10	5.23357	3.76063
Nc-1-IP_pe	chr10	8285185	8285524	340	8285305	11	6.03439	4.10251
Nc-1-IP_pe	chr10	8364131	8364395	265	8364316	8	3.264	2.772
Nc-1-IP_pe	chr10	8403917	8404213	297	8403969	7	3.06442	2.73501
Nc-1-IP_pe	chr10	8446869	8447330	462	8447071	13	6.62436	4.14003
Nc-1-IP_pe	chr10	8458548	8458839	292	8458648	11	5.89269	4.01672
Nc-1-IP_pe	chr10	8484185	8484827	643	8484277	9	4.46937	3.41876
Nc-1-IP_pe	chr10	8513907	8514273	367	8514128	14	6.06519	3.69269
Nc-1-IP_pe	chr10	8566555	8567068	514	8566975	12	5.61153	3.70794
Nc-1-IP_pe	chr10	8846309	8846580	272	8846471	11	6.03439	4.10251
Nc-1-IP_pe	chr10	9067666	9067920	255	9067748	12	5.23082	3.49289
Nc-1-IP_pe	chr10	9116824	9117232	409	9116936	11	6.03439	4.10251
Nc-1-IP_pe	chr10	9183175	9183499	325	9183443	15	5.66913	3.37372
Nc-1-IP_pe	chr10	9243908	9244241	334	9244029	10	5.23357	3.76063
Nc-1-IP_pe	chr10	9407304	9407602	299	9407325	11	4.00079	2.91908
Nc-1-IP_pe	chr10	9595971	9596261	291	9596085	10	5.23357	3.76063
Nc-1-IP_pe	chr10	9681419	9681700	282	9681546	13	7.7352	4.78626
Nc-1-IP_pe	chr10	9713163	9713441	279	9713383	10	4.99888	3.61677
Nc-1-IP_pe	chr10	9819537	9819928	392	9819700	10	5.10552	3.682
Nc-1-IP_pe	chr10	10160743	10161123	381	10161057	14	6.69566	4.03025
Nc-1-IP_pe	chr10	10161956	10162311	356	10162030	13	4.86362	3.18015
Nc-1-IP_pe	chr10	10266281	10266774	494	10266624	12	6.86906	4.44438
Nc-1-IP_pe	chr10	10409147	10409502	356	10409221	9	3.72192	2.95716
Nc-1-IP_pe	chr10	10603964	10604229	266	10604080	10	5.23357	3.76063
Nc-1-IP_pe	chr10	10666496	10666836	341	10666734	11	4.06403	2.95415
Nc-1-IP_pe	chr10	10719047	10719396	350	10719188	9	4.46937	3.41876
Nc-1-IP_pe	chr10	10765056	10765414	359	10765287	9	3.72192	2.95716
Nc-1-IP_pe	chr10	10821814	10822221	408	10821835	9	3.27326	2.68684
Nc-1-IP_pe	chr10	10973497	10973817	321	10973686	12	6.86906	4.44438
Nc-1-IP_pe	chr10	10983608	10983917	310	10983784	11	6.03439	4.10251
Nc-1-IP_pe	chr10	11012472	11012865	394	11012550	7	3.06442	2.73501
Nc-1-IP_pe	chr10	11017447	11017801	355	11017609	16	9.74731	5.37917
Nc-1-IP_pe	chr10	11018246	11018521	276	11018409	12	3.83688	2.74114
Nc-1-IP_pe	chr10	11161946	11162212	267	11162076	10	3.89064	2.95552
Nc-1-IP_pe	chr10	11165643	11166020	378	11165934	10	4.99888	3.61677
Nc-1-IP_pe	chr10	11177970	11178433	464	11178210	9	3.27326	2.68684
Nc-1-IP_pe	chr10	11191302	11191646	345	11191425	13	6.62436	4.14003
Nc-1-IP_pe	chr10	11191909	11192176	268	11192138	8	3.74505	3.07688
Nc-1-IP_pe	chr10	11207468	11207728	261	11207652	10	5.23357	3.76063
Nc-1-IP_pe	chr10	11242671	11243122	452	11242947	12	6.71369	4.35145

Nc-1-IP_pe	chr10	11247165	11248394	1230	11247440	13	6.62436	4.14003
Nc-1-IP_pe	chr10	11270552	11270829	278	11270668	10	4.99888	3.61677
Nc-1-IP_pe	chr10	11280190	11281066	877	11280358	11	6.03439	4.10251
Nc-1-IP_pe	chr10	11281490	11281968	479	11281649	11	6.03439	4.10251
Nc-1-IP_pe	chr10	11314221	11314644	424	11314468	10	4.99888	3.61677
Nc-1-IP_pe	chr10	11314910	11315238	329	11315009	11	5.10577	3.5486
Nc-1-IP_pe	chr10	11337471	11337941	471	11337566	10	4.39571	3.25288
Nc-1-IP_pe	chr10	11363173	11363447	275	11363264	9	4.46568	3.41644
Nc-1-IP_pe	chr10	11381292	11381806	515	11381647	14	6.69566	4.03025
Nc-1-IP_pe	chr10	11382618	11383099	482	11383072	9	4.27829	3.29955
Nc-1-IP_pe	chr10	11385344	11385680	337	11385500	8	3.51085	2.92785
Nc-1-IP_pe	chr10	11389104	11389523	420	11389282	10	4.39571	3.25288
Nc-1-IP_pe	chr10	11401309	11401688	380	11401502	13	7.7352	4.78626
Nc-1-IP_pe	chr10	11423218	11423513	296	11423380	10	3.4604	2.70797
Nc-1-IP_pe	chr10	11424060	11424384	325	11424229	20	12.6503	6.14111
Nc-1-IP_pe	chr10	11432204	11432501	298	11432361	10	4.98602	3.60892
Nc-1-IP_pe	chr10	11442056	11442475	420	11442160	10	4.75926	3.47115
Nc-1-IP_pe	chr10	11457358	11457641	284	11457482	10	5.23357	3.76063
Nc-1-IP_pe	chr10	11499506	11499813	308	11499700	11	6.03439	4.10251
Nc-1-IP_pe	chr10	11611048	11611465	418	11611199	16	7.61454	4.22416
Nc-1-IP_pe	chr10	11611820	11612098	279	11611976	14	5.03085	3.16286
Nc-1-IP_pe	chr10	11616228	11616566	339	11616399	13	7.7352	4.78626
Nc-1-IP_pe	chr10	11622263	11622587	325	11622574	9	3.27326	2.68684
Nc-1-IP_pe	chr10	11626211	11626576	366	11626374	11	2.96397	2.36091
Nc-1-IP_pe	chr10	11650416	11650736	321	11650527	10	4.87068	3.5387
Nc-1-IP_pe	chr10	11654826	11655215	390	11654900	9	3.68012	2.93175
Nc-1-IP_pe	chr10	11662561	11662843	283	11662619	12	5.84943	3.84431
Nc-1-IP_pe	chr10	11683510	11683909	400	11683814	12	3.83688	2.74114
Nc-1-IP_pe	chr10	11686542	11686797	256	11686678	11	4.50458	3.20175
Nc-1-IP_pe	chr10	11707075	11707348	274	11707139	12	6.10662	3.99337
Nc-1-IP_pe	chr10	11709120	11709433	314	11709362	10	3.83201	2.92147
Nc-1-IP_pe	chr10	11713204	11713466	263	11713448	8	3.08755	2.66145
Nc-1-IP_pe	chr10	11727799	11728211	413	11727908	9	4.46937	3.41876
Nc-1-IP_pe	chr10	11742528	11742828	301	11742636	14	5.03085	3.16286
Nc-1-IP_pe	chr10	11755466	11755773	308	11755688	12	6.58417	4.27436
Nc-1-IP_pe	chr10	11757634	11758123	490	11758059	9	3.43499	2.78365
Nc-1-IP_pe	chr10	11762372	11762864	493	11762597	9	4.24819	3.28084
Nc-1-IP_pe	chr10	11765236	11765838	603	11765736	12	6.58417	4.27436
Nc-1-IP_pe	chr10	11768894	11769239	346	11768977	7	3.06442	2.73501
Nc-1-IP_pe	chr10	11772797	11773201	405	11773132	12	5.23082	3.49289
Nc-1-IP_pe	chr10	11774932	11775232	301	11775012	9	3.79246	3.00015
Nc-1-IP_pe	chr10	11778114	11778392	279	11778263	12	6.54823	4.25303
Nc-1-IP_pe	chr10	11783942	11784219	278	11784031	10	3.89064	2.95552
Nc-1-IP_pe	chr10	11804591	11804963	373	11804754	12	5.01934	3.37518
Nc-1-IP_pe	chr10	11823260	11823812	553	11823659	12	6.12485	4.004

Nc-1-IP_pe	chr10	11825294	11825566	273	11825363	11	4.54404	3.2242
Nc-1-IP_pe	chr10	11826653	11827081	429	11826853	11	5.59692	3.83905
Nc-1-IP_pe	chr10	11839474	11840123	650	11839662	15	9.55381	5.47001
Nc-1-IP_pe	chr10	11856165	11856469	305	11856187	9	3.90961	3.07183
Nc-1-IP_pe	chr10	11859049	11859352	304	11859190	13	6.72991	4.20021
Nc-1-IP_pe	chr10	11859880	11860399	520	11860048	14	6.61011	3.98382
Nc-1-IP_pe	chr10	11869347	11869885	539	11869666	14	6.44441	3.89444
Nc-1-IP_pe	chr10	11870337	11870605	269	11870480	12	5.5796	3.68975
Nc-1-IP_pe	chr10	11877411	11877864	454	11877472	13	3.67377	2.58159
Nc-1-IP_pe	chr10	11879229	11880308	1080	11879376	14	5.47279	3.38553
Nc-1-IP_pe	chr10	11885233	11885586	354	11885560	23	5.5873	2.71945
Nc-1-IP_pe	chr10	11894587	11894958	372	11894765	15	7.39587	4.25918
Nc-1-IP_pe	chr10	11901852	11902120	269	11902004	15	7.58222	4.35946
Nc-1-IP_pe	chr10	11902377	11902708	332	11902657	11	4.59676	3.25427
Nc-1-IP_pe	chr10	11905993	11906262	270	11906111	11	5.10577	3.5486
Nc-1-IP_pe	chr10	11907908	11908664	757	11908193	12	2.87625	2.25568
Nc-1-IP_pe	chr10	11982508	11982783	276	11982552	8	3.56021	2.95918
Nc-1-IP_pe	chr10	11988430	11988684	255	11988643	11	3.28475	2.53029
Nc-1-IP_pe	chr10	12005608	12006111	504	12005714	9	3.92293	3.08
Nc-1-IP_pe	chr10	12018180	12018669	490	12018340	11	6.03439	4.10251
Nc-1-IP_pe	chr10	12025802	12026055	254	12025936	11	6.03439	4.10251
Nc-1-IP_pe	chr10	12048389	12048786	398	12048552	11	5.10577	3.5486
Nc-1-IP_pe	chr10	12068973	12069240	268	12069035	15	6.85973	3.97568
Nc-1-IP_pe	chr10	12076364	12076726	363	12076516	10	3.60969	2.79325
Nc-1-IP_pe	chr10	12080056	12080400	345	12080100	11	5.61473	3.84969
Nc-1-IP_pe	chr10	12112996	12113352	357	12113327	9	4.04582	3.15559
Nc-1-IP_pe	chr10	12118172	12118514	343	12118250	9	3.93633	3.08822
Nc-1-IP_pe	chr10	12122491	12122791	301	12122774	10	4.62142	3.388
Nc-1-IP_pe	chr10	12174810	12175238	429	12175162	8	3.7276	3.06575
Nc-1-IP_pe	chr10	12181728	12182098	371	12181897	9	4.25966	3.28797
Nc-1-IP_pe	chr10	12195572	12196686	1115	12196464	16	10.5028	5.81189
Nc-1-IP_pe	chr10	12202536	12203005	470	12202739	12	5.84943	3.84431
Nc-1-IP_pe	chr10	12205853	12206112	260	12205980	12	6.86906	4.44438
Nc-1-IP_pe	chr10	12223030	12223528	499	12223400	12	4.70082	3.20033
Nc-1-IP_pe	chr10	12226229	12227170	942	12226656	13	5.36853	3.44651
Nc-1-IP_pe	chr10	12228065	12228636	572	12228155	8	3.7135	3.05675
Nc-1-IP_pe	chr10	12234135	12234483	349	12234229	11	6.03439	4.10251
Nc-1-IP_pe	chr10	12239457	12239779	323	12239520	10	5.23357	3.76063
Nc-1-IP_pe	chr10	12253768	12254075	308	12253857	11	4.35236	3.11555
Nc-1-IP_pe	chr10	12288633	12288906	274	12288783	8	3.51085	2.92785
Nc-1-IP_pe	chr10	12290865	12291122	258	12290937	10	4.39571	3.25288
Nc-1-IP_pe	chr10	12298073	12298571	499	12298499	10	4.62142	3.388
Nc-1-IP_pe	chr10	12301678	12302153	476	12301823	12	5.84943	3.84431
Nc-1-IP_pe	chr10	12348928	12349721	794	12349321	16	5.37454	3.13479
Nc-1-IP_pe	chr10	12350754	12351243	490	12350832	10	4.20113	3.13749

Nc-1-IP_pe	chr10	12363139	12363478	340	12363385	11	5.19376	3.60018
Nc-1-IP_pe	chr10	12376719	12377036	318	12376813	11	5.44042	3.74585
Nc-1-IP_pe	chr10	12429258	12429657	400	12429458	14	4.73128	3.01506
Nc-1-IP_pe	chr10	12467071	12467349	279	12467242	11	5.97044	4.06374
Nc-1-IP_pe	chr10	12470519	12470840	322	12470677	11	4.06403	2.95415
Nc-1-IP_pe	chr10	12477745	12478200	456	12477781	12	4.70082	3.20033
Nc-1-IP_pe	chr10	12479655	12480087	433	12479930	9	3.92293	3.08
Nc-1-IP_pe	chr10	12489989	12490265	277	12490016	11	4.06403	2.95415
Nc-1-IP_pe	chr10	12493525	12493856	332	12493688	10	3.08907	2.49869
Nc-1-IP_pe	chr10	12502080	12502582	503	12502298	16	6.33285	3.58457
Nc-1-IP_pe	chr10	12519057	12520163	1107	12519134	26	10.439	4.19014
Nc-1-IP_pe	chr10	12522746	12523586	841	12523403	16	4.25252	2.63824
Nc-1-IP_pe	chr10	12538761	12539073	313	12538981	12	6.86906	4.44438
Nc-1-IP_pe	chr10	12542794	12543117	324	12543065	9	4.46937	3.41876
Nc-1-IP_pe	chr10	12560143	12560539	397	12560435	8	3.63049	3.00385
Nc-1-IP_pe	chr10	12589187	12589517	331	12589417	10	4.39571	3.25288
Nc-1-IP_pe	chr10	12625063	12625645	583	12625605	10	5.23357	3.76063
Nc-1-IP_pe	chr10	12672062	12672527	466	12672180	7	3.06442	2.73501
Nc-1-IP_pe	chr10	12708914	12709167	254	12708975	11	5.19376	3.60018
Nc-1-IP_pe	chr10	12713442	12713786	345	12713567	10	4.99888	3.61677
Nc-1-IP_pe	chr10	12729775	12730084	310	12729827	14	4.60011	2.95114
Nc-1-IP_pe	chr10	12741731	12741991	261	12741812	11	4.54404	3.2242
Nc-1-IP_pe	chr10	12751732	12752108	377	12751969	11	3.64839	2.72584
Nc-1-IP_pe	chr10	12753643	12754110	468	12753709	10	4.77495	3.48064
Nc-1-IP_pe	chr10	12758912	12759276	365	12759194	9	2.89249	2.46179
Nc-1-IP_pe	chr10	12761569	12761834	266	12761669	10	5.23357	3.76063
Nc-1-IP_pe	chr10	12766783	12767549	767	12767146	10	5.23357	3.76063
Nc-1-IP_pe	chr10	12770989	12771264	276	12771005	7	3.06442	2.73501
Nc-1-IP_pe	chr10	12775033	12775977	945	12775409	11	5.10577	3.5486
Nc-1-IP_pe	chr10	12777652	12777943	292	12777877	13	5.71736	3.63481
Nc-1-IP_pe	chr10	12788377	12788757	381	12788666	9	4.46568	3.41644
Nc-1-IP_pe	chr10	12814134	12814504	371	12814371	10	3.89064	2.95552
Nc-1-IP_pe	chr10	12838741	12839045	305	12838918	11	6.03439	4.10251
Nc-1-IP_pe	chr10	12839758	12840155	398	12839867	12	6.71369	4.35145
Nc-1-IP_pe	chr10	12841524	12842485	962	12841669	10	3.89064	2.95552
Nc-1-IP_pe	chr10	12847604	12847960	357	12847742	13	6.62436	4.14003
Nc-1-IP_pe	chr10	12848979	12849329	351	12849161	11	6.03439	4.10251
Nc-1-IP_pe	chr10	12872725	12873099	375	12872882	15	9.09658	5.20507
Nc-1-IP_pe	chr10	12885034	12885298	265	12885096	10	5.23357	3.76063
Nc-1-IP_pe	chr10	12893931	12894206	276	12894170	10	5.23357	3.76063
Nc-1-IP_pe	chr10	12906292	12906800	509	12906480	13	6.36468	3.99316
Nc-1-IP_pe	chr10	12908986	12909998	1013	12909602	13	5.36853	3.44651
Nc-1-IP_pe	chr10	12915805	12916079	275	12915899	14	5.98179	3.64885
Nc-1-IP_pe	chr10	12959131	12959462	332	12959341	8	3.51085	2.92785
Nc-1-IP_pe	chr10	12967921	12968185	265	12968103	12	6.86906	4.44438

Nc-1-IP_pe	chr10	13013891	13014187	297	13013957	10	5.23357	3.76063
Nc-1-IP_pe	chr10	13018562	13019131	570	13018703	9	4.46937	3.41876
Nc-1-IP_pe	chr10	13054529	13054969	441	13054576	8	3.56021	2.95918
Nc-1-IP_pe	chr10	13082245	13082785	541	13082685	10	3.4604	2.70797
Nc-1-IP_pe	chr10	13098780	13099307	528	13099219	11	5.44042	3.74585
Nc-1-IP_pe	chr10	13100247	13100747	501	13100528	13	5.71736	3.63481
Nc-1-IP_pe	chr10	13108497	13108783	287	13108641	12	6.86906	4.44438
Nc-1-IP_pe	chr10	13118560	13118834	275	13118639	15	9.55381	5.47001
Nc-1-IP_pe	chr10	13142912	13143276	365	13143041	13	7.43036	4.60624
Nc-1-IP_pe	chr10	13162099	13162648	550	13162187	9	4.46937	3.41876
Nc-1-IP_pe	chr10	13207529	13207783	255	13207714	9	4.46937	3.41876
Nc-1-IP_pe	chr10	13212259	13212565	307	13212478	11	6.03439	4.10251
Nc-1-IP_pe	chr10	13221964	13222236	273	13222171	11	4.00079	2.91908
Nc-1-IP_pe	chr10	13225531	13225956	426	13225834	12	5.98129	3.92053
Nc-1-IP_pe	chr10	13227808	13228094	287	13227991	10	4.80655	3.49978
Nc-1-IP_pe	chr10	13234212	13234550	339	13234326	12	5.89413	3.8701
Nc-1-IP_pe	chr10	13273761	13274732	972	13273894	13	7.08702	4.40581
Nc-1-IP_pe	chr10	13276200	13276589	390	13276420	13	7.7352	4.78626
Nc-1-IP_pe	chr10	13278528	13278788	261	13278714	11	5.10577	3.5486
Nc-1-IP_pe	chr10	13285825	13286313	489	13285863	11	4.06403	2.95415
Nc-1-IP_pe	chr10	13288349	13288710	362	13288511	13	7.56612	4.68618
Nc-1-IP_pe	chr10	13290473	13290760	288	13290734	9	2.89249	2.46179
Nc-1-IP_pe	chr10	13300430	13301290	861	13300590	14	5.51541	3.4073
Nc-1-IP_pe	chr10	13322554	13322844	291	13322679	12	4.51041	3.09723
Nc-1-IP_pe	chr10	13455549	13456356	808	13455924	12	4.63088	3.16234
Nc-1-IP_pe	chr10	13500747	13501224	478	13501078	10	5.23357	3.76063
Nc-1-IP_pe	chr10	13517465	13517850	386	13517516	10	5.23357	3.76063
Nc-1-IP_pe	chr10	13557428	13557763	336	13557707	8	3.74505	3.07688
Nc-1-IP_pe	chr10	13586952	13587600	649	13587492	14	5.03085	3.16286
Nc-1-IP_pe	chr10	13654509	13654946	438	13654717	14	8.34955	4.96348
Nc-1-IP_pe	chr10	13656642	13657034	393	13656671	10	3.4604	2.70797
Nc-1-IP_pe	chr10	13658487	13658771	285	13658612	13	6.62436	4.14003
Nc-1-IP_pe	chr10	13665652	13666162	511	13665978	10	5.14046	3.70342
Nc-1-IP_pe	chr10	13666687	13667461	775	13667265	9	4.25966	3.28797
Nc-1-IP_pe	chr10	13691612	13691875	264	13691779	11	5.27411	3.64746
Nc-1-IP_pe	chr10	13695668	13696163	496	13695840	11	4.06403	2.95415
Nc-1-IP_pe	chr10	13699449	13699781	333	13699483	8	3.57661	2.96959
Nc-1-IP_pe	chr10	13718181	13718563	383	13718461	9	2.93173	2.4848
Nc-1-IP_pe	chr10	13721232	13721679	448	13721333	10	4.99888	3.61677
Nc-1-IP_pe	chr10	13733902	13734159	258	13733933	7	3.01283	2.70125
Nc-1-IP_pe	chr10	13737101	13737406	306	13737209	11	5.10577	3.5486
Nc-1-IP_pe	chr10	13747378	13748256	879	13748076	11	6.03439	4.10251
Nc-1-IP_pe	chr10	13769451	13770119	669	13770093	11	5.3563	3.696
Nc-1-IP_pe	chr10	13790376	13790776	401	13790574	14	5.51541	3.4073
Nc-1-IP_pe	chr10	13791807	13792407	601	13792164	13	7.34205	4.55444

Nc-1-IP_pe	chr10	13812885	13813192	308	13813008	9	3.72192	2.95716
Nc-1-IP_pe	chr10	13829941	13830583	643	13830475	15	7.46994	4.29894
Nc-1-IP_pe	chr10	13839663	13839942	280	13839739	9	3.92293	3.08
Nc-1-IP_pe	chr10	13856360	13856641	282	13856548	13	7.42513	4.60316
Nc-1-IP_pe	chr10	13915531	13916030	500	13915935	9	4.46937	3.41876
Nc-1-IP_pe	chr10	13967763	13968089	327	13967859	11	6.03439	4.10251
Nc-1-IP_pe	chr10	13981412	13981976	565	13981890	9	4.46937	3.41876
Nc-1-IP_pe	chr10	13983797	13984229	433	13984043	12	6.86906	4.44438
Nc-1-IP_pe	chr10	14034150	14034434	285	14034269	12	6.86906	4.44438
Nc-1-IP_pe	chr10	14052053	14052676	624	14052152	9	4.46937	3.41876
Nc-1-IP_pe	chr10	14092128	14092526	399	14092428	9	4.35492	3.34727
Nc-1-IP_pe	chr10	14094404	14094683	280	14094519	9	2.89249	2.46179
Nc-1-IP_pe	chr10	14120812	14121071	260	14120823	11	3.28475	2.53029
Nc-1-IP_pe	chr10	14132026	14132555	530	14132175	9	4.05981	3.16422
Nc-1-IP_pe	chr10	14209619	14209902	284	14209764	9	4.46937	3.41876
Nc-1-IP_pe	chr10	14220046	14220313	268	14220175	13	6.54342	4.09407
Nc-1-IP_pe	chr10	14264823	14265092	270	14264990	11	4.06403	2.95415
Nc-1-IP_pe	chr10	14276644	14276944	301	14276714	8	3.74505	3.07688
Nc-1-IP_pe	chr10	14326835	14327116	282	14326906	11	5.03835	3.5092
Nc-1-IP_pe	chr10	14340452	14340956	505	14340604	13	6.62436	4.14003
Nc-1-IP_pe	chr10	14353884	14354145	262	14354013	12	6.86906	4.44438
Nc-1-IP_pe	chr10	14362831	14363261	431	14363224	10	4.99888	3.61677
Nc-1-IP_pe	chr10	14448042	14448432	391	14448223	11	6.03439	4.10251
Nc-1-IP_pe	chr10	14497562	14497901	340	14497820	11	5.83542	3.98217
Nc-1-IP_pe	chr10	14534265	14534655	391	14534569	11	5.3563	3.696
Nc-1-IP_pe	chr10	14540875	14541494	620	14541292	9	3.02445	2.53932
Nc-1-IP_pe	chr10	14548396	14548678	283	14548480	12	2.87625	2.25568
Nc-1-IP_pe	chr10	14561273	14561549	277	14561430	12	5.84943	3.84431
Nc-1-IP_pe	chr10	14582514	14583071	558	14582887	12	6.07041	3.97229
Nc-1-IP_pe	chr10	14588891	14589157	267	14589079	10	5.03671	3.63988
Nc-1-IP_pe	chr10	14631583	14632077	495	14631717	14	6.61011	3.98382
Nc-1-IP_pe	chr10	14648245	14648505	261	14648331	10	5.23357	3.76063
Nc-1-IP_pe	chr10	14685893	14686171	279	14685982	9	3.92293	3.08
Nc-1-IP_pe	chr10	14687990	14688266	277	14688067	11	5.83542	3.98217
Nc-1-IP_pe	chr10	14700088	14700345	258	14700146	10	5.23357	3.76063
Nc-1-IP_pe	chr10	14751682	14752260	579	14751823	12	4.24064	2.95299
Nc-1-IP_pe	chr10	14754090	14754374	285	14754205	10	5.07094	3.66082
Nc-1-IP_pe	chr10	14762270	14762692	423	14762277	9	4.05981	3.16422
Nc-1-IP_pe	chr10	14767082	14767366	285	14767218	11	6.03439	4.10251
Nc-1-IP_pe	chr10	14784560	14785017	458	14784923	7	3.06442	2.73501
Nc-1-IP_pe	chr10	14809457	14810150	694	14809504	8	3.14944	2.70014
Nc-1-IP_pe	chr10	14818627	14818919	293	14818726	12	6.86906	4.44438
Nc-1-IP_pe	chr10	14838317	14839195	879	14838970	16	7.44163	4.13536
Nc-1-IP_pe	chr10	14843379	14843743	365	14843451	12	4.24064	2.95299
Nc-1-IP_pe	chr10	14878791	14879132	342	14878907	16	5.82808	3.34463

Nc-1-IP_pe	chr10	14888216	14888486	271	14888316	10	3.08907	2.49869
Nc-1-IP_pe	chr10	14905699	14905997	299	14905858	13	5.9487	3.76157
Nc-1-IP_pe	chr10	14920697	14921018	322	14920786	8	3.51085	2.92785
Nc-1-IP_pe	chr10	14931932	14932243	312	14932121	10	5.23357	3.76063
Nc-1-IP_pe	chr10	14933492	14934061	570	14933596	10	5.23357	3.76063
Nc-1-IP_pe	chr10	14942818	14943089	272	14943043	9	4.00426	3.12999
Nc-1-IP_pe	chr10	14945680	14946195	516	14945816	11	5.03835	3.5092
Nc-1-IP_pe	chr10	14950644	14950906	263	14950782	14	6.69566	4.03025
Nc-1-IP_pe	chr10	14957430	14957774	345	14957663	11	4.69157	3.30855
Nc-1-IP_pe	chr10	15003807	15004243	437	15003842	10	4.99888	3.61677
Nc-1-IP_pe	chr10	15024281	15024705	425	15024700	9	3.27326	2.68684
Nc-1-IP_pe	chr10	15041645	15041926	282	15041757	10	3.89064	2.95552
Nc-1-IP_pe	chr10	15069223	15069620	398	15069554	9	3.72192	2.95716
Nc-1-IP_pe	chr10	15083556	15084118	563	15083940	10	5.10552	3.682
Nc-1-IP_pe	chr10	15093241	15093622	382	15093493	11	5.10577	3.5486
Nc-1-IP_pe	chr10	15097078	15097555	478	15097490	14	4.13959	2.7305
Nc-1-IP_pe	chr10	15106670	15106952	283	15106920	10	5.23357	3.76063
Nc-1-IP_pe	chr10	15109999	15110262	264	15110119	11	6.03439	4.10251
Nc-1-IP_pe	chr10	15126983	15127425	443	15127056	10	4.99888	3.61677
Nc-1-IP_pe	chr10	15134341	15134598	258	15134452	9	4.25966	3.28797
Nc-1-IP_pe	chr10	15153897	15154368	472	15154090	10	3.89064	2.95552
Nc-1-IP_pe	chr10	15167901	15168643	743	15168174	15	7.46994	4.29894
Nc-1-IP_pe	chr10	15187672	15187927	256	15187776	10	3.4604	2.70797
Nc-1-IP_pe	chr10	15199201	15199680	480	15199320	10	4.93609	3.57849
Nc-1-IP_pe	chr10	15206447	15206726	280	15206627	13	6.54342	4.09407
Nc-1-IP_pe	chr10	15218961	15219227	267	15219157	13	6.54342	4.09407
Nc-1-IP_pe	chr10	15222727	15223789	1063	15222861	11	6.03439	4.10251
Nc-1-IP_pe	chr10	15224132	15224435	304	15224387	9	3.32589	2.71826
Nc-1-IP_pe	chr10	15225865	15226189	325	15225972	11	4.06403	2.95415
Nc-1-IP_pe	chr10	15231797	15232051	255	15231893	7	2.97697	2.67781
Nc-1-IP_pe	chr10	15257659	15258228	570	15257926	12	5.84943	3.84431
Nc-1-IP_pe	chr10	15294177	15294443	267	15294364	9	4.46937	3.41876
Nc-1-IP_pe	chr10	15326275	15326637	363	15326599	11	3.64839	2.72584
Nc-1-IP_pe	chr10	15337595	15337944	350	15337703	11	5.77463	3.94557
Nc-1-IP_pe	chr10	15348049	15348647	599	15348152	9	4.46937	3.41876
Nc-1-IP_pe	chr10	15350698	15350983	286	15350761	10	5.23357	3.76063
Nc-1-IP_pe	chr10	15351268	15351707	440	15351331	9	3.72192	2.95716
Nc-1-IP_pe	chr10	15353939	15354616	678	15354450	9	4.46937	3.41876
Nc-1-IP_pe	chr10	15392254	15392544	291	15392388	11	5.3563	3.696
Nc-1-IP_pe	chr10	15398255	15398557	303	15398306	10	4.77495	3.48064
Nc-1-IP_pe	chr10	15425179	15425619	441	15425310	12	4.70082	3.20033
Nc-1-IP_pe	chr10	15445680	15445945	266	15445767	8	3.74505	3.07688
Nc-1-IP_pe	chr10	15456552	15456834	283	15456754	12	6.86906	4.44438
Nc-1-IP_pe	chr10	15626026	15626404	379	15626336	9	3.72192	2.95716
Nc-1-IP_pe	chr10	15719072	15720017	946	15719743	14	8.63073	5.12814

Nc-1-IP_pe	chr10	15743675	15744337	663	15743828	8	3.74505	3.07688
Nc-1-IP_pe	chr10	15860360	15860636	277	15860429	13	5.29187	3.40559
Nc-1-IP_pe	chr10	15920991	15921260	270	15921190	14	3.86727	2.6027
Nc-1-IP_pe	chr10	16177138	16177570	433	16177469	12	6.86906	4.44438
Nc-1-IP_pe	chr10	16438059	16438337	279	16438144	11	4.88957	3.42271
Nc-1-IP_pe	chr10	16457377	16457631	255	16457451	9	3.79246	3.00015
Nc-1-IP_pe	chr10	16519949	16520252	304	16520139	11	5.3563	3.696
Nc-1-IP_pe	chr10	16535360	16535637	278	16535543	11	6.03439	4.10251
Nc-1-IP_pe	chr10	16583484	16583774	291	16583523	10	4.39571	3.25288
Nc-1-IP_pe	chr10	16585134	16585652	519	16585534	8	3.74505	3.07688
Nc-1-IP_pe	chr10	16599197	16599744	548	16599364	12	6.7561	4.37677
Nc-1-IP_pe	chr10	16626097	16626459	363	16626369	8	3.74505	3.07688
Nc-1-IP_pe	chr10	16633572	16633842	271	16633631	9	4.46937	3.41876
Nc-1-IP_pe	chr10	16688554	16688811	258	16688580	7	3.06442	2.73501
Nc-1-IP_pe	chr10	16730311	16730571	261	16730371	12	6.86906	4.44438
Nc-1-IP_pe	chr10	16750891	16751207	317	16751042	16	9.11744	5.02718
Nc-1-IP_pe	chr10	16766629	16766888	260	16766648	7	3.06442	2.73501
Nc-1-IP_pe	chr10	16808059	16808367	309	16808227	10	4.99888	3.61677
Nc-1-IP_pe	chr10	16817069	16817347	279	16817321	17	6.64406	3.61808
Nc-1-IP_pe	chr10	16897523	16897830	308	16897718	11	5.77463	3.94557
Nc-1-IP_pe	chr10	16952813	16953143	331	16952933	14	8.63073	5.12814
Nc-1-IP_pe	chr10	16981349	16981706	358	16981473	12	6.86906	4.44438
Nc-1-IP_pe	chr10	16984983	16985470	488	16985207	12	5.23082	3.49289
Nc-1-IP_pe	chr10	17004768	17005057	290	17004963	10	5.23357	3.76063
Nc-1-IP_pe	chr10	17026566	17026865	300	17026774	8	3.74505	3.07688
Nc-1-IP_pe	chr10	17057863	17058147	285	17058038	12	6.58417	4.27436
Nc-1-IP_pe	chr10	17118411	17118744	334	17118533	14	8.63073	5.12814
Nc-1-IP_pe	chr10	17201278	17201778	501	17201684	22	12.3673	5.5949
Nc-1-IP_pe	chr10	17216830	17217095	266	17216855	10	4.47495	3.30017
Nc-1-IP_pe	chr10	17228275	17228859	585	17228754	16	8.17084	4.51499
Nc-1-IP_pe	chr10	17229401	17229861	461	17229755	25	4.15604	2.18719
Nc-1-IP_pe	chr10	17252093	17252407	315	17252405	11	3.28475	2.53029
Nc-1-IP_pe	chr10	17346164	17346550	387	17346400	11	5.3563	3.696
Nc-1-IP_pe	chr10	17390957	17391254	298	17391159	12	6.86906	4.44438
Nc-1-IP_pe	chr10	17453298	17454295	998	17453970	17	9.42237	5.01057
Nc-1-IP_pe	chr10	17496107	17496656	550	17496495	10	4.93609	3.57849
Nc-1-IP_pe	chr10	17517099	17517365	267	17517168	9	3.72192	2.95716
Nc-1-IP_pe	chr10	17590946	17591319	374	17591274	8	3.74505	3.07688
Nc-1-IP_pe	chr10	17600986	17601335	350	17601241	10	5.23357	3.76063
Nc-1-IP_pe	chr10	17616866	17617295	430	17617073	13	3.77558	2.63125
Nc-1-IP_pe	chr10	17628733	17629431	699	17628924	14	8.63073	5.12814
Nc-1-IP_pe	chr10	17629751	17630242	492	17629927	13	4.41954	2.952
Nc-1-IP_pe	chr10	17713747	17714049	303	17713898	15	9.55381	5.47001
Nc-1-IP_pe	chr10	17734243	17734514	272	17734392	15	7.46994	4.29894
Nc-1-IP_pe	chr10	17749837	17750192	356	17749893	8	3.64414	3.01254

Nc-1-IP_pe	chr10	17879421	17879673	253	17879431	13	4.02562	2.7544
Nc-1-IP_pe	chr10	17950654	17951108	455	17950995	10	5.23357	3.76063
Nc-1-IP_pe	chr10	17953229	17953507	279	17953372	11	4.06403	2.95415
Nc-1-IP_pe	chr10	17989551	17989903	353	17989864	12	4.70082	3.20033
Nc-1-IP_pe	chr10	18056275	18056544	270	18056487	9	4.05981	3.16422
Nc-1-IP_pe	chr10	18146443	18146893	451	18146828	8	3.08755	2.66145
Nc-1-IP_pe	chr10	18246927	18247241	315	18247118	13	5.9487	3.76157
Nc-1-IP_pe	chr10	18278718	18278990	273	18278812	12	6.86906	4.44438
Nc-1-IP_pe	chr10	18346513	18346768	256	18346634	10	5.23357	3.76063
Nc-1-IP_pe	chr10	18539215	18539601	387	18539337	9	4.46937	3.41876
Nc-1-IP_pe	chr10	18601702	18601965	264	18601829	11	6.03439	4.10251
Nc-1-IP_pe	chr10	18686509	18686847	339	18686654	10	5.23357	3.76063
Nc-1-IP_pe	chr10	18692492	18693010	519	18692550	10	5.23357	3.76063
Nc-1-IP_pe	chr10	18797854	18798117	264	18797940	10	5.23357	3.76063
Nc-1-IP_pe	chr10	18951240	18951624	385	18951409	16	7.11257	3.96852
Nc-1-IP_pe	chr10	19009951	19010215	265	19010062	9	2.89249	2.46179
Nc-1-IP_pe	chr10	19504727	19504993	267	19504900	15	7.46994	4.29894
Nc-1-IP_pe	chr10	19714128	19714601	474	19714317	10	3.89064	2.95552
Nc-1-IP_pe	chr10	19775936	19776219	284	19776005	8	3.74505	3.07688
Nc-1-IP_pe	chr10	19816646	19816982	337	19816735	13	6.62436	4.14003
Nc-1-IP_pe	chr10	19853964	19854259	296	19854065	13	7.7352	4.78626
Nc-1-IP_pe	chr10	20262705	20262957	253	20262861	14	8.63073	5.12814
Nc-1-IP_pe	chr10	20347841	20348162	322	20348027	12	6.58417	4.27436
Nc-1-IP_pe	chr10	20441693	20442207	515	20442035	9	4.46937	3.41876
Nc-1-IP_pe	chr10	20595186	20595455	270	20595283	10	5.23357	3.76063
Nc-1-IP_pe	chr10	20667806	20668231	426	20668065	11	6.03439	4.10251
Nc-1-IP_pe	chr10	20836370	20836641	272	20836517	8	3.74505	3.07688
Nc-1-IP_pe	chr10	20965501	20965832	332	20965570	11	5.03835	3.5092
Nc-1-IP_pe	chr10	20970345	20970629	285	20970449	14	8.29543	4.93196
Nc-1-IP_pe	chr10	21011594	21012183	590	21011666	10	4.07292	3.06202
Nc-1-IP_pe	chr10	21015642	21016142	501	21016107	8	3.56021	2.95918
Nc-1-IP_pe	chr10	21080974	21081238	265	21081036	10	5.23357	3.76063
Nc-1-IP_pe	chr10	21142710	21142964	255	21142832	11	5.10577	3.5486
Nc-1-IP_pe	chr10	21165502	21165900	399	21165616	13	6.62436	4.14003
Nc-1-IP_pe	chr10	21173464	21174648	1185	21173709	19	9.03708	4.48779
Nc-1-IP_pe	chr10	21181652	21181985	334	21181720	8	3.74505	3.07688
Nc-1-IP_pe	chr10	21189235	21189700	466	21189391	13	7.42513	4.60316
Nc-1-IP_pe	chr10	21192614	21192944	331	21192746	14	8.44792	5.0209
Nc-1-IP_pe	chr10	21259261	21259620	360	21259491	12	4.24064	2.95299
Nc-1-IP_pe	chr10	21266608	21267310	703	21266786	12	5.15877	3.45264
Nc-1-IP_pe	chr10	21278146	21278416	271	21278216	11	5.03835	3.5092
Nc-1-IP_pe	chr10	21297051	21297342	292	21297243	9	3.72947	2.96176
Nc-1-IP_pe	chr10	21299498	21299921	424	21299872	12	3.83688	2.74114
Nc-1-IP_pe	chr10	21305426	21305959	534	21305927	9	3.27326	2.68684
Nc-1-IP_pe	chr10	21327312	21327710	399	21327436	10	3.08907	2.49869

Nc-1-IP_pe	chr10	21348407	21348735	329	21348593	13	7.32024	4.54167
Nc-1-IP_pe	chr10	21350496	21351033	538	21350682	12	6.58417	4.27436
Nc-1-IP_pe	chr10	21352298	21352562	265	21352408	13	7.7352	4.78626
Nc-1-IP_pe	chr10	21360588	21360847	260	21360764	12	6.58898	4.27722
Nc-1-IP_pe	chr10	21362791	21363128	338	21362855	10	4.20113	3.13749
Nc-1-IP_pe	chr10	21371420	21371686	267	21371515	11	3.64839	2.72584
Nc-1-IP_pe	chr10	21396443	21396719	277	21396546	16	10.4665	5.79086
Nc-1-IP_pe	chr10	21398008	21398461	454	21398347	10	5.23357	3.76063
Nc-1-IP_pe	chr10	21401117	21401666	550	21401118	10	3.08907	2.49869
Nc-1-IP_pe	chr10	21402196	21402551	356	21402480	11	2.96397	2.36091
Nc-1-IP_pe	chr10	21405890	21406152	263	21405950	10	3.89064	2.95552
Nc-1-IP_pe	chr10	21415390	21415815	426	21415581	13	7.65891	4.74103
Nc-1-IP_pe	chr10	21416321	21416694	374	21416388	11	4.54404	3.2242
Nc-1-IP_pe	chr10	21426832	21427133	302	21427007	15	6.19428	3.63445
Nc-1-IP_pe	chr10	21430305	21430738	434	21430625	17	9.89113	5.26381
Nc-1-IP_pe	chr10	21439083	21439345	263	21439214	9	4.32403	3.32802
Nc-1-IP_pe	chr10	21457726	21458047	322	21457924	9	4.25966	3.28797
Nc-1-IP_pe	chr10	21471117	21471537	421	21471359	11	5.7051	3.90381
Nc-1-IP_pe	chr10	21473741	21474024	284	21473827	11	4.54404	3.2242
Nc-1-IP_pe	chr10	21495932	21497144	1213	21495997	18	5.33197	2.94862
Nc-1-IP_pe	chr10	21500252	21500810	559	21500364	14	5.43414	3.36584
Nc-1-IP_pe	chr10	21509848	21510596	749	21510004	16	9.11744	5.02718
Nc-1-IP_pe	chr10	21533684	21533955	272	21533748	15	4.70031	2.91253
Nc-1-IP_pe	chr10	21543417	21543769	353	21543617	12	6.86906	4.44438
Nc-1-IP_pe	chr10	21546261	21546563	303	21546470	11	5.89269	4.01672
Nc-1-IP_pe	chr10	21554755	21555169	415	21554946	10	5.23357	3.76063
Nc-1-IP_pe	chr10	21567930	21568193	264	21568064	10	3.94999	2.99009
Nc-1-IP_pe	chr10	21621231	21621684	454	21621495	9	4.46937	3.41876
Nc-1-IP_pe	chr10	21665206	21665567	362	21665401	9	3.27326	2.68684
Nc-1-IP_pe	chr10	21676251	21676638	388	21676484	12	6.86906	4.44438
Nc-1-IP_pe	chr10	21699009	21699270	262	21699123	11	6.03439	4.10251
Nc-1-IP_pe	chr10	21704961	21705802	842	21705526	11	5.19376	3.60018
Nc-1-IP_pe	chr10	21714511	21714767	257	21714579	11	6.03439	4.10251
Nc-1-IP_pe	chr10	21737696	21738198	503	21738047	10	5.23357	3.76063
Nc-1-IP_pe	chr10	21746799	21747127	329	21746924	8	3.53695	2.94441
Nc-1-IP_pe	chr10	21752244	21752759	516	21752639	15	7.46994	4.29894
Nc-1-IP_pe	chr10	21755277	21755704	428	21755325	9	4.2632	3.29017
Nc-1-IP_pe	chr10	21781393	21781742	350	21781503	9	4.05981	3.16422
Nc-1-IP_pe	chr10	21834384	21834738	355	21834551	18	6.65364	3.51391
Nc-1-IP_pe	chr10	21836930	21837543	614	21837064	16	6.33285	3.58457
Nc-1-IP_pe	chr10	21837969	21838245	277	21838127	14	5.51541	3.4073
Nc-1-IP_pe	chr10	21895980	21896285	306	21896225	9	4.46937	3.41876
Nc-1-IP_pe	chr10	22021846	22022129	284	22022078	11	5.19376	3.60018
Nc-1-IP_pe	chr10	22051363	22051677	315	22051570	8	3.56021	2.95918
Nc-1-IP_pe	chr10	22089475	22089836	362	22089520	10	5.23357	3.76063

Nc-1-IP_pe	chr10	22102931	22103185	255	22103091	9	4.46937	3.41876
Nc-1-IP_pe	chr10	22184806	22185102	297	22184857	8	3.08755	2.66145
Nc-1-IP_pe	chr10	22229256	22229575	320	22229403	14	6.06519	3.69269
Nc-1-IP_pe	chr10	22239102	22239373	272	22239141	10	5.23357	3.76063
Nc-1-IP_pe	chr10	22252307	22252736	430	22252592	13	4.90859	3.20357
Nc-1-IP_pe	chr10	22267465	22267934	470	22267785	13	7.7352	4.78626
Nc-1-IP_pe	chr10	22335995	22336922	928	22336768	9	4.46937	3.41876
Nc-1-IP_pe	chr10	22340533	22340988	456	22340933	14	3.55308	2.45762
Nc-1-IP_pe	chr10	22436614	22437304	691	22436782	17	9.42237	5.01057
Nc-1-IP_pe	chr10	22437567	22437942	376	22437849	11	6.03439	4.10251
Nc-1-IP_pe	chr10	22445095	22445366	272	22445248	12	4.24064	2.95299
Nc-1-IP_pe	chr10	22453025	22453340	316	22453141	10	5.23357	3.76063
Nc-1-IP_pe	chr10	22462742	22463097	356	22462774	8	3.74505	3.07688
Nc-1-IP_pe	chr10	22475781	22476292	512	22476056	10	3.4604	2.70797
Nc-1-IP_pe	chr10	22477708	22478251	544	22478017	15	8.26005	4.73146
Nc-1-IP_pe	chr10	22537865	22538183	319	22537910	9	3.72192	2.95716
Nc-1-IP_pe	chr10	22544291	22544571	281	22544378	12	4.70082	3.20033
Nc-1-IP_pe	chr10	22580908	22581264	357	22581090	11	4.54404	3.2242
Nc-1-IP_pe	chr10	22583329	22583589	261	22583544	10	3.94999	2.99009
Nc-1-IP_pe	chr10	22608064	22608507	444	22608352	11	6.03439	4.10251
Nc-1-IP_pe	chr10	22612822	22613620	799	22612947	13	5.9487	3.76157
Nc-1-IP_pe	chr10	22622331	22622587	257	22622490	13	6.62436	4.14003
Nc-1-IP_pe	chr10	22649473	22649837	365	22649571	12	5.23082	3.49289
Nc-1-IP_pe	chr10	22651155	22651811	657	22651374	11	6.03439	4.10251
Nc-1-IP_pe	chr10	22710247	22710591	345	22710465	7	2.89555	2.62467
Nc-1-IP_pe	chr10	22714714	22714998	285	22714911	12	6.16157	4.02543
Nc-1-IP_pe	chr10	22885692	22886223	532	22885855	12	5.84943	3.84431
Nc-1-IP_pe	chr10	22893298	22893734	437	22893403	11	5.10577	3.5486
Nc-1-IP_pe	chr10	23022786	23023115	330	23023046	10	5.23357	3.76063
Nc-1-IP_pe	chr10	23093772	23094034	263	23093872	9	4.25966	3.28797
Nc-1-IP_pe	chr10	23095502	23095901	400	23095742	16	10.5028	5.81189
Nc-1-IP_pe	chr10	23104341	23104891	551	23104412	9	4.46937	3.41876
Nc-1-IP_pe	chr10	23119055	23119339	285	23119230	9	4.25966	3.28797
Nc-1-IP_pe	chr10	23135651	23135916	266	23135721	8	3.74505	3.07688
Nc-1-IP_pe	chr10	23144339	23144628	290	23144423	8	3.74505	3.07688
Nc-1-IP_pe	chr10	23145710	23146086	377	23145913	14	8.63073	5.12814
Nc-1-IP_pe	chr10	23150963	23151360	398	23151193	11	3.64839	2.72584
Nc-1-IP_pe	chr10	23157454	23157912	459	23157524	9	3.66792	2.92434
Nc-1-IP_pe	chr10	23159038	23159446	409	23159149	9	4.46937	3.41876
Nc-1-IP_pe	chr10	23163477	23164252	776	23163917	9	3.92293	3.08
Nc-1-IP_pe	chr10	23171534	23171795	262	23171732	10	5.23357	3.76063
Nc-1-IP_pe	chr10	23197225	23197756	532	23197304	8	3.56021	2.95918
Nc-1-IP_pe	chr10	23199566	23199856	291	23199747	12	6.86906	4.44438
Nc-1-IP_pe	chr10	23282422	23282731	310	23282592	15	9.55381	5.47001
Nc-1-IP_pe	chr10	23301726	23302015	290	23301904	13	7.10772	4.41783

Nc-1-IP_pe	chr10	23316947	23317227	281	23317054	13	4.86362	3.18015
Nc-1-IP_pe	chr10	23390284	23390875	592	23390707	14	7.42849	4.43574
Nc-1-IP_pe	chr10	23439182	23439560	379	23439511	11	3.58722	2.69267
Nc-1-IP_pe	chr10	23444807	23445193	387	23445057	12	6.86906	4.44438
Nc-1-IP_pe	chr10	23449595	23449980	386	23449785	9	3.72192	2.95716
Nc-1-IP_pe	chr10	23694401	23695621	1221	23694710	13	7.7352	4.78626
Nc-1-IP_pe	chr10	24067816	24068138	323	24068079	10	4.99888	3.61677
Nc-1-IP_pe	chr10	24176747	24177005	259	24176923	12	4.70082	3.20033
Nc-1-IP_pe	chr10	24220537	24220957	421	24220668	8	3.74505	3.07688
Nc-1-IP_pe	chr10	24227838	24228175	338	24227902	9	4.25966	3.28797
Nc-1-IP_pe	chr10	24258558	24258915	358	24258733	12	5.23082	3.49289
Nc-1-IP_pe	chr10	24315419	24315751	333	24315647	10	5.23357	3.76063
Nc-1-IP_pe	chr10	24377090	24377357	268	24377187	8	3.74505	3.07688
Nc-1-IP_pe	chr10	24413289	24413594	306	24413442	10	3.89064	2.95552
Nc-1-IP_pe	chr10	24426389	24426664	276	24426472	11	6.03439	4.10251
Nc-1-IP_pe	chr10	24479764	24480056	293	24479972	11	5.77463	3.94557
Nc-1-IP_pe	chr10	24484455	24484879	425	24484615	9	4.35492	3.34727
Nc-1-IP_pe	chr10	24569284	24569549	266	24569486	9	4.25966	3.28797
Nc-1-IP_pe	chr10	24612572	24612858	287	24612750	10	5.23357	3.76063
Nc-1-IP_pe	chr10	24648448	24648729	282	24648593	11	5.77463	3.94557
Nc-1-IP_pe	chr10	24655720	24656540	821	24656094	25	17.8013	7.68862
Nc-1-IP_pe	chr10	24659433	24659749	317	24659629	10	4.39571	3.25288
Nc-1-IP_pe	chr10	24695842	24696403	562	24695857	9	3.12089	2.59629
Nc-1-IP_pe	chr10	24704452	24704720	269	24704565	12	5.23082	3.49289
Nc-1-IP_pe	chr10	24721587	24721944	358	24721689	15	8.38325	4.80024
Nc-1-IP_pe	chr10	24722648	24722998	351	24722804	13	4.45253	2.96876
Nc-1-IP_pe	chr10	24724078	24724476	399	24724383	15	4.90347	3.00714
Nc-1-IP_pe	chr10	24737541	24737906	366	24737680	13	5.9487	3.76157
Nc-1-IP_pe	chr10	24749158	24749761	604	24749329	9	3.92293	3.08
Nc-1-IP_pe	chr10	24785129	24785560	432	24785331	15	6.19428	3.63445
Nc-1-IP_pe	chr10	24796221	24796732	512	24796679	10	3.4604	2.70797
Nc-1-IP_pe	chr10	24820725	24821114	390	24821002	12	5.77527	3.80164
Nc-1-IP_pe	chr10	24852305	24852622	318	24852459	11	5.10577	3.5486
Nc-1-IP_pe	chr10	24859231	24859510	280	24859362	14	6.78215	4.0774
Nc-1-IP_pe	chr10	24923182	24923434	253	24923190	9	3.72192	2.95716
Nc-1-IP_pe	chr10	24944115	24944580	466	24944116	11	3.64839	2.72584
Nc-1-IP_pe	chr10	24950985	24951252	268	24951067	11	4.88957	3.42271
Nc-1-IP_pe	chr10	24972238	24972491	254	24972403	9	4.46937	3.41876
Nc-1-IP_pe	chr10	25062564	25063342	779	25063172	11	4.06403	2.95415
Nc-1-IP_pe	chr10	25077503	25077805	303	25077609	11	6.03439	4.10251
Nc-1-IP_pe	chr10	25102963	25103425	463	25103066	11	5.7051	3.90381
Nc-1-IP_pe	chr10	25143282	25143681	400	25143603	9	4.25966	3.28797
Nc-1-IP_pe	chr10	25151313	25151571	259	25151400	11	4.54404	3.2242
Nc-1-IP_pe	chr10	25174846	25176251	1406	25174906	11	5.03835	3.5092
Nc-1-IP_pe	chr10	25194180	25194475	296	25194382	10	5.23357	3.76063

Nc-1-IP_pe	chr10	25252521	25252860	340	25252604	15	6.19428	3.63445
Nc-1-IP_pe	chr10	25392071	25392436	366	25392399	11	6.03439	4.10251
Nc-1-IP_pe	chr10	25574695	25574968	274	25574801	9	4.25966	3.28797
Nc-1-IP_pe	chr10	25597996	25598298	303	25598057	9	3.72192	2.95716
Nc-1-IP_pe	chr10	25615688	25616080	393	25615934	9	4.20361	3.25317
Nc-1-IP_pe	chr10	25619934	25620472	539	25620319	13	7.12853	4.42991
Nc-1-IP_pe	chr10	25660883	25661236	354	25661049	9	4.20361	3.25317
Nc-1-IP_pe	chr10	25685096	25685409	314	25685254	14	7.42849	4.43574
Nc-1-IP_pe	chr10	25694380	25695148	769	25694574	12	6.12485	4.004
Nc-1-IP_pe	chr10	25870174	25870648	475	25870251	8	3.74505	3.07688
Nc-1-IP_pe	chr10	25901495	25901751	257	25901660	11	6.03439	4.10251
Nc-1-IP_pe	chr10	25934116	25935041	926	25934377	13	7.7352	4.78626
Nc-1-IP_pe	chr10	25995812	25996130	319	25995975	12	6.58417	4.27436
Nc-1-IP_pe	chr10	26118728	26119053	326	26118901	12	6.86906	4.44438
Nc-1-IP_pe	chr10	26175755	26176053	299	26175882	9	4.46937	3.41876
Nc-1-IP_pe	chr10	26177991	26178313	323	26178151	12	6.31185	4.11348
Nc-1-IP_pe	chr10	26211751	26212056	306	26211956	13	5.36853	3.44651
Nc-1-IP_pe	chr10	26214830	26215187	358	26214874	8	3.56021	2.95918
Nc-1-IP_pe	chr10	26217723	26218332	610	26217856	12	4.70082	3.20033
Nc-1-IP_pe	chr10	26227730	26228118	389	26227792	10	3.89064	2.95552
Nc-1-IP_pe	chr10	26236931	26237199	269	26236942	8	3.56021	2.95918
Nc-1-IP_pe	chr10	26244911	26245202	292	26244986	9	4.46937	3.41876
Nc-1-IP_pe	chr10	26401230	26401670	441	26401437	13	6.62436	4.14003
Nc-1-IP_pe	chr10	26480088	26480383	296	26480247	13	7.7352	4.78626
Nc-1-IP_pe	chr10	26505727	26505998	272	26505967	8	3.74505	3.07688
Nc-1-IP_pe	chr10	26534538	26534874	337	26534611	11	6.03439	4.10251
Nc-1-IP_pe	chr10	26551111	26551430	320	26551134	12	6.71369	4.35145
Nc-1-IP_pe	chr10	26579348	26579720	373	26579452	10	5.23357	3.76063
Nc-1-IP_pe	chr10	26610050	26610322	273	26610117	8	3.74505	3.07688
Nc-1-IP_pe	chr10	26640075	26640691	617	26640194	10	5.23357	3.76063
Nc-1-IP_pe	chr10	26643205	26643499	295	26643281	12	6.86906	4.44438
Nc-1-IP_pe	chr10	26652202	26652470	269	26652417	10	5.23357	3.76063
Nc-1-IP_pe	chr10	26653012	26653303	292	26653191	10	5.23357	3.76063
Nc-1-IP_pe	chr10	26697578	26698151	574	26697901	17	7.6273	4.08876
Nc-1-IP_pe	chr10	26707484	26708045	562	26707979	9	4.35492	3.34727
Nc-1-IP_pe	chr10	26709373	26709630	258	26709413	10	5.23357	3.76063
Nc-1-IP_pe	chr10	26715165	26715500	336	26715470	9	3.72192	2.95716
Nc-1-IP_pe	chr10	26718274	26718699	426	26718649	8	3.56021	2.95918
Nc-1-IP_pe	chr10	26730081	26730552	472	26730517	11	6.03439	4.10251
Nc-1-IP_pe	chr10	26731847	26732170	324	26731952	11	6.03439	4.10251
Nc-1-IP_pe	chr10	26800563	26800959	397	26800802	15	8.26005	4.73146
Nc-1-IP_pe	chr10	26820620	26820889	270	26820744	10	5.23357	3.76063
Nc-1-IP_pe	chr10	26860215	26860878	664	26860741	18	8.27052	4.2634
Nc-1-IP_pe	chr10	26868883	26869323	441	26869044	9	4.46937	3.41876
Nc-1-IP_pe	chr10	26887151	26887407	257	26887253	10	4.99888	3.61677

Nc-1-IP_pe	chr10	26890543	26890903	361	26890709	10	3.94999	2.99009
Nc-1-IP_pe	chr10	26892482	26892807	326	26892583	9	4.25966	3.28797
Nc-1-IP_pe	chr10	26935044	26935323	280	26935155	12	6.58417	4.27436
Nc-1-IP_pe	chr10	26946056	26946775	720	26946130	11	4.54404	3.2242
Nc-1-IP_pe	chr10	27016300	27016581	282	27016474	13	7.42513	4.60316
Nc-1-IP_pe	chr10	27059770	27060025	256	27059938	13	6.62436	4.14003
Nc-1-IP_pe	chr10	27071998	27072753	756	27072109	9	4.46937	3.41876
Nc-1-IP_pe	chr10	27111204	27111581	378	27111270	8	3.56021	2.95918
Nc-1-IP_pe	chr10	27132112	27132768	657	27132271	11	6.03439	4.10251
Nc-1-IP_pe	chr10	27155290	27155747	458	27155480	14	5.03085	3.16286
Nc-1-IP_pe	chr10	27178141	27178396	256	27178279	13	6.62436	4.14003
Nc-1-IP_pe	chr10	27181685	27182101	417	27181897	9	4.25966	3.28797
Nc-1-IP_pe	chr10	27216844	27217148	305	27217044	12	5.61153	3.70794
Nc-1-IP_pe	chr10	27233491	27233802	312	27233542	7	2.9043	2.63038
Nc-1-IP_pe	chr10	27250073	27250585	513	27250446	11	5.49185	3.77641
Nc-1-IP_pe	chr10	27258782	27259183	402	27259093	11	3.64839	2.72584
Nc-1-IP_pe	chr10	27296430	27297262	833	27296592	12	6.86906	4.44438
Nc-1-IP_pe	chr10	27299299	27299748	450	27299534	12	6.31185	4.11348
Nc-1-IP_pe	chr10	27319656	27319977	322	27319814	16	8.79222	4.84884
Nc-1-IP_pe	chr10	27357012	27357378	367	27357211	12	6.71369	4.35145
Nc-1-IP_pe	chr10	27387298	27387615	318	27387391	13	4.86362	3.18015
Nc-1-IP_pe	chr10	27389049	27389388	340	27389235	10	4.39571	3.25288
Nc-1-IP_pe	chr10	27413914	27414493	580	27414322	12	6.86906	4.44438
Nc-1-IP_pe	chr10	27504119	27504376	258	27504276	13	3.07238	2.29378
Nc-1-IP_pe	chr10	27587189	27587921	733	27587679	11	5.19376	3.60018
Nc-1-IP_pe	chr10	27599086	27599383	298	27599355	12	4.24064	2.95299
Nc-1-IP_pe	chr10	27742564	27743744	1181	27743568	12	5.84943	3.84431
Nc-1-IP_pe	chr10	27744238	27744490	253	27744346	9	4.20361	3.25317
Nc-1-IP_pe	chr10	27745224	27745780	557	27745591	12	5.77527	3.80164
Nc-1-IP_pe	chr10	27876022	27876461	440	27876254	10	5.23357	3.76063
Nc-1-IP_pe	chr10	27910477	27910737	261	27910595	11	6.03439	4.10251
Nc-1-IP_pe	chr10	27941856	27942155	300	27941999	11	4.54404	3.2242
Nc-1-IP_pe	chr10	28063492	28063745	254	28063614	11	6.03439	4.10251
Nc-1-IP_pe	chr10	28127706	28128019	314	28127868	9	4.46937	3.41876
Nc-1-IP_pe	chr10	28276080	28276378	299	28276205	7	3.06442	2.73501
Nc-1-IP_pe	chr10	28309725	28310549	825	28310380	10	5.23357	3.76063
Nc-1-IP_pe	chr10	28388589	28388846	258	28388815	8	3.56021	2.95918
Nc-1-IP_pe	chr10	28392268	28392598	331	28392415	12	6.86906	4.44438
Nc-1-IP_pe	chr10	28416253	28416596	344	28416449	12	6.86906	4.44438
Nc-1-IP_pe	chr10	28446168	28446440	273	28446193	7	3.06442	2.73501
Nc-1-IP_pe	chr10	28522809	28523158	350	28522834	8	3.64414	3.01254
Nc-1-IP_pe	chr10	28531923	28532693	771	28532395	19	8.19109	4.09114
Nc-1-IP_pe	chr10	28643277	28644015	739	28643563	14	5.51541	3.4073
Nc-1-IP_pe	chr10	28650290	28650584	295	28650552	8	3.14944	2.70014
Nc-1-IP_pe	chr10	28653205	28653528	324	28653294	12	6.86906	4.44438

Nc-1-IP_pe	chr10	28658267	28658746	480	28658338	10	4.33501	3.21677
Nc-1-IP_pe	chr10	28660359	28660651	293	28660515	14	5.51541	3.4073
Nc-1-IP_pe	chr10	28684242	28684576	335	28684362	11	5.77463	3.94557
Nc-1-IP_pe	chr10	28697379	28697739	361	28697622	12	6.40869	4.1705
Nc-1-IP_pe	chr10	28711680	28711998	319	28711961	9	4.46937	3.41876
Nc-1-IP_pe	chr10	28713223	28713499	277	28713309	14	6.69566	4.03025
Nc-1-IP_pe	chr10	28722132	28722676	545	28722597	12	6.86906	4.44438
Nc-1-IP_pe	chr10	28728857	28729342	486	28729251	10	5.23357	3.76063
Nc-1-IP_pe	chr10	28730615	28730901	287	28730868	8	3.74505	3.07688
Nc-1-IP_pe	chr10	28741017	28741317	301	28741148	11	6.03439	4.10251
Nc-1-IP_pe	chr10	28743442	28743780	339	28743582	12	5.23082	3.49289
Nc-1-IP_pe	chr10	28771335	28772098	764	28771718	15	5.20142	3.14789
Nc-1-IP_pe	chr10	28791963	28792229	267	28792127	10	4.20113	3.13749
Nc-1-IP_pe	chr10	28793676	28794032	357	28793791	8	3.08755	2.66145
Nc-1-IP_pe	chr10	28796752	28797385	634	28796886	10	5.23357	3.76063
Nc-1-IP_pe	chr10	28812956	28813295	340	28813092	12	4.24064	2.95299
Nc-1-IP_pe	chr10	28817988	28818271	284	28818157	14	7.42849	4.43574
Nc-1-IP_pe	chr10	28882664	28882932	269	28882764	17	11.4763	6.15376
Nc-1-IP_pe	chr10	28896730	28897286	557	28897197	14	7.42849	4.43574
Nc-1-IP_pe	chr10	28924870	28925166	297	28925034	14	7.9745	4.74633
Nc-1-IP_pe	chr10	28936314	28936987	674	28936514	11	6.03439	4.10251
Nc-1-IP_pe	chr10	29008843	29009111	269	29009031	9	4.46937	3.41876
Nc-1-IP_pe	chr10	29027987	29028559	573	29028405	11	3.28475	2.53029
Nc-1-IP_pe	chr10	29055971	29056324	354	29056104	9	3.72192	2.95716
Nc-1-IP_pe	chr10	29095308	29095734	427	29095673	8	3.74505	3.07688
Nc-1-IP_pe	chr10	29101656	29101919	264	29101777	13	6.92473	4.312
Nc-1-IP_pe	chr10	29156258	29156573	316	29156332	11	5.05359	3.5181
Nc-1-IP_pe	chr10	29189325	29189729	405	29189502	14	8.20556	4.87976
Nc-1-IP_pe	chr10	29198532	29198786	255	29198686	10	5.23357	3.76063
Nc-1-IP_pe	chr10	29201288	29201997	710	29201316	10	5.23357	3.76063
Nc-1-IP_pe	chr10	29206771	29207501	731	29207417	12	6.86906	4.44438
Nc-1-IP_pe	chr10	29229294	29229973	680	29229798	13	5.9487	3.76157
Nc-1-IP_pe	chr10	29250460	29250733	274	29250580	12	6.86906	4.44438
Nc-1-IP_pe	chr10	29381490	29381841	352	29381776	10	4.25398	3.16873
Nc-1-IP_pe	chr10	29393444	29393864	421	29393578	12	6.50787	4.22912
Nc-1-IP_pe	chr10	29394529	29394884	356	29394714	13	5.36853	3.44651
Nc-1-IP_pe	chr10	29395382	29395777	396	29395425	8	3.52386	2.93611
Nc-1-IP_pe	chr10	29427588	29427927	340	29427763	11	5.7051	3.90381
Nc-1-IP_pe	chr10	29428374	29428786	413	29428688	11	5.7051	3.90381
Nc-1-IP_pe	chr10	29438226	29438665	440	29438418	12	6.86906	4.44438
Nc-1-IP_pe	chr10	29463438	29463858	421	29463610	12	5.77527	3.80164
Nc-1-IP_pe	chr10	29464738	29465423	686	29465016	10	5.23357	3.76063
Nc-1-IP_pe	chr10	29469424	29470257	834	29469558	12	6.31185	4.11348
Nc-1-IP_pe	chr10	29479183	29479538	356	29479268	12	5.23082	3.49289
Nc-1-IP_pe	chr10	29480442	29481082	641	29480604	10	4.91962	3.56846

Nc-1-IP_pe	chr10	29487224	29487478	255	29487336	11	5.10577	3.5486
Nc-1-IP_pe	chr10	29493840	29494225	386	29493929	10	5.12294	3.69268
Nc-1-IP_pe	chr10	29495492	29495745	254	29495552	13	5.36853	3.44651
Nc-1-IP_pe	chr10	29498877	29499193	317	29498896	9	4.05981	3.16422
Nc-1-IP_pe	chr10	29510545	29510835	291	29510678	19	8.4638	4.21715
Nc-1-IP_pe	chr10	29516356	29516851	496	29516737	15	9.51988	5.45022
Nc-1-IP_pe	chr10	29518782	29519327	546	29519297	8	3.08755	2.66145
Nc-1-IP_pe	chr10	29523633	29523966	334	29523883	10	5.21145	3.74702
Nc-1-IP_pe	chr10	29562541	29562867	327	29562577	10	3.08907	2.49869
Nc-1-IP_pe	chr10	29563963	29564620	658	29564371	12	5.84943	3.84431
Nc-1-IP_pe	chr10	29565486	29565867	382	29565648	9	4.25966	3.28797
Nc-1-IP_pe	chr10	29574637	29574944	308	29574709	15	9.55381	5.47001
Nc-1-IP_pe	chr10	29579530	29579875	346	29579753	11	4.54404	3.2242
Nc-1-IP_pe	chr10	29584159	29584903	745	29584295	15	8.26005	4.73146
Nc-1-IP_pe	chr10	29608720	29608972	253	29608893	13	5.42865	3.47872
Nc-1-IP_pe	chr10	29631158	29631556	399	29631312	12	4.70082	3.20033
Nc-1-IP_pe	chr10	29669234	29669918	685	29669351	12	5.94619	3.9002
Nc-1-IP_pe	chr10	29680821	29681083	263	29680872	12	4.24064	2.95299
Nc-1-IP_pe	chr10	29696723	29697360	638	29696967	14	8.63073	5.12814
Nc-1-IP_pe	chr10	29726609	29726888	280	29726644	10	3.89064	2.95552
Nc-1-IP_pe	chr10	29728771	29729132	362	29728974	12	6.58417	4.27436
Nc-1-IP_pe	chr10	29734984	29735742	759	29735268	15	6.10627	3.59023
Nc-1-IP_pe	chr10	29778679	29779009	331	29778896	12	6.56855	4.26509
Nc-1-IP_pe	chr10	29813920	29814849	930	29814108	15	8.70419	4.98103
Nc-1-IP_pe	chr10	29821035	29821380	346	29821241	14	6.06519	3.69269
Nc-1-IP_pe	chr10	29843905	29844368	464	29844313	11	5.24175	3.6284
Nc-1-IP_pe	chr10	29868562	29868823	262	29868688	11	5.10577	3.5486
Nc-1-IP_pe	chr10	29886534	29886789	256	29886545	12	3.83688	2.74114
Nc-1-IP_pe	chr10	29921179	29921594	416	29921235	13	4.41954	2.952
Nc-1-IP_pe	chr10	29932091	29932642	552	29932476	15	9.19325	5.26076
Nc-1-IP_pe	chr10	29935040	29935320	281	29935183	11	4.54404	3.2242
Nc-1-IP_pe	chr10	29973969	29974233	265	29974175	9	4.40188	3.37657
Nc-1-IP_pe	chr10	29979644	29980423	780	29980303	13	7.7352	4.78626
Nc-1-IP_pe	chr10	30022092	30022363	272	30022219	13	7.7352	4.78626
Nc-1-IP_pe	chr10	30026257	30027061	805	30026890	16	6.80394	3.81462
Nc-1-IP_pe	chr10	30027364	30028271	908	30027474	13	4.67336	3.0817
Nc-1-IP_pe	chr10	30030227	30030479	253	30030312	11	5.03835	3.5092
Nc-1-IP_pe	chr10	30039104	30039816	713	30039757	15	5.20142	3.14789
Nc-1-IP_pe	chr10	30058600	30059344	745	30059149	21	9.29881	4.32834
Nc-1-IP_pe	chr10	30081424	30081687	264	30081626	10	5.00283	3.61918
Nc-1-IP_pe	chr10	30097280	30097622	343	30097482	12	5.64372	3.72631
Nc-1-IP_pe	chr10	30123538	30123920	383	30123760	8	3.74505	3.07688
Nc-1-IP_pe	chr10	30151788	30152097	310	30151871	13	6.19316	3.89711
Nc-1-IP_pe	chr10	30177552	30178215	664	30177889	10	4.39571	3.25288
Nc-1-IP_pe	chr10	30204417	30204686	270	30204618	8	3.74505	3.07688

Nc-1-IP_pe	chr10	30285717	30286082	366	30285824	10	4.62142	3.388
Nc-1-IP_pe	chr10	30332813	30333079	267	30332864	10	4.99888	3.61677
Nc-1-IP_pe	chr10	30348891	30349768	878	30348998	9	3.27326	2.68684
Nc-1-IP_pe	chr10	30355053	30355429	377	30355409	11	4.06403	2.95415
Nc-1-IP_pe	chr10	30356992	30357503	512	30357432	13	3.35769	2.42919
Nc-1-IP_pe	chr10	30364690	30365037	348	30364871	16	5.82808	3.34463
Nc-1-IP_pe	chr10	30365831	30366167	337	30365905	13	3.35769	2.42919
Nc-1-IP_pe	chr10	30366497	30366777	281	30366644	16	7.53857	4.18505
Nc-1-IP_pe	chr10	30370477	30371013	537	30370567	11	4.55694	3.23155
Nc-1-IP_pe	chr10	30374270	30374640	371	30374344	14	6.44441	3.89444
Nc-1-IP_pe	chr10	30403202	30403756	555	30403494	15	9.55381	5.47001
Nc-1-IP_pe	chr10	30427852	30428346	495	30428307	10	4.90322	3.55848
Nc-1-IP_pe	chr10	30455712	30456059	348	30455874	12	6.86906	4.44438
Nc-1-IP_pe	chr10	30471067	30471410	344	30471297	11	5.93136	4.04009
Nc-1-IP_pe	chr10	30531595	30531884	290	30531660	13	3.67377	2.58159
Nc-1-IP_pe	chr10	30575403	30575683	281	30575602	13	6.62436	4.14003
Nc-1-IP_pe	chr10	30587658	30587946	289	30587799	13	5.36853	3.44651
Nc-1-IP_pe	chr10	30589456	30589754	299	30589532	10	5.23357	3.76063
Nc-1-IP_pe	chr10	30593344	30593638	295	30593474	13	5.36853	3.44651
Nc-1-IP_pe	chr10	30605412	30605835	424	30605569	13	4.02562	2.7544
Nc-1-IP_pe	chr10	30611036	30611419	384	30611289	8	3.69948	3.0478
Nc-1-IP_pe	chr10	30614248	30614531	284	30614323	12	5.84943	3.84431
Nc-1-IP_pe	chr10	30617309	30617623	315	30617469	10	5.23357	3.76063
Nc-1-IP_pe	chr10	30626068	30626717	650	30626387	15	6.24734	3.66122
Nc-1-IP_pe	chr10	30643661	30644132	472	30644070	10	4.91962	3.56846
Nc-1-IP_pe	chr10	30677325	30677707	383	30677654	9	4.46937	3.41876
Nc-1-IP_pe	chr10	30691985	30692399	415	30692058	9	4.40188	3.37657
Nc-1-IP_pe	chr10	30705240	30705506	267	30705294	11	5.56156	3.81794
Nc-1-IP_pe	chr10	30710608	30711294	687	30711168	10	4.39571	3.25288
Nc-1-IP_pe	chr10	30716211	30716673	463	30716380	11	6.03439	4.10251
Nc-1-IP_pe	chr10	30738837	30739267	431	30739094	13	6.59853	4.12535
Nc-1-IP_pe	chr10	30748604	30749018	415	30748888	11	5.3563	3.696
Nc-1-IP_pe	chr10	30749484	30749745	262	30749590	11	5.10577	3.5486
Nc-1-IP_pe	chr10	30753205	30753641	437	30753544	10	4.93609	3.57849
Nc-1-IP_pe	chr10	30761228	30761855	628	30761443	9	4.46937	3.41876
Nc-1-IP_pe	chr10	30764712	30765074	363	30764908	13	5.9487	3.76157
Nc-1-IP_pe	chr10	30767468	30767738	271	30767554	9	4.46937	3.41876
Nc-1-IP_pe	chr10	30768494	30768844	351	30768674	12	6.86906	4.44438
Nc-1-IP_pe	chr10	30769271	30769599	329	30769471	12	4.70082	3.20033
Nc-1-IP_pe	chr10	30775426	30776150	725	30775670	9	3.72192	2.95716
Nc-1-IP_pe	chr10	30779389	30779904	516	30779596	11	5.77463	3.94557
Nc-1-IP_pe	chr10	30783986	30784392	407	30784167	11	5.81652	3.97078
Nc-1-IP_pe	chr10	30785088	30785640	553	30785450	12	5.84943	3.84431
Nc-1-IP_pe	chr10	30786836	30787231	396	30787033	13	4.41954	2.952
Nc-1-IP_pe	chr10	30800489	30800787	299	30800667	12	6.82053	4.41531

Nc-1-IP_pe	chr10	30805102	30805449	348	30805199	15	8.61047	4.928
Nc-1-IP_pe	chr10	30806842	30807175	334	30806967	13	7.7352	4.78626
Nc-1-IP_pe	chr10	30815828	30816111	284	30815902	12	5.84943	3.84431
Nc-1-IP_pe	chr10	30818347	30818619	273	30818509	12	5.3037	3.53374
Nc-1-IP_pe	chr10	30822436	30822843	408	30822456	9	3.54889	2.85226
Nc-1-IP_pe	chr10	30824998	30825363	366	30825223	12	5.99895	3.93077
Nc-1-IP_pe	chr10	30828918	30829472	555	30829092	13	7.17043	4.45427
Nc-1-IP_pe	chr10	30833449	30833858	410	30833527	9	4.3705	3.35698
Nc-1-IP_pe	chr10	30835684	30835986	303	30835840	9	3.72192	2.95716
Nc-1-IP_pe	chr10	30889392	30889671	280	30889609	11	4.06403	2.95415
Nc-1-IP_pe	chr10	30902711	30903353	643	30902838	14	5.51541	3.4073
Nc-1-IP_pe	chr10	30930397	30930658	262	30930517	13	7.7352	4.78626
Nc-1-IP_pe	chr10	30947752	30948092	341	30947971	11	6.03439	4.10251
Nc-1-IP_pe	chr10	31031734	31032264	531	31032199	15	4.7819	2.95039
Nc-1-IP_pe	chr10	31056791	31057084	294	31056946	9	3.72192	2.95716
Nc-1-IP_pe	chr10	31103394	31103750	357	31103699	10	4.33501	3.21677
Nc-1-IP_pe	chr10	31156122	31156420	299	31156238	7	3.06442	2.73501
Nc-1-IP_pe	chr10	31180553	31180819	267	31180600	9	4.46937	3.41876
Nc-1-IP_pe	chr10	31215881	31216159	279	31216154	8	3.08755	2.66145
Nc-1-IP_pe	chr10	31318571	31319005	435	31318616	16	3.94146	2.50592
Nc-1-IP_pe	chr10	31319331	31319721	391	31319661	12	4.24064	2.95299
Nc-1-IP_pe	chr10	31320072	31320616	545	31320490	11	5.10577	3.5486
Nc-1-IP_pe	chr10	31361514	31362130	617	31362117	12	4.39405	3.03475
Nc-1-IP_pe	chr10	31364115	31364408	294	31364267	14	6.61011	3.98382
Nc-1-IP_pe	chr10	31448751	31449116	366	31448906	14	4.21449	2.76599
Nc-1-IP_pe	chr10	31469220	31469896	677	31469608	12	6.71369	4.35145
Nc-1-IP_pe	chr10	31508462	31508908	447	31508779	14	4.60011	2.95114
Nc-1-IP_pe	chr10	31586776	31587214	439	31587183	9	3.61966	2.89507
Nc-1-IP_pe	chr10	31602606	31602928	323	31602715	15	4.7819	2.95039
Nc-1-IP_pe	chr10	31617040	31617353	314	31617160	13	6.96475	4.33508
Nc-1-IP_pe	chr10	31631674	31631931	258	31631818	12	6.86906	4.44438
Nc-1-IP_pe	chr10	31673819	31674259	441	31673850	10	3.89064	2.95552
Nc-1-IP_pe	chr10	31677750	31678100	351	31677806	7	3.06159	2.73315
Nc-1-IP_pe	chr10	31719034	31719300	267	31719091	12	5.01934	3.37518
Nc-1-IP_pe	chr10	31727594	31727989	396	31727765	11	5.10577	3.5486
Nc-1-IP_pe	chr10	31736104	31736674	571	31736602	10	3.4604	2.70797
Nc-1-IP_pe	chr10	31755238	31755625	388	31755546	15	5.66913	3.37372
Nc-1-IP_pe	chr10	31774727	31775014	288	31774966	10	3.89064	2.95552
Nc-1-IP_pe	chr10	31838552	31838830	279	31838691	9	4.46937	3.41876
Nc-1-IP_pe	chr10	31909636	31910116	481	31909749	11	6.03439	4.10251
Nc-1-IP_pe	chr10	31937918	31938224	307	31938036	11	6.03439	4.10251
Nc-1-IP_pe	chr10	31961233	31961500	268	31961359	10	4.25398	3.16873
Nc-1-IP_pe	chr10	31970161	31970451	291	31970361	10	4.46067	3.29163
Nc-1-IP_pe	chr10	31970783	31971544	762	31971430	17	7.02067	3.79543
Nc-1-IP_pe	chr10	31981270	31982034	765	31981385	12	5.84943	3.84431

Nc-1-IP_pe	chr10	31983924	31984203	280	31984013	11	5.52654	3.79706
Nc-1-IP_pe	chr10	31996251	31996520	270	31996400	13	6.62436	4.14003
Nc-1-IP_pe	chr10	32064116	32064417	302	32064328	10	5.23357	3.76063
Nc-1-IP_pe	chr10	32079652	32079965	314	32079795	9	4.46937	3.41876
Nc-1-IP_pe	chr10	32086536	32086790	255	32086636	12	4.70082	3.20033
Nc-1-IP_pe	chr10	32122861	32123154	294	32123042	9	4.0319	3.14701
Nc-1-IP_pe	chr10	32124784	32125145	362	32124949	14	7.42849	4.43574
Nc-1-IP_pe	chr10	32132364	32132680	317	32132556	14	8.29543	4.93196
Nc-1-IP_pe	chr10	32144070	32144430	361	32144217	9	4.2632	3.29017
Nc-1-IP_pe	chr10	32160871	32161208	338	32161047	9	3.83096	3.02367
Nc-1-IP_pe	chr10	32196000	32196318	319	32196140	14	8.63073	5.12814
Nc-1-IP_pe	chr10	32201172	32201588	417	32201440	7	3.06442	2.73501
Nc-1-IP_pe	chr10	32214763	32215114	352	32214978	9	2.89249	2.46179
Nc-1-IP_pe	chr10	32220402	32220699	298	32220572	13	7.42513	4.60316
Nc-1-IP_pe	chr10	32230326	32230702	377	32230630	9	4.15971	3.22596
Nc-1-IP_pe	chr10	32236093	32236642	550	32236613	8	3.74505	3.07688
Nc-1-IP_pe	chr10	32259371	32259648	278	32259519	11	6.03439	4.10251
Nc-1-IP_pe	chr10	32296616	32296879	264	32296775	11	5.10577	3.5486
Nc-1-IP_pe	chr10	32302088	32302528	441	32302521	9	3.27326	2.68684
Nc-1-IP_pe	chr10	32302957	32303291	335	32303105	13	7.7352	4.78626
Nc-1-IP_pe	chr10	32365862	32366155	294	32366019	10	5.23357	3.76063
Nc-1-IP_pe	chr10	32381359	32381788	430	32381662	13	7.7352	4.78626
Nc-1-IP_pe	chr10	32414815	32415103	289	32414994	10	4.39571	3.25288
Nc-1-IP_pe	chr10	32500604	32501071	468	32500663	12	3.16137	2.39719
Nc-1-IP_pe	chr10	32548195	32548524	330	32548361	12	5.84943	3.84431
Nc-1-IP_pe	chr10	32553850	32554190	341	32553864	11	4.06403	2.95415
Nc-1-IP_pe	chr10	32559198	32559569	372	32559365	13	4.86362	3.18015
Nc-1-IP_pe	chr10	32641933	32642347	415	32642294	10	5.23357	3.76063
Nc-1-IP_pe	chr10	32650588	32651188	601	32651026	9	4.46937	3.41876
Nc-1-IP_pe	chr10	32714921	32715386	466	32715159	14	8.63073	5.12814
Nc-1-IP_pe	chr10	32788711	32789070	360	32788875	11	4.54404	3.2242
Nc-1-IP_pe	chr10	32798395	32798984	590	32798942	9	4.46937	3.41876
Nc-1-IP_pe	chr10	32807750	32808428	679	32808298	12	6.86906	4.44438
Nc-1-IP_pe	chr10	32816382	32816746	365	32816592	12	6.58417	4.27436
Nc-1-IP_pe	chr10	32856660	32856937	278	32856674	8	3.56021	2.95918
Nc-1-IP_pe	chr10	32926455	32926766	312	32926618	11	6.03439	4.10251
Nc-1-IP_pe	chr10	32944028	32944393	366	32944194	9	4.46937	3.41876
Nc-1-IP_pe	chr10	32957457	32957952	496	32957692	13	6.36468	3.99316
Nc-1-IP_pe	chr10	32987803	32988060	258	32987989	8	3.74505	3.07688
Nc-1-IP_pe	chr10	33029383	33029696	314	33029524	10	5.23357	3.76063
Nc-1-IP_pe	chr10	33030313	33030617	305	33030526	10	4.99888	3.61677
Nc-1-IP_pe	chr10	33034650	33035025	376	33034924	8	3.4979	2.91965
Nc-1-IP_pe	chr10	33037694	33038017	324	33037921	9	2.89249	2.46179
Nc-1-IP_pe	chr10	33049251	33049644	394	33049465	12	3.83688	2.74114
Nc-1-IP_pe	chr10	33057915	33058209	295	33057993	10	4.99888	3.61677

Nc-1-IP_pe	chr10	33076559	33076958	400	33076706	11	5.65062	3.87116
Nc-1-IP_pe	chr10	33077552	33077830	279	33077710	16	7.53857	4.18505
Nc-1-IP_pe	chr10	33081506	33081767	262	33081621	12	6.65086	4.31401
Nc-1-IP_pe	chr10	33106838	33107104	267	33106973	11	5.7051	3.90381
Nc-1-IP_pe	chr10	33115975	33116442	468	33116073	13	5.36853	3.44651
Nc-1-IP_pe	chr10	33131496	33131832	337	33131799	12	3.47963	2.55766
Nc-1-IP_pe	chr10	33134236	33134728	493	33134677	11	5.16212	3.58161
Nc-1-IP_pe	chr10	33140529	33140859	331	33140720	12	6.29276	4.10227
Nc-1-IP_pe	chr10	33150588	33150887	300	33150799	12	5.15877	3.45264
Nc-1-IP_pe	chr10	33179385	33179832	448	33179386	11	2.96397	2.36091
Nc-1-IP_pe	chr10	33188481	33188746	266	33188541	11	4.06403	2.95415
Nc-1-IP_pe	chr10	33213007	33213393	387	33213196	12	5.01934	3.37518
Nc-1-IP_pe	chr10	33250906	33251328	423	33250994	12	6.71369	4.35145
Nc-1-IP_pe	chr10	33336683	33337070	388	33336766	15	6.39297	3.73508
Nc-1-IP_pe	chr10	33390149	33390403	255	33390294	11	5.7051	3.90381
Nc-1-IP_pe	chr10	33393528	33393914	387	33393689	10	4.39571	3.25288
Nc-1-IP_pe	chr10	33429012	33429332	321	33429233	10	3.83201	2.92147
Nc-1-IP_pe	chr10	33468048	33468488	441	33468403	13	5.36853	3.44651
Nc-1-IP_pe	chr10	33479190	33479683	494	33479403	12	5.3037	3.53374
Nc-1-IP_pe	chr10	33480867	33481301	435	33480900	10	3.94999	2.99009
Nc-1-IP_pe	chr10	33501888	33502192	305	33501913	11	5.03835	3.5092
Nc-1-IP_pe	chr10	33516156	33516415	260	33516176	10	3.08907	2.49869
Nc-1-IP_pe	chr10	33654105	33654407	303	33654226	13	7.7352	4.78626
Nc-1-IP_pe	chr10	33660454	33660838	385	33660701	14	8.25309	4.90735
Nc-1-IP_pe	chr10	33703004	33703275	272	33703113	11	5.03835	3.5092
Nc-1-IP_pe	chr10	33704097	33704368	272	33704195	10	5.17577	3.72509
Nc-1-IP_pe	chr10	33737293	33737556	264	33737381	10	5.23357	3.76063
Nc-1-IP_pe	chr10	33737828	33738200	373	33738118	9	2.89249	2.46179
Nc-1-IP_pe	chr10	33766941	33767200	260	33766972	9	4.05981	3.16422
Nc-1-IP_pe	chr10	33775862	33776133	272	33775879	8	3.64414	3.01254
Nc-1-IP_pe	chr10	33777746	33778195	450	33777892	9	4.25966	3.28797
Nc-1-IP_pe	chr10	33805315	33805603	289	33805540	11	4.54404	3.2242
Nc-1-IP_pe	chr10	33837557	33838106	550	33837879	10	5.23357	3.76063
Nc-1-IP_pe	chr10	33839897	33840362	466	33840259	9	4.46937	3.41876
Nc-1-IP_pe	chr10	33890614	33890943	330	33890775	11	6.03439	4.10251
Nc-1-IP_pe	chr10	33923516	33923775	260	33923586	14	8.29543	4.93196
Nc-1-IP_pe	chr10	33982041	33982454	414	33982227	10	5.23357	3.76063
Nc-1-IP_pe	chr10	34043489	34043823	335	34043565	12	6.58417	4.27436
Nc-1-IP_pe	chr10	34055745	34056193	449	34055935	12	4.24064	2.95299
Nc-1-IP_pe	chr10	34057092	34057410	319	34057177	9	3.32589	2.71826
Nc-1-IP_pe	chr10	34074793	34076227	1435	34075842	17	11.4763	6.15376
Nc-1-IP_pe	chr10	34085674	34085983	310	34085796	13	3.07238	2.29378
Nc-1-IP_pe	chr10	34094324	34094610	287	34094532	11	5.7051	3.90381
Nc-1-IP_pe	chr10	34096483	34096816	334	34096698	18	12.4731	6.49564
Nc-1-IP_pe	chr10	34119518	34119781	264	34119638	13	7.42513	4.60316

Nc-1-IP_pe	chr10	34129784	34130301	518	34129900	11	6.03439	4.10251
Nc-1-IP_pe	chr10	34132696	34133350	655	34133304	12	5.94619	3.9002
Nc-1-IP_pe	chr10	34221024	34221286	263	34221108	9	4.25966	3.28797
Nc-1-IP_pe	chr10	34252122	34252388	267	34252351	9	4.46937	3.41876
Nc-1-IP_pe	chr10	34307319	34307834	516	34307358	10	3.89064	2.95552
Nc-1-IP_pe	chr10	34308151	34308480	330	34308283	14	6.69566	4.03025
Nc-1-IP_pe	chr10	34391842	34392105	264	34391950	9	4.46937	3.41876
Nc-1-IP_pe	chr10	34538569	34538832	264	34538749	12	6.71369	4.35145
Nc-1-IP_pe	chr10	34642014	34642300	287	34642095	15	5.96953	3.52194
Nc-1-IP_pe	chr10	34664376	34664644	269	34664479	16	8.17084	4.51499
Nc-1-IP_pe	chr10	34670071	34670373	303	34670223	13	6.62436	4.14003
Nc-1-IP_pe	chr10	34694967	34695256	290	34695078	8	3.74505	3.07688
Nc-1-IP_pe	chr10	34698500	34698806	307	34698661	12	5.84943	3.84431
Nc-1-IP_pe	chr10	34718418	34718713	296	34718498	12	5.77527	3.80164
Nc-1-IP_pe	chr10	34765352	34765635	284	34765523	12	6.77747	4.38954
Nc-1-IP_pe	chr10	34809203	34809524	322	34809371	12	5.61153	3.70794
Nc-1-IP_pe	chr10	34829570	34829915	346	34829803	12	4.70082	3.20033
Nc-1-IP_pe	chr10	34831277	34831621	345	34831500	14	7.42849	4.43574
Nc-1-IP_pe	chr10	34838021	34838312	292	34838265	10	3.89064	2.95552
Nc-1-IP_pe	chr10	34842449	34842729	281	34842513	8	3.64414	3.01254
Nc-1-IP_pe	chr10	34854867	34855811	945	34855355	9	4.46937	3.41876
Nc-1-IP_pe	chr10	34906749	34907509	761	34906954	11	4.06403	2.95415
Nc-1-IP_pe	chr10	34928938	34929205	268	34929147	9	4.46937	3.41876
Nc-1-IP_pe	chr10	34938274	34938542	269	34938448	11	4.54404	3.2242
Nc-1-IP_pe	chr10	34957168	34957446	279	34957241	9	3.72192	2.95716
Nc-1-IP_pe	chr10	34961900	34962171	272	34962147	11	2.96397	2.36091
Nc-1-IP_pe	chr10	34969212	34969468	257	34969316	11	4.78913	3.36466
Nc-1-IP_pe	chr10	34976118	34976624	507	34976362	20	8.56319	4.1316
Nc-1-IP_pe	chr10	34983716	34984179	464	34983775	9	4.46937	3.41876
Nc-1-IP_pe	chr10	34986390	34986673	284	34986565	13	5.86991	3.71823
Nc-1-IP_pe	chr10	34997432	34997949	518	34997797	8	3.43415	2.87929
Nc-1-IP_pe	chr10	35065330	35065914	585	35065753	14	8.63073	5.12814
Nc-1-IP_pe	chr10	35073629	35074025	397	35073785	8	3.74505	3.07688
Nc-1-IP_pe	chr10	35090025	35090432	408	35090126	14	3.55308	2.45762
Nc-1-IP_pe	chr10	35110329	35110660	332	35110490	9	4.46937	3.41876
Nc-1-IP_pe	chr10	35112923	35113303	381	35113003	9	4.46937	3.41876
Nc-1-IP_pe	chr10	35114438	35115091	654	35114987	10	4.99888	3.61677
Nc-1-IP_pe	chr10	35127195	35127468	274	35127285	12	4.28158	2.97474
Nc-1-IP_pe	chr10	35156045	35156394	350	35156163	9	4.25966	3.28797
Nc-1-IP_pe	chr10	35198904	35199189	286	35199012	12	6.58417	4.27436
Nc-1-IP_pe	chr10	35216396	35216751	356	35216648	10	5.23357	3.76063
Nc-1-IP_pe	chr10	35237803	35238067	265	35237963	11	6.03439	4.10251
Nc-1-IP_pe	chr10	35276800	35277098	299	35277024	9	3.27326	2.68684
Nc-1-IP_pe	chr10	35335885	35336729	845	35336258	20	9.94634	4.77022
Nc-1-IP_pe	chr10	35358752	35359229	478	35358912	13	4.86362	3.18015

Nc-1-IP_pe	chr10	35406703	35407027	325	35406806	12	6.7561	4.37677
Nc-1-IP_pe	chr10	35449893	35450145	253	35450050	12	3.83688	2.74114
Nc-1-IP_pe	chr10	35451608	35451882	275	35451694	12	5.23082	3.49289
Nc-1-IP_pe	chr10	35473868	35474338	471	35474298	13	5.86991	3.71823
Nc-1-IP_pe	chr10	35531912	35532270	359	35532127	9	4.20361	3.25317
Nc-1-IP_pe	chr10	35533353	35533689	337	35533569	12	6.38914	4.15897
Nc-1-IP_pe	chr10	35549559	35549857	299	35549605	14	4.60011	2.95114
Nc-1-IP_pe	chr10	35550124	35550434	311	35550360	10	3.89064	2.95552
Nc-1-IP_pe	chr10	35564391	35564885	495	35564519	14	5.51541	3.4073
Nc-1-IP_pe	chr10	35566071	35566375	305	35566147	11	4.06403	2.95415
Nc-1-IP_pe	chr10	35568423	35568728	306	35568655	9	3.92293	3.08
Nc-1-IP_pe	chr10	35585022	35585322	301	35585152	14	5.51541	3.4073
Nc-1-IP_pe	chr10	35587570	35587830	261	35587732	10	4.95265	3.58858
Nc-1-IP_pe	chr10	35597742	35598287	546	35597763	10	4.39571	3.25288
Nc-1-IP_pe	chr10	35605514	35605865	352	35605810	11	5.3563	3.696
Nc-1-IP_pe	chr10	35608169	35608438	270	35608233	8	3.264	2.772
Nc-1-IP_pe	chr10	35613516	35613922	407	35613849	9	4.20361	3.25317
Nc-1-IP_pe	chr10	35632926	35633257	332	35633127	14	7.9745	4.74633
Nc-1-IP_pe	chr10	35639626	35640206	581	35639950	10	5.23357	3.76063
Nc-1-IP_pe	chr10	35641898	35642950	1053	35642834	16	9.01614	4.97137
Nc-1-IP_pe	chr10	35794487	35794779	293	35794677	9	4.46937	3.41876
Nc-1-IP_pe	chr10	35843717	35843977	261	35843746	9	4.46937	3.41876
Nc-1-IP_pe	chr10	35858753	35859163	411	35859084	15	4.7819	2.95039
Nc-1-IP_pe	chr10	35897289	35897622	334	35897420	13	6.62436	4.14003
Nc-1-IP_pe	chr10	35898342	35898620	279	35898523	11	5.10577	3.5486
Nc-1-IP_pe	chr10	35900874	35901248	375	35901069	15	6.78905	3.93887
Nc-1-IP_pe	chr10	36003840	36004127	288	36003919	11	6.03439	4.10251
Nc-1-IP_pe	chr10	36038401	36038678	278	36038506	12	6.86906	4.44438
Nc-1-IP_pe	chr10	36066321	36066813	493	36066342	11	5.10577	3.5486
Nc-1-IP_pe	chr10	36083697	36084258	562	36084092	10	5.23357	3.76063
Nc-1-IP_pe	chr10	36398391	36398692	302	36398551	13	7.7352	4.78626
Nc-1-IP_pe	chr10	36400248	36400551	304	36400311	10	5.23357	3.76063
Nc-1-IP_pe	chr10	36457038	36457370	333	36457167	9	4.46937	3.41876
Nc-1-IP_pe	chr10	36586678	36586994	317	36586867	12	6.71369	4.35145
Nc-1-IP_pe	chr10	36691390	36691835	446	36691589	11	6.03439	4.10251
Nc-1-IP_pe	chr10	36723446	36723784	339	36723709	7	3.06442	2.73501
Nc-1-IP_pe	chr10	36734584	36735012	429	36734828	10	4.39571	3.25288
Nc-1-IP_pe	chr10	36748325	36748646	322	36748591	9	4.46937	3.41876
Nc-1-IP_pe	chr10	36763559	36763872	314	36763656	9	4.46937	3.41876
Nc-1-IP_pe	chr10	36775190	36775442	253	36775322	11	6.03439	4.10251
Nc-1-IP_pe	chr10	36810752	36811073	322	36810937	10	4.39571	3.25288
Nc-1-IP_pe	chr10	36898254	36898664	411	36898541	9	3.66792	2.92434
Nc-1-IP_pe	chr10	37049965	37050629	665	37050209	11	6.03439	4.10251
Nc-1-IP_pe	chr10	37137554	37138056	503	37137718	13	7.34205	4.55444
Nc-1-IP_pe	chr10	37143338	37143598	261	37143441	11	5.10577	3.5486

Nc-1-IP_pe	chr10	37180085	37180355	271	37180094	12	3.47963	2.55766
Nc-1-IP_pe	chr10	37182167	37182907	741	37182276	15	6.78905	3.93887
Nc-1-IP_pe	chr10	37275258	37275707	450	37275535	15	9.55381	5.47001
Nc-1-IP_pe	chr10	37344804	37345296	493	37345248	8	3.74505	3.07688
Nc-1-IP_pe	chr10	37383858	37384168	311	37383953	9	3.72192	2.95716
Nc-1-IP_pe	chr10	37488494	37488851	358	37488696	9	3.27326	2.68684
Nc-1-IP_pe	chr10	37495786	37496159	374	37495869	8	3.74505	3.07688
Nc-1-IP_pe	chr10	37513969	37514240	272	37514078	10	5.23357	3.76063
Nc-1-IP_pe	chr10	37570647	37570935	289	37570706	10	5.23357	3.76063
Nc-1-IP_pe	chr10	37634683	37635294	612	37634706	11	3.64839	2.72584
Nc-1-IP_pe	chr10	37638837	37639658	822	37639018	14	7.42849	4.43574
Nc-1-IP_pe	chr10	37640192	37640451	260	37640267	12	6.86906	4.44438
Nc-1-IP_pe	chr10	37651423	37651956	534	37651514	13	7.7352	4.78626
Nc-1-IP_pe	chr10	37678229	37678525	297	37678414	14	8.63073	5.12814
Nc-1-IP_pe	chr10	37679315	37679683	369	37679603	9	3.27326	2.68684
Nc-1-IP_pe	chr10	37680485	37681347	863	37680638	12	5.77527	3.80164
Nc-1-IP_pe	chr10	37685810	37686203	394	37686065	10	5.23357	3.76063
Nc-1-IP_pe	chr10	37699097	37699379	283	37699271	15	9.55381	5.47001
Nc-1-IP_pe	chr10	37701626	37702252	627	37701779	16	10.5028	5.81189
Nc-1-IP_pe	chr10	37711335	37711624	290	37711498	10	5.23357	3.76063
Nc-1-IP_pe	chr10	37712335	37712596	262	37712543	9	3.32589	2.71826
Nc-1-IP_pe	chr10	37723047	37723331	285	37723182	12	6.86906	4.44438
Nc-1-IP_pe	chr10	37728683	37729265	583	37729147	12	5.23082	3.49289
Nc-1-IP_pe	chr10	37735104	37735706	603	37735427	10	4.39571	3.25288
Nc-1-IP_pe	chr10	37739054	37739475	422	37739236	13	7.7352	4.78626
Nc-1-IP_pe	chr10	37765952	37766280	329	37766225	11	4.54404	3.2242
Nc-1-IP_pe	chr10	37780565	37780888	324	37780841	8	3.74505	3.07688
Nc-1-IP_pe	chr10	37818096	37818447	352	37818297	9	4.05981	3.16422
Nc-1-IP_pe	chr10	37857787	37858194	408	37857815	15	4.40338	2.77622
Nc-1-IP_pe	chr10	37859055	37859534	480	37859476	12	5.3037	3.53374
Nc-1-IP_pe	chr10	37874618	37874876	259	37874762	10	5.23357	3.76063
Nc-1-IP_pe	chr10	37881534	37881937	404	37881775	11	6.03439	4.10251
Nc-1-IP_pe	chr10	37897123	37897424	302	37897279	12	6.86906	4.44438
Nc-1-IP_pe	chr10	37917609	37917873	265	37917738	8	3.74505	3.07688
Nc-1-IP_pe	chr10	37945250	37945831	582	37945674	10	5.23357	3.76063
Nc-1-IP_pe	chr10	37963667	37964075	409	37963972	7	3.06442	2.73501
Nc-1-IP_pe	chr10	37976234	37976636	403	37976384	16	9.11744	5.02718
Nc-1-IP_pe	chr10	37993362	37993633	272	37993465	11	5.97044	4.06374
Nc-1-IP_pe	chr10	38012455	38012710	256	38012606	12	6.86906	4.44438
Nc-1-IP_pe	chr10	38045854	38046127	274	38045973	10	5.23357	3.76063
Nc-1-IP_pe	chr10	38061756	38062142	387	38062099	10	4.07292	3.06202
Nc-1-IP_pe	chr10	38094519	38095591	1073	38095387	16	6.33285	3.58457
Nc-1-IP_pe	chr10	38156821	38157439	619	38157362	10	4.39571	3.25288
Nc-1-IP_pe	chr10	38292519	38292868	350	38292744	11	3.64839	2.72584
Nc-1-IP_pe	chr10	38298177	38298458	282	38298355	11	4.11366	2.98176

Nc-1-IP_pe	chr10	38313911	38314237	327	38314027	11	6.03439	4.10251
Nc-1-IP_pe	chr10	38356000	38356626	627	38356255	20	11.7013	5.64235
Nc-1-IP_pe	chr10	38402068	38402886	819	38402180	17	8.20861	4.37862
Nc-1-IP_pe	chr10	38408547	38409030	484	38408985	13	5.36853	3.44651
Nc-1-IP_pe	chr10	38410185	38410533	349	38410301	16	5.73763	3.30235
Nc-1-IP_pe	chr10	38410798	38411323	526	38411107	14	4.52309	2.91384
Nc-1-IP_pe	chr10	38430237	38430655	419	38430561	13	5.29187	3.40559
Nc-1-IP_pe	chr10	38436367	38436640	274	38436530	13	6.36468	3.99316
Nc-1-IP_pe	chr10	38443844	38444120	277	38444002	10	3.82047	2.91478
Nc-1-IP_pe	chr10	38449010	38449460	451	38449274	25	9.19615	3.83259
Nc-1-IP_pe	chr10	38450431	38450802	372	38450559	102	38.4502	4.88762
Nc-1-IP_pe	chr10	38453033	38453312	280	38453042	11	2.94464	2.3508
Nc-1-IP_pe	chr10	38455682	38456121	440	38455897	12	5.59553	3.69882
Nc-1-IP_pe	chr10	38604031	38604412	382	38604260	11	5.3563	3.696
Nc-1-IP_pe	chr10	38610928	38611221	294	38611099	17	9.09431	4.8363
Nc-1-IP_pe	chr10	38635592	38635998	407	38635915	14	7.42849	4.43574
Nc-1-IP_pe	chr10	38703038	38703592	555	38703241	12	5.23082	3.49289
Nc-1-IP_pe	chr10	38713650	38713959	310	38713819	13	7.7352	4.78626
Nc-1-IP_pe	chr10	38736113	38736556	444	38736266	12	5.23082	3.49289
Nc-1-IP_pe	chr10	38738215	38738829	615	38738665	15	6.5434	3.81197
Nc-1-IP_pe	chr10	38739858	38740326	469	38739942	12	3.47963	2.55766
Nc-1-IP_pe	chr10	38742086	38742482	397	38742390	19	6.78661	3.47027
Nc-1-IP_pe	chr10	38942680	38943516	837	38942929	28	10.6836	4.07065
Nc-1-IP_pe	chr10	38950852	38951189	338	38951010	14	5.51541	3.4073
Nc-1-IP_pe	chr10	38969586	38969935	350	38969789	14	5.69896	3.50164
Nc-1-IP_pe	chr10	38970210	38970518	309	38970308	14	3.55308	2.45762
Nc-1-IP_pe	chr10	38979811	38980152	342	38980076	16	6.8987	3.86161
Nc-1-IP_pe	chr10	39005084	39005507	424	39005153	15	5.66913	3.37372
Nc-1-IP_pe	chr10	39372722	39373082	361	39372832	15	4.50738	2.8237
Nc-1-IP_pe	chr10	39475435	39475693	259	39475567	11	5.03835	3.5092
Nc-1-IP_pe	chr10	41749642	41750140	499	41749991	13	4.02562	2.7544
Nc-1-IP_pe	chr10	42111082	42112184	1103	42111911	15	9.19325	5.26076
Nc-1-IP_pe	chr10	42112752	42113284	533	42112895	11	4.54404	3.2242
Nc-1-IP_pe	chr10	42131113	42131368	256	42131251	16	8.26996	4.56762
Nc-1-IP_pe	chr10	42148983	42149737	755	42149144	33	7.471	2.78059
Nc-1-IP_pe	chr10	42203025	42203410	386	42203176	8	3.74505	3.07688
Nc-1-IP_pe	chr10	42229931	42231222	1292	42231122	24	5.47323	2.62998
Nc-1-IP_pe	chr10	42232203	42232993	791	42232424	21	8.6741	4.05679
Nc-1-IP_pe	chr10	42233356	42233628	273	42233585	17	3.84884	2.41145
Nc-1-IP_pe	chr10	42236339	42237100	762	42236989	18	7.1522	3.73811
Nc-1-IP_pe	chr10	42241965	42242791	827	42242253	15	5.70822	3.39286
Nc-1-IP_pe	chr10	42248512	42248765	254	42248664	12	6.86906	4.44438
Nc-1-IP_pe	chr10	42263243	42263545	303	42263513	14	5.51541	3.4073
Nc-1-IP_pe	chr10	42281887	42282180	294	42282101	22	9.39426	4.24119
Nc-1-IP_pe	chr10	42293007	42293571	565	42293434	16	10.3789	5.74017

Nc-1-IP_pe	chr10	42349340	42349618	279	42349462	11	6.03439	4.10251
Nc-1-IP_pe	chr10	42367556	42368216	661	42367761	13	7.42513	4.60316
Nc-1-IP_pe	chr10	42377227	42377559	333	42377323	9	2.89249	2.46179
Nc-1-IP_pe	chr10	42380504	42380805	302	42380684	12	6.86906	4.44438
Nc-1-IP_pe	chr10	42397961	42398217	257	42398083	11	6.03439	4.10251
Nc-1-IP_pe	chr10	42431535	42431861	327	42431602	11	6.03439	4.10251
Nc-1-IP_pe	chr10	42435564	42435828	265	42435599	12	4.06753	2.86157
Nc-1-IP_pe	chr10	42465262	42465575	314	42465400	11	6.03439	4.10251
Nc-1-IP_pe	chr10	42466252	42466622	371	42466276	10	3.4604	2.70797
Nc-1-IP_pe	chr10	42482032	42482339	308	42482216	14	8.44792	5.0209
Nc-1-IP_pe	chr10	42531536	42531809	274	42531677	13	4.02562	2.7544
Nc-1-IP_pe	chr10	42551654	42551961	308	42551751	16	6.37501	3.60492
Nc-1-IP_pe	chr10	42570532	42570834	303	42570585	9	3.66792	2.92434
Nc-1-IP_pe	chr10	42587369	42588115	747	42587471	13	6.72991	4.20021
Nc-1-IP_pe	chr10	42602863	42603185	323	42603062	11	5.89269	4.01672
Nc-1-IP_pe	chr10	42604553	42604828	276	42604688	11	6.03439	4.10251
Nc-1-IP_pe	chr10	42639695	42640225	531	42640151	12	6.14317	4.01469
Nc-1-IP_pe	chr10	42690288	42690608	321	42690558	16	4.25252	2.63824
Nc-1-IP_pe	chr10	42691049	42691713	665	42691403	16	7.53857	4.18505
Nc-1-IP_pe	chr10	42722000	42722300	301	42722265	10	4.68196	3.42446
Nc-1-IP_pe	chr10	42746238	42746498	261	42746374	12	3.47963	2.55766
Nc-1-IP_pe	chr10	42754826	42755248	423	42755036	12	4.63088	3.16234
Nc-1-IP_pe	chr10	42758271	42758581	311	42758403	16	7.61454	4.22416
Nc-1-IP_pe	chr10	42768707	42769014	308	42768740	11	4.59676	3.25427
Nc-1-IP_pe	chr10	42771317	42771679	363	42771603	11	2.96397	2.36091
Nc-1-IP_pe	chr10	42782302	42783035	734	42782795	31	12.8059	4.49176
Nc-1-IP_pe	chr10	42812288	42812923	636	42812737	17	7.02067	3.79543
Nc-1-IP_pe	chr10	42816370	42816622	253	42816554	12	4.70082	3.20033
Nc-1-IP_pe	chr10	42836961	42837376	416	42837114	15	6.78905	3.93887
Nc-1-IP_pe	chr10	42847218	42847836	619	42847328	15	5.96953	3.52194
Nc-1-IP_pe	chr10	42858776	42859165	390	42858925	21	7.58657	3.60451
Nc-1-IP_pe	chr10	42866210	42866731	522	42866620	20	4.99691	2.68313
Nc-1-IP_pe	chr10	42867605	42868128	524	42867633	12	3.83688	2.74114
Nc-1-IP_pe	chr10	42876596	42877111	516	42877069	11	4.1834	3.02067
Nc-1-IP_pe	chr10	42877525	42877854	330	42877688	13	5.42865	3.47872
Nc-1-IP_pe	chr10	42898666	42899165	500	42898739	12	4.75569	3.23024
Nc-1-IP_pe	chr10	42900194	42900484	291	42900329	21	4.90204	2.59278
Nc-1-IP_pe	chr10	42903133	42903490	358	42903227	14	5.03085	3.16286
Nc-1-IP_pe	chr10	42927660	42928601	942	42928387	20	11.575	5.57735
Nc-1-IP_pe	chr10	42930267	42931086	820	42930501	14	6.61011	3.98382
Nc-1-IP_pe	chr10	42931951	42932648	698	42932440	14	6.28469	3.80898
Nc-1-IP_pe	chr10	42933283	42934778	1496	42933972	14	5.03085	3.16286
Nc-1-IP_pe	chr10	42935976	42936459	484	42936320	12	3.83688	2.74114
Nc-1-IP_pe	chr10	42949754	42950074	321	42949785	7	3.0493	2.72511
Nc-1-IP_pe	chr10	42951039	42952218	1180	42951512	21	8.6741	4.05679

Nc-1-IP_pe	chr10	42952838	42953274	437	42953025	13	6.09351	3.84167
Nc-1-IP_pe	chr10	42954893	42955502	610	42955417	12	3.16137	2.39719
Nc-1-IP_pe	chr10	42961992	42962997	1006	42962785	20	6.94658	3.44067
Nc-1-IP_pe	chr10	42969307	42971217	1911	42970908	14	4.95172	3.12357
Nc-1-IP_pe	chr10	42979988	42980425	438	42980268	13	5.10817	3.30823
Nc-1-IP_pe	chr10	42984671	42985254	584	42985066	15	6.49775	3.78858
Nc-1-IP_pe	chr10	42985764	42986078	315	42985969	11	3.8706	2.84726
Nc-1-IP_pe	chr10	42988579	42988957	379	42988731	12	3.83688	2.74114
Nc-1-IP_pe	chr10	42989734	42990559	826	42989942	20	8.56319	4.1316
Nc-1-IP_pe	chr10	42990831	42991117	287	42990900	12	4.24064	2.95299
Nc-1-IP_pe	chr10	42991857	42992254	398	42992079	11	2.96397	2.36091
Nc-1-IP_pe	chr10	42996320	42996801	482	42996495	12	4.91154	3.31567
Nc-1-IP_pe	chr10	42999044	42999634	591	42999113	13	4.41954	2.952
Nc-1-IP_pe	chr10	43010534	43010819	286	43010573	10	3.08907	2.49869
Nc-1-IP_pe	chr10	43020317	43020619	303	43020448	10	4.39571	3.25288
Nc-1-IP_pe	chr10	43024838	43025225	388	43024912	15	4.40338	2.77622
Nc-1-IP_pe	chr10	43026015	43026935	921	43026228	14	5.51541	3.4073
Nc-1-IP_pe	chr10	43028184	43028749	566	43028625	13	6.72991	4.20021
Nc-1-IP_pe	chr10	43036614	43037483	870	43037323	12	4.63088	3.16234
Nc-1-IP_pe	chr10	43038461	43038834	374	43038520	13	4.90859	3.20357
Nc-1-IP_pe	chr10	43041654	43041926	273	43041677	11	3.28475	2.53029
Nc-1-IP_pe	chr10	43042719	43043189	471	43042812	13	4.80086	3.14755
Nc-1-IP_pe	chr10	43049156	43049982	827	43049269	14	4.21449	2.76599
Nc-1-IP_pe	chr10	43056329	43056640	312	43056539	12	5.3037	3.53374
Nc-1-IP_pe	chr10	43058115	43058613	499	43058197	15	7.12899	4.11712
Nc-1-IP_pe	chr10	43058892	43059462	571	43059145	15	6.78905	3.93887
Nc-1-IP_pe	chr10	43064314	43064632	319	43064578	14	4.60011	2.95114
Nc-1-IP_pe	chr10	43065917	43066449	533	43066091	17	7.02067	3.79543
Nc-1-IP_pe	chr10	43068861	43069287	427	43069090	16	7.95994	4.40382
Nc-1-IP_pe	chr10	43070301	43071126	826	43070862	15	6.97567	4.03634
Nc-1-IP_pe	chr10	43073179	43073446	268	43073401	12	4.79401	3.25118
Nc-1-IP_pe	chr10	43077198	43077834	637	43077719	15	5.92932	3.50195
Nc-1-IP_pe	chr10	43078921	43079243	323	43079118	14	3.55308	2.45762
Nc-1-IP_pe	chr10	43082038	43082437	400	43082099	11	3.28475	2.53029
Nc-1-IP_pe	chr10	43082760	43083626	867	43083127	16	6.61333	3.72083
Nc-1-IP_pe	chr10	43086991	43087267	277	43087225	15	5.66913	3.37372
Nc-1-IP_pe	chr10	43089791	43090515	725	43090398	12	3.16137	2.39719
Nc-1-IP_pe	chr10	43102447	43102720	274	43102616	15	5.66913	3.37372
Nc-1-IP_pe	chr10	43104830	43106928	2099	43104993	19	6.78661	3.47027
Nc-1-IP_pe	chr10	43109973	43110229	257	43110057	14	5.10212	3.19839
Nc-1-IP_pe	chr10	43112227	43112646	420	43112351	15	5.34394	3.21607
Nc-1-IP_pe	chr10	43113242	43113767	526	43113243	13	3.07238	2.29378
Nc-1-IP_pe	chr10	43114668	43115572	905	43115314	19	6.78661	3.47027
Nc-1-IP_pe	chr10	43115941	43116227	287	43116061	15	5.78498	3.43058
Nc-1-IP_pe	chr10	43117919	43119065	1147	43118375	16	5.37454	3.13479

Nc-1-IP_pe	chr10	43119928	43120815	888	43120398	12	3.16137	2.39719
Nc-1-IP_pe	chr10	43122602	43123047	446	43122706	18	8.58688	4.41758
Nc-1-IP_pe	chr10	43146208	43146584	377	43146298	11	4.06403	2.95415
Nc-1-IP_pe	chr10	43176588	43176867	280	43176757	18	7.1522	3.73811
Nc-1-IP_pe	chr10	43192647	43193267	621	43192925	12	4.24064	2.95299
Nc-1-IP_pe	chr10	43196303	43196589	287	43196565	15	3.46204	2.35851
Nc-1-IP_pe	chr10	43197296	43197600	305	43197440	15	5.88948	3.4822
Nc-1-IP_pe	chr10	43198160	43198597	438	43198314	16	6.24025	3.54005
Nc-1-IP_pe	chr10	43199602	43199876	275	43199797	17	4.78101	2.79343
Nc-1-IP_pe	chr10	43200342	43200772	431	43200459	14	4.21449	2.76599
Nc-1-IP_pe	chr10	43201919	43202602	684	43202004	19	7.32961	3.70477
Nc-1-IP_pe	chr10	43204473	43204858	386	43204583	17	5.14803	2.94914
Nc-1-IP_pe	chr10	43205500	43206162	663	43205766	18	6.40661	3.40507
Nc-1-IP_pe	chr10	43207090	43207381	292	43207334	16	5.35101	3.12405
Nc-1-IP_pe	chr10	43208603	43208932	330	43208768	18	7.13091	3.72841
Nc-1-IP_pe	chr10	43214486	43215022	537	43214523	17	6.99309	3.78232
Nc-1-IP_pe	chr10	43215949	43216379	431	43216094	16	6.1093	3.47747
Nc-1-IP_pe	chr10	43221631	43221956	326	43221776	15	6.19428	3.63445
Nc-1-IP_pe	chr10	43227884	43229751	1868	43229266	22	9.63527	4.34392
Nc-1-IP_pe	chr10	43233657	43234173	517	43234060	18	6.15736	3.29676
Nc-1-IP_pe	chr10	43234557	43234899	343	43234730	13	3.77558	2.63125
Nc-1-IP_pe	chr10	43236713	43237975	1263	43237117	14	5.06689	3.18081
Nc-1-IP_pe	chr10	43243255	43243780	526	43243422	15	7.46994	4.29894
Nc-1-IP_pe	chr10	43250627	43250915	289	43250866	13	4.02562	2.7544
Nc-1-IP_pe	chr10	43251987	43252531	545	43252523	13	4.02562	2.7544
Nc-1-IP_pe	chr10	43253002	43253302	301	43253200	13	4.15529	2.81895
Nc-1-IP_pe	chr10	43255106	43255886	781	43255263	18	6.65364	3.51391
Nc-1-IP_pe	chr10	43256732	43257254	523	43256812	18	5.62894	3.07204
Nc-1-IP_pe	chr10	43258309	43258656	348	43258552	17	6.59042	3.59311
Nc-1-IP_pe	chr10	43258994	43259490	497	43259114	18	5.33197	2.94862
Nc-1-IP_pe	chr10	43262974	43263550	577	43263404	14	4.04994	2.68821
Nc-1-IP_pe	chr10	43265486	43265962	477	43265892	15	5.00915	3.05678
Nc-1-IP_pe	chr10	43267581	43268219	639	43268032	19	9.78988	4.85493
Nc-1-IP_pe	chr10	43274100	43274371	272	43274190	12	4.63088	3.16234
Nc-1-IP_pe	chr10	43274810	43275177	368	43274929	11	3.64839	2.72584
Nc-1-IP_pe	chr10	43283202	43284819	1618	43284672	18	7.1522	3.73811
Nc-1-IP_pe	chr10	43285685	43285991	307	43285908	16	5.37454	3.13479
Nc-1-IP_pe	chr10	43286417	43286846	430	43286597	17	3.84884	2.41145
Nc-1-IP_pe	chr10	43297533	43297862	330	43297714	18	9.1091	4.6774
Nc-1-IP_pe	chr10	43298416	43298758	343	43298680	14	4.60011	2.95114
Nc-1-IP_pe	chr10	43306898	43308224	1327	43307661	13	4.41954	2.952
Nc-1-IP_pe	chr10	43311988	43312534	547	43312121	17	7.02067	3.79543
Nc-1-IP_pe	chr10	43313847	43314520	674	43313944	14	5.71266	3.50871
Nc-1-IP_pe	chr10	43318961	43319693	733	43319062	15	5.96953	3.52194
Nc-1-IP_pe	chr10	43320021	43320313	293	43320109	12	3.16137	2.39719

Nc-1-IP_pe	chr10	43322985	43323472	488	43322994	12	4.28158	2.97474
Nc-1-IP_pe	chr10	43325834	43326232	399	43326083	16	5.82808	3.34463
Nc-1-IP_pe	chr10	43334149	43334788	640	43334659	16	6.74448	3.78526
Nc-1-IP_pe	chr10	43342627	43343033	407	43342786	18	5.62894	3.07204
Nc-1-IP_pe	chr10	43344947	43345208	262	43345038	17	7.08058	3.82398
Nc-1-IP_pe	chr10	43350596	43351156	561	43350762	21	8.55202	4.00473
Nc-1-IP_pe	chr10	43362710	43363054	345	43362834	24	8.05897	3.50918
Nc-1-IP_pe	chr10	43375896	43376236	341	43376114	19	6.32622	3.27682
Nc-1-IP_pe	chr10	43376563	43377205	643	43377092	17	7.06589	3.81697
Nc-1-IP_pe	chr10	43377876	43378226	351	43378046	15	3.74733	2.48291
Nc-1-IP_pe	chr10	43384347	43384632	286	43384499	16	5.07731	3.00018
Nc-1-IP_pe	chr10	43387344	43387932	589	43387596	20	7.43645	3.64378
Nc-1-IP_pe	chr10	43396494	43396800	307	43396654	17	5.56266	3.12894
Nc-1-IP_pe	chr10	43400756	43401009	254	43400934	13	5.38711	3.45645
Nc-1-IP_pe	chr10	43401689	43401968	280	43401830	18	9.61048	4.93296
Nc-1-IP_pe	chr10	43407145	43407526	382	43407524	14	3.00733	2.21111
Nc-1-IP_pe	chr10	43409442	43409788	347	43409510	21	8.40557	3.94271
Nc-1-IP_pe	chr10	43415989	43416402	414	43416293	13	3.07238	2.29378
Nc-1-IP_pe	chr10	43430086	43430566	481	43430169	11	4.06403	2.95415
Nc-1-IP_pe	chr10	43432446	43433144	699	43432976	12	4.24064	2.95299
Nc-1-IP_pe	chr10	43441302	43441890	589	43441723	17	7.50977	4.03119
Nc-1-IP_pe	chr10	43446228	43446926	699	43446488	18	5.73252	3.11557
Nc-1-IP_pe	chr10	43465212	43465492	281	43465304	27	9.006	3.58762
Nc-1-IP_pe	chr10	43473729	43474109	381	43473749	10	3.89064	2.95552
Nc-1-IP_pe	chr10	43478257	43478563	307	43478415	17	9.69915	5.1595
Nc-1-IP_pe	chr10	43481024	43481279	256	43481077	12	3.47963	2.55766
Nc-1-IP_pe	chr10	43499866	43500307	442	43499973	9	3.27326	2.68684
Nc-1-IP_pe	chr10	43506858	43507455	598	43506988	11	5.97044	4.06374
Nc-1-IP_pe	chr10	43532974	43533639	666	43533286	12	6.71369	4.35145
Nc-1-IP_pe	chr10	43543795	43544060	266	43543943	10	5.00283	3.61918
Nc-1-IP_pe	chr10	43546979	43547398	420	43547107	8	3.58997	2.97809
Nc-1-IP_pe	chr10	43556879	43557248	370	43556882	12	3.16137	2.39719
Nc-1-IP_pe	chr10	43573876	43574569	694	43574435	28	9.16687	3.56053
Nc-1-IP_pe	chr10	43581257	43581526	270	43581440	14	6.06519	3.69269
Nc-1-IP_pe	chr10	43581825	43582267	443	43581937	10	3.4604	2.70797
Nc-1-IP_pe	chr10	43595779	43596205	427	43595913	16	6.33285	3.58457
Nc-1-IP_pe	chr10	43606091	43606960	870	43606472	27	8.12936	3.29991
Nc-1-IP_pe	chr10	43648311	43648869	559	43648652	27	10.2626	4.02153
Nc-1-IP_pe	chr10	43650065	43650494	430	43650194	18	4.96968	2.80074
Nc-1-IP_pe	chr10	43668391	43668885	495	43668561	19	6.32622	3.27682
Nc-1-IP_pe	chr10	43670119	43670385	267	43670244	19	7.2357	3.66372
Nc-1-IP_pe	chr10	43674857	43675433	577	43674954	16	5.37454	3.13479
Nc-1-IP_pe	chr10	43678595	43679044	450	43678871	21	8.10596	3.8173
Nc-1-IP_pe	chr10	43688348	43689249	902	43688555	18	6.15736	3.29676
Nc-1-IP_pe	chr10	43689534	43690694	1161	43689799	22	8.24538	3.76835

Nc-1-IP_pe	chr10	43692259	43692582	324	43692368	21	7.58657	3.60451
Nc-1-IP_pe	chr10	43694467	43694773	307	43694600	14	4.53323	2.91874
Nc-1-IP_pe	chr10	43695397	43696271	875	43696154	18	5.33197	2.94862
Nc-1-IP_pe	chr10	43697292	43697687	396	43697472	17	6.47874	3.54137
Nc-1-IP_pe	chr10	43699010	43699267	258	43699143	13	4.34711	2.91533
Nc-1-IP_pe	chr10	43700056	43700361	306	43700236	13	4.02562	2.7544
Nc-1-IP_pe	chr10	43700938	43701519	582	43701447	22	5.39765	2.71064
Nc-1-IP_pe	chr10	43702347	43703653	1307	43702430	17	7.1696	3.86657
Nc-1-IP_pe	chr10	43709141	43709432	292	43709360	10	3.70758	2.84953
Nc-1-IP_pe	chr10	43710714	43710980	267	43710853	13	5.74741	3.65119
Nc-1-IP_pe	chr10	43711657	43712526	870	43712365	18	8.37884	4.31591
Nc-1-IP_pe	chr10	43713170	43713471	302	43713343	12	3.83688	2.74114
Nc-1-IP_pe	chr10	43728417	43728782	366	43728654	20	6.08336	3.09555
Nc-1-IP_pe	chr10	43730157	43730467	311	43730334	16	7.72115	4.27929
Nc-1-IP_pe	chr10	43759241	43759494	254	43759376	18	9.23591	4.74148
Nc-1-IP_pe	chr10	43780036	43780319	284	43780154	15	5.1177	3.10809
Nc-1-IP_pe	chr10	43781621	43782616	996	43781761	17	7.70506	4.12703
Nc-1-IP_pe	chr10	43782888	43783708	821	43783116	19	10.1703	5.04543
Nc-1-IP_pe	chr10	43799378	43799722	345	43799515	17	5.14803	2.94914
Nc-1-IP_pe	chr10	43807459	43808079	621	43807552	16	7.57949	4.20609
Nc-1-IP_pe	chr10	43819265	43819986	722	43819426	14	4.23163	2.77414
Nc-1-IP_pe	chr10	43825535	43826178	644	43826030	21	4.72684	2.53141
Nc-1-IP_pe	chr10	43836388	43836970	583	43836871	16	7.72115	4.27929
Nc-1-IP_pe	chr10	43838383	43838864	482	43838644	19	7.84735	3.93486
Nc-1-IP_pe	chr10	43841214	43842301	1088	43842084	18	5.1438	2.87144
Nc-1-IP_pe	chr10	43846410	43846807	398	43846574	20	8.56319	4.1316
Nc-1-IP_pe	chr10	43852742	43853145	404	43853002	20	5.70098	2.94771
Nc-1-IP_pe	chr10	43854750	43855045	296	43854886	23	7.01947	3.21526
Nc-1-IP_pe	chr10	43863181	43863623	443	43863381	20	4.71088	2.57831
Nc-1-IP_pe	chr10	43865569	43866072	504	43865809	30	10.9504	3.972
Nc-1-IP_pe	chr10	43866579	43866953	375	43866684	14	3.00312	2.20923
Nc-1-IP_pe	chr10	43869127	43870330	1204	43869795	24	9.77496	4.15836
Nc-1-IP_pe	chr10	43871568	43871857	290	43871633	21	8.6741	4.05679
Nc-1-IP_pe	chr10	43872763	43873235	473	43872885	14	3.86727	2.6027
Nc-1-IP_pe	chr10	43879804	43880370	567	43879991	14	4.32616	2.81919
Nc-1-IP_pe	chr10	43881445	43881844	400	43881635	20	6.49719	3.259
Nc-1-IP_pe	chr10	43884760	43885553	794	43885376	16	5.62031	3.24783
Nc-1-IP_pe	chr10	43892256	43892682	427	43892588	14	3.86727	2.6027
Nc-1-IP_pe	chr10	43899256	43899611	356	43899330	17	3.33947	2.20998
Nc-1-IP_pe	chr10	43904094	43905016	923	43904310	14	4.21449	2.76599
Nc-1-IP_pe	chr10	43906571	43906888	318	43906811	16	6.65665	3.74206
Nc-1-IP_pe	chr10	43910106	43910661	556	43910654	12	3.16137	2.39719
Nc-1-IP_pe	chr10	43941038	43941445	408	43941064	10	3.06046	2.48272
Nc-1-IP_pe	chr10	43944845	43945112	268	43945018	12	4.24064	2.95299
Nc-1-IP_pe	chr10	43954721	43955393	673	43955306	14	3.55308	2.45762

Nc-1-IP_pe	chr10	43956143	43956479	337	43956397	13	5.41476	3.47126
Nc-1-IP_pe	chr10	43958888	43959154	267	43958963	14	5.35794	3.32713
Nc-1-IP_pe	chr10	43962392	43962716	325	43962552	21	7.10961	3.41419
Nc-1-IP_pe	chr10	43969714	43970244	531	43969827	18	7.82684	4.0513
Nc-1-IP_pe	chr10	43973559	43973863	305	43973714	14	4.42328	2.86573
Nc-1-IP_pe	chr10	43980368	43980903	536	43980679	17	6.25544	3.43888
Nc-1-IP_pe	chr10	43984491	43985545	1055	43984917	17	4.44438	2.65333
Nc-1-IP_pe	chr10	44000010	44000311	302	44000048	16	3.65575	2.38624
Nc-1-IP_pe	chr10	44005066	44005347	282	44005173	16	5.39474	3.14402
Nc-1-IP_pe	chr10	44005705	44006309	605	44005951	14	4.32616	2.81919
Nc-1-IP_pe	chr10	44014793	44015146	354	44015104	15	3.20025	2.24588
Nc-1-IP_pe	chr10	44015822	44016335	514	44016113	15	5.20142	3.14789
Nc-1-IP_pe	chr10	44044959	44045243	285	44045092	13	3.95529	2.71958
Nc-1-IP_pe	chr10	44046518	44046932	415	44046661	19	8.98281	4.46183
Nc-1-IP_pe	chr10	44057078	44057723	646	44057513	23	11.3323	4.94433
Nc-1-IP_pe	chr10	44061007	44061458	452	44061360	17	5.54967	3.12324
Nc-1-IP_pe	chr10	44061887	44062419	533	44062254	15	5.20142	3.14789
Nc-1-IP_pe	chr10	44063569	44063960	392	44063668	16	4.59224	2.7853
Nc-1-IP_pe	chr10	44065257	44065997	741	44065679	17	5.9911	3.31919
Nc-1-IP_pe	chr10	44068665	44069155	491	44068748	12	4.61864	3.1557
Nc-1-IP_pe	chr10	44070588	44070937	350	44070811	21	9.3401	4.34659
Nc-1-IP_pe	chr10	44091247	44091774	528	44091309	20	6.94658	3.44067
Nc-1-IP_pe	chr10	44112372	44112710	339	44112600	14	6.19157	3.75949
Nc-1-IP_pe	chr10	44123249	44123571	323	44123367	15	6.19428	3.63445
Nc-1-IP_pe	chr10	44125590	44126044	455	44125753	17	9.69915	5.1595
Nc-1-IP_pe	chr10	44132420	44132685	266	44132546	18	9.94172	5.10499
Nc-1-IP_pe	chr10	44137456	44137909	454	44137770	17	5.9911	3.31919
Nc-1-IP_pe	chr10	44171732	44172066	335	44171914	19	9.43107	4.67828
Nc-1-IP_pe	chr10	44172723	44173012	290	44172834	11	3.69531	2.75136
Nc-1-IP_pe	chr10	44177582	44177909	328	44177861	15	6.06481	3.56947
Nc-1-IP_pe	chr10	44184785	44185250	466	44185186	15	5.58327	3.33181
Nc-1-IP_pe	chr10	44197724	44198022	299	44197922	12	3.81861	2.73167
Nc-1-IP_pe	chr10	44206417	44206919	503	44206570	20	9.45052	4.53626
Nc-1-IP_pe	chr10	44207264	44208104	841	44207930	20	9.94634	4.77022
Nc-1-IP_pe	chr10	44233421	44234032	612	44233789	17	6.64406	3.61808
Nc-1-IP_pe	chr10	44258059	44258376	318	44258199	20	10.887	5.2293
Nc-1-IP_pe	chr10	44259197	44259597	401	44259370	17	8.13632	4.34211
Nc-1-IP_pe	chr10	44260960	44262000	1041	44261324	15	5.66913	3.37372
Nc-1-IP_pe	chr10	44267104	44267414	311	44267239	18	7.32895	3.81908
Nc-1-IP_pe	chr10	44268510	44269088	579	44268671	18	3.77591	2.33276
Nc-1-IP_pe	chr10	44281332	44282094	763	44281767	19	8.03556	4.02008
Nc-1-IP_pe	chr10	44284354	44284723	370	44284456	13	5.36853	3.44651
Nc-1-IP_pe	chr10	44292380	44292775	396	44292500	16	8.22985	4.5463
Nc-1-IP_pe	chr10	44299961	44300235	275	44300120	16	8.45195	4.66488
Nc-1-IP_pe	chr10	44307947	44308452	506	44308412	13	4.02562	2.7544

Nc-1-IP_pe	chr10	44309571	44310327	757	44310175	19	5.90435	3.10381
Nc-1-IP_pe	chr10	44310805	44311301	497	44311198	21	3.85951	2.23441
Nc-1-IP_pe	chr10	44316561	44317114	554	44316956	22	7.27519	3.39036
Nc-1-IP_pe	chr10	44333544	44333836	293	44333736	15	6.69889	3.8921
Nc-1-IP_pe	chr10	44346332	44346714	383	44346477	19	7.59289	3.82098
Nc-1-IP_pe	chr10	44348805	44349112	308	44348937	20	9.39627	4.511
Nc-1-IP_pe	chr10	44352497	44352853	357	44352674	12	3.57938	2.60852
Nc-1-IP_pe	chr10	44358233	44358618	386	44358396	21	10.3528	4.8061
Nc-1-IP_pe	chr10	44359042	44359294	253	44359158	15	6.03738	3.55576
Nc-1-IP_pe	chr10	44362757	44363828	1072	44363474	19	6.78661	3.47027
Nc-1-IP_pe	chr10	44372066	44372361	296	44372357	14	3.33731	2.35937
Nc-1-IP_pe	chr10	44373449	44373741	293	44373567	19	6.22205	3.23373
Nc-1-IP_pe	chr10	44374195	44374679	485	44374200	19	3.48582	2.18208
Nc-1-IP_pe	chr10	44375081	44375358	278	44375225	14	5.02041	3.15766
Nc-1-IP_pe	chr10	44378469	44379204	736	44378819	13	4.83642	3.16601
Nc-1-IP_pe	chr10	44384504	44385735	1232	44384682	19	6.78661	3.47027
Nc-1-IP_pe	chr10	44386044	44387110	1067	44386899	23	12.3023	5.38534
Nc-1-IP_pe	chr10	44388024	44388278	255	44388166	19	7.29128	3.68799
Nc-1-IP_pe	chr10	44388656	44388977	322	44388795	18	7.1522	3.73811
Nc-1-IP_pe	chr10	44391808	44392261	454	44392035	15	5.20142	3.14789
Nc-1-IP_pe	chr10	44395064	44395470	407	44395263	15	3.74763	2.48304
Nc-1-IP_pe	chr10	44396621	44396981	361	44396845	15	5.81087	3.44334
Nc-1-IP_pe	chr10	44398858	44399338	481	44398974	16	6.65665	3.74206
Nc-1-IP_pe	chr10	44417754	44418113	360	44417951	20	9.63477	4.62254
Nc-1-IP_pe	chr10	44425324	44425705	382	44425513	16	5.98198	3.41707
Nc-1-IP_pe	chr10	44427800	44428354	555	44428043	13	4.98219	3.24203
Nc-1-IP_pe	chr10	44435010	44435542	533	44435270	17	5.54967	3.12324
Nc-1-IP_pe	chr10	44436119	44436388	270	44436307	16	5.18047	3.04664
Nc-1-IP_pe	chr10	44458515	44459086	572	44458935	20	7.43645	3.64378
Nc-1-IP_pe	chr10	44468048	44468346	299	44468169	19	6.32622	3.27682
Nc-1-IP_pe	chr10	44476048	44476756	709	44476195	17	4.44438	2.65333
Nc-1-IP_pe	chr10	44510505	44510763	259	44510670	15	5.66913	3.37372
Nc-1-IP_pe	chr10	44517320	44517617	298	44517446	16	5.50606	3.19508
Nc-1-IP_pe	chr10	44557847	44558517	671	44558338	22	10.3858	4.67178
Nc-1-IP_pe	chr10	44559336	44560022	687	44559741	16	5.50606	3.19508
Nc-1-IP_pe	chr10	44562374	44562631	258	44562560	14	5.20974	3.25233
Nc-1-IP_pe	chr10	44570696	44571202	507	44571078	14	5.35794	3.32713
Nc-1-IP_pe	chr10	44575138	44575678	541	44575259	16	6.51374	3.67221
Nc-1-IP_pe	chr10	44575942	44576334	393	44575955	16	3.1497	2.1782
Nc-1-IP_pe	chr10	44576850	44577117	268	44577023	14	5.03085	3.16286
Nc-1-IP_pe	chr10	44578051	44578616	566	44578257	15	6.39297	3.73508
Nc-1-IP_pe	chr10	44586317	44586621	305	44586400	17	6.13258	3.38303
Nc-1-IP_pe	chr10	44587150	44587596	447	44587556	14	3.55308	2.45762
Nc-1-IP_pe	chr10	44588002	44588273	272	44588165	13	4.56123	3.02417
Nc-1-IP_pe	chr10	44590603	44590880	278	44590748	19	8.58219	4.2724

Nc-1-IP_pe	chr10	44593993	44594614	622	44594297	16	3.94146	2.50592
Nc-1-IP_pe	chr10	44600629	44601404	776	44601303	13	3.52389	2.509
Nc-1-IP_pe	chr10	44601794	44602097	304	44601926	19	5.15822	2.80735
Nc-1-IP_pe	chr10	44632719	44633070	352	44632814	11	3.28475	2.53029
Nc-1-IP_pe	chr10	44633643	44634022	380	44633783	11	5.68685	3.89286
Nc-1-IP_pe	chr10	44647276	44647555	280	44647317	14	6.06519	3.69269
Nc-1-IP_pe	chr10	44651784	44652058	275	44651877	16	7.53857	4.18505
Nc-1-IP_pe	chr10	44666037	44666299	263	44666159	14	6.16097	3.74327
Nc-1-IP_pe	chr10	44699824	44700322	499	44700023	14	4.60011	2.95114
Nc-1-IP_pe	chr10	44715545	44715814	270	44715661	13	5.57038	3.55505
Nc-1-IP_pe	chr10	44757639	44758016	378	44757868	14	4.60011	2.95114
Nc-1-IP_pe	chr10	44784166	44784492	327	44784298	15	4.7819	2.95039
Nc-1-IP_pe	chr10	44815588	44815928	341	44815746	19	7.84735	3.93486
Nc-1-IP_pe	chr10	44818376	44819289	914	44819086	17	6.13258	3.38303
Nc-1-IP_pe	chr10	44822865	44823182	318	44822987	16	5.98198	3.41707
Nc-1-IP_pe	chr10	44826272	44826812	541	44826511	19	7.29128	3.68799
Nc-1-IP_pe	chr10	44827514	44827884	371	44827692	21	9.29881	4.32834
Nc-1-IP_pe	chr10	44829297	44829707	411	44829630	14	4.73128	3.01506
Nc-1-IP_pe	chr10	44848798	44849203	406	44849187	13	4.24481	2.8638
Nc-1-IP_pe	chr10	44853365	44853640	276	44853530	16	4.25252	2.63824
Nc-1-IP_pe	chr10	44855099	44855710	612	44855116	14	4.38411	2.84693
Nc-1-IP_pe	chr10	44856990	44857374	385	44857074	18	4.6358	2.66698
Nc-1-IP_pe	chr10	44859613	44860152	540	44859850	20	6.08336	3.09555
Nc-1-IP_pe	chr10	44864840	44865672	833	44865508	23	11.5921	5.06057
Nc-1-IP_pe	chr10	44875016	44875332	317	44875074	13	5.121	3.315
Nc-1-IP_pe	chr10	44876590	44877301	712	44877177	16	3.65575	2.38624
Nc-1-IP_pe	chr10	44880645	44881067	423	44880792	14	4.04994	2.68821
Nc-1-IP_pe	chr10	44889258	44889737	480	44889358	17	7.06589	3.81697
Nc-1-IP_pe	chr10	44890855	44891780	926	44891057	23	10.1326	4.42559
Nc-1-IP_pe	chr10	44895188	44895597	410	44895580	13	3.6894	2.58919
Nc-1-IP_pe	chr10	44895897	44896262	366	44895941	14	3.55308	2.45762
Nc-1-IP_pe	chr10	44896979	44897232	254	44897076	14	5.03085	3.16286
Nc-1-IP_pe	chr10	44902159	44902438	280	44902234	20	7.97282	3.87239
Nc-1-IP_pe	chr10	44907758	44908017	260	44907882	14	3.55308	2.45762
Nc-1-IP_pe	chr10	44911335	44911613	279	44911441	16	4.96462	2.94973
Nc-1-IP_pe	chr10	44912164	44912430	267	44912338	14	4.32616	2.81919
Nc-1-IP_pe	chr10	44912782	44913084	303	44912804	13	3.715	2.60167
Nc-1-IP_pe	chr10	44924074	44924530	457	44924130	19	7.18275	3.64066
Nc-1-IP_pe	chr10	44931359	44932644	1286	44932301	25	6.67663	2.96222
Nc-1-IP_pe	chr10	44935151	44935739	589	44935336	19	5.51632	2.94814
Nc-1-IP_pe	chr10	44950227	44950548	322	44950439	19	5.06054	2.7694
Nc-1-IP_pe	chr10	44959790	44960157	368	44960029	24	7.70148	3.38085
Nc-1-IP_pe	chr10	44962595	44963185	591	44962918	17	3.84884	2.41145
Nc-1-IP_pe	chr10	44969769	44970159	391	44970004	21	6.66991	3.24296
Nc-1-IP_pe	chr10	44970865	44971149	285	44971019	15	4.15779	2.66518

Nc-1-IP_pe	chr10	44972497	44972806	310	44972538	20	6.64096	3.31664
Nc-1-IP_pe	chr10	44974618	44974872	255	44974773	24	6.49734	2.96505
Nc-1-IP_pe	chr10	44975842	44976150	309	44976058	21	5.97042	2.97863
Nc-1-IP_pe	chr10	44976970	44978024	1055	44977612	21	5.5349	2.81884
Nc-1-IP_pe	chr10	44979593	44980165	573	44979760	26	6.47412	2.84038
Nc-1-IP_pe	chr10	44981970	44982484	515	44982259	25	3.74961	2.06877
Nc-1-IP_pe	chr10	44985537	44985803	267	44985709	19	6.23301	3.23825
Nc-1-IP_pe	chr10	44990484	44990980	497	44990550	18	6.27579	3.34803
Nc-1-IP_pe	chr10	44992160	44994117	1958	44992421	24	8.05897	3.50918
Nc-1-IP_pe	chr10	45014712	45015005	294	45014816	19	6.44601	3.32669
Nc-1-IP_pe	chr10	45045438	45045715	278	45045552	12	6.86906	4.44438
Nc-1-IP_pe	chr10	45047829	45048143	315	45047869	12	6.82053	4.41531
Nc-1-IP_pe	chr10	45060238	45060611	374	45060399	17	6.64406	3.61808
Nc-1-IP_pe	chr10	45062925	45063202	278	45063161	15	4.7819	2.95039
Nc-1-IP_pe	chr10	45087264	45087525	262	45087371	17	9.72281	5.17231
Nc-1-IP_pe	chr10	45092068	45092432	365	45092370	13	4.86362	3.18015
Nc-1-IP_pe	chr10	45114937	45115291	355	45115176	13	4.76564	3.12931
Nc-1-IP_pe	chr10	45121215	45121749	535	45121604	17	7.02067	3.79543
Nc-1-IP_pe	chr10	45124184	45124566	383	45124252	10	3.12201	2.51709
Nc-1-IP_pe	chr10	45151023	45152465	1443	45151793	19	6.78661	3.47027
Nc-1-IP_pe	chr10	45205267	45205871	605	45205596	20	6.07662	3.09292
Nc-1-IP_pe	chr10	45224322	45225030	709	45224549	19	7.29128	3.68799
Nc-1-IP_pe	chr10	45250517	45250973	457	45250594	18	6.91333	3.62993
Nc-1-IP_pe	chr10	45259343	45259798	456	45259544	17	4.96303	2.87025
Nc-1-IP_pe	chr10	45260122	45260600	479	45260286	16	4.25252	2.63824
Nc-1-IP_pe	chr10	45301266	45301975	710	45301286	15	3.74763	2.48304
Nc-1-IP_pe	chr10	45303611	45304120	510	45303825	21	7.98609	3.76767
Nc-1-IP_pe	chr10	45332909	45333887	979	45333055	18	7.62523	3.95652
Nc-1-IP_pe	chr10	45334314	45335194	881	45335052	18	6.15736	3.29676
Nc-1-IP_pe	chr10	45346328	45347096	769	45346723	16	3.94146	2.50592
Nc-1-IP_pe	chr10	45347386	45348103	718	45347525	20	7.85753	3.8227
Nc-1-IP_pe	chr10	45358749	45359424	676	45358903	19	7.84735	3.93486
Nc-1-IP_pe	chr10	45362451	45362920	470	45362740	19	3.71826	2.26621
Nc-1-IP_pe	chr10	45373240	45373938	699	45373848	28	8.5728	3.37037
Nc-1-IP_pe	chr10	45374370	45374642	273	45374464	25	5.3279	2.53752
Nc-1-IP_pe	chr10	45375678	45376055	378	45375829	22	7.7408	3.56938
Nc-1-IP_pe	chr10	45379131	45379840	710	45379213	20	4.15754	2.37952
Nc-1-IP_pe	chr10	45381617	45381892	276	45381717	19	5.72261	3.03049
Nc-1-IP_pe	chr10	45384104	45384728	625	45384218	18	4.04135	2.43445
Nc-1-IP_pe	chr10	45387580	45388629	1050	45388380	19	7.42515	3.74675
Nc-1-IP_pe	chr10	45391130	45392220	1091	45391519	25	8.22195	3.4832
Nc-1-IP_pe	chr10	45418149	45418723	575	45418561	23	4.4731	2.35612
Nc-1-IP_pe	chr10	45421561	45422569	1009	45422503	18	5.36045	2.96037
Nc-1-IP_pe	chr10	45423167	45423451	285	45423240	19	6.11371	3.18917
Nc-1-IP_pe	chr10	45423807	45424071	265	45423927	18	5.69081	3.09801

Nc-1-IP_pe	chr10	45425731	45426118	388	45425957	27	9.006	3.58762
Nc-1-IP_pe	chr10	45427404	45428041	638	45427958	18	6.15736	3.29676
Nc-1-IP_pe	chr10	45433206	45433501	296	45433332	19	6.78661	3.47027
Nc-1-IP_pe	chr10	45439482	45439810	329	45439576	20	7.19732	3.54396
Nc-1-IP_pe	chr10	45440696	45441262	567	45440923	16	4.97667	2.95511
Nc-1-IP_pe	chr10	45441525	45442303	779	45442046	20	7.61095	3.71744
Nc-1-IP_pe	chr10	45442968	45444489	1522	45443911	25	5.37675	2.55246
Nc-1-IP_pe	chr10	45445120	45445603	484	45445340	17	6.24301	3.43321
Nc-1-IP_pe	chr10	45446466	45447240	775	45446717	16	5.65518	3.264
Nc-1-IP_pe	chr10	45461142	45461404	263	45461244	16	4.9371	2.93746
Nc-1-IP_pe	chr10	45463188	45463587	400	45463278	19	4.5193	2.56258
Nc-1-IP_pe	chr10	45465322	45465749	428	45465562	25	8.22195	3.4832
Nc-1-IP_pe	chr10	45466833	45467106	274	45467001	19	5.51632	2.94814
Nc-1-IP_pe	chr10	45471942	45472211	270	45472137	17	5.9911	3.31919
Nc-1-IP_pe	chr10	45474735	45475138	404	45474991	16	3.39261	2.27748
Nc-1-IP_pe	chr10	45480412	45481126	715	45480540	21	10.4313	4.84266
Nc-1-IP_pe	chr10	45488014	45488471	458	45488063	15	5.70822	3.39286
Nc-1-IP_pe	chr10	45511525	45512739	1215	45512243	70	23.2909	4.17871
Nc-1-IP_pe	chr10	45533889	45534200	312	45534064	17	6.47874	3.54137
Nc-1-IP_pe	chr10	45543564	45543994	431	45543619	13	5.42865	3.47872
Nc-1-IP_pe	chr10	45554600	45554912	313	45554710	22	8.79433	3.99081
Nc-1-IP_pe	chr10	45568427	45568933	507	45568547	15	6.45255	3.76546
Nc-1-IP_pe	chr10	45571988	45572298	311	45572050	14	3.86727	2.6027
Nc-1-IP_pe	chr10	45594337	45595056	720	45594866	26	7.17069	3.05938
Nc-1-IP_pe	chr10	45601574	45601905	332	45601645	21	7.98609	3.76767
Nc-1-IP_pe	chr10	45624933	45625291	359	45625037	13	4.86362	3.18015
Nc-1-IP_pe	chr10	45637049	45637660	612	45637219	13	6.52521	4.08375
Nc-1-IP_pe	chr10	45650189	45650517	329	45650345	15	7.43258	4.27887
Nc-1-IP_pe	chr10	45698896	45699573	678	45699399	19	5.15822	2.80735
Nc-1-IP_pe	chr10	45713065	45713461	397	45713237	21	6.2632	3.08808
Nc-1-IP_pe	chr10	45726870	45728374	1505	45727210	51	16.058	3.82675
Nc-1-IP_pe	chr10	45749210	45749562	353	45749312	15	5.66913	3.37372
Nc-1-IP_pe	chr10	45787507	45787799	293	45787611	16	4.42092	2.71079
Nc-1-IP_pe	chr10	45812752	45813223	472	45813049	26	10.439	4.19014
Nc-1-IP_pe	chr10	45817072	45817568	497	45817195	25	8.22195	3.4832
Nc-1-IP_pe	chr10	45818575	45819263	689	45818898	22	7.86456	3.6177
Nc-1-IP_pe	chr10	45825953	45826317	365	45826242	24	6.80641	3.06942
Nc-1-IP_pe	chr10	45847378	45847716	339	45847381	18	2.88361	1.99878
Nc-1-IP_pe	chr10	45849717	45850217	501	45849799	16	5.82808	3.34463
Nc-1-IP_pe	chr10	45856171	45856541	371	45856374	16	5.94447	3.39936
Nc-1-IP_pe	chr10	45857761	45858252	492	45857924	14	4.60011	2.95114
Nc-1-IP_pe	chr10	45865292	45866313	1022	45866170	26	12.4493	4.97879
Nc-1-IP_pe	chr10	45868986	45869593	608	45869143	26	9.86937	3.97999
Nc-1-IP_pe	chr10	45887565	45887853	289	45887714	17	9.28874	4.93928
Nc-1-IP_pe	chr10	45914407	45914670	264	45914581	13	5.29187	3.40559

Nc-1-IP_pe	chr10	45921866	45922174	309	45922016	13	6.19316	3.89711
Nc-1-IP_pe	chr10	45971924	45972792	869	45972200	27	5.75532	2.57668
Nc-1-IP_pe	chr10	45992053	45992341	289	45992201	20	10.1399	4.86305
Nc-1-IP_pe	chr10	46023147	46023482	336	46023307	28	9.16687	3.56053
Nc-1-IP_pe	chr10	46030169	46030713	545	46030330	32	7.28348	2.77605
Nc-1-IP_pe	chr10	46031062	46031529	468	46031461	17	5.14803	2.94914
Nc-1-IP_pe	chr10	46036061	46036321	261	46036169	26	11.7219	4.68487
Nc-1-IP_pe	chr10	46039490	46039833	344	46039639	20	7.32334	3.59641
Nc-1-IP_pe	chr10	46040710	46041249	540	46040865	18	7.0346	3.68468
Nc-1-IP_pe	chr10	46044827	46045413	587	46045006	21	9.4928	4.41442
Nc-1-IP_pe	chr10	46052765	46053315	551	46053216	22	7.7408	3.56938
Nc-1-IP_pe	chr10	46055216	46055501	286	46055329	18	5.33197	2.94862
Nc-1-IP_pe	chr10	46060298	46060942	645	46060509	18	5.33197	2.94862
Nc-1-IP_pe	chr10	46076162	46076491	330	46076276	18	5.83846	3.16035
Nc-1-IP_pe	chr10	46079060	46079884	825	46079818	15	4.06016	2.62146
Nc-1-IP_pe	chr10	46091077	46091849	773	46091247	23	9.21857	4.05024
Nc-1-IP_pe	chr10	46092161	46092852	692	46092266	20	5.01743	2.69071
Nc-1-IP_pe	chr10	46095139	46095958	820	46095764	24	6.80641	3.06942
Nc-1-IP_pe	chr10	46097286	46097741	456	46097386	21	5.88587	2.94733
Nc-1-IP_pe	chr10	46106037	46106470	434	46106273	24	6.87207	3.0918
Nc-1-IP_pe	chr10	46107990	46108306	317	46108098	18	6.63038	3.50359
Nc-1-IP_pe	chr10	46126507	46127037	531	46126889	17	4.78101	2.79343
Nc-1-IP_pe	chr10	46129972	46130879	908	46130080	17	5.54967	3.12324
Nc-1-IP_pe	chr10	46133732	46134028	297	46133875	19	6.0077	3.14582
Nc-1-IP_pe	chr10	46204622	46204968	347	46204716	19	6.78661	3.47027
Nc-1-IP_pe	chr10	46214080	46214678	599	46214383	20	5.34663	2.81336
Nc-1-IP_pe	chr10	46215544	46215934	391	46215731	21	6.2632	3.08808
Nc-1-IP_pe	chr10	46216405	46217403	999	46217289	23	8.13503	3.62709
Nc-1-IP_pe	chr10	46221043	46221821	779	46221655	26	10.6054	4.25264
Nc-1-IP_pe	chr10	46223239	46224145	907	46223406	27	6.34328	2.7488
Nc-1-IP_pe	chr10	46225496	46225872	377	46225770	17	3.83544	2.40609
Nc-1-IP_pe	chr10	46228478	46228960	483	46228775	23	5.96384	2.84645
Nc-1-IP_pe	chr10	46232011	46232361	351	46232247	21	7.34645	3.50809
Nc-1-IP_pe	chr10	46239983	46241358	1376	46240969	20	7.0741	3.49302
Nc-1-IP_pe	chr10	46248718	46250101	1384	46249489	28	11.405	4.32623
Nc-1-IP_pe	chr10	46250492	46251645	1154	46251603	20	6.88252	3.4145
Nc-1-IP_pe	chr10	46252196	46252740	545	46252576	19	6.21111	3.22922
Nc-1-IP_pe	chr10	46253235	46253785	551	46253701	19	4.23334	2.45554
Nc-1-IP_pe	chr10	46254301	46255096	796	46254849	20	8.46117	4.08623
Nc-1-IP_pe	chr10	46264350	46267234	2885	46265687	22	7.27519	3.39036
Nc-1-IP_pe	chr10	46268570	46269054	485	46268874	21	6.2632	3.08808
Nc-1-IP_pe	chr10	46272013	46273200	1188	46273030	21	4.90204	2.59278
Nc-1-IP_pe	chr10	46274115	46274403	289	46274279	15	3.74763	2.48304
Nc-1-IP_pe	chr10	46278538	46278821	284	46278742	14	4.94039	3.11796
Nc-1-IP_pe	chr10	46282427	46283812	1386	46282837	37	18.8293	5.81888

Nc-1-IP_pe	chr10	46284707	46285186	480	46284943	34	10.5986	3.55474
Nc-1-IP_pe	chr10	46286160	46286773	614	46286619	22	7.2676	3.38748
Nc-1-IP_pe	chr10	46292369	46293276	908	46292934	21	7.05134	3.39126
Nc-1-IP_pe	chr10	46296785	46297227	443	46296988	26	8.13668	3.37521
Nc-1-IP_pe	chr10	46297810	46298166	357	46297961	21	5.2077	2.70109
Nc-1-IP_pe	chr10	46300156	46300662	507	46300397	21	6.76008	3.27774
Nc-1-IP_pe	chr10	46301772	46302465	694	46302201	24	9.18001	3.92694
Nc-1-IP_pe	chr10	46307228	46308317	1090	46308072	26	7.95585	3.31498
Nc-1-IP_pe	chr10	46308647	46309133	487	46309016	20	3.05104	1.99497
Nc-1-IP_pe	chr10	46309446	46310170	725	46309549	24	5.81603	2.74037
Nc-1-IP_pe	chr10	46311554	46312807	1254	46312667	30	7.40438	2.90308
Nc-1-IP_pe	chr10	46313881	46314948	1068	46314825	23	3.39514	2.01894
Nc-1-IP_pe	chr10	46315205	46315552	348	46315427	33	6.61114	2.56636
Nc-1-IP_pe	chr10	46326658	46327370	713	46327311	24	5.18704	2.5391
Nc-1-IP_pe	chr10	46331539	46332113	575	46331740	16	4.38656	2.69594
Nc-1-IP_pe	chr10	46334361	46334814	454	46334743	15	4.4329	2.78966
Nc-1-IP_pe	chr10	46336710	46337476	767	46337101	26	8.58731	3.52764
Nc-1-IP_pe	chr10	46357850	46358687	838	46358009	24	7.19588	3.20323
Nc-1-IP_pe	chr10	46361826	46362095	270	46361876	19	6.0182	3.1501
Nc-1-IP_pe	chr10	46363332	46363610	279	46363426	20	6.26143	3.16543
Nc-1-IP_pe	chr10	46364505	46365295	791	46364755	37	11.9932	3.73052
Nc-1-IP_pe	chr10	46366530	46367442	913	46366913	21	4.90204	2.59278
Nc-1-IP_pe	chr10	46367720	46369433	1714	46369083	25	6.28656	2.83671
Nc-1-IP_pe	chr10	46370865	46371642	778	46371059	28	7.53611	3.05077
Nc-1-IP_pe	chr10	46372359	46372688	330	46372465	20	5.16039	2.74372
Nc-1-IP_pe	chr10	46374831	46375268	438	46375170	21	5.28723	2.72953
Nc-1-IP_pe	chr10	46375738	46376601	864	46375825	24	5.58146	2.66465
Nc-1-IP_pe	chr10	46376906	46377827	922	46377705	31	6.23593	2.54618
Nc-1-IP_pe	chr10	46379706	46380281	576	46380064	28	7.52977	3.04887
Nc-1-IP_pe	chr10	46381134	46381564	431	46381407	25	4.60609	2.31999
Nc-1-IP_pe	chr10	46382576	46382922	347	46382645	27	6.89598	2.91461
Nc-1-IP_pe	chr10	46384544	46384849	306	46384703	24	3.21005	1.93677
Nc-1-IP_pe	chr10	46385271	46386208	938	46385949	26	7.04602	3.01966
Nc-1-IP_pe	chr10	46387005	46387278	274	46387211	24	4.37085	2.2857
Nc-1-IP_pe	chr10	46392318	46393728	1411	46393289	25	8.39794	3.54508
Nc-1-IP_pe	chr10	46397570	46398530	961	46397769	37	9.53791	3.10771
Nc-1-IP_pe	chr10	46411779	46412208	430	46411990	19	4.8268	2.67939
Nc-1-IP_pe	chr10	46412791	46413224	434	46413098	22	8.52649	3.88148
Nc-1-IP_pe	chr10	46418431	46418774	344	46418517	22	4.04626	2.25795
Nc-1-IP_pe	chr10	46425161	46425581	421	46425244	15	4.23553	2.70016
Nc-1-IP_pe	chr10	46425951	46426339	389	46426110	20	5.34663	2.81336
Nc-1-IP_pe	chr10	46427109	46427418	310	46427240	20	8.55484	4.12788
Nc-1-IP_pe	chr10	46437569	46438073	505	46437796	15	4.67074	2.89885
Nc-1-IP_pe	chr10	46438597	46439220	624	46439095	22	5.09279	2.60614
Nc-1-IP_pe	chr10	46443940	46445010	1071	46444004	20	6.45316	3.24143

Nc-1-IP_pe	chr10	46445624	46446105	482	46445970	18	6.12556	3.28305
Nc-1-IP_pe	chr10	46455123	46455495	373	46455172	16	4.78259	2.86892
Nc-1-IP_pe	chr10	46457048	46457326	279	46457239	20	7.92792	3.853
Nc-1-IP_pe	chr10	46462045	46462579	535	46462273	23	5.5873	2.71945
Nc-1-IP_pe	chr10	46464810	46465273	464	46465167	21	4.0961	2.31438
Nc-1-IP_pe	chr10	46465757	46466179	423	46466060	30	9.06325	3.38223
Nc-1-IP_pe	chr10	46489979	46490594	616	46490208	20	8.92783	4.2957
Nc-1-IP_pe	chr10	46491391	46492014	624	46491702	17	6.47874	3.54137
Nc-1-IP_pe	chr10	46497668	46498233	566	46497891	22	10.0534	4.52509
Nc-1-IP_pe	chr10	46500749	46501024	276	46500934	13	4.62808	3.05843
Nc-1-IP_pe	chr10	46510313	46510586	274	46510537	16	4.96462	2.94973
Nc-1-IP_pe	chr10	46514321	46514952	632	46514639	31	12.8059	4.49176
Nc-1-IP_pe	chr10	46517035	46517437	403	46517228	35	6.98573	2.58457
Nc-1-IP_pe	chr10	46519788	46520061	274	46519935	23	5.07748	2.55101
Nc-1-IP_pe	chr10	46520558	46521263	706	46521033	33	10.1907	3.50761
Nc-1-IP_pe	chr10	46521845	46522434	590	46522238	38	10.0829	3.1895
Nc-1-IP_pe	chr10	46523249	46523855	607	46523712	41	9.92358	3.01534
Nc-1-IP_pe	chr10	46524678	46524947	270	46524796	31	5.03387	2.24262
Nc-1-IP_pe	chr10	46526641	46527003	363	46526794	31	9.31262	3.3889
Nc-1-IP_pe	chr10	46527308	46527632	325	46527478	29	7.71914	3.04692
Nc-1-IP_pe	chr10	46527924	46528667	744	46528247	26	5.0372	2.40922
Nc-1-IP_pe	chr10	46534163	46534439	277	46534332	18	4.18715	2.49085
Nc-1-IP_pe	chr10	46540694	46541432	739	46541134	45	16.192	4.23311
Nc-1-IP_pe	chr10	46542740	46543531	792	46542879	25	4.60609	2.31999
Nc-1-IP_pe	chr10	46545147	46545706	560	46545317	31	6.22686	2.54384
Nc-1-IP_pe	chr10	46547891	46548827	937	46548012	27	5.26054	2.43494
Nc-1-IP_pe	chr10	46549232	46550768	1537	46549589	36	10.1498	3.31219
Nc-1-IP_pe	chr10	46552242	46552823	582	46552250	26	2.90503	1.80608
Nc-1-IP_pe	chr10	46553183	46553615	433	46553438	34	9.16271	3.16745
Nc-1-IP_pe	chr10	46554777	46555309	533	46554834	24	4.36495	2.2839
Nc-1-IP_pe	chr10	46555674	46558459	2786	46557636	38	12.063	3.68206
Nc-1-IP_pe	chr10	46558914	46559717	804	46559526	29	7.36869	2.94515
Nc-1-IP_pe	chr10	46564414	46564676	263	46564567	16	4.98663	2.95956
Nc-1-IP_pe	chr10	46572974	46573232	259	46573032	24	7.19588	3.20323
Nc-1-IP_pe	chr10	46573690	46574322	633	46573897	26	6.81227	2.94581
Nc-1-IP_pe	chr10	46578615	46579554	940	46579150	30	7.90234	3.04332
Nc-1-IP_pe	chr10	46579955	46581770	1816	46580295	23	6.25615	2.94664
Nc-1-IP_pe	chr10	46583578	46584931	1354	46584351	24	7.93912	3.4659
Nc-1-IP_pe	chr10	46585334	46586075	742	46585490	21	5.2077	2.70109
Nc-1-IP_pe	chr10	46586944	46587857	914	46587185	30	13.2618	4.7659
Nc-1-IP_pe	chr10	46595413	46596152	740	46595717	21	6.99413	3.36883
Nc-1-IP_pe	chr10	46597583	46598039	457	46597808	33	7.471	2.78059
Nc-1-IP_pe	chr10	46613495	46613911	417	46613895	15	3.20025	2.24588
Nc-1-IP_pe	chr10	46648803	46649182	380	46648990	15	4.7819	2.95039
Nc-1-IP_pe	chr10	46653594	46653890	297	46653762	22	7.7408	3.56938

Nc-1-IP_pe	chr10	46658273	46658534	262	46658387	22	6.44373	3.08129
Nc-1-IP_pe	chr10	46690160	46690850	691	46690223	17	4.44438	2.65333
Nc-1-IP_pe	chr10	46747299	46748190	892	46747348	14	3.26761	2.32785
Nc-1-IP_pe	chr10	46753409	46753856	448	46753778	14	4.59463	2.94848
Nc-1-IP_pe	chr10	46756364	46757500	1137	46757318	26	5.29477	2.48465
Nc-1-IP_pe	chr10	46761056	46761390	335	46761198	22	7.7408	3.56938
Nc-1-IP_pe	chr10	46763001	46763295	295	46763226	16	5.37454	3.13479
Nc-1-IP_pe	chr10	46767111	46767375	265	46767156	15	5.90272	3.48876
Nc-1-IP_pe	chr10	46773400	46774059	660	46773929	24	9.92801	4.219
Nc-1-IP_pe	chr10	46774819	46776287	1469	46775049	29	9.79421	3.68331
Nc-1-IP_pe	chr10	46783089	46783348	260	46783187	15	4.7819	2.95039
Nc-1-IP_pe	chr10	46786593	46787149	557	46786656	25	6.62684	2.94607
Nc-1-IP_pe	chr10	46805803	46806125	323	46805907	18	4.6358	2.66698
Nc-1-IP_pe	chr10	46807376	46807663	288	46807515	23	6.62483	3.0751
Nc-1-IP_pe	chr10	46816006	46816785	780	46816575	23	8.39	3.72457
Nc-1-IP_pe	chr10	46840837	46841529	693	46841313	19	5.90394	3.10364
Nc-1-IP_pe	chr10	46843857	46844313	457	46843919	14	4.742	3.02031
Nc-1-IP_pe	chr10	46865122	46865647	526	46865239	18	4.04135	2.43445
Nc-1-IP_pe	chr10	46871726	46872013	288	46871908	22	10.6105	4.7723
Nc-1-IP_pe	chr10	46881811	46882447	637	46882369	21	7.58657	3.60451
Nc-1-IP_pe	chr10	46883175	46883432	258	46883308	16	4.96462	2.94973
Nc-1-IP_pe	chr10	46906426	46906762	337	46906610	23	10.1636	4.43862
Nc-1-IP_pe	chr10	46908863	46909218	356	46909011	17	5.83879	3.25103
Nc-1-IP_pe	chr10	46911051	46911723	673	46911488	23	5.28318	2.6185
Nc-1-IP_pe	chr10	46920541	46920821	281	46920675	14	3.55308	2.45762
Nc-1-IP_pe	chr10	46948719	46949280	562	46948787	15	4.7819	2.95039
Nc-1-IP_pe	chr10	46965960	46966215	256	46966173	18	4.6358	2.66698
Nc-1-IP_pe	chr10	46973790	46974215	426	46973856	19	5.51632	2.94814
Nc-1-IP_pe	chr10	47012839	47013147	309	47012985	17	6.71194	3.64977
Nc-1-IP_pe	chr10	47013723	47014016	294	47013936	18	4.96968	2.80074
Nc-1-IP_pe	chr10	47016632	47016971	340	47016781	20	7.97282	3.87239
Nc-1-IP_pe	chr10	47022573	47023003	431	47022689	21	3.59899	2.14713
Nc-1-IP_pe	chr10	47024715	47025807	1093	47025672	20	5.9768	3.05405
Nc-1-IP_pe	chr10	47026591	47027798	1208	47027535	22	7.30177	3.40047
Nc-1-IP_pe	chr10	47031917	47032336	420	47032209	15	3.95045	2.57261
Nc-1-IP_pe	chr10	47033337	47033929	593	47033861	18	5.33197	2.94862
Nc-1-IP_pe	chr10	47035368	47036482	1115	47035738	19	6.57353	3.38013
Nc-1-IP_pe	chr10	47036794	47037217	424	47037142	22	8.24538	3.76835
Nc-1-IP_pe	chr10	47043224	47043598	375	47043407	18	4.6358	2.66698
Nc-1-IP_pe	chr10	47044001	47044415	415	47044275	23	6.25615	2.94664
Nc-1-IP_pe	chr10	47045224	47045910	687	47045338	17	4.44438	2.65333
Nc-1-IP_pe	chr10	47046698	47047159	462	47046789	16	4.09083	2.56919
Nc-1-IP_pe	chr10	47047466	47048737	1272	47048149	25	8.69165	3.64955
Nc-1-IP_pe	chr10	47049618	47049939	322	47049747	22	7.7403	3.56918
Nc-1-IP_pe	chr10	47051555	47051938	384	47051657	18	5.95782	3.21113

Nc-1-IP_pe	chr10	47054879	47055140	262	47054989	14	4.18531	2.75214
Nc-1-IP_pe	chr10	47059046	47060910	1865	47060419	17	3.84884	2.41145
Nc-1-IP_pe	chr10	47077594	47078426	833	47077967	18	7.18674	3.75388
Nc-1-IP_pe	chr10	47078797	47079175	379	47078995	21	8.10596	3.8173
Nc-1-IP_pe	chr10	47080856	47081147	292	47080867	17	3.58449	2.30632
Nc-1-IP_pe	chr10	47082151	47082594	444	47082213	17	4.44438	2.65333
Nc-1-IP_pe	chr10	47083366	47083960	595	47083407	16	5.46118	3.17446
Nc-1-IP_pe	chr10	47085299	47085575	277	47085470	20	6.94658	3.44067
Nc-1-IP_pe	chr10	47091106	47091484	379	47091440	15	3.90248	2.55134
Nc-1-IP_pe	chr10	47095152	47095776	625	47095271	23	8.92223	3.93219
Nc-1-IP_pe	chr10	47099465	47099753	289	47099587	19	5.80235	3.06257
Nc-1-IP_pe	chr10	47102720	47103017	298	47102962	12	2.87625	2.25568
Nc-1-IP_pe	chr10	47107212	47107804	593	47107660	17	4.13464	2.52661
Nc-1-IP_pe	chr10	47123844	47124425	582	47124018	19	7.29128	3.68799
Nc-1-IP_pe	chr10	47127185	47127545	361	47127477	14	3.00733	2.21111
Nc-1-IP_pe	chr10	47128715	47128990	276	47128906	19	9.05528	4.49651
Nc-1-IP_pe	chr10	47132697	47133046	350	47132931	19	7.64995	3.84638
Nc-1-IP_pe	chr10	47142075	47142405	331	47142195	22	5.72303	2.82387
Nc-1-IP_pe	chr10	47143377	47143767	391	47143406	19	4.89276	2.70468
Nc-1-IP_pe	chr10	47144628	47144883	256	47144764	21	5.2077	2.70109
Nc-1-IP_pe	chr10	47150578	47150893	316	47150682	18	4.04135	2.43445
Nc-1-IP_pe	chr10	47159919	47160819	901	47160578	20	6.08336	3.09555
Nc-1-IP_pe	chr10	47164480	47164831	352	47164653	25	9.7397	4.03495
Nc-1-IP_pe	chr10	47165325	47165823	499	47165507	23	7.01947	3.21526
Nc-1-IP_pe	chr10	47166282	47167378	1097	47166461	26	10.439	4.19014
Nc-1-IP_pe	chr10	47175086	47175970	885	47175495	21	8.702	4.06873
Nc-1-IP_pe	chr10	47176318	47176904	587	47176590	15	5.00915	3.05678
Nc-1-IP_pe	chr10	47178992	47179528	537	47179240	21	9.42449	4.38402
Nc-1-IP_pe	chr10	47184585	47185460	876	47184978	18	5.72638	3.11298
Nc-1-IP_pe	chr10	47187263	47188182	920	47187962	22	6.4249	3.07445
Nc-1-IP_pe	chr10	47191263	47191665	403	47191381	18	4.96968	2.80074
Nc-1-IP_pe	chr10	47192776	47193289	514	47192978	21	7.10914	3.414
Nc-1-IP_pe	chr10	47196189	47197230	1042	47196294	22	5.72303	2.82387
Nc-1-IP_pe	chr10	47218738	47219169	432	47218910	22	9.39426	4.24119
Nc-1-IP_pe	chr10	47219870	47220145	276	47220063	13	3.67377	2.58159
Nc-1-IP_pe	chr10	47222357	47222721	365	47222661	16	6.24025	3.54005
Nc-1-IP_pe	chr10	47226446	47226997	552	47226681	22	7.86456	3.6177
Nc-1-IP_pe	chr10	47227379	47228009	631	47227475	22	9.92229	4.46789
Nc-1-IP_pe	chr10	47238462	47238773	312	47238617	20	6.49719	3.259
Nc-1-IP_pe	chr10	47240025	47240402	378	47240234	17	5.70414	3.19126
Nc-1-IP_pe	chr10	47243387	47243961	575	47243810	18	4.32723	2.54542
Nc-1-IP_pe	chr10	47247170	47247469	300	47247195	19	4.5193	2.56258
Nc-1-IP_pe	chr10	47247770	47248109	340	47247947	22	8.63919	3.9273
Nc-1-IP_pe	chr10	47248723	47249380	658	47248800	19	6.02872	3.1544
Nc-1-IP_pe	chr10	47252084	47252515	432	47252165	17	5.03828	2.90225

Nc-1-IP_pe	chr10	47253938	47254254	317	47254091	23	4.99703	2.52478
Nc-1-IP_pe	chr10	47265108	47265390	283	47265286	16	6.64217	3.73495
Nc-1-IP_pe	chr10	47276324	47278010	1687	47276397	19	4.8268	2.67939
Nc-1-IP_pe	chr10	47279168	47279925	758	47279363	20	4.26111	2.41635
Nc-1-IP_pe	chr10	47282131	47282423	293	47282253	18	5.3029	2.93664
Nc-1-IP_pe	chr10	47287108	47287535	428	47287468	18	3.44647	2.20818
Nc-1-IP_pe	chr10	47287804	47288370	567	47287971	19	4.5193	2.56258
Nc-1-IP_pe	chr10	47288919	47289819	901	47288992	17	5.01934	2.89418
Nc-1-IP_pe	chr10	47295475	47295779	305	47295569	13	4.02562	2.7544
Nc-1-IP_pe	chr10	47300036	47301714	1679	47300501	26	9.86007	3.97661
Nc-1-IP_pe	chr10	47305173	47305707	535	47305575	18	5.56789	3.0465
Nc-1-IP_pe	chr10	47306805	47307067	263	47307004	19	5.92451	3.11198
Nc-1-IP_pe	chr10	47310030	47310538	509	47310342	25	7.24165	3.14818
Nc-1-IP_pe	chr10	47312248	47312582	335	47312444	26	6.81227	2.94581
Nc-1-IP_pe	chr10	47314406	47314865	460	47314778	16	4.23572	2.63104
Nc-1-IP_pe	chr10	47318807	47319136	330	47318986	21	7.69604	3.64887
Nc-1-IP_pe	chr10	47319702	47320092	391	47319948	14	3.72955	2.53881
Nc-1-IP_pe	chr10	47322512	47322781	270	47322637	20	5.16039	2.74372
Nc-1-IP_pe	chr10	47323763	47324031	269	47323859	21	7.09753	3.40943
Nc-1-IP_pe	chr10	47324803	47325989	1187	47325466	27	9.48843	3.75114
Nc-1-IP_pe	chr10	47328294	47330621	2328	47329307	23	8.39	3.72457
Nc-1-IP_pe	chr10	47331382	47331751	370	47331655	17	4.78065	2.79327
Nc-1-IP_pe	chr10	47335597	47335972	376	47335794	19	6.76483	3.46101
Nc-1-IP_pe	chr10	47350035	47351201	1167	47350209	28	9.24671	3.58649
Nc-1-IP_pe	chr10	47353465	47353726	262	47353574	20	5.34663	2.81336
Nc-1-IP_pe	chr10	47357255	47357515	261	47357375	17	5.34321	3.03326
Nc-1-IP_pe	chr10	47358537	47358897	361	47358789	22	4.53777	2.41958
Nc-1-IP_pe	chr10	47359256	47359560	305	47359432	19	5.14691	2.80294
Nc-1-IP_pe	chr10	47365001	47365313	313	47365154	21	4.61597	2.49283
Nc-1-IP_pe	chr10	47368907	47369315	409	47369120	25	7.5452	3.25021
Nc-1-IP_pe	chr10	47370854	47371120	267	47371011	23	9.15996	4.02675
Nc-1-IP_pe	chr10	47376482	47376739	258	47376734	17	3.56805	2.29983
Nc-1-IP_pe	chr10	47388653	47389102	450	47389001	17	6.72563	3.65618
Nc-1-IP_pe	chr10	47402091	47402376	286	47402117	19	4.50014	2.55537
Nc-1-IP_pe	chr10	47403445	47404144	700	47403493	25	4.26554	2.21932
Nc-1-IP_pe	chr10	47409152	47410030	879	47409474	31	11.6262	4.10013
Nc-1-IP_pe	chr10	47413609	47414262	654	47414087	27	7.72987	3.17269
Nc-1-IP_pe	chr10	47416254	47416865	612	47416364	20	4.61297	2.54276
Nc-1-IP_pe	chr10	47417179	47419085	1907	47417613	30	11.8375	4.26705
Nc-1-IP_pe	chr10	47419419	47419859	441	47419757	25	6.62684	2.94607
Nc-1-IP_pe	chr10	47420447	47421331	885	47420518	27	6.66155	2.84379
Nc-1-IP_pe	chr10	47425900	47426484	585	47425971	19	4.5193	2.56258
Nc-1-IP_pe	chr10	47433631	47434190	560	47434074	21	6.2632	3.08808
Nc-1-IP_pe	chr10	47442085	47442920	836	47442835	21	5.5349	2.81884
Nc-1-IP_pe	chr10	47449984	47450942	959	47450006	20	4.00033	2.32392

Nc-1-IP_pe	chr10	47451429	47452477	1049	47451950	25	6.5875	2.93334
Nc-1-IP_pe	chr10	47453740	47454253	514	47453777	20	4.71088	2.57831
Nc-1-IP_pe	chr10	47455475	47455832	358	47455649	20	5.34663	2.81336
Nc-1-IP_pe	chr10	47456984	47457710	727	47457475	29	11.9799	4.42221
Nc-1-IP_pe	chr10	47461591	47462277	687	47461769	30	7.07552	2.81206
Nc-1-IP_pe	chr10	47464588	47464891	304	47464755	17	4.78101	2.79343
Nc-1-IP_pe	chr10	47465679	47466012	334	47465724	15	3.82401	2.51666
Nc-1-IP_pe	chr10	47466318	47468018	1701	47467714	25	6.98838	3.0642
Nc-1-IP_pe	chr10	47468414	47469033	620	47468686	27	10.7399	4.19327
Nc-1-IP_pe	chr10	47471030	47471787	758	47471156	23	6.92762	3.18239
Nc-1-IP_pe	chr10	47472775	47473448	674	47472856	27	5.912	2.62214
Nc-1-IP_pe	chr10	47474422	47474813	392	47474555	21	3.63688	2.15978
Nc-1-IP_pe	chr10	47475431	47475752	322	47475722	20	4.77009	2.59989
Nc-1-IP_pe	chr10	47476763	47477344	582	47477157	25	7.5452	3.25021
Nc-1-IP_pe	chr10	47479243	47479908	666	47479812	21	4.31457	2.38889
Nc-1-IP_pe	chr10	47481159	47481636	478	47481240	19	4.89276	2.70468
Nc-1-IP_pe	chr10	47482319	47482748	430	47482552	30	9.06325	3.38223
Nc-1-IP_pe	chr10	47484651	47485036	386	47484784	17	4.8075	2.80456
Nc-1-IP_pe	chr10	47489479	47489962	484	47489723	19	4.8268	2.67939
Nc-1-IP_pe	chr10	47490779	47491146	368	47490966	24	10.7113	4.53656
Nc-1-IP_pe	chr10	47491600	47493076	1477	47492865	27	9.46411	3.74281
Nc-1-IP_pe	chr10	47493586	47493878	293	47493811	21	6.88144	3.32483
Nc-1-IP_pe	chr10	47495731	47496319	589	47495839	23	8.39	3.72457
Nc-1-IP_pe	chr10	47498703	47499084	382	47498945	20	6.94658	3.44067
Nc-1-IP_pe	chr10	47523833	47524111	279	47523968	20	6.49719	3.259
Nc-1-IP_pe	chr10	47535923	47536356	434	47536001	12	2.87625	2.25568
Nc-1-IP_pe	chr10	47544741	47545304	564	47545212	16	5.82808	3.34463
Nc-1-IP_pe	chr10	47553234	47553982	749	47553425	32	12.878	4.41117
Nc-1-IP_pe	chr10	47558264	47558544	281	47558404	15	4.40338	2.77622
Nc-1-IP_pe	chr10	47564006	47564300	295	47564127	24	8.53898	3.68518
Nc-1-IP_pe	chr10	47564941	47565376	436	47565209	22	5.13472	2.62042
Nc-1-IP_pe	chr10	47565657	47565991	335	47565758	22	3.82136	2.18497
Nc-1-IP_pe	chr10	47566334	47567555	1222	47566672	25	10.9652	4.51135
Nc-1-IP_pe	chr10	47568080	47568350	271	47568150	16	5.96945	3.41115
Nc-1-IP_pe	chr10	47568650	47569063	414	47568832	17	6.5771	3.58692
Nc-1-IP_pe	chr10	47572387	47572701	315	47572538	17	7.52577	4.03901
Nc-1-IP_pe	chr10	47580029	47580332	304	47580111	17	6.82252	3.70166
Nc-1-IP_pe	chr10	47582913	47583605	693	47583102	19	5.51632	2.94814
Nc-1-IP_pe	chr10	47584817	47585137	321	47585124	13	3.67377	2.58159
Nc-1-IP_pe	chr10	47586018	47586395	378	47586232	17	5.9911	3.31919
Nc-1-IP_pe	chr10	47592247	47593072	826	47592426	28	9.63986	3.71574
Nc-1-IP_pe	chr10	47625361	47625653	293	47625493	19	6.78661	3.47027
Nc-1-IP_pe	chr10	47649482	47650044	563	47649556	18	6.63038	3.50359
Nc-1-IP_pe	chr10	47655715	47656018	304	47655866	26	6.81227	2.94581
Nc-1-IP_pe	chr10	47659071	47660016	946	47659477	23	8.92223	3.93219

Nc-1-IP_pe	chr10	47680365	47680677	313	47680522	22	8.9597	4.05907
Nc-1-IP_pe	chr10	47705988	47706273	286	47706128	26	5.29477	2.48465
Nc-1-IP_pe	chr10	47727780	47728101	322	47727978	17	5.35342	3.03768
Nc-1-IP_pe	chr10	47735838	47736179	342	47736129	16	5.82808	3.34463
Nc-1-IP_pe	chr10	47741814	47742076	263	47741963	14	5.95264	3.63357
Nc-1-IP_pe	chr10	47746003	47746600	598	47746076	18	6.15736	3.29676
Nc-1-IP_pe	chr10	47748431	47748708	278	47748601	14	3.00733	2.21111
Nc-1-IP_pe	chr10	47752836	47753357	522	47752899	15	3.90248	2.55134
Nc-1-IP_pe	chr10	47753737	47754895	1159	47754259	26	5.29477	2.48465
Nc-1-IP_pe	chr10	47755870	47756251	382	47756099	26	7.31838	3.10673
Nc-1-IP_pe	chr10	47762128	47763190	1063	47762585	30	7.90234	3.04332
Nc-1-IP_pe	chr10	47763986	47764267	282	47764222	19	4.23334	2.45554
Nc-1-IP_pe	chr10	47765276	47765616	341	47765476	25	8.97807	3.75291
Nc-1-IP_pe	chr10	47767400	47768125	726	47767497	28	7.90909	3.16402
Nc-1-IP_pe	chr10	47871630	47871908	279	47871778	16	4.87846	2.91138
Nc-1-IP_pe	chr10	47881989	47883231	1243	47882714	21	7.10961	3.41419
Nc-1-IP_pe	chr10	47885765	47886019	255	47885891	14	5.19764	3.24624
Nc-1-IP_pe	chr10	47889285	47889726	442	47889444	18	5.33197	2.94862
Nc-1-IP_pe	chr10	47892591	47893342	752	47893199	16	5.50606	3.19508
Nc-1-IP_pe	chr10	47894828	47895324	497	47894918	17	3.11196	2.12137
Nc-1-IP_pe	chr10	47915120	47915476	357	47915398	15	4.50738	2.8237
Nc-1-IP_pe	chr10	47917876	47918454	579	47918031	16	4.59754	2.78762
Nc-1-IP_pe	chr10	47918846	47919961	1116	47919446	17	4.44438	2.65333
Nc-1-IP_pe	chr10	47922255	47922574	320	47922527	21	4.0961	2.31438
Nc-1-IP_pe	chr10	47924809	47925131	323	47925036	16	6.58466	3.7068
Nc-1-IP_pe	chr10	47929777	47930094	318	47929912	17	6.51104	3.5563
Nc-1-IP_pe	chr10	47938668	47938989	322	47938734	12	4.51041	3.09723
Nc-1-IP_pe	chr10	47940404	47940743	340	47940425	15	4.90347	3.00714
Nc-1-IP_pe	chr10	47945040	47945425	386	47945314	13	3.35769	2.42919
Nc-1-IP_pe	chr10	47946810	47947202	393	47946980	13	5.29187	3.40559
Nc-1-IP_pe	chr10	47971637	47972538	902	47971976	17	4.44438	2.65333
Nc-1-IP_pe	chr10	47987239	47987574	336	47987451	22	6.07094	2.94697
Nc-1-IP_pe	chr10	47988370	47988648	279	47988393	16	4.25218	2.63809
Nc-1-IP_pe	chr10	47989774	47990132	359	47990027	18	6.76888	3.56519
Nc-1-IP_pe	chr10	47992202	47992826	625	47992422	19	7.2357	3.66372
Nc-1-IP_pe	chr10	47995355	47995647	293	47995546	18	6.15736	3.29676
Nc-1-IP_pe	chr10	47996212	47996489	278	47996241	12	3.26128	2.44727
Nc-1-IP_pe	chr10	47999551	48000283	733	48000119	21	7.10914	3.414
Nc-1-IP_pe	chr10	48001863	48002439	577	48002358	24	8.4054	3.63577
Nc-1-IP_pe	chr10	48010154	48010516	363	48010337	21	9.98999	4.63886
Nc-1-IP_pe	chr10	48016063	48016378	316	48016226	17	5.54967	3.12324
Nc-1-IP_pe	chr10	48031242	48032021	780	48031291	16	3.65575	2.38624
Nc-1-IP_pe	chr10	48040366	48040894	529	48040725	20	9.67226	4.6402
Nc-1-IP_pe	chr10	48075755	48076119	365	48076029	13	5.41476	3.47126
Nc-1-IP_pe	chr10	48077407	48078042	636	48077549	15	6.78905	3.93887

Nc-1-IP_pe	chr10	48139893	48140526	634	48140343	26	10.439	4.19014
Nc-1-IP_pe	chr10	48175735	48176305	571	48175966	14	6.42818	3.88572
Nc-1-IP_pe	chr10	48189338	48189691	354	48189544	17	7.85637	4.20196
Nc-1-IP_pe	chr10	48223845	48224268	424	48223936	9	4.20361	3.25317
Nc-1-IP_pe	chr10	48235350	48235658	309	48235416	12	5.04681	3.39039
Nc-1-IP_pe	chr10	48239396	48239674	279	48239605	11	4.46586	3.17976
Nc-1-IP_pe	chr10	48246205	48246700	496	48246649	12	4.28158	2.97474
Nc-1-IP_pe	chr10	48288888	48289530	643	48289002	18	6.63038	3.50359
Nc-1-IP_pe	chr10	48303023	48303434	412	48303200	13	5.36853	3.44651
Nc-1-IP_pe	chr10	48334445	48334852	408	48334517	10	3.03453	2.46828
Nc-1-IP_pe	chr10	48346099	48347169	1071	48346465	14	6.61011	3.98382
Nc-1-IP_pe	chr10	48441728	48442252	525	48441785	15	4.7819	2.95039
Nc-1-IP_pe	chr10	48445831	48446104	274	48446035	15	5.20142	3.14789
Nc-1-IP_pe	chr10	48447530	48447877	348	48447619	13	4.78908	3.14145
Nc-1-IP_pe	chr10	48449301	48450036	736	48449863	18	8.10208	4.1823
Nc-1-IP_pe	chr10	48450407	48450673	267	48450466	15	5.22925	3.16116
Nc-1-IP_pe	chr10	48451322	48452005	684	48451416	15	6.05107	3.5626
Nc-1-IP_pe	chr10	48454175	48454508	334	48454235	14	4.60011	2.95114
Nc-1-IP_pe	chr10	48455518	48455826	309	48455672	16	5.98198	3.41707
Nc-1-IP_pe	chr10	48457210	48457690	481	48457275	11	3.71541	2.7623
Nc-1-IP_pe	chr10	48458811	48459240	430	48458922	16	4.25252	2.63824
Nc-1-IP_pe	chr10	48463903	48464529	627	48464330	17	5.54967	3.12324
Nc-1-IP_pe	chr10	48464952	48465596	645	48465340	14	5.03085	3.16286
Nc-1-IP_pe	chr10	48466059	48466962	904	48466210	19	6.32622	3.27682
Nc-1-IP_pe	chr10	48468956	48469370	415	48469288	11	2.96397	2.36091
Nc-1-IP_pe	chr10	48471015	48471346	332	48471083	13	3.35769	2.42919
Nc-1-IP_pe	chr10	48471906	48472378	473	48471958	12	4.24064	2.95299
Nc-1-IP_pe	chr10	48479235	48479765	531	48479445	13	5.86991	3.71823
Nc-1-IP_pe	chr10	48480576	48481432	857	48481286	12	6.19862	4.04709
Nc-1-IP_pe	chr10	48500605	48500863	259	48500676	11	4.07936	2.96267
Nc-1-IP_pe	chr10	48508881	48509683	803	48509577	15	5.66913	3.37372
Nc-1-IP_pe	chr10	48534036	48534530	495	48534428	12	4.70082	3.20033
Nc-1-IP_pe	chr10	48535870	48536375	506	48536259	15	4.06016	2.62146
Nc-1-IP_pe	chr10	48537710	48538132	423	48537835	15	6.24734	3.66122
Nc-1-IP_pe	chr10	48545545	48545839	295	48545792	11	3.68532	2.74592
Nc-1-IP_pe	chr10	48548602	48548926	325	48548627	11	3.64839	2.72584
Nc-1-IP_pe	chr10	48552228	48552651	424	48552604	14	3.55308	2.45762
Nc-1-IP_pe	chr10	48556133	48556455	323	48556268	18	7.93853	4.10423
Nc-1-IP_pe	chr10	48567293	48567631	339	48567603	10	3.4604	2.70797
Nc-1-IP_pe	chr10	48574524	48574922	399	48574695	16	10.0682	5.56166
Nc-1-IP_pe	chr10	48582966	48583248	283	48583124	14	3.86727	2.6027
Nc-1-IP_pe	chr10	48586892	48587364	473	48587027	19	11.4137	5.69049
Nc-1-IP_pe	chr10	48588687	48589089	403	48588892	17	9.26676	4.9276
Nc-1-IP_pe	chr10	48590217	48590552	336	48590306	17	9.53612	5.07157
Nc-1-IP_pe	chr10	48594505	48594807	303	48594575	9	3.12089	2.59629

Nc-1-IP_pe	chr10	48598429	48598782	354	48598646	10	3.08907	2.49869
Nc-1-IP_pe	chr10	48601034	48601319	286	48601082	13	4.41954	2.952
Nc-1-IP_pe	chr10	48603758	48604175	418	48603895	12	5.31851	3.54206
Nc-1-IP_pe	chr10	48604463	48605306	844	48604721	17	8.58498	4.57078
Nc-1-IP_pe	chr10	48608247	48608723	477	48608499	19	9.92774	4.92358
Nc-1-IP_pe	chr10	48609953	48610379	427	48610031	11	4.47871	3.18705
Nc-1-IP_pe	chr10	48620853	48621118	266	48620986	11	6.00992	4.08766
Nc-1-IP_pe	chr10	48635183	48635500	318	48635465	13	5.36853	3.44651
Nc-1-IP_pe	chr10	48655403	48655746	344	48655486	17	5.14803	2.94914
Nc-1-IP_pe	chr10	48656489	48657061	573	48657011	16	3.94146	2.50592
Nc-1-IP_pe	chr10	48658570	48659257	688	48658762	16	6.1093	3.47747
Nc-1-IP_pe	chr10	48665347	48665851	505	48665677	12	6.50787	4.22912
Nc-1-IP_pe	chr10	48668114	48668535	422	48668296	20	11.7013	5.64235
Nc-1-IP_pe	chr10	48682348	48682605	258	48682471	12	5.23082	3.49289
Nc-1-IP_pe	chr10	48684480	48685525	1046	48685414	12	5.84943	3.84431
Nc-1-IP_pe	chr10	48687751	48688061	311	48687901	13	7.29853	4.52898
Nc-1-IP_pe	chr10	48692668	48693004	337	48692763	11	4.88957	3.42271
Nc-1-IP_pe	chr10	48699041	48699533	493	48699154	11	4.54404	3.2242
Nc-1-IP_pe	chr10	48719822	48720304	483	48719950	15	6.19428	3.63445
Nc-1-IP_pe	chr10	48723584	48724189	606	48723658	13	5.42865	3.47872
Nc-1-IP_pe	chr10	48728160	48728759	600	48728644	11	3.28475	2.53029
Nc-1-IP_pe	chr10	48743095	48743708	614	48743306	16	5.37454	3.13479
Nc-1-IP_pe	chr10	48745698	48746096	399	48745854	18	9.82901	5.04617
Nc-1-IP_pe	chr10	48762345	48762657	313	48762425	11	5.13077	3.56323
Nc-1-IP_pe	chr10	48767855	48768419	565	48767954	11	3.64839	2.72584
Nc-1-IP_pe	chr10	48774218	48774738	521	48774473	15	6.78905	3.93887
Nc-1-IP_pe	chr10	48775071	48775624	554	48775478	10	3.4604	2.70797
Nc-1-IP_pe	chr10	48778120	48778501	382	48778379	13	5.71736	3.63481
Nc-1-IP_pe	chr10	48794104	48794392	289	48794220	17	5.9911	3.31919
Nc-1-IP_pe	chr10	48811643	48812165	523	48812010	11	5.03835	3.5092
Nc-1-IP_pe	chr10	48817120	48817409	290	48817216	16	8.39046	4.63193
Nc-1-IP_pe	chr10	48830232	48830848	617	48830803	13	5.36853	3.44651
Nc-1-IP_pe	chr10	48848078	48848869	792	48848137	12	6.0167	3.94107
Nc-1-IP_pe	chr10	48851568	48852048	481	48851686	8	3.53695	2.94441
Nc-1-IP_pe	chr10	48880303	48880721	419	48880466	12	5.01934	3.37518
Nc-1-IP_pe	chr10	48882052	48882363	312	48882180	14	5.03085	3.16286
Nc-1-IP_pe	chr10	48905575	48906070	496	48905600	13	4.41954	2.952
Nc-1-IP_pe	chr10	48907903	48908347	445	48908009	10	4.77495	3.48064
Nc-1-IP_pe	chr10	48923829	48924142	314	48924030	15	6.78905	3.93887
Nc-1-IP_pe	chr10	48926913	48927359	447	48927264	12	5.84943	3.84431
Nc-1-IP_pe	chr10	48933409	48934125	717	48933981	14	6.44441	3.89444
Nc-1-IP_pe	chr10	48938364	48938651	288	48938562	9	3.29405	2.69924
Nc-1-IP_pe	chr10	48955516	48955889	374	48955776	12	5.40861	3.59282
Nc-1-IP_pe	chr10	48968040	48968449	410	48968073	11	3.28475	2.53029
Nc-1-IP_pe	chr10	48971678	48971972	295	48971841	16	9.24948	5.10026

Nc-1-IP_pe	chr10	48974924	48975458	535	48975060	18	10.7376	5.5283
Nc-1-IP_pe	chr10	48976636	48976946	311	48976789	12	5.87693	3.86017
Nc-1-IP_pe	chr10	49000042	49000603	562	49000419	12	5.01934	3.37518
Nc-1-IP_pe	chr10	49001627	49002079	453	49001702	11	3.58722	2.69267
Nc-1-IP_pe	chr10	49025386	49025735	350	49025478	11	4.54404	3.2242
Nc-1-IP_pe	chr10	49026778	49027084	307	49026902	16	3.65575	2.38624
Nc-1-IP_pe	chr10	49029697	49030041	345	49029916	14	5.03085	3.16286
Nc-1-IP_pe	chr10	49057377	49057876	500	49057769	12	4.24064	2.95299
Nc-1-IP_pe	chr10	49058387	49059534	1148	49058830	15	6.69889	3.8921
Nc-1-IP_pe	chr10	49069219	49069543	325	49069521	8	3.4979	2.91965
Nc-1-IP_pe	chr10	49075012	49075918	907	49075824	13	5.36853	3.44651
Nc-1-IP_pe	chr10	49077031	49077364	334	49077203	12	5.89413	3.8701
Nc-1-IP_pe	chr10	49090777	49092237	1461	49091701	15	8.61047	4.928
Nc-1-IP_pe	chr10	49092834	49093316	483	49092843	10	3.08907	2.49869
Nc-1-IP_pe	chr10	49096728	49097300	573	49097240	10	3.60969	2.79325
Nc-1-IP_pe	chr10	49103898	49104741	844	49104178	11	5.10577	3.5486
Nc-1-IP_pe	chr10	49107270	49108097	828	49107748	16	9.11744	5.02718
Nc-1-IP_pe	chr10	49110715	49111370	656	49110832	13	7.52042	4.65922
Nc-1-IP_pe	chr10	49114833	49115222	390	49115021	10	5.10552	3.682
Nc-1-IP_pe	chr10	49115851	49116164	314	49115968	12	6.82053	4.41531
Nc-1-IP_pe	chr10	49131309	49131593	285	49131494	12	5.25962	3.50902
Nc-1-IP_pe	chr10	49152918	49153403	486	49153235	13	7.42513	4.60316
Nc-1-IP_pe	chr10	49161532	49161880	349	49161711	8	3.0402	2.6319
Nc-1-IP_pe	chr10	49167270	49167565	296	49167552	12	3.83688	2.74114
Nc-1-IP_pe	chr10	49176788	49177219	432	49177159	11	4.06403	2.95415
Nc-1-IP_pe	chr10	49177776	49178355	580	49177906	13	5.71736	3.63481
Nc-1-IP_pe	chr10	49180061	49180356	296	49180138	16	6.37501	3.60492
Nc-1-IP_pe	chr10	49225413	49225789	377	49225575	11	4.87504	3.4143
Nc-1-IP_pe	chr10	49226568	49226839	272	49226674	11	4.06403	2.95415
Nc-1-IP_pe	chr10	49227878	49228174	297	49227951	11	4.54404	3.2242
Nc-1-IP_pe	chr10	49262458	49263078	621	49262903	11	5.10577	3.5486
Nc-1-IP_pe	chr10	49270218	49270484	267	49270316	14	6.13058	3.7272
Nc-1-IP_pe	chr10	49281427	49281775	349	49281606	17	9.99923	5.32289
Nc-1-IP_pe	chr10	49283869	49284803	935	49284074	12	6.0167	3.94107
Nc-1-IP_pe	chr10	49286718	49287091	374	49286851	15	4.40338	2.77622
Nc-1-IP_pe	chr10	49293222	49293700	479	49293608	14	3.55308	2.45762
Nc-1-IP_pe	chr10	49295303	49295638	336	49295504	13	4.02562	2.7544
Nc-1-IP_pe	chr10	49301823	49302198	376	49301830	10	3.21261	2.56787
Nc-1-IP_pe	chr10	49308318	49309120	803	49309013	14	5.82389	3.56638
Nc-1-IP_pe	chr10	49311132	49311457	326	49311397	10	3.08907	2.49869
Nc-1-IP_pe	chr10	49318148	49318458	311	49318252	14	6.06519	3.69269
Nc-1-IP_pe	chr10	49318777	49319048	272	49318889	11	4.54404	3.2242
Nc-1-IP_pe	chr10	49322517	49322826	310	49322661	11	4.61014	3.26192
Nc-1-IP_pe	chr10	49324245	49324876	632	49324406	11	5.1152	3.55411
Nc-1-IP_pe	chr10	49328597	49328998	402	49328619	9	3.81807	3.01579

Nc-1-IP_pe	chr10	49348392	49348685	294	49348524	13	6.54342	4.09407
Nc-1-IP_pe	chr10	49349412	49349877	466	49349775	14	6.28469	3.80898
Nc-1-IP_pe	chr10	49352723	49353242	520	49352884	12	5.70894	3.7636
Nc-1-IP_pe	chr10	49360302	49361134	833	49360433	13	5.9487	3.76157
Nc-1-IP_pe	chr10	49365618	49366307	690	49366174	19	9.92774	4.92358
Nc-1-IP_pe	chr10	49369873	49370555	683	49370059	18	10.3148	5.30169
Nc-1-IP_pe	chr10	49381331	49381622	292	49381500	16	5.82808	3.34463
Nc-1-IP_pe	chr10	49382208	49382525	318	49382464	13	3.35769	2.42919
Nc-1-IP_pe	chr10	49385295	49385569	275	49385377	13	4.41954	2.952
Nc-1-IP_pe	chr10	49386409	49386934	526	49386438	14	3.86727	2.6027
Nc-1-IP_pe	chr10	49394912	49395273	362	49395206	14	4.73128	3.01506
Nc-1-IP_pe	chr10	49397207	49397724	518	49397311	11	3.64839	2.72584
Nc-1-IP_pe	chr10	49398262	49398583	322	49398407	15	6.69889	3.8921
Nc-1-IP_pe	chr10	49400727	49401018	292	49400757	10	3.4604	2.70797
Nc-1-IP_pe	chr10	49402342	49402646	305	49402475	12	5.48538	3.63624
Nc-1-IP_pe	chr10	49412626	49413126	501	49412953	20	7.97282	3.87239
Nc-1-IP_pe	chr10	49416908	49418081	1174	49417039	17	7.02067	3.79543
Nc-1-IP_pe	chr10	49424944	49425390	447	49425209	13	4.67336	3.0817
Nc-1-IP_pe	chr10	49428517	49428810	294	49428627	16	4.25252	2.63824
Nc-1-IP_pe	chr10	49439009	49439529	521	49439281	11	3.64839	2.72584
Nc-1-IP_pe	chr10	49444045	49444361	317	49444200	14	5.43414	3.36584
Nc-1-IP_pe	chr10	49476551	49477065	515	49476812	21	9.98999	4.63886
Nc-1-IP_pe	chr10	49520198	49520803	606	49520739	14	5.03085	3.16286
Nc-1-IP_pe	chr10	49524139	49524504	366	49524268	17	9.94011	5.29055
Nc-1-IP_pe	chr10	49537746	49538292	547	49538115	13	3.95529	2.71958
Nc-1-IP_pe	chr10	49538684	49539143	460	49538957	20	3.24562	2.0616
Nc-1-IP_pe	chr10	49556496	49556801	306	49556706	14	5.51541	3.4073
Nc-1-IP_pe	chr10	49576160	49576459	300	49576165	13	4.41954	2.952
Nc-1-IP_pe	chr10	49591895	49592215	321	49592129	12	4.81979	3.26529
Nc-1-IP_pe	chr10	49592792	49593056	265	49592966	16	5.50606	3.19508
Nc-1-IP_pe	chr10	49594983	49595268	286	49595178	11	3.60658	2.70315
Nc-1-IP_pe	chr10	49597551	49599218	1668	49597592	14	4.99738	3.14622
Nc-1-IP_pe	chr10	49607054	49607655	602	49607326	16	4.96462	2.94973
Nc-1-IP_pe	chr10	49609078	49609463	386	49609422	11	4.01189	2.92523
Nc-1-IP_pe	chr10	49610312	49611009	698	49610544	13	4.56123	3.02417
Nc-1-IP_pe	chr10	49614588	49615050	463	49614935	16	5.82808	3.34463
Nc-1-IP_pe	chr10	49620198	49620734	537	49620720	11	4.00079	2.91908
Nc-1-IP_pe	chr10	49622660	49623139	480	49622893	19	10.0197	4.96959
Nc-1-IP_pe	chr10	49631665	49632977	1313	49632882	16	4.59224	2.7853
Nc-1-IP_pe	chr10	49634237	49634632	396	49634536	17	7.02067	3.79543
Nc-1-IP_pe	chr10	49643822	49644241	420	49643866	17	5.4568	3.08264
Nc-1-IP_pe	chr10	49652027	49652959	933	49652220	15	4.7819	2.95039
Nc-1-IP_pe	chr10	49679293	49679841	549	49679469	17	11.4763	6.15376
Nc-1-IP_pe	chr10	49701679	49702195	517	49701709	10	3.4604	2.70797
Nc-1-IP_pe	chr10	49710654	49711105	452	49710733	11	4.54404	3.2242

Nc-1-IP_pe	chr10	49724956	49725239	284	49725134	12	6.25484	4.08002
Nc-1-IP_pe	chr10	49734192	49734469	278	49734401	12	3.77112	2.7071
Nc-1-IP_pe	chr10	49736773	49737315	543	49737099	17	5.54967	3.12324
Nc-1-IP_pe	chr10	49737958	49739130	1173	49738474	15	5.5343	3.308
Nc-1-IP_pe	chr10	49740612	49740976	365	49740882	13	3.67377	2.58159
Nc-1-IP_pe	chr10	49748706	49748968	263	49748796	17	7.35238	3.95466
Nc-1-IP_pe	chr10	49749802	49750129	328	49750073	11	3.28475	2.53029
Nc-1-IP_pe	chr10	49752408	49752769	362	49752578	16	4.25252	2.63824
Nc-1-IP_pe	chr10	49758243	49758827	585	49758335	14	5.51541	3.4073
Nc-1-IP_pe	chr10	49760995	49761323	329	49761106	12	4.39405	3.03475
Nc-1-IP_pe	chr10	49762323	49762649	327	49762400	16	6.65665	3.74206
Nc-1-IP_pe	chr10	49828368	49828649	282	49828537	12	6.71369	4.35145
Nc-1-IP_pe	chr10	49941340	49942345	1006	49942069	24	11.1	4.69865
Nc-1-IP_pe	chr10	49969769	49970801	1033	49970372	19	4.5193	2.56258
Nc-1-IP_pe	chr10	49986765	49987066	302	49986952	22	6.84408	3.22845
Nc-1-IP_pe	chr10	49988243	49989068	826	49988613	23	9.9969	4.3688
Nc-1-IP_pe	chr10	49992790	49993064	275	49992929	24	8.05897	3.50918
Nc-1-IP_pe	chr10	50009898	50010200	303	50010083	22	8.81204	3.99809
Nc-1-IP_pe	chr10	50011076	50011411	336	50011212	10	3.08907	2.49869
Nc-1-IP_pe	chr10	50012315	50012596	282	50012410	14	6.02589	3.672
Nc-1-IP_pe	chr10	50016344	50016783	440	50016702	15	6.5434	3.81197
Nc-1-IP_pe	chr10	50018052	50018676	625	50018489	13	4.45253	2.96876
Nc-1-IP_pe	chr10	50020177	50020793	617	50020651	15	6.3782	3.72756
Nc-1-IP_pe	chr10	50023885	50024209	325	50024006	14	3.86727	2.6027
Nc-1-IP_pe	chr10	50026961	50027420	460	50027177	17	6.78076	3.68203
Nc-1-IP_pe	chr10	50029387	50029662	276	50029653	14	3.33731	2.35937
Nc-1-IP_pe	chr10	50043913	50044190	278	50044113	12	6.03452	3.95142
Nc-1-IP_pe	chr10	50067620	50068479	860	50068000	30	13.3853	4.81064
Nc-1-IP_pe	chr10	50082410	50082787	378	50082559	10	4.46067	3.29163
Nc-1-IP_pe	chr10	50092378	50092674	297	50092523	17	6.47874	3.54137
Nc-1-IP_pe	chr10	50099383	50099905	523	50099770	13	5.36853	3.44651
Nc-1-IP_pe	chr10	50129479	50129756	278	50129650	11	4.23076	3.04719
Nc-1-IP_pe	chr10	50130539	50130796	258	50130735	11	4.38975	3.13666
Nc-1-IP_pe	chr10	50134155	50134457	303	50134291	13	6.72991	4.20021
Nc-1-IP_pe	chr10	50143622	50144091	470	50143735	10	3.89064	2.95552
Nc-1-IP_pe	chr10	50147079	50147346	268	50147118	9	3.54889	2.85226
Nc-1-IP_pe	chr10	50152227	50153090	864	50152602	19	7.84735	3.93486
Nc-1-IP_pe	chr10	50201035	50201603	569	50201465	10	4.93609	3.57849
Nc-1-IP_pe	chr10	50227827	50228087	261	50227930	10	4.90322	3.55848
Nc-1-IP_pe	chr10	50294253	50294973	721	50294869	14	5.51541	3.4073
Nc-1-IP_pe	chr10	50343773	50344159	387	50343861	14	4.21449	2.76599
Nc-1-IP_pe	chr10	50350511	50351058	548	50350957	15	6.19428	3.63445
Nc-1-IP_pe	chr10	50418063	50418328	266	50418210	15	6.78905	3.93887
Nc-1-IP_pe	chr10	50421497	50421804	308	50421633	10	5.00283	3.61918
Nc-1-IP_pe	chr10	50476703	50477218	516	50477135	12	5.77527	3.80164

Nc-1-IP_pe	chr10	50480495	50480806	312	50480588	16	6.33285	3.58457
Nc-1-IP_pe	chr10	50556277	50556582	306	50556418	17	9.09431	4.8363
Nc-1-IP_pe	chr10	50575985	50576456	472	50576406	10	5.05378	3.65032
Nc-1-IP_pe	chr10	50611442	50611764	323	50611528	15	4.06016	2.62146
Nc-1-IP_pe	chr10	50622879	50623924	1046	50623543	19	7.05262	3.58428
Nc-1-IP_pe	chr10	50624894	50625178	285	50625035	18	5.42854	2.98853
Nc-1-IP_pe	chr10	50650348	50650758	411	50650535	13	5.36853	3.44651
Nc-1-IP_pe	chr10	50658308	50659011	704	50658483	17	6.64406	3.61808
Nc-1-IP_pe	chr10	50660261	50660754	494	50660390	16	5.50606	3.19508
Nc-1-IP_pe	chr10	50678802	50679653	852	50678953	17	5.54967	3.12324
Nc-1-IP_pe	chr10	50682041	50682474	434	50682295	12	3.66916	2.65455
Nc-1-IP_pe	chr10	50683445	50683790	346	50683527	12	3.66006	2.64988
Nc-1-IP_pe	chr10	50687453	50688104	652	50687629	16	6.8793	3.85196
Nc-1-IP_pe	chr10	50695677	50695948	272	50695716	9	2.89249	2.46179
Nc-1-IP_pe	chr10	50696230	50696655	426	50696334	14	6.96097	4.17548
Nc-1-IP_pe	chr10	50699130	50699403	274	50699347	15	4.40338	2.77622
Nc-1-IP_pe	chr10	50702131	50702390	260	50702191	15	4.40338	2.77622
Nc-1-IP_pe	chr10	50709831	50710206	376	50710094	21	5.88587	2.94733
Nc-1-IP_pe	chr10	50738098	50738423	326	50738316	11	4.74697	3.34038
Nc-1-IP_pe	chr10	50739316	50740230	915	50739771	19	3.96692	2.35708
Nc-1-IP_pe	chr10	50787940	50788337	398	50788275	11	4.35236	3.11555
Nc-1-IP_pe	chr10	50794933	50795414	482	50795110	10	5.23357	3.76063
Nc-1-IP_pe	chr10	50923315	50923774	460	50923558	15	8.84797	5.06275
Nc-1-IP_pe	chr10	51163514	51164198	685	51163686	15	7.37761	4.24941
Nc-1-IP_pe	chr10	51205352	51205666	315	51205404	13	4.86362	3.18015
Nc-1-IP_pe	chr10	51377747	51378023	277	51377844	11	6.03439	4.10251
Nc-1-IP_pe	chr10	51392545	51392858	314	51392741	16	9.24948	5.10026
Nc-1-IP_pe	chr10	51515818	51516253	436	51515973	12	3.47963	2.55766
Nc-1-IP_pe	chr10	51604648	51605372	725	51604763	11	6.03439	4.10251
Nc-1-IP_pe	chr10	51616372	51616997	626	51616488	13	5.36853	3.44651
Nc-1-IP_pe	chr10	51699345	51699911	567	51699769	16	6.51374	3.67221
Nc-1-IP_pe	chr10	51711131	51711415	285	51711331	12	6.86906	4.44438
Nc-1-IP_pe	chr10	51726813	51727172	360	51727060	12	6.86906	4.44438
Nc-1-IP_pe	chr10	51756451	51756774	324	51756678	16	10.5028	5.81189
Nc-1-IP_pe	chr10	51760706	51760976	271	51760773	9	4.46937	3.41876
Nc-1-IP_pe	chr10	51824608	51824871	264	51824730	14	8.63073	5.12814
Nc-1-IP_pe	chr10	51946754	51947056	303	51946868	14	6.06519	3.69269
Nc-1-IP_pe	chr10	52036840	52037249	410	52037065	12	4.70082	3.20033
Nc-1-IP_pe	chr10	52054893	52055180	288	52055013	14	6.69566	4.03025
Nc-1-IP_pe	chr10	52110020	52110314	295	52110248	13	5.15976	3.33548
Nc-1-IP_pe	chr10	52180645	52181173	529	52180813	10	4.99888	3.61677
Nc-1-IP_pe	chr10	52183232	52183748	517	52183430	14	4.60011	2.95114
Nc-1-IP_pe	chr10	52196014	52196367	354	52196253	12	6.86906	4.44438
Nc-1-IP_pe	chr10	52207069	52207790	722	52207273	13	4.78908	3.14145
Nc-1-IP_pe	chr10	52278582	52278864	283	52278715	11	4.06403	2.95415

Nc-1-IP_pe	chr10	52365561	52366221	661	52366068	15	6.19428	3.63445
Nc-1-IP_pe	chr10	52374679	52375705	1027	52375558	16	7.53857	4.18505
Nc-1-IP_pe	chr10	52421308	52421577	270	52421529	10	4.39571	3.25288
Nc-1-IP_pe	chr10	52459574	52459867	294	52459736	12	5.67619	3.74486
Nc-1-IP_pe	chr10	52466610	52466871	262	52466778	11	5.19376	3.60018
Nc-1-IP_pe	chr10	52478691	52478986	296	52478843	16	8.79222	4.84884
Nc-1-IP_pe	chr10	52689259	52689634	376	52689479	15	7.37761	4.24941
Nc-1-IP_pe	chr10	52735666	52736323	658	52736097	14	6.69566	4.03025
Nc-1-IP_pe	chr10	52744036	52744318	283	52744226	13	5.55598	3.54727
Nc-1-IP_pe	chr10	52778428	52778703	276	52778669	13	4.02562	2.7544
Nc-1-IP_pe	chr10	52827819	52828295	477	52828133	11	3.28475	2.53029
Nc-1-IP_pe	chr10	52846155	52846445	291	52846234	13	5.9487	3.76157
Nc-1-IP_pe	chr10	52871437	52871999	563	52871636	11	3.64839	2.72584
Nc-1-IP_pe	chr10	52888585	52888865	281	52888748	15	8.01848	4.59761
Nc-1-IP_pe	chr10	52939114	52939383	270	52939156	9	4.46937	3.41876
Nc-1-IP_pe	chr10	52939815	52940180	366	52940125	14	4.21449	2.76599
Nc-1-IP_pe	chr10	52940516	52940778	263	52940690	15	5.70822	3.39286
Nc-1-IP_pe	chr10	52949898	52950785	888	52950007	12	5.77527	3.80164
Nc-1-IP_pe	chr10	52964622	52965304	683	52964933	15	5.58327	3.33181
Nc-1-IP_pe	chr10	52965629	52966012	384	52965717	12	4.88508	3.30112
Nc-1-IP_pe	chr10	52995658	52996558	901	52996229	12	4.70082	3.20033
Nc-1-IP_pe	chr10	53027083	53027422	340	53027334	16	9.24948	5.10026
Nc-1-IP_pe	chr10	53028684	53028988	305	53028831	12	6.86906	4.44438
Nc-1-IP_pe	chr10	53221782	53222389	608	53222315	12	5.77527	3.80164
Nc-1-IP_pe	chr10	53222962	53223463	502	53223262	15	6.19428	3.63445
Nc-1-IP_pe	chr10	53272104	53272370	267	53272172	12	2.87625	2.25568
Nc-1-IP_pe	chr10	53329995	53330268	274	53330173	15	6.78905	3.93887
Nc-1-IP_pe	chr10	53351715	53352030	316	53351851	12	5.23082	3.49289
Nc-1-IP_pe	chr10	53403371	53403984	614	53403645	15	8.16556	4.67894
Nc-1-IP_pe	chr10	53421935	53422532	598	53422032	9	4.46937	3.41876
Nc-1-IP_pe	chr10	53504263	53504607	345	53504311	9	3.54889	2.85226
Nc-1-IP_pe	chr10	53513294	53513551	258	53513331	10	5.23357	3.76063
Nc-1-IP_pe	chr10	53535293	53535578	286	53535470	14	8.63073	5.12814
Nc-1-IP_pe	chr10	53587933	53588209	277	53588042	9	4.25966	3.28797
Nc-1-IP_pe	chr10	53593724	53594321	598	53594018	15	6.19428	3.63445
Nc-1-IP_pe	chr10	53659978	53660357	380	53660095	10	4.33501	3.21677
Nc-1-IP_pe	chr10	53909830	53910333	504	53910205	12	3.96559	2.80816
Nc-1-IP_pe	chr10	53951773	53952207	435	53951818	10	3.08907	2.49869
Nc-1-IP_pe	chr10	53999675	54000060	386	53999890	12	6.86906	4.44438
Nc-1-IP_pe	chr10	54057037	54057524	488	54057300	10	5.23357	3.76063
Nc-1-IP_pe	chr10	54064497	54065073	577	54064613	14	5.03085	3.16286
Nc-1-IP_pe	chr10	54100055	54100368	314	54100166	12	6.86906	4.44438
Nc-1-IP_pe	chr10	54172057	54172336	280	54172254	12	5.23082	3.49289
Nc-1-IP_pe	chr10	54260549	54260877	329	54260669	11	4.54404	3.2242
Nc-1-IP_pe	chr10	54262712	54263007	296	54262950	12	5.23082	3.49289

Nc-1-IP_pe	chr10	54342161	54342769	609	54342711	9	4.25966	3.28797
Nc-1-IP_pe	chr10	54594710	54595109	400	54594727	10	3.4604	2.70797
Nc-1-IP_pe	chr10	54804067	54804359	293	54804259	11	5.77463	3.94557
Nc-1-IP_pe	chr10	54899534	54899800	267	54899685	11	6.03439	4.10251
Nc-1-IP_pe	chr10	54908176	54908494	319	54908320	12	6.86906	4.44438
Nc-1-IP_pe	chr10	54922735	54923198	464	54922844	12	6.86906	4.44438
Nc-1-IP_pe	chr10	54972313	54972657	345	54972421	9	3.72192	2.95716
Nc-1-IP_pe	chr10	54988875	54989430	556	54989318	11	5.52654	3.79706
Nc-1-IP_pe	chr10	55001363	55001838	476	55001748	12	5.84943	3.84431
Nc-1-IP_pe	chr10	55032995	55033372	378	55033249	11	5.10577	3.5486
Nc-1-IP_pe	chr10	55061775	55062041	267	55061899	11	5.10577	3.5486
Nc-1-IP_pe	chr10	55241014	55241688	675	55241175	11	6.03439	4.10251
Nc-1-IP_pe	chr10	55256429	55256996	568	55256561	10	3.89064	2.95552
Nc-1-IP_pe	chr10	55262296	55262584	289	55262461	11	5.10577	3.5486
Nc-1-IP_pe	chr10	55266471	55266822	352	55266719	16	6.33285	3.58457
Nc-1-IP_pe	chr10	55292546	55292901	356	55292661	16	6.51374	3.67221
Nc-1-IP_pe	chr10	55312604	55312865	262	55312754	14	7.42849	4.43574
Nc-1-IP_pe	chr10	55319542	55319902	361	55319830	11	5.19376	3.60018
Nc-1-IP_pe	chr10	55602047	55602680	634	55602591	12	5.84943	3.84431
Nc-1-IP_pe	chr10	55627499	55627757	259	55627544	10	4.39571	3.25288
Nc-1-IP_pe	chr10	55647353	55647722	370	55647503	11	4.06403	2.95415
Nc-1-IP_pe	chr10	55667181	55667667	487	55667573	14	6.61011	3.98382
Nc-1-IP_pe	chr10	55688418	55688986	569	55688600	14	5.51541	3.4073
Nc-1-IP_pe	chr10	55813478	55813854	377	55813696	12	6.50787	4.22912
Nc-1-IP_pe	chr10	55822424	55822712	289	55822461	8	3.56021	2.95918
Nc-1-IP_pe	chr10	55862254	55862507	254	55862331	13	5.36853	3.44651
Nc-1-IP_pe	chr10	55899818	55900474	657	55900368	18	11.3299	5.852
Nc-1-IP_pe	chr10	56109172	56109718	547	56109489	14	6.69566	4.03025
Nc-1-IP_pe	chr10	56154962	56155554	593	56155321	15	7.46994	4.29894
Nc-1-IP_pe	chr10	56164667	56165024	358	56164926	10	4.07292	3.06202
Nc-1-IP_pe	chr10	56186709	56186984	276	56186837	15	7.95672	4.56362
Nc-1-IP_pe	chr10	56212870	56213322	453	56213032	13	7.7352	4.78626
Nc-1-IP_pe	chr10	56360899	56361162	264	56360988	18	5.72638	3.11298
Nc-1-IP_pe	chr10	56447375	56447956	582	56447880	13	4.41954	2.952
Nc-1-IP_pe	chr10	56465201	56465497	297	56465350	9	4.46937	3.41876
Nc-1-IP_pe	chr10	56466110	56466374	265	56466168	11	4.06403	2.95415
Nc-1-IP_pe	chr10	56563812	56564560	749	56564439	10	4.99888	3.61677
Nc-1-IP_pe	chr10	56646409	56647310	902	56646479	14	6.69566	4.03025
Nc-1-IP_pe	chr10	56801853	56802214	362	56802107	11	5.10577	3.5486
Nc-1-IP_pe	chr10	56823691	56823963	273	56823902	9	3.72192	2.95716
Nc-1-IP_pe	chr10	56949076	56949463	388	56949145	12	4.24064	2.95299
Nc-1-IP_pe	chr10	57032376	57032743	368	57032586	13	7.12853	4.42991
Nc-1-IP_pe	chr10	57057039	57057391	353	57057185	13	6.62436	4.14003
Nc-1-IP_pe	chr10	57064926	57065281	356	57065040	13	7.7352	4.78626
Nc-1-IP_pe	chr10	57094860	57095718	859	57095456	13	5.86991	3.71823

Nc-1-IP_pe	chr10	57165482	57165778	297	57165563	11	5.10577	3.5486
Nc-1-IP_pe	chr10	57207123	57207438	316	57207285	13	5.9487	3.76157
Nc-1-IP_pe	chr10	57249652	57250165	514	57249863	18	10.9042	5.61861
Nc-1-IP_pe	chr10	57301365	57301678	314	57301491	18	12.0366	6.24715
Nc-1-IP_pe	chr10	57321465	57322086	622	57321653	16	6.33285	3.58457
Nc-1-IP_pe	chr10	57483026	57483315	290	57483154	10	5.21145	3.74702
Nc-1-IP_pe	chr10	57506586	57507367	782	57506881	10	4.33501	3.21677
Nc-1-IP_pe	chr10	57585636	57586101	466	57585641	11	4.00079	2.91908
Nc-1-IP_pe	chr10	57591291	57591609	319	57591453	11	5.10577	3.5486
Nc-1-IP_pe	chr10	57832896	57833202	307	57833035	16	9.01614	4.97137
Nc-1-IP_pe	chr10	57908047	57908563	517	57908150	12	6.58417	4.27436
Nc-1-IP_pe	chr10	58026657	58027071	415	58026744	13	6.62436	4.14003
Nc-1-IP_pe	chr10	58077325	58077667	343	58077514	10	3.4604	2.70797
Nc-1-IP_pe	chr10	58093563	58093868	306	58093783	10	5.23357	3.76063
Nc-1-IP_pe	chr10	58164322	58164889	568	58164768	12	5.61153	3.70794
Nc-1-IP_pe	chr10	58197004	58197388	385	58197233	11	4.54404	3.2242
Nc-1-IP_pe	chr10	58200136	58200412	277	58200200	9	4.46937	3.41876
Nc-1-IP_pe	chr10	58257070	58257408	339	58257345	10	5.15807	3.71423
Nc-1-IP_pe	chr10	58267103	58268114	1012	58267257	28	11.2618	4.27481
Nc-1-IP_pe	chr10	58326469	58327230	762	58327108	14	3.26761	2.32785
Nc-1-IP_pe	chr10	58335036	58335516	481	58335231	22	5.72303	2.82387
Nc-1-IP_pe	chr10	58360631	58360896	266	58360797	12	4.70082	3.20033
Nc-1-IP_pe	chr10	58426214	58426582	369	58426246	10	3.4604	2.70797
Nc-1-IP_pe	chr10	58448314	58448619	306	58448430	16	9.49293	5.236
Nc-1-IP_pe	chr10	58479970	58480260	291	58480080	14	8.44792	5.0209
Nc-1-IP_pe	chr10	58513299	58513565	267	58513396	14	4.21449	2.76599
Nc-1-IP_pe	chr10	58588805	58589172	368	58589072	13	4.02562	2.7544
Nc-1-IP_pe	chr10	58589454	58589747	294	58589478	8	3.51085	2.92785
Nc-1-IP_pe	chr10	58592619	58593083	465	58593032	10	4.93609	3.57849
Nc-1-IP_pe	chr10	58615963	58616973	1011	58616055	12	5.61153	3.70794
Nc-1-IP_pe	chr10	58629960	58630258	299	58630077	12	4.70082	3.20033
Nc-1-IP_pe	chr10	58676415	58676873	459	58676763	14	5.51541	3.4073
Nc-1-IP_pe	chr10	58687473	58688276	804	58687880	12	5.84943	3.84431
Nc-1-IP_pe	chr10	58837185	58837545	361	58837341	13	4.02562	2.7544
Nc-1-IP_pe	chr10	58905224	58905558	335	58905415	14	6.78215	4.0774
Nc-1-IP_pe	chr10	58990194	58990550	357	58990319	9	3.66792	2.92434
Nc-1-IP_pe	chr10	59026431	59026745	315	59026581	17	8.58498	4.57078
Nc-1-IP_pe	chr10	59033688	59033965	278	59033904	10	3.89064	2.95552
Nc-1-IP_pe	chr10	59176207	59176500	294	59176410	14	5.51541	3.4073
Nc-1-IP_pe	chr10	59176822	59177241	420	59177094	13	4.02562	2.7544
Nc-1-IP_pe	chr10	59211307	59211602	296	59211430	11	6.03439	4.10251
Nc-1-IP_pe	chr10	59283281	59283546	266	59283286	9	3.27326	2.68684
Nc-1-IP_pe	chr10	59291118	59291400	283	59291168	9	3.72192	2.95716
Nc-1-IP_pe	chr10	59306563	59306932	370	59306726	13	6.62436	4.14003
Nc-1-IP_pe	chr10	59501624	59501880	257	59501725	12	5.23082	3.49289

Nc-1-IP_pe	chr10	59538635	59539319	685	59538751	14	5.51541	3.4073
Nc-1-IP_pe	chr10	59564087	59564595	509	59564182	12	6.58898	4.27722
Nc-1-IP_pe	chr10	59577178	59577438	261	59577350	9	4.20361	3.25317
Nc-1-IP_pe	chr10	59591580	59592005	426	59591732	13	4.41954	2.952
Nc-1-IP_pe	chr10	59593170	59594103	934	59593598	15	9.09658	5.20507
Nc-1-IP_pe	chr10	59646047	59646357	311	59646173	9	3.92293	3.08
Nc-1-IP_pe	chr10	59646613	59647000	388	59646828	14	5.69896	3.50164
Nc-1-IP_pe	chr10	59696164	59696700	537	59696385	18	6.15736	3.29676
Nc-1-IP_pe	chr10	59697134	59697404	271	59697205	10	4.69727	3.4337
Nc-1-IP_pe	chr10	59708668	59710136	1469	59709056	19	8.4638	4.21715
Nc-1-IP_pe	chr10	59713417	59713682	266	59713557	15	5.66913	3.37372
Nc-1-IP_pe	chr10	59743905	59744406	502	59743962	18	6.05776	3.25389
Nc-1-IP_pe	chr10	59749386	59750330	945	59749738	12	6.58417	4.27436
Nc-1-IP_pe	chr10	59792835	59793110	276	59792974	16	6.8987	3.86161
Nc-1-IP_pe	chr10	59841717	59842260	544	59842120	16	8.57707	4.73221
Nc-1-IP_pe	chr10	59848459	59849014	556	59848814	11	6.03439	4.10251
Nc-1-IP_pe	chr10	59882602	59882920	319	59882803	16	9.27336	5.11352
Nc-1-IP_pe	chr10	59888139	59888493	355	59888215	12	3.47963	2.55766
Nc-1-IP_pe	chr10	59905843	59906811	969	59905934	20	7.58419	3.7061
Nc-1-IP_pe	chr10	59938607	59938861	255	59938769	11	4.47871	3.18705
Nc-1-IP_pe	chr10	59941446	59941792	347	59941646	19	8.4638	4.21715
Nc-1-IP_pe	chr10	60009739	60010005	267	60009893	13	6.34721	3.98335
Nc-1-IP_pe	chr10	60030160	60030520	361	60030212	11	5.52654	3.79706
Nc-1-IP_pe	chr10	60046833	60047116	284	60046965	12	5.23082	3.49289
Nc-1-IP_pe	chr10	60068805	60069106	302	60068991	11	4.23076	3.04719
Nc-1-IP_pe	chr10	60069811	60070095	285	60069927	9	4.23325	3.27156
Nc-1-IP_pe	chr10	60081144	60081412	269	60081279	17	6.47874	3.54137
Nc-1-IP_pe	chr10	60095934	60096203	270	60096089	12	6.50787	4.22912
Nc-1-IP_pe	chr10	60128299	60128576	278	60128448	13	6.19316	3.89711
Nc-1-IP_pe	chr10	60179350	60179786	437	60179487	17	9.72281	5.17231
Nc-1-IP_pe	chr10	60316876	60317382	507	60317285	18	4.6358	2.66698
Nc-1-IP_pe	chr10	60352962	60353252	291	60353112	14	5.29552	3.29555
Nc-1-IP_pe	chr10	60373762	60374025	264	60373869	19	8.4638	4.21715
Nc-1-IP_pe	chr10	60406444	60406713	270	60406604	16	7.17668	4.0008
Nc-1-IP_pe	chr10	60647874	60648147	274	60648020	16	7.34063	4.08385
Nc-1-IP_pe	chr10	60689267	60689558	292	60689446	14	5.30793	3.30182
Nc-1-IP_pe	chr10	60693291	60693700	410	60693465	25	8.69165	3.64955
Nc-1-IP_pe	chr10	60705879	60706433	555	60706052	14	6.61011	3.98382
Nc-1-IP_pe	chr10	60732916	60733700	785	60733131	30	7.90234	3.04332
Nc-1-IP_pe	chr10	60733976	60734234	259	60734045	15	4.92438	3.01694
Nc-1-IP_pe	chr10	60752301	60752572	272	60752442	14	5.51541	3.4073
Nc-1-IP_pe	chr10	60761340	60761640	301	60761458	12	4.89828	3.30838
Nc-1-IP_pe	chr10	60775306	60775864	559	60775377	16	6.33285	3.58457
Nc-1-IP_pe	chr10	60778475	60779089	615	60778700	36	10.9314	3.51495
Nc-1-IP_pe	chr10	60840657	60840973	317	60840689	13	4.41954	2.952

Nc-1-IP_pe	chr10	60866018	60866281	264	60866202	18	5.94685	3.20644
Nc-1-IP_pe	chr10	60872682	60873067	386	60872905	13	4.81267	3.15368
Nc-1-IP_pe	chr10	60933431	60933699	269	60933517	14	5.03085	3.16286
Nc-1-IP_pe	chr10	60943607	60944894	1288	60944681	25	7.78348	3.33136
Nc-1-IP_pe	chr10	61001364	61001987	624	61001800	31	11.6262	4.10013
Nc-1-IP_pe	chr10	61039195	61039564	370	61039409	23	7.4429	3.36882
Nc-1-IP_pe	chr10	61040360	61041096	737	61040838	28	9.85935	3.78895
Nc-1-IP_pe	chr10	61114799	61115059	261	61114963	13	4.41954	2.952
Nc-1-IP_pe	chr10	61189453	61189865	413	61189754	15	4.06016	2.62146
Nc-1-IP_pe	chr10	61228527	61228783	257	61228660	15	5.34394	3.21607
Nc-1-IP_pe	chr10	61348024	61348512	489	61348356	16	3.1497	2.1782
Nc-1-IP_pe	chr10	61376564	61377357	794	61377034	29	11.3772	4.21102
Nc-1-IP_pe	chr10	61378840	61379400	561	61379113	18	5.72638	3.11298
Nc-1-IP_pe	chr10	61410314	61410663	350	61410446	17	7.6273	4.08876
Nc-1-IP_pe	chr10	61452274	61453746	1473	61453131	23	8.38947	3.72437
Nc-1-IP_pe	chr10	61520295	61521019	725	61520525	21	6.2632	3.08808
Nc-1-IP_pe	chr10	61539644	61539969	326	61539834	15	8.06007	4.62056
Nc-1-IP_pe	chr10	61567905	61568161	257	61568033	21	8.12272	3.82426
Nc-1-IP_pe	chr10	61569419	61569788	370	61569517	15	3.74763	2.48304
Nc-1-IP_pe	chr10	61620286	61620576	291	61620474	18	10.1956	5.23853
Nc-1-IP_pe	chr10	61662990	61663347	358	61663148	25	7.37317	3.1922
Nc-1-IP_pe	chr10	61676773	61677030	258	61676938	14	6.06519	3.69269
Nc-1-IP_pe	chr10	61683033	61683417	385	61683130	14	5.03085	3.16286
Nc-1-IP_pe	chr10	61750971	61751401	431	61751085	11	2.98297	2.37087
Nc-1-IP_pe	chr10	61797576	61797971	396	61797849	12	3.96559	2.80816
Nc-1-IP_pe	chr10	61839163	61839428	266	61839228	15	4.40338	2.77622
Nc-1-IP_pe	chr10	61860044	61860945	902	61860307	17	5.54967	3.12324
Nc-1-IP_pe	chr10	61867507	61868173	667	61867881	25	9.19615	3.83259
Nc-1-IP_pe	chr10	61868691	61869116	426	61868704	14	3.00733	2.21111
Nc-1-IP_pe	chr10	61870665	61871046	382	61870855	17	7.35238	3.95466
Nc-1-IP_pe	chr10	61879776	61880053	278	61879948	16	5.82808	3.34463
Nc-1-IP_pe	chr10	61889417	61889692	276	61889571	15	4.70031	2.91253
Nc-1-IP_pe	chr10	61896901	61897579	679	61897390	31	9.66075	3.49133
Nc-1-IP_pe	chr10	61903196	61903579	384	61903351	29	8.89222	3.39931
Nc-1-IP_pe	chr10	61908456	61908754	299	61908587	16	7.68537	4.26075
Nc-1-IP_pe	chr10	61913883	61914143	261	61914022	19	6.78661	3.47027
Nc-1-IP_pe	chr10	61942460	61942722	263	61942601	19	6.32622	3.27682
Nc-1-IP_pe	chr10	61955720	61956112	393	61955855	18	8.27052	4.2634
Nc-1-IP_pe	chr10	62015788	62016061	274	62015912	17	4.78101	2.79343
Nc-1-IP_pe	chr10	62022288	62022544	257	62022418	18	7.1522	3.73811
Nc-1-IP_pe	chr10	62092162	62092631	470	62092353	22	5.09279	2.60614
Nc-1-IP_pe	chr10	62092893	62093257	365	62093037	16	5.18047	3.04664
Nc-1-IP_pe	chr10	62104838	62105390	553	62105251	19	5.15822	2.80735
Nc-1-IP_pe	chr10	62106342	62106599	258	62106457	21	6.2632	3.08808
Nc-1-IP_pe	chr10	62125467	62125764	298	62125666	13	6.88507	4.28917

Nc-1-IP_pe	chr10	62155138	62155558	421	62155177	12	4.70082	3.20033
Nc-1-IP_pe	chr10	62165380	62165658	279	62165514	11	4.54404	3.2242
Nc-1-IP_pe	chr10	62242609	62242874	266	62242740	14	8.63073	5.12814
Nc-1-IP_pe	chr10	62280261	62280953	693	62280669	25	9.7397	4.03495
Nc-1-IP_pe	chr10	62281596	62281877	282	62281627	13	4.41954	2.952
Nc-1-IP_pe	chr10	62288070	62288358	289	62288187	10	3.89064	2.95552
Nc-1-IP_pe	chr10	62307024	62307405	382	62307128	12	3.16137	2.39719
Nc-1-IP_pe	chr10	62312975	62313250	276	62313104	18	10.4117	5.35332
Nc-1-IP_pe	chr10	62315511	62315868	358	62315609	11	3.28475	2.53029
Nc-1-IP_pe	chr10	62351569	62351991	423	62351646	12	4.24064	2.95299
Nc-1-IP_pe	chr10	62374039	62375045	1007	62374624	15	6.10627	3.59023
Nc-1-IP_pe	chr10	62382576	62382879	304	62382727	14	5.03085	3.16286
Nc-1-IP_pe	chr10	62466233	62466531	299	62466488	11	3.28475	2.53029
Nc-1-IP_pe	chr10	62527025	62527368	344	62527208	11	5.00808	3.49156
Nc-1-IP_pe	chr10	62581507	62581924	418	62581785	13	5.9487	3.76157
Nc-1-IP_pe	chr10	62588465	62588747	283	62588605	11	6.03439	4.10251
Nc-1-IP_pe	chr10	62624799	62625247	449	62625222	9	3.18064	2.63172
Nc-1-IP_pe	chr10	62633313	62633646	334	62633534	11	4.06403	2.95415
Nc-1-IP_pe	chr10	62657311	62657570	260	62657535	15	4.40338	2.77622
Nc-1-IP_pe	chr10	62677737	62678168	432	62677947	12	5.23082	3.49289
Nc-1-IP_pe	chr10	62769646	62770084	439	62769704	9	3.27326	2.68684
Nc-1-IP_pe	chr10	62790088	62790418	331	62790262	19	7.29128	3.68799
Nc-1-IP_pe	chr10	62804807	62805773	967	62805249	25	5.96578	2.73518
Nc-1-IP_pe	chr10	62812978	62813548	571	62813086	16	7.27441	4.05022
Nc-1-IP_pe	chr10	62815588	62815872	285	62815717	17	6.51104	3.5563
Nc-1-IP_pe	chr10	62816465	62816726	262	62816566	13	4.34711	2.91533
Nc-1-IP_pe	chr10	62817165	62817483	319	62817339	20	6.49719	3.259
Nc-1-IP_pe	chr10	62818528	62818953	426	62818728	23	9.21857	4.05024
Nc-1-IP_pe	chr10	62819420	62819830	411	62819457	13	3.07238	2.29378
Nc-1-IP_pe	chr10	62827031	62827437	407	62827128	11	3.64839	2.72584
Nc-1-IP_pe	chr10	62878798	62879092	295	62878860	12	6.16157	4.02543
Nc-1-IP_pe	chr10	62886620	62887056	437	62886824	17	5.54967	3.12324
Nc-1-IP_pe	chr10	62924399	62924835	437	62924620	16	4.96462	2.94973
Nc-1-IP_pe	chr10	62928823	62929375	553	62929037	12	4.28158	2.97474
Nc-1-IP_pe	chr10	62949422	62949724	303	62949491	13	5.29187	3.40559
Nc-1-IP_pe	chr10	62962347	62962791	445	62962671	11	6.00992	4.08766
Nc-1-IP_pe	chr10	62974129	62974611	483	62974373	13	6.82624	4.25537
Nc-1-IP_pe	chr10	63038062	63038538	477	63038500	10	3.89064	2.95552
Nc-1-IP_pe	chr10	63044557	63044857	301	63044750	15	7.46994	4.29894
Nc-1-IP_pe	chr10	63090186	63090450	265	63090296	13	6.43528	4.03292
Nc-1-IP_pe	chr10	63092424	63092753	330	63092612	13	3.67377	2.58159
Nc-1-IP_pe	chr10	63095661	63096041	381	63095745	9	3.72192	2.95716
Nc-1-IP_pe	chr10	63097764	63098274	511	63098078	11	6.03439	4.10251
Nc-1-IP_pe	chr10	63099107	63099359	253	63099338	9	4.25966	3.28797
Nc-1-IP_pe	chr10	63105272	63105628	357	63105286	10	3.08907	2.49869

Nc-1-IP_pe	chr10	63130716	63131093	378	63130771	12	4.92484	3.32299
Nc-1-IP_pe	chr10	63133518	63134025	508	63133691	31	13.7952	4.83612
Nc-1-IP_pe	chr10	63225532	63225797	266	63225695	20	7.97282	3.87239
Nc-1-IP_pe	chr10	63226489	63226981	493	63226661	13	5.9487	3.76157
Nc-1-IP_pe	chr10	63244744	63245061	318	63244953	16	10.5028	5.81189
Nc-1-IP_pe	chr10	63249166	63249831	666	63249681	10	4.39571	3.25288
Nc-1-IP_pe	chr10	63270224	63270631	408	63270449	8	3.74505	3.07688
Nc-1-IP_pe	chr10	63298751	63299027	277	63298836	11	5.7051	3.90381
Nc-1-IP_pe	chr10	63307788	63308057	270	63307960	14	8.47281	5.03547
Nc-1-IP_pe	chr10	63334719	63334989	271	63334764	12	4.24064	2.95299
Nc-1-IP_pe	chr10	63336861	63337204	344	63336980	17	7.02067	3.79543
Nc-1-IP_pe	chr10	63353643	63354016	374	63353974	9	3.72192	2.95716
Nc-1-IP_pe	chr10	63364069	63364349	281	63364203	17	6.78076	3.68203
Nc-1-IP_pe	chr10	63386426	63386899	474	63386860	13	3.35769	2.42919
Nc-1-IP_pe	chr10	63403746	63404078	333	63403895	15	8.97083	5.13293
Nc-1-IP_pe	chr10	63411399	63412055	657	63411526	13	5.36853	3.44651
Nc-1-IP_pe	chr10	63418815	63419333	519	63418978	10	3.89064	2.95552
Nc-1-IP_pe	chr10	63422132	63422388	257	63422244	10	4.20113	3.13749
Nc-1-IP_pe	chr10	63427132	63427538	407	63427197	12	5.45448	3.61875
Nc-1-IP_pe	chr10	63493944	63494284	341	63494179	17	11.4763	6.15376
Nc-1-IP_pe	chr10	63609167	63609524	358	63609351	17	10.3998	5.544
Nc-1-IP_pe	chr10	63612413	63612676	264	63612585	15	8.26005	4.73146
Nc-1-IP_pe	chr10	63629918	63630223	306	63630189	16	3.86167	2.47233
Nc-1-IP_pe	chr10	63710841	63711141	301	63711111	12	4.06753	2.86157
Nc-1-IP_pe	chr10	63718114	63718572	459	63718274	14	6.13058	3.7272
Nc-1-IP_pe	chr10	63724319	63724571	253	63724419	17	5.54967	3.12324
Nc-1-IP_pe	chr10	63741832	63742160	329	63742032	13	5.36853	3.44651
Nc-1-IP_pe	chr10	63785736	63786130	395	63785916	12	6.27375	4.09111
Nc-1-IP_pe	chr10	63815691	63816087	397	63815747	9	3.32589	2.71826
Nc-1-IP_pe	chr10	63891665	63891964	300	63891815	12	5.23082	3.49289
Nc-1-IP_pe	chr10	63902456	63903324	869	63902536	11	5.10577	3.5486
Nc-1-IP_pe	chr10	63928659	63928970	312	63928766	12	4.70082	3.20033
Nc-1-IP_pe	chr10	63950103	63950785	683	63950276	14	6.69566	4.03025
Nc-1-IP_pe	chr10	63998208	63999104	897	63998603	16	7.53857	4.18505
Nc-1-IP_pe	chr10	64020720	64021443	724	64020931	14	8.20556	4.87976
Nc-1-IP_pe	chr10	64041393	64041857	465	64041759	21	7.58657	3.60451
Nc-1-IP_pe	chr10	64051968	64052453	486	64052032	12	4.70082	3.20033
Nc-1-IP_pe	chr10	64057358	64057647	290	64057495	15	9.55381	5.47001
Nc-1-IP_pe	chr10	64062050	64062344	295	64062218	18	7.73136	4.00629
Nc-1-IP_pe	chr10	64063739	64064005	267	64063923	12	6.42833	4.1821
Nc-1-IP_pe	chr10	64120425	64121023	599	64120731	16	5.82808	3.34463
Nc-1-IP_pe	chr10	64130042	64130343	302	64130132	10	3.4604	2.70797
Nc-1-IP_pe	chr10	64161770	64162027	258	64161960	14	8.63073	5.12814
Nc-1-IP_pe	chr10	64170798	64171164	367	64171012	13	7.7352	4.78626
Nc-1-IP_pe	chr10	64196795	64197538	744	64197428	14	6.06519	3.69269

Nc-1-IP_pe	chr10	64223739	64224063	325	64223815	12	4.1728	2.91706
Nc-1-IP_pe	chr10	64258866	64259317	452	64259221	12	6.12485	4.004
Nc-1-IP_pe	chr10	64273436	64273709	274	64273617	13	7.42513	4.60316
Nc-1-IP_pe	chr10	64464558	64464844	287	64464689	10	5.23357	3.76063
Nc-1-IP_pe	chr10	64566799	64567240	442	64567211	9	3.54889	2.85226
Nc-1-IP_pe	chr10	64567694	64568100	407	64568018	11	5.03835	3.5092
Nc-1-IP_pe	chr10	64577412	64577690	279	64577560	14	6.78215	4.0774
Nc-1-IP_pe	chr10	64618904	64619189	286	64618971	12	6.50787	4.22912
Nc-1-IP_pe	chr10	64628996	64629324	329	64629157	9	3.72192	2.95716
Nc-1-IP_pe	chr10	64641523	64641783	261	64641715	14	6.06519	3.69269
Nc-1-IP_pe	chr10	64643137	64643702	566	64643290	14	8.63073	5.12814
Nc-1-IP_pe	chr10	64671796	64672381	586	64671899	12	5.23082	3.49289
Nc-1-IP_pe	chr10	64731474	64731834	361	64731605	19	9.92774	4.92358
Nc-1-IP_pe	chr10	64784093	64784562	470	64784175	11	4.54404	3.2242
Nc-1-IP_pe	chr10	64809838	64810415	578	64810349	18	7.32895	3.81908
Nc-1-IP_pe	chr10	64826416	64826694	279	64826580	12	5.15877	3.45264
Nc-1-IP_pe	chr10	64924317	64924619	303	64924552	11	4.06403	2.95415
Nc-1-IP_pe	chr10	65035236	65035676	441	65035480	14	6.69566	4.03025
Nc-1-IP_pe	chr10	65051252	65051511	260	65051371	11	5.03835	3.5092
Nc-1-IP_pe	chr10	65051789	65052419	631	65052323	17	10.6711	5.69559
Nc-1-IP_pe	chr10	65053468	65054225	758	65053664	16	7.53857	4.18505
Nc-1-IP_pe	chr10	65070844	65071223	380	65071124	12	4.70082	3.20033
Nc-1-IP_pe	chr10	65079441	65079702	262	65079593	13	4.41954	2.952
Nc-1-IP_pe	chr10	65083400	65083743	344	65083480	11	2.96397	2.36091
Nc-1-IP_pe	chr10	65084637	65085206	570	65085127	13	4.41954	2.952
Nc-1-IP_pe	chr10	65100754	65101048	295	65101046	12	2.87625	2.25568
Nc-1-IP_pe	chr10	65151445	65151713	269	65151630	11	6.03439	4.10251
Nc-1-IP_pe	chr10	65157584	65157925	342	65157719	10	4.99888	3.61677
Nc-1-IP_pe	chr10	65194265	65194536	272	65194396	12	6.58417	4.27436
Nc-1-IP_pe	chr10	65279091	65279347	257	65279257	17	8.02964	4.28847
Nc-1-IP_pe	chr10	65286048	65286346	299	65286207	14	6.96097	4.17548
Nc-1-IP_pe	chr10	65350086	65350363	278	65350201	13	4.41954	2.952
Nc-1-IP_pe	chr10	65429332	65429980	649	65429817	21	14.2479	6.776
Nc-1-IP_pe	chr10	65570679	65571015	337	65570770	16	4.96462	2.94973
Nc-1-IP_pe	chr10	65580342	65580594	253	65580414	11	6.03439	4.10251
Nc-1-IP_pe	chr10	65581556	65581972	417	65581731	17	10.6711	5.69559
Nc-1-IP_pe	chr10	65623512	65623778	267	65623690	9	3.72192	2.95716
Nc-1-IP_pe	chr10	65748444	65748711	268	65748574	11	5.77463	3.94557
Nc-1-IP_pe	chr10	65760304	65760696	393	65760516	14	7.14701	4.27839
Nc-1-IP_pe	chr10	65779009	65779588	580	65779557	12	3.16137	2.39719
Nc-1-IP_pe	chr10	65821938	65822217	280	65822048	12	6.58417	4.27436
Nc-1-IP_pe	chr10	65831001	65831343	343	65831214	16	8.26996	4.56762
Nc-1-IP_pe	chr10	65920404	65920826	423	65920508	8	3.56021	2.95918
Nc-1-IP_pe	chr10	65958667	65959151	485	65958809	16	6.37501	3.60492
Nc-1-IP_pe	chr10	65968475	65968745	271	65968719	10	4.39571	3.25288

Nc-1-IP_pe	chr10	66123450	66123907	458	66123648	12	4.24064	2.95299
Nc-1-IP_pe	chr10	66129746	66130133	388	66129948	13	5.9487	3.76157
Nc-1-IP_pe	chr10	66230877	66231161	285	66231024	12	5.94619	3.9002
Nc-1-IP_pe	chr10	66245771	66246495	725	66246320	16	5.37454	3.13479
Nc-1-IP_pe	chr10	66335896	66336333	438	66335957	12	5.23082	3.49289
Nc-1-IP_pe	chr10	66343995	66344398	404	66344034	9	4.46937	3.41876
Nc-1-IP_pe	chr10	66354003	66354256	254	66354128	13	7.7352	4.78626
Nc-1-IP_pe	chr10	66495697	66495990	294	66495833	11	5.77463	3.94557
Nc-1-IP_pe	chr10	66513550	66513902	353	66513693	12	4.24064	2.95299
Nc-1-IP_pe	chr10	66549051	66549332	282	66549134	13	7.7352	4.78626
Nc-1-IP_pe	chr10	66651382	66651801	420	66651598	16	8.57707	4.73221
Nc-1-IP_pe	chr10	66690914	66691681	768	66690969	14	8.1351	4.83895
Nc-1-IP_pe	chr10	66720634	66721087	454	66720722	13	5.9487	3.76157
Nc-1-IP_pe	chr10	66868202	66868742	541	66868608	13	7.7352	4.78626
Nc-1-IP_pe	chr10	66899627	66900157	531	66899773	13	5.9487	3.76157
Nc-1-IP_pe	chr10	67074245	67074547	303	67074274	8	3.74505	3.07688
Nc-1-IP_pe	chr10	67142756	67143268	513	67142817	11	6.03439	4.10251
Nc-1-IP_pe	chr10	67187579	67187838	260	67187731	11	5.10577	3.5486
Nc-1-IP_pe	chr10	67224749	67225561	813	67224889	11	5.3563	3.696
Nc-1-IP_pe	chr10	67248143	67248635	493	67248589	9	4.46937	3.41876
Nc-1-IP_pe	chr10	67403088	67403891	804	67403582	17	9.65211	5.13407
Nc-1-IP_pe	chr10	67476887	67477432	546	67476932	15	5.20142	3.14789
Nc-1-IP_pe	chr10	67524146	67524398	253	67524271	12	6.50787	4.22912
Nc-1-IP_pe	chr10	67551076	67551396	321	67551341	16	4.59224	2.7853
Nc-1-IP_pe	chr10	67597744	67598152	409	67597941	14	5.838	3.57373
Nc-1-IP_pe	chr10	67632405	67632851	447	67632675	16	9.49293	5.236
Nc-1-IP_pe	chr10	67691347	67692329	983	67692086	16	10.2074	5.6414
Nc-1-IP_pe	chr10	67699992	67700484	493	67700364	15	5.70822	3.39286
Nc-1-IP_pe	chr10	67738031	67738303	273	67738122	13	6.19316	3.89711
Nc-1-IP_pe	chr10	67746632	67747219	588	67746770	16	6.24025	3.54005
Nc-1-IP_pe	chr10	67767512	67768249	738	67767592	14	6.28469	3.80898
Nc-1-IP_pe	chr10	67774443	67774708	266	67774618	13	5.9487	3.76157
Nc-1-IP_pe	chr10	67795796	67796081	286	67795956	9	3.70468	2.94668
Nc-1-IP_pe	chr10	67812718	67813223	506	67812869	10	5.17577	3.72509
Nc-1-IP_pe	chr10	67842661	67842941	281	67842797	13	4.86362	3.18015
Nc-1-IP_pe	chr10	67844540	67844798	259	67844581	10	3.80898	2.90812
Nc-1-IP_pe	chr10	67855481	67855775	295	67855641	19	6.32622	3.27682
Nc-1-IP_pe	chr10	67870110	67870395	286	67870236	17	5.9911	3.31919
Nc-1-IP_pe	chr10	67878270	67878605	336	67878378	12	4.70082	3.20033
Nc-1-IP_pe	chr10	67884663	67885277	615	67885176	20	4.71088	2.57831
Nc-1-IP_pe	chr10	67890687	67890961	275	67890895	9	2.89249	2.46179
Nc-1-IP_pe	chr10	67918900	67919216	317	67919113	14	4.21449	2.76599
Nc-1-IP_pe	chr10	67952388	67952805	418	67952490	11	4.00079	2.91908
Nc-1-IP_pe	chr10	67978508	67978789	282	67978623	20	8.56319	4.1316
Nc-1-IP_pe	chr10	68069778	68070034	257	68069906	14	7.42849	4.43574

Nc-1-IP_pe	chr10	68127049	68127733	685	68127468	15	9.43796	5.40251
Nc-1-IP_pe	chr10	68163032	68163442	411	68163120	12	5.92876	3.89011
Nc-1-IP_pe	chr10	68166363	68166618	256	68166556	11	5.66869	3.88198
Nc-1-IP_pe	chr10	68184532	68185082	551	68184882	20	12.3659	5.98975
Nc-1-IP_pe	chr10	68200435	68200737	303	68200664	11	4.54404	3.2242
Nc-1-IP_pe	chr10	68216819	68217184	366	68217010	15	6.78905	3.93887
Nc-1-IP_pe	chr10	68231156	68231822	667	68231447	15	5.96953	3.52194
Nc-1-IP_pe	chr10	68238409	68239255	847	68238574	10	3.08907	2.49869
Nc-1-IP_pe	chr10	68241066	68241821	756	68241223	11	4.74697	3.34038
Nc-1-IP_pe	chr10	68245838	68246138	301	68245875	10	4.39571	3.25288
Nc-1-IP_pe	chr10	68251086	68251423	338	68251224	13	3.35769	2.42919
Nc-1-IP_pe	chr10	68279015	68279707	693	68279516	16	5.37454	3.13479
Nc-1-IP_pe	chr10	68283729	68284037	309	68283859	17	6.47874	3.54137
Nc-1-IP_pe	chr10	68332369	68332987	619	68332736	20	7.71924	3.7635
Nc-1-IP_pe	chr10	68353358	68353613	256	68353443	13	6.62436	4.14003
Nc-1-IP_pe	chr10	68358836	68359168	333	68358860	10	4.57677	3.36116
Nc-1-IP_pe	chr10	68362237	68362568	332	68362256	9	3.32589	2.71826
Nc-1-IP_pe	chr10	68370941	68371497	557	68371234	10	4.72812	3.45232
Nc-1-IP_pe	chr10	68381553	68381823	271	68381737	13	7.42513	4.60316
Nc-1-IP_pe	chr10	68391545	68391806	262	68391603	11	2.96397	2.36091
Nc-1-IP_pe	chr10	68398020	68398608	589	68398416	12	3.47963	2.55766
Nc-1-IP_pe	chr10	68406479	68407630	1152	68407035	20	6.49719	3.259
Nc-1-IP_pe	chr10	68415949	68416311	363	68416096	12	5.45448	3.61875
Nc-1-IP_pe	chr10	68425328	68425623	296	68425520	12	4.70082	3.20033
Nc-1-IP_pe	chr10	68443817	68444278	462	68444133	10	4.47495	3.30017
Nc-1-IP_pe	chr10	68445145	68445405	261	68445241	11	5.3232	3.67643
Nc-1-IP_pe	chr10	68457882	68458522	641	68458365	13	6.26093	3.93497
Nc-1-IP_pe	chr10	68471649	68472107	459	68472066	16	4.25252	2.63824
Nc-1-IP_pe	chr10	68477161	68477662	502	68477591	14	5.03085	3.16286
Nc-1-IP_pe	chr10	68481083	68482110	1028	68481211	11	5.83542	3.98217
Nc-1-IP_pe	chr10	68518860	68519248	389	68519209	13	5.9487	3.76157
Nc-1-IP_pe	chr10	68523536	68524161	626	68523668	16	6.33285	3.58457
Nc-1-IP_pe	chr10	68527731	68528350	620	68527852	18	5.33197	2.94862
Nc-1-IP_pe	chr10	68529006	68529395	390	68529075	11	4.02303	2.9314
Nc-1-IP_pe	chr10	68532117	68532386	270	68532175	17	5.54967	3.12324
Nc-1-IP_pe	chr10	68560259	68560937	679	68560557	17	5.9911	3.31919
Nc-1-IP_pe	chr10	68575165	68575787	623	68575571	14	7.016	4.20583
Nc-1-IP_pe	chr10	68576054	68576653	600	68576201	12	5.64372	3.72631
Nc-1-IP_pe	chr10	68577039	68577735	697	68577150	11	5.14641	3.5724
Nc-1-IP_pe	chr10	68592001	68592293	293	68592156	10	4.39571	3.25288
Nc-1-IP_pe	chr10	68599127	68599713	587	68599371	17	4.44438	2.65333
Nc-1-IP_pe	chr10	68605094	68605719	626	68605261	16	8.94801	4.93397
Nc-1-IP_pe	chr10	68609026	68609304	279	68609151	11	4.74697	3.34038
Nc-1-IP_pe	chr10	68611070	68611331	262	68611202	15	4.06016	2.62146
Nc-1-IP_pe	chr10	68630370	68630677	308	68630513	18	9.94172	5.10499

Nc-1-IP_pe	chr10	68636543	68636960	418	68636698	14	6.06519	3.69269
Nc-1-IP_pe	chr10	68639615	68640079	465	68639747	10	3.89064	2.95552
Nc-1-IP_pe	chr10	68644094	68644449	356	68644264	13	5.36853	3.44651
Nc-1-IP_pe	chr10	68657050	68657649	600	68657531	13	7.17043	4.45427
Nc-1-IP_pe	chr10	68664566	68664956	391	68664915	10	3.40382	2.67582
Nc-1-IP_pe	chr10	68677620	68678005	386	68677654	10	4.87068	3.5387
Nc-1-IP_pe	chr10	68688151	68688621	471	68688516	12	5.84943	3.84431
Nc-1-IP_pe	chr10	68691110	68691485	376	68691225	12	3.47963	2.55766
Nc-1-IP_pe	chr10	68701675	68701946	272	68701718	12	4.88508	3.30112
Nc-1-IP_pe	chr10	68714770	68715053	284	68715043	10	3.83201	2.92147
Nc-1-IP_pe	chr10	68718950	68719215	266	68719078	16	8.57707	4.73221
Nc-1-IP_pe	chr10	68721275	68721864	590	68721523	20	4.71088	2.57831
Nc-1-IP_pe	chr10	68722548	68722847	300	68722719	14	4.60011	2.95114
Nc-1-IP_pe	chr10	68723598	68723915	318	68723639	9	3.84391	3.03159
Nc-1-IP_pe	chr10	68745310	68745646	337	68745364	12	3.47963	2.55766
Nc-1-IP_pe	chr10	68746332	68746836	505	68746505	10	4.62142	3.388
Nc-1-IP_pe	chr10	68752025	68752284	260	68752106	13	7.7352	4.78626
Nc-1-IP_pe	chr10	68766287	68766752	466	68766563	10	3.08907	2.49869
Nc-1-IP_pe	chr10	68769388	68769641	254	68769408	12	4.70082	3.20033
Nc-1-IP_pe	chr10	68771789	68772044	256	68771950	10	4.11081	3.08427
Nc-1-IP_pe	chr10	68773953	68774542	590	68774076	15	7.46994	4.29894
Nc-1-IP_pe	chr10	68777779	68778085	307	68777906	13	4.02562	2.7544
Nc-1-IP_pe	chr10	68781044	68781624	581	68781497	11	4.32767	3.10163
Nc-1-IP_pe	chr10	68799563	68799911	349	68799879	11	4.06403	2.95415
Nc-1-IP_pe	chr10	68815749	68816076	328	68815948	17	7.36791	3.96218
Nc-1-IP_pe	chr10	68818120	68818487	368	68818239	11	4.06802	2.95637
Nc-1-IP_pe	chr10	68827154	68827813	660	68827384	13	3.35769	2.42919
Nc-1-IP_pe	chr10	68828135	68828586	452	68828370	19	6.32622	3.27682
Nc-1-IP_pe	chr10	68832157	68832529	373	68832304	14	5.44699	3.37238
Nc-1-IP_pe	chr10	68844897	68845533	637	68845053	13	6.52521	4.08375
Nc-1-IP_pe	chr10	68854186	68854460	275	68854200	13	3.35769	2.42919
Nc-1-IP_pe	chr10	68880776	68881044	269	68880900	10	4.50371	3.31737
Nc-1-IP_pe	chr10	68905599	68905912	314	68905783	17	8.31233	4.43123
Nc-1-IP_pe	chr10	68917225	68917519	295	68917370	15	9.38402	5.37117
Nc-1-IP_pe	chr10	68955882	68956600	719	68956391	26	11.0543	4.42371
Nc-1-IP_pe	chr10	68967993	68968441	449	68968322	16	6.8987	3.86161
Nc-1-IP_pe	chr10	68982546	68982806	261	68982661	16	8.26996	4.56762
Nc-1-IP_pe	chr10	68999217	68999702	486	68999636	11	4.06403	2.95415
Nc-1-IP_pe	chr10	69018528	69018800	273	69018542	10	3.08907	2.49869
Nc-1-IP_pe	chr10	69021305	69021605	301	69021394	15	5.20142	3.14789
Nc-1-IP_pe	chr10	69023276	69023823	548	69023458	17	5.54967	3.12324
Nc-1-IP_pe	chr10	69041610	69041943	334	69041757	10	3.4604	2.70797
Nc-1-IP_pe	chr10	69042318	69042573	256	69042506	17	5.9911	3.31919
Nc-1-IP_pe	chr10	69047350	69047886	537	69047842	14	3.86727	2.6027
Nc-1-IP_pe	chr10	69048216	69048601	386	69048217	13	3.07238	2.29378

Nc-1-IP_pe	chr10	69077610	69078227	618	69077983	11	5.7051	3.90381
Nc-1-IP_pe	chr10	69108080	69108663	584	69108347	11	5.10577	3.5486
Nc-1-IP_pe	chr10	69109838	69110169	332	69109936	14	7.62616	4.54739
Nc-1-IP_pe	chr10	69123683	69124532	850	69124353	19	5.51632	2.94814
Nc-1-IP_pe	chr10	69126219	69126481	263	69126436	12	5.33338	3.55042
Nc-1-IP_pe	chr10	69131160	69131636	477	69131198	10	3.89064	2.95552
Nc-1-IP_pe	chr10	69142020	69142346	327	69142230	15	6.78905	3.93887
Nc-1-IP_pe	chr10	69150278	69150530	253	69150458	15	5.20142	3.14789
Nc-1-IP_pe	chr10	69154313	69154703	391	69154446	12	4.70082	3.20033
Nc-1-IP_pe	chr10	69159065	69159358	294	69159220	11	5.56156	3.81794
Nc-1-IP_pe	chr10	69180057	69180688	632	69180308	39	25.9655	8.18229
Nc-1-IP_pe	chr10	69184226	69184530	305	69184449	15	7.35943	4.23968
Nc-1-IP_pe	chr10	69199437	69199700	264	69199523	16	8.26996	4.56762
Nc-1-IP_pe	chr10	69205396	69205838	443	69205607	15	5.66913	3.37372
Nc-1-IP_pe	chr10	69234963	69235314	352	69235025	15	5.86312	3.46915
Nc-1-IP_pe	chr10	69239994	69240636	643	69240101	13	5.96425	3.77015
Nc-1-IP_pe	chr10	69243036	69243444	409	69243267	16	7.8117	4.32634
Nc-1-IP_pe	chr10	69255260	69255649	390	69255450	18	9.48302	4.86743
Nc-1-IP_pe	chr10	69265430	69266044	615	69265847	18	7.18674	3.75388
Nc-1-IP_pe	chr10	69273235	69273677	443	69273589	11	4.58343	3.24666
Nc-1-IP_pe	chr10	69295028	69295297	270	69295154	14	6.11547	3.71921
Nc-1-IP_pe	chr10	69297865	69298136	272	69298072	14	6.412	3.87704
Nc-1-IP_pe	chr10	69299608	69300018	411	69299871	12	4.24064	2.95299
Nc-1-IP_pe	chr10	69304441	69305230	790	69304519	15	6.39297	3.73508
Nc-1-IP_pe	chr10	69315833	69316230	398	69316145	19	5.51632	2.94814
Nc-1-IP_pe	chr10	69317976	69318560	585	69318063	21	7.4689	3.5571
Nc-1-IP_pe	chr10	69320035	69320730	696	69320628	19	5.15822	2.80735
Nc-1-IP_pe	chr10	69329738	69329996	259	69329816	14	5.35794	3.32713
Nc-1-IP_pe	chr10	69334261	69335237	977	69334703	20	6.95357	3.44353
Nc-1-IP_pe	chr10	69335550	69335804	255	69335680	15	5.29768	3.19388
Nc-1-IP_pe	chr10	69338137	69338410	274	69338374	16	4.96462	2.94973
Nc-1-IP_pe	chr10	69341395	69341919	525	69341551	13	4.26502	2.87396
Nc-1-IP_pe	chr10	69343420	69343803	384	69343637	15	5.77209	3.42424
Nc-1-IP_pe	chr10	69354613	69354880	268	69354814	13	4.86362	3.18015
Nc-1-IP_pe	chr10	69360189	69360726	538	69360340	20	8.56319	4.1316
Nc-1-IP_pe	chr10	69365679	69365984	306	69365759	12	3.94559	2.79771
Nc-1-IP_pe	chr10	69375888	69376301	414	69375897	14	3.00733	2.21111
Nc-1-IP_pe	chr10	69376972	69377262	291	69377120	13	4.9575	3.22911
Nc-1-IP_pe	chr10	69382271	69382658	388	69382575	18	9.31716	4.78273
Nc-1-IP_pe	chr10	69387104	69387507	404	69387382	13	4.55021	3.01854
Nc-1-IP_pe	chr10	69392213	69392483	271	69392440	16	3.1497	2.1782
Nc-1-IP_pe	chr10	69392752	69393553	802	69392775	22	6.04768	2.93867
Nc-1-IP_pe	chr10	69394531	69395200	670	69394712	16	3.94146	2.50592
Nc-1-IP_pe	chr10	69398538	69398979	442	69398751	20	8.56319	4.1316
Nc-1-IP_pe	chr10	69401379	69401670	292	69401501	13	4.57229	3.02983

Nc-1-IP_pe	chr10	69406155	69406465	311	69406260	16	6.17431	3.50848
Nc-1-IP_pe	chr10	69407357	69408318	962	69407741	19	6.32622	3.27682
Nc-1-IP_pe	chr10	69413918	69414238	321	69414038	16	6.24025	3.54005
Nc-1-IP_pe	chr10	69416741	69417050	310	69416931	15	4.7819	2.95039
Nc-1-IP_pe	chr10	69419772	69420067	296	69419816	16	4.96462	2.94973
Nc-1-IP_pe	chr10	69424338	69424743	406	69424723	12	2.87625	2.25568
Nc-1-IP_pe	chr10	69428083	69428359	277	69428172	14	3.86727	2.6027
Nc-1-IP_pe	chr10	69429238	69429529	292	69429493	14	6.22239	3.77584
Nc-1-IP_pe	chr10	69432806	69433295	490	69433113	26	11.0543	4.42371
Nc-1-IP_pe	chr10	69435093	69435665	573	69435581	16	5.39474	3.14402
Nc-1-IP_pe	chr10	69435950	69436612	663	69436340	21	7.59399	3.60751
Nc-1-IP_pe	chr10	69451665	69451960	296	69451751	20	5.01743	2.69071
Nc-1-IP_pe	chr10	69465934	69466291	358	69465990	17	6.23061	3.42756
Nc-1-IP_pe	chr10	69474067	69474608	542	69474586	14	4.52309	2.91384
Nc-1-IP_pe	chr10	69476764	69477054	291	69476967	16	5.37454	3.13479
Nc-1-IP_pe	chr10	69482234	69482714	481	69482306	14	5.06689	3.18081
Nc-1-IP_pe	chr10	69483757	69484062	306	69483887	14	5.63114	3.46667
Nc-1-IP_pe	chr10	69492802	69493393	592	69493353	18	5.23669	2.90944
Nc-1-IP_pe	chr10	69493821	69494098	278	69493840	15	4.06504	2.62364
Nc-1-IP_pe	chr10	69494925	69495446	522	69494988	15	2.95965	2.14351
Nc-1-IP_pe	chr10	69499906	69500237	332	69499957	16	5.37454	3.13479
Nc-1-IP_pe	chr10	69501651	69501943	293	69501741	15	5.07393	3.08736
Nc-1-IP_pe	chr10	69502318	69502790	473	69502430	19	7.72209	3.87861
Nc-1-IP_pe	chr10	69504726	69505013	288	69504946	14	4.21449	2.76599
Nc-1-IP_pe	chr10	69517342	69517610	269	69517397	13	4.41954	2.952
Nc-1-IP_pe	chr10	69544907	69545457	551	69545014	13	4.02562	2.7544
Nc-1-IP_pe	chr10	69558359	69559472	1114	69558498	18	7.1522	3.73811
Nc-1-IP_pe	chr10	69559930	69560322	393	69560145	15	6.78905	3.93887
Nc-1-IP_pe	chr10	69561283	69561596	314	69561453	17	6.47874	3.54137
Nc-1-IP_pe	chr10	69570348	69570882	535	69570719	12	4.79401	3.25118
Nc-1-IP_pe	chr10	69571730	69572972	1243	69572800	21	8.26251	3.88258
Nc-1-IP_pe	chr10	69573621	69574133	513	69574035	15	5.70822	3.39286
Nc-1-IP_pe	chr10	69574411	69574949	539	69574442	11	3.28475	2.53029
Nc-1-IP_pe	chr10	69576465	69576776	312	69576615	13	5.70242	3.62667
Nc-1-IP_pe	chr10	69577336	69578282	947	69577737	15	7.46994	4.29894
Nc-1-IP_pe	chr10	69593425	69593693	269	69593576	14	7.40051	4.42001
Nc-1-IP_pe	chr10	69605001	69605344	344	69605188	14	6.06519	3.69269
Nc-1-IP_pe	chr10	69612186	69612440	255	69612375	13	6.92473	4.312
Nc-1-IP_pe	chr10	69618672	69618935	264	69618787	15	5.66913	3.37372
Nc-1-IP_pe	chr10	69620672	69620980	309	69620852	14	6.06519	3.69269
Nc-1-IP_pe	chr10	69630196	69631301	1106	69630538	18	7.47506	3.88659
Nc-1-IP_pe	chr10	69636658	69637538	881	69636789	17	6.76692	3.67553
Nc-1-IP_pe	chr10	69655150	69655673	524	69655332	17	6.93559	3.75504
Nc-1-IP_pe	chr10	69657489	69657755	267	69657722	15	5.20142	3.14789
Nc-1-IP_pe	chr10	69662691	69663233	543	69663073	16	6.1482	3.49601

Nc-1-IP_pe	chr10	69666149	69666473	325	69666282	14	3.86727	2.6027
Nc-1-IP_pe	chr10	69669219	69669618	400	69669392	15	6.16217	3.61829
Nc-1-IP_pe	chr10	69677484	69677738	255	69677493	10	3.59902	2.78714
Nc-1-IP_pe	chr10	69684181	69685209	1029	69684305	12	4.73037	3.21642
Nc-1-IP_pe	chr10	69692812	69693124	313	69692953	16	6.24025	3.54005
Nc-1-IP_pe	chr10	69693721	69694034	314	69693861	18	7.1522	3.73811
Nc-1-IP_pe	chr10	69714064	69714331	268	69714267	10	4.0355	3.04008
Nc-1-IP_pe	chr10	69717636	69718479	844	69717871	12	5.01934	3.37518
Nc-1-IP_pe	chr10	69728724	69729200	477	69728803	15	6.23304	3.654
Nc-1-IP_pe	chr10	69729812	69730169	358	69729905	17	6.47874	3.54137
Nc-1-IP_pe	chr10	69736104	69736412	309	69736195	13	4.81267	3.15368
Nc-1-IP_pe	chr10	69743688	69743964	277	69743852	13	4.41954	2.952
Nc-1-IP_pe	chr10	69747371	69747966	596	69747768	12	4.75569	3.23024
Nc-1-IP_pe	chr10	69749240	69749505	266	69749436	14	4.21449	2.76599
Nc-1-IP_pe	chr10	69749961	69750410	450	69750284	15	4.40338	2.77622
Nc-1-IP_pe	chr10	69766051	69767432	1382	69766504	14	5.42134	3.35933
Nc-1-IP_pe	chr10	69767930	69768182	253	69768173	13	3.07238	2.29378
Nc-1-IP_pe	chr10	69801090	69801585	496	69801403	19	3.71826	2.26621
Nc-1-IP_pe	chr10	69802734	69803002	269	69802823	19	5.23499	2.8373
Nc-1-IP_pe	chr10	69803571	69804138	568	69803942	14	3.86727	2.6027
Nc-1-IP_pe	chr10	69806012	69806501	490	69806321	22	8.81204	3.99809
Nc-1-IP_pe	chr10	69810800	69811582	783	69811211	17	7.38348	3.96973
Nc-1-IP_pe	chr10	69813338	69813859	522	69813495	17	7.06589	3.81697
Nc-1-IP_pe	chr10	69817910	69818196	287	69818052	12	3.47963	2.55766
Nc-1-IP_pe	chr10	69819390	69819895	506	69819778	14	5.51541	3.4073
Nc-1-IP_pe	chr10	69829580	69829966	387	69829710	15	4.06016	2.62146
Nc-1-IP_pe	chr10	69836095	69836381	287	69836253	19	8.35089	4.16476
Nc-1-IP_pe	chr10	69839805	69840075	271	69840047	14	3.00733	2.21111
Nc-1-IP_pe	chr10	69843760	69844409	650	69844258	14	5.86637	3.5885
Nc-1-IP_pe	chr10	69847780	69848049	270	69847856	16	6.8987	3.86161
Nc-1-IP_pe	chr10	69848617	69849471	855	69848786	15	7.12899	4.11712
Nc-1-IP_pe	chr10	69850579	69850961	383	69850888	14	5.03085	3.16286
Nc-1-IP_pe	chr10	69854074	69854388	315	69854180	10	3.56726	2.76895
Nc-1-IP_pe	chr10	69860753	69861084	332	69860941	18	6.40661	3.40507
Nc-1-IP_pe	chr10	69862953	69863779	827	69863023	17	6.92131	3.74829
Nc-1-IP_pe	chr10	69867275	69867975	701	69867490	18	5.72638	3.11298
Nc-1-IP_pe	chr10	69872110	69872610	501	69872134	12	3.47963	2.55766
Nc-1-IP_pe	chr10	69880273	69881107	835	69880514	17	7.29073	3.92485
Nc-1-IP_pe	chr10	69889092	69889352	261	69889157	12	4.24064	2.95299
Nc-1-IP_pe	chr10	69893029	69893923	895	69893494	16	5.82808	3.34463
Nc-1-IP_pe	chr10	69896295	69896758	464	69896637	13	4.82453	3.15983
Nc-1-IP_pe	chr10	69897319	69897719	401	69897562	14	5.75402	3.53012
Nc-1-IP_pe	chr10	69904307	69904642	336	69904548	16	7.01793	3.92106
Nc-1-IP_pe	chr10	69909104	69909382	279	69909125	14	5.51541	3.4073
Nc-1-IP_pe	chr10	69917791	69918271	481	69917809	14	3.96259	2.64722

Nc-1-IP_pe	chr10	69922687	69923275	589	69923133	17	7.02067	3.79543
Nc-1-IP_pe	chr10	69928959	69929748	790	69929082	14	4.21449	2.76599
Nc-1-IP_pe	chr10	69937659	69938237	579	69937804	14	5.93814	3.62598
Nc-1-IP_pe	chr10	69943459	69943769	311	69943556	15	6.5434	3.81197
Nc-1-IP_pe	chr10	69954106	69954586	481	69954150	11	4.35236	3.11555
Nc-1-IP_pe	chr10	69957608	69958132	525	69958046	15	5.58327	3.33181
Nc-1-IP_pe	chr10	69962267	69962579	313	69962337	11	4.00079	2.91908
Nc-1-IP_pe	chr10	69962946	69963236	291	69963040	14	5.838	3.57373
Nc-1-IP_pe	chr10	69968413	69968699	287	69968579	14	5.98179	3.64885
Nc-1-IP_pe	chr10	70052684	70053602	919	70053433	20	8.60223	4.14902
Nc-1-IP_pe	chr10	70053901	70054419	519	70053917	14	4.21449	2.76599
Nc-1-IP_pe	chr10	70059633	70060622	990	70060468	15	6.19428	3.63445
Nc-1-IP_pe	chr10	70075413	70075867	455	70075605	17	7.02067	3.79543
Nc-1-IP_pe	chr10	70083143	70083779	637	70083624	15	4.40338	2.77622
Nc-1-IP_pe	chr10	70085107	70085495	389	70085368	16	7.79347	4.31685
Nc-1-IP_pe	chr10	70102189	70102500	312	70102359	15	5.66913	3.37372
Nc-1-IP_pe	chr10	70105347	70105616	270	70105483	14	4.62572	2.96359
Nc-1-IP_pe	chr10	70109287	70109748	462	70109404	17	7.77178	4.16
Nc-1-IP_pe	chr10	70111293	70111849	557	70111716	21	10.8799	5.05417
Nc-1-IP_pe	chr10	70112672	70113761	1090	70113140	14	5.49876	3.39879
Nc-1-IP_pe	chr10	70116105	70116377	273	70116162	15	4.7819	2.95039
Nc-1-IP_pe	chr10	70116683	70117028	346	70116871	16	5.50606	3.19508
Nc-1-IP_pe	chr10	70117685	70117963	279	70117837	13	4.02562	2.7544
Nc-1-IP_pe	chr10	70119911	70120278	368	70119942	12	3.92572	2.78735
Nc-1-IP_pe	chr10	70122438	70122707	270	70122567	15	6.17625	3.62538
Nc-1-IP_pe	chr10	70126572	70128049	1478	70127144	16	6.80394	3.81462
Nc-1-IP_pe	chr10	70131429	70131858	430	70131627	17	5.9911	3.31919
Nc-1-IP_pe	chr10	70132126	70132903	778	70132603	16	4.25252	2.63824
Nc-1-IP_pe	chr10	70133376	70133633	258	70133577	15	5.6578	3.36818
Nc-1-IP_pe	chr10	70139448	70139884	437	70139632	14	4.95172	3.12357
Nc-1-IP_pe	chr10	70145262	70145745	484	70145494	24	7.19588	3.20323
Nc-1-IP_pe	chr10	70146276	70146714	439	70146337	20	5.01743	2.69071
Nc-1-IP_pe	chr10	70152174	70152643	470	70152331	12	5.45448	3.61875
Nc-1-IP_pe	chr10	70162132	70162421	290	70162220	10	4.09813	3.07682
Nc-1-IP_pe	chr10	70185074	70185421	348	70185148	13	5.36853	3.44651
Nc-1-IP_pe	chr10	70220063	70220335	273	70220117	13	4.86362	3.18015
Nc-1-IP_pe	chr10	70232929	70233753	825	70233660	17	5.14803	2.94914
Nc-1-IP_pe	chr10	70235897	70236369	473	70236252	12	4.48682	3.08453
Nc-1-IP_pe	chr10	70240155	70240956	802	70240312	14	5.92369	3.61842
Nc-1-IP_pe	chr10	70254816	70255746	931	70255074	18	7.93853	4.10423
Nc-1-IP_pe	chr10	70260479	70260851	373	70260522	16	4.96462	2.94973
Nc-1-IP_pe	chr10	70274185	70274510	326	70274299	16	8.09318	4.47392
Nc-1-IP_pe	chr10	70280798	70281146	349	70281090	13	5.36853	3.44651
Nc-1-IP_pe	chr10	70283588	70284328	741	70283775	19	8.68415	4.32024
Nc-1-IP_pe	chr10	70294912	70295197	286	70294966	14	5.03085	3.16286

Nc-1-IP_pe	chr10	70299985	70300410	426	70300075	11	3.64839	2.72584
Nc-1-IP_pe	chr10	70300977	70301619	643	70301111	14	6.13058	3.7272
Nc-1-IP_pe	chr10	70309683	70309944	262	70309727	14	5.838	3.57373
Nc-1-IP_pe	chr10	70317562	70317979	418	70317712	14	6.01114	3.66425
Nc-1-IP_pe	chr10	70323212	70324018	807	70323856	20	3.6727	2.20918
Nc-1-IP_pe	chr10	70335171	70335634	464	70335579	17	5.54967	3.12324
Nc-1-IP_pe	chr10	70346910	70347641	732	70347453	19	7.29128	3.68799
Nc-1-IP_pe	chr10	70348740	70349257	518	70349148	18	7.73136	4.00629
Nc-1-IP_pe	chr10	70354555	70355109	555	70354710	16	7.35733	4.09235
Nc-1-IP_pe	chr10	70358265	70358765	501	70358357	17	4.44438	2.65333
Nc-1-IP_pe	chr10	70362699	70362979	281	70362722	12	3.76171	2.70223
Nc-1-IP_pe	chr10	70368935	70369255	321	70369250	10	3.49455	2.72742
Nc-1-IP_pe	chr10	70371531	70371935	405	70371893	13	3.67377	2.58159
Nc-1-IP_pe	chr10	70373853	70374211	359	70374032	17	4.13464	2.52661
Nc-1-IP_pe	chr10	70376018	70376365	348	70376173	15	6.2617	3.66848
Nc-1-IP_pe	chr10	70380304	70380991	688	70380621	15	5.20142	3.14789
Nc-1-IP_pe	chr10	70381913	70382471	559	70382196	20	6.95357	3.44353
Nc-1-IP_pe	chr10	70398148	70398607	460	70398178	12	3.47963	2.55766
Nc-1-IP_pe	chr10	70404276	70404814	539	70404696	25	10.3273	4.25987
Nc-1-IP_pe	chr10	70435304	70435721	418	70435661	13	3.35769	2.42919
Nc-1-IP_pe	chr10	70442013	70442757	745	70442290	17	4.13464	2.52661
Nc-1-IP_pe	chr10	70447505	70447776	272	70447559	13	4.67336	3.0817
Nc-1-IP_pe	chr10	70457850	70458327	478	70457986	15	5.67035	3.37431
Nc-1-IP_pe	chr10	70458736	70458988	253	70458841	20	5.70098	2.94771
Nc-1-IP_pe	chr10	70459403	70459753	351	70459579	14	4.862	3.07924
Nc-1-IP_pe	chr10	70466741	70467233	493	70467174	12	3.50082	2.56844
Nc-1-IP_pe	chr10	70472558	70472825	268	70472690	15	5.15085	3.12383
Nc-1-IP_pe	chr10	70478528	70478812	285	70478623	17	4.27863	2.58527
Nc-1-IP_pe	chr10	70480083	70481043	961	70480202	15	4.86199	2.98773
Nc-1-IP_pe	chr10	70481637	70482372	736	70481750	17	6.73936	3.66261
Nc-1-IP_pe	chr10	70483303	70483636	334	70483461	11	2.96397	2.36091
Nc-1-IP_pe	chr10	70488686	70489207	522	70488857	17	6.78076	3.68203
Nc-1-IP_pe	chr10	70493040	70493599	560	70493491	18	5.72638	3.11298
Nc-1-IP_pe	chr10	70496885	70497480	596	70497244	16	6.80394	3.81462
Nc-1-IP_pe	chr10	70499119	70499467	349	70499304	22	8.79433	3.99081
Nc-1-IP_pe	chr10	70500899	70501445	547	70501171	16	4.59224	2.7853
Nc-1-IP_pe	chr10	70508392	70508700	309	70508515	13	3.35769	2.42919
Nc-1-IP_pe	chr10	70510153	70510447	295	70510274	17	7.92501	4.23614
Nc-1-IP_pe	chr10	70513824	70514163	340	70513987	16	6.37501	3.60492
Nc-1-IP_pe	chr10	70515189	70515478	290	70515302	14	5.03085	3.16286
Nc-1-IP_pe	chr10	70519889	70520235	347	70519970	11	4.06802	2.95637
Nc-1-IP_pe	chr10	70523076	70523574	499	70523328	17	7.02067	3.79543
Nc-1-IP_pe	chr10	70529862	70530188	327	70530126	13	4.02562	2.7544
Nc-1-IP_pe	chr10	70541674	70542177	504	70541945	14	5.12578	3.21022
Nc-1-IP_pe	chr10	70544815	70545177	363	70545021	19	8.77046	4.36095

Nc-1-IP_pe	chr10	70549528	70550015	488	70549698	18	6.63038	3.50359
Nc-1-IP_pe	chr10	70553507	70554016	510	70553610	15	6.20456	3.63963
Nc-1-IP_pe	chr10	70561651	70562765	1115	70562604	20	7.43645	3.64378
Nc-1-IP_pe	chr10	70563364	70563746	383	70563406	12	3.47963	2.55766
Nc-1-IP_pe	chr10	70564129	70564662	534	70564322	16	5.37454	3.13479
Nc-1-IP_pe	chr10	70565294	70565735	442	70565566	11	3.92417	2.87675
Nc-1-IP_pe	chr10	70568619	70569032	414	70568857	16	6.65665	3.74206
Nc-1-IP_pe	chr10	70571341	70571853	513	70571491	20	8.56319	4.1316
Nc-1-IP_pe	chr10	70575486	70576278	793	70575586	16	7.53857	4.18505
Nc-1-IP_pe	chr10	70582712	70583381	670	70583217	15	7.46994	4.29894
Nc-1-IP_pe	chr10	70585159	70585857	699	70585310	15	8.22991	4.71468
Nc-1-IP_pe	chr10	70591204	70591533	330	70591255	13	4.86362	3.18015
Nc-1-IP_pe	chr10	70598456	70599107	652	70598554	15	4.7819	2.95039
Nc-1-IP_pe	chr10	70600456	70601163	708	70600705	12	5.27426	3.51722
Nc-1-IP_pe	chr10	70602498	70604009	1512	70603148	20	7.43645	3.64378
Nc-1-IP_pe	chr10	70622727	70623248	522	70623062	16	7.27441	4.05022
Nc-1-IP_pe	chr10	70633781	70634100	320	70634095	13	3.67377	2.58159
Nc-1-IP_pe	chr10	70638280	70638738	459	70638700	10	4.83846	3.51913
Nc-1-IP_pe	chr10	70647877	70648329	453	70648151	16	5.73763	3.30235
Nc-1-IP_pe	chr10	70655338	70655689	352	70655504	12	5.04681	3.39039
Nc-1-IP_pe	chr10	70672008	70672318	311	70672225	14	3.55308	2.45762
Nc-1-IP_pe	chr10	70672759	70673013	255	70672889	14	3.86727	2.6027
Nc-1-IP_pe	chr10	70674580	70675167	588	70674799	16	5.82808	3.34463
Nc-1-IP_pe	chr10	70675507	70675762	256	70675540	12	3.95558	2.80293
Nc-1-IP_pe	chr10	70681324	70681998	675	70681804	19	8.9469	4.44469
Nc-1-IP_pe	chr10	70685030	70685498	469	70685403	10	3.12201	2.51709
Nc-1-IP_pe	chr10	70689068	70689444	377	70689308	11	3.89191	2.85898
Nc-1-IP_pe	chr10	70690387	70691284	898	70690775	18	6.15736	3.29676
Nc-1-IP_pe	chr10	70693697	70694275	579	70694141	22	11.4793	5.17098
Nc-1-IP_pe	chr10	70698440	70698794	355	70698621	15	5.5956	3.33782
Nc-1-IP_pe	chr10	70700385	70700788	404	70700610	19	6.56181	3.3752
Nc-1-IP_pe	chr10	70703185	70703587	403	70703509	14	4.98591	3.14053
Nc-1-IP_pe	chr10	70706442	70706893	452	70706476	16	4.25252	2.63824
Nc-1-IP_pe	chr10	70709025	70709330	306	70709143	16	7.53857	4.18505
Nc-1-IP_pe	chr10	70710687	70710941	255	70710707	10	3.98634	3.01131
Nc-1-IP_pe	chr10	70712630	70712883	254	70712840	10	3.43373	2.6928
Nc-1-IP_pe	chr10	70723994	70724582	589	70724193	14	4.21449	2.76599
Nc-1-IP_pe	chr10	70731762	70732746	985	70732580	15	5.20142	3.14789
Nc-1-IP_pe	chr10	70736317	70736920	604	70736783	16	6.75928	3.79256
Nc-1-IP_pe	chr10	70742184	70742513	330	70742348	14	4.21449	2.76599
Nc-1-IP_pe	chr10	70743605	70744404	800	70743886	17	4.78101	2.79343
Nc-1-IP_pe	chr10	70747348	70747871	524	70747700	18	7.04825	3.69086
Nc-1-IP_pe	chr10	70751492	70752205	714	70752082	15	4.40338	2.77622
Nc-1-IP_pe	chr10	70754828	70755174	347	70754923	14	4.80698	3.05217
Nc-1-IP_pe	chr10	70757706	70758211	506	70758089	19	6.78661	3.47027

Nc-1-IP_pe	chr10	70760623	70760970	348	70760862	16	4.78259	2.86892
Nc-1-IP_pe	chr10	70763714	70764219	506	70763782	14	3.26761	2.32785
Nc-1-IP_pe	chr10	70766010	70766425	416	70766356	13	4.86362	3.18015
Nc-1-IP_pe	chr10	70772629	70773173	545	70772708	14	3.86727	2.6027
Nc-1-IP_pe	chr10	70774207	70774758	552	70774463	20	7.97282	3.87239
Nc-1-IP_pe	chr10	70775218	70775493	276	70775381	18	4.6358	2.66698
Nc-1-IP_pe	chr10	70777206	70777556	351	70777473	15	4.7819	2.95039
Nc-1-IP_pe	chr10	70779649	70779922	274	70779856	16	5.18047	3.04664
Nc-1-IP_pe	chr10	70781654	70781942	289	70781892	15	6.14813	3.61124
Nc-1-IP_pe	chr10	70806220	70806486	267	70806345	14	4.60011	2.95114
Nc-1-IP_pe	chr10	70809472	70809762	291	70809682	12	2.87625	2.25568
Nc-1-IP_pe	chr10	70820247	70820673	427	70820366	12	4.24064	2.95299
Nc-1-IP_pe	chr10	70861515	70862141	627	70861668	17	5.9911	3.31919
Nc-1-IP_pe	chr10	70879345	70879652	308	70879432	12	4.45173	3.06567
Nc-1-IP_pe	chr10	70887533	70888807	1275	70888315	24	5.47323	2.62998
Nc-1-IP_pe	chr10	70901392	70902282	891	70901688	13	4.02562	2.7544
Nc-1-IP_pe	chr10	70904810	70905564	755	70905264	18	7.73136	4.00629
Nc-1-IP_pe	chr10	70908224	70908634	411	70908266	16	4.25252	2.63824
Nc-1-IP_pe	chr10	70909810	70910237	428	70910044	15	5.58327	3.33181
Nc-1-IP_pe	chr10	70910542	70911413	872	70911133	20	9.56043	4.58764
Nc-1-IP_pe	chr10	70919199	70919488	290	70919385	17	7.13975	3.85227
Nc-1-IP_pe	chr10	70923894	70924247	354	70923943	15	4.40338	2.77622
Nc-1-IP_pe	chr10	70925031	70925595	565	70925499	17	6.51104	3.5563
Nc-1-IP_pe	chr10	70926427	70927170	744	70926648	18	5.72638	3.11298
Nc-1-IP_pe	chr10	70935866	70936430	565	70936232	14	4.8399	3.06836
Nc-1-IP_pe	chr10	70940828	70941104	277	70941052	18	5.33197	2.94862
Nc-1-IP_pe	chr10	70941828	70942187	360	70941862	12	3.86684	2.7567
Nc-1-IP_pe	chr10	70945869	70946356	488	70946214	12	3.96559	2.80816
Nc-1-IP_pe	chr10	70949895	70950418	524	70950310	12	5.03305	3.38277
Nc-1-IP_pe	chr10	70951226	70951500	275	70951406	11	4.54404	3.2242
Nc-1-IP_pe	chr10	70961193	70961470	278	70961355	16	6.24025	3.54005
Nc-1-IP_pe	chr10	70962876	70963333	458	70963153	12	4.01615	2.83462
Nc-1-IP_pe	chr10	70965622	70966027	406	70965730	12	4.06753	2.86157
Nc-1-IP_pe	chr10	70969432	70969703	272	70969613	14	5.03085	3.16286
Nc-1-IP_pe	chr10	70987825	70988317	493	70987936	16	6.95583	3.89005
Nc-1-IP_pe	chr10	70997954	70998767	814	70998529	15	5.34394	3.21607
Nc-1-IP_pe	chr10	71004101	71004608	508	71004499	15	5.58327	3.33181
Nc-1-IP_pe	chr10	71008869	71009330	462	71009074	17	5.9911	3.31919
Nc-1-IP_pe	chr10	71022128	71022450	323	71022317	14	6.97924	4.18554
Nc-1-IP_pe	chr10	71025872	71026163	292	71025976	13	4.56123	3.02417
Nc-1-IP_pe	chr10	71027988	71028281	294	71028172	17	8.58498	4.57078
Nc-1-IP_pe	chr10	71029209	71029486	278	71029230	12	3.86684	2.7567
Nc-1-IP_pe	chr10	71034089	71034393	305	71034183	13	4.24481	2.8638
Nc-1-IP_pe	chr10	71040030	71040377	348	71040260	14	5.43414	3.36584
Nc-1-IP_pe	chr10	71040749	71041127	379	71041106	12	4.04688	2.85073

Nc-1-IP_pe	chr10	71043690	71044406	717	71043891	14	5.48576	3.39215
Nc-1-IP_pe	chr10	71047007	71047274	268	71047083	13	4.82453	3.15983
Nc-1-IP_pe	chr10	71052510	71052791	282	71052781	13	3.07238	2.29378
Nc-1-IP_pe	chr10	71059378	71059688	311	71059477	15	6.94226	4.01882
Nc-1-IP_pe	chr10	71061422	71062254	833	71061666	15	6.69889	3.8921
Nc-1-IP_pe	chr10	71065037	71066077	1041	71065299	16	5.98198	3.41707
Nc-1-IP_pe	chr10	71069047	71069492	446	71069180	13	4.02562	2.7544
Nc-1-IP_pe	chr10	71070042	71070472	431	71070097	13	4.01121	2.74725
Nc-1-IP_pe	chr10	71074886	71075531	646	71075150	14	4.59463	2.94848
Nc-1-IP_pe	chr10	71076252	71076865	614	71076707	15	5.00915	3.05678
Nc-1-IP_pe	chr10	71078581	71078877	297	71078800	11	3.64839	2.72584
Nc-1-IP_pe	chr10	71079787	71080254	468	71079916	17	7.72166	4.13523
Nc-1-IP_pe	chr10	71080873	71081377	505	71080923	13	4.41954	2.952
Nc-1-IP_pe	chr10	71085252	71085545	294	71085490	15	4.40338	2.77622
Nc-1-IP_pe	chr10	71087613	71087898	286	71087865	15	4.06016	2.62146
Nc-1-IP_pe	chr10	71093683	71093948	266	71093830	19	6.78661	3.47027
Nc-1-IP_pe	chr10	71117783	71118093	311	71117866	15	6.19428	3.63445
Nc-1-IP_pe	chr10	71125256	71125913	658	71125520	14	4.60011	2.95114
Nc-1-IP_pe	chr10	71131641	71131975	335	71131657	11	4.00079	2.91908
Nc-1-IP_pe	chr10	71134825	71135250	426	71135132	12	4.95159	3.33774
Nc-1-IP_pe	chr10	71136127	71137084	958	71136241	16	5.82808	3.34463
Nc-1-IP_pe	chr10	71137456	71137742	287	71137648	12	4.70082	3.20033
Nc-1-IP_pe	chr10	71140422	71140784	363	71140707	11	3.68532	2.74592
Nc-1-IP_pe	chr10	71175801	71176154	354	71176004	15	6.24734	3.66122
Nc-1-IP_pe	chr10	71177599	71177891	293	71177767	11	4.24272	3.05389
Nc-1-IP_pe	chr10	71179224	71179648	425	71179550	10	3.37424	2.65905
Nc-1-IP_pe	chr10	71181964	71182335	372	71182109	15	5.66913	3.37372
Nc-1-IP_pe	chr10	71188154	71188487	334	71188358	20	9.94094	4.76764
Nc-1-IP_pe	chr10	71191548	71192008	461	71191594	12	3.47963	2.55766
Nc-1-IP_pe	chr10	71195756	71196013	258	71195872	12	5.18731	3.46856
Nc-1-IP_pe	chr10	71212277	71213385	1109	71212536	22	7.27519	3.39036
Nc-1-IP_pe	chr10	71217322	71217602	281	71217513	18	6.15736	3.29676
Nc-1-IP_pe	chr10	71220634	71221310	677	71220722	17	7.29073	3.92485
Nc-1-IP_pe	chr10	71225800	71226226	427	71225875	12	4.58219	3.13597
Nc-1-IP_pe	chr10	71229655	71229927	273	71229801	18	5.72638	3.11298
Nc-1-IP_pe	chr10	71232029	71232981	953	71232901	14	6.05554	3.68761
Nc-1-IP_pe	chr10	71234711	71235864	1154	71234989	16	5.82808	3.34463
Nc-1-IP_pe	chr10	71236555	71237247	693	71237136	15	5.96953	3.52194
Nc-1-IP_pe	chr10	71240391	71240698	308	71240472	13	4.57229	3.02983
Nc-1-IP_pe	chr10	71242028	71242851	824	71242159	18	7.47506	3.88659
Nc-1-IP_pe	chr10	71244517	71244940	424	71244788	15	4.98779	3.04672
Nc-1-IP_pe	chr10	71256203	71257465	1263	71256566	19	7.05262	3.58428
Nc-1-IP_pe	chr10	71262457	71262742	286	71262540	13	4.35751	2.92059
Nc-1-IP_pe	chr10	71267944	71268584	641	71268396	18	7.1522	3.73811
Nc-1-IP_pe	chr10	71270401	71270656	256	71270653	15	2.95965	2.14351

Nc-1-IP_pe	chr10	71271826	71272869	1044	71272440	17	5.54967	3.12324
Nc-1-IP_pe	chr10	71277123	71277551	429	71277405	19	8.03556	4.02008
Nc-1-IP_pe	chr10	71278212	71278567	356	71278394	19	9.16563	4.54954
Nc-1-IP_pe	chr10	71282195	71282819	625	71282341	15	5.4025	3.24424
Nc-1-IP_pe	chr10	71283831	71284369	539	71284220	20	7.43645	3.64378
Nc-1-IP_pe	chr10	71284710	71286282	1573	71285433	16	5.37454	3.13479
Nc-1-IP_pe	chr10	71287574	71287985	412	71287691	16	4.59224	2.7853
Nc-1-IP_pe	chr10	71290571	71290953	383	71290821	16	6.33285	3.58457
Nc-1-IP_pe	chr10	71293340	71293903	564	71293853	12	2.87625	2.25568
Nc-1-IP_pe	chr10	71295881	71296443	563	71296002	14	5.19764	3.24624
Nc-1-IP_pe	chr10	71297838	71298317	480	71298138	18	6.63038	3.50359
Nc-1-IP_pe	chr10	71298977	71299306	330	71299251	14	4.8399	3.06836
Nc-1-IP_pe	chr10	71300637	71301616	980	71301461	17	7.75502	4.15171
Nc-1-IP_pe	chr10	71304068	71304542	475	71304311	21	10.7754	5.00449
Nc-1-IP_pe	chr10	71316773	71317447	675	71317341	14	5.03085	3.16286
Nc-1-IP_pe	chr10	71326626	71326999	374	71326740	15	4.06016	2.62146
Nc-1-IP_pe	chr10	71327940	71328322	383	71328106	19	7.88414	3.95145
Nc-1-IP_pe	chr10	71337564	71338265	702	71337596	17	4.78101	2.79343
Nc-1-IP_pe	chr10	71340950	71341345	396	71341012	14	4.44302	2.87523
Nc-1-IP_pe	chr10	71345767	71346402	636	71345912	15	5.3092	3.1994
Nc-1-IP_pe	chr10	71347355	71347694	340	71347555	14	4.64659	2.97374
Nc-1-IP_pe	chr10	71353383	71353713	331	71353597	14	5.28315	3.2893
Nc-1-IP_pe	chr10	71354674	71355209	536	71354891	13	4.67336	3.0817
Nc-1-IP_pe	chr10	71357181	71357439	259	71357376	13	5.29187	3.40559
Nc-1-IP_pe	chr10	71362196	71362730	535	71362652	14	5.02041	3.15766
Nc-1-IP_pe	chr10	71367714	71367976	263	71367798	14	3.26761	2.32785
Nc-1-IP_pe	chr10	71370052	71370325	274	71370220	14	3.55308	2.45762
Nc-1-IP_pe	chr10	71377009	71377482	474	71377271	19	9.22155	4.57653
Nc-1-IP_pe	chr10	71380176	71380587	412	71380296	13	5.42865	3.47872
Nc-1-IP_pe	chr10	71383928	71384552	625	71384481	15	4.06016	2.62146
Nc-1-IP_pe	chr10	71385324	71385643	320	71385401	12	4.24064	2.95299
Nc-1-IP_pe	chr10	71386146	71386510	365	71386330	16	7.61454	4.22416
Nc-1-IP_pe	chr10	71389915	71390264	350	71390134	14	6.10041	3.71126
Nc-1-IP_pe	chr10	71391311	71391586	276	71391440	13	4.41954	2.952
Nc-1-IP_pe	chr10	71393302	71393702	401	71393655	15	3.46204	2.35851
Nc-1-IP_pe	chr10	71395163	71395449	287	71395327	16	6.57039	3.69983
Nc-1-IP_pe	chr10	71397877	71398157	281	71398004	15	6.55872	3.81984
Nc-1-IP_pe	chr10	71400054	71400391	338	71400197	13	5.29187	3.40559
Nc-1-IP_pe	chr10	71402822	71403148	327	71403004	11	3.64839	2.72584
Nc-1-IP_pe	chr10	71407304	71407621	318	71407425	13	4.41954	2.952
Nc-1-IP_pe	chr10	71422643	71422919	277	71422856	14	5.51541	3.4073
Nc-1-IP_pe	chr10	71423815	71424102	288	71424026	13	5.19892	3.35621
Nc-1-IP_pe	chr10	71430100	71430398	299	71430201	11	3.53939	2.66682
Nc-1-IP_pe	chr10	71439821	71440078	258	71439892	16	4.97667	2.95511
Nc-1-IP_pe	chr10	71442285	71443311	1027	71443075	16	6.33285	3.58457

Nc-1-IP_pe	chr10	71446051	71446445	395	71446185	15	6.74657	3.91681
Nc-1-IP_pe	chr10	71446974	71447425	452	71447049	12	4.32611	2.99846
Nc-1-IP_pe	chr10	71447709	71448787	1079	71448639	17	7.50977	4.03119
Nc-1-IP_pe	chr10	71452043	71452396	354	71452250	17	5.35342	3.03768
Nc-1-IP_pe	chr10	71459621	71460010	390	71459830	19	8.68415	4.32024
Nc-1-IP_pe	chr10	71460411	71460951	541	71460640	16	6.24025	3.54005
Nc-1-IP_pe	chr10	71461607	71462233	627	71462065	12	4.11973	2.88905
Nc-1-IP_pe	chr10	71472028	71472697	670	71472229	18	8.37884	4.31591
Nc-1-IP_pe	chr10	71475313	71476194	882	71475821	18	8.33933	4.29672
Nc-1-IP_pe	chr10	71481794	71482160	367	71481851	14	4.21449	2.76599
Nc-1-IP_pe	chr10	71483556	71484406	851	71483998	13	4.02562	2.7544
Nc-1-IP_pe	chr10	71491796	71492108	313	71491850	15	6.10627	3.59023
Nc-1-IP_pe	chr10	71492742	71493047	306	71493032	13	3.07238	2.29378
Nc-1-IP_pe	chr10	71494557	71495098	542	71494639	14	4.52309	2.91384
Nc-1-IP_pe	chr10	71497810	71498849	1040	71497960	17	6.47874	3.54137
Nc-1-IP_pe	chr10	71502951	71503273	323	71503137	14	6.36383	3.85124
Nc-1-IP_pe	chr10	71503589	71503895	307	71503813	12	5.06062	3.39805
Nc-1-IP_pe	chr10	71511807	71512601	795	71511959	14	5.14959	3.22214
Nc-1-IP_pe	chr10	71513411	71513663	253	71513505	14	3.86727	2.6027
Nc-1-IP_pe	chr10	71514265	71514597	333	71514532	12	3.88634	2.76684
Nc-1-IP_pe	chr10	71521143	71521480	338	71521209	17	6.13258	3.38303
Nc-1-IP_pe	chr10	71525380	71526509	1130	71526370	18	8.30482	4.27999
Nc-1-IP_pe	chr10	71527219	71527756	538	71527390	14	5.61771	3.45976
Nc-1-IP_pe	chr10	71528176	71528501	326	71528347	18	8.37884	4.31591
Nc-1-IP_pe	chr10	71528800	71530556	1757	71528910	15	5.20142	3.14789
Nc-1-IP_pe	chr10	71542184	71542577	394	71542551	15	3.74763	2.48304
Nc-1-IP_pe	chr10	71547568	71548237	670	71548175	12	3.67829	2.65924
Nc-1-IP_pe	chr10	71560296	71560551	256	71560412	12	5.17301	3.46058
Nc-1-IP_pe	chr10	71561831	71562304	474	71562022	17	8.31233	4.43123
Nc-1-IP_pe	chr10	71566023	71566361	339	71566139	18	7.04825	3.69086
Nc-1-IP_pe	chr10	71567483	71567809	327	71567624	13	4.86362	3.18015
Nc-1-IP_pe	chr10	71570113	71571258	1146	71571169	17	6.92131	3.74829
Nc-1-IP_pe	chr10	71576083	71576706	624	71576484	17	7.6273	4.08876
Nc-1-IP_pe	chr10	71579677	71580430	754	71580259	14	5.838	3.57373
Nc-1-IP_pe	chr10	71580907	71581407	501	71580959	11	3.7458	2.77889
Nc-1-IP_pe	chr10	71588017	71588305	289	71588176	14	6.46071	3.9032
Nc-1-IP_pe	chr10	71596502	71597347	846	71596820	15	5.66913	3.37372
Nc-1-IP_pe	chr10	71598233	71598516	284	71598414	14	4.21449	2.76599
Nc-1-IP_pe	chr10	71601523	71602033	511	71601652	12	4.16211	2.91142
Nc-1-IP_pe	chr10	71607614	71607928	315	71607841	17	5.9911	3.31919
Nc-1-IP_pe	chr10	71608666	71609065	400	71608971	14	3.55754	2.45966
Nc-1-IP_pe	chr10	71615488	71615868	381	71615695	19	5.51632	2.94814
Nc-1-IP_pe	chr10	71618640	71618915	276	71618700	17	6.47874	3.54137
Nc-1-IP_pe	chr10	71620235	71620894	660	71620680	22	8.24538	3.76835
Nc-1-IP_pe	chr10	71622622	71622928	307	71622840	15	5.22925	3.16116

Nc-1-IP_pe	chr10	71627574	71627845	272	71627590	12	3.16137	2.39719
Nc-1-IP_pe	chr10	71633891	71634225	335	71634095	20	6.08336	3.09555
Nc-1-IP_pe	chr10	71634637	71635123	487	71634832	11	4.29098	3.08099
Nc-1-IP_pe	chr10	71644997	71645721	725	71645527	16	6.1093	3.47747
Nc-1-IP_pe	chr10	71646936	71647275	340	71647008	12	4.06753	2.86157
Nc-1-IP_pe	chr10	71647835	71648797	963	71648736	17	4.96303	2.87025
Nc-1-IP_pe	chr10	71650223	71650520	298	71650431	11	3.45528	2.62151
Nc-1-IP_pe	chr10	71652833	71653179	347	71653115	12	3.74297	2.69256
Nc-1-IP_pe	chr10	71656072	71656352	281	71656154	19	7.05262	3.58428
Nc-1-IP_pe	chr10	71656946	71657304	359	71657153	13	3.35769	2.42919
Nc-1-IP_pe	chr10	71662310	71662990	681	71662523	12	4.63088	3.16234
Nc-1-IP_pe	chr10	71663268	71663571	304	71663490	17	7.6273	4.08876
Nc-1-IP_pe	chr10	71669965	71670225	261	71670102	12	4.15147	2.90579
Nc-1-IP_pe	chr10	71672235	71672799	565	71672387	13	3.35769	2.42919
Nc-1-IP_pe	chr10	71685376	71685714	339	71685552	18	5.62894	3.07204
Nc-1-IP_pe	chr10	71688464	71689325	862	71688795	33	14.7937	4.94465
Nc-1-IP_pe	chr10	71690209	71690678	470	71690428	19	8.82285	4.38574
Nc-1-IP_pe	chr10	71694665	71695446	782	71694781	18	6.15736	3.29676
Nc-1-IP_pe	chr10	71696273	71696681	409	71696641	14	4.62572	2.96359
Nc-1-IP_pe	chr10	71701903	71702498	596	71702331	16	5.39474	3.14402
Nc-1-IP_pe	chr10	71705000	71705621	622	71705064	16	5.98198	3.41707
Nc-1-IP_pe	chr10	71708130	71708540	411	71708359	12	3.16137	2.39719
Nc-1-IP_pe	chr10	71708894	71709528	635	71709056	19	6.80149	3.4766
Nc-1-IP_pe	chr10	71710247	71710699	453	71710487	16	4.25252	2.63824
Nc-1-IP_pe	chr10	71712761	71713747	987	71712819	17	4.96303	2.87025
Nc-1-IP_pe	chr10	71714535	71715492	958	71715247	16	4.59224	2.7853
Nc-1-IP_pe	chr10	71715980	71716688	709	71716455	15	4.40338	2.77622
Nc-1-IP_pe	chr10	71716959	71717418	460	71717128	17	6.72563	3.65618
Nc-1-IP_pe	chr10	71719707	71720203	497	71719743	18	3.77591	2.33276
Nc-1-IP_pe	chr10	71721243	71721867	625	71721327	17	7.35238	3.95466
Nc-1-IP_pe	chr10	71724322	71724684	363	71724601	16	5.82808	3.34463
Nc-1-IP_pe	chr10	71725283	71725702	420	71725537	21	8.6741	4.05679
Nc-1-IP_pe	chr10	71726176	71726711	536	71726690	16	5.37454	3.13479
Nc-1-IP_pe	chr10	71730857	71731163	307	71731025	14	4.52309	2.91384
Nc-1-IP_pe	chr10	71731958	71732261	304	71732133	17	5.9911	3.31919
Nc-1-IP_pe	chr10	71734301	71734632	332	71734469	16	5.18047	3.04664
Nc-1-IP_pe	chr10	71735057	71735317	261	71735162	19	4.8268	2.67939
Nc-1-IP_pe	chr10	71735833	71736153	321	71735942	16	4.97667	2.95511
Nc-1-IP_pe	chr10	71738246	71738829	584	71738308	18	6.90003	3.62395
Nc-1-IP_pe	chr10	71741347	71742219	873	71741845	18	7.53463	3.91426
Nc-1-IP_pe	chr10	71748672	71749295	624	71749134	15	5.83691	3.4562
Nc-1-IP_pe	chr10	71750737	71751306	570	71750896	16	5.82808	3.34463
Nc-1-IP_pe	chr10	71752360	71752737	378	71752594	22	10.4599	4.70482
Nc-1-IP_pe	chr10	71753494	71754269	776	71754106	18	8.56884	4.40872
Nc-1-IP_pe	chr10	71757319	71757949	631	71757534	16	7.00233	3.91326

Nc-1-IP_pe	chr10	71758435	71758799	365	71758493	14	5.98179	3.64885
Nc-1-IP_pe	chr10	71761657	71762553	897	71762389	17	7.22981	3.89549
Nc-1-IP_pe	chr10	71763477	71763849	373	71763570	12	3.62393	2.63134
Nc-1-IP_pe	chr10	71771784	71772286	503	71772086	19	6.32622	3.27682
Nc-1-IP_pe	chr10	71772725	71773086	362	71773058	17	3.84884	2.41145
Nc-1-IP_pe	chr10	71779236	71779511	276	71779365	12	4.06753	2.86157
Nc-1-IP_pe	chr10	71780388	71780687	300	71780462	14	5.25853	3.27688
Nc-1-IP_pe	chr10	71782030	71782360	331	71782191	15	5.1177	3.10809
Nc-1-IP_pe	chr10	71782873	71783399	527	71783172	13	4.92076	3.20992
Nc-1-IP_pe	chr10	71788653	71789544	892	71789282	16	7.24162	4.03361
Nc-1-IP_pe	chr10	71790107	71790362	256	71790236	17	5.14803	2.94914
Nc-1-IP_pe	chr10	71795770	71796419	650	71796270	13	4.02562	2.7544
Nc-1-IP_pe	chr10	71798705	71798979	275	71798709	12	3.58823	2.61305
Nc-1-IP_pe	chr10	71799498	71799789	292	71799680	15	6.52813	3.80414
Nc-1-IP_pe	chr10	71805728	71806307	580	71805799	18	4.32723	2.54542
Nc-1-IP_pe	chr10	71807760	71808182	423	71808107	12	3.79003	2.71687
Nc-1-IP_pe	chr10	71810494	71810826	333	71810584	15	5.01987	3.06184
Nc-1-IP_pe	chr10	71811345	71813387	2043	71812732	20	6.60733	3.30311
Nc-1-IP_pe	chr10	71815388	71815752	365	71815627	13	3.6894	2.58919
Nc-1-IP_pe	chr10	71817660	71818129	470	71817759	14	4.21449	2.76599
Nc-1-IP_pe	chr10	71818919	71819384	466	71819138	18	6.91333	3.62993
Nc-1-IP_pe	chr10	71821809	71822226	418	71822046	17	7.22981	3.89549
Nc-1-IP_pe	chr10	71824045	71824405	361	71824243	17	5.14803	2.94914
Nc-1-IP_pe	chr10	71825261	71825588	328	71825450	18	8.27052	4.2634
Nc-1-IP_pe	chr10	71850846	71851143	298	71850982	16	4.96462	2.94973
Nc-1-IP_pe	chr10	71851673	71852035	363	71851770	14	5.53803	3.41887
Nc-1-IP_pe	chr10	71852351	71853049	699	71852739	16	6.8987	3.86161
Nc-1-IP_pe	chr10	71869113	71869365	253	71869142	14	3.55308	2.45762
Nc-1-IP_pe	chr10	71874179	71874918	740	71874519	14	5.32037	3.30811
Nc-1-IP_pe	chr10	71875947	71876609	663	71876269	20	8.76288	4.22109
Nc-1-IP_pe	chr10	71891495	71891933	439	71891699	15	4.06016	2.62146
Nc-1-IP_pe	chr10	71899731	71901162	1432	71900993	17	7.6273	4.08876
Nc-1-IP_pe	chr10	71906981	71907504	524	71907257	21	10.8379	5.03418
Nc-1-IP_pe	chr10	71915179	71915677	499	71915506	13	4.86362	3.18015
Nc-1-IP_pe	chr10	71921659	71922033	375	71922021	14	4.73128	3.01506
Nc-1-IP_pe	chr10	71943372	71943655	284	71943518	13	5.86991	3.71823
Nc-1-IP_pe	chr10	71944028	71944308	281	71944164	14	5.838	3.57373
Nc-1-IP_pe	chr10	71955515	71955789	275	71955617	14	4.21449	2.76599
Nc-1-IP_pe	chr10	71956775	71957145	371	71957027	12	4.70523	3.20273
Nc-1-IP_pe	chr10	71971113	71971757	645	71971514	18	4.32723	2.54542
Nc-1-IP_pe	chr10	71972212	71972483	272	71972312	17	5.7822	3.22586
Nc-1-IP_pe	chr10	71974846	71975213	368	71974893	14	3.26761	2.32785
Nc-1-IP_pe	chr10	71975644	71975899	256	71975780	18	5.72638	3.11298
Nc-1-IP_pe	chr10	71979223	71979547	325	71979345	14	5.09034	3.19251
Nc-1-IP_pe	chr10	71982562	71983301	740	71983087	14	5.18557	3.24018

Nc-1-IP_pe	chr10	71985150	71985416	267	71985257	15	6.29054	3.68307
Nc-1-IP_pe	chr10	71987973	71988565	593	71988427	17	7.6273	4.08876
Nc-1-IP_pe	chr10	71997771	71998061	291	71997928	16	5.59722	3.23714
Nc-1-IP_pe	chr10	71998484	71998895	412	71998505	16	3.1497	2.1782
Nc-1-IP_pe	chr10	72007348	72007679	332	72007483	20	8.92783	4.2957
Nc-1-IP_pe	chr10	72008085	72008489	405	72008285	15	4.06016	2.62146
Nc-1-IP_pe	chr10	72012194	72012725	532	72012553	14	5.18557	3.24018
Nc-1-IP_pe	chr10	72034205	72034477	273	72034396	14	5.30793	3.30182
Nc-1-IP_pe	chr10	72035785	72036079	295	72036077	14	3.00733	2.21111
Nc-1-IP_pe	chr10	72039914	72040687	774	72040466	20	8.76288	4.22109
Nc-1-IP_pe	chr10	72044443	72045048	606	72044638	18	7.1522	3.73811
Nc-1-IP_pe	chr10	72052218	72052883	666	72052643	13	5.04465	3.2748
Nc-1-IP_pe	chr10	72053629	72053965	337	72053692	13	3.77558	2.63125
Nc-1-IP_pe	chr10	72058223	72058574	352	72058322	14	5.22188	3.25843
Nc-1-IP_pe	chr10	72059918	72060448	531	72060204	14	5.25853	3.27688
Nc-1-IP_pe	chr10	72061408	72061959	552	72061888	15	5.00915	3.05678
Nc-1-IP_pe	chr10	72062703	72063606	904	72062884	18	8.10208	4.1823
Nc-1-IP_pe	chr10	72064084	72064664	581	72064192	17	4.44438	2.65333
Nc-1-IP_pe	chr10	72067511	72068565	1055	72068012	17	6.47874	3.54137
Nc-1-IP_pe	chr10	72070229	72070509	281	72070425	15	5.20142	3.14789
Nc-1-IP_pe	chr10	72071061	72071435	375	72071120	13	4.04902	2.76601
Nc-1-IP_pe	chr10	72072380	72072640	261	72072539	14	3.00733	2.21111
Nc-1-IP_pe	chr10	72074692	72075123	432	72074924	18	7.02099	3.67851
Nc-1-IP_pe	chr10	72084154	72084493	340	72084364	13	3.07238	2.29378
Nc-1-IP_pe	chr10	72085833	72086105	273	72085932	14	5.03198	3.16342
Nc-1-IP_pe	chr10	72086509	72086795	287	72086581	17	7.52577	4.03901
Nc-1-IP_pe	chr10	72087943	72088633	691	72088289	14	4.04994	2.68821
Nc-1-IP_pe	chr10	72103167	72103667	501	72103279	11	3.64839	2.72584
Nc-1-IP_pe	chr10	72105092	72105404	313	72105267	13	5.36853	3.44651
Nc-1-IP_pe	chr10	72111230	72111991	762	72111807	18	11.6772	6.04507
Nc-1-IP_pe	chr10	72119952	72120205	254	72120011	13	5.15976	3.33548
Nc-1-IP_pe	chr10	72135260	72135695	436	72135332	12	4.24064	2.95299
Nc-1-IP_pe	chr10	72136640	72136973	334	72136910	10	4.68196	3.42446
Nc-1-IP_pe	chr10	72156104	72156653	550	72156231	16	10.117	5.58955
Nc-1-IP_pe	chr10	72188916	72189331	416	72189033	9	4.21839	3.26234
Nc-1-IP_pe	chr10	72205210	72206038	829	72205715	13	6.36468	3.99316
Nc-1-IP_pe	chr10	72208987	72209687	701	72209533	12	4.24064	2.95299
Nc-1-IP_pe	chr10	72234905	72235270	366	72235029	18	8.78911	4.51742
Nc-1-IP_pe	chr10	72241329	72241636	308	72241633	12	2.87625	2.25568
Nc-1-IP_pe	chr10	72242698	72243011	314	72242859	18	6.15736	3.29676
Nc-1-IP_pe	chr10	72246841	72247117	277	72246970	13	4.77734	3.13537
Nc-1-IP_pe	chr10	72255077	72255901	825	72255334	16	5.82808	3.34463
Nc-1-IP_pe	chr10	72262552	72263015	464	72262603	12	4.18351	2.92273
Nc-1-IP_pe	chr10	72265767	72266641	875	72266146	19	9.22155	4.57653
Nc-1-IP_pe	chr10	72267699	72268300	602	72267823	18	8.51506	4.38236

Nc-1-IP_pe	chr10	72270720	72271212	493	72271134	16	5.83378	3.3473
Nc-1-IP_pe	chr10	72274054	72274443	390	72274349	22	6.72623	3.18481
Nc-1-IP_pe	chr10	72282248	72282514	267	72282292	12	4.79401	3.25118
Nc-1-IP_pe	chr10	72286137	72286786	650	72286581	12	4.24064	2.95299
Nc-1-IP_pe	chr10	72287191	72287730	540	72287279	13	4.02562	2.7544
Nc-1-IP_pe	chr10	72290741	72291014	274	72290852	13	4.67336	3.0817
Nc-1-IP_pe	chr10	72296635	72297228	594	72297201	15	4.32392	2.74012
Nc-1-IP_pe	chr10	72297700	72298033	334	72297831	21	7.72183	3.65936
Nc-1-IP_pe	chr10	72303671	72304173	503	72303765	15	5.20142	3.14789
Nc-1-IP_pe	chr10	72310752	72311053	302	72310949	15	4.23553	2.70016
Nc-1-IP_pe	chr10	72317142	72317441	300	72317338	17	6.13258	3.38303
Nc-1-IP_pe	chr10	72319471	72320006	536	72319612	17	6.59042	3.59311
Nc-1-IP_pe	chr10	72321033	72322422	1390	72321562	18	7.15875	3.7411
Nc-1-IP_pe	chr10	72323254	72323641	388	72323325	15	4.7819	2.95039
Nc-1-IP_pe	chr10	72323958	72324403	446	72324083	18	4.6358	2.66698
Nc-1-IP_pe	chr10	72325668	72326095	428	72325963	19	5.90435	3.10381
Nc-1-IP_pe	chr10	72330685	72330937	253	72330837	16	5.11827	3.01859
Nc-1-IP_pe	chr10	72333427	72333697	271	72333558	17	5.90762	3.28176
Nc-1-IP_pe	chr10	72334897	72335516	620	72334979	15	4.40338	2.77622
Nc-1-IP_pe	chr10	72336258	72336585	328	72336508	15	4.06504	2.62364
Nc-1-IP_pe	chr10	72340589	72340856	268	72340708	17	5.35342	3.03768
Nc-1-IP_pe	chr10	72345120	72345428	309	72345267	13	4.14549	2.81406
Nc-1-IP_pe	chr10	72346348	72346750	403	72346588	19	7.84735	3.93486
Nc-1-IP_pe	chr10	72354154	72355050	897	72354799	40	22.6623	6.71749
Nc-1-IP_pe	chr10	72355643	72355914	272	72355779	16	6.84901	3.83694
Nc-1-IP_pe	chr10	72364125	72364398	274	72364334	14	4.42328	2.86573
Nc-1-IP_pe	chr10	72367945	72368260	316	72368057	14	5.838	3.57373
Nc-1-IP_pe	chr10	72371241	72371596	356	72371306	16	8.37012	4.62105
Nc-1-IP_pe	chr10	72385113	72385476	364	72385364	13	6.84576	4.26658
Nc-1-IP_pe	chr10	72386146	72386417	272	72386162	11	3.64839	2.72584
Nc-1-IP_pe	chr10	72393663	72393948	286	72393824	15	6.24734	3.66122
Nc-1-IP_pe	chr10	72435730	72436220	491	72435778	16	4.59224	2.7853
Nc-1-IP_pe	chr10	72454369	72454621	253	72454425	10	3.50482	2.73328
Nc-1-IP_pe	chr10	72472765	72473081	317	72472904	12	6.82053	4.41531
Nc-1-IP_pe	chr10	72482919	72483739	821	72483612	15	7.46994	4.29894
Nc-1-IP_pe	chr10	72496169	72496681	513	72496369	12	4.51041	3.09723
Nc-1-IP_pe	chr10	72546793	72547297	505	72546867	14	5.98179	3.64885
Nc-1-IP_pe	chr10	72586216	72586605	390	72586502	12	5.54793	3.67174
Nc-1-IP_pe	chr10	72617689	72618030	342	72617874	11	3.64839	2.72584
Nc-1-IP_pe	chr10	72623476	72623887	412	72623735	16	7.8853	4.36474
Nc-1-IP_pe	chr10	72651240	72651532	293	72651372	14	6.06519	3.69269
Nc-1-IP_pe	chr10	72652359	72652633	275	72652541	14	7.12806	4.26787
Nc-1-IP_pe	chr10	72656195	72656926	732	72656601	15	5.20142	3.14789
Nc-1-IP_pe	chr10	72661959	72662511	553	72662285	14	4.8399	3.06836
Nc-1-IP_pe	chr10	72682650	72682931	282	72682888	14	5.03085	3.16286

Nc-1-IP_pe	chr10	72692247	72692784	538	72692636	20	6.08336	3.09555
Nc-1-IP_pe	chr10	72711008	72711496	489	72711179	10	3.4604	2.70797
Nc-1-IP_pe	chr10	72751414	72752070	657	72751605	13	5.86991	3.71823
Nc-1-IP_pe	chr10	72755951	72756277	327	72756090	12	5.5322	3.6628
Nc-1-IP_pe	chr10	72786483	72786737	255	72786527	12	5.59553	3.69882
Nc-1-IP_pe	chr10	72787276	72787546	271	72787416	15	8.25154	4.72672
Nc-1-IP_pe	chr10	72806194	72806488	295	72806405	13	4.41954	2.952
Nc-1-IP_pe	chr10	72823855	72824129	275	72823990	11	4.54404	3.2242
Nc-1-IP_pe	chr10	72862233	72862834	602	72862700	18	10.2908	5.28894
Nc-1-IP_pe	chr10	72870279	72870608	330	72870403	10	5.23357	3.76063
Nc-1-IP_pe	chr10	72895512	72895817	306	72895622	18	8.37884	4.31591
Nc-1-IP_pe	chr10	72928870	72929131	262	72929107	10	3.89064	2.95552
Nc-1-IP_pe	chr10	72947816	72948135	320	72947957	13	4.41954	2.952
Nc-1-IP_pe	chr10	72967474	72967955	482	72967803	13	5.15976	3.33548
Nc-1-IP_pe	chr10	72980566	72981102	537	72980690	14	7.50175	4.47701
Nc-1-IP_pe	chr10	72981490	72981934	445	72981618	12	3.77112	2.7071
Nc-1-IP_pe	chr10	73017964	73019003	1040	73018808	12	3.83688	2.74114
Nc-1-IP_pe	chr10	73049923	73050187	265	73050025	12	5.84943	3.84431
Nc-1-IP_pe	chr10	73096146	73097178	1033	73096242	15	4.7819	2.95039
Nc-1-IP_pe	chr10	73112072	73112367	296	73112224	16	9.29734	5.12684
Nc-1-IP_pe	chr10	73117073	73117527	455	73117275	12	6.6717	4.32642
Nc-1-IP_pe	chr10	73120610	73120887	278	73120748	14	6.69566	4.03025
Nc-1-IP_pe	chr10	73125124	73125481	358	73125214	10	4.39571	3.25288
Nc-1-IP_pe	chr10	73155083	73155694	612	73155302	14	8.22927	4.89351
Nc-1-IP_pe	chr10	73167826	73168456	631	73168362	23	7.54072	3.40476
Nc-1-IP_pe	chr10	73175202	73175584	383	73175379	12	6.86906	4.44438
Nc-1-IP_pe	chr10	73190896	73191669	774	73191206	12	3.16137	2.39719
Nc-1-IP_pe	chr10	73206553	73207813	1261	73206689	15	6.19428	3.63445
Nc-1-IP_pe	chr10	73265027	73265355	329	73265096	14	7.9745	4.74633
Nc-1-IP_pe	chr10	73312956	73313248	293	73313103	14	7.42849	4.43574
Nc-1-IP_pe	chr10	73342907	73343195	289	73343020	14	5.51541	3.4073
Nc-1-IP_pe	chr10	73345630	73345890	261	73345834	14	5.03085	3.16286
Nc-1-IP_pe	chr10	73349317	73349594	278	73349464	13	7.38599	4.58019
Nc-1-IP_pe	chr10	73351262	73351595	334	73351375	11	3.64839	2.72584
Nc-1-IP_pe	chr10	73351857	73352114	258	73352000	14	6.61011	3.98382
Nc-1-IP_pe	chr10	73365289	73365617	329	73365418	18	6.15736	3.29676
Nc-1-IP_pe	chr10	73394549	73394804	256	73394658	16	10.264	5.67394
Nc-1-IP_pe	chr10	73399588	73400086	499	73399603	11	4.06403	2.95415
Nc-1-IP_pe	chr10	73404941	73405371	431	73405331	12	4.70082	3.20033
Nc-1-IP_pe	chr10	73412107	73412402	296	73412223	14	4.60011	2.95114
Nc-1-IP_pe	chr10	73417197	73417519	323	73417323	13	4.04902	2.76601
Nc-1-IP_pe	chr10	73528426	73528783	358	73528599	14	6.78215	4.0774
Nc-1-IP_pe	chr10	73559206	73559485	280	73559220	8	3.35977	2.83231
Nc-1-IP_pe	chr10	73567037	73567548	512	73567491	14	4.60011	2.95114
Nc-1-IP_pe	chr10	73573140	73573531	392	73573517	10	3.4604	2.70797

Nc-1-IP_pe	chr10	73584120	73584381	262	73584279	14	7.12806	4.26787
Nc-1-IP_pe	chr10	73613228	73613904	677	73613632	15	8.38325	4.80024
Nc-1-IP_pe	chr10	73618194	73618712	519	73618304	13	4.86362	3.18015
Nc-1-IP_pe	chr10	73625805	73626302	498	73626178	18	5.33197	2.94862
Nc-1-IP_pe	chr10	73629656	73630151	496	73629790	16	7.44163	4.13536
Nc-1-IP_pe	chr10	73632901	73633443	543	73633050	17	7.02067	3.79543
Nc-1-IP_pe	chr10	73641279	73641755	477	73641454	15	5.83691	3.4562
Nc-1-IP_pe	chr10	73646905	73648205	1301	73648099	19	7.05262	3.58428
Nc-1-IP_pe	chr10	73663450	73663811	362	73663655	25	11.1571	4.58851
Nc-1-IP_pe	chr10	73664291	73664788	498	73664692	20	6.72028	3.34863
Nc-1-IP_pe	chr10	73667973	73668280	308	73668055	16	5.37454	3.13479
Nc-1-IP_pe	chr10	73679037	73679357	321	73679305	13	4.65065	3.07002
Nc-1-IP_pe	chr10	73704555	73704810	256	73704693	15	6.78905	3.93887
Nc-1-IP_pe	chr10	73707268	73708478	1211	73708027	19	6.32622	3.27682
Nc-1-IP_pe	chr10	73715189	73715650	462	73715561	10	3.08907	2.49869
Nc-1-IP_pe	chr10	73730184	73730795	612	73730481	35	20.1905	6.63838
Nc-1-IP_pe	chr10	73731698	73732668	971	73732218	20	7.45226	3.65043
Nc-1-IP_pe	chr10	73733072	73733515	444	73733299	12	5.39344	3.58426
Nc-1-IP_pe	chr10	73734229	73735135	907	73734885	19	5.90435	3.10381
Nc-1-IP_pe	chr10	73743963	73744611	649	73744429	18	7.1522	3.73811
Nc-1-IP_pe	chr10	73747893	73748474	582	73748093	13	5.99619	3.78778
Nc-1-IP_pe	chr10	73757849	73758223	375	73758092	12	4.64316	3.169
Nc-1-IP_pe	chr10	73774331	73774791	461	73774753	11	3.89191	2.85898
Nc-1-IP_pe	chr10	73781775	73782371	597	73782191	19	3.26827	2.10398
Nc-1-IP_pe	chr10	73785575	73786334	760	73785893	20	9.16637	4.40471
Nc-1-IP_pe	chr10	73791193	73791646	454	73791527	12	3.75233	2.69739
Nc-1-IP_pe	chr10	73792024	73792662	639	73792240	19	6.56181	3.3752
Nc-1-IP_pe	chr10	73796976	73797596	621	73797509	14	5.57768	3.4392
Nc-1-IP_pe	chr10	73799496	73799815	320	73799638	14	5.68531	3.49459
Nc-1-IP_pe	chr10	73800954	73801597	644	73801122	14	5.69896	3.50164
Nc-1-IP_pe	chr10	73806389	73806780	392	73806711	14	4.21449	2.76599
Nc-1-IP_pe	chr10	73807667	73807953	287	73807788	15	5.5956	3.33782
Nc-1-IP_pe	chr10	73808347	73808686	340	73808546	18	6.15736	3.29676
Nc-1-IP_pe	chr10	73810834	73811931	1098	73811596	30	10.9504	3.972
Nc-1-IP_pe	chr10	73814865	73815403	539	73814994	20	8.82834	4.25062
Nc-1-IP_pe	chr10	73816718	73817358	641	73816853	16	6.61333	3.72083
Nc-1-IP_pe	chr10	73817626	73817905	280	73817844	16	4.59224	2.7853
Nc-1-IP_pe	chr10	73818258	73818816	559	73818785	17	4.78101	2.79343
Nc-1-IP_pe	chr10	73823922	73824945	1024	73824571	15	5.43804	3.26139
Nc-1-IP_pe	chr10	73826546	73827098	553	73826706	20	5.01743	2.69071
Nc-1-IP_pe	chr10	73827945	73828233	289	73828019	17	4.78101	2.79343
Nc-1-IP_pe	chr10	73836266	73836953	688	73836604	19	8.35089	4.16476
Nc-1-IP_pe	chr10	73838853	73840207	1355	73839960	16	4.68897	2.82769
Nc-1-IP_pe	chr10	73840651	73841401	751	73841233	16	4.96462	2.94973
Nc-1-IP_pe	chr10	73842025	73843222	1198	73842492	18	5.33197	2.94862

Nc-1-IP_pe	chr10	73847745	73848365	621	73847951	14	5.06689	3.18081
Nc-1-IP_pe	chr10	73848689	73849088	400	73849018	16	5.07731	3.00018
Nc-1-IP_pe	chr10	73850169	73850607	439	73850286	13	4.02562	2.7544
Nc-1-IP_pe	chr10	73852994	73853791	798	73853080	14	4.85093	3.07379
Nc-1-IP_pe	chr10	73858253	73858665	413	73858496	13	4.40998	2.94715
Nc-1-IP_pe	chr10	73859831	73860115	285	73860043	13	4.02562	2.7544
Nc-1-IP_pe	chr10	73866876	73867241	366	73867225	15	3.67238	2.45006
Nc-1-IP_pe	chr10	73867619	73868393	775	73868349	18	6.71734	3.54221
Nc-1-IP_pe	chr10	73871360	73871616	257	73871403	14	4.21449	2.76599
Nc-1-IP_pe	chr10	73872452	73872906	455	73872684	20	8.14433	3.94687
Nc-1-IP_pe	chr10	73874265	73874545	281	73874383	18	7.47506	3.88659
Nc-1-IP_pe	chr10	73875992	73876559	568	73876455	15	5.49794	3.29036
Nc-1-IP_pe	chr10	73911049	73911505	457	73911212	20	6.38846	3.2157
Nc-1-IP_pe	chr10	73913634	73914172	539	73913783	14	4.52309	2.91384
Nc-1-IP_pe	chr10	73916423	73916817	395	73916633	14	4.60011	2.95114
Nc-1-IP_pe	chr10	73921940	73922409	470	73921995	13	4.41954	2.952
Nc-1-IP_pe	chr10	73927017	73927563	547	73927394	15	5.66913	3.37372
Nc-1-IP_pe	chr10	73933094	73933389	296	73933269	14	5.37054	3.33352
Nc-1-IP_pe	chr10	73933837	73934098	262	73933955	17	7.02067	3.79543
Nc-1-IP_pe	chr10	73942203	73942461	259	73942349	14	5.06689	3.18081
Nc-1-IP_pe	chr10	73947261	73947681	421	73947422	12	4.24064	2.95299
Nc-1-IP_pe	chr10	73959182	73959606	425	73959380	15	5.20142	3.14789
Nc-1-IP_pe	chr10	73965211	73965689	479	73965305	16	4.96462	2.94973
Nc-1-IP_pe	chr10	73976237	73976541	305	73976411	16	5.82808	3.34463
Nc-1-IP_pe	chr10	73986126	73986523	398	73986413	13	6.48901	4.06327
Nc-1-IP_pe	chr10	73996227	73996563	337	73996436	15	6.29054	3.68307
Nc-1-IP_pe	chr10	73998672	73999427	756	73998764	19	6.22205	3.23373
Nc-1-IP_pe	chr10	74015492	74015985	494	74015784	13	6.7109	4.18935
Nc-1-IP_pe	chr10	74016289	74016632	344	74016508	15	8.26005	4.73146
Nc-1-IP_pe	chr10	74017117	74017429	313	74017246	17	10.2686	5.47119
Nc-1-IP_pe	chr10	74046236	74046529	294	74046316	10	4.39571	3.25288
Nc-1-IP_pe	chr10	74054673	74055221	549	74055155	15	7.46994	4.29894
Nc-1-IP_pe	chr10	74062728	74063121	394	74062794	11	4.54404	3.2242
Nc-1-IP_pe	chr10	74065174	74065652	479	74065538	13	7.23402	4.49131
Nc-1-IP_pe	chr10	74094308	74094577	270	74094423	16	8.17084	4.51499
Nc-1-IP_pe	chr10	74117450	74117819	370	74117460	11	3.81796	2.81836
Nc-1-IP_pe	chr10	74132967	74133926	960	74133192	16	6.33285	3.58457
Nc-1-IP_pe	chr10	74139859	74140286	428	74140216	14	3.55308	2.45762
Nc-1-IP_pe	chr10	74155215	74155474	260	74155411	16	7.97877	4.4137
Nc-1-IP_pe	chr10	74161809	74162115	307	74162098	11	3.28475	2.53029
Nc-1-IP_pe	chr10	74170516	74170797	282	74170634	14	7.42849	4.43574
Nc-1-IP_pe	chr10	74173265	74173707	443	74173329	11	3.64839	2.72584
Nc-1-IP_pe	chr10	74176679	74177287	609	74176802	18	4.04135	2.43445
Nc-1-IP_pe	chr10	74197386	74197682	297	74197550	11	5.68685	3.89286
Nc-1-IP_pe	chr10	74206917	74207180	264	74207062	14	5.03085	3.16286

Nc-1-IP_pe	chr10	74207953	74208281	329	74208136	15	4.7819	2.95039
Nc-1-IP_pe	chr10	74216966	74217364	399	74217043	12	4.70082	3.20033
Nc-1-IP_pe	chr10	74231508	74231770	263	74231686	11	6.03439	4.10251
Nc-1-IP_pe	chr10	74232429	74232698	270	74232618	7	3.06442	2.73501
Nc-1-IP_pe	chr10	74242853	74243285	433	74243145	14	7.14701	4.27839
Nc-1-IP_pe	chr10	74273517	74273784	268	74273605	13	5.36853	3.44651
Nc-1-IP_pe	chr10	74283791	74284421	631	74283955	13	3.07238	2.29378
Nc-1-IP_pe	chr10	74288365	74288679	315	74288538	13	7.63554	4.7272
Nc-1-IP_pe	chr10	74363272	74363903	632	74363428	14	4.60011	2.95114
Nc-1-IP_pe	chr10	74384346	74384599	254	74384488	10	4.39571	3.25288
Nc-1-IP_pe	chr10	74403158	74404082	925	74403553	12	6.31185	4.11348
Nc-1-IP_pe	chr10	74423400	74423797	398	74423751	13	4.41954	2.952
Nc-1-IP_pe	chr10	74474710	74474972	263	74474883	14	6.61011	3.98382
Nc-1-IP_pe	chr10	74507325	74507608	284	74507494	12	5.8598	3.85029
Nc-1-IP_pe	chr10	74515371	74516323	953	74515639	21	10.8379	5.03418
Nc-1-IP_pe	chr10	74550101	74550369	269	74550188	14	5.43414	3.36584
Nc-1-IP_pe	chr10	74580234	74580560	327	74580424	11	4.06403	2.95415
Nc-1-IP_pe	chr10	74598407	74598659	253	74598579	13	7.34205	4.55444
Nc-1-IP_pe	chr10	74608888	74609666	779	74609355	16	2.92499	2.08721
Nc-1-IP_pe	chr10	74648420	74649120	701	74648539	18	5.72638	3.11298
Nc-1-IP_pe	chr10	74655475	74655774	300	74655769	15	3.46204	2.35851
Nc-1-IP_pe	chr10	74660324	74660603	280	74660536	12	4.70082	3.20033
Nc-1-IP_pe	chr10	74668455	74668793	339	74668675	16	6.33285	3.58457
Nc-1-IP_pe	chr10	74682656	74682943	288	74682748	12	4.70082	3.20033
Nc-1-IP_pe	chr10	74686721	74687038	318	74686873	19	10.7389	5.33625
Nc-1-IP_pe	chr10	74688907	74689226	320	74689066	17	6.47874	3.54137
Nc-1-IP_pe	chr10	74707636	74708006	371	74707948	13	4.56123	3.02417
Nc-1-IP_pe	chr10	74711803	74712438	636	74712317	14	6.69531	4.03006
Nc-1-IP_pe	chr10	74744454	74744712	259	74744634	13	3.67377	2.58159
Nc-1-IP_pe	chr10	74749882	74750212	331	74750019	14	5.12578	3.21022
Nc-1-IP_pe	chr10	74753184	74753570	387	74753511	16	4.96462	2.94973
Nc-1-IP_pe	chr10	74759172	74759981	810	74759517	13	5.76252	3.65944
Nc-1-IP_pe	chr10	74761427	74761727	301	74761572	12	4.68027	3.18915
Nc-1-IP_pe	chr10	74767173	74767595	423	74767343	12	3.96559	2.80816
Nc-1-IP_pe	chr10	74771721	74772850	1130	74772155	13	5.85439	3.70972
Nc-1-IP_pe	chr10	74790309	74790668	360	74790387	11	2.96397	2.36091
Nc-1-IP_pe	chr10	74802342	74802650	309	74802508	15	5.66913	3.37372
Nc-1-IP_pe	chr10	74805979	74806298	320	74806056	13	5.15976	3.33548
Nc-1-IP_pe	chr10	74809399	74809936	538	74809500	12	3.83688	2.74114
Nc-1-IP_pe	chr10	74811772	74812168	397	74811871	11	4.58343	3.24666
Nc-1-IP_pe	chr10	74813213	74813736	524	74813605	15	6.39297	3.73508
Nc-1-IP_pe	chr10	74814242	74814638	397	74814414	18	9.1091	4.6774
Nc-1-IP_pe	chr10	74815290	74816007	718	74815436	16	8.21011	4.53581
Nc-1-IP_pe	chr10	74818699	74819140	442	74818869	21	11.1811	5.19865
Nc-1-IP_pe	chr10	74825012	74825738	727	74825201	25	9.73912	4.03473

Nc-1-IP_pe	chr10	74834211	74834706	496	74834653	14	4.21449	2.76599
Nc-1-IP_pe	chr10	74841239	74841492	254	74841353	13	3.07238	2.29378
Nc-1-IP_pe	chr10	74843947	74844471	525	74844177	16	8.49334	4.68711
Nc-1-IP_pe	chr10	74848001	74848323	323	74848113	16	8.66212	4.77818
Nc-1-IP_pe	chr10	74858563	74858870	308	74858797	17	6.78076	3.68203
Nc-1-IP_pe	chr10	74860744	74861134	391	74860901	18	9.76263	5.01166
Nc-1-IP_pe	chr10	74864113	74864775	663	74864233	18	9.61048	4.93296
Nc-1-IP_pe	chr10	74870667	74871425	759	74870796	17	7.6273	4.08876
Nc-1-IP_pe	chr10	74880748	74881266	519	74881226	18	4.04135	2.43445
Nc-1-IP_pe	chr10	74887294	74888040	747	74887500	14	4.21449	2.76599
Nc-1-IP_pe	chr10	74892925	74893221	297	74893066	19	7.29128	3.68799
Nc-1-IP_pe	chr10	74903239	74903651	413	74903521	13	4.45253	2.96876
Nc-1-IP_pe	chr10	74931703	74931996	294	74931828	17	7.02067	3.79543
Nc-1-IP_pe	chr10	74952060	74952427	368	74952227	17	6.47874	3.54137
Nc-1-IP_pe	chr10	74975547	74975969	423	74975846	11	5.74187	3.92588
Nc-1-IP_pe	chr10	74987079	74987365	287	74987252	16	5.82808	3.34463
Nc-1-IP_pe	chr10	74996388	74996653	266	74996545	16	6.8987	3.86161
Nc-1-IP_pe	chr10	75018102	75018518	417	75018488	15	2.95965	2.14351
Nc-1-IP_pe	chr10	75029088	75030147	1060	75029906	26	10.439	4.19014
Nc-1-IP_pe	chr10	75030607	75031098	492	75030970	17	8.31887	4.43455
Nc-1-IP_pe	chr10	75033529	75034085	557	75033932	22	8.79433	3.99081
Nc-1-IP_pe	chr10	75034982	75035352	371	75035081	14	4.60011	2.95114
Nc-1-IP_pe	chr10	75043737	75044009	273	75043869	21	6.2632	3.08808
Nc-1-IP_pe	chr10	75052838	75053144	307	75053071	13	4.67336	3.0817
Nc-1-IP_pe	chr10	75070642	75071078	437	75070926	18	5.33158	2.94846
Nc-1-IP_pe	chr10	75073567	75073982	416	75073796	15	4.40338	2.77622
Nc-1-IP_pe	chr10	75077275	75077569	295	75077391	17	6.47874	3.54137
Nc-1-IP_pe	chr10	75079467	75079800	334	75079543	16	6.33285	3.58457
Nc-1-IP_pe	chr10	75087730	75088157	428	75087931	19	5.32483	2.87252
Nc-1-IP_pe	chr10	75092640	75093206	567	75092735	14	5.27082	3.28308
Nc-1-IP_pe	chr10	75094527	75095705	1179	75094818	18	7.11704	3.7221
Nc-1-IP_pe	chr10	75100101	75101157	1057	75100140	21	4.90204	2.59278
Nc-1-IP_pe	chr10	75103224	75104300	1077	75103383	23	8.39	3.72457
Nc-1-IP_pe	chr10	75104947	75105888	942	75105103	21	5.5349	2.81884
Nc-1-IP_pe	chr10	75107632	75108309	678	75107858	20	7.06192	3.48801
Nc-1-IP_pe	chr10	75111779	75112044	266	75111858	25	6.62684	2.94607
Nc-1-IP_pe	chr10	75128161	75128471	311	75128298	16	7.53857	4.18505
Nc-1-IP_pe	chr10	75134312	75134624	313	75134575	14	5.43414	3.36584
Nc-1-IP_pe	chr10	75135276	75135820	545	75135385	13	4.41954	2.952
Nc-1-IP_pe	chr10	75146189	75146484	296	75146229	12	3.16137	2.39719
Nc-1-IP_pe	chr10	75152716	75153100	385	75152738	10	3.92601	2.97611
Nc-1-IP_pe	chr10	75161930	75162260	331	75162259	12	2.87625	2.25568
Nc-1-IP_pe	chr10	75169210	75169842	633	75169664	16	7.42466	4.12669
Nc-1-IP_pe	chr10	75187462	75187910	449	75187606	17	6.45883	3.53218
Nc-1-IP_pe	chr10	75189206	75189614	409	75189353	23	7.4429	3.36882

Nc-1-IP_pe	chr10	75193513	75194440	928	75194349	17	5.9911	3.31919
Nc-1-IP_pe	chr10	75200306	75200743	438	75200388	16	5.30773	3.10433
Nc-1-IP_pe	chr10	75210531	75211422	892	75211092	30	11.763	4.24182
Nc-1-IP_pe	chr10	75218178	75218721	544	75218347	17	7.65556	4.10265
Nc-1-IP_pe	chr10	75232231	75232485	255	75232311	17	5.14803	2.94914
Nc-1-IP_pe	chr10	75232898	75233261	364	75233087	19	6.58528	3.38508
Nc-1-IP_pe	chr10	75235395	75236462	1068	75235786	26	7.95585	3.31498
Nc-1-IP_pe	chr10	75237939	75238965	1027	75238100	20	4.71088	2.57831
Nc-1-IP_pe	chr10	75239508	75240032	525	75239924	15	4.67074	2.89885
Nc-1-IP_pe	chr10	75240435	75240701	267	75240625	14	4.38411	2.84693
Nc-1-IP_pe	chr10	75242054	75242621	568	75242503	17	5.80475	3.23588
Nc-1-IP_pe	chr10	75244678	75245110	433	75244972	23	8.87435	3.91328
Nc-1-IP_pe	chr10	75248327	75249059	733	75248416	23	5.5873	2.71945
Nc-1-IP_pe	chr10	75255148	75255631	484	75255601	16	2.92499	2.08721
Nc-1-IP_pe	chr10	75257862	75258379	518	75258246	18	8.23643	4.24693
Nc-1-IP_pe	chr10	75264735	75265352	618	75264956	17	5.14803	2.94914
Nc-1-IP_pe	chr10	75275980	75276641	662	75276422	20	8.29312	4.01203
Nc-1-IP_pe	chr10	75281353	75281826	474	75281510	14	4.60011	2.95114
Nc-1-IP_pe	chr10	75283206	75283606	401	75283509	13	4.76564	3.12931
Nc-1-IP_pe	chr10	75293140	75293446	307	75293219	16	5.62031	3.24783
Nc-1-IP_pe	chr10	75293806	75294177	372	75293971	23	10.6882	4.66214
Nc-1-IP_pe	chr10	75294789	75295381	593	75294812	18	2.88361	1.99878
Nc-1-IP_pe	chr10	75332891	75333621	731	75333091	19	10.7389	5.33625
Nc-1-IP_pe	chr10	75333892	75334195	304	75334092	13	3.35769	2.42919
Nc-1-IP_pe	chr10	75343149	75343746	598	75343536	15	5.34394	3.21607
Nc-1-IP_pe	chr10	75344213	75344533	321	75344239	13	3.67377	2.58159
Nc-1-IP_pe	chr10	75347944	75348334	391	75348053	17	5.9911	3.31919
Nc-1-IP_pe	chr10	75352508	75353094	587	75352585	18	6.15736	3.29676
Nc-1-IP_pe	chr10	75356387	75356763	377	75356574	17	5.9911	3.31919
Nc-1-IP_pe	chr10	75372738	75373361	624	75373271	13	4.41954	2.952
Nc-1-IP_pe	chr10	75378172	75378490	319	75378404	17	6.25544	3.43888
Nc-1-IP_pe	chr10	75382984	75383539	556	75383004	14	3.26761	2.32785
Nc-1-IP_pe	chr10	75383807	75385049	1243	75384671	17	7.36791	3.96218
Nc-1-IP_pe	chr10	75386861	75387219	359	75387031	23	11.5921	5.06057
Nc-1-IP_pe	chr10	75387665	75388135	471	75388038	13	4.56123	3.02417
Nc-1-IP_pe	chr10	75389170	75389459	290	75389283	16	6.48567	3.65855
Nc-1-IP_pe	chr10	75395006	75395441	436	75395220	19	3.48582	2.18208
Nc-1-IP_pe	chr10	75395700	75396415	716	75396356	21	5.2077	2.70109
Nc-1-IP_pe	chr10	75398648	75399652	1005	75399367	20	4.71088	2.57831
Nc-1-IP_pe	chr10	75401395	75401779	385	75401583	26	7.71458	3.23541
Nc-1-IP_pe	chr10	75402842	75403614	773	75403365	23	6.62483	3.0751
Nc-1-IP_pe	chr10	75408250	75408829	580	75408636	19	7.31609	3.69885
Nc-1-IP_pe	chr10	75409177	75409869	693	75409436	19	5.51632	2.94814
Nc-1-IP_pe	chr10	75413426	75413800	375	75413642	20	9.21699	4.428
Nc-1-IP_pe	chr10	75418435	75418992	558	75418704	19	7.18275	3.64066

Nc-1-IP_pe	chr10	75430937	75431965	1029	75431732	18	7.73136	4.00629
Nc-1-IP_pe	chr10	75446100	75446358	259	75446141	14	5.61771	3.45976
Nc-1-IP_pe	chr10	75455846	75456284	439	75456073	17	9.42237	5.01057
Nc-1-IP_pe	chr10	75463072	75463404	333	75463320	10	3.83201	2.92147
Nc-1-IP_pe	chr10	75470350	75470619	270	75470460	17	5.9911	3.31919
Nc-1-IP_pe	chr10	75477647	75478161	515	75477986	14	4.60011	2.95114
Nc-1-IP_pe	chr10	75479939	75480439	501	75480012	16	4.59224	2.7853
Nc-1-IP_pe	chr10	75506660	75506934	275	75506842	14	3.86727	2.6027
Nc-1-IP_pe	chr10	75521098	75521656	559	75521307	19	4.8268	2.67939
Nc-1-IP_pe	chr10	75563763	75564648	886	75564461	16	6.65665	3.74206
Nc-1-IP_pe	chr10	75575674	75575958	285	75575794	14	6.06519	3.69269
Nc-1-IP_pe	chr10	75581998	75582559	562	75582420	14	5.51541	3.4073
Nc-1-IP_pe	chr10	75600207	75600483	277	75600330	15	5.20142	3.14789
Nc-1-IP_pe	chr10	75657288	75657557	270	75657457	14	5.03085	3.16286
Nc-1-IP_pe	chr10	75670686	75670949	264	75670877	13	5.9487	3.76157
Nc-1-IP_pe	chr10	75681389	75681674	286	75681525	16	6.33285	3.58457
Nc-1-IP_pe	chr10	75689488	75689749	262	75689550	14	5.51541	3.4073
Nc-1-IP_pe	chr10	75740275	75740651	377	75740495	12	3.47963	2.55766
Nc-1-IP_pe	chr10	75749743	75750342	600	75750185	15	6.5434	3.81197
Nc-1-IP_pe	chr10	75750755	75751206	452	75750938	24	9.05651	3.87976
Nc-1-IP_pe	chr10	75753455	75753821	367	75753575	17	7.6273	4.08876
Nc-1-IP_pe	chr10	75769811	75770074	264	75769902	14	6.90659	4.14556
Nc-1-IP_pe	chr10	75770890	75771587	698	75771079	15	7.02624	4.06292
Nc-1-IP_pe	chr10	75808573	75808879	307	75808707	14	6.26903	3.80064
Nc-1-IP_pe	chr10	75831134	75831753	620	75831321	11	3.3117	2.54465
Nc-1-IP_pe	chr10	75866239	75866534	296	75866283	11	4.47871	3.18705
Nc-1-IP_pe	chr10	75875617	75876045	429	75875697	12	3.83688	2.74114
Nc-1-IP_pe	chr10	75884976	75885277	302	75885220	14	4.95172	3.12357
Nc-1-IP_pe	chr10	75902080	75902428	349	75902271	14	7.54286	4.50023
Nc-1-IP_pe	chr10	75924702	75925089	388	75924719	10	3.08907	2.49869
Nc-1-IP_pe	chr10	75926663	75927044	382	75926980	14	6.96097	4.17548
Nc-1-IP_pe	chr10	75934504	75934800	297	75934649	16	6.8987	3.86161
Nc-1-IP_pe	chr10	75967806	75968177	372	75967832	13	4.86362	3.18015
Nc-1-IP_pe	chr10	75973462	75973738	277	75973682	16	4.25252	2.63824
Nc-1-IP_pe	chr10	75983683	75984003	321	75983779	15	7.0945	4.09889
Nc-1-IP_pe	chr10	75988299	75988846	548	75988419	11	4.47871	3.18705
Nc-1-IP_pe	chr10	75997660	75997977	318	75997829	14	6.61011	3.98382
Nc-1-IP_pe	chr10	76000401	76000679	279	76000556	20	6.49719	3.259
Nc-1-IP_pe	chr10	76003132	76003443	312	76003261	16	8.59821	4.74362
Nc-1-IP_pe	chr10	76013663	76013962	300	76013767	13	5.65792	3.60248
Nc-1-IP_pe	chr10	76017775	76018097	323	76017864	10	4.20113	3.13749
Nc-1-IP_pe	chr10	76018542	76018798	257	76018678	11	4.91882	3.43967
Nc-1-IP_pe	chr10	76030581	76030857	277	76030808	14	5.56442	3.43239
Nc-1-IP_pe	chr10	76033430	76033901	472	76033490	11	4.30316	3.08784
Nc-1-IP_pe	chr10	76056324	76057068	745	76056549	14	3.86727	2.6027

Nc-1-IP_pe	chr10	76085422	76085762	341	76085551	14	6.94277	4.16546
Nc-1-IP_pe	chr10	76107177	76107632	456	76107289	12	3.16137	2.39719
Nc-1-IP_pe	chr10	76129213	76129598	386	76129403	13	5.86991	3.71823
Nc-1-IP_pe	chr10	76150140	76150509	370	76150287	11	4.00079	2.91908
Nc-1-IP_pe	chr10	76171190	76171534	345	76171499	10	3.4604	2.70797
Nc-1-IP_pe	chr10	76186221	76186689	469	76186329	8	3.25229	2.76464
Nc-1-IP_pe	chr10	76226405	76226694	290	76226511	12	5.23082	3.49289
Nc-1-IP_pe	chr10	76251403	76251702	300	76251540	14	5.51541	3.4073
Nc-1-IP_pe	chr10	76298079	76298408	330	76298315	12	4.24064	2.95299
Nc-1-IP_pe	chr10	76303860	76304154	295	76303969	13	6.36468	3.99316
Nc-1-IP_pe	chr10	76316058	76316542	485	76316425	16	6.33285	3.58457
Nc-1-IP_pe	chr10	76432795	76433237	443	76432905	11	4.24272	3.05389
Nc-1-IP_pe	chr10	76464026	76464296	271	76464145	15	7.21633	4.16341
Nc-1-IP_pe	chr10	76492958	76493443	486	76493393	9	4.20361	3.25317
Nc-1-IP_pe	chr10	76514274	76514662	389	76514448	10	5.01973	3.6295
Nc-1-IP_pe	chr10	76543605	76543902	298	76543741	10	4.47495	3.30017
Nc-1-IP_pe	chr10	76554011	76554265	255	76554176	10	3.94999	2.99009
Nc-1-IP_pe	chr10	76604818	76605112	295	76605013	12	5.84943	3.84431
Nc-1-IP_pe	chr10	76675959	76676342	384	76676165	18	8.99859	4.62187
Nc-1-IP_pe	chr10	76686840	76687132	293	76687008	15	7.46994	4.29894
Nc-1-IP_pe	chr10	76766569	76766881	313	76766862	8	3.02956	2.62527
Nc-1-IP_pe	chr10	76775534	76776071	538	76775652	9	3.94978	3.09648
Nc-1-IP_pe	chr10	76815240	76815560	321	76815432	15	6.78905	3.93887
Nc-1-IP_pe	chr10	76820777	76821155	379	76820884	14	5.03085	3.16286
Nc-1-IP_pe	chr10	76846220	76847101	882	76846804	16	3.94146	2.50592
Nc-1-IP_pe	chr10	76857074	76857326	253	76857171	16	9.11744	5.02718
Nc-1-IP_pe	chr10	76890866	76891273	408	76891152	9	3.99054	3.12154
Nc-1-IP_pe	chr10	76893446	76893785	340	76893570	13	7.42513	4.60316
Nc-1-IP_pe	chr10	76938043	76938488	446	76938403	13	5.71736	3.63481
Nc-1-IP_pe	chr10	76941909	76942206	298	76942136	10	3.08907	2.49869
Nc-1-IP_pe	chr10	76967336	76967692	357	76967474	15	6.95893	4.02756
Nc-1-IP_pe	chr10	77014870	77015266	397	77015114	23	8.92223	3.93219
Nc-1-IP_pe	chr10	77084318	77084666	349	77084567	12	4.71777	3.20956
Nc-1-IP_pe	chr10	77091287	77091619	333	77091480	12	3.47963	2.55766
Nc-1-IP_pe	chr10	77107942	77108332	391	77108237	12	4.96504	3.34516
Nc-1-IP_pe	chr10	77140197	77140456	260	77140327	15	4.7819	2.95039
Nc-1-IP_pe	chr10	77152445	77152750	306	77152680	16	6.33285	3.58457
Nc-1-IP_pe	chr10	77157682	77158025	344	77157875	21	8.6741	4.05679
Nc-1-IP_pe	chr10	77169933	77170532	600	77170249	20	11.3299	5.45222
Nc-1-IP_pe	chr10	77171171	77171438	268	77171327	12	3.16137	2.39719
Nc-1-IP_pe	chr10	77173786	77174172	387	77174046	15	6.69889	3.8921
Nc-1-IP_pe	chr10	77175802	77176080	279	77175884	17	6.01283	3.32896
Nc-1-IP_pe	chr10	77197598	77197951	354	77197800	15	7.2695	4.19169
Nc-1-IP_pe	chr10	77225312	77225985	674	77225554	17	4.78101	2.79343
Nc-1-IP_pe	chr10	77233576	77233833	258	77233725	14	7.14701	4.27839

Nc-1-IP_pe	chr10	77238052	77238312	261	77238191	16	4.96462	2.94973
Nc-1-IP_pe	chr10	77260172	77260602	431	77260389	15	6.39297	3.73508
Nc-1-IP_pe	chr10	77274670	77274996	327	77274832	13	6.02837	3.80557
Nc-1-IP_pe	chr10	77312005	77312762	758	77312384	14	6.22239	3.77584
Nc-1-IP_pe	chr10	77325272	77325784	513	77325440	15	4.06016	2.62146
Nc-1-IP_pe	chr10	77338362	77338626	265	77338564	14	4.60011	2.95114
Nc-1-IP_pe	chr10	77359105	77359467	363	77359284	14	3.86727	2.6027
Nc-1-IP_pe	chr10	77372477	77372765	289	77372594	16	7.53857	4.18505
Nc-1-IP_pe	chr10	77375561	77375926	366	77375680	18	7.32895	3.81908
Nc-1-IP_pe	chr10	77390566	77390863	298	77390748	19	8.66703	4.3122
Nc-1-IP_pe	chr10	77392879	77393292	414	77393230	19	6.0077	3.14582
Nc-1-IP_pe	chr10	77400113	77401529	1417	77401392	16	7.72115	4.27929
Nc-1-IP_pe	chr10	77403477	77403992	516	77403870	14	4.21449	2.76599
Nc-1-IP_pe	chr10	77407917	77408294	378	77408225	20	6.94658	3.44067
Nc-1-IP_pe	chr10	77408754	77409591	838	77408899	17	4.96303	2.87025
Nc-1-IP_pe	chr10	77410499	77410835	337	77410744	23	8.38947	3.72437
Nc-1-IP_pe	chr10	77415390	77415856	467	77415782	16	5.73763	3.30235
Nc-1-IP_pe	chr10	77419937	77420327	391	77420153	15	4.40338	2.77622
Nc-1-IP_pe	chr10	77423548	77423947	400	77423674	14	5.68531	3.49459
Nc-1-IP_pe	chr10	77425370	77425676	307	77425500	16	7.39088	4.10945
Nc-1-IP_pe	chr10	77439709	77440167	459	77439828	14	4.52309	2.91384
Nc-1-IP_pe	chr10	77442033	77442529	497	77442079	13	3.67377	2.58159
Nc-1-IP_pe	chr10	77442878	77443646	769	77443130	18	7.73136	4.00629
Nc-1-IP_pe	chr10	77444726	77445276	551	77444856	14	5.80982	3.55907
Nc-1-IP_pe	chr10	77446390	77447209	820	77447026	16	7.56205	4.19712
Nc-1-IP_pe	chr10	77455256	77455730	475	77455353	15	6.19428	3.63445
Nc-1-IP_pe	chr10	77456029	77456283	255	77456211	14	5.53803	3.41887
Nc-1-IP_pe	chr10	77457111	77457470	360	77457240	15	5.20142	3.14789
Nc-1-IP_pe	chr10	77463533	77463800	268	77463673	10	2.95	2.42133
Nc-1-IP_pe	chr10	77466089	77466384	296	77466260	12	2.87625	2.25568
Nc-1-IP_pe	chr10	77472846	77473422	577	77472925	18	8.01971	4.1429
Nc-1-IP_pe	chr10	77473774	77474035	262	77473824	13	4.5945	3.0412
Nc-1-IP_pe	chr10	77474794	77475321	528	77474975	18	6.15736	3.29676
Nc-1-IP_pe	chr10	77487988	77488819	832	77488741	18	5.83846	3.16035
Nc-1-IP_pe	chr10	77495608	77495897	290	77495797	18	5.33197	2.94862
Nc-1-IP_pe	chr10	77501589	77502395	807	77501694	17	5.9911	3.31919
Nc-1-IP_pe	chr10	77504058	77504489	432	77504397	17	7.6885	4.11887
Nc-1-IP_pe	chr10	77516551	77516853	303	77516686	18	5.33197	2.94862
Nc-1-IP_pe	chr10	77538376	77539045	670	77538512	16	5.82808	3.34463
Nc-1-IP_pe	chr10	77540776	77541039	264	77540893	15	6.94226	4.01882
Nc-1-IP_pe	chr10	77545527	77545830	304	77545737	17	6.47874	3.54137
Nc-1-IP_pe	chr10	77552602	77552939	338	77552763	15	6.48264	3.78084
Nc-1-IP_pe	chr10	77553421	77554288	868	77554098	16	6.33285	3.58457
Nc-1-IP_pe	chr10	77564980	77565248	269	77565036	19	5.90435	3.10381
Nc-1-IP_pe	chr10	77566314	77566603	290	77566345	14	4.23163	2.77414

Nc-1-IP_pe	chr10	77567021	77567435	415	77567375	14	4.95172	3.12357
Nc-1-IP_pe	chr10	77575867	77576473	607	77576063	21	9.17453	4.27363
Nc-1-IP_pe	chr10	77578929	77579294	366	77579188	16	6.00716	3.42898
Nc-1-IP_pe	chr10	77590125	77591438	1314	77591239	22	7.27519	3.39036
Nc-1-IP_pe	chr10	77598768	77599070	303	77599003	14	4.21449	2.76599
Nc-1-IP_pe	chr10	77602681	77603706	1026	77603658	15	4.7819	2.95039
Nc-1-IP_pe	chr10	77605093	77605685	593	77605515	23	5.5873	2.71945
Nc-1-IP_pe	chr10	77615261	77615823	563	77615422	15	3.46204	2.35851
Nc-1-IP_pe	chr10	77630048	77630411	364	77630208	14	5.18557	3.24018
Nc-1-IP_pe	chr10	77636876	77637791	916	77637133	23	10.3342	4.51071
Nc-1-IP_pe	chr10	77638350	77639442	1093	77639329	18	7.62523	3.95652
Nc-1-IP_pe	chr10	77642721	77643086	366	77642900	22	10.0534	4.52509
Nc-1-IP_pe	chr10	77649688	77650097	410	77650085	13	3.67377	2.58159
Nc-1-IP_pe	chr10	77660154	77660435	282	77660255	13	5.40091	3.46384
Nc-1-IP_pe	chr10	77681397	77682052	656	77681995	14	5.7958	3.55179
Nc-1-IP_pe	chr10	77686943	77687877	935	77687288	17	4.44438	2.65333
Nc-1-IP_pe	chr10	77696335	77696986	652	77696910	13	5.71736	3.63481
Nc-1-IP_pe	chr10	77701394	77701897	504	77701710	16	6.33285	3.58457
Nc-1-IP_pe	chr10	77709053	77709490	438	77709340	16	6.64217	3.73495
Nc-1-IP_pe	chr10	77710644	77711898	1255	77711204	26	9.34034	3.78992
Nc-1-IP_pe	chr10	77712697	77713312	616	77712788	15	5.4619	3.27292
Nc-1-IP_pe	chr10	77716031	77716531	501	77716184	17	6.51104	3.5563
Nc-1-IP_pe	chr10	77730704	77730983	280	77730835	18	4.32723	2.54542
Nc-1-IP_pe	chr10	77731608	77731980	373	77731868	15	4.7819	2.95039
Nc-1-IP_pe	chr10	77732303	77732737	435	77732460	12	4.35991	3.0165
Nc-1-IP_pe	chr10	77760621	77761245	625	77761085	12	3.96559	2.80816
Nc-1-IP_pe	chr10	77763850	77764200	351	77764154	14	4.42328	2.86573
Nc-1-IP_pe	chr10	77770157	77770409	253	77770300	16	4.42092	2.71079
Nc-1-IP_pe	chr10	77771508	77771903	396	77771763	16	4.96462	2.94973
Nc-1-IP_pe	chr10	77773902	77774492	591	77774088	16	6.33285	3.58457
Nc-1-IP_pe	chr10	77779859	77780367	509	77780315	16	4.96462	2.94973
Nc-1-IP_pe	chr10	77780709	77780981	273	77780887	15	5.58327	3.33181
Nc-1-IP_pe	chr10	77782294	77782569	276	77782467	15	5.83691	3.4562
Nc-1-IP_pe	chr10	77795022	77795713	692	77795178	16	5.46118	3.17446
Nc-1-IP_pe	chr10	77796536	77797016	481	77796649	16	4.25218	2.63809
Nc-1-IP_pe	chr10	77797521	77797802	282	77797667	18	6.50397	3.44778
Nc-1-IP_pe	chr10	77805287	77806095	809	77805455	18	6.78184	3.57098
Nc-1-IP_pe	chr10	77807970	77808296	327	77808099	17	6.38155	3.49661
Nc-1-IP_pe	chr10	77811411	77812348	938	77811515	17	6.18134	3.40515
Nc-1-IP_pe	chr10	77816187	77816731	545	77816479	21	5.5349	2.81884
Nc-1-IP_pe	chr10	77817729	77818152	424	77817961	16	3.65575	2.38624
Nc-1-IP_pe	chr10	77819192	77819487	296	77819387	13	4.02562	2.7544
Nc-1-IP_pe	chr10	77821717	77822543	827	77821799	18	6.63038	3.50359
Nc-1-IP_pe	chr10	77830359	77830724	366	77830610	18	5.72638	3.11298
Nc-1-IP_pe	chr10	77833514	77834082	569	77833865	21	5.5349	2.81884

Nc-1-IP_pe	chr10	77834345	77834660	316	77834424	18	6.17129	3.30277
Nc-1-IP_pe	chr10	77835158	77835515	358	77835381	22	5.39765	2.71064
Nc-1-IP_pe	chr10	77837820	77839033	1214	77838219	20	5.34663	2.81336
Nc-1-IP_pe	chr10	77840211	77840506	296	77840303	15	4.65115	2.8898
Nc-1-IP_pe	chr10	77841691	77842012	322	77841931	18	3.77591	2.33276
Nc-1-IP_pe	chr10	77846122	77846594	473	77846342	23	9.98044	4.36194
Nc-1-IP_pe	chr10	77859938	77860559	622	77860261	15	4.14926	2.66135
Nc-1-IP_pe	chr10	77869115	77869432	318	77869221	18	5.1438	2.87144
Nc-1-IP_pe	chr10	77873268	77873553	286	77873368	21	7.4689	3.5571
Nc-1-IP_pe	chr10	77875156	77875685	530	77875532	14	4.01473	2.67166
Nc-1-IP_pe	chr10	77877549	77877872	324	77877737	20	5.70098	2.94771
Nc-1-IP_pe	chr10	77879862	77880376	515	77880319	16	3.94146	2.50592
Nc-1-IP_pe	chr10	77884252	77884982	731	77884370	16	5.30773	3.10433
Nc-1-IP_pe	chr10	77890170	77891092	923	77890443	21	7.99961	3.77325
Nc-1-IP_pe	chr10	77893963	77894280	318	77894054	19	6.70429	3.43532
Nc-1-IP_pe	chr10	77894700	77895244	545	77894918	22	8.79433	3.99081
Nc-1-IP_pe	chr10	77901409	77901896	488	77901749	18	6.27579	3.34803
Nc-1-IP_pe	chr10	77908048	77908615	568	77908554	17	5.14803	2.94914
Nc-1-IP_pe	chr10	77914460	77914814	355	77914712	18	4.3886	2.56944
Nc-1-IP_pe	chr10	77922987	77923784	798	77923313	19	3.06441	2.03128
Nc-1-IP_pe	chr10	77925761	77926438	678	77926332	25	5.96578	2.73518
Nc-1-IP_pe	chr10	77938766	77939564	799	77938917	17	7.36791	3.96218
Nc-1-IP_pe	chr10	77942342	77942889	548	77942774	14	3.26761	2.32785
Nc-1-IP_pe	chr10	77943924	77944194	271	77944000	18	8.1187	4.19027
Nc-1-IP_pe	chr10	77966039	77966682	644	77966579	15	5.86312	3.46915
Nc-1-IP_pe	chr10	77967816	77968092	277	77967908	19	7.84735	3.93486
Nc-1-IP_pe	chr10	77975458	77975714	257	77975609	16	4.25252	2.63824
Nc-1-IP_pe	chr10	77977084	77977389	306	77977199	14	4.94039	3.11796
Nc-1-IP_pe	chr10	77979331	77979647	317	77979599	13	3.07238	2.29378
Nc-1-IP_pe	chr10	77981348	77981685	338	77981526	19	7.29128	3.68799
Nc-1-IP_pe	chr10	77987002	77987453	452	77987386	18	6.91333	3.62993
Nc-1-IP_pe	chr10	77987880	77988143	264	77988015	19	8.85804	4.40242
Nc-1-IP_pe	chr10	77990659	77991144	486	77990808	14	5.56442	3.43239
Nc-1-IP_pe	chr10	78004290	78004745	456	78004566	20	9.48695	4.55326
Nc-1-IP_pe	chr10	78028923	78029585	663	78029047	22	4.80668	2.5094
Nc-1-IP_pe	chr10	78036416	78036748	333	78036559	15	4.06016	2.62146
Nc-1-IP_pe	chr10	78054001	78054550	550	78054174	25	10.4937	4.3247
Nc-1-IP_pe	chr10	78061333	78061955	623	78061814	14	5.32037	3.30811
Nc-1-IP_pe	chr10	78067232	78068348	1117	78068331	17	3.11196	2.12137
Nc-1-IP_pe	chr10	78069277	78069794	518	78069470	17	5.54967	3.12324
Nc-1-IP_pe	chr10	78075435	78077114	1680	78075937	19	8.35089	4.16476
Nc-1-IP_pe	chr10	78082413	78082885	473	78082625	13	4.60566	3.04692
Nc-1-IP_pe	chr10	78084621	78084924	304	78084814	20	5.70098	2.94771
Nc-1-IP_pe	chr10	78085677	78086257	581	78086001	16	6.75928	3.79256
Nc-1-IP_pe	chr10	78094915	78095196	282	78095040	16	7.99767	4.42362

Nc-1-IP_pe	chr10	78096172	78096526	355	78096316	17	5.9911	3.31919
Nc-1-IP_pe	chr10	78105467	78105923	457	78105582	13	4.02562	2.7544
Nc-1-IP_pe	chr10	78116733	78117335	603	78116933	15	6.69889	3.8921
Nc-1-IP_pe	chr10	78134363	78134639	277	78134490	13	4.90859	3.20357
Nc-1-IP_pe	chr10	78139117	78139751	635	78139288	19	5.90435	3.10381
Nc-1-IP_pe	chr10	78173537	78174071	535	78173948	14	5.18557	3.24018
Nc-1-IP_pe	chr10	78178261	78179505	1245	78179438	20	5.70098	2.94771
Nc-1-IP_pe	chr10	78179945	78180581	637	78180559	14	4.07659	2.70076
Nc-1-IP_pe	chr10	78185872	78186152	281	78185975	16	6.09641	3.47134
Nc-1-IP_pe	chr10	78196166	78196748	583	78196349	17	5.4568	3.08264
Nc-1-IP_pe	chr10	78197954	78198517	564	78198383	21	9.29881	4.32834
Nc-1-IP_pe	chr10	78199795	78200107	313	78199933	13	4.75398	3.12328
Nc-1-IP_pe	chr10	78202829	78203250	422	78203077	14	4.32616	2.81919
Nc-1-IP_pe	chr10	78210173	78211446	1274	78211205	22	10.4599	4.70482
Nc-1-IP_pe	chr10	78212254	78212762	509	78212663	15	4.40338	2.77622
Nc-1-IP_pe	chr10	78215572	78216246	675	78215936	17	5.9911	3.31919
Nc-1-IP_pe	chr10	78222246	78222548	303	78222532	13	3.67377	2.58159
Nc-1-IP_pe	chr10	78228405	78228716	312	78228574	18	4.6358	2.66698
Nc-1-IP_pe	chr10	78239909	78240264	356	78240154	19	7.29128	3.68799
Nc-1-IP_pe	chr10	78243065	78243656	592	78243339	16	6.51374	3.67221
Nc-1-IP_pe	chr10	78249361	78249647	287	78249481	14	5.24628	3.27071
Nc-1-IP_pe	chr10	78252118	78252456	339	78252272	16	3.39261	2.27748
Nc-1-IP_pe	chr10	78261352	78261706	355	78261557	19	6.51517	3.35563
Nc-1-IP_pe	chr10	78269665	78270024	360	78269843	15	5.33233	3.21049
Nc-1-IP_pe	chr10	78274285	78274562	278	78274445	17	5.35342	3.03768
Nc-1-IP_pe	chr10	78281205	78281535	331	78281220	17	3.84884	2.41145
Nc-1-IP_pe	chr10	78283288	78283779	492	78283571	18	7.18674	3.75388
Nc-1-IP_pe	chr10	78289738	78290643	906	78290443	19	5.15822	2.80735
Nc-1-IP_pe	chr10	78292876	78293310	435	78293080	16	3.94146	2.50592
Nc-1-IP_pe	chr10	78301457	78301733	277	78301729	12	3.46657	2.55102
Nc-1-IP_pe	chr10	78304790	78305072	283	78304932	15	5.29768	3.19388
Nc-1-IP_pe	chr10	78327562	78328094	533	78328044	17	4.78101	2.79343
Nc-1-IP_pe	chr10	78328696	78329116	421	78328885	16	5.37454	3.13479
Nc-1-IP_pe	chr10	78332598	78333329	732	78332784	16	6.61333	3.72083
Nc-1-IP_pe	chr10	78335617	78336373	757	78335926	13	4.66199	3.07584
Nc-1-IP_pe	chr10	78337480	78337957	478	78337857	21	8.12272	3.82426
Nc-1-IP_pe	chr10	78341987	78342480	494	78342155	14	5.03085	3.16286
Nc-1-IP_pe	chr10	78359016	78359477	462	78359476	13	3.07238	2.29378
Nc-1-IP_pe	chr10	78367038	78367547	510	78367202	13	3.67377	2.58159
Nc-1-IP_pe	chr10	78367915	78368219	305	78368075	16	5.28625	3.09457
Nc-1-IP_pe	chr10	78369397	78369694	298	78369563	19	5.90435	3.10381
Nc-1-IP_pe	chr10	78370107	78370473	367	78370275	17	4.44438	2.65333
Nc-1-IP_pe	chr10	78372534	78373067	534	78372804	17	4.78101	2.79343
Nc-1-IP_pe	chr10	78374311	78374602	292	78374404	16	5.98198	3.41707
Nc-1-IP_pe	chr10	78375053	78375664	612	78375513	20	7.97282	3.87239

Nc-1-IP_pe	chr10	78381448	78381796	349	78381626	15	5.20142	3.14789
Nc-1-IP_pe	chr10	78399813	78400197	385	78399960	22	11.675	5.26301
Nc-1-IP_pe	chr10	78404138	78404506	369	78404317	14	3.55308	2.45762
Nc-1-IP_pe	chr10	78406391	78407016	626	78406845	18	6.15736	3.29676
Nc-1-IP_pe	chr10	78407352	78407855	504	78407490	18	4.32723	2.54542
Nc-1-IP_pe	chr10	78408548	78409232	685	78409027	20	6.07662	3.09292
Nc-1-IP_pe	chr10	78412590	78412882	293	78412792	14	5.00888	3.15193
Nc-1-IP_pe	chr10	78413524	78414019	496	78413764	12	3.66916	2.65455
Nc-1-IP_pe	chr10	78419379	78420125	747	78419582	17	6.6711	3.63069
Nc-1-IP_pe	chr10	78422931	78423209	279	78422983	13	4.45253	2.96876
Nc-1-IP_pe	chr10	78434049	78434358	310	78434186	18	5.33197	2.94862
Nc-1-IP_pe	chr10	78436055	78436416	362	78436326	21	6.66991	3.24296
Nc-1-IP_pe	chr10	78438630	78438975	346	78438726	15	5.22925	3.16116
Nc-1-IP_pe	chr10	78443325	78443839	515	78443700	16	4.59224	2.7853
Nc-1-IP_pe	chr10	78446327	78446696	370	78446502	22	10.4227	4.68824
Nc-1-IP_pe	chr10	78456666	78456930	265	78456750	16	5.37454	3.13479
Nc-1-IP_pe	chr10	78457654	78458028	375	78457788	17	6.38155	3.49661
Nc-1-IP_pe	chr10	78459497	78459820	324	78459748	15	4.7819	2.95039
Nc-1-IP_pe	chr10	78462672	78463073	402	78462816	16	5.82808	3.34463
Nc-1-IP_pe	chr10	78464668	78464956	289	78464912	13	3.90032	2.69247
Nc-1-IP_pe	chr10	78468413	78469542	1130	78468639	20	6.72028	3.34863
Nc-1-IP_pe	chr10	78472191	78472623	433	78472431	22	8.79433	3.99081
Nc-1-IP_pe	chr10	78476927	78478057	1131	78476985	15	4.06016	2.62146
Nc-1-IP_pe	chr10	78491478	78492068	591	78491884	16	6.65665	3.74206
Nc-1-IP_pe	chr10	78502783	78503041	259	78502908	15	7.04321	4.07185
Nc-1-IP_pe	chr10	78508676	78509564	889	78509305	16	6.57039	3.69983
Nc-1-IP_pe	chr10	78511674	78512349	676	78512226	18	6.52861	3.45863
Nc-1-IP_pe	chr10	78512696	78512956	261	78512873	14	4.21449	2.76599
Nc-1-IP_pe	chr10	78514012	78514667	656	78514224	19	6.78661	3.47027
Nc-1-IP_pe	chr10	78523274	78523628	355	78523500	21	8.6741	4.05679
Nc-1-IP_pe	chr10	78533792	78534584	793	78533990	15	5.4619	3.27292
Nc-1-IP_pe	chr10	78536963	78537278	316	78537225	15	5.1177	3.10809
Nc-1-IP_pe	chr10	78539205	78539641	437	78539344	17	5.54967	3.12324
Nc-1-IP_pe	chr10	78542566	78543337	772	78542780	17	5.14803	2.94914
Nc-1-IP_pe	chr10	78552269	78552924	656	78552756	17	7.21469	3.88822
Nc-1-IP_pe	chr10	78560313	78560694	382	78560329	13	3.36696	2.43362
Nc-1-IP_pe	chr10	78561048	78561537	490	78561401	12	3.41594	2.52533
Nc-1-IP_pe	chr10	78573255	78573613	359	78573284	13	3.52389	2.509
Nc-1-IP_pe	chr10	78577513	78577826	314	78577607	13	3.67377	2.58159
Nc-1-IP_pe	chr10	78582418	78582683	266	78582632	15	4.40338	2.77622
Nc-1-IP_pe	chr10	78597725	78597994	270	78597761	13	4.02562	2.7544
Nc-1-IP_pe	chr10	78599996	78600336	341	78600098	18	7.1522	3.73811
Nc-1-IP_pe	chr10	78601460	78602008	549	78601935	18	4.96968	2.80074
Nc-1-IP_pe	chr10	78602292	78602564	273	78602451	17	6.38155	3.49661
Nc-1-IP_pe	chr10	78612543	78613050	508	78612860	16	6.8987	3.86161

Nc-1-IP_pe	chr10	78614252	78614539	288	78614397	15	5.66913	3.37372
Nc-1-IP_pe	chr10	78615995	78616420	426	78616327	15	5.57097	3.32583
Nc-1-IP_pe	chr10	78618513	78618836	324	78618668	14	4.95172	3.12357
Nc-1-IP_pe	chr10	78619436	78619912	477	78619763	15	3.46204	2.35851
Nc-1-IP_pe	chr10	78624485	78624768	284	78624715	17	4.78101	2.79343
Nc-1-IP_pe	chr10	78656366	78657160	795	78657025	15	2.95965	2.14351
Nc-1-IP_pe	chr10	78658439	78658884	446	78658760	13	3.67377	2.58159
Nc-1-IP_pe	chr10	78681899	78682505	607	78682458	15	5.34394	3.21607
Nc-1-IP_pe	chr10	78688237	78688527	291	78688372	19	6.32622	3.27682
Nc-1-IP_pe	chr10	78689117	78689627	511	78689371	16	4.59224	2.7853
Nc-1-IP_pe	chr10	78690440	78690897	458	78690653	15	5.54649	3.31392
Nc-1-IP_pe	chr10	78695872	78696195	324	78695999	16	6.33285	3.58457
Nc-1-IP_pe	chr10	78700105	78701144	1040	78700991	15	6.87612	3.98423
Nc-1-IP_pe	chr10	78702210	78702657	448	78702331	16	5.37454	3.13479
Nc-1-IP_pe	chr10	78713051	78713387	337	78713290	16	6.59897	3.7138
Nc-1-IP_pe	chr10	78720083	78720357	275	78720164	14	3.55308	2.45762
Nc-1-IP_pe	chr10	78727120	78727488	369	78727353	15	4.7819	2.95039
Nc-1-IP_pe	chr10	78733380	78734282	903	78733851	18	8.05251	4.15857
Nc-1-IP_pe	chr10	78734868	78735243	376	78735102	12	3.47963	2.55766
Nc-1-IP_pe	chr10	78739893	78740365	473	78740154	14	3.86727	2.6027
Nc-1-IP_pe	chr10	78752589	78753030	442	78752634	13	4.02562	2.7544
Nc-1-IP_pe	chr10	78760722	78761761	1040	78760886	19	6.0077	3.14582
Nc-1-IP_pe	chr10	78765378	78766038	661	78765875	18	6.55337	3.46955
Nc-1-IP_pe	chr10	78769604	78769979	376	78769821	19	6.32622	3.27682
Nc-1-IP_pe	chr10	78779326	78780140	815	78779399	20	5.34663	2.81336
Nc-1-IP_pe	chr10	78799843	78800270	428	78799919	16	5.39474	3.14402
Nc-1-IP_pe	chr10	78801956	78802776	821	78802187	16	3.39261	2.27748
Nc-1-IP_pe	chr10	78803297	78804055	759	78803893	19	3.26827	2.10398
Nc-1-IP_pe	chr10	78806587	78808066	1480	78807491	15	4.7819	2.95039
Nc-1-IP_pe	chr10	78808942	78809353	412	78809217	15	5.98302	3.52865
Nc-1-IP_pe	chr10	78813426	78814727	1302	78814667	16	7.39088	4.10945
Nc-1-IP_pe	chr10	78818017	78818487	471	78818341	18	8.99859	4.62187
Nc-1-IP_pe	chr10	78819364	78819772	409	78819557	14	6.06519	3.69269
Nc-1-IP_pe	chr10	78846592	78846890	299	78846756	13	3.60553	2.54847
Nc-1-IP_pe	chr10	78851395	78851949	555	78851508	15	5.29768	3.19388
Nc-1-IP_pe	chr10	78852600	78853115	516	78852960	21	6.2632	3.08808
Nc-1-IP_pe	chr10	78857014	78857393	380	78857318	17	6.83651	3.70825
Nc-1-IP_pe	chr10	78859470	78859821	352	78859508	14	3.55308	2.45762
Nc-1-IP_pe	chr10	78865010	78865494	485	78865226	22	10.0441	4.52101
Nc-1-IP_pe	chr10	78865919	78867139	1221	78866947	21	8.12272	3.82426
Nc-1-IP_pe	chr10	78870067	78870331	265	78870186	19	5.90435	3.10381
Nc-1-IP_pe	chr10	78875086	78875494	409	78875156	10	3.57781	2.77499
Nc-1-IP_pe	chr10	78892213	78892593	381	78892271	13	4.41954	2.952
Nc-1-IP_pe	chr10	78895586	78895885	300	78895698	20	6.08336	3.09555
Nc-1-IP_pe	chr10	78898447	78899316	870	78899206	22	10.009	4.5057

Nc-1-IP_pe	chr10	78900105	78901154	1050	78900589	18	6.28754	3.35314
Nc-1-IP_pe	chr10	78902695	78903191	497	78903073	15	4.86199	2.98773
Nc-1-IP_pe	chr10	78904886	78905472	587	78904967	15	3.74763	2.48304
Nc-1-IP_pe	chr10	78905834	78906143	310	78905995	19	5.41648	2.90862
Nc-1-IP_pe	chr10	78910929	78911280	352	78911160	28	14.0728	5.34759
Nc-1-IP_pe	chr10	78911966	78912412	447	78912109	16	5.82808	3.34463
Nc-1-IP_pe	chr10	78912761	78914997	2237	78913568	16	4.59224	2.7853
Nc-1-IP_pe	chr10	78916335	78916607	273	78916501	15	3.46204	2.35851
Nc-1-IP_pe	chr10	78920049	78920322	274	78920148	22	4.2847	2.33597
Nc-1-IP_pe	chr10	78922388	78922890	503	78922705	23	10.4737	4.57008
Nc-1-IP_pe	chr10	78923437	78923828	392	78923652	18	6.55337	3.46955
Nc-1-IP_pe	chr10	78927468	78927871	404	78927687	20	4.71088	2.57831
Nc-1-IP_pe	chr10	78928410	78929157	748	78929033	19	6.68027	3.42515
Nc-1-IP_pe	chr10	78929961	78930624	664	78930514	15	4.63166	2.88081
Nc-1-IP_pe	chr10	78933115	78933625	511	78933439	20	6.83566	3.39542
Nc-1-IP_pe	chr10	78935309	78935604	296	78935502	18	5.42854	2.98853
Nc-1-IP_pe	chr10	78941447	78941921	475	78941780	22	9.42371	4.25368
Nc-1-IP_pe	chr10	78948414	78948825	412	78948463	15	3.74733	2.48291
Nc-1-IP_pe	chr10	78950884	78951499	616	78951256	17	5.4568	3.08264
Nc-1-IP_pe	chr10	78952733	78952986	254	78952974	13	3.07238	2.29378
Nc-1-IP_pe	chr10	78958502	78958761	260	78958696	16	5.38376	3.139
Nc-1-IP_pe	chr10	78959302	78959804	503	78959658	13	3.35769	2.42919
Nc-1-IP_pe	chr10	78960571	78961153	583	78961012	21	8.20621	3.85904
Nc-1-IP_pe	chr10	78962620	78962978	359	78962748	15	4.48862	2.81511
Nc-1-IP_pe	chr10	78966254	78967131	878	78966391	23	9.36735	4.11018
Nc-1-IP_pe	chr10	78967839	78968248	410	78968108	20	6.94658	3.44067
Nc-1-IP_pe	chr10	78972329	78972807	479	78972775	24	5.18704	2.5391
Nc-1-IP_pe	chr10	78974593	78975436	844	78974719	21	7.45653	3.55214
Nc-1-IP_pe	chr10	78977607	78977934	328	78977891	17	4.27863	2.58527
Nc-1-IP_pe	chr10	78981353	78981879	527	78981510	16	4.59224	2.7853
Nc-1-IP_pe	chr10	78985613	78986049	437	78985743	17	5.35342	3.03768
Nc-1-IP_pe	chr10	78987126	78987976	851	78987777	15	4.23553	2.70016
Nc-1-IP_pe	chr10	78988764	78989040	277	78988779	11	3.06138	2.41205
Nc-1-IP_pe	chr10	78992286	78993040	755	78992492	19	8.44891	4.21022
Nc-1-IP_pe	chr10	78996636	78996895	260	78996837	19	6.78661	3.47027
Nc-1-IP_pe	chr10	79004843	79005385	543	79005376	14	3.13218	2.26693
Nc-1-IP_pe	chr10	79006391	79006849	459	79006480	16	4.42092	2.71079
Nc-1-IP_pe	chr10	79007587	79007855	269	79007674	16	5.82808	3.34463
Nc-1-IP_pe	chr10	79009034	79009312	279	79009078	11	3.12586	2.44604
Nc-1-IP_pe	chr10	79010840	79011121	282	79011024	16	4.96462	2.94973
Nc-1-IP_pe	chr10	79020047	79020321	275	79020257	15	4.5357	2.83668
Nc-1-IP_pe	chr10	79022704	79023039	336	79022979	21	7.10961	3.41419
Nc-1-IP_pe	chr10	79024830	79025115	286	79025011	18	4.96968	2.80074
Nc-1-IP_pe	chr10	79047976	79048734	759	79048486	20	6.08336	3.09555
Nc-1-IP_pe	chr10	79048998	79049469	472	79049188	18	4.96968	2.80074

Nc-1-IP_pe	chr10	79049803	79050811	1009	79050026	16	5.64353	3.25859
Nc-1-IP_pe	chr10	79054002	79054377	376	79054298	13	3.44439	2.47074
Nc-1-IP_pe	chr10	79056229	79056798	570	79056601	20	8.27807	4.00541
Nc-1-IP_pe	chr10	79058374	79058822	449	79058675	16	5.60875	3.24248
Nc-1-IP_pe	chr10	79062055	79062482	428	79062116	18	5.33197	2.94862
Nc-1-IP_pe	chr10	79068045	79068493	449	79068117	19	5.32483	2.87252
Nc-1-IP_pe	chr10	79072815	79073391	577	79072895	17	4.41444	2.64099
Nc-1-IP_pe	chr10	79078866	79079752	887	79078928	17	4.52162	2.68525
Nc-1-IP_pe	chr10	79082399	79082682	284	79082559	18	5.50765	3.02139
Nc-1-IP_pe	chr10	79084516	79085048	533	79084957	21	5.59941	2.84228
Nc-1-IP_pe	chr10	79085651	79085963	313	79085863	22	8.09481	3.70843
Nc-1-IP_pe	chr10	79088163	79089357	1195	79089238	19	6.80149	3.4766
Nc-1-IP_pe	chr10	79090728	79091013	286	79090946	17	5.14803	2.94914
Nc-1-IP_pe	chr10	79092159	79092616	458	79092472	15	4.40535	2.77711
Nc-1-IP_pe	chr10	79094227	79095082	856	79094417	21	7.10961	3.41419
Nc-1-IP_pe	chr10	79095624	79096260	637	79096095	18	4.04135	2.43445
Nc-1-IP_pe	chr10	79099989	79101110	1122	79100177	23	9.21857	4.05024
Nc-1-IP_pe	chr10	79103065	79103576	512	79103163	17	5.15375	2.9516
Nc-1-IP_pe	chr10	79104109	79104701	593	79104220	23	9.38241	4.11627
Nc-1-IP_pe	chr10	79107333	79107883	551	79107650	24	10.2246	4.33784
Nc-1-IP_pe	chr10	79108783	79109448	666	79109195	18	6.24071	3.33281
Nc-1-IP_pe	chr10	79110510	79110812	303	79110710	16	5.08752	3.00476
Nc-1-IP_pe	chr10	79113002	79113555	554	79113090	18	4.6358	2.66698
Nc-1-IP_pe	chr10	79115318	79115716	399	79115435	17	5.22259	2.98117
Nc-1-IP_pe	chr10	79117684	79118050	367	79117859	23	7.01947	3.21526
Nc-1-IP_pe	chr10	79120840	79121239	400	79120956	15	4.34186	2.74826
Nc-1-IP_pe	chr10	79123630	79123960	331	79123662	15	3.67238	2.45006
Nc-1-IP_pe	chr10	79124531	79125398	868	79124894	19	6.78661	3.47027
Nc-1-IP_pe	chr10	79126722	79127225	504	79127166	14	4.11245	2.71767
Nc-1-IP_pe	chr10	79127541	79128363	823	79128217	20	7.81569	3.80474
Nc-1-IP_pe	chr10	79135140	79135412	273	79135395	16	3.65575	2.38624
Nc-1-IP_pe	chr10	79142794	79144102	1309	79143163	19	5.51632	2.94814
Nc-1-IP_pe	chr10	79149114	79149618	505	79149422	26	7.03684	3.01675
Nc-1-IP_pe	chr10	79153367	79154769	1403	79153805	20	4.94901	2.66548
Nc-1-IP_pe	chr10	79155459	79155937	479	79155541	21	5.2077	2.70109
Nc-1-IP_pe	chr10	79157947	79158853	907	79158301	22	6.4249	3.07445
Nc-1-IP_pe	chr10	79160515	79160991	477	79160846	19	3.06441	2.03128
Nc-1-IP_pe	chr10	79166241	79167131	891	79166406	26	6.15464	2.74223
Nc-1-IP_pe	chr10	79168208	79168557	350	79168265	16	4.25252	2.63824
Nc-1-IP_pe	chr10	79168902	79169279	378	79168999	20	5.70098	2.94771
Nc-1-IP_pe	chr10	79169973	79170710	738	79170488	17	5.38418	3.05103
Nc-1-IP_pe	chr10	79173997	79174426	430	79174186	21	7.85249	3.71272
Nc-1-IP_pe	chr10	79174711	79175571	861	79175228	25	6.28656	2.83671
Nc-1-IP_pe	chr10	79176938	79177239	302	79177040	19	6.14597	3.20241
Nc-1-IP_pe	chr10	79179509	79179860	352	79179695	21	5.68737	2.87437

Nc-1-IP_pe	chr10	79181082	79181454	373	79181156	21	5.77693	2.9072
Nc-1-IP_pe	chr10	79182830	79183168	339	79183035	16	2.92499	2.08721
Nc-1-IP_pe	chr10	79187006	79187619	614	79187552	18	5.322	2.94451
Nc-1-IP_pe	chr10	79188984	79189345	362	79189276	21	6.79291	3.29045
Nc-1-IP_pe	chr10	79189873	79190375	503	79190261	22	7.58251	3.50803
Nc-1-IP_pe	chr10	79193505	79194446	942	79193834	20	5.01743	2.69071
Nc-1-IP_pe	chr10	79195921	79197185	1265	79196366	20	5.9966	3.06174
Nc-1-IP_pe	chr10	79199013	79199303	291	79199255	14	3.32318	2.35297
Nc-1-IP_pe	chr10	79202587	79203165	579	79202821	23	5.91101	2.82849
Nc-1-IP_pe	chr10	79203517	79204100	584	79204009	16	4.63386	2.80351
Nc-1-IP_pe	chr10	79207256	79207752	497	79207565	18	5.62894	3.07204
Nc-1-IP_pe	chr10	79208325	79208580	256	79208453	20	7.61095	3.71744
Nc-1-IP_pe	chr10	79209396	79210986	1591	79210318	19	6.78924	3.47139
Nc-1-IP_pe	chr10	79211691	79212025	335	79211904	20	7.53105	3.68363
Nc-1-IP_pe	chr10	79214836	79215252	417	79214887	18	6.17129	3.30277
Nc-1-IP_pe	chr10	79215748	79216271	524	79216147	20	6.49719	3.259
Nc-1-IP_pe	chr10	79220301	79220751	451	79220508	22	5.09279	2.60614
Nc-1-IP_pe	chr10	79221603	79222054	452	79221803	16	5.34015	3.1191
Nc-1-IP_pe	chr10	79223090	79223543	454	79223194	14	4.04994	2.68821
Nc-1-IP_pe	chr10	79224352	79224807	456	79224429	18	6.41868	3.41035
Nc-1-IP_pe	chr10	79226146	79226448	303	79226266	15	4.40338	2.77622
Nc-1-IP_pe	chr10	79229799	79230156	358	79230104	15	4.73012	2.92634
Nc-1-IP_pe	chr10	79232945	79233295	351	79233048	14	3.59582	2.47721
Nc-1-IP_pe	chr10	79233716	79234068	353	79233914	20	6.83566	3.39542
Nc-1-IP_pe	chr10	79236995	79237319	325	79237104	20	5.70098	2.94771
Nc-1-IP_pe	chr10	79238889	79239810	922	79238989	19	6.06042	3.16735
Nc-1-IP_pe	chr10	79241758	79242421	664	79242322	23	5.28318	2.6185
Nc-1-IP_pe	chr10	79243720	79244383	664	79244174	20	7.09852	3.50309
Nc-1-IP_pe	chr10	79244696	79245534	839	79245368	20	6.08336	3.09555
Nc-1-IP_pe	chr10	79247012	79247513	502	79247384	17	5.23251	2.98544
Nc-1-IP_pe	chr10	79248276	79248767	492	79248356	18	6.00198	3.22999
Nc-1-IP_pe	chr10	79250484	79251504	1021	79250728	22	8.40205	3.8312
Nc-1-IP_pe	chr10	79254242	79255013	772	79254448	23	9.24806	4.06209
Nc-1-IP_pe	chr10	79264152	79264748	597	79264163	18	4.31276	2.53976
Nc-1-IP_pe	chr10	79265771	79266141	371	79266019	19	5.34301	2.87967
Nc-1-IP_pe	chr10	79270130	79270676	547	79270422	22	5.09279	2.60614
Nc-1-IP_pe	chr10	79272945	79273214	270	79273204	16	2.92499	2.08721
Nc-1-IP_pe	chr10	79273740	79274120	381	79273931	19	5.51001	2.94564
Nc-1-IP_pe	chr10	79274794	79275123	330	79274924	21	5.51301	2.8109
Nc-1-IP_pe	chr10	79276035	79276562	528	79276410	21	7.31021	3.49365
Nc-1-IP_pe	chr10	79277763	79278162	400	79278142	14	3.64238	2.49861
Nc-1-IP_pe	chr10	79278961	79279472	512	79279062	24	9.05596	3.87955
Nc-1-IP_pe	chr10	79286991	79287451	461	79287251	19	6.0182	3.1501
Nc-1-IP_pe	chr10	79290977	79291274	298	79291182	20	5.8596	3.00868
Nc-1-IP_pe	chr10	79292958	79293963	1006	79293838	21	5.5349	2.81884

Nc-1-IP_pe	chr10	79295045	79295725	681	79295324	24	8.17403	3.55098
Nc-1-IP_pe	chr10	79297736	79298362	627	79297843	20	5.61491	2.91485
Nc-1-IP_pe	chr10	79300717	79301320	604	79301013	28	9.98803	3.83221
Nc-1-IP_pe	chr10	79301673	79302333	661	79301932	18	4.3886	2.56944
Nc-1-IP_pe	chr10	79303539	79304397	859	79303672	21	6.41148	3.14416
Nc-1-IP_pe	chr10	79305306	79305661	356	79305440	21	5.2077	2.70109
Nc-1-IP_pe	chr10	79306803	79307207	405	79307130	17	5.19295	2.96842
Nc-1-IP_pe	chr10	79310556	79311038	483	79310695	17	5.35342	3.03768
Nc-1-IP_pe	chr10	79311352	79313307	1956	79313120	24	9.62514	4.09944
Nc-1-IP_pe	chr10	79313959	79314419	461	79314019	22	4.2847	2.33597
Nc-1-IP_pe	chr10	79315040	79315613	574	79315388	18	5.91407	3.19248
Nc-1-IP_pe	chr10	79317094	79317447	354	79317302	20	5.70098	2.94771
Nc-1-IP_pe	chr10	79318239	79318889	651	79318345	19	6.25501	3.24734
Nc-1-IP_pe	chr10	79327471	79327850	380	79327623	18	5.23669	2.90944
Nc-1-IP_pe	chr10	79328954	79329302	349	79329204	25	6.32814	2.84998
Nc-1-IP_pe	chr10	79335379	79335881	503	79335821	19	5.15822	2.80735
Nc-1-IP_pe	chr10	79337748	79338141	394	79337893	19	4.5193	2.56258
Nc-1-IP_pe	chr10	79339251	79339620	370	79339393	18	4.96968	2.80074
Nc-1-IP_pe	chr10	79342755	79343237	483	79342951	21	5.2077	2.70109
Nc-1-IP_pe	chr10	79345320	79346065	746	79345711	16	4.05159	2.55252
Nc-1-IP_pe	chr10	79346593	79346992	400	79346899	18	4.6358	2.66698
Nc-1-IP_pe	chr10	79347437	79347954	518	79347887	25	3.94771	2.12633
Nc-1-IP_pe	chr10	79348275	79348550	276	79348293	19	3.5722	2.21326
Nc-1-IP_pe	chr10	79350421	79350713	293	79350544	21	4.90204	2.59278
Nc-1-IP_pe	chr10	79351377	79351678	302	79351530	24	9.61033	4.09364
Nc-1-IP_pe	chr10	79352138	79352467	330	79352358	26	6.81227	2.94581
Nc-1-IP_pe	chr10	79353661	79354101	441	79353840	19	6.32622	3.27682
Nc-1-IP_pe	chr10	79360202	79360494	293	79360297	18	5.72638	3.11298
Nc-1-IP_pe	chr10	79377697	79378173	477	79377713	18	3.77591	2.33276
Nc-1-IP_pe	chr10	79378649	79379161	513	79379117	15	3.74763	2.48304
Nc-1-IP_pe	chr10	79380519	79381087	569	79380821	21	8.37669	3.93054
Nc-1-IP_pe	chr10	79381370	79381646	277	79381385	12	3.05529	2.34431
Nc-1-IP_pe	chr10	79384010	79384386	377	79384050	19	7.24901	3.66953
Nc-1-IP_pe	chr10	79384934	79385672	739	79385478	15	5.17309	3.1344
Nc-1-IP_pe	chr10	79386044	79386670	627	79386249	18	4.32723	2.54542
Nc-1-IP_pe	chr10	79387550	79388185	636	79387956	17	6.30549	3.46174
Nc-1-IP_pe	chr10	79388905	79389235	331	79388983	19	4.5193	2.56258
Nc-1-IP_pe	chr10	79390519	79391644	1126	79390747	23	8.39	3.72457
Nc-1-IP_pe	chr10	79397295	79398231	937	79398187	17	5.56266	3.12894
Nc-1-IP_pe	chr10	79399265	79400012	748	79399891	17	3.58449	2.30632
Nc-1-IP_pe	chr10	79400668	79401453	786	79401072	17	4.78065	2.79327
Nc-1-IP_pe	chr10	79403379	79403655	277	79403448	16	3.39261	2.27748
Nc-1-IP_pe	chr10	79404950	79406177	1228	79405608	19	6.1893	3.22023
Nc-1-IP_pe	chr10	79408128	79408677	550	79408305	17	5.29254	3.01133
Nc-1-IP_pe	chr10	79410681	79411152	472	79410684	16	3.1497	2.1782

Nc-1-IP_pe	chr10	79411961	79412374	414	79412074	20	8.14433	3.94687
Nc-1-IP_pe	chr10	79413170	79414179	1010	79413671	19	6.11371	3.18917
Nc-1-IP_pe	chr10	79418167	79419637	1471	79418734	19	8.44891	4.21022
Nc-1-IP_pe	chr10	79421656	79422208	553	79421803	13	4.26502	2.87396
Nc-1-IP_pe	chr10	79422474	79422818	345	79422650	12	3.98572	2.81868
Nc-1-IP_pe	chr10	79424342	79425388	1047	79424714	18	6.15736	3.29676
Nc-1-IP_pe	chr10	79431306	79431568	263	79431409	16	3.94146	2.50592
Nc-1-IP_pe	chr10	79431984	79432641	658	79432432	22	6.44373	3.08129
Nc-1-IP_pe	chr10	79433233	79433552	320	79433337	20	6.85904	3.40493
Nc-1-IP_pe	chr10	79433966	79434237	272	79434013	18	3.77591	2.33276
Nc-1-IP_pe	chr10	79435652	79435964	313	79435758	21	5.42814	2.78021
Nc-1-IP_pe	chr10	79436394	79436749	356	79436488	21	6.15208	3.04634
Nc-1-IP_pe	chr10	79437620	79437931	312	79437714	22	6.07094	2.94697
Nc-1-IP_pe	chr10	79440391	79440792	402	79440616	19	5.77229	3.05046
Nc-1-IP_pe	chr10	79464136	79464435	300	79464397	16	4.25252	2.63824
Nc-1-IP_pe	chr10	79465372	79465688	317	79465539	18	7.73287	4.007
Nc-1-IP_pe	chr10	79484117	79484700	584	79484209	16	3.39261	2.27748
Nc-1-IP_pe	chr10	79486634	79487129	496	79486714	16	5.72576	3.29682
Nc-1-IP_pe	chr10	79490456	79490944	489	79490578	14	4.13959	2.7305
Nc-1-IP_pe	chr10	79494097	79494911	815	79494234	14	4.32616	2.81919
Nc-1-IP_pe	chr10	79499635	79499927	293	79499723	15	3.46204	2.35851
Nc-1-IP_pe	chr10	79501745	79502123	379	79502003	26	7.55119	3.18205
Nc-1-IP_pe	chr10	79503447	79503752	306	79503515	20	5.70098	2.94771
Nc-1-IP_pe	chr10	79512969	79513570	602	79513142	19	9.03708	4.48779
Nc-1-IP_pe	chr10	79523817	79524150	334	79524059	17	4.13464	2.52661
Nc-1-IP_pe	chr10	79532607	79532923	317	79532821	13	5.36853	3.44651
Nc-1-IP_pe	chr10	79541730	79541996	267	79541839	11	3.96771	2.90079
Nc-1-IP_pe	chr10	79555335	79555625	291	79555481	21	9.20731	4.28803
Nc-1-IP_pe	chr10	79558991	79559414	424	79559020	17	4.13464	2.52661
Nc-1-IP_pe	chr10	79559812	79560221	410	79560078	19	4.8268	2.67939
Nc-1-IP_pe	chr10	79562501	79563018	518	79562646	23	7.01947	3.21526
Nc-1-IP_pe	chr10	79570494	79570779	286	79570624	18	5.72638	3.11298
Nc-1-IP_pe	chr10	79584629	79584918	290	79584793	13	5.51308	3.52412
Nc-1-IP_pe	chr10	79585721	79586016	296	79585781	13	4.78908	3.14145
Nc-1-IP_pe	chr10	79593445	79593713	269	79593561	13	5.85439	3.70972
Nc-1-IP_pe	chr10	79602865	79603586	722	79602923	13	4.02562	2.7544
Nc-1-IP_pe	chr10	79608584	79608970	387	79608759	14	5.56442	3.43239
Nc-1-IP_pe	chr10	79609848	79611108	1261	79610426	23	11.372	4.962
Nc-1-IP_pe	chr10	79611605	79613198	1594	79611813	23	4.99703	2.52478
Nc-1-IP_pe	chr10	79619649	79620405	757	79620291	16	8.64073	4.7666
Nc-1-IP_pe	chr10	79637593	79637854	262	79637697	14	5.838	3.57373
Nc-1-IP_pe	chr10	79686704	79686973	270	79686804	17	8.7831	4.67333
Nc-1-IP_pe	chr10	79692629	79693265	637	79693193	16	4.68897	2.82769
Nc-1-IP_pe	chr10	79699459	79699722	264	79699557	15	6.6517	3.86771
Nc-1-IP_pe	chr10	79700489	79700973	485	79700758	21	7.10961	3.41419

Nc-1-IP_pe	chr10	79709976	79710701	726	79710478	17	8.58498	4.57078
Nc-1-IP_pe	chr10	79712491	79712953	463	79712915	11	4.06403	2.95415
Nc-1-IP_pe	chr10	79713741	79714295	555	79714174	17	10.1401	5.40027
Nc-1-IP_pe	chr10	79838689	79839052	364	79838752	12	3.83688	2.74114
Nc-1-IP_pe	chr10	79846610	79847332	723	79846982	22	3.82136	2.18497
Nc-1-IP_pe	chr10	79847643	79847986	344	79847814	24	9.91256	4.21286
Nc-1-IP_pe	chr10	79849411	79849682	272	79849568	20	7.0741	3.49302
Nc-1-IP_pe	chr10	79849970	79850281	312	79850137	22	8.52649	3.88148
Nc-1-IP_pe	chr10	79851094	79852129	1036	79851585	27	8.12936	3.29991
Nc-1-IP_pe	chr10	79853114	79853409	296	79853349	13	3.07238	2.29378
Nc-1-IP_pe	chr10	79861201	79861507	307	79861459	19	4.5193	2.56258
Nc-1-IP_pe	chr10	79867077	79867338	262	79867086	13	3.07238	2.29378
Nc-1-IP_pe	chr10	79868280	79868636	357	79868499	15	7.43258	4.27887
Nc-1-IP_pe	chr10	79873291	79873786	496	79873475	12	4.70082	3.20033
Nc-1-IP_pe	chr10	79903766	79904679	914	79904567	21	4.0961	2.31438
Nc-1-IP_pe	chr10	79908545	79908822	278	79908691	15	7.30527	4.21075
Nc-1-IP_pe	chr10	79922030	79922969	940	79922872	21	6.15208	3.04634
Nc-1-IP_pe	chr10	79925898	79926398	501	79926001	20	8.56319	4.1316
Nc-1-IP_pe	chr10	79927481	79928217	737	79928039	16	5.73763	3.30235
Nc-1-IP_pe	chr10	79936699	79937131	433	79936919	16	7.68537	4.26075
Nc-1-IP_pe	chr10	79937634	79938152	519	79938117	14	4.13959	2.7305
Nc-1-IP_pe	chr10	79939960	79940831	872	79940728	16	5.37454	3.13479
Nc-1-IP_pe	chr10	79942470	79942798	329	79942652	15	3.74763	2.48304
Nc-1-IP_pe	chr10	79946270	79946734	465	79946728	14	3.26761	2.32785
Nc-1-IP_pe	chr10	79948870	79949141	272	79949118	12	3.58823	2.61305
Nc-1-IP_pe	chr10	79952071	79952334	264	79952251	16	6.80394	3.81462
Nc-1-IP_pe	chr10	79954370	79955181	812	79955092	13	5.76252	3.65944
Nc-1-IP_pe	chr10	79956204	79956962	759	79956392	16	4.59224	2.7853
Nc-1-IP_pe	chr10	79976238	79976688	451	79976413	16	7.8117	4.32634
Nc-1-IP_pe	chr10	79981598	79982150	553	79982032	21	7.58657	3.60451
Nc-1-IP_pe	chr10	79985999	79986396	398	79986193	21	9.17453	4.27363
Nc-1-IP_pe	chr10	79998200	79998558	359	79998466	10	3.98634	3.01131
Nc-1-IP_pe	chr10	80005758	80006154	397	80005795	13	4.90859	3.20357
Nc-1-IP_pe	chr10	80011544	80011815	272	80011677	18	9.67522	4.96638
Nc-1-IP_pe	chr10	80022196	80023322	1127	80023212	18	5.72638	3.11298
Nc-1-IP_pe	chr10	80030032	80030592	561	80030289	18	6.63038	3.50359
Nc-1-IP_pe	chr10	80038372	80038872	501	80038660	21	7.58657	3.60451
Nc-1-IP_pe	chr10	80052751	80053157	407	80052806	10	3.60969	2.79325
Nc-1-IP_pe	chr10	80056854	80057125	272	80057015	15	5.20142	3.14789
Nc-1-IP_pe	chr10	80059958	80060254	297	80060124	12	4.75569	3.23024
Nc-1-IP_pe	chr10	80069837	80070366	530	80070000	14	6.44441	3.89444
Nc-1-IP_pe	chr10	80078242	80079486	1245	80078854	22	10.782	4.84972
Nc-1-IP_pe	chr10	80100269	80100558	290	80100415	20	6.49675	3.25882
Nc-1-IP_pe	chr10	80108679	80109684	1006	80109566	13	3.67377	2.58159
Nc-1-IP_pe	chr10	80111875	80112392	518	80112313	13	5.01954	3.26161

Nc-1-IP_pe	chr10	80119297	80119617	321	80119452	16	7.97877	4.4137
Nc-1-IP_pe	chr10	80126733	80127117	385	80126872	21	8.6741	4.05679
Nc-1-IP_pe	chr10	80132337	80132883	547	80132531	14	3.86727	2.6027
Nc-1-IP_pe	chr10	80136050	80136396	347	80136166	18	6.63038	3.50359
Nc-1-IP_pe	chr10	80139116	80139440	325	80139241	11	3.42779	2.60674
Nc-1-IP_pe	chr10	80144050	80145075	1026	80144881	26	11.5681	4.62396
Nc-1-IP_pe	chr10	80148037	80148536	500	80148159	17	5.54967	3.12324
Nc-1-IP_pe	chr10	80152707	80153085	379	80152759	21	5.68737	2.87437
Nc-1-IP_pe	chr10	80154345	80154770	426	80154606	20	5.9768	3.05405
Nc-1-IP_pe	chr10	80164992	80165505	514	80165312	18	4.96968	2.80074
Nc-1-IP_pe	chr10	80166918	80167378	461	80167036	17	3.33947	2.20998
Nc-1-IP_pe	chr10	80172522	80172798	277	80172658	21	7.10961	3.41419
Nc-1-IP_pe	chr10	80173082	80173506	425	80173254	20	5.70098	2.94771
Nc-1-IP_pe	chr10	80174520	80175301	782	80174860	16	5.50606	3.19508
Nc-1-IP_pe	chr10	80176999	80178771	1773	80178550	24	12.4866	5.30135
Nc-1-IP_pe	chr10	80183013	80183326	314	80183208	20	6.08336	3.09555
Nc-1-IP_pe	chr10	80186478	80186804	327	80186614	21	5.86814	2.94078
Nc-1-IP_pe	chr10	80191577	80192200	624	80192080	22	7.27519	3.39036
Nc-1-IP_pe	chr10	80194586	80195590	1005	80194801	16	6.33285	3.58457
Nc-1-IP_pe	chr10	80204842	80205747	906	80205220	24	6.44146	2.94634
Nc-1-IP_pe	chr10	80207285	80207592	308	80207436	17	3.84884	2.41145
Nc-1-IP_pe	chr10	80214139	80214442	304	80214383	14	5.03085	3.16286
Nc-1-IP_pe	chr10	80221508	80221782	275	80221660	14	5.03085	3.16286
Nc-1-IP_pe	chr10	80228953	80229342	390	80229049	20	6.08336	3.09555
Nc-1-IP_pe	chr10	80232534	80232990	457	80232892	20	7.97282	3.87239
Nc-1-IP_pe	chr10	80235110	80235443	334	80235211	19	3.48582	2.18208
Nc-1-IP_pe	chr10	80237579	80238187	609	80238080	15	4.80057	2.95908
Nc-1-IP_pe	chr10	80247492	80247837	346	80247657	27	6.66155	2.84379
Nc-1-IP_pe	chr10	80252791	80253149	359	80252795	18	3.52899	2.23922
Nc-1-IP_pe	chr10	80260024	80260780	757	80260293	16	4.96462	2.94973
Nc-1-IP_pe	chr10	80261070	80261755	686	80261574	16	4.59754	2.78762
Nc-1-IP_pe	chr10	80263878	80264949	1072	80264767	19	5.90435	3.10381
Nc-1-IP_pe	chr10	80265788	80266673	886	80266472	22	7.7408	3.56938
Nc-1-IP_pe	chr10	80270380	80270766	387	80270580	15	6.13413	3.60421
Nc-1-IP_pe	chr10	80272210	80272771	562	80272218	15	2.95965	2.14351
Nc-1-IP_pe	chr10	80273309	80273750	442	80273419	20	6.49719	3.259
Nc-1-IP_pe	chr10	80277569	80278185	617	80278014	18	7.40151	3.85254
Nc-1-IP_pe	chr10	80283613	80284113	501	80283702	13	4.41954	2.952
Nc-1-IP_pe	chr10	80294215	80294591	377	80294416	16	8.17084	4.51499
Nc-1-IP_pe	chr10	80297930	80298186	257	80298021	11	3.28475	2.53029
Nc-1-IP_pe	chr10	80315344	80315789	446	80315552	20	6.08336	3.09555
Nc-1-IP_pe	chr10	80335429	80335906	478	80335831	12	5.56373	3.68072
Nc-1-IP_pe	chr10	80346672	80346990	319	80346907	12	5.77527	3.80164
Nc-1-IP_pe	chr10	80348942	80349213	272	80349060	13	3.35769	2.42919
Nc-1-IP_pe	chr10	80359465	80360010	546	80359786	11	4.59676	3.25427

Nc-1-IP_pe	chr10	80387977	80388410	434	80388095	13	4.76564	3.12931
Nc-1-IP_pe	chr10	80395284	80396110	827	80395940	21	7.58657	3.60451
Nc-1-IP_pe	chr10	80408223	80409127	905	80408786	21	6.15208	3.04634
Nc-1-IP_pe	chr10	80410888	80411178	291	80411036	20	5.34663	2.81336
Nc-1-IP_pe	chr10	80418588	80419142	555	80418719	15	3.20025	2.24588
Nc-1-IP_pe	chr10	80420119	80420476	358	80420209	20	8.29312	4.01203
Nc-1-IP_pe	chr10	80425794	80426180	387	80426107	18	6.60326	3.49159
Nc-1-IP_pe	chr10	80437146	80437430	285	80437272	15	4.7819	2.95039
Nc-1-IP_pe	chr10	80439394	80439732	339	80439541	16	5.82808	3.34463
Nc-1-IP_pe	chr10	80443467	80443725	259	80443659	15	5.12872	3.11332
Nc-1-IP_pe	chr10	80457576	80458534	959	80457693	21	6.64136	3.23197
Nc-1-IP_pe	chr10	80464849	80465275	427	80465049	18	5.33197	2.94862
Nc-1-IP_pe	chr10	80473295	80473573	279	80473474	16	5.96945	3.41115
Nc-1-IP_pe	chr10	80474382	80475125	744	80474592	19	5.15822	2.80735
Nc-1-IP_pe	chr10	80475620	80475999	380	80475842	18	7.04825	3.69086
Nc-1-IP_pe	chr10	80481748	80482000	253	80481760	18	3.77591	2.33276
Nc-1-IP_pe	chr10	80487427	80488097	671	80487633	15	4.97715	3.04172
Nc-1-IP_pe	chr10	80488455	80488806	352	80488654	16	5.88264	3.37024
Nc-1-IP_pe	chr10	80491381	80492006	626	80491421	14	4.21449	2.76599
Nc-1-IP_pe	chr10	80500570	80500884	315	80500628	16	3.39261	2.27748
Nc-1-IP_pe	chr10	80504035	80504604	570	80504301	18	5.9909	3.22526
Nc-1-IP_pe	chr10	80505381	80506129	749	80505505	21	6.05567	3.01032
Nc-1-IP_pe	chr10	80506873	80507491	619	80507332	24	8.94755	3.83837
Nc-1-IP_pe	chr10	80514286	80514562	277	80514398	20	6.94658	3.44067
Nc-1-IP_pe	chr10	80516256	80516941	686	80516823	15	5.00915	3.05678
Nc-1-IP_pe	chr10	80518675	80519259	585	80519029	16	6.36135	3.59832
Nc-1-IP_pe	chr10	80520126	80521421	1296	80521020	16	4.96462	2.94973
Nc-1-IP_pe	chr10	80524058	80524499	442	80524267	24	7.61242	3.34923
Nc-1-IP_pe	chr10	80526687	80527514	828	80526966	16	6.57039	3.69983
Nc-1-IP_pe	chr10	80528297	80529237	941	80529172	17	6.25544	3.43888
Nc-1-IP_pe	chr10	80529650	80530711	1062	80529798	15	5.96953	3.52194
Nc-1-IP_pe	chr10	80533454	80534552	1099	80533920	23	10.1326	4.42559
Nc-1-IP_pe	chr10	80535305	80536697	1393	80536064	19	7.88414	3.95145
Nc-1-IP_pe	chr10	80549572	80550528	957	80549632	13	3.67377	2.58159
Nc-1-IP_pe	chr10	80554628	80554971	344	80554785	18	10.6609	5.48691
Nc-1-IP_pe	chr10	80570157	80570456	300	80570346	12	5.61153	3.70794
Nc-1-IP_pe	chr10	80571797	80572135	339	80571923	15	7.91596	4.54123
Nc-1-IP_pe	chr10	80586007	80586265	259	80586123	16	7.8117	4.32634
Nc-1-IP_pe	chr10	80589425	80589900	476	80589813	14	4.21449	2.76599
Nc-1-IP_pe	chr10	80590184	80590554	371	80590416	12	4.80688	3.25822
Nc-1-IP_pe	chr10	80603382	80603887	506	80603680	14	4.21449	2.76599
Nc-1-IP_pe	chr10	80609358	80609982	625	80609768	19	7.59289	3.82098
Nc-1-IP_pe	chr10	80614678	80615006	329	80614826	12	3.86684	2.7567
Nc-1-IP_pe	chr10	80619151	80619639	489	80619190	15	5.1177	3.10809
Nc-1-IP_pe	chr10	80630523	80630811	289	80630757	11	3.28475	2.53029

Nc-1-IP_pe	chr10	80654836	80655245	410	80654935	12	3.47963	2.55766
Nc-1-IP_pe	chr10	80656898	80657382	485	80657009	13	4.02562	2.7544
Nc-1-IP_pe	chr10	80660623	80660926	304	80660735	16	6.77412	3.79988
Nc-1-IP_pe	chr10	80674170	80674531	362	80674306	13	3.67377	2.58159
Nc-1-IP_pe	chr10	80700603	80700871	269	80700693	20	7.85753	3.8227
Nc-1-IP_pe	chr10	80705272	80705584	313	80705333	13	4.86362	3.18015
Nc-1-IP_pe	chr10	80719391	80719691	301	80719589	15	7.41419	4.269
Nc-1-IP_pe	chr10	80727752	80728394	643	80727772	13	3.29152	2.39761
Nc-1-IP_pe	chr10	80732979	80733625	647	80733157	14	6.90659	4.14556
Nc-1-IP_pe	chr10	80813936	80814562	627	80814092	14	5.74019	3.52296
Nc-1-IP_pe	chr10	80825304	80825762	459	80825725	14	6.06519	3.69269
Nc-1-IP_pe	chr10	80842600	80843061	462	80842748	11	4.83181	3.3893
Nc-1-IP_pe	chr10	80855544	80855850	307	80855686	19	6.32622	3.27682
Nc-1-IP_pe	chr10	80859059	80859435	377	80859298	13	5.57038	3.55505
Nc-1-IP_pe	chr10	80881726	80882395	670	80881896	13	4.86362	3.18015
Nc-1-IP_pe	chr10	80888809	80889287	479	80889071	13	3.67377	2.58159
Nc-1-IP_pe	chr10	80894797	80895296	500	80895196	13	3.07238	2.29378
Nc-1-IP_pe	chr10	80994440	80994695	256	80994554	12	6.42833	4.1821
Nc-1-IP_pe	chr10	81021061	81021353	293	81021201	12	4.75569	3.23024
Nc-1-IP_pe	chr10	81027623	81027953	331	81027744	13	5.36853	3.44651
Nc-1-IP_pe	chr10	81068452	81068737	286	81068644	15	6.19428	3.63445
Nc-1-IP_pe	chr10	81082971	81083688	718	81082990	12	3.58823	2.61305
Nc-1-IP_pe	chr10	81086061	81086342	282	81086177	14	6.06519	3.69269
Nc-1-IP_pe	chr10	81092534	81093394	861	81092919	16	5.82808	3.34463
Nc-1-IP_pe	chr10	81112898	81113150	253	81112984	17	7.6273	4.08876
Nc-1-IP_pe	chr10	81139673	81139938	266	81139768	17	7.6273	4.08876
Nc-1-IP_pe	chr10	81175819	81176092	274	81175931	12	6.58417	4.27436
Nc-1-IP_pe	chr10	81265022	81265357	336	81265165	13	4.90859	3.20357
Nc-1-IP_pe	chr10	81570910	81571687	778	81571582	11	4.54404	3.2242
Nc-1-IP_pe	chr10	81614011	81614766	756	81614191	12	5.84943	3.84431
Nc-1-IP_pe	chr10	81704975	81705277	303	81705174	11	6.03439	4.10251
Nc-1-IP_pe	chr10	81709930	81710229	300	81710076	14	5.51541	3.4073
Nc-1-IP_pe	chr10	81735343	81735677	335	81735444	10	4.33501	3.21677
Nc-1-IP_pe	chr10	81742995	81743301	307	81743110	13	7.7352	4.78626
Nc-1-IP_pe	chr10	81772503	81772763	261	81772628	18	4.32723	2.54542
Nc-1-IP_pe	chr10	81784225	81784580	356	81784561	14	4.60011	2.95114
Nc-1-IP_pe	chr10	81862620	81863166	547	81863000	18	7.1522	3.73811
Nc-1-IP_pe	chr10	81894699	81895037	339	81894865	12	6.21727	4.05801
Nc-1-IP_pe	chr10	81923285	81923564	280	81923449	20	7.97282	3.87239
Nc-1-IP_pe	chr10	81962662	81962991	330	81962781	14	6.06519	3.69269
Nc-1-IP_pe	chr10	82011744	82012414	671	82011913	14	7.42849	4.43574
Nc-1-IP_pe	chr10	82062235	82062508	274	82062382	15	5.1177	3.10809
Nc-1-IP_pe	chr10	82085463	82085772	310	82085756	9	3.27326	2.68684
Nc-1-IP_pe	chr10	82150015	82150342	328	82150203	12	6.48784	4.21727
Nc-1-IP_pe	chr10	82189450	82189720	271	82189673	11	4.54404	3.2242

Nc-1-IP_pe	chr10	82200964	82201277	314	82201079	11	5.7051	3.90381
Nc-1-IP_pe	chr10	82233019	82233359	341	82233227	17	10.1401	5.40027
Nc-1-IP_pe	chr10	82261726	82262000	275	82261863	10	4.95265	3.58858
Nc-1-IP_pe	chr10	82333790	82334085	296	82333940	13	6.62436	4.14003
Nc-1-IP_pe	chr10	82363474	82363726	253	82363628	12	6.50787	4.22912
Nc-1-IP_pe	chr10	82495003	82495313	311	82495192	19	13.206	6.67523
Nc-1-IP_pe	chr10	82539765	82540022	258	82539873	13	7.43036	4.60624
Nc-1-IP_pe	chr10	82617196	82617472	277	82617387	14	6.69566	4.03025
Nc-1-IP_pe	chr10	82638709	82638994	286	82638861	16	7.53857	4.18505
Nc-1-IP_pe	chr10	82669590	82669904	315	82669727	19	13.1046	6.61797
Nc-1-IP_pe	chr10	82672655	82673231	577	82673121	10	3.89064	2.95552
Nc-1-IP_pe	chr10	82675038	82675314	277	82675289	15	4.7819	2.95039
Nc-1-IP_pe	chr10	82692612	82692929	318	82692660	10	4.99888	3.61677
Nc-1-IP_pe	chr10	82715182	82715449	268	82715296	11	6.03439	4.10251
Nc-1-IP_pe	chr10	82727372	82727823	452	82727521	11	5.52654	3.79706
Nc-1-IP_pe	chr10	82745966	82746507	542	82746359	18	7.73136	4.00629
Nc-1-IP_pe	chr10	82788285	82788590	306	82788451	14	4.60011	2.95114
Nc-1-IP_pe	chr10	82816323	82816749	427	82816580	16	6.8987	3.86161
Nc-1-IP_pe	chr10	82885864	82886152	289	82886095	10	4.77495	3.48064
Nc-1-IP_pe	chr10	83057602	83057901	300	83057671	9	4.05981	3.16422
Nc-1-IP_pe	chr10	83176087	83176391	305	83176264	11	4.54404	3.2242
Nc-1-IP_pe	chr10	83230040	83230367	328	83230162	10	4.99888	3.61677
Nc-1-IP_pe	chr10	83348494	83348750	257	83348688	11	3.64839	2.72584
Nc-1-IP_pe	chr10	83355343	83355602	260	83355444	12	5.77527	3.80164
Nc-1-IP_pe	chr10	83401076	83401353	278	83401206	13	7.42513	4.60316
Nc-1-IP_pe	chr10	83527097	83527587	491	83527459	13	7.7352	4.78626
Nc-1-IP_pe	chr10	83564162	83564488	327	83564308	11	4.54404	3.2242
Nc-1-IP_pe	chr10	83564849	83565386	538	83564987	12	6.86906	4.44438
Nc-1-IP_pe	chr10	83790369	83790720	352	83790520	8	3.74505	3.07688
Nc-1-IP_pe	chr10	83791029	83791863	835	83791456	13	6.54342	4.09407
Nc-1-IP_pe	chr10	83865586	83865853	268	83865827	9	3.72192	2.95716
Nc-1-IP_pe	chr10	83879072	83879777	706	83879541	10	5.23357	3.76063
Nc-1-IP_pe	chr10	83912755	83913167	413	83912961	12	5.84943	3.84431
Nc-1-IP_pe	chr10	83957726	83958185	460	83958057	8	3.74505	3.07688
Nc-1-IP_pe	chr10	83990854	83991193	340	83990911	10	5.23357	3.76063
Nc-1-IP_pe	chr10	84015088	84015340	253	84015185	12	6.86906	4.44438
Nc-1-IP_pe	chr10	84019805	84020138	334	84019998	13	4.86362	3.18015
Nc-1-IP_pe	chr10	84075404	84075921	518	84075849	9	4.46937	3.41876
Nc-1-IP_pe	chr10	84081460	84081927	468	84081686	11	6.03439	4.10251
Nc-1-IP_pe	chr10	84139545	84139880	336	84139691	22	6.07094	2.94697
Nc-1-IP_pe	chr10	84163168	84163435	268	84163402	9	4.25966	3.28797
Nc-1-IP_pe	chr10	84180890	84181149	260	84181040	10	4.96929	3.59872
Nc-1-IP_pe	chr10	84188737	84189032	296	84188914	11	6.03439	4.10251
Nc-1-IP_pe	chr10	84195837	84196212	376	84195892	11	4.23076	3.04719
Nc-1-IP_pe	chr10	84201270	84201727	458	84201566	12	5.77527	3.80164

Nc-1-IP_pe	chr10	84215098	84215382	285	84215261	10	3.08907	2.49869
Nc-1-IP_pe	chr10	84216818	84217282	465	84216910	8	3.74505	3.07688
Nc-1-IP_pe	chr10	84224424	84224818	395	84224649	12	4.70082	3.20033
Nc-1-IP_pe	chr10	84232745	84233278	534	84232779	9	3.72192	2.95716
Nc-1-IP_pe	chr10	84237142	84237795	654	84237699	15	6.78905	3.93887
Nc-1-IP_pe	chr10	84257359	84257904	546	84257449	13	6.19316	3.89711
Nc-1-IP_pe	chr10	84261014	84261835	822	84261241	14	8.62483	5.12466
Nc-1-IP_pe	chr10	84262475	84262739	265	84262698	8	3.74505	3.07688
Nc-1-IP_pe	chr10	84280151	84280758	608	84280628	13	5.9487	3.76157
Nc-1-IP_pe	chr10	84293399	84293811	413	84293616	10	5.23357	3.76063
Nc-1-IP_pe	chr10	84321326	84321736	411	84321584	12	6.86906	4.44438
Nc-1-IP_pe	chr10	84400495	84400889	395	84400754	14	5.03085	3.16286
Nc-1-IP_pe	chr10	84415631	84416323	693	84416112	19	7.29128	3.68799
Nc-1-IP_pe	chr10	84490997	84491814	818	84491447	23	5.91101	2.82849
Nc-1-IP_pe	chr10	84502412	84502685	274	84502569	16	3.65575	2.38624
Nc-1-IP_pe	chr10	84525442	84525748	307	84525597	22	6.84408	3.22845
Nc-1-IP_pe	chr10	84533986	84534521	536	84534501	17	3.11196	2.12137
Nc-1-IP_pe	chr10	84538025	84538286	262	84538171	18	6.87353	3.61204
Nc-1-IP_pe	chr10	84540365	84541297	933	84540677	22	6.84408	3.22845
Nc-1-IP_pe	chr10	84550223	84550499	277	84550232	11	3.8706	2.84726
Nc-1-IP_pe	chr10	84565302	84565707	406	84565547	13	6.21	3.90651
Nc-1-IP_pe	chr10	84572227	84572546	320	84572421	14	6.06519	3.69269
Nc-1-IP_pe	chr10	84582246	84582715	470	84582351	13	3.07238	2.29378
Nc-1-IP_pe	chr10	84583239	84583505	267	84583422	16	6.33285	3.58457
Nc-1-IP_pe	chr10	84584793	84585283	491	84585271	12	3.83688	2.74114
Nc-1-IP_pe	chr10	84589548	84590365	818	84589692	18	6.17129	3.30277
Nc-1-IP_pe	chr10	84594186	84594737	552	84594319	12	5.37834	3.57574
Nc-1-IP_pe	chr10	84596400	84596675	276	84596450	14	6.13058	3.7272
Nc-1-IP_pe	chr10	86592016	86592269	254	86592080	9	4.46937	3.41876
Nc-1-IP_pe	chr10	86960635	86961158	524	86960787	9	4.46937	3.41876
Nc-1-IP_pe	chr10	87080696	87081086	391	87080856	13	7.7352	4.78626
Nc-1-IP_pe	chr10	87229817	87230089	273	87229952	14	5.03085	3.16286
Nc-1-IP_pe	chr10	87231503	87232052	550	87231768	14	8.63073	5.12814
Nc-1-IP_pe	chr10	87232403	87232873	471	87232582	20	14.0444	6.90474
Nc-1-IP_pe	chr10	87234264	87234642	379	87234394	9	3.27326	2.68684
Nc-1-IP_pe	chr10	87342088	87342462	375	87342328	12	4.24064	2.95299
Nc-1-IP_pe	chr10	87359478	87359864	387	87359600	16	8.26996	4.56762
Nc-1-IP_pe	chr10	87362091	87363059	969	87362221	14	6.06519	3.69269
Nc-1-IP_pe	chr10	87363611	87364229	619	87364081	15	6.78905	3.93887
Nc-1-IP_pe	chr10	87365846	87366741	896	87366624	13	5.36853	3.44651
Nc-1-IP_pe	chr10	87368787	87369160	374	87368829	9	4.25966	3.28797
Nc-1-IP_pe	chr10	87391115	87391391	277	87391286	12	6.86906	4.44438
Nc-1-IP_pe	chr10	88616379	88616943	565	88616846	9	4.46937	3.41876
Nc-1-IP_pe	chr10	88689091	88689454	364	88689238	15	8.16556	4.67894
Nc-1-IP_pe	chr10	88880120	88881000	881	88880369	17	5.9911	3.31919

Nc-1-IP_pe	chr10	88933294	88933557	264	88933456	9	4.46937	3.41876
Nc-1-IP_pe	chr10	89018857	89019109	253	89019029	10	4.07292	3.06202
Nc-1-IP_pe	chr10	89168370	89168669	300	89168506	13	7.6824	4.75494
Nc-1-IP_pe	chr10	89207951	89208685	735	89208069	14	5.03085	3.16286
Nc-1-IP_pe	chr10	89219694	89220275	582	89220136	11	6.03439	4.10251
Nc-1-IP_pe	chr10	89235291	89235574	284	89235535	12	4.24064	2.95299
Nc-1-IP_pe	chr10	89251409	89251793	385	89251689	20	9.94634	4.77022
Nc-1-IP_pe	chr10	89263979	89264239	261	89264058	17	6.47874	3.54137
Nc-1-IP_pe	chr10	89265167	89265485	319	89265411	13	6.02837	3.80557
Nc-1-IP_pe	chr10	89271795	89272143	349	89271968	11	5.89269	4.01672
Nc-1-IP_pe	chr10	89322712	89323021	310	89322905	11	5.19376	3.60018
Nc-1-IP_pe	chr10	89351053	89351309	257	89351199	9	4.32403	3.32802
Nc-1-IP_pe	chr10	89412151	89412510	360	89412377	10	3.4604	2.70797
Nc-1-IP_pe	chr10	89414861	89415372	512	89414899	13	4.04902	2.76601
Nc-1-IP_pe	chr10	89459610	89459938	329	89459717	13	6.62436	4.14003
Nc-1-IP_pe	chr10	89530482	89530781	300	89530631	15	7.46994	4.29894
Nc-1-IP_pe	chr10	89535550	89536173	624	89535714	12	5.3037	3.53374
Nc-1-IP_pe	chr10	89656062	89656318	257	89656131	9	3.72192	2.95716
Nc-1-IP_pe	chr10	89673571	89673883	313	89673648	12	5.84943	3.84431
Nc-1-IP_pe	chr10	89701218	89701648	431	89701539	14	5.51541	3.4073
Nc-1-IP_pe	chr10	89746393	89746703	311	89746620	11	6.03439	4.10251
Nc-1-IP_pe	chr10	89748085	89748343	259	89748201	9	4.46937	3.41876
Nc-1-IP_pe	chr10	89781373	89781666	294	89781572	13	6.72991	4.20021
Nc-1-IP_pe	chr10	89815441	89815767	327	89815682	9	3.27326	2.68684
Nc-1-IP_pe	chr10	89820626	89820990	365	89820809	10	5.23357	3.76063
Nc-1-IP_pe	chr10	89837126	89837734	609	89837520	10	3.89064	2.95552
Nc-1-IP_pe	chr10	89837993	89838409	417	89838198	8	3.74505	3.07688
Nc-1-IP_pe	chr10	89848672	89848934	263	89848808	11	5.77463	3.94557
Nc-1-IP_pe	chr10	89852556	89852940	385	89852936	8	3.51085	2.92785
Nc-1-IP_pe	chr10	89898882	89899238	357	89899220	8	3.74505	3.07688
Nc-1-IP_pe	chr10	89950801	89951067	267	89951004	11	4.74697	3.34038
Nc-1-IP_pe	chr10	89978764	89979120	357	89978925	12	5.84943	3.84431
Nc-1-IP_pe	chr10	90003931	90004222	292	90004030	13	7.42513	4.60316
Nc-1-IP_pe	chr10	90011329	90011810	482	90011498	18	9.39952	4.82471
Nc-1-IP_pe	chr10	90041302	90042238	937	90041490	11	5.52654	3.79706
Nc-1-IP_pe	chr10	90051485	90051817	333	90051557	11	6.03439	4.10251
Nc-1-IP_pe	chr10	90086260	90086759	500	90086323	13	6.72991	4.20021
Nc-1-IP_pe	chr10	90119774	90120388	615	90120232	12	3.83688	2.74114
Nc-1-IP_pe	chr10	90140792	90141199	408	90141109	9	3.27326	2.68684
Nc-1-IP_pe	chr10	90159079	90159364	286	90159185	11	4.06403	2.95415
Nc-1-IP_pe	chr10	90250668	90251075	408	90251000	12	4.24064	2.95299
Nc-1-IP_pe	chr10	90297257	90297580	324	90297453	14	6.69566	4.03025
Nc-1-IP_pe	chr10	90610206	90610482	277	90610378	10	5.23357	3.76063
Nc-1-IP_pe	chr10	90743006	90743624	619	90743431	14	7.42849	4.43574
Nc-1-IP_pe	chr10	90831218	90831520	303	90831322	12	6.86906	4.44438

Nc-1-IP_pe	chr10	90832064	90833027	964	90832239	13	6.92473	4.312
Nc-1-IP_pe	chr10	90857013	90857817	805	90857488	14	6.06519	3.69269
Nc-1-IP_pe	chr10	90882398	90882709	312	90882547	13	7.42513	4.60316
Nc-1-IP_pe	chr10	90920163	90920435	273	90920385	10	5.23357	3.76063
Nc-1-IP_pe	chr10	90964701	90965049	349	90964793	13	6.19316	3.89711
Nc-1-IP_pe	chr10	90999022	90999355	334	90999202	10	5.23357	3.76063
Nc-1-IP_pe	chr10	91103079	91103336	258	91103169	14	7.54286	4.50023
Nc-1-IP_pe	chr10	91103934	91104259	326	91104231	8	3.44677	2.88727
Nc-1-IP_pe	chr10	91160827	91161424	598	91161136	11	4.88957	3.42271
Nc-1-IP_pe	chr10	91164062	91164359	298	91164201	13	7.10772	4.41783
Nc-1-IP_pe	chr10	91177486	91177988	503	91177539	11	5.77463	3.94557
Nc-1-IP_pe	chr10	91220864	91221200	337	91220927	14	4.60011	2.95114
Nc-1-IP_pe	chr10	91290413	91290919	507	91290768	15	8.77559	5.02156
Nc-1-IP_pe	chr10	91306066	91306531	466	91306121	8	3.61691	2.99521
Nc-1-IP_pe	chr10	91309526	91310075	550	91309985	11	5.10577	3.5486
Nc-1-IP_pe	chr10	91323301	91323592	292	91323470	12	4.70082	3.20033
Nc-1-IP_pe	chr10	91327256	91327529	274	91327387	14	5.98179	3.64885
Nc-1-IP_pe	chr10	91337004	91337394	391	91337369	10	5.23357	3.76063
Nc-1-IP_pe	chr10	91503340	91503629	290	91503466	14	7.14701	4.27839
Nc-1-IP_pe	chr10	91526202	91526469	268	91526348	13	5.71736	3.63481
Nc-1-IP_pe	chr10	91540888	91541168	281	91541008	11	6.03439	4.10251
Nc-1-IP_pe	chr10	91581958	91582251	294	91582221	10	3.4604	2.70797
Nc-1-IP_pe	chr10	91600135	91600641	507	91600168	10	3.89064	2.95552
Nc-1-IP_pe	chr10	91632770	91633198	429	91633190	11	2.96397	2.36091
Nc-1-IP_pe	chr10	91634661	91634942	282	91634773	10	5.23357	3.76063
Nc-1-IP_pe	chr10	91639333	91639683	351	91639528	11	6.03439	4.10251
Nc-1-IP_pe	chr10	91653899	91654158	260	91654001	10	4.99888	3.61677
Nc-1-IP_pe	chr10	91689922	91690239	318	91690088	13	7.7352	4.78626
Nc-1-IP_pe	chr10	91693603	91693933	331	91693662	10	3.89064	2.95552
Nc-1-IP_pe	chr10	91699482	91699828	347	91699795	8	3.74505	3.07688
Nc-1-IP_pe	chr10	91714428	91714700	273	91714498	8	3.74505	3.07688
Nc-1-IP_pe	chr10	91722001	91722300	300	91722160	11	6.03439	4.10251
Nc-1-IP_pe	chr10	91788098	91788490	393	91788286	13	6.54342	4.09407
Nc-1-IP_pe	chr10	91848898	91849166	269	91849079	8	3.74505	3.07688
Nc-1-IP_pe	chr10	91850298	91850677	380	91850514	11	6.03439	4.10251
Nc-1-IP_pe	chr10	91887177	91887543	367	91887339	15	4.7819	2.95039
Nc-1-IP_pe	chr10	91908572	91908924	353	91908835	21	6.2632	3.08808
Nc-1-IP_pe	chr10	91936942	91937281	340	91937097	11	4.54404	3.2242
Nc-1-IP_pe	chr10	91969629	91969932	304	91969676	9	4.46937	3.41876
Nc-1-IP_pe	chr10	92047964	92048438	475	92048156	14	7.42849	4.43574
Nc-1-IP_pe	chr10	92056973	92057277	305	92057231	12	4.63088	3.16234
Nc-1-IP_pe	chr10	92073248	92073654	407	92073490	11	6.03439	4.10251
Nc-1-IP_pe	chr10	92094072	92094375	304	92094242	13	5.36853	3.44651
Nc-1-IP_pe	chr10	92115042	92115384	343	92115186	11	5.10577	3.5486
Nc-1-IP_pe	chr10	92215669	92215967	299	92215847	10	5.23357	3.76063

Nc-1-IP_pe	chr10	92227763	92228057	295	92227878	12	6.79895	4.40238
Nc-1-IP_pe	chr10	92239485	92240321	837	92239669	16	5.98198	3.41707
Nc-1-IP_pe	chr10	92279851	92280203	353	92279994	12	5.23082	3.49289
Nc-1-IP_pe	chr10	92289239	92289498	260	92289374	10	4.95265	3.58858
Nc-1-IP_pe	chr10	92291264	92291796	533	92291499	13	4.56123	3.02417
Nc-1-IP_pe	chr10	92313812	92314520	709	92313856	11	4.06403	2.95415
Nc-1-IP_pe	chr10	92336084	92336353	270	92336189	11	6.03439	4.10251
Nc-1-IP_pe	chr10	92372880	92373369	490	92373225	12	6.6095	4.28941
Nc-1-IP_pe	chr10	92376785	92377045	261	92376951	16	8.26996	4.56762
Nc-1-IP_pe	chr10	92391581	92391847	267	92391731	14	8.29543	4.93196
Nc-1-IP_pe	chr10	92393942	92394197	256	92393988	8	3.65786	3.02128
Nc-1-IP_pe	chr10	92395535	92395997	463	92395817	13	5.29187	3.40559
Nc-1-IP_pe	chr10	92398092	92398472	381	92398187	10	5.23357	3.76063
Nc-1-IP_pe	chr10	92401963	92402494	532	92402385	12	6.86906	4.44438
Nc-1-IP_pe	chr10	92407593	92407913	321	92407806	12	6.50787	4.22912
Nc-1-IP_pe	chr10	92410235	92410495	261	92410394	13	7.14943	4.44205
Nc-1-IP_pe	chr10	92419763	92420066	304	92419870	13	7.17043	4.45427
Nc-1-IP_pe	chr10	92428439	92428803	365	92428606	8	3.264	2.772
Nc-1-IP_pe	chr10	92440929	92441521	593	92441075	13	6.62436	4.14003
Nc-1-IP_pe	chr10	92451315	92451618	304	92451594	10	3.89064	2.95552
Nc-1-IP_pe	chr10	92462499	92462966	468	92462538	9	3.72192	2.95716
Nc-1-IP_pe	chr10	92466606	92466901	296	92466789	9	4.46937	3.41876
Nc-1-IP_pe	chr10	92536532	92536984	453	92536913	10	4.20113	3.13749
Nc-1-IP_pe	chr10	92543929	92544195	267	92543999	11	6.03439	4.10251
Nc-1-IP_pe	chr10	92556352	92556837	486	92556627	13	7.7352	4.78626
Nc-1-IP_pe	chr10	92557195	92557472	278	92557328	11	6.03439	4.10251
Nc-1-IP_pe	chr10	92571050	92571302	253	92571204	12	5.50092	3.64505
Nc-1-IP_pe	chr10	92574427	92574757	331	92574611	12	3.16137	2.39719
Nc-1-IP_pe	chr10	92577792	92578352	561	92577882	13	5.9487	3.76157
Nc-1-IP_pe	chr10	92593277	92593543	267	92593358	17	9.65211	5.13407
Nc-1-IP_pe	chr10	92666852	92667204	353	92667019	16	10.117	5.58955
Nc-1-IP_pe	chr10	92674850	92675171	322	92675078	10	3.89064	2.95552
Nc-1-IP_pe	chr10	92675518	92675823	306	92675701	14	4.21449	2.76599
Nc-1-IP_pe	chr10	92679754	92680644	891	92680198	12	3.47963	2.55766
Nc-1-IP_pe	chr10	92689511	92689783	273	92689601	14	5.69896	3.50164
Nc-1-IP_pe	chr10	92692329	92692656	328	92692363	13	5.29187	3.40559
Nc-1-IP_pe	chr10	92695768	92696132	365	92695940	13	4.02562	2.7544
Nc-1-IP_pe	chr10	92698640	92698995	356	92698831	13	7.47517	4.63258
Nc-1-IP_pe	chr10	92711728	92712077	350	92711846	11	6.03439	4.10251
Nc-1-IP_pe	chr10	92714666	92715023	358	92715010	10	4.39571	3.25288
Nc-1-IP_pe	chr10	92718192	92718454	263	92718313	12	6.18005	4.03623
Nc-1-IP_pe	chr10	92747775	92748249	475	92747848	9	4.20361	3.25317
Nc-1-IP_pe	chr10	92752535	92753102	568	92753004	10	3.4604	2.70797
Nc-1-IP_pe	chr10	92759429	92759746	318	92759598	12	6.58417	4.27436
Nc-1-IP_pe	chr10	92766718	92766992	275	92766878	10	5.23357	3.76063

Nc-1-IP_pe	chr10	92821174	92821635	462	92821274	10	5.23357	3.76063
Nc-1-IP_pe	chr10	92829052	92829416	365	92829256	12	5.23082	3.49289
Nc-1-IP_pe	chr10	92848827	92849292	466	92848925	17	4.13464	2.52661
Nc-1-IP_pe	chr10	92865257	92865615	359	92865400	12	5.23082	3.49289
Nc-1-IP_pe	chr10	92974596	92975969	1374	92975314	19	13.4918	6.83751
Nc-1-IP_pe	chr10	92981808	92982107	300	92981964	14	6.69566	4.03025
Nc-1-IP_pe	chr10	93050265	93050533	269	93050366	12	5.23082	3.49289
Nc-1-IP_pe	chr10	93062699	93063115	417	93062927	17	9.55913	5.08395
Nc-1-IP_pe	chr10	93066012	93066410	399	93066176	15	7.19874	4.15407
Nc-1-IP_pe	chr10	93069047	93069587	541	93069313	14	6.05554	3.68761
Nc-1-IP_pe	chr10	93074059	93074826	768	93074736	12	3.83688	2.74114
Nc-1-IP_pe	chr10	93079786	93080046	261	93080017	12	3.16137	2.39719
Nc-1-IP_pe	chr10	93091198	93091529	332	93091205	11	3.64839	2.72584
Nc-1-IP_pe	chr10	93093213	93093685	473	93093509	10	4.91962	3.56846
Nc-1-IP_pe	chr10	93097504	93097802	299	93097655	11	5.3563	3.696
Nc-1-IP_pe	chr10	93098776	93099144	369	93098990	15	9.55381	5.47001
Nc-1-IP_pe	chr10	93121592	93122159	568	93121645	10	3.08907	2.49869
Nc-1-IP_pe	chr10	93150872	93151157	286	93151023	9	4.20361	3.25317
Nc-1-IP_pe	chr10	93160776	93161148	373	93160928	12	6.86906	4.44438
Nc-1-IP_pe	chr10	93176690	93177257	568	93176895	11	3.64839	2.72584
Nc-1-IP_pe	chr10	93212248	93212543	296	93212332	15	5.66913	3.37372
Nc-1-IP_pe	chr10	93215316	93215869	554	93215719	13	7.7352	4.78626
Nc-1-IP_pe	chr10	93220907	93221362	456	93221191	13	6.92473	4.312
Nc-1-IP_pe	chr10	93276109	93276580	472	93276207	11	5.42344	3.73577
Nc-1-IP_pe	chr10	93307689	93308224	536	93308092	11	5.10577	3.5486
Nc-1-IP_pe	chr10	93334574	93335153	580	93334785	12	5.11638	3.42903
Nc-1-IP_pe	chr10	93350497	93350921	425	93350605	11	5.77463	3.94557
Nc-1-IP_pe	chr10	93386989	93387548	560	93387055	9	3.66792	2.92434
Nc-1-IP_pe	chr10	93413323	93413988	666	93413558	9	3.97689	3.11315
Nc-1-IP_pe	chr10	93422317	93422652	336	93422576	9	4.00426	3.12999
Nc-1-IP_pe	chr10	93423708	93424130	423	93424041	13	5.42865	3.47872
Nc-1-IP_pe	chr10	93425561	93425889	329	93425746	11	5.1152	3.55411
Nc-1-IP_pe	chr10	93449861	93450512	652	93450060	11	5.03835	3.5092
Nc-1-IP_pe	chr10	93453585	93453952	368	93453656	11	4.74697	3.34038
Nc-1-IP_pe	chr10	93457999	93458942	944	93458781	12	3.83688	2.74114
Nc-1-IP_pe	chr10	93459978	93460458	481	93460315	9	3.81807	3.01579
Nc-1-IP_pe	chr10	93477545	93477800	256	93477619	12	3.47963	2.55766
Nc-1-IP_pe	chr10	93497614	93498101	488	93497977	15	7.46994	4.29894
Nc-1-IP_pe	chr10	93521356	93521612	257	93521483	12	5.23082	3.49289
Nc-1-IP_pe	chr10	93535010	93535462	453	93535153	12	6.86906	4.44438
Nc-1-IP_pe	chr10	93556638	93556972	335	93556704	12	5.77527	3.80164
Nc-1-IP_pe	chr10	93557394	93557744	351	93557683	12	4.70082	3.20033
Nc-1-IP_pe	chr10	93559387	93559691	305	93559611	9	2.89249	2.46179
Nc-1-IP_pe	chr10	93566966	93567244	279	93567018	16	3.65575	2.38624
Nc-1-IP_pe	chr10	93568163	93568600	438	93568484	15	6.78905	3.93887

Nc-1-IP_pe	chr10	93600307	93600612	306	93600554	12	3.47963	2.55766
Nc-1-IP_pe	chr10	93601278	93601554	277	93601446	11	4.06403	2.95415
Nc-1-IP_pe	chr10	93641317	93641714	398	93641452	12	6.86906	4.44438
Nc-1-IP_pe	chr10	93702421	93702816	396	93702616	19	6.32622	3.27682
Nc-1-IP_pe	chr10	93712096	93712621	526	93712110	9	4.25966	3.28797
Nc-1-IP_pe	chr10	93751206	93751539	334	93751272	10	4.39571	3.25288
Nc-1-IP_pe	chr10	93773846	93774215	370	93774058	13	7.7352	4.78626
Nc-1-IP_pe	chr10	93783273	93783608	336	93783475	9	4.20361	3.25317
Nc-1-IP_pe	chr10	93810665	93810991	327	93810854	12	6.6095	4.28941
Nc-1-IP_pe	chr10	93839991	93840614	624	93840417	12	6.86906	4.44438
Nc-1-IP_pe	chr10	93845021	93845685	665	93845402	17	9.99923	5.32289
Nc-1-IP_pe	chr10	93855993	93856328	336	93856122	10	4.39571	3.25288
Nc-1-IP_pe	chr10	93958891	93959309	419	93959149	12	5.23082	3.49289
Nc-1-IP_pe	chr10	93959660	93960770	1111	93959752	12	5.23082	3.49289
Nc-1-IP_pe	chr10	93963287	93963602	316	93963375	12	6.50787	4.22912
Nc-1-IP_pe	chr10	93970345	93971078	734	93970920	17	10.6711	5.69559
Nc-1-IP_pe	chr10	93982649	93982905	257	93982711	10	4.99888	3.61677
Nc-1-IP_pe	chr10	93993507	93993809	303	93993803	11	2.96397	2.36091
Nc-1-IP_pe	chr10	93999682	94000028	347	93999936	10	5.23357	3.76063
Nc-1-IP_pe	chr10	94009821	94010182	362	94009909	14	6.06519	3.69269
Nc-1-IP_pe	chr10	94078531	94078791	261	94078674	9	4.46937	3.41876
Nc-1-IP_pe	chr10	94085508	94085861	354	94085715	8	3.14944	2.70014
Nc-1-IP_pe	chr10	94087756	94088085	330	94087886	13	4.41954	2.952
Nc-1-IP_pe	chr10	94101556	94101884	329	94101684	13	6.62436	4.14003
Nc-1-IP_pe	chr10	94108228	94108481	254	94108324	11	5.10577	3.5486
Nc-1-IP_pe	chr10	94210917	94211249	333	94211170	13	6.67312	4.1678
Nc-1-IP_pe	chr10	94222684	94223095	412	94223019	10	5.23357	3.76063
Nc-1-IP_pe	chr10	94228902	94229537	636	94229379	13	6.62436	4.14003
Nc-1-IP_pe	chr10	94234849	94235222	374	94235166	8	3.74505	3.07688
Nc-1-IP_pe	chr10	94288658	94288917	260	94288890	11	4.00079	2.91908
Nc-1-IP_pe	chr10	94310289	94310581	293	94310388	11	5.19376	3.60018
Nc-1-IP_pe	chr10	94314013	94314526	514	94314391	11	6.03439	4.10251
Nc-1-IP_pe	chr10	94322455	94322782	328	94322638	10	5.23357	3.76063
Nc-1-IP_pe	chr10	94381166	94381475	310	94381287	10	4.39571	3.25288
Nc-1-IP_pe	chr10	94384818	94385230	413	94385028	15	8.26005	4.73146
Nc-1-IP_pe	chr10	94403084	94403716	633	94403464	17	5.14803	2.94914
Nc-1-IP_pe	chr10	94411433	94411879	447	94411517	9	4.46937	3.41876
Nc-1-IP_pe	chr10	94458711	94459050	340	94458831	10	4.39571	3.25288
Nc-1-IP_pe	chr10	94459331	94459869	539	94459458	17	9.09431	4.8363
Nc-1-IP_pe	chr10	94578439	94578700	262	94578572	10	5.23357	3.76063
Nc-1-IP_pe	chr10	94616712	94617122	411	94616870	10	4.39571	3.25288
Nc-1-IP_pe	chr10	94624313	94625070	758	94624388	10	4.62142	3.388
Nc-1-IP_pe	chr10	94639711	94640098	388	94639894	9	4.46937	3.41876
Nc-1-IP_pe	chr10	94662881	94663336	456	94662987	11	4.74697	3.34038
Nc-1-IP_pe	chr10	94695940	94696314	375	94696060	11	5.52654	3.79706

Nc-1-IP_pe	chr10	94703809	94704155	347	94703937	15	8.26005	4.73146
Nc-1-IP_pe	chr10	94793828	94794274	447	94794160	12	2.87625	2.25568
Nc-1-IP_pe	chr10	94857859	94858512	654	94858136	11	5.77463	3.94557
Nc-1-IP_pe	chr10	94879152	94880001	850	94879808	17	10.3998	5.544
Nc-1-IP_pe	chr10	94898110	94898456	347	94898229	9	4.05981	3.16422
Nc-1-IP_pe	chr10	94972396	94972661	266	94972557	15	9.55381	5.47001
Nc-1-IP_pe	chr10	95079637	95080005	369	95079884	12	4.24064	2.95299
Nc-1-IP_pe	chr10	95135956	95136319	364	95136034	11	4.74697	3.34038
Nc-1-IP_pe	chr10	95136754	95137037	284	95136866	13	5.86991	3.71823
Nc-1-IP_pe	chr10	95195961	95196372	412	95196237	12	6.58417	4.27436
Nc-1-IP_pe	chr10	95230893	95231521	629	95231018	15	4.06016	2.62146
Nc-1-IP_pe	chr10	95236671	95237213	543	95237169	13	3.67377	2.58159
Nc-1-IP_pe	chr10	95241398	95241843	446	95241612	9	3.27326	2.68684
Nc-1-IP_pe	chr10	95250957	95251403	447	95251119	12	6.29276	4.10227
Nc-1-IP_pe	chr10	95268541	95268853	313	95268716	16	7.53857	4.18505
Nc-1-IP_pe	chr10	95269129	95269427	299	95269169	9	3.27326	2.68684
Nc-1-IP_pe	chr10	95289871	95290163	293	95289967	14	3.86727	2.6027
Nc-1-IP_pe	chr10	95295077	95295742	666	95295411	13	5.03208	3.26819
Nc-1-IP_pe	chr10	95332623	95332904	282	95332759	12	5.79203	3.81127
Nc-1-IP_pe	chr10	95336295	95336737	443	95336552	19	7.84735	3.93486
Nc-1-IP_pe	chr10	95353549	95353811	263	95353789	10	4.47495	3.30017
Nc-1-IP_pe	chr10	95384432	95384693	262	95384652	11	5.03835	3.5092
Nc-1-IP_pe	chr10	95407300	95407593	294	95407418	11	4.54404	3.2242
Nc-1-IP_pe	chr10	95415460	95415779	320	95415590	9	3.9633	3.10479
Nc-1-IP_pe	chr10	95478952	95479257	306	95479204	8	3.38431	2.8478
Nc-1-IP_pe	chr10	95495851	95496155	305	95496111	10	4.93609	3.57849
Nc-1-IP_pe	chr10	95519074	95519344	271	95519252	14	8.25309	4.90735
Nc-1-IP_pe	chr10	95530836	95531294	459	95530892	10	3.08907	2.49869
Nc-1-IP_pe	chr10	95533548	95533919	372	95533706	11	6.03439	4.10251
Nc-1-IP_pe	chr10	95534198	95534517	320	95534367	12	6.86906	4.44438
Nc-1-IP_pe	chr10	95541002	95541585	584	95541249	12	3.83688	2.74114
Nc-1-IP_pe	chr10	95552116	95552376	261	95552197	10	5.23357	3.76063
Nc-1-IP_pe	chr10	95560465	95561184	720	95560821	17	8.02964	4.28847
Nc-1-IP_pe	chr10	95591800	95592130	331	95591900	10	4.62142	3.388
Nc-1-IP_pe	chr10	95605313	95605728	416	95605597	12	3.47963	2.55766
Nc-1-IP_pe	chr10	95656010	95656786	777	95656490	17	5.54967	3.12324
Nc-1-IP_pe	chr10	95662805	95663252	448	95663150	8	3.74505	3.07688
Nc-1-IP_pe	chr10	95694713	95695199	487	95694824	10	4.99888	3.61677
Nc-1-IP_pe	chr10	95707063	95707325	263	95707136	14	6.44441	3.89444
Nc-1-IP_pe	chr10	95806810	95807634	825	95806906	13	7.38599	4.58019
Nc-1-IP_pe	chr10	95809134	95810144	1011	95809909	15	8.16556	4.67894
Nc-1-IP_pe	chr10	95822221	95822632	412	95822290	11	5.7051	3.90381
Nc-1-IP_pe	chr10	95933724	95934198	475	95933965	12	5.45448	3.61875
Nc-1-IP_pe	chr10	95934594	95934847	254	95934762	12	4.70082	3.20033
Nc-1-IP_pe	chr10	96062609	96062887	279	96062789	9	4.25966	3.28797

Nc-1-IP_pe	chr10	96089753	96090227	475	96090024	12	4.39405	3.03475
Nc-1-IP_pe	chr10	96097872	96098135	264	96097942	13	7.7352	4.78626
Nc-1-IP_pe	chr10	96185728	96186050	323	96185928	11	3.28475	2.53029
Nc-1-IP_pe	chr10	96214182	96214702	521	96214348	13	6.72991	4.20021
Nc-1-IP_pe	chr10	96224192	96224493	302	96224372	11	5.77463	3.94557
Nc-1-IP_pe	chr10	96227342	96227716	375	96227518	14	6.06519	3.69269
Nc-1-IP_pe	chr10	96235799	96236407	609	96235885	12	4.24064	2.95299
Nc-1-IP_pe	chr10	96249412	96249729	318	96249481	9	4.46937	3.41876
Nc-1-IP_pe	chr10	96266385	96266762	378	96266573	15	6.19428	3.63445
Nc-1-IP_pe	chr10	96371654	96371929	276	96371809	13	5.65792	3.60248
Nc-1-IP_pe	chr10	96373493	96373945	453	96373629	11	2.96397	2.36091
Nc-1-IP_pe	chr10	96384464	96384910	447	96384789	13	5.9487	3.76157
Nc-1-IP_pe	chr10	96390447	96390729	283	96390618	10	5.08818	3.67138
Nc-1-IP_pe	chr10	96394263	96394543	281	96394434	10	4.33501	3.21677
Nc-1-IP_pe	chr10	96396922	96397218	297	96397009	11	5.79772	3.95946
Nc-1-IP_pe	chr10	96406082	96407243	1162	96406398	12	3.16137	2.39719
Nc-1-IP_pe	chr10	96414260	96414814	555	96414419	10	4.33501	3.21677
Nc-1-IP_pe	chr10	96420782	96421044	263	96420891	11	5.93136	4.04009
Nc-1-IP_pe	chr10	96421423	96421680	258	96421591	9	3.72192	2.95716
Nc-1-IP_pe	chr10	96422513	96422765	253	96422676	14	5.51541	3.4073
Nc-1-IP_pe	chr10	96441131	96441716	586	96441162	9	3.72192	2.95716
Nc-1-IP_pe	chr10	96442213	96442636	424	96442426	10	4.39571	3.25288
Nc-1-IP_pe	chr10	96463481	96463922	442	96463686	12	6.86906	4.44438
Nc-1-IP_pe	chr10	96486764	96487382	619	96486915	14	7.42849	4.43574
Nc-1-IP_pe	chr10	96493766	96494796	1031	96494155	11	6.03439	4.10251
Nc-1-IP_pe	chr10	96510858	96511752	895	96511283	11	5.3563	3.696
Nc-1-IP_pe	chr10	96513224	96514271	1048	96514094	13	4.86362	3.18015
Nc-1-IP_pe	chr10	96563267	96563527	261	96563436	7	3.06442	2.73501
Nc-1-IP_pe	chr10	96586821	96587253	433	96587009	15	4.7819	2.95039
Nc-1-IP_pe	chr10	96601960	96602415	456	96602287	9	4.46937	3.41876
Nc-1-IP_pe	chr10	96609694	96610118	425	96609975	12	6.31185	4.11348
Nc-1-IP_pe	chr10	96610996	96611464	469	96611162	13	5.71736	3.63481
Nc-1-IP_pe	chr10	96615753	96616200	448	96615819	10	4.93609	3.57849
Nc-1-IP_pe	chr10	96704280	96704533	254	96704462	9	3.27326	2.68684
Nc-1-IP_pe	chr10	96726492	96726846	355	96726584	11	5.77463	3.94557
Nc-1-IP_pe	chr10	96776452	96776738	287	96776604	13	7.7352	4.78626
Nc-1-IP_pe	chr10	96799464	96799753	290	96799499	9	4.46937	3.41876
Nc-1-IP_pe	chr10	96808835	96809157	323	96809021	10	5.23357	3.76063
Nc-1-IP_pe	chr10	96819059	96819529	471	96819458	11	5.10577	3.5486
Nc-1-IP_pe	chr10	96826976	96827477	502	96827306	12	6.58417	4.27436
Nc-1-IP_pe	chr10	96855730	96856173	444	96856050	15	6.19428	3.63445
Nc-1-IP_pe	chr10	96862207	96862481	275	96862476	11	3.28475	2.53029
Nc-1-IP_pe	chr10	96878858	96879115	258	96878989	10	3.08907	2.49869
Nc-1-IP_pe	chr10	96933557	96933823	267	96933713	11	6.03439	4.10251
Nc-1-IP_pe	chr10	96940727	96941043	317	96940849	22	10.0534	4.52509

Nc-1-IP_pe	chr10	96941497	96941869	373	96941677	22	7.38228	3.43115
Nc-1-IP_pe	chr10	96965076	96965664	589	96965540	12	6.86906	4.44438
Nc-1-IP_pe	chr10	96982222	96982527	306	96982319	11	6.03439	4.10251
Nc-1-IP_pe	chr10	96995048	96995374	327	96995264	11	6.03439	4.10251
Nc-1-IP_pe	chr10	96998742	96999516	775	96999412	14	5.51541	3.4073
Nc-1-IP_pe	chr10	97000009	97000628	620	97000562	13	4.86362	3.18015
Nc-1-IP_pe	chr10	97003380	97004012	633	97003490	10	4.11081	3.08427
Nc-1-IP_pe	chr10	97010926	97011656	731	97011146	13	7.7352	4.78626
Nc-1-IP_pe	chr10	97012532	97012846	315	97012606	11	4.54404	3.2242
Nc-1-IP_pe	chr10	97013644	97014841	1198	97014223	13	6.19316	3.89711
Nc-1-IP_pe	chr10	97016992	97017329	338	97017103	13	6.62436	4.14003
Nc-1-IP_pe	chr10	97019216	97019623	408	97019543	14	5.03085	3.16286
Nc-1-IP_pe	chr10	97035456	97035859	404	97035603	16	6.1093	3.47747
Nc-1-IP_pe	chr10	97037259	97037564	306	97037268	9	3.43499	2.78365
Nc-1-IP_pe	chr10	97039842	97040217	376	97039917	11	4.54404	3.2242
Nc-1-IP_pe	chr10	97047569	97048267	699	97047931	14	7.90726	4.70771
Nc-1-IP_pe	chr10	97049048	97049637	590	97049377	15	6.5434	3.81197
Nc-1-IP_pe	chr10	97059011	97059301	291	97059171	12	5.01934	3.37518
Nc-1-IP_pe	chr10	97060505	97060811	307	97060685	13	4.41954	2.952
Nc-1-IP_pe	chr10	97062315	97062802	488	97062769	12	4.1728	2.91706
Nc-1-IP_pe	chr10	97073383	97073651	269	97073569	15	7.45104	4.28878
Nc-1-IP_pe	chr10	97098194	97098746	553	97098271	10	4.51818	3.32604
Nc-1-IP_pe	chr10	97102564	97102877	314	97102854	10	3.4604	2.70797
Nc-1-IP_pe	chr10	97106886	97107431	546	97106974	11	4.54404	3.2242
Nc-1-IP_pe	chr10	97111810	97112396	587	97112357	11	4.06403	2.95415
Nc-1-IP_pe	chr10	97115488	97115941	454	97115577	12	4.75569	3.23024
Nc-1-IP_pe	chr10	97116298	97116666	369	97116514	11	4.06403	2.95415
Nc-1-IP_pe	chr10	97128403	97128752	350	97128447	10	4.30776	3.2006
Nc-1-IP_pe	chr10	97135457	97135729	273	97135671	11	5.10577	3.5486
Nc-1-IP_pe	chr10	97139005	97139365	361	97139168	13	7.65891	4.74103
Nc-1-IP_pe	chr10	97140145	97140675	531	97140496	10	5.23357	3.76063
Nc-1-IP_pe	chr10	97151047	97151636	590	97151238	15	8.63375	4.94115
Nc-1-IP_pe	chr10	97156119	97156500	382	97156187	11	3.64839	2.72584
Nc-1-IP_pe	chr10	97163824	97164089	266	97163926	15	5.20142	3.14789
Nc-1-IP_pe	chr10	97169241	97169736	496	97169542	14	5.98179	3.64885
Nc-1-IP_pe	chr10	97174065	97174635	571	97174552	16	6.33285	3.58457
Nc-1-IP_pe	chr10	97185056	97185349	294	97185339	12	3.16137	2.39719
Nc-1-IP_pe	chr10	97187810	97188487	678	97188163	12	3.16137	2.39719
Nc-1-IP_pe	chr10	97189167	97189443	277	97189272	11	4.54404	3.2242
Nc-1-IP_pe	chr10	97196638	97196996	359	97196691	12	3.16137	2.39719
Nc-1-IP_pe	chr10	97203830	97204207	378	97203980	9	3.68012	2.93175
Nc-1-IP_pe	chr10	97267622	97267911	290	97267813	12	6.86906	4.44438
Nc-1-IP_pe	chr10	97275338	97277136	1799	97276909	16	8.17084	4.51499
Nc-1-IP_pe	chr10	97292318	97292578	261	97292482	20	6.49719	3.259
Nc-1-IP_pe	chr10	97302122	97302569	448	97302452	13	6.54342	4.09407

Nc-1-IP_pe	chr10	97303272	97303587	316	97303434	9	4.33944	3.33761
Nc-1-IP_pe	chr10	97321064	97321526	463	97321407	14	5.03085	3.16286
Nc-1-IP_pe	chr10	97330475	97330914	440	97330768	10	4.33501	3.21677
Nc-1-IP_pe	chr10	97332793	97333084	292	97332962	10	4.39571	3.25288
Nc-1-IP_pe	chr10	97333821	97334109	289	97333942	14	5.838	3.57373
Nc-1-IP_pe	chr10	97335547	97335970	424	97335757	9	4.4496	3.40639
Nc-1-IP_pe	chr10	97345649	97346107	459	97345710	12	6.08847	3.9828
Nc-1-IP_pe	chr10	97359904	97360224	321	97359995	12	5.15877	3.45264
Nc-1-IP_pe	chr10	97381382	97381892	511	97381569	12	5.64372	3.72631
Nc-1-IP_pe	chr10	97385483	97386037	555	97385931	14	7.6052	4.5355
Nc-1-IP_pe	chr10	97390832	97391085	254	97391025	13	6.62436	4.14003
Nc-1-IP_pe	chr10	97407473	97407808	336	97407699	12	6.19862	4.04709
Nc-1-IP_pe	chr10	97418829	97419184	356	97418898	11	4.73303	3.33236
Nc-1-IP_pe	chr10	97432397	97432671	275	97432535	17	7.02067	3.79543
Nc-1-IP_pe	chr10	97436757	97437213	457	97437079	13	6.96475	4.33508
Nc-1-IP_pe	chr10	97445515	97445844	330	97445791	14	4.21449	2.76599
Nc-1-IP_pe	chr10	97446785	97447192	408	97447023	12	5.45448	3.61875
Nc-1-IP_pe	chr10	97447571	97447849	279	97447661	12	3.83688	2.74114
Nc-1-IP_pe	chr10	97451610	97452288	679	97451731	13	4.41954	2.952
Nc-1-IP_pe	chr10	97461733	97462048	316	97461901	8	3.27576	2.7794
Nc-1-IP_pe	chr10	97468673	97468943	271	97468848	11	5.61473	3.84969
Nc-1-IP_pe	chr10	97473097	97473350	254	97473160	12	6.86906	4.44438
Nc-1-IP_pe	chr10	97473843	97474503	661	97473929	11	4.06403	2.95415
Nc-1-IP_pe	chr10	97477866	97478310	445	97477920	8	3.74505	3.07688
Nc-1-IP_pe	chr10	97484702	97484957	256	97484757	11	6.00992	4.08766
Nc-1-IP_pe	chr10	97493047	97493335	289	97493116	7	2.98886	2.68558
Nc-1-IP_pe	chr10	97498982	97499247	266	97499145	19	6.0077	3.14582
Nc-1-IP_pe	chr10	97560987	97561258	272	97561103	9	4.46937	3.41876
Nc-1-IP_pe	chr10	97570425	97571111	687	97570895	17	5.9911	3.31919
Nc-1-IP_pe	chr10	97607211	97607475	265	97607432	8	3.5501	2.95276
Nc-1-IP_pe	chr10	97609405	97609735	331	97609615	11	4.06403	2.95415
Nc-1-IP_pe	chr10	97616068	97616328	261	97616207	11	3.64839	2.72584
Nc-1-IP_pe	chr10	97617521	97618224	704	97617725	11	4.23076	3.04719
Nc-1-IP_pe	chr10	97640759	97641289	531	97640930	16	5.50606	3.19508
Nc-1-IP_pe	chr10	97660077	97660547	471	97660361	9	2.89249	2.46179
Nc-1-IP_pe	chr10	97714706	97715229	524	97715102	14	5.30793	3.30182
Nc-1-IP_pe	chr10	97736649	97738069	1421	97737425	24	11.6643	4.93931
Nc-1-IP_pe	chr10	97744346	97745374	1029	97744673	14	6.69566	4.03025
Nc-1-IP_pe	chr10	97749309	97749820	512	97749602	14	5.51541	3.4073
Nc-1-IP_pe	chr10	97752952	97753283	332	97753154	11	5.22568	3.61895
Nc-1-IP_pe	chr10	97755265	97755603	339	97755457	13	6.24389	3.92544
Nc-1-IP_pe	chr10	97761114	97761392	279	97761177	10	3.76347	2.88178
Nc-1-IP_pe	chr10	97762690	97762980	291	97762818	13	5.29187	3.40559
Nc-1-IP_pe	chr10	97764921	97765856	936	97765593	13	6.04456	3.81453
Nc-1-IP_pe	chr10	97770174	97770702	529	97770513	16	6.80394	3.81462

Nc-1-IP_pe	chr10	97774557	97775068	512	97774858	11	4.06403	2.95415
Nc-1-IP_pe	chr10	97782510	97783179	670	97782592	10	3.89064	2.95552
Nc-1-IP_pe	chr10	97786206	97786758	553	97786371	14	5.51541	3.4073
Nc-1-IP_pe	chr10	97789696	97790092	397	97789879	14	7.05305	4.22631
Nc-1-IP_pe	chr10	97803517	97803879	363	97803841	9	3.71705	2.9542
Nc-1-IP_pe	chr10	97811498	97812133	636	97812061	12	5.72541	3.77304
Nc-1-IP_pe	chr10	97816648	97817004	357	97816955	10	3.50482	2.73328
Nc-1-IP_pe	chr10	97823148	97823617	470	97823452	11	4.77502	3.35653
Nc-1-IP_pe	chr10	97824199	97824591	393	97824446	15	4.7819	2.95039
Nc-1-IP_pe	chr10	97825980	97826546	567	97826281	12	5.03305	3.38277
Nc-1-IP_pe	chr10	97832096	97832805	710	97832128	11	5.10577	3.5486
Nc-1-IP_pe	chr10	97849653	97850560	908	97850383	16	5.37454	3.13479
Nc-1-IP_pe	chr10	97851476	97851870	395	97851782	9	4.04582	3.15559
Nc-1-IP_pe	chr10	97869323	97869593	271	97869476	14	4.21449	2.76599
Nc-1-IP_pe	chr10	97880786	97882745	1960	97881648	13	4.86362	3.18015
Nc-1-IP_pe	chr10	97883517	97883815	299	97883796	10	3.91409	2.96917
Nc-1-IP_pe	chr10	97891421	97891787	367	97891489	9	3.72192	2.95716
Nc-1-IP_pe	chr10	97896693	97897389	697	97896773	13	6.62436	4.14003
Nc-1-IP_pe	chr10	97900948	97901475	528	97901392	10	3.94999	2.99009
Nc-1-IP_pe	chr10	97903466	97904694	1229	97904232	12	5.77527	3.80164
Nc-1-IP_pe	chr10	97906796	97907268	473	97906940	12	5.77527	3.80164
Nc-1-IP_pe	chr10	97913606	97914269	664	97913814	12	5.61153	3.70794
Nc-1-IP_pe	chr10	97914801	97915499	699	97915031	17	7.02067	3.79543
Nc-1-IP_pe	chr10	97915850	97916132	283	97916064	12	5.84943	3.84431
Nc-1-IP_pe	chr10	97917501	97917791	291	97917621	14	6.96097	4.17548
Nc-1-IP_pe	chr10	97925887	97926245	359	97926158	12	5.23082	3.49289
Nc-1-IP_pe	chr10	97974968	97975249	282	97975073	10	5.23357	3.76063
Nc-1-IP_pe	chr10	97975504	97976062	559	97975956	12	5.23082	3.49289
Nc-1-IP_pe	chr10	97979089	97979421	333	97979130	13	5.36853	3.44651
Nc-1-IP_pe	chr10	97985567	97985912	346	97985618	11	5.77463	3.94557
Nc-1-IP_pe	chr10	97999071	97999415	345	97999193	12	6.86906	4.44438
Nc-1-IP_pe	chr10	98000012	98000637	626	98000483	11	6.03439	4.10251
Nc-1-IP_pe	chr10	98010006	98010311	306	98010172	11	5.89269	4.01672
Nc-1-IP_pe	chr10	98031251	98031576	326	98031359	12	5.64372	3.72631
Nc-1-IP_pe	chr10	98049110	98049478	369	98049144	9	4.46937	3.41876
Nc-1-IP_pe	chr10	98080679	98080964	286	98080864	15	7.56326	4.34922
Nc-1-IP_pe	chr10	98081869	98082155	287	98082017	10	5.23357	3.76063
Nc-1-IP_pe	chr10	98083279	98083635	357	98083492	9	4.46937	3.41876
Nc-1-IP_pe	chr10	98086166	98086683	518	98086167	9	2.89249	2.46179
Nc-1-IP_pe	chr10	98088615	98089002	388	98088653	11	5.77463	3.94557
Nc-1-IP_pe	chr10	98091922	98092202	281	98092131	10	5.14046	3.70342
Nc-1-IP_pe	chr10	98134530	98135187	658	98134709	12	4.24064	2.95299
Nc-1-IP_pe	chr10	98152818	98153076	259	98152971	10	4.33501	3.21677
Nc-1-IP_pe	chr10	98259633	98259893	261	98259781	11	4.02303	2.9314
Nc-1-IP_pe	chr10	98261593	98262304	712	98261703	16	7.97877	4.4137

Nc-1-IP_pe chr10	98264380	98265202	823	98264962	14	4.60011	2.95114
Nc-1-IP_pe chr10	98267829	98268109	281	98267868	13	4.56123	3.02417
Nc-1-IP_pe chr10	98270259	98271234	976	98270966	15	7.85543	4.50806
Nc-1-IP_pe chr10	98274940	98275602	663	98275046	10	3.89064	2.95552
Nc-1-IP_pe chr10	98293553	98293841	289	98293676	11	3.64839	2.72584
Nc-1-IP_pe chr10	98296205	98296462	258	98296207	9	3.27326	2.68684
Nc-1-IP_pe chr10	98300621	98300938	318	98300700	12	5.84943	3.84431
Nc-1-IP_pe chr10	98301701	98301956	256	98301815	11	4.54404	3.2242
Nc-1-IP_pe chr10	98311035	98311352	318	98311221	13	3.35769	2.42919
Nc-1-IP_pe chr10	98311813	98312152	340	98311996	10	4.30776	3.2006
Nc-1-IP_pe chr10	98312428	98312971	544	98312475	9	3.47995	2.81069
Nc-1-IP_pe chr10	98315482	98316011	530	98315802	12	5.94619	3.9002
Nc-1-IP_pe chr10	98320932	98321235	304	98321144	10	3.4604	2.70797
Nc-1-IP_pe chr10	98326915	98327424	510	98327050	12	4.24064	2.95299
Nc-1-IP_pe chr10	98357120	98357482	363	98357275	13	7.29853	4.52898
Nc-1-IP_pe chr10	98363840	98364378	539	98363933	10	4.39571	3.25288
Nc-1-IP_pe chr10	98364700	98365930	1231	98365879	9	4.25966	3.28797
Nc-1-IP_pe chr10	98375022	98375565	544	98375274	15	8.26005	4.73146
Nc-1-IP_pe chr10	98382921	98383397	477	98383185	13	7.36397	4.56728
Nc-1-IP_pe chr10	98387671	98388021	351	98387861	15	6.78905	3.93887
Nc-1-IP_pe chr10	98389671	98390260	590	98390222	9	3.46864	2.80388
Nc-1-IP_pe chr10	98405372	98405768	397	98405541	10	4.75926	3.47115
Nc-1-IP_pe chr10	98410552	98410842	291	98410571	8	3.08328	2.65878
Nc-1-IP_pe chr10	98417146	98417459	314	98417306	13	6.19316	3.89711
Nc-1-IP_pe chr10	98422403	98422763	361	98422642	11	4.90416	3.43117
Nc-1-IP_pe chr10	98423426	98423802	377	98423635	13	4.86362	3.18015
Nc-1-IP_pe chr10	98430357	98430680	324	98430596	12	5.8598	3.85029
Nc-1-IP_pe chr10	98430975	98431481	507	98431281	15	7.46994	4.29894
Nc-1-IP_pe chr10	98442953	98443213	261	98443005	12	4.24064	2.95299
Nc-1-IP_pe chr10	98446773	98447720	948	98446939	16	7.44163	4.13536
Nc-1-IP_pe chr10	98466987	98467283	297	98467273	10	3.40382	2.67582
Nc-1-IP_pe chr10	98510335	98510644	310	98510454	9	4.20361	3.25317
Nc-1-IP_pe chr10	98539214	98539497	284	98539387	13	5.9487	3.76157
Nc-1-IP_pe chr10	98541423	98541723	301	98541717	9	3.27326	2.68684
Nc-1-IP_pe chr10	98550311	98550598	288	98550435	9	4.46937	3.41876
Nc-1-IP_pe chr10	98598309	98599390	1082	98598439	11	5.77463	3.94557
Nc-1-IP_pe chr10	98796548	98796868	321	98796684	12	6.58417	4.27436
Nc-1-IP_pe chr10	98937142	98937541	400	98937339	16	8.26996	4.56762
Nc-1-IP_pe chr10	99067237	99067557	321	99067442	9	4.46937	3.41876
Nc-1-IP_pe chr10	99185484	99185739	256	99185687	10	5.23357	3.76063
Nc-1-IP_pe chr10	99232305	99232920	616	99232502	10	4.39571	3.25288
Nc-1-IP_pe chr10	99233759	99234536	778	99234349	12	6.86906	4.44438
Nc-1-IP_pe chr10	99239542	99240006	465	99239676	9	4.46937	3.41876
Nc-1-IP_pe chr10	99246426	99246685	260	99246538	10	5.23357	3.76063
Nc-1-IP_pe chr10	99315940	99316268	329	99316067	12	6.58417	4.27436

Nc-1-IP_pe	chr10	99329474	99329830	357	99329516	13	4.45253	2.96876
Nc-1-IP_pe	chr10	99343525	99343871	347	99343649	8	3.7135	3.05675
Nc-1-IP_pe	chr10	99360754	99361030	277	99360850	11	5.77463	3.94557
Nc-1-IP_pe	chr10	99362131	99362514	384	99362177	11	4.00079	2.91908
Nc-1-IP_pe	chr10	99388012	99388271	260	99388113	11	4.61014	3.26192
Nc-1-IP_pe	chr10	99444387	99444940	554	99444861	9	4.46937	3.41876
Nc-1-IP_pe	chr10	99509223	99509544	322	99509392	11	6.03439	4.10251
Nc-1-IP_pe	chr10	99531006	99531407	402	99531124	10	4.77495	3.48064
Nc-1-IP_pe	chr10	99534788	99535337	550	99535124	17	9.65211	5.13407
Nc-1-IP_pe	chr10	99535711	99536259	549	99536088	12	6.31185	4.11348
Nc-1-IP_pe	chr10	99540393	99541177	785	99541119	12	3.47963	2.55766
Nc-1-IP_pe	chr10	99542230	99542777	548	99542696	12	5.23082	3.49289
Nc-1-IP_pe	chr10	99558893	99559185	293	99558956	12	5.77527	3.80164
Nc-1-IP_pe	chr10	99581018	99581307	290	99581167	17	8.31233	4.43123
Nc-1-IP_pe	chr10	99610787	99611257	471	99611156	13	5.9487	3.76157
Nc-1-IP_pe	chr10	99646155	99646572	418	99646237	14	5.03085	3.16286
Nc-1-IP_pe	chr10	99658691	99659037	347	99658944	15	4.40338	2.77622
Nc-1-IP_pe	chr10	99697913	99698167	255	99698020	12	6.12485	4.004
Nc-1-IP_pe	chr10	99781784	99782044	261	99781850	9	4.4496	3.40639
Nc-1-IP_pe	chr10	99795308	99795576	269	99795501	12	5.23082	3.49289
Nc-1-IP_pe	chr10	99816141	99816908	768	99816688	15	9.55381	5.47001
Nc-1-IP_pe	chr10	99827010	99827271	262	99827140	14	6.69566	4.03025
Nc-1-IP_pe	chr10	99834738	99835176	439	99834829	13	5.8082	3.68441
Nc-1-IP_pe	chr10	99838780	99839369	590	99839209	71	29.2853	5.16915
Nc-1-IP_pe	chr10	99846709	99847125	417	99846865	15	6.85973	3.97568
Nc-1-IP_pe	chr10	99879682	99880228	547	99880120	12	4.75569	3.23024
Nc-1-IP_pe	chr10	99885165	99885679	515	99885333	14	8.59917	5.10958
Nc-1-IP_pe	chr10	99891755	99892259	505	99891819	13	5.86991	3.71823
Nc-1-IP_pe	chr10	99917678	99918147	470	99917748	10	5.23357	3.76063
Nc-1-IP_pe	chr10	99920686	99920953	268	99920881	9	3.66792	2.92434
Nc-1-IP_pe	chr10	99950972	99951484	513	99951127	10	4.90322	3.55848
Nc-1-IP_pe	chr10	99955422	99955768	347	99955643	11	4.06403	2.95415
Nc-1-IP_pe	chr10	99956405	99956784	380	99956620	11	5.52654	3.79706
Nc-1-IP_pe	chr10	99960646	99960958	313	99960774	10	4.39571	3.25288
Nc-1-IP_pe	chr10	99961375	99961666	292	99961563	12	6.58417	4.27436
Nc-1-IP_pe	chr10	99981681	99982319	639	99982177	14	5.51541	3.4073
Nc-1-IP_pe	chr10	1E+08	1E+08	391	1E+08	16	8.26996	4.56762
Nc-1-IP_pe	chr10	1E+08	1E+08	384	1E+08	9	2.89249	2.46179
Nc-1-IP_pe	chr10	1E+08	1E+08	510	1E+08	10	4.77495	3.48064
Nc-1-IP_pe	chr10	1E+08	1E+08	348	1E+08	11	5.10577	3.5486
Nc-1-IP_pe	chr10	1E+08	1E+08	275	1E+08	13	5.9487	3.76157
Nc-1-IP_pe	chr10	1E+08	1E+08	309	1E+08	9	3.27326	2.68684
Nc-1-IP_pe	chr10	1E+08	1E+08	309	1E+08	12	5.77527	3.80164
Nc-1-IP_pe	chr10	1E+08	1E+08	693	1E+08	13	4.24481	2.8638
Nc-1-IP_pe	chr10	1E+08	1E+08	372	1E+08	13	7.70601	4.76894

Nc-1-IP_pe	chr10	1E+08	1E+08	1014	1E+08	15	9.55381	5.47001
Nc-1-IP_pe	chr10	1E+08	1E+08	690	1E+08	10	4.33501	3.21677
Nc-1-IP_pe	chr10	1E+08	1E+08	299	1E+08	14	8.63073	5.12814
Nc-1-IP_pe	chr10	1E+08	1E+08	279	1E+08	9	4.46937	3.41876
Nc-1-IP_pe	chr10	1E+08	1E+08	371	1E+08	25	11.5115	4.73286
Nc-1-IP_pe	chr10	1E+08	1E+08	276	1E+08	14	7.34079	4.38651
Nc-1-IP_pe	chr10	1E+08	1E+08	342	1E+08	20	7.32334	3.59641
Nc-1-IP_pe	chr10	1E+08	1E+08	472	1E+08	13	5.36853	3.44651
Nc-1-IP_pe	chr10	1E+08	1E+08	452	1E+08	10	4.39571	3.25288
Nc-1-IP_pe	chr10	1E+08	1E+08	1369	1E+08	16	10.3499	5.72347
Nc-1-IP_pe	chr10	1E+08	1E+08	299	1E+08	12	4.63088	3.16234
Nc-1-IP_pe	chr10	1E+08	1E+08	284	1E+08	15	7.46994	4.29894
Nc-1-IP_pe	chr10	1E+08	1E+08	562	1E+08	13	6.54342	4.09407
Nc-1-IP_pe	chr10	1E+08	1E+08	297	1E+08	13	4.78908	3.14145
Nc-1-IP_pe	chr10	1E+08	1E+08	280	1E+08	13	7.12853	4.42991
Nc-1-IP_pe	chr10	1.01E+08	1.01E+08	283	1.01E+08	10	4.33501	3.21677
Nc-1-IP_pe	chr10	1.01E+08	1.01E+08	411	1.01E+08	11	5.10577	3.5486
Nc-1-IP_pe	chr10	1.01E+08	1.01E+08	268	1.01E+08	14	7.54286	4.50023
Nc-1-IP_pe	chr10	1.01E+08	1.01E+08	601	1.01E+08	11	5.77463	3.94557
Nc-1-IP_pe	chr10	1.01E+08	1.01E+08	628	1.01E+08	10	4.75926	3.47115
Nc-1-IP_pe	chr10	1.01E+08	1.01E+08	334	1.01E+08	12	5.92876	3.89011
Nc-1-IP_pe	chr10	1.01E+08	1.01E+08	1335	1.01E+08	18	7.73136	4.00629
Nc-1-IP_pe	chr10	1.01E+08	1.01E+08	436	1.01E+08	10	3.83201	2.92147
Nc-1-IP_pe	chr10	1.01E+08	1.01E+08	396	1.01E+08	9	4.25966	3.28797
Nc-1-IP_pe	chr10	1.01E+08	1.01E+08	287	1.01E+08	15	8.16556	4.67894
Nc-1-IP_pe	chr10	1.01E+08	1.01E+08	282	1.01E+08	10	4.99888	3.61677
Nc-1-IP_pe	chr10	1.01E+08	1.01E+08	600	1.01E+08	10	4.56202	3.35231
Nc-1-IP_pe	chr10	1.01E+08	1.01E+08	349	1.01E+08	11	5.37297	3.70587
Nc-1-IP_pe	chr10	1.01E+08	1.01E+08	484	1.01E+08	15	7.95672	4.56362
Nc-1-IP_pe	chr10	1.01E+08	1.01E+08	507	1.01E+08	11	5.19376	3.60018
Nc-1-IP_pe	chr10	1.01E+08	1.01E+08	281	1.01E+08	12	4.70082	3.20033
Nc-1-IP_pe	chr10	1.01E+08	1.01E+08	374	1.01E+08	18	4.6358	2.66698
Nc-1-IP_pe	chr10	1.01E+08	1.01E+08	647	1.01E+08	10	4.95265	3.58858
Nc-1-IP_pe	chr10	1.01E+08	1.01E+08	450	1.01E+08	13	6.72991	4.20021
Nc-1-IP_pe	chr10	1.01E+08	1.01E+08	593	1.01E+08	15	8.79961	5.03521
Nc-1-IP_pe	chr10	1.01E+08	1.01E+08	422	1.01E+08	11	4.06403	2.95415
Nc-1-IP_pe	chr10	1.01E+08	1.01E+08	1248	1.01E+08	9	3.79246	3.00015
Nc-1-IP_pe	chr10	1.01E+08	1.01E+08	454	1.01E+08	11	4.54404	3.2242
Nc-1-IP_pe	chr10	1.01E+08	1.01E+08	314	1.01E+08	8	3.51085	2.92785
Nc-1-IP_pe	chr10	1.01E+08	1.01E+08	296	1.01E+08	12	6.50787	4.22912
Nc-1-IP_pe	chr10	1.01E+08	1.01E+08	292	1.01E+08	10	4.80655	3.49978
Nc-1-IP_pe	chr10	1.01E+08	1.01E+08	392	1.01E+08	11	5.10577	3.5486
Nc-1-IP_pe	chr10	1.01E+08	1.01E+08	536	1.01E+08	10	4.39571	3.25288
Nc-1-IP_pe	chr10	1.01E+08	1.01E+08	645	1.01E+08	14	8.63073	5.12814
Nc-1-IP_pe	chr10	1.01E+08	1.01E+08	373	1.01E+08	12	6.58417	4.27436

Nc-1-IP_pe	chr10	1.01E+08	1.01E+08	542	1.01E+08	10	4.62142	3.388
Nc-1-IP_pe	chr10	1.01E+08	1.01E+08	271	1.01E+08	11	5.10577	3.5486
Nc-1-IP_pe	chr10	1.01E+08	1.01E+08	290	1.01E+08	8	3.14944	2.70014
Nc-1-IP_pe	chr10	1.01E+08	1.01E+08	273	1.01E+08	11	5.10577	3.5486
Nc-1-IP_pe	chr10	1.01E+08	1.01E+08	256	1.01E+08	11	4.11366	2.98176
Nc-1-IP_pe	chr10	1.01E+08	1.01E+08	670	1.01E+08	13	6.92473	4.312
Nc-1-IP_pe	chr10	1.01E+08	1.01E+08	276	1.01E+08	13	6.62436	4.14003
Nc-1-IP_pe	chr10	1.01E+08	1.01E+08	291	1.01E+08	10	4.07292	3.06202
Nc-1-IP_pe	chr10	1.01E+08	1.01E+08	358	1.01E+08	11	6.03439	4.10251
Nc-1-IP_pe	chr10	1.01E+08	1.01E+08	316	1.01E+08	15	6.5434	3.81197
Nc-1-IP_pe	chr10	1.01E+08	1.01E+08	388	1.01E+08	12	5.23082	3.49289
Nc-1-IP_pe	chr10	1.01E+08	1.01E+08	336	1.01E+08	11	6.03439	4.10251
Nc-1-IP_pe	chr10	1.01E+08	1.01E+08	291	1.01E+08	11	2.96397	2.36091
Nc-1-IP_pe	chr10	1.01E+08	1.01E+08	619	1.01E+08	11	5.89269	4.01672
Nc-1-IP_pe	chr10	1.01E+08	1.01E+08	284	1.01E+08	12	6.03452	3.95142
Nc-1-IP_pe	chr10	1.01E+08	1.01E+08	611	1.01E+08	15	8.77559	5.02156
Nc-1-IP_pe	chr10	1.01E+08	1.01E+08	425	1.01E+08	9	4.21839	3.26234
Nc-1-IP_pe	chr10	1.01E+08	1.01E+08	455	1.01E+08	10	3.08907	2.49869
Nc-1-IP_pe	chr10	1.01E+08	1.01E+08	637	1.01E+08	13	6.52521	4.08375
Nc-1-IP_pe	chr10	1.01E+08	1.01E+08	276	1.01E+08	20	7.97282	3.87239
Nc-1-IP_pe	chr10	1.01E+08	1.01E+08	395	1.01E+08	13	5.9487	3.76157
Nc-1-IP_pe	chr10	1.01E+08	1.01E+08	444	1.01E+08	12	5.94619	3.9002
Nc-1-IP_pe	chr10	1.01E+08	1.01E+08	336	1.01E+08	10	3.94999	2.99009
Nc-1-IP_pe	chr10	1.01E+08	1.01E+08	411	1.01E+08	12	5.42384	3.60142
Nc-1-IP_pe	chr10	1.01E+08	1.01E+08	267	1.01E+08	11	4.06403	2.95415
Nc-1-IP_pe	chr10	1.01E+08	1.01E+08	403	1.01E+08	16	5.62031	3.24783
Nc-1-IP_pe	chr10	1.01E+08	1.01E+08	437	1.01E+08	11	4.61014	3.26192
Nc-1-IP_pe	chr10	1.01E+08	1.01E+08	384	1.01E+08	8	3.08755	2.66145
Nc-1-IP_pe	chr10	1.01E+08	1.01E+08	283	1.01E+08	10	4.39571	3.25288
Nc-1-IP_pe	chr10	1.01E+08	1.01E+08	290	1.01E+08	12	4.70082	3.20033
Nc-1-IP_pe	chr10	1.01E+08	1.01E+08	313	1.01E+08	13	7.7352	4.78626
Nc-1-IP_pe	chr10	1.01E+08	1.01E+08	427	1.01E+08	9	4.46937	3.41876
Nc-1-IP_pe	chr10	1.01E+08	1.01E+08	337	1.01E+08	12	6.14317	4.01469
Nc-1-IP_pe	chr10	1.01E+08	1.01E+08	857	1.01E+08	19	7.29128	3.68799
Nc-1-IP_pe	chr10	1.01E+08	1.01E+08	359	1.01E+08	12	5.15877	3.45264
Nc-1-IP_pe	chr10	1.01E+08	1.01E+08	394	1.01E+08	12	4.70082	3.20033
Nc-1-IP_pe	chr10	1.01E+08	1.01E+08	310	1.01E+08	12	4.91154	3.31567
Nc-1-IP_pe	chr10	1.01E+08	1.01E+08	378	1.01E+08	15	4.7819	2.95039
Nc-1-IP_pe	chr10	1.01E+08	1.01E+08	626	1.01E+08	24	11.6162	4.91857
Nc-1-IP_pe	chr10	1.01E+08	1.01E+08	419	1.01E+08	10	3.55676	2.76294
Nc-1-IP_pe	chr10	1.01E+08	1.01E+08	279	1.01E+08	11	4.26676	3.06738
Nc-1-IP_pe	chr10	1.01E+08	1.01E+08	297	1.01E+08	13	5.59935	3.57072
Nc-1-IP_pe	chr10	1.01E+08	1.01E+08	254	1.01E+08	10	4.12355	3.09176
Nc-1-IP_pe	chr10	1.01E+08	1.01E+08	697	1.01E+08	13	4.45253	2.96876
Nc-1-IP_pe	chr10	1.01E+08	1.01E+08	757	1.01E+08	14	6.13058	3.7272

Nc-1-IP_pe	chr10	1.01E+08	1.01E+08	688	1.01E+08	15	6.19428	3.63445
Nc-1-IP_pe	chr10	1.01E+08	1.01E+08	457	1.01E+08	10	3.4604	2.70797
Nc-1-IP_pe	chr10	1.01E+08	1.01E+08	294	1.01E+08	12	3.17548	2.40425
Nc-1-IP_pe	chr10	1.01E+08	1.01E+08	312	1.01E+08	10	3.4604	2.70797
Nc-1-IP_pe	chr10	1.01E+08	1.01E+08	261	1.01E+08	14	6.06519	3.69269
Nc-1-IP_pe	chr10	1.01E+08	1.01E+08	421	1.01E+08	16	7.53857	4.18505
Nc-1-IP_pe	chr10	1.01E+08	1.01E+08	282	1.01E+08	17	5.9911	3.31919
Nc-1-IP_pe	chr10	1.01E+08	1.01E+08	441	1.01E+08	15	4.06016	2.62146
Nc-1-IP_pe	chr10	1.01E+08	1.01E+08	612	1.01E+08	14	6.28469	3.80898
Nc-1-IP_pe	chr10	1.01E+08	1.01E+08	426	1.01E+08	13	4.41954	2.952
Nc-1-IP_pe	chr10	1.01E+08	1.01E+08	284	1.01E+08	16	9.6698	5.33541
Nc-1-IP_pe	chr10	1.01E+08	1.01E+08	311	1.01E+08	11	5.89269	4.01672
Nc-1-IP_pe	chr10	1.01E+08	1.01E+08	398	1.01E+08	10	4.59158	3.37006
Nc-1-IP_pe	chr10	1.01E+08	1.01E+08	355	1.01E+08	13	5.36853	3.44651
Nc-1-IP_pe	chr10	1.01E+08	1.01E+08	417	1.01E+08	12	3.77112	2.7071
Nc-1-IP_pe	chr10	1.01E+08	1.01E+08	434	1.01E+08	13	5.61391	3.57861
Nc-1-IP_pe	chr10	1.01E+08	1.01E+08	297	1.01E+08	11	4.47871	3.18705
Nc-1-IP_pe	chr10	1.01E+08	1.01E+08	272	1.01E+08	9	3.54889	2.85226
Nc-1-IP_pe	chr10	1.01E+08	1.01E+08	398	1.01E+08	10	4.33501	3.21677
Nc-1-IP_pe	chr10	1.01E+08	1.01E+08	788	1.01E+08	12	5.84275	3.84046
Nc-1-IP_pe	chr10	1.01E+08	1.01E+08	305	1.01E+08	11	3.78682	2.80131
Nc-1-IP_pe	chr10	1.01E+08	1.01E+08	253	1.01E+08	11	3.64839	2.72584
Nc-1-IP_pe	chr10	1.01E+08	1.01E+08	1075	1.01E+08	17	9.99923	5.32289
Nc-1-IP_pe	chr10	1.01E+08	1.01E+08	294	1.01E+08	10	3.60969	2.79325
Nc-1-IP_pe	chr10	1.01E+08	1.01E+08	316	1.01E+08	14	6.13058	3.7272
Nc-1-IP_pe	chr10	1.01E+08	1.01E+08	367	1.01E+08	9	3.66792	2.92434
Nc-1-IP_pe	chr10	1.01E+08	1.01E+08	496	1.01E+08	10	4.99888	3.61677
Nc-1-IP_pe	chr10	1.01E+08	1.01E+08	315	1.01E+08	16	8.37012	4.62105
Nc-1-IP_pe	chr10	1.01E+08	1.01E+08	262	1.01E+08	9	3.75449	2.977
Nc-1-IP_pe	chr10	1.01E+08	1.01E+08	381	1.01E+08	14	7.42849	4.43574
Nc-1-IP_pe	chr10	1.01E+08	1.01E+08	672	1.01E+08	10	4.96929	3.59872
Nc-1-IP_pe	chr10	1.01E+08	1.01E+08	584	1.01E+08	12	3.83688	2.74114
Nc-1-IP_pe	chr10	1.01E+08	1.01E+08	709	1.01E+08	10	4.48929	3.30875
Nc-1-IP_pe	chr10	1.01E+08	1.01E+08	351	1.01E+08	12	5.23082	3.49289
Nc-1-IP_pe	chr10	1.01E+08	1.01E+08	565	1.01E+08	10	4.44645	3.28314
Nc-1-IP_pe	chr10	1.01E+08	1.01E+08	426	1.01E+08	15	8.16556	4.67894
Nc-1-IP_pe	chr10	1.01E+08	1.01E+08	318	1.01E+08	11	4.93354	3.44821
Nc-1-IP_pe	chr10	1.01E+08	1.01E+08	280	1.01E+08	13	3.67377	2.58159
Nc-1-IP_pe	chr10	1.01E+08	1.01E+08	1450	1.01E+08	16	6.8987	3.86161
Nc-1-IP_pe	chr10	1.01E+08	1.01E+08	520	1.01E+08	11	3.64839	2.72584
Nc-1-IP_pe	chr10	1.01E+08	1.01E+08	647	1.01E+08	12	4.70082	3.20033
Nc-1-IP_pe	chr10	1.01E+08	1.01E+08	502	1.01E+08	14	6.06519	3.69269
Nc-1-IP_pe	chr10	1.01E+08	1.01E+08	345	1.01E+08	12	4.24064	2.95299
Nc-1-IP_pe	chr10	1.01E+08	1.01E+08	327	1.01E+08	14	6.06519	3.69269
Nc-1-IP_pe	chr10	1.01E+08	1.01E+08	269	1.01E+08	14	6.69566	4.03025

Nc-1-IP_pe	chr10	1.01E+08	1.01E+08	410	1.01E+08	14	6.57647	3.96562
Nc-1-IP_pe	chr10	1.01E+08	1.01E+08	426	1.01E+08	12	5.01934	3.37518
Nc-1-IP_pe	chr10	1.01E+08	1.01E+08	254	1.01E+08	12	5.70894	3.7636
Nc-1-IP_pe	chr10	1.01E+08	1.01E+08	297	1.01E+08	11	5.49185	3.77641
Nc-1-IP_pe	chr10	1.01E+08	1.01E+08	348	1.01E+08	10	4.33501	3.21677
Nc-1-IP_pe	chr10	1.01E+08	1.01E+08	556	1.01E+08	11	5.76039	3.93701
Nc-1-IP_pe	chr10	1.01E+08	1.01E+08	315	1.01E+08	12	5.77527	3.80164
Nc-1-IP_pe	chr10	1.01E+08	1.01E+08	333	1.01E+08	9	3.27326	2.68684
Nc-1-IP_pe	chr10	1.01E+08	1.01E+08	487	1.01E+08	12	5.15877	3.45264
Nc-1-IP_pe	chr10	1.01E+08	1.01E+08	345	1.01E+08	12	6.86906	4.44438
Nc-1-IP_pe	chr10	1.01E+08	1.01E+08	557	1.01E+08	10	5.23357	3.76063
Nc-1-IP_pe	chr10	1.01E+08	1.01E+08	273	1.01E+08	11	6.03439	4.10251
Nc-1-IP_pe	chr10	1.01E+08	1.01E+08	378	1.01E+08	9	3.66792	2.92434
Nc-1-IP_pe	chr10	1.02E+08	1.02E+08	433	1.02E+08	9	3.79246	3.00015
Nc-1-IP_pe	chr10	1.02E+08	1.02E+08	260	1.02E+08	12	6.86906	4.44438
Nc-1-IP_pe	chr10	1.02E+08	1.02E+08	523	1.02E+08	16	4.25252	2.63824
Nc-1-IP_pe	chr10	1.02E+08	1.02E+08	358	1.02E+08	12	6.18005	4.03623
Nc-1-IP_pe	chr10	1.02E+08	1.02E+08	298	1.02E+08	11	4.45305	3.17249
Nc-1-IP_pe	chr10	1.02E+08	1.02E+08	527	1.02E+08	10	3.8904	2.95538
Nc-1-IP_pe	chr10	1.02E+08	1.02E+08	260	1.02E+08	11	5.3563	3.696
Nc-1-IP_pe	chr10	1.02E+08	1.02E+08	385	1.02E+08	14	6.96097	4.17548
Nc-1-IP_pe	chr10	1.02E+08	1.02E+08	259	1.02E+08	10	4.39571	3.25288
Nc-1-IP_pe	chr10	1.02E+08	1.02E+08	299	1.02E+08	12	6.12485	4.004
Nc-1-IP_pe	chr10	1.02E+08	1.02E+08	375	1.02E+08	9	2.89249	2.46179
Nc-1-IP_pe	chr10	1.02E+08	1.02E+08	337	1.02E+08	12	4.70082	3.20033
Nc-1-IP_pe	chr10	1.02E+08	1.02E+08	512	1.02E+08	20	7.43645	3.64378
Nc-1-IP_pe	chr10	1.02E+08	1.02E+08	280	1.02E+08	19	5.51632	2.94814
Nc-1-IP_pe	chr10	1.02E+08	1.02E+08	263	1.02E+08	13	6.54342	4.09407
Nc-1-IP_pe	chr10	1.02E+08	1.02E+08	524	1.02E+08	8	3.74505	3.07688
Nc-1-IP_pe	chr10	1.02E+08	1.02E+08	279	1.02E+08	12	4.24064	2.95299
Nc-1-IP_pe	chr10	1.02E+08	1.02E+08	265	1.02E+08	11	6.03439	4.10251
Nc-1-IP_pe	chr10	1.02E+08	1.02E+08	486	1.02E+08	14	3.86727	2.6027
Nc-1-IP_pe	chr10	1.02E+08	1.02E+08	356	1.02E+08	10	3.71867	2.85592
Nc-1-IP_pe	chr10	1.02E+08	1.02E+08	309	1.02E+08	12	4.88508	3.30112
Nc-1-IP_pe	chr10	1.02E+08	1.02E+08	522	1.02E+08	19	9.81043	4.86513
Nc-1-IP_pe	chr10	1.02E+08	1.02E+08	370	1.02E+08	14	7.09041	4.24699
Nc-1-IP_pe	chr10	1.02E+08	1.02E+08	347	1.02E+08	13	6.21	3.90651
Nc-1-IP_pe	chr10	1.02E+08	1.02E+08	518	1.02E+08	14	5.838	3.57373
Nc-1-IP_pe	chr10	1.02E+08	1.02E+08	626	1.02E+08	14	6.39588	3.8684
Nc-1-IP_pe	chr10	1.02E+08	1.02E+08	273	1.02E+08	19	9.92774	4.92358
Nc-1-IP_pe	chr10	1.02E+08	1.02E+08	479	1.02E+08	8	3.56021	2.95918
Nc-1-IP_pe	chr10	1.02E+08	1.02E+08	258	1.02E+08	10	5.23357	3.76063
Nc-1-IP_pe	chr10	1.02E+08	1.02E+08	723	1.02E+08	16	6.37501	3.60492
Nc-1-IP_pe	chr10	1.02E+08	1.02E+08	675	1.02E+08	13	6.62436	4.14003
Nc-1-IP_pe	chr10	1.02E+08	1.02E+08	254	1.02E+08	10	4.83846	3.51913

Nc-1-IP_pe	chr10	1.02E+08	1.02E+08	940	1.02E+08	12	4.75569	3.23024
Nc-1-IP_pe	chr10	1.02E+08	1.02E+08	436	1.02E+08	14	5.03085	3.16286
Nc-1-IP_pe	chr10	1.02E+08	1.02E+08	280	1.02E+08	9	3.27326	2.68684
Nc-1-IP_pe	chr10	1.02E+08	1.02E+08	269	1.02E+08	8	3.51085	2.92785
Nc-1-IP_pe	chr10	1.02E+08	1.02E+08	632	1.02E+08	8	3.74505	3.07688
Nc-1-IP_pe	chr10	1.02E+08	1.02E+08	260	1.02E+08	11	6.03439	4.10251
Nc-1-IP_pe	chr10	1.02E+08	1.02E+08	263	1.02E+08	10	5.23357	3.76063
Nc-1-IP_pe	chr10	1.02E+08	1.02E+08	253	1.02E+08	10	4.22744	3.15303
Nc-1-IP_pe	chr10	1.02E+08	1.02E+08	386	1.02E+08	15	4.90347	3.00714
Nc-1-IP_pe	chr10	1.02E+08	1.02E+08	453	1.02E+08	15	4.7819	2.95039
Nc-1-IP_pe	chr10	1.02E+08	1.02E+08	309	1.02E+08	9	3.03392	2.54491
Nc-1-IP_pe	chr10	1.02E+08	1.02E+08	266	1.02E+08	12	6.16157	4.02543
Nc-1-IP_pe	chr10	1.02E+08	1.02E+08	473	1.02E+08	11	5.76039	3.93701
Nc-1-IP_pe	chr10	1.02E+08	1.02E+08	284	1.02E+08	9	3.72192	2.95716
Nc-1-IP_pe	chr10	1.02E+08	1.02E+08	297	1.02E+08	11	5.06889	3.52703
Nc-1-IP_pe	chr10	1.02E+08	1.02E+08	290	1.02E+08	11	2.96397	2.36091
Nc-1-IP_pe	chr10	1.02E+08	1.02E+08	602	1.02E+08	13	5.36853	3.44651
Nc-1-IP_pe	chr10	1.02E+08	1.02E+08	582	1.02E+08	14	6.78215	4.0774
Nc-1-IP_pe	chr10	1.02E+08	1.02E+08	346	1.02E+08	10	3.03453	2.46828
Nc-1-IP_pe	chr10	1.02E+08	1.02E+08	601	1.02E+08	14	4.52309	2.91384
Nc-1-IP_pe	chr10	1.02E+08	1.02E+08	295	1.02E+08	9	3.28353	2.69296
Nc-1-IP_pe	chr10	1.02E+08	1.02E+08	892	1.02E+08	10	4.29423	3.19257
Nc-1-IP_pe	chr10	1.02E+08	1.02E+08	385	1.02E+08	13	4.90859	3.20357
Nc-1-IP_pe	chr10	1.02E+08	1.02E+08	511	1.02E+08	16	3.94146	2.50592
Nc-1-IP_pe	chr10	1.02E+08	1.02E+08	512	1.02E+08	11	4.11366	2.98176
Nc-1-IP_pe	chr10	1.02E+08	1.02E+08	354	1.02E+08	11	2.96397	2.36091
Nc-1-IP_pe	chr10	1.02E+08	1.02E+08	263	1.02E+08	10	4.95265	3.58858
Nc-1-IP_pe	chr10	1.02E+08	1.02E+08	344	1.02E+08	10	4.82246	3.50943
Nc-1-IP_pe	chr10	1.02E+08	1.02E+08	687	1.02E+08	17	8.31233	4.43123
Nc-1-IP_pe	chr10	1.02E+08	1.02E+08	617	1.02E+08	9	4.46937	3.41876
Nc-1-IP_pe	chr10	1.02E+08	1.02E+08	420	1.02E+08	12	5.23082	3.49289
Nc-1-IP_pe	chr10	1.02E+08	1.02E+08	306	1.02E+08	14	5.03085	3.16286
Nc-1-IP_pe	chr10	1.02E+08	1.02E+08	1435	1.02E+08	18	5.33158	2.94846
Nc-1-IP_pe	chr10	1.02E+08	1.02E+08	320	1.02E+08	14	4.21449	2.76599
Nc-1-IP_pe	chr10	1.02E+08	1.02E+08	344	1.02E+08	13	5.03208	3.26819
Nc-1-IP_pe	chr10	1.02E+08	1.02E+08	338	1.02E+08	17	6.92131	3.74829
Nc-1-IP_pe	chr10	1.02E+08	1.02E+08	338	1.02E+08	13	7.7352	4.78626
Nc-1-IP_pe	chr10	1.02E+08	1.02E+08	300	1.02E+08	9	4.46937	3.41876
Nc-1-IP_pe	chr10	1.02E+08	1.02E+08	325	1.02E+08	11	5.10577	3.5486
Nc-1-IP_pe	chr10	1.02E+08	1.02E+08	290	1.02E+08	17	8.31233	4.43123
Nc-1-IP_pe	chr10	1.02E+08	1.02E+08	790	1.02E+08	12	6.86906	4.44438
Nc-1-IP_pe	chr10	1.02E+08	1.02E+08	366	1.02E+08	8	3.02956	2.62527
Nc-1-IP_pe	chr10	1.02E+08	1.02E+08	349	1.02E+08	9	3.22129	2.65588
Nc-1-IP_pe	chr10	1.02E+08	1.02E+08	525	1.02E+08	11	4.06403	2.95415
Nc-1-IP_pe	chr10	1.02E+08	1.02E+08	318	1.02E+08	11	4.06403	2.95415

Nc-1-IP_pe	chr10	1.02E+08	1.02E+08	812	1.02E+08	16	5.73763	3.30235
Nc-1-IP_pe	chr10	1.02E+08	1.02E+08	279	1.02E+08	10	3.19423	2.55755
Nc-1-IP_pe	chr10	1.02E+08	1.02E+08	836	1.02E+08	17	7.1249	3.84516
Nc-1-IP_pe	chr10	1.02E+08	1.02E+08	386	1.02E+08	13	4.81267	3.15368
Nc-1-IP_pe	chr10	1.02E+08	1.02E+08	633	1.02E+08	13	5.13388	3.3218
Nc-1-IP_pe	chr10	1.02E+08	1.02E+08	369	1.02E+08	11	4.04544	2.94383
Nc-1-IP_pe	chr10	1.02E+08	1.02E+08	564	1.02E+08	16	7.53857	4.18505
Nc-1-IP_pe	chr10	1.02E+08	1.02E+08	371	1.02E+08	13	5.36853	3.44651
Nc-1-IP_pe	chr10	1.02E+08	1.02E+08	567	1.02E+08	13	5.93256	3.75268
Nc-1-IP_pe	chr10	1.02E+08	1.02E+08	396	1.02E+08	18	7.47506	3.88659
Nc-1-IP_pe	chr10	1.02E+08	1.02E+08	477	1.02E+08	24	8.53898	3.68518
Nc-1-IP_pe	chr10	1.02E+08	1.02E+08	474	1.02E+08	15	5.22925	3.16116
Nc-1-IP_pe	chr10	1.02E+08	1.02E+08	403	1.02E+08	15	6.74657	3.91681
Nc-1-IP_pe	chr10	1.02E+08	1.02E+08	571	1.02E+08	15	6.5434	3.81197
Nc-1-IP_pe	chr10	1.02E+08	1.02E+08	412	1.02E+08	10	3.4604	2.70797
Nc-1-IP_pe	chr10	1.02E+08	1.02E+08	539	1.02E+08	15	6.85973	3.97568
Nc-1-IP_pe	chr10	1.02E+08	1.02E+08	412	1.02E+08	13	5.36853	3.44651
Nc-1-IP_pe	chr10	1.02E+08	1.02E+08	515	1.02E+08	11	4.00079	2.91908
Nc-1-IP_pe	chr10	1.02E+08	1.02E+08	370	1.02E+08	13	4.34711	2.91533
Nc-1-IP_pe	chr10	1.02E+08	1.02E+08	316	1.02E+08	13	5.99619	3.78778
Nc-1-IP_pe	chr10	1.02E+08	1.02E+08	570	1.02E+08	12	3.47963	2.55766
Nc-1-IP_pe	chr10	1.02E+08	1.02E+08	777	1.02E+08	16	6.65665	3.74206
Nc-1-IP_pe	chr10	1.02E+08	1.02E+08	272	1.02E+08	14	5.98179	3.64885
Nc-1-IP_pe	chr10	1.02E+08	1.02E+08	326	1.02E+08	11	4.70533	3.31645
Nc-1-IP_pe	chr10	1.03E+08	1.03E+08	325	1.03E+08	13	4.41954	2.952
Nc-1-IP_pe	chr10	1.03E+08	1.03E+08	696	1.03E+08	11	4.54404	3.2242
Nc-1-IP_pe	chr10	1.03E+08	1.03E+08	421	1.03E+08	11	5.25789	3.63791
Nc-1-IP_pe	chr10	1.03E+08	1.03E+08	352	1.03E+08	11	4.54404	3.2242
Nc-1-IP_pe	chr10	1.03E+08	1.03E+08	670	1.03E+08	8	3.29947	2.79431
Nc-1-IP_pe	chr10	1.03E+08	1.03E+08	289	1.03E+08	8	3.56332	2.96115
Nc-1-IP_pe	chr10	1.03E+08	1.03E+08	337	1.03E+08	15	3.74763	2.48304
Nc-1-IP_pe	chr10	1.03E+08	1.03E+08	362	1.03E+08	10	4.32135	3.20866
Nc-1-IP_pe	chr10	1.03E+08	1.03E+08	586	1.03E+08	15	6.77862	3.93345
Nc-1-IP_pe	chr10	1.03E+08	1.03E+08	347	1.03E+08	15	6.19428	3.63445
Nc-1-IP_pe	chr10	1.03E+08	1.03E+08	1109	1.03E+08	19	7.84735	3.93486
Nc-1-IP_pe	chr10	1.03E+08	1.03E+08	281	1.03E+08	15	3.46204	2.35851
Nc-1-IP_pe	chr10	1.03E+08	1.03E+08	263	1.03E+08	12	4.47508	3.07822
Nc-1-IP_pe	chr10	1.03E+08	1.03E+08	436	1.03E+08	26	15.1007	6.13314
Nc-1-IP_pe	chr10	1.03E+08	1.03E+08	560	1.03E+08	12	4.24064	2.95299
Nc-1-IP_pe	chr10	1.03E+08	1.03E+08	307	1.03E+08	13	5.47064	3.50127
Nc-1-IP_pe	chr10	1.03E+08	1.03E+08	390	1.03E+08	15	3.74763	2.48304
Nc-1-IP_pe	chr10	1.03E+08	1.03E+08	331	1.03E+08	14	6.34788	3.84271
Nc-1-IP_pe	chr10	1.03E+08	1.03E+08	1143	1.03E+08	14	7.16603	4.28896
Nc-1-IP_pe	chr10	1.03E+08	1.03E+08	699	1.03E+08	15	7.56326	4.34922
Nc-1-IP_pe	chr10	1.03E+08	1.03E+08	546	1.03E+08	14	7.52226	4.48859

Nc-1-IP_pe	chr10	1.03E+08	1.03E+08	1129	1.03E+08	13	5.36853	3.44651
Nc-1-IP_pe	chr10	1.03E+08	1.03E+08	533	1.03E+08	12	4.24064	2.95299
Nc-1-IP_pe	chr10	1.03E+08	1.03E+08	385	1.03E+08	10	3.50482	2.73328
Nc-1-IP_pe	chr10	1.03E+08	1.03E+08	485	1.03E+08	10	4.74365	3.46171
Nc-1-IP_pe	chr10	1.03E+08	1.03E+08	380	1.03E+08	9	4.46937	3.41876
Nc-1-IP_pe	chr10	1.03E+08	1.03E+08	265	1.03E+08	21	7.10961	3.41419
Nc-1-IP_pe	chr10	1.03E+08	1.03E+08	304	1.03E+08	12	6.86906	4.44438
Nc-1-IP_pe	chr10	1.03E+08	1.03E+08	743	1.03E+08	9	4.46937	3.41876
Nc-1-IP_pe	chr10	1.03E+08	1.03E+08	318	1.03E+08	11	6.03439	4.10251
Nc-1-IP_pe	chr10	1.03E+08	1.03E+08	380	1.03E+08	10	4.99888	3.61677
Nc-1-IP_pe	chr10	1.03E+08	1.03E+08	262	1.03E+08	13	4.14549	2.81406
Nc-1-IP_pe	chr10	1.03E+08	1.03E+08	369	1.03E+08	9	3.77975	2.99239
Nc-1-IP_pe	chr10	1.03E+08	1.03E+08	334	1.03E+08	15	7.75613	4.45384
Nc-1-IP_pe	chr10	1.03E+08	1.03E+08	454	1.03E+08	13	7.70601	4.76894
Nc-1-IP_pe	chr10	1.03E+08	1.03E+08	668	1.03E+08	12	6.58417	4.27436
Nc-1-IP_pe	chr10	1.03E+08	1.03E+08	385	1.03E+08	10	5.14046	3.70342
Nc-1-IP_pe	chr10	1.03E+08	1.03E+08	272	1.03E+08	10	3.83201	2.92147
Nc-1-IP_pe	chr10	1.03E+08	1.03E+08	396	1.03E+08	11	5.25789	3.63791
Nc-1-IP_pe	chr10	1.03E+08	1.03E+08	584	1.03E+08	8	3.64414	3.01254
Nc-1-IP_pe	chr10	1.03E+08	1.03E+08	661	1.03E+08	11	3.64839	2.72584
Nc-1-IP_pe	chr10	1.03E+08	1.03E+08	656	1.03E+08	10	4.39571	3.25288
Nc-1-IP_pe	chr10	1.03E+08	1.03E+08	296	1.03E+08	11	5.97044	4.06374
Nc-1-IP_pe	chr10	1.03E+08	1.03E+08	330	1.03E+08	10	5.23357	3.76063
Nc-1-IP_pe	chr10	1.03E+08	1.03E+08	267	1.03E+08	11	5.10577	3.5486
Nc-1-IP_pe	chr10	1.03E+08	1.03E+08	271	1.03E+08	14	6.06519	3.69269
Nc-1-IP_pe	chr10	1.03E+08	1.03E+08	422	1.03E+08	11	4.8033	3.37283
Nc-1-IP_pe	chr10	1.03E+08	1.03E+08	289	1.03E+08	15	5.20142	3.14789
Nc-1-IP_pe	chr10	1.03E+08	1.03E+08	531	1.03E+08	13	7.29853	4.52898
Nc-1-IP_pe	chr10	1.03E+08	1.03E+08	337	1.03E+08	14	8.63073	5.12814
Nc-1-IP_pe	chr10	1.03E+08	1.03E+08	517	1.03E+08	10	4.93609	3.57849
Nc-1-IP_pe	chr10	1.03E+08	1.03E+08	303	1.03E+08	10	5.23357	3.76063
Nc-1-IP_pe	chr10	1.03E+08	1.03E+08	347	1.03E+08	11	6.03439	4.10251
Nc-1-IP_pe	chr10	1.03E+08	1.03E+08	255	1.03E+08	18	5.62894	3.07204
Nc-1-IP_pe	chr10	1.03E+08	1.03E+08	271	1.03E+08	8	3.74505	3.07688
Nc-1-IP_pe	chr10	1.03E+08	1.03E+08	454	1.03E+08	9	3.27326	2.68684
Nc-1-IP_pe	chr10	1.03E+08	1.03E+08	377	1.03E+08	12	6.71369	4.35145
Nc-1-IP_pe	chr10	1.03E+08	1.03E+08	280	1.03E+08	9	4.46937	3.41876
Nc-1-IP_pe	chr10	1.03E+08	1.03E+08	307	1.03E+08	10	4.24068	3.16086
Nc-1-IP_pe	chr10	1.03E+08	1.03E+08	481	1.03E+08	15	6.69889	3.8921
Nc-1-IP_pe	chr10	1.03E+08	1.03E+08	498	1.03E+08	12	6.58417	4.27436
Nc-1-IP_pe	chr10	1.03E+08	1.03E+08	283	1.03E+08	13	7.34205	4.55444
Nc-1-IP_pe	chr10	1.03E+08	1.03E+08	500	1.03E+08	14	6.69566	4.03025
Nc-1-IP_pe	chr10	1.03E+08	1.03E+08	355	1.03E+08	10	4.80655	3.49978
Nc-1-IP_pe	chr10	1.03E+08	1.03E+08	729	1.03E+08	13	7.42513	4.60316
Nc-1-IP_pe	chr10	1.03E+08	1.03E+08	518	1.03E+08	14	5.69896	3.50164

Nc-1-IP_pe	chr10	1.03E+08	1.03E+08	309	1.03E+08	7	2.97697	2.67781
Nc-1-IP_pe	chr10	1.03E+08	1.03E+08	274	1.03E+08	10	4.53273	3.33475
Nc-1-IP_pe	chr10	1.03E+08	1.03E+08	785	1.03E+08	16	7.44163	4.13536
Nc-1-IP_pe	chr10	1.03E+08	1.03E+08	428	1.03E+08	10	4.44645	3.28314
Nc-1-IP_pe	chr10	1.03E+08	1.03E+08	511	1.03E+08	10	4.99888	3.61677
Nc-1-IP_pe	chr10	1.03E+08	1.03E+08	341	1.03E+08	13	6.47103	4.0531
Nc-1-IP_pe	chr10	1.03E+08	1.03E+08	304	1.03E+08	10	4.33501	3.21677
Nc-1-IP_pe	chr10	1.03E+08	1.03E+08	279	1.03E+08	30	12.0847	4.35139
Nc-1-IP_pe	chr10	1.03E+08	1.03E+08	850	1.03E+08	10	5.23357	3.76063
Nc-1-IP_pe	chr10	1.03E+08	1.03E+08	253	1.03E+08	11	3.64839	2.72584
Nc-1-IP_pe	chr10	1.03E+08	1.03E+08	319	1.03E+08	9	4.088	3.18162
Nc-1-IP_pe	chr10	1.03E+08	1.03E+08	490	1.03E+08	8	3.69948	3.0478
Nc-1-IP_pe	chr10	1.03E+08	1.03E+08	269	1.03E+08	8	3.08755	2.66145
Nc-1-IP_pe	chr10	1.03E+08	1.03E+08	331	1.03E+08	13	7.42513	4.60316
Nc-1-IP_pe	chr10	1.03E+08	1.03E+08	314	1.03E+08	14	6.69566	4.03025
Nc-1-IP_pe	chr10	1.03E+08	1.03E+08	504	1.03E+08	10	5.23357	3.76063
Nc-1-IP_pe	chr10	1.03E+08	1.03E+08	359	1.03E+08	9	4.46937	3.41876
Nc-1-IP_pe	chr10	1.03E+08	1.03E+08	436	1.03E+08	12	6.86906	4.44438
Nc-1-IP_pe	chr10	1.03E+08	1.03E+08	563	1.03E+08	10	4.39571	3.25288
Nc-1-IP_pe	chr10	1.03E+08	1.03E+08	273	1.03E+08	10	4.99888	3.61677
Nc-1-IP_pe	chr10	1.03E+08	1.03E+08	608	1.03E+08	17	5.14803	2.94914
Nc-1-IP_pe	chr10	1.03E+08	1.03E+08	662	1.03E+08	13	4.86362	3.18015
Nc-1-IP_pe	chr10	1.03E+08	1.03E+08	357	1.03E+08	10	4.99888	3.61677
Nc-1-IP_pe	chr10	1.03E+08	1.03E+08	513	1.03E+08	10	4.62142	3.388
Nc-1-IP_pe	chr10	1.03E+08	1.03E+08	545	1.03E+08	15	5.20142	3.14789
Nc-1-IP_pe	chr10	1.03E+08	1.03E+08	319	1.03E+08	15	8.54126	4.88897
Nc-1-IP_pe	chr10	1.03E+08	1.03E+08	416	1.03E+08	14	7.28181	4.3535
Nc-1-IP_pe	chr10	1.03E+08	1.03E+08	282	1.03E+08	11	4.54404	3.2242
Nc-1-IP_pe	chr10	1.03E+08	1.03E+08	370	1.03E+08	11	4.06403	2.95415
Nc-1-IP_pe	chr10	1.03E+08	1.03E+08	754	1.03E+08	12	6.86906	4.44438
Nc-1-IP_pe	chr10	1.03E+08	1.03E+08	264	1.03E+08	10	5.23357	3.76063
Nc-1-IP_pe	chr10	1.03E+08	1.03E+08	292	1.03E+08	14	6.61011	3.98382
Nc-1-IP_pe	chr10	1.03E+08	1.03E+08	632	1.03E+08	13	6.14303	3.86919
Nc-1-IP_pe	chr10	1.03E+08	1.03E+08	606	1.03E+08	13	3.86418	2.67468
Nc-1-IP_pe	chr10	1.03E+08	1.03E+08	262	1.03E+08	16	5.39474	3.14402
Nc-1-IP_pe	chr10	1.03E+08	1.03E+08	428	1.03E+08	16	6.52784	3.67908
Nc-1-IP_pe	chr10	1.03E+08	1.03E+08	281	1.03E+08	13	4.45253	2.96876
Nc-1-IP_pe	chr10	1.03E+08	1.03E+08	427	1.03E+08	12	5.25962	3.50902
Nc-1-IP_pe	chr10	1.03E+08	1.03E+08	277	1.03E+08	14	4.21449	2.76599
Nc-1-IP_pe	chr10	1.03E+08	1.03E+08	635	1.03E+08	14	6.16097	3.74327
Nc-1-IP_pe	chr10	1.03E+08	1.03E+08	342	1.03E+08	16	5.82808	3.34463
Nc-1-IP_pe	chr10	1.03E+08	1.03E+08	395	1.03E+08	9	3.42387	2.77697
Nc-1-IP_pe	chr10	1.03E+08	1.03E+08	473	1.03E+08	12	5.42384	3.60142
Nc-1-IP_pe	chr10	1.03E+08	1.03E+08	261	1.03E+08	14	6.85284	4.11607
Nc-1-IP_pe	chr10	1.03E+08	1.03E+08	986	1.03E+08	22	10.8426	4.87724

Nc-1-IP_pe chr10	1.04E+08	1.04E+08	534	1.04E+08	13	5.6727	3.61051
Nc-1-IP_pe chr10	1.04E+08	1.04E+08	365	1.04E+08	13	4.41954	2.952
Nc-1-IP_pe chr10	1.04E+08	1.04E+08	353	1.04E+08	13	4.41954	2.952
Nc-1-IP_pe chr10	1.04E+08	1.04E+08	398	1.04E+08	9	3.84391	3.03159
Nc-1-IP_pe chr10	1.04E+08	1.04E+08	733	1.04E+08	16	6.80394	3.81462
Nc-1-IP_pe chr10	1.04E+08	1.04E+08	564	1.04E+08	15	6.78905	3.93887
Nc-1-IP_pe chr10	1.04E+08	1.04E+08	516	1.04E+08	10	3.08907	2.49869
Nc-1-IP_pe chr10	1.04E+08	1.04E+08	726	1.04E+08	17	7.36791	3.96218
Nc-1-IP_pe chr10	1.04E+08	1.04E+08	383	1.04E+08	16	7.97877	4.4137
Nc-1-IP_pe chr10	1.04E+08	1.04E+08	315	1.04E+08	12	3.47963	2.55766
Nc-1-IP_pe chr10	1.04E+08	1.04E+08	536	1.04E+08	12	3.47963	2.55766
Nc-1-IP_pe chr10	1.04E+08	1.04E+08	522	1.04E+08	11	4.13673	2.99462
Nc-1-IP_pe chr10	1.04E+08	1.04E+08	416	1.04E+08	10	3.83201	2.92147
Nc-1-IP_pe chr10	1.04E+08	1.04E+08	910	1.04E+08	19	11.7093	5.84867
Nc-1-IP_pe chr10	1.04E+08	1.04E+08	609	1.04E+08	17	7.6273	4.08876
Nc-1-IP_pe chr10	1.04E+08	1.04E+08	313	1.04E+08	12	3.16137	2.39719
Nc-1-IP_pe chr10	1.04E+08	1.04E+08	897	1.04E+08	17	6.92131	3.74829
Nc-1-IP_pe chr10	1.04E+08	1.04E+08	338	1.04E+08	12	2.95438	2.29425
Nc-1-IP_pe chr10	1.04E+08	1.04E+08	387	1.04E+08	13	6.15968	3.87845
Nc-1-IP_pe chr10	1.04E+08	1.04E+08	574	1.04E+08	14	6.69566	4.03025
Nc-1-IP_pe chr10	1.04E+08	1.04E+08	446	1.04E+08	16	5.37454	3.13479
Nc-1-IP_pe chr10	1.04E+08	1.04E+08	267	1.04E+08	10	3.03453	2.46828
Nc-1-IP_pe chr10	1.04E+08	1.04E+08	779	1.04E+08	16	7.59698	4.21511
Nc-1-IP_pe chr10	1.04E+08	1.04E+08	811	1.04E+08	16	7.8853	4.36474
Nc-1-IP_pe chr10	1.04E+08	1.04E+08	592	1.04E+08	16	7.79347	4.31685
Nc-1-IP_pe chr10	1.04E+08	1.04E+08	269	1.04E+08	14	6.61011	3.98382
Nc-1-IP_pe chr10	1.04E+08	1.04E+08	480	1.04E+08	15	7.62034	4.38009
Nc-1-IP_pe chr10	1.04E+08	1.04E+08	422	1.04E+08	13	3.67377	2.58159
Nc-1-IP_pe chr10	1.04E+08	1.04E+08	430	1.04E+08	17	6.47874	3.54137
Nc-1-IP_pe chr10	1.04E+08	1.04E+08	351	1.04E+08	12	4.63088	3.16234
Nc-1-IP_pe chr10	1.04E+08	1.04E+08	282	1.04E+08	12	4.63088	3.16234
Nc-1-IP_pe chr10	1.04E+08	1.04E+08	370	1.04E+08	9	3.27326	2.68684
Nc-1-IP_pe chr10	1.04E+08	1.04E+08	610	1.04E+08	11	4.19517	3.02726
Nc-1-IP_pe chr10	1.04E+08	1.04E+08	603	1.04E+08	12	5.23082	3.49289
Nc-1-IP_pe chr10	1.04E+08	1.04E+08	568	1.04E+08	11	3.49235	2.64145
Nc-1-IP_pe chr10	1.04E+08	1.04E+08	287	1.04E+08	17	4.78101	2.79343
Nc-1-IP_pe chr10	1.04E+08	1.04E+08	327	1.04E+08	17	9.09431	4.8363
Nc-1-IP_pe chr10	1.04E+08	1.04E+08	514	1.04E+08	13	6.12646	3.85997
Nc-1-IP_pe chr10	1.04E+08	1.04E+08	496	1.04E+08	12	5.23082	3.49289
Nc-1-IP_pe chr10	1.04E+08	1.04E+08	785	1.04E+08	13	4.90859	3.20357
Nc-1-IP_pe chr10	1.04E+08	1.04E+08	402	1.04E+08	12	4.70523	3.20273
Nc-1-IP_pe chr10	1.04E+08	1.04E+08	515	1.04E+08	12	4.70082	3.20033
Nc-1-IP_pe chr10	1.04E+08	1.04E+08	310	1.04E+08	13	4.78908	3.14145
Nc-1-IP_pe chr10	1.04E+08	1.04E+08	448	1.04E+08	17	8.02964	4.28847
Nc-1-IP_pe chr10	1.04E+08	1.04E+08	447	1.04E+08	13	5.15976	3.33548

Nc-1-IP_pe	chr10	1.04E+08	1.04E+08	418	1.04E+08	14	5.51541	3.4073
Nc-1-IP_pe	chr10	1.04E+08	1.04E+08	566	1.04E+08	15	3.74763	2.48304
Nc-1-IP_pe	chr10	1.04E+08	1.04E+08	276	1.04E+08	13	5.9487	3.76157
Nc-1-IP_pe	chr10	1.04E+08	1.04E+08	281	1.04E+08	14	5.03085	3.16286
Nc-1-IP_pe	chr10	1.04E+08	1.04E+08	360	1.04E+08	11	3.68532	2.74592
Nc-1-IP_pe	chr10	1.04E+08	1.04E+08	547	1.04E+08	15	4.7819	2.95039
Nc-1-IP_pe	chr10	1.04E+08	1.04E+08	322	1.04E+08	12	4.88508	3.30112
Nc-1-IP_pe	chr10	1.04E+08	1.04E+08	275	1.04E+08	14	6.72985	4.04886
Nc-1-IP_pe	chr10	1.04E+08	1.04E+08	580	1.04E+08	13	5.03208	3.26819
Nc-1-IP_pe	chr10	1.04E+08	1.04E+08	271	1.04E+08	17	7.6273	4.08876
Nc-1-IP_pe	chr10	1.04E+08	1.04E+08	367	1.04E+08	13	6.14303	3.86919
Nc-1-IP_pe	chr10	1.04E+08	1.04E+08	372	1.04E+08	13	6.32982	3.97358
Nc-1-IP_pe	chr10	1.04E+08	1.04E+08	312	1.04E+08	16	6.8987	3.86161
Nc-1-IP_pe	chr10	1.04E+08	1.04E+08	702	1.04E+08	16	8.26996	4.56762
Nc-1-IP_pe	chr10	1.04E+08	1.04E+08	440	1.04E+08	12	4.70082	3.20033
Nc-1-IP_pe	chr10	1.04E+08	1.04E+08	280	1.04E+08	10	5.23357	3.76063
Nc-1-IP_pe	chr10	1.04E+08	1.04E+08	254	1.04E+08	8	3.74505	3.07688
Nc-1-IP_pe	chr10	1.04E+08	1.04E+08	274	1.04E+08	16	10.2925	5.69036
Nc-1-IP_pe	chr10	1.04E+08	1.04E+08	255	1.04E+08	13	5.36853	3.44651
Nc-1-IP_pe	chr10	1.04E+08	1.04E+08	276	1.04E+08	14	6.69566	4.03025
Nc-1-IP_pe	chr10	1.04E+08	1.04E+08	444	1.04E+08	10	4.16209	3.11446
Nc-1-IP_pe	chr10	1.04E+08	1.04E+08	342	1.04E+08	13	5.9487	3.76157
Nc-1-IP_pe	chr10	1.04E+08	1.04E+08	450	1.04E+08	15	5.66913	3.37372
Nc-1-IP_pe	chr10	1.04E+08	1.04E+08	778	1.04E+08	13	4.86362	3.18015
Nc-1-IP_pe	chr10	1.04E+08	1.04E+08	410	1.04E+08	14	5.30793	3.30182
Nc-1-IP_pe	chr10	1.04E+08	1.04E+08	395	1.04E+08	15	6.60497	3.84363
Nc-1-IP_pe	chr10	1.04E+08	1.04E+08	331	1.04E+08	15	8.26005	4.73146
Nc-1-IP_pe	chr10	1.04E+08	1.04E+08	285	1.04E+08	11	6.03439	4.10251
Nc-1-IP_pe	chr10	1.04E+08	1.04E+08	261	1.04E+08	11	6.03439	4.10251
Nc-1-IP_pe	chr10	1.04E+08	1.04E+08	297	1.04E+08	12	6.86906	4.44438
Nc-1-IP_pe	chr10	1.04E+08	1.04E+08	260	1.04E+08	9	4.25966	3.28797
Nc-1-IP_pe	chr10	1.04E+08	1.04E+08	285	1.04E+08	8	3.56021	2.95918
Nc-1-IP_pe	chr10	1.04E+08	1.04E+08	346	1.04E+08	18	6.15736	3.29676
Nc-1-IP_pe	chr10	1.04E+08	1.04E+08	301	1.04E+08	13	5.36853	3.44651
Nc-1-IP_pe	chr10	1.04E+08	1.04E+08	460	1.04E+08	16	4.59224	2.7853
Nc-1-IP_pe	chr10	1.04E+08	1.04E+08	273	1.04E+08	10	5.17577	3.72509
Nc-1-IP_pe	chr10	1.04E+08	1.04E+08	308	1.04E+08	10	3.89064	2.95552
Nc-1-IP_pe	chr10	1.04E+08	1.04E+08	322	1.04E+08	10	3.08907	2.49869
Nc-1-IP_pe	chr10	1.04E+08	1.04E+08	317	1.04E+08	14	5.56442	3.43239
Nc-1-IP_pe	chr10	1.04E+08	1.04E+08	267	1.04E+08	12	5.17301	3.46058
Nc-1-IP_pe	chr10	1.04E+08	1.04E+08	280	1.04E+08	12	4.24064	2.95299
Nc-1-IP_pe	chr10	1.04E+08	1.04E+08	966	1.04E+08	14	6.412	3.87704
Nc-1-IP_pe	chr10	1.04E+08	1.04E+08	786	1.04E+08	17	4.78101	2.79343
Nc-1-IP_pe	chr10	1.04E+08	1.04E+08	285	1.04E+08	10	3.89064	2.95552
Nc-1-IP_pe	chr10	1.04E+08	1.04E+08	278	1.04E+08	12	4.70082	3.20033

Nc-1-IP_pe	chr10	1.05E+08	1.05E+08	435	1.05E+08	12	6.58417	4.27436
Nc-1-IP_pe	chr10	1.05E+08	1.05E+08	346	1.05E+08	10	4.99888	3.61677
Nc-1-IP_pe	chr10	1.05E+08	1.05E+08	548	1.05E+08	10	3.89064	2.95552
Nc-1-IP_pe	chr10	1.05E+08	1.05E+08	339	1.05E+08	8	3.74505	3.07688
Nc-1-IP_pe	chr10	1.05E+08	1.05E+08	254	1.05E+08	10	5.23357	3.76063
Nc-1-IP_pe	chr10	1.05E+08	1.05E+08	682	1.05E+08	12	4.70082	3.20033
Nc-1-IP_pe	chr10	1.05E+08	1.05E+08	365	1.05E+08	15	6.78905	3.93887
Nc-1-IP_pe	chr10	1.05E+08	1.05E+08	261	1.05E+08	8	3.52386	2.93611
Nc-1-IP_pe	chr10	1.05E+08	1.05E+08	346	1.05E+08	8	3.0402	2.6319
Nc-1-IP_pe	chr10	1.05E+08	1.05E+08	358	1.05E+08	14	6.69566	4.03025
Nc-1-IP_pe	chr10	1.05E+08	1.05E+08	299	1.05E+08	8	3.56021	2.95918
Nc-1-IP_pe	chr10	1.05E+08	1.05E+08	261	1.05E+08	11	3.64839	2.72584
Nc-1-IP_pe	chr10	1.05E+08	1.05E+08	303	1.05E+08	9	4.25966	3.28797
Nc-1-IP_pe	chr10	1.05E+08	1.05E+08	284	1.05E+08	8	3.56021	2.95918
Nc-1-IP_pe	chr10	1.05E+08	1.05E+08	483	1.05E+08	10	3.08907	2.49869
Nc-1-IP_pe	chr10	1.05E+08	1.05E+08	373	1.05E+08	11	4.54404	3.2242
Nc-1-IP_pe	chr10	1.05E+08	1.05E+08	336	1.05E+08	23	7.4429	3.36882
Nc-1-IP_pe	chr10	1.06E+08	1.06E+08	482	1.06E+08	15	6.85973	3.97568
Nc-1-IP_pe	chr10	1.06E+08	1.06E+08	288	1.06E+08	11	6.03439	4.10251
Nc-1-IP_pe	chr10	1.06E+08	1.06E+08	310	1.06E+08	11	5.10577	3.5486
Nc-1-IP_pe	chr10	1.07E+08	1.07E+08	597	1.07E+08	10	5.23357	3.76063
Nc-1-IP_pe	chr10	1.07E+08	1.07E+08	427	1.07E+08	10	4.99888	3.61677
Nc-1-IP_pe	chr10	1.07E+08	1.07E+08	475	1.07E+08	11	6.03439	4.10251
Nc-1-IP_pe	chr10	1.07E+08	1.07E+08	260	1.07E+08	9	4.46937	3.41876
Nc-1-IP_pe	chr10	1.07E+08	1.07E+08	384	1.07E+08	9	4.46937	3.41876
Nc-1-IP_pe	chr10	1.08E+08	1.08E+08	264	1.08E+08	10	3.89064	2.95552
Nc-1-IP_pe	chr10	1.08E+08	1.08E+08	267	1.08E+08	9	3.72192	2.95716
Nc-1-IP_pe	chr10	1.09E+08	1.09E+08	313	1.09E+08	10	5.23357	3.76063
Nc-1-IP_pe	chr10	1.09E+08	1.09E+08	285	1.09E+08	10	5.23357	3.76063
Nc-1-IP_pe	chr10	1.09E+08	1.09E+08	381	1.09E+08	10	4.93609	3.57849
Nc-1-IP_pe	chr10	1.09E+08	1.09E+08	647	1.09E+08	10	5.23357	3.76063
Nc-1-IP_pe	chr10	1.1E+08	1.1E+08	609	1.1E+08	8	3.74505	3.07688
Nc-1-IP_pe	chr10	1.1E+08	1.1E+08	671	1.1E+08	10	5.23357	3.76063
Nc-1-IP_pe	chr10	1.1E+08	1.1E+08	537	1.1E+08	18	12.0366	6.24715
Nc-1-IP_pe	chr10	1.1E+08	1.1E+08	580	1.1E+08	12	6.86906	4.44438
Nc-1-IP_pe	chr10	1.1E+08	1.1E+08	409	1.1E+08	10	5.23357	3.76063
Nc-1-IP_pe	chr10	1.1E+08	1.1E+08	834	1.1E+08	12	6.86906	4.44438
Nc-1-IP_pe	chr10	1.1E+08	1.1E+08	581	1.1E+08	9	4.25966	3.28797
Nc-1-IP_pe	chr10	1.1E+08	1.1E+08	276	1.1E+08	11	6.03439	4.10251
Nc-1-IP_pe	chr10	1.1E+08	1.1E+08	390	1.1E+08	13	7.7352	4.78626
Nc-1-IP_pe	chr10	1.1E+08	1.1E+08	1037	1.1E+08	9	4.46937	3.41876
Nc-1-IP_pe	chr10	1.1E+08	1.1E+08	445	1.1E+08	12	6.86906	4.44438
Nc-1-IP_pe	chr10	1.1E+08	1.1E+08	632	1.1E+08	9	4.46937	3.41876
Nc-1-IP_pe	chr10	1.1E+08	1.1E+08	491	1.1E+08	8	3.56021	2.95918
Nc-1-IP_pe	chr10	1.1E+08	1.1E+08	333	1.1E+08	10	5.23357	3.76063

Nc-1-IP_pe	chr10	1.1E+08	1.1E+08	397	1.1E+08	9	3.72192	2.95716
Nc-1-IP_pe	chr10	1.1E+08	1.1E+08	321	1.1E+08	9	3.27326	2.68684
Nc-1-IP_pe	chr10	1.1E+08	1.1E+08	310	1.1E+08	10	4.79071	3.49018
Nc-1-IP_pe	chr10	1.1E+08	1.1E+08	291	1.1E+08	10	4.93609	3.57849
Nc-1-IP_pe	chr10	1.1E+08	1.1E+08	256	1.1E+08	8	3.0402	2.6319
Nc-1-IP_pe	chr10	1.1E+08	1.1E+08	300	1.1E+08	11	5.95085	4.05188
Nc-1-IP_pe	chr10	1.1E+08	1.1E+08	255	1.1E+08	10	3.89064	2.95552
Nc-1-IP_pe	chr10	1.1E+08	1.1E+08	272	1.1E+08	10	5.23357	3.76063
Nc-1-IP_pe	chr10	1.1E+08	1.1E+08	270	1.1E+08	16	6.33285	3.58457
Nc-1-IP_pe	chr10	1.11E+08	1.11E+08	274	1.11E+08	13	7.7352	4.78626
Nc-1-IP_pe	chr10	1.11E+08	1.11E+08	262	1.11E+08	13	7.19153	4.46655
Nc-1-IP_pe	chr10	1.11E+08	1.11E+08	458	1.11E+08	13	5.9487	3.76157
Nc-1-IP_pe	chr10	1.11E+08	1.11E+08	276	1.11E+08	8	3.74505	3.07688
Nc-1-IP_pe	chr10	1.11E+08	1.11E+08	569	1.11E+08	8	3.74505	3.07688
Nc-1-IP_pe	chr10	1.11E+08	1.11E+08	765	1.11E+08	13	7.7352	4.78626
Nc-1-IP_pe	chr10	1.11E+08	1.11E+08	323	1.11E+08	14	8.20556	4.87976
Nc-1-IP_pe	chr10	1.11E+08	1.11E+08	372	1.11E+08	17	10.6711	5.69559
Nc-1-IP_pe	chr10	1.11E+08	1.11E+08	255	1.11E+08	11	5.10577	3.5486
Nc-1-IP_pe	chr10	1.11E+08	1.11E+08	525	1.11E+08	11	5.10577	3.5486
Nc-1-IP_pe	chr10	1.11E+08	1.11E+08	312	1.11E+08	9	3.27326	2.68684
Nc-1-IP_pe	chr10	1.11E+08	1.11E+08	340	1.11E+08	10	5.23357	3.76063
Nc-1-IP_pe	chr10	1.11E+08	1.11E+08	433	1.11E+08	9	4.46937	3.41876
Nc-1-IP_pe	chr10	1.12E+08	1.12E+08	485	1.12E+08	14	8.20556	4.87976
Nc-1-IP_pe	chr10	1.12E+08	1.12E+08	553	1.12E+08	11	4.54404	3.2242
Nc-1-IP_pe	chr10	1.12E+08	1.12E+08	340	1.12E+08	9	4.25966	3.28797
Nc-1-IP_pe	chr10	1.12E+08	1.12E+08	393	1.12E+08	11	6.03439	4.10251
Nc-1-IP_pe	chr10	1.12E+08	1.12E+08	276	1.12E+08	11	6.03439	4.10251
Nc-1-IP_pe	chr10	1.12E+08	1.12E+08	262	1.12E+08	7	3.06442	2.73501
Nc-1-IP_pe	chr10	1.12E+08	1.12E+08	515	1.12E+08	10	4.39571	3.25288
Nc-1-IP_pe	chr10	1.12E+08	1.12E+08	633	1.12E+08	21	11.4957	5.35164
Nc-1-IP_pe	chr10	1.13E+08	1.13E+08	392	1.13E+08	8	3.08755	2.66145
Nc-1-IP_pe	chr10	1.13E+08	1.13E+08	526	1.13E+08	9	3.72192	2.95716
Nc-1-IP_pe	chr10	1.13E+08	1.13E+08	498	1.13E+08	9	4.46937	3.41876
Nc-1-IP_pe	chr10	1.13E+08	1.13E+08	812	1.13E+08	14	8.63073	5.12814
Nc-1-IP_pe	chr10	1.13E+08	1.13E+08	354	1.13E+08	10	5.23357	3.76063
Nc-1-IP_pe	chr10	1.13E+08	1.13E+08	507	1.13E+08	8	3.74505	3.07688
Nc-1-IP_pe	chr10	1.13E+08	1.13E+08	483	1.13E+08	10	5.23357	3.76063
Nc-1-IP_pe	chr10	1.13E+08	1.13E+08	411	1.13E+08	9	4.35492	3.34727
Nc-1-IP_pe	chr10	1.14E+08	1.14E+08	279	1.14E+08	10	4.39571	3.25288
Nc-1-IP_pe	chr10	1.14E+08	1.14E+08	313	1.14E+08	8	3.74505	3.07688
Nc-1-IP_pe	chr10	1.14E+08	1.14E+08	509	1.14E+08	10	5.23357	3.76063
Nc-1-IP_pe	chr10	1.14E+08	1.14E+08	370	1.14E+08	8	3.74505	3.07688
Nc-1-IP_pe	chr10	1.14E+08	1.14E+08	292	1.14E+08	11	6.03439	4.10251
Nc-1-IP_pe	chr10	1.14E+08	1.14E+08	304	1.14E+08	9	4.25966	3.28797
Nc-1-IP_pe	chr10	1.14E+08	1.14E+08	359	1.14E+08	9	4.46937	3.41876

Nc-1-IP_pe	chr10	1.14E+08	1.14E+08	351	1.14E+08	10	5.23357	3.76063
Nc-1-IP_pe	chr10	1.14E+08	1.14E+08	470	1.14E+08	12	6.86906	4.44438
Nc-1-IP_pe	chr10	1.14E+08	1.14E+08	254	1.14E+08	10	5.23357	3.76063
Nc-1-IP_pe	chr10	1.14E+08	1.14E+08	324	1.14E+08	10	4.99888	3.61677
Nc-1-IP_pe	chr10	1.14E+08	1.14E+08	332	1.14E+08	13	5.9487	3.76157
Nc-1-IP_pe	chr10	1.14E+08	1.14E+08	435	1.14E+08	9	3.27326	2.68684
Nc-1-IP_pe	chr10	1.14E+08	1.14E+08	671	1.14E+08	10	5.23357	3.76063
Nc-1-IP_pe	chr10	1.14E+08	1.14E+08	364	1.14E+08	11	6.03439	4.10251
Nc-1-IP_pe	chr10	1.14E+08	1.14E+08	998	1.14E+08	10	5.23357	3.76063
Nc-1-IP_pe	chr10	1.14E+08	1.14E+08	266	1.14E+08	12	6.86906	4.44438
Nc-1-IP_pe	chr10	1.14E+08	1.14E+08	320	1.14E+08	8	3.74505	3.07688
Nc-1-IP_pe	chr10	1.14E+08	1.14E+08	323	1.14E+08	10	4.39571	3.25288
Nc-1-IP_pe	chr10	1.14E+08	1.14E+08	310	1.14E+08	8	3.56021	2.95918
Nc-1-IP_pe	chr10	1.14E+08	1.14E+08	288	1.14E+08	14	7.42849	4.43574
Nc-1-IP_pe	chr10	1.14E+08	1.14E+08	427	1.14E+08	9	4.46937	3.41876
Nc-1-IP_pe	chr10	1.14E+08	1.14E+08	271	1.14E+08	10	5.23357	3.76063
Nc-1-IP_pe	chr10	1.15E+08	1.15E+08	522	1.15E+08	8	3.74505	3.07688
Nc-1-IP_pe	chr10	1.15E+08	1.15E+08	291	1.15E+08	11	5.7051	3.90381
Nc-1-IP_pe	chr10	1.15E+08	1.15E+08	624	1.15E+08	9	4.46937	3.41876
Nc-1-IP_pe	chr10	1.15E+08	1.15E+08	425	1.15E+08	13	6.62436	4.14003
Nc-1-IP_pe	chr10	1.15E+08	1.15E+08	303	1.15E+08	8	3.74505	3.07688
Nc-1-IP_pe	chr10	1.15E+08	1.15E+08	532	1.15E+08	11	6.03439	4.10251
Nc-1-IP_pe	chr10	1.15E+08	1.15E+08	292	1.15E+08	9	4.25966	3.28797
Nc-1-IP_pe	chr10	1.15E+08	1.15E+08	396	1.15E+08	14	8.29543	4.93196
Nc-1-IP_pe	chr10	1.15E+08	1.15E+08	396	1.15E+08	9	4.46937	3.41876
Nc-1-IP_pe	chr10	1.16E+08	1.16E+08	258	1.16E+08	10	5.23357	3.76063
Nc-1-IP_pe	chr10	1.16E+08	1.16E+08	417	1.16E+08	9	4.46937	3.41876
Nc-1-IP_pe	chr10	1.16E+08	1.16E+08	350	1.16E+08	8	3.74505	3.07688
Nc-1-IP_pe	chr10	1.16E+08	1.16E+08	392	1.16E+08	9	4.46937	3.41876
Nc-1-IP_pe	chr10	1.16E+08	1.16E+08	284	1.16E+08	8	3.74505	3.07688
Nc-1-IP_pe	chr10	1.16E+08	1.16E+08	276	1.16E+08	12	6.86906	4.44438
Nc-1-IP_pe	chr10	1.17E+08	1.17E+08	499	1.17E+08	9	4.46937	3.41876
Nc-1-IP_pe	chr10	1.17E+08	1.17E+08	315	1.17E+08	12	5.84943	3.84431
Nc-1-IP_pe	chr10	1.17E+08	1.17E+08	416	1.17E+08	7	2.9043	2.63038
Nc-1-IP_pe	chr10	1.17E+08	1.17E+08	303	1.17E+08	10	3.89064	2.95552
Nc-1-IP_pe	chr10	1.17E+08	1.17E+08	453	1.17E+08	9	4.04582	3.15559
Nc-1-IP_pe	chr10	1.17E+08	1.17E+08	299	1.17E+08	8	3.61691	2.99521
Nc-1-IP_pe	chr10	1.17E+08	1.17E+08	499	1.17E+08	11	6.03439	4.10251
Nc-1-IP_pe	chr10	1.17E+08	1.17E+08	462	1.17E+08	10	4.33501	3.21677
Nc-1-IP_pe	chr10	1.17E+08	1.17E+08	343	1.17E+08	9	3.72192	2.95716
Nc-1-IP_pe	chr10	1.17E+08	1.17E+08	300	1.17E+08	9	3.92293	3.08
Nc-1-IP_pe	chr10	1.17E+08	1.17E+08	492	1.17E+08	8	3.74505	3.07688
Nc-1-IP_pe	chr10	1.17E+08	1.17E+08	369	1.17E+08	8	3.74505	3.07688
Nc-1-IP_pe	chr10	1.17E+08	1.17E+08	526	1.17E+08	10	5.23357	3.76063
Nc-1-IP_pe	chr10	1.17E+08	1.17E+08	611	1.17E+08	11	6.03439	4.10251

Nc-1-IP_pe	chr10	1.17E+08	1.17E+08	724	1.17E+08	15	5.66913	3.37372
Nc-1-IP_pe	chr10	1.17E+08	1.17E+08	441	1.17E+08	9	4.46937	3.41876
Nc-1-IP_pe	chr10	1.17E+08	1.17E+08	698	1.17E+08	12	6.86906	4.44438
Nc-1-IP_pe	chr10	1.17E+08	1.17E+08	416	1.17E+08	9	4.46937	3.41876
Nc-1-IP_pe	chr10	1.17E+08	1.17E+08	406	1.17E+08	14	8.63073	5.12814
Nc-1-IP_pe	chr10	1.17E+08	1.17E+08	625	1.17E+08	8	3.74505	3.07688
Nc-1-IP_pe	chr10	1.17E+08	1.17E+08	277	1.17E+08	11	5.7051	3.90381
Nc-1-IP_pe	chr10	1.17E+08	1.17E+08	396	1.17E+08	9	4.05981	3.16422
Nc-1-IP_pe	chr10	1.17E+08	1.17E+08	311	1.17E+08	11	6.03439	4.10251
Nc-1-IP_pe	chr10	1.18E+08	1.18E+08	409	1.18E+08	11	6.03439	4.10251
Nc-1-IP_pe	chr10	1.18E+08	1.18E+08	332	1.18E+08	11	6.03439	4.10251
Nc-1-IP_pe	chr10	1.18E+08	1.18E+08	478	1.18E+08	10	5.23357	3.76063
Nc-1-IP_pe	chr10	1.18E+08	1.18E+08	427	1.18E+08	12	6.86906	4.44438
Nc-1-IP_pe	chr10	1.18E+08	1.18E+08	376	1.18E+08	8	3.74505	3.07688
Nc-1-IP_pe	chr10	1.18E+08	1.18E+08	344	1.18E+08	10	5.23357	3.76063
Nc-1-IP_pe	chr10	1.18E+08	1.18E+08	455	1.18E+08	13	7.42513	4.60316
Nc-1-IP_pe	chr10	1.18E+08	1.18E+08	992	1.18E+08	9	2.89249	2.46179
Nc-1-IP_pe	chr10	1.18E+08	1.18E+08	353	1.18E+08	8	3.74505	3.07688
Nc-1-IP_pe	chr10	1.18E+08	1.18E+08	681	1.18E+08	9	4.46937	3.41876
Nc-1-IP_pe	chr10	1.18E+08	1.18E+08	285	1.18E+08	9	4.46937	3.41876
Nc-1-IP_pe	chr10	1.18E+08	1.18E+08	280	1.18E+08	13	6.62436	4.14003
Nc-1-IP_pe	chr10	1.18E+08	1.18E+08	859	1.18E+08	17	11.4763	6.15376
Nc-1-IP_pe	chr10	1.18E+08	1.18E+08	326	1.18E+08	11	6.03439	4.10251
Nc-1-IP_pe	chr10	1.18E+08	1.18E+08	259	1.18E+08	9	4.25966	3.28797
Nc-1-IP_pe	chr10	1.19E+08	1.19E+08	441	1.19E+08	9	4.46937	3.41876
Nc-1-IP_pe	chr10	1.19E+08	1.19E+08	263	1.19E+08	12	6.86906	4.44438
Nc-1-IP_pe	chr10	1.19E+08	1.19E+08	253	1.19E+08	10	3.89064	2.95552
Nc-1-IP_pe	chr10	1.19E+08	1.19E+08	309	1.19E+08	9	4.46937	3.41876
Nc-1-IP_pe	chr10	1.19E+08	1.19E+08	560	1.19E+08	10	4.39571	3.25288
Nc-1-IP_pe	chr10	1.19E+08	1.19E+08	359	1.19E+08	7	3.06442	2.73501
Nc-1-IP_pe	chr10	1.19E+08	1.19E+08	324	1.19E+08	11	6.03439	4.10251
Nc-1-IP_pe	chr10	1.19E+08	1.19E+08	295	1.19E+08	7	3.06442	2.73501
Nc-1-IP_pe	chr10	1.19E+08	1.19E+08	349	1.19E+08	15	8.26005	4.73146
Nc-1-IP_pe	chr10	1.19E+08	1.19E+08	525	1.19E+08	8	3.74505	3.07688
Nc-1-IP_pe	chr10	1.19E+08	1.19E+08	263	1.19E+08	7	3.06442	2.73501
Nc-1-IP_pe	chr10	1.19E+08	1.19E+08	367	1.19E+08	8	3.74505	3.07688
Nc-1-IP_pe	chr10	1.19E+08	1.19E+08	731	1.19E+08	9	4.46937	3.41876
Nc-1-IP_pe	chr10	1.19E+08	1.19E+08	431	1.19E+08	10	4.77495	3.48064
Nc-1-IP_pe	chr10	1.19E+08	1.19E+08	307	1.19E+08	9	4.46937	3.41876
Nc-1-IP_pe	chr10	1.19E+08	1.19E+08	831	1.19E+08	11	6.03439	4.10251
Nc-1-IP_pe	chr10	1.19E+08	1.19E+08	454	1.19E+08	11	4.06403	2.95415
Nc-1-IP_pe	chr10	1.19E+08	1.19E+08	498	1.19E+08	11	5.63263	3.8604
Nc-1-IP_pe	chr10	1.19E+08	1.19E+08	313	1.19E+08	8	3.56021	2.95918
Nc-1-IP_pe	chr10	1.19E+08	1.19E+08	441	1.19E+08	11	4.54404	3.2242
Nc-1-IP_pe	chr10	1.19E+08	1.19E+08	366	1.19E+08	10	3.4604	2.70797

Nc-1-IP_pe	chr10	1.19E+08	1.19E+08	260	1.19E+08	10	5.23357	3.76063
Nc-1-IP_pe	chr10	1.19E+08	1.19E+08	342	1.19E+08	10	3.89064	2.95552
Nc-1-IP_pe	chr10	1.19E+08	1.19E+08	642	1.19E+08	9	4.25966	3.28797
Nc-1-IP_pe	chr10	1.19E+08	1.19E+08	277	1.19E+08	12	6.58417	4.27436
Nc-1-IP_pe	chr10	1.19E+08	1.19E+08	540	1.19E+08	9	4.46937	3.41876
Nc-1-IP_pe	chr10	1.19E+08	1.19E+08	320	1.19E+08	9	4.25966	3.28797
Nc-1-IP_pe	chr10	1.19E+08	1.19E+08	310	1.19E+08	10	5.23357	3.76063
Nc-1-IP_pe	chr10	1.19E+08	1.19E+08	465	1.19E+08	12	5.84943	3.84431
Nc-1-IP_pe	chr10	1.19E+08	1.19E+08	472	1.19E+08	9	3.27326	2.68684
Nc-1-IP_pe	chr10	1.19E+08	1.19E+08	412	1.19E+08	30	10.9504	3.972
Nc-1-IP_pe	chr10	1.19E+08	1.19E+08	289	1.19E+08	13	7.34205	4.55444
Nc-1-IP_pe	chr10	1.19E+08	1.19E+08	322	1.19E+08	7	2.98886	2.68558
Nc-1-IP_pe	chr10	1.19E+08	1.19E+08	296	1.19E+08	10	4.77495	3.48064
Nc-1-IP_pe	chr10	1.19E+08	1.19E+08	270	1.19E+08	10	5.23357	3.76063
Nc-1-IP_pe	chr10	1.19E+08	1.19E+08	455	1.19E+08	11	4.06403	2.95415
Nc-1-IP_pe	chr10	1.19E+08	1.19E+08	808	1.19E+08	10	5.23357	3.76063
Nc-1-IP_pe	chr10	1.19E+08	1.19E+08	434	1.19E+08	9	4.46937	3.41876
Nc-1-IP_pe	chr10	1.19E+08	1.19E+08	466	1.19E+08	11	5.77463	3.94557
Nc-1-IP_pe	chr10	1.19E+08	1.19E+08	257	1.19E+08	10	3.89064	2.95552
Nc-1-IP_pe	chr10	1.19E+08	1.19E+08	338	1.19E+08	10	3.94999	2.99009
Nc-1-IP_pe	chr10	1.19E+08	1.19E+08	781	1.19E+08	10	5.23357	3.76063
Nc-1-IP_pe	chr10	1.19E+08	1.19E+08	496	1.19E+08	8	3.74505	3.07688
Nc-1-IP_pe	chr10	1.19E+08	1.19E+08	652	1.19E+08	9	3.72192	2.95716
Nc-1-IP_pe	chr10	1.19E+08	1.19E+08	323	1.19E+08	9	3.72192	2.95716
Nc-1-IP_pe	chr10	1.19E+08	1.19E+08	347	1.19E+08	12	6.86906	4.44438
Nc-1-IP_pe	chr10	1.19E+08	1.19E+08	254	1.19E+08	10	5.14046	3.70342
Nc-1-IP_pe	chr10	1.19E+08	1.19E+08	418	1.19E+08	10	4.39571	3.25288
Nc-1-IP_pe	chr10	1.19E+08	1.19E+08	396	1.19E+08	9	4.46937	3.41876
Nc-1-IP_pe	chr10	1.19E+08	1.19E+08	363	1.19E+08	9	4.46937	3.41876
Nc-1-IP_pe	chr10	1.19E+08	1.19E+08	465	1.19E+08	9	3.72192	2.95716
Nc-1-IP_pe	chr10	1.19E+08	1.19E+08	349	1.19E+08	13	6.72991	4.20021
Nc-1-IP_pe	chr10	1.19E+08	1.19E+08	275	1.19E+08	10	3.89064	2.95552
Nc-1-IP_pe	chr10	1.2E+08	1.2E+08	454	1.2E+08	9	4.25966	3.28797
Nc-1-IP_pe	chr10	1.2E+08	1.2E+08	304	1.2E+08	9	4.25966	3.28797
Nc-1-IP_pe	chr10	1.2E+08	1.2E+08	408	1.2E+08	12	5.23082	3.49289
Nc-1-IP_pe	chr10	1.2E+08	1.2E+08	377	1.2E+08	14	6.69566	4.03025
Nc-1-IP_pe	chr10	1.2E+08	1.2E+08	514	1.2E+08	11	6.03439	4.10251
Nc-1-IP_pe	chr10	1.2E+08	1.2E+08	370	1.2E+08	8	3.08755	2.66145
Nc-1-IP_pe	chr10	1.2E+08	1.2E+08	654	1.2E+08	9	4.46937	3.41876
Nc-1-IP_pe	chr10	1.2E+08	1.2E+08	504	1.2E+08	11	6.03439	4.10251
Nc-1-IP_pe	chr10	1.2E+08	1.2E+08	289	1.2E+08	9	3.54889	2.85226
Nc-1-IP_pe	chr10	1.2E+08	1.2E+08	546	1.2E+08	11	4.74697	3.34038
Nc-1-IP_pe	chr10	1.2E+08	1.2E+08	332	1.2E+08	7	3.06442	2.73501
Nc-1-IP_pe	chr10	1.2E+08	1.2E+08	994	1.2E+08	12	5.84943	3.84431
Nc-1-IP_pe	chr10	1.2E+08	1.2E+08	470	1.2E+08	10	4.99888	3.61677

Nc-1-IP_pe	chr10	1.2E+08	1.2E+08	514	1.2E+08	8	3.74505	3.07688
Nc-1-IP_pe	chr10	1.2E+08	1.2E+08	371	1.2E+08	9	4.25966	3.28797
Nc-1-IP_pe	chr10	1.2E+08	1.2E+08	313	1.2E+08	12	6.86906	4.44438
Nc-1-IP_pe	chr10	1.2E+08	1.2E+08	537	1.2E+08	9	4.46937	3.41876
Nc-1-IP_pe	chr10	1.2E+08	1.2E+08	400	1.2E+08	10	4.99888	3.61677
Nc-1-IP_pe	chr10	1.2E+08	1.2E+08	296	1.2E+08	10	5.23357	3.76063
Nc-1-IP_pe	chr10	1.2E+08	1.2E+08	389	1.2E+08	10	5.23357	3.76063
Nc-1-IP_pe	chr10	1.21E+08	1.21E+08	418	1.21E+08	16	6.8987	3.86161
Nc-1-IP_pe	chr10	1.21E+08	1.21E+08	506	1.21E+08	15	7.46994	4.29894
Nc-1-IP_pe	chr10	1.21E+08	1.21E+08	436	1.21E+08	8	3.74505	3.07688
Nc-1-IP_pe	chr10	1.21E+08	1.21E+08	272	1.21E+08	9	4.46937	3.41876
Nc-1-IP_pe	chr10	1.21E+08	1.21E+08	325	1.21E+08	11	6.03439	4.10251
Nc-1-IP_pe	chr10	1.21E+08	1.21E+08	516	1.21E+08	9	4.46937	3.41876
Nc-1-IP_pe	chr10	1.21E+08	1.21E+08	489	1.21E+08	9	4.46937	3.41876
Nc-1-IP_pe	chr10	1.21E+08	1.21E+08	316	1.21E+08	10	5.23357	3.76063
Nc-1-IP_pe	chr10	1.22E+08	1.22E+08	297	1.22E+08	8	3.74505	3.07688
Nc-1-IP_pe	chr10	1.22E+08	1.22E+08	328	1.22E+08	8	3.74505	3.07688
Nc-1-IP_pe	chr10	1.22E+08	1.22E+08	296	1.22E+08	10	4.99888	3.61677
Nc-1-IP_pe	chr10	1.22E+08	1.22E+08	253	1.22E+08	9	4.46937	3.41876
Nc-1-IP_pe	chr10	1.22E+08	1.22E+08	511	1.22E+08	10	5.23357	3.76063
Nc-1-IP_pe	chr10	1.22E+08	1.22E+08	540	1.22E+08	13	4.56123	3.02417
Nc-1-IP_pe	chr10	1.22E+08	1.22E+08	428	1.22E+08	10	4.62142	3.388
Nc-1-IP_pe	chr10	1.22E+08	1.22E+08	268	1.22E+08	9	4.46937	3.41876
Nc-1-IP_pe	chr10	1.22E+08	1.22E+08	264	1.22E+08	9	4.46937	3.41876
Nc-1-IP_pe	chr10	1.22E+08	1.22E+08	269	1.22E+08	10	4.93609	3.57849
Nc-1-IP_pe	chr10	1.22E+08	1.22E+08	306	1.22E+08	10	3.40382	2.67582
Nc-1-IP_pe	chr10	1.22E+08	1.22E+08	853	1.22E+08	14	8.63073	5.12814
Nc-1-IP_pe	chr10	1.22E+08	1.22E+08	278	1.22E+08	11	6.03439	4.10251
Nc-1-IP_pe	chr10	1.22E+08	1.22E+08	1023	1.22E+08	12	6.71369	4.35145
Nc-1-IP_pe	chr10	1.22E+08	1.22E+08	428	1.22E+08	8	3.74505	3.07688
Nc-1-IP_pe	chr10	1.22E+08	1.22E+08	665	1.22E+08	8	3.74505	3.07688
Nc-1-IP_pe	chr10	1.22E+08	1.22E+08	307	1.22E+08	9	4.46937	3.41876
Nc-1-IP_pe	chr10	1.22E+08	1.22E+08	268	1.22E+08	9	4.46937	3.41876
Nc-1-IP_pe	chr10	1.22E+08	1.22E+08	656	1.22E+08	8	3.08755	2.66145
Nc-1-IP_pe	chr10	1.22E+08	1.22E+08	298	1.22E+08	9	4.46937	3.41876
Nc-1-IP_pe	chr10	1.22E+08	1.22E+08	358	1.22E+08	12	6.86906	4.44438
Nc-1-IP_pe	chr10	1.22E+08	1.22E+08	301	1.22E+08	9	4.46937	3.41876
Nc-1-IP_pe	chr10	1.22E+08	1.22E+08	478	1.22E+08	12	6.86906	4.44438
Nc-1-IP_pe	chr10	1.22E+08	1.22E+08	288	1.22E+08	10	5.23357	3.76063
Nc-1-IP_pe	chr10	1.22E+08	1.22E+08	359	1.22E+08	8	3.74505	3.07688
Nc-1-IP_pe	chr10	1.22E+08	1.22E+08	333	1.22E+08	15	5.20142	3.14789
Nc-1-IP_pe	chr10	1.22E+08	1.22E+08	367	1.22E+08	11	6.03439	4.10251
Nc-1-IP_pe	chr10	1.22E+08	1.22E+08	269	1.22E+08	12	6.86906	4.44438
Nc-1-IP_pe	chr10	1.23E+08	1.23E+08	263	1.23E+08	8	3.56021	2.95918
Nc-1-IP_pe	chr10	1.23E+08	1.23E+08	294	1.23E+08	9	4.46937	3.41876

Nc-1-IP_pe	chr10	1.23E+08	1.23E+08	398	1.23E+08	8	3.08755	2.66145
Nc-1-IP_pe	chr10	1.23E+08	1.23E+08	666	1.23E+08	10	5.23357	3.76063
Nc-1-IP_pe	chr10	1.23E+08	1.23E+08	479	1.23E+08	9	4.20361	3.25317
Nc-1-IP_pe	chr10	1.23E+08	1.23E+08	270	1.23E+08	10	5.23357	3.76063
Nc-1-IP_pe	chr10	1.23E+08	1.23E+08	300	1.23E+08	8	3.74505	3.07688
Nc-1-IP_pe	chr10	1.23E+08	1.23E+08	280	1.23E+08	10	5.23357	3.76063
Nc-1-IP_pe	chr10	1.23E+08	1.23E+08	338	1.23E+08	10	4.39571	3.25288
Nc-1-IP_pe	chr10	1.23E+08	1.23E+08	304	1.23E+08	11	6.03439	4.10251
Nc-1-IP_pe	chr10	1.23E+08	1.23E+08	484	1.23E+08	10	4.39571	3.25288
Nc-1-IP_pe	chr10	1.23E+08	1.23E+08	308	1.23E+08	11	6.03439	4.10251
Nc-1-IP_pe	chr10	1.23E+08	1.23E+08	366	1.23E+08	12	5.84943	3.84431
Nc-1-IP_pe	chr10	1.23E+08	1.23E+08	314	1.23E+08	7	3.06442	2.73501
Nc-1-IP_pe	chr10	1.23E+08	1.23E+08	274	1.23E+08	14	7.14701	4.27839
Nc-1-IP_pe	chr10	1.23E+08	1.23E+08	482	1.23E+08	9	4.46937	3.41876
Nc-1-IP_pe	chr10	1.23E+08	1.23E+08	385	1.23E+08	9	4.46937	3.41876
Nc-1-IP_pe	chr10	1.23E+08	1.23E+08	605	1.23E+08	12	4.70082	3.20033
Nc-1-IP_pe	chr10	1.23E+08	1.23E+08	453	1.23E+08	9	3.27326	2.68684
Nc-1-IP_pe	chr10	1.23E+08	1.23E+08	281	1.23E+08	11	6.03439	4.10251
Nc-1-IP_pe	chr10	1.23E+08	1.23E+08	507	1.23E+08	9	4.46937	3.41876
Nc-1-IP_pe	chr10	1.23E+08	1.23E+08	506	1.23E+08	8	3.74505	3.07688
Nc-1-IP_pe	chr10	1.23E+08	1.23E+08	303	1.23E+08	12	6.86906	4.44438
Nc-1-IP_pe	chr10	1.23E+08	1.23E+08	429	1.23E+08	11	5.10577	3.5486
Nc-1-IP_pe	chr10	1.23E+08	1.23E+08	270	1.23E+08	12	6.86906	4.44438
Nc-1-IP_pe	chr10	1.23E+08	1.23E+08	497	1.23E+08	8	3.74505	3.07688
Nc-1-IP_pe	chr10	1.23E+08	1.23E+08	258	1.23E+08	11	6.03439	4.10251
Nc-1-IP_pe	chr10	1.24E+08	1.24E+08	449	1.24E+08	8	3.64414	3.01254
Nc-1-IP_pe	chr10	1.24E+08	1.24E+08	329	1.24E+08	9	4.46937	3.41876
Nc-1-IP_pe	chr10	1.24E+08	1.24E+08	491	1.24E+08	8	3.74505	3.07688
Nc-1-IP_pe	chr10	1.24E+08	1.24E+08	415	1.24E+08	8	3.74505	3.07688
Nc-1-IP_pe	chr10	1.24E+08	1.24E+08	447	1.24E+08	8	3.74505	3.07688
Nc-1-IP_pe	chr10	1.24E+08	1.24E+08	354	1.24E+08	8	3.56021	2.95918
Nc-1-IP_pe	chr10	1.24E+08	1.24E+08	378	1.24E+08	9	4.46937	3.41876
Nc-1-IP_pe	chr10	1.24E+08	1.24E+08	357	1.24E+08	10	4.39571	3.25288
Nc-1-IP_pe	chr10	1.24E+08	1.24E+08	414	1.24E+08	10	5.23357	3.76063
Nc-1-IP_pe	chr10	1.24E+08	1.24E+08	358	1.24E+08	10	4.93609	3.57849
Nc-1-IP_pe	chr10	1.24E+08	1.24E+08	521	1.24E+08	14	8.29543	4.93196
Nc-1-IP_pe	chr10	1.24E+08	1.24E+08	644	1.24E+08	8	3.08755	2.66145
Nc-1-IP_pe	chr10	1.24E+08	1.24E+08	352	1.24E+08	12	6.86906	4.44438
Nc-1-IP_pe	chr10	1.24E+08	1.24E+08	294	1.24E+08	9	4.46937	3.41876
Nc-1-IP_pe	chr10	1.24E+08	1.24E+08	817	1.24E+08	11	5.10577	3.5486
Nc-1-IP_pe	chr10	1.24E+08	1.24E+08	383	1.24E+08	11	6.03439	4.10251
Nc-1-IP_pe	chr10	1.24E+08	1.24E+08	493	1.24E+08	11	5.3563	3.696
Nc-1-IP_pe	chr10	1.24E+08	1.24E+08	1161	1.24E+08	11	6.03439	4.10251
Nc-1-IP_pe	chr10	1.24E+08	1.24E+08	312	1.24E+08	8	3.74505	3.07688
Nc-1-IP_pe	chr10	1.24E+08	1.24E+08	517	1.24E+08	12	6.58417	4.27436

Nc-1-IP_pe	chr10	1.24E+08	1.24E+08	276	1.24E+08	8	3.74505	3.07688
Nc-1-IP_pe	chr10	1.24E+08	1.24E+08	304	1.24E+08	9	4.25966	3.28797
Nc-1-IP_pe	chr10	1.24E+08	1.24E+08	326	1.24E+08	10	5.23357	3.76063
Nc-1-IP_pe	chr10	1.24E+08	1.24E+08	254	1.24E+08	11	5.7051	3.90381
Nc-1-IP_pe	chr10	1.24E+08	1.24E+08	575	1.24E+08	10	5.23357	3.76063
Nc-1-IP_pe	chr10	1.24E+08	1.24E+08	259	1.24E+08	11	6.03439	4.10251
Nc-1-IP_pe	chr10	1.24E+08	1.24E+08	346	1.24E+08	7	2.93007	2.64719
Nc-1-IP_pe	chr10	1.24E+08	1.24E+08	394	1.24E+08	11	5.52654	3.79706
Nc-1-IP_pe	chr10	1.24E+08	1.24E+08	461	1.24E+08	7	3.06442	2.73501
Nc-1-IP_pe	chr10	1.24E+08	1.24E+08	278	1.24E+08	10	5.10552	3.682
Nc-1-IP_pe	chr10	1.24E+08	1.24E+08	469	1.24E+08	13	7.7352	4.78626
Nc-1-IP_pe	chr10	1.24E+08	1.24E+08	265	1.24E+08	11	6.03439	4.10251
Nc-1-IP_pe	chr10	1.24E+08	1.24E+08	297	1.24E+08	8	3.74505	3.07688
Nc-1-IP_pe	chr10	1.24E+08	1.24E+08	427	1.24E+08	11	6.03439	4.10251
Nc-1-IP_pe	chr10	1.24E+08	1.24E+08	448	1.24E+08	12	5.23082	3.49289
Nc-1-IP_pe	chr10	1.24E+08	1.24E+08	657	1.24E+08	12	6.86906	4.44438
Nc-1-IP_pe	chr10	1.24E+08	1.24E+08	640	1.24E+08	8	3.74505	3.07688
Nc-1-IP_pe	chr10	1.24E+08	1.24E+08	582	1.24E+08	11	4.54404	3.2242
Nc-1-IP_pe	chr10	1.24E+08	1.24E+08	798	1.24E+08	11	5.7051	3.90381
Nc-1-IP_pe	chr10	1.24E+08	1.24E+08	298	1.24E+08	8	3.74505	3.07688
Nc-1-IP_pe	chr10	1.24E+08	1.24E+08	282	1.24E+08	11	5.10577	3.5486
Nc-1-IP_pe	chr10	1.24E+08	1.24E+08	609	1.24E+08	9	3.72192	2.95716
Nc-1-IP_pe	chr10	1.24E+08	1.24E+08	310	1.24E+08	12	5.84943	3.84431
Nc-1-IP_pe	chr10	1.24E+08	1.24E+08	255	1.24E+08	11	4.47871	3.18705
Nc-1-IP_pe	chr10	1.24E+08	1.24E+08	381	1.24E+08	10	5.23357	3.76063
Nc-1-IP_pe	chr10	1.25E+08	1.25E+08	282	1.25E+08	8	3.38431	2.8478
Nc-1-IP_pe	chr10	1.25E+08	1.25E+08	261	1.25E+08	9	4.25966	3.28797
Nc-1-IP_pe	chr10	1.25E+08	1.25E+08	256	1.25E+08	12	6.58417	4.27436
Nc-1-IP_pe	chr10	1.25E+08	1.25E+08	438	1.25E+08	8	3.58997	2.97809
Nc-1-IP_pe	chr10	1.25E+08	1.25E+08	319	1.25E+08	9	3.27326	2.68684
Nc-1-IP_pe	chr10	1.25E+08	1.25E+08	362	1.25E+08	9	4.46937	3.41876
Nc-1-IP_pe	chr10	1.25E+08	1.25E+08	256	1.25E+08	11	4.54404	3.2242
Nc-1-IP_pe	chr10	1.25E+08	1.25E+08	596	1.25E+08	12	5.61153	3.70794
Nc-1-IP_pe	chr10	1.25E+08	1.25E+08	456	1.25E+08	7	3.06442	2.73501
Nc-1-IP_pe	chr10	1.25E+08	1.25E+08	518	1.25E+08	10	5.23357	3.76063
Nc-1-IP_pe	chr10	1.25E+08	1.25E+08	555	1.25E+08	9	3.72192	2.95716
Nc-1-IP_pe	chr10	1.25E+08	1.25E+08	312	1.25E+08	9	4.46937	3.41876
Nc-1-IP_pe	chr10	1.25E+08	1.25E+08	662	1.25E+08	12	6.86906	4.44438
Nc-1-IP_pe	chr10	1.25E+08	1.25E+08	413	1.25E+08	8	3.53695	2.94441
Nc-1-IP_pe	chr10	1.25E+08	1.25E+08	296	1.25E+08	8	3.51085	2.92785
Nc-1-IP_pe	chr10	1.25E+08	1.25E+08	540	1.25E+08	10	4.91962	3.56846
Nc-1-IP_pe	chr10	1.25E+08	1.25E+08	276	1.25E+08	11	4.06403	2.95415
Nc-1-IP_pe	chr10	1.25E+08	1.25E+08	281	1.25E+08	15	7.46994	4.29894
Nc-1-IP_pe	chr10	1.25E+08	1.25E+08	298	1.25E+08	10	5.10552	3.682
Nc-1-IP_pe	chr10	1.25E+08	1.25E+08	280	1.25E+08	8	3.74505	3.07688

Nc-1-IP_pe	chr10	1.25E+08	1.25E+08	264	1.25E+08	13	7.42513	4.60316
Nc-1-IP_pe	chr10	1.25E+08	1.25E+08	585	1.25E+08	13	7.42513	4.60316
Nc-1-IP_pe	chr10	1.25E+08	1.25E+08	316	1.25E+08	11	5.54401	3.80747
Nc-1-IP_pe	chr10	1.25E+08	1.25E+08	581	1.25E+08	10	5.23357	3.76063
Nc-1-IP_pe	chr10	1.25E+08	1.25E+08	260	1.25E+08	9	4.46937	3.41876
Nc-1-IP_pe	chr10	1.25E+08	1.25E+08	418	1.25E+08	13	6.62436	4.14003
Nc-1-IP_pe	chr10	1.25E+08	1.25E+08	411	1.25E+08	8	3.07244	2.65201
Nc-1-IP_pe	chr10	1.25E+08	1.25E+08	383	1.25E+08	9	3.72192	2.95716
Nc-1-IP_pe	chr10	1.25E+08	1.25E+08	340	1.25E+08	8	3.67166	3.03007
Nc-1-IP_pe	chr10	1.25E+08	1.25E+08	257	1.25E+08	8	3.74505	3.07688
Nc-1-IP_pe	chr10	1.25E+08	1.25E+08	535	1.25E+08	12	6.86906	4.44438
Nc-1-IP_pe	chr10	1.25E+08	1.25E+08	266	1.25E+08	12	3.83688	2.74114
Nc-1-IP_pe	chr10	1.25E+08	1.25E+08	645	1.25E+08	8	3.56021	2.95918
Nc-1-IP_pe	chr10	1.25E+08	1.25E+08	427	1.25E+08	11	5.77463	3.94557
Nc-1-IP_pe	chr10	1.25E+08	1.25E+08	313	1.25E+08	9	4.46937	3.41876
Nc-1-IP_pe	chr10	1.25E+08	1.25E+08	623	1.25E+08	13	5.36853	3.44651
Nc-1-IP_pe	chr10	1.25E+08	1.25E+08	513	1.25E+08	16	6.8987	3.86161
Nc-1-IP_pe	chr10	1.25E+08	1.25E+08	441	1.25E+08	9	4.46937	3.41876
Nc-1-IP_pe	chr10	1.25E+08	1.25E+08	270	1.25E+08	10	5.23357	3.76063
Nc-1-IP_pe	chr10	1.25E+08	1.25E+08	305	1.25E+08	8	3.74505	3.07688
Nc-1-IP_pe	chr10	1.25E+08	1.25E+08	263	1.25E+08	8	3.74505	3.07688
Nc-1-IP_pe	chr10	1.25E+08	1.25E+08	271	1.25E+08	8	3.74505	3.07688
Nc-1-IP_pe	chr10	1.25E+08	1.25E+08	387	1.25E+08	10	4.56202	3.35231
Nc-1-IP_pe	chr10	1.25E+08	1.25E+08	632	1.25E+08	11	6.03439	4.10251
Nc-1-IP_pe	chr10	1.25E+08	1.25E+08	593	1.25E+08	9	4.33944	3.33761
Nc-1-IP_pe	chr10	1.25E+08	1.25E+08	434	1.25E+08	8	3.08755	2.66145
Nc-1-IP_pe	chr10	1.25E+08	1.25E+08	298	1.25E+08	11	4.06403	2.95415
Nc-1-IP_pe	chr10	1.25E+08	1.25E+08	274	1.25E+08	10	5.23357	3.76063
Nc-1-IP_pe	chr10	1.25E+08	1.25E+08	490	1.25E+08	10	4.39571	3.25288
Nc-1-IP_pe	chr10	1.25E+08	1.25E+08	472	1.25E+08	13	7.7352	4.78626
Nc-1-IP_pe	chr10	1.25E+08	1.25E+08	725	1.25E+08	12	5.61153	3.70794
Nc-1-IP_pe	chr10	1.25E+08	1.25E+08	678	1.25E+08	8	3.7135	3.05675
Nc-1-IP_pe	chr10	1.25E+08	1.25E+08	261	1.25E+08	11	6.03439	4.10251
Nc-1-IP_pe	chr10	1.25E+08	1.25E+08	330	1.25E+08	8	3.74505	3.07688
Nc-1-IP_pe	chr10	1.25E+08	1.25E+08	450	1.25E+08	8	3.74505	3.07688
Nc-1-IP_pe	chr10	1.25E+08	1.25E+08	453	1.25E+08	10	3.89064	2.95552
Nc-1-IP_pe	chr10	1.25E+08	1.25E+08	575	1.25E+08	10	5.23357	3.76063
Nc-1-IP_pe	chr10	1.25E+08	1.25E+08	256	1.25E+08	11	5.3563	3.696
Nc-1-IP_pe	chr10	1.25E+08	1.25E+08	303	1.25E+08	14	6.69566	4.03025
Nc-1-IP_pe	chr10	1.25E+08	1.25E+08	679	1.25E+08	9	4.46937	3.41876
Nc-1-IP_pe	chr10	1.25E+08	1.25E+08	283	1.25E+08	10	5.23357	3.76063
Nc-1-IP_pe	chr10	1.25E+08	1.25E+08	325	1.25E+08	11	5.77463	3.94557
Nc-1-IP_pe	chr10	1.25E+08	1.25E+08	1034	1.25E+08	10	5.23357	3.76063
Nc-1-IP_pe	chr10	1.25E+08	1.25E+08	310	1.25E+08	10	5.23357	3.76063
Nc-1-IP_pe	chr10	1.25E+08	1.25E+08	446	1.25E+08	10	5.23357	3.76063

Nc-1-IP_pe	chr10	1.25E+08	1.25E+08	353	1.25E+08	11	6.03439	4.10251
Nc-1-IP_pe	chr10	1.25E+08	1.25E+08	400	1.25E+08	13	7.7352	4.78626
Nc-1-IP_pe	chr10	1.25E+08	1.25E+08	263	1.25E+08	8	3.74505	3.07688
Nc-1-IP_pe	chr10	1.26E+08	1.26E+08	275	1.26E+08	10	5.23357	3.76063
Nc-1-IP_pe	chr10	1.26E+08	1.26E+08	384	1.26E+08	13	7.7352	4.78626
Nc-1-IP_pe	chr10	1.26E+08	1.26E+08	332	1.26E+08	11	6.03439	4.10251
Nc-1-IP_pe	chr10	1.26E+08	1.26E+08	316	1.26E+08	12	5.84943	3.84431
Nc-1-IP_pe	chr10	1.26E+08	1.26E+08	395	1.26E+08	10	5.23357	3.76063
Nc-1-IP_pe	chr10	1.26E+08	1.26E+08	355	1.26E+08	11	6.03439	4.10251
Nc-1-IP_pe	chr10	1.26E+08	1.26E+08	313	1.26E+08	12	6.58417	4.27436
Nc-1-IP_pe	chr10	1.26E+08	1.26E+08	287	1.26E+08	10	4.99888	3.61677
Nc-1-IP_pe	chr10	1.26E+08	1.26E+08	406	1.26E+08	15	9.55381	5.47001
Nc-1-IP_pe	chr10	1.26E+08	1.26E+08	268	1.26E+08	8	3.74505	3.07688
Nc-1-IP_pe	chr10	1.26E+08	1.26E+08	372	1.26E+08	11	5.77463	3.94557
Nc-1-IP_pe	chr10	1.26E+08	1.26E+08	305	1.26E+08	11	6.03439	4.10251
Nc-1-IP_pe	chr10	1.26E+08	1.26E+08	317	1.26E+08	9	2.89249	2.46179
Nc-1-IP_pe	chr10	1.26E+08	1.26E+08	522	1.26E+08	8	3.74505	3.07688
Nc-1-IP_pe	chr10	1.26E+08	1.26E+08	353	1.26E+08	12	6.58417	4.27436
Nc-1-IP_pe	chr10	1.26E+08	1.26E+08	363	1.26E+08	15	6.69889	3.8921
Nc-1-IP_pe	chr10	1.26E+08	1.26E+08	259	1.26E+08	12	4.60645	3.1491
Nc-1-IP_pe	chr10	1.26E+08	1.26E+08	1009	1.26E+08	33	11.8946	4.00703
Nc-1-IP_pe	chr10	1.26E+08	1.26E+08	362	1.26E+08	9	4.25966	3.28797
Nc-1-IP_pe	chr10	1.26E+08	1.26E+08	516	1.26E+08	10	4.99888	3.61677
Nc-1-IP_pe	chr10	1.26E+08	1.26E+08	311	1.26E+08	11	6.03439	4.10251
Nc-1-IP_pe	chr10	1.26E+08	1.26E+08	472	1.26E+08	13	5.9487	3.76157
Nc-1-IP_pe	chr10	1.26E+08	1.26E+08	579	1.26E+08	11	4.74697	3.34038
Nc-1-IP_pe	chr10	1.26E+08	1.26E+08	291	1.26E+08	9	4.46937	3.41876
Nc-1-IP_pe	chr10	1.26E+08	1.26E+08	352	1.26E+08	9	4.46937	3.41876
Nc-1-IP_pe	chr10	1.26E+08	1.26E+08	668	1.26E+08	14	7.42849	4.43574
Nc-1-IP_pe	chr10	1.26E+08	1.26E+08	480	1.26E+08	7	3.06442	2.73501
Nc-1-IP_pe	chr10	1.26E+08	1.26E+08	262	1.26E+08	12	4.70082	3.20033
Nc-1-IP_pe	chr10	1.26E+08	1.26E+08	311	1.26E+08	9	4.46937	3.41876
Nc-1-IP_pe	chr10	1.26E+08	1.26E+08	502	1.26E+08	14	7.54286	4.50023
Nc-1-IP_pe	chr10	1.26E+08	1.26E+08	315	1.26E+08	13	4.67336	3.0817
Nc-1-IP_pe	chr10	1.26E+08	1.26E+08	335	1.26E+08	10	5.23357	3.76063
Nc-1-IP_pe	chr10	1.26E+08	1.26E+08	589	1.26E+08	12	6.86906	4.44438
Nc-1-IP_pe	chr10	1.27E+08	1.27E+08	284	1.27E+08	10	4.39571	3.25288
Nc-1-IP_pe	chr10	1.27E+08	1.27E+08	321	1.27E+08	14	6.69566	4.03025
Nc-1-IP_pe	chr10	1.27E+08	1.27E+08	294	1.27E+08	10	4.39571	3.25288
Nc-1-IP_pe	chr10	1.27E+08	1.27E+08	300	1.27E+08	8	3.74505	3.07688
Nc-1-IP_pe	chr10	1.27E+08	1.27E+08	255	1.27E+08	9	3.27326	2.68684
Nc-1-IP_pe	chr10	1.27E+08	1.27E+08	359	1.27E+08	8	3.56021	2.95918
Nc-1-IP_pe	chr10	1.27E+08	1.27E+08	264	1.27E+08	14	8.63073	5.12814
Nc-1-IP_pe	chr10	1.27E+08	1.27E+08	785	1.27E+08	36	3.21524	1.72785
Nc-1-IP_pe	chr10	1.27E+08	1.27E+08	494	1.27E+08	10	5.23357	3.76063

Nc-1-IP_pe	chr10	1.27E+08	1.27E+08	262	1.27E+08	10	5.23357	3.76063
Nc-1-IP_pe	chr10	1.27E+08	1.27E+08	277	1.27E+08	10	5.23357	3.76063
Nc-1-IP_pe	chr10	1.27E+08	1.27E+08	413	1.27E+08	9	4.46937	3.41876
Nc-1-IP_pe	chr10	1.27E+08	1.27E+08	336	1.27E+08	9	4.46937	3.41876
Nc-1-IP_pe	chr10	1.27E+08	1.27E+08	369	1.27E+08	10	4.93609	3.57849
Nc-1-IP_pe	chr10	1.27E+08	1.27E+08	272	1.27E+08	11	5.10577	3.5486
Nc-1-IP_pe	chr10	1.27E+08	1.27E+08	363	1.27E+08	9	4.46937	3.41876
Nc-1-IP_pe	chr10	1.27E+08	1.27E+08	675	1.27E+08	10	5.23357	3.76063
Nc-1-IP_pe	chr10	1.27E+08	1.27E+08	261	1.27E+08	9	4.20361	3.25317
Nc-1-IP_pe	chr10	1.27E+08	1.27E+08	373	1.27E+08	8	3.74505	3.07688
Nc-1-IP_pe	chr10	1.27E+08	1.27E+08	494	1.27E+08	16	6.8987	3.86161
Nc-1-IP_pe	chr10	1.27E+08	1.27E+08	260	1.27E+08	9	4.46937	3.41876
Nc-1-IP_pe	chr10	1.27E+08	1.27E+08	715	1.27E+08	10	5.23357	3.76063
Nc-1-IP_pe	chr10	1.27E+08	1.27E+08	380	1.27E+08	11	6.03439	4.10251
Nc-1-IP_pe	chr10	1.28E+08	1.28E+08	693	1.28E+08	12	4.24064	2.95299
Nc-1-IP_pe	chr10	1.28E+08	1.28E+08	285	1.28E+08	8	3.74505	3.07688
Nc-1-IP_pe	chr10	1.28E+08	1.28E+08	278	1.28E+08	8	3.74505	3.07688
Nc-1-IP_pe	chr10	1.28E+08	1.28E+08	551	1.28E+08	8	3.56021	2.95918
Nc-1-IP_pe	chr10	1.28E+08	1.28E+08	264	1.28E+08	10	5.23357	3.76063
Nc-1-IP_pe	chr10	1.28E+08	1.28E+08	279	1.28E+08	10	3.08907	2.49869
Nc-1-IP_pe	chr10	1.28E+08	1.28E+08	443	1.28E+08	9	4.46937	3.41876
Nc-1-IP_pe	chr10	1.28E+08	1.28E+08	584	1.28E+08	10	5.10552	3.682
Nc-1-IP_pe	chr10	1.28E+08	1.28E+08	669	1.28E+08	10	3.89064	2.95552
Nc-1-IP_pe	chr10	1.28E+08	1.28E+08	291	1.28E+08	10	4.99888	3.61677
Nc-1-IP_pe	chr10	1.28E+08	1.28E+08	304	1.28E+08	9	4.25966	3.28797
Nc-1-IP_pe	chr10	1.28E+08	1.28E+08	567	1.28E+08	8	3.56021	2.95918
Nc-1-IP_pe	chr10	1.28E+08	1.28E+08	376	1.28E+08	10	3.08907	2.49869
Nc-1-IP_pe	chr10	1.28E+08	1.28E+08	607	1.28E+08	10	5.23357	3.76063
Nc-1-IP_pe	chr10	1.28E+08	1.28E+08	346	1.28E+08	12	6.86906	4.44438
Nc-1-IP_pe	chr10	1.28E+08	1.28E+08	303	1.28E+08	9	4.38615	3.36674
Nc-1-IP_pe	chr10	1.28E+08	1.28E+08	305	1.28E+08	10	4.39571	3.25288
Nc-1-IP_pe	chr10	1.28E+08	1.28E+08	497	1.28E+08	9	3.27326	2.68684
Nc-1-IP_pe	chr10	1.28E+08	1.28E+08	263	1.28E+08	8	3.74505	3.07688
Nc-1-IP_pe	chr10	1.28E+08	1.28E+08	636	1.28E+08	12	6.86906	4.44438
Nc-1-IP_pe	chr10	1.28E+08	1.28E+08	532	1.28E+08	15	9.19325	5.26076
Nc-1-IP_pe	chr10	1.28E+08	1.28E+08	270	1.28E+08	10	4.39571	3.25288
Nc-1-IP_pe	chr10	1.29E+08	1.29E+08	312	1.29E+08	7	2.9043	2.63038
Nc-1-IP_pe	chr10	1.29E+08	1.29E+08	267	1.29E+08	8	3.74505	3.07688
Nc-1-IP_pe	chr10	1.29E+08	1.29E+08	344	1.29E+08	9	4.46937	3.41876
Nc-1-IP_pe	chr10	1.29E+08	1.29E+08	325	1.29E+08	8	3.74505	3.07688
Nc-1-IP_pe	chr10	1.29E+08	1.29E+08	273	1.29E+08	8	3.56021	2.95918
Nc-1-IP_pe	chr10	1.29E+08	1.29E+08	398	1.29E+08	10	5.23357	3.76063
Nc-1-IP_pe	chr10	1.29E+08	1.29E+08	299	1.29E+08	9	4.25966	3.28797
Nc-1-IP_pe	chr10	1.29E+08	1.29E+08	290	1.29E+08	10	5.23357	3.76063
Nc-1-IP_pe	chr10	1.29E+08	1.29E+08	379	1.29E+08	11	5.77463	3.94557

Nc-1-IP_pe	chr10	1.29E+08	1.29E+08	260	1.29E+08	8	3.74505	3.07688
Nc-1-IP_pe	chr10	1.29E+08	1.29E+08	406	1.29E+08	11	6.03439	4.10251
Nc-1-IP_pe	chr10	1.29E+08	1.29E+08	457	1.29E+08	10	4.99888	3.61677
Nc-1-IP_pe	chr10	1.29E+08	1.29E+08	325	1.29E+08	8	3.74505	3.07688
Nc-1-IP_pe	chr10	1.3E+08	1.3E+08	370	1.3E+08	10	4.99888	3.61677
Nc-1-IP_pe	chr10	1.3E+08	1.3E+08	257	1.3E+08	8	3.14944	2.70014
Nc-1-IP_pe	chr10	1.3E+08	1.3E+08	394	1.3E+08	11	5.77463	3.94557
Nc-1-IP_pe	chr10	1.3E+08	1.3E+08	253	1.3E+08	11	4.54404	3.2242
Nc-1-IP_pe	chr10	1.3E+08	1.3E+08	668	1.3E+08	10	4.99888	3.61677
Nc-1-IP_pe	chr10	1.3E+08	1.3E+08	587	1.3E+08	11	5.77463	3.94557
Nc-1-IP_pe	chr10	1.3E+08	1.3E+08	257	1.3E+08	13	7.7352	4.78626
Nc-1-IP_pe	chr10	1.3E+08	1.3E+08	523	1.3E+08	9	3.66792	2.92434
Nc-1-IP_pe	chr10	1.3E+08	1.3E+08	326	1.3E+08	12	6.71369	4.35145
Nc-1-IP_pe	chr10	1.3E+08	1.3E+08	316	1.3E+08	10	4.99888	3.61677
Nc-1-IP_pe	chr10	1.3E+08	1.3E+08	467	1.3E+08	8	3.74505	3.07688
Nc-1-IP_pe	chr10	1.3E+08	1.3E+08	318	1.3E+08	8	3.74505	3.07688
Nc-1-IP_pe	chr10	1.3E+08	1.3E+08	363	1.3E+08	10	4.99888	3.61677
Nc-1-IP_pe	chr10	1.3E+08	1.3E+08	256	1.3E+08	8	3.74505	3.07688
Nc-1-IP_pe	chr10	1.3E+08	1.3E+08	344	1.3E+08	10	4.99888	3.61677
Nc-1-IP_pe	chr10	1.3E+08	1.3E+08	254	1.3E+08	9	4.46937	3.41876
Nc-1-IP_pe	chr10	1.3E+08	1.3E+08	287	1.3E+08	9	4.46937	3.41876
Nc-1-IP_pe	chr10	1.3E+08	1.3E+08	386	1.3E+08	13	6.62436	4.14003
Nc-1-IP_pe	chr10	1.3E+08	1.3E+08	402	1.3E+08	10	5.23357	3.76063
Nc-1-IP_pe	chr10	1.3E+08	1.3E+08	305	1.3E+08	12	6.86906	4.44438
Nc-1-IP_pe	chr10	1.3E+08	1.3E+08	463	1.3E+08	8	3.74505	3.07688
Nc-1-IP_pe	chr10	1.3E+08	1.3E+08	282	1.3E+08	11	6.03439	4.10251
Nc-1-IP_pe	chr10	1.3E+08	1.3E+08	292	1.3E+08	7	3.06442	2.73501
Nc-1-IP_pe	chr10	1.3E+08	1.3E+08	678	1.3E+08	11	6.03439	4.10251
Nc-1-IP_pe	chr10	1.3E+08	1.3E+08	291	1.3E+08	9	4.25966	3.28797
Nc-1-IP_pe	chr10	1.3E+08	1.3E+08	274	1.3E+08	11	6.03439	4.10251
Nc-1-IP_pe	chr10	1.3E+08	1.3E+08	822	1.3E+08	12	6.86906	4.44438
Nc-1-IP_pe	chr10	1.3E+08	1.3E+08	794	1.3E+08	13	6.02837	3.80557
Nc-1-IP_pe	chr10	1.3E+08	1.3E+08	308	1.3E+08	12	6.86906	4.44438
Nc-1-IP_pe	chr10	1.3E+08	1.3E+08	457	1.3E+08	12	4.70082	3.20033
Nc-1-IP_pe	chr10	1.3E+08	1.3E+08	273	1.3E+08	9	4.46937	3.41876
Nc-1-IP_pe	chr10	1.3E+08	1.3E+08	279	1.3E+08	7	2.9043	2.63038
Nc-1-IP_pe	chr10	1.3E+08	1.3E+08	313	1.3E+08	12	6.86906	4.44438
Nc-1-IP_pe	chr10	1.3E+08	1.3E+08	275	1.3E+08	9	4.46937	3.41876
Nc-1-IP_pe	chr10	1.3E+08	1.3E+08	281	1.3E+08	12	6.86906	4.44438
Nc-1-IP_pe	chr10	1.3E+08	1.3E+08	280	1.3E+08	8	3.74505	3.07688
Nc-1-IP_pe	chr10	1.3E+08	1.3E+08	357	1.3E+08	9	4.46937	3.41876
Nc-1-IP_pe	chr10	1.3E+08	1.3E+08	348	1.3E+08	9	4.46937	3.41876
Nc-1-IP_pe	chr10	1.31E+08	1.31E+08	258	1.31E+08	8	3.56021	2.95918
Nc-1-IP_pe	chr10	1.31E+08	1.31E+08	289	1.31E+08	12	6.86906	4.44438
Nc-1-IP_pe	chr10	1.31E+08	1.31E+08	450	1.31E+08	8	3.74505	3.07688

Nc-1-IP_pe	chr10	1.31E+08	1.31E+08	362	1.31E+08	11	6.03439	4.10251
Nc-1-IP_pe	chr10	1.31E+08	1.31E+08	307	1.31E+08	8	3.74505	3.07688
Nc-1-IP_pe	chr10	1.31E+08	1.31E+08	353	1.31E+08	8	3.74505	3.07688
Nc-1-IP_pe	chr10	1.31E+08	1.31E+08	303	1.31E+08	12	6.86906	4.44438
Nc-1-IP_pe	chr10	1.31E+08	1.31E+08	261	1.31E+08	12	6.86906	4.44438
Nc-1-IP_pe	chr10	1.31E+08	1.31E+08	283	1.31E+08	10	5.23357	3.76063
Nc-1-IP_pe	chr10	1.31E+08	1.31E+08	452	1.31E+08	11	6.03439	4.10251
Nc-1-IP_pe	chr10	1.31E+08	1.31E+08	346	1.31E+08	8	3.74505	3.07688
Nc-1-IP_pe	chr10	1.31E+08	1.31E+08	528	1.31E+08	11	6.03439	4.10251
Nc-1-IP_pe	chr10	1.31E+08	1.31E+08	268	1.31E+08	8	3.74505	3.07688
Nc-1-IP_pe	chr10	1.31E+08	1.31E+08	356	1.31E+08	9	4.46937	3.41876
Nc-1-IP_pe	chr10	1.31E+08	1.31E+08	505	1.31E+08	13	5.36853	3.44651
Nc-1-IP_pe	chr10	1.31E+08	1.31E+08	529	1.31E+08	8	3.74505	3.07688
Nc-1-IP_pe	chr10	1.31E+08	1.31E+08	514	1.31E+08	10	5.23357	3.76063
Nc-1-IP_pe	chr10	1.31E+08	1.31E+08	591	1.31E+08	9	4.46937	3.41876
Nc-1-IP_pe	chr10	1.31E+08	1.31E+08	281	1.31E+08	10	4.39571	3.25288
Nc-1-IP_pe	chr10	1.31E+08	1.31E+08	446	1.31E+08	10	5.23357	3.76063
Nc-1-IP_pe	chr10	1.31E+08	1.31E+08	311	1.31E+08	11	6.03439	4.10251
Nc-1-IP_pe	chr10	1.31E+08	1.31E+08	257	1.31E+08	9	4.46937	3.41876
Nc-1-IP_pe	chr10	1.31E+08	1.31E+08	293	1.31E+08	7	3.06442	2.73501
Nc-1-IP_pe	chr10	1.31E+08	1.31E+08	326	1.31E+08	10	5.23357	3.76063
Nc-1-IP_pe	chr10	1.31E+08	1.31E+08	452	1.31E+08	9	4.46937	3.41876
Nc-1-IP_pe	chr10	1.31E+08	1.31E+08	575	1.31E+08	9	4.46937	3.41876
Nc-1-IP_pe	chr10	1.31E+08	1.31E+08	292	1.31E+08	9	4.25966	3.28797
Nc-1-IP_pe	chr10	1.32E+08	1.32E+08	310	1.32E+08	11	4.54404	3.2242
Nc-1-IP_pe	chr10	1.32E+08	1.32E+08	508	1.32E+08	11	6.03439	4.10251
Nc-1-IP_pe	chr10	1.32E+08	1.32E+08	287	1.32E+08	12	6.86906	4.44438
Nc-1-IP_pe	chr10	1.32E+08	1.32E+08	281	1.32E+08	11	6.03439	4.10251
Nc-1-IP_pe	chr10	1.32E+08	1.32E+08	309	1.32E+08	10	4.62142	3.388
Nc-1-IP_pe	chr10	1.32E+08	1.32E+08	321	1.32E+08	12	4.70082	3.20033
Nc-1-IP_pe	chr10	1.32E+08	1.32E+08	273	1.32E+08	10	5.23357	3.76063
Nc-1-IP_pe	chr10	1.32E+08	1.32E+08	298	1.32E+08	8	3.74505	3.07688
Nc-1-IP_pe	chr10	1.32E+08	1.32E+08	289	1.32E+08	12	6.86906	4.44438
Nc-1-IP_pe	chr10	1.32E+08	1.32E+08	263	1.32E+08	10	5.23357	3.76063
Nc-1-IP_pe	chr10	1.32E+08	1.32E+08	808	1.32E+08	10	4.39571	3.25288
Nc-1-IP_pe	chr10	1.32E+08	1.32E+08	720	1.32E+08	11	6.03439	4.10251
Nc-1-IP_pe	chr10	1.32E+08	1.32E+08	480	1.32E+08	9	4.46937	3.41876
Nc-1-IP_pe	chr10	1.32E+08	1.32E+08	580	1.32E+08	14	3.86727	2.6027
Nc-1-IP_pe	chr10	1.32E+08	1.32E+08	303	1.32E+08	9	4.46937	3.41876
Nc-1-IP_pe	chr10	1.32E+08	1.32E+08	278	1.32E+08	9	4.46937	3.41876
Nc-1-IP_pe	chr10	1.32E+08	1.32E+08	429	1.32E+08	10	5.23357	3.76063
Nc-1-IP_pe	chr10	1.32E+08	1.32E+08	512	1.32E+08	11	3.64839	2.72584
Nc-1-IP_pe	chr10	1.32E+08	1.32E+08	300	1.32E+08	8	3.56021	2.95918
Nc-1-IP_pe	chr10	1.32E+08	1.32E+08	263	1.32E+08	7	3.06442	2.73501
Nc-1-IP_pe	chr10	1.32E+08	1.32E+08	344	1.32E+08	10	3.4604	2.70797

Nc-1-IP_pe	chr10	1.32E+08	1.32E+08	313	1.32E+08	9	3.72192	2.95716
Nc-1-IP_pe	chr10	1.32E+08	1.32E+08	355	1.32E+08	7	2.98886	2.68558
Nc-1-IP_pe	chr10	1.32E+08	1.32E+08	793	1.32E+08	22	5.39765	2.71064
Nc-1-IP_pe	chr10	1.32E+08	1.32E+08	746	1.32E+08	9	4.46937	3.41876
Nc-1-IP_pe	chr10	1.32E+08	1.32E+08	306	1.32E+08	9	4.46937	3.41876
Nc-1-IP_pe	chr10	1.32E+08	1.32E+08	692	1.32E+08	11	6.03439	4.10251
Nc-1-IP_pe	chr10	1.32E+08	1.32E+08	296	1.32E+08	13	7.7352	4.78626
Nc-1-IP_pe	chr10	1.32E+08	1.32E+08	332	1.32E+08	9	4.46937	3.41876
Nc-1-IP_pe	chr10	1.32E+08	1.32E+08	433	1.32E+08	9	4.46937	3.41876
Nc-1-IP_pe	chr10	1.32E+08	1.32E+08	842	1.32E+08	16	7.97877	4.4137
Nc-1-IP_pe	chr10	1.32E+08	1.32E+08	258	1.32E+08	9	4.46937	3.41876
Nc-1-IP_pe	chr10	1.32E+08	1.32E+08	608	1.32E+08	10	4.07292	3.06202
Nc-1-IP_pe	chr10	1.32E+08	1.32E+08	271	1.32E+08	11	5.03835	3.5092
Nc-1-IP_pe	chr10	1.32E+08	1.32E+08	324	1.32E+08	8	3.56021	2.95918
Nc-1-IP_pe	chr10	1.32E+08	1.32E+08	311	1.32E+08	8	3.74505	3.07688
Nc-1-IP_pe	chr10	1.32E+08	1.32E+08	393	1.32E+08	8	3.74505	3.07688
Nc-1-IP_pe	chr10	1.32E+08	1.32E+08	421	1.32E+08	11	6.03439	4.10251
Nc-1-IP_pe	chr10	1.32E+08	1.32E+08	474	1.32E+08	10	4.39571	3.25288
Nc-1-IP_pe	chr10	1.32E+08	1.32E+08	356	1.32E+08	10	4.39571	3.25288
Nc-1-IP_pe	chr10	1.32E+08	1.32E+08	281	1.32E+08	12	6.25484	4.08002
Nc-1-IP_pe	chr10	1.32E+08	1.32E+08	624	1.32E+08	10	4.98602	3.60892
Nc-1-IP_pe	chr10	1.32E+08	1.32E+08	291	1.32E+08	9	4.46937	3.41876
Nc-1-IP_pe	chr10	1.32E+08	1.32E+08	596	1.32E+08	8	3.264	2.772
Nc-1-IP_pe	chr10	1.32E+08	1.32E+08	376	1.32E+08	8	3.74505	3.07688
Nc-1-IP_pe	chr10	1.32E+08	1.32E+08	378	1.32E+08	9	3.27326	2.68684
Nc-1-IP_pe	chr10	1.32E+08	1.32E+08	350	1.32E+08	8	3.56021	2.95918
Nc-1-IP_pe	chr10	1.32E+08	1.32E+08	349	1.32E+08	9	4.46937	3.41876
Nc-1-IP_pe	chr10	1.32E+08	1.32E+08	375	1.32E+08	10	3.89064	2.95552
Nc-1-IP_pe	chr10	1.32E+08	1.32E+08	452	1.32E+08	8	3.51085	2.92785
Nc-1-IP_pe	chr10	1.32E+08	1.32E+08	284	1.32E+08	13	4.02562	2.7544
Nc-1-IP_pe	chr10	1.32E+08	1.32E+08	395	1.32E+08	10	3.4604	2.70797
Nc-1-IP_pe	chr10	1.32E+08	1.32E+08	782	1.32E+08	12	6.86906	4.44438
Nc-1-IP_pe	chr10	1.32E+08	1.32E+08	397	1.32E+08	9	3.27326	2.68684
Nc-1-IP_pe	chr10	1.32E+08	1.32E+08	302	1.32E+08	10	3.89064	2.95552
Nc-1-IP_pe	chr10	1.32E+08	1.32E+08	450	1.32E+08	9	4.05981	3.16422
Nc-1-IP_pe	chr10	1.32E+08	1.32E+08	997	1.32E+08	9	4.46937	3.41876
Nc-1-IP_pe	chr10	1.32E+08	1.32E+08	456	1.32E+08	9	4.05981	3.16422
Nc-1-IP_pe	chr10	1.32E+08	1.32E+08	329	1.32E+08	12	5.23082	3.49289
Nc-1-IP_pe	chr10	1.32E+08	1.32E+08	481	1.32E+08	10	5.23357	3.76063
Nc-1-IP_pe	chr10	1.32E+08	1.32E+08	268	1.32E+08	10	5.23357	3.76063
Nc-1-IP_pe	chr10	1.32E+08	1.32E+08	612	1.32E+08	9	4.46937	3.41876
Nc-1-IP_pe	chr10	1.32E+08	1.32E+08	418	1.32E+08	8	3.264	2.772
Nc-1-IP_pe	chr10	1.32E+08	1.32E+08	255	1.32E+08	8	3.74505	3.07688
Nc-1-IP_pe	chr10	1.32E+08	1.32E+08	294	1.32E+08	13	6.02837	3.80557
Nc-1-IP_pe	chr10	1.32E+08	1.32E+08	573	1.32E+08	8	3.08755	2.66145

Nc-1-IP_pe	chr10	1.32E+08	1.32E+08	274	1.32E+08	11	6.03439	4.10251
Nc-1-IP_pe	chr10	1.32E+08	1.32E+08	413	1.32E+08	14	8.29543	4.93196
Nc-1-IP_pe	chr10	1.32E+08	1.32E+08	736	1.32E+08	9	4.25966	3.28797
Nc-1-IP_pe	chr10	1.32E+08	1.32E+08	274	1.32E+08	11	3.78682	2.80131
Nc-1-IP_pe	chr10	1.32E+08	1.32E+08	755	1.32E+08	10	4.91962	3.56846
Nc-1-IP_pe	chr10	1.32E+08	1.32E+08	285	1.32E+08	12	5.23082	3.49289
Nc-1-IP_pe	chr10	1.32E+08	1.32E+08	347	1.32E+08	9	2.89249	2.46179
Nc-1-IP_pe	chr10	1.32E+08	1.32E+08	287	1.32E+08	13	5.9487	3.76157
Nc-1-IP_pe	chr10	1.32E+08	1.32E+08	256	1.32E+08	7	3.06442	2.73501
Nc-1-IP_pe	chr10	1.32E+08	1.32E+08	401	1.32E+08	12	6.58417	4.27436
Nc-1-IP_pe	chr10	1.32E+08	1.32E+08	713	1.32E+08	16	9.15497	5.04791
Nc-1-IP_pe	chr10	1.32E+08	1.32E+08	651	1.32E+08	12	5.3037	3.53374
Nc-1-IP_pe	chr10	1.32E+08	1.32E+08	378	1.32E+08	13	6.92473	4.312
Nc-1-IP_pe	chr10	1.32E+08	1.32E+08	428	1.32E+08	10	3.4604	2.70797
Nc-1-IP_pe	chr10	1.32E+08	1.32E+08	319	1.32E+08	12	5.62759	3.7171
Nc-1-IP_pe	chr10	1.32E+08	1.32E+08	287	1.32E+08	14	6.13058	3.7272
Nc-1-IP_pe	chr10	1.32E+08	1.32E+08	307	1.32E+08	12	5.84275	3.84046
Nc-1-IP_pe	chr10	1.32E+08	1.32E+08	616	1.32E+08	14	6.69566	4.03025
Nc-1-IP_pe	chr10	1.32E+08	1.32E+08	674	1.32E+08	11	4.06403	2.95415
Nc-1-IP_pe	chr10	1.32E+08	1.32E+08	363	1.32E+08	12	6.86906	4.44438
Nc-1-IP_pe	chr10	1.32E+08	1.32E+08	407	1.32E+08	10	3.89064	2.95552
Nc-1-IP_pe	chr10	1.32E+08	1.32E+08	281	1.32E+08	12	4.70082	3.20033
Nc-1-IP_pe	chr10	1.32E+08	1.32E+08	1186	1.32E+08	11	5.19376	3.60018
Nc-1-IP_pe	chr10	1.32E+08	1.32E+08	747	1.32E+08	11	4.74697	3.34038
Nc-1-IP_pe	chr10	1.32E+08	1.32E+08	415	1.32E+08	12	6.36968	4.14751
Nc-1-IP_pe	chr10	1.32E+08	1.32E+08	1156	1.32E+08	10	3.60969	2.79325
Nc-1-IP_pe	chr10	1.32E+08	1.32E+08	569	1.32E+08	14	8.27703	4.92126
Nc-1-IP_pe	chr10	1.32E+08	1.32E+08	307	1.32E+08	9	4.3705	3.35698
Nc-1-IP_pe	chr10	1.32E+08	1.32E+08	396	1.32E+08	10	3.4604	2.70797
Nc-1-IP_pe	chr10	1.32E+08	1.32E+08	315	1.32E+08	14	7.42849	4.43574
Nc-1-IP_pe	chr10	1.32E+08	1.32E+08	257	1.32E+08	9	4.46937	3.41876
Nc-1-IP_pe	chr10	1.32E+08	1.32E+08	781	1.32E+08	12	4.75569	3.23024
Nc-1-IP_pe	chr10	1.32E+08	1.32E+08	767	1.32E+08	9	3.72192	2.95716
Nc-1-IP_pe	chr10	1.33E+08	1.33E+08	331	1.33E+08	8	3.56021	2.95918
Nc-1-IP_pe	chr10	1.33E+08	1.33E+08	431	1.33E+08	11	6.03439	4.10251
Nc-1-IP_pe	chr10	1.33E+08	1.33E+08	486	1.33E+08	11	5.10577	3.5486
Nc-1-IP_pe	chr10	1.33E+08	1.33E+08	342	1.33E+08	13	7.7352	4.78626
Nc-1-IP_pe	chr10	1.33E+08	1.33E+08	295	1.33E+08	8	3.45945	2.8953
Nc-1-IP_pe	chr10	1.33E+08	1.33E+08	825	1.33E+08	15	6.19428	3.63445
Nc-1-IP_pe	chr10	1.33E+08	1.33E+08	640	1.33E+08	9	4.25966	3.28797
Nc-1-IP_pe	chr10	1.33E+08	1.33E+08	721	1.33E+08	11	5.10577	3.5486
Nc-1-IP_pe	chr10	1.33E+08	1.33E+08	691	1.33E+08	8	3.74505	3.07688
Nc-1-IP_pe	chr10	1.33E+08	1.33E+08	343	1.33E+08	9	4.46937	3.41876
Nc-1-IP_pe	chr10	1.33E+08	1.33E+08	486	1.33E+08	11	4.35236	3.11555
Nc-1-IP_pe	chr10	1.33E+08	1.33E+08	253	1.33E+08	9	4.46937	3.41876

Nc-1-IP_pe	chr10	1.33E+08	1.33E+08	265	1.33E+08	10	3.89064	2.95552
Nc-1-IP_pe	chr10	1.33E+08	1.33E+08	393	1.33E+08	9	3.72192	2.95716
Nc-1-IP_pe	chr10	1.33E+08	1.33E+08	1012	1.33E+08	10	5.23357	3.76063
Nc-1-IP_pe	chr10	1.33E+08	1.33E+08	307	1.33E+08	12	5.77527	3.80164
Nc-1-IP_pe	chr10	1.33E+08	1.33E+08	631	1.33E+08	13	6.62436	4.14003
Nc-1-IP_pe	chr10	1.33E+08	1.33E+08	610	1.33E+08	9	4.25966	3.28797
Nc-1-IP_pe	chr10	1.33E+08	1.33E+08	302	1.33E+08	10	4.77495	3.48064
Nc-1-IP_pe	chr10	1.33E+08	1.33E+08	408	1.33E+08	9	4.46937	3.41876
Nc-1-IP_pe	chr10	1.33E+08	1.33E+08	729	1.33E+08	12	6.58417	4.27436
Nc-1-IP_pe	chr10	1.33E+08	1.33E+08	498	1.33E+08	9	3.72192	2.95716
Nc-1-IP_pe	chr10	1.33E+08	1.33E+08	749	1.33E+08	9	4.25966	3.28797
Nc-1-IP_pe	chr10	1.33E+08	1.33E+08	588	1.33E+08	13	7.7352	4.78626
Nc-1-IP_pe	chr10	1.33E+08	1.33E+08	266	1.33E+08	7	3.06442	2.73501
Nc-1-IP_pe	chr10	1.33E+08	1.33E+08	919	1.33E+08	11	5.3563	3.696
Nc-1-IP_pe	chr10	1.33E+08	1.33E+08	431	1.33E+08	9	4.46937	3.41876
Nc-1-IP_pe	chr10	1.33E+08	1.33E+08	985	1.33E+08	8	3.51085	2.92785
Nc-1-IP_pe	chr10	1.33E+08	1.33E+08	382	1.33E+08	12	6.86906	4.44438
Nc-1-IP_pe	chr10	1.33E+08	1.33E+08	506	1.33E+08	9	4.35492	3.34727
Nc-1-IP_pe	chr10	1.33E+08	1.33E+08	561	1.33E+08	12	6.31185	4.11348
Nc-1-IP_pe	chr10	1.33E+08	1.33E+08	542	1.33E+08	11	5.10577	3.5486
Nc-1-IP_pe	chr10	1.33E+08	1.33E+08	1108	1.33E+08	11	6.03439	4.10251
Nc-1-IP_pe	chr10	1.33E+08	1.33E+08	362	1.33E+08	11	4.54404	3.2242
Nc-1-IP_pe	chr10	1.33E+08	1.33E+08	404	1.33E+08	12	6.50787	4.22912
Nc-1-IP_pe	chr10	1.33E+08	1.33E+08	262	1.33E+08	10	5.23357	3.76063
Nc-1-IP_pe	chr10	1.33E+08	1.33E+08	257	1.33E+08	12	6.86906	4.44438
Nc-1-IP_pe	chr10	1.33E+08	1.33E+08	351	1.33E+08	9	4.4496	3.40639
Nc-1-IP_pe	chr10	1.33E+08	1.33E+08	567	1.33E+08	9	4.20361	3.25317
Nc-1-IP_pe	chr10	1.33E+08	1.33E+08	301	1.33E+08	9	4.46937	3.41876
Nc-1-IP_pe	chr10	1.33E+08	1.33E+08	883	1.33E+08	11	5.10577	3.5486
Nc-1-IP_pe	chr10	1.33E+08	1.33E+08	287	1.33E+08	9	4.46937	3.41876
Nc-1-IP_pe	chr10	1.33E+08	1.33E+08	544	1.33E+08	10	3.89064	2.95552
Nc-1-IP_pe	chr10	1.33E+08	1.33E+08	575	1.33E+08	11	6.03439	4.10251
Nc-1-IP_pe	chr10	1.33E+08	1.33E+08	325	1.33E+08	11	6.03439	4.10251
Nc-1-IP_pe	chr10	1.33E+08	1.33E+08	280	1.33E+08	7	3.06442	2.73501
Nc-1-IP_pe	chr10	1.33E+08	1.33E+08	494	1.33E+08	10	5.23357	3.76063
Nc-1-IP_pe	chr10	1.33E+08	1.33E+08	457	1.33E+08	10	5.23357	3.76063
Nc-1-IP_pe	chr10	1.33E+08	1.33E+08	393	1.33E+08	10	4.99888	3.61677
Nc-1-IP_pe	chr10	1.33E+08	1.33E+08	284	1.33E+08	10	3.4604	2.70797
Nc-1-IP_pe	chr10	1.33E+08	1.33E+08	740	1.33E+08	8	3.74505	3.07688
Nc-1-IP_pe	chr10	1.33E+08	1.33E+08	287	1.33E+08	8	3.74505	3.07688
Nc-1-IP_pe	chr10	1.33E+08	1.33E+08	577	1.33E+08	9	2.89249	2.46179
Nc-1-IP_pe	chr10	1.33E+08	1.33E+08	271	1.33E+08	13	7.12853	4.42991
Nc-1-IP_pe	chr10	1.33E+08	1.33E+08	333	1.33E+08	10	4.99888	3.61677
Nc-1-IP_pe	chr10	1.33E+08	1.33E+08	281	1.33E+08	10	4.99888	3.61677
Nc-1-IP_pe	chr10	1.33E+08	1.33E+08	397	1.33E+08	15	9.19325	5.26076

Nc-1-IP_pe	chr10	1.33E+08	1.33E+08	574	1.33E+08	10	5.23357	3.76063
Nc-1-IP_pe	chr10	1.33E+08	1.33E+08	341	1.33E+08	12	6.86906	4.44438
Nc-1-IP_pe	chr10	1.33E+08	1.33E+08	394	1.33E+08	9	3.72192	2.95716
Nc-1-IP_pe	chr10	1.33E+08	1.33E+08	274	1.33E+08	16	10.5028	5.81189
Nc-1-IP_pe	chr10	1.33E+08	1.33E+08	434	1.33E+08	10	5.23357	3.76063
Nc-1-IP_pe	chr10	1.33E+08	1.33E+08	323	1.33E+08	12	6.7561	4.37677
Nc-1-IP_pe	chr10	1.33E+08	1.33E+08	331	1.33E+08	11	6.03439	4.10251
Nc-1-IP_pe	chr10	1.33E+08	1.33E+08	394	1.33E+08	12	5.84943	3.84431
Nc-1-IP_pe	chr10	1.33E+08	1.33E+08	279	1.33E+08	9	3.79246	3.00015
Nc-1-IP_pe	chr10	1.33E+08	1.33E+08	253	1.33E+08	8	3.56021	2.95918
Nc-1-IP_pe	chr10	1.33E+08	1.33E+08	374	1.33E+08	9	2.89249	2.46179
Nc-1-IP_pe	chr10	1.33E+08	1.33E+08	599	1.33E+08	9	4.46937	3.41876
Nc-1-IP_pe	chr10	1.33E+08	1.33E+08	342	1.33E+08	10	3.89064	2.95552
Nc-1-IP_pe	chr10	1.33E+08	1.33E+08	307	1.33E+08	11	5.52654	3.79706
Nc-1-IP_pe	chr10	1.33E+08	1.33E+08	442	1.33E+08	9	4.25966	3.28797
Nc-1-IP_pe	chr10	1.33E+08	1.33E+08	1364	1.33E+08	13	7.12853	4.42991
Nc-1-IP_pe	chr10	1.33E+08	1.33E+08	258	1.33E+08	9	4.46937	3.41876
Nc-1-IP_pe	chr10	1.33E+08	1.33E+08	293	1.33E+08	10	4.99888	3.61677
Nc-1-IP_pe	chr10	1.33E+08	1.33E+08	271	1.33E+08	8	3.74505	3.07688
Nc-1-IP_pe	chr10	1.33E+08	1.33E+08	371	1.33E+08	10	5.10552	3.682
Nc-1-IP_pe	chr10	1.33E+08	1.33E+08	285	1.33E+08	8	3.74505	3.07688
Nc-1-IP_pe	chr10	1.33E+08	1.33E+08	371	1.33E+08	8	3.74505	3.07688
Nc-1-IP_pe	chr10	1.33E+08	1.33E+08	350	1.33E+08	10	5.23357	3.76063
Nc-1-IP_pe	chr10	1.33E+08	1.33E+08	569	1.33E+08	12	6.58417	4.27436
Nc-1-IP_pe	chr10	1.33E+08	1.33E+08	468	1.33E+08	8	3.74505	3.07688
Nc-1-IP_pe	chr10	1.33E+08	1.33E+08	267	1.33E+08	11	6.03439	4.10251
Nc-1-IP_pe	chr10	1.33E+08	1.33E+08	663	1.33E+08	11	5.77463	3.94557
Nc-1-IP_pe	chr10	1.33E+08	1.33E+08	890	1.33E+08	11	5.77463	3.94557
Nc-1-IP_pe	chr10	1.33E+08	1.33E+08	303	1.33E+08	10	5.00283	3.61918
Nc-1-IP_pe	chr10	1.33E+08	1.33E+08	462	1.33E+08	11	4.54404	3.2242
Nc-1-IP_pe	chr10	1.33E+08	1.33E+08	850	1.33E+08	12	4.70082	3.20033
Nc-1-IP_pe	chr10	1.33E+08	1.33E+08	468	1.33E+08	9	3.92293	3.08
Nc-1-IP_pe	chr10	1.33E+08	1.33E+08	501	1.33E+08	9	3.72192	2.95716
Nc-1-IP_pe	chr10	1.33E+08	1.33E+08	609	1.33E+08	8	3.74505	3.07688
Nc-1-IP_pe	chr10	1.33E+08	1.33E+08	326	1.33E+08	11	6.03439	4.10251
Nc-1-IP_pe	chr10	1.33E+08	1.33E+08	393	1.33E+08	12	5.23082	3.49289
Nc-1-IP_pe	chr10	1.33E+08	1.33E+08	281	1.33E+08	8	3.74505	3.07688
Nc-1-IP_pe	chr10	1.33E+08	1.33E+08	282	1.33E+08	9	3.72192	2.95716
Nc-1-IP_pe	chr10	1.33E+08	1.33E+08	434	1.33E+08	10	5.23357	3.76063
Nc-1-IP_pe	chr10	1.33E+08	1.33E+08	329	1.33E+08	10	5.23357	3.76063
Nc-1-IP_pe	chr10	1.33E+08	1.33E+08	464	1.33E+08	8	3.7276	3.06575
Nc-1-IP_pe	chr10	1.33E+08	1.33E+08	283	1.33E+08	10	4.33501	3.21677
Nc-1-IP_pe	chr10	1.33E+08	1.33E+08	662	1.33E+08	9	4.05981	3.16422
Nc-1-IP_pe	chr10	1.33E+08	1.33E+08	382	1.33E+08	13	5.9487	3.76157
Nc-1-IP_pe	chr10	1.33E+08	1.33E+08	386	1.33E+08	9	4.46937	3.41876

Nc-1-IP_pe	chr10	1.33E+08	1.33E+08	256	1.33E+08	11	5.19376	3.60018
Nc-1-IP_pe	chr10	1.33E+08	1.33E+08	419	1.33E+08	11	4.54404	3.2242
Nc-1-IP_pe	chr10	1.33E+08	1.33E+08	264	1.33E+08	10	4.33501	3.21677
Nc-1-IP_pe	chr10	1.33E+08	1.33E+08	807	1.33E+08	9	4.46937	3.41876
Nc-1-IP_pe	chr10	1.33E+08	1.33E+08	398	1.33E+08	11	5.30676	3.66673
Nc-1-IP_pe	chr10	1.33E+08	1.33E+08	277	1.33E+08	9	3.72947	2.96176
Nc-1-IP_pe	chr10	1.33E+08	1.33E+08	828	1.33E+08	12	5.15877	3.45264
Nc-1-IP_pe	chr10	1.33E+08	1.33E+08	344	1.33E+08	13	5.15976	3.33548
Nc-1-IP_pe	chr10	1.33E+08	1.33E+08	392	1.33E+08	8	3.74505	3.07688
Nc-1-IP_pe	chr10	1.33E+08	1.33E+08	693	1.33E+08	11	5.10577	3.5486
Nc-1-IP_pe	chr10	1.33E+08	1.33E+08	601	1.33E+08	7	3.06442	2.73501
Nc-1-IP_pe	chr10	1.33E+08	1.33E+08	743	1.33E+08	12	5.23082	3.49289
Nc-1-IP_pe	chr10	1.33E+08	1.33E+08	442	1.33E+08	9	3.72192	2.95716
Nc-1-IP_pe	chr10	1.33E+08	1.33E+08	369	1.33E+08	13	3.35769	2.42919
Nc-1-IP_pe	chr10	1.33E+08	1.33E+08	372	1.33E+08	13	7.7352	4.78626
Nc-1-IP_pe	chr10	1.33E+08	1.33E+08	628	1.33E+08	13	7.7352	4.78626
Nc-1-IP_pe	chr10	1.33E+08	1.33E+08	341	1.33E+08	12	6.86906	4.44438
Nc-1-IP_pe	chr10	1.33E+08	1.33E+08	302	1.33E+08	10	4.33501	3.21677
Nc-1-IP_pe	chr10	1.33E+08	1.33E+08	310	1.33E+08	8	3.56021	2.95918
Nc-1-IP_pe	chr10	1.33E+08	1.33E+08	533	1.33E+08	13	6.62436	4.14003
Nc-1-IP_pe	chr10	1.33E+08	1.33E+08	347	1.33E+08	10	4.82246	3.50943
Nc-1-IP_pe	chr10	1.33E+08	1.33E+08	818	1.33E+08	14	5.03085	3.16286
Nc-1-IP_pe	chr10	1.33E+08	1.33E+08	486	1.33E+08	12	5.15877	3.45264
Nc-1-IP_pe	chr10	1.33E+08	1.33E+08	342	1.33E+08	13	6.84576	4.26658
Nc-1-IP_pe	chr10	1.33E+08	1.33E+08	281	1.33E+08	9	4.2632	3.29017
Nc-1-IP_pe	chr10	1.33E+08	1.33E+08	313	1.33E+08	10	4.39571	3.25288
Nc-1-IP_pe	chr10	1.33E+08	1.33E+08	267	1.33E+08	16	7.53857	4.18505
Nc-1-IP_pe	chr10	1.33E+08	1.33E+08	776	1.33E+08	12	6.58417	4.27436
Nc-1-IP_pe	chr10	1.33E+08	1.33E+08	552	1.33E+08	10	5.10552	3.682
Nc-1-IP_pe	chr10	1.33E+08	1.33E+08	464	1.33E+08	8	3.56021	2.95918
Nc-1-IP_pe	chr10	1.33E+08	1.33E+08	469	1.33E+08	8	3.56021	2.95918
Nc-1-IP_pe	chr10	1.33E+08	1.33E+08	487	1.33E+08	11	6.03439	4.10251
Nc-1-IP_pe	chr10	1.33E+08	1.33E+08	944	1.33E+08	13	7.34205	4.55444
Nc-1-IP_pe	chr10	1.33E+08	1.33E+08	695	1.33E+08	10	3.89064	2.95552
Nc-1-IP_pe	chr10	1.33E+08	1.33E+08	484	1.33E+08	10	5.23357	3.76063
Nc-1-IP_pe	chr10	1.33E+08	1.33E+08	311	1.33E+08	9	4.46937	3.41876
Nc-1-IP_pe	chr10	1.33E+08	1.33E+08	354	1.33E+08	9	4.25966	3.28797
Nc-1-IP_pe	chr10	1.33E+08	1.33E+08	315	1.33E+08	11	6.03439	4.10251
Nc-1-IP_pe	chr10	1.33E+08	1.33E+08	309	1.33E+08	8	3.74505	3.07688
Nc-1-IP_pe	chr10	1.33E+08	1.33E+08	722	1.33E+08	9	4.32403	3.32802
Nc-1-IP_pe	chr10	1.33E+08	1.33E+08	278	1.33E+08	13	7.34205	4.55444
Nc-1-IP_pe	chr10	1.33E+08	1.33E+08	349	1.33E+08	9	4.46937	3.41876
Nc-1-IP_pe	chr10	1.33E+08	1.33E+08	424	1.33E+08	9	4.46937	3.41876
Nc-1-IP_pe	chr10	1.33E+08	1.33E+08	1143	1.33E+08	18	4.32723	2.54542
Nc-1-IP_pe	chr10	1.34E+08	1.34E+08	371	1.34E+08	8	3.74505	3.07688

Nc-1-IP_pe	chr10	1.34E+08	1.34E+08	384	1.34E+08	13	5.9487	3.76157
Nc-1-IP_pe	chr10	1.34E+08	1.34E+08	374	1.34E+08	9	4.46937	3.41876
Nc-1-IP_pe	chr10	1.34E+08	1.34E+08	410	1.34E+08	9	4.46937	3.41876
Nc-1-IP_pe	chr10	1.34E+08	1.34E+08	718	1.34E+08	16	7.53857	4.18505
Nc-1-IP_pe	chr10	1.34E+08	1.34E+08	499	1.34E+08	21	13.7467	6.50576
Nc-1-IP_pe	chr10	1.34E+08	1.34E+08	301	1.34E+08	17	7.02067	3.79543
Nc-1-IP_pe	chr10	1.34E+08	1.34E+08	740	1.34E+08	39	13.2663	3.92686
Nc-1-IP_pe	chr10	1.34E+08	1.34E+08	538	1.34E+08	20	9.94094	4.76764
Nc-1-IP_pe	chr10	1.34E+08	1.34E+08	645	1.34E+08	32	10.7391	3.73924
Nc-1-IP_pe	chr10	1.34E+08	1.34E+08	271	1.34E+08	12	3.30117	2.46734
Nc-1-IP_pe	chr10	1.34E+08	1.34E+08	510	1.34E+08	23	7.4429	3.36882
Nc-1-IP_pe	chr10	1.34E+08	1.34E+08	389	1.34E+08	16	5.47236	3.17959
Nc-1-IP_pe	chr10	1.34E+08	1.34E+08	582	1.34E+08	20	6.08336	3.09555
Nc-1-IP_pe	chr10	1.34E+08	1.34E+08	1361	1.34E+08	33	15.9401	5.3479
Nc-1-IP_pe	chr10	1.34E+08	1.34E+08	537	1.34E+08	17	6.48487	3.5442
Nc-1-IP_pe	chr10	1.34E+08	1.34E+08	1313	1.34E+08	33	12.439	4.17441
Nc-1-IP_pe	chr10	1.34E+08	1.34E+08	555	1.34E+08	24	9.05651	3.87976
Nc-1-IP_pe	chr10	1.34E+08	1.34E+08	1317	1.34E+08	26	6.47412	2.84038
Nc-1-IP_pe	chr10	1.34E+08	1.34E+08	267	1.34E+08	22	8.23987	3.76615
Nc-1-IP_pe	chr10	1.34E+08	1.34E+08	372	1.34E+08	28	6.23052	2.6687
Nc-1-IP_pe	chr10	1.34E+08	1.34E+08	270	1.34E+08	15	5.66913	3.37372
Nc-1-IP_pe	chr10	1.34E+08	1.34E+08	475	1.34E+08	20	6.94658	3.44067
Nc-1-IP_pe	chr10	1.34E+08	1.34E+08	902	1.34E+08	21	4.34782	2.40029
Nc-1-IP_pe	chr10	1.34E+08	1.34E+08	501	1.34E+08	17	6.29293	3.456
Nc-1-IP_pe	chr10	1.34E+08	1.34E+08	531	1.34E+08	32	9.82842	3.47157
Nc-1-IP_pe	chr10	1.34E+08	1.34E+08	437	1.34E+08	16	5.45004	3.16935
Nc-1-IP_pe	chr10	1.34E+08	1.34E+08	1327	1.34E+08	30	12.0847	4.35139
Nc-1-IP_pe	chr10	1.34E+08	1.34E+08	498	1.34E+08	22	9.61876	4.33685
Nc-1-IP_pe	chr10	1.34E+08	1.34E+08	762	1.34E+08	34	9.75686	3.32495
Nc-1-IP_pe	chr10	1.34E+08	1.34E+08	585	1.34E+08	23	10.5623	4.608
Nc-1-IP_pe	chr10	1.34E+08	1.34E+08	1520	1.34E+08	27	11.7969	4.58755
Nc-1-IP_pe	chr10	1.34E+08	1.34E+08	477	1.34E+08	18	8.46177	4.35631
Nc-1-IP_pe	chr10	1.34E+08	1.34E+08	1135	1.34E+08	38	11.2687	3.47999
Nc-1-IP_pe	chr10	1.34E+08	1.34E+08	891	1.34E+08	34	16.5513	5.43166
Nc-1-IP_pe	chr11	123163	123525	363	123375	18	9.1091	4.6774
Nc-1-IP_pe	chr11	123788	124733	946	124240	18	9.94172	5.10499
Nc-1-IP_pe	chr11	126545	126815	271	126783	11	4.47871	3.18705
Nc-1-IP_pe	chr11	127677	127982	306	127792	16	7.53857	4.18505
Nc-1-IP_pe	chr11	134456	134748	293	134589	27	7.72987	3.17269
Nc-1-IP_pe	chr11	135441	135742	302	135594	13	5.36853	3.44651
Nc-1-IP_pe	chr11	145870	146192	323	146108	18	9.1091	4.6774
Nc-1-IP_pe	chr11	165765	166458	694	165816	11	3.28475	2.53029
Nc-1-IP_pe	chr11	166900	167200	301	167005	12	4.63088	3.16234
Nc-1-IP_pe	chr11	175494	176467	974	176299	16	7.11257	3.96852
Nc-1-IP_pe	chr11	189351	190274	924	189783	22	9.39426	4.24119

Nc-1-IP_pe	chr11	192329	192769	441	192730	16	4.96462	2.94973
Nc-1-IP_pe	chr11	197196	197450	255	197297	13	6.14303	3.86919
Nc-1-IP_pe	chr11	198267	198804	538	198676	11	5.19376	3.60018
Nc-1-IP_pe	chr11	199115	199499	385	199213	13	4.86362	3.18015
Nc-1-IP_pe	chr11	200909	201576	668	201556	12	4.24064	2.95299
Nc-1-IP_pe	chr11	203334	204134	801	203982	16	6.24025	3.54005
Nc-1-IP_pe	chr11	207072	207348	277	207259	16	4.96462	2.94973
Nc-1-IP_pe	chr11	209474	210515	1042	209566	19	5.90435	3.10381
Nc-1-IP_pe	chr11	230474	230769	296	230521	11	5.3232	3.67643
Nc-1-IP_pe	chr11	236991	237299	309	237148	14	5.51541	3.4073
Nc-1-IP_pe	chr11	247509	247820	312	247674	12	4.70082	3.20033
Nc-1-IP_pe	chr11	265256	265751	496	265610	14	6.69566	4.03025
Nc-1-IP_pe	chr11	270837	271125	289	271007	13	4.41954	2.952
Nc-1-IP_pe	chr11	278027	279652	1626	278204	16	5.82808	3.34463
Nc-1-IP_pe	chr11	280794	281204	411	280876	13	5.9487	3.76157
Nc-1-IP_pe	chr11	282814	283230	417	282817	10	3.89064	2.95552
Nc-1-IP_pe	chr11	288747	289222	476	288904	15	6.24734	3.66122
Nc-1-IP_pe	chr11	289539	290113	575	289699	18	8.80785	4.52672
Nc-1-IP_pe	chr11	292891	293216	326	292936	11	3.64839	2.72584
Nc-1-IP_pe	chr11	295348	295600	253	295483	15	6.69889	3.8921
Nc-1-IP_pe	chr11	296142	296638	497	296370	16	5.73763	3.30235
Nc-1-IP_pe	chr11	299207	299716	510	299311	17	9.09431	4.8363
Nc-1-IP_pe	chr11	299983	300494	512	300458	13	4.86362	3.18015
Nc-1-IP_pe	chr11	303931	304222	292	304056	13	6.29523	3.95418
Nc-1-IP_pe	chr11	306845	307549	705	307477	14	6.44441	3.89444
Nc-1-IP_pe	chr11	307984	309257	1274	308334	15	3.46204	2.35851
Nc-1-IP_pe	chr11	310843	311856	1014	311360	24	13.903	5.95621
Nc-1-IP_pe	chr11	314315	315417	1103	314323	12	3.83688	2.74114
Nc-1-IP_pe	chr11	315866	316482	617	316000	14	5.89493	3.6034
Nc-1-IP_pe	chr11	318181	319271	1091	319055	25	4.60609	2.31999
Nc-1-IP_pe	chr11	320492	320745	254	320590	19	5.51632	2.94814
Nc-1-IP_pe	chr11	322171	322589	419	322459	15	4.06016	2.62146
Nc-1-IP_pe	chr11	327168	327488	321	327358	11	4.74697	3.34038
Nc-1-IP_pe	chr11	328051	328705	655	328212	14	5.51541	3.4073
Nc-1-IP_pe	chr11	332717	333209	493	333060	9	3.74196	2.96936
Nc-1-IP_pe	chr11	337316	337916	601	337432	12	6.86906	4.44438
Nc-1-IP_pe	chr11	339186	339500	315	339342	9	4.46937	3.41876
Nc-1-IP_pe	chr11	345917	346176	260	346105	8	3.74505	3.07688
Nc-1-IP_pe	chr11	351426	351859	434	351525	12	3.83688	2.74114
Nc-1-IP_pe	chr11	353050	353615	566	353500	10	3.89064	2.95552
Nc-1-IP_pe	chr11	355322	355959	638	355487	15	6.10627	3.59023
Nc-1-IP_pe	chr11	364750	365320	571	364797	13	3.44439	2.47074
Nc-1-IP_pe	chr11	367241	367631	391	367470	9	3.59586	2.88066
Nc-1-IP_pe	chr11	368289	368771	483	368740	15	5.66913	3.37372
Nc-1-IP_pe	chr11	370267	371329	1063	371232	12	5.94619	3.9002

Nc-1-IP_pe	chr11	375402	375989	588	375864	9	4.46937	3.41876
Nc-1-IP_pe	chr11	376944	377606	663	377139	12	6.86906	4.44438
Nc-1-IP_pe	chr11	379860	380612	753	380515	10	4.62142	3.388
Nc-1-IP_pe	chr11	382413	382728	316	382602	11	6.00992	4.08766
Nc-1-IP_pe	chr11	385326	386147	822	386123	13	6.02837	3.80557
Nc-1-IP_pe	chr11	391779	392098	320	391788	10	3.83201	2.92147
Nc-1-IP_pe	chr11	399936	400593	658	400416	9	4.46937	3.41876
Nc-1-IP_pe	chr11	406111	406366	256	406151	12	5.84943	3.84431
Nc-1-IP_pe	chr11	407801	408160	360	408024	13	5.71736	3.63481
Nc-1-IP_pe	chr11	418863	419148	286	418896	11	5.19376	3.60018
Nc-1-IP_pe	chr11	429392	430143	752	429422	10	5.23357	3.76063
Nc-1-IP_pe	chr11	432732	433075	344	432868	11	6.03439	4.10251
Nc-1-IP_pe	chr11	434364	435057	694	434627	10	5.23357	3.76063
Nc-1-IP_pe	chr11	437347	438217	871	437703	14	8.29543	4.93196
Nc-1-IP_pe	chr11	439241	439980	740	439778	9	3.27326	2.68684
Nc-1-IP_pe	chr11	441187	441610	424	441207	9	4.46937	3.41876
Nc-1-IP_pe	chr11	443018	443549	532	443171	10	4.62142	3.388
Nc-1-IP_pe	chr11	445862	446355	494	446298	9	3.80523	3.00795
Nc-1-IP_pe	chr11	455294	455681	388	455507	11	5.76039	3.93701
Nc-1-IP_pe	chr11	485739	486213	475	486052	10	3.4604	2.70797
Nc-1-IP_pe	chr11	498404	498731	328	498461	9	3.53727	2.84525
Nc-1-IP_pe	chr11	499610	500672	1063	500640	11	4.06403	2.95415
Nc-1-IP_pe	chr11	501709	502456	748	502116	12	5.8598	3.85029
Nc-1-IP_pe	chr11	503933	504496	564	504429	12	6.07041	3.97229
Nc-1-IP_pe	chr11	519498	520315	818	520218	28	9.16687	3.56053
Nc-1-IP_pe	chr11	530418	531081	664	531081	21	3.04296	1.96307
Nc-1-IP_pe	chr11	532861	533475	615	533091	11	4.38975	3.13666
Nc-1-IP_pe	chr11	535801	536630	830	536086	16	9.11744	5.02718
Nc-1-IP_pe	chr11	537702	538476	775	537967	12	6.31185	4.11348
Nc-1-IP_pe	chr11	540663	541138	476	540825	10	4.91962	3.56846
Nc-1-IP_pe	chr11	551879	552334	456	551966	12	4.70082	3.20033
Nc-1-IP_pe	chr11	553689	554554	866	554226	9	3.63165	2.90233
Nc-1-IP_pe	chr11	555425	555873	449	555715	16	7.53857	4.18505
Nc-1-IP_pe	chr11	560159	560716	558	560525	16	7.97877	4.4137
Nc-1-IP_pe	chr11	562127	562616	490	562510	11	3.28475	2.53029
Nc-1-IP_pe	chr11	575485	575767	283	575662	17	10.2946	5.4856
Nc-1-IP_pe	chr11	588120	588486	367	588304	14	6.05554	3.68761
Nc-1-IP_pe	chr11	596343	596989	647	596841	9	4.05981	3.16422
Nc-1-IP_pe	chr11	597685	598093	409	597777	13	6.19316	3.89711
Nc-1-IP_pe	chr11	605284	605930	647	605749	14	6.69566	4.03025
Nc-1-IP_pe	chr11	606375	608278	1904	606530	14	4.21449	2.76599
Nc-1-IP_pe	chr11	609081	609586	506	609401	14	7.42849	4.43574
Nc-1-IP_pe	chr11	612260	613014	755	612335	11	4.8033	3.37283
Nc-1-IP_pe	chr11	613855	614422	568	613955	11	4.8033	3.37283
Nc-1-IP_pe	chr11	625711	626424	714	625845	10	5.07094	3.66082

Nc-1-IP_pe	chr11	627409	628426	1018	627719	11	6.03439	4.10251
Nc-1-IP_pe	chr11	636623	637116	494	636670	9	4.05981	3.16422
Nc-1-IP_pe	chr11	641529	641973	445	641824	9	4.05981	3.16422
Nc-1-IP_pe	chr11	642710	643257	548	642813	22	8.24538	3.76835
Nc-1-IP_pe	chr11	645427	645744	318	645680	9	3.72192	2.95716
Nc-1-IP_pe	chr11	660388	660899	512	660523	9	4.46937	3.41876
Nc-1-IP_pe	chr11	662640	663183	544	662804	12	6.86404	4.44138
Nc-1-IP_pe	chr11	689694	690052	359	689882	10	5.23357	3.76063
Nc-1-IP_pe	chr11	694436	694708	273	694481	10	4.99888	3.61677
Nc-1-IP_pe	chr11	695861	696310	450	695947	14	6.44441	3.89444
Nc-1-IP_pe	chr11	705629	706416	788	706231	14	5.51541	3.4073
Nc-1-IP_pe	chr11	707245	707533	289	707474	13	3.67377	2.58159
Nc-1-IP_pe	chr11	711734	712644	911	712343	14	7.99713	4.75934
Nc-1-IP_pe	chr11	719624	720045	422	719964	11	4.54404	3.2242
Nc-1-IP_pe	chr11	720735	721353	619	720792	11	5.03835	3.5092
Nc-1-IP_pe	chr11	725362	726005	644	725536	12	5.84943	3.84431
Nc-1-IP_pe	chr11	728314	728754	441	728394	15	5.20142	3.14789
Nc-1-IP_pe	chr11	737542	737982	441	737959	8	3.38431	2.8478
Nc-1-IP_pe	chr11	763636	764052	417	763908	14	5.51541	3.4073
Nc-1-IP_pe	chr11	765577	765882	306	765795	10	4.20113	3.13749
Nc-1-IP_pe	chr11	772327	772951	625	772426	12	6.18005	4.03623
Nc-1-IP_pe	chr11	773453	774194	742	773676	11	5.22568	3.61895
Nc-1-IP_pe	chr11	776604	777916	1313	777287	11	5.19376	3.60018
Nc-1-IP_pe	chr11	778973	779430	458	779375	10	3.60969	2.79325
Nc-1-IP_pe	chr11	780111	780606	496	780232	12	5.61153	3.70794
Nc-1-IP_pe	chr11	782022	782331	310	782266	10	3.4604	2.70797
Nc-1-IP_pe	chr11	786550	789307	2758	787795	15	9.33061	5.34018
Nc-1-IP_pe	chr11	799620	799905	286	799898	10	3.4604	2.70797
Nc-1-IP_pe	chr11	802665	803531	867	803285	11	5.65062	3.87116
Nc-1-IP_pe	chr11	805612	806153	542	806077	11	5.42344	3.73577
Nc-1-IP_pe	chr11	810095	810669	575	810221	18	9.94172	5.10499
Nc-1-IP_pe	chr11	818376	819280	905	819235	14	3.86727	2.6027
Nc-1-IP_pe	chr11	819569	820021	453	819733	19	9.60791	4.76497
Nc-1-IP_pe	chr11	823998	824558	561	824514	11	3.89191	2.85898
Nc-1-IP_pe	chr11	825736	826110	375	825906	12	3.16137	2.39719
Nc-1-IP_pe	chr11	830369	831085	717	830836	12	5.43913	3.61006
Nc-1-IP_pe	chr11	835975	836963	989	836143	12	5.79203	3.81127
Nc-1-IP_pe	chr11	838466	838781	316	838583	15	7.02624	4.06292
Nc-1-IP_pe	chr11	840287	840585	299	840371	12	5.23082	3.49289
Nc-1-IP_pe	chr11	842190	842970	781	842849	13	5.82354	3.69281
Nc-1-IP_pe	chr11	846105	846491	387	846105	10	3.40382	2.67582
Nc-1-IP_pe	chr11	847713	848226	514	847927	14	7.40051	4.42001
Nc-1-IP_pe	chr11	852581	853017	437	852955	9	3.72192	2.95716
Nc-1-IP_pe	chr11	860234	860990	757	860931	12	5.84943	3.84431
Nc-1-IP_pe	chr11	861911	862565	655	862427	11	3.64839	2.72584

Nc-1-IP_pe	chr11	862860	863860	1001	862984	11	5.40654	3.72575
Nc-1-IP_pe	chr11	868566	868964	399	868625	10	5.19356	3.73603
Nc-1-IP_pe	chr11	891067	891725	659	891440	13	6.62436	4.14003
Nc-1-IP_pe	chr11	893502	893871	370	893625	9	4.33944	3.33761
Nc-1-IP_pe	chr11	895789	896523	735	895849	10	5.10552	3.682
Nc-1-IP_pe	chr11	904569	905222	654	904648	16	6.8987	3.86161
Nc-1-IP_pe	chr11	910506	910796	291	910618	13	5.03208	3.26819
Nc-1-IP_pe	chr11	919812	920559	748	920272	9	4.46937	3.41876
Nc-1-IP_pe	chr11	921442	921815	374	921512	11	6.03439	4.10251
Nc-1-IP_pe	chr11	925149	925595	447	925288	14	4.60011	2.95114
Nc-1-IP_pe	chr11	925867	926935	1069	925994	20	14.5316	7.17939
Nc-1-IP_pe	chr11	937871	938196	326	937956	9	4.46937	3.41876
Nc-1-IP_pe	chr11	956154	956408	255	956343	12	5.94619	3.9002
Nc-1-IP_pe	chr11	961849	962127	279	961973	12	4.92484	3.32299
Nc-1-IP_pe	chr11	964924	965261	338	965152	69	11.9794	2.57374
Nc-1-IP_pe	chr11	974917	975631	715	975481	25	8.22195	3.4832
Nc-1-IP_pe	chr11	978581	978851	271	978738	9	3.94978	3.09648
Nc-1-IP_pe	chr11	985363	985674	312	985456	10	4.95265	3.58858
Nc-1-IP_pe	chr11	990075	990380	306	990248	14	8.20556	4.87976
Nc-1-IP_pe	chr11	994753	995083	331	994877	14	8.20556	4.87976
Nc-1-IP_pe	chr11	996873	997145	273	996894	9	3.27326	2.68684
Nc-1-IP_pe	chr11	1002540	1003002	463	1002614	13	4.41954	2.952
Nc-1-IP_pe	chr11	1007824	1008466	643	1008212	12	5.84943	3.84431
Nc-1-IP_pe	chr11	1008977	1009434	458	1009319	9	4.46937	3.41876
Nc-1-IP_pe	chr11	1009971	1010570	600	1010364	11	6.03439	4.10251
Nc-1-IP_pe	chr11	1012577	1012984	408	1012844	13	5.31884	3.41997
Nc-1-IP_pe	chr11	1016692	1018010	1319	1017363	31	6.23593	2.54618
Nc-1-IP_pe	chr11	1021846	1022358	513	1022302	11	4.78913	3.36466
Nc-1-IP_pe	chr11	1024431	1024726	296	1024526	14	6.44441	3.89444
Nc-1-IP_pe	chr11	1025981	1026799	819	1026595	10	4.33501	3.21677
Nc-1-IP_pe	chr11	1028317	1028578	262	1028541	10	3.40382	2.67582
Nc-1-IP_pe	chr11	1036351	1036708	358	1036574	13	4.02562	2.7544
Nc-1-IP_pe	chr11	1037049	1037311	263	1037266	9	3.80523	3.00795
Nc-1-IP_pe	chr11	1037773	1038034	262	1037969	9	3.79246	3.00015
Nc-1-IP_pe	chr11	1039919	1040538	620	1040435	11	4.06403	2.95415
Nc-1-IP_pe	chr11	1041172	1041781	610	1041563	11	4.35236	3.11555
Nc-1-IP_pe	chr11	1047943	1048250	308	1048206	8	3.37201	2.84003
Nc-1-IP_pe	chr11	1050590	1050855	266	1050792	15	5.70822	3.39286
Nc-1-IP_pe	chr11	1053177	1053879	703	1053671	15	8.38325	4.80024
Nc-1-IP_pe	chr11	1060212	1060635	424	1060458	9	3.92293	3.08
Nc-1-IP_pe	chr11	1071261	1071548	288	1071332	8	3.64414	3.01254
Nc-1-IP_pe	chr11	1072109	1072379	271	1072121	10	4.39571	3.25288
Nc-1-IP_pe	chr11	1073454	1074174	721	1073621	13	7.42513	4.60316
Nc-1-IP_pe	chr11	1077134	1077677	544	1077331	11	6.03439	4.10251
Nc-1-IP_pe	chr11	1084111	1084442	332	1084171	12	5.3037	3.53374

Nc-1-IP_pe	chr11	1090395	1091030	636	1090929	10	4.66672	3.41527
Nc-1-IP_pe	chr11	1092750	1093083	334	1093027	9	3.50273	2.82441
Nc-1-IP_pe	chr11	1095824	1097230	1407	1096109	19	7.29128	3.68799
Nc-1-IP_pe	chr11	1099264	1099674	411	1099501	28	14.9958	5.7296
Nc-1-IP_pe	chr11	1104876	1105293	418	1104922	11	5.50915	3.78671
Nc-1-IP_pe	chr11	1107781	1108275	495	1108074	11	5.65062	3.87116
Nc-1-IP_pe	chr11	1113769	1114123	355	1113894	13	5.9487	3.76157
Nc-1-IP_pe	chr11	1114743	1115160	418	1114795	10	4.47495	3.30017
Nc-1-IP_pe	chr11	1119021	1119504	484	1119136	13	7.34205	4.55444
Nc-1-IP_pe	chr11	1120865	1121977	1113	1121697	11	5.7051	3.90381
Nc-1-IP_pe	chr11	1122456	1122781	326	1122626	10	4.20113	3.13749
Nc-1-IP_pe	chr11	1126654	1127293	640	1126905	12	6.12485	4.004
Nc-1-IP_pe	chr11	1128493	1129191	699	1128868	15	7.02624	4.06292
Nc-1-IP_pe	chr11	1129995	1130299	305	1130290	11	3.40056	2.59215
Nc-1-IP_pe	chr11	1130810	1131178	369	1130874	11	3.64839	2.72584
Nc-1-IP_pe	chr11	1153851	1154478	628	1153877	13	4.41954	2.952
Nc-1-IP_pe	chr11	1156843	1158892	2050	1157031	12	6.58417	4.27436
Nc-1-IP_pe	chr11	1162113	1162473	361	1162326	12	5.23082	3.49289
Nc-1-IP_pe	chr11	1167232	1167489	258	1167389	9	4.20361	3.25317
Nc-1-IP_pe	chr11	1169782	1170150	369	1170029	11	6.03439	4.10251
Nc-1-IP_pe	chr11	1170674	1171133	460	1170801	10	5.23357	3.76063
Nc-1-IP_pe	chr11	1176234	1177202	969	1176356	10	3.89064	2.95552
Nc-1-IP_pe	chr11	1178190	1178499	310	1178343	11	5.3563	3.696
Nc-1-IP_pe	chr11	1188481	1188751	271	1188526	10	4.99888	3.61677
Nc-1-IP_pe	chr11	1194233	1194666	434	1194465	9	3.27326	2.68684
Nc-1-IP_pe	chr11	1197135	1197829	695	1197553	14	8.57364	5.09458
Nc-1-IP_pe	chr11	1198713	1199231	519	1199106	9	3.60774	2.88785
Nc-1-IP_pe	chr11	1207317	1207864	548	1207582	16	8.26996	4.56762
Nc-1-IP_pe	chr11	1208300	1209308	1009	1208750	9	4.46937	3.41876
Nc-1-IP_pe	chr11	1220295	1220643	349	1220405	10	4.47495	3.30017
Nc-1-IP_pe	chr11	1221053	1221475	423	1221102	9	3.83096	3.02367
Nc-1-IP_pe	chr11	1221881	1222260	380	1222027	12	5.54793	3.67174
Nc-1-IP_pe	chr11	1225381	1225851	471	1225449	11	5.00808	3.49156
Nc-1-IP_pe	chr11	1227339	1227707	369	1227496	13	6.41752	4.02291
Nc-1-IP_pe	chr11	1231561	1232497	937	1232342	12	6.12485	4.004
Nc-1-IP_pe	chr11	1235687	1236799	1113	1236181	15	8.84797	5.06275
Nc-1-IP_pe	chr11	1237488	1237998	511	1237492	11	3.64839	2.72584
Nc-1-IP_pe	chr11	1238934	1239268	335	1239005	10	4.40419	3.25794
Nc-1-IP_pe	chr11	1241643	1241930	288	1241820	9	3.63165	2.90233
Nc-1-IP_pe	chr11	1242608	1243176	569	1242773	11	3.28475	2.53029
Nc-1-IP_pe	chr11	1243734	1244007	274	1243782	17	5.9911	3.31919
Nc-1-IP_pe	chr11	1244266	1245238	973	1244721	14	6.49348	3.92083
Nc-1-IP_pe	chr11	1246721	1247175	455	1247091	11	4.35236	3.11555
Nc-1-IP_pe	chr11	1249148	1249500	353	1249328	16	5.82808	3.34463
Nc-1-IP_pe	chr11	1250412	1250739	328	1250558	13	6.01225	3.79665

Nc-1-IP_pe	chr11	1252497	1253660	1164	1252562	15	7.46994	4.29894
Nc-1-IP_pe	chr11	1256897	1257149	253	1257093	10	4.39571	3.25288
Nc-1-IP_pe	chr11	1258153	1258607	455	1258425	11	4.54404	3.2242
Nc-1-IP_pe	chr11	1261298	1261687	390	1261628	14	5.03085	3.16286
Nc-1-IP_pe	chr11	1265462	1266002	541	1265919	9	4.14522	3.217
Nc-1-IP_pe	chr11	1271762	1272756	995	1272092	12	6.86404	4.44138
Nc-1-IP_pe	chr11	1276816	1277135	320	1276899	12	6.71369	4.35145
Nc-1-IP_pe	chr11	1281381	1281924	544	1281709	10	4.99888	3.61677
Nc-1-IP_pe	chr11	1288054	1288347	294	1288233	9	3.27326	2.68684
Nc-1-IP_pe	chr11	1295728	1296054	327	1295871	12	4.92484	3.32299
Nc-1-IP_pe	chr11	1297944	1298370	427	1298172	11	4.45305	3.17249
Nc-1-IP_pe	chr11	1306288	1306601	314	1306499	11	5.05359	3.5181
Nc-1-IP_pe	chr11	1309521	1310213	693	1309573	12	3.16137	2.39719
Nc-1-IP_pe	chr11	1337435	1337699	265	1337596	9	4.05981	3.16422
Nc-1-IP_pe	chr11	1340291	1340561	271	1340366	8	3.74505	3.07688
Nc-1-IP_pe	chr11	1343560	1343872	313	1343630	8	3.74505	3.07688
Nc-1-IP_pe	chr11	1349163	1349498	336	1349301	11	5.77463	3.94557
Nc-1-IP_pe	chr11	1353254	1353984	731	1353620	10	3.89064	2.95552
Nc-1-IP_pe	chr11	1368893	1369978	1086	1369185	14	5.51541	3.4073
Nc-1-IP_pe	chr11	1375094	1375463	370	1375356	11	6.03439	4.10251
Nc-1-IP_pe	chr11	1388595	1388975	381	1388927	12	4.51041	3.09723
Nc-1-IP_pe	chr11	1396720	1397009	290	1396766	8	3.56332	2.96115
Nc-1-IP_pe	chr11	1399228	1400322	1095	1399767	11	5.85441	3.99362
Nc-1-IP_pe	chr11	1404211	1404707	497	1404611	10	5.23357	3.76063
Nc-1-IP_pe	chr11	1411779	1412575	797	1412315	12	6.6095	4.28941
Nc-1-IP_pe	chr11	1415468	1415720	253	1415531	8	3.74505	3.07688
Nc-1-IP_pe	chr11	1417645	1418101	457	1418005	8	3.08755	2.66145
Nc-1-IP_pe	chr11	1419907	1420178	272	1420122	9	4.46937	3.41876
Nc-1-IP_pe	chr11	1422485	1422738	254	1422549	10	5.23357	3.76063
Nc-1-IP_pe	chr11	1426063	1426387	325	1426210	10	4.77495	3.48064
Nc-1-IP_pe	chr11	1426744	1427194	451	1426919	11	6.03439	4.10251
Nc-1-IP_pe	chr11	1429213	1429663	451	1429623	10	3.4604	2.70797
Nc-1-IP_pe	chr11	1439803	1440177	375	1440039	12	6.58417	4.27436
Nc-1-IP_pe	chr11	1448340	1448605	266	1448451	12	5.61153	3.70794
Nc-1-IP_pe	chr11	1451051	1451345	295	1451272	12	5.84943	3.84431
Nc-1-IP_pe	chr11	1453664	1454079	416	1453743	10	4.59158	3.37006
Nc-1-IP_pe	chr11	1455589	1456122	534	1455792	13	7.32024	4.54167
Nc-1-IP_pe	chr11	1465473	1465815	343	1465531	9	3.66792	2.92434
Nc-1-IP_pe	chr11	1468791	1469153	363	1468968	12	6.12485	4.004
Nc-1-IP_pe	chr11	1478124	1478588	465	1478172	9	2.89249	2.46179
Nc-1-IP_pe	chr11	1479635	1479964	330	1479793	9	3.72192	2.95716
Nc-1-IP_pe	chr11	1481940	1482272	333	1482152	9	3.27326	2.68684
Nc-1-IP_pe	chr11	1487840	1488259	420	1488123	14	6.06519	3.69269
Nc-1-IP_pe	chr11	1509996	1510419	424	1510254	26	8.84747	3.61717
Nc-1-IP_pe	chr11	1514976	1515541	566	1515210	12	6.29276	4.10227

Nc-1-IP_pe	chr11	1520373	1520649	277	1520485	13	7.47517	4.63258
Nc-1-IP_pe	chr11	1525062	1525458	397	1525397	9	4.46937	3.41876
Nc-1-IP_pe	chr11	1526390	1526670	281	1526572	11	3.64839	2.72584
Nc-1-IP_pe	chr11	1541193	1542223	1031	1541390	10	4.39571	3.25288
Nc-1-IP_pe	chr11	1543456	1543820	365	1543607	10	5.23357	3.76063
Nc-1-IP_pe	chr11	1544240	1544614	375	1544481	9	4.46937	3.41876
Nc-1-IP_pe	chr11	1546348	1547114	767	1546921	13	6.92473	4.312
Nc-1-IP_pe	chr11	1548818	1550184	1367	1549349	13	7.7352	4.78626
Nc-1-IP_pe	chr11	1554390	1554671	282	1554606	11	3.64839	2.72584
Nc-1-IP_pe	chr11	1559553	1559953	401	1559759	11	5.10577	3.5486
Nc-1-IP_pe	chr11	1571289	1571563	275	1571343	11	5.81652	3.97078
Nc-1-IP_pe	chr11	1573561	1574077	517	1573871	8	3.74505	3.07688
Nc-1-IP_pe	chr11	1587262	1587643	382	1587507	10	4.99888	3.61677
Nc-1-IP_pe	chr11	1596614	1596972	359	1596702	9	4.46937	3.41876
Nc-1-IP_pe	chr11	1597633	1598514	882	1598218	14	8.63073	5.12814
Nc-1-IP_pe	chr11	1624837	1625140	304	1625037	11	6.03439	4.10251
Nc-1-IP_pe	chr11	1629673	1629971	299	1629747	10	4.99888	3.61677
Nc-1-IP_pe	chr11	1630412	1630680	269	1630634	9	4.46937	3.41876
Nc-1-IP_pe	chr11	1632298	1632604	307	1632365	12	6.58417	4.27436
Nc-1-IP_pe	chr11	1639905	1640464	560	1640283	10	5.23357	3.76063
Nc-1-IP_pe	chr11	1642565	1642911	347	1642715	10	4.99888	3.61677
Nc-1-IP_pe	chr11	1651197	1651578	382	1651569	8	3.08755	2.66145
Nc-1-IP_pe	chr11	1653157	1653547	391	1653417	11	5.77463	3.94557
Nc-1-IP_pe	chr11	1654269	1654716	448	1654663	8	3.74505	3.07688
Nc-1-IP_pe	chr11	1659435	1660153	719	1659986	8	3.64414	3.01254
Nc-1-IP_pe	chr11	1660689	1661144	456	1660794	10	5.23357	3.76063
Nc-1-IP_pe	chr11	1670615	1671064	450	1670674	31	5.48389	2.35492
Nc-1-IP_pe	chr11	1678277	1678623	347	1678604	9	4.46937	3.41876
Nc-1-IP_pe	chr11	1684948	1685356	409	1685210	12	6.58417	4.27436
Nc-1-IP_pe	chr11	1686254	1686746	493	1686525	17	6.47874	3.54137
Nc-1-IP_pe	chr11	1695001	1695316	316	1695060	12	6.50787	4.22912
Nc-1-IP_pe	chr11	1706879	1707315	437	1707230	11	5.10577	3.5486
Nc-1-IP_pe	chr11	1730244	1730828	585	1730421	10	5.23357	3.76063
Nc-1-IP_pe	chr11	1747163	1747480	318	1747405	11	5.24175	3.6284
Nc-1-IP_pe	chr11	1749290	1749854	565	1749606	12	5.75858	3.79206
Nc-1-IP_pe	chr11	1761587	1762169	583	1761954	18	11.5887	5.99562
Nc-1-IP_pe	chr11	1763670	1763967	298	1763787	16	5.82808	3.34463
Nc-1-IP_pe	chr11	1766329	1766616	288	1766365	7	2.9043	2.63038
Nc-1-IP_pe	chr11	1768705	1768975	271	1768804	10	5.10552	3.682
Nc-1-IP_pe	chr11	1779253	1779597	345	1779421	13	4.86362	3.18015
Nc-1-IP_pe	chr11	1793695	1794065	371	1793971	9	4.46937	3.41876
Nc-1-IP_pe	chr11	1825552	1826148	597	1825591	13	4.02562	2.7544
Nc-1-IP_pe	chr11	1826624	1827050	427	1827044	10	3.30648	2.62073
Nc-1-IP_pe	chr11	1833183	1833889	707	1833533	11	4.35236	3.11555
Nc-1-IP_pe	chr11	1835275	1835950	676	1835688	13	5.96425	3.77015

Nc-1-IP_pe	chr11	1837659	1838058	400	1837765	12	5.42384	3.60142
Nc-1-IP_pe	chr11	1838336	1838588	253	1838509	12	4.70082	3.20033
Nc-1-IP_pe	chr11	1843081	1843385	305	1843236	12	5.3037	3.53374
Nc-1-IP_pe	chr11	1848828	1849228	401	1848925	9	3.27326	2.68684
Nc-1-IP_pe	chr11	1849599	1849995	397	1849756	11	3.28475	2.53029
Nc-1-IP_pe	chr11	1855325	1855738	414	1855534	10	4.48929	3.30875
Nc-1-IP_pe	chr11	1864451	1864715	265	1864556	12	5.74196	3.78252
Nc-1-IP_pe	chr11	1866055	1867011	957	1866614	12	5.39344	3.58426
Nc-1-IP_pe	chr11	1870059	1870356	298	1870271	11	5.03835	3.5092
Nc-1-IP_pe	chr11	1873519	1874082	564	1873985	12	5.69253	3.7542
Nc-1-IP_pe	chr11	1875169	1875810	642	1875701	10	4.32135	3.20866
Nc-1-IP_pe	chr11	1876711	1876983	273	1876875	13	5.42865	3.47872
Nc-1-IP_pe	chr11	1877570	1877830	261	1877778	15	5.20142	3.14789
Nc-1-IP_pe	chr11	1878573	1879042	470	1878758	11	5.10577	3.5486
Nc-1-IP_pe	chr11	1879914	1880669	756	1880018	12	6.14317	4.01469
Nc-1-IP_pe	chr11	1882007	1882652	646	1882538	11	6.03439	4.10251
Nc-1-IP_pe	chr11	1883912	1884258	347	1884099	8	3.74505	3.07688
Nc-1-IP_pe	chr11	1889221	1889729	509	1889287	9	3.72192	2.95716
Nc-1-IP_pe	chr11	1904110	1904473	364	1904227	11	6.03439	4.10251
Nc-1-IP_pe	chr11	1918319	1918635	317	1918479	11	4.54404	3.2242
Nc-1-IP_pe	chr11	1921800	1922240	441	1922070	8	3.74505	3.07688
Nc-1-IP_pe	chr11	1924663	1925130	468	1924923	11	5.7051	3.90381
Nc-1-IP_pe	chr11	1926640	1926995	356	1926839	10	4.99888	3.61677
Nc-1-IP_pe	chr11	1936172	1936573	402	1936376	16	10.5028	5.81189
Nc-1-IP_pe	chr11	1947389	1947689	301	1947549	12	5.84943	3.84431
Nc-1-IP_pe	chr11	1949240	1949495	256	1949309	9	3.27326	2.68684
Nc-1-IP_pe	chr11	1951532	1951953	422	1951676	10	4.50371	3.31737
Nc-1-IP_pe	chr11	1954593	1954923	331	1954772	10	3.89064	2.95552
Nc-1-IP_pe	chr11	1958100	1958666	567	1958552	10	4.39571	3.25288
Nc-1-IP_pe	chr11	1961265	1961545	281	1961275	8	3.52386	2.93611
Nc-1-IP_pe	chr11	1965444	1965723	280	1965459	10	3.08907	2.49869
Nc-1-IP_pe	chr11	1970469	1970958	490	1970509	10	4.30776	3.2006
Nc-1-IP_pe	chr11	1973023	1973622	600	1973143	11	5.3232	3.67643
Nc-1-IP_pe	chr11	1983188	1984056	869	1983259	13	7.21273	4.47889
Nc-1-IP_pe	chr11	1985458	1985911	454	1985544	10	4.4323	3.2747
Nc-1-IP_pe	chr11	1987587	1988701	1115	1988554	11	4.06403	2.95415
Nc-1-IP_pe	chr11	1989557	1989928	372	1989755	15	6.10627	3.59023
Nc-1-IP_pe	chr11	1990477	1990926	450	1990601	13	5.29187	3.40559
Nc-1-IP_pe	chr11	1992037	1992425	389	1992191	15	7.02624	4.06292
Nc-1-IP_pe	chr11	1992932	1993470	539	1993213	14	7.34079	4.38651
Nc-1-IP_pe	chr11	1996057	1996324	268	1996138	13	6.62436	4.14003
Nc-1-IP_pe	chr11	1997487	1997858	372	1997745	13	7.04588	4.38198
Nc-1-IP_pe	chr11	2001929	2002203	275	2002019	7	2.9043	2.63038
Nc-1-IP_pe	chr11	2002685	2003155	471	2002770	9	4.25966	3.28797
Nc-1-IP_pe	chr11	2004608	2005489	882	2005144	8	3.74505	3.07688

Nc-1-IP_pe	chr11	2014114	2014374	261	2014264	10	4.39571	3.25288
Nc-1-IP_pe	chr11	2019222	2019591	370	2019516	11	3.64839	2.72584
Nc-1-IP_pe	chr11	2025316	2025678	363	2025676	9	3.72192	2.95716
Nc-1-IP_pe	chr11	2028826	2029266	441	2029229	9	4.20361	3.25317
Nc-1-IP_pe	chr11	2029724	2030314	591	2029817	10	5.10552	3.682
Nc-1-IP_pe	chr11	2036711	2036976	266	2036893	14	4.21449	2.76599
Nc-1-IP_pe	chr11	2092197	2092558	362	2092358	9	4.05981	3.16422
Nc-1-IP_pe	chr11	2093599	2094741	1143	2094068	14	5.03085	3.16286
Nc-1-IP_pe	chr11	2097750	2098242	493	2098109	12	4.70082	3.20033
Nc-1-IP_pe	chr11	2099350	2099723	374	2099639	9	4.15971	3.22596
Nc-1-IP_pe	chr11	2123430	2123861	432	2123616	8	3.56021	2.95918
Nc-1-IP_pe	chr11	2127745	2128065	321	2127968	9	4.46937	3.41876
Nc-1-IP_pe	chr11	2144000	2144916	917	2144708	12	6.54823	4.25303
Nc-1-IP_pe	chr11	2145224	2145568	345	2145567	10	3.08907	2.49869
Nc-1-IP_pe	chr11	2146037	2146332	296	2146149	10	4.99888	3.61677
Nc-1-IP_pe	chr11	2148229	2148521	293	2148419	9	4.33944	3.33761
Nc-1-IP_pe	chr11	2150876	2151551	676	2151427	12	4.75569	3.23024
Nc-1-IP_pe	chr11	2152265	2153168	904	2152411	12	6.50787	4.22912
Nc-1-IP_pe	chr11	2154132	2154557	426	2154339	9	3.92293	3.08
Nc-1-IP_pe	chr11	2155767	2156035	269	2155795	9	4.24819	3.28084
Nc-1-IP_pe	chr11	2164036	2164307	272	2164275	11	3.49235	2.64145
Nc-1-IP_pe	chr11	2177052	2177541	490	2177340	14	6.67814	4.02073
Nc-1-IP_pe	chr11	2202464	2202783	320	2202732	13	5.9487	3.76157
Nc-1-IP_pe	chr11	2204869	2205230	362	2204984	14	6.28469	3.80898
Nc-1-IP_pe	chr11	2209234	2209509	276	2209429	10	4.87068	3.5387
Nc-1-IP_pe	chr11	2217037	2217358	322	2217148	10	5.14046	3.70342
Nc-1-IP_pe	chr11	2236599	2236925	327	2236722	10	4.99888	3.61677
Nc-1-IP_pe	chr11	2248319	2248602	284	2248425	10	4.99888	3.61677
Nc-1-IP_pe	chr11	2249048	2249317	270	2249286	9	4.46937	3.41876
Nc-1-IP_pe	chr11	2261122	2261533	412	2261330	8	3.22905	2.75004
Nc-1-IP_pe	chr11	2269575	2271631	2057	2269844	14	8.63073	5.12814
Nc-1-IP_pe	chr11	2277963	2278330	368	2278171	12	6.86906	4.44438
Nc-1-IP_pe	chr11	2296318	2297028	711	2296657	10	5.23357	3.76063
Nc-1-IP_pe	chr11	2299085	2299603	519	2299213	16	9.24948	5.10026
Nc-1-IP_pe	chr11	2300815	2301173	359	2300941	11	5.95085	4.05188
Nc-1-IP_pe	chr11	2302903	2303288	386	2303057	11	3.64839	2.72584
Nc-1-IP_pe	chr11	2304579	2305052	474	2304780	11	6.03439	4.10251
Nc-1-IP_pe	chr11	2314158	2314523	366	2314452	9	3.72192	2.95716
Nc-1-IP_pe	chr11	2316292	2316581	290	2316436	11	6.03439	4.10251
Nc-1-IP_pe	chr11	2317429	2317730	302	2317530	11	6.03439	4.10251
Nc-1-IP_pe	chr11	2328282	2328679	398	2328413	11	4.06403	2.95415
Nc-1-IP_pe	chr11	2329566	2329836	271	2329662	11	6.03439	4.10251
Nc-1-IP_pe	chr11	2331783	2332106	324	2332021	13	7.7352	4.78626
Nc-1-IP_pe	chr11	2345966	2346636	671	2346320	10	5.23357	3.76063
Nc-1-IP_pe	chr11	2360286	2360703	418	2360669	10	5.23357	3.76063

Nc-1-IP_pe	chr11	2362519	2362814	296	2362674	9	4.46937	3.41876
Nc-1-IP_pe	chr11	2363130	2363853	724	2363293	10	4.99888	3.61677
Nc-1-IP_pe	chr11	2368686	2369055	370	2368745	11	6.03439	4.10251
Nc-1-IP_pe	chr11	2377550	2377824	275	2377683	13	7.65891	4.74103
Nc-1-IP_pe	chr11	2383554	2383909	356	2383681	12	6.58417	4.27436
Nc-1-IP_pe	chr11	2384702	2384962	261	2384845	7	3.06442	2.73501
Nc-1-IP_pe	chr11	2385323	2386267	945	2386137	11	6.03439	4.10251
Nc-1-IP_pe	chr11	2387120	2387684	565	2387242	8	3.74505	3.07688
Nc-1-IP_pe	chr11	2392450	2392894	445	2392806	11	5.19376	3.60018
Nc-1-IP_pe	chr11	2395747	2396087	341	2395989	10	3.89064	2.95552
Nc-1-IP_pe	chr11	2396608	2397201	594	2396878	12	6.6717	4.32642
Nc-1-IP_pe	chr11	2399584	2400980	1397	2400155	13	4.41954	2.952
Nc-1-IP_pe	chr11	2402261	2402729	469	2402347	10	5.23357	3.76063
Nc-1-IP_pe	chr11	2403745	2404008	264	2403750	9	3.27326	2.68684
Nc-1-IP_pe	chr11	2405047	2406122	1076	2405445	9	4.46937	3.41876
Nc-1-IP_pe	chr11	2406944	2407207	264	2407072	11	6.03439	4.10251
Nc-1-IP_pe	chr11	2426692	2427286	595	2426799	10	4.47495	3.30017
Nc-1-IP_pe	chr11	2427748	2428015	268	2427870	11	4.54404	3.2242
Nc-1-IP_pe	chr11	2440767	2441708	942	2441306	8	3.74505	3.07688
Nc-1-IP_pe	chr11	2443930	2444295	366	2444122	8	3.51085	2.92785
Nc-1-IP_pe	chr11	2460955	2461260	306	2461239	8	3.74505	3.07688
Nc-1-IP_pe	chr11	2473324	2473955	632	2473326	9	3.27326	2.68684
Nc-1-IP_pe	chr11	2509495	2509880	386	2509761	10	3.89064	2.95552
Nc-1-IP_pe	chr11	2516006	2516462	457	2516239	10	5.23357	3.76063
Nc-1-IP_pe	chr11	2522106	2522409	304	2522227	11	6.03439	4.10251
Nc-1-IP_pe	chr11	2526182	2526550	369	2526425	10	5.23357	3.76063
Nc-1-IP_pe	chr11	2526902	2527154	253	2527022	9	4.25966	3.28797
Nc-1-IP_pe	chr11	2549961	2550342	382	2550142	10	4.47495	3.30017
Nc-1-IP_pe	chr11	2561106	2561464	359	2561409	10	4.77495	3.48064
Nc-1-IP_pe	chr11	2565844	2566549	706	2566322	13	7.34205	4.55444
Nc-1-IP_pe	chr11	2568310	2569204	895	2569077	11	6.03439	4.10251
Nc-1-IP_pe	chr11	2583561	2583840	280	2583656	10	4.99888	3.61677
Nc-1-IP_pe	chr11	2585452	2585763	312	2585587	13	5.36853	3.44651
Nc-1-IP_pe	chr11	2586892	2587179	288	2586996	14	8.63073	5.12814
Nc-1-IP_pe	chr11	2590362	2590660	299	2590515	11	5.52654	3.79706
Nc-1-IP_pe	chr11	2639962	2640343	382	2640168	13	6.62436	4.14003
Nc-1-IP_pe	chr11	2645398	2645988	591	2645510	12	5.61153	3.70794
Nc-1-IP_pe	chr11	2663388	2663736	349	2663591	9	4.25966	3.28797
Nc-1-IP_pe	chr11	2665840	2666239	400	2665974	10	3.89064	2.95552
Nc-1-IP_pe	chr11	2667737	2667991	255	2667818	9	4.46937	3.41876
Nc-1-IP_pe	chr11	2671556	2672015	460	2671698	9	3.72192	2.95716
Nc-1-IP_pe	chr11	2681357	2681706	350	2681641	8	3.74505	3.07688
Nc-1-IP_pe	chr11	2685243	2685643	401	2685520	13	4.41954	2.952
Nc-1-IP_pe	chr11	2692614	2692930	317	2692648	9	4.46937	3.41876
Nc-1-IP_pe	chr11	2699257	2699947	691	2699915	14	5.03085	3.16286

Nc-1-IP_pe	chr11	2708673	2708999	327	2708817	8	3.64414	3.01254
Nc-1-IP_pe	chr11	2713567	2713931	365	2713729	8	3.74505	3.07688
Nc-1-IP_pe	chr11	2714531	2714964	434	2714896	9	4.46937	3.41876
Nc-1-IP_pe	chr11	2718086	2718500	415	2718351	16	10.5028	5.81189
Nc-1-IP_pe	chr11	2721964	2722339	376	2722187	13	5.9487	3.76157
Nc-1-IP_pe	chr11	2734335	2734705	371	2734630	11	6.03439	4.10251
Nc-1-IP_pe	chr11	2738608	2739247	640	2738966	10	5.23357	3.76063
Nc-1-IP_pe	chr11	2748262	2748841	580	2748470	10	4.66672	3.41527
Nc-1-IP_pe	chr11	2752480	2752808	329	2752663	13	5.36853	3.44651
Nc-1-IP_pe	chr11	2769354	2770172	819	2769624	11	5.19376	3.60018
Nc-1-IP_pe	chr11	2770781	2771127	347	2771024	8	3.08755	2.66145
Nc-1-IP_pe	chr11	2771947	2772229	283	2772064	10	4.82246	3.50943
Nc-1-IP_pe	chr11	2774709	2774992	284	2774895	9	2.89249	2.46179
Nc-1-IP_pe	chr11	2777536	2778085	550	2777670	12	5.94619	3.9002
Nc-1-IP_pe	chr11	2779038	2779829	792	2779099	10	4.88691	3.54856
Nc-1-IP_pe	chr11	2780185	2780441	257	2780343	10	3.83201	2.92147
Nc-1-IP_pe	chr11	2781636	2782103	468	2781695	10	5.23357	3.76063
Nc-1-IP_pe	chr11	2788025	2788368	344	2788194	12	6.58417	4.27436
Nc-1-IP_pe	chr11	2790372	2790805	434	2790783	9	4.46937	3.41876
Nc-1-IP_pe	chr11	2792021	2792788	768	2792704	10	3.4604	2.70797
Nc-1-IP_pe	chr11	2800453	2800919	467	2800601	15	9.55381	5.47001
Nc-1-IP_pe	chr11	2802129	2802615	487	2802515	8	3.74505	3.07688
Nc-1-IP_pe	chr11	2811199	2811739	541	2811681	9	4.46937	3.41876
Nc-1-IP_pe	chr11	2812006	2813015	1010	2812036	11	4.54404	3.2242
Nc-1-IP_pe	chr11	2814949	2815218	270	2815059	9	4.46937	3.41876
Nc-1-IP_pe	chr11	2815477	2815952	476	2815847	9	4.25966	3.28797
Nc-1-IP_pe	chr11	2820864	2821346	483	2821080	12	5.23082	3.49289
Nc-1-IP_pe	chr11	2826147	2826610	464	2826463	11	5.7051	3.90381
Nc-1-IP_pe	chr11	2834958	2836193	1236	2835487	11	5.7051	3.90381
Nc-1-IP_pe	chr11	2844443	2845243	801	2844502	10	4.77495	3.48064
Nc-1-IP_pe	chr11	2845654	2846216	563	2845843	13	7.34205	4.55444
Nc-1-IP_pe	chr11	2850129	2850420	292	2850251	9	4.1889	3.24405
Nc-1-IP_pe	chr11	2855017	2855280	264	2855199	9	4.14522	3.217
Nc-1-IP_pe	chr11	2869675	2870047	373	2869877	8	3.08755	2.66145
Nc-1-IP_pe	chr11	2870609	2871779	1171	2870952	11	5.19376	3.60018
Nc-1-IP_pe	chr11	2878956	2879339	384	2879200	11	5.77463	3.94557
Nc-1-IP_pe	chr11	2880665	2880937	273	2880773	7	2.93007	2.64719
Nc-1-IP_pe	chr11	2885460	2885787	328	2885544	15	4.40338	2.77622
Nc-1-IP_pe	chr11	2890663	2891111	449	2890953	12	6.50787	4.22912
Nc-1-IP_pe	chr11	2891392	2891701	310	2891452	10	4.93609	3.57849
Nc-1-IP_pe	chr11	2892097	2893259	1163	2892892	14	7.75387	4.62
Nc-1-IP_pe	chr11	2896515	2897009	495	2896894	11	6.03439	4.10251
Nc-1-IP_pe	chr11	2898177	2898737	561	2898250	11	5.68685	3.89286
Nc-1-IP_pe	chr11	2901264	2901676	413	2901490	12	5.70894	3.7636
Nc-1-IP_pe	chr11	2907993	2908455	463	2908309	13	5.93256	3.75268

Nc-1-IP_pe	chr11	2909987	2910557	571	2910141	12	4.70082	3.20033
Nc-1-IP_pe	chr11	2921497	2922597	1101	2922491	9	4.4177	3.38645
Nc-1-IP_pe	chr11	2927953	2928228	276	2928105	13	4.02562	2.7544
Nc-1-IP_pe	chr11	2939793	2940545	753	2940193	10	3.4604	2.70797
Nc-1-IP_pe	chr11	2941051	2941993	943	2941878	11	5.97044	4.06374
Nc-1-IP_pe	chr11	2944705	2945255	551	2944798	11	4.00079	2.91908
Nc-1-IP_pe	chr11	2960252	2960538	287	2960401	12	6.86906	4.44438
Nc-1-IP_pe	chr11	2963262	2963834	573	2963757	9	4.25966	3.28797
Nc-1-IP_pe	chr11	2991836	2992089	254	2992023	13	3.67377	2.58159
Nc-1-IP_pe	chr11	3010365	3011118	754	3011065	10	5.23357	3.76063
Nc-1-IP_pe	chr11	3014144	3014522	379	3014386	10	5.23357	3.76063
Nc-1-IP_pe	chr11	3026855	3027118	264	3026999	12	6.86906	4.44438
Nc-1-IP_pe	chr11	3030420	3030742	323	3030440	10	3.89064	2.95552
Nc-1-IP_pe	chr11	3039108	3039591	484	3039242	8	3.08755	2.66145
Nc-1-IP_pe	chr11	3049462	3049795	334	3049482	10	4.51818	3.32604
Nc-1-IP_pe	chr11	3056791	3057515	725	3057189	16	6.51374	3.67221
Nc-1-IP_pe	chr11	3060288	3060781	494	3060349	12	5.23082	3.49289
Nc-1-IP_pe	chr11	3065223	3065496	274	3065348	12	6.86906	4.44438
Nc-1-IP_pe	chr11	3067573	3068204	632	3068125	11	6.03439	4.10251
Nc-1-IP_pe	chr11	3087818	3088258	441	3088108	8	3.45945	2.8953
Nc-1-IP_pe	chr11	3092918	3093884	967	3093651	13	7.02546	4.37016
Nc-1-IP_pe	chr11	3094203	3094698	496	3094542	16	9.90554	5.46889
Nc-1-IP_pe	chr11	3103103	3103389	287	3103229	10	5.23357	3.76063
Nc-1-IP_pe	chr11	3109575	3109841	267	3109841	8	2.93592	2.56703
Nc-1-IP_pe	chr11	3126452	3126808	357	3126637	13	7.19153	4.46655
Nc-1-IP_pe	chr11	3133604	3133872	269	3133731	10	5.23357	3.76063
Nc-1-IP_pe	chr11	3136699	3137333	635	3136798	10	5.23357	3.76063
Nc-1-IP_pe	chr11	3137904	3138417	514	3138261	9	4.46937	3.41876
Nc-1-IP_pe	chr11	3139049	3139358	310	3139149	12	6.86906	4.44438
Nc-1-IP_pe	chr11	3147239	3147594	356	3147413	11	5.74187	3.92588
Nc-1-IP_pe	chr11	3148270	3148597	328	3148359	8	3.61691	2.99521
Nc-1-IP_pe	chr11	3151246	3151508	263	3151376	8	3.74505	3.07688
Nc-1-IP_pe	chr11	3153770	3154302	533	3153867	10	5.22943	3.75809
Nc-1-IP_pe	chr11	3156338	3156904	567	3156586	9	3.79246	3.00015
Nc-1-IP_pe	chr11	3157655	3158384	730	3158082	16	9.01614	4.97137
Nc-1-IP_pe	chr11	3164642	3165071	430	3164921	16	9.6187	5.30662
Nc-1-IP_pe	chr11	3170470	3171021	552	3170507	9	4.088	3.18162
Nc-1-IP_pe	chr11	3182449	3182717	269	3182497	9	4.46937	3.41876
Nc-1-IP_pe	chr11	3220020	3220325	306	3220038	8	3.0402	2.6319
Nc-1-IP_pe	chr11	3225351	3226177	827	3225464	11	5.7051	3.90381
Nc-1-IP_pe	chr11	3227382	3227915	534	3227458	8	3.74505	3.07688
Nc-1-IP_pe	chr11	3257950	3258238	289	3258053	10	5.23357	3.76063
Nc-1-IP_pe	chr11	3270960	3271378	419	3271136	10	3.08907	2.49869
Nc-1-IP_pe	chr11	3281038	3281314	277	3281300	8	3.08755	2.66145
Nc-1-IP_pe	chr11	3338695	3338971	277	3338896	10	5.23357	3.76063

Nc-1-IP_pe	chr11	3346536	3346811	276	3346734	9	4.46937	3.41876
Nc-1-IP_pe	chr11	3357364	3357832	469	3357512	12	6.86906	4.44438
Nc-1-IP_pe	chr11	3397299	3397585	287	3397452	8	3.74505	3.07688
Nc-1-IP_pe	chr11	3406486	3406770	285	3406694	10	5.23357	3.76063
Nc-1-IP_pe	chr11	3439325	3439609	285	3439366	7	3.06442	2.73501
Nc-1-IP_pe	chr11	3440417	3440808	392	3440666	10	3.4604	2.70797
Nc-1-IP_pe	chr11	3480570	3480834	265	3480764	9	4.46937	3.41876
Nc-1-IP_pe	chr11	3498437	3498713	277	3498604	8	3.74505	3.07688
Nc-1-IP_pe	chr11	3567980	3568245	266	3568165	10	5.23357	3.76063
Nc-1-IP_pe	chr11	3576527	3576839	313	3576587	8	3.56332	2.96115
Nc-1-IP_pe	chr11	3579666	3580096	431	3579799	15	7.75613	4.45384
Nc-1-IP_pe	chr11	3625055	3625333	279	3625258	9	4.46937	3.41876
Nc-1-IP_pe	chr11	3661856	3662213	358	3662206	10	3.08907	2.49869
Nc-1-IP_pe	chr11	3787316	3787798	483	3787427	11	6.03439	4.10251
Nc-1-IP_pe	chr11	3788470	3788747	278	3788533	8	3.74505	3.07688
Nc-1-IP_pe	chr11	3826705	3827466	762	3826781	8	3.74505	3.07688
Nc-1-IP_pe	chr11	3867758	3868011	254	3867942	10	5.23357	3.76063
Nc-1-IP_pe	chr11	3999670	4000045	376	3999994	7	3.06442	2.73501
Nc-1-IP_pe	chr11	4016518	4017149	632	4016943	11	6.03439	4.10251
Nc-1-IP_pe	chr11	4094883	4095463	581	4095169	12	5.23082	3.49289
Nc-1-IP_pe	chr11	4116122	4116590	469	4116468	8	3.74505	3.07688
Nc-1-IP_pe	chr11	4166213	4166472	260	4166447	8	3.264	2.772
Nc-1-IP_pe	chr11	4168264	4168594	331	4168498	8	3.74505	3.07688
Nc-1-IP_pe	chr11	4282574	4282852	279	4282725	9	4.05981	3.16422
Nc-1-IP_pe	chr11	4335067	4335630	564	4335251	12	4.70082	3.20033
Nc-1-IP_pe	chr11	4431254	4431520	267	4431488	8	3.56021	2.95918
Nc-1-IP_pe	chr11	4531883	4532312	430	4531914	10	5.23357	3.76063
Nc-1-IP_pe	chr11	4601871	4602280	410	4601944	9	4.46937	3.41876
Nc-1-IP_pe	chr11	4653088	4653419	332	4653316	9	4.46937	3.41876
Nc-1-IP_pe	chr11	4681963	4682231	269	4682103	8	3.74505	3.07688
Nc-1-IP_pe	chr11	4722601	4722877	277	4722641	7	3.06442	2.73501
Nc-1-IP_pe	chr11	5422563	5422848	286	5422755	11	5.77463	3.94557
Nc-1-IP_pe	chr11	5544862	5545147	286	5544945	9	4.46937	3.41876
Nc-1-IP_pe	chr11	5929491	5929801	311	5929639	11	6.03439	4.10251
Nc-1-IP_pe	chr11	5930633	5930956	324	5930701	8	3.74505	3.07688
Nc-1-IP_pe	chr11	5960739	5961295	557	5961116	12	6.86906	4.44438
Nc-1-IP_pe	chr11	6210092	6210367	276	6210198	13	5.9487	3.76157
Nc-1-IP_pe	chr11	6217355	6217822	468	6217441	11	6.03439	4.10251
Nc-1-IP_pe	chr11	6222368	6222891	524	6222435	8	3.74505	3.07688
Nc-1-IP_pe	chr11	6259808	6260136	329	6259910	10	5.23357	3.76063
Nc-1-IP_pe	chr11	6401109	6402210	1102	6401220	11	6.03439	4.10251
Nc-1-IP_pe	chr11	6404880	6405188	309	6405077	10	5.23357	3.76063
Nc-1-IP_pe	chr11	6411076	6411742	667	6411134	10	3.89064	2.95552
Nc-1-IP_pe	chr11	6412838	6413128	291	6412971	12	5.84943	3.84431
Nc-1-IP_pe	chr11	6418445	6419334	890	6418940	18	11.6181	6.01202

Nc-1-IP_pe	chr11	6437036	6437570	535	6437148	11	6.03439	4.10251
Nc-1-IP_pe	chr11	6473533	6474392	860	6474188	11	5.77463	3.94557
Nc-1-IP_pe	chr11	6481287	6482333	1047	6481853	15	6.19428	3.63445
Nc-1-IP_pe	chr11	6497150	6497415	266	6497246	9	3.72192	2.95716
Nc-1-IP_pe	chr11	6544835	6545239	405	6545013	10	3.89064	2.95552
Nc-1-IP_pe	chr11	6557811	6558286	476	6557990	13	7.7352	4.78626
Nc-1-IP_pe	chr11	6566014	6566433	420	6566173	8	3.74505	3.07688
Nc-1-IP_pe	chr11	6617471	6617772	302	6617609	9	4.46937	3.41876
Nc-1-IP_pe	chr11	6622837	6623102	266	6622856	8	3.74505	3.07688
Nc-1-IP_pe	chr11	6626067	6626410	344	6626311	10	5.23357	3.76063
Nc-1-IP_pe	chr11	6626778	6627256	479	6627058	11	6.03439	4.10251
Nc-1-IP_pe	chr11	6646199	6647143	945	6647055	10	5.23357	3.76063
Nc-1-IP_pe	chr11	6654799	6655183	385	6655029	9	4.46937	3.41876
Nc-1-IP_pe	chr11	7019794	7020102	309	7019933	13	7.7352	4.78626
Nc-1-IP_pe	chr11	7536951	7537208	258	7537100	10	5.23357	3.76063
Nc-1-IP_pe	chr11	7540208	7540536	329	7540361	9	4.46937	3.41876
Nc-1-IP_pe	chr11	7547179	7547650	472	7547278	8	3.74505	3.07688
Nc-1-IP_pe	chr11	7607006	7607394	389	7607168	10	5.23357	3.76063
Nc-1-IP_pe	chr11	7653207	7653595	389	7653505	9	3.72192	2.95716
Nc-1-IP_pe	chr11	7665507	7665859	353	7665576	7	2.9043	2.63038
Nc-1-IP_pe	chr11	7667575	7667855	281	7667702	13	7.7352	4.78626
Nc-1-IP_pe	chr11	7673909	7674233	325	7674022	10	5.23357	3.76063
Nc-1-IP_pe	chr11	7780977	7781276	300	7781166	11	6.03439	4.10251
Nc-1-IP_pe	chr11	7987113	7987670	558	7987566	19	9.15219	4.54307
Nc-1-IP_pe	chr11	8031051	8031303	253	8031233	10	5.23357	3.76063
Nc-1-IP_pe	chr11	8039598	8039865	268	8039758	11	5.10577	3.5486
Nc-1-IP_pe	chr11	8059092	8059364	273	8059252	8	3.74505	3.07688
Nc-1-IP_pe	chr11	8061346	8061760	415	8061449	8	3.74505	3.07688
Nc-1-IP_pe	chr11	8062862	8063192	331	8063005	9	4.25966	3.28797
Nc-1-IP_pe	chr11	8066745	8067001	257	8066896	11	4.74697	3.34038
Nc-1-IP_pe	chr11	8095637	8095941	305	8095713	8	3.08755	2.66145
Nc-1-IP_pe	chr11	8100312	8100610	299	8100363	8	3.51085	2.92785
Nc-1-IP_pe	chr11	8168728	8169088	361	8168955	11	6.03439	4.10251
Nc-1-IP_pe	chr11	8204120	8204374	255	8204313	10	5.10552	3.682
Nc-1-IP_pe	chr11	8228274	8228589	316	8228505	9	3.27326	2.68684
Nc-1-IP_pe	chr11	8230052	8230344	293	8230230	11	3.64839	2.72584
Nc-1-IP_pe	chr11	8231159	8231771	613	8231548	10	4.62142	3.388
Nc-1-IP_pe	chr11	8232691	8233089	399	8233006	13	6.92473	4.312
Nc-1-IP_pe	chr11	8234618	8235049	432	8234884	14	7.75387	4.62
Nc-1-IP_pe	chr11	8237388	8237813	426	8237603	14	8.29543	4.93196
Nc-1-IP_pe	chr11	8243380	8243916	537	8243480	10	5.23357	3.76063
Nc-1-IP_pe	chr11	8248758	8249309	552	8249159	10	3.89064	2.95552
Nc-1-IP_pe	chr11	8257323	8257856	534	8257406	9	3.72192	2.95716
Nc-1-IP_pe	chr11	8267898	8268174	277	8268150	7	3.06442	2.73501
Nc-1-IP_pe	chr11	8291710	8292268	559	8291950	13	5.9487	3.76157

Nc-1-IP_pe	chr11	8298073	8298407	335	8298245	9	4.46937	3.41876
Nc-1-IP_pe	chr11	8313422	8313720	299	8313602	8	3.74505	3.07688
Nc-1-IP_pe	chr11	8326719	8327009	291	8326855	8	3.74505	3.07688
Nc-1-IP_pe	chr11	8336034	8336306	273	8336202	10	4.77495	3.48064
Nc-1-IP_pe	chr11	8522752	8523565	814	8523146	10	5.23357	3.76063
Nc-1-IP_pe	chr11	8593849	8594125	277	8594038	14	6.69566	4.03025
Nc-1-IP_pe	chr11	8678821	8679108	288	8678867	8	3.74505	3.07688
Nc-1-IP_pe	chr11	8682423	8682881	459	8682672	12	5.77527	3.80164
Nc-1-IP_pe	chr11	8701856	8702188	333	8701995	15	6.78905	3.93887
Nc-1-IP_pe	chr11	8707238	8708006	769	8707362	11	5.52654	3.79706
Nc-1-IP_pe	chr11	8714305	8714562	258	8714537	8	3.58997	2.97809
Nc-1-IP_pe	chr11	8726542	8726859	318	8726804	11	6.03439	4.10251
Nc-1-IP_pe	chr11	8741170	8741449	280	8741397	9	4.46937	3.41876
Nc-1-IP_pe	chr11	8766243	8766521	279	8766359	11	6.03439	4.10251
Nc-1-IP_pe	chr11	8807948	8808250	303	8808040	9	4.46937	3.41876
Nc-1-IP_pe	chr11	8828329	8828694	366	8828547	11	6.03439	4.10251
Nc-1-IP_pe	chr11	8889476	8889869	394	8889641	7	3.06442	2.73501
Nc-1-IP_pe	chr11	8910343	8910697	355	8910541	11	5.77463	3.94557
Nc-1-IP_pe	chr11	8929291	8929566	276	8929410	10	4.39571	3.25288
Nc-1-IP_pe	chr11	8936496	8936995	500	8936861	12	4.24064	2.95299
Nc-1-IP_pe	chr11	8964274	8964729	456	8964407	16	8.26996	4.56762
Nc-1-IP_pe	chr11	8977633	8978274	642	8978146	13	7.7352	4.78626
Nc-1-IP_pe	chr11	8978803	8979422	620	8979008	15	9.55381	5.47001
Nc-1-IP_pe	chr11	9003090	9003714	625	9003552	11	5.10577	3.5486
Nc-1-IP_pe	chr11	9030913	9031183	271	9030962	10	4.93609	3.57849
Nc-1-IP_pe	chr11	9047344	9047609	266	9047486	10	5.23357	3.76063
Nc-1-IP_pe	chr11	9069272	9069576	305	9069343	11	5.52654	3.79706
Nc-1-IP_pe	chr11	9091076	9091401	326	9091184	13	7.7352	4.78626
Nc-1-IP_pe	chr11	9092216	9092494	279	9092259	10	3.4604	2.70797
Nc-1-IP_pe	chr11	9189037	9189294	258	9189079	8	3.74505	3.07688
Nc-1-IP_pe	chr11	9265423	9266053	631	9265912	11	5.10577	3.5486
Nc-1-IP_pe	chr11	9266713	9266990	278	9266826	7	3.06442	2.73501
Nc-1-IP_pe	chr11	9293227	9293808	582	9293673	9	4.46937	3.41876
Nc-1-IP_pe	chr11	9306136	9306634	499	9306242	9	3.66792	2.92434
Nc-1-IP_pe	chr11	9408917	9409204	288	9409168	8	3.08755	2.66145
Nc-1-IP_pe	chr11	9421511	9421922	412	9421722	10	4.99888	3.61677
Nc-1-IP_pe	chr11	9472038	9472549	512	9472492	9	4.46937	3.41876
Nc-1-IP_pe	chr11	9526789	9527187	399	9527047	8	3.74505	3.07688
Nc-1-IP_pe	chr11	9535660	9535992	333	9535837	11	6.03439	4.10251
Nc-1-IP_pe	chr11	9572941	9573325	385	9573169	10	4.77495	3.48064
Nc-1-IP_pe	chr11	9606148	9606411	264	9606280	9	4.20361	3.25317
Nc-1-IP_pe	chr11	9607279	9607644	366	9607376	11	6.03439	4.10251
Nc-1-IP_pe	chr11	9613419	9614383	965	9614231	11	4.54404	3.2242
Nc-1-IP_pe	chr11	9663713	9664210	498	9663936	9	4.46937	3.41876
Nc-1-IP_pe	chr11	9758655	9758971	317	9758902	14	5.51541	3.4073

Nc-1-IP_pe	chr11	9983608	9983871	264	9983682	12	5.01934	3.37518
Nc-1-IP_pe	chr11	10134724	10135150	427	10134813	7	2.9043	2.63038
Nc-1-IP_pe	chr11	10155969	10156444	476	10156205	10	5.23357	3.76063
Nc-1-IP_pe	chr11	10173152	10173594	443	10173213	8	3.74505	3.07688
Nc-1-IP_pe	chr11	10294033	10294483	451	10294374	14	7.9745	4.74633
Nc-1-IP_pe	chr11	10306593	10307047	455	10306885	9	3.85693	3.03955
Nc-1-IP_pe	chr11	10307419	10307821	403	10307736	10	4.20113	3.13749
Nc-1-IP_pe	chr11	10366684	10367137	454	10366994	9	4.46937	3.41876
Nc-1-IP_pe	chr11	10396725	10397302	578	10396909	8	3.74505	3.07688
Nc-1-IP_pe	chr11	10442571	10442827	257	10442694	9	4.46937	3.41876
Nc-1-IP_pe	chr11	10473632	10473956	325	10473727	9	4.46937	3.41876
Nc-1-IP_pe	chr11	10483204	10483739	536	10483271	10	5.23357	3.76063
Nc-1-IP_pe	chr11	10491007	10491314	308	10491041	12	4.24064	2.95299
Nc-1-IP_pe	chr11	10493157	10493720	564	10493477	16	10.2925	5.69036
Nc-1-IP_pe	chr11	10496813	10497580	768	10496971	10	5.10552	3.682
Nc-1-IP_pe	chr11	10499839	10500152	314	10499896	11	5.3563	3.696
Nc-1-IP_pe	chr11	10541123	10541492	370	10541150	10	3.4604	2.70797
Nc-1-IP_pe	chr11	10566011	10566290	280	10566142	9	4.25966	3.28797
Nc-1-IP_pe	chr11	10586489	10586769	281	10586631	9	4.46937	3.41876
Nc-1-IP_pe	chr11	10607397	10607849	453	10607487	9	4.46937	3.41876
Nc-1-IP_pe	chr11	10653647	10654286	640	10654210	12	6.86906	4.44438
Nc-1-IP_pe	chr11	10693468	10693781	314	10693663	9	4.25966	3.28797
Nc-1-IP_pe	chr11	10694079	10694500	422	10694375	11	6.03439	4.10251
Nc-1-IP_pe	chr11	10751065	10751410	346	10751223	12	5.61153	3.70794
Nc-1-IP_pe	chr11	10786095	10786399	305	10786351	13	4.41954	2.952
Nc-1-IP_pe	chr11	10808291	10809616	1326	10808466	13	5.71736	3.63481
Nc-1-IP_pe	chr11	10903325	10903603	279	10903451	11	4.54404	3.2242
Nc-1-IP_pe	chr11	10987225	10987639	415	10987517	7	3.06442	2.73501
Nc-1-IP_pe	chr11	10993042	10993399	358	10993140	8	3.74505	3.07688
Nc-1-IP_pe	chr11	11038548	11038851	304	11038623	9	4.05981	3.16422
Nc-1-IP_pe	chr11	11067626	11067906	281	11067789	9	4.46937	3.41876
Nc-1-IP_pe	chr11	11269666	11270248	583	11269729	8	3.74505	3.07688
Nc-1-IP_pe	chr11	11282388	11282645	258	11282467	10	5.23357	3.76063
Nc-1-IP_pe	chr11	11283144	11283443	300	11283332	10	5.23357	3.76063
Nc-1-IP_pe	chr11	11343904	11344171	268	11344064	9	4.46937	3.41876
Nc-1-IP_pe	chr11	11345548	11345983	436	11345951	8	3.08755	2.66145
Nc-1-IP_pe	chr11	11404731	11404984	254	11404863	8	3.08755	2.66145
Nc-1-IP_pe	chr11	11428308	11428569	262	11428558	7	3.06442	2.73501
Nc-1-IP_pe	chr11	11484707	11485035	329	11484861	9	4.46937	3.41876
Nc-1-IP_pe	chr11	11510998	11511310	313	11511116	9	4.46937	3.41876
Nc-1-IP_pe	chr11	11519629	11520062	434	11519950	11	6.03439	4.10251
Nc-1-IP_pe	chr11	11811338	11811713	376	11811595	8	3.56021	2.95918
Nc-1-IP_pe	chr11	11966616	11966922	307	11966861	14	7.42849	4.43574
Nc-1-IP_pe	chr11	11967263	11967696	434	11967565	8	3.74505	3.07688
Nc-1-IP_pe	chr11	11973851	11974337	487	11974245	10	5.23357	3.76063

Nc-1-IP_pe	chr11	11975546	11975954	409	11975697	12	6.86906	4.44438
Nc-1-IP_pe	chr11	11977175	11977863	689	11977663	10	4.99888	3.61677
Nc-1-IP_pe	chr11	11982138	11982534	397	11982377	9	4.46937	3.41876
Nc-1-IP_pe	chr11	12008783	12009256	474	12009117	11	5.77463	3.94557
Nc-1-IP_pe	chr11	12010075	12010440	366	12010415	9	3.27326	2.68684
Nc-1-IP_pe	chr11	12052443	12052836	394	12052790	8	3.74505	3.07688
Nc-1-IP_pe	chr11	12061966	12062221	256	12062109	10	5.23357	3.76063
Nc-1-IP_pe	chr11	12137170	12137533	364	12137335	9	4.46937	3.41876
Nc-1-IP_pe	chr11	12159932	12160185	254	12160157	10	4.39571	3.25288
Nc-1-IP_pe	chr11	12162356	12162610	255	12162563	11	5.77463	3.94557
Nc-1-IP_pe	chr11	12218208	12218533	326	12218335	13	7.7352	4.78626
Nc-1-IP_pe	chr11	12222117	12222672	556	12222225	9	4.46937	3.41876
Nc-1-IP_pe	chr11	12223328	12223605	278	12223479	11	6.03439	4.10251
Nc-1-IP_pe	chr11	12224462	12224911	450	12224601	10	5.23357	3.76063
Nc-1-IP_pe	chr11	12245109	12245564	456	12245315	12	6.86906	4.44438
Nc-1-IP_pe	chr11	12255712	12256080	369	12255875	14	8.29543	4.93196
Nc-1-IP_pe	chr11	12268944	12269314	371	12269195	9	4.46937	3.41876
Nc-1-IP_pe	chr11	12271879	12272226	348	12272129	12	5.77527	3.80164
Nc-1-IP_pe	chr11	12280982	12281240	259	12281158	11	3.28475	2.53029
Nc-1-IP_pe	chr11	12356499	12356894	396	12356721	8	3.74505	3.07688
Nc-1-IP_pe	chr11	12490016	12490296	281	12490259	7	3.06442	2.73501
Nc-1-IP_pe	chr11	12500339	12500659	321	12500615	8	3.74505	3.07688
Nc-1-IP_pe	chr11	12541016	12541766	751	12541305	9	4.46937	3.41876
Nc-1-IP_pe	chr11	12681589	12681973	385	12681711	9	4.46937	3.41876
Nc-1-IP_pe	chr11	12724637	12724925	289	12724776	11	6.03439	4.10251
Nc-1-IP_pe	chr11	12785887	12786313	427	12785996	9	4.46937	3.41876
Nc-1-IP_pe	chr11	12814160	12814451	292	12814301	9	2.89249	2.46179
Nc-1-IP_pe	chr11	12882659	12883074	416	12882894	8	3.74505	3.07688
Nc-1-IP_pe	chr11	12908198	12908468	271	12908428	11	6.03439	4.10251
Nc-1-IP_pe	chr11	13010227	13010657	431	13010386	12	4.63088	3.16234
Nc-1-IP_pe	chr11	13297997	13298256	260	13298168	10	4.93609	3.57849
Nc-1-IP_pe	chr11	13315609	13316036	428	13315837	9	4.46937	3.41876
Nc-1-IP_pe	chr11	13462625	13462974	350	13462741	10	4.62142	3.388
Nc-1-IP_pe	chr11	13534019	13534315	297	13534278	8	3.74505	3.07688
Nc-1-IP_pe	chr11	13582293	13582636	344	13582465	8	3.74505	3.07688
Nc-1-IP_pe	chr11	13665225	13665543	319	13665462	10	5.23357	3.76063
Nc-1-IP_pe	chr11	13668976	13669279	304	13669097	11	5.3563	3.696
Nc-1-IP_pe	chr11	13963931	13964359	429	13964181	13	5.36853	3.44651
Nc-1-IP_pe	chr11	13991815	13992069	255	13991882	9	4.46937	3.41876
Nc-1-IP_pe	chr11	14091271	14092076	806	14091893	11	5.10577	3.5486
Nc-1-IP_pe	chr11	14133662	14133971	310	14133909	9	4.46937	3.41876
Nc-1-IP_pe	chr11	14154133	14154550	418	14154422	11	6.03439	4.10251
Nc-1-IP_pe	chr11	14242164	14242489	326	14242394	8	3.74505	3.07688
Nc-1-IP_pe	chr11	14259182	14259675	494	14259340	12	6.58417	4.27436
Nc-1-IP_pe	chr11	14519951	14520399	449	14520173	9	3.72192	2.95716

Nc-1-IP_pe	chr11	14590887	14591271	385	14591060	10	5.23357	3.76063
Nc-1-IP_pe	chr11	14643774	14644073	300	14644035	10	4.99888	3.61677
Nc-1-IP_pe	chr11	14716331	14716733	403	14716434	12	3.47963	2.55766
Nc-1-IP_pe	chr11	14801521	14801782	262	14801710	11	6.03439	4.10251
Nc-1-IP_pe	chr11	14806301	14806726	426	14806490	9	4.46937	3.41876
Nc-1-IP_pe	chr11	14892007	14892375	369	14892142	10	3.83201	2.92147
Nc-1-IP_pe	chr11	14938846	14939137	292	14939104	9	4.46937	3.41876
Nc-1-IP_pe	chr11	15235157	15235460	304	15235268	9	4.46937	3.41876
Nc-1-IP_pe	chr11	15375247	15375510	264	15375378	12	6.58417	4.27436
Nc-1-IP_pe	chr11	15412905	15413160	256	15412971	8	3.74505	3.07688
Nc-1-IP_pe	chr11	15527602	15527869	268	15527705	8	3.74505	3.07688
Nc-1-IP_pe	chr11	15594469	15594868	400	15594757	11	5.10577	3.5486
Nc-1-IP_pe	chr11	15900744	15901174	431	15900883	9	4.46937	3.41876
Nc-1-IP_pe	chr11	15905268	15905560	293	15905527	10	3.4604	2.70797
Nc-1-IP_pe	chr11	15939287	15939702	416	15939630	11	4.06403	2.95415
Nc-1-IP_pe	chr11	16070790	16071065	276	16070969	8	3.74505	3.07688
Nc-1-IP_pe	chr11	16141795	16142126	332	16141878	8	3.74505	3.07688
Nc-1-IP_pe	chr11	16484102	16484450	349	16484411	7	3.06442	2.73501
Nc-1-IP_pe	chr11	16495228	16495839	612	16495458	11	6.03439	4.10251
Nc-1-IP_pe	chr11	16520660	16521040	381	16520986	9	2.89249	2.46179
Nc-1-IP_pe	chr11	16604130	16604672	543	16604621	9	4.46937	3.41876
Nc-1-IP_pe	chr11	16780162	16780427	266	16780309	13	7.7352	4.78626
Nc-1-IP_pe	chr11	16782526	16782802	277	16782584	9	3.66792	2.92434
Nc-1-IP_pe	chr11	16783532	16783854	323	16783610	10	4.39571	3.25288
Nc-1-IP_pe	chr11	16786223	16786523	301	16786449	12	4.70082	3.20033
Nc-1-IP_pe	chr11	16790807	16791204	398	16790933	9	3.72192	2.95716
Nc-1-IP_pe	chr11	16847211	16847512	302	16847272	11	5.77463	3.94557
Nc-1-IP_pe	chr11	16850049	16850468	420	16850190	13	7.7352	4.78626
Nc-1-IP_pe	chr11	16888060	16888357	298	16888167	13	6.62436	4.14003
Nc-1-IP_pe	chr11	16906995	16907471	477	16907039	21	4.61597	2.49283
Nc-1-IP_pe	chr11	16923318	16923876	559	16923682	11	5.10577	3.5486
Nc-1-IP_pe	chr11	16925276	16925812	537	16925581	16	6.80394	3.81462
Nc-1-IP_pe	chr11	16947437	16947706	270	16947460	13	5.03208	3.26819
Nc-1-IP_pe	chr11	16948656	16949105	450	16948794	14	6.69566	4.03025
Nc-1-IP_pe	chr11	16959982	16960355	374	16960075	16	7.39088	4.10945
Nc-1-IP_pe	chr11	16964463	16964734	272	16964541	18	7.95467	4.11191
Nc-1-IP_pe	chr11	16967463	16967752	290	16967715	13	4.86362	3.18015
Nc-1-IP_pe	chr11	16978866	16979183	318	16979114	17	7.09531	3.83101
Nc-1-IP_pe	chr11	16992300	16992607	308	16992375	14	5.51541	3.4073
Nc-1-IP_pe	chr11	17033829	17034108	280	17033970	15	7.46994	4.29894
Nc-1-IP_pe	chr11	17034379	17034634	256	17034510	14	7.2429	4.33177
Nc-1-IP_pe	chr11	17035851	17036125	275	17036118	10	3.03453	2.46828
Nc-1-IP_pe	chr11	17047713	17048006	294	17047923	11	5.08426	3.53601
Nc-1-IP_pe	chr11	17048844	17049106	263	17049007	12	5.23082	3.49289
Nc-1-IP_pe	chr11	17049771	17050340	570	17050201	16	7.97877	4.4137

Nc-1-IP_pe	chr11	17050901	17051488	588	17050956	14	5.06689	3.18081
Nc-1-IP_pe	chr11	17064074	17064418	345	17064171	14	7.09041	4.24699
Nc-1-IP_pe	chr11	17066031	17066312	282	17066213	12	5.39344	3.58426
Nc-1-IP_pe	chr11	17066840	17067440	601	17067277	11	4.11366	2.98176
Nc-1-IP_pe	chr11	17078189	17078750	562	17078285	15	5.22925	3.16116
Nc-1-IP_pe	chr11	17081591	17081992	402	17081710	11	3.64839	2.72584
Nc-1-IP_pe	chr11	17105478	17106082	605	17105901	14	8.06563	4.79881
Nc-1-IP_pe	chr11	17120808	17121277	470	17120940	11	6.03439	4.10251
Nc-1-IP_pe	chr11	17162979	17163275	297	17163124	13	4.86362	3.18015
Nc-1-IP_pe	chr11	17183596	17183860	265	17183711	8	3.64414	3.01254
Nc-1-IP_pe	chr11	17190052	17190478	427	17190186	13	6.45312	4.04299
Nc-1-IP_pe	chr11	17195511	17195960	450	17195575	16	4.25252	2.63824
Nc-1-IP_pe	chr11	17198688	17198984	297	17198846	11	5.00808	3.49156
Nc-1-IP_pe	chr11	17201068	17201360	293	17201134	11	5.16212	3.58161
Nc-1-IP_pe	chr11	17207671	17208059	389	17207810	21	5.2077	2.70109
Nc-1-IP_pe	chr11	17220389	17220676	288	17220562	10	3.89064	2.95552
Nc-1-IP_pe	chr11	17224281	17224867	587	17224360	10	4.53273	3.33475
Nc-1-IP_pe	chr11	17255617	17255921	305	17255707	15	6.78905	3.93887
Nc-1-IP_pe	chr11	17264432	17264719	288	17264560	18	5.72638	3.11298
Nc-1-IP_pe	chr11	17273951	17274400	450	17274294	12	4.52227	3.10362
Nc-1-IP_pe	chr11	17292939	17293193	255	17293049	15	7.19874	4.15407
Nc-1-IP_pe	chr11	17312352	17312772	421	17312580	12	5.23082	3.49289
Nc-1-IP_pe	chr11	17315988	17316288	301	17316146	12	6.86906	4.44438
Nc-1-IP_pe	chr11	17353364	17353852	489	17353460	21	8.6741	4.05679
Nc-1-IP_pe	chr11	17355879	17356411	533	17355974	11	4.42758	3.15806
Nc-1-IP_pe	chr11	17381882	17382239	358	17382077	14	4.8399	3.06836
Nc-1-IP_pe	chr11	17382598	17383019	422	17382722	14	3.00733	2.21111
Nc-1-IP_pe	chr11	17384134	17384533	400	17384262	19	8.4638	4.21715
Nc-1-IP_pe	chr11	17386987	17388289	1303	17387383	15	6.30504	3.69041
Nc-1-IP_pe	chr11	17388599	17390252	1654	17388888	18	5.33197	2.94862
Nc-1-IP_pe	chr11	17390831	17391186	356	17391025	16	6.8987	3.86161
Nc-1-IP_pe	chr11	17393047	17393634	588	17393553	13	3.67377	2.58159
Nc-1-IP_pe	chr11	17400131	17400385	255	17400221	13	3.67377	2.58159
Nc-1-IP_pe	chr11	17402857	17403200	344	17403174	15	5.20142	3.14789
Nc-1-IP_pe	chr11	17406548	17406918	371	17406660	17	7.52577	4.03901
Nc-1-IP_pe	chr11	17408331	17409040	710	17408827	13	5.73236	3.64298
Nc-1-IP_pe	chr11	17415184	17415938	755	17415594	13	5.9487	3.76157
Nc-1-IP_pe	chr11	17420294	17420606	313	17420475	10	3.89064	2.95552
Nc-1-IP_pe	chr11	17423175	17423622	448	17423287	13	5.9487	3.76157
Nc-1-IP_pe	chr11	17427038	17427627	590	17427351	14	6.49348	3.92083
Nc-1-IP_pe	chr11	17428478	17428901	424	17428710	16	6.33285	3.58457
Nc-1-IP_pe	chr11	17429832	17431027	1196	17430311	17	9.60544	5.10888
Nc-1-IP_pe	chr11	17460332	17461322	991	17461125	16	7.27441	4.05022
Nc-1-IP_pe	chr11	17481884	17482202	319	17482006	12	5.5322	3.6628
Nc-1-IP_pe	chr11	17486037	17486340	304	17486093	19	9.15219	4.54307

Nc-1-IP_pe	chr11	17492679	17493214	536	17493038	12	3.16137	2.39719
Nc-1-IP_pe	chr11	17495007	17495289	283	17495124	16	5.28625	3.09457
Nc-1-IP_pe	chr11	17499529	17499892	364	17499753	15	6.07859	3.57636
Nc-1-IP_pe	chr11	17501189	17501786	598	17501334	14	5.68531	3.49459
Nc-1-IP_pe	chr11	17502081	17502491	411	17502315	19	9.22155	4.57653
Nc-1-IP_pe	chr11	17504802	17505135	334	17504881	12	4.28158	2.97474
Nc-1-IP_pe	chr11	17509311	17509658	348	17509322	10	3.60969	2.79325
Nc-1-IP_pe	chr11	17513209	17513509	301	17513304	12	5.27426	3.51722
Nc-1-IP_pe	chr11	17517044	17517586	543	17517386	15	6.73062	3.90854
Nc-1-IP_pe	chr11	17520055	17520415	361	17520062	13	3.67377	2.58159
Nc-1-IP_pe	chr11	17521540	17521805	266	17521641	14	5.63114	3.46667
Nc-1-IP_pe	chr11	17522858	17523128	271	17522966	16	5.82808	3.34463
Nc-1-IP_pe	chr11	17525638	17525944	307	17525709	14	6.61011	3.98382
Nc-1-IP_pe	chr11	17529551	17530099	549	17529733	12	4.89828	3.30838
Nc-1-IP_pe	chr11	17538862	17539253	392	17538994	10	3.76347	2.88178
Nc-1-IP_pe	chr11	17540391	17540777	387	17540500	11	3.28475	2.53029
Nc-1-IP_pe	chr11	17542985	17543391	407	17543029	11	4.42758	3.15806
Nc-1-IP_pe	chr11	17543652	17543933	282	17543917	14	3.26761	2.32785
Nc-1-IP_pe	chr11	17548820	17549078	259	17548995	14	5.51541	3.4073
Nc-1-IP_pe	chr11	17553117	17553719	603	17553272	16	7.8668	4.35507
Nc-1-IP_pe	chr11	17574347	17574975	629	17574888	13	4.90859	3.20357
Nc-1-IP_pe	chr11	17577069	17577433	365	17577257	13	5.4426	3.4862
Nc-1-IP_pe	chr11	17587747	17588000	254	17587901	10	3.83201	2.92147
Nc-1-IP_pe	chr11	17593453	17593862	410	17593514	11	4.50458	3.20175
Nc-1-IP_pe	chr11	17602764	17603627	864	17603553	15	6.48264	3.78084
Nc-1-IP_pe	chr11	17609567	17610001	435	17609853	11	3.95677	2.89474
Nc-1-IP_pe	chr11	17612048	17612515	468	17612239	14	6.44441	3.89444
Nc-1-IP_pe	chr11	17612899	17613183	285	17613027	14	5.838	3.57373
Nc-1-IP_pe	chr11	17633656	17634767	1112	17634363	17	7.6273	4.08876
Nc-1-IP_pe	chr11	17635200	17635871	672	17635367	11	3.28475	2.53029
Nc-1-IP_pe	chr11	17640695	17641282	588	17641209	12	5.23082	3.49289
Nc-1-IP_pe	chr11	17641661	17642293	633	17642042	13	5.71736	3.63481
Nc-1-IP_pe	chr11	17643093	17643363	271	17643161	15	7.02624	4.06292
Nc-1-IP_pe	chr11	17645300	17645565	266	17645351	15	6.19428	3.63445
Nc-1-IP_pe	chr11	17645862	17646301	440	17645943	13	4.90859	3.20357
Nc-1-IP_pe	chr11	17686171	17686426	256	17686240	10	4.24068	3.16086
Nc-1-IP_pe	chr11	17695661	17696171	511	17695928	14	6.55975	3.95658
Nc-1-IP_pe	chr11	17698302	17698654	353	17698449	14	6.49348	3.92083
Nc-1-IP_pe	chr11	17709263	17710084	822	17709893	16	8.64073	4.7666
Nc-1-IP_pe	chr11	17711038	17711462	425	17711279	18	8.37884	4.31591
Nc-1-IP_pe	chr11	17721057	17721724	668	17721532	14	5.57768	3.4392
Nc-1-IP_pe	chr11	17730856	17731333	478	17731099	19	7.84735	3.93486
Nc-1-IP_pe	chr11	17733831	17734142	312	17733980	16	7.84837	4.34545
Nc-1-IP_pe	chr11	17734428	17735190	763	17734607	14	5.98179	3.64885
Nc-1-IP_pe	chr11	17735946	17736638	693	17736147	16	6.95583	3.89005

Nc-1-IP_pe	chr11	17740668	17741140	473	17740735	10	3.29694	2.61535
Nc-1-IP_pe	chr11	17744675	17745192	518	17744961	11	3.68532	2.74592
Nc-1-IP_pe	chr11	17750001	17750321	321	17750161	15	5.70822	3.39286
Nc-1-IP_pe	chr11	17753151	17753580	430	17753221	13	5.36853	3.44651
Nc-1-IP_pe	chr11	17760528	17761057	530	17760663	16	4.96462	2.94973
Nc-1-IP_pe	chr11	17762083	17762619	537	17762272	13	5.82354	3.69281
Nc-1-IP_pe	chr11	17764750	17765302	553	17765256	13	4.41954	2.952
Nc-1-IP_pe	chr11	17767474	17767849	376	17767650	13	4.41954	2.952
Nc-1-IP_pe	chr11	17769871	17770754	884	17770123	15	6.48264	3.78084
Nc-1-IP_pe	chr11	17771496	17772244	749	17771817	22	11.8987	5.36917
Nc-1-IP_pe	chr11	17772704	17773062	359	17772914	14	5.03085	3.16286
Nc-1-IP_pe	chr11	17774628	17775214	587	17775004	16	5.82808	3.34463
Nc-1-IP_pe	chr11	17777056	17777505	450	17777480	12	3.83688	2.74114
Nc-1-IP_pe	chr11	17780115	17780496	382	17780463	13	3.95529	2.71958
Nc-1-IP_pe	chr11	17789005	17789268	264	17789098	17	7.02067	3.79543
Nc-1-IP_pe	chr11	17806751	17807067	317	17806880	13	5.51308	3.52412
Nc-1-IP_pe	chr11	17808107	17808465	359	17808422	10	3.70758	2.84953
Nc-1-IP_pe	chr11	17811966	17812568	603	17812171	11	4.25472	3.06062
Nc-1-IP_pe	chr11	17867693	17867981	289	17867825	13	3.67377	2.58159
Nc-1-IP_pe	chr11	17873726	17874102	377	17873932	19	7.84735	3.93486
Nc-1-IP_pe	chr11	17973020	17973444	425	17973252	16	5.37454	3.13479
Nc-1-IP_pe	chr11	18032619	18033252	634	18033149	16	8.26996	4.56762
Nc-1-IP_pe	chr11	18043439	18043837	399	18043499	11	4.06403	2.95415
Nc-1-IP_pe	chr11	18060924	18061191	268	18061040	14	7.28181	4.3535
Nc-1-IP_pe	chr11	18065317	18065782	466	18065544	15	7.37761	4.24941
Nc-1-IP_pe	chr11	18097580	18098064	485	18097773	15	4.7819	2.95039
Nc-1-IP_pe	chr11	18105730	18106461	732	18105991	26	10.439	4.19014
Nc-1-IP_pe	chr11	18118847	18119333	487	18119002	14	6.36383	3.85124
Nc-1-IP_pe	chr11	18120539	18120979	441	18120653	14	6.67814	4.02073
Nc-1-IP_pe	chr11	18128025	18128739	715	18128153	19	11.3353	5.64883
Nc-1-IP_pe	chr11	18137264	18137742	479	18137618	14	4.21449	2.76599
Nc-1-IP_pe	chr11	18158269	18158730	462	18158343	15	4.06016	2.62146
Nc-1-IP_pe	chr11	18173339	18173931	593	18173783	14	5.69896	3.50164
Nc-1-IP_pe	chr11	18189296	18190124	829	18189312	15	3.74763	2.48304
Nc-1-IP_pe	chr11	18203812	18204680	869	18204532	17	7.36791	3.96218
Nc-1-IP_pe	chr11	18204990	18205343	354	18205142	14	3.86727	2.6027
Nc-1-IP_pe	chr11	18214557	18214900	344	18214709	15	8.18692	4.69079
Nc-1-IP_pe	chr11	18245591	18245995	405	18245652	15	6.31957	3.69778
Nc-1-IP_pe	chr11	18246639	18247227	589	18247039	23	10.1326	4.42559
Nc-1-IP_pe	chr11	18247941	18248278	338	18248125	17	6.38155	3.49661
Nc-1-IP_pe	chr11	18255034	18255349	316	18255048	13	3.35769	2.42919
Nc-1-IP_pe	chr11	18266588	18266952	365	18266786	13	4.70769	3.09938
Nc-1-IP_pe	chr11	18269214	18270186	973	18269367	18	6.15736	3.29676
Nc-1-IP_pe	chr11	18276095	18276669	575	18276312	22	7.99141	3.66755
Nc-1-IP_pe	chr11	18281300	18281645	346	18281419	14	7.48133	4.4655

Nc-1-IP_pe	chr11	18336940	18337208	269	18337133	11	4.06403	2.95415
Nc-1-IP_pe	chr11	18340170	18340546	377	18340394	14	7.28181	4.3535
Nc-1-IP_pe	chr11	18352998	18353413	416	18353389	12	2.87625	2.25568
Nc-1-IP_pe	chr11	18354845	18355107	263	18354922	14	7.36061	4.39762
Nc-1-IP_pe	chr11	18361406	18361675	270	18361578	13	5.36853	3.44651
Nc-1-IP_pe	chr11	18364984	18365290	307	18365047	11	5.30676	3.66673
Nc-1-IP_pe	chr11	18381609	18381873	265	18381709	14	4.21449	2.76599
Nc-1-IP_pe	chr11	18382209	18382784	576	18382625	13	3.81073	2.64845
Nc-1-IP_pe	chr11	18385551	18386102	552	18385944	14	3.00733	2.21111
Nc-1-IP_pe	chr11	18393989	18395578	1590	18395356	26	7.17069	3.05938
Nc-1-IP_pe	chr11	18398729	18399047	319	18398921	19	7.29128	3.68799
Nc-1-IP_pe	chr11	18437519	18437965	447	18437846	12	5.84943	3.84431
Nc-1-IP_pe	chr11	18448316	18448613	298	18448438	15	6.78905	3.93887
Nc-1-IP_pe	chr11	18448886	18449183	298	18448965	13	6.36468	3.99316
Nc-1-IP_pe	chr11	18455731	18456588	858	18455866	18	8.10208	4.1823
Nc-1-IP_pe	chr11	18458017	18458352	336	18458139	14	6.05554	3.68761
Nc-1-IP_pe	chr11	18488986	18489346	361	18489190	11	4.06403	2.95415
Nc-1-IP_pe	chr11	18516488	18516981	494	18516838	13	6.62436	4.14003
Nc-1-IP_pe	chr11	18526509	18527221	713	18526640	26	6.81227	2.94581
Nc-1-IP_pe	chr11	18528576	18528980	405	18528882	12	4.24064	2.95299
Nc-1-IP_pe	chr11	18533632	18533900	269	18533779	12	4.70082	3.20033
Nc-1-IP_pe	chr11	18538391	18539394	1004	18538880	12	6.19862	4.04709
Nc-1-IP_pe	chr11	18549471	18549829	359	18549589	13	5.73236	3.64298
Nc-1-IP_pe	chr11	18554236	18554674	439	18554625	13	5.8082	3.68441
Nc-1-IP_pe	chr11	18556952	18557487	536	18557390	13	5.77769	3.66773
Nc-1-IP_pe	chr11	18561517	18561801	285	18561767	12	3.83688	2.74114
Nc-1-IP_pe	chr11	18583045	18583297	253	18583084	12	4.95159	3.33774
Nc-1-IP_pe	chr11	18614743	18615433	691	18614851	16	4.96462	2.94973
Nc-1-IP_pe	chr11	18620359	18620676	318	18620475	9	3.54889	2.85226
Nc-1-IP_pe	chr11	18622639	18623016	378	18622836	18	8.37884	4.31591
Nc-1-IP_pe	chr11	18627395	18627755	361	18627749	15	3.46204	2.35851
Nc-1-IP_pe	chr11	18634083	18634417	335	18634302	26	8.47181	3.48826
Nc-1-IP_pe	chr11	18645193	18645464	272	18645366	17	5.54967	3.12324
Nc-1-IP_pe	chr11	18656599	18656855	257	18656775	16	5.39474	3.14402
Nc-1-IP_pe	chr11	18674627	18674900	274	18674764	15	6.5434	3.81197
Nc-1-IP_pe	chr11	18687259	18687520	262	18687335	13	5.36853	3.44651
Nc-1-IP_pe	chr11	18698452	18699719	1268	18698536	17	5.14803	2.94914
Nc-1-IP_pe	chr11	18701424	18701806	383	18701494	16	5.50606	3.19508
Nc-1-IP_pe	chr11	18705686	18706633	948	18705850	22	10.516	4.7299
Nc-1-IP_pe	chr11	18713823	18714137	315	18713910	22	7.27519	3.39036
Nc-1-IP_pe	chr11	18717855	18718131	277	18717995	14	3.86727	2.6027
Nc-1-IP_pe	chr11	18721052	18722430	1379	18721425	21	4.61597	2.49283
Nc-1-IP_pe	chr11	18725703	18726181	479	18726069	16	4.96462	2.94973
Nc-1-IP_pe	chr11	18727255	18727730	476	18727404	18	6.52861	3.45863
Nc-1-IP_pe	chr11	18729942	18730428	487	18730070	12	3.34163	2.48774

Nc-1-IP_pe	chr11	18732159	18732560	402	18732550	15	2.95965	2.14351
Nc-1-IP_pe	chr11	18742415	18742862	448	18742829	16	4.25252	2.63824
Nc-1-IP_pe	chr11	18743365	18744156	792	18743956	15	3.20025	2.24588
Nc-1-IP_pe	chr11	18749670	18749966	297	18749783	17	5.6711	3.17666
Nc-1-IP_pe	chr11	18752795	18753296	502	18753203	14	5.38318	3.33994
Nc-1-IP_pe	chr11	18760212	18760912	701	18760361	16	6.80394	3.81462
Nc-1-IP_pe	chr11	18761842	18762302	461	18762159	17	6.47874	3.54137
Nc-1-IP_pe	chr11	18763099	18763474	376	18763315	18	8.73326	4.48975
Nc-1-IP_pe	chr11	18768764	18769040	277	18768922	11	3.64839	2.72584
Nc-1-IP_pe	chr11	18775463	18775994	532	18775722	19	7.29128	3.68799
Nc-1-IP_pe	chr11	18779654	18780304	651	18780115	15	5.34394	3.21607
Nc-1-IP_pe	chr11	18780645	18781101	457	18780762	12	4.20505	2.93413
Nc-1-IP_pe	chr11	18791139	18791925	787	18791343	15	6.39297	3.73508
Nc-1-IP_pe	chr11	18792234	18792628	395	18792483	20	9.63477	4.62254
Nc-1-IP_pe	chr11	18793000	18793278	279	18793042	13	5.121	3.315
Nc-1-IP_pe	chr11	18808145	18808399	255	18808243	16	8.26996	4.56762
Nc-1-IP_pe	chr11	18834137	18835107	971	18834713	16	5.39474	3.14402
Nc-1-IP_pe	chr11	18867761	18868059	299	18867926	18	7.73136	4.00629
Nc-1-IP_pe	chr11	18871143	18871988	846	18871490	12	4.24064	2.95299
Nc-1-IP_pe	chr11	18887028	18887639	612	18887464	14	5.838	3.57373
Nc-1-IP_pe	chr11	18897407	18897967	561	18897789	15	6.10627	3.59023
Nc-1-IP_pe	chr11	18931992	18932249	258	18932097	21	9.75545	4.53231
Nc-1-IP_pe	chr11	18940482	18940745	264	18940661	12	2.87625	2.25568
Nc-1-IP_pe	chr11	18943533	18944095	563	18943916	16	5.82808	3.34463
Nc-1-IP_pe	chr11	18972382	18972655	274	18972538	14	4.52309	2.91384
Nc-1-IP_pe	chr11	19005821	19006097	277	19005986	15	7.37761	4.24941
Nc-1-IP_pe	chr11	19039809	19040315	507	19039843	13	4.86362	3.18015
Nc-1-IP_pe	chr11	19109437	19109707	271	19109603	16	4.59224	2.7853
Nc-1-IP_pe	chr11	19116897	19117636	740	19117288	19	4.5193	2.56258
Nc-1-IP_pe	chr11	19130919	19131291	373	19131126	13	5.9487	3.76157
Nc-1-IP_pe	chr11	19135430	19135897	468	19135539	18	8.27052	4.2634
Nc-1-IP_pe	chr11	19185543	19186359	817	19185721	16	7.35733	4.09235
Nc-1-IP_pe	chr11	19201942	19202206	265	19202074	20	9.21699	4.428
Nc-1-IP_pe	chr11	19206820	19207101	282	19206993	17	7.02067	3.79543
Nc-1-IP_pe	chr11	19229629	19229933	305	19229751	17	5.89608	3.2766
Nc-1-IP_pe	chr11	19233645	19233919	275	19233648	14	3.00733	2.21111
Nc-1-IP_pe	chr11	19241241	19242223	983	19241379	29	9.32978	3.53561
Nc-1-IP_pe	chr11	19275063	19275616	554	19275186	16	4.17061	2.60318
Nc-1-IP_pe	chr11	19285000	19285328	329	19285052	17	4.44438	2.65333
Nc-1-IP_pe	chr11	19286106	19286411	306	19286269	22	5.39765	2.71064
Nc-1-IP_pe	chr11	19305683	19306074	392	19305832	20	9.90187	4.74901
Nc-1-IP_pe	chr11	19308397	19308722	326	19308515	22	8.66768	3.93893
Nc-1-IP_pe	chr11	19313987	19314390	404	19314105	17	6.85055	3.71486
Nc-1-IP_pe	chr11	19315530	19315889	360	19315635	18	7.81107	4.04385
Nc-1-IP_pe	chr11	19316503	19317251	749	19317048	21	9.50999	4.42209

Nc-1-IP_pe	chr11	19326828	19327186	359	19326945	20	5.70098	2.94771
Nc-1-IP_pe	chr11	19329046	19329470	425	19329231	24	8.05897	3.50918
Nc-1-IP_pe	chr11	19344659	19345025	367	19345021	19	3.26827	2.10398
Nc-1-IP_pe	chr11	19345606	19345929	324	19345684	22	5.69596	2.81438
Nc-1-IP_pe	chr11	19346234	19346693	460	19346470	20	8.5706	4.1349
Nc-1-IP_pe	chr11	19355781	19356080	300	19356031	16	4.3356	2.67395
Nc-1-IP_pe	chr11	19378003	19378406	404	19378305	21	7.98609	3.76767
Nc-1-IP_pe	chr11	19382094	19382529	436	19382117	16	3.39261	2.27748
Nc-1-IP_pe	chr11	19404033	19404438	406	19404200	16	5.82808	3.34463
Nc-1-IP_pe	chr11	19407776	19408145	370	19407836	14	4.21449	2.76599
Nc-1-IP_pe	chr11	19421000	19421324	325	19421224	18	4.32723	2.54542
Nc-1-IP_pe	chr11	19426095	19426354	260	19426229	18	7.22901	3.7732
Nc-1-IP_pe	chr11	19446562	19446952	391	19446760	16	3.39261	2.27748
Nc-1-IP_pe	chr11	19447728	19448208	481	19448098	19	7.18275	3.64066
Nc-1-IP_pe	chr11	19457992	19458327	336	19458163	18	6.91333	3.62993
Nc-1-IP_pe	chr11	19482560	19482837	278	19482663	13	3.07238	2.29378
Nc-1-IP_pe	chr11	19484613	19485051	439	19484812	19	7.73663	3.88512
Nc-1-IP_pe	chr11	19485985	19486243	259	19486028	13	4.9575	3.22911
Nc-1-IP_pe	chr11	19514632	19514889	258	19514813	17	4.78101	2.79343
Nc-1-IP_pe	chr11	19520573	19521207	635	19520869	15	5.00915	3.05678
Nc-1-IP_pe	chr11	19524189	19524584	396	19524473	14	4.21449	2.76599
Nc-1-IP_pe	chr11	19533484	19534112	629	19533582	14	4.77435	3.03616
Nc-1-IP_pe	chr11	19541417	19541778	362	19541614	21	9.59661	4.46083
Nc-1-IP_pe	chr11	19549493	19549817	325	19549693	24	7.48326	3.30363
Nc-1-IP_pe	chr11	19551033	19551569	537	19551233	18	4.96968	2.80074
Nc-1-IP_pe	chr11	19553579	19553903	325	19553664	16	3.65575	2.38624
Nc-1-IP_pe	chr11	19558339	19558823	485	19558615	21	7.10961	3.41419
Nc-1-IP_pe	chr11	19571996	19572281	286	19572175	18	4.96968	2.80074
Nc-1-IP_pe	chr11	19573519	19573992	474	19573828	14	4.69929	2.99943
Nc-1-IP_pe	chr11	19586754	19587390	637	19587004	21	7.98609	3.76767
Nc-1-IP_pe	chr11	19589582	19589890	309	19589688	22	6.84408	3.22845
Nc-1-IP_pe	chr11	19596296	19596565	270	19596460	16	5.50606	3.19508
Nc-1-IP_pe	chr11	19607021	19607485	465	19607270	13	3.95529	2.71958
Nc-1-IP_pe	chr11	19616354	19616762	409	19616576	15	6.78905	3.93887
Nc-1-IP_pe	chr11	19624463	19624740	278	19624498	12	2.87625	2.25568
Nc-1-IP_pe	chr11	19628195	19628500	306	19628314	16	6.8987	3.86161
Nc-1-IP_pe	chr11	19631778	19632106	329	19632019	13	4.02562	2.7544
Nc-1-IP_pe	chr11	19633230	19633762	533	19633671	22	4.80668	2.5094
Nc-1-IP_pe	chr11	19636511	19636798	288	19636653	16	6.8987	3.86161
Nc-1-IP_pe	chr11	19652204	19652732	529	19652300	13	5.34601	3.43447
Nc-1-IP_pe	chr11	19662497	19663005	509	19662866	18	6.63038	3.50359
Nc-1-IP_pe	chr11	19666358	19666628	271	19666555	19	5.90435	3.10381
Nc-1-IP_pe	chr11	19670370	19670641	272	19670513	15	5.1177	3.10809
Nc-1-IP_pe	chr11	19679866	19680176	311	19679966	17	8.04728	4.29732
Nc-1-IP_pe	chr11	19684340	19685220	881	19684529	14	4.60011	2.95114

Nc-1-IP_pe	chr11	19685807	19686151	345	19686058	16	3.94146	2.50592
Nc-1-IP_pe	chr11	19691531	19692290	760	19691998	17	7.21469	3.88822
Nc-1-IP_pe	chr11	19694719	19695144	426	19694864	13	4.89646	3.19725
Nc-1-IP_pe	chr11	19697724	19698119	396	19697975	16	6.33285	3.58457
Nc-1-IP_pe	chr11	19714107	19714694	588	19714147	17	4.13464	2.52661
Nc-1-IP_pe	chr11	19715012	19715423	412	19715257	17	5.25243	2.99402
Nc-1-IP_pe	chr11	19715794	19716056	263	19715886	15	4.7819	2.95039
Nc-1-IP_pe	chr11	19724278	19724530	253	19724437	13	4.33674	2.9101
Nc-1-IP_pe	chr11	19738303	19738652	350	19738333	13	3.07238	2.29378
Nc-1-IP_pe	chr11	19776984	19778037	1054	19777390	18	5.94685	3.20644
Nc-1-IP_pe	chr11	19778978	19779347	370	19779031	13	4.71921	3.10532
Nc-1-IP_pe	chr11	19801319	19801645	327	19801336	18	3.52899	2.23922
Nc-1-IP_pe	chr11	19817531	19818033	503	19817950	18	7.1522	3.73811
Nc-1-IP_pe	chr11	19823057	19823729	673	19823623	12	4.24064	2.95299
Nc-1-IP_pe	chr11	19832131	19832627	497	19832516	11	4.54377	3.22405
Nc-1-IP_pe	chr11	19859527	19860694	1168	19860091	25	11.6948	4.8085
Nc-1-IP_pe	chr11	19872638	19873010	373	19872817	16	7.30741	4.06697
Nc-1-IP_pe	chr11	19894299	19894708	410	19894405	12	4.70082	3.20033
Nc-1-IP_pe	chr11	19902874	19903275	402	19903058	18	7.1522	3.73811
Nc-1-IP_pe	chr11	19925396	19925717	322	19925579	13	7.36397	4.56728
Nc-1-IP_pe	chr11	19948716	19948977	262	19948897	18	6.28754	3.35314
Nc-1-IP_pe	chr11	19953273	19953551	279	19953459	15	4.7819	2.95039
Nc-1-IP_pe	chr11	19960788	19961044	257	19960988	19	6.78661	3.47027
Nc-1-IP_pe	chr11	19976303	19976845	543	19976504	14	5.37054	3.33352
Nc-1-IP_pe	chr11	19990159	19990546	388	19990183	12	4.45173	3.06567
Nc-1-IP_pe	chr11	19999110	19999694	585	19999184	14	4.72059	3.00983
Nc-1-IP_pe	chr11	20000756	20001043	288	20000943	15	3.20025	2.24588
Nc-1-IP_pe	chr11	20014447	20014730	284	20014630	17	7.95967	4.25344
Nc-1-IP_pe	chr11	20024057	20024336	280	20024176	18	7.93853	4.10423
Nc-1-IP_pe	chr11	20034482	20035191	710	20034986	16	3.94146	2.50592
Nc-1-IP_pe	chr11	20042796	20043644	849	20043162	17	7.43048	3.99256
Nc-1-IP_pe	chr11	20048610	20049076	467	20048787	14	4.8399	3.06836
Nc-1-IP_pe	chr11	20049844	20050244	401	20050014	21	8.10596	3.8173
Nc-1-IP_pe	chr11	20092604	20093181	578	20093044	18	9.03749	4.64139
Nc-1-IP_pe	chr11	20102832	20103166	335	20103076	19	7.29128	3.68799
Nc-1-IP_pe	chr11	20107516	20107846	331	20107658	13	3.67377	2.58159
Nc-1-IP_pe	chr11	20112067	20112364	298	20112206	16	5.82808	3.34463
Nc-1-IP_pe	chr11	20125882	20126177	296	20126033	14	5.18557	3.24018
Nc-1-IP_pe	chr11	20128759	20129372	614	20128979	15	4.06016	2.62146
Nc-1-IP_pe	chr11	20131383	20132436	1054	20132183	20	7.43645	3.64378
Nc-1-IP_pe	chr11	20157055	20157404	350	20157305	18	7.47506	3.88659
Nc-1-IP_pe	chr11	20160107	20160374	268	20160233	17	6.38155	3.49661
Nc-1-IP_pe	chr11	20163370	20163647	278	20163596	15	4.40338	2.77622
Nc-1-IP_pe	chr11	20165256	20165589	334	20165484	12	4.71777	3.20956
Nc-1-IP_pe	chr11	20208565	20208819	255	20208753	13	5.29187	3.40559

Nc-1-IP_pe	chr11	20237538	20237898	361	20237647	13	4.04902	2.76601
Nc-1-IP_pe	chr11	20245430	20245977	548	20245562	11	5.03835	3.5092
Nc-1-IP_pe	chr11	20277092	20277370	279	20277302	13	4.86362	3.18015
Nc-1-IP_pe	chr11	20295914	20296186	273	20296016	12	6.69264	4.3389
Nc-1-IP_pe	chr11	20301811	20302171	361	20302025	13	6.36468	3.99316
Nc-1-IP_pe	chr11	20317812	20318368	557	20318237	15	8.26005	4.73146
Nc-1-IP_pe	chr11	20363705	20364111	407	20363894	25	3.56113	2.01424
Nc-1-IP_pe	chr11	20387148	20388241	1094	20387917	25	10.0317	4.14592
Nc-1-IP_pe	chr11	20398433	20398698	266	20398560	15	8.77559	5.02156
Nc-1-IP_pe	chr11	20423907	20424182	276	20424136	16	6.8987	3.86161
Nc-1-IP_pe	chr11	20433654	20433911	258	20433750	13	6.69197	4.17855
Nc-1-IP_pe	chr11	20437649	20437993	345	20437871	15	3.74763	2.48304
Nc-1-IP_pe	chr11	20495049	20495364	316	20495188	13	5.9487	3.76157
Nc-1-IP_pe	chr11	20507595	20508113	519	20507846	12	3.83688	2.74114
Nc-1-IP_pe	chr11	20512265	20512531	267	20512453	15	7.46994	4.29894
Nc-1-IP_pe	chr11	20513133	20513406	274	20513288	15	6.78905	3.93887
Nc-1-IP_pe	chr11	20546754	20547146	393	20547070	12	6.07041	3.97229
Nc-1-IP_pe	chr11	20555615	20556013	399	20555806	12	3.83688	2.74114
Nc-1-IP_pe	chr11	20596930	20597293	364	20596983	13	3.86418	2.67468
Nc-1-IP_pe	chr11	20598140	20598410	271	20598322	12	4.70082	3.20033
Nc-1-IP_pe	chr11	20601251	20601697	447	20601392	13	4.74235	3.11727
Nc-1-IP_pe	chr11	20604177	20604910	734	20604749	23	11.6559	5.08929
Nc-1-IP_pe	chr11	20605661	20606087	427	20605881	14	3.00733	2.21111
Nc-1-IP_pe	chr11	20618485	20618833	349	20618719	13	4.41954	2.952
Nc-1-IP_pe	chr11	20622758	20623347	590	20623205	14	4.52309	2.91384
Nc-1-IP_pe	chr11	20625339	20625651	313	20625488	15	6.5434	3.81197
Nc-1-IP_pe	chr11	20626396	20626699	304	20626560	13	3.67377	2.58159
Nc-1-IP_pe	chr11	20643589	20643918	330	20643803	13	4.41954	2.952
Nc-1-IP_pe	chr11	20650804	20651096	293	20650944	11	4.54404	3.2242
Nc-1-IP_pe	chr11	20752262	20752549	288	20752430	12	6.86906	4.44438
Nc-1-IP_pe	chr11	20763642	20763895	254	20763734	14	5.03085	3.16286
Nc-1-IP_pe	chr11	20789152	20789535	384	20789329	12	4.24064	2.95299
Nc-1-IP_pe	chr11	20790590	20790906	317	20790680	14	6.28469	3.80898
Nc-1-IP_pe	chr11	20808395	20808897	503	20808398	12	2.87625	2.25568
Nc-1-IP_pe	chr11	20825007	20825534	528	20825341	17	7.6273	4.08876
Nc-1-IP_pe	chr11	20854582	20854870	289	20854720	12	5.87693	3.86017
Nc-1-IP_pe	chr11	20863706	20863960	255	20863726	14	4.21449	2.76599
Nc-1-IP_pe	chr11	20873703	20874304	602	20873931	17	9.99923	5.32289
Nc-1-IP_pe	chr11	20935849	20936243	395	20935904	9	4.07387	3.17289
Nc-1-IP_pe	chr11	21094258	21094794	537	21094325	10	3.89064	2.95552
Nc-1-IP_pe	chr11	21169755	21170043	289	21169862	13	7.12853	4.42991
Nc-1-IP_pe	chr11	21182108	21182396	289	21182223	13	7.7352	4.78626
Nc-1-IP_pe	chr11	21204995	21205558	564	21205275	13	4.41954	2.952
Nc-1-IP_pe	chr11	21280952	21281621	670	21281268	15	5.20142	3.14789
Nc-1-IP_pe	chr11	21433735	21434022	288	21433900	13	4.41954	2.952

Nc-1-IP_pe	chr11	21476722	21477154	433	21476901	13	5.36853	3.44651
Nc-1-IP_pe	chr11	21485276	21485669	394	21485279	12	3.16137	2.39719
Nc-1-IP_pe	chr11	21593924	21594529	606	21594445	11	4.47871	3.18705
Nc-1-IP_pe	chr11	21627146	21627780	635	21627666	12	5.84943	3.84431
Nc-1-IP_pe	chr11	21674880	21675275	396	21675054	13	5.36853	3.44651
Nc-1-IP_pe	chr11	21810959	21811308	350	21811014	9	3.27326	2.68684
Nc-1-IP_pe	chr11	21871755	21872425	671	21872319	14	8.29543	4.93196
Nc-1-IP_pe	chr11	21876360	21876796	437	21876467	9	4.46937	3.41876
Nc-1-IP_pe	chr11	22001266	22001904	639	22001755	12	5.72541	3.77304
Nc-1-IP_pe	chr11	22021226	22021971	746	22021442	15	7.56326	4.34922
Nc-1-IP_pe	chr11	22025224	22025625	402	22025378	12	4.88508	3.30112
Nc-1-IP_pe	chr11	22055638	22055954	317	22055745	18	6.63038	3.50359
Nc-1-IP_pe	chr11	22096641	22096899	259	22096821	11	4.06403	2.95415
Nc-1-IP_pe	chr11	22128604	22128979	376	22128875	12	5.61153	3.70794
Nc-1-IP_pe	chr11	22142114	22142460	347	22142210	10	4.77495	3.48064
Nc-1-IP_pe	chr11	22150134	22150497	364	22150325	11	5.03835	3.5092
Nc-1-IP_pe	chr11	22192727	22193025	299	22192817	18	6.78184	3.57098
Nc-1-IP_pe	chr11	22193382	22193780	399	22193562	15	6.24734	3.66122
Nc-1-IP_pe	chr11	22284799	22285275	477	22285102	15	4.40338	2.77622
Nc-1-IP_pe	chr11	22294107	22294464	358	22294303	15	9.55381	5.47001
Nc-1-IP_pe	chr11	22298518	22298814	297	22298760	8	3.74505	3.07688
Nc-1-IP_pe	chr11	22341095	22341873	779	22341667	19	5.51632	2.94814
Nc-1-IP_pe	chr11	22343766	22344048	283	22343810	12	3.86684	2.7567
Nc-1-IP_pe	chr11	22432656	22432981	326	22432724	14	6.06519	3.69269
Nc-1-IP_pe	chr11	22518853	22519119	267	22518999	14	6.06519	3.69269
Nc-1-IP_pe	chr11	22617799	22618119	321	22618014	16	7.97877	4.4137
Nc-1-IP_pe	chr11	22624944	22625303	360	22625175	25	6.28656	2.83671
Nc-1-IP_pe	chr11	22634401	22634728	328	22634508	13	4.86362	3.18015
Nc-1-IP_pe	chr11	22652526	22653016	491	22652639	17	8.20861	4.37862
Nc-1-IP_pe	chr11	22662992	22663351	360	22663218	12	4.24064	2.95299
Nc-1-IP_pe	chr11	22666448	22667128	681	22667001	13	7.34205	4.55444
Nc-1-IP_pe	chr11	22711375	22711887	513	22711799	11	5.3563	3.696
Nc-1-IP_pe	chr11	22739204	22739519	316	22739341	8	3.74505	3.07688
Nc-1-IP_pe	chr11	22773158	22773538	381	22773499	10	3.94999	2.99009
Nc-1-IP_pe	chr11	22860243	22860535	293	22860468	11	5.03835	3.5092
Nc-1-IP_pe	chr11	22861072	22861341	270	22861171	16	9.11744	5.02718
Nc-1-IP_pe	chr11	22942294	22942867	574	22942471	15	7.95672	4.56362
Nc-1-IP_pe	chr11	22984600	22984866	267	22984745	11	6.03439	4.10251
Nc-1-IP_pe	chr11	22988002	22988505	504	22988062	11	4.54404	3.2242
Nc-1-IP_pe	chr11	23043979	23044242	264	23044150	12	6.58417	4.27436
Nc-1-IP_pe	chr11	23202741	23203278	538	23203214	14	5.30793	3.30182
Nc-1-IP_pe	chr11	23243282	23243563	282	23243442	10	4.39571	3.25288
Nc-1-IP_pe	chr11	23321025	23321412	388	23321167	13	5.9487	3.76157
Nc-1-IP_pe	chr11	23331645	23332403	759	23331860	15	5.83691	3.4562
Nc-1-IP_pe	chr11	23370393	23370699	307	23370599	12	4.24064	2.95299

Nc-1-IP_pe	chr11	23372916	23373220	305	23373063	13	7.42513	4.60316
Nc-1-IP_pe	chr11	23400178	23400528	351	23400391	13	5.36853	3.44651
Nc-1-IP_pe	chr11	23403476	23403998	523	23403839	11	5.10577	3.5486
Nc-1-IP_pe	chr11	23405073	23405580	508	23405282	13	5.36853	3.44651
Nc-1-IP_pe	chr11	23507747	23508162	416	23507998	16	4.59224	2.7853
Nc-1-IP_pe	chr11	23679949	23680208	260	23680040	15	7.02624	4.06292
Nc-1-IP_pe	chr11	23707583	23707893	311	23707797	14	6.69566	4.03025
Nc-1-IP_pe	chr11	23717924	23718521	598	23717970	9	4.05981	3.16422
Nc-1-IP_pe	chr11	23730478	23731060	583	23730859	17	8.39361	4.47264
Nc-1-IP_pe	chr11	23731702	23732594	893	23732481	15	5.66913	3.37372
Nc-1-IP_pe	chr11	23753564	23753943	380	23753758	14	8.29543	4.93196
Nc-1-IP_pe	chr11	23816777	23817208	432	23816980	11	5.19376	3.60018
Nc-1-IP_pe	chr11	23879736	23880054	319	23879785	9	4.35492	3.34727
Nc-1-IP_pe	chr11	23883685	23884042	358	23883868	17	6.47874	3.54137
Nc-1-IP_pe	chr11	23934789	23935184	396	23934992	21	7.58657	3.60451
Nc-1-IP_pe	chr11	23942236	23942742	507	23942601	17	7.02067	3.79543
Nc-1-IP_pe	chr11	23961689	23962224	536	23962031	19	5.15822	2.80735
Nc-1-IP_pe	chr11	23993666	23994747	1082	23993982	15	6.69889	3.8921
Nc-1-IP_pe	chr11	24008516	24008769	254	24008659	12	5.84943	3.84431
Nc-1-IP_pe	chr11	24150274	24150617	344	24150514	11	5.3563	3.696
Nc-1-IP_pe	chr11	24235058	24235326	269	24235196	14	5.98179	3.64885
Nc-1-IP_pe	chr11	24314752	24315097	346	24314907	16	10.5028	5.81189
Nc-1-IP_pe	chr11	24327971	24328328	358	24328125	18	10.9042	5.61861
Nc-1-IP_pe	chr11	24405859	24407200	1342	24406214	16	6.8987	3.86161
Nc-1-IP_pe	chr11	24416211	24416497	287	24416445	16	4.25252	2.63824
Nc-1-IP_pe	chr11	24475024	24475302	279	24475239	15	4.06016	2.62146
Nc-1-IP_pe	chr11	24497027	24497455	429	24497205	23	12.3023	5.38534
Nc-1-IP_pe	chr11	24562536	24562856	321	24562661	9	3.72192	2.95716
Nc-1-IP_pe	chr11	24722629	24722893	265	24722716	12	5.23082	3.49289
Nc-1-IP_pe	chr11	24765719	24765993	275	24765848	11	4.54404	3.2242
Nc-1-IP_pe	chr11	24823902	24824254	353	24824095	15	8.16556	4.67894
Nc-1-IP_pe	chr11	24844871	24845165	295	24844887	10	3.50482	2.73328
Nc-1-IP_pe	chr11	24859667	24860275	609	24860028	23	6.25615	2.94664
Nc-1-IP_pe	chr11	24874268	24874562	295	24874455	13	4.02562	2.7544
Nc-1-IP_pe	chr11	24880051	24880376	326	24880230	15	6.85973	3.97568
Nc-1-IP_pe	chr11	24924102	24924388	287	24924182	12	6.58417	4.27436
Nc-1-IP_pe	chr11	25006716	25007001	286	25006893	14	5.82389	3.56638
Nc-1-IP_pe	chr11	25039278	25039705	428	25039469	15	7.46994	4.29894
Nc-1-IP_pe	chr11	25094487	25094771	285	25094652	17	8.31233	4.43123
Nc-1-IP_pe	chr11	25280667	25280958	292	25280713	15	4.70031	2.91253
Nc-1-IP_pe	chr11	25349742	25350218	477	25349812	13	6.36468	3.99316
Nc-1-IP_pe	chr11	25350629	25351495	867	25351278	18	7.47506	3.88659
Nc-1-IP_pe	chr11	25437201	25437601	401	25437359	10	5.23357	3.76063
Nc-1-IP_pe	chr11	25610591	25610871	281	25610734	12	6.86906	4.44438
Nc-1-IP_pe	chr11	25627837	25628091	255	25627951	14	5.30793	3.30182

Nc-1-IP_pe	chr11	25723950	25724427	478	25724148	12	5.84275	3.84046
Nc-1-IP_pe	chr11	25728990	25729378	389	25729182	18	5.72638	3.11298
Nc-1-IP_pe	chr11	25755953	25756517	565	25756326	14	5.51541	3.4073
Nc-1-IP_pe	chr11	25758108	25758648	541	25758338	13	4.41954	2.952
Nc-1-IP_pe	chr11	25781096	25781608	513	25781528	12	4.28158	2.97474
Nc-1-IP_pe	chr11	25831428	25832010	583	25831662	14	3.26761	2.32785
Nc-1-IP_pe	chr11	25923835	25924125	291	25924040	8	3.74505	3.07688
Nc-1-IP_pe	chr11	25943572	25943944	373	25943826	13	4.02562	2.7544
Nc-1-IP_pe	chr11	25944310	25945387	1078	25945050	13	4.86362	3.18015
Nc-1-IP_pe	chr11	26042334	26042630	297	26042402	14	4.21449	2.76599
Nc-1-IP_pe	chr11	26113984	26114367	384	26114154	19	8.51517	4.24108
Nc-1-IP_pe	chr11	26167270	26167596	327	26167398	13	7.7352	4.78626
Nc-1-IP_pe	chr11	26193074	26193458	385	26193275	14	8.63073	5.12814
Nc-1-IP_pe	chr11	26222362	26222797	436	26222490	9	4.20361	3.25317
Nc-1-IP_pe	chr11	26280418	26280675	258	26280521	10	5.23357	3.76063
Nc-1-IP_pe	chr11	26292715	26292986	272	26292906	14	7.42849	4.43574
Nc-1-IP_pe	chr11	26294980	26295267	288	26295056	16	9.11744	5.02718
Nc-1-IP_pe	chr11	26300879	26301154	276	26301028	12	6.86906	4.44438
Nc-1-IP_pe	chr11	26306364	26306623	260	26306574	9	4.46937	3.41876
Nc-1-IP_pe	chr11	26331991	26332334	344	26332179	9	3.92293	3.08
Nc-1-IP_pe	chr11	26374848	26375133	286	26374850	9	2.89249	2.46179
Nc-1-IP_pe	chr11	26435986	26436585	600	26436125	11	4.54404	3.2242
Nc-1-IP_pe	chr11	26451236	26451939	704	26451405	14	7.42849	4.43574
Nc-1-IP_pe	chr11	26586537	26586835	299	26586695	14	6.69566	4.03025
Nc-1-IP_pe	chr11	26732986	26733300	315	26733232	12	4.70082	3.20033
Nc-1-IP_pe	chr11	26818931	26819556	626	26819461	11	6.03439	4.10251
Nc-1-IP_pe	chr11	26823731	26824143	413	26824082	10	5.23357	3.76063
Nc-1-IP_pe	chr11	26950112	26950487	376	26950310	16	9.11744	5.02718
Nc-1-IP_pe	chr11	26994145	26994762	618	26994311	19	10.9318	5.43653
Nc-1-IP_pe	chr11	27124159	27124797	639	27124196	11	6.03439	4.10251
Nc-1-IP_pe	chr11	27142879	27143417	539	27143240	15	8.26005	4.73146
Nc-1-IP_pe	chr11	27177225	27177664	440	27177451	16	9.01614	4.97137
Nc-1-IP_pe	chr11	27202869	27203306	438	27202892	11	3.78682	2.80131
Nc-1-IP_pe	chr11	27204323	27204637	315	27204508	14	6.90659	4.14556
Nc-1-IP_pe	chr11	27219849	27220124	276	27219998	16	4.25252	2.63824
Nc-1-IP_pe	chr11	27286449	27286711	263	27286642	12	5.45448	3.61875
Nc-1-IP_pe	chr11	27356070	27356356	287	27356231	10	5.19356	3.73603
Nc-1-IP_pe	chr11	27362830	27363271	442	27363047	17	5.35342	3.03768
Nc-1-IP_pe	chr11	27472276	27472723	448	27472437	18	4.6358	2.66698
Nc-1-IP_pe	chr11	27506397	27506747	351	27506569	21	6.66991	3.24296
Nc-1-IP_pe	chr11	27630872	27631203	332	27630994	12	5.48538	3.63624
Nc-1-IP_pe	chr11	27657825	27658436	612	27657985	16	8.17084	4.51499
Nc-1-IP_pe	chr11	27665610	27666027	418	27665638	14	4.60011	2.95114
Nc-1-IP_pe	chr11	27699832	27700870	1039	27700720	17	5.54967	3.12324
Nc-1-IP_pe	chr11	27712644	27712954	311	27712738	15	9.55381	5.47001

Nc-1-IP_pe	chr11	27719074	27719388	315	27719317	15	4.06016	2.62146
Nc-1-IP_pe	chr11	27722085	27722566	482	27722185	16	7.97877	4.4137
Nc-1-IP_pe	chr11	27722911	27723229	319	27723045	18	7.73136	4.00629
Nc-1-IP_pe	chr11	27775563	27775887	325	27775746	16	9.90554	5.46889
Nc-1-IP_pe	chr11	27851411	27851688	278	27851551	16	9.46811	5.22211
Nc-1-IP_pe	chr11	27854879	27855149	271	27855052	12	5.23082	3.49289
Nc-1-IP_pe	chr11	27855731	27856339	609	27856314	11	3.28475	2.53029
Nc-1-IP_pe	chr11	27864824	27865511	688	27864971	16	6.51374	3.67221
Nc-1-IP_pe	chr11	28110074	28110516	443	28110274	44	42.548	14.5574
Nc-1-IP_pe	chr11	28115243	28115565	323	28115446	13	7.7352	4.78626
Nc-1-IP_pe	chr11	28134331	28134638	308	28134557	13	5.36853	3.44651
Nc-1-IP_pe	chr11	28238003	28238695	693	28238546	15	4.40338	2.77622
Nc-1-IP_pe	chr11	28258234	28258870	637	28258361	12	5.84943	3.84431
Nc-1-IP_pe	chr11	28377887	28378611	725	28378297	15	6.39297	3.73508
Nc-1-IP_pe	chr11	28382182	28382490	309	28382291	14	6.96097	4.17548
Nc-1-IP_pe	chr11	28451335	28451605	271	28451434	15	7.56326	4.34922
Nc-1-IP_pe	chr11	28455691	28456691	1001	28456246	10	5.01973	3.6295
Nc-1-IP_pe	chr11	28501769	28502084	316	28502011	15	8.25154	4.72672
Nc-1-IP_pe	chr11	28512246	28512854	609	28512530	15	6.39297	3.73508
Nc-1-IP_pe	chr11	28525742	28526192	451	28526024	18	7.93853	4.10423
Nc-1-IP_pe	chr11	28751687	28751961	275	28751711	10	3.89064	2.95552
Nc-1-IP_pe	chr11	28792661	28792956	296	28792941	13	3.67377	2.58159
Nc-1-IP_pe	chr11	28814484	28814751	268	28814671	11	6.03439	4.10251
Nc-1-IP_pe	chr11	28848748	28849016	269	28848817	13	7.42513	4.60316
Nc-1-IP_pe	chr11	28857337	28857849	513	28857711	12	5.84943	3.84431
Nc-1-IP_pe	chr11	28944248	28944526	279	28944363	12	5.84943	3.84431
Nc-1-IP_pe	chr11	29051058	29051622	565	29051457	18	7.1522	3.73811
Nc-1-IP_pe	chr11	29056729	29057152	424	29057078	13	7.56612	4.68618
Nc-1-IP_pe	chr11	29075883	29076946	1064	29076544	13	6.36468	3.99316
Nc-1-IP_pe	chr11	29133886	29134154	269	29133954	12	5.84943	3.84431
Nc-1-IP_pe	chr11	29224616	29225235	620	29224799	19	7.29128	3.68799
Nc-1-IP_pe	chr11	29261403	29261978	576	29261818	18	7.1522	3.73811
Nc-1-IP_pe	chr11	29266941	29267390	450	29267166	13	5.9487	3.76157
Nc-1-IP_pe	chr11	29394741	29395056	316	29394918	12	5.84943	3.84431
Nc-1-IP_pe	chr11	29446350	29446780	431	29446387	10	3.4604	2.70797
Nc-1-IP_pe	chr11	29522363	29522681	319	29522562	10	4.80655	3.49978
Nc-1-IP_pe	chr11	29570784	29571273	490	29571099	9	3.72192	2.95716
Nc-1-IP_pe	chr11	29600150	29600485	336	29600324	16	6.24025	3.54005
Nc-1-IP_pe	chr11	29632446	29632712	267	29632663	10	3.08907	2.49869
Nc-1-IP_pe	chr11	29771045	29771442	398	29771398	9	2.89249	2.46179
Nc-1-IP_pe	chr11	29794856	29795298	443	29794867	11	3.28475	2.53029
Nc-1-IP_pe	chr11	29817384	29817666	283	29817423	8	3.56021	2.95918
Nc-1-IP_pe	chr11	29827370	29827772	403	29827633	15	6.78905	3.93887
Nc-1-IP_pe	chr11	30000521	30000818	298	30000720	12	5.23082	3.49289
Nc-1-IP_pe	chr11	30016705	30017701	997	30017125	15	9.07119	5.19048

Nc-1-IP_pe	chr11	30056683	30057073	391	30056867	23	8.92223	3.93219
Nc-1-IP_pe	chr11	30067332	30067636	305	30067501	10	3.4604	2.70797
Nc-1-IP_pe	chr11	30163066	30163367	302	30163203	12	4.70082	3.20033
Nc-1-IP_pe	chr11	30295093	30295415	323	30295289	12	4.24064	2.95299
Nc-1-IP_pe	chr11	30297220	30297727	508	30297289	9	4.25966	3.28797
Nc-1-IP_pe	chr11	30322915	30323383	469	30323238	19	7.84735	3.93486
Nc-1-IP_pe	chr11	30448677	30448932	256	30448805	11	6.03439	4.10251
Nc-1-IP_pe	chr11	30526879	30527185	307	30526991	12	6.86906	4.44438
Nc-1-IP_pe	chr11	30527557	30527893	337	30527704	14	7.42849	4.43574
Nc-1-IP_pe	chr11	30585938	30586245	308	30586127	14	5.51541	3.4073
Nc-1-IP_pe	chr11	30614033	30614323	291	30614121	13	7.7352	4.78626
Nc-1-IP_pe	chr11	30673662	30673957	296	30673804	14	5.98179	3.64885
Nc-1-IP_pe	chr11	30854483	30854807	325	30854526	11	3.28475	2.53029
Nc-1-IP_pe	chr11	30911551	30911807	257	30911690	11	5.10577	3.5486
Nc-1-IP_pe	chr11	30913302	30913563	262	30913455	9	3.54889	2.85226
Nc-1-IP_pe	chr11	30970337	30970986	650	30970621	18	10.7892	5.55624
Nc-1-IP_pe	chr11	31048516	31048773	258	31048603	8	3.56021	2.95918
Nc-1-IP_pe	chr11	31049887	31050289	403	31049908	10	4.77495	3.48064
Nc-1-IP_pe	chr11	31075814	31076149	336	31076004	14	6.69566	4.03025
Nc-1-IP_pe	chr11	31173953	31174585	633	31174266	13	6.72991	4.20021
Nc-1-IP_pe	chr11	31175204	31175652	449	31175541	13	4.86362	3.18015
Nc-1-IP_pe	chr11	31186160	31186726	567	31186436	12	4.70082	3.20033
Nc-1-IP_pe	chr11	31321220	31321639	420	31321311	19	5.90435	3.10381
Nc-1-IP_pe	chr11	31369526	31370064	539	31369807	18	6.15736	3.29676
Nc-1-IP_pe	chr11	31385869	31386127	259	31385950	12	4.70082	3.20033
Nc-1-IP_pe	chr11	31386544	31387104	561	31386847	15	6.19428	3.63445
Nc-1-IP_pe	chr11	31390115	31390596	482	31390484	9	3.43499	2.78365
Nc-1-IP_pe	chr11	31397855	31398141	287	31397955	13	6.62436	4.14003
Nc-1-IP_pe	chr11	31452739	31453015	277	31452846	14	7.42849	4.43574
Nc-1-IP_pe	chr11	31473020	31473356	337	31473174	13	7.63554	4.7272
Nc-1-IP_pe	chr11	31493918	31494385	468	31494072	12	5.61153	3.70794
Nc-1-IP_pe	chr11	31506832	31507255	424	31507075	17	6.47874	3.54137
Nc-1-IP_pe	chr11	31509484	31510138	655	31509917	19	11.9896	6.0003
Nc-1-IP_pe	chr11	31514140	31514454	315	31514292	17	11.0652	5.91835
Nc-1-IP_pe	chr11	31515690	31516087	398	31516000	10	3.4604	2.70797
Nc-1-IP_pe	chr11	31717515	31717803	289	31717629	12	6.86906	4.44438
Nc-1-IP_pe	chr11	31775309	31776046	738	31775829	15	8.77559	5.02156
Nc-1-IP_pe	chr11	31798400	31799166	767	31798699	17	8.7831	4.67333
Nc-1-IP_pe	chr11	31800528	31800875	348	31800642	16	6.33285	3.58457
Nc-1-IP_pe	chr11	31802912	31803193	282	31803031	20	6.94658	3.44067
Nc-1-IP_pe	chr11	31805249	31805618	370	31805408	15	6.19428	3.63445
Nc-1-IP_pe	chr11	31805881	31806420	540	31806277	18	8.51506	4.38236
Nc-1-IP_pe	chr11	31811505	31811874	370	31811606	16	4.59754	2.78762
Nc-1-IP_pe	chr11	31813800	31814303	504	31814025	16	4.97667	2.95511
Nc-1-IP_pe	chr11	31818974	31819271	298	31819117	14	5.7679	3.53731

Nc-1-IP_pe	chr11	31823549	31823934	386	31823691	13	5.77769	3.66773
Nc-1-IP_pe	chr11	31826975	31827394	420	31827243	15	4.40338	2.77622
Nc-1-IP_pe	chr11	31844325	31844598	274	31844454	10	5.23357	3.76063
Nc-1-IP_pe	chr11	31861053	31861407	355	31861198	12	4.51041	3.09723
Nc-1-IP_pe	chr11	31886022	31886499	478	31886118	13	5.36853	3.44651
Nc-1-IP_pe	chr11	31966761	31967019	259	31966931	15	6.19428	3.63445
Nc-1-IP_pe	chr11	31984861	31985128	268	31985083	15	6.19428	3.63445
Nc-1-IP_pe	chr11	31986981	31987490	510	31987215	18	7.73136	4.00629
Nc-1-IP_pe	chr11	31988528	31988787	260	31988688	14	5.69896	3.50164
Nc-1-IP_pe	chr11	32005841	32006200	360	32006100	13	4.02562	2.7544
Nc-1-IP_pe	chr11	32056458	32056729	272	32056470	13	4.41954	2.952
Nc-1-IP_pe	chr11	32065742	32066102	361	32065835	11	4.06403	2.95415
Nc-1-IP_pe	chr11	32068849	32069181	333	32069083	11	5.24175	3.6284
Nc-1-IP_pe	chr11	32070348	32070699	352	32070510	18	11.6476	6.0285
Nc-1-IP_pe	chr11	32090922	32091784	863	32091125	23	6.62483	3.0751
Nc-1-IP_pe	chr11	32118049	32118702	654	32118559	18	6.63038	3.50359
Nc-1-IP_pe	chr11	32126403	32126692	290	32126551	14	7.7972	4.64473
Nc-1-IP_pe	chr11	32168784	32169064	281	32168983	9	3.88314	3.0556
Nc-1-IP_pe	chr11	32173029	32173500	472	32173373	15	4.7819	2.95039
Nc-1-IP_pe	chr11	32179372	32179632	261	32179495	12	5.87693	3.86017
Nc-1-IP_pe	chr11	32195109	32195479	371	32195241	18	6.15736	3.29676
Nc-1-IP_pe	chr11	32214230	32214550	321	32214315	12	6.46791	4.20548
Nc-1-IP_pe	chr11	32223372	32223673	302	32223596	12	2.87625	2.25568
Nc-1-IP_pe	chr11	32295914	32296277	364	32296154	12	5.3037	3.53374
Nc-1-IP_pe	chr11	32308008	32308536	529	32308445	15	5.66913	3.37372
Nc-1-IP_pe	chr11	32313228	32313880	653	32313769	13	6.72991	4.20021
Nc-1-IP_pe	chr11	32334604	32335250	647	32334789	16	6.51374	3.67221
Nc-1-IP_pe	chr11	32345678	32346105	428	32345845	9	4.25966	3.28797
Nc-1-IP_pe	chr11	32366094	32366363	270	32366268	12	3.47963	2.55766
Nc-1-IP_pe	chr11	32368648	32368935	288	32368885	12	4.24064	2.95299
Nc-1-IP_pe	chr11	32374403	32374880	478	32374716	16	5.37454	3.13479
Nc-1-IP_pe	chr11	32379998	32380275	278	32380155	11	5.20969	3.60954
Nc-1-IP_pe	chr11	32381112	32381717	606	32381176	14	4.21449	2.76599
Nc-1-IP_pe	chr11	32383083	32383391	309	32383295	15	7.50683	4.31879
Nc-1-IP_pe	chr11	32384451	32384717	267	32384686	13	4.34711	2.91533
Nc-1-IP_pe	chr11	32399707	32399974	268	32399782	12	4.75569	3.23024
Nc-1-IP_pe	chr11	32426315	32427581	1267	32427507	15	6.78905	3.93887
Nc-1-IP_pe	chr11	32430525	32431007	483	32430859	16	6.8987	3.86161
Nc-1-IP_pe	chr11	32433929	32434202	274	32434100	12	5.61153	3.70794
Nc-1-IP_pe	chr11	32435327	32436643	1317	32435507	15	7.81548	4.48622
Nc-1-IP_pe	chr11	32437908	32438173	266	32438060	11	4.35236	3.11555
Nc-1-IP_pe	chr11	32444763	32445029	267	32444941	9	4.46937	3.41876
Nc-1-IP_pe	chr11	32459921	32460389	469	32460332	11	5.10577	3.5486
Nc-1-IP_pe	chr11	32461130	32461531	402	32461178	12	6.27375	4.09111
Nc-1-IP_pe	chr11	32470682	32471184	503	32471058	12	5.36329	3.56726

Nc-1-IP_pe	chr11	32474982	32475587	606	32475440	10	4.47495	3.30017
Nc-1-IP_pe	chr11	32481050	32481586	537	32481564	12	4.1728	2.91706
Nc-1-IP_pe	chr11	32489256	32489672	417	32489497	13	5.9487	3.76157
Nc-1-IP_pe	chr11	32502246	32502768	523	32502709	12	5.84943	3.84431
Nc-1-IP_pe	chr11	32521126	32521507	382	32521155	10	4.93609	3.57849
Nc-1-IP_pe	chr11	32537806	32538073	268	32537994	13	7.54321	4.67266
Nc-1-IP_pe	chr11	32543101	32543383	283	32543162	12	6.31185	4.11348
Nc-1-IP_pe	chr11	32545775	32546261	487	32545909	9	4.38615	3.36674
Nc-1-IP_pe	chr11	32552122	32552633	512	32552264	13	4.41954	2.952
Nc-1-IP_pe	chr11	32558814	32559068	255	32558956	12	5.36329	3.56726
Nc-1-IP_pe	chr11	32583738	32584277	540	32584067	23	9.50079	4.16433
Nc-1-IP_pe	chr11	32608395	32609019	625	32608660	16	8.26996	4.56762
Nc-1-IP_pe	chr11	32639461	32639745	285	32639538	13	6.36468	3.99316
Nc-1-IP_pe	chr11	32666884	32667157	274	32666989	14	5.30793	3.30182
Nc-1-IP_pe	chr11	32695040	32695356	317	32695158	8	3.08755	2.66145
Nc-1-IP_pe	chr11	32704020	32705144	1125	32704484	18	8.62313	4.4354
Nc-1-IP_pe	chr11	32727580	32727885	306	32727735	14	8.37396	4.97771
Nc-1-IP_pe	chr11	32747449	32747872	424	32747814	10	3.4604	2.70797
Nc-1-IP_pe	chr11	32750289	32750992	704	32750440	16	4.59224	2.7853
Nc-1-IP_pe	chr11	32762774	32763268	495	32763206	11	3.68532	2.74592
Nc-1-IP_pe	chr11	32794463	32794733	271	32794610	15	5.20142	3.14789
Nc-1-IP_pe	chr11	32798962	32799269	308	32799002	9	4.088	3.18162
Nc-1-IP_pe	chr11	32812283	32812758	476	32812386	12	5.77527	3.80164
Nc-1-IP_pe	chr11	32823841	32824121	281	32823959	14	7.42849	4.43574
Nc-1-IP_pe	chr11	32826901	32827162	262	32826998	12	5.31851	3.54206
Nc-1-IP_pe	chr11	32829997	32830270	274	32830203	16	3.65575	2.38624
Nc-1-IP_pe	chr11	32857065	32857453	389	32857281	14	7.14701	4.27839
Nc-1-IP_pe	chr11	32864023	32864405	383	32864228	9	4.46937	3.41876
Nc-1-IP_pe	chr11	32868063	32868357	295	32868184	13	7.7352	4.78626
Nc-1-IP_pe	chr11	32874391	32874734	344	32874590	15	4.06016	2.62146
Nc-1-IP_pe	chr11	32885664	32886219	556	32885804	12	3.83688	2.74114
Nc-1-IP_pe	chr11	32888198	32888713	516	32888336	12	5.50092	3.64505
Nc-1-IP_pe	chr11	32892537	32892993	457	32892701	19	6.78661	3.47027
Nc-1-IP_pe	chr11	32898234	32898999	766	32898790	15	4.7819	2.95039
Nc-1-IP_pe	chr11	32909458	32909736	279	32909557	15	5.20142	3.14789
Nc-1-IP_pe	chr11	32913277	32913549	273	32913409	14	7.7972	4.64473
Nc-1-IP_pe	chr11	32946895	32947193	299	32947061	12	3.83688	2.74114
Nc-1-IP_pe	chr11	32959951	32960461	511	32960341	12	6.36968	4.14751
Nc-1-IP_pe	chr11	33007751	33008235	485	33007883	13	6.45312	4.04299
Nc-1-IP_pe	chr11	33015620	33016513	894	33016054	16	4.97667	2.95511
Nc-1-IP_pe	chr11	33055911	33056193	283	33056010	12	4.99209	3.3601
Nc-1-IP_pe	chr11	33060917	33061205	289	33061114	13	4.90859	3.20357
Nc-1-IP_pe	chr11	33067562	33067820	259	33067654	12	4.70082	3.20033
Nc-1-IP_pe	chr11	33068553	33069021	469	33068783	14	4.21449	2.76599
Nc-1-IP_pe	chr11	33082925	33083446	522	33083276	12	5.23082	3.49289

Nc-1-IP_pe	chr11	33088449	33088767	319	33088542	13	7.12853	4.42991
Nc-1-IP_pe	chr11	33099857	33100192	336	33100106	12	4.24064	2.95299
Nc-1-IP_pe	chr11	33147071	33147426	356	33147231	14	4.21449	2.76599
Nc-1-IP_pe	chr11	33207573	33208000	428	33207895	12	6.56855	4.26509
Nc-1-IP_pe	chr11	33220385	33220660	276	33220496	12	6.44808	4.19375
Nc-1-IP_pe	chr11	33225931	33226189	259	33226027	11	5.68685	3.89286
Nc-1-IP_pe	chr11	33233918	33234180	263	33234126	9	4.40188	3.37657
Nc-1-IP_pe	chr11	33240790	33241066	277	33240823	10	3.92601	2.97611
Nc-1-IP_pe	chr11	33243305	33244028	724	33243800	20	6.08336	3.09555
Nc-1-IP_pe	chr11	33249950	33250227	278	33250175	11	4.67786	3.30068
Nc-1-IP_pe	chr11	33258392	33258736	345	33258470	13	4.56123	3.02417
Nc-1-IP_pe	chr11	33267303	33267606	304	33267467	11	5.3563	3.696
Nc-1-IP_pe	chr11	33272904	33273207	304	33272959	8	3.64414	3.01254
Nc-1-IP_pe	chr11	33282188	33282598	411	33282321	15	4.06016	2.62146
Nc-1-IP_pe	chr11	33304001	33304557	557	33304470	13	6.69197	4.17855
Nc-1-IP_pe	chr11	33330339	33330621	283	33330461	15	9.19325	5.26076
Nc-1-IP_pe	chr11	33331523	33332056	534	33332005	9	4.46937	3.41876
Nc-1-IP_pe	chr11	33337439	33337960	522	33337828	19	9.15219	4.54307
Nc-1-IP_pe	chr11	33343274	33343539	266	33343431	10	4.39571	3.25288
Nc-1-IP_pe	chr11	33349568	33349831	264	33349684	14	3.86727	2.6027
Nc-1-IP_pe	chr11	33371631	33372052	422	33371799	13	5.83893	3.70124
Nc-1-IP_pe	chr11	33373723	33374102	380	33373778	12	3.47963	2.55766
Nc-1-IP_pe	chr11	33375404	33376116	713	33375816	14	5.43414	3.36584
Nc-1-IP_pe	chr11	33397395	33397750	356	33397556	12	5.51653	3.6539
Nc-1-IP_pe	chr11	33401908	33402311	404	33402173	17	5.54967	3.12324
Nc-1-IP_pe	chr11	33418522	33419058	537	33418952	12	3.83688	2.74114
Nc-1-IP_pe	chr11	33428992	33429261	270	33429188	13	6.56172	4.10444
Nc-1-IP_pe	chr11	33452375	33452929	555	33452419	12	5.84943	3.84431
Nc-1-IP_pe	chr11	33459561	33459820	260	33459748	17	7.6885	4.11887
Nc-1-IP_pe	chr11	33465131	33465426	296	33465265	12	6.31185	4.11348
Nc-1-IP_pe	chr11	33504039	33504605	567	33504567	11	3.28475	2.53029
Nc-1-IP_pe	chr11	33520878	33521184	307	33521103	14	6.69566	4.03025
Nc-1-IP_pe	chr11	33542240	33542931	692	33542371	16	6.1093	3.47747
Nc-1-IP_pe	chr11	33609447	33609841	395	33609469	12	3.47963	2.55766
Nc-1-IP_pe	chr11	33631510	33631773	264	33631554	11	4.11366	2.98176
Nc-1-IP_pe	chr11	33645823	33646145	323	33646001	15	7.46994	4.29894
Nc-1-IP_pe	chr11	33650451	33650791	341	33650551	15	5.20142	3.14789
Nc-1-IP_pe	chr11	33658695	33658984	290	33658785	17	7.02067	3.79543
Nc-1-IP_pe	chr11	33674272	33674575	304	33674545	12	3.47963	2.55766
Nc-1-IP_pe	chr11	33700613	33701104	492	33700731	19	6.33279	3.27955
Nc-1-IP_pe	chr11	33723479	33723749	271	33723653	12	3.83688	2.74114
Nc-1-IP_pe	chr11	33735991	33736398	408	33736202	18	6.63038	3.50359
Nc-1-IP_pe	chr11	33737085	33737447	363	33737309	15	5.58327	3.33181
Nc-1-IP_pe	chr11	33773889	33774492	604	33774219	27	14.5965	5.7276
Nc-1-IP_pe	chr11	33820082	33820350	269	33820245	11	4.00079	2.91908

Nc-1-IP_pe	chr11	33820998	33821376	379	33821067	13	5.57038	3.55505
Nc-1-IP_pe	chr11	33828822	33829384	563	33829226	19	6.78661	3.47027
Nc-1-IP_pe	chr11	33852494	33853020	527	33852513	13	4.02562	2.7544
Nc-1-IP_pe	chr11	33859244	33859723	480	33859647	10	4.20113	3.13749
Nc-1-IP_pe	chr11	33861392	33861739	348	33861577	14	7.58433	4.52368
Nc-1-IP_pe	chr11	33862113	33862423	311	33862194	12	5.80886	3.82095
Nc-1-IP_pe	chr11	33864530	33865092	563	33864904	12	5.37834	3.57574
Nc-1-IP_pe	chr11	33869357	33869885	529	33869787	17	5.14803	2.94914
Nc-1-IP_pe	chr11	33870218	33870900	683	33870547	20	9.63477	4.62254
Nc-1-IP_pe	chr11	33875385	33875945	561	33875610	10	4.51818	3.32604
Nc-1-IP_pe	chr11	33892961	33893231	271	33893067	17	7.02067	3.79543
Nc-1-IP_pe	chr11	33901804	33902118	315	33901870	13	5.36853	3.44651
Nc-1-IP_pe	chr11	33913351	33913738	388	33913519	16	8.26996	4.56762
Nc-1-IP_pe	chr11	33914461	33915086	626	33914623	12	4.24064	2.95299
Nc-1-IP_pe	chr11	33925837	33926163	327	33925996	15	8.72788	4.99447
Nc-1-IP_pe	chr11	33935512	33936158	647	33935743	14	4.60011	2.95114
Nc-1-IP_pe	chr11	33980289	33980627	339	33980426	13	6.39984	4.01295
Nc-1-IP_pe	chr11	33990015	33990580	566	33990452	17	6.47874	3.54137
Nc-1-IP_pe	chr11	33994594	33994923	330	33994910	12	3.16137	2.39719
Nc-1-IP_pe	chr11	33997889	33998238	350	33997961	17	5.14803	2.94914
Nc-1-IP_pe	chr11	34010971	34011349	379	34011204	10	4.95265	3.58858
Nc-1-IP_pe	chr11	34051451	34051923	473	34051635	17	7.6273	4.08876
Nc-1-IP_pe	chr11	34052462	34052840	379	34052729	16	6.33285	3.58457
Nc-1-IP_pe	chr11	34105640	34106094	455	34105686	13	3.07238	2.29378
Nc-1-IP_pe	chr11	34120365	34121273	909	34120526	13	7.42513	4.60316
Nc-1-IP_pe	chr11	34130542	34131127	586	34130934	10	3.89064	2.95552
Nc-1-IP_pe	chr11	34137851	34138156	306	34137885	12	4.28158	2.97474
Nc-1-IP_pe	chr11	34149316	34150183	868	34149426	14	7.07169	4.23662
Nc-1-IP_pe	chr11	34153687	34154392	706	34153982	13	5.91681	3.74401
Nc-1-IP_pe	chr11	34154920	34155406	487	34155106	12	3.47963	2.55766
Nc-1-IP_pe	chr11	34162259	34162697	439	34162635	13	3.07238	2.29378
Nc-1-IP_pe	chr11	34166478	34167427	950	34166864	13	5.04465	3.2748
Nc-1-IP_pe	chr11	34169616	34170062	447	34169748	11	4.1834	3.02067
Nc-1-IP_pe	chr11	34173704	34174924	1221	34174710	15	7.46994	4.29894
Nc-1-IP_pe	chr11	34178110	34178400	291	34178263	8	3.0402	2.6319
Nc-1-IP_pe	chr11	34181171	34181754	584	34181543	16	8.26996	4.56762
Nc-1-IP_pe	chr11	34182981	34183632	652	34183593	10	4.0355	3.04008
Nc-1-IP_pe	chr11	34230042	34230573	532	34230195	17	8.7831	4.67333
Nc-1-IP_pe	chr11	34240343	34240727	385	34240529	16	7.53857	4.18505
Nc-1-IP_pe	chr11	34248782	34249153	372	34248959	19	10.5055	5.21599
Nc-1-IP_pe	chr11	34257668	34258118	451	34258010	13	7.00513	4.3584
Nc-1-IP_pe	chr11	34268349	34268717	369	34268644	13	6.56172	4.10444
Nc-1-IP_pe	chr11	34294522	34295387	866	34294653	12	6.6717	4.32642
Nc-1-IP_pe	chr11	34304607	34304877	271	34304697	15	8.26005	4.73146
Nc-1-IP_pe	chr11	34306328	34306643	316	34306400	10	4.39571	3.25288

Nc-1-IP_pe	chr11	34322457	34323124	668	34323075	10	4.62142	3.388
Nc-1-IP_pe	chr11	34328016	34328410	395	34328311	9	3.94978	3.09648
Nc-1-IP_pe	chr11	34343581	34344067	487	34343921	10	4.33501	3.21677
Nc-1-IP_pe	chr11	34345143	34345437	295	34345248	12	5.74196	3.78252
Nc-1-IP_pe	chr11	34356626	34357776	1151	34357321	15	5.66913	3.37372
Nc-1-IP_pe	chr11	34363257	34363726	470	34363673	11	3.64839	2.72584
Nc-1-IP_pe	chr11	34395351	34395990	640	34395855	13	6.47103	4.0531
Nc-1-IP_pe	chr11	34435606	34435959	354	34435848	16	6.33285	3.58457
Nc-1-IP_pe	chr11	34438909	34439279	371	34439048	21	4.0961	2.31438
Nc-1-IP_pe	chr11	34483113	34483452	340	34483326	12	6.84223	4.4283
Nc-1-IP_pe	chr11	34485796	34486360	565	34486257	8	3.56021	2.95918
Nc-1-IP_pe	chr11	34495783	34496312	530	34495984	12	4.63088	3.16234
Nc-1-IP_pe	chr11	34504472	34504781	310	34504686	14	7.32104	4.37545
Nc-1-IP_pe	chr11	34505322	34505639	318	34505444	12	6.14317	4.01469
Nc-1-IP_pe	chr11	34509182	34509575	394	34509561	9	4.1889	3.24405
Nc-1-IP_pe	chr11	34595051	34595345	295	34595098	10	4.39571	3.25288
Nc-1-IP_pe	chr11	34628884	34629151	268	34629050	13	4.86362	3.18015
Nc-1-IP_pe	chr11	34677370	34677631	262	34677459	10	5.23357	3.76063
Nc-1-IP_pe	chr11	34692247	34692499	253	34692364	19	7.29128	3.68799
Nc-1-IP_pe	chr11	34702304	34702632	329	34702503	15	8.84797	5.06275
Nc-1-IP_pe	chr11	34713230	34713745	516	34713632	11	5.7051	3.90381
Nc-1-IP_pe	chr11	34738004	34738611	608	34738104	10	4.77495	3.48064
Nc-1-IP_pe	chr11	34790284	34790582	299	34790449	8	3.64414	3.01254
Nc-1-IP_pe	chr11	34818685	34819045	361	34818998	8	3.74505	3.07688
Nc-1-IP_pe	chr11	34874870	34875388	519	34874973	10	3.4604	2.70797
Nc-1-IP_pe	chr11	34898405	34899150	746	34898960	14	8.29543	4.93196
Nc-1-IP_pe	chr11	34915763	34916237	475	34916161	14	4.21449	2.76599
Nc-1-IP_pe	chr11	34941780	34942050	271	34941892	13	6.62436	4.14003
Nc-1-IP_pe	chr11	35021745	35022482	738	35022078	14	5.51541	3.4073
Nc-1-IP_pe	chr11	35048934	35049489	556	35049408	14	6.06519	3.69269
Nc-1-IP_pe	chr11	35116726	35117067	342	35116916	15	3.46204	2.35851
Nc-1-IP_pe	chr11	35126034	35126359	326	35126226	8	3.74505	3.07688
Nc-1-IP_pe	chr11	35128984	35129534	551	35129418	14	7.9745	4.74633
Nc-1-IP_pe	chr11	35138593	35138919	327	35138746	13	5.36853	3.44651
Nc-1-IP_pe	chr11	35166361	35166658	298	35166488	15	7.19874	4.15407
Nc-1-IP_pe	chr11	35176518	35176838	321	35176773	12	6.86906	4.44438
Nc-1-IP_pe	chr11	35278390	35278891	502	35278735	15	8.26005	4.73146
Nc-1-IP_pe	chr11	35351653	35351935	283	35351813	12	6.86906	4.44438
Nc-1-IP_pe	chr11	35371129	35371493	365	35371148	8	3.4722	2.90337
Nc-1-IP_pe	chr11	35419252	35420061	810	35419912	13	5.57038	3.55505
Nc-1-IP_pe	chr11	35470599	35471006	408	35470862	12	6.58417	4.27436
Nc-1-IP_pe	chr11	35525836	35526105	270	35525859	11	4.11366	2.98176
Nc-1-IP_pe	chr11	35590540	35590894	355	35590761	15	6.78905	3.93887
Nc-1-IP_pe	chr11	35618766	35619728	963	35619341	20	6.08336	3.09555
Nc-1-IP_pe	chr11	35662801	35663233	433	35662921	24	7.61242	3.34923

Nc-1-IP_pe	chr11	35685688	35685995	308	35685853	17	7.6273	4.08876
Nc-1-IP_pe	chr11	35702883	35703306	424	35703047	14	5.03085	3.16286
Nc-1-IP_pe	chr11	35749410	35749735	326	35749545	16	6.33285	3.58457
Nc-1-IP_pe	chr11	35793348	35793768	421	35793395	12	5.45448	3.61875
Nc-1-IP_pe	chr11	35800203	35800719	517	35800275	11	5.37297	3.70587
Nc-1-IP_pe	chr11	35851347	35851617	271	35851521	13	5.96425	3.77015
Nc-1-IP_pe	chr11	35859686	35860619	934	35860517	13	3.07238	2.29378
Nc-1-IP_pe	chr11	35880703	35880997	295	35880849	17	8.31233	4.43123
Nc-1-IP_pe	chr11	35888155	35888484	330	35888189	13	3.67377	2.58159
Nc-1-IP_pe	chr11	35891156	35891453	298	35891240	14	5.51541	3.4073
Nc-1-IP_pe	chr11	35915655	35915921	267	35915801	13	6.62436	4.14003
Nc-1-IP_pe	chr11	35960668	35961440	773	35960804	16	4.59224	2.7853
Nc-1-IP_pe	chr11	35973539	35973799	261	35973740	14	5.838	3.57373
Nc-1-IP_pe	chr11	35999377	35999795	419	35999476	16	7.61454	4.22416
Nc-1-IP_pe	chr11	36036972	36037331	360	36037169	18	7.1522	3.73811
Nc-1-IP_pe	chr11	36044529	36044796	268	36044740	14	6.61011	3.98382
Nc-1-IP_pe	chr11	36068537	36068822	286	36068585	14	4.60011	2.95114
Nc-1-IP_pe	chr11	36090468	36091134	667	36090693	11	4.06403	2.95415
Nc-1-IP_pe	chr11	36101841	36102113	273	36101982	13	4.86362	3.18015
Nc-1-IP_pe	chr11	36103319	36103574	256	36103486	13	5.86991	3.71823
Nc-1-IP_pe	chr11	36113741	36114012	272	36113864	12	4.70082	3.20033
Nc-1-IP_pe	chr11	36123564	36123841	278	36123702	13	6.62436	4.14003
Nc-1-IP_pe	chr11	36125911	36126172	262	36126045	16	6.24025	3.54005
Nc-1-IP_pe	chr11	36129370	36129622	253	36129494	13	5.36853	3.44651
Nc-1-IP_pe	chr11	36130679	36131314	636	36130861	14	6.71255	4.03944
Nc-1-IP_pe	chr11	36143978	36144353	376	36144218	15	4.7819	2.95039
Nc-1-IP_pe	chr11	36156241	36156510	270	36156392	11	4.84616	3.39759
Nc-1-IP_pe	chr11	36159810	36160252	443	36160204	13	5.71736	3.63481
Nc-1-IP_pe	chr11	36180551	36180853	303	36180767	12	5.13046	3.43687
Nc-1-IP_pe	chr11	36215864	36216212	349	36216084	16	7.11257	3.96852
Nc-1-IP_pe	chr11	36221036	36221418	383	36221374	15	3.74763	2.48304
Nc-1-IP_pe	chr11	36228798	36229284	487	36229175	17	7.21469	3.88822
Nc-1-IP_pe	chr11	36239700	36240030	331	36239903	12	4.66786	3.1824
Nc-1-IP_pe	chr11	36252195	36252594	400	36252426	16	8.26996	4.56762
Nc-1-IP_pe	chr11	36257537	36257833	297	36257690	16	7.61454	4.22416
Nc-1-IP_pe	chr11	36258338	36258733	396	36258446	17	6.92131	3.74829
Nc-1-IP_pe	chr11	36260117	36260601	485	36260282	12	5.17301	3.46058
Nc-1-IP_pe	chr11	36262597	36262872	276	36262719	15	6.5895	3.83567
Nc-1-IP_pe	chr11	36271790	36272122	333	36271926	15	3.74763	2.48304
Nc-1-IP_pe	chr11	36276095	36276449	355	36276265	14	6.332	3.83422
Nc-1-IP_pe	chr11	36289389	36289855	467	36289705	16	5.37454	3.13479
Nc-1-IP_pe	chr11	36294548	36294808	261	36294730	11	3.64839	2.72584
Nc-1-IP_pe	chr11	36301119	36301582	464	36301268	18	6.15736	3.29676
Nc-1-IP_pe	chr11	36345111	36345402	292	36345373	10	3.4604	2.70797
Nc-1-IP_pe	chr11	36369789	36370158	370	36370134	9	4.25966	3.28797

Nc-1-IP_pe	chr11	36376625	36377435	811	36376844	15	4.32392	2.74012
Nc-1-IP_pe	chr11	36397824	36398211	388	36398020	10	3.4604	2.70797
Nc-1-IP_pe	chr11	36399967	36400245	279	36400106	11	4.8033	3.37283
Nc-1-IP_pe	chr11	36411209	36411754	546	36411598	13	6.26093	3.93497
Nc-1-IP_pe	chr11	36422336	36422598	263	36422503	11	2.96397	2.36091
Nc-1-IP_pe	chr11	36424841	36425136	296	36424929	10	4.59158	3.37006
Nc-1-IP_pe	chr11	36427914	36428242	329	36428056	18	9.1091	4.6774
Nc-1-IP_pe	chr11	36454697	36454951	255	36454716	14	5.18557	3.24018
Nc-1-IP_pe	chr11	36509582	36510348	767	36510156	19	4.8268	2.67939
Nc-1-IP_pe	chr11	36556967	36557248	282	36557068	20	6.49719	3.259
Nc-1-IP_pe	chr11	36560249	36560659	411	36560631	11	3.68532	2.74592
Nc-1-IP_pe	chr11	36573637	36574149	513	36574038	11	4.61014	3.26192
Nc-1-IP_pe	chr11	36594477	36594847	371	36594675	19	8.4638	4.21715
Nc-1-IP_pe	chr11	36694176	36694841	666	36694712	13	5.9487	3.76157
Nc-1-IP_pe	chr11	36914006	36914301	296	36914155	15	8.84797	5.06275
Nc-1-IP_pe	chr11	36922091	36922498	408	36922231	14	6.06519	3.69269
Nc-1-IP_pe	chr11	37067383	37067727	345	37067582	14	8.44792	5.0209
Nc-1-IP_pe	chr11	37145554	37145994	441	37145737	12	4.70082	3.20033
Nc-1-IP_pe	chr11	37168081	37168391	311	37168236	12	6.58417	4.27436
Nc-1-IP_pe	chr11	37219124	37219876	753	37219264	11	5.10577	3.5486
Nc-1-IP_pe	chr11	37362370	37362671	302	37362426	12	5.23082	3.49289
Nc-1-IP_pe	chr11	37381265	37381533	269	37381422	10	5.23357	3.76063
Nc-1-IP_pe	chr11	37431609	37432160	552	37431740	13	4.56123	3.02417
Nc-1-IP_pe	chr11	37435194	37435710	517	37435288	13	6.02837	3.80557
Nc-1-IP_pe	chr11	37519290	37519627	338	37519456	11	5.77463	3.94557
Nc-1-IP_pe	chr11	37545492	37545861	370	37545528	10	4.33501	3.21677
Nc-1-IP_pe	chr11	37608172	37608452	281	37608371	9	4.46937	3.41876
Nc-1-IP_pe	chr11	37812383	37812895	513	37812407	14	3.86727	2.6027
Nc-1-IP_pe	chr11	37947806	37948315	510	37947937	11	3.78682	2.80131
Nc-1-IP_pe	chr11	38114555	38114816	262	38114678	15	5.66913	3.37372
Nc-1-IP_pe	chr11	38116499	38117205	707	38116612	12	4.63088	3.16234
Nc-1-IP_pe	chr11	38148532	38149229	698	38148586	15	7.46994	4.29894
Nc-1-IP_pe	chr11	38170084	38170556	473	38170273	12	6.82053	4.41531
Nc-1-IP_pe	chr11	38176644	38177033	390	38176894	11	4.88957	3.42271
Nc-1-IP_pe	chr11	38192118	38192625	508	38192284	19	9.92774	4.92358
Nc-1-IP_pe	chr11	38295695	38296085	391	38295927	14	5.03085	3.16286
Nc-1-IP_pe	chr11	38324265	38324632	368	38324438	19	9.92774	4.92358
Nc-1-IP_pe	chr11	38346436	38346754	319	38346626	10	4.39571	3.25288
Nc-1-IP_pe	chr11	38395088	38395353	266	38395220	12	6.86906	4.44438
Nc-1-IP_pe	chr11	38428838	38429142	305	38428972	13	7.34205	4.55444
Nc-1-IP_pe	chr11	38461814	38462496	683	38461906	15	6.19428	3.63445
Nc-1-IP_pe	chr11	38503005	38503275	271	38503153	15	3.46204	2.35851
Nc-1-IP_pe	chr11	38601834	38602231	398	38602019	10	4.33501	3.21677
Nc-1-IP_pe	chr11	38667976	38668241	266	38668130	12	5.15877	3.45264
Nc-1-IP_pe	chr11	38696583	38697230	648	38696687	12	5.84943	3.84431

Nc-1-IP_pe	chr11	38756471	38756835	365	38756594	13	4.41954	2.952
Nc-1-IP_pe	chr11	38768158	38768846	689	38768726	14	4.95172	3.12357
Nc-1-IP_pe	chr11	38774738	38775192	455	38775002	17	8.31233	4.43123
Nc-1-IP_pe	chr11	38821678	38822494	817	38822047	12	5.61153	3.70794
Nc-1-IP_pe	chr11	38823700	38823979	280	38823888	15	5.66913	3.37372
Nc-1-IP_pe	chr11	38867091	38867499	409	38867287	11	2.96397	2.36091
Nc-1-IP_pe	chr11	38969836	38970334	499	38969956	11	6.03439	4.10251
Nc-1-IP_pe	chr11	38986031	38986991	961	38986222	16	7.61454	4.22416
Nc-1-IP_pe	chr11	39046248	39046522	275	39046492	9	4.46937	3.41876
Nc-1-IP_pe	chr11	39061650	39061925	276	39061792	11	4.06403	2.95415
Nc-1-IP_pe	chr11	39072120	39072397	278	39072263	10	5.23357	3.76063
Nc-1-IP_pe	chr11	39078432	39078901	470	39078669	14	6.69566	4.03025
Nc-1-IP_pe	chr11	39161477	39161905	429	39161655	12	5.23082	3.49289
Nc-1-IP_pe	chr11	39170154	39170423	270	39170295	13	7.7352	4.78626
Nc-1-IP_pe	chr11	39192044	39193000	957	39192709	15	6.78905	3.93887
Nc-1-IP_pe	chr11	39311923	39312292	370	39312168	14	6.61011	3.98382
Nc-1-IP_pe	chr11	39333137	39333624	488	39333443	19	9.92774	4.92358
Nc-1-IP_pe	chr11	39341560	39341886	327	39341689	11	6.03439	4.10251
Nc-1-IP_pe	chr11	39347546	39348248	703	39347686	11	6.03439	4.10251
Nc-1-IP_pe	chr11	39410497	39410755	259	39410639	12	6.86906	4.44438
Nc-1-IP_pe	chr11	39442042	39442613	572	39442315	15	9.35725	5.35563
Nc-1-IP_pe	chr11	39536203	39536665	463	39536438	11	5.19376	3.60018
Nc-1-IP_pe	chr11	39651981	39652310	330	39652168	13	6.62436	4.14003
Nc-1-IP_pe	chr11	39663692	39664268	577	39663918	15	6.19428	3.63445
Nc-1-IP_pe	chr11	39716237	39716567	331	39716314	11	5.52654	3.79706
Nc-1-IP_pe	chr11	39755099	39755374	276	39755321	10	3.4604	2.70797
Nc-1-IP_pe	chr11	39786018	39787094	1077	39786227	15	7.95672	4.56362
Nc-1-IP_pe	chr11	39809776	39810620	845	39809986	12	5.84943	3.84431
Nc-1-IP_pe	chr11	39846814	39847247	434	39847157	17	6.47874	3.54137
Nc-1-IP_pe	chr11	39881109	39881808	700	39881712	16	7.97877	4.4137
Nc-1-IP_pe	chr11	39946921	39947234	314	39946928	8	3.56021	2.95918
Nc-1-IP_pe	chr11	39958558	39958901	344	39958882	8	3.264	2.772
Nc-1-IP_pe	chr11	39983882	39984507	626	39984178	11	5.10577	3.5486
Nc-1-IP_pe	chr11	40020388	40020681	294	40020480	10	5.23357	3.76063
Nc-1-IP_pe	chr11	40099879	40100133	255	40100012	12	6.58417	4.27436
Nc-1-IP_pe	chr11	40101719	40101971	253	40101799	9	4.35492	3.34727
Nc-1-IP_pe	chr11	40186588	40186842	255	40186746	9	4.25966	3.28797
Nc-1-IP_pe	chr11	40194574	40195019	446	40194686	10	5.10552	3.682
Nc-1-IP_pe	chr11	40336648	40336926	279	40336742	10	5.23357	3.76063
Nc-1-IP_pe	chr11	40354385	40355123	739	40354604	10	4.93609	3.57849
Nc-1-IP_pe	chr11	40658959	40659415	457	40659167	17	7.6273	4.08876
Nc-1-IP_pe	chr11	40710520	40711321	802	40710746	14	8.63073	5.12814
Nc-1-IP_pe	chr11	40814025	40814482	458	40814355	10	3.89064	2.95552
Nc-1-IP_pe	chr11	40971567	40971834	268	40971654	13	6.62436	4.14003
Nc-1-IP_pe	chr11	41304977	41305388	412	41305211	15	7.46994	4.29894

Nc-1-IP_pe	chr11	41370276	41370536	261	41370416	11	6.03439	4.10251
Nc-1-IP_pe	chr11	41464592	41464864	273	41464754	16	10.5028	5.81189
Nc-1-IP_pe	chr11	41516489	41516802	314	41516661	12	5.84943	3.84431
Nc-1-IP_pe	chr11	41591876	41592203	328	41592166	10	3.4604	2.70797
Nc-1-IP_pe	chr11	41593647	41593905	259	41593741	9	4.46937	3.41876
Nc-1-IP_pe	chr11	41641733	41642075	343	41642014	9	4.46937	3.41876
Nc-1-IP_pe	chr11	41650427	41650699	273	41650558	14	6.78215	4.0774
Nc-1-IP_pe	chr11	41728093	41728599	507	41728570	11	4.35236	3.11555
Nc-1-IP_pe	chr11	41780560	41780832	273	41780688	9	4.25966	3.28797
Nc-1-IP_pe	chr11	41788122	41788767	646	41788429	21	12.6115	5.91104
Nc-1-IP_pe	chr11	41902353	41902914	562	41902706	13	5.9487	3.76157
Nc-1-IP_pe	chr11	41917561	41918464	904	41918241	15	7.02624	4.06292
Nc-1-IP_pe	chr11	42066021	42066710	690	42066329	14	5.03085	3.16286
Nc-1-IP_pe	chr11	42407413	42407752	340	42407630	12	6.58417	4.27436
Nc-1-IP_pe	chr11	42434631	42434976	346	42434838	21	10.2367	4.75227
Nc-1-IP_pe	chr11	42475967	42476267	301	42476092	10	4.93609	3.57849
Nc-1-IP_pe	chr11	42480073	42480424	352	42480100	8	3.74505	3.07688
Nc-1-IP_pe	chr11	42503593	42504153	561	42503713	10	4.93609	3.57849
Nc-1-IP_pe	chr11	42635956	42636420	465	42636279	12	6.58417	4.27436
Nc-1-IP_pe	chr11	42661343	42661909	567	42661555	11	4.88957	3.42271
Nc-1-IP_pe	chr11	42876379	42876685	307	42876530	14	6.06519	3.69269
Nc-1-IP_pe	chr11	42881021	42881669	649	42881359	19	12.587	6.32844
Nc-1-IP_pe	chr11	42940484	42941224	741	42940828	8	3.56021	2.95918
Nc-1-IP_pe	chr11	43100205	43101105	901	43100342	11	6.03439	4.10251
Nc-1-IP_pe	chr11	43130629	43131672	1044	43131171	12	6.86906	4.44438
Nc-1-IP_pe	chr11	43207113	43207375	263	43207123	10	3.83201	2.92147
Nc-1-IP_pe	chr11	43211934	43212460	527	43212288	13	5.9487	3.76157
Nc-1-IP_pe	chr11	43270097	43270375	279	43270185	10	5.10552	3.682
Nc-1-IP_pe	chr11	43275284	43275651	368	43275436	16	10.117	5.58955
Nc-1-IP_pe	chr11	43300886	43301195	310	43301137	9	2.89249	2.46179
Nc-1-IP_pe	chr11	43305226	43305535	310	43305465	11	4.54404	3.2242
Nc-1-IP_pe	chr11	43311962	43312410	449	43311994	17	5.14803	2.94914
Nc-1-IP_pe	chr11	43351719	43352105	387	43351866	13	4.67336	3.0817
Nc-1-IP_pe	chr11	43358886	43359181	296	43358980	21	7.72183	3.65936
Nc-1-IP_pe	chr11	43371286	43371796	511	43371634	10	4.62142	3.388
Nc-1-IP_pe	chr11	43452274	43452545	272	43452420	18	9.1091	4.6774
Nc-1-IP_pe	chr11	43460912	43461307	396	43461149	8	3.64414	3.01254
Nc-1-IP_pe	chr11	43478757	43479094	338	43478908	16	9.11744	5.02718
Nc-1-IP_pe	chr11	43481869	43482138	270	43482014	15	9.35725	5.35563
Nc-1-IP_pe	chr11	43504114	43504392	279	43504219	16	7.79347	4.31685
Nc-1-IP_pe	chr11	43515046	43515425	380	43515256	14	6.06519	3.69269
Nc-1-IP_pe	chr11	43516105	43516976	872	43516455	17	6.64406	3.61808
Nc-1-IP_pe	chr11	43521613	43522428	816	43521786	14	6.78215	4.0774
Nc-1-IP_pe	chr11	43547333	43548742	1410	43547508	17	8.58498	4.57078
Nc-1-IP_pe	chr11	43579204	43579943	740	43579772	21	12.22	5.71184

Nc-1-IP_pe	chr11	43583335	43584206	872	43583542	11	4.54377	3.22405
Nc-1-IP_pe	chr11	43644065	43644689	625	43644290	39	21.7559	6.55365
Nc-1-IP_pe	chr11	43651568	43651865	298	43651661	10	5.22943	3.75809
Nc-1-IP_pe	chr11	43652404	43652725	322	43652511	11	5.83542	3.98217
Nc-1-IP_pe	chr11	43658782	43659301	520	43659118	14	3.00733	2.21111
Nc-1-IP_pe	chr11	43732598	43732938	341	43732816	9	4.07387	3.17289
Nc-1-IP_pe	chr11	43733737	43734339	603	43734219	16	6.65665	3.74206
Nc-1-IP_pe	chr11	43774228	43774498	271	43774369	15	8.26005	4.73146
Nc-1-IP_pe	chr11	43828059	43828426	368	43828177	12	6.86906	4.44438
Nc-1-IP_pe	chr11	43868207	43868472	266	43868283	13	5.9487	3.76157
Nc-1-IP_pe	chr11	43880321	43881181	861	43880806	37	25.9492	8.63183
Nc-1-IP_pe	chr11	43890341	43890715	375	43890617	11	5.77463	3.94557
Nc-1-IP_pe	chr11	43898522	43898864	343	43898767	14	5.98179	3.64885
Nc-1-IP_pe	chr11	43924206	43924956	751	43924702	21	9.98999	4.63886
Nc-1-IP_pe	chr11	43928125	43928680	556	43928565	14	5.57768	3.4392
Nc-1-IP_pe	chr11	43931161	43931561	401	43931430	14	5.03085	3.16286
Nc-1-IP_pe	chr11	43933708	43934027	320	43933919	16	6.32064	3.57869
Nc-1-IP_pe	chr11	43936753	43937066	314	43936976	15	5.83691	3.4562
Nc-1-IP_pe	chr11	43941176	43941572	397	43941453	13	4.58338	3.0355
Nc-1-IP_pe	chr11	43942047	43942410	364	43942217	17	6.47874	3.54137
Nc-1-IP_pe	chr11	43944226	43944501	276	43944308	18	5.52764	3.02971
Nc-1-IP_pe	chr11	43952914	43953483	570	43953280	14	6.06519	3.69269
Nc-1-IP_pe	chr11	43959250	43959977	728	43959704	13	6.48901	4.06327
Nc-1-IP_pe	chr11	43969080	43969366	287	43969318	13	7.29853	4.52898
Nc-1-IP_pe	chr11	43982695	43982951	257	43982837	11	3.64839	2.72584
Nc-1-IP_pe	chr11	43983702	43984057	356	43983801	13	4.41954	2.952
Nc-1-IP_pe	chr11	43987412	43987989	578	43987770	16	7.53857	4.18505
Nc-1-IP_pe	chr11	43989071	43989448	378	43989426	13	3.35769	2.42919
Nc-1-IP_pe	chr11	43991461	43991793	333	43991670	10	3.08907	2.49869
Nc-1-IP_pe	chr11	44002190	44002653	464	44002409	11	4.33999	3.10858
Nc-1-IP_pe	chr11	44003903	44004384	482	44004172	12	4.70082	3.20033
Nc-1-IP_pe	chr11	44013342	44013603	262	44013531	13	5.38711	3.45645
Nc-1-IP_pe	chr11	44033939	44034282	344	44034060	16	8.03566	4.44361
Nc-1-IP_pe	chr11	44048005	44048321	317	44048150	15	8.16556	4.67894
Nc-1-IP_pe	chr11	44060603	44061130	528	44061051	10	4.50371	3.31737
Nc-1-IP_pe	chr11	44073229	44073975	747	44073386	16	6.8987	3.86161
Nc-1-IP_pe	chr11	44075662	44076145	484	44075947	11	4.47871	3.18705
Nc-1-IP_pe	chr11	44081097	44081480	384	44081327	12	4.63088	3.16234
Nc-1-IP_pe	chr11	44096184	44096557	374	44096380	21	5.88587	2.94733
Nc-1-IP_pe	chr11	44107450	44107960	511	44107845	14	5.838	3.57373
Nc-1-IP_pe	chr11	44119507	44119889	383	44119535	11	5.24175	3.6284
Nc-1-IP_pe	chr11	44146066	44146649	584	44146543	13	6.62436	4.14003
Nc-1-IP_pe	chr11	44235299	44235562	264	44235450	11	5.77463	3.94557
Nc-1-IP_pe	chr11	44258908	44259190	283	44259017	12	5.15877	3.45264
Nc-1-IP_pe	chr11	44259934	44260343	410	44260172	13	4.41954	2.952

Nc-1-IP_pe	chr11	44262039	44262391	353	44262202	10	3.49455	2.72742
Nc-1-IP_pe	chr11	44263189	44263573	385	44263451	12	3.83688	2.74114
Nc-1-IP_pe	chr11	44263891	44264566	676	44264020	17	5.9911	3.31919
Nc-1-IP_pe	chr11	44267145	44267595	451	44267475	14	5.57768	3.4392
Nc-1-IP_pe	chr11	44269574	44269944	371	44269834	14	4.60011	2.95114
Nc-1-IP_pe	chr11	44272433	44272701	269	44272438	11	2.96397	2.36091
Nc-1-IP_pe	chr11	44276146	44276545	400	44276389	18	8.10208	4.1823
Nc-1-IP_pe	chr11	44280072	44280471	400	44280259	17	5.9911	3.31919
Nc-1-IP_pe	chr11	44281291	44281546	256	44281318	10	3.53586	2.751
Nc-1-IP_pe	chr11	44286461	44287098	638	44286862	16	6.80394	3.81462
Nc-1-IP_pe	chr11	44288874	44289177	304	44289035	11	4.06403	2.95415
Nc-1-IP_pe	chr11	44293644	44294670	1027	44293706	15	7.50683	4.31879
Nc-1-IP_pe	chr11	44295452	44295990	539	44295909	12	5.23082	3.49289
Nc-1-IP_pe	chr11	44301429	44302554	1126	44302082	16	6.1093	3.47747
Nc-1-IP_pe	chr11	44303265	44303554	290	44303352	15	4.32392	2.74012
Nc-1-IP_pe	chr11	44304156	44304511	356	44304318	15	6.02372	3.54894
Nc-1-IP_pe	chr11	44308397	44310112	1716	44310038	19	6.32622	3.27682
Nc-1-IP_pe	chr11	44310821	44312049	1229	44311449	17	6.25544	3.43888
Nc-1-IP_pe	chr11	44316804	44317095	292	44316948	18	4.96968	2.80074
Nc-1-IP_pe	chr11	44317526	44317779	254	44317619	11	3.69531	2.75136
Nc-1-IP_pe	chr11	44318730	44319361	632	44319261	16	4.59224	2.7853
Nc-1-IP_pe	chr11	44320020	44320561	542	44320167	13	4.24481	2.8638
Nc-1-IP_pe	chr11	44327848	44328571	724	44328462	19	9.58801	4.75518
Nc-1-IP_pe	chr11	44333891	44334905	1015	44334678	18	9.94172	5.10499
Nc-1-IP_pe	chr11	44339104	44340371	1268	44340147	15	8.16556	4.67894
Nc-1-IP_pe	chr11	44347778	44348060	283	44347965	14	6.06519	3.69269
Nc-1-IP_pe	chr11	44348595	44348862	268	44348604	10	3.70758	2.84953
Nc-1-IP_pe	chr11	44350433	44350878	446	44350573	13	5.65792	3.60248
Nc-1-IP_pe	chr11	44354686	44355205	520	44355054	11	4.57016	3.23909
Nc-1-IP_pe	chr11	44362026	44362389	364	44362231	13	5.9487	3.76157
Nc-1-IP_pe	chr11	44365574	44366036	463	44365883	14	5.51541	3.4073
Nc-1-IP_pe	chr11	44381427	44381873	447	44381728	12	4.51041	3.09723
Nc-1-IP_pe	chr11	44384785	44385044	260	44384886	10	4.33501	3.21677
Nc-1-IP_pe	chr11	44396688	44397585	898	44397105	17	8.20861	4.37862
Nc-1-IP_pe	chr11	44407228	44407743	516	44407371	11	4.54404	3.2242
Nc-1-IP_pe	chr11	44418205	44418784	580	44418290	12	5.98129	3.92053
Nc-1-IP_pe	chr11	44423132	44423431	300	44423210	15	6.5895	3.83567
Nc-1-IP_pe	chr11	44443877	44444675	799	44444613	12	5.91141	3.88008
Nc-1-IP_pe	chr11	44446861	44447550	690	44447255	15	5.66913	3.37372
Nc-1-IP_pe	chr11	44451813	44452205	393	44452011	14	6.61011	3.98382
Nc-1-IP_pe	chr11	44454028	44454311	284	44454199	15	6.69889	3.8921
Nc-1-IP_pe	chr11	44460637	44461007	371	44460848	13	3.67377	2.58159
Nc-1-IP_pe	chr11	44465422	44465682	261	44465602	11	4.14834	3.00109
Nc-1-IP_pe	chr11	44467553	44468085	533	44467975	12	4.92484	3.32299
Nc-1-IP_pe	chr11	44473130	44473664	535	44473217	12	4.24064	2.95299

Nc-1-IP_pe	chr11	44474320	44474582	263	44474498	13	5.15976	3.33548
Nc-1-IP_pe	chr11	44483871	44484429	559	44484066	15	4.40338	2.77622
Nc-1-IP_pe	chr11	44489493	44489825	333	44489695	11	4.96316	3.46542
Nc-1-IP_pe	chr11	44506339	44506952	614	44506800	18	5.72638	3.11298
Nc-1-IP_pe	chr11	44514918	44515206	289	44515139	14	3.86727	2.6027
Nc-1-IP_pe	chr11	44522982	44523236	255	44523110	15	5.83691	3.4562
Nc-1-IP_pe	chr11	44523658	44524042	385	44523697	12	2.87625	2.25568
Nc-1-IP_pe	chr11	44527386	44527712	327	44527561	12	4.10922	2.88351
Nc-1-IP_pe	chr11	44536795	44537071	277	44536972	12	4.29266	2.98064
Nc-1-IP_pe	chr11	44539985	44540489	505	44540279	12	3.83688	2.74114
Nc-1-IP_pe	chr11	44562987	44563380	394	44563195	17	8.04728	4.29732
Nc-1-IP_pe	chr11	44566041	44566877	837	44566343	16	6.86413	3.84444
Nc-1-IP_pe	chr11	44568455	44569262	808	44568958	15	4.7819	2.95039
Nc-1-IP_pe	chr11	44569888	44571290	1403	44570094	21	11.8007	5.502
Nc-1-IP_pe	chr11	44572654	44573242	589	44572981	16	8.59821	4.74362
Nc-1-IP_pe	chr11	44579705	44580066	362	44579898	17	7.6885	4.11887
Nc-1-IP_pe	chr11	44580840	44581399	560	44581337	14	5.5512	3.42562
Nc-1-IP_pe	chr11	44590712	44591721	1010	44591333	13	5.36853	3.44651
Nc-1-IP_pe	chr11	44593228	44593534	307	44593240	15	4.06016	2.62146
Nc-1-IP_pe	chr11	44594358	44595020	663	44594643	14	5.03085	3.16286
Nc-1-IP_pe	chr11	44596112	44596544	433	44596418	12	4.24064	2.95299
Nc-1-IP_pe	chr11	44598744	44598999	256	44598915	11	4.01189	2.92523
Nc-1-IP_pe	chr11	44599527	44600174	648	44599639	13	5.29187	3.40559
Nc-1-IP_pe	chr11	44601042	44602148	1107	44601616	15	6.60497	3.84363
Nc-1-IP_pe	chr11	44603363	44603631	269	44603392	14	4.8399	3.06836
Nc-1-IP_pe	chr11	44606947	44607217	271	44607072	17	4.78101	2.79343
Nc-1-IP_pe	chr11	44611305	44611790	486	44611458	16	6.89451	3.85952
Nc-1-IP_pe	chr11	44614813	44615154	342	44615068	18	5.72638	3.11298
Nc-1-IP_pe	chr11	44617798	44618350	553	44617974	16	5.82808	3.34463
Nc-1-IP_pe	chr11	44618662	44619238	577	44619109	15	6.03738	3.55576
Nc-1-IP_pe	chr11	44619966	44620431	466	44620168	17	7.80546	4.17669
Nc-1-IP_pe	chr11	44621474	44623012	1539	44621648	15	4.40338	2.77622
Nc-1-IP_pe	chr11	44628808	44629081	274	44628893	15	6.5434	3.81197
Nc-1-IP_pe	chr11	44630293	44630791	499	44630392	11	3.64839	2.72584
Nc-1-IP_pe	chr11	44692326	44692947	622	44692439	12	4.70082	3.20033
Nc-1-IP_pe	chr11	44694112	44694602	491	44694572	14	3.55308	2.45762
Nc-1-IP_pe	chr11	44701363	44701626	264	44701537	11	3.89191	2.85898
Nc-1-IP_pe	chr11	44707676	44707968	293	44707784	14	5.7679	3.53731
Nc-1-IP_pe	chr11	44709003	44709400	398	44709245	13	4.86362	3.18015
Nc-1-IP_pe	chr11	44718763	44719051	289	44718850	15	7.63952	4.39047
Nc-1-IP_pe	chr11	44722368	44722750	383	44722647	11	4.6642	3.29286
Nc-1-IP_pe	chr11	44724964	44725405	442	44725256	15	7.19874	4.15407
Nc-1-IP_pe	chr11	44726362	44726649	288	44726468	15	4.06016	2.62146
Nc-1-IP_pe	chr11	44727865	44728229	365	44728030	16	4.59224	2.7853
Nc-1-IP_pe	chr11	44739807	44740675	869	44740209	16	5.37454	3.13479

Nc-1-IP_pe	chr11	44742494	44742789	296	44742683	12	4.24064	2.95299
Nc-1-IP_pe	chr11	44743486	44743786	301	44743565	15	6.19428	3.63445
Nc-1-IP_pe	chr11	44746860	44747355	496	44747119	16	8.57707	4.73221
Nc-1-IP_pe	chr11	44747983	44748344	362	44748177	11	4.88957	3.42271
Nc-1-IP_pe	chr11	44755306	44755566	261	44755442	13	5.71736	3.63481
Nc-1-IP_pe	chr11	44761824	44762174	351	44762086	10	4.25398	3.16873
Nc-1-IP_pe	chr11	44762931	44763374	444	44763097	11	2.96397	2.36091
Nc-1-IP_pe	chr11	44764609	44764863	255	44764726	13	4.45253	2.96876
Nc-1-IP_pe	chr11	44770989	44771273	285	44771136	12	4.88508	3.30112
Nc-1-IP_pe	chr11	44785797	44786542	746	44786220	14	4.21449	2.76599
Nc-1-IP_pe	chr11	44790968	44791856	889	44791093	14	4.21449	2.76599
Nc-1-IP_pe	chr11	44792865	44793191	327	44793061	10	3.76347	2.88178
Nc-1-IP_pe	chr11	44795755	44796021	267	44795934	13	5.85439	3.70972
Nc-1-IP_pe	chr11	44798365	44798619	255	44798552	14	5.98179	3.64885
Nc-1-IP_pe	chr11	44804812	44805093	282	44804928	15	5.70822	3.39286
Nc-1-IP_pe	chr11	44807743	44808038	296	44807855	15	6.82712	3.95868
Nc-1-IP_pe	chr11	44812829	44813265	437	44813146	15	7.37761	4.24941
Nc-1-IP_pe	chr11	44834926	44835314	389	44835125	9	2.89249	2.46179
Nc-1-IP_pe	chr11	44851573	44851954	382	44851720	13	3.35769	2.42919
Nc-1-IP_pe	chr11	44854399	44854726	328	44854520	14	5.7264	3.51582
Nc-1-IP_pe	chr11	44868459	44868921	463	44868733	13	3.07238	2.29378
Nc-1-IP_pe	chr11	44874890	44875238	349	44874960	12	4.39405	3.03475
Nc-1-IP_pe	chr11	44876931	44877212	282	44877100	15	4.06016	2.62146
Nc-1-IP_pe	chr11	44880664	44880979	316	44880832	21	7.10961	3.41419
Nc-1-IP_pe	chr11	44882769	44883058	290	44882973	13	4.02562	2.7544
Nc-1-IP_pe	chr11	44883395	44883668	274	44883497	14	5.03085	3.16286
Nc-1-IP_pe	chr11	44884420	44884742	323	44884553	13	3.35769	2.42919
Nc-1-IP_pe	chr11	44888448	44888726	279	44888602	13	5.13388	3.3218
Nc-1-IP_pe	chr11	44892142	44893426	1285	44893014	15	6.20456	3.63963
Nc-1-IP_pe	chr11	44899526	44899976	451	44899858	13	5.22525	3.37017
Nc-1-IP_pe	chr11	44901066	44901469	404	44901330	12	4.39405	3.03475
Nc-1-IP_pe	chr11	44902544	44902837	294	44902567	16	3.94146	2.50592
Nc-1-IP_pe	chr11	44909896	44910474	579	44910070	15	5.66913	3.37372
Nc-1-IP_pe	chr11	44911585	44911924	340	44911813	19	7.84735	3.93486
Nc-1-IP_pe	chr11	44916266	44916518	253	44916390	17	8.6634	4.61125
Nc-1-IP_pe	chr11	44916954	44917425	472	44917149	11	3.64839	2.72584
Nc-1-IP_pe	chr11	44918715	44919081	367	44919062	11	3.64839	2.72584
Nc-1-IP_pe	chr11	44922706	44923160	455	44923002	14	4.62572	2.96359
Nc-1-IP_pe	chr11	44924356	44924658	303	44924461	15	5.90272	3.48876
Nc-1-IP_pe	chr11	44927016	44927491	476	44927407	16	4.09083	2.56919
Nc-1-IP_pe	chr11	44927799	44928077	279	44927921	19	6.32622	3.27682
Nc-1-IP_pe	chr11	44934728	44935457	730	44935102	19	8.71852	4.33643
Nc-1-IP_pe	chr11	44936661	44937697	1037	44936987	20	7.32334	3.59641
Nc-1-IP_pe	chr11	44938315	44939397	1083	44938962	19	7.84735	3.93486
Nc-1-IP_pe	chr11	44941001	44941625	625	44941513	13	4.02562	2.7544

Nc-1-IP_pe	chr11	44942499	44943245	747	44943003	17	4.78101	2.79343
Nc-1-IP_pe	chr11	44946386	44946669	284	44946488	17	6.1569	3.39405
Nc-1-IP_pe	chr11	44947327	44947747	421	44947586	15	4.14926	2.66135
Nc-1-IP_pe	chr11	44948789	44949699	911	44949580	13	3.76686	2.62698
Nc-1-IP_pe	chr11	44954571	44954992	422	44954925	15	3.98281	2.58698
Nc-1-IP_pe	chr11	44957011	44957440	430	44957223	17	6.64406	3.61808
Nc-1-IP_pe	chr11	44960011	44960340	330	44960046	13	3.44439	2.47074
Nc-1-IP_pe	chr11	44962863	44963511	649	44963105	18	7.82684	4.0513
Nc-1-IP_pe	chr11	44972196	44973066	871	44972933	13	5.25176	3.38425
Nc-1-IP_pe	chr11	44976770	44977286	517	44977184	13	3.86418	2.67468
Nc-1-IP_pe	chr11	44979454	44980036	583	44979656	18	4.04135	2.43445
Nc-1-IP_pe	chr11	44980815	44981192	378	44980887	12	3.47963	2.55766
Nc-1-IP_pe	chr11	44989970	44990473	504	44990408	13	4.67336	3.0817
Nc-1-IP_pe	chr11	44990923	44991528	606	44991381	15	6.89257	3.99282
Nc-1-IP_pe	chr11	44992229	44992688	460	44992426	15	6.8109	3.95023
Nc-1-IP_pe	chr11	45017226	45017562	337	45017411	16	5.98198	3.41707
Nc-1-IP_pe	chr11	45027015	45027514	500	45027377	17	7.52577	4.03901
Nc-1-IP_pe	chr11	45036595	45037036	442	45036814	18	10.5351	5.41929
Nc-1-IP_pe	chr11	45044157	45044523	367	45044278	14	5.86637	3.5885
Nc-1-IP_pe	chr11	45049080	45049403	324	45049113	12	3.86684	2.7567
Nc-1-IP_pe	chr11	45052055	45052596	542	45052234	16	7.11257	3.96852
Nc-1-IP_pe	chr11	45053090	45053650	561	45053231	17	5.9911	3.31919
Nc-1-IP_pe	chr11	45054077	45054504	428	45054273	14	3.55308	2.45762
Nc-1-IP_pe	chr11	45069012	45069526	515	45069210	15	7.69749	4.42193
Nc-1-IP_pe	chr11	45080170	45081398	1229	45080460	13	3.67377	2.58159
Nc-1-IP_pe	chr11	45085896	45086439	544	45086316	16	5.73763	3.30235
Nc-1-IP_pe	chr11	45088047	45088336	290	45088192	15	5.20142	3.14789
Nc-1-IP_pe	chr11	45091807	45092295	489	45091927	11	4.11366	2.98176
Nc-1-IP_pe	chr11	45094827	45095626	800	45095434	20	9.82443	4.71218
Nc-1-IP_pe	chr11	45098142	45098442	301	45098233	13	4.81267	3.15368
Nc-1-IP_pe	chr11	45101670	45102140	471	45102137	27	3.22048	1.87297
Nc-1-IP_pe	chr11	45109197	45109643	447	45109340	16	7.08082	3.95257
Nc-1-IP_pe	chr11	45133631	45134060	430	45133926	13	6.19316	3.89711
Nc-1-IP_pe	chr11	45147364	45147750	387	45147456	19	6.78661	3.47027
Nc-1-IP_pe	chr11	45184961	45185237	277	45184993	12	3.94559	2.79771
Nc-1-IP_pe	chr11	45194700	45194994	295	45194878	11	5.05359	3.5181
Nc-1-IP_pe	chr11	45195595	45195862	268	45195732	12	4.70082	3.20033
Nc-1-IP_pe	chr11	45206066	45206494	429	45206417	12	4.24064	2.95299
Nc-1-IP_pe	chr11	45206850	45207127	278	45207004	15	4.06016	2.62146
Nc-1-IP_pe	chr11	45208984	45209326	343	45209172	15	5.20142	3.14789
Nc-1-IP_pe	chr11	45210000	45210595	596	45210510	11	3.28475	2.53029
Nc-1-IP_pe	chr11	45212829	45213099	271	45213026	22	6.07094	2.94697
Nc-1-IP_pe	chr11	45217459	45217786	328	45217590	16	7.64983	4.24237
Nc-1-IP_pe	chr11	45219422	45219855	434	45219674	14	5.03085	3.16286
Nc-1-IP_pe	chr11	45224623	45224920	298	45224736	14	5.69896	3.50164

Nc-1-IP_pe	chr11	45226140	45226792	653	45226689	13	6.07713	3.83258
Nc-1-IP_pe	chr11	45227801	45228083	283	45227933	12	5.59553	3.69882
Nc-1-IP_pe	chr11	45232367	45232867	501	45232576	11	4.8033	3.37283
Nc-1-IP_pe	chr11	45233288	45233910	623	45233788	19	6.78661	3.47027
Nc-1-IP_pe	chr11	45243982	45244297	316	45244156	14	7.42849	4.43574
Nc-1-IP_pe	chr11	45245829	45246560	732	45245951	11	5.00808	3.49156
Nc-1-IP_pe	chr11	45251832	45252803	972	45252363	16	5.37454	3.13479
Nc-1-IP_pe	chr11	45267483	45268289	807	45267584	14	3.86727	2.6027
Nc-1-IP_pe	chr11	45281487	45282198	712	45281734	12	3.16137	2.39719
Nc-1-IP_pe	chr11	45285981	45286374	394	45286178	19	7.73663	3.88512
Nc-1-IP_pe	chr11	45321786	45322381	596	45321931	11	4.35236	3.11555
Nc-1-IP_pe	chr11	45338101	45338471	371	45338299	12	5.40861	3.59282
Nc-1-IP_pe	chr11	45342611	45342986	376	45342981	12	5.77527	3.80164
Nc-1-IP_pe	chr11	45355297	45355787	491	45355378	10	4.39571	3.25288
Nc-1-IP_pe	chr11	45368875	45369509	635	45369024	15	6.85973	3.97568
Nc-1-IP_pe	chr11	45369928	45370195	268	45369935	10	3.08907	2.49869
Nc-1-IP_pe	chr11	45371203	45371661	459	45371385	15	5.20142	3.14789
Nc-1-IP_pe	chr11	45384262	45385863	1602	45385517	12	4.88508	3.30112
Nc-1-IP_pe	chr11	45390841	45391906	1066	45391450	10	3.66365	2.82424
Nc-1-IP_pe	chr11	45393668	45394168	501	45393990	13	5.86991	3.71823
Nc-1-IP_pe	chr11	45398404	45398982	579	45398582	15	7.56326	4.34922
Nc-1-IP_pe	chr11	45407189	45407523	335	45407382	15	5.20142	3.14789
Nc-1-IP_pe	chr11	45412278	45412824	547	45412340	12	4.95159	3.33774
Nc-1-IP_pe	chr11	45427884	45428337	454	45428143	13	6.72991	4.20021
Nc-1-IP_pe	chr11	45431141	45431432	292	45431362	11	5.14641	3.5724
Nc-1-IP_pe	chr11	45444550	45445066	517	45444708	13	5.36853	3.44651
Nc-1-IP_pe	chr11	45464289	45464548	260	45464362	15	6.69889	3.8921
Nc-1-IP_pe	chr11	45467980	45468327	348	45468186	11	4.81752	3.38105
Nc-1-IP_pe	chr11	45469290	45469562	273	45469445	12	5.33338	3.55042
Nc-1-IP_pe	chr11	45470505	45471134	630	45470691	13	6.02837	3.80557
Nc-1-IP_pe	chr11	45482261	45483038	778	45482401	12	4.52227	3.10362
Nc-1-IP_pe	chr11	45487732	45488212	481	45488170	10	4.17505	3.1221
Nc-1-IP_pe	chr11	45494843	45495406	564	45495222	12	6.58417	4.27436
Nc-1-IP_pe	chr11	45501693	45502484	792	45502231	14	5.51541	3.4073
Nc-1-IP_pe	chr11	45505952	45506317	366	45506264	11	4.06403	2.95415
Nc-1-IP_pe	chr11	45511825	45512083	259	45511887	15	5.20142	3.14789
Nc-1-IP_pe	chr11	45514026	45514483	458	45514374	11	5.45748	3.75598
Nc-1-IP_pe	chr11	45527612	45527976	365	45527806	16	7.75718	4.29799
Nc-1-IP_pe	chr11	45548474	45548732	259	45548583	14	6.13058	3.7272
Nc-1-IP_pe	chr11	45551172	45551516	345	45551350	23	14.3414	6.37411
Nc-1-IP_pe	chr11	45563955	45564251	297	45564090	12	4.57012	3.12944
Nc-1-IP_pe	chr11	45571991	45572380	390	45572240	13	5.42865	3.47872
Nc-1-IP_pe	chr11	45574335	45574597	263	45574434	13	5.29187	3.40559
Nc-1-IP_pe	chr11	45574943	45575244	302	45575026	11	4.47871	3.18705
Nc-1-IP_pe	chr11	45576442	45576807	366	45576619	14	6.69566	4.03025

Nc-1-IP_pe	chr11	45582140	45582396	257	45582305	15	5.66913	3.37372
Nc-1-IP_pe	chr11	45584622	45585307	686	45585153	12	5.84275	3.84046
Nc-1-IP_pe	chr11	45590883	45591561	679	45591354	12	5.23082	3.49289
Nc-1-IP_pe	chr11	45593016	45593523	508	45593066	10	3.8436	2.92819
Nc-1-IP_pe	chr11	45604163	45604558	396	45604354	16	4.59224	2.7853
Nc-1-IP_pe	chr11	45607909	45608556	648	45608432	14	6.23788	3.78407
Nc-1-IP_pe	chr11	45620891	45621777	887	45621712	15	5.20142	3.14789
Nc-1-IP_pe	chr11	45624274	45624540	267	45624359	15	6.19428	3.63445
Nc-1-IP_pe	chr11	45635679	45636009	331	45635878	11	4.54404	3.2242
Nc-1-IP_pe	chr11	45644876	45645501	626	45645041	11	4.78913	3.36466
Nc-1-IP_pe	chr11	45646350	45646618	269	45646567	11	4.49162	3.19439
Nc-1-IP_pe	chr11	45649262	45649720	459	45649700	12	5.23082	3.49289
Nc-1-IP_pe	chr11	45650545	45651049	505	45650874	18	7.73136	4.00629
Nc-1-IP_pe	chr11	45652168	45652920	753	45652302	15	7.37761	4.24941
Nc-1-IP_pe	chr11	45655354	45656111	758	45655620	12	3.47963	2.55766
Nc-1-IP_pe	chr11	45658862	45659590	729	45658914	13	3.35769	2.42919
Nc-1-IP_pe	chr11	45661840	45662424	585	45662046	12	4.70523	3.20273
Nc-1-IP_pe	chr11	45664088	45665535	1448	45664826	17	7.6885	4.11887
Nc-1-IP_pe	chr11	45670079	45670652	574	45670133	12	4.88508	3.30112
Nc-1-IP_pe	chr11	45678051	45678516	466	45678335	14	3.86727	2.6027
Nc-1-IP_pe	chr11	45678987	45679285	299	45679112	10	3.44377	2.69851
Nc-1-IP_pe	chr11	45681054	45681436	383	45681241	17	5.9911	3.31919
Nc-1-IP_pe	chr11	45692593	45692881	289	45692865	12	5.01934	3.37518
Nc-1-IP_pe	chr11	45696469	45697018	550	45696882	12	3.16137	2.39719
Nc-1-IP_pe	chr11	45716640	45717048	409	45716721	16	7.11257	3.96852
Nc-1-IP_pe	chr11	45737993	45738271	279	45738152	16	7.79347	4.31685
Nc-1-IP_pe	chr11	45738706	45739104	399	45738906	16	4.96462	2.94973
Nc-1-IP_pe	chr11	45755490	45755848	359	45755752	13	4.67336	3.0817
Nc-1-IP_pe	chr11	45758428	45758754	327	45758744	10	3.4604	2.70797
Nc-1-IP_pe	chr11	45771529	45772119	591	45771626	17	7.36791	3.96218
Nc-1-IP_pe	chr11	45788563	45788817	255	45788714	11	5.03835	3.5092
Nc-1-IP_pe	chr11	45793034	45793659	626	45793146	9	3.93633	3.08822
Nc-1-IP_pe	chr11	45803969	45805292	1324	45804189	24	9.05651	3.87976
Nc-1-IP_pe	chr11	45807785	45808938	1154	45807965	17	6.64406	3.61808
Nc-1-IP_pe	chr11	45810985	45811282	298	45811037	14	3.55308	2.45762
Nc-1-IP_pe	chr11	45816999	45817256	258	45817076	13	5.25176	3.38425
Nc-1-IP_pe	chr11	45823965	45824286	322	45824122	16	6.33285	3.58457
Nc-1-IP_pe	chr11	45831754	45832200	447	45831972	15	7.34131	4.22999
Nc-1-IP_pe	chr11	45847469	45847901	433	45847631	24	6.49734	2.96505
Nc-1-IP_pe	chr11	45869322	45869586	265	45869495	12	3.16137	2.39719
Nc-1-IP_pe	chr11	45887765	45888469	705	45887874	12	4.24064	2.95299
Nc-1-IP_pe	chr11	45894342	45895557	1216	45895375	15	6.09241	3.58328
Nc-1-IP_pe	chr11	45895883	45896787	905	45896490	21	6.66991	3.24296
Nc-1-IP_pe	chr11	45897610	45898234	625	45897850	20	9.21699	4.428
Nc-1-IP_pe	chr11	45898881	45900593	1713	45900506	18	5.94685	3.20644

Nc-1-IP_pe	chr11	45902091	45902350	260	45902248	12	3.96559	2.80816
Nc-1-IP_pe	chr11	45902727	45903363	637	45902897	18	7.73287	4.007
Nc-1-IP_pe	chr11	45905687	45906284	598	45906148	19	6.32622	3.27682
Nc-1-IP_pe	chr11	45908215	45908579	365	45908406	14	3.55308	2.45762
Nc-1-IP_pe	chr11	45908948	45909780	833	45909701	19	7.67869	3.85921
Nc-1-IP_pe	chr11	45910785	45911307	523	45910983	16	5.18047	3.04664
Nc-1-IP_pe	chr11	45914284	45914769	486	45914368	22	6.44373	3.08129
Nc-1-IP_pe	chr11	45917430	45917777	348	45917674	23	7.3183	3.32328
Nc-1-IP_pe	chr11	45929510	45929880	371	45929616	17	5.9911	3.31919
Nc-1-IP_pe	chr11	45930156	45930875	720	45930654	19	7.88414	3.95145
Nc-1-IP_pe	chr11	45931920	45932302	383	45932076	14	4.52309	2.91384
Nc-1-IP_pe	chr11	45933926	45934275	350	45934138	19	6.32622	3.27682
Nc-1-IP_pe	chr11	45934829	45935310	482	45935043	13	4.67336	3.0817
Nc-1-IP_pe	chr11	45967126	45967423	298	45967301	13	5.29187	3.40559
Nc-1-IP_pe	chr11	45993804	45994125	322	45993979	12	5.87693	3.86017
Nc-1-IP_pe	chr11	46021208	46021663	456	46021565	9	3.27326	2.68684
Nc-1-IP_pe	chr11	46022673	46022932	260	46022753	10	4.46067	3.29163
Nc-1-IP_pe	chr11	46121917	46122417	501	46122188	16	6.8987	3.86161
Nc-1-IP_pe	chr11	46134656	46134911	256	46134801	12	5.23082	3.49289
Nc-1-IP_pe	chr11	46136163	46136538	376	46136502	11	3.89191	2.85898
Nc-1-IP_pe	chr11	46143004	46143675	672	46143235	19	7.84735	3.93486
Nc-1-IP_pe	chr11	46186153	46186440	288	46186298	18	10.3389	5.3145
Nc-1-IP_pe	chr11	46201182	46201450	269	46201368	14	5.43414	3.36584
Nc-1-IP_pe	chr11	46207651	46207967	317	46207754	20	10.7672	5.16976
Nc-1-IP_pe	chr11	46213349	46213738	390	46213516	19	10.5515	5.23961
Nc-1-IP_pe	chr11	46216830	46217167	338	46216940	17	9.20132	4.89288
Nc-1-IP_pe	chr11	46238044	46238741	698	46238204	16	4.3356	2.67395
Nc-1-IP_pe	chr11	46239030	46239590	561	46239161	20	7.32334	3.59641
Nc-1-IP_pe	chr11	46251378	46252186	809	46251478	14	5.53803	3.41887
Nc-1-IP_pe	chr11	46260309	46261114	806	46260358	15	6.82712	3.95868
Nc-1-IP_pe	chr11	46271784	46272117	334	46271911	14	4.60011	2.95114
Nc-1-IP_pe	chr11	46273898	46274754	857	46274234	14	5.04358	3.16919
Nc-1-IP_pe	chr11	46278242	46278809	568	46278325	17	5.14803	2.94914
Nc-1-IP_pe	chr11	46289712	46290540	829	46290381	17	5.14803	2.94914
Nc-1-IP_pe	chr11	46290862	46291137	276	46290903	15	5.1177	3.10809
Nc-1-IP_pe	chr11	46291771	46292416	646	46292275	17	5.14803	2.94914
Nc-1-IP_pe	chr11	46294151	46294440	290	46294366	15	5.58327	3.33181
Nc-1-IP_pe	chr11	46294748	46295534	787	46295186	18	5.83846	3.16035
Nc-1-IP_pe	chr11	46296106	46297273	1168	46296299	16	6.29369	3.56571
Nc-1-IP_pe	chr11	46299996	46300816	821	46300200	18	6.15736	3.29676
Nc-1-IP_pe	chr11	46308828	46309213	386	46308999	18	6.15736	3.29676
Nc-1-IP_pe	chr11	46312666	46313142	477	46313022	12	4.13028	2.89461
Nc-1-IP_pe	chr11	46316762	46317919	1158	46317530	14	5.30793	3.30182
Nc-1-IP_pe	chr11	46318891	46319359	469	46318920	17	3.33947	2.20998
Nc-1-IP_pe	chr11	46322712	46323141	430	46322924	13	4.02562	2.7544

Nc-1-IP_pe	chr11	46327949	46328283	335	46328168	13	4.14549	2.81406
Nc-1-IP_pe	chr11	46329709	46329971	263	46329744	12	3.88634	2.76684
Nc-1-IP_pe	chr11	46332646	46334014	1369	46333108	22	6.07094	2.94697
Nc-1-IP_pe	chr11	46335610	46336079	470	46335922	16	4.96462	2.94973
Nc-1-IP_pe	chr11	46340679	46341065	387	46340699	14	3.33731	2.35937
Nc-1-IP_pe	chr11	46345227	46345727	501	46345524	16	4.87846	2.91138
Nc-1-IP_pe	chr11	46348733	46349336	604	46348917	15	4.80057	2.95908
Nc-1-IP_pe	chr11	46351363	46352649	1287	46352439	18	7.61002	3.94942
Nc-1-IP_pe	chr11	46353348	46354187	840	46353849	17	5.54967	3.12324
Nc-1-IP_pe	chr11	46355398	46356460	1063	46355646	13	4.34711	2.91533
Nc-1-IP_pe	chr11	46357437	46358082	646	46357826	20	7.43645	3.64378
Nc-1-IP_pe	chr11	46366022	46366324	303	46366275	16	4.75427	2.85643
Nc-1-IP_pe	chr11	46366631	46368243	1613	46366780	19	4.5193	2.56258
Nc-1-IP_pe	chr11	46370023	46370353	331	46370170	16	5.6319	3.2532
Nc-1-IP_pe	chr11	46371017	46371727	711	46371288	17	6.33071	3.47329
Nc-1-IP_pe	chr11	46372056	46372737	682	46372243	16	5.93204	3.39349
Nc-1-IP_pe	chr11	46373697	46373980	284	46373881	15	4.06016	2.62146
Nc-1-IP_pe	chr11	46375595	46375925	331	46375833	17	6.25544	3.43888
Nc-1-IP_pe	chr11	46376219	46376928	710	46376274	16	5.42783	3.15917
Nc-1-IP_pe	chr11	46378061	46378968	908	46378125	17	5.83879	3.25103
Nc-1-IP_pe	chr11	46379620	46380223	604	46379909	22	6.07094	2.94697
Nc-1-IP_pe	chr11	46380798	46381530	733	46381301	27	12.8757	5.01024
Nc-1-IP_pe	chr11	46384444	46384762	319	46384630	18	4.04135	2.43445
Nc-1-IP_pe	chr11	46385254	46385636	383	46385415	16	4.59224	2.7853
Nc-1-IP_pe	chr11	46386197	46386567	371	46386449	18	7.11704	3.7221
Nc-1-IP_pe	chr11	46387533	46388141	609	46387777	20	8.36892	4.04542
Nc-1-IP_pe	chr11	46389184	46390911	1728	46390471	19	7.94423	3.97862
Nc-1-IP_pe	chr11	46395773	46396398	626	46396254	17	6.47874	3.54137
Nc-1-IP_pe	chr11	46396710	46396985	276	46396862	13	4.36794	2.92586
Nc-1-IP_pe	chr11	46397475	46398163	689	46397607	17	6.47874	3.54137
Nc-1-IP_pe	chr11	46404762	46405151	390	46404859	13	4.86362	3.18015
Nc-1-IP_pe	chr11	46408480	46408790	311	46408580	17	6.47874	3.54137
Nc-1-IP_pe	chr11	46414456	46414745	290	46414599	14	4.52309	2.91384
Nc-1-IP_pe	chr11	46424583	46425131	549	46425057	13	4.86362	3.18015
Nc-1-IP_pe	chr11	46425592	46425894	303	46425859	10	4.11081	3.08427
Nc-1-IP_pe	chr11	46428708	46429395	688	46428962	18	4.04135	2.43445
Nc-1-IP_pe	chr11	46447191	46447470	280	46447295	16	7.97877	4.4137
Nc-1-IP_pe	chr11	46468304	46469139	836	46468897	11	4.99304	3.4828
Nc-1-IP_pe	chr11	46471301	46471788	488	46471384	14	6.96097	4.17548
Nc-1-IP_pe	chr11	46484759	46485383	625	46484895	12	4.24064	2.95299
Nc-1-IP_pe	chr11	46505667	46506013	347	46505765	12	4.28158	2.97474
Nc-1-IP_pe	chr11	46525076	46525608	533	46525526	12	3.77112	2.7071
Nc-1-IP_pe	chr11	46534297	46534889	593	46534766	18	7.73136	4.00629
Nc-1-IP_pe	chr11	46542477	46542820	344	46542542	12	3.86684	2.7567
Nc-1-IP_pe	chr11	46551889	46552168	280	46551905	12	3.47963	2.55766

Nc-1-IP_pe	chr11	46567292	46567635	344	46567557	14	5.06689	3.18081
Nc-1-IP_pe	chr11	46571794	46572255	462	46572206	11	3.28475	2.53029
Nc-1-IP_pe	chr11	46573059	46573330	272	46573158	14	6.92464	4.15548
Nc-1-IP_pe	chr11	46578542	46578806	265	46578656	13	5.9487	3.76157
Nc-1-IP_pe	chr11	46586006	46586355	350	46586228	12	5.91141	3.88008
Nc-1-IP_pe	chr11	46593388	46594123	736	46593640	24	11.6643	4.93931
Nc-1-IP_pe	chr11	46595085	46595443	359	46595295	13	4.86362	3.18015
Nc-1-IP_pe	chr11	46595705	46596545	841	46596197	15	6.19428	3.63445
Nc-1-IP_pe	chr11	46605800	46606056	257	46605849	11	3.64839	2.72584
Nc-1-IP_pe	chr11	46606395	46606761	367	46606399	10	3.40382	2.67582
Nc-1-IP_pe	chr11	46611117	46611402	286	46611171	10	3.82047	2.91478
Nc-1-IP_pe	chr11	46623022	46623332	311	46623230	14	5.51541	3.4073
Nc-1-IP_pe	chr11	46633438	46633724	287	46633630	12	5.37834	3.57574
Nc-1-IP_pe	chr11	46634269	46634611	343	46634376	12	5.01934	3.37518
Nc-1-IP_pe	chr11	46647463	46647743	281	46647559	12	5.92876	3.89011
Nc-1-IP_pe	chr11	46654547	46655298	752	46654816	18	9.82901	5.04617
Nc-1-IP_pe	chr11	46667145	46667403	259	46667246	15	6.19428	3.63445
Nc-1-IP_pe	chr11	46668277	46668586	310	46668550	12	3.16137	2.39719
Nc-1-IP_pe	chr11	46675728	46676030	303	46675890	14	5.27082	3.28308
Nc-1-IP_pe	chr11	46676505	46676874	370	46676541	11	3.28559	2.53074
Nc-1-IP_pe	chr11	46678578	46679231	654	46678868	15	4.80057	2.95908
Nc-1-IP_pe	chr11	46679670	46680001	332	46679801	18	7.59486	3.94234
Nc-1-IP_pe	chr11	46682211	46682545	335	46682394	15	4.80057	2.95908
Nc-1-IP_pe	chr11	46686366	46686934	569	46686819	15	5.20142	3.14789
Nc-1-IP_pe	chr11	46688134	46688438	305	46688265	13	5.36853	3.44651
Nc-1-IP_pe	chr11	46694267	46694572	306	46694365	19	6.78661	3.47027
Nc-1-IP_pe	chr11	46695119	46695390	272	46695207	14	5.33286	3.31443
Nc-1-IP_pe	chr11	46695690	46695972	283	46695961	14	3.86727	2.6027
Nc-1-IP_pe	chr11	46697004	46698073	1070	46697563	18	7.64048	3.96366
Nc-1-IP_pe	chr11	46699908	46701630	1723	46700934	22	7.04484	3.3034
Nc-1-IP_pe	chr11	46705011	46705482	472	46705074	16	3.94146	2.50592
Nc-1-IP_pe	chr11	46705759	46706029	271	46705833	13	4.80086	3.14755
Nc-1-IP_pe	chr11	46719548	46719834	287	46719756	19	7.59289	3.82098
Nc-1-IP_pe	chr11	46725867	46726472	606	46726316	17	5.9911	3.31919
Nc-1-IP_pe	chr11	46727758	46728217	460	46728023	12	3.83688	2.74114
Nc-1-IP_pe	chr11	46735153	46735450	298	46735319	16	6.8987	3.86161
Nc-1-IP_pe	chr11	46740144	46740821	678	46740406	15	6.19428	3.63445
Nc-1-IP_pe	chr11	46756909	46757369	461	46757317	14	7.30138	4.36445
Nc-1-IP_pe	chr11	46785408	46785788	381	46785477	11	6.00992	4.08766
Nc-1-IP_pe	chr11	46821505	46821780	276	46821704	8	3.74505	3.07688
Nc-1-IP_pe	chr11	46831660	46831923	264	46831852	10	4.39571	3.25288
Nc-1-IP_pe	chr11	46833794	46834075	282	46833928	14	8.44792	5.0209
Nc-1-IP_pe	chr11	46848115	46848438	324	46848403	11	3.28475	2.53029
Nc-1-IP_pe	chr11	46858862	46859251	390	46859137	13	4.41954	2.952
Nc-1-IP_pe	chr11	46868899	46869245	347	46869125	16	7.54467	4.18818

Nc-1-IP_pe	chr11	46872239	46872497	259	46872349	13	3.07238	2.29378
Nc-1-IP_pe	chr11	46873253	46873508	256	46873317	15	4.06016	2.62146
Nc-1-IP_pe	chr11	46876419	46877148	730	46876612	17	7.06589	3.81697
Nc-1-IP_pe	chr11	46878808	46879358	551	46878964	12	3.58823	2.61305
Nc-1-IP_pe	chr11	46897888	46898259	372	46898224	14	3.26733	2.32773
Nc-1-IP_pe	chr11	46899261	46899581	321	46899305	18	3.88695	2.37515
Nc-1-IP_pe	chr11	46899855	46900159	305	46900114	17	5.9911	3.31919
Nc-1-IP_pe	chr11	46902698	46902957	260	46902849	16	5.82808	3.34463
Nc-1-IP_pe	chr11	46903821	46904164	344	46903999	17	7.02067	3.79543
Nc-1-IP_pe	chr11	46906056	46906419	364	46906234	18	6.63038	3.50359
Nc-1-IP_pe	chr11	46917631	46917911	281	46917674	16	4.25218	2.63809
Nc-1-IP_pe	chr11	46918281	46918544	264	46918364	15	4.06016	2.62146
Nc-1-IP_pe	chr11	46918824	46919161	338	46918859	15	6.07859	3.57636
Nc-1-IP_pe	chr11	46928216	46928732	517	46928633	18	7.73136	4.00629
Nc-1-IP_pe	chr11	46939885	46940210	326	46940075	14	6.69566	4.03025
Nc-1-IP_pe	chr11	46979542	46979868	327	46979765	13	3.67377	2.58159
Nc-1-IP_pe	chr11	47006723	47007089	367	47006951	10	3.83201	2.92147
Nc-1-IP_pe	chr11	47037625	47038789	1165	47038229	30	16.2625	5.92252
Nc-1-IP_pe	chr11	47119022	47119340	319	47119201	15	6.5434	3.81197
Nc-1-IP_pe	chr11	47149037	47149524	488	47149124	15	3.46204	2.35851
Nc-1-IP_pe	chr11	47150547	47150851	305	47150766	15	6.36347	3.72007
Nc-1-IP_pe	chr11	47154296	47154599	304	47154553	11	3.78682	2.80131
Nc-1-IP_pe	chr11	47155945	47156259	315	47156170	14	5.82389	3.56638
Nc-1-IP_pe	chr11	47157659	47157945	287	47157804	13	4.57229	3.02983
Nc-1-IP_pe	chr11	47160794	47161453	660	47161320	20	5.34663	2.81336
Nc-1-IP_pe	chr11	47164129	47164468	340	47164397	12	3.78056	2.71198
Nc-1-IP_pe	chr11	47166062	47166665	604	47166510	15	5.75924	3.41792
Nc-1-IP_pe	chr11	47168324	47168778	455	47168715	14	4.62572	2.96359
Nc-1-IP_pe	chr11	47172725	47173084	360	47173003	14	4.21449	2.76599
Nc-1-IP_pe	chr11	47174251	47174956	706	47174872	17	6.47183	3.53818
Nc-1-IP_pe	chr11	47176414	47177254	841	47176531	26	6.81227	2.94581
Nc-1-IP_pe	chr11	47178034	47178752	719	47178442	20	7.84355	3.81669
Nc-1-IP_pe	chr11	47179163	47179510	348	47179342	19	6.69226	3.43023
Nc-1-IP_pe	chr11	47184759	47185306	548	47184816	18	4.79108	2.72889
Nc-1-IP_pe	chr11	47185894	47186670	777	47186011	24	7.27253	3.22987
Nc-1-IP_pe	chr11	47198910	47199454	545	47199059	16	7.20904	4.01714
Nc-1-IP_pe	chr11	47200451	47200840	390	47200665	22	10.782	4.84972
Nc-1-IP_pe	chr11	47201306	47201571	266	47201334	10	3.27796	2.60465
Nc-1-IP_pe	chr11	47208207	47208474	268	47208397	13	4.78908	3.14145
Nc-1-IP_pe	chr11	47209800	47210183	384	47209954	17	7.21469	3.88822
Nc-1-IP_pe	chr11	47223341	47223629	289	47223559	13	4.02562	2.7544
Nc-1-IP_pe	chr11	47228548	47228863	316	47228705	13	5.70242	3.62667
Nc-1-IP_pe	chr11	47232396	47233482	1087	47233245	16	3.65575	2.38624
Nc-1-IP_pe	chr11	47233824	47234318	495	47234032	12	3.47963	2.55766
Nc-1-IP_pe	chr11	47238368	47238653	286	47238504	12	3.16137	2.39719

Nc-1-IP_pe	chr11	47239427	47239719	293	47239599	17	5.05731	2.91036
Nc-1-IP_pe	chr11	47248543	47249274	732	47248684	19	5.80235	3.06257
Nc-1-IP_pe	chr11	47254219	47254664	446	47254470	13	4.14549	2.81406
Nc-1-IP_pe	chr11	47258117	47258559	443	47258261	16	5.98198	3.41707
Nc-1-IP_pe	chr11	47260432	47260759	328	47260534	17	5.25243	2.99402
Nc-1-IP_pe	chr11	47261596	47261855	260	47261764	17	6.75312	3.66906
Nc-1-IP_pe	chr11	47269878	47270617	740	47270094	17	7.35238	3.95466
Nc-1-IP_pe	chr11	47274640	47275028	389	47274875	18	6.15736	3.29676
Nc-1-IP_pe	chr11	47289012	47289367	356	47289211	16	7.27441	4.05022
Nc-1-IP_pe	chr11	47293433	47294125	693	47293882	12	4.63088	3.16234
Nc-1-IP_pe	chr11	47295580	47295992	413	47295942	10	4.80655	3.49978
Nc-1-IP_pe	chr11	47305663	47306466	804	47306098	16	3.65575	2.38624
Nc-1-IP_pe	chr11	47313869	47314152	284	47314016	12	5.3037	3.53374
Nc-1-IP_pe	chr11	47315509	47316026	518	47315596	10	4.39571	3.25288
Nc-1-IP_pe	chr11	47320355	47320768	414	47320571	16	8.41088	4.64286
Nc-1-IP_pe	chr11	47327813	47328188	376	47328002	15	6.06481	3.56947
Nc-1-IP_pe	chr11	47328575	47328858	284	47328708	14	4.8399	3.06836
Nc-1-IP_pe	chr11	47330516	47330768	253	47330586	16	6.81892	3.82203
Nc-1-IP_pe	chr11	47331187	47331916	730	47331293	15	6.12018	3.5972
Nc-1-IP_pe	chr11	47334625	47334889	265	47334697	16	6.43001	3.63154
Nc-1-IP_pe	chr11	47342693	47343679	987	47343371	16	5.98198	3.41707
Nc-1-IP_pe	chr11	47345005	47345298	294	47345059	14	4.23163	2.77414
Nc-1-IP_pe	chr11	47347492	47347918	427	47347829	16	4.96462	2.94973
Nc-1-IP_pe	chr11	47348326	47348682	357	47348494	17	6.83651	3.70825
Nc-1-IP_pe	chr11	47349661	47350111	451	47349939	18	5.33197	2.94862
Nc-1-IP_pe	chr11	47354976	47355296	321	47355171	14	4.12147	2.72194
Nc-1-IP_pe	chr11	47355982	47356427	446	47356124	23	8.92223	3.93219
Nc-1-IP_pe	chr11	47358826	47359136	311	47359065	18	5.05286	2.83443
Nc-1-IP_pe	chr11	47374833	47376558	1726	47375246	14	4.95172	3.12357
Nc-1-IP_pe	chr11	47377449	47379063	1615	47377616	18	4.6358	2.66698
Nc-1-IP_pe	chr11	47381751	47382036	286	47381824	12	4.87193	3.29389
Nc-1-IP_pe	chr11	47383249	47383716	468	47383413	20	6.94658	3.44067
Nc-1-IP_pe	chr11	47387878	47389229	1352	47388369	15	5.66913	3.37372
Nc-1-IP_pe	chr11	47390519	47391294	776	47390963	14	5.60432	3.45288
Nc-1-IP_pe	chr11	47391756	47392122	367	47391813	14	5.59098	3.44602
Nc-1-IP_pe	chr11	47394268	47396200	1933	47394457	22	9.39426	4.24119
Nc-1-IP_pe	chr11	47398187	47398478	292	47398224	14	4.21449	2.76599
Nc-1-IP_pe	chr11	47399985	47400531	547	47400025	14	5.838	3.57373
Nc-1-IP_pe	chr11	47403196	47403507	312	47403365	14	5.838	3.57373
Nc-1-IP_pe	chr11	47404532	47404996	465	47404538	13	3.35769	2.42919
Nc-1-IP_pe	chr11	47408204	47408512	309	47408346	16	5.82808	3.34463
Nc-1-IP_pe	chr11	47410061	47410395	335	47410266	19	6.32622	3.27682
Nc-1-IP_pe	chr11	47412109	47412543	435	47412229	20	7.43645	3.64378
Nc-1-IP_pe	chr11	47413203	47413927	725	47413735	16	4.59224	2.7853
Nc-1-IP_pe	chr11	47415951	47416210	260	47416159	18	5.72638	3.11298

Nc-1-IP_pe	chr11	47418075	47418689	615	47418254	13	4.03953	2.7613
Nc-1-IP_pe	chr11	47419116	47419424	309	47419331	15	5.15085	3.12383
Nc-1-IP_pe	chr11	47420319	47420848	530	47420660	15	5.16195	3.12911
Nc-1-IP_pe	chr11	47422736	47423021	286	47422885	16	2.92499	2.08721
Nc-1-IP_pe	chr11	47425712	47426298	587	47426256	22	5.39765	2.71064
Nc-1-IP_pe	chr11	47433332	47433639	308	47433474	15	6.19428	3.63445
Nc-1-IP_pe	chr11	47434811	47435103	293	47435034	11	4.06403	2.95415
Nc-1-IP_pe	chr11	47436779	47437463	685	47436967	13	5.27845	3.39845
Nc-1-IP_pe	chr11	47437773	47438600	828	47437951	17	7.6273	4.08876
Nc-1-IP_pe	chr11	47438877	47439227	351	47439188	14	5.03085	3.16286
Nc-1-IP_pe	chr11	47447009	47447281	273	47447081	14	6.11547	3.71921
Nc-1-IP_pe	chr11	47449148	47449648	501	47449507	16	4.96462	2.94973
Nc-1-IP_pe	chr11	47455611	47455866	256	47455833	12	4.28158	2.97474
Nc-1-IP_pe	chr11	47462776	47463249	474	47462871	13	5.57038	3.55505
Nc-1-IP_pe	chr11	47466786	47467144	359	47467128	16	3.65575	2.38624
Nc-1-IP_pe	chr11	47480062	47480389	328	47480318	11	4.88957	3.42271
Nc-1-IP_pe	chr11	47490460	47491392	933	47491094	18	9.1091	4.6774
Nc-1-IP_pe	chr11	47502487	47502933	447	47502744	14	4.60011	2.95114
Nc-1-IP_pe	chr11	47511926	47512222	297	47512047	12	4.70082	3.20033
Nc-1-IP_pe	chr11	47520451	47520946	496	47520696	14	5.51541	3.4073
Nc-1-IP_pe	chr11	47530696	47531146	451	47530781	13	5.15976	3.33548
Nc-1-IP_pe	chr11	47540585	47540851	267	47540699	14	4.8399	3.06836
Nc-1-IP_pe	chr11	47545041	47545300	260	47545125	13	6.07713	3.83258
Nc-1-IP_pe	chr11	47552719	47553182	464	47552986	17	4.78065	2.79327
Nc-1-IP_pe	chr11	47557000	47557413	414	47557184	16	7.79347	4.31685
Nc-1-IP_pe	chr11	47561024	47561392	369	47561333	12	4.75569	3.23024
Nc-1-IP_pe	chr11	47564049	47564462	414	47564236	13	4.92076	3.20992
Nc-1-IP_pe	chr11	47565344	47566179	836	47565460	19	5.51632	2.94814
Nc-1-IP_pe	chr11	47572909	47573238	330	47573132	16	6.33285	3.58457
Nc-1-IP_pe	chr11	47574594	47575052	459	47574657	11	4.06403	2.95415
Nc-1-IP_pe	chr11	47578444	47579445	1002	47579039	48	29.0383	7.60391
Nc-1-IP_pe	chr11	47584262	47584537	276	47584417	14	5.39586	3.34637
Nc-1-IP_pe	chr11	47587768	47588179	412	47588172	12	3.86684	2.7567
Nc-1-IP_pe	chr11	47589297	47589903	607	47589704	17	5.54967	3.12324
Nc-1-IP_pe	chr11	47595227	47595601	375	47595408	13	3.07238	2.29378
Nc-1-IP_pe	chr11	47597757	47598061	305	47597999	13	4.83642	3.16601
Nc-1-IP_pe	chr11	47608475	47608766	292	47608603	15	6.19428	3.63445
Nc-1-IP_pe	chr11	47634860	47635224	365	47634968	13	5.36853	3.44651
Nc-1-IP_pe	chr11	47658094	47658539	446	47658365	14	4.60011	2.95114
Nc-1-IP_pe	chr11	47662291	47662708	418	47662552	13	6.7109	4.18935
Nc-1-IP_pe	chr11	47663595	47664208	614	47664094	14	7.34079	4.38651
Nc-1-IP_pe	chr11	47675618	47675934	317	47675783	15	6.78905	3.93887
Nc-1-IP_pe	chr11	47695390	47695646	257	47695533	10	4.20113	3.13749
Nc-1-IP_pe	chr11	47697236	47698121	886	47697321	14	3.86727	2.6027
Nc-1-IP_pe	chr11	47703949	47704382	434	47704204	12	5.77527	3.80164

Nc-1-IP_pe	chr11	47732191	47733134	944	47732901	16	9.11744	5.02718
Nc-1-IP_pe	chr11	47737576	47738074	499	47737685	14	7.28181	4.3535
Nc-1-IP_pe	chr11	47740089	47740377	289	47740280	11	4.06403	2.95415
Nc-1-IP_pe	chr11	47740668	47740968	301	47740914	13	5.86991	3.71823
Nc-1-IP_pe	chr11	47752329	47752801	473	47752339	10	4.13634	3.09929
Nc-1-IP_pe	chr11	47757310	47757663	354	47757521	13	3.67377	2.58159
Nc-1-IP_pe	chr11	47795856	47796116	261	47795903	11	4.94832	3.45679
Nc-1-IP_pe	chr11	47799179	47799443	265	47799351	13	6.86537	4.27785
Nc-1-IP_pe	chr11	47799782	47800207	426	47800044	16	8.26996	4.56762
Nc-1-IP_pe	chr11	47805595	47805870	276	47805692	12	4.28158	2.97474
Nc-1-IP_pe	chr11	47808790	47809079	290	47808866	14	6.69566	4.03025
Nc-1-IP_pe	chr11	47824918	47825439	522	47825151	12	4.70082	3.20033
Nc-1-IP_pe	chr11	47830850	47831146	297	47830949	14	6.44441	3.89444
Nc-1-IP_pe	chr11	47834747	47835054	308	47834930	15	8.03923	4.60906
Nc-1-IP_pe	chr11	47838411	47838664	254	47838560	12	4.88508	3.30112
Nc-1-IP_pe	chr11	47841698	47841983	286	47841890	11	4.24272	3.05389
Nc-1-IP_pe	chr11	47842590	47842943	354	47842872	12	3.96559	2.80816
Nc-1-IP_pe	chr11	47845183	47845435	253	47845258	14	5.61771	3.45976
Nc-1-IP_pe	chr11	47847940	47848545	606	47848196	30	13.9338	5.01228
Nc-1-IP_pe	chr11	47858615	47858883	269	47858833	18	4.96968	2.80074
Nc-1-IP_pe	chr11	47868868	47869134	267	47869000	13	6.8068	4.24423
Nc-1-IP_pe	chr11	47871091	47871435	345	47871342	10	3.89064	2.95552
Nc-1-IP_pe	chr11	47874997	47875454	458	47875165	11	3.28475	2.53029
Nc-1-IP_pe	chr11	47882967	47883395	429	47883348	10	4.39571	3.25288
Nc-1-IP_pe	chr11	47892676	47892982	307	47892860	16	5.73763	3.30235
Nc-1-IP_pe	chr11	47910045	47910981	937	47910768	18	7.1522	3.73811
Nc-1-IP_pe	chr11	47912593	47912860	268	47912786	11	4.49162	3.19439
Nc-1-IP_pe	chr11	47922790	47923044	255	47922987	13	5.70242	3.62667
Nc-1-IP_pe	chr11	47925455	47925759	305	47925595	14	7.05305	4.22631
Nc-1-IP_pe	chr11	47931025	47931432	408	47931332	9	3.72192	2.95716
Nc-1-IP_pe	chr11	47933664	47934201	538	47933795	13	6.02837	3.80557
Nc-1-IP_pe	chr11	47951154	47951494	341	47951272	17	5.9911	3.31919
Nc-1-IP_pe	chr11	47958904	47959302	399	47959046	10	3.40382	2.67582
Nc-1-IP_pe	chr11	47961371	47961698	328	47961587	19	8.4638	4.21715
Nc-1-IP_pe	chr11	47975342	47975705	364	47975670	11	3.64839	2.72584
Nc-1-IP_pe	chr11	47979097	47979501	405	47979210	14	5.838	3.57373
Nc-1-IP_pe	chr11	47984893	47985152	260	47985058	12	3.83688	2.74114
Nc-1-IP_pe	chr11	47993068	47993536	469	47993467	12	4.58219	3.13597
Nc-1-IP_pe	chr11	48005884	48006214	331	48006199	12	2.87625	2.25568
Nc-1-IP_pe	chr11	48010009	48010614	606	48010236	12	4.83276	3.2724
Nc-1-IP_pe	chr11	48018363	48018967	605	48018775	17	8.46937	4.51138
Nc-1-IP_pe	chr11	48032090	48032617	528	48032114	11	4.10219	2.97537
Nc-1-IP_pe	chr11	48035062	48035430	369	48035298	14	3.86727	2.6027
Nc-1-IP_pe	chr11	48043728	48044070	343	48044024	13	4.45253	2.96876
Nc-1-IP_pe	chr11	48060728	48061399	672	48060850	16	6.8987	3.86161

Nc-1-IP_pe	chr11	48075474	48075760	287	48075706	14	5.06689	3.18081
Nc-1-IP_pe	chr11	48084271	48084679	409	48084473	13	5.23848	3.3772
Nc-1-IP_pe	chr11	48086326	48086604	279	48086413	15	4.40338	2.77622
Nc-1-IP_pe	chr11	48090152	48090790	639	48090542	13	5.86991	3.71823
Nc-1-IP_pe	chr11	48091941	48092211	271	48091973	9	3.18064	2.63172
Nc-1-IP_pe	chr11	48124887	48125163	277	48125039	17	8.74292	4.65245
Nc-1-IP_pe	chr11	48125799	48126129	331	48125999	16	7.53857	4.18505
Nc-1-IP_pe	chr11	48127016	48127323	308	48127207	10	3.94999	2.99009
Nc-1-IP_pe	chr11	48139428	48139801	374	48139575	12	4.70082	3.20033
Nc-1-IP_pe	chr11	48140230	48140654	425	48140276	12	4.24064	2.95299
Nc-1-IP_pe	chr11	48153156	48153421	266	48153233	12	5.94619	3.9002
Nc-1-IP_pe	chr11	48172496	48173510	1015	48172708	17	6.47874	3.54137
Nc-1-IP_pe	chr11	48175522	48175950	429	48175720	11	5.77463	3.94557
Nc-1-IP_pe	chr11	48176327	48176941	615	48176600	10	4.99888	3.61677
Nc-1-IP_pe	chr11	48179276	48179705	430	48179466	14	8.34955	4.96348
Nc-1-IP_pe	chr11	48191080	48191370	291	48191205	10	3.89064	2.95552
Nc-1-IP_pe	chr11	48228660	48229054	395	48228825	20	11.7013	5.64235
Nc-1-IP_pe	chr11	48236033	48236702	670	48236271	12	5.23082	3.49289
Nc-1-IP_pe	chr11	48264179	48264490	312	48264280	11	5.10577	3.5486
Nc-1-IP_pe	chr11	48292583	48293471	889	48292743	14	7.42849	4.43574
Nc-1-IP_pe	chr11	48305358	48305655	298	48305459	16	10.117	5.58955
Nc-1-IP_pe	chr11	48345682	48346841	1160	48346373	12	5.23082	3.49289
Nc-1-IP_pe	chr11	48356182	48356550	369	48356206	12	3.47963	2.55766
Nc-1-IP_pe	chr11	48378016	48378605	590	48378435	12	5.84943	3.84431
Nc-1-IP_pe	chr11	48415451	48415976	526	48415774	11	4.74697	3.34038
Nc-1-IP_pe	chr11	48453309	48453861	553	48453418	10	4.39571	3.25288
Nc-1-IP_pe	chr11	48595678	48595966	289	48595905	8	3.08755	2.66145
Nc-1-IP_pe	chr11	48729361	48729875	515	48729782	14	7.54286	4.50023
Nc-1-IP_pe	chr11	48882421	48882688	268	48882471	13	4.45253	2.96876
Nc-1-IP_pe	chr11	48883446	48883770	325	48883532	10	3.71867	2.85592
Nc-1-IP_pe	chr11	48884765	48885573	809	48885033	16	6.95583	3.89005
Nc-1-IP_pe	chr11	48885992	48886694	703	48886193	14	6.44441	3.89444
Nc-1-IP_pe	chr11	48894116	48894424	309	48894330	17	7.6273	4.08876
Nc-1-IP_pe	chr11	48894950	48895349	400	48895008	11	4.91882	3.43967
Nc-1-IP_pe	chr11	48898185	48898684	500	48898483	17	8.58498	4.57078
Nc-1-IP_pe	chr11	48899940	48901228	1289	48900520	16	6.65665	3.74206
Nc-1-IP_pe	chr11	48901778	48902154	377	48902011	13	4.86362	3.18015
Nc-1-IP_pe	chr11	48955781	48956055	275	48955911	18	8.27052	4.2634
Nc-1-IP_pe	chr11	48963813	48964427	615	48963934	9	4.25966	3.28797
Nc-1-IP_pe	chr11	48964967	48965335	369	48965142	11	6.03439	4.10251
Nc-1-IP_pe	chr11	48968525	48968800	276	48968634	12	5.23082	3.49289
Nc-1-IP_pe	chr11	49045796	49046548	753	49045891	13	5.86991	3.71823
Nc-1-IP_pe	chr11	49049238	49049807	570	49049355	11	4.74697	3.34038
Nc-1-IP_pe	chr11	49061607	49062169	563	49061767	9	4.46937	3.41876
Nc-1-IP_pe	chr11	49102381	49102680	300	49102543	16	7.61454	4.22416

Nc-1-IP_pe	chr11	49160514	49160774	261	49160613	10	4.99888	3.61677
Nc-1-IP_pe	chr11	49273863	49274141	279	49273878	11	3.64839	2.72584
Nc-1-IP_pe	chr11	49298433	49298698	266	49298457	11	4.06403	2.95415
Nc-1-IP_pe	chr11	49434359	49434654	296	49434465	12	6.58417	4.27436
Nc-1-IP_pe	chr11	49498509	49498836	328	49498788	9	4.46937	3.41876
Nc-1-IP_pe	chr11	49560895	49561763	869	49561092	13	7.12853	4.42991
Nc-1-IP_pe	chr11	49780494	49780765	272	49780643	13	7.7352	4.78626
Nc-1-IP_pe	chr11	49817804	49818327	524	49817986	21	10.7603	4.99737
Nc-1-IP_pe	chr11	49824434	49824819	386	49824552	10	4.93609	3.57849
Nc-1-IP_pe	chr11	49851759	49852420	662	49852039	18	9.94172	5.10499
Nc-1-IP_pe	chr11	49854035	49854378	344	49854317	14	3.55308	2.45762
Nc-1-IP_pe	chr11	49886430	49887270	841	49886594	9	4.46937	3.41876
Nc-1-IP_pe	chr11	49888157	49889657	1501	49889478	12	6.58417	4.27436
Nc-1-IP_pe	chr11	49893000	49893804	805	49893582	14	8.44792	5.0209
Nc-1-IP_pe	chr11	49911182	49911708	527	49911665	13	3.67377	2.58159
Nc-1-IP_pe	chr11	49941678	49942904	1227	49942740	13	7.34205	4.55444
Nc-1-IP_pe	chr11	50005072	50005738	667	50005171	11	3.64839	2.72584
Nc-1-IP_pe	chr11	50027120	50027431	312	50027408	10	3.4604	2.70797
Nc-1-IP_pe	chr11	50029229	50029608	380	50029459	11	5.77463	3.94557
Nc-1-IP_pe	chr11	50035287	50035540	254	50035486	8	3.56021	2.95918
Nc-1-IP_pe	chr11	50061564	50061859	296	50061770	8	3.74505	3.07688
Nc-1-IP_pe	chr11	50063221	50063751	531	50063703	14	5.51541	3.4073
Nc-1-IP_pe	chr11	50071484	50073012	1529	50072857	17	7.6273	4.08876
Nc-1-IP_pe	chr11	50075136	50075674	539	50075262	13	4.04902	2.76601
Nc-1-IP_pe	chr11	50077104	50077707	604	50077157	12	4.88508	3.30112
Nc-1-IP_pe	chr11	50078264	50078522	259	50078471	12	4.1728	2.91706
Nc-1-IP_pe	chr11	50082274	50082748	475	50082407	10	4.90322	3.55848
Nc-1-IP_pe	chr11	50142696	50143044	349	50142825	14	7.9745	4.74633
Nc-1-IP_pe	chr11	50148805	50149597	793	50149369	15	6.78905	3.93887
Nc-1-IP_pe	chr11	50199492	50199873	382	50199756	16	9.74731	5.37917
Nc-1-IP_pe	chr11	50232827	50233098	272	50232918	14	8.29543	4.93196
Nc-1-IP_pe	chr11	50242363	50242663	301	50242389	8	3.63049	3.00385
Nc-1-IP_pe	chr11	50245855	50246396	542	50245956	11	5.03835	3.5092
Nc-1-IP_pe	chr11	50247038	50247407	370	50247236	15	6.39297	3.73508
Nc-1-IP_pe	chr11	50298185	50298655	471	50298387	10	3.08907	2.49869
Nc-1-IP_pe	chr11	50301298	50301769	472	50301493	13	6.36468	3.99316
Nc-1-IP_pe	chr11	50306674	50307221	548	50306826	11	5.63263	3.8604
Nc-1-IP_pe	chr11	50329362	50330477	1116	50330374	14	3.86727	2.6027
Nc-1-IP_pe	chr11	50335179	50335775	597	50335632	10	4.39571	3.25288
Nc-1-IP_pe	chr11	50695113	50695368	256	50695228	13	6.36468	3.99316
Nc-1-IP_pe	chr11	54624455	54624942	488	54624649	12	5.23082	3.49289
Nc-1-IP_pe	chr11	54626619	54626922	304	54626715	11	5.72344	3.91481
Nc-1-IP_pe	chr11	54641553	54642050	498	54641876	13	6.02837	3.80557
Nc-1-IP_pe	chr11	54714179	54714439	261	54714306	12	4.70082	3.20033
Nc-1-IP_pe	chr11	54832533	54832999	467	54832852	14	6.28469	3.80898

Nc-1-IP_pe	chr11	54838066	54838688	623	54838243	10	4.62142	3.388
Nc-1-IP_pe	chr11	54850218	54850823	606	54850684	12	6.86906	4.44438
Nc-1-IP_pe	chr11	54930904	54931177	274	54931067	24	9.77496	4.15836
Nc-1-IP_pe	chr11	55084447	55085181	735	55084718	16	9.11744	5.02718
Nc-1-IP_pe	chr11	55173166	55173527	362	55173307	12	5.77527	3.80164
Nc-1-IP_pe	chr11	55279392	55279986	595	55279887	11	3.28475	2.53029
Nc-1-IP_pe	chr11	55282718	55283234	517	55283144	12	5.98129	3.92053
Nc-1-IP_pe	chr11	55334652	55335367	716	55334851	12	6.86906	4.44438
Nc-1-IP_pe	chr11	55356042	55356358	317	55356177	15	7.95672	4.56362
Nc-1-IP_pe	chr11	55371189	55371651	463	55371578	10	4.39571	3.25288
Nc-1-IP_pe	chr11	55460073	55460498	426	55460404	21	6.66991	3.24296
Nc-1-IP_pe	chr11	55665497	55665939	443	55665799	12	6.86906	4.44438
Nc-1-IP_pe	chr11	55747500	55747809	310	55747586	14	5.43414	3.36584
Nc-1-IP_pe	chr11	55811813	55812321	509	55812229	9	4.46937	3.41876
Nc-1-IP_pe	chr11	55827306	55827632	327	55827376	9	4.46937	3.41876
Nc-1-IP_pe	chr11	55857269	55857540	272	55857330	12	5.84943	3.84431
Nc-1-IP_pe	chr11	55873310	55873632	323	55873457	13	4.56123	3.02417
Nc-1-IP_pe	chr11	55970775	55971115	341	55970893	13	6.62436	4.14003
Nc-1-IP_pe	chr11	56032941	56033215	275	56033088	10	5.23357	3.76063
Nc-1-IP_pe	chr11	56033499	56033979	481	56033907	8	3.08755	2.66145
Nc-1-IP_pe	chr11	56143788	56144055	268	56143890	14	6.06519	3.69269
Nc-1-IP_pe	chr11	56152318	56152609	292	56152461	12	6.58417	4.27436
Nc-1-IP_pe	chr11	56173216	56173645	430	56173531	9	4.46937	3.41876
Nc-1-IP_pe	chr11	56219864	56220222	359	56220073	15	9.07119	5.19048
Nc-1-IP_pe	chr11	56259564	56260061	498	56259926	11	6.03439	4.10251
Nc-1-IP_pe	chr11	56277326	56277888	563	56277449	13	7.42513	4.60316
Nc-1-IP_pe	chr11	56332094	56332505	412	56332203	12	3.83688	2.74114
Nc-1-IP_pe	chr11	56337036	56337463	428	56337399	12	6.58417	4.27436
Nc-1-IP_pe	chr11	56362967	56363305	339	56363214	13	7.56612	4.68618
Nc-1-IP_pe	chr11	56401809	56402528	720	56402309	14	8.63073	5.12814
Nc-1-IP_pe	chr11	56409800	56410191	392	56409980	7	3.06442	2.73501
Nc-1-IP_pe	chr11	56496492	56497553	1062	56497235	18	6.63038	3.50359
Nc-1-IP_pe	chr11	56554516	56554894	379	56554664	13	5.36853	3.44651
Nc-1-IP_pe	chr11	56569675	56570119	445	56569745	9	3.79246	3.00015
Nc-1-IP_pe	chr11	56640566	56640833	268	56640755	12	6.86906	4.44438
Nc-1-IP_pe	chr11	56690329	56690663	335	56690423	10	5.23357	3.76063
Nc-1-IP_pe	chr11	56705395	56705709	315	56705537	15	5.58327	3.33181
Nc-1-IP_pe	chr11	56845174	56845426	253	56845307	17	5.14803	2.94914
Nc-1-IP_pe	chr11	56873784	56874073	290	56873963	14	4.60011	2.95114
Nc-1-IP_pe	chr11	56945460	56946053	594	56945973	12	3.86684	2.7567
Nc-1-IP_pe	chr11	56968377	56968646	270	56968621	12	4.39405	3.03475
Nc-1-IP_pe	chr11	57006097	57006634	538	57006115	15	3.74763	2.48304
Nc-1-IP_pe	chr11	57022345	57022851	507	57022574	12	5.23082	3.49289
Nc-1-IP_pe	chr11	57062800	57063279	480	57063192	14	4.60011	2.95114
Nc-1-IP_pe	chr11	57091963	57092370	408	57092091	16	7.61454	4.22416

Nc-1-IP_pe	chr11	57106270	57106735	466	57106428	12	5.99895	3.93077
Nc-1-IP_pe	chr11	57148382	57148677	296	57148414	13	5.15976	3.33548
Nc-1-IP_pe	chr11	57169612	57169969	358	57169915	12	4.63088	3.16234
Nc-1-IP_pe	chr11	57180017	57180467	451	57180264	14	6.28469	3.80898
Nc-1-IP_pe	chr11	57181589	57182033	445	57181608	13	4.24481	2.8638
Nc-1-IP_pe	chr11	57182344	57182894	551	57182444	14	6.06519	3.69269
Nc-1-IP_pe	chr11	57184509	57184938	430	57184553	13	4.41954	2.952
Nc-1-IP_pe	chr11	57188104	57188390	287	57188276	14	6.44441	3.89444
Nc-1-IP_pe	chr11	57207243	57207524	282	57207376	14	6.69566	4.03025
Nc-1-IP_pe	chr11	57210803	57211155	353	57210863	10	3.60969	2.79325
Nc-1-IP_pe	chr11	57213293	57213707	415	57213603	16	7.61454	4.22416
Nc-1-IP_pe	chr11	57219541	57219867	327	57219745	11	4.23076	3.04719
Nc-1-IP_pe	chr11	57227761	57228048	288	57227954	11	5.10577	3.5486
Nc-1-IP_pe	chr11	57236992	57237689	698	57237155	17	7.02067	3.79543
Nc-1-IP_pe	chr11	57240720	57241059	340	57240880	18	8.80785	4.52672
Nc-1-IP_pe	chr11	57297529	57297953	425	57297891	14	4.60011	2.95114
Nc-1-IP_pe	chr11	57302354	57302745	392	57302538	21	6.66991	3.24296
Nc-1-IP_pe	chr11	57305646	57306201	556	57305830	13	4.08727	2.78503
Nc-1-IP_pe	chr11	57308514	57309360	847	57308954	19	5.51632	2.94814
Nc-1-IP_pe	chr11	57309684	57310542	859	57310356	22	8.81204	3.99809
Nc-1-IP_pe	chr11	57311629	57311893	265	57311685	17	5.56266	3.12894
Nc-1-IP_pe	chr11	57320052	57320620	569	57320523	19	6.32622	3.27682
Nc-1-IP_pe	chr11	57325283	57325574	292	57325378	21	10.3334	4.79704
Nc-1-IP_pe	chr11	57326376	57326730	355	57326441	12	4.10922	2.88351
Nc-1-IP_pe	chr11	57328232	57328492	261	57328284	13	4.20473	2.84369
Nc-1-IP_pe	chr11	57330230	57330552	323	57330325	22	8.79433	3.99081
Nc-1-IP_pe	chr11	57335223	57335865	643	57335415	27	11.7969	4.58755
Nc-1-IP_pe	chr11	57341650	57341925	276	57341841	13	4.41954	2.952
Nc-1-IP_pe	chr11	57346620	57347022	403	57346967	16	5.50606	3.19508
Nc-1-IP_pe	chr11	57347296	57347958	663	57347390	16	4.59224	2.7853
Nc-1-IP_pe	chr11	57348787	57349153	367	57348899	17	5.9911	3.31919
Nc-1-IP_pe	chr11	57349653	57350077	425	57349978	17	5.54967	3.12324
Nc-1-IP_pe	chr11	57350340	57351001	662	57350614	18	8.37884	4.31591
Nc-1-IP_pe	chr11	57358932	57359372	441	57359274	16	4.25252	2.63824
Nc-1-IP_pe	chr11	57369470	57369902	433	57369667	13	5.25176	3.38425
Nc-1-IP_pe	chr11	57371461	57371809	349	57371645	19	9.15219	4.54307
Nc-1-IP_pe	chr11	57379525	57380082	558	57380000	15	6.39297	3.73508
Nc-1-IP_pe	chr11	57381613	57381996	384	57381623	12	3.16137	2.39719
Nc-1-IP_pe	chr11	57387497	57387932	436	57387667	11	3.64839	2.72584
Nc-1-IP_pe	chr11	57405582	57406043	462	57405739	14	5.40858	3.35284
Nc-1-IP_pe	chr11	57409168	57409504	337	57409466	17	5.89608	3.2766
Nc-1-IP_pe	chr11	57419591	57419844	254	57419700	16	5.18047	3.04664
Nc-1-IP_pe	chr11	57435739	57436165	427	57435895	13	4.47401	2.97968
Nc-1-IP_pe	chr11	57437965	57438583	619	57438572	12	2.87625	2.25568
Nc-1-IP_pe	chr11	57448613	57449067	455	57448839	15	6.19428	3.63445

Nc-1-IP_pe	chr11	57454720	57455251	532	57454900	17	7.18458	3.87376
Nc-1-IP_pe	chr11	57460449	57461279	831	57460875	25	5.66296	2.64066
Nc-1-IP_pe	chr11	57463415	57463680	266	57463497	16	5.34015	3.1191
Nc-1-IP_pe	chr11	57464921	57465587	667	57465099	17	6.76692	3.67553
Nc-1-IP_pe	chr11	57467806	57468682	877	57467902	20	3.6727	2.20918
Nc-1-IP_pe	chr11	57471015	57471410	396	57471383	14	3.86727	2.6027
Nc-1-IP_pe	chr11	57471960	57472227	268	57472195	15	4.06016	2.62146
Nc-1-IP_pe	chr11	57474421	57475011	591	57474943	20	8.91114	4.28812
Nc-1-IP_pe	chr11	57476013	57477286	1274	57476171	22	7.27519	3.39036
Nc-1-IP_pe	chr11	57477635	57477975	341	57477970	14	3.26761	2.32785
Nc-1-IP_pe	chr11	57479331	57479640	310	57479515	15	4.06016	2.62146
Nc-1-IP_pe	chr11	57481947	57483331	1385	57482995	18	7.73136	4.00629
Nc-1-IP_pe	chr11	57487039	57487388	350	57487183	18	8.10208	4.1823
Nc-1-IP_pe	chr11	57493460	57494322	863	57493743	18	5.94685	3.20644
Nc-1-IP_pe	chr11	57503130	57503461	332	57503335	15	5.81087	3.44334
Nc-1-IP_pe	chr11	57506130	57506572	443	57506427	10	3.7298	2.86234
Nc-1-IP_pe	chr11	57514228	57514513	286	57514397	16	4.50821	2.74867
Nc-1-IP_pe	chr11	57514937	57515341	405	57515184	18	5.23669	2.90944
Nc-1-IP_pe	chr11	57515799	57516064	266	57515851	18	3.77591	2.33276
Nc-1-IP_pe	chr11	57528768	57529028	261	57528842	13	6.34721	3.98335
Nc-1-IP_pe	chr11	57533017	57533524	508	57533381	12	6.10662	3.99337
Nc-1-IP_pe	chr11	57534529	57534925	397	57534776	10	4.39023	3.24962
Nc-1-IP_pe	chr11	57547440	57547909	470	57547663	13	5.55598	3.54727
Nc-1-IP_pe	chr11	57562199	57562553	355	57562440	15	6.24734	3.66122
Nc-1-IP_pe	chr11	57567337	57567755	419	57567619	15	6.24734	3.66122
Nc-1-IP_pe	chr11	57568059	57568704	646	57568132	18	5.72638	3.11298
Nc-1-IP_pe	chr11	57582293	57582659	367	57582522	7	2.9043	2.63038
Nc-1-IP_pe	chr11	57587215	57587505	291	57587391	12	5.23082	3.49289
Nc-1-IP_pe	chr11	57597917	57598952	1036	57598705	12	6.58417	4.27436
Nc-1-IP_pe	chr11	57600071	57600372	302	57600278	10	5.14046	3.70342
Nc-1-IP_pe	chr11	57603716	57604004	289	57603787	10	4.39571	3.25288
Nc-1-IP_pe	chr11	57609128	57609446	319	57609288	12	6.58898	4.27722
Nc-1-IP_pe	chr11	57614402	57614754	353	57614487	11	5.19376	3.60018
Nc-1-IP_pe	chr11	57648102	57648895	794	57648704	15	8.26005	4.73146
Nc-1-IP_pe	chr11	57652247	57652586	340	57652393	8	3.08755	2.66145
Nc-1-IP_pe	chr11	57659655	57659983	329	57659863	10	3.08907	2.49869
Nc-1-IP_pe	chr11	57668003	57668651	649	57668425	19	8.19109	4.09114
Nc-1-IP_pe	chr11	57686981	57687570	590	57687037	8	3.74505	3.07688
Nc-1-IP_pe	chr11	57696461	57696919	459	57696566	14	6.06519	3.69269
Nc-1-IP_pe	chr11	57698647	57699344	698	57699013	16	7.53857	4.18505
Nc-1-IP_pe	chr11	57699814	57700204	391	57699945	12	3.83688	2.74114
Nc-1-IP_pe	chr11	57716841	57717531	691	57717445	13	5.9487	3.76157
Nc-1-IP_pe	chr11	57719896	57720151	256	57720015	10	5.23357	3.76063
Nc-1-IP_pe	chr11	57743947	57744217	271	57744133	13	6.02837	3.80557
Nc-1-IP_pe	chr11	57747699	57748069	371	57748048	9	3.77975	2.99239

Nc-1-IP_pe	chr11	57752275	57752575	301	57752456	10	3.71867	2.85592
Nc-1-IP_pe	chr11	57789830	57790267	438	57790121	7	3.06442	2.73501
Nc-1-IP_pe	chr11	57791193	57791663	471	57791404	14	8.63073	5.12814
Nc-1-IP_pe	chr11	57839992	57840256	265	57840015	7	3.06442	2.73501
Nc-1-IP_pe	chr11	57866480	57866774	295	57866638	10	4.93609	3.57849
Nc-1-IP_pe	chr11	57873451	57873813	363	57873599	10	4.77495	3.48064
Nc-1-IP_pe	chr11	57876580	57876974	395	57876666	10	5.23357	3.76063
Nc-1-IP_pe	chr11	57886959	57887229	271	57887071	11	4.06403	2.95415
Nc-1-IP_pe	chr11	57894277	57894685	409	57894477	10	5.23357	3.76063
Nc-1-IP_pe	chr11	57943697	57944324	628	57944090	16	5.82808	3.34463
Nc-1-IP_pe	chr11	57952890	57953460	571	57953354	11	5.10577	3.5486
Nc-1-IP_pe	chr11	57953733	57954133	401	57953789	9	4.46937	3.41876
Nc-1-IP_pe	chr11	57992091	57992439	349	57992156	10	4.93609	3.57849
Nc-1-IP_pe	chr11	57999303	58000305	1003	57999470	15	6.19428	3.63445
Nc-1-IP_pe	chr11	58006861	58007234	374	58007007	10	5.23357	3.76063
Nc-1-IP_pe	chr11	58040734	58041414	681	58041391	11	5.10577	3.5486
Nc-1-IP_pe	chr11	58065946	58067329	1384	58067011	11	5.77463	3.94557
Nc-1-IP_pe	chr11	58228136	58228900	765	58228324	14	5.51541	3.4073
Nc-1-IP_pe	chr11	58267032	58267623	592	58267388	11	4.06403	2.95415
Nc-1-IP_pe	chr11	58390661	58391089	429	58390688	13	3.67377	2.58159
Nc-1-IP_pe	chr11	58444643	58444970	328	58444862	11	6.03439	4.10251
Nc-1-IP_pe	chr11	58529787	58530519	733	58529973	12	6.86906	4.44438
Nc-1-IP_pe	chr11	58579412	58579809	398	58579604	16	4.25252	2.63824
Nc-1-IP_pe	chr11	58608633	58608903	271	58608803	13	7.7352	4.78626
Nc-1-IP_pe	chr11	58645716	58646017	302	58645748	9	3.27326	2.68684
Nc-1-IP_pe	chr11	58703253	58703651	399	58703465	13	7.7352	4.78626
Nc-1-IP_pe	chr11	58706350	58706780	431	58706525	10	4.47495	3.30017
Nc-1-IP_pe	chr11	58780522	58780810	289	58780615	10	4.62142	3.388
Nc-1-IP_pe	chr11	58815975	58816257	283	58816100	13	7.7352	4.78626
Nc-1-IP_pe	chr11	58905114	58905407	294	58905131	8	3.08755	2.66145
Nc-1-IP_pe	chr11	58933720	58934292	573	58933868	12	6.58417	4.27436
Nc-1-IP_pe	chr11	58934644	58935118	475	58934797	11	3.64839	2.72584
Nc-1-IP_pe	chr11	58972509	58972894	386	58972629	10	4.99888	3.61677
Nc-1-IP_pe	chr11	59030043	59030306	264	59030165	11	5.77463	3.94557
Nc-1-IP_pe	chr11	59050593	59050864	272	59050703	14	8.29543	4.93196
Nc-1-IP_pe	chr11	59062819	59063181	363	59062978	9	4.46937	3.41876
Nc-1-IP_pe	chr11	59076130	59076783	654	59076734	11	4.06403	2.95415
Nc-1-IP_pe	chr11	59103276	59103696	421	59103482	14	8.29543	4.93196
Nc-1-IP_pe	chr11	59131807	59132090	284	59131809	9	3.72192	2.95716
Nc-1-IP_pe	chr11	59135832	59136096	265	59135919	11	4.74697	3.34038
Nc-1-IP_pe	chr11	59142545	59142804	260	59142643	18	6.40661	3.40507
Nc-1-IP_pe	chr11	59180110	59180591	482	59180269	12	4.70082	3.20033
Nc-1-IP_pe	chr11	59182207	59182599	393	59182244	11	3.58722	2.69267
Nc-1-IP_pe	chr11	59184928	59185353	426	59185212	13	6.36468	3.99316
Nc-1-IP_pe	chr11	59193794	59194178	385	59193978	15	8.27326	4.73882

Nc-1-IP_pe	chr11	59201325	59201651	327	59201403	9	3.72192	2.95716
Nc-1-IP_pe	chr11	59206586	59206937	352	59206771	13	7.12853	4.42991
Nc-1-IP_pe	chr11	59233686	59233960	275	59233777	12	4.24064	2.95299
Nc-1-IP_pe	chr11	59260815	59261491	677	59261426	9	4.04582	3.15559
Nc-1-IP_pe	chr11	59266990	59267324	335	59267162	13	6.65435	4.1571
Nc-1-IP_pe	chr11	59271673	59272229	557	59271774	12	6.48784	4.21727
Nc-1-IP_pe	chr11	59292461	59292739	279	59292665	11	5.03835	3.5092
Nc-1-IP_pe	chr11	59355044	59355503	460	59355191	11	4.47871	3.18705
Nc-1-IP_pe	chr11	59367222	59367686	465	59367374	12	6.86906	4.44438
Nc-1-IP_pe	chr11	59387260	59387524	265	59387370	15	9.55381	5.47001
Nc-1-IP_pe	chr11	59443319	59443771	453	59443710	11	4.54404	3.2242
Nc-1-IP_pe	chr11	59504015	59504595	581	59504332	12	5.77527	3.80164
Nc-1-IP_pe	chr11	59512550	59512807	258	59512743	9	4.46937	3.41876
Nc-1-IP_pe	chr11	59532006	59532637	632	59532192	11	6.03439	4.10251
Nc-1-IP_pe	chr11	59568944	59569216	273	59569098	12	6.86906	4.44438
Nc-1-IP_pe	chr11	59570463	59570716	254	59570493	7	3.03708	2.71711
Nc-1-IP_pe	chr11	59585002	59585289	288	59585181	13	7.7352	4.78626
Nc-1-IP_pe	chr11	59604543	59604845	303	59604644	13	6.62436	4.14003
Nc-1-IP_pe	chr11	59640526	59641009	484	59640871	12	5.23082	3.49289
Nc-1-IP_pe	chr11	59680323	59680592	270	59680519	12	4.28158	2.97474
Nc-1-IP_pe	chr11	59699201	59700382	1182	59699870	14	6.69566	4.03025
Nc-1-IP_pe	chr11	59705968	59706334	367	59706209	10	5.23357	3.76063
Nc-1-IP_pe	chr11	59748825	59749277	453	59749253	12	4.51041	3.09723
Nc-1-IP_pe	chr11	59753705	59754139	435	59753828	13	5.68753	3.61857
Nc-1-IP_pe	chr11	59755124	59755642	519	59755502	18	6.40661	3.40507
Nc-1-IP_pe	chr11	59765397	59765699	303	59765556	11	5.81652	3.97078
Nc-1-IP_pe	chr11	59793107	59793361	255	59793217	12	6.46791	4.20548
Nc-1-IP_pe	chr11	59795443	59796383	941	59795478	10	5.17577	3.72509
Nc-1-IP_pe	chr11	59798528	59798792	265	59798670	11	6.03439	4.10251
Nc-1-IP_pe	chr11	59810320	59810971	652	59810779	19	10.9318	5.43653
Nc-1-IP_pe	chr11	59812973	59813237	265	59813096	11	6.03439	4.10251
Nc-1-IP_pe	chr11	59813521	59813788	268	59813782	8	3.08755	2.66145
Nc-1-IP_pe	chr11	59847160	59847533	374	59847459	8	3.74505	3.07688
Nc-1-IP_pe	chr11	59898012	59898319	308	59898208	10	3.50482	2.73328
Nc-1-IP_pe	chr11	59899560	59899860	301	59899619	8	3.74505	3.07688
Nc-1-IP_pe	chr11	59931701	59932477	777	59932266	13	7.56612	4.68618
Nc-1-IP_pe	chr11	60020450	60020964	515	60020834	11	4.54404	3.2242
Nc-1-IP_pe	chr11	60108257	60108690	434	60108422	15	7.46994	4.29894
Nc-1-IP_pe	chr11	60168241	60169295	1055	60168744	10	4.33501	3.21677
Nc-1-IP_pe	chr11	60180071	60180344	274	60180242	10	5.23357	3.76063
Nc-1-IP_pe	chr11	60288689	60289450	762	60288949	14	6.69566	4.03025
Nc-1-IP_pe	chr11	60360290	60360583	294	60360444	11	5.77463	3.94557
Nc-1-IP_pe	chr11	60367992	60368248	257	60368155	12	6.86906	4.44438
Nc-1-IP_pe	chr11	60450967	60451402	436	60451298	20	5.70098	2.94771
Nc-1-IP_pe	chr11	60590842	60591119	278	60590979	9	3.72192	2.95716

Nc-1-IP_pe	chr11	60615472	60615823	352	60615703	9	4.35492	3.34727
Nc-1-IP_pe	chr11	60618293	60618866	574	60618728	10	5.23357	3.76063
Nc-1-IP_pe	chr11	60621646	60623094	1449	60623038	14	6.69566	4.03025
Nc-1-IP_pe	chr11	60632238	60632498	261	60632274	11	5.7051	3.90381
Nc-1-IP_pe	chr11	60692370	60692645	276	60692420	9	3.27326	2.68684
Nc-1-IP_pe	chr11	60703476	60703774	299	60703655	9	4.46937	3.41876
Nc-1-IP_pe	chr11	60714077	60714348	272	60714252	8	3.74505	3.07688
Nc-1-IP_pe	chr11	60714873	60715159	287	60714987	12	6.58417	4.27436
Nc-1-IP_pe	chr11	60739023	60740301	1279	60739868	15	5.20142	3.14789
Nc-1-IP_pe	chr11	60743740	60743995	256	60743864	16	7.79347	4.31685
Nc-1-IP_pe	chr11	60747810	60748693	884	60747962	15	7.71696	4.43252
Nc-1-IP_pe	chr11	60755421	60755769	349	60755527	10	3.08907	2.49869
Nc-1-IP_pe	chr11	60767043	60767679	637	60767490	18	7.1522	3.73811
Nc-1-IP_pe	chr11	60771003	60771492	490	60771417	12	4.04688	2.85073
Nc-1-IP_pe	chr11	60772059	60772316	258	60772287	13	4.56123	3.02417
Nc-1-IP_pe	chr11	60775818	60776226	409	60775980	13	3.77558	2.63125
Nc-1-IP_pe	chr11	60782266	60782840	575	60782283	10	4.63645	3.39704
Nc-1-IP_pe	chr11	60790246	60791257	1012	60791159	17	8.15431	4.35118
Nc-1-IP_pe	chr11	60793980	60794945	966	60794945	11	2.96397	2.36091
Nc-1-IP_pe	chr11	60797801	60798376	576	60797932	14	6.44441	3.89444
Nc-1-IP_pe	chr11	60803978	60804242	265	60804090	13	5.9487	3.76157
Nc-1-IP_pe	chr11	60836885	60837176	292	60837079	11	3.64839	2.72584
Nc-1-IP_pe	chr11	60839854	60840117	264	60840019	16	6.33285	3.58457
Nc-1-IP_pe	chr11	60844000	60844530	531	60844374	21	8.85564	4.13481
Nc-1-IP_pe	chr11	60848806	60849074	269	60849022	15	5.20142	3.14789
Nc-1-IP_pe	chr11	60849562	60850041	480	60849651	14	5.30793	3.30182
Nc-1-IP_pe	chr11	60858408	60859090	683	60859002	13	4.9575	3.22911
Nc-1-IP_pe	chr11	60875243	60875548	306	60875473	13	4.88438	3.19095
Nc-1-IP_pe	chr11	60889276	60890254	979	60890104	19	10.6445	5.28749
Nc-1-IP_pe	chr11	60893354	60893822	469	60893741	11	5.02318	3.50036
Nc-1-IP_pe	chr11	60900543	60901305	763	60900991	13	3.67377	2.58159
Nc-1-IP_pe	chr11	60906181	60906450	270	60906361	16	5.07731	3.00018
Nc-1-IP_pe	chr11	60915385	60915734	350	60915495	16	5.73763	3.30235
Nc-1-IP_pe	chr11	60918764	60919048	285	60918862	18	7.65577	3.97082
Nc-1-IP_pe	chr11	60921747	60922707	961	60921920	17	7.11008	3.83807
Nc-1-IP_pe	chr11	60924458	60925786	1329	60925457	20	8.61811	4.15612
Nc-1-IP_pe	chr11	60926846	60927835	990	60927219	19	7.75121	3.89165
Nc-1-IP_pe	chr11	60928682	60929016	335	60928864	21	5.88587	2.94733
Nc-1-IP_pe	chr11	60931768	60932325	558	60931886	16	6.12223	3.48363
Nc-1-IP_pe	chr11	60933549	60934004	456	60933630	15	4.98779	3.04672
Nc-1-IP_pe	chr11	60934810	60935141	332	60934982	17	5.9911	3.31919
Nc-1-IP_pe	chr11	60943413	60943983	571	60943832	13	4.02562	2.7544
Nc-1-IP_pe	chr11	60944853	60945145	293	60944985	12	4.79401	3.25118
Nc-1-IP_pe	chr11	60946406	60946910	505	60946862	14	6.06519	3.69269
Nc-1-IP_pe	chr11	60948703	60949152	450	60948817	12	3.47963	2.55766

Nc-1-IP_pe	chr11	60950493	60951282	790	60951044	19	9.41173	4.66885
Nc-1-IP_pe	chr11	60951700	60952098	399	60951923	17	5.14803	2.94914
Nc-1-IP_pe	chr11	60955338	60955709	372	60955683	10	3.08907	2.49869
Nc-1-IP_pe	chr11	60967060	60967559	500	60967116	11	3.64839	2.72584
Nc-1-IP_pe	chr11	60970993	60971285	293	60971128	17	7.6273	4.08876
Nc-1-IP_pe	chr11	60971793	60972083	291	60971872	14	3.86727	2.6027
Nc-1-IP_pe	chr11	60972850	60973451	602	60973345	11	4.17167	3.01412
Nc-1-IP_pe	chr11	60974405	60974962	558	60974560	15	6.10627	3.59023
Nc-1-IP_pe	chr11	60977284	60977548	265	60977430	11	4.81752	3.38105
Nc-1-IP_pe	chr11	60982537	60982874	338	60982642	12	5.06062	3.39805
Nc-1-IP_pe	chr11	60984775	60985425	651	60984812	12	4.743	3.22332
Nc-1-IP_pe	chr11	60993025	60993335	311	60993090	15	7.37761	4.24941
Nc-1-IP_pe	chr11	60996301	60996710	410	60996509	14	6.06519	3.69269
Nc-1-IP_pe	chr11	61007600	61008582	983	61007734	14	4.21449	2.76599
Nc-1-IP_pe	chr11	61018071	61018441	371	61018356	14	5.43414	3.36584
Nc-1-IP_pe	chr11	61019090	61019453	364	61019265	15	4.06016	2.62146
Nc-1-IP_pe	chr11	61020794	61021046	253	61020863	11	3.64839	2.72584
Nc-1-IP_pe	chr11	61022229	61023394	1166	61023221	17	8.31233	4.43123
Nc-1-IP_pe	chr11	61026900	61028079	1180	61027144	15	7.62034	4.38009
Nc-1-IP_pe	chr11	61040467	61040838	372	61040701	14	6.99758	4.19566
Nc-1-IP_pe	chr11	61041140	61041454	315	61041238	10	4.16209	3.11446
Nc-1-IP_pe	chr11	61052534	61053060	527	61052975	14	6.06519	3.69269
Nc-1-IP_pe	chr11	61053369	61053656	288	61053502	11	4.20699	3.03387
Nc-1-IP_pe	chr11	61058363	61058736	374	61058662	12	4.93819	3.33035
Nc-1-IP_pe	chr11	61066471	61066749	279	61066660	9	3.24186	2.66813
Nc-1-IP_pe	chr11	61068589	61068941	353	61068753	18	7.93853	4.10423
Nc-1-IP_pe	chr11	61081091	61081519	429	61081246	13	5.36853	3.44651
Nc-1-IP_pe	chr11	61090028	61090416	389	61090271	13	4.02562	2.7544
Nc-1-IP_pe	chr11	61091728	61092013	286	61091868	18	7.47506	3.88659
Nc-1-IP_pe	chr11	61093293	61093579	287	61093403	12	5.31851	3.54206
Nc-1-IP_pe	chr11	61101916	61102199	284	61102031	11	3.11772	2.44174
Nc-1-IP_pe	chr11	61113285	61113541	257	61113421	14	4.21449	2.76599
Nc-1-IP_pe	chr11	61118125	61119059	935	61118715	13	4.41954	2.952
Nc-1-IP_pe	chr11	61123722	61124133	412	61123764	12	4.75569	3.23024
Nc-1-IP_pe	chr11	61125342	61125953	612	61125581	17	7.70506	4.12703
Nc-1-IP_pe	chr11	61127927	61128312	386	61128291	12	3.94559	2.79771
Nc-1-IP_pe	chr11	61131440	61132102	663	61131690	14	4.21449	2.76599
Nc-1-IP_pe	chr11	61133417	61133747	331	61133477	13	4.04902	2.76601
Nc-1-IP_pe	chr11	61134171	61135588	1418	61135412	19	5.90435	3.10381
Nc-1-IP_pe	chr11	61138372	61138693	322	61138462	13	5.48474	3.50885
Nc-1-IP_pe	chr11	61150720	61151188	469	61150952	18	6.40661	3.40507
Nc-1-IP_pe	chr11	61160328	61160922	595	61160915	16	4.25218	2.63809
Nc-1-IP_pe	chr11	61166624	61167406	783	61166647	19	2.87318	1.96343
Nc-1-IP_pe	chr11	61175124	61175423	300	61175246	16	7.75718	4.29799
Nc-1-IP_pe	chr11	61176160	61176618	459	61176366	12	4.24064	2.95299

Nc-1-IP_pe	chr11	61193704	61194015	312	61193746	11	3.22564	2.49886
Nc-1-IP_pe	chr11	61195740	61196201	462	61195827	15	5.70822	3.39286
Nc-1-IP_pe	chr11	61199851	61200248	398	61199955	15	4.7819	2.95039
Nc-1-IP_pe	chr11	61203245	61203600	356	61203444	16	5.39474	3.14402
Nc-1-IP_pe	chr11	61204427	61205093	667	61204645	27	6.34328	2.7488
Nc-1-IP_pe	chr11	61205932	61206773	842	61206449	18	5.72638	3.11298
Nc-1-IP_pe	chr11	61207199	61207870	672	61207409	25	7.78348	3.33136
Nc-1-IP_pe	chr11	61209744	61210136	393	61210031	19	5.51632	2.94814
Nc-1-IP_pe	chr11	61211089	61211359	271	61211187	23	5.63184	2.73435
Nc-1-IP_pe	chr11	61212354	61212638	285	61212411	21	5.5349	2.81884
Nc-1-IP_pe	chr11	61214444	61214724	281	61214655	18	4.84215	2.74937
Nc-1-IP_pe	chr11	61215460	61216207	748	61215570	26	8.84747	3.61717
Nc-1-IP_pe	chr11	61221050	61221423	374	61221082	14	3.71352	2.5314
Nc-1-IP_pe	chr11	61225031	61225487	457	61225334	19	4.8268	2.67939
Nc-1-IP_pe	chr11	61228406	61230184	1779	61229020	22	6.84408	3.22845
Nc-1-IP_pe	chr11	61231193	61231477	285	61231262	24	3.76759	2.10307
Nc-1-IP_pe	chr11	61233151	61233539	389	61233364	22	8.38838	3.82569
Nc-1-IP_pe	chr11	61234345	61234982	638	61234535	22	5.39765	2.71064
Nc-1-IP_pe	chr11	61244849	61245231	383	61245193	16	3.39261	2.27748
Nc-1-IP_pe	chr11	61246001	61246481	481	61246035	16	4.25218	2.63809
Nc-1-IP_pe	chr11	61248557	61248812	256	61248608	12	3.61497	2.62674
Nc-1-IP_pe	chr11	61265297	61266000	704	61265642	14	4.21449	2.76599
Nc-1-IP_pe	chr11	61266923	61267363	441	61267017	15	7.02624	4.06292
Nc-1-IP_pe	chr11	61273074	61274051	978	61273192	16	5.82808	3.34463
Nc-1-IP_pe	chr11	61274641	61274914	274	61274762	15	5.70822	3.39286
Nc-1-IP_pe	chr11	61280809	61281404	596	61280977	18	6.65364	3.51391
Nc-1-IP_pe	chr11	61283538	61283791	254	61283688	15	5.916	3.49534
Nc-1-IP_pe	chr11	61287573	61287847	275	61287699	15	5.83691	3.4562
Nc-1-IP_pe	chr11	61302332	61302880	549	61302462	12	2.87625	2.25568
Nc-1-IP_pe	chr11	61303335	61303687	353	61303512	11	5.24175	3.6284
Nc-1-IP_pe	chr11	61333064	61333945	882	61333770	14	5.43414	3.36584
Nc-1-IP_pe	chr11	61335629	61336029	401	61335967	13	5.38711	3.45645
Nc-1-IP_pe	chr11	61342653	61343611	959	61343559	12	4.70082	3.20033
Nc-1-IP_pe	chr11	61345166	61345907	742	61345316	11	5.02318	3.50036
Nc-1-IP_pe	chr11	61346510	61346775	266	61346577	11	4.84616	3.39759
Nc-1-IP_pe	chr11	61349191	61349497	307	61349257	10	3.08907	2.49869
Nc-1-IP_pe	chr11	61356626	61357007	382	61356884	15	6.97567	4.03634
Nc-1-IP_pe	chr11	61383852	61384153	302	61383894	10	4.62142	3.388
Nc-1-IP_pe	chr11	61392366	61393049	684	61392649	18	9.19569	4.72112
Nc-1-IP_pe	chr11	61396248	61396533	286	61396346	13	6.72991	4.20021
Nc-1-IP_pe	chr11	61403982	61404242	261	61404205	9	3.92293	3.08
Nc-1-IP_pe	chr11	61412376	61412643	268	61412433	10	4.98602	3.60892
Nc-1-IP_pe	chr11	61414863	61415203	341	61415039	13	7.7352	4.78626
Nc-1-IP_pe	chr11	61418876	61419173	298	61418914	8	3.64414	3.01254
Nc-1-IP_pe	chr11	61429408	61429757	350	61429540	16	6.1093	3.47747

Nc-1-IP_pe	chr11	61435806	61436084	279	61435888	12	5.61153	3.70794
Nc-1-IP_pe	chr11	61443798	61444105	308	61443866	10	4.47495	3.30017
Nc-1-IP_pe	chr11	61457979	61458803	825	61458019	8	3.08755	2.66145
Nc-1-IP_pe	chr11	61471021	61471334	314	61471178	13	5.9487	3.76157
Nc-1-IP_pe	chr11	61475037	61475570	534	61475079	13	5.36853	3.44651
Nc-1-IP_pe	chr11	61477582	61478179	598	61477774	17	8.31233	4.43123
Nc-1-IP_pe	chr11	61482541	61482801	261	61482650	13	4.86362	3.18015
Nc-1-IP_pe	chr11	61483845	61484124	280	61483976	15	7.11172	4.10799
Nc-1-IP_pe	chr11	61485210	61485658	449	61485351	11	3.73563	2.77334
Nc-1-IP_pe	chr11	61496113	61496425	313	61496220	12	4.63088	3.16234
Nc-1-IP_pe	chr11	61514360	61514755	396	61514635	13	4.90859	3.20357
Nc-1-IP_pe	chr11	61518651	61519007	357	61518718	10	4.01082	3.02563
Nc-1-IP_pe	chr11	61523484	61524551	1068	61524387	13	5.86991	3.71823
Nc-1-IP_pe	chr11	61524912	61525196	285	61524970	10	3.21261	2.56787
Nc-1-IP_pe	chr11	61527855	61528373	519	61528222	11	4.69157	3.30855
Nc-1-IP_pe	chr11	61530981	61531697	717	61531292	11	4.71915	3.32439
Nc-1-IP_pe	chr11	61532042	61532530	489	61532157	12	5.15877	3.45264
Nc-1-IP_pe	chr11	61535125	61535719	595	61535284	14	6.71255	4.03944
Nc-1-IP_pe	chr11	61537216	61538072	857	61537596	13	5.59935	3.57072
Nc-1-IP_pe	chr11	61539529	61540255	727	61539705	15	6.95893	4.02756
Nc-1-IP_pe	chr11	61545103	61545489	387	61545324	13	5.4426	3.4862
Nc-1-IP_pe	chr11	61545776	61546274	499	61545826	13	4.41954	2.952
Nc-1-IP_pe	chr11	61546574	61547156	583	61546663	15	6.85973	3.97568
Nc-1-IP_pe	chr11	61547715	61548254	540	61548135	11	4.35236	3.11555
Nc-1-IP_pe	chr11	61550876	61551679	804	61551390	14	4.21449	2.76599
Nc-1-IP_pe	chr11	61553231	61553517	287	61553381	13	4.02562	2.7544
Nc-1-IP_pe	chr11	61559609	61559881	273	61559843	10	3.89064	2.95552
Nc-1-IP_pe	chr11	61560220	61560794	575	61560629	17	9.37749	4.98658
Nc-1-IP_pe	chr11	61563138	61563401	264	61563256	12	5.61153	3.70794
Nc-1-IP_pe	chr11	61567797	61568066	270	61567927	12	3.83688	2.74114
Nc-1-IP_pe	chr11	61574836	61575201	366	61575014	12	5.31851	3.54206
Nc-1-IP_pe	chr11	61576151	61576599	449	61576265	11	4.54404	3.2242
Nc-1-IP_pe	chr11	61579986	61580661	676	61580448	12	4.87193	3.29389
Nc-1-IP_pe	chr11	61581465	61581826	362	61581600	13	5.58484	3.56287
Nc-1-IP_pe	chr11	61583159	61583672	514	61583500	16	7.39088	4.10945
Nc-1-IP_pe	chr11	61587737	61588098	362	61587968	14	4.95172	3.12357
Nc-1-IP_pe	chr11	61588569	61589091	523	61588708	17	7.36791	3.96218
Nc-1-IP_pe	chr11	61595182	61595490	309	61595332	14	6.96097	4.17548
Nc-1-IP_pe	chr11	61596154	61596697	544	61596570	11	4.54404	3.2242
Nc-1-IP_pe	chr11	61599194	61600157	964	61599430	18	9.44113	4.84598
Nc-1-IP_pe	chr11	61609050	61609629	580	61609414	14	6.83507	4.10633
Nc-1-IP_pe	chr11	61610155	61610868	714	61610839	12	3.47963	2.55766
Nc-1-IP_pe	chr11	61611449	61611786	338	61611628	12	5.70894	3.7636
Nc-1-IP_pe	chr11	61612152	61612443	292	61612249	16	8.26996	4.56762
Nc-1-IP_pe	chr11	61614043	61614373	331	61614217	13	5.03208	3.26819

Nc-1-IP_pe	chr11	61620721	61621034	314	61620841	13	5.91681	3.74401
Nc-1-IP_pe	chr11	61633650	61633998	349	61633669	11	2.96397	2.36091
Nc-1-IP_pe	chr11	61646177	61646496	320	61646370	10	3.89064	2.95552
Nc-1-IP_pe	chr11	61651195	61651453	259	61651423	14	3.71352	2.5314
Nc-1-IP_pe	chr11	61652579	61652876	298	61652762	12	3.83688	2.74114
Nc-1-IP_pe	chr11	61654430	61654866	437	61654700	10	4.39571	3.25288
Nc-1-IP_pe	chr11	61661874	61662256	383	61661959	9	3.27326	2.68684
Nc-1-IP_pe	chr11	61665539	61665815	277	61665718	10	3.89064	2.95552
Nc-1-IP_pe	chr11	61667357	61667768	412	61667739	10	4.20113	3.13749
Nc-1-IP_pe	chr11	61669252	61669644	393	61669561	11	4.06403	2.95415
Nc-1-IP_pe	chr11	61679896	61680909	1014	61680080	20	11.3299	5.45222
Nc-1-IP_pe	chr11	61693834	61694430	597	61694112	18	6.63038	3.50359
Nc-1-IP_pe	chr11	61699160	61699710	551	61699307	19	7.84735	3.93486
Nc-1-IP_pe	chr11	61706656	61707232	577	61706801	12	3.47963	2.55766
Nc-1-IP_pe	chr11	61717167	61717466	300	61717215	10	3.71867	2.85592
Nc-1-IP_pe	chr11	61721578	61721996	419	61721982	12	4.57012	3.12944
Nc-1-IP_pe	chr11	61726757	61727048	292	61726890	16	6.8987	3.86161
Nc-1-IP_pe	chr11	61733723	61735107	1385	61733828	15	6.66737	3.87581
Nc-1-IP_pe	chr11	61737153	61738132	980	61737781	16	5.37454	3.13479
Nc-1-IP_pe	chr11	61741231	61741726	496	61741389	14	5.51541	3.4073
Nc-1-IP_pe	chr11	61745125	61745836	712	61745767	14	6.06519	3.69269
Nc-1-IP_pe	chr11	61747960	61748642	683	61748322	17	6.38155	3.49661
Nc-1-IP_pe	chr11	61762904	61763166	263	61763005	14	6.57647	3.96562
Nc-1-IP_pe	chr11	61769199	61769961	763	61769757	20	10.8645	5.21807
Nc-1-IP_pe	chr11	61773888	61774155	268	61774026	14	6.19157	3.75949
Nc-1-IP_pe	chr11	61775466	61775756	291	61775601	13	5.38711	3.45645
Nc-1-IP_pe	chr11	61776013	61776459	447	61776412	13	5.03208	3.26819
Nc-1-IP_pe	chr11	61777536	61778055	520	61777541	12	3.50082	2.56844
Nc-1-IP_pe	chr11	61779650	61780166	517	61779780	12	4.53417	3.11004
Nc-1-IP_pe	chr11	61787210	61788000	791	61787832	11	3.78682	2.80131
Nc-1-IP_pe	chr11	61790180	61790480	301	61790183	12	2.87625	2.25568
Nc-1-IP_pe	chr11	61792397	61793360	964	61792810	20	7.85753	3.8227
Nc-1-IP_pe	chr11	61816396	61816729	334	61816589	17	4.43891	2.65107
Nc-1-IP_pe	chr11	61825639	61825983	345	61825828	10	3.08907	2.49869
Nc-1-IP_pe	chr11	61827106	61828014	909	61827367	15	6.10627	3.59023
Nc-1-IP_pe	chr11	61828943	61829206	264	61829054	15	3.74763	2.48304
Nc-1-IP_pe	chr11	61837451	61837961	511	61837706	17	5.14803	2.94914
Nc-1-IP_pe	chr11	61853630	61854077	448	61853743	17	5.54967	3.12324
Nc-1-IP_pe	chr11	61856362	61856790	429	61856418	12	3.77112	2.7071
Nc-1-IP_pe	chr11	61857417	61858094	678	61857973	13	4.52827	3.00733
Nc-1-IP_pe	chr11	61860357	61860812	456	61860391	13	3.6894	2.58919
Nc-1-IP_pe	chr11	61862092	61864085	1994	61862477	16	4.96462	2.94973
Nc-1-IP_pe	chr11	61865227	61865596	370	61865357	13	5.10817	3.30823
Nc-1-IP_pe	chr11	61866501	61867220	720	61866867	15	4.40338	2.77622
Nc-1-IP_pe	chr11	61869139	61869491	353	61869241	13	4.89646	3.19725

Nc-1-IP_pe	chr11	61869829	61870285	457	61869974	13	4.67336	3.0817
Nc-1-IP_pe	chr11	61871135	61872020	886	61871378	16	5.82808	3.34463
Nc-1-IP_pe	chr11	61872909	61873231	323	61873062	17	7.6273	4.08876
Nc-1-IP_pe	chr11	61877917	61878270	354	61878258	14	4.13959	2.7305
Nc-1-IP_pe	chr11	61878927	61879207	281	61879031	15	4.70031	2.91253
Nc-1-IP_pe	chr11	61890238	61890793	556	61890549	18	8.10208	4.1823
Nc-1-IP_pe	chr11	61897966	61898366	401	61898198	14	5.43414	3.36584
Nc-1-IP_pe	chr11	61901232	61901615	384	61901455	16	6.92507	3.87472
Nc-1-IP_pe	chr11	61905875	61906602	728	61906169	13	4.02562	2.7544
Nc-1-IP_pe	chr11	61907791	61908144	354	61907815	11	4.06403	2.95415
Nc-1-IP_pe	chr11	61911095	61911772	678	61911257	10	4.47495	3.30017
Nc-1-IP_pe	chr11	61913087	61913701	615	61913447	11	4.81752	3.38105
Nc-1-IP_pe	chr11	61916612	61917545	934	61916772	14	6.50995	3.92971
Nc-1-IP_pe	chr11	61919392	61919728	337	61919581	12	3.47963	2.55766
Nc-1-IP_pe	chr11	61920605	61920992	388	61920711	10	3.41375	2.68146
Nc-1-IP_pe	chr11	61922542	61923011	470	61922742	18	9.13587	4.6909
Nc-1-IP_pe	chr11	61932815	61933109	295	61932915	13	7.7352	4.78626
Nc-1-IP_pe	chr11	61939953	61940228	276	61940120	8	3.08755	2.66145
Nc-1-IP_pe	chr11	61940487	61940845	359	61940612	13	7.42513	4.60316
Nc-1-IP_pe	chr11	61945318	61945656	339	61945585	13	6.72991	4.20021
Nc-1-IP_pe	chr11	61947302	61947578	277	61947413	15	7.75613	4.45384
Nc-1-IP_pe	chr11	61949944	61950522	579	61950084	10	4.60647	3.37901
Nc-1-IP_pe	chr11	61951369	61951917	549	61951637	14	7.30138	4.36445
Nc-1-IP_pe	chr11	61954653	61954992	340	61954945	12	3.47963	2.55766
Nc-1-IP_pe	chr11	61955552	61955816	265	61955621	13	4.67336	3.0817
Nc-1-IP_pe	chr11	61956547	61957155	609	61957051	14	6.06519	3.69269
Nc-1-IP_pe	chr11	61959776	61960126	351	61959863	15	4.40338	2.77622
Nc-1-IP_pe	chr11	61967326	61967611	286	61967460	38	15.3772	4.5963
Nc-1-IP_pe	chr11	61973388	61973681	294	61973414	11	3.58722	2.69267
Nc-1-IP_pe	chr11	61975527	61975801	275	61975604	11	4.40231	3.14376
Nc-1-IP_pe	chr11	61976481	61977298	818	61977188	14	6.87069	4.12585
Nc-1-IP_pe	chr11	61983661	61984355	695	61983797	14	5.51541	3.4073
Nc-1-IP_pe	chr11	62000951	62001585	635	62001188	14	5.51541	3.4073
Nc-1-IP_pe	chr11	62011980	62012252	273	62012217	11	4.61014	3.26192
Nc-1-IP_pe	chr11	62028924	62029186	263	62029096	10	4.57677	3.36116
Nc-1-IP_pe	chr11	62058280	62058595	316	62058457	9	3.54889	2.85226
Nc-1-IP_pe	chr11	62067900	62068694	795	62068497	10	4.29423	3.19257
Nc-1-IP_pe	chr11	62077912	62078385	474	62078031	10	5.01973	3.6295
Nc-1-IP_pe	chr11	62102156	62102496	341	62102359	10	5.23357	3.76063
Nc-1-IP_pe	chr11	62128254	62128511	258	62128476	16	4.59224	2.7853
Nc-1-IP_pe	chr11	62129799	62130382	584	62129877	16	6.80394	3.81462
Nc-1-IP_pe	chr11	62131630	62131995	366	62131895	12	4.24064	2.95299
Nc-1-IP_pe	chr11	62136351	62136646	296	62136563	13	6.01225	3.79665
Nc-1-IP_pe	chr11	62137873	62138389	517	62138002	14	6.13058	3.7272
Nc-1-IP_pe	chr11	62138648	62139488	841	62139377	11	4.93354	3.44821

Nc-1-IP_pe	chr11	62141411	62141697	287	62141602	13	5.36853	3.44651
Nc-1-IP_pe	chr11	62144895	62145344	450	62145056	15	8.40553	4.81272
Nc-1-IP_pe	chr11	62148325	62148829	505	62148747	14	7.68959	4.58341
Nc-1-IP_pe	chr11	62150191	62150892	702	62150760	15	6.19428	3.63445
Nc-1-IP_pe	chr11	62151769	62152075	307	62151944	11	6.03439	4.10251
Nc-1-IP_pe	chr11	62157863	62158349	487	62158026	12	6.86906	4.44438
Nc-1-IP_pe	chr11	62166988	62167875	888	62167471	10	4.39571	3.25288
Nc-1-IP_pe	chr11	62189951	62190352	402	62190203	9	3.12089	2.59629
Nc-1-IP_pe	chr11	62192439	62193040	602	62192907	15	6.19428	3.63445
Nc-1-IP_pe	chr11	62193932	62194581	650	62194531	10	5.23357	3.76063
Nc-1-IP_pe	chr11	62209401	62209734	334	62209596	10	3.89064	2.95552
Nc-1-IP_pe	chr11	62278851	62279860	1010	62279612	13	7.56612	4.68618
Nc-1-IP_pe	chr11	62308380	62308678	299	62308527	12	6.86906	4.44438
Nc-1-IP_pe	chr11	62312547	62313025	479	62312766	9	4.25966	3.28797
Nc-1-IP_pe	chr11	62323404	62323660	257	62323587	12	5.84943	3.84431
Nc-1-IP_pe	chr11	62367453	62367886	434	62367498	9	4.25966	3.28797
Nc-1-IP_pe	chr11	62371173	62371664	492	62371281	11	4.06403	2.95415
Nc-1-IP_pe	chr11	62371982	62372702	721	62372180	18	11.6181	6.01202
Nc-1-IP_pe	chr11	62385811	62386176	366	62386077	19	12.2819	6.16
Nc-1-IP_pe	chr11	62398056	62398772	717	62398473	11	4.54404	3.2242
Nc-1-IP_pe	chr11	62399466	62400138	673	62399719	13	4.02562	2.7544
Nc-1-IP_pe	chr11	62413209	62413889	681	62413418	15	7.45104	4.28878
Nc-1-IP_pe	chr11	62426264	62426768	505	62426697	12	4.24064	2.95299
Nc-1-IP_pe	chr11	62427250	62427556	307	62427412	11	4.24272	3.05389
Nc-1-IP_pe	chr11	62434365	62434844	480	62434655	14	8.06563	4.79881
Nc-1-IP_pe	chr11	62439873	62440810	938	62440044	13	6.36468	3.99316
Nc-1-IP_pe	chr11	62448013	62448460	448	62448387	12	5.23082	3.49289
Nc-1-IP_pe	chr11	62459894	62460233	340	62459942	14	4.60011	2.95114
Nc-1-IP_pe	chr11	62469113	62469395	283	62469298	14	5.03085	3.16286
Nc-1-IP_pe	chr11	62471151	62471663	513	62471658	10	3.87863	2.94853
Nc-1-IP_pe	chr11	62472125	62472608	484	62472519	13	4.02562	2.7544
Nc-1-IP_pe	chr11	62485320	62485572	253	62485474	13	5.9487	3.76157
Nc-1-IP_pe	chr11	62490272	62490705	434	62490553	9	3.72192	2.95716
Nc-1-IP_pe	chr11	62494618	62494908	291	62494748	13	5.9487	3.76157
Nc-1-IP_pe	chr11	62505252	62505658	407	62505327	16	6.80394	3.81462
Nc-1-IP_pe	chr11	62509329	62509766	438	62509486	13	6.27805	3.94455
Nc-1-IP_pe	chr11	62517111	62517476	366	62517174	12	4.417	3.04704
Nc-1-IP_pe	chr11	62530758	62531032	275	62530897	17	7.06589	3.81697
Nc-1-IP_pe	chr11	62536761	62537092	332	62536802	10	3.08907	2.49869
Nc-1-IP_pe	chr11	62537800	62538244	445	62537937	14	5.74019	3.52296
Nc-1-IP_pe	chr11	62539551	62539810	260	62539654	15	5.83691	3.4562
Nc-1-IP_pe	chr11	62546124	62546696	573	62546491	18	6.28754	3.35314
Nc-1-IP_pe	chr11	62559548	62559827	280	62559783	12	4.51041	3.09723
Nc-1-IP_pe	chr11	62581808	62582113	306	62582093	11	3.64839	2.72584
Nc-1-IP_pe	chr11	62593070	62593404	335	62593356	13	4.29558	2.88934

Nc-1-IP_pe	chr11	62593800	62594439	640	62593934	16	6.12223	3.48363
Nc-1-IP_pe	chr11	62596869	62597281	413	62597054	15	4.06016	2.62146
Nc-1-IP_pe	chr11	62601403	62601785	383	62601535	25	8.22195	3.4832
Nc-1-IP_pe	chr11	62602469	62603321	853	62602657	17	6.13258	3.38303
Nc-1-IP_pe	chr11	62610449	62611980	1532	62611898	23	7.42848	3.36353
Nc-1-IP_pe	chr11	62613266	62613855	590	62613502	20	3.90729	2.29119
Nc-1-IP_pe	chr11	62615343	62615854	512	62615452	17	6.13258	3.38303
Nc-1-IP_pe	chr11	62616474	62617598	1125	62617160	19	7.42515	3.74675
Nc-1-IP_pe	chr11	62633146	62634252	1107	62633993	16	5.73763	3.30235
Nc-1-IP_pe	chr11	62646115	62646597	483	62646387	21	7.10961	3.41419
Nc-1-IP_pe	chr11	62647266	62647813	548	62647427	20	8.4457	4.07937
Nc-1-IP_pe	chr11	62648613	62649155	543	62648760	14	4.59463	2.94848
Nc-1-IP_pe	chr11	62652892	62653374	483	62653082	18	6.40661	3.40507
Nc-1-IP_pe	chr11	62654881	62655146	266	62655089	13	4.41954	2.952
Nc-1-IP_pe	chr11	62656091	62656447	357	62656352	16	5.37454	3.13479
Nc-1-IP_pe	chr11	62657416	62657897	482	62657566	12	5.07448	3.40574
Nc-1-IP_pe	chr11	62659318	62659737	420	62659437	14	5.18557	3.24018
Nc-1-IP_pe	chr11	62660990	62661310	321	62661179	14	6.06519	3.69269
Nc-1-IP_pe	chr11	62665143	62666950	1808	62666866	16	6.33285	3.58457
Nc-1-IP_pe	chr11	62669570	62670011	442	62669640	15	4.80057	2.95908
Nc-1-IP_pe	chr11	62670619	62670906	288	62670726	12	3.83688	2.74114
Nc-1-IP_pe	chr11	62671414	62671801	388	62671580	17	5.4568	3.08264
Nc-1-IP_pe	chr11	62673813	62674219	407	62674134	12	3.35798	2.496
Nc-1-IP_pe	chr11	62678460	62679138	679	62678616	22	6.44373	3.08129
Nc-1-IP_pe	chr11	62684251	62684656	406	62684469	17	7.6273	4.08876
Nc-1-IP_pe	chr11	62692275	62692933	659	62692833	16	7.66757	4.25154
Nc-1-IP_pe	chr11	62694112	62695138	1027	62694484	17	8.24512	4.3971
Nc-1-IP_pe	chr11	62707423	62707912	490	62707830	17	6.25544	3.43888
Nc-1-IP_pe	chr11	62708602	62709124	523	62708912	17	6.25544	3.43888
Nc-1-IP_pe	chr11	62715833	62716090	258	62715964	15	7.2695	4.19169
Nc-1-IP_pe	chr11	62717531	62717793	263	62717715	13	5.9487	3.76157
Nc-1-IP_pe	chr11	62720255	62720630	376	62720350	16	6.37501	3.60492
Nc-1-IP_pe	chr11	62722011	62722281	271	62722146	15	6.6517	3.86771
Nc-1-IP_pe	chr11	62726620	62726905	286	62726732	22	4.53777	2.41958
Nc-1-IP_pe	chr11	62740571	62741239	669	62740693	17	8.7831	4.67333
Nc-1-IP_pe	chr11	62756585	62756893	309	62756717	15	5.62037	3.34989
Nc-1-IP_pe	chr11	62761563	62762075	513	62761931	20	6.60733	3.30311
Nc-1-IP_pe	chr11	62763339	62763724	386	62763482	14	3.86727	2.6027
Nc-1-IP_pe	chr11	62775731	62777068	1338	62776620	14	5.47279	3.38553
Nc-1-IP_pe	chr11	62778034	62778361	328	62778134	14	5.75402	3.53012
Nc-1-IP_pe	chr11	62782574	62782872	299	62782750	15	5.66913	3.37372
Nc-1-IP_pe	chr11	62786813	62787073	261	62786913	21	5.1031	2.66385
Nc-1-IP_pe	chr11	62791233	62791594	362	62791413	16	4.53479	2.76024
Nc-1-IP_pe	chr11	62795326	62795738	413	62795387	13	3.58081	2.5365
Nc-1-IP_pe	chr11	62797337	62797943	607	62797401	15	4.70031	2.91253

Nc-1-IP_pe	chr11	62798526	62799046	521	62798992	17	4.78101	2.79343
Nc-1-IP_pe	chr11	62799362	62799615	254	62799591	13	2.94177	2.23242
Nc-1-IP_pe	chr11	62805106	62805791	686	62805264	29	11.0682	4.10499
Nc-1-IP_pe	chr11	62806649	62807585	937	62806787	16	4.59754	2.78762
Nc-1-IP_pe	chr11	62811318	62811774	457	62811713	12	3.16137	2.39719
Nc-1-IP_pe	chr11	62821157	62822476	1320	62821190	17	6.47874	3.54137
Nc-1-IP_pe	chr11	62824033	62824628	596	62824503	17	6.55057	3.57461
Nc-1-IP_pe	chr11	62830997	62831616	620	62831174	25	7.78348	3.33136
Nc-1-IP_pe	chr11	62832203	62833039	837	62832294	21	5.2077	2.70109
Nc-1-IP_pe	chr11	62833540	62833796	257	62833611	16	5.48356	3.18474
Nc-1-IP_pe	chr11	62839318	62839697	380	62839545	40	15.586	4.47327
Nc-1-IP_pe	chr11	62855883	62856220	338	62856187	19	3.71826	2.26621
Nc-1-IP_pe	chr11	62861512	62861818	307	62861715	15	6.01011	3.54215
Nc-1-IP_pe	chr11	62863142	62863508	367	62863284	15	4.7819	2.95039
Nc-1-IP_pe	chr11	62869916	62870386	471	62870116	11	3.28475	2.53029
Nc-1-IP_pe	chr11	62871081	62871447	367	62871293	13	5.73236	3.64298
Nc-1-IP_pe	chr11	62887291	62888261	971	62888119	16	5.98198	3.41707
Nc-1-IP_pe	chr11	62888608	62889110	503	62888658	14	4.21449	2.76599
Nc-1-IP_pe	chr11	62889441	62889965	525	62889544	16	6.24025	3.54005
Nc-1-IP_pe	chr11	62890925	62891419	495	62891165	11	3.28475	2.53029
Nc-1-IP_pe	chr11	62895566	62896105	540	62895948	16	8.21011	4.53581
Nc-1-IP_pe	chr11	62903571	62904060	490	62903746	11	4.06403	2.95415
Nc-1-IP_pe	chr11	62910115	62910851	737	62910307	14	4.73128	3.01506
Nc-1-IP_pe	chr11	62912401	62912723	323	62912686	14	5.39586	3.34637
Nc-1-IP_pe	chr11	62917017	62917356	340	62917290	12	3.96559	2.80816
Nc-1-IP_pe	chr11	62921552	62922026	475	62921725	14	5.0786	3.18665
Nc-1-IP_pe	chr11	62923657	62924126	470	62923958	14	5.09034	3.19251
Nc-1-IP_pe	chr11	62927217	62927471	255	62927392	14	4.8399	3.06836
Nc-1-IP_pe	chr11	62929956	62930529	574	62930126	14	5.30793	3.30182
Nc-1-IP_pe	chr11	62938864	62939136	273	62938942	11	3.64839	2.72584
Nc-1-IP_pe	chr11	62948901	62949155	255	62948987	20	7.97282	3.87239
Nc-1-IP_pe	chr11	62957776	62958072	297	62957985	13	4.41954	2.952
Nc-1-IP_pe	chr11	62959306	62960034	729	62959482	14	5.03085	3.16286
Nc-1-IP_pe	chr11	62961588	62961874	287	62961761	11	3.64839	2.72584
Nc-1-IP_pe	chr11	62975308	62975600	293	62975424	12	4.47508	3.07822
Nc-1-IP_pe	chr11	62976933	62977389	457	62976972	14	4.04994	2.68821
Nc-1-IP_pe	chr11	62984031	62984363	333	62984083	10	3.57781	2.77499
Nc-1-IP_pe	chr11	62991688	62992121	434	62991774	14	6.19157	3.75949
Nc-1-IP_pe	chr11	62993327	62993595	269	62993357	12	4.57012	3.12944
Nc-1-IP_pe	chr11	62995377	62995876	500	62995807	12	2.87625	2.25568
Nc-1-IP_pe	chr11	62997479	62997892	414	62997661	14	5.43414	3.36584
Nc-1-IP_pe	chr11	63009087	63009477	391	63009265	15	5.66913	3.37372
Nc-1-IP_pe	chr11	63010810	63011144	335	63010969	16	4.25252	2.63824
Nc-1-IP_pe	chr11	63014530	63015402	873	63015311	15	6.46757	3.77313
Nc-1-IP_pe	chr11	63015811	63016380	570	63016176	16	6.33285	3.58457

Nc-1-IP_pe	chr11	63017265	63017839	575	63017536	13	5.38711	3.45645
Nc-1-IP_pe	chr11	63023864	63024263	400	63024007	17	5.14803	2.94914
Nc-1-IP_pe	chr11	63026700	63027151	452	63026967	13	5.36853	3.44651
Nc-1-IP_pe	chr11	63047527	63047920	394	63047721	8	3.4979	2.91965
Nc-1-IP_pe	chr11	63052220	63052586	367	63052479	17	5.9911	3.31919
Nc-1-IP_pe	chr11	63078337	63078997	661	63078510	31	22.6493	8.59787
Nc-1-IP_pe	chr11	63087671	63088216	546	63087907	12	6.21727	4.05801
Nc-1-IP_pe	chr11	63105128	63105691	564	63105210	10	4.39571	3.25288
Nc-1-IP_pe	chr11	63115099	63115534	436	63115317	11	5.03835	3.5092
Nc-1-IP_pe	chr11	63163660	63163957	298	63163825	13	5.36853	3.44651
Nc-1-IP_pe	chr11	63236175	63236795	621	63236633	15	5.66913	3.37372
Nc-1-IP_pe	chr11	63278060	63278541	482	63278412	12	5.61153	3.70794
Nc-1-IP_pe	chr11	63278949	63279266	318	63279085	10	3.08907	2.49869
Nc-1-IP_pe	chr11	63283169	63283472	304	63283186	12	3.58823	2.61305
Nc-1-IP_pe	chr11	63313325	63313796	472	63313604	22	10.0534	4.52509
Nc-1-IP_pe	chr11	63320960	63321322	363	63321263	10	5.00283	3.61918
Nc-1-IP_pe	chr11	63349741	63350114	374	63349893	14	5.43414	3.36584
Nc-1-IP_pe	chr11	63417458	63418070	613	63417698	10	3.08907	2.49869
Nc-1-IP_pe	chr11	63418876	63419245	370	63419112	14	6.61011	3.98382
Nc-1-IP_pe	chr11	63430973	63431269	297	63431089	14	5.51541	3.4073
Nc-1-IP_pe	chr11	63435389	63435702	314	63435553	26	11.0543	4.42371
Nc-1-IP_pe	chr11	63460960	63461233	274	63461068	10	5.10552	3.682
Nc-1-IP_pe	chr11	63463971	63464282	312	63464190	11	5.66869	3.88198
Nc-1-IP_pe	chr11	63464873	63465232	360	63465022	13	7.12853	4.42991
Nc-1-IP_pe	chr11	63490576	63491346	771	63491067	19	10.6915	5.31176
Nc-1-IP_pe	chr11	63511247	63511687	441	63511550	13	3.67377	2.58159
Nc-1-IP_pe	chr11	63513898	63514432	535	63514007	11	4.11366	2.98176
Nc-1-IP_pe	chr11	63515460	63516352	893	63516250	13	3.35769	2.42919
Nc-1-IP_pe	chr11	63523802	63524070	269	63523950	13	5.52733	3.5318
Nc-1-IP_pe	chr11	63530753	63531055	303	63530924	14	6.07045	3.69546
Nc-1-IP_pe	chr11	63537514	63539006	1493	63537981	15	5.70822	3.39286
Nc-1-IP_pe	chr11	63547140	63547545	406	63547279	13	4.02562	2.7544
Nc-1-IP_pe	chr11	63549881	63550156	276	63549975	17	5.56266	3.12894
Nc-1-IP_pe	chr11	63566162	63566543	382	63566369	17	8.30034	4.42513
Nc-1-IP_pe	chr11	63597076	63597353	278	63597168	15	4.7819	2.95039
Nc-1-IP_pe	chr11	63601709	63602231	523	63602130	13	4.78908	3.14145
Nc-1-IP_pe	chr11	63608242	63608552	311	63608442	15	5.83691	3.4562
Nc-1-IP_pe	chr11	63618203	63618509	307	63618423	12	4.54611	3.11648
Nc-1-IP_pe	chr11	63621654	63622262	609	63621965	13	4.41954	2.952
Nc-1-IP_pe	chr11	63639991	63640289	299	63640112	14	6.06519	3.69269
Nc-1-IP_pe	chr11	63645786	63646066	281	63645894	12	5.45448	3.61875
Nc-1-IP_pe	chr11	63674224	63674646	423	63674412	17	7.6885	4.11887
Nc-1-IP_pe	chr11	63686447	63686736	290	63686720	10	3.87863	2.94853
Nc-1-IP_pe	chr11	63696250	63696526	277	63696414	12	4.70082	3.20033
Nc-1-IP_pe	chr11	63700653	63700958	306	63700864	12	3.96559	2.80816

Nc-1-IP_pe	chr11	63711629	63711948	320	63711881	12	3.47963	2.55766
Nc-1-IP_pe	chr11	63728614	63728874	261	63728679	12	5.3037	3.53374
Nc-1-IP_pe	chr11	63732143	63732471	329	63732342	11	5.93136	4.04009
Nc-1-IP_pe	chr11	63733213	63733510	298	63733343	14	6.06519	3.69269
Nc-1-IP_pe	chr11	63748873	63749296	424	63749177	10	3.82047	2.91478
Nc-1-IP_pe	chr11	63754091	63754397	307	63754203	13	6.29523	3.95418
Nc-1-IP_pe	chr11	63756486	63756988	503	63756838	16	8.97063	4.94637
Nc-1-IP_pe	chr11	63762999	63763830	832	63763316	20	7.85753	3.8227
Nc-1-IP_pe	chr11	63767909	63768370	462	63768158	19	8.28644	4.135
Nc-1-IP_pe	chr11	63768826	63769905	1080	63769519	24	9.33472	3.98646
Nc-1-IP_pe	chr11	63772646	63773010	365	63772758	13	4.78908	3.14145
Nc-1-IP_pe	chr11	63790351	63790819	469	63790742	10	4.37633	3.24134
Nc-1-IP_pe	chr11	63794162	63794694	533	63794320	12	5.59553	3.69882
Nc-1-IP_pe	chr11	63797416	63797689	274	63797604	12	3.16137	2.39719
Nc-1-IP_pe	chr11	63809244	63809567	324	63809451	13	4.89646	3.19725
Nc-1-IP_pe	chr11	63813083	63813363	281	63813239	15	5.58327	3.33181
Nc-1-IP_pe	chr11	63818210	63819803	1594	63819602	19	8.2227	4.10566
Nc-1-IP_pe	chr11	63826401	63827299	899	63826997	17	7.02067	3.79543
[truncated: 742,650 more chars]
